# A novel one-class SVM based negative data sampling method for reconstructing proteome-wide HTLV-human protein interaction networks

**DOI:** 10.1038/srep08034

**Published:** 2015-01-26

**Authors:** Suyu Mei, Hao Zhu

**Affiliations:** 1 Software College, Shenyang Normal University, Shenyang, 110034, China; 2 Bioinformatics Section, School of Biomedical Sciences, Southern Medical University, Guangzhou, 510515, China

## Abstract

Protein-protein interaction (PPI) prediction is generally treated as a problem of binary classification wherein negative data sampling is still an open problem to be addressed. The commonly used random sampling is prone to yield less representative negative data with considerable false negatives. Meanwhile rational constraints are seldom exerted on model selection to reduce the risk of false positive predictions for most of the existing computational methods. In this work, we propose a novel negative data sampling method based on one-class SVM (support vector machine, SVM) to predict proteome-wide protein interactions between HTLV retrovirus and Homo sapiens, wherein one-class SVM is used to choose reliable and representative negative data, and two-class SVM is used to yield proteome-wide outcomes as predictive feedback for rational model selection. Computational results suggest that one-class SVM is more suited to be used as negative data sampling method than two-class PPI predictor, and the predictive feedback constrained model selection helps to yield a rational predictive model that reduces the risk of false positive predictions. Some predictions have been validated by the recent literature. Lastly, gene ontology based clustering of the predicted PPI networks is conducted to provide valuable cues for the pathogenesis of HTLV retrovirus.

Protein-protein interaction (PPI) plays an important role in mediating biological processes, cellular signaling pathways and development of organismal systems. Accurate mapping of the proteome-wide interactome is a central problem of proteomics and system biology. Although recent years have witnessed much progress in experimental identification and computational prediction of PPIs^1^, high risk of false discovery rate is still a problem to be effectively addressed^1^,^2^. For instances, *in vitro* detection methods such as affinity purification are prone to capture false interactions, *in vivo* yeast two-hybrid (Y2H) is likely biased towards non-specific interactions^3^ and gene co-expression that could induce synthetic lethality is not efficient to detect pathogen-host protein interactions^4^,^5^. Recent critical assessments of experimentally obtained PPI data suggest that these data exhibit an unacceptably high fraction of false positives and low agreement between each other^6^,^7^,^8^. Meanwhile, computational methods also takes the risk of high false discovery rate for the following reasons. Firstly, the experimentally identified PPI data are likely to contain a certain level of noise (false interactions). Secondly, the negative data needed for two-class PPI prediction are usually obtained by random sampling^9^,^10^,^11^,^12^,^13^,^14^,^15^, which may introduce considerable false negative. Thirdly, model selection is generally conducted by cross validation on the training PPI data, and the trained models, if used for proteome-wide predictions, are prone to overpredictions. For pathogen-host PPI prediction, these issues become worse because the training data available are much smaller and less representative. Thus the intra-species models^12^,^13^,^14^,^15^,^16^,^17^,^18^,^19^,^20^ are likely to yield more false positive predictions than the inter-species PPI prediction models^9^,^10^,^11^.

At present the negative data required for computational reconstruction of PPI networks are in general not available. Recently some negative data from biological experiments have been collected into database, e.g. the reference set of negatome^21^, but the negative data are not enough train a two-class classifier. To meet the need of computational modeling, random sampling is often used to generate negative data^9^,^10^,^11^,^12^,^13^,^14^,^15^. The assumption behind random sampling is that the non-interactome space is much larger than the interactome space, so that random sampling could hit the non-interactome space with a large probability to sample true negatives (non-interactions). However, random sampling is supposed to introduce uncertainty and complexity to the model behaviour, simple as it is. There are several major factors that affect model performance, such as the learning algorithm, feature construction method and the data quality. The uncertainty introduced by random sampling makes it hard to discriminate which factor leads to the poor model performance. For instance, Yu et al.^22^ cast a doubt on the PPI predictive ability of simple sequence *k*-mer feature construction, while Park et al.^23^ argued that it was not the *k*-mer feature construction but the random sampling method that resulted in poor model performance. No matter whether the arguments catch the point, the quality of negative data is undoubtedly critical to the model performance. To obtain reliable negative data, Ben-Hur et al.^24^ proposed to exclude those subcellular co-localized proteins, and Mei^25^ further showed that exclusiveness of subcellular co-localized proteins outperformed random sampling without introducing predictive bias. Intuitively, the negative data obtained by excluding those subcellular co-localized proteins seem to be more reliable but less representative, because the negative data do not represent the proteins pairs that are subcellular co-localized but do not interact. To make a detour around negative data sampling, one-class learning/clustering methods have been proposed for PPI prediction, e.g. association rule mining^17^, one-class SVM^26^,^27^, ensemble non-negative matrix factorization based clustering^28^, etc. These methods, though much simplified, are more likely to yield a large fraction of false positive predictions, because they do not learn the negative (non-interaction) patterns. A wise choice is not to evade negative data sampling but to properly ensure that the obtained negative data are reliable and representative.

Model selection is a second critical concern of computational modeling for PPI prediction. Most of the existing methods generally conduct model selection by optimizing model parameters and empirically tuning hyper-parameters merely on the training data^9^,^10^,^11^,^12^,^13^,^14^,^15^,^16^,^17^,^18^,^19^. The assumption behind the practice is that a model optimally trained on the training PPI data can generalize well to the gigantic unseen space of protein pairs. This assumption does not always hold true, especially when the training PPI data is rather small. To gain knowledge about the quality of model selection, one simple and natural method is to use the model to predict all possible (proteome-wide) or a large percentage of protein pairs, and then check the false positives. However, lack of experimental evidences makes it hard for us to determine the false positive rate. Nevertheless, the rationality of the predictions still can be estimated through the predicted positive rate. Jansen et al.^2^ has estimated that the expected number of negatives (non-interacting protein pairs) is several orders of magnitude higher than the number of positives (interacting protein pairs). This estimation can be used to check the quality of model selection. If the predicted positives account for a large percentage of the proteome-wide protein pairs (e.g. >50%), we can infer that the predictions go against the estimation in ref 2 and thus there is a large fraction of false positive predictions. Moreover, large predicted positive rate contradicts with the assumption of large negative (small positive) space behind random sampling. If the model is trained on the negative data sampled by random sampling (small positive space) and the model yields a large percentage of positives (large positive space), we can see an obvious paradox between the assumption of random sampling and its outcome. After checking the outcomes of the random forest method^18^, we find that the 25 *Salmonella* proteins are predicted to interact with 22,651 human proteins (nearly all known human proteins), indicating a certain degree of overprediction. We can see that it is necessary to analyse the proteome-wide predictions and impose rational constraints on model selection. For large-scale intra-species PPI prediction, the computation of model selection will be daunting, but the computation is acceptable for pathogen-host PPI prediction.

Feature construction is a third important concern of computational modeling for PPI prediction. As compared to intra-species PPI networks reconstruction (e.g. yeast PPI network^9^, *Arabidopsis thaliana* PPI network^10^, human PPI network^11^, etc.), inter-species pathogen-host PPI networks reconstruction is more challenging in that the pathogen-host PPI data available is generally much smaller. To improve the model performance, most of the existing methods generally leverage a catalog of biological feature information, e.g. binding motif, gene expression profile, gene co-expression, gene ontology, sequence *k*-mer, post-translational modification, protein structural information and PPI network topology^12^,^13^,^14^,^29^,^30^, etc. Among these types of feature information, the sequence information of protein achieves relatively moderate discriminative ability^22^,^23^, though less expensive to obtain. Tastan et al.^12^ has claimed that gene ontology (*GO*) is one of the strongest indicators for host-pathogen PPI prediction when combined with other feature information. Moreover, gene ontology alone has been reported to achieve satisfactory performance for pathogen-host PPI prediction^25^ and intra-species PPI prediction^29^. In spite of strong discriminative ability, non-sequence information (e.g. gene ontology, spatial structural information, gene co-expression, etc.) has the drawback that the feature information is generally not complete. To overcome the drawback, proper substitution of incomplete feature information has been deliberately proposed^18^,^25^.

In this work, we address the two concerns of negative data sampling and rational constraints on model selection to reliably reconstruct the proteome-wide protein interaction networks between HTLV retrovirus and Homo sapiens. We use one-class SVM to sample reliable and representative negative examples, and use two-class SVM proteome-wide predictive feedback as constraints on one-class SVM model selection. Reliability demands that the negative examples are distributed far away from the positive examples with low risk of false negatives, and representativeness demands that the negative examples supporting two-class decision boundary should be near to the positive examples so as to reduce the risk of false positives. The two seemingly opposite requirements suggest that a proper negative data sampling method should achieve good trade-off between reliability and representativeness. Here we propose two-class SVM proteome-wide predictive feedback to guide the search of one-class SVM hyperparameter space, such that the constrained model selection reduces the risk of false positive predictions. As for feature construction, we use gene ontology (*GO*) here to represent proteins in view of its strong discriminative ability of PPI prediction. To enrich *GO* feature information and make up for totally unannotated proteins, we conduct homolog knowledge transfer via independent homolog instances as reported in^31^. Lastly, we conduct gene ontology based clustering analysis of the predicted HTLV-human PPI networks to provide valuable cues for understanding the pathogenesis of HTLV retrovirus.

## Methods

### Data

Human T-cell lymphotropic viruses (HTLV) belong to the family of retroviruses. The type 1 HTLV virus (HTLV-1) can induce Adult T-cell Leukemia/Lymphoma and the type 2 HTLV virus (HTLV-2) does not show known pathogenesis, though closely related to HTLV-1^31^. Simonis et al.^32^ used high-throughput yeast-two-hybrid (HT-Y2H)^33^,^34^ to identify 166 interactions between HTLV and human proteins. There are only three interactions related to HTLV-1 Tax (Nup62, MAD1L1, Cdc23) that overlap with the 145 interactions from VirusMINT^35^ and VirHostNet^36^, accounting for 2.1% recognition rate.

For the convenience of reference, we call *S*1*_pos_* the data from^32^ and *S*2*_pos_* the data from^35^,^36^. Additionally, we call *S*3*_pos_* the data from^37^. We check the three datasets against UniprotKB database (http://www.uniprot.org/uniprot/), and remove those putative HTLV proteins and those HTLV proteins that have no corresponding accessions in Swissprot database (manually annotated and reviewed part of UniprotKB). After filtration, *S*1*_pos_* is reduced to 155 interactions, *S*2*_pos_* is reduced to 144 interactions and *S*3*_pos_* contains the HTLV protein p30 only with 42 interactions. We call *S_pos_* (*S_pos_* = *S*1*_pos_* ∪ *S*2*_pos_* ∪ *S*3*_pos_*) the union of the three dataset, and thus *S_pos_* contains 341 interactions. We sample the equal number of negative data for each HTLV protein in *S_pos_* and thus obtain the corresponding negative data *S_neg_* The union of *S_pos_* and *S_neg_*, called *S* (*S* = *S_pos_* ∪ *S_neg_*) is used to train two-class SVM for proteome-wide HTLV-human PPI networks reconstruction. To stringently demonstrate the model performance, we also use *S*1*_pos_* and *S*2*_pos_* as mutual independent test data and use *S*3*_pos_* as literature validation.

### *GO* feature construction

Gene ontology (*GO*) is used as indicator of HTLV-human PPI prediction and *GO* feature construction is conducted as^31^. The homolog *GO* knowledge is treated as independent instance (called homolog instance) to augment the target instance (the *GO* information of the proteins themselves). The homologs are extracted from SwissProt 57.3 database^38^ using PSI-Blast with default *E-value* = 10^39^ against all species, and the *GO* terms are extracted from GOA database^40^. For each protein *i*, there are two sets of *GO* terms, one set denoted as homolog set 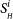 contains the *GO* terms from the homologs, and the other set denoted as target set 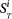 contains the *GO* terms from the protein itself. Based on the denotations, we can formally define two feature vectors for each protein pair (*i*_1_, *i*_2_) as follows:
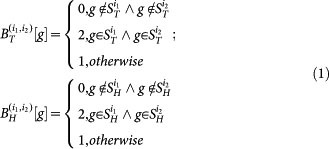
where 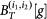 denotes component *g* of the target instance 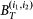 and 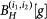 denotes component *g* of the homolog instance 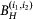. Formula (1) means that if the protein pair (*i*_1_, *i*_2_) shares the same *GO* term *g*, then the corresponding component in the feature vector 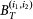 or 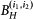 is set 2; if neither protein in the protein pair possesses the *GO* term *g*, then the component is set 0; otherwise the component is set 1. The above definition is symmetrical, so that protein pair (*i*_1_, *i*_2_) and protein pair (*i*_2_, *i*_1_) have identical feature representation. If either set of *GO* terms is empty, the feature vector is defined as null and should be removed:
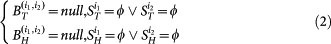


### One-class SVM based negative data sampling

One-class SVM was originally proposed for estimating the support of a high-dimensional distribution^41^ and detecting novelty/outlier^42^. Unlike two-class classification, one-class SVM attempts to derive from the positive data alone one decision boundary, one side of which is positive and the other side is outlier. The decision boundary can be assumed as a hyperplane^41^,^42^ or a hypersphere^43^. The assumption of hyperplane is to map the data into a kernel space so as to construct a hyperplane that is maximally distant from the origin. Given the training vectors *x_i_* ∈ *R^n^*, *i* = 1, 2, …, *l* that possess positive labels only, the primal problem of one-class SVM is formally defined as the following quadratic program^42^:
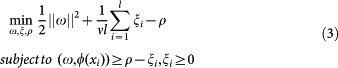
where *ν* ∈ (0,1) controls the upper bound on the fraction of outliers and the lower bound on the fraction of support vectors. *ξ_i_* is slack variable, *ρ* denotes offset, *ϕ*(*x_i_*) is mapping function and *ω* is instance weight. The prime problem (2) corresponds to the following dual problem^42^:
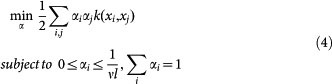


After the coefficients of the support vectors (*α_i_* > 0) are obtained, the decision function is then defined as follows:
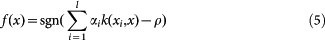
where the kernel function *k*(*x*, *y*) is defined as the inner product of two mapping functions, i.e. *k*(*x*, *y*) = (*ϕ*(*x*)·*ϕ*(*y*)), for instance, *Gaussian* kernel assumes the form:
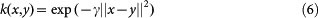
where ||Δ|| denotes 2-norm of vector Δ and the hyperparameter *γ* controls the flexibility of kernel.

One-class SVM is originally developed to learn the patterns inherent in the positive data and then use the patterns to discriminative outliers from the positive data^42^. Recently, one-class SVM has been used as two-class classification^26^,^27^ to avoid negative data sampling, the idea behind which is that the negative class is actually treated equally as the positive outliers. Unfortunately, the negative data generally do not share similar patterns with the positive outliers and one-class SVM can not properly define the two-class decision boundary without learning the negative patterns. Here we use one-class SVM instead to roughly confine the positive (+) region that contains the positive data and then sample negative data outside the region. The question is how much the space of the positive (+) region should be. For the convenience of description, we denote as positive (+) region the opposite side of the hyperplane from the origin, and accordingly negative (-) region the other side of the hyperplane. The more distant the hyperplane is from the origin, the larger the positive (+) region will be. In this case, the space of the negative (-) region is reduced and the sampling in this space is supposed to be more reliable, but the positive (+) region is supposed to contain more errors (outliers and false positives). On the contrary, if the hyperplane is nearer to the origin, the positive (+) region is reduced and the the negative (-) region is supposed to contain more false negatives. In a word, the dilemma is that we should choose the hyperplane far away from the origin or near to the origin, or to say, choose reliable negative data with high false positive rate or choose reliable positive data with high false negative rate. The dilemma, though theoretically unresolved^42^, can be effectively solved by empirically tuning the parameter *ν* ∈ (0,1). One simple method is to define a series of parameter *ν* ∈ (0,1) values to control the space of the positive (+) region. For each parameter *ν* ∈ (0,1) value, together with the kernel parameter *γ*, we train a one-class SVM model to predict proteome-wide HTLV-human protein pairs and then choose a portion of reliable and representative negative data from the negative outcomes (predicted non-interactions). To achieve a proper trade off between reliability and representativeness, we choose the predicted negatives that are centered around the negative outcomes, too far or too near negatives are discarded. Assuming there are *n* predicted *negative* data with outcomes *R_i_* < 0, *i* = 1, …, *n*, the mean and standard variance of the outcomes are defined as follows:
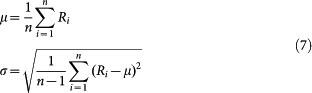
Then the negative data are chosen within the following data indices:
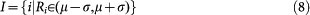
To reduce the risk of model bias, the size of the chosen negative data is equal to the size of positive data (assuming *N*). We further choose the negative data within the indices defined by formula (7) with large outcome values.
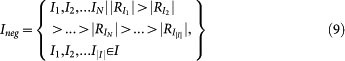
where |*I*| denotes the cardinality of set *I*. Using the above described negative sampling method, we obtain the corresponding negative data for *S*1*_pos_*, *S*2*_pos_* and *S*3*_pos_*, denoted as *S*1*_neg_*, *S*2*_neg_* and *S*3*_neg_*, respectively. Then the three datasets for two-class SVM training are defined as *S*1 = *S*1*_pos_* ∪ *S*1*_neg_*, *S*2 = *S*2*_pos_* ∪ *S*2*_neg_* and *S*3 = *S*3*_pos_* ∪ *S*3*_neg_*. The final training data for proteome-wide HTLV-human PPI prediction is defined as follows:
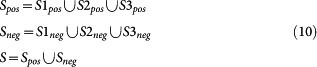


### Two-class SVM prediction

For each parameter pair (*ν*, *γ*), one-class SVM yields one negative dataset *S_neg_*, based on which we train a two-class SVM for novel HTLV-human PPI prediction. Unlike one-class SVM, two-class SVM attempts to maximize the margin between two-class hyperplanes. The prime problem of two-class SVM is defined as follows^44^:
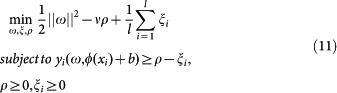
where *y_i_* denotes the class label of data point *x_i_*, the parameter *ν* achieves trade-off between the upper bound on the fraction of training errors and the lower bound of the fraction of support vectors. The parameter *ν* of one-class SVM affects the quality of sampled negative data while the parameter *ν* of two-class SVM affects the generalization ability of two-class predictive model. Comparing formula (3) with formula (11), we can see that two-class SVM needs the information of data label but one-class SVM does not. The prime problem of formula (11) is converted to the following the dual problem:
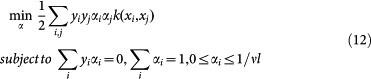
Solving the optimization problem, we can obtain the coefficients of the support vectors (*α_i_* > 0) and further define the decision function as follows:
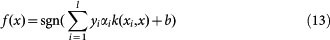
Like one-class SVM, two-class SVM also has one parameter pair (*ν*, *γ*) to be empirically tuned on the training data (*γ* denotes *Gaussian* kernel parameter). Here leave-one-out cross validation (LOOCV) is used to tune the parameter pair (*ν*, *γ*). After parameter tuning, the trained two-class SVM is used to predict proteome-wide HTLV-human protein pairs. As described in formula (1) and formula (2), each test protein pair (*i*_1_, *i*_2_) is represented by the target instance 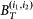 and the homolog instance 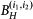, thus two-class SVM decision function *f* yields two outputs for the two instances 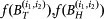. The final decision value for protein pair (*i*_1_, *i*_2_) is defined as follows:
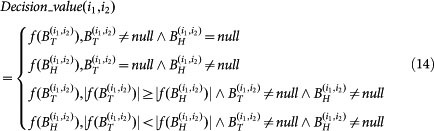
where |·| denotes the absolute value, and then the final label for protein pair (*i*_1_, *i*_2_) is defined as follows:
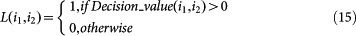


### Proteome-wide predictive feedback constrained model selection

A series of one-class SVM parameter pair (*ν*, *γ*) values yield a series of candidate negative data *S_neg_*. The question is how to determine the quality of the negative data. The common practice is to conduct model evaluation by *k*-fold cross validation or leave-one-out cross validation (LOOCV) on the training data *S* = *S_pos_* ∪ *S_neg_*, and then choose the negative data *S_neg_* that achieves the best model performance. However, cross validation model evaluation on the training data is not enough to demonstrate the true generalization ability. A model that behaves well on the training data is still likely to yield overpredictions like the random forest method for pathogen-host PPI prediction^18^. The rationality of the predictions should be very necessarily verified. Jansen et al.^2^ has proposed a doctrine that the expected number of negatives (non-interacting protein pairs) is several orders of magnitude higher than the number of positives (interacting protein pairs). The doctrine can be used for us to check the rationality of proteome-wide predictions. Assuming there are *p* protein pairs to be predicted, *p*_1_ pairs are predicted as positive (interactions) and *p*_2_ pairs are predicted as negative (non-interactions) (*p* = *p*_1_ + *p*_2_), the model can be accepted only if the following rule is observed:
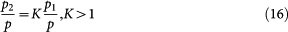
Otherwise, there is a high risk of false positive predictions. Here we use formula (16) as constraint on the model selection of one-class SVM. The parameter pair (*ν*, *γ*) of with larger *K* and good two-class SVM LOOCV performance is preferred.

Two-class SVM LOOCV performance is estimated with multiple performance metrics, such as ROC-AUC (Receiver Operating Characteristic - Area Under Curve), PR-AUC (Precision recall curve AUC), SP (Specificity), SE (Sensitivity) and MCC (Matthews correlation coefficient). SP, SE and MCC can be derived confusion matrix *M*. Formula (17) defines several intermediate variables, from which we can calculate SP_l_, SE_l_ and MCC_l_ for each label as formula (18), and calculate overall MCC as formula (19).
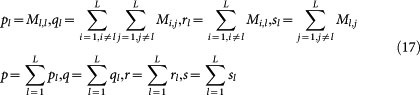

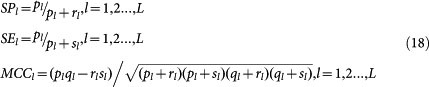

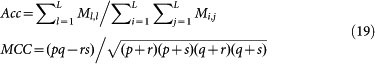
where the confusion matrix *M_i_*_,*j*_ records the counts that class *i* are classified to class *j*, and *L* denotes the number of labels. AUC is calculated based on the decision values of two-class SVM.

## Results

### Proteome-wide negative data sampling

#### One-class SVM parameter pair (ν, γ) tuning

**Figure 1:**
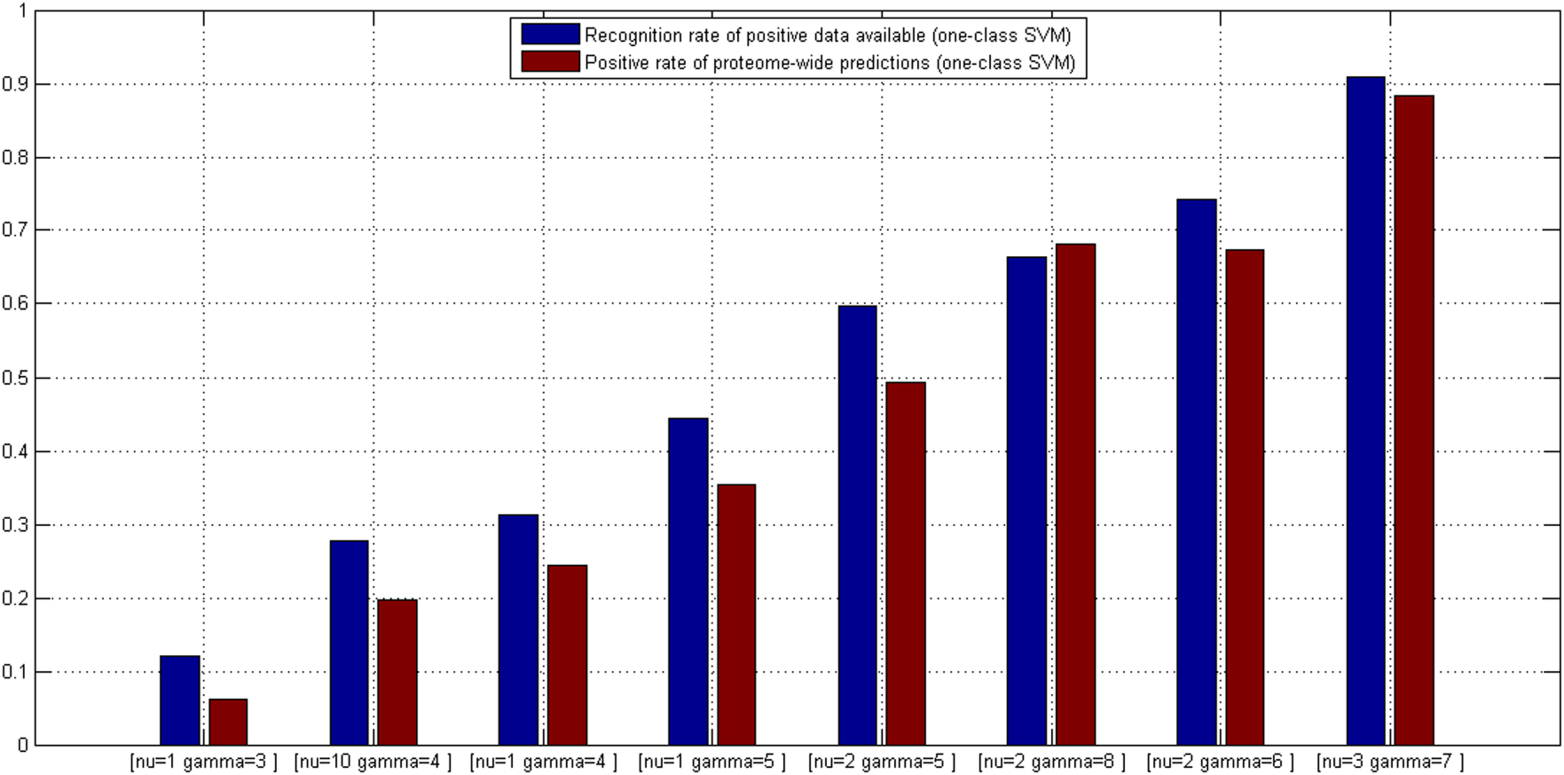
One-class SVM *OCSVM_(ν, γ)_* parameters tuning. Eight representative parameter pairs *(ν, γ)* are chosen from the parameter space according to *OCSVM_(ν, γ)_* LOOCV performance. The blue bars illustrate the recognition rate of the training positive data and the brown bars illustrate the predicted positive rate of proteome-wide predictions by *OCSVM_(ν, γ)_*.

The search space of one-class SVM parameter pair (*ν*, *γ*) is daunting. To reduce the computational complexity, we narrow down the space of *ν* and *γ* to the set {2*^m^* | − 11 ≤ *m* ≤ −1, *m* ∈ *Z*}. For simplicity of annotations, the set is sorted in a descending order, and we use *ν* and *γ* to denote the index of the set elements. *ν* = *i* denotes that *ν* assumes the value 2^−*i*^, *γ* = *j* denotes that *γ* assumes the value 2^−*j*^. The two parameters are empirically tuned by leave-one-out cross validation on the positive data *S_pos_* (*S_pos_* = *S*1*_pos_* ∪ *S*2*_pos_* ∪ *S*3*_pos_*). Each parameter pair (*ν*, *γ*) value trains one one-class SVM model (denoted as *OCSVM*_(*ν*, *γ*)_) and *OCSVM*_(*ν*, *γ*)_ yields corresponding LOOCV performance, e.g. recognition rate of the known PPIs. We split all the achieved LOOCV performances into eight ranges (0.1, 0.2), (0.2, 0.3), (0.3, 0.4), (0.4, 0.5), (0.5, 0.6), (0.6, 0.7), (0.7, 0.8) and (0.9, 1). The range (0.8, 0.9) is omitted because no LOOCV performance falls into the range. In general, more than one *OCSVM*_(*ν*, *γ*)_ achieves equivalent LOOCV performance, i.e their LOOCV performances fall in the same range. For instance, *OCSVM*_(*ν* = 1, *γ* = 3)_ achieves 11.99% recognition rate, *OCSVM*_(*ν* = 10, *γ* = 3)_ achieves 14.62% recognition rate and *OCSVM*_(*ν* = 8, *γ* = 3)_ achieves 11.70% recognition rate, all of which fall in the same range (0.1, 0.2). For each range, we randomly select only one *OCSVM*_(*ν*, *γ*)_ as representative, e.g. *OCSVM*_(*ν* = 1, *γ* = 3)_ for the range (0.1, 0.2), and treat the corresponding (*ν*, *γ*) as *representative parameter pair* (*ν*, *γ*). Thus we choose total eight representative parameter pairs (*ν*, *γ*) as illustrated in Figure 1. The eight representative parameter pairs (*ν*, *γ*) are arranged in the order of ascending recognition rate (see dark blue bars in Figure 1). The *OCSVM*_(*ν*, *γ*)_ that achieves higher recognition rate is supposed to yield smaller negative (-) region, implying that sampling negative data in this region will be more reliable but less representative. After obtaining the eight trained *OCSVM*_(*ν*, *γ*)_ models, we then use *OCSVM*_(*ν*, *γ*)_ to conduct proteome-wide negative data sampling.

#### Negative data sampling from OCSVM_(ν, γ)_ predicted negatives

*N*ow we use the trained *OCSVM*_(*ν*, *γ*)_ models to predict all unseen HTLV-human protein pairs, and then obtain eight negative datasets from the predicted negatives according to formula (7–9). There are 10 HTLV proteins in the training data *S_pos_* and the human proteins are taken from Swissprot database^38^. After excluding those known HTLV-targeted human proteins in *S_pos_* and those protein pairs (*i*_1_, *i*_2_) that satisfy 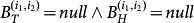, we obtain the whole search space for each HTLV protein as shown in Table 1. The predicted positive rates yielded by the eight trained *OCSVM*_(*ν*, *γ*)_ models are illustrated with brown bars in Figure 1. From Figure 1, we can see that the better LOOCV performance (recognition rate of positive data, bars in brown) *OCSVM*_(*ν*, *γ*)_ achieves, the more protein pairs are predicted to be positive (bars in dark blue). Moreover, with the increase of LOOCV performance, the ratio of predicted negative rate to predicted positive rate decreases to be less than 1 (see the latter four representative parameter pairs (*ν*, *γ*)), which does not observe the rule (*K* > 1) defined in formula (16). For instance, *OCSVM*_(*ν* = 3, *γ* = 7)_ achieves 88.35% predicted positive rate (bar in brown), which is far beyond rational scope. If we choose *OCSVM*_(*ν* = 3, *γ* = 7)_ only because of its 90.94% LOOCV performance (bar in dark blue), we will take the risk of high false positive predictions. Thus it should be cautious to accept a trained one-class SVM only based on its cross validation performance on the training data without examining the rationality of proteome-wide predictions.

In this work, we use one-class SVM to confine negative data sampling. For each representative parameter pairs (*ν*, *γ*), we obtain the negative data 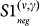, 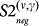, 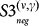 and 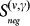 from *OCSVM*_(*ν*, *γ*)_ predicted negatives according to formula (7–9). Based on the sampled negative data, we construct three training datasets 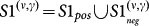, 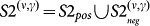, 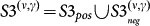 and *S*^(*ν*, *γ*)^ = *S*1^(*ν*, *γ*)^ ∪ *S*2^(*ν*, *γ*)^ ∪ *S*3^(*ν*, *γ*)^ to train and validate two-class SVM *TCSVM*_(*ν*, *γ*)_.

### Proteome-wide predictive feedback constrained model selection

#### Two-class SVM performance evaluation

**Figure 2:**
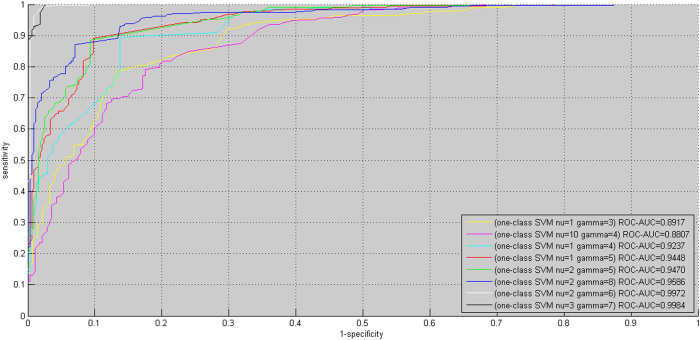
Two-class SVM *TCSVM*_(*ν*, *γ*)_ LOOCV ROC curves. For each representative parameter pair *(ν, γ)*, one negative dataset is sampled from the predicted outcomes of the correponding *OCSVM*_(*ν*, *γ*)_. The sampled negative data are merged with the positive data to train two-class SVM *TCSVM*_(*ν*, *γ*)_. The ROC curves and corresponding AUC scores are used to estimate the quality of the negative data sampled by *OCSVM*_(*ν*, *γ*)_.

**Figure 3:**
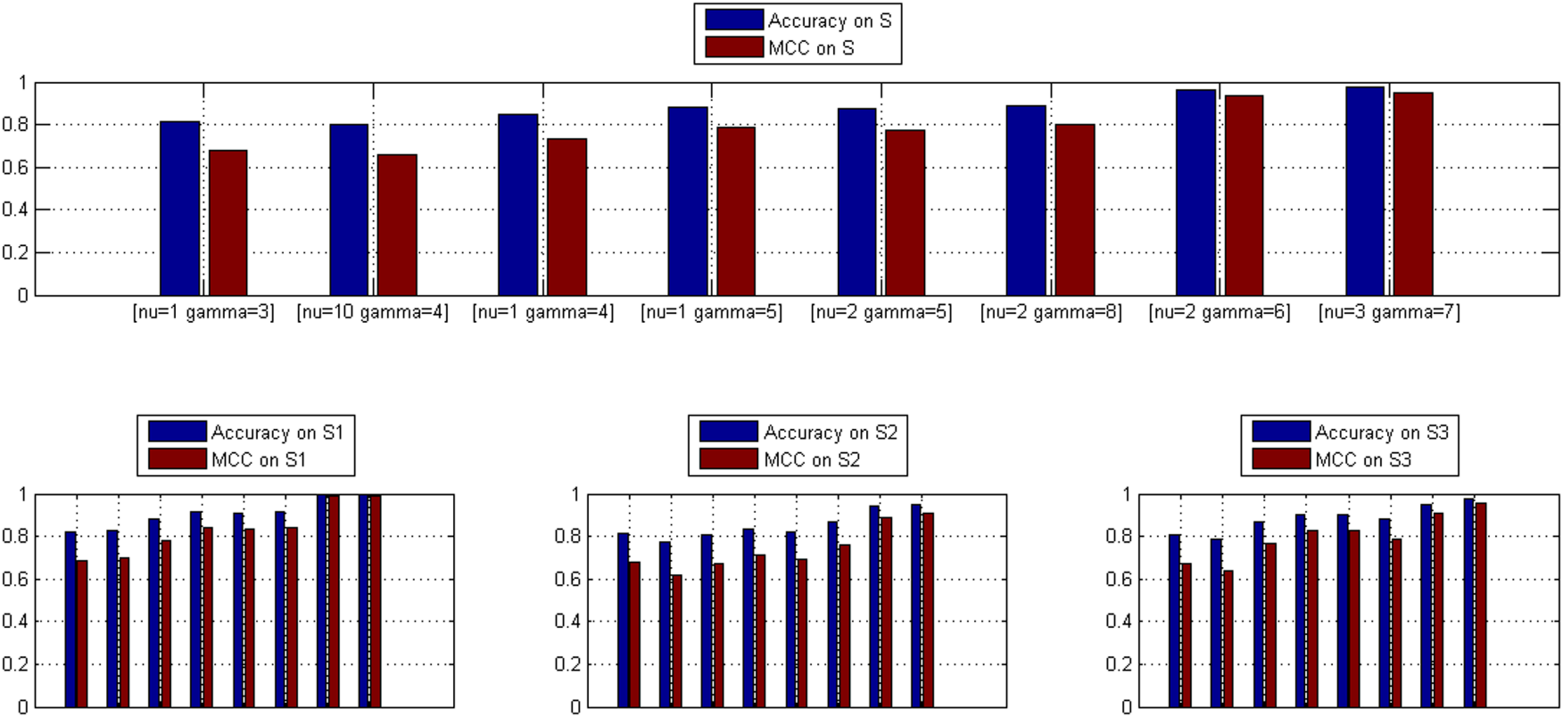
Two-class SVM *TCSVM*_(*ν*, *γ*)_ LOOCV performance on dataset S, S1, S2 and S3. For each representative parameter pair (ν, γ), negative datasets are sampled from the predicted outcomes of the correponding *OCSVM*_(*ν*, *γ*)_ to be the negative data of dataset S, S1, S2 and S3, and then train four two-class SVM *TCSVM*_(*ν*, *γ*)_. The blue bars denote *TCSVM*_(*ν*, *γ*)_ LOOCV Accuracy and the brown bars denote *TCSVM*_(*ν*, *γ*)_ LOOCV MCC.

For each representative parameter pairs (*ν*, *γ*), *OCSVM*_(*ν*, *γ*)_ yields one training data *S*^(*ν*, *γ*)^, based on which we train one two-class SVM denoted as *TCSVM*_(*ν*, *γ*)_. Like one-class SVM, two-class SVM also has two parameters (*ν*, *γ*) to be empirically tuned, denoted as (*ν′*, *γ′*) to be distinguished from one-class SVM parameters (*ν*, *γ*). Here (*ν′*, *γ′*) is tuned by leave-one-out cross validation within the parameter space {2*^m^* | − 5 ≤ *m* ≤ −1, *m* ∈ *Z*}. Since (*ν′*, *γ′*) is trivial to us, (*ν′*, *γ′*) will not be mentioned any more. The LOOCV ROC curves of the eight *TCSVM*_(*ν*, *γ*)_ models are illustrated in Figure 2. From the points of view of AUC scores, the eight *TCSVM*_(*ν*, *γ*)_ models all achieve sound LOOCV performance with the AUC score ≥ 0.8807. Other LOOCV performance metrics (Accuracy, MCC) are shown in Figure 3. The *upper* sub-part plots the bar chart of Accuracy and MCC for *S*^(*ν*, *γ*)^ and the *lower* three sub-parts for *S*1^(*ν*, *γ*)^, *S*2^(*ν*, *γ*)^ and *S*3^(*ν*, *γ*)^. Except the second *TCSVM*_(*ν* = 10, *γ* = 4)_, all the other *TCSVM*_(*ν*, *γ*)_ models achieve >80% Accuracy and >0.68 MCC.

Comparing Figure 1, Figure 2 and Figure 3, we can see that the higher LOOCV performance *OCSVM*_(*ν*, *γ*)_ achieves, the higher LOOCV performance *TCSVM*_(*ν*, *γ*)_ also will achieve on the negative data yielded by *OCSVM*_(*ν*, *γ*)_. The results are not surprising. Higher *OCSVM*_(*ν*, *γ*)_ LOOCV performance suggests that *OCSVM*_(*ν*, *γ*)_ achieves larger positive (+) region and smaller negative (-) region of the hyperplane. Thus the negative data predicted by *OCSVM*_(*ν*, *γ*)_ are more reliable and more easily discriminated from the positive data by *TCSVM*_(*ν*, *γ*)_. However, the negative sampled in smaller negative (-) region of the hyperplane are supposed to be less representative, so that many so-called unreliable negative data will be misclassified to positive class, i.e. false positive predictions or overpredictions. For the reason, the quality of the negative data yielded by *OCSVM*_(*ν*, *γ*)_ should be subjected to further verification by proteome-wide *TCSVM*_(*ν*, *γ*)_ predictive feedback.

#### TCSVM_(ν, γ)_ outcomes constrained model selection

**Figure 4:**
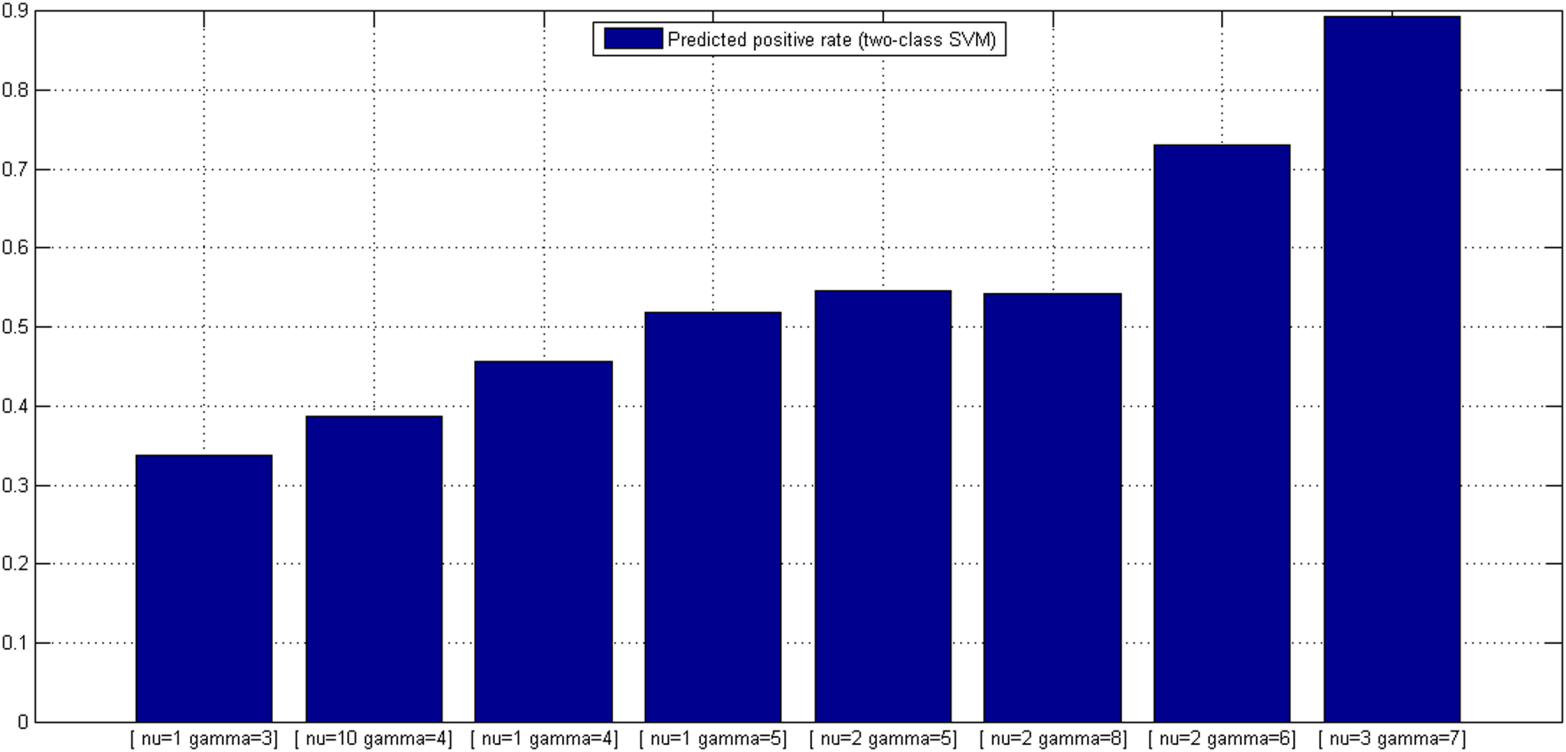
Two-class SVM *TCSVM*_(*ν*, *γ*)_ proteome-wide predicted positive rates. From the predicted posisitve rates, *K* values derived are derived to be used as constraint on *OCSVM*_(*ν*, *γ*)_ model selection. Lower bar signifies higher *K* value.

Similar to *OCSVM*_(*ν*, *γ*)_, the proteome-wide prediction space for each HTLV protein is collected by excluding those human proteins in *S*^(*ν*, *γ*)^ that the HTLV protein interacts with and does not interact with. For most HTLV proteins, the number of human proteins to be predicted is over 20,000, thus there are more than 200,000 protein pairs to be predicted. The predicted positive rates for the eight *TCSVM*_(*ν*, *γ*)_ models are shown in Figure 4. Except the former three *TCSVM*_(*ν*, *γ*)_ models, the latter five *TCSVM*_(*ν*, *γ*)_ from [*ν* = 1, *γ* = 5] to [*ν* = 3, *γ* = 7] all achieve > 50% predicted positive rate with constant *K* less than 1 (*K* is defined in formula (16)), thus out of our options. The first *TCSVM*_(*ν* = 1, *γ* = 3)_ achieves 33.70% predicted positive rate (*K* = 1.97), the second *TCSVM*_(*ν* = 10, *γ* = 4)_ achieves 38.65% predicted positive rate (*K* = 1.59) and the third *TCSVM*_(*ν* = 4, *γ* = 1)_ achieves 45.69% predicted positive rate (*K* = 1.19). The three two-class SVM models, i.e. *TCSVM*_(*ν* = 1, *γ* = 3)_, *TCSVM*_(*ν* = 10, *γ* = 4)_ and *TCSVM*_(*ν* = 1, *γ* = 3)_ should be subjected to further survey for the final model selection.

**Figure 5:**
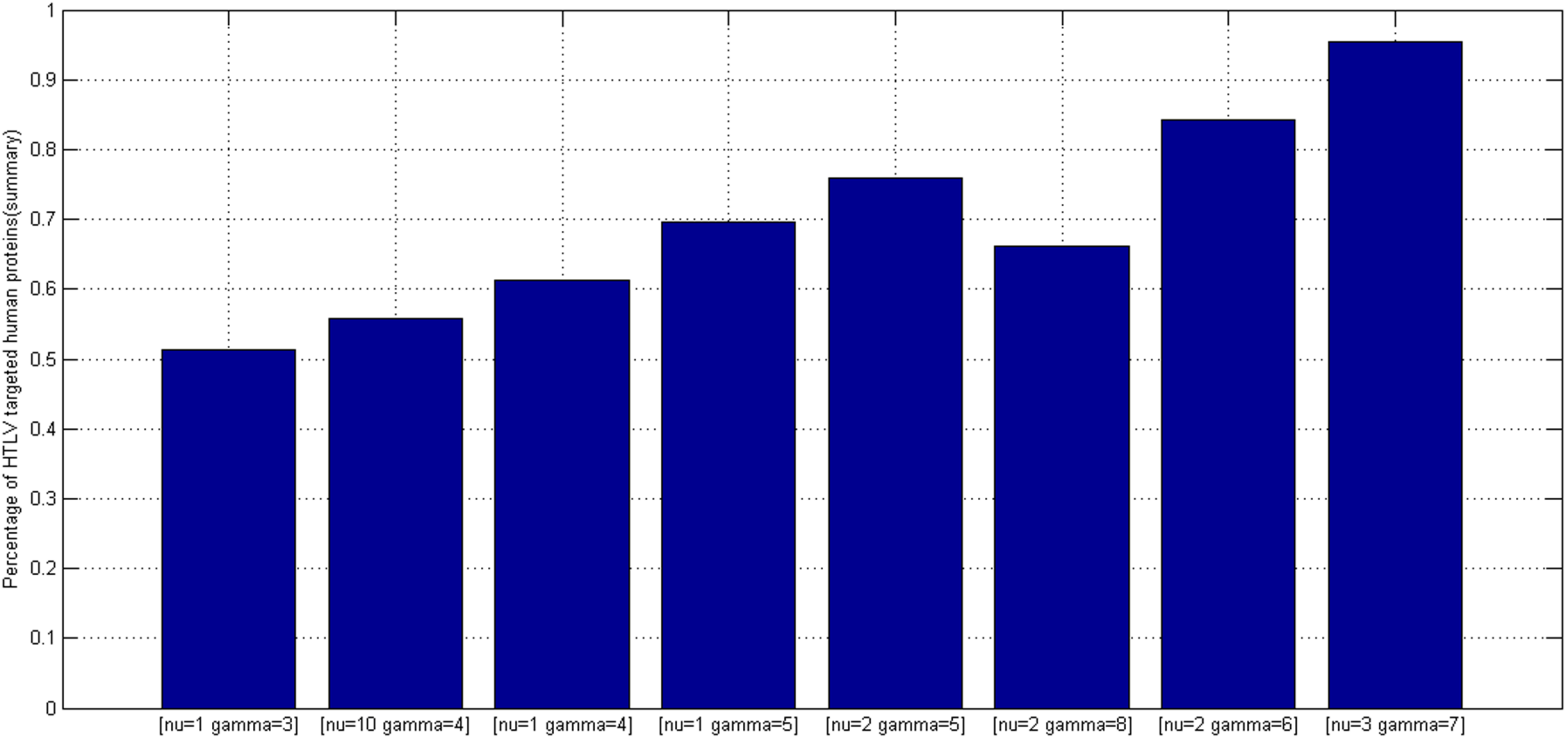
Percentage of HTLV targeted human proteins predicted by two-class SVM *TCSVM*_(*ν*, *γ*)_. Lower bars are supposed to signify lower risk of false positive predictions. The metric together with *K* value is used as constraint on model selection of one-class SVM *OCSVM*_(*ν*, *γ*)_.

Proteome-wide predicted positive rate is an effective metric to validate the rationality of predictions. To choose a proper model from *TCSVM*_(*ν* = 1, *γ* = 3)_, *TCSVM*_(*ν* = 10, *γ* = 4)_ and *TCSVM*_(*ν* = 1, *γ* = 3)_, we further propose the metric *percentage of HTLV-targeted human proteins* for the final model selection (see Figure 5). As shown in Figure 5, the latter six *TCSVM*_(*ν*, *γ*)_ models all predict > 60% human proteins to be targeted by HTLV proteins, the first *TCSVM*_(*ν* = 1, *γ* = 3)_ predicts 51.25% interacting human partners and the second *TCSVM*_(*ν* = 10, *γ* = 4)_ predicts 55.81% interacting human partners. The percentage of predicted human partners seems to be relatively high, partly because the known PPI dataset is small and the sampled negative data are still less representative. However, as compared with the random forest method^18^, which predicted 22,651 human proteins out of 22,654 human proteins to be targeted by *Salmonella* proteins, 51.25% predicted human partners suggest much lower risk of overprediction.

**Figure 6:**
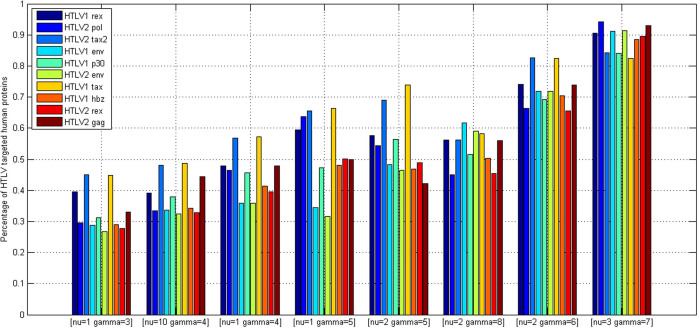
Details of percentage of HTLV targeted human proteins predicted by *TCSVM*_(*ν*, *γ*)_. From the metric, *K* value can be derived for each HTLV protein to conduct fine-grained model selection of one-class SVM *OCSVM*_(*ν*, *γ*)_. The parameter pair *(ν, γ)* with more lower bars are preferred.

To further choose the final model from *TCSVM*_(*ν* = 1, *γ* = 3)_ and *TCSVM*_(*ν* = 10, *γ* = 4)_, we provide in Figure 6 the details of percentage of human partners predicted to be targeted by each HTLV protein. As shown in Figure 6, *TCSVM*_(*ν* = 1, *γ* = 3)_ generally shows lower risk of false positive predictions. Five HTLV proteins (HTLV2 pol, HTLV1 env, HTLV1 hbz, HTLV2 rex) are predicted to be targeted by less than 30% human partners, two HTLV proteins (HTLV1 rex, HTLV2 gag) are predicted to be targeted by over 30% but less than 40% human partners, and two HTLV proteins (HTLV2 tax2, HTLV1 tax) are predicted to be targeted by over 40% but less than 50% human partners. Comparatively, *TCSVM*_(*ν* = 10, *γ* = 4)_ shows a little higher risk of false positive predictions (see Figure 4 ~ Figure 6) and a little decrease of LOOCV performance (see Figure 2 and Figure 3). For the reason, we are inclined to choose *TCSVM*_(*ν* = 3, *γ* = 1)_ as the final predictive model. The details of percentage of human partners predicted by *TCSVM*_(*ν* = 3, *γ* = 1)_ are given in Table 1.

#### Further validation of TCSVM_(ν = 1, γ = 3)_

We have conducted LOOCV model estimation on *TCSVM*_(*ν* = 1, *γ* = 3)_ and analysed the rationality of proteome-wide predictions by *TCSVM*_(*ν* = 1, *γ* = 3)_. To gain knowledge about the generalization ability of *TCSVM*_(*ν* = 3, *γ* = 1)_, we need to further conduct independent test using experimental evidences from recent literature. Because of the scarcity of experimental data, we make full use of three PPI: (1) train a model on *S*1 (denoted as 
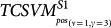
) and validate 
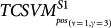
 using *S*2*_pos_*; (2) train a model on *S*2 (denoted as 
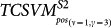
) and validate 
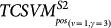
 using *S*1*_pos_*; (3) train a model on *S*1 ∪ *S*2 (denoted as 
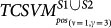
) and validate 
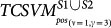
 using *S*3*_pos_*. Before independent tests, we conduct LOOCV estimation on 
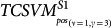
, 
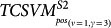
 and 
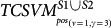
 (see Table 2). The results of independent tests show that 
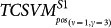
 completely recognizes *S*2*_pos_*, far better than 2.1% recognition rate by HT-Y2H [32]. 
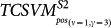
 also completely recognizes *S*1*_pos_*, but 
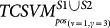
 achieves only 33.33% recognition rate on *S*3*_pos_*. The test data *S*3*_pos_* contains HTLV p30 only and the training data *S*1 ∪ *S*2 does not contain HTLV p30, so it is not surprising that 
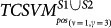
 achieves low recognition rate on *S*3*_pos_*. But the result is still promising as compared to experimental siRNA screens (10% recognition rate)^13^.

#### Comparison with random sampling

**Figure 7:**
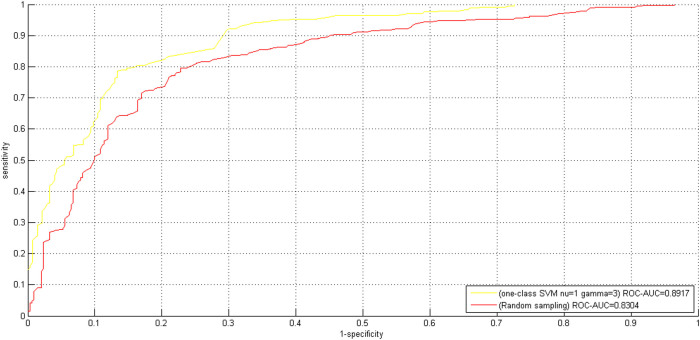
Comparative ROC curves between the final model *TCSVM*_(*ν = 1*, *γ = 3*)_ and random sampling model *TCSVM_random_*. From the points of view of AUC scores, *TCSVM*_(*ν* = 1, *γ* = 3)_ outperforms *TCSVM_random_*.

Random sampling is simple and unbiased, but is prone to be less reliable and less representative. For comparison, a negative data 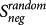 with equal size to the positive data *S_pos_* is obtained using random sampling. Then we train a two-class SVM denoted as *TCSVM_random_* on the data 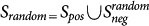. The comparative LOOCV ROC curves between *TCSVM*_(*ν* = 1, *γ* = 3)_ and *TCSVM_random_* are shown in Figure 7. We can see that *TCSVM*_(*ν* = 1, *γ* = 3)_ performs better than *TCSVM_random_* with AUC score 0.8917 versus 0.8304. *TCSVM*_(*ν* = 1, *γ* = 3)_ also shows better LOOCV performance than *TCSVM_random_* with (Accuracy = 0.8158, MCC = 0.6812) versus (Accuracy = 0.7778, MCC = 0.6239). In addition, we also conduct proteome-wide predictions using *TCSVM_random_*. The computational results show that *TCSVM_random_* achieves 24.97% proteome-wide predicted positive rate, relatively lower than *TCSVM*_(*ν* = 1, *γ* = 3)_ (33.70%), suggesting a relatively lower risk of false positive predictions. *TCSVM_random_* achieves 3.00 *K* value, higher than *TCSVM*_(*ν* = 1, *γ* = 3)_ *K* value (1.97). The *K* value defined in formula (16) is proposed to roughly estimate the rationality of predictions. In general, low *K* value (≤1) suggests a high risk of false positive predictions, which can be used as constraint on model selection. It is hard to accurately define the upper bound and the lower bound of *K* value, high *K* value does not always imply good model. Too high *K* value may suggest high false negative rate and insufficiency of model predictive ability. We should obtain a proper trade-off between proteome-wide prediction based *K* value, training data based cross validation performance and literature evidence based independent test performance. Here we might as well choose *TCSVM*_(*ν* = 1, *γ* = 3)_ for the reasons: (1) *TCSVM*_(*ν* = 1, *γ* = 3)_ achieves better LOOCV performance; (2) *TCSVM*_(*ν* = 1, *γ* = 3)_ confines the space of negative data sampling, thus the obtained negative data are more reliable and representative; (3) *TCSVM*_(*ν* = 1, *γ* = 3)_ and *OCSVM*_(*ν* = 1, *γ* = 3)_attempts to achieve a proper trade-off between false positives and false negatives.

### Proteome-wide HTLV-human PPI networks reconstruction

#### PPI networks reconstruction

As described above, *TCSVM*_(*ν* = 1, *γ* = 3)_ that is trained on the constructed data *S*^(*ν* = 1, *γ* = 3)^ is chosen as the final model. The proteome-wide predictions are given in the Supplementary Section 1 (predicted interactions) and Supplementary Section 2 (predicted non-interactions). Among the total 201,969 protein pairs, *TCSVM*_(*ν* = 1, *γ* = 3)_ predicts 68,054 interactions and 133,915 non-interactions with predicted positive rate accounting for 33.70%. If we define *Decision*_*value*(*i*_1_, *i*_2_) > *δ*, *δ* > 0 as positive class and *Decision*_*value*(*i*_1_, *i*_2_) < −*δ*, *δ* > 0 as negative class, e.g. *δ* = 0.1 (*Decision*_*value*(*i*_1_, *i*_2_) see formula (14)), the predicted interactions and the predicted non-interactions will be more reliable with lower risk of false predictions. The rapidly reconstructed HTLV-human PPI networks provide valuable cues for further biomedical research. Gene ontology based clustering analysis of the predicted networks will be discussed in the next section.

#### Literature validation of PPI predictions

*K* value is useful to check the rationality of proteome-wide predictions and literature validation is further needed to check the reliability of proteome-wide predictions. However, the fact that the existing experimental evidences are sparsely scattered over hundreds of biomedical literature makes it hard for us to collect enough data to validate the predictions. Nevertheless, we still manage to find 20 novel experimental PPIs that are correctly recognized by our proposed *TCSVM*_(*ν* = 1, *γ* = 3)_ (see Table 3). The PPIs given in Table 3 have not been collected into the training data *S*^(*ν* = 1, *γ* = 3)^, though some PPIs were found much earlier than^32^. For instances, HTLV1 p30 is found to interact with Cyclin E and CDK2 to affect their complex formation and thus to delay S phase entry^45^. Nakano et al.^46^ proposed that HTLV1 p30 may interact with nucleoporin NUP62 and tumor suppressor LZTS2. HTLV1 tax has been found to interact with NEMO, OPTN, RELB and IKKE^47^ and the interaction between HTLV1 tax and Mdm2 results in the degradation of FoxO4, a transcription factor and tumor suppressor of Akt signaling pathway^48^. In^49^, HTLV1 hbz is reported to directly inhibit the acetyl transferase activity of p300/CBP. In^50^, HTLV1 hbz is reported to interact with SMAD2/3/4. In^51^, HTLV2 tax2 is reported to interact the key component of autophagy pathways BECN1 to connect the IKK complex to autophagy pathways. In^52^, it is reported that the direct interaction between CIITA with Tax2 inhibits the oncogenic retrovirus replication in infected cells. It is hard to manually extract all the related experimental PPIs from so many scattered literature, so we give only dozens of examples as shown in Table 2. The 20 experimental evidences help to validate the reliability of *TCSVM*_(*ν* = 1, *γ* = 3)_ proteome-wide predictions.

The number of experimental direct PPIs is very limited, so we also find some indirect evidences to further validate the reliability of *TCSVM*_(*ν* = 1, *γ* = 3)_ predictions. Taylor et al.^53^ assessed the effect of p30 on cellular RNA transcript expression and their nuclear export, and reported the related down-regulated genes and the up-regulated genes regulated by HTLV1 protein p30. The alteration of the host cellular transcript expression may indicate that there is a direct or functional (indirect) interaction between p30 and the up- or down-regulated genes. Hence we conduct overlap analysis between *TCSVM*_(*ν* = 1, *γ* = 3)_ predictions with the results^53^, and the predictions supported by gene expression are given in Supplementary Section 3 ~ Section 6.

## Discussion

Biological experiments generally focus on positive phenomena such as interaction, binding, modification, activation, expression, response, etc., whereas the corresponding negative phenomena arouse less attentions. Actually the negative phenomena also benefit our understanding of the positive patterns and especially facilitate computational modeling. Because experimental negative data are seldom available, proper negative data sampling method is highly desired to sample reliable and representative negative data. In this work, we use one-class SVM to confine the space of negative data sampling for the sake of reliability and sample the centred negatives (*μ* − *σ*, *μ* + *σ*) for the sake of representativeness. To validate the quality of sampled negative data or to select proper one-class SVM parameter pair (*ν*, *γ*), we calculate the *K* value and the predicted positive rate of two-class SVM proteome-wide predictions, based on which to exert constraints on one-class SVM model selection. The computational results show that the final *OCSVM*_(*ν* = 1, *γ* = 3)_ yields a quality negative data to train the predictive model *TCSVM*_(*ν* = 1, *γ* = 3)_. *TCSVM*_(*ν* = 1, *γ* = 3)_ has been empirically demonstrated to show good LOOCV performance, good independent test performance and rational proteome-wide predictions. Here we further conduct gene ontology based clustering analysis of predicted HTLV-human PPI networks to gain the insight of general patterns that HTLV viruses attack human proteins.

To further validate the sampled negative data, we conduct leave-one-out cross validation (LOOCV) and literature validation. The performance metrics ROC-AUC, SP, SE, Accuracy and MCC demonstrate that the two-class SVM *TCSVM*_(*ν* = 1, *γ* = 3)_ trained on the obtained negative data achieve good LOOCV performance and rational predicted positive rate, yielding low risk of false positive predictions.

Lastly, gene ontology based clustering analysis of the predictions reveals some HTLV-targeted significant signaling pathways and human proteins that fulfil critical molecular functions, which provides much insight into the pathogenesis of HTLV retroviruses. To gain knowledge about how the HTLV proteins interfere with the host signaling pathways, what host cellular functions the HTLV proteins are prone to do harm with, and where the interactions occur, we cluster all the predicted interactions into thee major classes according to GO terms, i.e. biological processes (P), molecular functions (F) and cellular compartments (C). Here we use gene ontology term (GO term) as distance metric, i.e. the human partners that possess the same GO term are assigned to the same cluster. Thus each cluster of human proteins defines a biological module that reveals the general behaviour patterns of HTLV viruses. To distinguish the patterns that all the 10 HTLV proteins observe and the patterns that several HTLV proteins observe, we further split each cluster into two sub-clusters, one sub-cluster embraces all the 10 HTLV viruses (denoted as P1, F1 and C1), and the other sub-cluster embraces only a part of viruses (denoted as P2, F2 and C2). P1, F1 and C1 are given in Supplementary Section 7, Section 8 and Section 9, respectively. P2, F2 and C2 are given in the Supplementary Section 10, Section 11 and Section 12, respectively. For the sake of large number of biological modules (clusters), we only demonstrate several biological modules as examples, interested readers are referred to Supplementary Section 7 ~ Supplementary Section 12 for other biological cues.

### PPI Sub-network GO:0007219 - Notch signaling pathway

**Figure 8:**
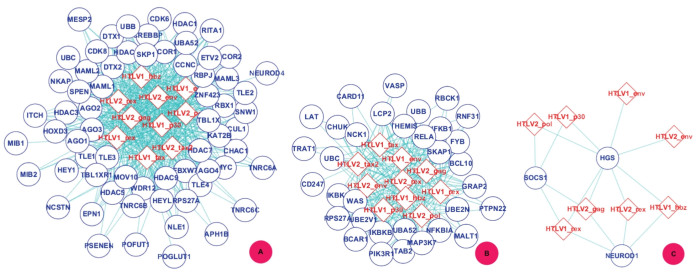
Gene ontology based clustering of predicted PPI subnetworks - biological processes. Three human signaling pathways predicted to be targeted by HTLV proteins are illustrated as examples: 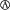 GO:0007219 - Notch signaling pathway. 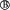 GO:0050852 - T cell receptor signaling pathway. 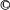 GO:0046426 - negative regulation of JAK-STAT cascade. The diamond denotes HTLV proteins and the ecllipse circle denotes human proteins.

Notch signaling pathway plays an important role in cell proliferation, differentiation and apoptosis. Recent research has suggested that constitutive activation of Notch signaling pathway is essential to the pathogenesis of HTLV-1 associated adult T-cell leukemia (ATL), and the inhibition of Notch signaling by Γ-secretase inhibitors reduces tumor cell proliferation and tumor formation in ATL-engrafted mice^54^. In this work, *TCSVM*_(*ν* = 1, *γ* = 3)_ predicts 545 interactions between the 10 HTLV proteins and 65 human proteins that are involved in Notch signaling pathway. We use the biological processes *GO* term GO:0007219 to denote the predicted PPI sub-network. The PPI sub-network GO:0007219 is extracted from Supplementary Section 7 and is illustrated by 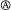 in Figure 8. The HTLV proteins are denoted with diamond and the human protein are denoted with eclipse. From Figure 8, we can see that the 10 HTLV proteins are densely connected with 50 ~ 60 Notch signaling proteins. Interestingly, it is predicted many times that the 10 HTLV proteins simultaneously target the same human protein, i.e. the degree of the human protein is 10 in the PPI Sub-network GO:0007219. In the predicted PPI sub-network, there are 40 human proteins with degree 10 and 10 human proteins with degree 9. In the experimental network *S_pos_*, we also find the phenomena that more than one HTLV proteins target the same human protein. In *S_pos_*, there are 43 human proteins that interact with more than one HTLV protein, e.g. the human protein EWS is targeted by 5 HTLV proteins {HTLV1 rex; HTLV1 tax; HTLV2 gag; HTLV2 rex; HTLV2 tax2}. A human protein that is targeted by multiple HTLV proteins may play a critical role in a protein complex, functional model or signaling pathway. Such the human proteins provide some insight into the pathogenesis and druggable target. For instance, EWSR1 (Q01844), a EWS oncogene, may play a role in the tumorigenic process, causing Ewing sarcoma, a highly malignant, metastatic, primitive small round cell tumor of bone and soft tissue that affects children and adolescents (http://www.uniprot.org/uniprot/Q01844). SKP1 (P63208) is predicted to be targeted by all the 10 HTLV proteins. According to Uniprot annotation (http://www.uniprot.org/uniprot/P63208), SKP1 is an essential component of the SCF (SKP1-CUL1-F-box protein) ubiquitin ligase complex, which mediates the ubiquitination of proteins (e.g. cyclin E, NOTCH1 released notch intracellular domain (NICD)) involved in cell cycle progression (e.g. G1/S, G2/M), signal transduction and transcription. HTLV-1 Tax has been experimentally demonstrated to bind key cell cycle regulators to influence T lymphocyte G1-S progression^32^,^55^,^56^.

### PPI Sub-network GO:0050852 - T cell receptor signaling pathway

Researches in the last decade have demonstrated that there is close connection between HTLV infection and the activation of T cell receptor signaling pathway^57^,^58^. In^57^, it is reported that immune stimuli on T cell receptor signaling pathway may activate HTLV-1 gene expression and cellular gene expression. In^58^, it is stated that HTLV-1 dysregulates common T-cell activation pathways for the virus to establish persistent infection. In this work, *TCSVM*_(*ν* = 1, *γ* = 3)_ predicts 250 interactions between the 10 HTLV proteins and 33 human proteins that are involved in T cell receptor signaling pathway. PPI Sub-network GO:0050852 is extracted from Supplementary Section 7 and is illustrated by 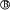 in Figure 8. The predicted PPI sub-network is less densely connected than PPI Sub-network GO:0007219. There are 10 human proteins with degree 10 and 14 human proteins with degree 9. The human proteins targeted by multiple HTLV proteins may also fulfil critical molecular functions. For example, the human protein THMS1 (Q8N1K5) is predicted to be targeted by all the 10 HTLV proteins. According to Uniprot annotations, THMS1 plays a central role in late thymocyte development and regulates T-cell development through T-cell antigen receptor (TCR) signaling (http://www.uniprot.org/uniprot/Q8N1K5).

### PPI Sub-network GO:0046426 - negative regulation of JAK-STAT cascade

JAK-STAT signalling pathway plays a critical role in the transduction of extracellular signals from cytokines and growth factors that are involved in hematopoiesis, immune regulation, fertility, lactation, growth and embryogenesis. Negative regulators of JAK–STAT pathways include tyrosine phosphatases, protein inhibitors of activated STATs, suppressors of cytokine signalling proteins, and cytokine-inducible SH2-containing protein^49^. It has been reported that HTLV-1 Tax protein suppresses apoptosis through constitutive activation of the NFκB pathway, which in turn activates JAK3-STAT5 pathway to cause lymphocyte proliferation and adult T-cell lymphoma/leukemia^59^. In this work, *TCSVM*_(*ν* = 1, *γ* = 3)_ predicts 16 interactions between 8 HTLV proteins and 3 human proteins that are involved in negative regulation of JAK-STAT cascade. It may be inferred that the 8 HTLV proteins repress the 3 negative regulators of JAK-STAT cascade to keep constitutive activation of the JAK-STAT signaling pathway. PPI Sub-network GO:0046426 is extracted from Supplementary Section 10 and is illustrated by 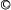 in Figure 8. The predicted sub-network is rather sparsely connected. There is one human protein with degree 8 and two human proteins with degree 4. Two HTLV proteins {HTLV1 tax, HTLV2 tax2} are predicted not to interact with the pathway related human proteins. We only extract only three signaling pathways as illustrated in Figure 8, interested readers are referred to Supplementary Section 7 and Supplementary Section 10 for other signaling pathways or biological processes.

### PPI Sub-network GO:0017124 - SH3 domain binding

**Figure 9:**
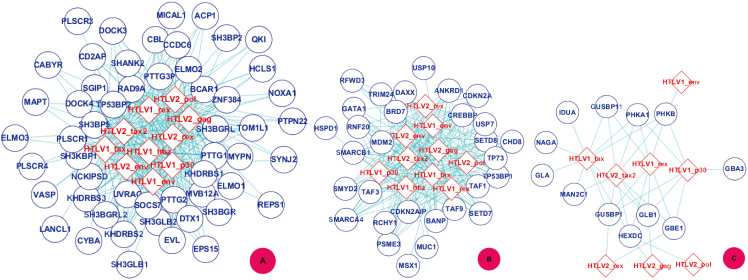
Gene ontology based clustering of predicted PPI subnetworks - molecular functions. Three human Molecular functional modules predicted to be targeted by HTLV proteins are illustrated as examples: 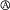 GO:0017124 - SH3 domain binding. 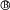 GO:0002039 - p53 binding. 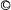 GO:0004553 - hydrolase activity. The diamond denotes HTLV proteins and the ecllipse circle denotes human proteins.

It has been stated that HTLV pathogenesis is closely related to the interaction between HTLV protein and SH3 domain containing proteins^60^. In this work, *TCSVM*_(*ν* = 1, *γ* = 3)_ predicts 343 interactions between the 10 HTLV proteins and 53 SH3 domain binding proteins. It may be inferred that HTLV proteins interrupt the normal functions of the SH3 domain containing proteins by interacting with the corresponding SH3 domain binding proteins. PPI Sub-network GO:0017124 is extracted from Supplementary Section 11 and is illustrated by 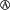 in Figure 9. In the predicted PPI sub-network, there are 15 human proteins with degree 10, 4 human proteins with degree 9 and 8 human proteins with degree 8. The human protein PTTG1 (O95997) predicted to be targeted by the 10 HTLV proteins acts as regulatory protein and plays a central role in chromosome stability, in the p53/TP53 pathway, and in DNA repair. During the mitosis, PTTG1 blocks Separase/ESPL1 function, preventing the proteolysis of the cohesin complex and the subsequent segregation of the chromosomes (http://www.uniprot.org/uniprot/O95997).

### PPI Sub-network GO:0002039 - p53 binding

In^61^, the experimental results suggest that p53 function is inactivated by HTLV Tax protein to induce statistically significant prevalence of tumorigenesis. In^62^, the authors stated that HTLV Tax does not co-immunoprecipitate with p53 and there may be an indirect mechanism to reduce the activity of p53. The assumption is validated in^63^, where it is stated that HTLV-I Tax induces a novel interaction between p65/RelA and p53 to inhibit p53 transcriptional activity. In this work, *TCSVM*_(*ν* = 1, *γ* = 3)_ predicts 238 interactions between the 10 HTLV proteins and 30 p53 binding proteins. The results suggest that interaction with p53 binding proteins is another indirect mechanism to inactivate p53 function. PPI Sub-network GO:0002039 is extracted from Supplementary Section 11 and is illustrated by 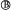 in Figure 9. In the predicted sub-network, there are 17 human proteins with degree 10 and 3 human proteins with degree 8. p53 binding proteins may be indispensible for p53 to be co-complexed for proper transcription activity. For instance, the human protein BRD7 (Q9NPI1) predicted to interact with HTLV proteins is actually a coactivator for TP53-mediated activation of transcription of a set of target genes, and BRD7 is required for TP53-mediated cell-cycle arrest in response to oncogene activation (http://www.uniprot.org/uniprot/Q9NPI1). If HTLV proteins interfere with Q9NPI1 function, there would be much adverse affect on p53 transcription activity.

### PPI Sub-network GO:0004553 - O-glycosyl hydrolase activity

*TCSVM*_(*ν* = 1, *γ* = 3)_ predicts that some HTLV proteins interact with some human proteins fulfilling the function of O-glycosyl hydrolase activity. PPI Sub-network GO:0004553 is extracted from Supplementary Section 10 and is illustrated by 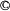 in Figure 9. In the PPI sub-network, there are 37 interactions between 8 HTLV proteins and 12 human proteins. There are 4 human proteins that are targeted by 5 HTLV proteins. For instance, GLB1 (P16278) cleaves beta-linked terminal galactosyl residues from gangliosides, glycoproteins and glycosaminoglycans (http://www.uniprot.org/uniprot/P16278).

## Supplementary Material

Supplementary Information A novel one-class SVM based negative data sampling method for reconstructing proteome-wide HTLV-human protein interaction networks-Mei S, Zhu H

## Figures and Tables

**Table 1:** Statistics of human proteins to be predicted and percentage of predicted interactions for each HTLV protein. The upper part shows the number of protein pairs to be predicted by *OCSVM*_(*ν*, *γ*)_, whose predicted negatives will be sampled as negative data. The lower part shows the predicted positive rate achieved by *TCSVM*_(*ν* = 1, *γ* = 3)_, which is used as constraint on *OCSVM*_(*ν*, *γ*)_ model selection

Statistics of human proteins to be predicted by one-class SVM														
HTLV1 rex	HTLV2 pol	HTLV2 tax2	HTLV1 env	HTLV1 p30	HTLV2 env	HTLV1 tax	HTLV1 hbz		HTLV2 rex		HTLV2 gag		Total	
20,229	20,271	20,151	20,265	20,211	20,280	19,767	20,244		20,274		20,277		201,969	
**Percentage of predicted interactions by two-class SVM (nu = 1, gamma = 3)**														
39.47%	29.67%	44.92%	28.79%	31.17%	26.70%	44.73%		29.01%		27.77%		33.10%		33.70%

**Table 2:** LOOCV performance achieved by *TCSVM*_(*ν* = 1, *γ* = 3)_ on training datasets. The performance metrics are used as a profile to demonstrate the reliability of proteome-wide predictions

	*S*^(*ν* = 1, *γ* = 3)^			*S*1^(*ν* = 1, *γ* = 3)^			*S*2^(*ν* = 1, *γ* = 3)^			*S*3^(*ν* = 1, *γ* = 3)^		
	SP	SE	MCC	SP	SE	MCC	SP	SE	MCC	SP	SE	MCC
**Positive**	0.8775	0.7749	0.6895	0.8986	0.8000	0.6975	0.8538	0.7655	0.6805	0.8824	0.7143	0.6724
**Negative**	0.7571	0.8664	0.6827	0.7156	0.8478	0.6654	0.7848	0.8671	0.6882	0.7600	0.9048	0.6889
**[*Acc; MCC*]**	[0.8158; 0.6812]			[0.8178; 0.6843]			[0.8160, 0.6815]			[0.8095; 0.6716]		

**Table 3:** Predictions validated by recent literature. The square bracketed number that follows the targeted human gene name denotes the literature reference number

HTLV proteins	Targeted human proteins
HTLV1 p30	CDK2[45];LZTS2[46];NUP62[46]
HTLV1 tax	NEMO[47]; OPTN[47]; RELB[47]; IKKE[47]; MDM2[48]; HDAC3[48]
HTLV1 hbz	RELA[48]; CCND1[49]; CBP[50]; SMAD4[50] ; SMAD3[50] ; SMAD2[50] ;
HTLV1 rex	SRSF1[46]
HTLV2 tax2	BECN1[51]; UVRAG[51]; CIITA[52]
HTLV2 gag	WWP1[48]
