# Supplementary material for: A novel one-class SVM based negative data sampling method for reconstructing proteome-wide HTLV-human protein interaction networks
Source: Sci Rep. 2015 Jan 26;5:8034. doi: 10.1038/srep08034 (PMC5379509; doi:10.1038/srep08034)
Supplement: Supplementary Information — A novel one-class SVM based negative data sampling method for reconstructing proteome-wide HTLV-human protein interaction networks-Mei S, Zhu H [file srep08034-s1.pdf]

A novel one-class SVM based negative data sampling method for reconstructing proteome-wide HTLV-human protein interaction networks

Suyu Mei[①②*], Hao Zhu[②§]

[①]Software College, Shenyang Normal University, Shenyang, 110034, China

[②] Bioinformatics Section, School of Biomedical Sciences, Southern Medical University, Guangzhou, 510515, China

Email addresses: [*]meisygle@gmail.com, [§]zhuhao@smu.edu.cn

[SECTION 1] Predicted Interactions

P0C206	Q9UID6	0.67061
P0C206	P27695	0.51734
P0C206	Q8N5I9	0.68336
P0C206	Q9Y536	0.22458
P0C206	Q9BZ01	0.2964
P0C206	P49589	0.47065
P0C206	Q15513	0.45994
P0C206	Q9Y234	0.3439
P0C206	A7MCY6	0.67345
P0C206	O95363	0.57495
P0C206	P33316	0.69159
P0C206	Q02386	0.28307
P0C206	Q96P71	0.64026
P0C206	Q9UBW7	0.85719
P0C206	Q9BUJ2	1.0681
P0C206	Q99990	0.70466
P0C206	O15481	0.027659
P0C206	Q96HA7	0.93145
P0C206	Q8HWS3	0.40281
P0C206	Q8N9K5	0.36383
P0C206	Q8N6G1	0.45994
P0C206	Q13439	1.0269
P0C206	Q96MC4	1.0112
P0C206	Q8TE85	0.34588
P0C206	O95271	0.73353
P0C206	Q5VUA4	0.70389
P0C206	Q76L83	0.71065
P0C206	Q5BJF2	0.43929
P0C206	Q9BPU9	0.30493
P0C206	Q9UNS1	0.68758
P0C206	O75293	0.37461
P0C206	P61289	0.73647
P0C206	Q96AQ1	0.84842
P0C206	Q9H714	0.57011
P0C206	Q96NJ1	0.45994
P0C206	Q8TCI5	0.73614
P0C206	Q53GS7	0.87269
P0C206	Q9NQC7	0.28937
P0C206	O43598	0.26694
P0C206	Q9NUA8	0.27909
P0C206	Q8TF30	0.071289
P0C206	A6NGQ2	0.42903
P0C206	Q7Z3C6	0.41401
P0C206	Q4L180	0.39487
P0C206	O43829	0.87864
P0C206	Q8N720	1.1512
P0C206	Q96G28	0.44707
P0C206	Q6IEG0	0.4779
P0C206	O43679	0.31594
P0C206	O43542	0.74222
P0C206	Q5TG92	0.65844
P0C206	Q96TC7	0.99999
P0C206	Q9NY87	0.74123
P0C206	Q9BRJ6	0.51679
P0C206	O75340	0.48407
P0C206	Q5JTW2	0.52138
P0C206	Q13112	1.5933
P0C206	P06733	0.98293
P0C206	Q8N5N7	0.62111
P0C206	Q8IWA6	0.18518
P0C206	Q6P2C0	0.4657
P0C206	O00488	0.38651
P0C206	Q9Y3S2	0.36636
P0C206	Q9UNN5	0.71115
P0C206	P28698	0.89834
P0C206	Q9H1K1	1.0692
P0C206	Q16763	0.83794
P0C206	Q8IXW0	0.55678
P0C206	Q9P1G2	0.44026
P0C206	Q6NUN9	0.95859
P0C206	Q8WY50	0.45994
P0C206	Q5SW79	1.0399
P0C206	A9UHW6	0.78563

P0C206	Q9BT56	0.37449
P0C206	Q15072	0.41433
P0C206	P59074	0.039144
P0C206	Q86XA9	0.36601
P0C206	Q9NUC0	0.19791
P0C206	P61572	0.80216
P0C206	Q9C000	0.27633
P0C206	Q92688	0.33985
P0C206	Q96EE3	0.67238
P0C206	Q9BQ66	0.72851
P0C206	Q6PJG2	0.93996
P0C206	Q8IW03	0.081664
P0C206	Q9BYR7	1.134
P0C206	O14604	0.69497
P0C206	Q8TC94	0.13938
P0C206	Q9Y253	0.4797
P0C206	Q96A33	0.19594
P0C206	Q9HBM1	0.86714
P0C206	Q6UXU6	1.1632
P0C206	Q6NXT1	0.54531
P0C206	Q86VD1	0.47043
P0C206	O14964	0.8046
P0C206	Q9BRT6	0.35611
P0C206	Q8IYT1	0.53808
P0C206	O60829	0.79463
P0C206	Q9UNP9	0.21623
P0C206	Q9BRG1	1.3028
P0C206	Q9NQC8	0.17507
P0C206	Q16831	0.17485
P0C206	P46937	0.84873
P0C206	Q16851	0.44973
P0C206	Q8WXE1	0.87575
P0C206	Q9Y468	1.0498
P0C206	Q8TAE8	1.0676
P0C206	P41091	0.13888
P0C206	Q8TAG6	0.4246
P0C206	O14645	0.21241
P0C206	O14662	0.65038
P0C206	Q9BV19	0.45122
P0C206	Q15723	1.3012
P0C206	A0JNW5	0.70674
P0C206	Q9Y2X8	0.93071
P0C206	Q9UKC9	0.60052
P0C206	Q6P1L8	0.85532
P0C206	O15511	0.28236
P0C206	O43502	0.41548
P0C206	P0CJ74	0.67101
P0C206	Q9Y5M1	0.19037
P0C206	Q13875	0.6853
P0C206	Q12982	0.30711
P0C206	Q7L2H7	0.68394
P0C206	P09132	0.17852
P0C206	O60645	0.5535
P0C206	Q6L8H2	0.45994
P0C206	Q13887	0.50426
P0C206	Q02833	0.51845
P0C206	P84022	0.57284
P0C206	Q8WXW3	0.79188
P0C206	P0CG35	0.66868
P0C206	Q15287	0.86904
P0C206	Q9BUE0	1.1227
P0C206	Q9NPI8	0.98238
P0C206	Q9UK61	0.64028
P0C206	Q92597	0.68892
P0C206	Q9H6X5	0.028304
P0C206	Q3T8J9	0.81385
P0C206	Q9Y224	1.6016
P0C206	Q6ZVT0	0.012682
P0C206	Q9UJT9	0.57074
P0C206	P53680	0.24304
P0C206	P40429	0.78167
P0C206	Q2LD37	0.18625
P0C206	Q8IYB0	0.45994
P0C206	Q7Z7H3	0.1051
P0C206	Q9UJ94	0.45994
P0C206	Q9BWH6	0.63517

P0C206	O75486	0.71005
P0C206	Q6L8G4	0.45994
P0C206	Q8IVJ8	0.81741
P0C206	Q9NZ63	0.54755
P0C206	Q8IXH6	1.2536
P0C206	K7EIZ4	0.45994
P0C206	Q9H857	0.52334
P0C206	Q9HCU9	1.2682
P0C206	Q9NQX1	0.73127
P0C206	Q969U7	0.69863
P0C206	Q3KNS6	0.2989
P0C206	Q3KP66	1.1831
P0C206	Q8N6Y0	0.75334
P0C206	Q86W11	0.30118
P0C206	Q8NBJ7	0.25999
P0C206	Q3LI73	0.70835
P0C206	Q9UFF9	0.56287
P0C206	Q96F63	0.68336
P0C206	Q9BZQ8	0.69938
P0C206	Q9Y2X9	0.77032
P0C206	P61313	0.8892
P0C206	P63010	0.51511
P0C206	Q12872	0.67104
P0C206	Q8N9T8	1.3412
P0C206	A8MUQ0	1.0377
P0C206	Q9NQG7	0.36962
P0C206	Q5JQC4	0.45994
P0C206	Q96JL9	0.33847
P0C206	Q9H0S4	0.056318
P0C206	O60716	0.56186
P0C206	Q9H8H0	0.32773
P0C206	Q14320	0.078139
P0C206	P53567	0.71762
P0C206	P78358	0.80113
P0C206	Q9P016	0.58204
P0C206	A8K8V0	0.39436
P0C206	Q6EEV4	0.45994
P0C206	Q99436	0.57107
P0C206	Q5PRF9	0.80159
P0C206	Q9UJZ1	0.016446
P0C206	O15182	0.593
P0C206	P28062	0.21946
P0C206	Q9NPA3	1.2846
P0C206	O75477	0.46911
P0C206	Q9BSB4	0.42833
P0C206	P10144	0.41861
P0C206	Q86Y01	0.7533
P0C206	Q9UFN0	0.79252
P0C206	Q8N0V3	0.82504
P0C206	P0C7V9	0.77807
P0C206	Q9BVM2	0.20192
P0C206	Q07687	0.49892
P0C206	Q2T9F4	0.13623
P0C206	Q68DK2	1.156
P0C206	P0CG34	0.66868
P0C206	B2RV13	0.61457
P0C206	Q96ME1	0.19532
P0C206	Q5T035	0.58114
P0C206	Q8NEG4	0.62332
P0C206	P10588	0.35225
P0C206	Q96RS0	0.66212
P0C206	Q9UK12	0.34842
P0C206	Q6UXP7	0.33577
P0C206	Q8NA77	0.81689
P0C206	Q9BV90	0.22007
P0C206	A6NEM1	0.88867
P0C206	Q8N371	0.38891
P0C206	Q6U949	0.45994
P0C206	K7EJ46	0.45994
P0C206	Q96NT1	1.0164
P0C206	Q9Y3A3	0.7485
P0C206	O75683	0.50039
P0C206	Q2YD98	0.33205
P0C206	P05787	1.4502
P0C206	Q6ZUC2	0.32049
P0C206	Q14789	0.34053

P0C206	O43541	0.65635
P0C206	Q5H9J7	0.97135
P0C206	Q9BW71	0.8224
P0C206	Q96AX9	0.29805
P0C206	Q9P2N7	0.23934
P0C206	Q8TAI1	0.40544
P0C206	O60216	0.93424
P0C206	P24863	1.1994
P0C206	P99999	0.755
P0C206	Q9NPE3	0.81368
P0C206	Q6ZTR5	0.27219
P0C206	Q9UNM6	0.13435
P0C206	Q9NX58	0.34631
P0C206	Q96DG6	0.22518
P0C206	P62324	0.77389
P0C206	Q9Y232	0.23072
P0C206	Q6P9A3	0.30987
P0C206	Q92621	1.1527
P0C206	Q9BUL9	1.0131
P0C206	Q8NG57	0.39141
P0C206	Q5SRI9	0.69489
P0C206	Q9BVK6	0.18074
P0C206	P48643	0.16075
P0C206	P31949	0.40998
P0C206	Q9H765	0.2938
P0C206	Q9NZV6	0.10156
P0C206	Q6P161	0.21655
P0C206	Q8N137	1.0638
P0C206	Q49AG3	0.65406
P0C206	Q86XR8	1.1533
P0C206	A6NKD9	1.201
P0C206	Q99848	0.32377
P0C206	Q8WUF5	1.025
P0C206	P0CJ87	0.386
P0C206	Q9NSE4	0.32388
P0C206	Q9UBW5	0.66879
P0C206	Q15435	0.26891
P0C206	Q8N9M1	0.28103
P0C206	Q4G0F5	0.30498
P0C206	Q9HBL0	0.52112
P0C206	B1APH4	0.45983
P0C206	P07195	0.54372
P0C206	A6NEY8	0.32165
P0C206	Q86VZ6	0.29361
P0C206	Q969F1	0.71247
P0C206	Q9BPX3	1.0856
P0C206	P46939	0.83516
P0C206	Q96A35	0.68811
P0C206	Q9P031	1.2277
P0C206	Q8WXF5	0.66636
P0C206	Q53T94	0.53027
P0C206	P67809	0.57066
P0C206	Q08J23	0.77108
P0C206	Q9NQ87	0.53323
P0C206	Q14188	0.87541
P0C206	P68871	0.30606
P0C206	Q9NSB2	0.39837
P0C206	Q96L92	0.83366
P0C206	Q8N5N4	0.69495
P0C206	Q9NUP9	0.37129
P0C206	Q6NXS1	0.91273
P0C206	Q5XKR9	0.77251
P0C206	Q86V15	0.60347
P0C206	Q969W8	0.47516
P0C206	Q9UJV8	0.028716
P0C206	O43324	1.2413
P0C206	Q9UMS4	0.64995
P0C206	O14579	0.55167
P0C206	Q06190	0.17358
P0C206	P56181	0.62825
P0C206	Q9H4L7	0.43906
P0C206	Q9P1U0	0.38439
P0C206	Q8IX15	0.6368
P0C206	Q16385	0.9068
P0C206	Q96PU5	0.26529
P0C206	O14628	0.52463

P0C206	Q86WH2	0.37152
P0C206	P10914	0.43194
P0C206	P33240	0.40851
P0C206	Q9HCM1	0.6667
P0C206	Q8IXQ5	0.41066
P0C206	Q9Y3E1	0.51174
P0C206	Q13501	0.55543
P0C206	Q13829	0.82664
P0C206	Q8N5M1	0.58353
P0C206	Q92769	0.39868
P0C206	Q13541	0.86687
P0C206	P0CG48	0.41704
P0C206	Q96HY6	0.4278
P0C206	P54278	0.49377
P0C206	Q8WYP5	0.77989
P0C206	Q96NL6	0.2748
P0C206	P07814	0.41236
P0C206	Q5VT33	0.12438
P0C206	Q92539	0.66176
P0C206	P08048	0.20949
P0C206	Q9NS56	0.43052
P0C206	Q5VZQ5	0.6703
P0C206	Q8NF67	0.46235
P0C206	P60002	1.0154
P0C206	P07438	0.47357
P0C206	Q9BZE0	0.30232
P0C206	P13640	0.82287
P0C206	Q16655	0.45818
P0C206	Q6MZT1	0.92787
P0C206	Q7Z4U5	0.87009
P0C206	P0CJ71	0.67101
P0C206	Q63HQ0	0.45994
P0C206	O96002	0.45994
P0C206	P86479	0.45994
P0C206	O95081	0.35694
P0C206	Q2M3D2	0.28158
P0C206	Q01082	0.53065
P0C206	Q9P2N5	0.23657
P0C206	Q9NWK9	0.59676
P0C206	Q6IPX3	1.1152
P0C206	O15131	1.2183
P0C206	Q8NDC0	0.63909
P0C206	Q9Y291	0.47102
P0C206	P78424	0.33964
P0C206	Q8NB25	0.1761
P0C206	Q9NPJ3	0.17288
P0C206	Q9BXL5	0.46735
P0C206	Q14657	1.3397
P0C206	Q9H074	0.43198
P0C206	Q8N0X7	0.20408
P0C206	Q14525	0.41992
P0C206	O60291	0.60906
P0C206	Q9H5H4	0.33269
P0C206	Q96AJ1	1.2845
P0C206	Q9P0U4	0.49775
P0C206	Q9GZW5	0.57424
P0C206	Q538Z0	0.45994
P0C206	Q16649	0.24556
P0C206	Q9HBQ8	0.93098
P0C206	P0C7M6	0.073065
P0C206	Q8WWL7	1.0749
P0C206	O43933	0.35753
P0C206	Q96GT9	0.71558
P0C206	Q96KC9	0.64013
P0C206	Q99733	0.87369
P0C206	P58340	0.60452
P0C206	Q8IXW5	0.8092
P0C206	O43309	0.33771
P0C206	Q6ZNB5	0.1866
P0C206	Q9C005	0.78315
P0C206	P60368	0.45515
P0C206	O15127	0.891
P0C206	Q8N140	1.1101
P0C206	Q9H8Y1	0.52775
P0C206	P62875	0.31128
P0C206	Q9HB71	0.63894

P0C206	Q03060	0.47668
P0C206	Q5XG87	0.30864
P0C206	Q9NZH5	0.53988
P0C206	Q6ICL3	0.099619
P0C206	O95201	0.86737
P0C206	Q96JM7	0.53724
P0C206	Q15468	0.32937
P0C206	O94915	0.28047
P0C206	Q9UHP9	0.20888
P0C206	A8K010	0.25207
P0C206	Q86TP1	0.97074
P0C206	Q9BZK3	0.41367
P0C206	Q9BSN8	0.50345
P0C206	Q9NR46	0.55287
P0C206	Q9Y2Z0	0.54924
P0C206	Q9UI72	0.45994
P0C206	Q9Y5L4	0.016007
P0C206	Q14019	0.28773
P0C206	Q9BTE3	0.90696
P0C206	Q8TC21	0.32989
P0C206	Q5SZD1	0.12872
P0C206	Q6IQ49	0.33063
P0C206	P62877	0.64775
P0C206	Q9BYE4	0.45994
P0C206	Q9Y3M9	0.23915
P0C206	Q9Y3D5	0.61053
P0C206	P05062	0.25346
P0C206	O95273	1.0948
P0C206	P0DI82	0.4905
P0C206	Q9UN30	0.41743
P0C206	Q9NPD8	0.80903
P0C206	Q69384	0.86244
P0C206	A6NDR6	0.31537
P0C206	Q9NYJ1	1.1893
P0C206	O75698	0.45994
P0C206	Q8N8A2	0.44439
P0C206	A6NHQ2	0.23653
P0C206	O75530	0.73632
P0C206	Q9Y5L0	1.1469
P0C206	Q0VDF9	0.39722
P0C206	Q8N988	0.55714
P0C206	A6NCC3	0.41952
P0C206	P57076	0.28612
P0C206	Q5T1N1	1.0121
P0C206	Q9UKG1	0.50509
P0C206	Q6ZTY9	0.28282
P0C206	P98168	0.84163
P0C206	Q96SE7	0.39234
P0C206	Q9Y678	0.72757
P0C206	P0CB33	0.5334
P0C206	Q8IUR6	1.0323
P0C206	O75528	0.71161
P0C206	Q15437	0.52378
P0C206	P83876	1.005
P0C206	Q9BQM9	0.077973
P0C206	Q14587	0.26368
P0C206	P53805	0.69083
P0C206	Q6B0K9	0.40461
P0C206	Q99613	0.74204
P0C206	Q9H8Q6	0.79714
P0C206	P10746	0.3271
P0C206	P53675	0.41261
P0C206	Q9BQ61	0.68336
P0C206	Q9NQ35	0.96274
P0C206	Q8IXH7	1.3016
P0C206	Q8NAV1	0.83314
P0C206	Q86WZ0	0.54693
P0C206	Q9UI54	0.45994
P0C206	O75952	0.090693
P0C206	Q8N9L7	0.23033
P0C206	A8MZG2	0.68336
P0C206	O00148	0.38512
P0C206	Q96JC4	0.38091
P0C206	Q6JBY9	0.39109
P0C206	Q9P2B4	0.38893
P0C206	Q9Y4H4	0.43341

P0C206	Q96D05	0.98342
P0C206	Q2NKJ3	0.58404
P0C206	Q8N0U7	0.34594
P0C206	Q96S07	0.45994
P0C206	P51854	0.2138
P0C206	Q96Q83	0.62022
P0C206	Q7Z353	0.31537
P0C206	Q9Y3F4	0.89589
P0C206	Q8WUX9	0.61766
P0C206	Q6ZS46	0.8224
P0C206	Q3LI70	0.45994
P0C206	Q9Y6B7	0.39937
P0C206	Q7Z3Y9	0.48752
P0C206	Q6ZMY3	0.16639
P0C206	Q15270	0.2532
P0C206	Q9BTP6	0.62062
P0C206	Q9UN37	0.61146
P0C206	Q9Y399	1.0633
P0C206	Q96M02	0.21817
P0C206	Q05195	0.63866
P0C206	P16383	0.87218
P0C206	Q8IXS0	0.68336
P0C206	Q19AV6	1.2692
P0C206	Q96FZ2	1.2242
P0C206	Q96MP5	0.39903
P0C206	Q9HCK8	0.49375
P0C206	Q8N8E3	0.35035
P0C206	P60709	0.17471
P0C206	Q15329	1.3819
P0C206	P50402	0.45303
P0C206	Q9NQE9	0.49508
P0C206	Q9UKF6	0.70266
P0C206	Q8NHW5	0.2549
P0C206	Q6ZUT1	0.45994
P0C206	Q13601	0.47874
P0C206	Q92575	1.0929
P0C206	Q8N9N2	0.42895
P0C206	Q96T37	0.28563
P0C206	O95359	0.48586
P0C206	O60481	0.55928
P0C206	Q96G21	0.99509
P0C206	O94986	1.3638
P0C206	Q9Y2M0	0.52643
P0C206	Q9HB19	0.7107
P0C206	P28715	0.40204
P0C206	O43159	0.95767
P0C206	Q14103	0.73151
P0C206	P13010	0.54323
P0C206	Q86Y37	0.094785
P0C206	Q9BW61	0.66643
P0C206	Q68DH5	0.79899
P0C206	Q8N187	0.77288
P0C206	Q9NWM3	0.2067
P0C206	Q96L21	0.60033
P0C206	Q00444	0.34845
P0C206	P27635	0.63172
P0C206	Q9BQE6	0.52637
P0C206	Q9H6K5	0.68336
P0C206	P49336	0.44785
P0C206	Q96BV0	0.23399
P0C206	P0CJ90	0.386
P0C206	Q587I9	0.46076
P0C206	Q9NX46	0.56967
P0C206	Q9UM54	0.055852
P0C206	P59991	0.86576
P0C206	Q12988	0.52849
P0C206	Q9NQV6	0.20949
P0C206	P14678	0.72003
P0C206	Q9ULR5	0.61475
P0C206	P17661	0.51697
P0C206	P69208	0.45994
P0C206	Q496M5	0.40439
P0C206	P31483	0.28104
P0C206	P78352	0.63814
P0C206	O95478	0.45005
P0C206	Q9Y295	0.27848

P0C206	Q32MQ0	0.64868
P0C206	Q49AM1	0.50031
P0C206	Q8WTP9	1.1532
P0C206	P54725	0.71405
P0C206	Q96DB5	0.56768
P0C206	Q6P1M9	0.46746
P0C206	Q9BVI4	0.50628
P0C206	Q9Y2D1	1.2663
P0C206	O15172	0.26256
P0C206	Q8IZD9	0.27167
P0C206	Q8WU58	0.21718
P0C206	P06396	0.45927
P0C206	Q63HK3	0.54321
P0C206	Q9HCK5	0.67384
P0C206	Q7Z7K0	0.13369
P0C206	Q6K0P9	0.37056
P0C206	Q9Y5J6	0.47754
P0C206	Q9BVA0	0.35273
P0C206	Q9UM00	1.3473
P0C206	O60508	0.36514
P0C206	P0C025	0.61014
P0C206	Q7Z2X4	0.30391
P0C206	Q9GZV4	0.62652
P0C206	Q5VU69	0.3862
P0C206	P0CG12	1.4208
P0C206	Q8N9S9	0.26884
P0C206	Q7Z3I7	0.30827
P0C206	Q8TA86	0.95792
P0C206	Q9HAH7	0.60808
P0C206	Q9UHL9	0.44636
P0C206	Q7L0X2	0.35135
P0C206	Q8TAF3	0.78763
P0C206	P50452	0.71182
P0C206	Q8WWH5	0.42218
P0C206	Q9NRH1	0.33653
P0C206	Q9BTK2	0.0096923
P0C206	P62253	0.72675
P0C206	Q96NI8	0.47036
P0C206	Q15466	0.68631
P0C206	Q96LV5	0.086646
P0C206	Q6PCD5	0.33805
P0C206	Q9UJN7	0.32372
P0C206	P82930	0.85892
P0C206	Q5SWW7	0.45994
P0C206	Q9NSG2	0.55589
P0C206	Q96G23	0.28723
P0C206	Q8NA47	0.68336
P0C206	Q9H4A6	0.53062
P0C206	O75934	0.72801
P0C206	Q6PD62	0.72652
P0C206	Q96MW1	0.68336
P0C206	Q9UPV7	0.31596
P0C206	Q17RA5	0.45994
P0C206	P01358	0.45994
P0C206	Q8WU90	1.154
P0C206	O75832	0.37767
P0C206	Q99865	0.57702
P0C206	Q9BZI7	0.64865
P0C206	Q96N22	0.53747
P0C206	Q504Q3	0.21137
P0C206	A8MXY4	0.4071
P0C206	Q96AY2	0.97003
P0C206	Q9BUY5	0.45613
P0C206	Q96RY5	1.058
P0C206	P53701	0.57953
P0C206	Q8N9N5	0.46538
P0C206	Q6IPU0	0.67548
P0C206	O15360	0.97249
P0C206	P45973	0.81314
P0C206	Q96RT1	0.3785
P0C206	Q13568	1.0643
P0C206	Q0VDD7	1.3978
P0C206	A3QJZ6	0.073657
P0C206	Q8TB03	0.72008
P0C206	Q96MM3	0.2781
P0C206	O60384	0.57495

P0C206	Q8NAQ8	1.3423
P0C206	P35398	0.50244
P0C206	P23193	1.3679
P0C206	E9PQR5	0.08936
P0C206	Q17RS7	0.14099
P0C206	Q92830	0.71513
P0C206	P20042	0.44997
P0C206	O94842	0.78534
P0C206	Q6NSI4	0.15933
P0C206	Q9UDY4	0.92012
P0C206	P0C841	0.45994
P0C206	Q96RK0	1.1504
P0C206	P83436	0.59233
P0C206	E9PJI5	0.72101
P0C206	O94973	0.48509
P0C206	O75192	0.49899
P0C206	P0CL81	0.23762
P0C206	Q5XKP0	0.42743
P0C206	Q96AB6	0.26133
P0C206	Q96D15	0.34601
P0C206	Q96PG8	0.45994
P0C206	Q96PE7	0.11384
P0C206	Q15365	0.9329
P0C206	P36543	0.30358
P0C206	P09917	0.26797
P0C206	Q14588	0.40539
P0C206	Q9HCK1	0.64559
P0C206	P08779	0.80166
P0C206	Q9UHN1	0.23799
P0C206	Q5SZL2	0.44555
P0C206	P18124	0.59832
P0C206	O00167	0.8628
P0C206	Q96HR3	0.83525
P0C206	P61024	0.78423
P0C206	Q7Z398	0.31903
P0C206	Q8WW35	0.44439
P0C206	Q06546	1.3303
P0C206	Q8NEZ2	0.94221
P0C206	Q13077	0.43458
P0C206	Q96DC7	1.4428
P0C206	P49790	1.1523
P0C206	Q9GZZ1	0.53205
P0C206	Q9BYG3	0.28447
P0C206	Q8WXI4	0.017703
P0C206	Q9NSP4	0.22121
P0C206	Q8TEB1	0.37887
P0C206	Q6IEE7	1.0593
P0C206	Q96ST3	0.83196
P0C206	O14828	0.64174
P0C206	Q9HB65	0.67616
P0C206	Q8WWY6	0.58122
P0C206	P07196	0.5814
P0C206	Q8N2H9	0.37097
P0C206	Q6IS14	0.34917
P0C206	Q9C0C7	0.23304
P0C206	P39687	1.4932
P0C206	Q9NPJ4	1.5675
P0C206	Q03924	0.31278
P0C206	Q15545	1.0289
P0C206	Q495Z4	0.32772
P0C206	P0CJ76	0.67101
P0C206	Q96N21	0.71403
P0C206	Q8WV22	0.39066
P0C206	Q07955	0.50015
P0C206	Q9Y520	0.51481
P0C206	Q9UI43	0.78209
P0C206	O95149	0.45696
P0C206	Q6ZMR3	0.52228
P0C206	Q8NA19	0.52448
P0C206	Q8NEF9	0.82072
P0C206	P40305	0.60192
P0C206	Q9H2F5	0.66307
P0C206	O43711	0.4056
P0C206	Q86TB9	0.85038
P0C206	O14920	0.57448
P0C206	Q7Z6M4	0.61351

P0C206	Q5MJ09	0.21947
P0C206	Q8IX21	0.049865
P0C206	Q14739	0.34408
P0C206	Q9BUG6	0.29094
P0C206	P17026	0.27941
P0C206	Q6ZW61	0.17608
P0C206	P26447	0.3198
P0C206	P26368	0.43622
P0C206	P52298	0.47738
P0C206	Q14687	0.44439
P0C206	Q8IYM2	0.36694
P0C206	Q9Y296	0.34086
P0C206	Q8NA31	0.68632
P0C206	Q8IXM3	0.54106
P0C206	Q92556	0.36733
P0C206	Q96S65	1.0506
P0C206	Q99828	0.26431
P0C206	Q96EL3	0.21655
P0C206	Q8WUD4	0.19077
P0C206	P35527	0.48752
P0C206	P31269	0.71297
P0C206	Q7Z2W9	0.079886
P0C206	P31314	0.66102
P0C206	Q96IR2	0.41874
P0C206	Q96SN8	0.58654
P0C206	Q9H201	0.26258
P0C206	Q7RTU3	0.48008
P0C206	O14603	0.0072885
P0C206	Q5U649	0.12751
P0C206	Q16514	0.9371
P0C206	Q8IXM6	0.60415
P0C206	Q9H4P4	0.32954
P0C206	Q9H790	0.27405
P0C206	Q7Z2F6	0.4218
P0C206	Q9GZM3	0.15285
P0C206	Q6IV72	0.45163
P0C206	P0CL82	0.23762
P0C206	Q9BXL8	0.30845
P0C206	P55265	0.40497
P0C206	Q6VMQ6	1.0129
P0C206	Q96HT8	1.5614
P0C206	Q9NQ90	0.53974
P0C206	Q15493	0.20555
P0C206	O95453	0.43914
P0C206	P55198	1.1319
P0C206	A6NCN8	0.68336
P0C206	Q9P2Y5	0.49528
P0C206	O00221	0.27519
P0C206	P61576	0.50359
P0C206	P38935	0.6136
P0C206	Q6ZW54	1.1004
P0C206	Q96F07	0.57279
P0C206	P07864	0.54372
P0C206	Q9H693	0.58133
P0C206	Q5VWP2	0.12024
P0C206	Q5JNZ3	0.39086
P0C206	Q9Y324	0.85222
P0C206	P55789	0.10759
P0C206	A6NEL3	0.022964
P0C206	Q96GI7	0.68336
P0C206	P06401	0.30268
P0C206	Q5VZL5	0.91626
P0C206	Q86TC9	0.51442
P0C206	Q9ULE0	0.12162
P0C206	Q03701	0.54114
P0C206	Q9H444	0.86899
P0C206	Q06455	0.9131
P0C206	Q9UHA4	0.1679
P0C206	Q92844	0.61463
P0C206	Q9GZU2	0.67638
P0C206	Q9BYJ9	0.52927
P0C206	P31749	0.56152
P0C206	P78537	0.9721
P0C206	Q8N6T3	0.15997
P0C206	Q5T5D7	0.33408
P0C206	Q5JSJ4	1.2839

P0C206	Q92890	0.90286
P0C206	O95251	1.0155
P0C206	Q9Y6K1	0.49497
P0C206	P62854	0.85697
P0C206	O14867	0.47326
P0C206	Q12796	1.55
P0C206	Q5T1B0	0.016983
P0C206	Q9UBI1	0.44439
P0C206	Q8N883	0.2859
P0C206	Q5T754	0.45994
P0C206	Q96CS4	0.32718
P0C206	Q5VWN6	0.24601
P0C206	P27797	0.54923
P0C206	Q7LBR1	1.0679
P0C206	P40306	0.31308
P0C206	O75865	0.74928
P0C206	Q7Z3K6	0.75643
P0C206	Q9UBC1	0.59693
P0C206	O60393	0.23761
P0C206	Q01538	0.2152
P0C206	Q9HA92	0.41303
P0C206	Q6A162	0.34578
P0C206	Q9NWS8	0.64495
P0C206	O75794	0.98134
P0C206	Q9NXH3	0.35522
P0C206	Q9Y2G2	0.5242
P0C206	Q9H0U6	0.45345
P0C206	Q14CS0	0.56088
P0C206	Q8N535	0.49689
P0C206	Q9NPF5	1.0678
P0C206	Q9UHQ1	0.80194
P0C206	Q8WV60	0.28447
P0C206	Q5SY85	0.31086
P0C206	Q96GA3	0.87235
P0C206	P56715	0.19423
P0C206	A6NGH7	0.36826
P0C206	Q9C099	1.2464
P0C206	Q8NC54	1.2653
P0C206	Q5SRE5	0.72093
P0C206	Q68E01	0.73664
P0C206	P17677	0.19424
P0C206	Q86VG3	0.7041
P0C206	Q06330	0.60595
P0C206	Q13469	1.1816
P0C206	Q9UMS5	0.81781
P0C206	Q8TD26	0.37272
P0C206	Q9NP71	0.52818
P0C206	Q86YG4	0.51948
P0C206	P01008	0.32425
P0C206	O75496	1.0106
P0C206	Q9H299	0.21036
P0C206	P49959	0.26337
P0C206	Q9NP97	0.77463
P0C206	Q9Y2Z4	0.31227
P0C206	Q9ULX3	0.69101
P0C206	Q7Z7J5	0.91923
P0C206	Q9BXW4	0.41665
P0C206	P28065	0.53911
P0C206	Q86X60	0.39678
P0C206	Q9HC77	0.97753
P0C206	Q9Y6J3	0.45994
P0C206	P40425	0.80071
P0C206	Q502W6	0.050097
P0C206	Q68DK7	0.86262
P0C206	Q9BSV6	0.34842
P0C206	Q9NYT6	0.29385
P0C206	Q96GS4	1.1382
P0C206	Q9NS18	0.27549
P0C206	Q9UBV8	0.24574
P0C206	Q14997	0.40744
P0C206	Q75L30	0.45994
P0C206	Q9UH92	0.97047
P0C206	Q6UB98	0.54755
P0C206	Q9H305	0.15928
P0C206	P36639	0.54269
P0C206	Q15543	1.1135

P0C206	Q9H7T9	0.93227
P0C206	Q9UBU8	0.93011
P0C206	Q8N1P7	0.24644
P0C206	Q86SQ7	1.0758
P0C206	Q9Y6Q3	0.27243
P0C206	P0CW23	0.49733
P0C206	A8MTB1	0.45994
P0C206	P98182	0.30392
P0C206	Q9H0K4	0.74381
P0C206	Q8NEQ6	0.40139
P0C206	O14530	0.40442
P0C206	Q14008	1.0527
P0C206	Q99504	0.71889
P0C206	Q9UBP6	1.1592
P0C206	Q9H840	0.8946
P0C206	Q9NUQ3	0.8041
P0C206	Q8N3X6	0.61212
P0C206	Q92567	0.43814
P0C206	Q9Y2A0	0.3178
P0C206	Q9UBB4	0.94934
P0C206	Q9NVN8	0.40151
P0C206	Q9BUP3	0.17578
P0C206	O14576	0.56588
P0C206	Q96Q89	0.65006
P0C206	O95260	0.21089
P0C206	O75396	0.39552
P0C206	Q9NWS6	0.83133
P0C206	Q9UL45	0.1916
P0C206	Q8NFH5	0.73463
P0C206	Q93100	0.31573
P0C206	A8MXV4	0.55689
P0C206	P0C5W0	0.27799
P0C206	Q86UW9	1.1102
P0C206	Q8NB50	0.41874
P0C206	Q9GZQ3	1.0456
P0C206	Q13432	0.60193
P0C206	Q5U5X0	0.93861
P0C206	Q9NRF9	0.15056
P0C206	Q9UJQ4	0.26416
P0C206	Q9Y2V7	0.11443
P0C206	A4D1U4	0.39358
P0C206	Q5VXD3	0.13086
P0C206	Q9HAZ2	0.51229
P0C206	Q9GZL8	0.40853
P0C206	Q7Z4G1	1.4614
P0C206	Q9BQ15	0.82136
P0C206	Q12905	1.1105
P0C206	Q9H4X1	0.93504
P0C206	Q8TDN4	0.84469
P0C206	Q96CB9	0.7484
P0C206	Q9P032	0.59748
P0C206	Q96DX5	1.1972
P0C206	O00512	0.95264
P0C206	Q9HC52	0.92994
P0C206	Q8N9F8	0.35984
P0C206	P61581	0.95266
P0C206	Q8WW12	0.744
P0C206	P0DKV0	0.075957
P0C206	Q96IZ0	0.89949
P0C206	Q9BSU1	0.71197
P0C206	Q5UIP0	0.96233
P0C206	Q13127	0.58578
P0C206	Q9NRG9	1.0109
P0C206	Q6P575	0.10533
P0C206	Q9BSF8	0.54755
P0C206	Q9UJX3	0.85404
P0C206	Q9NZI5	0.6508
P0C206	O60504	0.81164
P0C206	O95780	0.56457
P0C206	P60673	0.32917
P0C206	Q16222	0.2834
P0C206	Q9BQC3	1.0327
P0C206	P13682	0.35632
P0C206	Q9BV47	0.50639
P0C206	Q96ST8	1.1272
P0C206	Q53H82	0.1428

P0C206	Q8TF63	0.62813
P0C206	Q7RTS9	1.2509
P0C206	A6NK44	0.096969
P0C206	Q96EU6	0.3161
P0C206	Q9NUU7	0.21206
P0C206	P50213	0.053586
P0C206	Q9BUX1	0.85715
P0C206	P49848	0.83232
P0C206	Q9NTX9	0.14151
P0C206	Q15397	0.17861
P0C206	Q9Y6K9	0.97425
P0C206	O75251	0.16788
P0C206	Q9UPN9	0.6416
P0C206	Q96EL2	0.4343
P0C206	Q6ZN84	0.23342
P0C206	Q9Y5Q3	0.92965
P0C206	Q8IYN0	0.38973
P0C206	P61326	0.84228
P0C206	P14921	0.84886
P0C206	Q05481	0.4071
P0C206	Q9UQ84	0.38843
P0C206	P78385	0.43636
P0C206	Q15911	0.33207
P0C206	Q9H900	0.66357
P0C206	Q8N228	0.49155
P0C206	Q9BYG7	0.32773
P0C206	Q5U4N7	0.45994
P0C206	Q9NXJ5	0.42188
P0C206	P0C867	0.37401
P0C206	P46976	0.51379
P0C206	P47756	0.066274
P0C206	O75420	0.44518
P0C206	Q9Y6Q5	0.49988
P0C206	P24522	0.69876
P0C206	Q9HAY2	0.1174
P0C206	Q8N815	0.010575
P0C206	Q01081	0.51628
P0C206	P49207	0.98382
P0C206	Q8WZA8	0.45994
P0C206	P78318	0.88865
P0C206	Q9NY43	0.49939
P0C206	Q96AP0	0.16702
P0C206	Q3SY56	0.24797
P0C206	Q9NSU2	0.15836
P0C206	Q96LP2	0.12638
P0C206	Q99766	0.70933
P0C206	Q86Z20	1.1206
P0C206	Q92845	0.31085
P0C206	Q8IWE5	0.53434
P0C206	Q9NSI6	0.95353
P0C206	Q8TF01	0.89109
P0C206	Q0VGE8	0.30062
P0C206	P56278	0.18714
P0C206	P51532	0.50506
P0C206	P26651	0.80832
P0C206	P81274	0.65032
P0C206	Q8IZH2	0.20087
P0C206	Q06416	0.43565
P0C206	Q9NPI1	1.0999
P0C206	O00291	0.63072
P0C206	Q14681	0.13145
P0C206	Q9NZM5	0.44224
P0C206	Q9NV88	0.70712
P0C206	Q9Y4L5	0.55355
P0C206	Q12815	0.47407
P0C206	Q9BQA1	1.0734
P0C206	Q8TCS8	0.24059
P0C206	Q6ZTR7	0.069073
P0C206	P35251	0.51174
P0C206	Q86W50	0.47207
P0C206	O15061	0.82205
P0C206	P60174	0.19452
P0C206	Q96HA9	0.55851
P0C206	Q9P2G1	0.49231
P0C206	Q07627	0.4802
P0C206	O00743	0.90805

P0C206	Q5TI25	1.4923
P0C206	Q6PF04	0.30822
P0C206	Q9UBP0	0.33163
P0C206	Q5SNX5	0.45994
P0C206	Q5BJF6	0.64643
P0C206	Q9UMY1	1.0385
P0C206	Q8NCN2	0.41273
P0C206	Q92572	0.57581
P0C206	Q7Z6J0	0.43295
P0C206	Q9NSA3	0.86458
P0C206	O95684	0.9284
P0C206	Q8WTX7	0.54932
P0C206	Q17RN3	0.39948
P0C206	Q9UBC9	0.61185
P0C206	Q6V702	0.26627
P0C206	Q9Y2D5	0.25268
P0C206	Q9HAW0	0.69666
P0C206	Q9NZ42	0.42733
P0C206	Q9Y4A8	0.60133
P0C206	Q86UU0	0.9748
P0C206	Q13114	0.49486
P0C206	Q9Y242	0.45995
P0C206	P0CB43	0.33728
P0C206	Q5K130	0.068669
P0C206	Q9BQ48	0.83316
P0C206	Q08AG5	0.61551
P0C206	Q9NV44	0.16914
P0C206	Q96KR4	0.1757
P0C206	Q5MJ68	0.41741
P0C206	Q8WWX9	0.66333
P0C206	Q9NS25	0.86383
P0C206	Q9HAA7	0.067235
P0C206	Q5VY09	0.86655
P0C206	Q9Y6R9	0.70707
P0C206	Q16537	0.48604
P0C206	Q6FI81	0.56472
P0C206	Q9ULM3	0.90573
P0C206	Q9NR55	0.45577
P0C206	Q9H993	0.053384
P0C206	Q9H6Q3	0.33829
P0C206	Q9BYE3	0.45994
P0C206	Q49B96	0.49621
P0C206	O43592	0.44506
P0C206	Q9UEU5	0.42333
P0C206	Q96C86	0.9379
P0C206	O43791	0.64067
P0C206	B4DU55	0.39689
P0C206	Q86U38	0.21007
P0C206	Q9NVW2	0.53706
P0C206	O43255	0.49319
P0C206	Q8TC71	0.56135
P0C206	Q6NX49	0.27546
P0C206	Q9UC06	0.33909
P0C206	Q9NZL9	0.15157
P0C206	Q8TAK5	0.62337
P0C206	Q9BV20	0.36437
P0C206	Q8N8U3	0.29451
P0C206	O60784	0.47803
P0C206	P62879	0.57557
P0C206	Q969F2	0.19475
P0C206	Q9GZU7	0.6601
P0C206	Q6L9T8	0.43017
P0C206	Q16186	0.69026
P0C206	Q6ZRS4	0.65291
P0C206	Q641Q2	0.92761
P0C206	P36954	0.31415
P0C206	Q9BV86	0.45907
P0C206	O75123	0.29797
P0C206	Q9Y3B6	0.83813
P0C206	Q9NX53	0.44439
P0C206	Q02078	0.47642
P0C206	P35325	0.45994
P0C206	Q147U1	0.34723
P0C206	Q96N11	0.27204
P0C206	Q9UDW1	0.17374
P0C206	Q14586	0.29385

P0C206	O15266	0.61793
P0C206	P55036	0.44751
P0C206	Q969W3	0.97891
P0C206	Q9ULP0	0.21086
P0C206	Q9UNK9	0.25515
P0C206	Q8ND56	0.79396
P0C206	Q99708	0.82683
P0C206	Q9P086	1.1227
P0C206	Q96PE6	0.29519
P0C206	A8MVM7	0.45994
P0C206	Q9GZN7	0.39004
P0C206	P43358	0.50382
P0C206	A6NNA5	0.16039
P0C206	Q13263	0.30807
P0C206	Q9UKK6	0.82358
P0C206	Q8N7L0	0.32028
P0C206	Q9Y3D8	0.2607
P0C206	Q8N726	0.65143
P0C206	Q9BRT2	0.51252
P0C206	Q13526	0.63955
P0C206	Q15361	0.90031
P0C206	A6NKC0	0.49928
P0C206	Q9UII5	0.39483
P0C206	P50750	0.50326
P0C206	Q8WVI7	0.15166
P0C206	Q15696	0.2593
P0C206	P0CJ72	0.67101
P0C206	Q15019	0.27956
P0C206	Q3LI77	0.12421
P0C206	Q5TGS1	0.59711
P0C206	Q8NA96	0.15893
P0C206	O94864	0.6355
P0C206	Q8N6I1	0.83629
P0C206	P52741	0.37667
P0C206	Q53H64	0.13726
P0C206	O95602	0.82053
P0C206	Q8IVM0	0.66879
P0C206	P30307	0.76403
P0C206	O75474	1.136
P0C206	Q9Y2N7	0.64748
P0C206	P43356	1.0607
P0C206	Q9H981	1.3632
P0C206	Q96KS0	0.16001
P0C206	Q5VUG0	0.451
P0C206	A6NGD5	0.37883
P0C206	Q8IYW5	0.37874
P0C206	Q3C1V9	0.41351
P0C206	Q9BRQ4	0.2872
P0C206	Q86Y26	0.69223
P0C206	Q5TEA3	0.2803
P0C206	O95376	0.50131
P0C206	P13533	0.26858
P0C206	B7Z6K7	0.28545
P0C206	Q8NAJ2	0.11112
P0C206	Q9HD34	0.66602
P0C206	Q9Y3E7	0.91701
P0C206	Q9BX40	0.80798
P0C206	Q9NR28	0.9096
P0C206	Q9BTE1	0.3044
P0C206	Q8TD86	0.41191
P0C206	Q14CZ0	1.4626
P0C206	O96017	0.80044
P0C206	Q7Z2V1	0.45994
P0C206	Q9H6F5	0.41544
P0C206	Q5JQC9	0.32566
P0C206	Q8IYU4	0.053808
P0C206	Q9H4I2	0.92884
P0C206	P02100	0.41469
P0C206	Q9BT43	0.040658
P0C206	Q8WUU5	0.46223
P0C206	B1AL46	0.75244
P0C206	Q9UNH7	0.32572
P0C206	P15407	0.63396
P0C206	Q9BVM4	0.37741
P0C206	P02538	0.77159
P0C206	A6NFF2	0.18976

P0C206	P57088	0.76745
P0C206	Q9Y6Q2	0.25026
P0C206	P78364	0.22691
P0C206	Q9BZR8	0.52756
P0C206	Q8N699	0.76264
P0C206	Q13309	0.54771
P0C206	Q9Y5U8	0.45702
P0C206	P62081	0.97133
P0C206	P20226	0.96825
P0C206	P12525	0.32741
P0C206	P69892	0.41469
P0C206	Q5TD94	0.3165
P0C206	A8MTZ7	0.29274
P0C206	Q8N3V7	0.80028
P0C206	Q9Y3D3	0.74208
P0C206	Q9UPM9	0.059991
P0C206	Q9BT92	0.77193
P0C206	O00308	0.23642
P0C206	O75145	0.57629
P0C206	Q14137	0.91295
P0C206	Q13642	0.57289
P0C206	P11161	0.53121
P0C206	P04156	0.59323
P0C206	Q8IYX0	0.56946
P0C206	Q8NDZ9	0.45994
P0C206	Q9UBB5	1.311
P0C206	Q8IYN6	0.80301
P0C206	Q8NC96	0.40991
P0C206	Q6X4T0	0.29568
P0C206	O43365	0.47977
P0C206	P17031	0.36831
P0C206	Q09161	0.69
P0C206	Q9P015	0.61549
P0C206	Q9BXY8	0.7721
P0C206	Q9HAU4	0.39974
P0C206	Q86VK4	0.20949
P0C206	A8MWP4	0.1332
P0C206	Q9NZT2	0.19663
P0C206	Q86UT8	1.1498
P0C206	Q6ZNM6	0.85534
P0C206	Q9UDY8	0.27929
P0C206	Q96MT3	1.1208
P0C206	Q04743	0.28418
P0C206	P18545	0.44613
P0C206	P53618	0.8281
P0C206	Q9P299	0.43683
P0C206	Q9BVP2	0.30254
P0C206	O43747	0.77238
P0C206	O43920	0.84323
P0C206	Q15544	1.1293
P0C206	Q5VSL9	0.9274
P0C206	Q6XQN6	0.3036
P0C206	Q8IVE0	0.19595
P0C206	P0CW24	0.27799
P0C206	Q9HCC9	0.61181
P0C206	Q9UHQ7	0.52631
P0C206	Q9BXF6	0.77774
P0C206	P30050	0.67945
P0C206	E7EW31	0.45994
P0C206	P25786	0.4628
P0C206	Q9H511	0.28047
P0C206	Q9H9Q2	0.35853
P0C206	P05114	0.40302
P0C206	Q9Y287	0.32435
P0C206	Q9NY12	0.3828
P0C206	Q9NY30	0.36849
P0C206	Q9BVC5	0.44263
P0C206	O60759	0.52477
P0C206	Q8WY54	0.97638
P0C206	P18583	0.2879
P0C206	A2RRD8	0.39805
P0C206	Q9H9E3	0.62136
P0C206	P55769	0.77565
P0C206	P16220	0.86516
P0C206	Q9P2X8	0.16535
P0C206	Q9NW07	0.32527

P0C206	Q9Y5T5	0.41927
P0C206	Q8NEU8	0.5843
P0C206	O14524	0.79548
P0C206	Q6P050	0.85709
P0C206	P17275	0.904
P0C206	Q9Y5R6	0.033891
P0C206	Q9P000	0.38183
P0C206	Q6P6B1	0.68336
P0C206	Q9H579	0.020269
P0C206	Q9NVV5	0.49977
P0C206	O14948	0.50862
P0C206	Q9GZT9	0.314
P0C206	O60518	1.1138
P0C206	Q68DL7	0.45994
P0C206	O95633	0.24449
P0C206	P09067	0.15039
P0C206	Q9UN79	1.0245
P0C206	Q5PR19	0.32463
P0C206	P10074	0.57889
P0C206	Q06787	0.8521
P0C206	A6NIY4	0.51209
P0C206	Q1A5X6	0.68336
P0C206	Q8IYW2	0.45994
P0C206	O75884	1.2258
P0C206	O75607	0.79643
P0C206	O94855	0.53122
P0C206	P30153	0.57027
P0C206	Q8TAY7	1.6967
P0C206	P39656	0.389
P0C206	Q9UL42	0.96895
P0C206	O43516	0.36421
P0C206	Q9NR33	0.1454
P0C206	Q8IVT2	0.87342
P0C206	Q3SYG4	0.56789
P0C206	O14737	0.41164
P0C206	P62166	0.32164
P0C206	Q9HD36	0.52209
P0C206	Q92681	0.63372
P0C206	A8MS94	0.18056
P0C206	Q9Y6D9	1.0958
P0C206	Q9UBT6	0.19037
P0C206	Q9UK13	0.52141
P0C206	Q5JXB2	0.64704
P0C206	E9PB15	0.15943
P0C206	Q5T3J3	1.1546
P0C206	O43151	0.4893
P0C206	Q8TAM2	0.27841
P0C206	Q15700	0.24372
P0C206	Q9BTP7	1.2326
P0C206	Q3ZCV2	0.68336
P0C206	Q9UHJ9	0.74693
P0C206	Q9UFV3	0.16958
P0C206	O43422	0.44493
P0C206	Q96GX8	0.68336
P0C206	Q8NI27	0.58597
P0C206	Q96N23	0.57802
P0C206	Q9BRA0	0.33985
P0C206	O14602	0.42323
P0C206	Q9HAU0	0.35046
P0C206	P35869	0.42108
P0C206	Q9UPZ3	0.68865
P0C206	O15527	0.54574
P0C206	O14862	0.57712
P0C206	P0C838	0.53888
P0C206	O95810	0.41061
P0C206	Q86WI3	0.58204
P0C206	P40121	0.51931
P0C206	Q8NA72	0.47201
P0C206	Q96SL8	0.3695
P0C206	Q8WXD5	0.89494
P0C206	Q14692	0.42338
P0C206	P17544	1.1882
P0C206	O75030	0.60954
P0C206	A6NMK8	0.70781
P0C206	Q7L1V2	1.0651
P0C206	Q9H5F2	0.39281

P0C206	Q9UMX1	0.83756
P0C206	Q96DT7	0.23605
P0C206	P62736	0.17471
P0C206	Q17R31	0.22442
P0C206	Q5TG53	0.45994
P0C206	Q9H6J7	0.33913
P0C206	Q9UGU0	0.62207
P0C206	Q10567	0.75369
P0C206	Q6DCA0	0.056086
P0C206	P0C7X5	0.37522
P0C206	Q8TAD8	1.424
P0C206	Q86W67	0.10662
P0C206	Q96C90	0.76396
P0C206	O75925	0.73553
P0C206	O94964	0.95936
P0C206	Q8WUA4	0.26386
P0C206	P09874	0.87324
P0C206	Q15744	0.38969
P0C206	Q6ZW49	0.54511
P0C206	P25788	0.57763
P0C206	O43427	0.15892
P0C206	Q6IN85	0.44065
P0C206	O14653	0.21863
P0C206	Q9NWN3	0.47762
P0C206	P54920	0.50775
P0C206	Q86YC2	0.82878
P0C206	Q9BVV6	0.45994
P0C206	P20290	1.2628
P0C206	Q5EBL4	0.53363
P0C206	Q6NUQ1	0.70658
P0C206	Q9NVU7	0.58422
P0C206	Q5TC84	0.46292
P0C206	Q93008	0.5447
P0C206	Q96JG6	0.1211
P0C206	Q8IV20	0.37376
P0C206	Q5H9R7	1.0285
P0C206	Q9UDV7	0.2761
P0C206	Q9ULL0	0.56295
P0C206	Q92562	0.39743
P0C206	P51161	0.28262
P0C206	Q9H4B0	0.63438
P0C206	Q6ZWJ1	0.35408
P0C206	P22392	0.30386
P0C206	P29144	0.04937
P0C206	Q9Y248	0.8092
P0C206	P25490	0.35169
P0C206	Q9BZZ5	0.53608
P0C206	O43313	0.33183
P0C206	Q8IY51	0.047276
P0C206	Q9Y623	0.33462
P0C206	O75290	0.51617
P0C206	P15622	0.35755
P0C206	Q8NEH6	0.92279
P0C206	Q9BR76	0.42234
P0C206	Q32M84	0.23261
P0C206	O43815	0.34237
P0C206	P68036	0.76104
P0C206	Q6ICC9	0.58561
P0C206	Q9H8V8	0.45994
P0C206	Q9Y5L5	1.1565
P0C206	O14933	0.74262
P0C206	P43357	0.44439
P0C206	Q9BYQ3	0.72851
P0C206	Q8N9Z0	0.4933
P0C206	Q9NSD4	0.35148
P0C206	O60783	0.6312
P0C206	Q5VXH4	0.41864
P0C206	Q8NG27	0.41186
P0C206	Q05D60	0.15401
P0C206	Q96G03	0.60072
P0C206	Q9UIC8	0.37924
P0C206	Q9UJ55	0.1174
P0C206	Q8IXP5	0.68336
P0C206	Q8NDL9	0.11238
P0C206	O75161	1.1865
P0C206	Q969M7	0.5414

P0C206	Q02446	0.24041
P0C206	Q86UP8	0.23143
P0C206	Q8N8Z8	0.30186
P0C206	Q96I23	0.38753
P0C206	Q8N6L7	1.1172
P0C206	P08651	0.38651
P0C206	Q9ULW0	1.0585
P0C206	Q9ULD0	0.28768
P0C206	Q9UNA4	0.52035
P0C206	Q9BU61	0.81398
P0C206	P22061	0.51562
P0C206	Q96RT7	0.54231
P0C206	Q3SXR2	0.23216
P0C206	Q04323	0.37073
P0C206	Q08AD1	0.70422
P0C206	Q7L5A3	0.50914
P0C206	O75444	0.5264
P0C206	Q96PI1	0.79757
P0C206	Q7Z4W3	0.45994
P0C206	Q9UK73	0.82379
P0C206	Q96B49	0.82081
P0C206	Q9HBE1	0.31465
P0C206	P82914	0.76238
P0C206	Q9UJL9	0.29335
P0C206	Q7Z6I5	0.84166
P0C206	Q9HCU8	0.5684
P0C206	Q9UGC7	0.45074
P0C206	O95424	0.54864
P0C206	Q5T5A8	0.45994
P0C206	P79522	0.56154
P0C206	P18754	1.2725
P0C206	P02794	0.82237
P0C206	P18850	0.68405
P0C206	Q9UL41	0.70306
P0C206	Q8IY31	0.68651
P0C206	Q9BPY3	0.28704
P0C206	Q96HI0	0.67507
P0C206	Q330K2	0.3817
P0C206	Q9UHV2	1.5855
P0C206	O60664	0.7902
P0C206	O75170	0.88321
P0C206	Q8IUF8	0.62298
P0C206	Q07021	0.86745
P0C206	Q14966	0.10912
P0C206	Q96F45	0.30231
P0C206	P49910	0.92544
P0C206	O43759	0.60017
P0C206	O95164	0.83078
P0C206	Q13084	1.1842
P0C206	Q9H4M3	0.35409
P0C206	Q8N635	0.041279
P0C206	Q8NCF5	0.64262
P0C206	Q7L2Z9	0.21203
P0C206	O75419	0.99746
P0C206	Q9UNY4	0.64547
P0C206	Q96HJ5	0.15878
P0C206	Q92733	0.97586
P0C206	Q8IWL8	0.90287
P0C206	P0C7V6	0.7778
P0C206	Q9P243	0.53273
P0C206	P41236	0.5748
P0C206	Q14690	0.54878
P0C206	Q9BV29	0.1336
P0C206	Q96E40	0.077606
P0C206	O15040	0.44439
P0C206	O75334	0.6677
P0C206	Q8NDA8	0.64213
P0C206	P0C7T7	0.45994
P0C206	Q9NTU4	0.50369
P0C206	Q8WUN7	0.54755
P0C206	Q3SY46	0.60142
P0C206	Q9NPD3	0.29865
P0C206	Q86XK2	0.91878
P0C206	Q92664	0.27567
P0C206	A1L190	0.94073
P0C206	Q96PV7	0.79661

P0C206	A5PLL1	0.33985
P0C206	Q5T686	0.6175
P0C206	Q5SQN1	0.47129
P0C206	Q6ZSB9	0.28894
P0C206	O60225	0.26627
P0C206	O97980	0.15893
P0C206	P41252	0.70722
P0C206	Q9BRU2	0.62874
P0C206	Q96FX8	0.69182
P0C206	Q9NRS6	0.14736
P0C206	P57060	0.57309
P0C206	Q13156	0.30586
P0C206	P78395	0.84526
P0C206	Q8N814	0.9729
P0C206	Q86YF9	0.27052
P0C206	Q5T1J5	0.95874
P0C206	O95721	0.52346
P0C206	P51955	0.37674
P0C206	Q7Z5B4	0.67867
P0C206	Q13023	0.69275
P0C206	Q8N944	1.1573
P0C206	Q9BU68	0.57426
P0C206	Q8N5C8	0.61256
P0C206	Q8NDZ2	0.32649
P0C206	Q8N7R0	0.43565
P0C206	Q96KN1	0.54457
P0C206	Q08752	0.44414
P0C206	P86496	0.45994
P0C206	Q5JTH9	0.44558
P0C206	O60907	0.8609
P0C206	Q8N6T0	0.37134
P0C206	Q56P42	0.33985
P0C206	P30419	0.30553
P0C206	P15502	0.30878
P0C206	Q56P03	0.59057
P0C206	O43402	0.84672
P0C206	Q8WYH8	0.73603
P0C206	Q96N95	0.50258
P0C206	Q99622	0.29006
P0C206	O95049	0.61011
P0C206	Q7L5Y6	0.11206
P0C206	Q8TB69	0.53104
P0C206	Q9BT49	0.73716
P0C206	Q9Y4P1	0.41875
P0C206	O43812	0.43559
P0C206	Q14258	1.0829
P0C206	Q6W2J9	0.51593
P0C206	A0PJX2	0.42503
P0C206	Q6PI48	0.60863
P0C206	O94913	0.33916
P0C206	Q86XL3	0.47179
P0C206	Q9Y6N9	0.40421
P0C206	Q49AA0	0.48721
P0C206	P41212	0.90467
P0C206	Q9UD71	0.90743
P0C206	Q9UGP4	0.60355
P0C206	Q9C0B9	0.25202
P0C206	Q6UN15	1.0007
P0C206	Q9H9T3	0.93305
P0C206	P62241	0.64704
P0C206	Q5RKV6	0.15456
P0C206	Q4U2R6	0.9481
P0C206	Q7L190	0.92378
P0C206	Q9BV68	0.49231
P0C206	Q9NSC2	0.36996
P0C206	Q8N5R6	0.6717
P0C206	O75154	0.99987
P0C206	Q96I27	0.28365
P0C206	P54105	0.94855
P0C206	Q96HG1	0.45994
P0C206	A8MVP8	0.10538
P0C206	O14521	0.55207
P0C206	P48382	1.0915
P0C206	Q96BP3	0.26092
P0C206	Q5VXU3	0.51967
P0C206	Q99873	0.67475

P0C206	Q9NRR8	0.7446
P0C206	Q9NV92	0.20038
P0C206	P25205	0.48836
P0C206	A6NNH0	0.87489
P0C206	Q969F9	0.94986
P0C206	O75381	0.50595
P0C206	Q5T871	0.45994
P0C206	P60371	0.72851
P0C206	P62304	0.62822
P0C206	Q9BT04	0.59002
P0C206	P52701	0.11164
P0C206	Q9UC07	0.37223
P0C206	A6NEV1	0.6609
P0C206	P13489	0.58126
P0C206	Q05066	0.62321
P0C206	Q9Y2G0	0.45836
P0C206	Q9NR11	0.28626
P0C206	Q9Y6D6	0.69685
P0C206	Q96BW1	0.33985
P0C206	O75582	0.32125
P0C206	Q9BYP8	0.45994
P0C206	Q9Y2Y9	0.97249
P0C206	Q8IUI4	0.31467
P0C206	Q9NVD7	0.3458
P0C206	Q86WC6	0.34507
P0C206	Q15697	0.66939
P0C206	Q8N3X1	0.32773
P0C206	P62244	0.79259
P0C206	O14744	1.0179
P0C206	Q5W0A0	0.3226
P0C206	Q96EP1	0.74911
P0C206	Q5HYJ3	0.50408
P0C206	P25787	0.54434
P0C206	Q99933	0.85789
P0C206	Q5CZA5	0.39689
P0C206	P51587	0.2186
P0C206	P78414	0.31537
P0C206	Q05519	0.5716
P0C206	Q9BYP9	0.72851
P0C206	Q9Y2M2	0.44787
P0C206	Q86VP1	0.52862
P0C206	O75306	0.56994
P0C206	Q15233	0.63651
P0C206	A4D161	0.0026307
P0C206	Q14315	0.40431
P0C206	P04732	0.45193
P0C206	A6NDB9	0.4627
P0C206	Q8IWD4	0.68336
P0C206	P48634	1.0004
P0C206	Q5F1R6	0.18027
P0C206	Q8IYB3	0.44399
P0C206	Q9C0D2	0.86056
P0C206	A6NCL1	0.33539
P0C206	Q8IY85	0.43791
P0C206	Q8NG48	0.079141
P0C206	A9QM74	0.73044
P0C206	Q96AV8	0.47687
P0C206	Q5T5S1	0.59845
P0C206	P12074	0.33271
P0C206	P45984	0.38796
P0C206	P50914	0.92005
P0C206	Q15121	0.58584
P0C206	Q01860	0.75656
P0C206	Q6ZS72	0.19224
P0C206	Q32P41	0.49726
P0C206	Q9H7E2	0.58973
P0C206	Q8NEP4	0.68336
P0C206	Q96A04	1.0116
P0C206	Q7Z7K2	0.25189
P0C206	O15258	0.49144
P0C206	Q9Y4Z2	0.29028
P0C206	Q6P3X8	0.61242
P0C206	Q9UJX5	0.88962
P0C206	Q5TZA2	0.82286
P0C206	A1A4T8	0.00048364
P0C206	P51531	0.75402

P0C206	Q96GJ1	0.22519
P0C206	P56589	0.048978
P0C206	O75382	0.53571
P0C206	Q9BYC8	0.91791
P0C206	Q6P2H3	0.79443
P0C206	Q8N2G8	0.29649
P0C206	Q9H091	0.456
P0C206	A6NE82	0.28047
P0C206	Q3KRA9	0.17778
P0C206	Q8N9P6	0.45994
P0C206	Q96IF1	0.51583
P0C206	P41182	0.26217
P0C206	O75638	1.3885
P0C206	A6NC42	0.48726
P0C206	P17024	0.37336
P0C206	Q05516	0.84154
P0C206	Q7Z3B4	0.951
P0C206	Q96MF6	0.42453
P0C206	O43913	0.43575
P0C206	Q9UPA5	0.33875
P0C206	P49901	0.45994
P0C206	Q9NXV2	1.385
P0C206	Q6V9R5	0.53376
P0C206	Q9Y2J0	0.26895
P0C206	Q6ZV56	0.45994
P0C206	Q13868	0.54366
P0C206	Q9NQW6	0.47917
P0C206	Q13615	0.19197
P0C206	Q9NZN8	0.82681
P0C206	O95202	0.3604
P0C206	Q8TD31	0.78743
P0C206	P08865	0.8316
P0C206	Q5ST30	0.27647
P0C206	Q9NQR7	0.45994
P0C206	P52743	0.43791
P0C206	P54821	0.23679
P0C206	Q9UQK1	0.42174
P0C206	Q86XZ4	0.46469
P0C206	Q8N1K5	0.60143
P0C206	O60927	0.93006
P0C206	P56211	0.40024
P0C206	Q8TCU4	0.13109
P0C206	Q08211	0.27466
P0C206	Q86WV5	0.53854
P0C206	Q6NS38	0.42249
P0C206	Q9UIV1	1.2546
P0C206	P08727	0.55133
P0C206	Q6AWC8	0.54243
P0C206	Q96CV9	0.65502
P0C206	Q9H869	1.5805
P0C206	Q96FF7	0.45994
P0C206	Q13228	1.3113
P0C206	P41219	0.33044
P0C206	P41227	0.43135
P0C206	Q9C010	0.64319
P0C206	Q8NA69	0.0016508
P0C206	P01858	0.45994
P0C206	Q9HB03	0.65817
P0C206	P62750	0.7193
P0C206	Q5H9J9	0.24017
P0C206	O60294	0.43617
P0C206	Q9UJX2	0.71399
P0C206	Q6ZNA4	0.70885
P0C206	Q9BTV5	0.7262
P0C206	C9J6K1	0.44531
P0C206	P49639	0.28345
P0C206	Q96CG3	0.44734
P0C206	Q8N1E6	0.47623
P0C206	Q9GZL7	0.78961
P0C206	Q8IY63	0.53567
P0C206	Q96CW5	1.0276
P0C206	Q86YA3	0.94109
P0C206	Q13515	0.34311
P0C206	Q15554	0.59257
P0C206	A6NKU9	0.51209
P0C206	Q9BYQ0	0.72851

P0C206	Q9H7R0	0.50051
P0C206	Q9BS31	0.23477
P0C206	Q13625	0.52624
P0C206	O75807	0.53332
P0C206	O75717	0.57574
P0C206	Q8WUE5	0.075006
P0C206	Q06323	0.70662
P0C206	Q9UMX0	0.85557
P0C206	Q32MH5	0.56851
P0C206	Q99583	0.47898
P0C206	O43681	0.21936
P0C206	Q96B36	0.89425
P0C206	Q969P5	0.26941
P0C206	Q9NR00	0.43665
P0C206	P05204	0.53685
P0C206	Q2VPB7	0.63984
P0C206	Q8NAA6	0.0029259
P0C206	P14317	0.32655
P0C206	Q2VWA4	0.25152
P0C206	P18621	0.66084
P0C206	P09012	0.24179
P0C206	P62380	0.24275
P0C206	Q9UHK0	0.41216
P0C206	Q8N7W2	0.63911
P0C206	Q96K75	0.31949
P0C206	Q9UQR0	0.37991
P0C206	Q9H1E3	0.83954
P0C206	Q9NQZ8	0.32223
P0C206	Q96D03	0.30884
P0C206	Q15327	0.48365
P0C206	Q7L8S5	0.39413
P0C206	P52657	1.2318
P0C206	Q9NR83	0.38208
P0C206	O14713	0.54989
P0C206	P50995	0.9443
P0C206	Q6ZUV0	0.03673
P0C206	Q7L3S4	0.33647
P0C206	Q8WTT2	0.38413
P0C206	Q8N9R6	0.029276
P0C206	Q7Z5Q5	0.11481
P0C206	Q9H7N4	0.84733
P0C206	Q6NZ36	0.42986
P0C206	Q9H492	0.36266
P0C206	Q9UH99	0.27506
P0C206	Q6ZMK1	0.21715
P0C206	Q15172	0.39529
P0C206	Q9NXT0	0.31562
P0C206	Q8TC05	0.7679
P0C206	Q3KP22	0.16813
P0C206	Q8TE04	0.50374
P0C206	Q8IV48	0.035121
P0C206	Q9BQ65	0.47744
P0C206	Q8N9E0	0.68336
P0C206	Q96CX2	0.11906
P0C206	Q9UKL3	1.0696
P0C206	Q8NHG7	0.76257
P0C206	O15382	0.1146
P0C206	O43752	0.40145
P0C206	P51861	0.44523
P0C206	Q99598	0.1161
P0C206	Q5VVM6	0.091009
P0C206	P22531	0.60746
P0C206	Q96LI5	0.6228
P0C206	P48147	0.51163
P0C206	Q96T59	0.15893
P0C206	Q5T5B0	0.45994
P0C206	O75787	0.29734
P0C206	O94822	0.2338
P0C206	Q9NRX3	0.81087
P0C206	Q14CX7	0.47465
P0C206	Q6XD76	0.40647
P0C206	Q9BTE7	0.58919
P0C206	I3L1E1	0.33839
P0C206	P46781	0.95287
P0C206	O14529	0.47387
P0C206	Q3LI54	0.45994

P0C206	P30279	0.82797
P0C206	Q8NEE0	0.45994
P0C206	Q9BQY9	0.61178
P0C206	Q96B97	0.46928
P0C206	Q96JE9	0.3022
P0C206	P78560	0.64285
P0C206	O95983	1.4182
P0C206	P62328	1.0506
P0C206	Q8NE18	1.277
P0C206	Q00613	0.98359
P0C206	O00470	0.69206
P0C206	Q6PII5	0.4668
P0C206	P35226	0.71414
P0C206	Q9H0R1	0.32484
P0C206	Q13111	1.1911
P0C206	Q9UKL0	1.3089
P0C206	P0C7T4	0.45994
P0C206	Q9UBX2	0.386
P0C206	Q04446	0.30624
P0C206	O75818	0.40502
P0C206	Q6ZTZ1	0.3899
P0C206	Q96DB2	0.37553
P0C206	O75608	0.24089
P0C206	Q9UHR5	1.3822
P0C206	Q9NRG4	0.92332
P0C206	Q6PJP8	0.9054
P0C206	Q86X55	0.76772
P0C206	Q5T0W9	0.2538
P0C206	O60934	0.27105
P0C206	Q9Y6G5	0.44439
P0C206	P14406	0.26143
P0C206	Q9P2X7	0.45994
P0C206	Q0D2H9	0.38935
P0C206	Q8N3F0	0.44772
P0C206	Q8N0T1	0.52163
P0C206	O76075	0.1568
P0C206	Q9C0D7	0.55202
P0C206	A6H8Y1	0.86067
P0C206	Q02548	0.26612
P0C206	Q5T753	0.45994
P0C206	Q8N1X5	0.45994
P0C206	O00629	0.71595
P0C206	Q63HN1	0.39628
P0C206	Q9P021	0.28883
P0C206	O75604	0.4099
P0C206	O60684	0.57093
P0C206	P52737	0.86047
P0C206	Q16836	0.44334
P0C206	O95232	0.55195
P0C206	P62266	0.93565
P0C206	P02008	0.38521
P0C206	Q9Y5M6	0.45994
P0C206	Q9Y2L5	0.14054
P0C206	Q02543	0.67945
P0C206	Q9BUH6	0.58209
P0C206	Q9Y4I5	0.37023
P0C206	Q9H8T0	0.58791
P0C206	Q9UKA1	0.34251
P0C206	Q9BTW9	0.29062
P0C206	Q9HB96	0.99145
P0C206	Q496Y0	0.27498
P0C206	Q2V2M9	0.43519
P0C206	Q9P2K1	0.34621
P0C206	Q14232	0.17859
P0C206	P49754	0.66539
P0C206	P60409	0.72851
P0C206	O43493	0.7849
P0C206	Q0VDE8	0.54291
P0C206	O75469	0.30603
P0C206	Q8WUB2	0.34257
P0C206	P52565	0.1236
P0C206	Q9BRP7	0.28324
P0C206	Q8N1I8	0.45994
P0C206	Q99615	0.92277
P0C206	Q9BQ89	1.2539
P0C206	Q6ZN32	0.43565

P0C206	A2IDD5	0.65648
P0C206	Q9NR56	0.47843
P0C206	P63167	1.4528
P0C206	P59923	0.40539
P0C206	Q66LE6	0.55584
P0C206	Q8N5L8	0.97801
P0C206	E9PAV3	0.55374
P0C206	Q13057	0.28683
P0C206	Q8WXB4	0.6229
P0C206	Q9UK33	0.20949
P0C206	Q15170	1.5389
P0C206	Q9Y6K5	0.19606
P0C206	Q15691	0.82927
P0C206	P36542	0.32021
P0C206	P41567	0.89126
P0C206	Q8IWA0	0.32773
P0C206	Q8IYI8	0.41963
P0C206	P0CJ92	0.27537
P0C206	Q9UMX3	0.62011
P0C206	O94811	0.59525
P0C206	Q96DX8	0.114
P0C206	O60477	0.68027
P0C206	P09467	0.37604
P0C206	Q96JI7	0.74793
P0C206	A5D6W6	0.78576
P0C206	O75081	1.399
P0C206	Q9Y2V2	0.48246
P0C206	Q9H1K0	0.14266
P0C206	Q96JK9	1.0598
P0C206	Q8NBR6	1.6069
P0C206	Q8NE86	0.37604
P0C206	A8MUA0	0.37224
P0C206	Q9P2F9	0.25557
P0C206	Q9BY77	0.40469
P0C206	Q8WXI9	0.73503
P0C206	O75533	0.27126
P0C206	Q5TCQ9	0.6571
P0C206	Q9H6L5	0.76953
P0C206	Q86VW0	0.32274
P0C206	P14866	0.40398
P0C206	Q8NFW9	0.45906
P0C206	P34932	0.27921
P0C206	Q9BXQ6	0.45994
P0C206	Q9BR77	0.48235
P0C206	Q9NZY2	0.45994
P0C206	Q9Y315	0.6021
P0C206	Q9C0H6	0.39455
P0C206	C9JE40	0.88391
P0C206	O60826	0.7604
P0C206	Q4G0A6	1.2081
P0C206	Q9NRR3	0.69292
P0C206	P29374	0.29415
P0C206	Q9Y2Q1	0.54871
P0C206	P52655	1.1798
P0C206	Q9BQP7	0.25419
P0C206	Q96QR8	0.36919
P0C206	Q99619	0.6544
P0C206	Q8N910	0.065439
P0C206	Q92665	0.8929
P0C206	Q96CT7	0.10345
P0C206	Q5HYW2	0.77913
P0C206	Q8IYD8	0.47941
P0C206	O00255	0.21625
P0C206	Q9UL58	0.63014
P0C206	Q8N0U4	0.36673
P0C206	Q00536	0.096644
P0C206	Q9NX55	0.33714
P0C206	Q96HZ7	0.45994
P0C206	O95397	1.1213
P0C206	Q9H9L3	0.51143
P0C206	Q8N2C7	0.86465
P0C206	O60732	0.44439
P0C206	O95997	1.029
P0C206	P40692	0.062407
P0C206	Q9H081	0.86382
P0C206	Q658K8	0.61431

P0C206	Q9Y2D9	0.86737
P0C206	O95999	0.2467
P0C206	Q8IWV1	0.34164
P0C206	Q9HDB8	0.419
P0C206	Q9BU19	0.20949
P0C206	Q8TEQ6	0.68576
P0C206	P61966	0.40222
P0C206	Q495Y8	0.51209
P0C206	Q9UIL1	0.86178
P0C206	Q9H7B4	0.23754
P0C206	Q6ZVW7	0.10428
P0C206	Q53G26	0.68745
P0C206	Q8WTR7	0.75727
P0C206	Q9H2H0	0.32324
P0C206	Q9NRI7	0.45994
P0C206	P60413	0.72851
P0C206	Q13045	1.2968
P0C206	Q7Z7H8	0.25126
P0C206	Q04726	1.1484
P0C206	Q9Y4C2	0.72535
P0C206	Q9UHB7	1.4275
P0C206	Q9UMS6	0.73345
P0C206	Q9NVD3	0.039369
P0C206	Q8WXE9	0.67857
P0C206	Q8N4U5	0.10465
P0C206	Q9NX40	0.5858
P0C206	P21980	0.36968
P0C206	Q99623	1.2601
P0C206	P49720	0.35315
P0C206	P61978	0.89724
P0C206	Q03933	0.79318
P0C206	P04264	0.35707
P0C206	Q9Y6H1	0.95874
P0C206	Q99717	0.47756
P0C206	Q9H1Z4	0.54755
P0C206	Q9Y697	0.68577
P0C206	P15559	0.15856
P0C206	Q9UMQ3	0.82561
P0C206	Q9UEE9	0.55774
P0C206	P17019	0.53784
P0C206	Q8IWB7	0.23807
P0C206	Q5VIY5	0.53104
P0C206	Q5XG85	0.45994
P0C206	P63162	0.61452
P0C206	P19388	0.70435
P0C206	A6NFY7	0.59733
P0C206	Q3LHN2	0.45994
P0C206	Q6ZRF7	0.23685
P0C206	O60870	0.66741
P0C206	Q9Y676	0.71519
P0C206	Q6P387	0.54755
P0C206	Q14602	0.40764
P0C206	Q7Z4H9	0.37786
P0C206	Q9NZ32	0.15042
P0C206	Q5XKK7	0.28971
P0C206	Q9NPA5	0.20949
P0C206	Q6STE5	0.76489
P0C206	O14613	0.18896
P0C206	Q9P2M1	0.66879
P0C206	Q9UBS4	0.4554
P0C206	P61218	0.35813
P0C206	Q9P266	0.52829
P0C206	Q9H5P4	0.51848
P0C206	Q14974	1.1443
P0C206	Q6P1Q0	0.053322
P0C206	P05412	0.78712
P0C206	O95600	0.20949
P0C206	Q5TFE4	0.20214
P0C206	O14777	0.7355
P0C206	Q8IY22	0.21416
P0C206	Q9NWU2	0.11206
P0C206	Q05BQ5	0.53112
P0C206	Q9P2D1	0.26704
P0C206	A2RUB6	0.18514
P0C206	Q9HAV7	0.46229
P0C206	Q5T1R4	0.42473

P0C206	Q8IYH5	0.76632
P0C206	O15534	0.3538
P0C206	Q8IUQ4	0.22286
P0C206	Q8IYF1	0.38657
P0C206	Q2M2I5	0.48752
P0C206	Q9NS64	0.5879
P0C206	O75940	0.60914
P0C206	Q8N684	0.34567
P0C206	O95394	0.81167
P0C206	Q9UKV0	0.54929
P0C206	Q8IYE0	0.72457
P0C206	Q14678	0.62714
P0C206	Q8WW38	0.68378
P0C206	P0CJ75	0.67101
P0C206	P46100	0.83537
P0C206	Q658T7	0.18901
P0C206	P09001	0.63364
P0C206	O43463	0.91428
P0C206	Q8NBC4	0.45994
P0C206	P25963	0.64644
P0C206	Q9BUK6	0.17142
P0C206	Q8N8E2	0.19083
P0C206	O95630	0.3382
P0C206	P46013	0.33788
P0C206	Q13394	0.31811
P0C206	Q8N0S6	0.46426
P0C206	Q9H5L6	0.45127
P0C206	O00567	0.86244
P0C206	O43670	0.27585
P0C206	Q05086	0.34569
P0C206	Q15185	0.55432
P0C206	A6NHJ4	0.57913
P0C206	Q6ZPA2	0.45994
P0C206	Q8N2W9	1.3368
P0C206	Q86XF7	0.60768
P0C206	Q8TBZ0	0.52396
P0C206	Q9NSI2	0.68336
P0C206	O94892	0.27604
P0C206	Q6NSI3	0.95158
P0C206	Q66PJ3	0.71947
P0C206	Q6ZV60	0.45994
P0C206	Q9HC62	0.63043
P0C206	Q9H4T2	0.29094
P0C206	O00268	1.1101
P0C206	P78411	0.23995
P0C206	O95059	0.39967
P0C206	Q14146	0.45994
P0C206	Q86UD1	0.27482
P0C206	C9JLR9	0.52254
P0C206	Q96HA1	1.0038
P0C206	Q9BSQ5	0.381
P0C206	Q9Y5V3	0.86435
P0C206	P19429	0.074674
P0C206	Q8IVF1	0.92123
P0C206	Q9NXV6	0.82838
P0C206	O95867	0.57591
P0C206	A6NI87	0.62538
P0C206	Q460N5	0.53024
P0C206	Q8TAV0	0.89109
P0C206	A6NIX2	1.2574
P0C206	Q9UPP1	0.4974
P0C206	Q9Y3Y4	0.85487
P0C206	Q9HD40	0.60789
P0C206	Q9UQN3	0.59523
P0C206	P0DMC3	0.45994
P0C206	Q86T29	0.27283
P0C206	P13645	0.46879
P0C206	Q9C0J9	0.22782
P0C206	Q9H425	0.47978
P0C206	Q07020	1.0088
P0C206	Q9NW08	0.13762
P0C206	P07311	0.70743
P0C206	Q96J02	0.27072
P0C206	Q8N8A8	0.24418
P0C206	Q13137	1.2675
P0C206	Q9P0M9	0.66322

P0C206	P19419	1.3603
P0C206	Q6PI98	0.81415
P0C206	Q9BWU0	0.63741
P0C206	Q9NWB6	0.68336
P0C206	A2A288	0.59298
P0C206	O14832	0.2659
P0C206	Q6PH85	0.054344
P0C206	Q8NFP0	0.35694
P0C206	P83881	0.78841
P0C206	P50221	0.64237
P0C206	Q96PU8	0.24203
P0C206	Q9UM63	0.43145
P0C206	P80303	0.26454
P0C206	Q3LI83	0.82782
P0C206	Q8IYD1	0.30154
P0C206	Q6ZS27	0.31657
P0C206	P46778	0.5404
P0C206	Q9Y2P0	0.33802
P0C206	Q969G5	0.49389
P0C206	Q8N4M7	0.8892
P0C206	Q9BRS8	0.36917
P0C206	Q96MH2	1.3957
P0C206	Q13951	0.72914
P0C206	Q52MB2	1.1267
P0C206	Q9Y2K5	0.39535
P0C206	P58505	0.5238
P0C206	Q96MT4	0.77288
P0C206	P54198	1.1704
P0C206	Q9UL49	0.38073
P0C206	A2RTX5	0.53365
P0C206	Q8N8U2	0.5251
P0C206	P0CF97	0.52929
P0C206	Q6ZVD7	0.46075
P0C206	Q8TBF2	0.23628
P0C206	Q86VY4	0.58242
P0C206	P78559	0.87252
P0C206	Q14011	0.2529
P0C206	O95561	0.69605
P0C206	Q96SK3	0.38367
P0C206	Q8WYK2	0.42834
P0C206	O75177	0.79201
P0C206	Q9HAT8	0.66492
P0C206	Q96MX3	0.29643
P0C206	O95182	0.20428
P0C206	Q96SY0	0.083767
P0C206	Q8NFJ8	0.51524
P0C206	Q9Y4K1	0.85146
P0C206	A6NP11	0.55334
P0C206	A6NJJ6	0.68336
P0C206	O95070	0.31443
P0C206	Q8IXR5	0.47374
P0C206	P59998	0.21967
P0C206	Q3MIS6	0.52141
P0C206	Q8WXX7	0.38857
P0C206	P28838	0.63855
P0C206	Q15038	1.4303
P0C206	Q9NQ89	0.34123
P0C206	P55212	0.50915
P0C206	Q7Z4H3	0.35284
P0C206	P51816	0.34456
P0C206	Q9UL59	0.32313
P0C206	Q9NUW8	0.81815
P0C206	Q9H6R7	0.13594
P0C206	Q96LA8	0.78589
P0C206	Q9UH90	0.55845
P0C206	Q6ZUI0	0.23887
P0C206	Q9NWF9	0.95069
P0C206	Q3SYF9	0.45994
P0C206	Q6IA86	0.73827
P0C206	Q9BZP3	0.17085
P0C206	Q15173	0.41935
P0C206	Q9H8Y8	0.49329
P0C206	Q6NV74	0.68336
P0C206	P10242	0.99475
P0C206	Q7Z5K2	0.86118
P0C206	Q9NRX4	0.10285

P0C206	Q9BZK8	0.45994
P0C206	P60660	0.34028
P0C206	Q8NCR6	0.44005
P0C206	Q9H1A3	0.44439
P0C206	O00193	1.0445
P0C206	Q5EBM0	0.56883
P0C206	Q8N1G0	0.41273
P0C206	O75764	0.44592
P0C206	Q09666	0.79412
P0C206	Q9BYR4	0.72851
P0C206	Q14442	0.20071
P0C206	Q5T5Y3	0.45536
P0C206	Q9Y6Z5	0.71488
P0C206	Q6ZS17	0.57638
P0C206	Q6MZN7	0.45994
P0C206	P42695	0.49881
P0C206	Q96PS1	0.51435
P0C206	Q5R3I4	0.18354
P0C206	Q92791	0.15615
P0C206	Q96A73	1.0191
P0C206	Q96QU8	1.1459
P0C206	Q8NI51	0.79718
P0C206	Q9H307	0.70902
P0C206	Q2M238	0.12559
P0C206	Q9UIF3	0.26542
P0C206	O14879	0.75182
P0C206	Q9Y2Z2	0.96892
P0C206	O00505	1.1089
P0C206	Q9NXW9	0.35992
P0C206	Q8TD06	0.18407
P0C206	Q12768	0.11388
P0C206	Q9Y614	0.13938
P0C206	Q75NE6	1.1463
P0C206	Q9Y448	1.0902
P0C206	Q9Y462	0.84534
P0C206	Q1ED39	1.0445
P0C206	P14651	0.27385
P0C206	P26196	0.55291
P0C206	Q9H7H1	0.2639
P0C206	Q96T51	0.37623
P0C206	Q92988	0.61456
P0C206	Q8N196	0.81602
P0C206	Q9NS39	0.17902
P0C206	Q0D2K3	0.87061
P0C206	Q9P278	0.90375
P0C206	P59942	0.67835
P0C206	Q9Y3C8	0.40369
P0C206	Q99618	1.1814
P0C206	Q16342	0.28263
P0C206	Q9BT25	0.80565
P0C206	Q9UGM6	0.14938
P0C206	Q96EN9	0.69097
P0C206	Q9BRQ3	0.73285
P0C206	Q8N1Q8	0.60148
P0C206	Q9H2L5	0.87062
P0C206	P08729	1.4732
P0C206	Q8N661	0.64377
P0C206	P50749	0.89964
P0C206	O15499	0.2556
P0C206	Q8TC90	0.79065
P0C206	Q6ZMY9	0.53257
P0C206	Q29RF7	1.1672
P0C206	P53041	0.7766
P0C206	Q3SYA9	0.51591
P0C206	Q9ULJ6	0.90636
P0C206	Q15637	0.42301
P0C206	P10073	0.44562
P0C206	O60220	0.64749
P0C206	Q03468	0.51472
P0C206	Q6XXX2	0.11382
P0C206	Q96K30	0.99188
P0C206	Q3KNT7	0.30509
P0C206	Q8N660	1.4923
P0C206	O60269	0.17214
P0C206	Q8NFZ5	0.99344
P0C206	Q9NW81	0.10451

P0C206	Q96KP1	0.419
P0C206	Q5HYW3	1.0332
P0C206	Q8WZ33	1.0745
P0C206	Q52LR7	0.64687
P0C206	Q53GL7	0.60105
P0C206	O94850	0.41823
P0C206	Q5JXC2	0.16439
P0C206	Q96BP2	1.3466
P0C206	B2RUZ4	1.2147
P0C206	Q96AA8	0.49055
P0C206	O15265	1.2917
P0C206	Q9H9R9	0.16163
P0C206	Q68EA5	0.55028
P0C206	Q9BYG5	0.79147
P0C206	P23409	0.087497
P0C206	Q96JF6	0.27659
P0C206	Q9UK59	0.053719
P0C206	P0C853	0.45994
P0C206	Q9BPZ2	0.59481
P0C206	Q7RTT3	0.26627
P0C206	Q3SY52	0.32283
P0C206	Q96G75	0.076523
P0C206	Q9Y383	0.62702
P0C206	Q6PGQ7	1.3886
P0C206	Q9HCX3	0.26061
P0C206	Q86VQ0	0.52885
P0C206	A6NFQ2	0.63765
P0C206	Q9NYP9	0.53848
P0C206	A6NFD8	0.32363
P0C206	Q16890	0.48262
P0C206	Q9HCI6	0.43332
P0C206	Q14457	0.67604
P0C206	O43318	0.65041
P0C206	Q9Y5Z7	0.54355
P0C206	Q9NVH6	0.31804
P0C206	P61568	0.44044
P0C206	P56277	0.82577
P0C206	P0C852	0.45994
P0C206	Q8N680	0.20949
P0C206	Q8WX93	0.38321
P0C206	Q9NSQ0	0.36238
P0C206	Q86VN1	0.65634
P0C206	O75175	1.4632
P0C206	O60493	0.22931
P0C206	A1L168	0.68336
P0C206	Q15021	1.0189
P0C206	Q96PY6	0.46267
P0C206	Q96JN2	0.72128
P0C206	O75586	0.83148
P0C206	A6NDD8	0.90881
P0C206	Q8IZW8	0.49972
P0C206	Q9UBC0	0.23085
P0C206	P36551	0.16535
P0C206	Q96EZ4	0.45994
P0C206	P63151	0.43061
P0C206	O60828	0.99879
P0C206	P59090	0.45994
P0C206	Q15654	1.0726
P0C206	Q96P70	1.149
P0C206	Q16666	0.70329
P0C206	P15336	0.9487
P0C206	P19338	0.56667
P0C206	P53673	0.51208
P0C206	Q9HC44	1.435
P0C206	P49755	0.19472
P0C206	P12980	0.96557
P0C206	Q86T23	0.055708
P0C206	Q6PDB4	0.58046
P0C206	Q8NCU1	0.26306
P0C206	Q86UD4	0.27655
P0C206	Q6ECI4	0.41273
P0C206	Q2VPJ9	0.68336
P0C206	O00444	0.28609
P0C206	P62263	0.53273
P0C206	Q9NP98	0.5815
P0C206	Q5JPH6	0.56976

P0C206	Q53EQ6	0.56891
P0C206	P0CJ86	0.386
P0C206	Q96TA1	0.45104
P0C206	Q9UJW8	0.29181
P0C206	Q6UW56	0.25807
P0C206	Q9NP81	0.3615
P0C206	Q9GZP4	0.02976
P0C206	O95886	0.57042
P0C206	P55327	0.40226
P0C206	O00232	0.092063
P0C206	Q9NZJ4	0.18688
P0C206	Q17RM4	0.39376
P0C206	Q9NQG5	1.2325
P0C206	Q6ICB4	0.30783
P0C206	P26358	0.50204
P0C206	Q5JRA6	0.032758
P0C206	Q969H0	0.73456
P0C206	Q27J81	0.29328
P0C206	Q9H320	0.91525
P0C206	Q6WBX8	0.56512
P0C206	Q13536	0.45994
P0C206	Q12901	0.43551
P0C206	Q9UPQ9	0.40862
P0C206	O75690	0.45994
P0C206	Q969J2	0.32173
P0C206	Q86TU7	0.36308
P0C206	Q8TAB5	0.68336
P0C206	P60410	1.0016
P0C206	Q9BUB7	0.73128
P0C206	Q86WU2	0.073055
P0C206	P06746	0.67989
P0C206	P29728	0.15181
P0C206	P52738	0.30066
P0C206	Q1W209	0.45994
P0C206	P30154	0.42373
P0C206	Q8N7G0	0.43565
P0C206	Q15653	1.0885
P0C206	Q96CM4	0.23855
P0C206	O75157	0.36156
P0C206	Q9UIH9	0.55088
P0C206	Q16822	0.16136
P0C206	Q9UFW8	0.55794
P0C206	O95848	0.65973
P0C206	P48553	0.1353
P0C206	Q13243	0.67525
P0C206	Q12874	0.5154
P0C206	Q9Y2J8	0.58482
P0C206	Q16629	0.62748
P0C206	Q96RM1	0.45994
P0C206	Q7KZF4	0.96832
P0C206	Q92541	1.1349
P0C206	Q04727	0.76896
P0C206	Q8TBZ6	0.4219
P0C206	Q96NU1	0.11775
P0C206	P62273	0.82478
P0C206	Q8IU53	0.47048
P0C206	Q86U42	0.58898
P0C206	Q92800	0.63799
P0C206	P0C2Y1	0.17444
P0C206	Q3LHN0	1.0016
P0C206	Q6P656	0.14473
P0C206	Q9BYD5	0.108
P0C206	Q99698	0.29216
P0C206	Q2M389	0.28525
P0C206	Q15572	0.80593
P0C206	Q709C8	0.64944
P0C206	Q15631	0.75011
P0C206	Q9UJY4	0.64329
P0C206	P55055	0.39149
P0C206	P17027	0.27283
P0C206	H3BMG3	0.27581
P0C206	Q8TF09	0.7026
P0C206	Q9NWQ4	0.60952
P0C206	P54098	0.51246
P0C206	Q8WVV9	0.088662
P0C206	Q9Y2B5	0.31806

P0C206	P51508	0.33397
P0C206	P57086	0.56824
P0C206	Q92670	0.31064
P0C206	Q9Y2G4	0.26133
P0C206	Q9UER7	1.0211
P0C206	Q66K80	0.45994
P0C206	Q00610	0.93224
P0C206	Q8N8I6	0.45994
P0C206	Q9H6U6	0.95531
P0C206	Q6QNY1	0.84053
P0C206	Q96MY7	1.2292
P0C206	P00915	0.24082
P0C206	Q9Y3C1	0.31029
P0C206	Q5THK1	0.083824
P0C206	Q8NFT6	0.22346
P0C206	Q8N7M2	0.55028
P0C206	Q96QG7	0.39214
P0C206	B7Z1M9	0.014963
P0C206	O60765	0.36178
P0C206	Q5W1A9	0.48572
P0C206	Q9BZD4	0.90396
P0C206	Q9NPI6	1.0231
P0C206	Q7LBC6	0.26783
P0C206	P62314	0.76079
P0C206	Q6ZRS2	0.51058
P0C206	Q6P2C8	1.0826
P0C206	Q3SY05	0.0422
P0C206	P62316	0.76246
P0C206	P20248	1.1559
P0C206	Q9P215	0.32086
P0C206	O96007	0.78161
P0C206	Q13278	0.64714
P0C206	A8MUU9	0.34884
P0C206	Q5SXM1	0.41197
P0C206	Q8IWE4	0.317
P0C206	Q5H9B9	0.045861
P0C206	P19012	0.78982
P0C206	O43390	0.47776
P0C206	Q5VWM5	0.81386
P0C206	Q86U28	0.077271
P0C206	Q6P597	0.27115
P0C206	Q8N9R0	0.45994
P0C206	Q5BKU9	0.095875
P0C206	A6NKN8	0.95298
P0C206	Q6ICG6	0.18351
P0C206	Q8IWZ3	1.0245
P0C206	Q8IXT1	0.32349
P0C206	P60900	0.32303
P0C206	O43734	0.68336
P0C206	O75618	1.3068
P0C206	A1A580	0.70835
P0C206	O00625	0.64288
P0C206	Q9Y3C7	1.0339
P0C206	Q9BRJ7	0.71047
P0C206	A6NEY3	0.93098
P0C206	P43268	0.55392
P0C206	Q96T23	0.81684
P0C206	Q96LR5	0.7632
P0C206	Q5TAA0	0.040521
P0C206	Q8NE31	0.17286
P0C206	P61201	0.62358
P0C206	Q9UHC1	0.54636
P0C206	Q96DE0	1.0862
P0C206	Q96MG7	0.99205
P0C206	Q6ZW13	0.45994
P0C206	Q9UBD5	0.64381
P0C206	Q9H761	0.093689
P0C206	I6L9F6	0.25827
P0C206	Q9HCG1	0.42023
P0C206	O75467	0.28839
P0C206	Q8N999	0.3399
P0C206	Q9NVR7	0.28514
P0C206	Q9BS34	0.28876
P0C206	Q96C00	0.55449
P0C206	Q92922	1.0752
P0C206	Q8NHZ7	0.55385

P0C206	Q6UVJ0	1.0219
P0C206	Q9BSF4	0.0023501
P0C206	O15397	0.98986
P0C206	Q9BUI4	0.31323
P0C206	Q5H9R4	1.0721
P0C206	Q6KB66	0.30526
P0C206	Q8IZY5	0.21369
P0C206	Q9UDY2	1.0609
P0C206	Q02878	1.1159
P0C206	Q70EL1	0.47204
P0C206	P62837	0.93071
P0C206	O60318	0.63092
P0C206	Q96JB2	0.96883
P0C206	Q13895	0.92999
P0C206	Q9UHY8	0.40338
P0C206	Q9H9E1	0.36017
P0C206	Q15306	0.88347
P0C206	Q9Y5Z9	0.39263
P0C206	O14531	0.24153
P0C206	Q7Z7F1	0.29253
P0C206	Q9UHZ2	0.45994
P0C206	O60831	1.129
P0C206	Q8IUD2	1.0006
P0C206	A8MYP8	0.35945
P0C206	P21953	0.15545
P0C206	Q9BZE7	0.68336
P0C206	P15621	0.53327
P0C206	O75626	0.33872
P0C206	Q05BU3	0.45994
P0C206	P53804	0.84379
P0C206	Q9NVH2	0.36981
P0C206	Q9Y5B9	0.42455
P0C206	Q9NS91	0.39985
P0C206	Q5W5X9	0.39734
P0C206	E9PI22	0.69362
P0C206	P42771	0.59403
P0C206	Q9H6Y2	0.53407
P0C206	P55735	0.65456
P0C206	Q8N1A6	0.019386
P0C206	Q9Y5J9	0.65827
P0C206	Q0VD86	0.54755
P0C206	Q9HA77	0.52634
P0C206	Q6ZSR6	0.33889
P0C206	P09565	0.3124
P0C206	Q96GG9	0.38469
P0C206	Q8NHU2	0.032616
P0C206	Q9Y6Z4	0.15001
P0C206	A6NLF2	0.39141
P0C206	Q9P0J6	0.68062
P0C206	Q9Y5Y2	0.44977
P0C206	P56555	0.69079
P0C206	Q9UKV8	0.82059
P0C206	Q9P2K3	1.3927
P0C206	P38117	0.33071
P0C206	Q9P2R7	0.40907
P0C206	P43360	0.50382
P0C206	Q9NWU5	0.66712
P0C206	Q6ZNE5	0.49781
P0C206	Q8N7K0	0.40539
P0C206	Q8NDZ0	0.20799
P0C206	Q8WXQ3	1.0819
P0C206	Q7Z794	0.46471
P0C206	O94829	1.1898
P0C206	Q569K4	0.17322
P0C206	A8MWP6	0.45994
P0C206	Q13342	0.89721
P0C206	A8K8P3	0.90776
P0C206	Q3LI67	0.45994
P0C206	Q9ULJ3	0.83273
P0C206	P51522	0.32168
P0C206	Q9BZA5	0.94725
P0C206	Q13316	0.34493
P0C206	P17535	1.0243
P0C206	P68543	0.32228
P0C206	Q99909	0.26627
P0C206	Q16543	0.46836

P0C206	Q9H098	0.82754
P0C206	Q9NRL2	1.222
P0C206	A8MSI8	1.3223
P0C206	Q9UPV0	1.8082
P0C206	Q13547	0.78569
P0C206	O94777	0.52097
P0C206	Q96LM6	0.11206
P0C206	Q8NEK8	0.77807
P0C206	Q99550	0.60888
P0C206	P78386	0.45381
P0C206	Q96Q35	0.54627
P0C206	P43355	1.2455
P0C206	Q5T653	0.60285
P0C206	Q8N0Y2	0.29094
P0C206	Q86SY8	0.45994
P0C206	Q9H6V9	0.527
P0C206	Q86YD7	0.42457
P0C206	Q8TAF7	0.51209
P0C206	P34897	0.20651
P0C206	A6NGG3	0.45994
P0C206	Q96JN8	0.40632
P0C206	Q01844	0.7475
P0C206	Q14186	1.3226
P0C206	Q6ZP65	0.24207
P0C206	P13056	0.81925
P0C206	Q9Y2T2	0.69821
P0C206	Q99962	0.32481
P0C206	P04792	0.41545
P0C206	Q8NHR7	0.26593
P0C206	Q8IZT6	0.54086
P0C206	Q96SD1	0.17067
P0C206	Q13066	0.23762
P0C206	Q16531	0.74861
P0C206	Q9UI66	0.45994
P0C206	Q9BSI4	0.50666
P0C206	Q7Z624	0.20259
P0C206	P17023	0.29427
P0C206	Q9NPC3	0.26363
P0C206	Q15035	0.25162
P0C206	Q7LG56	0.33395
P0C206	Q9P267	0.31141
P0C206	A7E2V4	1.4143
P0C206	Q13416	0.60613
P0C206	Q96KM6	0.41956
P0C206	P49715	0.22832
P0C206	Q9H410	1.0647
P0C206	Q14746	0.51887
P0C206	Q8N0Z6	0.52183
P0C206	Q9BWN1	0.68336
P0C206	Q6ZN30	0.42433
P0C206	Q9UBX3	0.37001
P0C206	Q9UKJ5	0.42177
P0C206	O95671	0.63932
P0C206	Q8N729	0.35747
P0C206	Q8TAT6	0.6314
P0C206	Q6ZS49	0.45994
P0C206	Q9C0F0	0.58025
P0C206	P35754	0.42635
P0C206	Q03936	0.53574
P0C206	Q9BQ87	0.58122
P0C206	O94776	1.177
P0C206	Q9ULL5	0.45994
P0C206	Q9UBZ4	0.29883
P0C206	Q8N1L1	0.45994
P0C206	Q96G27	0.49089
P0C206	P31268	0.38707
P0C206	P36956	0.27605
P0C206	Q9UKA2	0.59519
P0C206	Q13398	0.52124
P0C206	Q9UGK8	0.80995
P0C206	Q9H9L7	0.40402
P0C206	P07320	0.93459
P0C206	P17483	0.16045
P0C206	Q9Y4U1	0.19633
P0C206	Q14140	1.5563
P0C206	P10072	0.3328

P0C206	P29373	0.24027
P0C206	Q86TX2	0.24718
P0C206	A2A368	0.039454
P0C206	Q6ICG8	1.3903
P0C206	P42772	0.90994
P0C206	Q9HCS4	0.25075
P0C206	Q96MB7	0.48821
P0C206	Q9H503	0.46075
P0C206	Q6P1X6	0.75667
P0C206	P98170	0.33575
P0C206	Q8N888	0.83536
P0C206	Q7Z6G3	0.54852
P0C206	P50991	0.411
P0C206	Q96DZ1	0.35861
P0C206	P62495	0.55699
P0C206	Q6ZTI0	0.45994
P0C206	A6QL64	0.55812
P0C206	Q13596	0.41023
P0C206	P30305	0.51785
P0C206	O15164	0.32369
P0C206	Q8IUW3	0.85025
P0C206	Q93009	0.4233
P0C206	Q9H668	0.3687
P0C206	Q5T5F5	0.45994
P0C206	O75494	0.5249
P0C206	Q5SVJ3	0.2338
P0C206	P19484	0.6575
P0C206	O94952	0.6443
P0C206	Q9UJ98	0.54911
P0C206	C9JN71	0.39006
P0C206	Q5T953	0.86655
P0C206	Q8NFR7	0.64513
P0C206	Q9UM07	0.58459
P0C206	Q9H9L4	0.36815
P0C206	P0CJ69	0.67101
P0C206	Q17RH7	0.45994
P0C206	Q9BYR6	1.0016
P0C206	Q7RTV3	0.56017
P0C206	Q14591	0.24995
P0C206	Q96A22	0.45221
P0C206	P35237	0.42166
P0C206	Q6PF18	0.15746
P0C206	Q13021	0.29567
P0C206	P49902	0.087987
P0C206	Q8N9U9	0.060157
P0C206	Q9UBL0	0.2437
P0C206	Q6ZUU3	0.45994
P0C206	Q5VWQ0	1.2857
P0C206	O60524	0.9166
P0C206	Q96T92	0.42986
P0C206	P62826	0.67984
P0C206	P12524	0.32741
P0C206	Q9BQE9	0.68913
P0C206	Q9H816	0.70382
P0C206	Q99816	0.52126
P0C206	Q8IUD6	0.51069
P0C206	Q2TAZ0	0.44439
P0C206	Q8IXQ6	0.92623
P0C206	C9JBD0	0.58414
P0C206	Q5JR59	1.3495
P0C206	Q9BZS9	0.45994
P0C206	P82673	0.18636
P0C206	P0CL83	0.68606
P0C206	Q2KHM9	0.20739
P0C206	P41970	1.0167
P0C206	Q5HYI7	1.1742
P0C206	Q9H496	0.21607
P0C206	Q9NWZ5	0.11483
P0C206	O60813	0.38437
P0C206	Q9NQX0	0.12031
P0C206	Q5VWM3	0.085585
P0C206	Q04760	0.06161
P0C206	Q86VE0	1.2946
P0C206	P20962	0.60982
P0C206	O94888	0.55039
P0C206	O75437	0.51743

P0C206	Q8TDH9	0.27092
P0C206	P58512	0.61091
P0C206	Q9NRX2	0.48633
P0C206	Q04725	0.98146
P0C206	Q6AI39	0.74106
P0C206	Q0P6D6	0.03045
P0C206	P0CJ73	0.67101
P0C206	Q9Y3Q8	1.3793
P0C206	A8MTA8	0.24999
P0C206	Q9P1Z2	0.94589
P0C206	Q12968	0.74526
P0C206	P56693	0.63031
P0C206	Q6ZUF6	0.27483
P0C206	Q14590	0.29385
P0C206	Q04917	0.35958
P0C206	P51003	0.20674
P0C206	O95905	0.93793
P0C206	Q537H7	0.069229
P0C206	P62280	0.84312
P0C206	O15524	0.25138
P0C206	Q9P287	0.85041
P0C206	O95155	0.25947
P0C206	Q92993	0.98982
P0C206	P27348	0.43062
P0C206	Q8NBZ0	1.4644
P0C206	Q14699	0.47757
P0C206	P43490	0.30482
P0C206	E7ETH6	0.32476
P0C206	P57740	0.8515
P0C206	Q96GV9	0.56692
P0C206	Q86WV6	0.13154
P0C206	O15205	0.60029
P0C206	Q9BXT5	0.58786
P0C206	Q96S94	0.47475
P0C206	Q6PGN9	0.92335
P0C206	Q6R2W3	0.47807
P0C206	O60613	0.49346
P0C206	Q8N8E1	0.36926
P0C206	P58004	0.46723
P0C206	D6RGH6	1.3583
P0C206	Q12824	0.98361
P0C206	P43364	1.0004
P0C206	P49454	0.66133
P0C206	P18846	0.96544
P0C206	Q5T1B1	1.1299
P0C206	P11441	0.66463
P0C206	A6NN73	0.32306
P0C206	Q16891	0.20144
P0C206	Q9HB07	0.32821
P0C206	Q13158	0.44934
P0C206	P48380	0.45026
P0C206	Q8TAE6	0.35522
P0C206	P49005	0.66594
P0C206	P0CJ89	0.386
P0C206	Q9BZW7	0.41339
P0C206	A6NLC5	0.27556
P0C206	Q9NRY5	1.1724
P0C206	Q9UI08	0.23113
P0C206	Q8WZA9	0.68336
P0C206	O95678	0.37896
P0C206	Q68DY1	0.53784
P0C206	Q9H5V9	0.60085
P0C206	Q96QE5	0.97319
P0C206	O14770	0.56053
P0C206	P22626	0.436
P0C206	Q9UL03	0.99436
P0C206	P49321	0.83618
P0C206	Q9H2U6	0.35839
P0C206	Q9Y592	1.0089
P0C206	Q5T0U0	0.027102
P0C206	O75896	0.48326
P0C206	Q16589	0.55459
P0C206	P01009	0.37949
P0C206	Q9Y376	0.27399
P0C206	Q96BK5	0.65534
P0C206	B4DZS4	0.30093

P0C206	O75558	0.73039
P0C206	A6NFK2	0.14453
P0C206	D6RB28	0.88867
P0C206	O15318	0.81372
P0C206	Q86YP4	0.97844
P0C206	Q9BXH1	0.3776
P0C206	P55771	0.97535
P0C206	Q99996	0.68659
P0C206	Q5JTJ3	0.17879
P0C206	P59036	0.45994
P0C206	Q9HCM7	1.1293
P0C206	P31939	0.38186
P0C206	A6NNW6	0.58577
P0C206	A1A519	0.84457
P0C206	Q9BZ23	0.51497
P0C206	Q92542	0.37049
P0C206	Q2VY69	0.37537
P0C206	P50453	0.69564
P0C206	Q9H5L9	0.10486
P0C206	P41250	0.40305
P0C206	O15525	0.47068
P0C206	Q8WYA0	0.71247
P0C206	P49406	0.54712
P0C206	P26641	0.43345
P0C206	Q9Y3R5	0.7984
P0C206	P54845	0.44281
P0C206	Q9BX70	0.70649
P0C206	Q3LI64	0.45994
P0C206	Q9Y6J9	0.92242
P0C206	A8K830	0.45994
P0C206	Q8NE65	0.47185
P0C206	Q07666	0.49311
P0C206	P17987	0.34931
P0C206	Q8TD19	0.33775
P0C206	Q6ZN06	0.52928
P0C206	A8MQ11	0.27064
P0C206	Q9UET6	0.78754
P0C206	Q9C0B2	0.34993
P0C206	Q96HE9	0.15381
P0C206	Q9UI09	0.08845
P0C206	Q9UNN4	0.56845
P0C206	Q9H0H0	0.2646
P0C206	Q8N0U6	0.45994
P0C206	Q9UI47	0.35308
P0C206	Q13616	0.5787
P0C206	Q86WR0	0.31338
P0C206	Q6NUI1	0.78014
P0C206	O00716	1.1542
P0C206	Q8NA57	0.986
P0C206	Q6ZNB6	0.38992
P0C206	O95751	0.70721
P0C206	Q8N9N8	0.17369
P0C206	Q13330	0.73149
P0C206	Q6ZVX7	0.65428
P0C206	Q96A26	0.18473
P0C206	Q12933	0.59313
P0C206	P15976	0.35432
P0C206	Q6P5R6	0.30234
P0C206	Q6PGP7	0.88537
P0C206	Q14533	0.886
P0C206	Q96BQ5	0.84515
P0C206	Q02040	0.4553
P0C206	P14621	0.017215
P0C206	Q8NFR3	0.34031
P0C206	P34949	0.42497
P0C206	O60884	0.57406
P0C206	Q9BTL3	0.58488
P0C206	Q5TCZ1	0.24648
P0C206	P10768	0.38233
P0C206	Q01201	1.1864
P0C206	Q5W111	0.17909
P0C206	O60299	0.47425
P0C206	A6NNF4	0.4071
P0C206	Q96LT6	0.31725
P0C206	Q8N3I7	0.3313
P0C206	Q9H4I3	0.00066993

P0C206	Q92783	0.7337
P0C206	P49642	0.31987
P0C206	Q86UE3	0.39234
P0C206	Q96G04	0.067794
P0C206	Q99497	0.10505
P0C206	Q9Y4E1	0.63197
P0C206	Q8N7B6	0.57
P0C206	O75529	0.67976
P0C206	Q92925	0.67212
P0C206	A1A5D9	0.84853
P0C206	P61956	0.79842
P0C206	A6NJI1	0.27123
P0C206	O43809	0.63192
P0C206	Q9BYA6	0.45994
P0C206	Q99829	0.3117
P0C206	Q86VX2	1.0265
P0C206	Q9H1H9	0.7666
P0C206	Q9BQS6	0.96806
P0C206	Q9H0I3	0.61188
P0C206	Q86YL5	0.33365
P0C206	Q86XK3	0.85437
P0C206	P0C7T5	0.45264
P0C206	Q86X83	0.60104
P0C206	Q13287	0.62191
P0C206	P49116	0.70739
P0C206	Q9ULX6	0.59579
P0C206	P67775	0.79541
P0C206	Q96BZ8	0.68336
P0C206	O00458	0.88505
P0C206	Q8TB72	0.54766
P0C206	Q969E2	0.64316
P0C206	Q9BWD3	0.88264
P0C206	P41218	0.82589
P0C206	Q9H3J6	0.90521
P0C206	Q96B26	0.6731
P0C206	O15145	0.1868
P0C206	Q5HYR2	0.85318
P0C206	Q15532	1.1108
P0C206	Q6ICB0	0.81632
P0C206	Q17RB0	0.6858
P0C206	Q15050	0.79557
P0C206	Q9UQF2	0.53399
P0C206	Q9H0G5	0.53064
P0C206	Q96KQ4	1.0547
P0C206	Q6PK04	0.99682
P0C206	Q6IPT2	1.2191
P0C206	Q5EBM4	0.20949
P0C206	Q9HB58	0.5606
P0C206	P30622	0.4145
P0C206	P47224	0.29706
P0C206	Q9HD15	0.70263
P0C206	P62857	0.66383
P0C206	Q8N9K7	0.45994
P0C206	P0DKX0	0.477
P0C206	O14686	0.52308
P0C206	Q8IZU1	0.66819
P0C206	Q8IZ13	0.14922
P0C206	Q9BSC4	0.32773
P0C206	Q6P444	0.63251
P0C206	P22736	0.4029
P0C206	A1A4V9	0.34969
P0C206	Q15910	0.52586
P0C206	Q96HM7	0.79714
P0C206	Q86YW9	0.49467
P0C206	Q5VVS0	0.98012
P0C206	Q15014	1.4149
P0C206	P62851	0.91109
P0C206	P0CI01	0.51209
P0C206	Q9P2H0	0.44439
P0C206	Q8WY36	0.49155
P0C206	P43359	1.0367
P0C206	Q9BYN8	0.991
P0C206	P11166	0.57063
P0C206	P30793	0.22707
P0C206	Q86U86	1.2144
P0C206	P11055	0.31733

P0C206	Q5TKA1	0.53705
P0C206	Q9BYQ7	0.91057
P0C206	Q9NZU7	0.48332
P0C206	P19367	0.31308
P0C206	A6NGN4	0.29113
P0C206	O94818	1.0445
P0C206	C9J069	0.066669
P0C206	P29084	1.3909
P0C206	Q1HG44	0.92859
P0C206	A8MQ14	0.36754
P0C206	Q9NZI7	0.36759
P0C206	P50542	0.23937
P0C206	Q460N3	0.42719
P0C206	Q9UHV7	0.57714
P0C206	Q6W349	0.60429
P0C206	Q8TDN6	0.42946
P0C206	Q7Z5W3	0.80472
P0C206	P19532	0.84279
P0C206	Q10587	0.37088
P0C206	Q8N5U6	1.3383
P0C206	Q7L8W6	0.46352
P0C206	Q96KG9	0.4938
P0C206	P41223	0.13465
P0C206	Q9Y2R2	0.24635
P0C206	Q5TYX0	0.41864
P0C206	P78317	0.74048
P0C206	P78316	1.1278
P0C206	O43237	0.23588
P0C206	Q99471	0.94754
P0C206	Q8WVM0	0.68005
P0C206	Q96QA6	0.32773
P0C206	Q8IZ73	0.31172
P0C206	O43242	0.11693
P0C206	Q9Y6G3	0.32344
P0C206	P81877	0.58842
P0C206	Q15699	0.51512
P0C206	Q9Y530	0.3599
P0C206	Q5JTD0	1.2792
P0C206	O00321	0.39625
P0C206	Q15796	0.66595
P0C206	B9A014	0.18893
P0C206	O95996	0.48447
P0C206	P62910	0.65917
P0C206	A8MUZ8	0.59145
P0C206	Q9Y586	0.19007
P0C206	Q969I3	0.60619
P0C206	P04733	0.45193
P0C206	Q8N8F4	0.24913
P0C206	Q5JVF3	0.24315
P0C206	A6NLX3	0.24652
P0C206	Q9BYQ6	0.72851
P0C206	O43865	0.27662
P0C206	P10636	0.13016
P0C206	P0CV98	0.32628
P0C206	Q9UBB9	0.36928
P0C206	Q99741	0.6216
P0C206	Q9H9A5	0.06863
P0C206	Q96A56	1.0261
P0C206	Q96A65	0.29127
P0C206	Q6ZUT4	0.50147
P0C206	O95238	0.36295
P0C206	Q8N4B4	0.22172
P0C206	Q9BTT0	0.34648
P0C206	Q8TAQ2	1.383
P0C206	Q13257	0.86876
P0C206	P54252	0.55534
P0C206	Q15326	1.4408
P0C206	Q96HB5	0.39212
P0C206	Q13487	1.4552
P0C206	Q4VC05	0.63459
P0C206	Q5TYW1	0.26368
P0C206	Q16829	0.34535
P0C206	Q9H446	0.44439
P0C206	Q8N9G6	0.92666
P0C206	O43257	0.47494
P0C206	Q96E14	0.70969

P0C206	Q9Y603	1.3379
P0C206	Q8WV37	0.71616
P0C206	Q8NDV7	0.23684
P0C206	Q86SX3	1.4221
P0C206	Q5SQ13	0.0040292
P0C206	P61582	1.0517
P0C206	Q96H40	0.51926
P0C206	Q8N9W8	1.2191
P0C206	Q5CZC0	1.1397
P0C206	Q9H6K1	0.19652
P0C206	Q9BX26	1.1574
P0C206	Q92973	1.1311
P0C206	Q14596	0.33684
P0C206	Q00994	1.0345
P0C206	Q8IZU3	0.33176
P0C206	Q9Y2G7	0.5788
P0C206	Q9NXR8	0.48153
P0C206	P35326	0.51897
P0C206	P32189	0.25082
P0C206	A6NCD4	0.30056
P0C206	Q5T0J3	0.10335
P0C206	Q92831	0.81883
P0C206	Q96LC9	0.62231
P0C206	Q8WY41	0.55785
P0C206	Q9H0E7	0.37764
P0C206	Q5T5M9	0.11206
P0C206	Q96IS3	0.33461
P0C206	Q9BRH9	0.34446
P0C206	Q96BN8	0.12848
P0C206	Q9BQA5	0.93949
P0C206	O96020	1.0743
P0C206	Q17RC7	0.53082
P0C206	Q6PI47	0.13145
P0C206	Q86TG7	0.78905
P0C206	Q3MJ62	0.29094
P0C206	O95476	0.37816
P0C206	Q8NA92	0.61368
P0C206	Q9Y4P8	0.98447
P0C206	Q7L2K0	0.20624
P0C206	Q86UQ8	0.58122
P0C206	Q5BKZ1	0.61404
P0C206	P49354	0.37173
P0C206	Q8TCJ0	1.008
P0C206	Q9Y3V2	1.0004
P0C206	A2RTY3	0.32017
P0C206	P49427	0.79465
P0C206	Q9HA90	0.68336
P0C206	Q3B726	0.35438
P0C206	Q0VG06	0.71215
P0C206	Q14669	0.4245
P0C206	Q9NNW5	0.65734
P0C206	Q8IV54	0.35556
P0C206	P33076	0.59218
P0C206	O15226	0.81296
P0C206	P26640	0.26931
P0C206	Q9NX65	0.26719
P0C206	Q9BU64	0.4362
P0C206	Q9UIY3	0.094029
P0C206	Q8WWC4	0.70504
P0C206	Q9C0D0	0.45365
P0C206	Q9BQI6	0.28621
P0C206	P30260	1.1408
P0C206	Q8WVJ9	0.67069
P0C206	Q5VTR2	0.75166
P0C206	Q4VX62	0.33955
P0C206	Q9H160	0.59381
P0C206	A2RUQ5	0.18342
P0C206	O14633	0.45994
P0C206	Q86XG9	0.16288
P0C206	Q9BPX7	0.45626
P0C206	P55145	0.48564
P0C206	P24855	0.14065
P0C206	A1L188	0.68242
P0C206	Q14241	0.4043
P0C206	Q9P2D3	0.79245
P0C206	Q5VZR2	1.098

P0C206	P0C879	0.45994
P0C206	Q6YHU6	0.059722
P0C206	P80217	0.75022
P0C206	O00482	0.36546
P0C206	Q8WTZ3	0.56398
P0C206	Q15036	0.28724
P0C206	Q13216	0.51634
P0C206	Q14141	0.2487
P0C206	Q9NRN9	0.26375
P0C206	Q9UPV9	0.87548
P0C206	O95125	0.31582
P0C206	P57055	0.68169
P0C206	Q96HZ4	0.27029
P0C206	A2BFH1	0.28126
P0C206	Q03252	0.43829
P0C206	Q6P3W7	0.24933
P0C206	O94901	0.29372
P0C206	Q96FJ8	0.45994
P0C206	O75791	1.1023
P0C206	Q9Y6X8	0.85674
P0C206	Q02241	0.83505
P0C206	Q13123	0.76216
P0C206	Q5T8I9	0.264
P0C206	P27986	0.55744
P0C206	A1L3X4	0.45994
P0C206	Q8WZ26	0.021157
P0C206	Q96IV0	0.67741
P0C206	Q8N3J9	0.31162
P0C206	P52740	0.26368
P0C206	P38646	0.64224
P0C206	Q8N1G1	0.15957
P0C206	Q14781	0.91292
P0C206	Q8N8H1	0.49281
P0C206	Q8N912	0.82784
P0C206	Q9P2Y4	0.35488
P0C206	O00410	1.2589
P0C206	Q9Y6X9	0.50702
P0C206	Q8WWQ0	0.66188
P0C206	A8MT70	0.58813
P0C206	Q9Y6X3	1.1479
P0C206	Q9H808	0.70051
P0C206	Q9Y4Y9	0.67158
P0C206	Q8NB91	0.39748
P0C206	Q9BYQ2	0.72851
P0C206	Q5VYM1	0.4654
P0C206	Q13233	0.57513
P0C206	O15519	0.31703
P0C206	Q9GZM8	0.16151
P0C206	Q6PB30	0.76895
P0C206	Q8TC84	0.54755
P0C206	Q96KH6	0.53824
P0C206	Q9BRT9	0.42774
P0C206	Q8NFI3	0.32365
P0C206	Q6P2D0	0.30421
P0C206	Q96N16	1.0764
P0C206	Q6ZUA9	0.7285
P0C206	Q9BQ69	0.56812
P0C206	Q9NR50	0.5654
P0C206	Q13620	0.88786
P0C206	Q9BZE9	0.87399
P0C206	Q9Y512	0.20006
P0C206	Q9UI14	0.089521
P0C206	A6NH13	0.37068
P0C206	Q8IZL8	1.0083
P0C206	Q8IZX4	0.43142
P0C206	Q13409	0.61351
P0C206	Q14938	0.3111
P0C206	O00422	1.3793
P0C206	Q9UHX1	0.60489
P0C206	P62993	0.25364
P0C206	Q16254	1.1172
P0C206	Q9BW66	1.1113
P0C206	Q96K62	0.29584
P0C206	Q96E29	0.43518
P0C206	P83111	0.2185
P0C206	Q5ZEY9	0.58368

P0C206	Q6AI08	0.59281
P0C206	Q9UJG1	1.0958
P0C206	P46782	0.68501
P0C206	Q96RU7	0.46593
P0C206	O95045	0.34162
P0C206	Q5T870	0.784
P0C206	O60284	0.45026
P0C206	Q99551	0.79243
P0C206	P53801	0.76447
P0C206	Q8N9B5	0.92155
P0C206	Q6BDS2	0.42287
P0C206	Q7Z3D6	0.056741
P0C206	Q86XN7	0.68336
P0C206	Q9Y5Q9	0.26386
P0C206	O43236	0.42938
P0C206	Q2TAY7	0.33985
P0C206	Q96HJ3	0.68336
P0C206	A4QMS7	0.24641
P0C206	K7EIQ3	0.94197
P0C206	Q8TAT8	0.22504
P0C206	O94762	0.23216
P0C206	Q59F38	0.15994
P0C206	Q5T7N7	0.45994
P0C206	O95628	0.6848
P0C206	Q76KX8	0.53394
P0C206	Q9ULD9	1.1309
P0C206	Q9ULE4	0.574
P0C206	Q8NEL0	0.10668
P0C206	P02728	0.45994
P0C206	Q9Y6X2	0.92682
P0C206	O95153	0.77281
P0C206	Q8N123	0.60181
P0C206	H3BU77	0.49955
P0C206	Q86TJ5	0.30287
P0C206	Q8N4Q1	0.19557
P0C206	A5LHX3	0.23353
P0C206	O43261	0.44439
P0C206	P07315	0.71245
P0C206	Q7Z4Q2	0.99221
P0C206	Q9HAV4	1.1601
P0C206	P40424	0.72465
P0C206	Q8WUQ7	0.13095
P0C206	Q8NBJ4	0.46963
P0C206	Q9NNW7	0.2548
P0C206	Q9P2I0	0.78001
P0C206	Q14738	0.84609
P0C206	Q8IVM7	0.45994
P0C206	Q14145	1.4501
P0C206	Q5RGN0	0.90881
P0C206	A8MPS7	0.093281
P0C206	O43299	1.3855
P0C206	Q9Y5J7	0.43284
P0C206	Q9Y2D8	0.75428
P0C206	P50461	0.27252
P0C206	B4DXR9	0.53359
P0C206	Q13495	1.2196
P0C206	P49591	0.40511
P0C206	Q9NZE8	0.76125
P0C206	Q8IX90	0.8595
P0C206	P23786	0.11495
P0C206	Q9H5Y0	0.67091
P0C206	Q0VDD5	0.45994
P0C206	P06730	0.50635
P0C206	P28749	1.4166
P0C206	P13984	0.65665
P0C206	Q5TA31	0.66137
P0C206	Q969G2	0.27874
P0C206	A6NJV1	0.58232
P0C206	A8MYZ0	0.73709
P0C206	P09496	0.33678
P0C206	Q6ZUG5	0.28595
P0C206	Q9UGJ1	0.44715
P0C206	Q9H706	0.44439
P0C206	Q5BN46	0.15368
P0C206	Q9Y4Z0	0.61692
P0C206	A4GXA9	0.80911

P0C206	Q9UKJ3	0.76689
P0C206	Q8N6F7	0.51035
P0C206	B3EWG3	0.68336
P0C206	Q5T2W1	0.57272
P0C206	Q66K74	0.24452
P0C206	P36952	0.27508
P0C206	Q8N6N3	0.456
P0C206	Q8N813	0.067618
P0C206	P61247	1.0558
P0C206	Q9Y535	0.41794
P0C206	Q5SRD1	0.3211
P0C206	Q6ZSG2	0.3943
P0C206	P0CJ85	0.386
P0C206	Q6PF06	0.87164
P0C206	Q9Y5Q8	0.82232
P0C206	Q0VG73	0.45994
P0C206	Q6P589	0.50566
P0C206	Q9BY32	0.51489
P0C206	Q8WXC7	0.45806
P0C206	Q15814	0.2472
P0C206	Q9H6K4	0.40565
P0C206	O15405	1.0823
P0C206	Q9H5J4	0.65817
P0C206	Q9NVK5	0.27133
P0C206	Q9HAB8	0.67753
P0C206	Q8IZQ1	0.82634
P0C206	P11308	0.65659
P0C206	Q86VS8	0.5236
P0C206	Q9UBW8	0.55205
P0C206	Q8N4S0	0.68336
P0C206	Q9Y283	0.42316
P0C206	Q8IY42	1.0739
P0C206	P48200	0.48106
P0C206	Q9P2R6	1.2624
P0C206	Q9NYA3	0.33373
P0C206	Q7Z340	0.30987
P0C206	Q6TFL3	0.4088
P0C206	Q9NP61	0.26204
P0C206	Q96NM4	0.49155
P0C206	Q8NC69	0.13145
P0C206	Q9UGP5	0.093393
P0C206	O95865	0.48643
P0C206	Q8ND07	1.0516
P0C206	Q7L775	0.40954
P0C206	P57721	0.64715
P0C206	Q9BWM5	0.33811
P0C206	P10075	0.30021
P0C206	P13807	0.42624
P0C206	Q8TDB6	0.63539
P0C206	A6NNP5	0.063046
P0C206	O43504	0.27043
P0C206	P0DL12	0.26658
P0C206	Q15124	0.10658
P0C206	Q7Z3Z0	0.48752
P0C206	Q9NR77	0.60464
P0C206	Q15269	0.27913
P0C206	O75943	0.38337
P0C206	Q6ZUT6	0.30843
P0C206	Q9UJU3	0.25472
P0C206	Q53HV7	0.7954
P0C206	Q9NRY2	0.9571
P0C206	Q9NVI1	0.88149
P0C206	Q9Y4X0	0.24042
P0C206	Q9UK41	0.85399
P0C206	Q96B23	0.95954
P0C206	Q8TAD7	0.68336
P0C206	Q9Y5K5	0.43453
P0C206	Q9P1Q0	0.5191
P0C206	Q03111	1.4734
P0C206	Q9Y6F1	0.4208
P0C206	Q96DM1	0.86405
P0C206	Q9Y587	0.1608
P0C206	A6NDZ8	0.28047
P0C206	Q1A5X7	0.0020418
P0C206	Q96SQ5	0.33635
P0C206	P0C7X3	0.7077

P0C206	A8MWS5	0.028533
P0C206	P18146	0.80419
P0C206	Q3LI58	0.45994
P0C206	Q96KN7	0.077247
P0C206	O14786	0.27449
P0C206	P07992	0.19921
P0C206	Q6N069	0.3054
P0C206	O94929	0.31285
P0C206	A8MXT2	0.088734
P0C206	Q86XW9	0.10589
P0C206	K9M1U5	0.45994
P0C206	Q92624	1.1146
P0C206	P83369	0.64149
P0C206	Q5T124	0.13742
P0C206	P62633	0.79848
P0C206	Q8NEF3	0.33277
P0C206	P12755	0.23314
P0C206	Q9NZL3	0.88507
P0C206	Q96MA1	0.39399
P0C206	O95298	0.67505
P0C206	Q9NRJ2	0.45994
P0C206	O75360	0.53623
P0C206	O75971	1.1498
P0C206	Q8NEA6	0.42268
P0C206	Q96ED9	0.63032
P0C206	Q96JB5	0.41139
P0C206	Q8NEK5	0.33647
P0C206	Q6VUC0	0.5631
P0C206	P30042	0.25299
P0C206	Q8WYA6	0.78514
P0C206	Q15386	0.29649
P0C206	Q9UF83	0.43136
P0C206	O75475	1.432
P0C206	P51398	1.2847
P0C206	Q9HD33	0.92802
P0C206	O43290	0.96369
P0C206	O75534	0.17344
P0C206	P17025	0.32223
P0C206	Q658L1	0.98539
P0C206	O96004	0.50291
P0C206	A6NDV4	0.27403
P0C206	Q9H9H4	0.77542
P0C206	P61586	0.33654
P0C206	Q8N2I2	0.34327
P0C206	Q9P1Y5	0.70647
P0C206	Q8TEP8	0.94936
P0C206	O75069	0.1487
P0C206	P19447	0.21011
P0C206	Q9UHV9	0.90608
P0C206	P22695	0.13561
P0C206	P31946	0.58303
P0C206	Q9BYE0	0.22822
P0C206	Q9HBT7	0.37081
P0C206	O43776	0.61392
P0C206	Q92793	0.46409
P0C206	Q96B18	0.29091
P0C206	Q3KQV3	0.37298
P0C206	Q5JVG2	0.24088
P0C206	Q9UBF6	0.93115
P0C206	Q0VFX4	0.44031
P0C206	Q16587	0.30577
P0C206	Q6EEV6	0.12583
P0C206	P69891	0.41469
P0C206	Q13144	0.53341
P0C206	A6PW82	0.62801
P0C206	Q9BXW3	0.45994
P0C206	A6NHU5	0.16442
P0C206	Q9NVX0	1.2823
P0C206	O43399	0.6701
P0C206	Q9UFG5	0.44439
P0C206	A8MUL3	0.45994
P0C206	Q3LI81	1.0016
P0C206	Q8NC56	0.43573
P0C206	Q9UJJ7	0.45729
P0C206	Q969H6	0.9932
P0C206	P25815	0.63019

P0C206	Q7Z6R9	0.4536
P0C206	P18077	0.43517
P0C206	P61163	0.15677
P0C206	O95816	0.37663
P0C206	Q92616	0.2295
P0C206	Q96NT3	0.4783
P0C206	P14653	0.41435
P0C206	Q96LR2	1.0711
P0C206	P17861	0.73104
P0C206	Q8N2M8	0.892
P0C206	Q9BWK5	0.86655
P0C206	Q9UL01	0.50374
P0C206	Q96EZ8	0.99627
P0C206	Q8N159	0.22989
P0C206	Q2KHR2	0.36338
P0C206	Q9NYH9	0.11186
P0C206	Q8IWC1	0.77621
P0C206	Q01534	0.33807
P0C206	Q96LW7	0.90031
P0C206	A6NJL1	0.29094
P0C206	O76014	0.40644
P0C206	P54259	0.99992
P0C206	Q8N1B3	0.69758
P0C206	P10589	0.47486
P0C206	Q9BT81	0.50896
P0C206	Q8N6T7	0.82703
P0C206	Q92754	0.55569
P0C206	Q06730	0.22742
P0C206	Q14244	0.90662
P0C206	O95777	0.55946
P0C206	Q75MW2	0.70219
P0C206	Q9BZE3	0.5378
P0C206	P22103	0.45994
P0C206	Q9C086	1.5584
P0C206	Q9GZV5	1.1088
P0C206	Q9H583	1.1305
P0C206	Q9NX70	1.2
P0C206	A8MTY0	0.55067
P0C206	Q32M78	0.47573
P0C206	Q8IXQ3	0.4754
P0C206	O60308	0.57086
P0C206	Q9Y4X5	0.55947
P0C206	P0CH99	0.68153
P0C206	Q5TA76	0.45994
P0C206	O75920	1.1722
P0C206	O15091	0.010182
P0C206	Q13769	0.60283
P0C206	Q15773	0.90067
P0C206	P0C7Q3	0.10108
P0C206	Q9NUL5	0.22055
P0C206	O43513	1.2489
P0C206	Q8WV41	0.22921
P0C206	Q86VQ1	0.35605
P0C206	F5H284	0.28126
P0C206	A4D997	0.61306
P0C206	Q6NSZ9	0.29094
P0C206	Q9H4A5	1.1097
P0C206	Q86V48	1.0919
P0C206	P05413	0.32866
P0C206	Q9BWX5	0.53051
P0C206	A5PL33	0.58899
P0C206	Q9NX95	0.57667
P0C206	Q6ZUJ4	0.38058
P0C206	O75346	0.48795
P0C206	Q8N3Y1	0.3556
P0C206	A6NDU8	0.30097
P0C206	O75459	0.30344
P0C206	Q96DU7	0.34786
P0C206	P51580	0.22232
P0C206	O95163	0.80637
P0C206	Q63HK5	0.43031
P0C206	Q12857	0.44611
P0C206	E9PIF3	0.040509
P0C206	Q49AQ9	0.26335
P0C206	O14561	0.39574
P0C206	Q9NPC7	0.24904

P0C206	Q9Y421	0.46527
P0C206	A8MV65	0.74461
P0C206	P0CG29	0.44089
P0C206	Q6P280	0.58588
P0C206	Q69YQ0	0.4797
P0C206	Q86UT5	0.26027
P0C206	Q9BRJ2	0.46635
P0C206	Q92934	0.10885
P0C206	Q6P1K8	0.77891
P0C206	Q8IZC7	0.28803
P0C206	Q9Y316	0.62941
P0C206	Q16775	0.4668
P0C206	Q9H0E3	0.93882
P0C206	Q56VL3	0.70283
P0C206	Q08999	1.5304
P0C206	P62913	0.98242
P0C206	Q8IYI0	0.46038
P0C206	Q5SW24	0.29091
P0C206	Q99638	0.68626
P0C206	Q8NDW8	0.10037
P0C206	Q9C002	0.31135
P0C206	Q9Y675	0.7095
P0C206	Q14135	0.58122
P0C206	Q9H5J8	1.1504
P0C206	Q6PCE3	0.52623
P0C206	P54922	0.6632
P0C206	Q96AT1	0.68336
P0C206	Q8N393	0.3278
P0C206	P30047	0.73194
P0C206	Q96M61	0.44439
P0C206	Q5TYW8	0.1357
P0C206	Q8NEJ9	0.35838
P0C206	Q8N895	0.72557
P0C206	P61927	0.61873
P0C206	P0DI83	0.54567
P0C206	P25789	0.57763
P0C206	Q96N58	0.36429
P0C206	Q9BW04	0.16109
P0C206	Q16548	0.66246
P0C206	Q9H0A8	0.76825
P0C206	Q13867	0.37376
P0C206	Q9UNQ2	0.51306
P0C206	Q9C075	0.35532
P0C206	P32519	1.4189
P0C206	O14907	0.60884
P0C206	P49593	0.2686
P0C206	A4D263	0.34989
P0C206	O14519	0.39447
P0C206	Q6P1L6	0.28459
P0C206	A6NCJ1	0.22547
P0C206	P17022	0.29094
P0C206	Q9BSG1	0.43598
P0C206	Q712K3	0.7632
P0C206	P0DJH9	0.07807
P0C206	Q6ZR85	0.45994
P0C206	Q9NQ32	0.5516
P0C206	Q96FF9	0.99884
P0C206	Q9Y5B6	0.79172
P0C206	Q5T7N2	0.23296
P0C206	Q6Y7W6	0.44981
P0C206	Q569H4	0.68336
P0C206	Q6ZRM9	0.66705
P0C206	Q9NPJ8	0.41349
P0C206	Q9UJP4	0.45214
P0C206	Q6ZMW2	0.31629
P0C206	Q9Y6Y0	0.59049
P0C206	O75151	0.7155
P0C206	Q6ZN17	1.3039
P0C206	P23610	0.45994
P0C206	Q9BS18	0.59081
P0C206	P14649	0.28835
P0C206	Q9Y333	0.58334
P0C206	O95801	0.39019
P0C206	O00161	0.98449
P0C206	Q969R8	0.051817
P0C206	Q7L4I2	1.6549

P0C206	O60356	0.96334
P0C206	Q9H8M2	0.24056
P0C206	O60248	0.63158
P0C206	O14974	0.44896
P0C206	Q9BZE2	1.1149
P0C206	A1L443	1.098
P0C206	Q9NQ29	0.57287
P0C206	Q15853	0.65321
P0C206	Q9NVX2	0.35728
P0C206	Q8TBZ9	1.1146
P0C206	Q9P0W5	0.74087
P0C206	Q9UJY1	0.54865
P0C206	Q9HCE1	0.75031
P0C206	P61353	0.65917
P0C206	Q9Y608	0.43981
P0C206	P17029	0.45471
P0C206	Q9H3K2	0.11973
P0C206	Q1RMZ1	0.42835
P0C206	Q8NDX6	0.30231
P0C206	Q8N655	0.45994
P0C206	B3EWF7	0.45994
P0C206	Q9C0C6	0.83422
P0C206	Q9BWJ2	0.15599
P0C206	Q96D46	0.8006
P0C206	Q15003	0.94646
P0C206	Q5TA82	0.45994
P0C206	Q96QF7	0.28476
P0C206	A1L162	0.21203
P0C206	Q8N365	0.68336
P0C206	Q9NQV7	0.3508
P0C206	Q14839	0.91153
P0C206	Q9HBT8	0.2876
P0C206	Q15742	0.45417
P0C206	P30291	0.26786
P0C206	O76041	0.22807
P0C206	Q8N7R1	0.85189
P0C206	Q8TEA1	0.41502
P0C206	O43918	1.1715
P0C206	P51965	0.7632
P0C206	A6NN90	0.30716
P0C206	P62888	0.6716
P0C206	Q8NFH8	0.53952
P0C206	Q6ZN28	0.77414
P0C206	Q96S66	0.35108
P0C206	Q99689	0.31929
P0C206	Q6ZMV8	0.57235
P0C206	P46020	0.1931
P0C206	P41134	0.40527
P0C206	Q8IVN3	0.88663
P0C206	Q6ZN57	0.29438
P0C206	P23246	0.82752
P0C206	P59780	0.22404
P0C206	Q70Z53	0.7674
P0C206	O95567	0.45994
P0C206	Q9H7S9	0.89838
P0C206	Q96KF2	0.45994
P0C206	Q8N2H4	1.3885
P0C206	Q9H0A9	1.4579
P0C206	P43362	0.1174
P0C206	O75122	0.43077
P0C206	P0DMB2	0.69504
P0C206	Q9NQZ5	0.22924
P0C206	P57071	0.20949
P0C206	Q02556	0.48286
P0C206	Q9H6R4	0.39558
P0C206	Q96H79	0.76613
P0C206	P53677	0.40499
P0C206	Q9NPG2	0.20567
P0C206	Q12837	0.62944
P0C206	O60437	0.91384
P0C206	P14652	0.0066297
P0C206	Q68CL5	0.13637
P0C206	O60304	0.30116
P0C206	Q9NUU6	0.081122
P0C206	O95292	0.13706
P0C206	A8MW95	0.073918

P0C206	P68133	0.17471
P0C206	Q86Y91	0.44633
P0C206	Q14999	1.0127
P0C206	Q03989	0.43297
P0C206	O95954	0.2
P0C206	Q8TE76	0.48497
P0C206	O95989	0.41535
P0C206	G3V211	0.80203
P0C206	Q9NZD2	0.55867
P0C206	P49366	0.42225
P0C206	Q92674	0.46463
P0C206	Q6NVV1	0.37752
P0C206	A6NEJ1	0.26627
P0C206	O14599	0.55054
P0C206	Q5JUW0	0.41139
P0C206	A6NK06	0.26285
P0C206	Q9UM82	1.1809
P0C206	Q9UJT2	0.66399
P0C206	Q9HCZ1	0.3304
P0C206	O14593	0.83548
P0C206	Q5T0D9	0.3764
P0C206	Q8N5H3	0.68336
P0C206	Q92782	0.8729
P0C206	Q6ZR54	0.45994
P0C206	Q9Y3C0	0.39843
P0C206	Q9UK11	0.43186
P0C206	Q96JB3	0.30227
P0C206	Q05215	0.221
P0C206	O00570	0.98762
P0C206	O95073	1.2277
P0C206	Q8WUM0	0.76248
P0C206	Q5SXM2	0.73072
P0C206	Q9H607	0.43129
P0C206	Q6PKG0	0.42969
P0C206	P80294	0.95457
P0C206	Q14532	0.30235
P0C206	A6NMD2	0.17753
P0C206	Q8N5Q1	1.2191
P0C206	Q9BYK8	0.53001
P0C206	Q9UBQ0	0.58132
P0C206	Q6TGC4	0.44306
P0C206	Q8IXR9	0.13021
P0C206	Q8TAP6	1.1059
P0C206	Q16670	0.37883
P0C206	O75427	0.60855
P0C206	P21673	0.27992
P0C206	O75410	0.93825
P0C206	A8MZ59	0.31537
P0C206	Q8N3R9	0.25809
P0C206	Q5SXJ2	1.4923
P0C206	Q969Q6	0.40355
P0C206	Q9BY27	0.55714
P0C206	Q9GZN2	0.52547
P0C206	Q99426	0.29128
P0C206	Q7Z3G6	0.36074
P0C206	Q92785	0.88103
P0C206	Q8WTV1	1.0082
P0C206	P69905	0.31764
P0C206	Q13435	0.78478
P0C206	Q9UQ35	0.55166
P0C206	P59091	0.36311
P0C206	Q9UKT4	1.0116
P0C206	P23511	1.2441
P0C206	Q5EE01	0.75877
P0C206	O14787	1.1115
P0C206	Q8N6Q8	0.62847
P0C206	O60930	0.40794
P0C206	Q3LI72	0.45994
P0C206	Q9HCD5	1.2277
P0C206	P51114	0.087226
P0C206	O15355	1.0554
P0C206	P54274	0.54258
P0C206	Q8N7Y1	0.45994
P0C206	A6NMV5	0.10192
P0C206	H3BQB6	0.98773
P0C206	Q15916	0.20949

P0C206	Q6PJG6	0.85877
P0C206	P42773	0.44139
P0C206	A6NHX0	0.87489
P0C206	Q96EY4	0.46692
P0C206	Q99547	0.73329
P0C206	Q6MZQ0	0.11031
P0C206	O00213	0.45346
P0C206	Q5H9K5	0.17993
P0C206	O60238	0.35843
P0C206	P60608	0.57003
P0C206	Q9NPG3	0.89484
P0C206	Q32MZ4	1.29
P0C206	Q8IZ40	0.7251
P0C206	Q13064	0.42498
P0C206	O15105	0.32356
P0C206	Q9NRL3	0.57188
P0C206	Q8N9H6	0.28047
P0C206	Q92797	0.89236
P0C206	Q8TEW0	0.28039
P0C206	O43615	0.28024
P0C206	P54840	0.51162
P0C206	O76013	0.41992
P0C206	Q9Y247	0.11206
P0C206	P54277	0.16912
P0C206	O43715	0.73058
P0C206	Q8NDW4	0.31449
P0C206	Q8TEV9	0.6528
P0C206	Q9Y2H0	0.18781
P0C206	A6NKD2	0.32628
P0C206	A6NMN3	0.38803
P0C206	Q9NWS9	0.29094
P0C206	Q96SN7	0.25003
P0C206	Q8N812	0.50233
P0C206	O75531	1.0006
P0C206	O00327	0.29725
P0C206	P23490	0.87239
P0C206	Q5JQS6	0.49067
P0C206	O14646	0.81068
P0C206	Q13610	0.24191
P0C206	O75394	0.50183
P0C206	P46060	0.35161
P0C206	Q8TCY9	0.52129
P0C206	P63104	1.0258
P0C206	P11168	0.082967
P0C206	Q9H7P6	1.0834
P0C206	Q6P1K2	1.3082
P0C206	Q9H1A4	0.90004
P0C206	Q3ZCQ8	0.13601
P0C206	Q9NQ03	0.24705
P0C206	Q9BYV9	0.43802
P0C206	Q96QF0	0.69117
P0C206	Q99767	0.37717
P0C206	A8MX80	0.83808
P0C206	Q9P1D8	0.45994
P0C206	Q15599	0.57017
P0C206	Q9Y4J8	0.26098
P0C206	Q969H9	0.45994
P0C206	Q9H9Z2	0.82694
P0C206	P24666	0.19923
P0C206	Q15061	0.32773
P0C206	Q9BYE7	0.99499
P0C206	P43351	0.83984
P0C206	Q4KMZ1	0.30785
P0C206	Q96LX8	0.33799
P0C206	Q9H825	0.32074
P0C206	Q9UIE0	0.34255
P0C206	Q9BTY7	0.33728
P0C206	Q5VVC0	0.38386
P0C206	Q92610	0.30501
P0C206	P82664	1.0238
P0C206	Q13952	0.71425
P0C206	P20132	0.11422
P0C206	O95718	0.32275
P0C206	Q6DKK2	1.2526
P0C206	D6RF30	0.17325
P0C206	Q9Y2X3	0.78046

P0C206	P49792	0.925
P0C206	P78337	0.5322
P0C206	Q6ZU15	0.29437
P0C206	Q6ZMZ3	0.22893
P0C206	Q92622	0.13587
P0C206	Q15025	0.58894
P0C206	O14497	0.71463
P0C206	Q9UPQ4	0.38535
P0C206	Q5W0V3	0.83274
P0C206	P11172	0.50879
P0C206	P51825	1.0651
P0C206	Q8N2C9	0.080872
P0C206	P19013	0.48846
P0C206	Q9UGK3	0.87898
P0C206	Q9H609	0.20949
P0C206	O14681	0.86069
P0C206	Q8IZD4	1.0332
P0C206	P43897	1.2693
P0C206	Q7Z7C7	0.23185
P0C206	P05423	0.29885
P0C206	Q9NVU0	0.46246
P0C206	A1L170	0.45994
P0C206	O60749	0.26957
P0C206	P0C7V5	0.45922
P0C206	Q9P2J8	0.38367
P0C206	Q8TF40	1.009
P0C206	P12036	0.47604
P0C206	Q9H0W9	0.81787
P0C206	P36578	1.0247
P0C206	Q96B45	0.10723
P0C206	P60228	1.0237
P0C206	O96019	1.0613
P0C206	Q5T7P3	0.45994
P0C206	P53803	0.78876
P0C206	Q6UUV9	1.5887
P0C206	Q5BKY6	0.45994
P0C206	Q9HBK9	0.22651
P0C206	Q96N20	0.30524
P0C206	P49770	0.27956
P0C206	Q6ZNG2	0.27096
P0C206	Q9BWG6	0.32077
P0C206	Q13522	0.2544
P0C206	Q13619	0.46921
P0C206	A6NGY1	0.79384
P0C206	Q92784	0.27867
P0C206	Q13884	0.65815
P0C206	Q12983	0.18114
P0C206	Q13569	0.62463
P0C206	P17021	0.30568
P0C206	O43248	0.39474
P0C206	Q6ZU80	0.2035
P0C206	Q13166	0.45994
P0C206	Q8NBT2	0.88733
P0C206	O60682	0.41086
P0C206	Q8TEM1	0.3713
P0C206	Q9BUZ4	0.31045
P0C206	Q6P4D5	0.83905
P0C206	P0CL85	0.26555
P0C206	Q8IYJ3	0.27578
P0C206	Q9NRD1	0.28324
P0C206	Q96FA7	0.91329
P0C206	P49640	0.26603
P0C206	Q9BR11	0.17619
P0C206	P16157	0.30313
P0C206	Q8NBZ9	0.22592
P0C206	P11137	0.13703
P0C206	Q6ZU52	0.44439
P0C206	Q8N8B7	0.13249
P0C206	A6NE21	0.49928
P0C206	O60447	0.39058
P0C206	Q9H814	0.76404
P0C206	Q8WV07	0.40351
P0C206	Q8WUW1	0.25164
P0C206	A8MVW5	1.0311
P0C206	Q701N2	0.45994
P0C206	Q96GC5	0.73505

P0C206	Q14202	0.58004
P0C206	Q7Z3T8	0.23031
P0C206	Q14209	1.2272
P0C206	Q9NX63	0.94407
P0C206	Q96QU4	0.7095
P0C206	Q6PJ61	1.0107
P0C206	Q15651	0.85195
P0C206	P41162	0.43759
P0C206	P27816	0.64364
P0C206	O43345	0.39483
P0C206	Q9Y605	1.5382
P0C206	Q9BPX1	0.21914
P0C206	Q7L3B6	0.30964
P0C206	Q9BTX9	0.91258
P0C206	Q96BD8	0.8166
P0C206	Q1HG43	0.74154
P0C206	Q9BR46	0.77019
P0C206	O15151	0.3502
P0C206	P0C1Z6	1.4615
P0C206	Q8N865	0.30285
P0C206	Q9NVS2	0.74101
P0C206	Q86TJ2	0.70579
P0C206	Q8IYS8	0.6146
P0C206	Q92750	0.74789
P0C206	Q8N6M3	0.65899
P0C206	Q8NF99	0.50258
P0C206	Q96EH3	0.25114
P0C206	P27815	0.43145
P0C206	Q6VEQ5	0.4653
P0C206	Q93015	0.70967
P0C206	Q96HQ2	1.043
P0C206	Q9BTK6	0.58427
P0C206	Q8WYR4	0.46307
P0C206	Q9UK58	0.47475
P0C206	Q8TD35	0.68336
P0C206	Q96NS1	0.32773
P0C206	Q9UKT7	0.48901
P0C206	O60936	0.84415
P0C206	Q8N823	0.40539
P0C206	Q5BKY1	0.24014
P0C206	Q96DF8	0.99071
P0C206	Q5VZB9	0.25921
P0C206	A6NKF1	1.1555
P0C206	Q9UK28	0.57074
P0C206	P06744	0.64303
P0C206	Q8TBN0	0.37709
P0C206	Q52LA3	0.59661
P0C206	Q02080	0.52004
P0C206	P78347	0.68534
P0C206	Q9NWW0	0.33985
P0C206	Q9UHL3	0.13851
P0C206	Q9H0E2	0.28195
P0C206	Q99457	0.57048
P0C206	Q9UKX7	1.1464
P0C206	Q9H3R5	0.64137
P0C206	P61088	0.78602
P0C206	Q5T752	0.45994
P0C206	Q9GZR1	0.51748
P0C206	Q9P260	0.045074
P0C206	Q6P587	0.12096
P0C206	P50502	0.67925
P0C206	Q9UK45	0.65746
P0C206	Q49A33	0.50284
P0C206	Q96MF4	0.38328
P0C206	Q8NAE3	0.43252
P0C206	P82970	0.81314
P0C206	P14324	0.38893
P0C206	Q9Y3R4	0.30627
P0C206	Q8N9T2	0.45994
P0C206	P62861	1.0961
P0C206	P05198	0.64501
P0C206	Q8N1V8	0.03347
P0C206	Q3KNV8	1.1858
P0C206	Q96NY9	0.83894
P0C206	Q8TAL5	0.31499
P0C206	Q93075	0.69053

P0C206	Q12888	0.89379
P0C206	Q8WYJ6	0.3694
P0C206	Q9HD47	0.32373
P0C206	Q2M2Z5	0.94169
P0C206	O75688	0.41466
P0C206	P30084	0.18542
P0C206	Q969S3	0.95232
P0C206	Q16656	0.52242
P0C206	Q13617	0.65279
P0C206	P19838	0.76312
P0C206	O60443	0.073761
P0C206	Q96T49	0.33015
P0C206	Q9ULC6	0.88611
P0C206	Q9Y285	0.66673
P0C206	Q9H089	0.18286
P0C206	Q5T2T1	0.26784
P0C206	Q96NB3	0.73024
P0C206	Q8IWS0	0.80053
P0C206	B1AK76	0.94214
P0C206	Q9P1F3	0.8063
P0C206	O75663	0.58503
P0C206	Q7Z591	1.1596
P0C206	Q86US8	0.66947
P0C206	P0C860	0.51862
P0C206	Q6U7Q0	0.42986
P0C206	A6NNJ1	0.49928
P0C206	Q6ZTY8	0.17069
P0C206	Q5VYV7	0.44439
P0C206	Q14204	0.50882
P0C206	Q99743	0.47581
P0C206	Q86W56	0.84003
P0C206	A8MQ03	0.6472
P0C206	Q9H4S2	0.32678
P0C206	Q16633	0.7264
P0C206	Q14164	0.31474
P0C206	Q8NCQ2	0.64139
P0C206	Q8WWB7	0.37884
P0C206	O43298	0.20949
P0C206	Q8NI08	0.91921
P0C206	Q96SW2	0.67981
P0C206	Q9H0B6	0.78409
P0C206	Q6R327	0.17256
P0C206	P49917	0.1787
P0C206	O14490	0.32645
P0C206	P36508	0.19173
P0C206	Q8IV36	0.68651
P0C206	Q16526	0.533
P0C206	O15514	0.13893
P0C206	Q16082	1.0223
P0C206	Q8IXT2	0.25921
P0C206	Q717R9	0.52768
P0C206	Q8NF91	0.65476
P0C206	Q6PJW8	0.07951
P0C206	Q15054	0.69615
P0C206	Q969V6	1.4963
P0C206	O60925	0.1551
P0C206	Q9NVE4	0.26125
P0C206	Q9UPM8	0.41534
P0C206	Q9NYG5	0.99088
P0C206	Q96PQ6	0.53155
P0C206	Q92979	0.4195
P0C206	Q99581	0.061551
P0C206	Q14919	0.14894
P0C206	Q86SE9	1.3222
P0C206	Q5T280	1.2015
P0C206	O60841	0.12362
P0C206	Q9H9Q4	0.61121
P0C206	Q9H8S9	0.61333
P0C206	Q99612	0.43369
P0C206	Q9H943	0.026514
P0C206	Q9BW85	1.0118
P0C206	Q96FX7	0.3413
P0C206	P05060	0.31992
P0C206	Q9NVR5	0.74813
P0C206	P12883	0.26858
P0C206	Q92843	0.51684

P0C206	P35269	1.1729
P0C206	Q8ND04	0.62314
P0C206	O15516	0.27011
P0C206	Q9NZ81	1.1716
P0C206	Q96GX2	0.7244
P0C206	B1AMM8	0.19356
P0C206	Q9BRR8	0.88502
P0C206	Q9Y3P8	0.51574
P0C206	Q9NYF8	1.3537
P0C206	A6NK53	0.41273
P0C206	Q7Z5J4	0.23444
P0C206	Q9BXN6	0.33985
P0C206	Q6AW86	0.2778
P0C206	Q86X95	1.2736
P0C206	P10828	0.67234
P0C206	Q92686	0.25452
P0C206	Q0D2I5	0.12421
P0C206	Q9Y6J0	0.27507
P0C206	P55854	0.68663
P0C206	O00483	0.20655
P0C206	Q13428	1.1057
P0C206	Q5DJT8	0.26814
P0C206	Q6ZTQ3	0.25131
P0C206	Q5T7W7	0.68336
P0C206	Q9BQE4	0.71613
P0C206	Q96JZ2	0.52286
P0C206	Q6ZN04	0.30202
P0C206	P22415	0.4952
P0C206	A2RUG3	0.82909
P0C206	Q9UNH5	0.8206
P0C206	Q9H4D5	0.60011
P0C206	Q9H568	0.077725
P0C206	A0AUZ9	0.46107
P0C206	Q96FV9	0.8212
P0C206	Q6ZT77	0.35641
P0C206	P62508	0.65003
P0C206	P59037	0.74968
P0C206	P10244	0.71709
P0C206	Q8N3L3	0.86804
P0C206	P05813	0.30684
P0C206	Q9Y5J1	0.25418
P0C206	Q15041	0.047833
P0C206	Q8N6M9	0.38811
P0C206	Q9Y5B0	0.63748
P0C206	Q9Y4P9	0.31929
P0C206	Q8WW59	0.29505
P0C206	Q6P1N0	0.51236
P0C206	Q15650	1.071
P0C206	Q2VPK5	0.84919
P0C206	Q9BXU3	0.63451
P0C206	Q3LI66	0.45994
P0C206	P19387	0.59314
P0C206	O43379	0.34564
P0C206	Q6ZWC4	0.21522
P0C206	Q6PI26	1.2398
P0C206	P25063	0.45994
P0C206	Q2TAL8	0.78803
P0C206	P35711	1.0419
P0C206	A6NGK3	0.31056
P0C206	Q96KN4	0.079756
P0C206	Q9UP83	1.416
P0C206	A8MX34	0.35494
P0C206	Q8WW22	0.56222
P0C206	Q8TA94	0.55886
P0C206	Q9ULV5	0.5032
P0C206	Q9H788	1.1182
P0C206	Q15070	0.34006
P0C206	Q9UJX4	0.87893
P0C206	P08238	0.56017
P0C206	P40426	0.40797
P0C206	P04053	0.39241
P0C206	Q7Z333	0.29647
P0C206	Q13888	0.69243
P0C206	Q86V59	0.51418
P0C206	Q13907	0.36144
P0C206	Q93083	0.47357

P0C206	Q8TAX9	0.20719
P0C206	Q5JWF8	0.097304
P0C206	Q6ICG7	0.45994
P0C206	Q9NX04	0.44035
P0C206	Q0VFZ6	0.68336
P0C206	Q5H9T9	0.4475
P0C206	Q96N77	0.6229
P0C206	P38159	0.54139
P0C206	Q9NSJ1	0.36975
P0C206	Q9ULU8	0.24483
P0C206	P86480	0.45994
P0C206	Q86XI2	0.85207
P0C206	Q9NRR5	0.76918
P0C206	Q05048	0.66085
P0C206	Q7Z4T9	0.84274
P0C206	Q9BZQ2	0.51444
P0C206	A6NKF2	0.7984
P0C206	Q3I5F7	0.17496
P0C206	Q6VN20	0.47516
P0C206	O75446	1.289
P0C206	Q03112	0.3179
P0C206	Q6UWF3	0.65506
P0C206	Q9NWT6	0.4872
P0C206	A5D8V6	0.45627
P0C206	Q5TH74	0.53815
P0C206	P0DM35	0.89436
P0C206	Q8TCB0	0.089889
P0C206	Q7Z7G2	0.41332
P0C206	P53999	1.391
P0C206	Q9ULW6	0.49789
P0C206	Q6ULP2	0.83282
P0C206	P13647	0.9048
P0C206	Q9BXS1	0.31036
P0C206	Q96RN5	1.4104
P0C206	Q15007	0.55485
P0C206	Q5VTA0	0.47258
P0C206	Q9UFE4	0.27522
P0C206	Q8WZ19	0.13338
P0C206	Q6A163	0.40644
P0C206	O15287	1.0982
P0C206	H3BR10	0.45994
P0C206	Q96M63	0.27327
P0C206	Q96LJ8	0.62286
P0C206	O60506	0.29091
P0C206	P09086	0.41071
P0C206	E5RJ46	0.45994
P0C206	O00712	0.28945
P0C206	Q8IV33	0.46021
P0C206	Q9UGN5	0.34006
P0C206	Q5SYE7	0.55766
P0C206	Q9BXF3	0.75752
P0C206	Q5MNV8	0.50849
P0C206	P54368	0.098952
P0C206	Q9BU76	0.66606
P0C206	O43361	0.30608
P0C206	Q8NCT3	0.8329
P0C206	Q693B1	0.096805
P0C206	Q8TF20	0.39483
P0C206	Q9GZX7	0.43649
P0C206	Q8TF74	0.60103
P0C206	Q9ULV3	0.4487
P0C206	Q9BXS5	0.83035
P0C206	P08133	0.76312
P0C206	Q96LT7	0.74854
P0C206	Q9H8G2	0.68553
P0C206	Q9P0W2	0.82375
P0C206	Q9Y3D0	1.1419
P0C206	Q7L5Y9	0.31333
P0C206	Q99081	0.47871
P0C206	Q13296	0.098113
P0C206	P11844	0.90346
P0C206	P0CW21	0.45994
P0C206	Q8N5X7	0.08823
P0C206	Q9H0A6	0.66106
P0C206	O15155	0.27948
P0C206	Q5HYK7	1.3048

P0C206	Q8N4J0	1.0032
P0C206	P56645	0.36894
P0C206	Q8IYA8	0.68336
P0C206	Q96HA8	1.0556
P0C206	Q8NE22	1.0102
P0C206	Q6VY07	0.63012
P0C206	Q8N7H5	1.0424
P0C206	P35321	0.54597
P0C206	Q7Z6J8	0.40895
P0C206	P17035	0.28063
P0C206	Q96C74	0.42051
P0C206	Q9UPY8	1.185
P0C206	Q9NTI7	0.64868
P0C206	Q9H1I8	0.55624
P0C206	Q3ZCQ2	0.45994
P0C206	Q9NVA1	0.2674
P0C206	Q9UQE7	0.29863
P0C206	P60604	0.93071
P0C206	Q16719	0.35141
P0C206	Q5XKL5	0.32773
P0C206	Q96M11	1.0496
P0C206	O76087	0.23762
P0C206	Q8NF64	1.5587
P0C206	Q5JVS0	0.85409
P0C206	Q6RW13	0.88595
P0C206	O96018	0.57378
P0C206	Q9UJW0	0.69544
P0C206	P22681	0.44771
P0C206	Q9NVM1	0.86178
P0C206	Q93045	0.2812
P0C206	Q9NSD9	0.84645
P0C206	Q6ZRH9	0.45994
P0C206	Q13542	0.49143
P0C206	Q13310	0.38907
P0C206	Q6ZV77	0.45994
P0C206	P83916	0.74095
P0C206	O95402	1.1604
P0C206	Q5QJE6	0.99724
P0C206	P50613	0.25345
P0C206	O75153	0.19984
P0C206	Q9BYC9	0.80322
P0C206	Q6Q795	0.45994
P0C206	Q9NR09	0.54087
P0C206	O00165	0.87138
P0C206	Q9BQG0	0.83305
P0C206	Q9H4I8	0.2729
P0C206	Q68G74	0.33607
P0C206	Q86Y97	0.54552
P0C206	Q9BSY9	0.36269
P0C206	Q13618	0.44718
P0C206	Q8WYQ3	0.68242
P0C206	Q9Y3D7	0.23277
P0C206	Q92917	0.92427
P0C206	Q9BZR9	0.45061
P0C206	Q9H147	0.47875
P0C206	Q9UIS9	0.71629
P0C206	Q96Q05	0.47094
P0C206	Q6ZRH3	0.35646
P0C206	P0CW20	0.2511
P0C206	P0C7M3	0.15893
P0C206	P49450	0.31234
P0C206	Q9HA82	0.41229
P0C206	P17010	0.52855
P0C206	Q96S19	0.28471
P0C206	Q9Y4A5	0.9016
P0C206	Q6RSH7	0.4316
P0C206	Q9BUV8	0.12291
P0C206	Q6NT46	0.23762
P0C206	Q86XI8	0.7572
P0C206	O14972	0.35924
P0C206	P0C2W7	0.45994
P0C206	Q6XYB7	0.43565
P0C206	A8MQB3	0.0095262
P0C206	O75564	0.48386
P0C206	Q9GZS3	1.2793
P0C206	O14598	0.8605

P0C206	Q9NVM4	0.3253
P0C206	P16989	0.34645
P0C206	A6XGL0	0.47098
P0C206	P35268	0.78274
P0C206	O95400	0.79279
P0C206	P17568	0.10093
P0C206	O95347	0.60197
P0C206	Q8N9V7	0.7569
P0C206	Q15053	0.057025
P0C206	Q12980	0.68791
P0C206	Q8IXJ9	0.90605
P0C206	A6NFQ7	0.43565
P0C206	Q8NAX2	0.68336
P0C206	P59047	0.36334
P0C206	Q5HYN5	0.29372
P0C206	Q8N344	0.75643
P0C206	Q8NAF0	0.20949
P0C206	P23396	0.71639
P0C206	Q9BVW5	1.2168
P0C206	O95613	0.90754
P0C206	P39023	0.91777
P0C206	Q5T8A7	0.82147
P0C206	O75113	1.0291
P0C206	Q15560	1.277
P0C206	Q8WXA3	0.34631
P0C206	Q13068	0.11267
P0C206	P63000	0.54957
P0C206	O95257	0.42914
P0C206	Q9H3M9	0.37626
P0C206	Q7L0Y3	1.0305
P0C206	Q14106	0.36625
P0C206	Q96EP0	0.27199
P0C206	Q9NSC5	0.57
P0C206	P42166	0.31717
P0C206	P62258	0.86502
P0C206	Q86UQ0	0.29395
P0C206	Q13835	0.29256
P0C206	Q7L9B9	0.32404
P0C206	Q8NHY6	0.42368
P0C206	Q8IW36	0.46793
P0C206	Q96HW7	0.70712
P0C206	Q9BRP1	0.86821
P0C206	O95486	0.53122
P0C206	A8MZ97	0.84707
P0C206	Q5SWL7	0.73382
P0C206	Q0IIN9	0.2444
P0C206	P50549	0.97475
P0C206	Q9UKK3	0.79626
P0C206	O15083	0.69096
P0C206	Q92990	0.22119
P0C206	Q9H0W8	0.59647
P0C206	Q9UNY5	0.49332
P0C206	Q8NHY2	1.2316
P0C206	O95619	0.90177
P0C206	Q969M3	0.32187
P0C206	P20618	0.57107
P0C206	Q96F83	0.55795
P0C206	Q8N9Z2	0.57656
P0C206	A8MZF0	0.68336
P0C206	Q5BKX5	0.68336
P0C206	Q16658	0.18733
P0C206	Q86XE5	0.7395
P0C206	Q9Y543	0.46899
P0C206	Q13573	0.60901
P0C206	Q9ULW3	0.81591
P0C206	Q3LIE5	0.46467
P0C206	A6NEF3	0.93098
P0C206	Q9BWT1	1.0181
P0C206	Q499Z4	0.37429
P0C206	P32243	0.79466
P0C206	Q96LQ0	0.48832
P0C206	Q8IYE1	0.030614
P0C206	A0PG75	0.05827
P0C206	Q8N6V4	0.30866
P0C206	Q8WTW3	0.68148
P0C206	Q969X6	1.3684

P0C206	Q9P1J3	0.45994
P0C206	Q9UKN5	0.43032
P0C206	Q9BZV1	1.2326
P0C206	Q6RFH5	0.36266
P0C206	Q9BWJ5	0.65271
P0C206	Q8IWN7	0.30341
P0C206	P17676	0.85531
P0C206	P0DMB1	0.69362
P0C206	Q99700	0.79644
P0C206	P78413	0.23633
P0C206	Q86WP2	0.59489
P0C206	Q9BVS5	0.37686
P0C206	O94993	0.33056
P0C206	P35080	0.50559
P0C206	Q8N9Z9	0.75125
P0C206	Q9BUN5	0.55751
P0C206	O95990	0.29101
P0C206	Q9Y3C6	0.27642
P0C206	Q9H322	0.68886
P0C206	A6NDE8	0.23762
P0C206	Q53TS8	0.43389
P0C206	Q3LI68	0.87218
P0C206	Q5TA79	0.45994
P0C206	Q6ZVK8	0.49905
P0C206	Q3BBW0	0.20018
P0C206	O76011	0.48752
P0C206	Q5VIR6	0.88905
P0C206	Q13838	0.17632
P0C206	A5YM69	0.023663
P0C206	Q9UPU9	0.35081
P0C206	O00124	0.29443
P0C206	Q15973	0.30557
P0C206	Q8N490	0.51873
P0C206	Q9XRX5	0.44439
P0C206	Q6IBW4	0.36958
P0C206	Q8N769	0.17031
P0C206	Q6L8G8	0.45994
P0C206	Q9Y2R9	0.98732
P0C206	P51397	0.50028
P0C206	Q8WVB3	0.24465
P0C206	Q9BZL1	0.37783
P0C206	Q8IWV7	0.43621
P0C206	Q9UNE7	0.32817
P0C206	Q8IUC3	0.90014
P0C206	P12757	0.44888
P0C206	Q8WU20	0.22549
P0C206	Q8IZP0	0.31158
P0C206	Q6L8G9	0.45994
P0C206	Q4KN68	0.12735
P0C206	P32969	0.78651
P0C206	A6NHS1	0.47057
P0C206	Q5VSR9	0.80268
P0C206	Q9Y255	0.23374
P0C206	A1A4G5	0.45994
P0C206	Q8NC26	0.49425
P0C206	O95831	0.75591
P0C206	Q8N7X2	0.45994
P0C206	Q06265	0.20042
P0C206	O14929	0.88143
P0C206	Q5SZB4	0.11191
P0C206	Q9ULG1	0.48269
P0C206	Q5VT98	0.077528
P0C206	Q9NS69	0.18229
P0C206	Q13405	0.73505
P0C206	P60331	0.72851
P0C206	Q52M93	0.30231
P0C206	Q9HA58	0.45994
P0C206	P43487	0.44794
P0C206	O43296	0.36707
P0C206	Q14181	0.54035
P0C206	Q9BXG8	0.70375
P0C206	Q86WZ6	0.28863
P0C206	Q6UXR8	0.45994
P0C206	P15515	0.26644
P0C206	Q96H20	1.1384
P0C206	Q6ZVE7	0.5365

P0C206	A6NJ46	0.386
P0C206	I3L0S3	0.4602
P0C206	A6NKX1	0.45994
P0C206	H3BQW9	0.5764
P0C206	Q6PGQ1	0.47181
P0C206	Q9NZ52	0.62539
P0C206	Q13425	0.66843
P0C206	O95429	0.27813
P0C206	O94992	1.4055
P0C206	O75112	0.49453
P0C206	Q8TF47	0.51209
P0C206	F5H4B4	0.90147
P0C206	P63208	0.61718
P0C206	O14813	0.40321
P0C206	Q9UI56	0.45994
P0C206	P48668	0.38367
P0C206	P09497	0.52016
P0C206	Q9ULU4	0.78479
P0C206	Q8IZL2	1.014
P0C206	P82909	0.44136
P0C206	Q8IXS2	0.18333
P0C206	Q9UBU7	0.31615
P0C206	Q14139	0.63217
P0C206	P35232	0.60748
P0C206	O43708	0.4337
P0C206	Q8IZ20	0.20949
P0C206	Q9C0F3	0.6229
P0C206	Q5XUX1	0.44439
P0C206	Q13200	0.38561
P0C206	Q05BW5	0.29038
P0C206	P14136	0.36702
P0C206	Q9NR48	0.58159
P0C206	Q13015	0.63598
P0C206	Q8IV03	1.1762
P0C206	Q9UBU9	0.43356
P0C206	O15379	0.63598
P0C206	Q9H1Y0	0.4832
P0C206	O00110	0.30231
P0C206	Q5D0E6	0.82901
P0C206	Q8TAA3	0.26601
P0C206	Q92564	0.21134
P0C206	Q58FF3	0.39062
P0C206	P0C7W8	0.32934
P0C206	Q9GZY1	0.70629
P0C206	Q96CJ1	1.3064
P0C206	Q53HI1	0.59313
P0C206	Q9Y6D5	0.23125
P0C206	Q9BXX3	0.33372
P0C206	P52742	0.36979
P0C206	Q5JT18	0.45994
P0C206	P08047	0.82116
P0C206	Q9UEG4	0.35112
P0C206	A8MV72	0.36237
P0C206	Q9NP66	1.1378
P0C206	A6NJQ4	0.4045
P0C206	Q9Y305	0.67096
P0C206	P17482	1.1847
P0C206	P24278	1.0328
P0C206	Q14CW9	1.0145
P0C206	P18615	0.92636
P0C206	P51959	0.56096
P0C206	A6NFI3	0.28116
P0C206	O14556	0.36735
P0C206	P52597	0.37386
P0C206	O76021	0.5461
P0C206	Q711Q0	0.45994
P0C206	P80723	0.61666
P0C206	Q9BVV2	0.067231
P0C206	Q9BPZ3	0.59815
P0C206	Q9BWW4	0.77466
P0C206	Q13427	0.49771
P0C206	O95373	1.1369
P0C206	P60372	0.72851
P0C206	Q96M43	0.25901
P0C206	Q8IWE2	0.77533
P0C206	O75330	0.2985

P0C206	Q5VZK9	0.28348
P0C206	Q9NZM4	1.1771
P0C206	Q96C23	0.026409
P0C206	P62701	0.41834
P0C206	F8W1W9	0.35075
P0C206	Q8NCD3	0.66428
P0C206	P28070	0.37946
P0C206	Q9Y5J3	0.37147
P0C206	Q96JN0	0.6718
P0C206	H3BNL8	0.93601
P0C206	P13349	0.35328
P0C206	P07900	0.52104
P0C206	Q3YBM2	0.90535
P0C206	P49459	0.81893
P0C206	Q16630	0.22232
P0C206	Q9Y2S0	0.23167
P0C206	Q9NZV7	0.60776
P0C206	P51808	0.57153
P0C206	Q8IYX7	0.8441
P0C206	Q8N972	0.40539
P0C206	Q9UK10	0.40539
P0C206	Q12986	0.41996
P0C206	Q3C1V1	0.68336
P0C206	P29083	1.3429
P0C206	Q5T319	0.45994
P0C206	P04731	0.47357
P0C206	P51610	0.55127
P0C206	Q9P0M2	0.1548
P0C206	Q86SG2	0.35519
P0C206	Q7L4P6	0.41634
P0C206	Q8N488	0.76578
P0C206	A8MU93	0.68336
P0C206	Q7Z7B7	0.89864
P0C206	Q9UPT5	0.7014
P0C206	Q13426	0.42463
P0C206	O75391	0.72377
P0C206	Q6P087	0.29984
P0C206	P35606	0.53525
P0C206	Q8N8D1	0.72728
P0C206	O00264	0.059176
P0C206	Q5VYS4	0.1855
P0C206	Q6ZVQ6	1.2094
P0C206	Q5JUK9	0.22951
P0C206	Q9BVC6	0.73107
P0C206	P55196	0.24886
P0C206	Q14691	0.4
P0C206	Q9H3E2	0.16337
P0C206	Q86SZ2	0.88184
P0C206	O75694	0.74304
P0C206	O00159	0.1801
P0C206	O43900	0.48351
P0C206	Q9UKN8	0.62584
P0C206	A6NJG6	0.43565
P0C206	P0CZ20	0.39488
P0C206	Q96MN9	0.81858
P0C206	Q7Z7G8	0.096706
P0C206	Q9P2K5	0.15233
P0C206	Q96BR9	0.23725
P0C206	Q8WXF8	1.1552
P0C206	Q86XI6	0.22895
P0C206	Q6PJT7	1.081
P0C206	Q14683	0.55246
P0C206	Q8N141	0.5435
P0C206	Q9UHF7	0.67248
P0C206	O95922	0.019576
P0C206	Q86UC2	0.56503
P0C206	Q9Y6A5	0.086982
P0C206	Q6P2E9	1.1336
P0C206	Q5VTH2	0.6284
P0C206	Q96EV8	0.29861
P0C206	Q7RTU5	0.50915
P0C206	Q6NSH3	0.59951
P0C206	Q9UHY7	0.27376
P0C206	Q6ZRT6	0.68336
P0C206	Q9HAP2	0.82693
P0C206	Q8WVZ9	0.50382

P0C206	Q15906	1.0919
P0C206	Q9H944	1.1305
P0C206	Q9HAI6	0.69656
P0C206	Q9H582	0.20949
P0C206	Q9H974	1.3555
P0C206	P61575	0.80216
P0C206	Q9H171	0.22969
P0C206	Q5VWI1	0.98374
P0C206	Q9BTE6	0.63156
P0C206	P05386	1.0234
P0C206	P50616	0.46387
P0C206	Q96KR1	0.37318
P0C206	Q14593	0.5656
P0C206	Q9H175	1.212
P0C206	Q92985	0.57902
P0C206	Q8N4B5	0.68635
P0C206	Q969W0	0.49937
P0C206	Q9HCE0	0.30833
P0C206	Q6ZQT0	0.086017
P0C206	Q9UKH3	0.82824
P0C206	P86478	0.45994
P0C206	Q8N357	0.40828
P0C206	Q9P013	0.50631
P0C206	Q9NWB7	0.77896
P0C206	A8MPV3	0.10664
P0C206	P40855	0.55025
P0C206	Q9Y3B7	1.1155
P0C206	Q9BRD0	0.82072
P0C206	Q9H1H1	0.40703
P0C206	Q7RTU4	0.48008
P0C206	Q5TEC3	0.33372
P0C206	P53370	0.59716
P0C206	Q8N7J2	1.1573
P0C206	Q99445	0.40617
P0C206	O95178	0.60375
P0C206	Q6NSJ2	0.44628
P0C206	Q6RFH8	0.92562
P0C206	Q6IQ32	0.5855
P0C206	P33947	0.4147
P0C206	O60888	0.51926
P0C206	Q53QV2	0.59021
P0C206	Q8N268	0.050805
P0C206	O60885	0.80666
P0C206	Q15011	0.63703
P0C206	P59051	0.16593
P0C206	Q4G0N7	0.66422
P0C206	Q96FW1	0.15129
P0C206	Q5H9F3	0.8047
P0C206	Q8N5P1	0.36729
P0C206	O95707	0.90619
P0C206	Q9UBS8	0.80297
P0C206	Q9UJY5	0.65976
P0C206	O15049	1.084
P0C206	Q14149	0.36145
P0C206	Q7Z5V6	0.48543
P0C206	Q8WXF1	0.96982
P0C206	P63261	0.17471
P0C206	Q6IQ19	0.43059
P0C206	O95190	0.70446
P0C206	P48059	0.38354
P0C206	Q8WUI4	0.49694
P0C206	Q01658	0.60564
P0C206	Q17RY6	0.82071
P0C206	P34896	0.28512
P0C206	Q14160	0.63436
P0C206	P28290	0.5014
P0C206	Q96LK0	0.27468
P0C206	Q96JE7	1.0689
P0C206	Q86XT2	0.19313
P0C206	P22557	0.16499
P0C206	Q6DHV5	0.28137
P0C206	Q107X0	0.51471
P0C206	Q08ER8	0.39689
P0C206	Q9NPJ6	0.92227
P0C206	Q99543	0.63185
P0C206	O00461	0.62557

P0C206	P45381	0.19605
P0C206	Q14676	0.80784
P0C206	P17032	0.31775
P0C206	Q9UHJ3	0.451
P0C206	Q96BJ3	0.67235
P0C206	Q6ZS52	0.45994
P0C206	Q99569	0.47258
P0C206	P35680	0.28977
P0C206	O60239	0.46196
P0C206	F7VJQ1	0.45994
P0C206	Q8N377	0.45994
P0C206	Q7RTU0	0.48008
P0C206	P49789	0.67985
P0C206	Q9Y5A9	0.46655
P0C206	Q96T88	0.42785
P0C206	O15315	0.28773
P0C206	P49711	0.35397
P0C206	Q9NWV8	0.79881
P0C206	P35548	0.6867
P0C206	Q86TS9	0.43447
P0C206	P57081	1.0426
P0C206	Q8TD23	0.71907
P0C206	Q96EK5	0.3185
P0C206	Q9HC78	0.43624
P0C206	Q6NZI2	1.142
P0C206	Q96IY1	1.1014
P0C206	Q8N9H9	0.45994
P0C206	P61571	0.61046
P0C206	P01100	0.52638
P0C206	O14656	0.28006
P0C206	Q96JP2	0.30973
P0C206	Q96F86	0.61752
P0C206	Q71F78	0.53301
P0C206	Q8N7U7	0.43471
P0C206	P50440	0.34045
P0C206	P22090	0.31567
P0C206	Q9H2C1	0.27224
P0C206	Q04206	0.65727
P0C206	Q495M9	0.37243
P0C206	Q06587	0.93393
P0C206	H3BRN8	0.056726
P0C206	P0C5Y4	0.4802
P0C206	O60739	0.38675
P0C206	Q9NV06	0.34724
P0C206	Q4LE39	1.0669
P0C206	Q9P2J3	0.37131
P0C206	Q9UI12	0.15504
P0C206	P16415	0.38175
P0C206	Q86VX9	1.0501
P0C206	Q9H2K0	0.38471
P0C206	P49006	1.0265
P0C206	P53990	0.71323
P0C206	B5ME19	0.28162
P0C206	P86452	0.67443
P0C206	Q7Z4G4	0.19214
P0C206	Q5VVH5	0.79647
P0C206	O75505	0.31537
P0C206	Q8IYX8	0.20337
P0C206	P24390	0.68011
P0C206	B9ZVM9	1.1654
P0C206	Q6PH81	0.24982
P0C206	Q9Y5R5	0.30099
P0C206	P09105	0.40461
P0C206	Q6P047	0.76076
P0C206	O95931	0.9497
P0C206	Q9BYD6	0.57035
P0C206	Q9NQR1	0.82339
P0C206	Q9UHU1	0.45994
P0C206	P32929	0.30483
P0C206	P49257	0.26086
P0C206	Q86VP3	0.49996
P0C206	Q16204	0.29906
P0C206	Q5HY98	0.49074
P0C206	Q9UPW6	0.85473
P0C206	Q92889	0.34715
P0C206	Q9Y2L8	0.55283

P0C206	Q8IVG9	0.18859
P0C206	Q8TEX9	0.65402
P0C206	Q86VI1	0.27642
P0C206	Q86X51	0.44834
P0C206	O95487	0.84112
P0C206	O14508	0.34916
P0C206	P25800	0.36521
P0C206	P84098	0.61276
P0C206	A4D1B5	0.11905
P0C206	Q86WV1	0.71631
P0C206	Q96AQ6	0.93192
P0C206	Q9Y3B8	0.1937
P0C206	Q9NZL4	0.44112
P0C206	P09913	0.63506
P0C206	Q9Y6F6	0.12637
P0C206	A6NFC5	0.51588
P0C206	Q7Z589	1.0104
P0C206	A6NJ88	0.90288
P0C206	P01215	0.29446
P0C206	Q8TDQ7	0.15216
P0C206	Q9HCH0	1.4168
P0C206	P62945	0.67945
P0C206	Q8TDG2	0.16231
P0C206	O60763	0.57347
P0C206	Q9H4B6	0.68876
P0C206	Q8TAT5	0.2905
P0C206	Q69YL0	0.45994
P0C206	Q9NWW7	0.77878
P0C206	O94874	0.93948
P0C206	Q8N8K9	0.24842
P0C206	O75152	0.46784
P0C206	O60260	0.5197
P0C206	P17017	0.28063
P0C206	Q96CN0	0.45994
P0C206	Q9UIF8	1.155
P0C206	Q9UHT4	0.45994
P0C206	Q8IZ63	0.45994
P0C206	Q8IZU0	0.84618
P0C206	Q96MC9	1.1153
P0C206	Q9BTC0	0.51199
P0C206	Q9BYD2	0.78575
P0C206	Q9UG56	0.3986
P0C206	O75817	0.97918
P0C206	Q99460	0.11513
P0C206	P10155	0.52106
P0C206	Q9Y3D2	0.33509
P0C206	O75792	0.97029
P0C206	Q8TDP1	0.51919
P0C206	Q8TED0	0.46792
P0C206	Q9H6W3	0.46751
P0C206	Q8TCD5	0.18959
P0C206	Q9NQR4	0.41447
P0C206	O15225	0.45994
P0C206	Q16740	0.41892
P0C206	O60268	0.30213
P0C206	F5GYI3	1.0063
P0C206	Q9UKI9	1.3562
P0C206	Q01831	0.80848
P0C206	Q7KZ85	0.41251
P0C206	P0CJ88	0.386
P0C206	Q96CW1	0.61226
P0C206	O60535	0.6725
P0C206	Q9P0L1	0.37443
P0C206	Q6ZSR3	0.45994
P0C206	P0C7P2	0.18874
P0C206	Q96MK2	0.084068
P0C206	P52744	0.3559
P0C206	P15923	0.57673
P0C206	Q9BTX1	0.6462
P0C206	P26373	0.86825
P0C206	Q9H211	0.81134
P0C206	Q15942	0.46789
P0C206	Q71UM5	0.77074
P0C206	Q9Y6G9	0.29161
P0C206	Q9BQA9	0.15915
P0C206	Q8TF50	0.30966

4713	P0C206	Q8ND90	0.8325
4714	P0C206	Q5D1E8	0.8771
4715	P0C206	O00198	0.74072
4716	P0C206	Q96RS6	0.33985
4717	P0C206	Q96PK6	0.7538
4718	P0C206	Q01094	0.82878
4719	P0C206	Q3ZCT1	0.33566
4720	P0C206	Q9NZ72	0.12813
4721	P0C206	Q9BQK8	0.13754
4722	P0C206	Q9UIF9	0.80454
4723	P0C206	Q5T036	0.12374
4724	P0C206	Q9P219	0.54844
4725	P0C206	P26998	0.85146
4726	P0C206	Q14152	1.1684
4727	P0C206	Q9Y3B9	0.49809
4728	P0C206	Q15293	0.35233
4729	P0C206	Q16626	0.15499
4730	P0C206	Q15102	0.28067
4731	P0C206	Q9NY33	0.29028
4732	P0C206	Q0PNE2	0.68495
4733	P0C206	Q14686	0.89247
4734	P0C206	Q7Z7K6	0.71407
4735	P0C206	Q9UJ68	0.14179
4736	P0C206	Q8TCC3	0.27125
4737	P0C206	P12882	0.33462
4738	P0C206	P40925	0.54352
4739	P0C206	Q5JPI3	0.68553
4740	P0C206	Q8N8P6	0.3568
4741	P0C206	Q5SXM8	0.22305
4742	P0C206	Q96BT3	0.69807
4743	P0C206	Q969Z0	0.39821
4744	P0C206	O00204	0.50195
4745	P0C206	Q5TA77	0.45994
4746	P0C206	Q9UQR1	1.0078
4747	P0C206	P35637	0.45298
4748	P0C206	O60296	0.63827
4749	P0C206	A0A183	0.45994
4750	P0C206	O43491	0.37085
4751	P0C206	Q6ZVN8	0.29878
4752	P0C206	P60411	0.72851
4753	P0C206	Q9H4Z2	0.86737
4754	P0C206	O14908	0.24014
4755	P0C206	Q6MZP7	0.78142
4756	P0C206	Q5SV97	0.75732
4757	P0C206	P61570	0.86244
4758	P0C206	Q99807	0.2134
4759	P0C206	Q96P15	0.27508
4760	P0C206	Q8WUY8	0.59761
4761	P0C206	O96028	0.55939
4762	P0C206	P23526	0.18456
4763	P0C206	P61981	0.59939
4764	P0C206	Q7Z3K3	0.74203
4765	P0C206	O00151	0.45305
4766	P0C206	Q9HCI7	0.32211
4767	P0C206	O94905	0.38987
4768	P0C206	A0AVK6	0.47791
4769	P0C206	Q8TCE6	1.1096
4770	P0C206	A6NFL8	0.93098
4771	P0C206	O95361	0.26559
4772	P0C206	P0C7W0	0.68336
4773	P0C206	Q8TEV8	0.45994
4774	P0C206	Q96CX3	0.33813
4775	P0C206	Q8IZ16	0.26251
4776	P0C206	P15884	0.67008
4777	P0C206	O15078	0.68377
4778	P0C206	Q9H0P0	0.44798
4779	P0C206	Q9BR84	0.57913
4780	P0C206	A6NEN9	0.05351
4781	P0C206	Q9Y692	0.93419
4782	P0C206	Q9BUN8	0.2693
4783	P0C206	Q12816	0.42409
4784	P0C206	O96006	0.84028
4785	P0C206	Q8TEL6	0.50904
4786	P0C206	Q96F24	0.83436
4787	P0C206	O00757	0.07202
4788	P0C206	O43395	0.84755

P0C206	O15111	0.63954
P0C206	Q9NRI5	0.47613
P0C206	Q8WTS6	0.57897
P0C206	A8MUI8	0.46956
P0C206	Q9H963	0.43883
P0C206	P20823	0.2487
P0C206	Q502W7	0.30328
P0C206	A6NGY3	0.12155
P0C206	O14618	0.57886
P0C206	Q99728	0.36906
P0C206	P46777	1.117
P0C206	O14734	0.3652
P0C206	Q9NUP7	0.88757
P0C206	O95278	0.2662
P0C206	Q6ZU67	0.4231
P0C206	P05388	1.1511
P0C206	Q9NRX1	0.19092
P0C206	Q6ZP68	0.84164
P0C206	Q8WWW0	0.60248
P0C206	Q9UI10	0.37993
P0C206	Q5TYM5	0.32638
P0C206	Q9HD64	0.27851
P0C206	Q9NWT8	0.9571
P0C206	O94916	0.31431
P0C206	O95416	0.54207
P0C206	Q76FK4	0.32789
P0C206	P62979	0.45057
P0C206	P50539	0.36072
P0C206	Q9GZP9	0.23816
P0C206	P28347	1.1
P0C206	Q8NDF8	0.55645
P0C206	P13073	0.37206
P0C206	Q8NA54	0.24308
P0C206	P17020	0.25974
P0C206	Q99590	0.76132
P0C206	Q7Z739	0.53545
P0C206	P78345	0.97918
P0C206	Q9BWT7	0.25208
P0C206	P53539	0.12219
P0C206	P59022	0.67301
P0C206	Q96NL3	0.35755
P0C206	Q96MF0	0.18409
P0C206	Q8N9R8	0.80181
P0C206	Q96MU7	0.74915
P0C206	Q8IZS6	0.2429
P0C206	Q6T4R5	0.33364
P0C206	P13994	0.52156
P0C206	Q8NCA9	0.20949
P0C206	Q92546	1.0138
P0C206	Q96FH0	0.42103
P0C206	P58557	1.0551
P0C206	Q3SY89	0.17848
P0C206	Q8N616	0.45994
P0C206	Q9NS37	0.94781
P0C206	P17039	0.27283
P0C206	Q8TBC5	0.29094
P0C206	Q7Z417	0.82087
P0C206	Q02447	0.61327
P0C206	Q8TF32	0.55334
P0C206	Q9Y5N6	0.6727
P0C206	P16452	0.23951
P0C206	Q9Y6X0	0.028716
P0C206	Q06547	1.1562
P0C206	Q9BTL4	0.56161
P0C206	Q9H7Z3	0.21239
P0C206	Q15834	1.3154
P0C206	Q9P0U1	0.49482
P0C206	Q8N693	0.53418
P0C206	Q6P2Q9	0.45987
P0C206	P05783	1.3462
P0C206	Q6PUV4	0.28156
P0C206	Q9UJK0	1.0047
P0C206	Q9BUV0	0.68336
P0C206	A6ZKI3	0.62913
P0C206	O95405	0.25716
P0C206	P57772	0.5566

P0C206	Q96GY0	0.662
P0C206	Q5T230	1.486
P0C206	Q3C1V8	0.78154
P0C206	A8MXK9	0.15499
P0C206	Q9BRU9	0.39571
P0C206	Q86TU6	0.9777
P0C206	Q5T1C6	0.80091
P0C206	Q9Y2J4	0.39527
P0C206	Q6ZQT7	0.45994
P0C206	P67870	0.37114
P0C206	Q9BVL4	1.3352
P0C206	Q6ZSS3	0.279
P0C206	Q8NI35	0.42662
P0C206	Q92759	0.77192
P0C206	Q75VX8	1.0064
P0C206	P35789	0.50318
P0C206	Q96JJ3	0.32186
P0C206	Q96IR3	0.72264
P0C206	Q8WVC0	1.1244
P0C206	Q66GS9	0.52238
P0C206	P55273	0.28751
P0C206	P30039	0.21998
P0C206	Q15486	0.54467
P0C206	Q9Y2B2	0.78901
P0C206	Q9NYF3	0.4064
P0C206	Q6J272	1.0957
P0C206	P46527	0.39374
P0C206	Q9H1X1	0.39988
P0C206	O43633	0.36151
P0C206	O95551	0.95904
P0C206	Q8N841	0.19093
P0C206	Q8IXQ4	0.68336
P0C206	Q8WU49	0.45994
P0C206	Q8N128	0.45994
P0C206	Q9BZQ4	0.2441
P0C206	P28324	1.5804
P0C206	A6NMX2	0.037569
P0C206	Q8NFD4	0.14704
P0C206	O75969	0.41249
P0C206	Q8IUC1	0.30526
P0C206	Q6ZRH4	1.5889
P0C206	P23284	0.39453
P0C206	B7ZBB8	0.13188
P0C206	Q03052	0.41024
P0C206	O95793	0.27497
P0C206	Q5T160	0.70526
P0C206	O15446	0.34982
P0C206	Q659C4	0.56868
P0C206	O43768	0.32032
P0C206	Q96LB3	0.78666
P0C206	Q9NVP1	0.13612
P0C206	Q8TBB1	0.97989
P0C206	Q96CS2	0.80565
P0C206	Q96LL4	0.68336
P0C206	Q9H2M3	0.03719
P0C206	Q8N1D0	0.14821
P0C206	Q8WU76	0.15448
P0C206	Q8TDY2	0.98617
P0C206	O43663	1.0607
P0C206	Q9Y2W1	0.73772
P0C206	P62256	0.74623
P0C206	P04406	0.41315
P0C206	Q14CN4	0.31106
P0C206	A8MV24	0.50351
P0C206	Q96AX1	0.22328
P0C206	O75956	0.29837
P0C206	Q96BY2	0.56108
P0C206	Q8IW35	1.3957
P0C206	P62318	0.72466
P0C206	Q8N554	0.20949
P0C206	Q9UPX8	0.42981
P0C206	P04198	0.81801
P0C206	Q96HP8	1.1453
P0C206	Q5H9L2	1.2277
P0C206	Q96M60	0.29278
P0C206	Q9BT78	1.1541

P0C206	Q9H8N7	0.42183
P0C206	P17030	0.33951
P0C206	Q5VV17	0.17182
P0C206	Q14790	0.2675
P0C206	P48444	0.58562
P0C206	Q495D7	0.32782
P0C206	P54727	0.8319
P0C206	Q6NZY4	0.72855
P0C206	Q5H9L4	0.17749
P0C206	Q6ZSA8	0.45994
P0C206	Q8ND99	0.13861
P0C206	Q15024	0.6489
P0C206	Q92558	0.47921
P0C206	Q14582	0.4215
P0C206	Q9BW92	0.49916
P0C206	Q13352	0.65877
P0C206	Q96IT1	0.84723
P0C206	Q6ZVL8	0.45994
P0C206	O15371	0.71222
P0C206	Q9HCL3	0.53058
P0C206	O14771	0.63717
P0C206	Q6IB77	0.35659
P0C206	P35749	0.33462
P0C206	Q7Z4H7	1.1466
P0C206	Q6PEW1	0.28176
P0C206	Q5TA45	0.37411
P0C206	Q96ST2	0.3888
P0C206	Q8N7Q3	0.43586
P0C206	Q8IVC4	0.36355
P0C206	Q15006	0.45827
P0C206	P40222	0.74313
P0C206	Q68DY9	0.36892
P0C206	P0CJ79	0.38918
P0C206	Q01546	0.41667
P0C206	Q9UJ04	0.31262
P0C206	Q7RTV0	0.4518
P0C206	Q02083	0.11846
P0C206	Q5FBB7	0.94348
P0C206	Q9Y4X4	0.33478
P0C206	P57075	0.42682
P0C206	Q6PCB5	0.23535
P0C206	Q15370	1.1563
P0C206	Q5VZE5	0.40518
P0C206	H0Y354	0.43017
P0C206	P40926	0.23112
P0C206	Q9ULD2	0.63661
P0C206	Q5T6V5	0.22478
P0C206	Q5VZT2	0.19534
P0C206	Q13956	0.52038
P0C206	A6NK75	0.41937
P0C206	P10909	0.10316
P0C206	O15234	0.93284
P0C206	Q8WVR3	0.68336
P0C206	O14893	0.63494
P0C206	Q8IYB1	0.39325
P0C206	Q8WW14	0.44797
P0C206	P78545	0.87139
P0C206	Q6P9F0	0.42526
P0C206	P22830	0.095865
P0C206	Q6PIY2	0.42568
P0C206	Q64ET8	0.85785
P0C206	Q2M3G4	0.066982
P0C206	Q6ZSJ8	0.68336
P0C206	Q8N9Q2	0.41337
P0C206	Q9BUL8	0.777
P0C206	Q8NAB5	0.45994
P0C206	Q5VT06	0.82803
P0C206	Q6PD74	0.42207
P0C206	Q9Y2V0	0.17182
P0C206	A6NK89	0.89922
P0C206	Q16143	0.4774
P0C206	Q08378	0.53603
P0C206	O14925	0.39503
P0C206	Q6P1N9	0.37
P0C206	Q8N157	0.24884
P0C206	P49916	0.31568

P0C206	Q9H1B4	0.41318
P0C206	Q9NUL3	0.69341
P0C206	Q13609	0.093667
P0C206	P17947	0.73132
P0C206	Q96HQ0	0.33367
P0C206	Q8N0Y7	0.75109
P0C206	Q02930	0.9136
P0C206	E9PJ23	0.10942
P0C206	Q8TDR4	0.52229
P0C206	Q13901	1.131
P0C206	A6NK39	0.34893
P0C206	Q9ULN7	1.0222
P0C206	Q92870	0.82209
P0C206	Q16534	0.30395
P0C206	Q8NHM5	0.3022
P0C206	P28066	0.54848
P0C206	Q99440	0.37966
P0C206	Q9NPB6	0.8197
P0C206	Q9BYV8	0.7642
P0C206	Q8N9W5	0.17026
P0C206	O94903	0.65873
P0C206	P62899	0.66929
P0C206	O76003	0.34974
P0C206	Q7Z713	0.33985
P0C206	P17028	0.88874
P0C206	P38936	0.28811
P0C206	Q9NQ92	1.0672
P0C206	Q5VWG9	1.0406
P0C206	Q9UM11	1.2672
P0C206	Q96S55	0.4012
P0C206	Q99608	0.24094
P0C206	Q86T82	0.45754
P0C206	Q6UWH4	0.98868
P0C206	Q96GC6	1.3555
P0C206	B3EWG5	0.68336
P0C206	O60921	0.98525
P0C206	Q9NQ33	0.65215
P0C206	P31040	0.57403
P0C206	O75635	0.34057
P0C206	O15063	0.23321
P0C206	P51523	0.41273
P0C206	Q7L2R6	0.53104
P0C206	Q8NHW3	0.3583
P0C206	Q9UI30	0.49774
P0C206	Q12996	0.10267
P0C206	Q5C9Z4	0.72799
P0C206	Q96AQ7	0.64324
P0C206	P08107	0.57013
P0C206	A6NLW8	0.386
P0C206	P24539	0.28142
P0C206	P35579	0.68663
P0C206	Q9BPZ7	0.71757
P0C206	O00401	0.69013
P0C206	P82933	0.68659
P0C206	Q9UL16	0.26915
P0C206	A6NF01	1.1841
P0C206	Q9UBB6	0.602
P0C206	Q9H2U2	0.54608
P0C206	Q15819	0.66117
P0C206	Q9BUH8	0.58757
P0C206	Q8N8J6	0.20949
P0C206	P56182	0.7535
P0C206	P47813	1.0039
P0C206	P56537	1.2932
P0C206	Q9HCN2	0.45994
P0C206	P17152	0.50752
P0C206	Q8TAA5	0.46229
P0C206	Q8TF05	0.21502
P0C206	Q04864	1.2562
P0C206	Q969T4	0.73274
P0C206	Q8N1L9	0.9262
P0C206	O43167	0.86737
P0C206	Q9H4Z3	0.19866
P0C206	O94927	0.80409
P0C206	Q9UHT9	0.45994
P0C206	Q4VC44	0.472

5093	P0C206	Q9NXP7	0.30922
5094	P0C206	P58317	0.35364
5095	P0C206	A5YKK6	1.1349
5096	P0C206	Q6P9G9	0.29094
5097	P0C206	P49750	1.1805
5098	P0C206	P62714	0.8807
5099	P0C206	Q5VU43	1.1879
5100	P0C206	Q9HAF1	1.1977
5101	P0C206	P0C5K6	0.45994
5102	P0C206	Q9BWD1	0.28891
5103	P0C206	O43294	0.23169
5104	P0C206	A6NN06	0.45994
5105	P0C206	Q96DT0	0.14972
5106	P0C206	P55895	0.63515
5107	P0C206	Q9NY27	1.0073
5108	P0C206	A6H8Z2	0.29736
5109	P0C206	Q9BRV8	0.66808
5110	P0C206	Q9NYJ8	0.65406
5111	P0C206	Q9Y6I3	0.16013
5112	P0C206	Q9P2F5	0.36522
5113	P0C206	Q15628	0.69742
5114	P0C206	Q9Y222	1.1541
5115	P0C206	Q8NBF1	0.45387
5116	P0C206	Q96S99	0.27541
5117	P0C206	Q53HL2	1.1063
5118	P0C206	O15069	0.51846
5119	P0C206	Q659A1	0.2276
5120	P0C206	Q6GQQ9	0.20086
5121	P0C206	O00762	0.58324
5122	P0C206	Q96NJ3	0.30696
5123	P0C206	Q92614	0.25156
5124	P0C206	Q8IWF6	0.58929
5125	P0C206	Q8N7M0	0.43093
5126	P0C206	Q92552	0.82405
5127	P0C206	A6NGU7	0.15182
5128	P0C206	Q9NRR4	0.46332
5129	P0C206	Q96A49	1.2364
5130	P0C206	Q86SE8	0.16819
5131	P0C206	O60281	0.40475
5132	P0C206	A1A4F0	0.017119
5133	P0C206	Q5VWZ2	0.088054
5134	P0C206	O43482	0.61231
5135	P0C206	Q9UKX2	0.33462
5136	P0C206	P83731	0.67945
5137	P0C206	A0MZ66	0.65103
5138	P0C206	Q9UHA2	0.37687
5139	P0C206	Q9HA38	0.24465
5140	P0C206	Q6ZQV5	0.40539
5141	P0C206	Q96MF7	0.81449
5142	P0C206	Q9Y3D9	0.51247
5143	P0C206	Q9H665	0.39633
5144	P0C206	P26371	0.45994
5145	P0C206	Q9NPB8	0.041763
5146	P0C206	A6NJB7	1.103
5147	P0C206	Q9Y3A0	0.26417
5148	P0C206	Q8N998	0.33985
5149	P0C206	Q96QH2	0.22926
5150	P0C206	Q9Y2L1	0.48028
5151	P0C206	Q96ES7	0.2939
5152	P0C206	H0UI37	0.26185
5153	P0C206	P42574	0.24802
5154	P0C206	Q13505	0.98825
5155	P0C206	P0C5J1	0.18753
5156	P0C206	Q96JM3	0.55848
5157	P0C206	Q6NUP7	0.57893
5158	P0C206	Q9BXM0	0.79929
5159	P0C206	Q9BZK7	0.99877
5160	P0C206	Q86XN8	0.26288
5161	P0C206	Q9H5I1	0.72552
5162	P0C206	Q9NVF7	0.80301
5163	P0C206	C9JC47	0.45994
5164	P0C206	O95900	0.73237
5165	P0C206	Q96KD3	1.2191
5166	P0C206	Q92625	0.26484
5167	P0C206	Q15562	0.9138
5168	P0C206	Q9BV57	0.69553

P0C206	Q9BZG8	0.33933
P0C206	O75376	0.44226
P0C206	Q9HCE5	0.20362
P0C206	Q12893	0.3106
P0C206	P82663	0.80083
P0C206	P42568	1.0129
P0C206	Q9H8V3	0.26916
P0C206	P82675	0.56522
P0C206	A6NI86	0.86722
P0C206	Q49MG5	1.2084
P0C206	Q96MU6	0.29467
P0C206	Q8WWY3	0.32455
P0C206	Q8TC76	1.6967
P0C206	Q9BYN7	0.41273
P0C206	Q9H165	0.31682
P0C206	Q9BQI5	0.32795
P0C206	Q7Z3V5	0.39234
P0C206	Q6ZNK6	1.1005
P0C206	Q8WWB5	0.96709
P0C206	Q6ZQR2	1.216
P0C206	Q9HBH7	0.69665
P0C206	Q8WUH2	0.82203
P0C206	Q9BV73	0.76716
P0C206	Q9NUQ6	0.54755
P0C206	Q92841	0.65876
P0C206	Q9Y2P8	0.50374
P0C206	Q9Y6R6	0.40539
P0C206	Q96C57	0.38994
P0C206	Q5SNT6	0.83662
P0C206	P07902	0.63044
P0C206	Q9GZX5	1.0339
P0C206	Q8N205	0.56567
P0C206	Q8N782	0.49209
P0C206	Q6X4W1	1.3755
P0C206	Q6ZNG1	0.41874
P0C206	P55795	0.061802
P0C206	O00267	1.1387
P0C206	Q9Y375	0.70603
P0C206	A6NEA5	0.047385
P0C206	A6NNL0	0.75244
P0C206	Q9NP87	0.2423
P0C206	P20809	0.25253
P0C206	P39019	0.77233
P0C206	Q92901	0.5404
P0C206	P0CW19	0.2511
P0C206	P02810	0.45994
P0C206	Q16533	0.62467
P0C206	Q5TAQ9	0.37887
P0C206	Q5MJ10	0.80268
P0C206	Q5SWA1	0.65062
P0C206	Q16612	0.31
P0C206	Q5TBB1	0.90435
P0C206	P02792	0.088351
P0C206	Q9NVV4	0.58754
P0C206	P51826	0.53786
P0C206	Q5JUQ0	0.12271
P0C206	P05387	0.5404
P0C206	Q5TAX3	0.86187
P0C206	Q92804	0.29927
P0C206	Q8NHU0	0.26814
P0C206	P20810	0.27082
P0C206	Q9H9A7	0.69851
P0C206	Q5TEZ4	0.45994
P0C206	Q15424	0.78483
P0C206	Q9BQY4	0.95314
P0C206	Q9UNZ5	0.68336
P0C206	Q15388	0.26743
P0C206	Q86XU0	0.34053
P0C206	O60381	0.30859
P0C206	O43736	0.39848
P0C206	A6NLC8	1.1278
P0C206	Q06732	0.22742
P0C206	Q6IN84	0.36757
P0C206	Q9BSM1	1.3061
P0C206	Q8NHV4	0.9705
P0C206	Q9H3F6	0.63936

P0C206	Q6ZR52	0.4071
P0C206	P61565	0.86244
P0C206	Q8N9Y4	1.0099
P0C206	O60832	0.67836
P0C206	Q53EL6	1.0341
P0C206	O14818	0.5376
P0C206	O75478	0.57056
P0C206	P46783	1.3761
P0C206	Q96AN2	0.22886
P0C206	Q8WU03	0.62298
P0C206	Q96FI4	0.37956
P0C206	P37802	0.46286
P0C206	O00399	0.27224
P0C206	Q7Z745	0.65728
P0C206	Q2NKX9	0.68336
P0C206	Q96H12	1.0065
P0C206	Q9NRC8	1.4412
P0C206	Q17RG1	0.13145
P0C206	Q9H2G4	0.83772
P0C206	P15036	0.85052
P0C206	O15392	0.36528
P0C206	Q5MCW4	0.27837
P0C206	Q14872	0.94021
P0C206	O14910	0.74759
P0C206	B1ATL7	0.25409
P0C206	O14896	0.57992
P0C206	Q7Z392	0.50298
P0C206	Q7Z465	0.76165
P0C206	Q9C035	0.72854
P0C206	Q5K131	0.17148
P0C206	Q6P0N0	0.90113
P0C206	A6NH52	0.98927
P0C206	P23327	0.41994
P0C206	Q5T5N4	0.088066
P0C206	Q9GZU8	0.45233
P0C206	Q5FWF6	0.54896
P0C206	Q4G0G2	0.42202
P0C206	P40763	0.32124
P0C206	Q5T8I3	0.20991
P0C206	P22670	0.96129
P0C206	Q15573	0.62513
P0C206	P31941	0.11275
P0C206	Q9UDV6	0.79316
P0C206	Q15937	0.32658
P0C206	Q6NSW5	1.0314
P0C206	Q6P9A1	0.48353
P0C206	Q86UN6	0.29409
P0C206	P60520	0.32186
P0C206	O43597	0.027603
P0C206	Q9H2T7	0.58364
P0C206	Q96AB3	0.83949
P0C206	Q13485	0.64689
P0C206	Q6P1L5	1.3885
P0C206	Q86UZ6	0.30231
P0C206	Q9NQX7	0.33777
P0C206	Q9H3P2	1.1872
P0C206	Q96L14	0.64137
P0C206	Q9BTA9	1.0397
P0C206	Q9Y4R8	0.32518
P0C206	Q496A3	0.28657
P0C206	Q9BZI1	0.26678
P0C206	Q8N1N5	0.63499
P0C206	Q8TAQ5	0.40539
P0C206	P25208	0.72405
P0C206	P56192	0.35279
P0C206	Q9UBZ9	0.66141
P0C206	Q9Y5A6	0.88936
P0C206	Q9H707	0.36276
P0C206	P48454	0.22433
P0C206	A2RU37	0.29388
P0C206	P42766	0.84165
P0C206	Q12840	0.39706
P0C206	Q96B42	0.31701
P0C206	P55209	0.78425
P0C206	P61244	0.94789
P0C206	Q8IWY8	0.31234

P0C206	Q8N6V9	0.061507
P0C206	Q9NRJ4	0.24757
P0C206	P0C7M4	0.31537
P0C206	Q9UPR3	1.0733
P0C206	Q6ISU1	0.50357
P0C206	Q8N3R3	0.27794
P0C206	Q9H008	0.53239
P0C206	Q15633	0.76556
P0C206	Q9H1Q7	0.71164
P0C206	P61960	0.35968
P0C206	Q8IWZ8	0.17176
P0C206	P55072	0.27077
P0C206	Q96MR7	1.2756
P0C206	Q08AF8	0.55886
P0C206	Q8N5J4	0.62347
P0C206	Q13129	0.93322
P0C206	Q9UKY0	0.21861
P0C206	Q9Y5N5	0.3844
P0C206	P60953	0.54053
P0C206	Q9NSK0	0.17092
P0C206	Q8TF45	0.27283
P0C206	Q8IWV8	0.36008
P0C206	Q53SZ7	0.68336
P0C206	P60866	0.81082
P0C206	Q17RF5	1.0753
P0C206	Q9NP92	1.0328
P0C206	Q14240	0.093275
P0C206	Q9H489	0.48222
P0C206	P23258	0.40823
P0C206	Q6UYE1	0.88528
P0C206	Q8IUR0	1.1307
P0C206	P0C866	0.065582
P0C206	O00139	0.56662
P0C206	P23760	0.9378
P0C206	Q9UKZ1	0.77878
P0C206	P23434	0.22312
P0C206	Q5TG08	0.90147
P0C206	Q9NTI5	1.0334
P0C206	Q9BUR4	0.84719
P0C206	P25791	0.62369
P0C206	Q9Y657	0.23043
P0C206	P52435	0.69376
P0C206	Q9BR09	0.17808
P0C206	Q92600	1.2623
P0C206	Q96EK6	0.46548
P0C206	Q5T5J6	0.75438
P0C206	Q71SY5	1.1863
P0C206	Q9BXP5	1.2605
P0C206	P61962	0.81278
P0C206	P0CW71	0.45994
P0C206	Q8TAD1	0.33985
P0C206	Q86WQ0	0.60367
P0C206	Q8N0S2	0.1779
P0C206	Q9BTT4	0.74411
P0C206	P25440	0.62252
P0C206	O15535	0.29094
P0C206	O95295	0.49287
P0C206	Q7Z614	0.59717
P0C206	Q8IXJ6	0.53947
P0C206	Q9NTK5	0.64954
P0C206	Q12765	0.59428
P0C206	Q8IVK1	0.45994
P0C206	Q96E16	0.90187
P0C206	Q9BYH8	0.47833
P0C206	O00463	0.50409
P0C206	Q13489	0.25078
P0C206	Q8TF39	0.5435
P0C206	Q96BU1	0.19589
P0C206	Q6NXR4	0.25501
P0C206	O43617	0.45329
P0C206	Q9NZH4	0.76259
P0C206	Q9NR22	0.42086
P0C206	Q8IYJ2	0.63187
P0C206	Q9NX01	0.28951
P0C206	O76009	0.40644
P0C206	Q53FN2	0.40198

P0C206	Q9NZJ0	0.89886
P0C206	Q9C0H9	0.14665
P0C206	O14776	1.2469
P0C206	Q9BSK1	0.28383
P0C206	P62306	0.24949
P0C206	Q5VWT5	0.76438
P0C206	P31150	0.20721
P0C206	Q86V42	0.4846
P0C206	Q7Z3Y8	0.4284
P0C206	Q8WYA1	0.4359
P0C206	Q9NWS0	0.40678
P0C206	Q8N612	0.69575
P0C206	Q9BZE4	0.41171
P0C206	Q9ULR0	0.90098
P0C206	Q15022	0.80716
P0C206	Q5HYK9	0.378
P0C206	Q9NUD7	0.076804
P0C206	P04818	0.12177
P0C206	Q96CF2	0.34798
P0C206	Q5HYM0	0.23217
P0C206	Q8TBE0	0.93327
P0C206	Q9H116	0.58093
P0C206	Q7L5D6	0.95499
P0C206	Q5BJE1	0.30885
P0C206	Q9HB21	0.30112
P0C206	P62487	0.4058
P0C206	Q2M1K9	0.42585
P0C206	Q9NUJ3	0.25904
P0C206	O95782	0.51194
P0C206	Q9HB75	0.78603
P0C206	Q6L8Q7	0.29183
P0C206	Q96HR8	1.1764
P0C206	Q701N4	0.45994
P0C206	Q9H6Z4	1.2604
P0C206	P28370	0.60793
P0C206	Q9BXU0	0.30982
P0C206	P60014	0.91057
P0C206	O14709	0.40539
P0C206	O15453	0.54639
P0C206	Q13148	0.46453
P0C206	Q8N5F7	1.4584
P0C206	O43172	0.67892
P0C206	P98171	0.26023
P0C206	Q9P258	0.86382
P0C206	Q9NYP8	0.25833
P0C206	P45985	0.23085
P0C206	Q7Z3Y7	0.44615
P0C206	Q9P0J0	0.55505
P0C206	Q96CB8	0.75073
P0C206	Q719I0	0.24463
P0C206	Q9H0C5	0.6808
P0C206	Q14527	0.47251
P0C206	Q156A1	0.45994
P0C206	Q5T9C2	0.68336
P0C206	Q96T58	0.6259
P0C206	Q9BRA2	0.59951
P0C206	P62841	0.67167
P0C206	Q15652	0.49083
P0C206	O60869	0.6874
P0C206	Q9H7L9	0.70678
P0C206	Q9H0U9	0.50685
P0C206	Q8WWM1	0.71216
P0C206	Q9NQY0	0.53062
P0C206	A6NNV3	0.51209
P0C206	Q14677	0.60754
P0C206	Q9UJ41	0.48809
P0C206	Q15366	0.87971
P0C206	O00541	0.47847
P0C206	Q9NPR9	0.93858
P0C206	Q99567	0.85614
P0C206	Q99627	0.55205
P0C206	Q9H5N1	0.59717
P0C206	A6NGG8	0.64957
P0C206	Q8NFG4	0.88726
P0C206	P62140	0.86025
P0C206	O75665	0.83336

P0C206	Q9UJX6	0.62689
P0C206	P32780	0.85027
P0C206	Q99704	0.17371
P0C206	Q04724	0.95574
P0C206	P0CG47	0.4264
P0C206	Q8NE71	0.42015
P0C206	A6NH11	0.62033
P0C206	A6PVY3	0.17601
P0C206	Q3LI59	0.45994
P0C206	Q13356	0.25029
P0C206	O60825	0.083956
P0C206	Q96H22	0.37495
P0C206	Q8N7B7	0.22959
P0C206	Q99459	1.1054
P0C206	Q99684	0.51441
P0C206	O60287	1.0501
P0C206	O15144	0.32999
P0C206	Q9Y3B2	0.75661
P0C206	Q9ULD5	0.25562
P0C206	Q9Y5K8	0.072028
P0C206	Q5VVP1	1.225
P0C206	Q8NC38	0.10721
P0C206	O60551	0.25416
P0C206	Q9NQ48	0.35637
P0C206	Q15542	1.0287
P0C206	Q5T0L3	1.2739
P0C206	Q8N8L2	0.3003
P0C206	Q96BN6	0.17179
P0C206	Q9C0C2	0.35815
P0C206	Q6NSI8	0.59371
P0C206	Q92966	0.64729
P0C206	P61457	1.1101
P0C206	O75373	0.38296
P0C206	A8MVJ9	0.51937
P0C206	Q8N587	0.48487
P0C206	Q9HAN9	0.35216
P0C206	Q49AS3	0.38116
P0C206	Q9HAU5	0.85638
P0C206	Q9NXE8	0.65719
P0C206	Q96N38	0.52001
P0C206	Q14934	0.55756
P0C206	Q6ZNR8	0.45994
P0C206	Q9UI32	0.38256
P0C206	Q9NXK8	0.71426
P0C206	Q8N336	0.3291
P0C206	P16333	0.67125
P0C206	Q9BY31	0.22565
P0C206	Q13561	0.9954
P0C206	Q96T76	1.0391
P0C206	O75909	1.2443
P0C206	Q8N4W9	0.40539
P0C206	Q96A00	0.35522
P0C206	O43447	0.624
P0C206	P55060	1.0108
P0C206	Q8NHS9	0.25665
P0C206	Q96BY7	0.22733
P0C206	O00472	0.72069
P0C206	Q8IXS8	0.84064
P0C206	P17096	0.54071
P0C206	B1ANY3	0.28306
P0C206	Q96MR9	0.4694
P0C206	Q33E94	1.0322
P0C206	Q9Y5P3	0.47228
P0C206	O95411	0.45994
P0C206	O15370	0.53383
P0C206	Q9BYR3	0.72851
P0C206	Q76N32	0.30141
P0C206	E9PKD4	0.024608
P0C206	Q8N1V2	0.63058
P0C206	P61580	0.95266
P0C206	Q9BSH5	0.47434
P0C206	A6NM28	0.30567
P0C206	Q9HCG8	0.14011
P0C206	Q6NTF7	0.03419
P0C206	Q75QN2	0.70712
P0C206	Q9UJ78	0.23072

P0C206	O43731	0.42686
P0C206	Q9H875	0.2941
P0C206	Q9H2W6	0.473
P0C206	Q14157	0.61857
P0C206	Q16864	0.079976
P0C206	Q13562	0.24403
P0C206	E9PGG2	0.31537
P0C206	Q16384	0.50903
P0C206	P36405	0.38968
P0C206	Q14592	0.34664
P0C206	Q9Y3F1	0.45994
P0C206	B3EWG6	0.68336
P0C206	Q8TE02	1.3787
P0C206	Q9H4G8	0.43556
P0C206	Q9H3H1	0.50094
P0C206	P23769	0.47608
P0C206	Q8IXM2	0.14791
P0C206	Q9H2G9	0.54558
P0C206	Q6UXP9	0.00049604
P0C206	Q9Y6B2	1.1298
P0C206	Q96MP8	0.35881
P0C206	Q9UH36	0.5371
P0C206	Q8N0V1	0.3691
P0C206	A4D1E9	0.87529
P0C206	Q9NYB0	0.82428
P0C206	Q8NFH4	1.034
P0C206	Q99747	0.66557
P0C206	Q96GX9	0.44841
P0C206	Q86V71	0.42186
P0C206	Q03923	0.58644
P0C206	Q8IVU9	0.12683
P0C206	Q9H7E9	0.68336
P0C206	Q8TBR4	0.56716
P0C206	A6NIH7	0.45561
P0C206	Q96RK1	0.80647
P0C206	Q5VYK3	0.39485
P0C206	P61583	0.95202
P0C206	Q14264	0.41125
P0C206	Q8IUC6	0.27757
P0C206	Q8IYI6	0.59555
P0C206	Q8NDH3	0.56842
P0C206	P29474	0.59885
P0C206	Q9Y2P7	0.55762
P0C206	Q9BUW7	0.68336
P0C206	Q8TEK3	0.49096
P0C206	Q8WUM4	1.157
P0C206	Q96K17	0.56371
P0C206	Q15013	1.0025
P0C206	Q9Y606	1.0684
P0C206	Q3SY00	0.87125
P0C206	Q9BX66	0.43444
P0C206	Q5T440	0.31566
P0C206	Q5TAB7	0.80293
P0C206	Q8N584	0.90418
P0C206	P11831	0.62153
P0C206	Q96E11	0.79488
P0C206	Q9BQ67	0.54755
P0C206	Q9UJM3	0.51723
P0C206	Q8WU39	0.35621
P0C206	Q6P2S7	0.14335
P0C206	Q9ULG3	0.45994
P0C206	Q9BWG4	0.058212
P0C206	O96011	0.23394
P0C206	P62072	0.4127
P0C206	Q14194	0.48609
P0C206	Q9H7T3	0.32239
P0C206	Q08050	0.51312
P0C206	P86481	0.45994
P0C206	O60244	0.57714
P0C206	Q00978	1.0615
P0C206	P28072	0.38948
P0C206	P33552	0.52981
P0C206	Q9H0R8	0.36585
P0C206	Q13098	0.34993
P0C206	P0CG08	0.61911
P0C206	Q494X1	0.30517

P0C206	P15924	0.35626
P0C206	Q9NV31	0.86955
P0C206	Q12981	0.21756
P0C206	Q8IX12	1.046
P0C206	Q9H379	0.65269
P0C206	O15162	0.36145
P0C206	Q96LI9	0.49018
P0C206	Q9H9G7	0.67384
P0C206	P46087	0.71128
P0C206	Q9BY43	0.4232
P0C206	Q7L1Q6	0.52352
P0C206	P06703	0.24714
P0C206	Q63HM1	0.42213
P0C206	Q9H7B2	0.8349
P0C206	Q6ZU65	0.36664
P0C206	Q9H009	0.51846
P0C206	Q0D2J5	0.35148
P0C206	Q59GR2	0.31537
P0C206	Q9UHP3	0.35554
P0C206	Q5THR3	0.4759
P0C206	Q9BYR0	0.72851
P0C206	Q63ZY3	1.066
P0C206	Q7Z7L9	0.36282
P0C206	Q9NVV2	0.45994
P0C206	Q3MHD2	0.44999
P0C206	O95336	0.33253
P0C206	Q5MJ07	0.80268
P0C206	O75368	0.28923
P0C206	Q8NEG7	0.48593
P0C206	Q9H6T0	0.17804
P0C206	P30304	0.68947
P0C206	Q8N9P0	0.083548
P0C206	O43678	0.65021
P0C206	Q9NX31	0.026652
P0C206	Q6ZQP7	0.45994
P0C206	I3L1I5	0.50898
P0C206	Q96JC9	1.1208
P0C206	A4D2B0	0.64247
P0C206	Q96C11	0.22864
P0C206	Q86YI8	0.83653
P0C206	O60315	0.4169
P0C206	Q6P1X5	1.0731
P0C206	Q8IZ26	0.33954
P0C206	Q16520	1.0532
P0C206	Q9H7C4	0.46169
P0C206	Q8WVF5	0.13145
P0C206	O94868	0.44439
P0C206	P61574	0.80216
P0C206	Q96N28	0.076529
P0C206	Q5T4H9	0.45994
P0C206	Q6NW29	0.22868
P0C206	Q9BVG4	0.33461
P0C206	P42566	0.29854
P0C206	Q9NS26	0.75209
P0C206	Q9HC38	0.28371
P0C206	Q96IZ7	1.1313
P0C206	Q6IE81	1.2953
P0C206	Q8IYX3	0.90868
P0C206	Q8NA42	0.42986
P0C206	Q15797	0.24586
P0C206	Q8N6M0	0.26612
P0C206	Q9UID3	0.18775
P0C206	Q969S2	0.24613
P0C206	Q13422	0.18062
P0C206	Q9BYS1	0.4802
P0C206	P63241	0.44971
P0C206	Q53HC0	1.151
P0C206	Q9UNX3	1.1315
P0C206	Q01664	0.58662
P0C206	Q9Y237	0.4545
P0C206	Q99966	0.22705
P0C206	Q2NL68	0.68336
P0C206	Q9NPD7	0.54461
P0C206	Q09FC8	0.53327
P0C206	Q8NDQ6	0.42986
P0C206	Q9UKT8	0.37932

P0C206	Q5T215	0.51431
P0C206	Q676U5	0.80252
P0C206	Q5TB30	0.3249
P0C206	Q569G3	0.68336
P0C206	Q5VX52	0.23748
P0C206	Q86W54	0.37343
P0C206	P43243	0.22598
P0C206	Q8N859	0.53502
P0C206	Q9BZE1	0.60944
P0C206	Q9Y294	1.1735
P0C206	P02808	0.42103
P0C206	Q562F6	0.90532
P0C206	Q9NZI6	0.64194
P0C206	O75691	0.90613
P0C206	P54136	0.76201
P0C206	Q8N1I0	0.36338
P0C206	Q9H9F9	1.4291
P0C206	P0CL80	0.23762
P0C206	Q49AJ0	0.2771
P0C206	Q9UHD4	0.67422
P0C206	Q5T4T6	1.1113
P0C206	Q6P582	0.82911
P0C206	Q8IV56	0.45082
P0C206	Q9BXF9	0.18718
P0C206	Q569K6	0.25664
P0C206	Q9H0F6	0.22803
P0C206	Q9UK76	1.0797
P0C206	Q6NSW7	0.43565
P0C206	Q15274	0.11595
P0C206	Q96NL8	0.65693
P0C206	Q9BQ90	0.88905
P0C206	Q96N53	0.45994
P0C206	Q5VT28	0.41181
P0C206	Q99502	0.23615
P0C206	P59282	0.46083
P0C206	P51786	0.31995
P0C206	Q15928	0.55147
P0C206	Q8WWR8	0.1869
P0C206	P0C671	0.11018
P0C206	O95521	0.51099
P0C206	O76015	0.40644
P0C206	Q9GZS1	0.59751
P0C206	O95218	0.56196
P0C206	P20749	0.43132
P0C206	Q6NT89	0.96202
P0C206	O43823	0.44161
P0C206	Q9ULK4	1.2915
P0C206	Q9Y5K6	0.39138
P0C206	O95639	0.44106
P0C206	P84103	0.48967
P0C206	O94925	0.55909
P0C206	A4D0V7	0.91412
P0C206	Q14674	0.40113
P0C206	A1L429	0.23762
P0C206	Q07157	0.74864
P0C206	P18847	1.2562
P0C206	O75400	0.97275
P0C206	Q8N5J2	1.3101
P0C206	O75489	0.24981
P0C206	O15260	0.11494
P0C206	Q9H1L0	0.62125
P0C206	Q8IX07	0.4285
P0C206	Q9NVR2	1.1022
P0C206	Q3B820	0.69104
P0C206	Q9BX82	0.51482
P0C206	Q86Y25	0.34698
P0C206	Q9NPF4	0.19873
P0C206	Q96QD9	0.75777
P0C206	Q9UL36	0.2914
P0C206	O75953	0.78833
P0C206	Q4G112	0.43565
P0C206	Q9UJA5	0.94784
P0C206	O94817	0.63151
P0C206	P02545	0.77813
P0C206	Q96M91	1.1295
P0C206	Q8N1G4	0.296

P0C206	Q96BR6	0.46795
P0C206	Q96M83	0.1153
P0C206	Q9UBF2	0.44806
P0C206	P23588	0.21065
P0C206	P16278	0.17823
P0C206	Q6SJ96	0.3786
P0C206	P0DJG4	0.85083
P0C206	Q9BYR5	0.72851
P0C206	Q8N6K4	0.45994
P0C206	A6NNC1	1.0312
P0C206	P07316	0.091647
P0C206	Q9UHR6	0.9696
P0C206	Q13889	0.38706
P0C206	Q719H9	0.91163
P0C206	P82650	0.3137
P0C206	Q4LEZ3	1.1799
P0C206	O95379	0.28736
P0C206	Q7Z6J9	0.68451
P0C206	Q02952	0.37037
P0C206	Q01850	1.0186
P0C206	Q8NFJ9	0.23751
P0C206	Q53ET0	1.6413
P0C206	Q5XKE5	0.37714
P0C206	F2Z3M2	0.45994
P0C206	P22532	0.45994
P0C206	Q9P0V3	0.67726
P0C206	Q6L8G5	0.45994
P0C206	Q9BRR0	0.52174
P0C206	P0DM63	0.72101
P0C206	P09972	0.63008
P0C206	Q969Q0	0.2329
P0C206	Q9H5Q4	0.51437
P0C206	Q9UBL3	0.4968
P0C206	Q86WJ1	0.54055
P0C206	A6NP61	0.766
P0C206	Q3BBV2	0.90881
P0C206	O60293	0.13539
P0C206	Q7Z7A1	1.3454
P0C206	P61513	0.67275
P0C206	Q2TAM9	1.098
P0C206	Q8IXK0	0.4634
P0C206	O75436	0.81866
P0C206	Q8WUH1	0.038898
P0C206	Q8NDH6	0.39448
P0C206	Q14154	0.60088
P0C206	Q6PI77	0.79872
P0C206	P07199	0.80823
P0C206	A6NDX5	0.39585
P0C206	P24534	0.62337
P0C206	Q9UK39	0.53831
P0C206	P20292	0.15203
P0C206	A6NI79	1.2873
P0C206	Q96FA3	0.71404
P0C206	P49368	0.20803
P0C206	Q8WXH0	0.2635
P0C206	Q69YH5	1.0042
P0C206	Q5TZK3	0.12167
P0C206	Q96JS3	0.43614
P0C206	Q8N3S3	0.48197
P0C206	P49716	0.49087
P0C206	Q5VSY0	0.51783
P0C206	Q9HCM9	0.5865
P0C206	Q6P2D8	0.3591
P0C206	P42226	0.87646
P0C206	Q6ZSN7	0.30036
P0C206	Q9NXG0	0.19257
P0C206	Q96PQ5	0.22398
P0C206	Q7Z5D8	0.63658
P0C206	Q96DE9	0.18424
P0C206	Q9BSY4	0.53355
P0C206	B2CW77	0.45994
P0C206	Q86UB2	0.74974
P0C206	Q8N7H1	0.0296
P0C206	Q96C28	0.48412
P0C206	Q6ZRQ5	0.75812
P0C206	Q96D09	0.1148

P0C206	Q9BXV9	0.45994
P0C206	P78396	1.0052
P0C206	Q08495	0.23569
P0C206	Q32NC0	0.41717
P0C206	Q9NU23	0.095934
P0C206	Q9H7M6	0.42061
P0C206	Q86TL0	0.35004
P0C206	Q9BYQ5	0.72851
P0C206	Q9BY42	0.84516
P0C206	Q8IY57	1.2971
P0C206	A6NGB9	0.35397
P0C206	A6NME0	0.24412
P0C206	Q6ISB3	0.89197
P0C206	Q5I0X4	0.68336
P0C206	Q9BQW3	0.17216
P0C206	Q5TD97	0.29144
P0C206	P53672	0.90346
P0C206	A6NIN4	0.45994
P0C206	Q9UPN7	1.1185
P0C206	Q9UKW6	0.56907
P0C206	Q9H6D7	0.80409
P0C206	Q8N8R7	0.66879
P0C206	P78344	0.38919
P0C206	Q9H7D7	0.54755
P0C206	Q8NET4	0.84911
P0C206	Q9HC24	0.082903
P0C206	P35659	0.41263
P0C206	Q96D98	0.80694
P0C206	Q6PIJ6	0.86025
P0C206	Q8N4B1	0.30783
P0C206	Q92900	0.68059
P0C206	Q9H3M7	0.86786
P0C206	Q8NI29	0.39252
P0C206	O75390	0.20711
P0C206	Q9Y2Q9	0.58063
P0C206	Q9BW11	0.50307
P0C206	Q9Y613	0.71824
P0C206	A8MYA2	0.31453
P0C206	Q6ZR93	0.45994
P0C206	Q04837	0.62883
P0C206	Q15058	0.52882
P0C206	Q9Y3A5	0.7692
P0C206	Q8IYW4	0.21744
P0C206	Q9NZC7	0.381
P0C206	Q9UPP2	0.27822
P0C206	Q9Y6D0	0.50467
P0C206	Q8N5T8	0.59395
P0C206	Q96NJ6	0.29991
P0C206	P0C7X2	0.57532
P0C206	Q9H6Z9	0.49771
P0C206	O75362	0.93828
P0C206	O14829	0.3866
P0C206	O75800	0.71565
P0C206	Q8IWP9	1.0615
P0C206	P49458	0.65452
P0C206	Q96IM9	0.13687
P0C206	Q86YD1	0.82113
P0C206	Q7L8J4	0.37124
P0C206	Q5U5X8	0.90106
P0C206	P98169	0.30392
P0C206	Q99871	0.80327
P0C206	Q969X5	0.19171
P0C206	Q00653	1.287
P0C206	Q9BYG8	0.21522
P0C206	Q6IQ21	0.41507
P0C206	Q9HCE3	0.20949
P0C206	P07498	0.84702
P0C206	A6NCI8	1.0758
P0C206	Q8N1D5	0.29025
P0C206	Q86Y79	0.61529
P0C206	Q13362	0.19715
P0C206	P28702	0.52797
P0C206	Q13155	0.70753
P0C206	P41208	0.76585
P0C206	Q9Y3C4	0.52973
P0C206	P16118	0.11517

P0C206	Q5JR98	0.83236
P0C206	Q8WW01	0.35074
P0C206	Q5VWM1	0.47258
P0C206	P05121	0.2749
P0C206	Q99676	0.38367
P0C206	Q15596	0.71401
P0C206	Q9BUA3	0.52254
P0C206	Q5T5P2	0.25936
P0C206	Q6P1W5	0.68624
P0C206	Q8NFC6	1.005
P0C206	Q969E8	1.1776
P0C206	P37275	0.91126
P0C206	Q9BYR9	0.23291
P0C206	O60671	0.6666
P0C206	Q969R5	0.43599
P0C206	Q5T751	0.45994
P0C206	Q99417	1.6432
P0C206	Q9H777	0.44127
P0C206	Q8WX92	1.5895
P0C206	Q8IYM9	0.28712
P0C206	P0C880	0.45994
P0C206	O60678	0.057897
P0C206	Q8WUB8	0.64383
P0C206	Q9ULQ0	1.0873
P0C206	O60810	0.71545
P0C206	Q49A88	0.49706
P0C206	Q15059	1.2722
P0C206	Q16576	0.5165
P0C206	Q8N7Z2	0.53933
P0C206	Q5JQF7	0.45994
P0C206	O95817	0.50772
P0C206	Q9BXW6	0.31289
P0C206	O60809	0.5053
P0C206	Q96M95	0.071913
P0C206	P06454	0.66933
P0C206	Q5H9S7	0.68257
P0C206	Q6ZVT6	0.25026
P0C206	A4D2H0	0.043398
P0C206	P35712	0.565
P0C206	Q86U90	0.61213
P0C206	Q96M34	0.11399
P0C206	Q13185	0.88492
P0C206	A6NG73	0.34893
P0C206	Q16236	0.69062
P0C206	Q4V348	0.26368
P0C206	Q9UKD1	1.0193
P0C206	P56282	0.31184
P0C206	Q9BSU3	0.65979
P0C206	P0CG31	0.35064
P0C206	P20265	0.7055
P0C206	O15055	0.35446
P0C206	P0DKL9	0.86213
P0C206	P22087	0.62026
P0C206	P31271	0.20386
P0C206	O15350	0.37909
P0C206	Q96GM5	0.79497
P0C206	Q6ZRC1	0.45994
P0C206	Q9H7X3	0.30447
P0C206	P10243	0.70714
P0C206	Q16621	0.53001
P0C206	Q6PK81	0.35022
P0C206	Q13415	0.34385
P0C206	P26639	0.56882
P0C206	Q9HBX3	0.45994
P0C206	Q9UPG8	0.46788
P0C206	Q01826	0.81468
P0C206	Q6ZN11	0.31071
P0C206	Q9H832	0.79463
P0C206	Q96M85	0.45994
P0C206	Q7Z2Z1	0.8756
P0C206	Q8NFQ8	0.62913
P0C206	Q8IYF3	0.51825
P0C206	Q9P242	0.84349
P0C206	Q8N3U4	0.83339
P0C206	P61758	0.72525
P0C206	Q00597	0.96011

6005	P0C206	Q96KT6	0.45994
6006	P0C206	Q53F19	1.0037
6007	P0C206	P14672	0.74417
6008	P0C206	Q8N9K3	0.68336
6009	P0C206	Q8N300	0.19405
6010	P0C206	P56381	0.12451
6011	P0C206	Q9UHD1	0.36082
6012	P0C206	P48378	0.49155
6013	P0C206	A6NKH3	0.39141
6014	P0C206	Q99687	0.57125
6015	P0C206	Q9NQX6	0.353
6016	P0C206	Q9BVT8	0.98737
6017	P0C206	Q9NPI7	0.19176
6018	P0C206	Q8WWL2	0.17075
6019	P0C206	O43543	0.22368
6020	P0C206	Q9ULE6	0.79252
6021	P0C206	Q8WZ82	0.038858
6022	P0C206	Q03393	0.13115
6023	P0C206	Q86UW6	0.20626
6024	P0C206	Q9H2U1	0.39759
6025	P0C206	Q9BYD1	0.9481
6026	P0C206	Q9NXF7	0.37887
6027	P0C206	Q9GZN8	0.18863
6028	P0C206	P59990	0.72851
6029	P0C206	Q13465	0.13224
6030	P0C206	Q96MY1	0.73513
6031	P0C206	Q6UWZ7	0.62329
6032	P0C206	Q3BBV0	0.38485
6033	P0C206	Q96FX2	1.0889
6034	P0C206	Q96CN9	0.70932
6035	P0C206	Q96LS8	0.28493
6036	P0C206	Q5UE93	0.62321
6037	P0C206	Q9NRZ9	0.26071
6038	P0C206	A6NFT4	0.062842
6039	P0C206	Q86WB0	1.4098
6040	P0C206	Q9HBM6	0.7837
6041	P0C206	O75164	1.1373
6042	P0C206	Q86UE4	0.74241
6043	P0C206	H3BN30	0.45994
6044	P0C206	Q68CQ4	0.16423
6045	P0C206	Q5H9I0	0.62003
6046	P0C206	P11277	0.25658
6047	P0C206	P0C7I6	1.0308
6048	P0C206	I3L3R5	0.45994
6049	P0C206	P07741	0.46227
6050	P0C206	Q99575	0.79836
6051	P0C206	Q08379	0.46672
6052	P0C206	Q5VWM6	0.93208
6053	P0C206	Q8NCY6	0.11206
6054	P0C206	Q9Y2B4	0.36669
6055	P0C206	Q96LW9	0.30488
6056	P0C206	A6NIE6	0.50634
6057	P0C206	H0Y7S4	0.16577
6058	P0C206	Q15629	0.48554
6059	P0C206	A6NJR5	0.51209
6060	P0C206	Q8IUB9	0.45994
6061	P0C206	P0CG13	0.10506
6062	P0C206	O14617	0.18873
6063	P0C206	Q9P253	0.29516
6064	P0C206	O00287	0.58044
6065	P0C206	Q8N987	0.41191
6066	P0C206	Q92636	0.43377
6067	P0C206	O14901	0.29778
6068	P0C206	Q9NYF0	0.31856
6069	P0C206	Q96CA5	0.34547
6070	P0C206	O95198	0.5777
6071	P0C206	A6NDN3	0.22107
6072	P0C206	Q96GD0	0.13383
6073	P0C206	P27540	0.46434
6074	P0C206	Q14863	0.30712
6075	P0C206	Q5T6F0	0.25095
6076	P0C206	P63313	0.66868
6077	P0C206	Q8NB12	0.58141
6078	P0C206	Q6TDU7	0.42116
6079	P0C206	Q2QGD7	0.81115
6080	P0C206	Q5PSV4	0.53614

P0C206	O75208	0.54071
P0C206	O95447	0.41135
P0C206	Q9P2D6	0.088668
P0C206	Q9ULZ2	0.94616
P0C206	Q8NEM1	0.53784
P0C206	Q6ZN79	0.5083
P0C206	Q9BY44	0.48621
P0C206	Q96FN9	0.64479
P0C206	Q86V20	0.91316
P0C206	Q9Y2G9	0.77172
P0C206	Q3Y452	0.45994
P0C206	Q5VZ46	0.1103
P0C206	Q5TA81	0.45994
P0C206	P48595	0.34057
P0C206	C9JFL3	0.45994
P0C206	P17097	0.23993
P0C206	Q9GZR5	0.36899
P0C206	Q6ZRN7	0.45994
P0C206	Q9BYZ2	0.54372
P0C206	Q9NVC6	0.60612
P0C206	Q9P2B7	0.68336
P0C206	Q8IV53	0.10641
P0C206	P15172	0.35904
P0C206	P78406	0.59881
P0C206	Q9NWY4	0.60805
P0C206	P20700	0.47011
P0C206	Q96KW2	0.91313
P0C206	Q9NV79	0.083963
P0C206	Q9P2M7	0.56726
P0C206	Q6RVD6	0.45994
P0C206	Q9NQ50	0.8255
P0C206	Q06609	0.55026
P0C206	Q674X7	0.58257
P0C206	P17040	0.84799
P0C206	Q6P9B6	0.44439
P0C206	Q8N715	0.10767
P0C206	Q96P63	0.34169
P0C206	P28074	0.45294
P0C206	Q96EI5	0.82975
P0C206	Q9H204	1.3865
P0C206	Q8TBK2	0.62261
P0C206	Q3SXZ3	0.36975
P0C206	Q5SNV9	0.44497
P0C206	Q14129	0.59656
P0C206	Q14151	0.74266
P0C206	P42704	1.0155
P0C206	Q96PM5	0.56595
P0C206	Q9BQD3	0.49772
P0C206	Q969G3	1.0879
P0C206	Q8NA58	0.51585
P0C206	Q6ZV80	0.060794
P0C206	O95625	0.3931
P0C206	Q8WXC6	0.25526
P0C206	P62277	1.0873
P0C206	Q9BRP4	0.50913
P0C206	P27469	0.73968
P0C206	P10412	0.071351
P0C206	Q14397	0.71393
P0C206	P46926	0.079101
P0C206	Q13772	0.48432
P0C206	Q9BXR0	1.3218
P0C206	Q9HBD4	0.71873
P0C206	Q9UKD2	0.21339
P0C206	Q6S9Z5	0.74428
P0C206	O94906	0.20625
P0C206	Q8N0Z3	0.77925
P0C206	Q5JST6	0.45351
P0C206	B1AJZ1	0.022873
P0C206	Q96EB1	1.2275
P0C206	Q96QC0	0.98996
P0C206	Q8IUC0	0.54406
P0C206	Q9BRT7	0.45994
P0C206	Q9UHB9	0.22869
P0C206	P57077	0.89374
P0C206	Q9BYU5	0.42567
P0C206	Q9UBS3	0.68583

P0C206	A6NEH8	0.28073
P0C206	Q32M92	0.10622
P0C206	Q8N573	0.15163
P0C206	Q92994	0.52823
P0C206	Q8NDP4	0.37513
P0C206	Q7L9L4	0.91835
P0C206	Q8NEY3	0.6789
P0C206	P63302	0.28945
P0C206	Q2M3X9	0.33397
P0C206	P25685	0.41874
P0C206	O94830	0.13946
P0C206	Q8TC20	0.033297
P0C206	P27694	0.67305
P0C206	A7MD48	0.46862
P0C206	Q3LI60	0.45994
P0C206	P20585	0.13422
P0C206	A4D1E1	0.53808
P0C206	P42575	0.32232
P0C206	Q53HM4	0.33654
P0C206	Q8TDY3	0.16231
P0C206	Q9UKT9	0.60443
P0C206	Q8WXS3	1.1761
P0C206	O95067	0.70247
P0C206	Q5VTD9	0.42414
P0C206	A1L020	0.35367
P0C206	Q9NP64	0.42819
P0C206	Q9NXR1	0.47489
P0C206	Q9P270	0.62781
P0C206	P52747	0.9684
P0C206	Q9BWF2	0.39564
P0C206	P56945	0.63194
P0C206	Q9H910	1.0797
P0C206	Q00765	0.11856
P0C206	Q4V328	0.59105
P0C206	A8K554	0.10204
P0C206	Q4KMQ1	0.65467
P0C206	P0C6E6	0.044903
P0C206	O75843	0.75383
P0C206	Q9H9S0	0.27796
P0C206	Q53GI3	0.34372
P0C206	Q86U70	0.28799
P0C206	P45877	0.28126
P0C206	Q9H0M5	0.29385
P0C206	Q3MJ40	0.083875
P0C206	Q6ZP82	0.44439
P0C206	Q96EA4	0.9324
P0C206	Q9HD42	1.1921
P0C206	O43364	0.27301
P0C206	Q14192	0.7831
P0C206	Q14749	0.67188
P0C206	Q96T17	0.75492
P0C206	Q9Y3E5	0.61262
P0C206	O15255	0.45994
P0C206	Q9BXK5	0.5174
P0C206	Q15849	0.37628
P0C206	Q8N1N4	0.42034
P0C206	Q9BYQ9	0.72851
P0C206	Q6ZRV2	1.0921
P0C206	Q9H501	1.1304
P0C206	Q96NA2	1.2379
P0C206	Q09028	0.7177
P0C206	Q8NDD1	0.203
P0C206	Q86TS7	0.45994
P0C206	Q86UD0	0.54755
P0C206	P49419	0.58954
P0C206	Q9H6Q4	0.45693
P0C206	O60346	0.28498
P0C206	Q9H246	0.40295
P0C206	Q5MIZ7	0.62921
P0C206	Q96BN2	0.84459
P0C206	Q9HC16	0.15476
P0C206	Q08209	0.45481
P0C206	Q8N1F1	0.45994
P0C206	O75448	0.5253
P0C206	O14639	0.80351
P0C206	P21127	0.29726

P0C206	Q6PII3	0.45773
P0C206	Q8TBY8	0.73692
P0C206	Q9H1K6	0.093775
P0C206	Q13438	0.1496
P0C206	Q6ZPB5	0.45994
P0C206	Q8NEG0	0.31434
P0C206	Q5T6X4	0.98162
P0C206	Q14134	0.71652
P0C206	Q9BR61	0.28628
P0C206	P0C843	0.4733
P0C206	Q8WUX2	0.20548
P0C206	Q8NEM2	0.41048
P0C206	Q9H9Y2	0.2509
P0C206	O60573	0.54257
P0C206	Q15031	0.33865
P0C206	Q86VF2	0.28084
P0C206	Q2T9L4	0.37411
P0C206	Q70YC4	0.5291
P0C206	Q9C0G0	0.20949
P0C206	Q5EBL2	0.2607
P0C206	Q14814	0.62059
P0C206	Q69YN4	0.43664
P0C206	Q8N8G6	0.29045
P0C206	Q5JVX7	0.66132
P0C206	Q5JY77	0.66879
P0C206	Q92734	0.27403
P0C206	Q96BF6	0.48933
P0C206	Q3SY84	0.38429
P0C206	Q14197	0.10666
P0C206	Q96DE5	1.1323
P0C206	P31274	0.18931
P0C206	P11388	0.28679
P0C206	Q14765	0.47075
P0C206	Q96RQ1	0.14614
P0C206	Q96EY9	0.36031
P0C206	B3KS81	0.26849
P0C206	Q8N801	0.04685
P0C206	P46776	0.8892
P0C206	Q9NWA0	1.4398
P0C206	Q15847	0.45994
P0C206	Q9HAJ7	1.2602
P0C206	Q9UBR4	0.27435
P0C206	O43325	1.043
P0C206	P51814	0.23524
P0C206	O60663	0.75767
P0C206	Q7Z7C8	0.53885
P0C206	Q6XZF7	0.33559
P0C206	Q6NYC8	0.3069
P0C206	Q8ND76	0.73249
P0C206	P11474	0.53404
P0C206	P26678	0.56779
P0C206	Q96B86	0.48034
P0C206	Q8NFD5	1.0068
P0C206	Q9NUX5	0.17048
P0C206	Q8NDN9	0.83201
P0C206	Q9H9J2	0.2124
P0C206	Q9Y2M5	0.92287
P0C206	Q9UN42	0.14098
P0C206	P84101	1.0956
P0C206	O75031	0.3691
P0C206	Q53S33	0.85624
P0C206	P51815	0.2952
P0C206	Q9NZ38	0.14207
P0C206	Q96BW5	0.44628
P0C206	Q6W0C5	0.28179
P0C206	Q92530	0.53995
P0C206	P21506	0.36831
P0C206	A8MT65	0.49384
P0C206	P35663	0.3756
P0C206	Q6ZUL3	0.45994
P0C206	Q9UHI6	0.42604
P0C206	P15927	0.56848
P0C206	Q9UHA3	0.40361
P0C206	Q9UL33	0.43177
P0C206	Q6UWI2	0.63456
P0C206	Q8TAP9	1.7307

P0C206	Q12962	1.1539
P0C206	Q9ULC4	0.51497
P0C206	Q9UK99	0.45167
P0C206	Q96H86	0.31076
P0C206	O60303	0.20166
P0C206	P01106	0.4567
P0C206	Q3LI62	0.45994
P0C206	Q8NEG2	0.59115
P0C206	Q9P2T0	0.64303
P0C206	P0CW01	0.32628
P0C206	Q71F56	0.52218
P0C206	Q9GZY8	0.33622
P0C206	Q8NCX0	0.60772
P0C206	P24928	0.94568
P0C206	Q9NZP6	1.1841
P0C206	Q96M29	0.48602
P0C206	Q8N983	0.59491
P0C206	Q8N2Z9	0.71743
P0C206	Q5TA50	0.31353
P0C206	P62906	0.66333
P0C206	A6NHR8	0.45994
P0C206	Q9UBK9	1.1548
P0C206	Q5XUX0	0.58011
P0C206	Q86WR7	0.79661
P0C206	P62312	0.74143
P0C206	C9J3V5	0.47542
P0C206	Q8N831	0.5659
P0C206	Q8N319	1.382
P0C206	O15194	0.11085
P0C206	Q8IYA6	0.95624
P0C206	Q6ZYL4	0.87718
P0C206	Q86X67	0.031823
P0C206	Q5JRK9	1.1532
P0C206	Q16181	0.26738
P0C206	Q15555	0.33347
P0C206	O15504	0.72761
P0C206	Q9H2H8	0.27642
P0C206	Q8IW19	0.55069
P0C206	Q96K80	0.46784
P0C206	Q9P2Z0	0.50669
P0C206	P41214	0.61479
P0C206	Q9BQQ7	0.23552
P0C206	A6NEW6	0.4045
P0C206	Q9NX18	0.94648
P0C206	Q8IZT9	0.84618
P0C206	Q70YC5	0.47911
P0C206	Q96AE4	0.57915
P0C206	Q8WV44	0.54702
P0C206	Q9H7H0	0.76787
P0C206	Q04637	0.474
P0C206	Q07352	1.0275
P0C206	Q8NCU7	0.99021
P0C206	Q52M58	0.65693
P0C206	P61077	0.93071
P0C206	Q9P209	0.95296
P0C206	Q68EN5	0.13505
P0C206	O60756	0.45994
P0C206	P15121	0.24879
P0C206	Q8TBZ8	0.37111
P0C206	Q9BTV7	0.15207
P0C206	Q15750	0.56055
P0C206	Q9UQ80	1.0727
P0C206	Q9NYW8	0.32175
P0C206	Q3LI76	0.77252
P0C206	Q15047	0.9013
P0C206	Q99932	0.45994
P0C206	P49721	0.35805
P0C206	Q9UN36	0.61235
P0C206	Q504T8	0.32773
P0C206	P52746	0.23725
P0C206	Q14696	0.43554
P0C206	Q96LI6	0.63282
P0C206	Q5HYC2	0.13525
P0C206	O15042	0.25346
P0C206	Q9BUK0	0.98366
P0C206	Q96NL0	0.45238

P0C206	Q5SQS8	0.41727
P0C206	Q92832	0.26572
P0C206	P46108	0.52473
P0C206	Q9NP79	0.43758
P0C206	P63173	0.75582
P0C206	A8CG34	0.50029
P0C206	Q9BXU2	0.43609
P0C206	Q6L8H1	0.45994
P0C206	Q2M329	0.48256
P0C206	Q96QK1	0.88722
P0C206	Q8IWL3	0.66613
P0C206	Q96C01	0.19081
P0C206	Q5JSH3	0.3198
P0C206	Q9NUP1	0.75237
P0C206	Q12798	0.89844
P0C206	Q9NSB4	0.32897
P0C206	Q9ULM2	0.33154
P0C206	Q3ZCX4	0.3556
P0C206	O14595	0.37623
P0C206	Q58F21	0.37232
P0C206	Q9UGL9	0.45994
P0C206	Q9Y6P5	0.57951
P0C206	P06748	0.44383
P0C206	Q9UIA9	0.63683
P0C206	P59797	0.89776
P0C206	Q8TCH9	0.32929
P0C206	P0CE67	0.45994
P0C206	Q9NZZ3	1.1099
P0C206	P82932	0.60985
P0C206	O43583	0.9911
P0C206	Q9H0N5	0.88897
P0C206	Q6P9B9	0.48967
P0C206	A6NLJ0	0.91681
P0C206	Q9UBC3	0.36245
P0C206	Q5T6F2	0.96758
P0C206	P84090	0.34419
P0C206	Q9C0E2	1.0399
P0C206	P49756	0.4263
P0C206	P55199	1.1058
P0C206	Q99594	1.2175
P0C206	O75398	0.35969
P0C206	P0CG24	0.30614
P0C206	Q14331	0.53072
P0C206	Q12769	1.0706
P0C206	Q92796	0.39278
P0C206	P14635	0.34921
P0C206	Q9BYD9	0.35257
P0C206	Q9H8K7	0.36064
P0C206	O95456	0.6156
P0C206	Q9UPP5	0.58232
P0C206	Q96QA5	0.29599
P0C206	Q96FV2	0.69222
P0C206	A6NJ64	0.43431
P0C206	Q93034	0.74085
P0C206	Q8NFP7	0.011653
P0C206	Q8TAP4	0.3011
P0C206	Q92945	0.70661
P0C206	Q9NY74	1.0102
P0C206	Q12972	0.77509
P0C206	Q9UJC3	0.17971
P0C206	Q8N485	0.23713
P0C206	B2RXF5	0.58935
P0C206	Q9BQI9	1.069
P0C206	Q5TID7	0.47352
P0C206	P01011	0.35973
P0C206	P0CF75	0.11258
P0C206	Q9NZC4	0.098612
P0C206	Q8NCE0	0.67912
P0C206	Q96N06	0.45285
P0C206	Q2M3C7	0.64122
P0C206	P59103	0.20885
P0C206	Q96QT6	1.1553
P0C206	Q8NBB4	0.36224
P0C206	A6NC62	0.1941
P0C206	Q9H321	0.8605
P0C206	Q96A09	0.61707

P0C206	Q7L804	0.72079
P0C206	O00170	0.75924
P0C206	Q96DI7	0.61153
P0C206	Q9H8W5	0.30581
P0C206	Q14584	0.34316
P0C206	P60006	0.64415
P0C206	Q5SRH9	1.0084
P0C206	Q92466	0.7612
P0C206	Q7Z6K5	0.1517
P0C206	Q8N5U0	0.45994
P0C206	Q6RUI8	0.41276
P0C206	Q6DD87	0.2773
P0C206	Q7L266	0.3976
P0C206	A4D0Y5	0.45994
P0C206	Q96RL1	0.89733
P0C206	Q9UMR2	0.27502
P0C206	Q99814	0.41467
P0C206	Q9BY41	0.60431
P0C206	P39880	0.69201
P0C206	Q8IUM7	0.30441
P0C206	Q9NYK5	0.58209
P0C206	Q13503	0.77565
P0C206	O15198	0.49081
P0C206	Q96SC8	0.28102
P0C206	Q14585	0.30439
P0C206	Q86V25	0.096286
P0C206	H0YL14	0.51886
P0C206	Q96K83	0.3294
P0C206	Q86X53	0.22585
P0C206	Q96B67	0.44832
P0C206	Q7Z2X7	0.65491
P0C206	P84550	0.043882
P0C206	Q5T3Y7	0.19896
P0C206	P09629	1.0333
P0C206	Q9UPW5	0.1184
P0C206	Q9P0T4	0.48789
P0C206	P56279	0.7811
P0C206	Q7Z6Z7	0.38531
P0C206	Q8N5W9	0.32415
P0C206	O15015	0.86737
P0C206	F8WFD2	0.45121
P0C206	P53992	1.1277
P0C206	Q6QNY0	0.70823
P0C206	Q96FZ7	0.14742
P0C206	P08670	0.16668
P0C206	P60510	1.0644
P0C206	Q14689	0.42652
P0C206	P63165	1.1007
P0C206	Q8WV99	0.46731
P0C206	Q15363	0.19138
P0C206	Q3LI61	0.45994
P0C206	O95644	0.87628
P0C206	Q9BXS6	0.84359
P0C206	Q8N4L8	0.33983
P0C206	Q6B0B8	0.50786
P0C206	P0C7H8	0.23291
P0C206	Q12912	0.97062
P0C206	Q9NT62	0.49786
P0C206	Q9P127	0.32109
P0C206	P09661	0.52868
P0C206	Q92529	0.2918
P0C206	O15084	0.87993
P0C206	O75179	0.29167
P0C206	Q9NQ55	0.4904
P0C206	P55345	0.39092
P0C206	P11532	0.61764
P0C206	Q53QW1	0.68336
P0C206	P11387	0.7356
P0C206	O43684	0.82289
P0C206	Q99798	0.12573
P0C206	Q8IZS5	0.34373
P0C206	Q2TAK8	0.82177
P0C206	O43660	0.31223
P0C206	Q9Y483	0.28201
P0C206	Q86UU5	0.65131
P0C206	Q9NV70	0.52809

P0C206	B2RD01	0.11891
P0C206	Q14549	0.45255
P0C206	Q1MSJ5	1.3184
P0C206	P62987	0.7163
P0C206	Q9H8W4	0.31555
P0C206	Q17R26	0.33385
P0C206	E9PQ53	0.86464
P0C206	Q14865	0.35928
P0C206	Q9NYZ3	0.76848
P0C206	Q8TAP8	0.44537
P0C206	Q8N184	0.30612
P0C206	Q9NQT4	0.44347
P0C206	Q9H2P0	0.29226
P0C206	Q9NV56	0.75737
P0C206	P04259	0.28425
P0C206	Q86UL8	0.36926
P0C206	Q9Y2R5	0.53087
P0C206	Q14257	0.39015
P0C206	P56524	0.38593
P0C206	P42167	0.33519
P0C206	Q9BYX4	0.32267
P0C206	Q8NFW8	0.22808
P0C206	Q13033	0.77721
P0C206	O60583	1.1291
P0C206	Q16762	0.23296
P0C206	Q8WXU2	0.86726
P0C206	Q15018	0.26812
P0C206	O75347	1.1537
P0C206	P61968	0.38815
P0C206	O43439	1.1078
P0C206	Q9Y3I1	0.96862
P0C206	Q13404	1.0697
P0C206	Q6ZVF9	0.63464
P0C206	P38919	0.17755
P0C206	Q9Y5R8	0.12241
P0C206	Q14526	0.74761
P0C206	O75335	0.2367
P0C206	Q7RTT5	0.26627
P0C206	Q9NV72	0.55067
P0C206	Q96GE5	0.50104
P0C206	Q6P435	0.38603
P0C206	P61086	0.72531
P0C206	Q53HR8	0.15998
P0C206	Q9Y2X0	0.54557
P0C206	P13196	0.32491
P0C206	P28356	0.26688
P0C206	Q8N5D0	0.62117
P0C206	O75821	0.45918
P0C206	Q7Z6K1	0.73741
P0C206	Q9Y5U9	1.0605
P0C206	Q9UFB7	0.31909
P0C206	Q96PV6	0.78664
P0C206	Q13094	0.65132
P0C206	Q16352	0.43294
P0C206	P41238	0.063219
P0C206	Q8N1A0	0.32596
P0C206	Q9Y3B1	0.062818
P0C206	O75886	1.2852
P0C206	O00257	1.1208
P0C206	Q96IT6	0.45994
P0C206	Q9Y2Y0	1.1787
P0C206	O60543	0.39087
P0C206	Q9BWC9	0.96503
P0C206	Q9BSK0	0.96663
P0C206	Q96C10	0.22842
P0C206	P28069	0.77043
P0C206	Q6NVH7	0.73133
P0C206	Q7RTS7	0.30526
P0C206	Q16637	0.82328
P0C206	Q9UND3	0.48024
P0C206	P14373	0.43527
P0C206	Q86WS4	0.45994
P0C206	Q96S59	1.034
P0C206	A9YTQ3	0.42495
P0C206	P23297	0.65544
P0C206	Q96EF9	0.36043

P0C206	Q6AZW8	0.31456
P0C206	Q9NYZ1	0.98927
P0C206	Q8WY22	0.76544
P0C206	Q9BY65	0.59957
P0C206	Q9BXW9	0.9612
P0C206	Q9ULW8	0.50612
P0C206	P48788	0.59844
P0C206	Q7L3T8	0.39396
P0C206	P62308	0.62822
P0C206	Q00987	0.27735
P0C206	Q6ZRP0	0.68336
P0C206	Q86V87	0.61532
P0C206	Q13106	0.31962
P0C206	Q99614	0.31625
P0C206	O14965	0.40063
P0C206	P02511	0.43726
P0C206	Q9BQQ3	0.30027
P0C206	P10599	0.37568
P0C206	Q9Y473	0.50016
P0C206	P62891	0.5984
P0C206	Q14980	1.2562
P0C206	Q6NX45	0.32044
P0C206	O95873	0.68336
P0C206	P49247	0.6919
P0C206	Q93062	0.79515
P0C206	Q13363	0.41786
P0C206	Q7L2J0	0.18102
P0C206	P62269	0.73801
P0C206	Q9P2J5	0.31434
P0C206	Q9NS86	0.30362
P0C206	Q8N1B4	0.73891
P0C206	P39748	0.36792
P0C206	Q9Y4B6	0.97175
P0C206	Q9BSH4	1.0354
P0C206	Q8NCP5	0.20949
P0C206	Q9H5Z6	0.68336
P0C206	Q9UHB4	0.64815
P0C206	Q92837	0.89239
P0C206	O43805	0.71176
P0C206	Q8N4C6	0.52454
P0C206	Q6ZNH5	0.34661
P0C206	Q5T619	0.31292
P0C206	Q8WUZ0	0.63879
P0C206	Q96AQ9	0.43121
P0C206	P61925	0.23138
P0C206	Q9HCH5	0.72576
P0C206	A8MVA2	0.72851
P0C206	P41220	0.48624
P0C206	Q52LG2	0.25905
P0C206	Q9GZV8	0.12332
P0C206	Q96SZ4	0.32373
P0C206	P42768	0.38933
P0C206	Q9GZR2	0.20129
P0C206	P29692	0.37183
P0C206	Q9P206	0.77913
P0C206	P35244	0.67884
P0C206	P07737	0.91204
P0C206	Q5SVZ6	0.91033
P0C206	Q96GM8	0.3686
P0C206	Q7Z2W4	0.56336
P0C206	Q5JRC9	0.66423
P0C206	Q8N0Z8	0.4593
P0C206	A6NGR9	0.49792
P0C206	Q8TF68	0.34497
P0C206	Q9UM13	0.77695
P0C206	Q08AN1	0.40539
P0C206	Q86XN6	0.40471
P0C206	Q96MT8	1.2654
P0C206	P62753	0.97999
P0C206	Q7Z3E2	0.59105
P0C206	Q96I24	0.54083
P0C206	O14512	0.59184
P0C206	A6NEQ2	0.45994
P0C206	Q86YV9	1.1
P0C206	O75525	0.78463
P0C206	Q6PEY1	0.89197

P0C206	P08397	0.29405
P0C206	Q5TA78	0.45994
P0C206	O75570	0.45254
P0C206	Q7Z460	0.53173
P0C206	Q15390	0.26256
P0C206	A6NF36	0.57969
P0C206	Q6ZRP5	0.45994
P0C206	Q9H6B1	0.51176
P0C206	Q7Z5Y7	0.13145
P0C206	Q9HBH1	0.73255
P0C206	Q9NVM9	1.3132
P0C206	A6NL46	0.9591
P0C206	A1L3Z2	0.15876
P0C206	Q02577	0.72154
P0C206	O76031	0.61295
P0C206	Q9C0F1	0.47609
P0C206	Q7Z6E9	0.61163
P0C206	Q9NTG7	0.69813
P0C206	P20719	0.22596
P0C206	Q96B01	0.64647
P0C206	O75351	0.42459
P0C206	C9JLW8	0.61032
P0C206	Q9NTX7	0.6529
P0C206	Q9NS23	0.59524
P0C206	O95391	0.26739
P0C206	Q8N339	0.37631
P0C206	Q8TCN5	0.20949
P0C206	Q7Z6B0	0.63073
P0C206	P08151	0.40796
P0C206	Q9BZF9	0.6052
P0C206	P20396	0.50987
P0C206	Q969T9	1.2402
P0C206	Q5VZV4	0.46953
P0C206	O43924	0.21637
P0C206	A6NES4	0.42105
P0C206	Q8WWB3	0.012471
P0C206	Q9Y2W2	0.75704
P0C206	Q13118	0.49748
P0C206	Q8NC74	0.41707
P0C206	Q8TAC9	0.64174
P0C206	P07738	0.78224
P0C206	Q8TAU0	0.2486
P0C206	Q15929	0.52526
P0C206	Q05877	0.84893
P0C206	Q13424	0.35119
P0C206	Q8N668	0.93529
P0C206	P08621	0.251
P0C206	Q7L5N1	1.1956
P0C206	P0CAT3	0.016902
P0C206	Q8IU81	0.44908
P0C206	O75143	1.1597
P0C206	Q8NHX4	0.55286
P0C206	P02795	0.74749
P0C206	O95685	0.37167
P0C206	Q9P2D0	0.35201
P0C206	P48552	0.70134
P0C206	Q5TFG8	0.38327
P0C206	Q6ZMV5	0.56283
P0C206	Q06250	0.45994
P0C206	Q49AN0	0.27497
P0C206	Q5JVG8	0.50739
P0C206	Q9NUK0	0.096735
P0C206	P54826	0.23919
P0C206	Q9UN81	0.50318
P0C206	Q9Y221	0.81213
P0C206	Q5VSD8	0.14569
P0C206	A1L4L8	0.64331
P0C206	Q96ER9	0.2556
P0C206	Q9HBF5	0.83987
P0C206	Q13347	0.74204
P0C206	P87889	0.16645
P0C206	P48539	0.27078
P0C206	Q6ZN55	0.30741
P0C206	P46779	1.0234
P0C206	Q9NQS7	1.0845
P0C206	Q14203	1.1036

P0C206	Q5VV52	0.26569
P0C206	Q9BQ83	0.78748
P0C206	Q6ZS94	0.45994
P0C206	Q9UJ72	0.1565
P0C206	Q9NTW7	0.50505
P0C206	Q6ZNG0	0.32699
P0C206	P17038	0.4071
P0C206	Q9NPL8	0.7007
P0C206	Q00403	1.2514
P0C206	Q59EK9	0.55905
P0C206	Q96PP4	0.13742
P0C206	Q9BYM8	0.23391
P0C206	A6NK58	0.35252
P0C206	Q8NEZ5	0.81499
P0C206	Q8WWM7	0.627
P0C206	Q53G59	0.59697
P0C206	Q01130	0.66096
P0C206	P30405	0.28126
P0C206	A6NN14	0.4071
P0C206	O15117	0.70943
P0C206	Q6XCG6	0.45994
P0C206	Q01469	0.29927
P0C206	Q8TEE9	1.0906
P0C206	A6NL82	0.3701
P0C206	O60675	0.54125
P0C206	Q5JU67	0.13201
P0C206	Q86VM9	0.35131
P0C206	O14802	0.28777
P0C206	Q9NQZ2	0.19991
P0C206	Q8ND61	0.90084
P0C206	P15822	1.1087
P0C206	Q9H3H3	0.28043
P0C206	Q8NB42	0.53541
P0C206	P07954	0.17441
P0C206	Q6ZV65	0.33241
P0C206	A6NEQ9	0.45994
P0C206	A7E2U8	0.48763
P0C206	O75935	1.0824
P0C206	Q9UL46	0.31616
P0C206	Q9NZD4	0.64093
P0C206	Q96M53	1.1874
P0C206	Q9UGB4	0.45994
P0C206	Q15776	0.68852
P0C206	Q66K89	0.93943
P0C206	O43310	0.51385
P0C206	O60711	0.64748
P0C206	P31948	1.0927
P0C206	Q15154	0.67339
P0C206	Q6Q759	0.77148
P0C206	Q96PT4	0.27096
P0C206	Q9BY89	0.23834
P0C206	Q8NCK3	0.37259
P0C206	Q96B02	0.34952
P0C206	O14558	0.86766
P0C206	Q16613	0.26459
P0C206	Q8NFH3	1.034
P0C206	Q92547	0.83777
P0C206	Q9NX05	0.51473
P0C206	Q9BYR8	1.0016
P0C206	P02042	0.41469
P0C206	P52294	1.1214
P0C206	Q5VTE6	0.091748
P0C206	Q8N6H7	0.23091
P0C206	Q68DI1	0.30556
P0C206	A5PLN9	0.53713
P0C206	Q9NXB0	0.94993
P0C206	Q6PG37	0.54509
P0C206	Q93073	0.73854
P0C206	Q15323	0.41992
P0C206	P21912	0.25193
P0C206	Q8NA03	0.55898
P0C206	O15297	0.71257
P0C206	Q2NL82	0.59362
P0C206	Q96Q06	0.90603
P0C206	P63172	0.93571
P0C206	Q3YBR2	0.61709

P0C206	Q00059	0.92652
P0C206	Q8IYK2	0.75655
P0C206	Q13105	0.62535
P0C206	Q06830	0.27731
P0C206	P41161	0.27295
P0C206	Q86WG3	0.38408
P0C206	Q3KQU3	0.79987
P0C206	Q5TCM9	0.45994
P0C206	A0JLT2	1.0197
P0C206	P63220	0.59441
P0C206	O14609	0.85783
P0C206	P0CG30	0.44089
P0C206	P36873	0.82811
P0C206	Q13042	1.3754
P0C206	H0YKK7	0.88867
P0C206	Q6NVV0	0.72147
P0C206	P0CG00	0.37883
P0C206	Q8N4C0	0.097618
P0C206	Q8IYB7	0.26316
P0C206	A8K0S8	0.31537
P0C206	Q9BRQ6	1.2716
P0C206	O14513	0.65996
P0C206	Q9Y252	0.57815
P0C206	P31249	0.58925
P0C206	Q6NUR6	0.67636
P0C206	Q8N443	0.40274
P0C206	P12035	0.4573
P0C206	P42224	0.23617
P0C206	P60412	0.72851
P0C206	P62310	0.57834
P0C206	Q5SZD4	0.6401
P0C206	O15126	0.45843
P0C206	P47928	0.4938
P0C206	Q969S8	0.90939
P0C206	Q9NRP2	0.92384
P0C206	P60842	0.066905
P0C206	P02489	0.39106
P0C206	Q9P2X3	0.16346
P0C206	P58005	0.34143
P0C206	Q5RHP9	0.43445
P0C206	Q9H1B7	1.0357
P0C206	Q96IU4	0.56782
P0C206	Q8WV92	0.49319
P0C206	Q9H4W6	0.14295
P0C206	Q9UK53	0.25193
P0C206	Q9Y2Y1	0.20983
P0C206	P52736	0.81752
P0C206	Q9H040	0.72896
P0C206	O95177	0.20572
P0C206	A8MVS1	0.56088
P0C206	Q96E09	0.80301
P0C206	Q3KRA6	1.1722
P0C206	P50548	0.47897
P0C206	Q5JNZ5	0.62971
P0C206	Q6VB84	0.52107
P0C206	O76064	0.40914
P0C206	Q9UBX0	0.25859
P0C206	Q8IYL2	0.53639
P0C206	Q96PC3	0.45645
P0C206	Q9Y6I8	0.31941
P0C206	Q8WXG1	0.10013
P0C206	Q9HBM0	0.61028
P0C206	Q96MW7	0.44813
P0C206	Q9BXD5	0.68658
P0C206	Q5TC12	0.56479
P0C206	Q9NPC6	0.89755
P0C206	Q9Y6C7	0.45994
P0C206	A3KN83	0.95479
P0C206	Q9BSW2	0.92473
P0C206	Q4G163	0.12969
P0C206	Q0P140	0.26483
P0C206	A5A3E0	0.16333
P0C206	Q96A44	0.61261
P0C206	P52758	0.61083
P0C206	Q9H469	0.34026
P0C206	Q9H9Y6	0.83436

6917	P0C206	P0CW26	0.39488
6918	P0C206	Q8ND83	1.195
6919	P0C206	Q96DY2	0.16979
6920	P0C206	P13051	1.1504
6921	P0C206	Q99576	0.57737
6922	P0C206	Q92540	1.0369
6923	P0C206	Q92499	0.43934
6924	P0C206	A8MUX0	0.49553
6925	P0C206	Q8NHQ1	1.535
6926	P0C206	Q8TDC0	0.61674
6927	P0C206	Q5BKX8	0.18463
6928	P0C206	Q8NHQ8	0.90673
6929	P0C206	A6NNA2	0.72905
6930	P0C206	Q8IY18	0.57529
6931	P0C206	Q8TB05	0.68336
6932	P0C206	Q969H4	0.26131
6933	P0C206	P22033	0.51424
6934	P0C206	Q9UMS0	1.1867
6935	P0C206	Q9BXJ9	0.7744
6936	P0C206	Q99549	0.80996
6937	P0C206	Q15643	1.1636
6938	P0C206	Q8TDZ2	0.25382
6939	P0C206	Q9H8M1	0.17829
6940	P0C206	Q9BU89	0.27007
6941	P0C206	P50748	0.9594
6942	P0C206	P49356	0.22985
6943	P0C206	O60563	1.5947
6944	P0C206	Q9BXM8	0.45994
6945	P0C206	Q5VWK0	0.079262
6946	P0C206	Q15398	1.1239
6947	P0C206	Q96EY1	0.47406
6948	P0C206	Q96K31	0.4838
6949	P0C206	Q14693	0.41629
6950	P0C206	Q68DN1	0.34873
6951	P0C206	Q9BXY0	0.32773
6952	P0C206	Q9ULK2	0.82891
6953	P0C206	Q8WUY3	0.46383
6954	P0C206	Q96IK1	0.40745
6955	P0C206	Q9BSJ6	0.63617
6956	P0C206	Q96DM3	0.27643
6957	P0C206	Q9Y6M1	0.34142
6958	P0C206	Q9H3C7	1.0302
6959	P0C206	O43593	0.34784
6960	P0C206	Q96K58	0.28309
6961	P0C206	O00423	0.64474
6962	P0C206	Q5SWL8	0.26699
6963	P0C206	Q9UGR2	0.58324
6964	P0C206	Q6GPH4	0.62649
6965	P0C206	O00479	0.68165
6966	P0C206	P15880	0.87489
6967	P0C206	Q53H80	0.4795
6968	P0C206	B4DX44	0.57753
6969	P0C206	Q58A44	0.45994
6970	P0C206	Q96G25	1.0597
6971	P0C206	Q9Y244	0.50774
6972	P0C206	Q8WYQ5	0.70603
6973	P0C206	Q96RE9	0.29783
6974	P0C206	P35580	0.28432
6975	P0C206	Q96MD2	0.18785
6976	P0C206	O15027	0.79164
6977	P0C206	P12956	0.45675
6978	P0C206	P55197	1.6586
6979	P0C206	Q86YS3	0.25862
6980	P0C206	Q96NB1	0.29293
6981	P0C206	Q494X3	0.55028
6982	P0C206	Q96N03	0.44439
6983	P0C206	Q8IY92	0.52473
6984	P0C206	Q9P2N6	0.36304
6985	P0C206	Q9BYQ4	0.72851
6986	P0C206	Q9HAW4	0.58003
6987	P0C206	Q13227	1.1246
6988	P0C206	O43156	0.63871
6989	P0C206	Q9HAK2	0.14295
6990	P0C206	Q7RTT6	0.47586
6991	P0C206	Q9BW19	0.39724
6992	P0C206	Q96FT9	0.11978

P0C206	Q8NFV5	0.51209
P0C206	A7XYQ1	0.48467
P0C206	Q9NY61	0.98927
P0C206	O43189	0.80238
P0C206	Q6P3W6	0.80594
P0C206	Q8NCN5	0.072023
P0C206	Q96PM9	0.6229
P0C206	Q9BU02	0.80849
P0C206	Q9BQN1	1.0776
P0C206	Q9Y3X0	0.54851
P0C206	Q14119	0.42428
P0C206	P17014	0.30003
P0C206	Q9NZF1	0.33436
P0C206	P23528	0.76639
P0C206	A8K5M9	1.1171
P0C206	Q9NX20	0.54016
P0C206	H3BNL1	0.54687
P0C206	Q00005	0.62069
P0C206	P00338	0.54372
P0C206	Q9UBI9	0.72448
P0C206	Q9NU63	0.28849
P0C206	Q8NCE8	0.66614
P0C206	P42025	0.5174
P0C206	Q00839	0.29228
P0C206	Q15051	0.76707
P0C206	Q9Y2T7	0.2166
P0C206	Q15833	0.40791
P0C206	P78346	0.7873
P0C206	Q9NZU5	0.51507
P0C206	P46379	0.28476
P0C206	P13535	0.26858
P0C206	Q96KT0	0.0096923
P0C206	Q86T03	1.0188
P0C206	Q6P1R3	0.61896
P0C206	Q6ZT83	0.19163
P0C206	P53611	0.13549
P0C206	Q92618	0.20949
P0C206	P55210	0.28791
P0C206	Q96GK7	0.50112
P0C206	Q9H019	0.63251
P0C206	Q8NBA8	0.24992
P0C206	Q14498	0.69489
P0C206	Q96EH5	0.6927
P0C206	Q96CS3	0.66048
P0C206	Q8TAF5	0.45994
P0C206	Q8NEP9	0.51617
P0C206	Q9BQ52	0.30548
P0C206	A6ND36	0.024854
P0C206	Q8NAV2	0.17049
P0C206	Q96ME7	0.20762
P0C206	P0CAP2	0.305
P0C206	O95159	0.7351
P0C206	Q9ULM6	1.0473
P0C206	Q9C029	0.29992
P0C206	Q9UI36	0.48517
P0C206	Q8N1F7	0.73489
P0C206	Q8TAU3	0.33635
P0C206	Q96IP4	0.61338
P0C206	O43148	0.87227
P0C206	P25398	0.68562
P0C206	Q9UQB3	0.66476
P0C206	Q9UQC2	0.34024
P0C206	O00303	0.31117
P0C206	Q494R0	0.45994
P0C206	Q9H094	0.016113
P0C206	Q8IVS8	0.13105
P0C206	O75167	0.29087
P0C206	Q9NPA8	0.53241
P0C206	Q9Y6Y1	0.70375
P0C206	Q96CP2	0.23934
P0C206	Q6ZR03	0.34508
P0C206	Q9NRD5	0.2319
P0C206	P61579	0.80216
P0C206	A5PLN7	0.68336
P0C206	Q86VP6	0.83507
P0C206	O94983	1.4967

P0C206	Q68DX3	0.61933
P0C206	Q71F23	0.72431
P0C206	Q8IYB9	0.42186
P0C206	Q96D70	0.89679
P0C206	Q5JYT7	0.028656
P0C206	Q8N0Z2	0.46229
P0C206	Q6ZNA1	0.39234
P0C206	Q8TF46	0.29394
P0C206	P20073	0.45146
P0C206	P08708	0.42942
P0C206	Q8N3J5	0.031269
P0C206	Q6NT76	0.59309
P0C206	O75674	0.51498
P0C206	Q14153	0.66895
P0C206	Q6NT04	0.36518
P0C206	Q14493	0.79895
P0C206	O75461	0.43526
P0C206	Q96EF6	0.68262
P0C206	C9J302	0.31078
P0C206	Q1RN00	0.45994
P0C206	Q15935	0.59919
P0C206	Q15004	0.95404
P0C206	Q14DG7	0.60901
P0C206	O95876	0.38753
P0C206	O43264	0.72197
P0C206	Q96B54	0.75951
P0C206	P10515	0.4044
P0C206	Q7L945	0.36636
P0C206	Q96NS8	0.1544
P0C206	Q6IQ23	0.4693
P0C206	P24864	0.65297
P0C206	Q7Z3B3	0.22639
P0C206	Q9HCS7	0.50795
P0C206	Q9Y5P8	0.72052
P0C206	Q5T9G4	1.2902
P0C206	Q01105	0.82895
P0C206	P09016	0.18582
P0C206	Q9UL40	0.75129
P0C206	Q01892	0.48285
P0C206	P24941	0.70518
P0C206	P78412	0.31537
P0C206	Q15369	1.4029
P0C206	Q15788	0.29258
P0C206	Q08AG7	0.87507
P0C206	Q08945	1.3558
P0C206	Q86YT6	0.24726
P0C206	Q15759	0.30449
P0C206	Q9NYV6	0.60031
P0C206	Q8N3U1	0.45994
P0C206	O75820	0.28063
P0C206	Q9Y5A7	0.76609
P0C206	Q9NZ09	0.13763
P0C206	Q8TEA8	0.9133
P0C206	Q96SR6	0.33397
P0C206	O00189	0.50669
P0C206	Q8WWK9	0.4917
P0C206	Q8TDD2	0.51466
P0C206	Q8TD16	0.46378
P0C206	Q9BSJ2	1.3337
P0C206	Q14061	0.52057
P0C206	Q5T0J7	0.271
P0C206	Q96MG8	0.029555
P0C206	O43639	0.46023
P0C206	Q9UKT5	0.4597
P0C206	P62937	0.66381
P0C206	O43765	0.70613
P0C206	Q1ZZU3	0.85437
P0C206	Q8TBK6	0.46758
P0C206	Q9HCI5	0.73611
P0C206	Q9H1D9	0.64115
P0C206	Q9UNX4	0.42361
P0C206	O96000	0.47814
P0C206	Q13765	1.2392
P0C206	O43745	0.86192
P0C206	Q5TBC7	0.29189
P0C206	A0AUK8	0.24246

P0C206	O95071	0.28494
P0C206	P60896	0.45994
P0C206	P13929	0.20897
P0C206	Q9BUT9	1.2905
P0C206	Q9BQ95	1.2188
P0C206	Q2M2W7	0.68336
P0C206	Q6UUV7	1.6413
P0C206	Q9H4R4	0.10546
P0C206	Q9UBF1	0.94092
P0C206	P62952	0.33364
P0C206	Q9UDW3	0.4984
P0C206	Q5T750	0.26479
P0C206	P23381	0.31867
P0C206	P16455	0.32127
P0C206	P02533	0.87109
P0C206	O75312	0.43373
P0C206	Q7Z2E3	0.65478
P0C206	Q5HY64	0.11084
P0C206	A8MYR8	0.090088
P0C206	Q2M218	0.33397
P0C206	Q93074	0.52293
P0C206	Q68CZ6	0.80409
P0C206	Q6NXP2	1.0591
P0C206	Q14249	0.048089
P0C206	Q6BDI9	0.60054
P0C206	P51668	0.58319
P0C206	Q08AI8	0.68336
P0C206	Q6ZVN7	0.07209
P0C206	P0CG33	0.3004
P0C206	Q9Y3T9	1.1777
P0C206	Q9ULJ1	0.24144
P0C206	P36402	0.56528
P0C206	Q5BIV9	0.50729
P0C206	Q9H633	0.36817
P0C206	Q8N163	0.76934
P0C206	P0CJ68	0.67101
P0C206	Q15459	0.52585
P0C206	Q8NCA5	0.46522
P0C206	Q14929	0.32132
P0C206	Q8TD17	0.26214
P0C206	Q9UH77	0.69196
P0C206	P11940	0.28803
P0C206	Q9H9C1	0.69673
P0C206	A8MTZ0	0.44326
P0C206	Q14566	0.28482
P0C206	A6NCW3	0.63324
P0C206	P18848	1.1867
P0C206	Q8N4H5	0.41064
P0C206	O15304	1.1667
P0C206	P11142	0.36997
P0C206	A6NC78	0.33004
P0C206	Q6ZTN6	0.47738
P0C206	Q8N328	0.074542
P0C206	Q12959	0.84297
P0C206	Q5QP74	0.45994
P0C206	Q8WVM7	0.94388
P0C206	Q0VG99	0.32028
P0C206	Q9NQ31	0.74141
P0C206	Q96DP5	0.57758
P0C206	Q5VZF2	0.10158
P0C206	Q9NRM2	0.16664
P0C206	Q96EK4	1.4778
P0C206	Q8N3F8	0.73813
P0C206	Q8WVK2	0.64617
P0C206	Q99732	0.3779
P0C206	P50135	0.20673
P0C206	Q9NXZ1	0.32773
P0C206	Q96KR7	0.42467
P0C206	Q6ZSN1	0.38203
P0C206	Q9NTJ3	0.30467
P0C206	Q2KHT3	0.65507
P0C206	Q6IPW1	0.61883
P0C206	Q53FT3	0.46399
P0C206	A2VDF0	0.31063
P0C206	Q68CZ2	0.46821
P0C206	Q6P4R8	1.3299

P0C206	Q969E4	0.58122
P0C206	Q8IUE0	0.43565
P0C206	Q8NG31	0.76262
P0C206	Q14C87	0.74524
P0C206	Q5T681	0.98451
P0C206	Q8N5A5	1.0366
P0C206	P61970	0.54135
P0C206	Q96RE7	1.1985
P0C206	Q9Y615	0.52493
P0C206	Q9H2K2	0.49632
P0C206	P22735	0.25384
P0C206	O60422	0.40162
P0C206	Q68D10	0.68336
P0C206	Q96EK7	0.44019
P0C206	Q8NEY8	0.93484
P0C206	Q96ND8	0.54826
P0C206	Q86UY8	0.1551
P0C206	Q8TAB7	0.89129
P0C206	Q8N1G2	0.42617
P0C206	Q09MP3	0.35354
P0C206	Q9UBD0	1.2797
P0C206	Q6ZQX7	0.014649
P0C206	Q9Y3A2	0.98495
P0C206	Q96GN5	1.0209
P0C206	Q9Y3Y2	1.0504
P0C206	Q8IWN6	0.30824
P0C206	Q7Z401	0.46846
P0C206	Q12830	1.1559
P0C206	O60516	0.35247
P0C206	O00273	0.34005
P0C206	Q9BRX2	0.56306
P0C206	P06132	0.60704
P0C206	Q9UBG7	0.28382
P0C206	Q6ZTI6	0.76624
P0C206	Q8N945	0.1127
P0C206	Q15714	0.88562
P0C206	Q96IU2	1.1068
P0C206	O43674	0.48065
P0C206	Q96LR7	0.98628
P0C206	Q8IZM0	0.32852
P0C206	Q3LI63	0.45994
P0C206	Q8NC51	0.73761
P0C206	Q96LW1	0.51209
P0C206	Q9Y266	1.1081
P0C206	P0CL84	0.26555
P0C206	Q9NP85	0.11483
P0C206	P41002	1.1478
P0C206	Q9NVN3	0.12215
P0C206	H3BTG2	0.51403
P0C206	Q9HD26	1.0106
P0C206	Q9NQS1	0.27723
P0C206	P56385	0.29911
P0C206	Q92834	0.47378
P0C206	Q8IU60	0.91072
P0C206	Q86SQ9	0.18473
P0C206	Q5T655	0.45994
P0C206	O60806	0.54659
P0C206	Q6VVB1	0.51083
P0C206	Q96IL0	0.34485
P0C206	Q6EMB2	0.28999
P0C206	Q9H8M7	0.92846
P0C206	Q9NYY8	0.24182
P0C206	O43763	0.26881
P0C206	Q8N7B1	0.71676
P0C206	P29372	0.60352
P0C206	Q3MIV0	0.45994
P0C206	Q9BTD1	0.32681
P0C206	P48728	0.21514
P0C206	Q9Y4W2	0.92789
P0C206	Q7L2E3	0.34196
P0C206	O00233	0.4967
P0C206	P52292	1.0256
P0C206	O15442	0.12895
P0C206	Q86T75	0.90881
P0C206	A2AJT9	0.48288
P0C206	P38432	0.8231

P0C206	Q7Z7F7	0.70285
P0C206	Q99750	0.52602
P0C206	O95571	0.66773
P0C206	P47914	1.1539
P0C206	Q13136	0.39748
P0C206	Q8N5Y2	0.1114
P0C206	O75150	0.64265
P0C206	Q86Y46	0.31106
P0C206	Q8IXQ9	0.3695
P0C206	P07476	0.56872
P0C206	Q5TB80	1.1356
P0C206	Q96CM3	0.61779
P0C206	Q9Y618	1.1671
P0C206	Q6EKJ0	0.23143
P0C206	P11802	0.80246
P0C206	O95789	0.78244
P0C206	Q9UIL8	0.60244
P0C206	P61578	0.62585
P0C206	O95714	0.31135
P0C206	Q8WY91	0.5773
P0C206	P37837	0.74472
P0C206	Q9P0R6	0.40115
P0C206	Q6ZRI6	0.53109
P0C206	Q86TA1	0.47492
P0C206	Q5U623	0.9839
P0C206	Q13472	0.14748
P0C206	Q6P3V2	0.30231
P0C206	Q86WW8	0.49654
P0C206	P68104	0.78122
P0C206	Q9BYI3	0.65476
P0C206	P14316	1.0423
P0C206	Q8WW33	0.70586
P0C206	Q9BYR2	0.72851
P0C206	O15235	0.78804
P0C206	O60232	1.2019
P0C206	Q9Y6X4	1.1498
P0C206	Q8WTU0	0.43611
P0C206	O95243	0.88402
P0C206	Q5T6S3	0.60244
P0C206	Q15528	1.3329
P0C206	Q5JSQ8	0.23043
P0C206	Q9BQC6	0.89389
P0C206	P48556	0.38485
P0C206	Q9NV39	0.47861
P0C206	Q01543	0.50578
P0C206	Q9H4E7	0.23604
P0C206	O75901	0.52857
P0C206	A8MXZ3	0.45994
P0C206	Q6P1J9	0.74607
P0C206	Q15257	0.51558
P0C206	Q5VZ66	0.48699
P0C206	Q9NYP3	0.60429
P0C206	Q9NUD5	0.3582
P0C206	P60602	0.50796
P0C206	A6NHQ4	0.58616
P0C206	Q9Y250	1.1175
P0C206	Q658Y4	0.61384
P0C206	Q9HBF4	0.40682
P0C206	Q49AR2	0.27105
P0C206	P09234	0.62568
P0C206	Q2M243	0.059371
P0C206	Q2M1V0	0.43565
P0C206	Q8TCF1	0.23888
P0C206	P10124	0.37373
P0C206	P15408	0.66561
P0C206	Q92560	0.2453
P0C206	Q8N398	0.69901
P0C206	Q12906	1.1401
P0C206	Q9BVC3	0.70103
P0C206	Q9Y6Z2	0.45994
P0C206	Q93052	0.62076
P0C206	Q9H5X1	0.24261
P0C206	Q8NCR3	0.35438
P0C206	Q99607	1.207
P0C206	P22674	0.53432
P0C206	Q8IYL3	0.91681

P0C206	P13646	0.44369
P0C206	Q4W4Y0	0.13115
P0C206	P51116	0.6349
P0C206	P11473	0.49894
P0C206	Q9Y534	0.34965
P0C206	Q6P1M3	1.1225
P0C206	O96015	0.064731
P0C206	P17480	1.1505
P0C206	Q96DN6	0.70974
P0C206	Q8NAP3	0.56817
P0C206	Q13761	0.88231
P0C206	Q8IY81	0.82445
P0C206	Q9P1T7	1.3961
P0C206	Q9P255	0.40543
P0C206	Q8TAW3	0.3367
P0C206	P35658	1.0465
P0C206	Q9UHD9	0.95922
P0C206	O95868	0.67664
P0C206	Q9H6S1	0.080271
P0C206	Q9UJW9	1.5012
P0C206	Q5VWX1	0.74558
P0C206	Q7Z699	0.41057
P0C206	Q12772	0.27787
P0C206	Q5T0F9	0.56148
P0C206	Q9H902	0.24572
P0C206	Q9H2C0	0.7263
P0C206	Q12789	0.9661
P0C206	A6NJ08	0.17099
P0C206	P23025	0.34795
P0C206	Q8N960	0.21305
P0C206	Q8IVI9	0.36431
P0C206	Q5SY13	0.45994
P0C206	Q9H3H9	0.98343
P0C206	Q8IVD9	0.5348
P0C206	Q9BRX8	0.069612
P0C206	O00534	0.34708
P0C206	Q5VT03	0.75244
P0C206	Q2TAC2	0.022078
P0C206	O94921	0.27813
P0C206	Q8WYN3	0.67724
P0C206	Q9BTV4	0.57443
P0C206	Q5TAP6	0.53951
P0C206	P47897	0.44582
P0C206	Q8N6N6	0.077688
P0C206	Q3LHN1	0.45994
P0C206	P14868	0.69221
P0C206	Q14511	0.87212
P0C206	Q9UII2	0.66075
P0C206	P62847	0.65946
P0C206	Q9Y6H5	0.88285
P0C206	Q8IW40	0.18647
P0C206	Q9C073	0.99714
P0C206	Q8N8X9	0.42521
P0C206	Q6ZTU2	0.65823
P0C206	Q86Y13	0.41345
P0C206	P00480	0.26043
P0C206	A6NDY2	0.51817
P0C206	Q6L8H4	0.45994
P0C206	Q9UIF7	0.53677
P0C206	Q9NS73	0.87657
P0C206	P47974	0.39938
P0C206	Q8TC56	1.2191
P0C206	Q9NZB2	0.72508
P0C206	Q96RJ6	0.50307
P0C206	Q96FQ6	0.41303
P0C206	O95166	0.35824
P0C206	Q9Y2A4	0.53032
P0C206	P52630	0.76461
P0C206	Q9H267	0.38606
P0C206	Q9Y2G5	0.31847
P0C206	Q5T200	0.82629
P0C206	A8MU10	0.45994
P0C206	P78371	0.65283
P0C206	Q9Y262	0.90247
P0C206	O14978	0.61557
P0C206	Q9H8G1	0.5656

P0C206	P13667	0.32609
P0C206	Q66K41	0.82861
P0C206	Q9HBL8	0.059505
P0C206	Q14159	1.423
P0C206	Q6TAR4	0.28101
P0C206	Q9NQP4	0.98427
P0C206	A8MWA4	0.57688
P0C206	Q7Z5L9	1.0357
P0C206	Q9C0I3	0.99028
P0C206	Q16401	0.76743
P0C206	Q9H7Y2	0.2572
P0C206	Q9BRX5	0.069799
P0C206	P30740	0.35971
P0C206	Q9NNX9	0.97625
P0C206	Q86WC4	0.99821
P0C206	Q9NP50	0.61271
P0C206	Q9H0A0	0.44277
P0C206	P35900	0.91573
P0C206	Q86X02	0.36531
P0C206	O14990	0.47346
P0C206	Q8TCZ7	0.5171
P0C206	Q6ZN08	0.52324
P0C206	Q8IW50	0.68336
P0C206	O00471	0.38442
P0C206	P0CG42	0.45994
P0C206	Q9BZD3	0.33259
P0C206	P31943	0.31368
P0C206	Q12800	0.4373
P0C206	Q9Y3C5	0.66822
P0C206	Q12834	0.89264
P0C206	Q8IWJ2	0.81715
P0C206	Q9BV97	0.34344
P0C206	Q5HYK3	0.40033
P0C206	Q7Z6I8	0.062544
P0C206	Q9BX63	0.34776
P0C206	P61254	0.96471
P0C206	Q8WVK7	0.80425
P0C206	Q6Q0C0	0.38855
P0C206	P0CJ70	0.67101
P0C206	Q8IVP9	0.31636
P0C206	Q13367	0.65647
P0C206	P08235	0.51164
P0C206	Q69YI7	1.2573
P0C206	P13985	0.45994
P0C206	Q9H0E9	0.37849
P0C206	Q9P2W9	0.37298
P0C206	P0CJ78	0.23862
P0C206	Q5TAG4	0.034477
P0C206	Q9H7J1	0.59416
P0C206	Q8NDH2	0.063596
P0C206	Q9H3L0	0.74782
P0C206	O43316	0.30202
P0C206	Q5VTL8	0.79556
P0C206	Q8NG50	0.15857
P0C206	Q6ZU35	0.68336
P0C206	P56556	0.37777
P0C206	A6NHP3	0.51209
P0C206	Q6NUN7	0.45994
P0C206	Q96LZ2	0.025788
P0C206	Q9NTK1	0.79765
P0C206	P31689	0.57168
P0C206	Q4W5G0	0.59254
P0C206	Q9BZL4	0.66879
P0C206	Q969T7	0.08705
P0C206	Q6I9Y2	0.78098
P0C206	O95171	0.36572
P0C206	Q05084	0.47159
P0C206	Q9Y6E7	0.53604
P0C206	A8MUV8	0.52256
P0C206	Q8IWT0	0.083489
P0C206	Q02221	0.20811
P0C206	Q6ZS92	0.19782
P0C206	Q9BYT5	0.17342
P0C206	Q5MJ70	0.51209
P0C206	Q8IXM7	0.25551
P0C206	Q99456	0.48752

P0C206	Q96S06	0.7423
P0C206	Q96C03	0.47435
P0C206	O95677	0.27849
P0C206	Q8WWM9	0.24398
P0C206	Q5VT52	0.30778
P0C206	Q9H467	1.188
P0C206	P51504	0.35271
P0C206	Q99856	0.7858
P0C206	Q96DV4	0.36958
P0C206	Q9Y2J2	0.52127
P0C206	A8MU76	0.92281
P0C206	Q9UI95	1.4135
P0C206	Q9BYG4	0.8455
P0C206	Q6P5X5	0.88253
P0C206	Q9BQJ4	0.7933
P0C206	Q9BSH3	0.2779
P0C206	A6NF83	0.78844
P0C206	Q9UI25	0.45994
P0C206	Q8NHY5	1.0382
P0C206	Q969U6	0.25122
P0C206	P52434	0.45663
P0C206	P58304	0.36091
P0C206	Q9BQ70	1.5118
P0C206	P35638	0.81954
P0C206	Q6IPR3	0.26169
P0C206	Q9H0B3	0.26921
P0C206	Q08AE8	0.16393
P0C206	Q9NP62	0.2104
P0C206	P50570	0.35824
P0C206	Q99496	0.61277
P0C206	P82912	0.57307
P0C206	O94805	0.78976
P0C206	Q5MJ08	0.061293
P0C206	Q3B7T1	0.66571
P0C206	Q8N6U2	0.43651
P0C206	Q96RL7	0.92397
P0C206	Q6NZ67	0.91007
P0C206	P17706	0.26003
P0C206	Q96C34	0.9469
P0C206	Q86XD5	0.25204
P0C206	Q15276	0.26935
P0C206	Q8IWF7	0.30996
P0C206	O76042	0.66938
P0C206	Q9NXX6	0.57448
P0C206	Q8N108	1.3095
P0C206	Q9UKY7	0.92901
P0C206	Q3KP31	0.50104
P0C206	Q8WWR9	0.35176
P0C206	O15116	0.74536
P0C206	Q8NBV4	0.55902
P0C206	P18887	0.79679
P0C206	Q8TAG9	0.1418
P0C206	P21399	0.3935
P0C206	P60369	0.72851
P0C206	O60224	0.26627
P0C206	Q5VTQ0	0.29677
P0C206	P29966	1.208
P0C206	Q6ZMS7	0.38136
P0C206	Q8IV32	0.57656
P0C206	Q99640	0.2503
P0C206	Q86WX3	1.2765
P0C206	Q6ZVC0	0.84349
P0C206	Q0P670	1.1394
P0C206	Q96EG3	0.25958
P0C206	Q9BTA0	0.68336
P0C206	Q5T0Z8	0.68336
P0C206	Q8N0W5	0.29506
P0C206	P0CW00	0.32628
P0C206	Q15561	0.94707
P0C206	Q8IUG1	0.50253
P0C206	Q5SVQ8	0.38197
P0C206	Q9ULH7	0.99853
P0C206	P17098	0.51603
P0C206	Q9BVC4	0.49528
P0C206	P82094	1.5566
P0C206	Q6ZRV3	0.45994

P0C206	O15372	0.7122
P0C206	Q9BW27	0.86754
P0C206	O15160	0.20779
P0C206	Q14207	0.99891
P0C206	Q2KJ03	0.26385
P0C206	Q9BRT3	0.24806
P0C206	Q96PU4	0.50382
P0C206	Q7L592	0.4683
P0C206	Q9BW30	0.33786
P0C206	Q8N3C0	0.48318
P0C206	Q92698	0.43634
P0C206	Q8NHZ8	1.3037
P0C206	Q9H7U1	1.1003
P0C206	Q9GZU3	1.2197
P0C206	Q69383	0.80216
P0C206	Q6ZN01	0.67833
P0C206	Q8IX03	1.2133
P0C206	Q8TAX7	0.45994
P0C206	Q9BSD3	0.37515
P0C206	Q969L2	0.31372
P0C206	P18669	0.80366
P0C206	A8MT69	0.6828
P0C206	O75323	0.63277
P0C206	Q9NWV4	0.66994
P0C206	Q96EY5	0.61417
P0C206	O75182	1.2483
P0C206	P59052	0.41658
P0C206	P33992	0.34217
P0C206	Q9UJW7	0.29385
P0C206	Q8NCU4	0.43511
P0C206	Q96GW9	0.4341
P0C206	A6NJ78	0.77807
P0C206	Q5VWP3	0.9287
P0C206	O00522	0.29423
P0C206	P31260	0.37103
P0C206	Q9P1A6	0.26639
P0C206	Q8N402	0.12863
P0C206	Q5T5A4	0.39017
P0C206	Q03188	0.59556
P0C206	Q9UJC5	0.53308
P0C206	Q9BWX1	0.59369
P0C206	P0CI00	0.67195
P0C206	P55957	0.49642
P0C206	Q5T0B9	0.20949
P0C206	O60688	0.22952
P0C206	Q9H1A7	0.15285
P0C206	Q9UKS6	0.3345
P0C206	Q5BKY9	0.68336
P0C206	Q16600	0.32332
P0C206	O95396	0.08442
P0C206	P17041	0.27985
P0C206	Q12904	0.1442
P0C206	P14618	0.2093
P0C206	P0C7V0	0.45994
P0C206	P62136	0.72208
P0C206	Q2NL98	0.89064
P0C206	Q9H2J4	0.74719
P0C206	Q9HCK0	0.23725
P0C206	Q969G9	0.22043
P0C206	Q5T6M2	0.8434
P0C206	Q8WW24	0.39786
P0C206	Q9H095	0.35529
P0C206	Q5JWR5	0.3562
P0C206	Q8N201	0.4179
P0C206	P0CG20	0.23867
P0C206	Q9UJW3	0.74836
P0C206	Q96NG5	0.51573
P0C206	Q9UNL4	0.57235
P0C206	Q92585	1.0677
P0C206	Q9UKA4	0.14678
P0C206	Q495Y7	0.073896
P0C206	Q9UGY1	0.34343
P0C206	Q8N8G2	1.0443
P0C206	O94763	0.84483
P0C206	Q9BVL2	0.7726
P0C206	Q96CW6	0.84275

P0C206	Q10571	0.26385
P0C206	Q9P2G4	0.68336
P0C206	Q9NVV9	1.3175
P0C206	P56377	0.48133
P0C206	O75822	0.74204
P0C206	O75425	0.75722
P0C206	P35908	0.4069
P0C206	Q9Y2S7	0.83144
P0C206	Q96JM2	0.42182
P0C206	Q6H8Q1	0.55944
P0C206	Q3ZLR7	0.7778
P0C206	Q92748	1.207
P0C206	O15014	1.0886
P0C206	Q15428	0.43152
P0C206	Q5TBE3	0.37602
P0C206	Q93050	0.26309
P0C206	Q8IZD2	0.44689
P0C206	Q05DH4	1.0937
P0C206	Q9Y5S9	0.60335
P0C206	Q9NZQ3	0.80319
P0C206	Q86V81	0.63704
P0C206	P0CJ77	0.67101
P0C206	Q9NW38	0.51629
P0C206	Q14671	0.18798
P0C206	P11171	0.64506
P0C206	Q12873	0.87824
P0C206	Q99986	0.25538
P0C206	O60290	0.56193
P0C206	Q9NSK7	0.97842
P0C206	Q9UKV3	0.64597
P0C206	P10827	0.34346
P0C206	Q6GMV2	0.3918
P0C206	Q86X76	0.17173
P0C206	P61604	0.84296
P0C206	O00566	0.6578
P0C206	Q14695	0.51287
P0C206	Q2TAP0	0.29426
P0C206	Q9UI26	0.56175
P0C206	Q9HBZ2	0.40155
P0C206	Q7Z7A3	0.63681
P0C206	Q9P2W6	0.45994
P0C206	Q8IXY8	0.24731
P0C206	Q96T21	0.5514
P0C206	B3EWG4	0.68336
P0C206	Q53FD0	0.87774
P0C206	Q902F8	0.8421
P0C206	Q8N2N9	0.55812
P0C206	Q9NTM9	0.4297
P0C206	Q96GY3	0.61356
P0C206	Q5JS54	0.61395
P0C206	O14746	0.66943
P0C206	Q5JSZ5	0.84433
P0C206	P0CV99	0.32628
P0C206	P36871	0.25165
P0C206	Q96NG8	0.35005
P0C206	Q6PRD7	0.45994
P0C206	Q14684	0.46932
P0C206	Q3BBV1	1.4923
P0C206	Q96LM1	0.81214
P0C206	Q9H7Z6	0.27502
P0C206	Q9UIG0	0.26285
P0C206	Q9HB09	1.2521
P0C206	Q9NWZ8	0.88007
P0C206	Q9Y238	0.12607
P0C206	P21675	0.46893
P0C206	Q53TQ3	0.79253
P0C206	O76095	0.43598
P0C206	Q96BD5	0.76061
P0C206	P14859	1.2679
P0C206	O43505	0.6143
P0C206	A6NDK9	0.1912
P0C206	Q9UNS2	0.79038
P0C206	P56177	0.24586
P0C206	A6NGS2	0.457
P0C206	Q4LE28	0.96648
P0C206	O00231	0.35283

P0C206	Q07866	0.78869
P0C206	P47944	0.41429
P0C206	P51946	0.66998
P0C206	Q8IUE1	0.43565
P0C206	Q86UB9	0.87834
P0C206	P15259	0.75109
P0C206	P35813	0.34172
P0C206	Q9H079	0.49239
P0C206	Q9BYQ8	0.72851
P0C206	Q9GZP8	0.45994
P0C206	Q6ZQY7	0.50777
P0C206	Q96AH0	0.46009
P0C206	Q6P3S1	0.10641
P0C206	Q8WVP5	1.0327
P0C206	Q14764	1.1836
P0C206	Q10586	0.48038
P0C206	P80297	0.47297
P0C206	Q5VUE5	0.50608
P0C206	Q7RTT4	0.26627
P0C206	P0DI81	1.2158
P0C206	Q0VF49	0.45994
P0C206	O75190	0.78545
P0C206	O60264	0.30944
P0C206	Q9ULX9	0.52864
P0C206	Q9NRT5	0.22803
P0C206	O00186	0.82949
P0C206	P61923	0.5279
P0C206	P63272	1.129
P0C206	Q68CJ9	0.86862
P0C206	Q04695	0.47527
P0C206	Q5T749	0.81382
P0C206	O43790	0.45381
P0C206	P42677	0.93671
P0C206	Q9NXR7	0.54862
P0C206	A1L3X0	0.65817
P0C206	Q5JTC6	1.0895
P0C206	Q70IA6	0.59819
P0C206	O15488	0.13013
P0C206	Q14978	0.7238
P0C206	Q9Y312	0.35423
P0C206	P62829	1.1285
P0C206	P55822	0.58892
P0C206	O15037	0.61118
P0C206	A8MSY0	0.34893
P0C206	P40337	0.91049
P0C206	Q13794	0.50698
P0C206	Q96LZ7	0.1497
P0C206	Q96CD0	0.60244
P0C206	Q8NAP1	0.68336
P0C206	P60370	0.72851
P0C206	Q5M9Q1	0.93939
P0C206	Q96I59	0.55501
P0C206	Q15583	0.35857
P0C206	Q9Y284	0.81956
P0C206	Q9H4H8	0.63847
P0C206	P31350	0.37143
P0C206	Q9NP77	0.34893
P0C206	Q9BUE6	0.037409
P0C206	Q92764	0.34578
P0C206	Q9UKY1	0.55035
P0C206	P12270	0.75114
P0C206	Q9UL18	0.97721
P0C206	P08697	0.37832
P0C206	P17036	0.50233
P0C206	Q9NRA8	1.2317
P0C206	Q9NRN7	0.55779
P0C206	Q5VT40	0.3416
P0C206	Q96GE4	0.33507
P0C206	Q96NU7	0.25655
P0C206	Q68CP9	0.76285
P0C206	Q9UBY9	1.0946
P0C206	Q9BTC8	0.92295
P0C206	Q969L4	0.6061
P0C206	O95503	1.082
P0C206	Q5JSS6	0.23298
P0C206	P51948	0.57323

7829	P0C206	Q6P2I7	0.060383
7830	P0C206	Q86V97	0.50382
7831	P0C206	Q9BRP8	0.92348
7832	P0C206	Q9NUS5	0.70921
7833	P0C206	P23508	0.52186
7834	P0C206	P61236	0.3795
7835	P0C206	Q8TBZ5	0.34774
7836	P0C206	P48443	0.29027
7837	P0C206	B7ZAQ6	0.61911
7838	P0C206	Q8N1W2	0.22413
7839	P0C206	Q01954	0.34359
7840	P0C206	Q13506	0.55295
7841	P0C206	Q9Y2H8	0.33886
7842	P0C206	Q6IPR1	0.48502
7843	P0C206	Q9Y2I6	0.32768
7844	P0C206	Q05932	0.26108
7845	P0C206	Q9H063	0.43492
7846	P0C206	Q8N3J3	0.32175
7847	P0C206	P31276	0.6341
7848	P0C206	Q1W6H9	1.3813
7849	P0C206	P14625	0.30573
7850	P0C206	P63146	0.81893
7851	P0C206	Q9Y2K1	0.20949
7852	P0C206	P78543	0.76838
7853	P0C206	P52815	0.56666
7854	P0C206	Q6ZTC4	0.45994
7855	P0C206	A6NCS6	0.55499
7856	P0C206	P24385	0.64753
7857	P0C206	Q96G01	0.23953
7858	P0C206	Q8WTW4	0.47303
7859	P0C206	Q8IWT3	0.29825
7860	P0C206	Q76KD6	0.55246
7861	P0C206	Q08AM6	0.26509
7862	P0C206	Q13360	0.33149
7863	P0C206	O00425	0.40557
7864	P0C206	O95229	0.94428
7865	P0C206	Q9BSF0	0.46311
7866	P0C206	P54253	1.0008
7867	P0C206	P19474	0.47762
7868	P0C206	Q6ZP98	0.39347
7869	P0C206	Q9BYD3	0.82048
7870	P0C206	Q08E93	0.45994
7871	P0C206	B1AH88	0.45994
7872	P0C206	Q9NYK6	0.45025
7873	P0C206	Q12894	0.5051
7874	P0C206	Q86UY5	0.22114
7875	P0C206	Q6ZVN6	0.051699
7876	P0C206	Q8NDT2	0.73828
7877	P0C206	Q8NFU3	0.23177
7878	P0C206	A2RUB1	0.68336
7879	P0C206	Q9NWD9	0.4639
7880	P0C206	P29401	0.068023
7881	P0C206	Q9BY60	0.13637
7882	P0C206	Q15046	0.75705
7883	P0C206	Q8WXC3	0.74589
7884	P0C206	Q8WW18	0.68336
7885	P0C206	Q9BU70	0.11493
7886	P0C206	Q8IUC2	0.90014
7887	P0C206	Q96BW9	0.55759
7888	P0C206	Q9H8X3	0.049727
7889	P0C206	O15060	0.20949
7890	P0C206	Q9H3K6	0.47487
7891	P0C206	P35227	0.85901
7892	P0C206	Q5H9M0	1.0384
7893	P0C206	O00411	0.61685
7894	P0C206	Q10570	0.72808
7895	P0C206	Q9Y2S6	0.45994
7896	P0C206	Q5T7V8	1.701
7897	P0C206	O75363	0.2364
7898	P0C206	Q6PIF2	0.42768
7899	P0C206	Q5T7P2	0.45994
7900	P0C206	Q9BTM9	0.93139
7901	P0C206	Q8WTZ4	0.021903
7902	P0C206	Q9Y281	0.62051
7903	P0C206	Q00534	0.63317
7904	P0C206	Q16594	0.67631

P0C206	Q8WV24	0.80638
P0C206	P62249	0.83277
P0C206	Q14653	0.65527
P0C206	Q9BRL7	0.67751
P0C206	Q2M3W8	0.5435
P0C206	Q8WVY7	0.38205
P0C206	Q15527	0.17244
P0C206	Q86YE8	0.40539
P0C206	A2RU30	0.65291
P0C206	Q70EK8	0.64437
P0C206	P62424	0.99094
P0C206	Q9BW60	0.65817
P0C206	P07197	0.85912
P0C206	P22528	0.37258
P0C206	Q8NEL9	0.06738
P0C206	P82921	0.74736
P0C206	Q6ZQN3	0.80621
P0C206	Q96PT3	0.27096
P0C206	Q96GU1	0.37431
P0C206	Q8WTQ4	0.061851
P0C206	P09914	0.37344
P0C206	Q9BVN2	0.74957
P0C206	Q3L8U1	1.0631
P0C206	Q9H1J1	0.84165
P0C206	Q59GN2	0.32831
P0C206	Q8TDR0	0.97778
P0C206	P0CG43	0.45994
P0C206	P16083	0.13671
P0C206	Q9Y2Y4	0.50756
P0C206	Q9H7R5	0.41874
P0C206	Q9BPW8	0.64303
P0C206	P12004	0.28779
P0C206	Q92905	0.79732
P0C206	Q8TDB4	0.35332
P0C206	P30281	0.92974
P0C206	P06400	0.70731
P0C206	Q9H9D4	0.79316
P0C206	Q9BVJ6	0.89511
P0C206	Q9H9H5	0.72048
P0C206	O43303	0.99069
P0C206	Q8IXF0	0.45145
P0C206	Q7L590	1.2039
P0C206	Q9NX08	0.44439
P0C206	Q6ZN19	0.27283
P0C206	Q13490	0.50533
P0C206	Q8NDY6	0.62032
P0C206	Q00587	0.36944
P0C206	Q00577	0.57911
P0C206	Q15029	0.28415
P0C206	C9J3I9	0.51775
P0C206	Q6DKI1	0.058094
P0C206	Q5T6C5	0.82891
P0C206	Q03938	0.53826
P0C206	Q96EL1	0.64868
P0C206	Q96BR5	0.078182
P0C206	Q02410	0.61747
P0C206	Q8TAZ6	0.5689
P0C206	P55347	0.51178
P0C206	Q9H8W3	0.5232
P0C206	Q9H2A3	0.25121
P0C206	Q96LY2	0.84842
P0C206	P61573	0.80216
P0C206	Q15181	0.38757
P0C206	O14715	0.045857
P0C206	Q9Y314	1.0711
P0C206	Q7L8L6	0.27276
P0C206	Q9Y3U8	0.87663
P0C206	Q8N8C0	0.36333
P0C206	P35573	0.53448
P0C206	O43830	0.27592
P0C206	Q9UQL6	0.57594
P0C206	Q96LX7	0.094487
P0C206	A8MZA4	0.53933
P0C206	Q96DY7	0.41724
P0C206	P49918	0.77138
P0C206	Q9NWS1	1.1834

P0C206	Q7Z7L8	1.2381
P0C206	Q9Y5U2	0.65534
P0C206	P50479	0.49231
P0C206	Q8WYR1	0.2995
P0C206	Q8N9A8	0.72891
P0C206	Q5TC04	0.082546
P0C206	Q9H606	0.34636
P0C206	P30876	0.73679
P0C206	Q8NDM7	0.6302
P0C206	Q8TE69	0.44439
P0C206	P78549	0.42306
P0C206	O75132	0.42104
P0C206	A8MTY7	0.72851
P0C206	Q9HAH1	0.47044
P0C206	Q96S95	0.61463
P0C206	Q8TES7	0.76608
P0C206	Q8IVV7	0.41679
P0C206	Q9UBQ5	1.0735
P0C206	Q6ZV50	0.49155
P0C206	P62917	0.99235
P0C206	Q9Y651	0.37484
P0C206	Q8NDY4	0.45994
P0C206	Q15436	0.78797
P0C206	O75155	0.8629
P0C206	Q9UMX2	0.26279
P0C206	Q6PEX3	0.51229
P0C206	Q9H0C8	0.57354
P0C206	O95948	0.44228
P0C206	Q01804	0.13613
P0C206	A6NCN2	0.43165
P0C206	Q6UX04	0.24926
P0C206	P50222	0.67048
P0C206	P56270	0.69951
P0C206	P57678	0.94104
P03363	Q8TDR0	0.77173
P03363	Q8N6F7	0.39965
P03363	Q1KMD3	0.34716
P03363	A2RRD8	0.22259
P03363	O60763	0.36379
P03363	Q9NWY4	0.22504
P03363	Q53FT3	0.36587
P03363	P80294	0.5938
P03363	Q719H9	0.56673
P03363	Q8IZX4	0.28062
P03363	Q96B26	0.61787
P03363	Q86TJ5	0.22259
P03363	Q49AA0	0.22259
P03363	A6NGQ2	0.23345
P03363	Q86X95	1.0271
P03363	Q6IE81	0.61757
P03363	Q9BQ89	0.75948
P03363	Q4G0X4	0.21694
P03363	Q96D46	0.61542
P03363	Q14139	0.68174
P03363	Q6XQN6	0.19021
P03363	Q9Y316	0.26634
P03363	O95376	0.54308
P03363	Q9P021	0.13901
P03363	Q5BJF6	0.58545
P03363	Q92886	0.26747
P03363	Q16236	0.52964
P03363	Q9BSD3	0.35801
P03363	Q9NQX1	0.50212
P03363	Q16587	0.17938
P03363	O95777	0.81209
P03363	P22681	0.34247
P03363	Q9H5Y0	0.042292
P03363	Q6NXP2	0.043997
P03363	Q9UJX2	0.29071
P03363	Q5H9J7	0.24431
P03363	Q9NPF5	0.81987
P03363	P56179	0.21693
P03363	Q6PRD7	0.24431
P03363	Q5TGS1	0.4337
P03363	Q6ZMW3	0.34776
P03363	Q6WQI6	0.24431

8057	P03363	O60832	0.64303
8058	P03363	Q96ST2	0.24232
8059	P03363	Q9Y6N9	0.35555
8060	P03363	Q15654	0.63493
8061	P03363	Q7RTS1	0.2634
8062	P03363	Q86VP1	0.42634
8063	P03363	Q8ND83	0.086557
8064	P03363	P49754	0.36641
8065	P03363	Q9UNN5	0.52784
8066	P03363	Q32P41	0.37586
8067	P03363	P46379	0.33115
8068	P03363	Q08AE8	0.27636
8069	P03363	Q3YBR2	0.55726
8070	P03363	Q9UBY9	0.79824
8071	P03363	Q96S94	0.43606
8072	P03363	Q13112	0.95197
8073	P03363	Q9NWF9	0.63121
8074	P03363	Q504Q3	0.2705
8075	P03363	Q5JUW0	0.24352
8076	P03363	Q9Y6Y0	0.57582
8077	P03363	Q86VE0	0.62696
8078	P03363	P78385	0.83681
8079	P03363	O15370	0.41953
8080	P03363	Q3Y452	0.12519
8081	P03363	Q15020	0.68132
8082	P03363	Q92547	0.72667
8083	P03363	O75940	0.8234
8084	P03363	P17706	0.25628
8085	P03363	Q9H8G2	0.044835
8086	P03363	P62913	0.60272
8087	P03363	O75717	0.55486
8088	P03363	P61236	0.29721
8089	P03363	O75179	0.45455
8090	P03363	Q8IXM2	0.29363
8091	P03363	Q6ZTI6	0.028146
8092	P03363	Q9UNQ2	0.17552
8093	P03363	Q86XU0	0.22259
8094	P03363	P05783	1.003
8095	P03363	Q9NX24	0.27349
8096	P03363	Q8TAK6	0.28947
8097	P03363	O75190	0.66194
8098	P03363	O75410	0.68272
8099	P03363	Q9H816	0.46952
8100	P03363	Q9Y2B5	0.23937
8101	P03363	Q15911	0.28491
8102	P03363	Q9Y5A6	0.55289
8103	P03363	Q9Y614	0.34579
8104	P03363	Q9NQT4	0.44956
8105	P03363	Q9NYG5	0.31856
8106	P03363	Q9H160	0.26193
8107	P03363	P80297	0.1646
8108	P03363	P49918	0.5455
8109	P03363	Q5T7N3	0.24431
8110	P03363	Q9P0L1	0.268
8111	P03363	Q14653	0.38243
8112	P03363	Q9Y6E7	0.25021
8113	P03363	Q96EA4	0.50567
8114	P03363	O14734	0.068572
8115	P03363	O95926	0.3599
8116	P03363	Q14146	0.29721
8117	P03363	Q8NB42	0.22259
8118	P03363	Q14978	0.35752
8119	P03363	O75069	0.15709
8120	P03363	Q86VX2	0.49242
8121	P03363	Q9Y2Z4	0.055714
8122	P03363	Q9Y6J0	0.29864
8123	P03363	P82650	0.25518
8124	P03363	Q86U70	0.1705
8125	P03363	A6NFF2	0.15989
8126	P03363	Q8WXW3	0.12015
8127	P03363	Q6L8H2	0.2934
8128	P03363	P43364	0.92347
8129	P03363	A8MQ14	0.22259
8130	P03363	Q9UNN4	0.43596
8131	P03363	Q53QV2	0.36283
8132	P03363	Q9NR55	0.61486

P03363	Q9Y6K5	0.21182
P03363	P62879	0.39672
P03363	A8K5M9	0.034826
P03363	Q9Y3P8	0.064846
P03363	A6NEF3	0.019317
P03363	Q5VTD9	0.37365
P03363	Q9BUL8	0.52554
P03363	Q14789	0.1103
P03363	P62314	0.84962
P03363	Q12959	0.35698
P03363	P41227	0.50079
P03363	O43399	0.70714
P03363	O75123	0.22259
P03363	Q15699	0.60924
P03363	P51858	0.22813
P03363	Q9C0F3	0.44662
P03363	Q92979	0.42834
P03363	Q96M53	0.14397
P03363	P52630	0.58456
P03363	Q9BQ50	0.16496
P03363	A6NGY1	0.22504
P03363	O95619	0.65624
P03363	P12956	0.21582
P03363	P23284	0.28725
P03363	Q14CX7	0.24431
P03363	Q9NPJ3	0.18058
P03363	Q9UBC0	0.27689
P03363	Q8N9T8	0.11913
P03363	Q8NHY6	0.22259
P03363	O95990	0.21151
P03363	O94762	0.2482
P03363	Q9NQG7	0.33974
P03363	Q16836	0.14818
P03363	Q9BSU1	0.39887
P03363	Q5T0B9	0.22259
P03363	Q86VS8	0.44293
P03363	O75953	0.72075
P03363	Q96JP0	0.29591
P03363	Q96QT6	0.56114
P03363	P13682	0.22474
P03363	Q6SJ96	0.48044
P03363	O75820	0.303
P03363	Q969F1	0.61051
P03363	Q96A44	0.74302
P03363	Q3KNS6	0.22259
P03363	Q9UGK3	0.69082
P03363	Q8TEE9	0.61357
P03363	Q8TE69	0.53508
P03363	Q86Y01	0.47894
P03363	Q9Y3C0	0.49798
P03363	Q9NW38	0.2845
P03363	Q9UFW8	0.42068
P03363	O15266	0.7583
P03363	Q92845	0.30935
P03363	Q99684	0.28853
P03363	Q96RS0	0.36181
P03363	O14787	0.94307
P03363	P18887	0.60263
P03363	Q06547	0.91527
P03363	Q3LI72	0.27762
P03363	Q52LG2	0.27762
P03363	Q16342	0.22031
P03363	P31273	0.31629
P03363	O43812	0.55694
P03363	Q9HAU0	0.46098
P03363	Q9H875	0.19914
P03363	A4D1E1	0.14739
P03363	Q03923	0.27255
P03363	Q32MZ4	1.0323
P03363	Q9BX63	0.32205
P03363	Q15233	0.73762
P03363	O60888	0.48139
P03363	Q8IUM7	0.25313
P03363	P43897	0.12704
P03363	Q15528	1.0199
P03363	P52758	0.35524

P03363	O00411	0.42155
P03363	Q9NVI1	0.62246
P03363	Q9Y5P8	0.55672
P03363	P02533	1.0007
P03363	Q9NVR2	0.90186
P03363	Q9Y535	0.20735
P03363	Q6P9A3	0.22259
P03363	G3V211	0.050543
P03363	Q99615	0.83659
P03363	Q9UKD1	0.59277
P03363	Q8NCK3	0.22259
P03363	A7MCY6	0.5149
P03363	Q9NZ09	0.24431
P03363	Q06455	0.55471
P03363	Q9Y2R5	0.23718
P03363	Q13490	0.25842
P03363	Q86SX3	0.14849
P03363	Q99829	0.25779
P03363	Q6ZMY9	0.22259
P03363	P17535	0.88492
P03363	Q8TB72	0.68055
P03363	Q5T953	0.0055957
P03363	Q5SZL2	0.38184
P03363	P35789	0.24904
P03363	O14901	0.2942
P03363	Q9UBW5	0.7338
P03363	O43395	0.93424
P03363	A8MSY0	0.43424
P03363	O60927	0.20425
P03363	O75373	0.22259
P03363	Q5T681	0.53508
P03363	Q5XKR9	0.029325
P03363	P62952	0.18889
P03363	O60829	0.11203
P03363	Q9BZZ5	0.537
P03363	Q9Y252	0.40938
P03363	Q5SVQ8	0.22259
P03363	Q6ZN17	0.83575
P03363	P98168	0.52218
P03363	Q9GZN2	0.54669
P03363	A6NKF1	0.75965
P03363	Q9Y2I6	0.34634
P03363	Q9H8N7	0.26814
P03363	P18848	0.85603
P03363	Q8NFU3	0.26239
P03363	Q9UIW0	0.35761
P03363	Q8TAX9	0.24431
P03363	Q7L273	0.21694
P03363	Q8N8G2	0.70463
P03363	Q8TDB6	0.54786
P03363	Q9UDT6	0.32715
P03363	Q9BUT9	0.10707
P03363	Q96IV0	0.52118
P03363	Q9ULW3	0.7299
P03363	Q16186	0.48564
P03363	O14744	0.64958
P03363	Q9UID3	0.27045
P03363	O94915	0.22927
P03363	O95751	0.67606
P03363	Q9BYD2	0.19909
P03363	Q69YH5	0.60055
P03363	Q8NI29	0.43705
P03363	Q15834	0.94443
P03363	P11137	0.32777
P03363	P08048	0.22259
P03363	Q9H9A7	0.67327
P03363	O75461	0.4772
P03363	Q8IXJ9	0.56005
P03363	Q8TC05	0.25312
P03363	Q14691	0.34898
P03363	O15205	0.56926
P03363	A8MWD9	0.416
P03363	Q96HM7	0.031479
P03363	Q9BYR9	0.2934
P03363	P09132	0.32763
P03363	Q9Y3E1	0.44627

P03363	Q9H7Z3	0.11203
P03363	Q96JC9	0.85391
P03363	Q86TC9	0.55008
P03363	Q9BUR4	0.796
P03363	O60739	0.10419
P03363	O75335	0.49568
P03363	P18621	0.36446
P03363	Q96H12	0.0592
P03363	P20810	0.33674
P03363	Q13105	0.42796
P03363	A6NKF2	0.69559
P03363	Q9NWT8	0.64164
P03363	Q8WXQ3	0.12034
P03363	Q9BZD4	0.47958
P03363	Q06787	0.8025
P03363	P35453	0.30181
P03363	Q8IZA3	0.1173
P03363	Q8IW19	0.44756
P03363	Q8N9Z9	0.70185
P03363	Q5T5P2	0.16099
P03363	P08779	0.96299
P03363	Q8IZP0	0.34846
P03363	Q13309	0.39624
P03363	Q9NZH5	0.34548
P03363	P29474	0.18845
P03363	P46060	0.28934
P03363	Q96JM7	0.38209
P03363	O75159	0.24017
P03363	P60370	0.2934
P03363	Q8IYX3	0.018765
P03363	O75081	0.63559
P03363	O75347	0.2422
P03363	Q9NVN8	0.38925
P03363	Q8NFW5	0.25249
P03363	Q8IZT9	0.22504
P03363	Q68DK7	0.2333
P03363	Q9H832	0.2068
P03363	Q8N9N2	0.46004
P03363	Q01850	0.93133
P03363	Q9BRT2	0.4199
P03363	P02808	0.063181
P03363	Q9BVC4	0.49008
P03363	Q5SVZ6	0.14658
P03363	P0C7T5	0.30512
P03363	Q9H2U2	0.2147
P03363	P17027	0.22259
P03363	Q15051	0.68176
P03363	P62258	0.4932
P03363	P49716	0.58724
P03363	P13994	0.55703
P03363	P0CH99	0.22259
P03363	Q3L8U1	0.78736
P03363	P55957	0.29844
P03363	P05386	0.72485
P03363	Q8N3V7	0.30129
P03363	Q9Y6H5	0.733
P03363	Q13151	0.23825
P03363	Q9UJ98	0.10034
P03363	Q96IK1	0.25402
P03363	Q9Y3F4	0.78198
P03363	Q5VUA4	0.41122
P03363	Q6N069	0.27389
P03363	P43360	0.56434
P03363	Q701N2	0.2934
P03363	O43493	0.26873
P03363	Q6NWY9	0.32049
P03363	Q9H2D6	0.26372
P03363	Q9NVV4	0.29245
P03363	O75363	0.24431
P03363	P46939	0.40565
P03363	Q9NSD4	0.22259
P03363	Q03989	0.3494
P03363	Q13595	0.23625
P03363	Q16082	0.85932
P03363	Q8N9F8	0.22259
P03363	O94972	0.27187

P03363	Q9HCC9	0.39787
P03363	Q13042	0.75265
P03363	Q9HD40	0.58364
P03363	O14907	0.48893
P03363	Q96RE7	0.70724
P03363	Q9Y4Y9	0.74947
P03363	Q86V71	0.22259
P03363	Q6IQ21	0.22259
P03363	Q9BXP5	0.93054
P03363	Q16649	0.44162
P03363	P49454	0.29207
P03363	Q96EP1	0.38403
P03363	Q8WUA4	0.2357
P03363	Q6VMQ6	0.68101
P03363	Q14765	0.48469
P03363	Q9Y592	0.11203
P03363	P63131	0.15425
P03363	Q9H201	0.333
P03363	Q12830	0.61081
P03363	Q5RKV6	0.24352
P03363	Q8NFW8	0.18298
P03363	Q8N5U6	0.73489
P03363	Q53GS7	0.57325
P03363	O95049	0.48007
P03363	Q6P0N0	0.24635
P03363	Q9C010	0.0080524
P03363	O75638	0.14619
P03363	Q9BZE2	0.19595
P03363	Q96IY1	0.54882
P03363	Q8IYA6	0.12015
P03363	Q8N2Y8	0.29635
P03363	Q8N5Y2	0.30857
P03363	Q7Z3B4	0.49492
P03363	Q8NHW5	0.37392
P03363	Q99592	0.23685
P03363	Q8N1G0	0.27378
P03363	Q92973	0.8559
P03363	Q9GZV4	0.48289
P03363	P10828	0.45501
P03363	Q9H963	0.22259
P03363	O15371	0.65979
P03363	Q92917	0.86194
P03363	P51965	0.30868
P03363	Q9Y263	0.32134
P03363	Q9UJQ4	0.21989
P03363	P63162	0.79978
P03363	Q9Y4Z0	0.69005
P03363	Q99909	0.39008
P03363	P62750	0.51408
P03363	Q9BQQ7	0.34077
P03363	Q15366	0.77583
P03363	Q969J2	0.268
P03363	P69905	0.19602
P03363	Q9BSH3	0.27292
P03363	Q9NVR5	0.17684
P03363	Q9Y4Z2	0.35277
P03363	Q13427	0.45447
P03363	Q9Y5B6	0.49974
P03363	Q8N988	0.22259
P03363	Q9C0I3	0.055685
P03363	Q8N4M7	0.085802
P03363	Q96RT1	0.35482
P03363	Q4W5G0	0.40039
P03363	Q9NSI8	0.24098
P03363	Q8WWL7	0.71724
P03363	O43736	0.15709
P03363	Q9BVJ6	0.70473
P03363	O00273	0.34077
P03363	Q07666	0.47227
P03363	P27348	0.54339
P03363	O75161	0.51349
P03363	Q96B36	0.69436
P03363	Q9UHF7	0.5491
P03363	P29374	0.39379
P03363	Q6P1K2	0.63531
P03363	Q8WTT2	0.30989

P03363	P23396	0.43504
P03363	Q9UL42	0.76224
P03363	Q9H825	0.41753
P03363	Q8N2I2	0.22259
P03363	P63119	0.19177
P03363	Q9UHL9	0.47756
P03363	Q96LW1	0.22259
P03363	P28698	0.61204
P03363	P02538	0.83203
P03363	Q53ET0	0.63393
P03363	Q8IZC7	0.22259
P03363	O60543	0.25962
P03363	O75368	0.33007
P03363	Q96IZ7	0.87588
P03363	Q92985	0.34525
P03363	Q9H4D5	0.52168
P03363	P42677	0.38878
P03363	Q9UL16	0.21533
P03363	Q8NE65	0.36172
P03363	Q9BUP3	0.29391
P03363	Q9BWF2	0.22842
P03363	Q9UGK8	0.71308
P03363	P19338	0.82045
P03363	A8MUX0	0.2934
P03363	Q8N0X2	0.32132
P03363	Q9H7H0	0.60932
P03363	Q96AQ7	0.42943
P03363	Q96N22	0.22259
P03363	Q5JTC6	0.49174
P03363	Q9NQZ8	0.24904
P03363	P62380	0.36957
P03363	Q96DB5	0.40339
P03363	Q8N5Z5	0.18177
P03363	P62136	0.31734
P03363	Q9Y2W2	0.88492
P03363	O95948	0.45949
P03363	Q9NYP9	0.33467
P03363	P63173	0.29783
P03363	O60809	0.10142
P03363	Q9NXZ1	0.29721
P03363	P49419	0.35926
P03363	Q8NF91	0.44515
P03363	O15287	0.73205
P03363	Q14264	0.16462
P03363	Q96J01	0.3057
P03363	P22531	0.74011
P03363	Q6DD87	0.22259
P03363	Q9GZS3	0.88668
P03363	Q9UHP9	0.31355
P03363	Q9H5J4	0.080996
P03363	Q9NWH9	0.36034
P03363	Q59GR2	0.55275
P03363	P19622	0.49559
P03363	P08865	0.48173
P03363	P23246	0.87297
P03363	Q9H902	0.24603
P03363	Q99623	0.78672
P03363	P29083	0.64123
P03363	Q7Z7K6	0.24083
P03363	P29084	0.9701
P03363	Q13835	0.33898
P03363	Q9H9R9	0.24431
P03363	Q8TAQ5	0.22259
P03363	Q9GZU8	0.22504
P03363	Q96D09	0.23126
P03363	Q96NU1	0.22504
P03363	Q96T58	0.64398
P03363	P27694	0.31801
P03363	Q8WTV1	0.57301
P03363	P07197	0.85189
P03363	P62937	0.42062
P03363	Q86UA1	0.24064
P03363	Q14533	0.8697
P03363	Q92766	0.24775
P03363	Q9Y2T2	0.53121
P03363	P0CL84	0.22504

P03363	P13807	0.35129
P03363	Q03924	0.24904
P03363	Q8WXK3	0.41574
P03363	Q6ZNA1	0.22259
P03363	Q6ZNK6	0.076511
P03363	O43933	0.33777
P03363	Q9UGN5	0.28899
P03363	P60604	0.21521
P03363	O94818	0.29739
P03363	Q6ZYL4	0.4604
P03363	Q96GC5	0.60871
P03363	Q9UPV9	0.65069
P03363	Q9Y2J8	0.13873
P03363	Q9HAW4	0.45401
P03363	O15355	0.62142
P03363	A5A3E0	0.18581
P03363	Q9GZY1	0.4314
P03363	Q9BYR6	0.42225
P03363	Q9UNH7	0.3316
P03363	P47902	0.46042
P03363	P31040	0.25378
P03363	Q9BRU9	0.27944
P03363	P36575	0.26926
P03363	Q96S65	0.50028
P03363	P35711	0.76164
P03363	Q13487	0.50615
P03363	Q14244	0.14365
P03363	Q99962	0.26467
P03363	Q9NZR4	0.27714
P03363	Q9H8E8	0.71705
P03363	Q9P2I0	0.71123
P03363	Q96I24	0.50731
P03363	Q9BXM0	0.6493
P03363	P69208	0.20014
P03363	Q96RJ6	0.41504
P03363	P54198	0.88754
P03363	Q14929	0.24904
P03363	Q8NDY6	0.51016
P03363	A2IDD5	0.23077
P03363	Q9Y5B0	0.2763
P03363	Q9UH92	0.63272
P03363	P78415	0.39253
P03363	Q9Y5K5	0.23815
P03363	O60443	0.1664
P03363	Q7Z333	0.29591
P03363	Q6XYB7	0.51853
P03363	P54652	0.25985
P03363	O60664	0.55695
P03363	O15527	0.26061
P03363	Q6ZVK8	0.37697
P03363	Q7Z7A1	0.8901
P03363	Q6PIY2	0.1102
P03363	P0CJ87	0.49158
P03363	Q9H3S7	0.29355
P03363	Q9NQX6	0.22259
P03363	Q86YD1	0.43951
P03363	A6NC42	0.1532
P03363	Q9NYP3	0.1705
P03363	P0CJ88	0.49158
P03363	P38919	0.41972
P03363	Q15370	0.73225
P03363	Q9BYU1	0.40426
P03363	P09067	0.34931
P03363	P12525	0.49594
P03363	P55771	0.67846
P03363	Q9BZR9	0.31064
P03363	Q7Z340	0.22259
P03363	Q5T8P6	0.24134
P03363	Q14938	0.49812
P03363	B4DU55	0.22259
P03363	Q9H091	0.18835
P03363	P41223	0.27643
P03363	Q8N859	0.22259
P03363	Q92925	0.39552
P03363	A8K8P3	0.75273
P03363	Q16589	0.36692

P03363	Q19AV6	0.42825
P03363	Q9UFG5	0.53508
P03363	Q6P9B6	0.53508
P03363	Q0VDD7	0.44617
P03363	Q9NWZ5	0.23133
P03363	P0DJD3	0.53971
P03363	P11277	0.30126
P03363	Q15466	0.51197
P03363	O75360	0.52419
P03363	P41252	0.62668
P03363	P07900	0.23771
P03363	Q8TF42	0.25571
P03363	Q9Y5J3	0.27463
P03363	O14908	0.23969
P03363	Q96DF8	0.38214
P03363	O14978	0.40676
P03363	Q5HYW2	0.017352
P03363	Q14CS0	0.34779
P03363	Q9Y6X0	0.27018
P03363	Q6ZN06	0.22259
P03363	Q96GA3	0.49651
P03363	Q2TAM9	0.051971
P03363	Q04323	0.44207
P03363	Q9Y6D9	0.51707
P03363	P56715	0.27474
P03363	Q4LE28	0.47281
P03363	Q2M3G4	0.2291
P03363	P63167	0.79408
P03363	P54727	0.5915
P03363	Q86Y79	0.011679
P03363	Q96EU6	0.26859
P03363	P13645	0.9637
P03363	P46087	0.64512
P03363	Q6ZMZ3	0.29611
P03363	P53801	0.16367
P03363	Q7L804	0.41803
P03363	Q86W11	0.268
P03363	Q9ULX3	0.10525
P03363	P62847	0.31092
P03363	Q9Y2X3	0.64893
P03363	Q9HC77	0.65501
P03363	Q9UNW9	0.2354
P03363	Q8NA42	0.40237
P03363	Q14865	0.24905
P03363	Q9UBB6	0.20299
P03363	O75570	0.23242
P03363	P11441	0.66916
P03363	O60645	0.57359
P03363	Q96NS5	0.41574
P03363	P18077	0.29481
P03363	Q13503	0.48177
P03363	O15360	0.78995
P03363	Q8TB45	0.2829
P03363	Q14525	0.89041
P03363	Q9NZL3	0.60385
P03363	P06744	0.14552
P03363	O60684	0.50939
P03363	Q9NQS1	0.28107
P03363	Q9H299	0.33548
P03363	Q15438	0.241
P03363	Q8NCU1	0.0012662
P03363	Q9GZL7	0.56282
P03363	Q6EMB2	0.34773
P03363	Q6P387	0.49651
P03363	P82664	0.23424
P03363	Q9UNH5	0.50918
P03363	P31274	0.38817
P03363	Q14145	0.87769
P03363	Q9Y2D9	0.63436
P03363	Q96ED9	0.6293
P03363	Q8NAP3	0.40863
P03363	P17021	0.22259
P03363	Q9GZM3	0.23378
P03363	Q10570	0.7881
P03363	Q16352	0.80866
P03363	O14920	0.23986

P03363	P17010	0.27223
P03363	Q08J23	0.49967
P03363	Q9H987	0.25697
P03363	P62714	0.52609
P03363	Q15050	0.27594
P03363	A2BFH1	0.21453
P03363	Q8IVP9	0.22259
P03363	Q5SRE5	0.38433
P03363	O43296	0.22259
P03363	Q8NA92	0.13635
P03363	O14529	0.49417
P03363	Q8WWQ0	0.50621
P03363	Q04206	0.26187
P03363	Q15853	0.57955
P03363	O14771	0.268
P03363	Q16222	0.2413
P03363	Q9H4X1	0.27382
P03363	Q9BQ95	0.86211
P03363	Q96CV9	0.53456
P03363	Q8NAV2	0.22504
P03363	P05387	0.36325
P03363	Q99598	0.23952
P03363	P22532	0.22177
P03363	P35237	0.32557
P03363	Q9NRA8	0.98864
P03363	Q9BR09	0.2201
P03363	O15519	0.40803
P03363	Q7Z591	0.43182
P03363	Q86VQ0	0.62215
P03363	O95780	0.22259
P03363	O95996	0.4124
P03363	Q96K80	0.42886
P03363	P51580	0.17582
P03363	O14896	0.52038
P03363	A6NL28	0.25697
P03363	P27540	0.34217
P03363	Q66K74	0.39693
P03363	Q7RTS9	0.67517
P03363	Q86VK4	0.22259
P03363	O60356	0.16774
P03363	P49848	0.65994
P03363	P02795	0.43442
P03363	Q9Y383	0.18172
P03363	Q86WB0	0.58107
P03363	Q96EL2	0.31759
P03363	Q8WYR1	0.20174
P03363	A6NK75	0.22259
P03363	P17676	0.57751
P03363	P12270	0.42053
P03363	P52738	0.24904
P03363	Q9UI47	0.59086
P03363	Q9H081	0.44958
P03363	Q76N32	0.36506
P03363	Q5TA50	0.21406
P03363	Q96GG9	0.49285
P03363	P35321	0.676
P03363	O75683	0.35064
P03363	Q92575	0.71376
P03363	P18669	0.21728
P03363	Q9BXR0	0.25034
P03363	Q5T5M9	0.22504
P03363	O14829	0.27823
P03363	Q8TAM2	0.28176
P03363	O75474	0.13502
P03363	P49459	0.21223
P03363	P10265	0.085848
P03363	P52565	0.31873
P03363	P06454	0.47682
P03363	O75643	0.38419
P03363	Q15468	0.12981
P03363	Q9NUA8	0.19079
P03363	Q96DZ1	0.34088
P03363	P59282	0.24431
P03363	Q6NSZ9	0.268
P03363	Q5C9Z4	0.74235
P03363	Q8IY21	0.29741

P03363	Q8NC51	0.72946
P03363	Q12901	0.24904
P03363	P26447	0.47357
P03363	O60481	0.53466
P03363	Q9NSD9	0.083221
P03363	P18146	0.51399
P03363	Q5JUK2	0.22111
P03363	Q9BYV8	0.60806
P03363	A6NKD9	0.12259
P03363	Q13360	0.27693
P03363	P49116	0.38593
P03363	Q68DC2	0.25197
P03363	Q969Q6	0.43063
P03363	Q0VDE8	0.063335
P03363	Q8TF40	0.70913
P03363	Q9P2Y5	0.44785
P03363	O00712	0.29943
P03363	Q969U7	0.61348
P03363	Q9H981	0.72627
P03363	Q9P2G1	0.29734
P03363	O95628	0.64286
P03363	Q8IVI9	0.34885
P03363	Q8WXX7	0.11203
P03363	Q9ULG1	0.26233
P03363	Q9HCU9	0.91248
P03363	Q8NG50	0.47954
P03363	A2RU30	0.20113
P03363	Q9BX66	0.30095
P03363	P0CJ86	0.49158
P03363	Q96KD3	0.062103
P03363	Q8WXE9	0.5718
P03363	P19388	0.46556
P03363	O95453	0.35973
P03363	O95050	0.22135
P03363	Q3LI81	0.27762
P03363	Q9H9P5	0.41847
P03363	P36873	0.24301
P03363	Q2VIQ3	0.30945
P03363	Q9BY44	0.49154
P03363	Q9BVT8	0.17461
P03363	Q6ECI4	0.27378
P03363	Q9UBX2	0.49158
P03363	Q9UHB4	0.543
P03363	P84098	0.36325
P03363	Q12972	0.65482
P03363	Q9H9C1	0.5428
P03363	O15061	0.88234
P03363	Q96C01	0.23887
P03363	Q7Z624	0.23796
P03363	P42766	0.48764
P03363	Q6P2D0	0.22259
P03363	Q52LA3	0.09678
P03363	O75764	0.31031
P03363	P24864	0.23999
P03363	Q3B820	0.3998
P03363	Q8NEK5	0.22259
P03363	P14649	0.33342
P03363	Q02386	0.14923
P03363	Q16851	0.26964
P03363	P51784	0.37214
P03363	P0C5Y4	0.2934
P03363	O15524	0.23252
P03363	Q9NQ55	0.508
P03363	Q13367	0.383
P03363	Q6ICB4	0.23681
P03363	Q5VV41	0.31906
P03363	Q13148	0.72993
P03363	Q8IXV7	0.24431
P03363	Q8TBZ9	0.083102
P03363	Q16537	0.5639
P03363	Q5VT06	0.60172
P03363	Q96JS3	0.3764
P03363	Q8N5J2	0.074921
P03363	Q8N0V3	0.071949
P03363	P0C7V9	0.062282
P03363	Q3BBV2	0.24431

P03363	Q969W3	0.046013
P03363	Q86UR1	0.27764
P03363	Q96JC4	0.22259
P03363	Q9P2W6	0.24431
P03363	Q8IXQ6	0.78228
P03363	P59797	0.012359
P03363	Q8NHU6	0.42789
P03363	P02549	0.24602
P03363	O60315	0.32795
P03363	Q6P1K8	0.21734
P03363	Q12872	0.44323
P03363	Q92674	0.33525
P03363	Q8TAB7	0.073674
P03363	P83916	0.53709
P03363	Q13435	0.54284
P03363	P33316	0.39249
P03363	Q9HBF5	0.032026
P03363	Q8NFH4	0.47201
P03363	Q8WZ33	0.71377
P03363	O00221	0.27067
P03363	O95411	0.29603
P03363	Q6STE5	0.42958
P03363	Q02930	0.68052
P03363	Q9UHB7	0.94778
P03363	Q5PRF9	0.61357
P03363	Q9BXW9	0.66555
P03363	Q75VX8	0.08127
P03363	O95391	0.35765
P03363	O60224	0.39008
P03363	Q9P0V3	0.57295
P03363	Q00597	0.7147
P03363	Q8NG57	0.19772
P03363	Q99576	0.62084
P03363	C9JR72	0.24431
P03363	Q969X6	0.93113
P03363	Q13428	0.83316
P03363	O14682	0.35489
P03363	Q8TAF3	0.73053
P03363	Q9UND3	0.34378
P03363	Q9BSV6	0.28865
P03363	A8MTY7	0.2934
P03363	Q9NZE8	0.19909
P03363	P98169	0.19314
P03363	Q9UMS0	0.82176
P03363	O75391	0.35279
P03363	P13984	0.47875
P03363	O43310	0.68276
P03363	P02545	0.58136
P03363	Q7Z3Z4	0.24553
P03363	A2RTX5	0.12473
P03363	Q96M43	0.24431
P03363	Q8N9A8	0.47588
P03363	Q7Z4U5	0.024605
P03363	O00762	0.29463
P03363	Q9P2J8	0.22259
P03363	P60709	0.26985
P03363	Q9NZJ4	0.18309
P03363	Q15021	0.65628
P03363	P62249	0.27497
P03363	Q86UP3	0.30694
P03363	Q9NXG0	0.17396
P03363	P49321	0.36663
P03363	Q9UJV8	0.27018
P03363	Q9Y468	0.47785
P03363	Q9H175	0.94514
P03363	Q6NT04	0.40039
P03363	Q9UP83	0.94879
P03363	P35227	0.5687
P03363	Q5K131	0.24431
P03363	O95400	0.77743
P03363	P52597	0.86404
P03363	Q9BYD1	0.70406
P03363	Q5U5Q3	0.23279
P03363	Q9C000	0.31178
P03363	Q7Z353	0.55275
P03363	O60563	0.85171

P03363	Q9HAU4	0.31773
P03363	Q96T92	0.40237
P03363	Q9NXX6	0.48695
P03363	Q92543	0.29104
P03363	Q9H321	0.29721
P03363	Q02083	0.17092
P03363	P0CG31	0.22259
P03363	Q99676	0.22259
P03363	Q3ZCT1	0.33748
P03363	P21127	0.25179
P03363	P36543	0.25223
P03363	Q92900	0.32966
P03363	Q6V9R5	0.22259
P03363	P60411	0.2934
P03363	Q9BTK6	0.61218
P03363	P82921	0.20437
P03363	Q96HR3	0.45517
P03363	Q9NW13	0.26123
P03363	O95229	0.53252
P03363	Q9H9Q2	0.4189
P03363	Q9Y4W2	0.39268
P03363	O60291	0.35106
P03363	P48539	0.267
P03363	Q99626	0.27069
P03363	Q6GQQ9	0.23718
P03363	Q9H8M2	0.32716
P03363	Q14582	0.45529
P03363	Q9NRL2	0.57361
P03363	Q13227	0.63385
P03363	P53567	0.54321
P03363	P48382	0.95837
P03363	Q8N5D0	0.50445
P03363	P50502	0.57965
P03363	Q9C0A1	0.30694
P03363	Q96DE5	0.57272
P03363	Q14526	0.65038
P03363	P05388	0.76644
P03363	A6NHJ4	0.22259
P03363	P68871	0.13772
P03363	Q9BYR7	0.42225
P03363	Q8IZP2	0.24431
P03363	Q9UMS5	0.34258
P03363	Q9BR46	0.030925
P03363	Q9UPY6	0.23868
P03363	P35080	0.5487
P03363	Q8N7R0	0.51853
P03363	O43747	0.45256
P03363	P33176	0.49905
P03363	Q58F21	0.24784
P03363	O60535	0.58527
P03363	C9J3V5	0.19361
P03363	Q9HAV4	0.90036
P03363	Q9Y4R8	0.45672
P03363	O60841	0.35355
P03363	Q9BZF3	0.27268
P03363	Q9UN79	0.92764
P03363	Q9H9Y6	0.46246
P03363	P58004	0.40313
P03363	P0CG35	0.26541
P03363	P78559	0.825
P03363	Q9Y4H4	0.18763
P03363	Q9BRK4	0.50364
P03363	Q9Y2R2	0.28396
P03363	Q96MX3	0.22259
P03363	A8MXZ3	0.2934
P03363	Q9NZU5	0.26504
P03363	Q9BYV9	0.52046
P03363	Q9H7U1	0.037873
P03363	P16157	0.31254
P03363	Q14194	0.44229
P03363	Q9HB71	0.61939
P03363	Q4G0A6	0.0456
P03363	Q92764	0.78878
P03363	P60896	0.24624
P03363	Q9UJY4	0.38848
P03363	P35579	0.26808

P03363	Q96MC9	0.045357
P03363	Q8IU81	0.27476
P03363	P61970	0.58902
P03363	P14373	0.38494
P03363	Q96FC7	0.23887
P03363	Q13516	0.33206
P03363	Q9UHI6	0.56335
P03363	Q15554	0.36669
P03363	Q9UJ04	0.15989
P03363	Q6NZY4	0.70291
P03363	Q96IU4	0.45816
P03363	O75340	0.36952
P03363	Q14781	0.71371
P03363	Q9NZL4	0.4488
P03363	Q9NY87	0.44815
P03363	Q9H707	0.22259
P03363	Q99996	0.53613
P03363	P56177	0.49327
P03363	Q7L4I2	0.15034
P03363	Q9H094	0.24431
P03363	Q2KHR2	0.46264
P03363	Q3C1V8	0.73672
P03363	Q96NG8	0.22259
P03363	Q14247	0.49424
P03363	Q9NVV9	0.76418
P03363	Q9UMX1	0.37268
P03363	P31150	0.33702
P03363	P36402	0.47615
P03363	P36578	0.61773
P03363	O75607	0.78051
P03363	O14786	0.088598
P03363	Q86YA3	0.030796
P03363	Q5TID7	0.29721
P03363	Q9UC06	0.24904
P03363	P78424	0.47247
P03363	Q96SE7	0.22259
P03363	Q9Y3C5	0.4394
P03363	P60412	0.2934
P03363	P09012	0.73095
P03363	Q9ULR5	0.67568
P03363	Q9UPZ3	0.24973
P03363	O94811	0.60715
P03363	Q96HW7	0.66095
P03363	A2A288	0.25151
P03363	P12524	0.49594
P03363	Q96IZ0	0.65086
P03363	P48444	0.34934
P03363	Q71SY5	0.80211
P03363	Q96T49	0.32285
P03363	Q330K2	0.26859
P03363	Q86YP4	0.51356
P03363	A6NJB7	0.010954
P03363	P49406	0.39181
P03363	O75886	0.82862
P03363	Q9BZQ8	0.25578
P03363	Q63HQ0	0.2528
P03363	P0CB48	0.27018
P03363	Q9ULP0	0.20401
P03363	Q6ZS46	0.016696
P03363	P54259	0.76921
P03363	Q96LW7	0.25057
P03363	Q9BRR0	0.27601
P03363	Q9Y375	0.61766
P03363	Q13347	0.68795
P03363	Q4VC05	0.091333
P03363	Q8IUE1	0.51853
P03363	P78412	0.55275
P03363	Q99543	0.42213
P03363	P0DI81	0.81958
P03363	P08151	0.28425
P03363	Q7L3S4	0.22259
P03363	Q8N448	0.38768
P03363	Q8NEZ5	0.52123
P03363	Q3LI68	0.27762
P03363	Q9H5I1	0.39947
P03363	Q3SY46	0.27762

P03363	P0CB47	0.33983
P03363	Q8N5M1	0.52667
P03363	P30154	0.51162
P03363	Q9H7D7	0.49651
P03363	Q99704	0.34472
P03363	P55199	0.70266
P03363	Q9H7S9	0.53871
P03363	Q96K62	0.22259
P03363	P86452	0.49815
P03363	P99999	0.35094
P03363	Q96L92	0.68992
P03363	Q96MT8	0.75421
P03363	Q9BY41	0.28015
P03363	Q5T7V8	0.9515
P03363	Q9HCM1	0.031545
P03363	Q9NS39	0.22949
P03363	Q96BD8	0.47453
P03363	P43378	0.30377
P03363	Q9BTE3	0.45945
P03363	P11387	0.5085
P03363	P53539	0.44039
P03363	Q99742	0.30683
P03363	Q9NYK6	0.026447
P03363	P07316	0.30454
P03363	O95059	0.23233
P03363	Q9NVQ4	0.29642
P03363	Q96JF6	0.22259
P03363	Q9UKV3	0.64173
P03363	Q14590	0.22259
P03363	Q96MU7	0.77943
P03363	P60673	0.42339
P03363	Q969E4	0.40525
P03363	Q9Y2V2	0.51161
P03363	Q9H5F2	0.24993
P03363	Q9H4L7	0.35467
P03363	O95561	0.018091
P03363	P62753	0.59175
P03363	Q7Z2Z1	0.59808
P03363	O15078	0.48406
P03363	Q9UHV9	0.74496
P03363	Q92539	0.24986
P03363	Q7L190	0.74576
P03363	Q99848	0.23603
P03363	Q9H492	0.40967
P03363	Q9H693	0.070131
P03363	O94992	0.93348
P03363	P22314	0.26719
P03363	Q9UJT9	0.37794
P03363	Q86UE3	0.22259
P03363	Q9BRU2	0.40004
P03363	Q8N1B4	0.4726
P03363	O43924	0.35572
P03363	P62312	0.80462
P03363	Q8WX92	0.97697
P03363	Q96BK5	0.49669
P03363	P17098	0.27166
P03363	P62140	0.36618
P03363	Q969T7	0.25497
P03363	Q9BTP7	0.8364
P03363	Q6IA86	0.5709
P03363	P24863	0.79914
P03363	Q69YI7	0.2575
P03363	Q9GZP8	0.17372
P03363	Q92530	0.12129
P03363	Q96DG6	0.27319
P03363	Q9H869	1.1148
P03363	Q08AN1	0.22259
P03363	P33552	0.094086
P03363	Q5TBB1	0.26532
P03363	Q14397	0.44287
P03363	Q8TBZ0	0.3089
P03363	P31267	0.38817
P03363	Q86V48	0.18393
P03363	Q3LI76	0.27762
P03363	Q9BYE4	0.22177
P03363	Q9UMY1	0.28674

P03363	Q15928	0.26735
P03363	Q9UHP3	0.37382
P03363	Q9NWW0	0.4314
P03363	Q8IYK2	0.12321
P03363	Q8ND04	0.53671
P03363	Q14863	0.38392
P03363	Q8WV37	0.42268
P03363	Q96JZ2	0.37135
P03363	Q02952	0.34899
P03363	Q7Z398	0.22259
P03363	O43365	0.37669
P03363	Q68EA5	0.22259
P03363	Q96AB6	0.32272
P03363	Q9H765	0.44922
P03363	Q8NHV4	0.53181
P03363	Q86WV1	0.53054
P03363	O15015	0.63436
P03363	Q9UKN5	0.3036
P03363	Q96SR6	0.20871
P03363	Q96EP0	0.25625
P03363	P62633	0.42539
P03363	Q9ULM2	0.22259
P03363	P61201	0.66158
P03363	Q02447	0.27682
P03363	Q9GZS0	0.27211
P03363	P08651	0.54762
P03363	P62081	0.64475
P03363	Q96ND8	0.22259
P03363	O60268	0.24431
P03363	O43805	0.54347
P03363	Q08170	0.38525
P03363	P40925	0.14545
P03363	A6NKH3	0.11974
P03363	Q8N3J9	0.21805
P03363	Q8N187	0.57185
P03363	Q9NNW7	0.2431
P03363	Q969Q5	0.30191
P03363	Q96GS4	0.095381
P03363	Q9ULJ3	0.58154
P03363	P62910	0.38002
P03363	P50452	0.64767
P03363	Q8TAY7	0.16215
P03363	Q96LC9	0.48067
P03363	Q15022	0.46526
P03363	Q86Y13	0.48105
P03363	Q13362	0.28025
P03363	Q06546	1.0523
P03363	O60308	0.34026
P03363	O75794	0.30998
P03363	Q9NXH3	0.19151
P03363	Q8NBT0	0.28241
P03363	Q15562	0.65812
P03363	Q9Y324	0.27944
P03363	Q63ZY3	0.76114
P03363	Q9BSH4	0.038259
P03363	A8MTQ0	0.32961
P03363	Q13952	0.61973
P03363	Q8N1G4	0.5108
P03363	O60306	0.30211
P03363	Q9BYR3	0.2934
P03363	Q9Y3D0	0.90135
P03363	Q6P2D8	0.43184
P03363	Q92990	0.24506
P03363	Q9Y3R5	0.07289
P03363	P50539	0.44312
P03363	O95232	0.61235
P03363	Q6PG37	0.22259
P03363	Q9Y2K5	0.35279
P03363	Q9BV90	0.33749
P03363	P58340	0.62882
P03363	Q96I59	0.17374
P03363	Q1MSJ5	0.36327
P03363	O75618	0.91266
P03363	Q96MT3	0.52947
P03363	Q9BYQ6	0.2934
P03363	P30876	0.36417

P03363	Q8TBZ2	0.30201
P03363	Q0VG99	0.39445
P03363	P30279	0.39242
P03363	Q3LI77	0.27762
P03363	Q7RTS7	0.42225
P03363	Q8IYN6	0.0041725
P03363	Q13228	0.98041
P03363	Q96PS1	0.0029525
P03363	Q5T6F2	0.023357
P03363	O76014	0.88908
P03363	A9QM74	0.55798
P03363	Q69YU3	0.24431
P03363	O75112	0.38475
P03363	Q8IXM3	0.33508
P03363	Q93075	0.12345
P03363	A9UHW6	0.86014
P03363	O14933	0.39987
P03363	Q96LA8	0.44816
P03363	P0CG30	0.42083
P03363	Q9Y2M0	0.31143
P03363	Q99575	0.61005
P03363	O43167	0.63436
P03363	Q969V4	0.26324
P03363	Q6ZR52	0.22259
P03363	Q9BW85	0.093894
P03363	Q14331	0.38135
P03363	Q09028	0.43536
P03363	P13010	0.2494
P03363	Q02556	0.39174
P03363	Q5VY09	0.0055957
P03363	Q92831	0.35003
P03363	Q03933	0.68803
P03363	Q8WYP5	0.47412
P03363	Q8WVC0	0.73638
P03363	Q3ZLR7	0.029024
P03363	Q86WQ0	0.59613
P03363	Q9H444	0.67311
P03363	Q96EK4	1.0421
P03363	P14868	0.51439
P03363	Q96RL1	0.56654
P03363	P25963	0.30951
P03363	Q8IX01	0.4444
P03363	O60422	0.48374
P03363	Q9HAN9	0.27236
P03363	Q9BUH8	0.63704
P03363	Q9UKW6	0.64663
P03363	P41236	0.40351
P03363	P51991	0.41079
P03363	Q9NQW6	0.3517
P03363	Q96LI6	0.69875
P03363	Q15291	0.27952
P03363	Q9UD71	0.10934
P03363	P12757	0.40275
P03363	Q9HCS7	0.6116
P03363	O95478	0.3856
P03363	P62508	0.35502
P03363	Q6ZU65	0.29721
P03363	O60232	0.59071
P03363	Q9BR76	0.60315
P03363	Q14457	0.40216
P03363	P14651	0.37196
P03363	Q15744	0.36578
P03363	Q5EBL2	0.24904
P03363	Q9Y2W6	0.25348
P03363	Q01831	0.62845
P03363	Q66GS9	0.40799
P03363	P20248	0.68905
P03363	Q9H9Y2	0.2976
P03363	Q7Z569	0.23048
P03363	O75170	0.68162
P03363	Q96GY3	0.55814
P03363	O75817	0.69177
P03363	P0CG00	0.23235
P03363	Q9HC52	0.65861
P03363	Q8NEM7	0.50162
P03363	Q13561	0.59303

P03363	Q96NJ6	0.23615
P03363	P62328	0.4696
P03363	Q86VF2	0.39326
P03363	Q8WW12	0.71797
P03363	P25205	0.31248
P03363	Q03112	0.21692
P03363	P61081	0.21863
P03363	Q13888	0.29944
P03363	P60369	0.2934
P03363	Q9UEG4	0.22259
P03363	O60663	0.52574
P03363	Q8N998	0.4314
P03363	Q9NS37	0.81551
P03363	Q6UX04	0.23513
P03363	O60437	0.73932
P03363	O00213	0.24302
P03363	Q14657	0.11203
P03363	Q86X83	0.53508
P03363	Q7L1V2	0.53508
P03363	O95568	0.23591
P03363	O95219	0.27247
P03363	Q9H706	0.53508
P03363	P09914	0.39272
P03363	Q14258	0.71712
P03363	Q96QC0	0.12226
P03363	Q8N0U4	0.24431
P03363	Q9HAH1	0.22259
P03363	Q9BXL5	0.53594
P03363	Q6UWZ7	0.51381
P03363	Q6IPT2	0.062103
P03363	Q92734	0.48973
P03363	Q96RY5	0.39755
P03363	P53672	0.084102
P03363	Q9H7L9	0.54776
P03363	Q8IYI6	0.58947
P03363	Q9UQF2	0.36837
P03363	P41218	0.5617
P03363	Q8N7U7	0.55275
P03363	Q92783	0.54832
P03363	Q9Y606	0.31774
P03363	Q15014	0.8952
P03363	O43347	0.28766
P03363	Q9BWW7	0.24904
P03363	Q8WWK9	0.40815
P03363	Q8NCE8	0.042292
P03363	O95347	0.4209
P03363	P56279	0.55437
P03363	P02728	0.11203
P03363	Q86Y46	0.48919
P03363	Q75QN2	0.66095
P03363	A6NHS1	0.031371
P03363	Q9BQC3	0.22979
P03363	Q6NX49	0.22259
P03363	P18847	0.92731
P03363	Q8NFH3	0.47201
P03363	P45973	0.50869
P03363	Q13794	0.2216
P03363	Q99929	0.34906
P03363	P14866	0.87459
P03363	Q05048	0.7758
P03363	Q9Y2P0	0.22259
P03363	Q3LI62	0.27762
P03363	Q96HY6	0.40763
P03363	P61513	0.29453
P03363	P49589	0.35416
P03363	P46776	0.61631
P03363	Q9UKX2	0.2721
P03363	O43303	0.76562
P03363	Q96BW1	0.4314
P03363	Q9HD15	0.5654
P03363	Q9P2N7	0.20346
P03363	Q15170	0.49089
P03363	Q86XI2	0.35534
P03363	Q5U623	0.66899
P03363	O75346	0.20871
P03363	Q9GZL8	0.11203

P03363	Q9BYT5	0.2934
P03363	P13640	0.50003
P03363	Q5VZK9	0.32262
P03363	O60281	0.33141
P03363	Q13356	0.15851
P03363	Q15024	0.60038
P03363	Q9HBT8	0.22259
P03363	Q10586	0.51727
P03363	Q15393	0.42415
P03363	P23610	0.24993
P03363	Q9UPP2	0.30119
P03363	Q9NRF9	0.39837
P03363	Q6ZV50	0.43182
P03363	O75132	0.50836
P03363	O43763	0.47248
P03363	Q9UPX8	0.39325
P03363	Q92879	0.52939
P03363	P60372	0.2934
P03363	Q6UWF3	0.082721
P03363	Q13405	0.60871
P03363	Q14980	0.69467
P03363	Q13111	0.72587
P03363	Q9NVU7	0.41814
P03363	P37275	0.67919
P03363	Q9Y266	0.36647
P03363	Q96NG3	0.24431
P03363	O75604	0.26611
P03363	A5YKK6	0.81423
P03363	Q9GZQ3	0.68145
P03363	Q06330	0.34904
P03363	Q8WVE0	0.19118
P03363	Q96B97	0.41687
P03363	P32780	0.48357
P03363	Q969W8	0.22259
P03363	Q99547	0.71778
P03363	Q9BRX5	0.19116
P03363	Q96AB3	0.69294
P03363	O14980	0.58115
P03363	Q8TDD2	0.42044
P03363	Q86US8	0.34808
P03363	Q8TAQ2	0.8015
P03363	P20700	0.38444
P03363	Q8IYF1	0.25394
P03363	P37802	0.42506
P03363	P0CJ89	0.49158
P03363	Q16531	0.70295
P03363	Q7L9B9	0.16157
P03363	Q3KNV8	0.58621
P03363	Q8N1V2	0.68162
P03363	Q96GM8	0.24981
P03363	Q8WW35	0.53508
P03363	P56182	0.24032
P03363	O00471	0.38182
P03363	Q9Y5R4	0.17651
P03363	Q8NDW4	0.22259
P03363	Q5TKA1	0.32123
P03363	P63313	0.26541
P03363	O76003	0.34877
P03363	Q8IWA0	0.29721
P03363	Q6ZRH4	0.21212
P03363	Q969T9	0.53819
P03363	Q8NFW9	0.24294
P03363	Q9NWZ8	0.8766
P03363	P09913	0.45875
P03363	Q9Y4A8	0.58318
P03363	Q92759	0.45965
P03363	Q9P2K1	0.12567
P03363	P60368	0.2934
P03363	Q9UI10	0.33727
P03363	P35637	0.58619
P03363	P28356	0.36577
P03363	Q12769	0.58428
P03363	O00410	0.9085
P03363	Q9BYD3	0.33395
P03363	O60303	0.15233
P03363	O75533	0.47189

P03363	Q6MZP7	0.48905
P03363	Q8WV24	0.5738
P03363	P23760	0.82864
P03363	P19387	0.42829
P03363	Q13283	0.35547
P03363	Q2LD37	0.32448
P03363	Q6ZRS2	0.31456
P03363	Q9BU19	0.22259
P03363	Q00005	0.51561
P03363	Q9NQ87	0.40245
P03363	Q96QK1	0.65432
P03363	Q96MR7	0.19561
P03363	Q13439	0.63397
P03363	Q9ULT8	0.40895
P03363	P42167	0.38589
P03363	O15111	0.26614
P03363	Q9Y3B2	0.67734
P03363	Q96DX8	0.34077
P03363	P09086	0.46646
P03363	P41250	0.1205
P03363	Q9H2P0	0.23965
P03363	Q96A65	0.21524
P03363	Q9NX65	0.22259
P03363	P82914	0.23236
P03363	P17947	0.49636
P03363	Q7RTV3	0.46321
P03363	Q8N720	0.77042
P03363	Q64ET8	0.22504
P03363	O15379	0.33766
P03363	Q9H2F5	0.35654
P03363	P23490	0.81536
P03363	Q9Y678	0.44128
P03363	Q9H0G5	0.74233
P03363	Q01892	0.60294
P03363	P78413	0.47984
P03363	Q9NZW5	0.25208
P03363	Q9GZR2	0.36039
P03363	O15304	0.53435
P03363	Q96BP3	0.31536
P03363	O95600	0.22259
P03363	Q9BXW4	0.49972
P03363	Q99750	0.5628
P03363	O94888	0.51791
P03363	Q96LX8	0.22259
P03363	P53677	0.25223
P03363	O14910	0.51012
P03363	Q86Z20	0.23247
P03363	Q9H2T7	0.49712
P03363	Q7L592	0.065964
P03363	Q9UBI9	0.053627
P03363	Q9UBF9	0.30651
P03363	P50135	0.23356
P03363	Q69383	0.55152
P03363	P61254	0.30144
P03363	Q6NXT1	0.3971
P03363	Q9P0J6	0.19909
P03363	Q9NVU0	0.20632
P03363	P62273	0.27332
P03363	Q9NR46	0.62994
P03363	Q6UW56	0.27163
P03363	Q6P2E9	0.81787
P03363	Q13618	0.31323
P03363	Q9HCH0	0.078419
P03363	Q9UHC1	0.41095
P03363	O00505	0.75292
P03363	Q9H9G7	0.78857
P03363	Q8IWF6	0.18807
P03363	Q9UHB9	0.31806
P03363	Q15691	0.46332
P03363	O60784	0.52557
P03363	P51532	0.28719
P03363	Q8N9V7	0.18084
P03363	Q99856	0.71906
P03363	Q15388	0.17197
P03363	Q96AH0	0.34766
P03363	Q9P2M1	0.7338

P03363	P61218	0.21827
P03363	Q16514	0.578
P03363	Q6PF06	0.076158
P03363	Q3SY84	0.67214
P03363	Q9BV86	0.37626
P03363	P17024	0.22259
P03363	Q96JP2	0.34823
P03363	Q15929	0.22259
P03363	P20719	0.21786
P03363	O95155	0.29362
P03363	P09651	0.44564
P03363	P59991	0.2934
P03363	Q9NX01	0.37014
P03363	Q6ZQV5	0.22259
P03363	P32969	0.49565
P03363	Q9H7C9	0.30037
P03363	O15049	0.12181
P03363	Q9NRN9	0.19118
P03363	Q7RTS3	0.23351
P03363	Q8IWP9	0.12329
P03363	P78537	0.50712
P03363	Q96MY1	0.032717
P03363	Q9BWG4	0.27432
P03363	Q01082	0.40697
P03363	O14925	0.2129
P03363	P28347	0.84678
P03363	Q14738	0.78523
P03363	O95684	0.67602
P03363	Q92901	0.36325
P03363	Q8TE02	1.0341
P03363	O95257	0.44497
P03363	Q56P03	0.40055
P03363	Q9NYJ8	0.23982
P03363	P31271	0.24737
P03363	P08579	0.67949
P03363	Q0D2K3	0.22914
P03363	P06733	0.55389
P03363	Q9NZ63	0.49651
P03363	Q9H0N5	0.40544
P03363	Q6P582	0.36547
P03363	P62736	0.16255
P03363	Q7Z2W4	0.33493
P03363	Q9P243	0.38776
P03363	Q494X3	0.22259
P03363	Q15459	0.73864
P03363	Q8IZL8	0.8314
P03363	Q14093	0.13285
P03363	P82970	0.43823
P03363	Q9H1D9	0.46685
P03363	Q3LI83	0.42225
P03363	Q8TE68	0.29734
P03363	Q5T8I9	0.2648
P03363	Q14160	0.33772
P03363	Q8N5N7	0.22496
P03363	O75909	0.77864
P03363	Q9UJW0	0.557
P03363	P78352	0.31602
P03363	Q9BQN1	0.0077299
P03363	Q7Z6E9	0.62903
P03363	Q9NZ52	0.38779
P03363	P59022	0.11203
P03363	Q9BQG0	0.48602
P03363	Q3SXZ3	0.22259
P03363	Q969S8	0.55005
P03363	Q9H8M7	0.71136
P03363	Q9BZK7	0.62021
P03363	Q8N1G2	0.56339
P03363	Q9NYB0	0.48993
P03363	Q9HBH7	0.36878
P03363	Q8IY67	0.33526
P03363	Q9NX02	0.36095
P03363	Q8NHW3	0.37255
P03363	Q9NX70	0.86967
P03363	Q9Y253	0.268
P03363	Q12933	0.49134
P03363	Q9GZT8	0.67801

P03363	Q96BP2	0.96284
P03363	Q9ULW0	0.6575
P03363	Q86SE9	0.6351
P03363	O43439	0.6948
P03363	O75818	0.27957
P03363	Q96BU1	0.15936
P03363	Q9NUU7	0.34231
P03363	Q8N8B7	0.23135
P03363	P04198	0.66142
P03363	Q8IYD1	0.3868
P03363	P46778	0.36325
P03363	Q96MR9	0.22259
P03363	P61583	0.22504
P03363	O94925	0.34759
P03363	Q15056	0.50955
P03363	P18850	0.48214
P03363	Q9H2W2	0.2434
P03363	Q9H6Z9	0.28584
P03363	O95793	0.46241
P03363	P51946	0.30671
P03363	Q14847	0.27755
P03363	Q9ULD0	0.21264
P03363	Q9H7T9	0.80817
P03363	Q8NCP5	0.22259
P03363	Q9H7E2	0.49575
P03363	Q70Z53	0.71827
P03363	Q13045	0.95508
P03363	Q9BXL8	0.22504
P03363	Q6IPX3	0.40525
P03363	Q5HYK9	0.22259
P03363	Q92597	0.50818
P03363	Q5T1R4	0.30864
P03363	Q8N9U0	0.22504
P03363	Q9Y3C6	0.16819
P03363	Q9BYG8	0.26118
P03363	Q9UL59	0.22259
P03363	P25490	0.25603
P03363	Q9Y5Q9	0.2357
P03363	Q8IUB9	0.27762
P03363	Q9NVS2	0.20437
P03363	Q6EKJ0	0.34087
P03363	O43264	0.32891
P03363	Q3SYF9	0.27762
P03363	Q01826	0.62592
P03363	Q99733	0.81107
P03363	Q9NY12	0.25742
P03363	Q12988	0.48126
P03363	A6NDD8	0.24431
P03363	P53805	0.74837
P03363	Q8IYH5	0.31869
P03363	Q8TCI5	0.60829
P03363	Q9NUL3	0.69484
P03363	Q9UBC9	0.72501
P03363	O95201	0.63436
P03363	Q7Z739	0.53508
P03363	O14558	0.82731
P03363	Q9Y3D5	0.20437
P03363	Q3LI70	0.27762
P03363	Q8TES7	0.71462
P03363	Q96HZ4	0.33571
P03363	Q9UKI9	1.1092
P03363	Q6PB30	0.043362
P03363	Q01469	0.50439
P03363	Q5T4T6	0.29721
P03363	Q99550	0.34285
P03363	Q9H1X1	0.12748
P03363	P61927	0.30094
P03363	O95397	0.21406
P03363	Q9P215	0.33341
P03363	Q8NHQ1	1.037
P03363	O75436	0.60086
P03363	O60765	0.25814
P03363	O00463	0.36759
P03363	O00399	0.32273
P03363	Q8NBT2	0.47259
P03363	Q13625	0.49183

P03363	Q5T5D7	0.22259
P03363	Q2YD98	0.37765
P03363	Q96BT7	0.2333
P03363	O75526	0.29576
P03363	O14602	0.53394
P03363	Q7Z4V5	0.24232
P03363	O95922	0.2015
P03363	P82930	0.22496
P03363	P17481	0.26245
P03363	Q16384	0.4106
P03363	Q9HC35	0.36243
P03363	Q9Y6X8	0.58757
P03363	Q8NDQ6	0.40237
P03363	Q9BUX1	0.61271
P03363	P50479	0.29734
P03363	P09972	0.35995
P03363	Q5SXJ2	0.24431
P03363	Q9HCL3	0.22259
P03363	Q7RTV0	0.48831
P03363	P53041	0.43173
P03363	Q9UBL3	0.41386
P03363	Q14154	0.49395
P03363	P78358	0.7338
P03363	P50570	0.2511
P03363	Q8N140	0.78308
P03363	Q9UBQ5	0.81189
P03363	Q5VUG0	0.43003
P03363	O14497	0.39214
P03363	P31946	0.36331
P03363	Q9Y2Q9	0.23415
P03363	Q52LR7	0.37752
P03363	Q9BWD1	0.24074
P03363	O75143	0.61596
P03363	O75376	0.30261
P03363	O60447	0.4447
P03363	O60671	0.36482
P03363	Q96GN5	0.67923
P03363	Q8TCN5	0.22259
P03363	Q5JTD0	0.78877
P03363	Q6BDS2	0.47569
P03363	Q9P2D0	0.31387
P03363	O95613	0.59348
P03363	Q9H0C5	0.75422
P03363	Q9BRR8	0.23307
P03363	O60907	0.50996
P03363	Q8NHR9	0.2591
P03363	Q9UJC5	0.21789
P03363	P29558	0.55892
P03363	P57723	0.41994
P03363	Q9H6U6	0.88506
P03363	Q86VQ1	0.24431
P03363	Q9UPR3	0.81883
P03363	Q8N883	0.22259
P03363	Q8N9N5	0.23982
P03363	Q16520	0.95025
P03363	Q53EQ6	0.4261
P03363	Q9NTK5	0.50737
P03363	O00425	0.64297
P03363	Q9UN36	0.48931
P03363	Q16629	0.55727
P03363	Q01543	0.47995
P03363	Q99456	1.0007
P03363	Q9HCK5	0.78857
P03363	Q7Z589	0.42212
P03363	P62891	0.36325
P03363	P42704	0.86102
P03363	Q6PJG2	0.6171
P03363	Q15398	0.84392
P03363	Q9Y2P8	0.26821
P03363	Q8IUC3	0.27762
P03363	O94986	0.94906
P03363	Q9Y462	0.59365
P03363	E7ETH6	0.20893
P03363	Q9HCD5	0.89393
P03363	Q15910	0.3586
P03363	P15621	0.40375

P03363	Q8NBB4	0.268
P03363	Q6P9A1	0.22259
P03363	P68363	0.36174
P03363	Q7Z6K1	0.2902
P03363	Q15545	0.67254
P03363	Q9UMX0	0.64738
P03363	O60902	0.30469
P03363	Q9BPX5	0.24407
P03363	Q9Y3A5	0.60694
P03363	Q9NTX7	0.52266
P03363	Q6NUQ1	0.52415
P03363	P49354	0.38697
P03363	A6NF01	0.39059
P03363	Q96FI4	0.17657
P03363	Q96P70	0.94159
P03363	O94967	0.23182
P03363	P17030	0.22259
P03363	Q16667	0.25565
P03363	Q6NT89	0.25225
P03363	Q92889	0.24903
P03363	B9ZVM9	0.091375
P03363	Q9UC07	0.24904
P03363	Q8ND76	0.42973
P03363	Q99607	0.92871
P03363	Q9UBS3	0.54164
P03363	Q96I25	0.67233
P03363	Q06190	0.26673
P03363	Q8N8C0	0.22259
P03363	Q9C0J9	0.23878
P03363	O95905	0.65638
P03363	Q13568	0.84885
P03363	Q9UL33	0.13632
P03363	Q13769	0.64494
P03363	Q9H3M7	0.59742
P03363	Q14204	0.35428
P03363	Q9BZE4	0.38529
P03363	P35659	0.28873
P03363	P31949	0.50315
P03363	Q14CM0	0.31823
P03363	Q9ULW8	0.34389
P03363	Q9Y657	0.15856
P03363	Q08AG5	0.22259
P03363	Q9Y3Y2	0.42825
P03363	P59990	0.2934
P03363	Q92791	0.23624
P03363	P52815	0.49458
P03363	Q08752	0.53062
P03363	O00567	0.75553
P03363	P62318	0.76996
P03363	P78317	0.33503
P03363	Q9NPC3	0.41199
P03363	O75884	0.3872
P03363	P60409	0.2934
P03363	P10768	0.27408
P03363	Q0D2J5	0.22259
P03363	Q15776	0.46864
P03363	Q9Y294	0.77731
P03363	Q16254	0.66991
P03363	Q9UBF1	0.84949
P03363	P06132	0.14895
P03363	Q8NEH6	0.46401
P03363	P54920	0.38445
P03363	Q969G9	0.25449
P03363	P11474	0.3064
P03363	P56545	0.26188
P03363	P16220	0.42384
P03363	Q10571	0.098472
P03363	A6NGD5	0.23235
P03363	B2CW77	0.27794
P03363	Q9Y2L8	0.268
P03363	Q96HQ0	0.22259
P03363	Q9H1E3	0.22504
P03363	Q8WYA6	0.72599
P03363	Q9H9E3	0.46061
P03363	Q8WV28	0.24808
P03363	Q9H6Q3	0.30494

P03363	Q9UHK0	0.35461
P03363	O95900	0.10262
P03363	P35251	0.24228
P03363	Q96MU6	0.22259
P03363	Q9Y3C1	0.26346
P03363	Q9Y2D1	1.0697
P03363	Q86WX3	0.99156
P03363	Q9BTM9	0.6843
P03363	P78414	0.55275
P03363	Q9Y295	0.41947
P03363	Q68DI1	0.22259
P03363	P23025	0.29454
P03363	Q9Y605	0.9281
P03363	Q9BQD3	0.54341
P03363	P42768	0.44853
P03363	Q5JVG2	0.22259
P03363	Q5SXM1	0.22259
P03363	Q8N814	0.017063
P03363	Q8N196	0.86815
P03363	Q86WV5	0.48083
P03363	O96007	0.26203
P03363	Q9NPG3	0.75431
P03363	Q8N8D1	0.25562
P03363	Q8NHZ8	0.71803
P03363	Q9NVK5	0.2976
P03363	Q9UBF6	0.62803
P03363	Q659C4	0.065211
P03363	Q15121	0.63708
P03363	Q8WV44	0.58341
P03363	Q9BRV8	0.67647
P03363	Q9UDV7	0.20871
P03363	O94864	0.36081
P03363	A8MTY0	0.22259
P03363	O75558	0.53017
P03363	Q8N0X7	0.22614
P03363	Q3KP31	0.22259
P03363	Q9H307	0.76051
P03363	Q13033	0.56435
P03363	P17031	0.22259
P03363	Q9NXV2	1.0701
P03363	Q9H7B2	0.29721
P03363	Q8TAV0	0.023766
P03363	Q96QU4	0.22504
P03363	Q14152	0.88084
P03363	Q13342	0.51145
P03363	Q53EL6	0.83793
P03363	Q9H6D7	0.4726
P03363	Q8WWY6	0.40525
P03363	Q96I23	0.34493
P03363	Q8N680	0.22259
P03363	P78543	0.54178
P03363	P13051	0.1473
P03363	Q96MG8	0.22121
P03363	Q6P5R6	0.21422
P03363	Q96JK9	0.59831
P03363	Q5JT82	0.22831
P03363	Q9NV31	0.80406
P03363	O43316	0.32459
P03363	Q5MJ68	0.42926
P03363	Q9Y6K9	0.47963
P03363	Q9BT78	0.92881
P03363	Q6NSW5	0.10199
P03363	Q8NCN2	0.27378
P03363	Q02241	0.47433
P03363	Q15436	0.34477
P03363	Q13901	0.83508
P03363	O95602	0.33815
P03363	Q16621	0.37739
P03363	Q9H063	0.38995
P03363	P58304	0.44716
P03363	Q3B7T1	0.40525
P03363	Q99612	0.34587
P03363	Q9Y5M1	0.10939
P03363	Q9P0U4	0.23883
P03363	Q59GN2	0.21422
P03363	Q8N141	0.22259

P03363	P48200	0.33832
P03363	Q7Z713	0.4314
P03363	Q9UDY4	0.6655
P03363	Q9NPB6	0.44395
P03363	O00743	0.55761
P03363	Q309B1	0.24431
P03363	Q02080	0.46629
P03363	Q14C87	0.17204
P03363	Q86V15	0.29673
P03363	Q9UKV8	0.4476
P03363	Q9UJY1	0.62296
P03363	P15515	0.12501
P03363	Q8N2Z9	0.39531
P03363	Q5BKY1	0.22798
P03363	Q1ZZU3	0.68407
P03363	Q9P258	0.40205
P03363	Q9UJX3	0.3917
P03363	Q6ZN55	0.22259
P03363	Q2M3D2	0.11203
P03363	Q5VTR2	0.37808
P03363	P80217	0.63177
P03363	Q96K30	0.83344
P03363	Q8N488	0.57768
P03363	Q8IV54	0.29551
P03363	Q8TA86	0.58014
P03363	O75145	0.63823
P03363	Q9BT25	0.46589
P03363	P61353	0.38002
P03363	Q9NW07	0.18862
P03363	Q66K89	0.59101
P03363	O95671	0.30495
P03363	Q9BRX9	0.49849
P03363	Q8N1K5	0.27872
P03363	Q96C90	0.19151
P03363	Q9NUS5	0.51564
P03363	Q8IVW6	0.29101
P03363	P63121	0.030594
P03363	P47897	0.10198
P03363	Q4LE39	0.93944
P03363	A6NCN2	0.80822
P03363	P57077	0.76257
P03363	P24928	0.46701
P03363	Q9Y2D8	0.66578
P03363	Q96QR8	0.28891
P03363	Q9Y3M2	0.36765
P03363	Q13536	0.22504
P03363	Q8N2G8	0.22009
P03363	Q9UL41	0.20501
P03363	Q15631	0.73486
P03363	P98171	0.35591
P03363	P68133	0.21742
P03363	O75437	0.27693
P03363	P19419	0.90573
P03363	Q8IZY5	0.23882
P03363	O95456	0.58033
P03363	Q9C0F0	0.27176
P03363	Q9Y2L5	0.15699
P03363	O94983	1.034
P03363	Q16630	0.59631
P03363	A8CG34	0.34646
P03363	P83436	0.42245
P03363	Q76KX8	0.22259
P03363	Q9UEU5	0.50921
P03363	P60328	0.2934
P03363	Q9UQL6	0.2235
P03363	Q04726	0.78359
P03363	O76009	0.88908
P03363	Q13617	0.50462
P03363	Q13015	0.02117
P03363	A6NC98	0.30739
P03363	Q3LI66	0.27762
P03363	Q96F07	0.67612
P03363	Q9C0D0	0.21162
P03363	O75475	0.79297
P03363	O95153	0.67648
P03363	Q96DC7	0.11992

P03363	Q96EL3	0.22496
P03363	Q9Y597	0.21694
P03363	Q9UL40	0.64141
P03363	Q5U5X8	0.015116
P03363	P46109	0.26624
P03363	Q9NQ33	0.4568
P03363	Q96SC8	0.34884
P03363	Q4G0J3	0.50492
P03363	O60293	0.31008
P03363	P14921	0.55063
P03363	Q9NSV4	0.22983
P03363	P54252	0.3259
P03363	O75164	0.48218
P03363	Q9NYF0	0.31155
P03363	Q5EBL4	0.38366
P03363	Q9UER7	0.45483
P03363	P35548	0.37156
P03363	Q9Y6X4	0.070219
P03363	Q9HBZ2	0.39645
P03363	Q8IWL8	0.4314
P03363	Q9H4H8	0.42179
P03363	P0DJG4	0.045188
P03363	Q96D70	0.35279
P03363	O15151	0.25574
P03363	Q15427	0.64534
P03363	P61580	0.22504
P03363	Q96RU3	0.24517
P03363	Q99496	0.34465
P03363	P49366	0.41279
P03363	Q8WU76	0.23161
P03363	Q8TEK3	0.47984
P03363	Q9C099	0.71803
P03363	Q9H1Z4	0.49651
P03363	Q53HL2	0.57522
P03363	Q5F1R6	0.28736
P03363	Q9H9J2	0.13567
P03363	Q5JPH6	0.06083
P03363	Q156A1	0.2609
P03363	Q9BW71	0.68952
P03363	Q00610	0.36601
P03363	Q460N3	0.30552
P03363	Q96A35	0.21263
P03363	Q9Y5P3	0.53823
P03363	P07741	0.20393
P03363	Q9Y613	0.75526
P03363	Q9UPN9	0.4047
P03363	Q15041	0.19521
P03363	P0DI83	0.29721
P03363	O43422	0.16657
P03363	P13489	0.63192
P03363	Q00587	0.34945
P03363	Q5VU43	0.87894
P03363	Q8WVK7	0.48354
P03363	P0C7Q3	0.21644
P03363	P61289	0.22001
P03363	O00268	0.68061
P03363	Q8TAP6	0.82432
P03363	Q96JI7	0.54269
P03363	Q9BVC3	0.59267
P03363	Q8IY63	0.52423
P03363	Q9Y6K1	0.33384
P03363	Q15532	0.78083
P03363	P50548	0.59951
P03363	P62304	0.68761
P03363	Q96IS3	0.43824
P03363	Q9NZZ3	0.78062
P03363	Q8N9Z0	0.22259
P03363	Q7L3B6	0.24431
P03363	O43679	0.27836
P03363	Q9UET6	0.053192
P03363	Q8TC56	0.49651
P03363	Q6FI81	0.52141
P03363	P04732	0.15112
P03363	O14593	0.71769
P03363	Q9BXS6	0.44469
P03363	Q9Y467	0.25158

P03363	Q9Y603	1.0636
P03363	O75530	0.52502
P03363	Q6NUN9	0.67684
P03363	Q9UK11	0.21344
P03363	P49005	0.39685
P03363	P78395	0.55185
P03363	Q8WUF5	0.78467
P03363	Q86T75	0.24431
P03363	Q96MB7	0.29819
P03363	Q9HB65	0.42831
P03363	Q5VZR2	0.051971
P03363	O43318	0.21707
P03363	Q15700	0.27495
P03363	Q3KQV3	0.22259
P03363	P11172	0.086071
P03363	Q6L8G8	0.2934
P03363	Q9UL03	0.63748
P03363	Q9UKX7	0.66191
P03363	O15069	0.52433
P03363	O15105	0.15582
P03363	Q96M02	0.24601
P03363	Q9BZR8	0.52157
P03363	P57060	0.11203
P03363	Q9BXM8	0.11203
P03363	Q9BV68	0.29734
P03363	Q8IXK0	0.33109
P03363	Q96M29	0.26324
P03363	Q96PQ6	0.22259
P03363	Q9Y6B7	0.24686
P03363	Q15306	0.58598
P03363	Q01130	0.85631
P03363	Q86UW6	0.40027
P03363	Q0VD86	0.49651
P03363	O14579	0.33902
P03363	P50453	0.57045
P03363	Q8N782	0.22259
P03363	Q8TD10	0.22504
P03363	A6NDZ8	0.22927
P03363	Q9H410	0.60941
P03363	Q13522	0.35293
P03363	Q6P2Q9	0.76331
P03363	Q5TYW1	0.22259
P03363	O43776	0.18797
P03363	O00267	0.6682
P03363	Q9BX70	0.74013
P03363	O14713	0.48983
P03363	O14746	0.38324
P03363	Q9Y2A4	0.22558
P03363	Q8IWY8	0.268
P03363	P24941	0.16657
P03363	P78411	0.31264
P03363	Q99417	1.0307
P03363	Q96JN0	0.57321
P03363	Q96GC6	0.9017
P03363	Q9NP98	0.49778
P03363	Q8NHY3	0.21872
P03363	Q9H467	0.97472
P03363	P50748	0.53621
P03363	Q9ULL5	0.10474
P03363	Q6NT76	0.55279
P03363	Q8N5L8	0.35279
P03363	Q15797	0.17877
P03363	Q7RTT5	0.39008
P03363	Q8TF01	0.26782
P03363	P04156	0.16432
P03363	Q9C0E2	0.93943
P03363	Q5TH74	0.01442
P03363	O95073	0.89393
P03363	Q96LK0	0.34008
P03363	Q9UM63	0.31517
P03363	Q9Y4P9	0.2985
P03363	Q9GZS1	0.49488
P03363	Q969V6	1.0251
P03363	Q86U44	0.25684
P03363	Q14181	0.20852
P03363	Q96JN8	0.34026

P03363	Q9NW75	0.18926
P03363	Q9Y6M1	0.60667
P03363	Q8IYW5	0.22759
P03363	Q9HCI6	0.18106
P03363	P32243	0.56524
P03363	Q96A04	0.23268
P03363	Q5TAQ9	0.56144
P03363	Q9BZE1	0.31668
P03363	Q8TBB1	0.70166
P03363	Q9HCK0	0.23615
P03363	Q9NPC7	0.24904
P03363	Q3ZCQ8	0.2116
P03363	O15047	0.2542
P03363	Q5T2T1	0.27757
P03363	Q8IVS8	0.17493
P03363	Q5VWZ2	0.23943
P03363	Q9Y2K1	0.22259
P03363	Q14201	0.16851
P03363	P09234	0.58341
P03363	O14867	0.50022
P03363	O14948	0.46969
P03363	O15446	0.25049
P03363	O60479	0.36349
P03363	Q96EG3	0.22259
P03363	Q9BSQ5	0.52781
P03363	P08708	0.25253
P03363	Q6ZMV8	0.22259
P03363	Q6VVB1	0.33251
P03363	Q9NPJ4	1.0366
P03363	P35244	0.41425
P03363	P36508	0.21482
P03363	Q9UKZ1	0.1371
P03363	P63146	0.1209
P03363	Q6BEB4	0.26643
P03363	Q7Z3K6	0.56356
P03363	O76011	1.0007
P03363	O95231	0.42544
P03363	Q3LHN1	0.27762
P03363	O60381	0.39011
P03363	Q9Y2H8	0.22259
P03363	Q9H5P4	0.58418
P03363	Q04724	0.64458
P03363	Q9BYU5	0.2934
P03363	Q9BSI4	0.29473
P03363	Q9NQ50	0.37844
P03363	P51522	0.24904
P03363	Q9UJX5	0.38687
P03363	Q15596	0.43519
P03363	P49756	0.7641
P03363	Q9BYZ2	0.30317
P03363	O43309	0.268
P03363	Q6PGQ7	0.53844
P03363	P01858	0.04695
P03363	Q13765	0.97013
P03363	Q9BQ66	0.2934
P03363	Q9BPZ2	0.23489
P03363	Q52MB2	0.24431
P03363	O95486	0.1923
P03363	Q14839	0.51542
P03363	Q8N302	0.22812
P03363	P48668	0.44563
P03363	Q8NFV5	0.0070677
P03363	Q8N336	0.29436
P03363	Q8N693	0.56714
P03363	Q5TD94	0.33113
P03363	Q9P2H0	0.53508
P03363	Q9H322	0.11203
P03363	P16415	0.22259
P03363	Q9HCZ1	0.22259
P03363	Q9HCS4	0.2454
P03363	P43268	0.40069
P03363	Q86UE4	0.32137
P03363	Q14695	0.0095832
P03363	Q9UBU9	0.52842
P03363	Q9NSJ1	0.22259
P03363	Q9BX82	0.22259

P03363	Q9UJW7	0.22259
P03363	Q14588	0.22259
P03363	Q99828	0.23833
P03363	P55347	0.65493
P03363	P41208	0.50154
P03363	P17028	0.63369
P03363	O75312	0.1438
P03363	Q92558	0.5119
P03363	Q14681	0.21694
P03363	O43639	0.35902
P03363	O43248	0.41079
P03363	Q8NEA6	0.4515
P03363	P15880	0.54899
P03363	Q96CS3	0.55664
P03363	Q9NTU4	0.17686
P03363	Q7Z3Y8	0.88908
P03363	Q49MG5	0.65542
P03363	E9PGG2	0.55275
P03363	Q9Y3Y4	0.48975
P03363	Q8IZ26	0.22259
P03363	Q96N20	0.21805
P03363	P51161	0.24764
P03363	O75506	0.32285
P03363	P11940	0.56047
P03363	Q96L21	0.33428
P03363	Q9Y2X9	0.52774
P03363	Q9UBK9	0.88149
P03363	Q49AM1	0.2456
P03363	Q96KQ4	0.77602
P03363	Q86U42	0.79381
P03363	Q9H4A6	0.24787
P03363	Q9NR56	0.52696
P03363	Q8WWY3	0.58534
P03363	P25815	0.66559
P03363	Q15369	0.80802
P03363	Q9NT62	0.58163
P03363	Q13526	0.44349
P03363	O14893	0.69464
P03363	Q13601	0.55666
P03363	P53618	0.49556
P03363	Q9BSJ6	0.59219
P03363	P51826	0.39545
P03363	Q68DK2	0.61371
P03363	A6NCW3	0.0073048
P03363	P49639	0.28701
P03363	Q9P209	0.75097
P03363	Q5H9I0	0.5655
P03363	Q13158	0.33944
P03363	O75791	0.78839
P03363	P19883	0.23163
P03363	Q9HC44	0.43182
P03363	Q14686	0.29102
P03363	Q8IZD4	0.73552
P03363	Q7Z4S6	0.47577
P03363	P59923	0.268
P03363	Q99569	0.37973
P03363	Q16695	0.30502
P03363	P15927	0.3215
P03363	Q8TBK2	0.5604
P03363	O75970	0.29245
P03363	Q99471	0.84264
P03363	Q8IXW5	0.39653
P03363	P60520	0.31965
P03363	Q5TBC7	0.28678
P03363	P23508	0.4691
P03363	Q96MM3	0.31703
P03363	Q8WY41	0.42368
P03363	O00193	0.11203
P03363	P17041	0.22259
P03363	Q9BYP8	0.27762
P03363	Q9NWS9	0.268
P03363	P0C7V6	0.029024
P03363	Q8WXS3	0.4778
P03363	O95476	0.26573
P03363	Q8NI27	0.63816
P03363	Q9BYQ7	0.2934

P03363	Q9NWV4	0.11203
P03363	Q8N4B1	0.23681
P03363	Q9BWF3	0.27418
P03363	Q32P51	0.52567
P03363	Q9Y5R6	0.17363
P03363	P23497	0.54962
P03363	Q8N2H9	0.18345
P03363	Q8TD06	0.12523
P03363	P30260	0.69054
P03363	P49427	0.49514
P03363	A6NLC8	0.12118
P03363	P11844	0.02649
P03363	Q9UGY1	0.32192
P03363	Q8N1N5	0.45787
P03363	P06400	0.24443
P03363	Q9HAP2	0.54903
P03363	P40337	0.55377
P03363	Q15327	0.35116
P03363	A1L020	0.24888
P03363	P57086	0.4427
P03363	P48378	0.43182
P03363	Q8IUF8	0.51925
P03363	Q9HCX3	0.22259
P03363	Q8WU90	0.77646
P03363	Q14689	0.4943
P03363	Q7Z5Y7	0.21694
P03363	P61575	0.55152
P03363	Q15633	0.65091
P03363	Q86YE8	0.22259
P03363	Q6ZP82	0.53508
P03363	Q96H20	0.6477
P03363	Q7Z7F7	0.31668
P03363	Q13185	0.56447
P03363	Q8TCC3	0.22496
P03363	O43765	0.72635
P03363	Q96BV0	0.22259
P03363	Q15323	0.89041
P03363	Q96NT1	0.15989
P03363	Q9BUG6	0.268
P03363	Q9BYG4	0.5496
P03363	Q9Y6I0	0.030594
P03363	Q0P6D6	0.12015
P03363	Q9Y697	0.41777
P03363	Q8WUY3	0.25338
P03363	Q3SY89	0.19772
P03363	Q15652	0.26986
P03363	Q96FV9	0.81434
P03363	P17483	0.31803
P03363	P01106	0.22789
P03363	Q6L8H1	0.2934
P03363	Q6ZNH5	0.22259
P03363	Q9UMY4	0.32094
P03363	Q9NS26	0.35383
P03363	P63000	0.18541
P03363	P05787	1.1475
P03363	Q5H9M0	0.070039
P03363	Q49A88	0.12015
P03363	Q5JXB2	0.23751
P03363	Q9Y4X4	0.43321
P03363	P23381	0.40526
P03363	Q8N1L9	0.97635
P03363	P0CG24	0.22259
P03363	Q96GE4	0.37923
P03363	Q8NI51	0.53491
P03363	Q6UN15	0.29561
P03363	Q7L5A3	0.22504
P03363	Q9BQ69	0.43298
P03363	Q9UQ80	0.90551
P03363	Q96I27	0.22259
P03363	Q96T21	0.25023
P03363	O14717	0.22439
P03363	Q9NZN8	0.56747
P03363	Q9H2K2	0.25051
P03363	Q9BXS5	0.40876
P03363	Q96E29	0.2369
P03363	Q14593	0.22259

P03363	P50616	0.57311
P03363	Q8IXQ9	0.23591
P03363	Q96BF6	0.37504
P03363	Q9BRJ7	0.22637
P03363	P10144	0.37527
P03363	Q8NDV7	0.21253
P03363	Q9H4S2	0.45025
P03363	Q969E8	0.48884
P03363	Q9NPD3	0.32714
P03363	Q99743	0.43106
P03363	P17019	0.22259
P03363	Q07157	0.47387
P03363	Q7Z3Y9	1.0007
P03363	Q9BQE9	0.040875
P03363	Q07866	0.77456
P03363	A6H8Y1	0.57652
P03363	O75691	0.71089
P03363	Q7Z7K2	0.22259
P03363	B4DXR9	0.22259
P03363	A8K0S8	0.55275
P03363	Q04727	0.52747
P03363	Q96KF2	0.22504
P03363	Q14807	0.31898
P03363	Q16548	0.72895
P03363	O00409	0.38702
P03363	Q8NFH8	0.62465
P03363	Q92620	0.2815
P03363	Q9H0S4	0.4151
P03363	Q7KZF4	0.55009
P03363	Q12986	0.431
P03363	Q9BRP8	0.79685
P03363	P06748	0.34735
P03363	Q9H5H4	0.25992
P03363	Q7L576	0.23723
P03363	O00629	0.54083
P03363	P40222	0.24409
P03363	O94913	0.41923
P03363	Q96IG2	0.24431
P03363	Q8WYJ6	0.47723
P03363	Q9P031	0.89393
P03363	O14879	0.58966
P03363	O60493	0.37151
P03363	Q05086	0.36209
P03363	Q8IX90	0.56658
P03363	Q9H079	0.11503
P03363	Q8WYK2	0.50885
P03363	O00423	0.70485
P03363	P28749	0.85318
P03363	P41002	0.85129
P03363	Q9NXF7	0.56144
P03363	Q9UNY5	0.268
P03363	Q8NFC6	0.10474
P03363	P27815	0.25226
P03363	Q9Y6R7	0.33815
P03363	Q9HD47	0.3536
P03363	O94875	0.26606
P03363	Q96DV4	0.3842
P03363	Q63HM1	0.28776
P03363	Q9H489	0.15989
P03363	P35326	0.17378
P03363	A6NDR6	0.55275
P03363	O15372	0.65977
P03363	Q9GZW5	0.5002
P03363	O43189	0.41155
P03363	P20073	0.32585
P03363	P0CJ90	0.49158
P03363	Q9Y6I7	0.40597
P03363	Q96T51	0.22334
P03363	P01358	0.064654
P03363	P36952	0.15608
P03363	Q92890	0.77526
P03363	B1APH4	0.22259
P03363	Q9HAF1	0.81789
P03363	O43447	0.74643
P03363	Q9UNY4	0.6519
P03363	Q9C040	0.35146

P03363	Q6P3W6	0.24431
P03363	P35869	0.32434
P03363	Q9P299	0.29634
P03363	Q9H0U6	0.17909
P03363	Q9BZA5	0.035336
P03363	O95260	0.32814
P03363	P17275	0.55216
P03363	Q93052	0.39342
P03363	Q9NVA2	0.28202
P03363	Q8TE67	0.24431
P03363	Q93045	0.24838
P03363	Q6P050	0.10575
P03363	P42226	0.77381
P03363	Q9Y4G6	0.37468
P03363	Q96AP0	0.2411
P03363	P41091	0.21119
P03363	Q86WC6	0.44327
P03363	Q9UJX6	0.26157
P03363	Q9UGV2	0.13976
P03363	P13349	0.38212
P03363	P41567	0.23513
P03363	P21673	0.32544
P03363	Q96PK6	0.65563
P03363	O95571	0.22673
P03363	Q8IXH7	0.80315
P03363	P17509	0.35108
P03363	P31249	0.50815
P03363	P23588	0.32344
P03363	Q93015	0.18401
P03363	Q99729	0.40596
P03363	Q3B726	0.22236
P03363	Q504T8	0.29721
P03363	Q9C005	0.51491
P03363	P39687	0.92288
P03363	O43504	0.40683
P03363	O94973	0.26892
P03363	Q96PU8	0.49824
P03363	P49711	0.21681
P03363	Q9NR77	0.29794
P03363	P61923	0.31682
P03363	P57772	0.073828
P03363	P50995	0.57266
P03363	Q96RK1	0.46218
P03363	Q7Z4H3	0.2525
P03363	Q9P1Q0	0.50652
P03363	Q9BTE7	0.11203
P03363	Q9NTW7	0.22259
P03363	Q9BQ67	0.49651
P03363	Q9P2N5	0.28371
P03363	Q7L1Q6	0.43315
P03363	Q9HD33	0.35496
P03363	P62495	0.53503
P03363	Q92797	0.74212
P03363	Q7L2R6	0.22259
P03363	Q6VY07	0.41381
P03363	P18615	0.67433
P03363	O94900	0.27018
P03363	O00198	0.15525
P03363	Q08495	0.28628
P03363	O43818	0.30647
P03363	P61326	0.87544
P03363	Q96LL4	0.53508
P03363	P56645	0.40082
P03363	P54098	0.4153
P03363	O75150	0.42466
P03363	Q8N0Z2	0.26613
P03363	Q8TBC5	0.268
P03363	Q5T0L3	0.0065104
P03363	Q96TC7	0.45915
P03363	A6NK39	0.43424
P03363	P54845	0.5034
P03363	Q8IWZ8	0.56314
P03363	P81274	0.58568
P03363	Q5FBB7	0.45215
P03363	Q9BYE0	0.28014
P03363	P48788	0.47932

P03363	Q9UK39	0.53489
P03363	Q96IF1	0.23636
P03363	Q9UNX4	0.33175
P03363	Q8IYL2	0.1933
P03363	Q15750	0.31356
P03363	Q66LE6	0.32388
P03363	Q9HCS5	0.34705
P03363	Q15773	0.40941
P03363	Q9UM11	0.66448
P03363	Q8TEP8	0.72943
P03363	Q6ZNA4	0.39103
P03363	P0DI82	0.3031
P03363	P52657	0.69202
P03363	Q9NZV7	0.23191
P03363	Q8N668	0.63772
P03363	Q8NAQ8	0.1891
P03363	Q9HCN2	0.068021
P03363	P35754	0.2725
P03363	Q9UK10	0.22259
P03363	Q8IWE5	0.68353
P03363	Q9UJK0	0.041588
P03363	O95817	0.52882
P03363	P61244	0.68282
P03363	Q8NAA4	0.30524
P03363	O00488	0.22647
P03363	Q13425	0.5993
P03363	Q8NCA9	0.22259
P03363	O00321	0.42668
P03363	P07199	0.49301
P03363	Q6ZTU2	0.11917
P03363	Q5TYM5	0.23887
P03363	P33992	0.26734
P03363	Q14019	0.52475
P03363	Q9P0M2	0.24532
P03363	P62306	0.46036
P03363	P05198	0.58167
P03363	Q7Z7A3	0.5517
P03363	Q8N3H0	0.24431
P03363	Q9BX40	0.050431
P03363	P15924	0.37781
P03363	Q86VZ6	0.26271
P03363	Q6NXS1	0.035209
P03363	Q49B96	0.29635
P03363	P46100	0.44851
P03363	Q9BSB4	0.54684
P03363	O15116	0.81881
P03363	Q7KZ85	0.38784
P03363	O60296	0.4815
P03363	Q9UN30	0.44089
P03363	P14653	0.4555
P03363	Q05519	0.75142
P03363	O95707	0.54373
P03363	P78314	0.25744
P03363	Q9UNL4	0.23223
P03363	P50221	0.65907
P03363	Q13895	0.79124
P03363	Q8IWC1	0.59775
P03363	P10588	0.29335
P03363	Q9Y3B7	0.73363
P03363	Q6ZP65	0.3162
P03363	P26373	0.59745
P03363	O94850	0.2851
P03363	Q8N1B3	0.66973
P03363	Q13127	0.27517
P03363	Q3I5F7	0.26068
P03363	Q96PT4	0.52563
P03363	P35269	0.6731
P03363	Q8IY57	0.61955
P03363	Q9BZE3	0.52764
P03363	Q9H0W9	0.54311
P03363	Q9Y2Y9	0.70748
P03363	Q9C086	0.79875
P03363	P35908	0.59258
P03363	O60749	0.32867
P03363	P11473	0.317
P03363	O75920	0.11942

10565	P03363	Q8NEY8	0.4758
10566	P03363	Q8IYE0	0.054376
10567	P03363	Q53G26	0.2491
10568	P03363	Q16666	0.43511
10569	P03363	O75792	0.1628
10570	P03363	Q9UJ41	0.35909
10571	P03363	P50991	0.31156
10572	P03363	Q8N3Z6	0.39128
10573	P03363	Q8N8R7	0.7338
10574	P03363	Q9BS18	0.39867
10575	P03363	Q86UA6	0.46277
10576	P03363	P52272	0.57335
10577	P03363	Q6ZMV9	0.47577
10578	P03363	A6NNF4	0.22259
10579	P03363	Q07021	0.51413
10580	P03363	P54105	0.73448
10581	P03363	Q7Z6J8	0.31103
10582	P03363	Q13155	0.66249
10583	P03363	P40425	0.66358
10584	P03363	Q3YBM2	0.090985
10585	P03363	Q6ZVD7	0.52533
10586	P03363	Q3BBV1	0.24431
10587	P03363	Q8NG27	0.4694
10588	P03363	P83369	0.67349
10589	P03363	Q3LHN0	0.27762
10590	P03363	Q96BD5	0.3323
10591	P03363	Q9C0G0	0.22259
10592	P03363	Q13495	0.39509
10593	P03363	Q99608	0.20603
10594	P03363	Q4U2R6	0.70406
10595	P03363	Q9UJX4	0.38375
10596	P03363	Q6ZN11	0.22259
10597	P03363	O96017	0.21455
10598	P03363	Q7Z6Z7	0.53226
10599	P03363	Q8N9W6	0.32386
10600	P03363	Q96QD9	0.7642
10601	P03363	P0C7X5	0.22259
10602	P03363	P17029	0.24401
10603	P03363	Q9BXN6	0.4314
10604	P03363	Q15029	0.60753
10605	P03363	Q9Y3Q8	1.057
10606	P03363	Q9NX07	0.32936
10607	P03363	Q9H633	0.26638
10608	P03363	O60264	0.2266
10609	P03363	Q15287	0.82994
10610	P03363	Q96CD0	0.53508
10611	P03363	P61088	0.45707
10612	P03363	Q12800	0.46404
10613	P03363	Q9H7C4	0.50887
10614	P03363	Q14746	0.37633
10615	P03363	Q15435	0.36379
10616	P03363	P06396	0.25407
10617	P03363	Q6L8G5	0.2934
10618	P03363	Q9NWB7	0.64239
10619	P03363	O75663	0.65848
10620	P03363	P25791	0.34242
10621	P03363	Q92529	0.32948
10622	P03363	O75351	0.24984
10623	P03363	Q9BQI6	0.2946
10624	P03363	Q9UPN7	0.90864
10625	P03363	Q9H1A7	0.23378
10626	P03363	Q5JR59	0.84049
10627	P03363	Q9UM07	0.29502
10628	P03363	Q96BR6	0.22259
10629	P03363	Q9H900	0.34933
10630	P03363	A4D263	0.11203
10631	P03363	Q8IZS6	0.31738
10632	P03363	Q9P2Z0	0.13635
10633	P03363	Q9BYQ9	0.2934
10634	P03363	O60813	0.10142
10635	P03363	P57678	0.79503
10636	P03363	Q9P2W9	0.19804
10637	P03363	P60331	0.2934
10638	P03363	Q6P1L6	0.22259
10639	P03363	Q5BKZ1	0.37781
10640	P03363	A6NK53	0.27378

P03363	Q6IV72	0.38353
P03363	Q9BTL3	0.67638
P03363	O60282	0.59438
P03363	Q9BQA1	0.72345
P03363	P83881	0.49029
P03363	C9JE40	0.92739
P03363	P51786	0.303
P03363	Q9BYP9	0.2934
P03363	Q9Y2J4	0.35586
P03363	Q96EH5	0.19237
P03363	Q8TF45	0.22259
P03363	Q96RM1	0.22177
P03363	P30304	0.34481
P03363	Q9H320	0.30058
P03363	P10072	0.11993
P03363	Q96P63	0.16116
P03363	Q8WV41	0.43009
P03363	P29372	0.41431
P03363	Q9H1A3	0.53508
P03363	B3EWF7	0.22504
P03363	Q14159	0.063244
P03363	Q8NEU8	0.4687
P03363	O76021	0.22585
P03363	Q96QF7	0.22504
P03363	Q12962	0.71656
P03363	O75157	0.29405
P03363	P10911	0.25109
P03363	P48728	0.16724
P03363	Q3MIV0	0.27762
P03363	Q8IWZ3	0.95224
P03363	Q96BW9	0.11388
P03363	Q8N684	0.66975
P03363	Q9BXF3	0.62564
P03363	Q9NZN9	0.24048
P03363	O75400	0.74099
P03363	Q9Y608	0.50498
P03363	O95405	0.22359
P03363	Q9Y6X2	0.60612
P03363	Q03936	0.30118
P03363	Q9BZF9	0.46732
P03363	Q9H4M3	0.4625
P03363	Q9H9F9	0.91748
P03363	P63151	0.39317
P03363	Q5TCZ1	0.47607
P03363	Q92572	0.38332
P03363	Q9NQ03	0.29518
P03363	Q5VV67	0.36049
P03363	Q6NZ36	0.28746
P03363	O43524	0.3249
P03363	Q03252	0.44084
P03363	O14599	0.56913
P03363	Q5TZA2	0.7381
P03363	Q9C075	0.79043
P03363	Q96EX3	0.24431
P03363	Q56P42	0.4314
P03363	P15923	0.34925
P03363	Q9NVV5	0.15709
P03363	P35900	1.0007
P03363	Q96RE9	0.22259
P03363	O60287	0.3193
P03363	Q9UGP4	0.32931
P03363	Q6ZN19	0.22259
P03363	P15336	0.44309
P03363	Q9BPX1	0.31201
P03363	O60885	0.68229
P03363	O75334	0.69923
P03363	Q15326	0.75237
P03363	Q8NDN9	0.58738
P03363	A6NF36	0.08959
P03363	Q9UD57	0.42544
P03363	Q13951	0.71046
P03363	P63104	0.61146
P03363	Q9BQ90	0.76096
P03363	Q2M1K9	0.35777
P03363	Q6A162	0.78878
P03363	Q8TDY3	0.29593

P03363	Q8TDC0	0.69364
P03363	Q6PGP7	0.83401
P03363	Q13748	0.31729
P03363	Q96T23	0.28845
P03363	P43358	0.56434
P03363	Q9H379	0.025527
P03363	Q9H305	0.21411
P03363	A6NFL8	0.019317
P03363	Q9Y3B4	0.28046
P03363	Q6AI39	0.0011991
P03363	Q9H0R8	0.31711
P03363	Q9P2T0	0.44317
P03363	Q14684	0.24799
P03363	P17023	0.24904
P03363	Q14690	0.65118
P03363	Q86T29	0.22259
P03363	Q9UK58	0.43606
P03363	Q9BRJ2	0.31882
P03363	Q00534	0.25413
P03363	P07196	0.67178
P03363	Q9BSN8	0.24352
P03363	P20749	0.27272
P03363	Q6RSH7	0.35546
P03363	Q9HBK9	0.21159
P03363	Q9UKA2	0.49193
P03363	Q96EF6	0.43705
P03363	Q5EE01	0.44556
P03363	Q92636	0.56781
P03363	Q96D15	0.34375
P03363	Q9HD36	0.39797
P03363	Q9NX08	0.53508
P03363	Q13867	0.37978
P03363	Q969L2	0.15277
P03363	Q16543	0.51436
P03363	Q9ULM6	0.57285
P03363	Q00403	0.55093
P03363	Q8NAF0	0.22259
P03363	A6NFY7	0.056662
P03363	Q9UBV8	0.42868
P03363	Q96DT7	0.27693
P03363	P61578	0.55152
P03363	Q13609	0.13506
P03363	Q15937	0.21805
P03363	Q92560	0.23439
P03363	Q9NRR5	0.59731
P03363	O15499	0.51754
P03363	Q8TAT6	0.33231
P03363	Q15424	0.77447
P03363	Q9UBI1	0.53508
P03363	Q9Y2G9	0.20898
P03363	O15117	0.5325
P03363	Q5VYS4	0.24287
P03363	Q8NEF9	0.6258
P03363	Q9NWU5	0.20117
P03363	Q96LY2	0.0079666
P03363	Q15653	0.67783
P03363	Q8TE85	0.29363
P03363	Q13619	0.40627
P03363	Q9ULU4	0.48315
P03363	Q9BXY0	0.29721
P03363	Q9Y4E1	0.11
P03363	Q9HB75	0.75004
P03363	P0CL85	0.22504
P03363	Q9P1T7	0.79648
P03363	Q96P71	0.46738
P03363	P55212	0.28431
P03363	Q9BWJ5	0.41785
P03363	O75446	0.7982
P03363	O43615	0.24579
P03363	Q9NRG9	0.52135
P03363	Q8NCR6	0.33932
P03363	Q16204	0.28924
P03363	Q495Y8	0.0070677
P03363	P08727	1.0007
P03363	O75031	0.10433
P03363	P22090	0.24437

P03363	Q5JTW2	0.42673
P03363	Q96FH0	0.47935
P03363	Q92904	0.34172
P03363	O15144	0.50442
P03363	P32519	1.0963
P03363	P15822	0.82045
P03363	Q6P2S7	0.24431
P03363	Q14140	0.47223
P03363	Q07352	0.70109
P03363	Q9C0C7	0.40869
P03363	P61163	0.16655
P03363	P04053	0.30509
P03363	Q9H112	0.30292
P03363	Q9P032	0.49115
P03363	P48552	0.38968
P03363	Q9Y3I1	0.84782
P03363	Q9UBU8	0.5822
P03363	Q8IZU1	0.29721
P03363	P0C7V5	0.22259
P03363	Q6PGN9	0.57388
P03363	Q9BSF8	0.49651
P03363	Q9Y333	0.70799
P03363	Q8WY36	0.43182
P03363	Q9H0U9	0.47026
P03363	Q14562	0.49856
P03363	O43593	0.2562
P03363	A8MUZ8	0.22259
P03363	Q5JY77	0.7338
P03363	P62861	0.31766
P03363	Q92832	0.3351
P03363	Q2M1V0	0.51853
P03363	Q05066	0.59554
P03363	Q8N228	0.43182
P03363	Q9NVM4	0.21835
P03363	Q9BSU3	0.42889
P03363	Q8N7G0	0.51853
P03363	Q8N6M9	0.20313
P03363	Q2VY69	0.22259
P03363	Q06250	0.11203
P03363	P62841	0.40736
P03363	Q9Y5Z7	0.48522
P03363	Q6ZQR2	0.11822
P03363	P62316	0.76043
P03363	Q8WXI9	0.31644
P03363	A8MTZ0	0.53376
P03363	Q3SYA9	0.11203
P03363	Q8WUM0	0.37921
P03363	P61457	0.69157
P03363	Q13242	0.37397
P03363	Q5JSS6	0.1103
P03363	Q9UNX3	0.33188
P03363	A8MVW5	0.52788
P03363	Q9BVL2	0.35837
P03363	Q9NR50	0.47896
P03363	Q9BW30	0.23412
P03363	O94952	0.13521
P03363	P08133	0.61836
P03363	Q96Q83	0.30015
P03363	Q9H0B6	0.89545
P03363	Q96QU8	0.84819
P03363	Q8N456	0.24431
P03363	Q96JM2	0.32823
P03363	Q96J87	0.3157
P03363	A6NEJ1	0.39008
P03363	Q99549	0.44189
P03363	Q9UBP6	0.75027
P03363	A3KN83	0.11203
P03363	Q99708	0.43769
P03363	O43830	0.24904
P03363	Q8TC84	0.49651
P03363	Q5VSR9	0.01487
P03363	Q96E66	0.24431
P03363	Q9BRP1	0.2724
P03363	Q99567	0.42724
P03363	Q14602	0.43052
P03363	Q9BTC8	0.59842

P03363	O75529	0.46553
P03363	Q96EW2	0.24431
P03363	O95218	0.57228
P03363	P10155	0.57953
P03363	Q96JM3	0.39133
P03363	C9JN71	0.18593
P03363	Q96SI1	0.21694
P03363	Q08050	0.36749
P03363	Q96LW9	0.268
P03363	P13378	0.34195
P03363	Q96F86	0.64897
P03363	P22103	0.09071
P03363	A6NNW6	0.094981
P03363	Q92688	0.4314
P03363	P30622	0.27264
P03363	A6NH11	0.21406
P03363	Q9HCU8	0.34626
P03363	Q99457	0.17795
P03363	Q9NSA3	0.66163
P03363	Q8IYB9	0.22259
P03363	P20585	0.16363
P03363	P07320	0.64176
P03363	Q14669	0.52045
P03363	P0CJ85	0.49158
P03363	Q8N660	0.24431
P03363	Q8IY85	0.43013
P03363	Q9NX18	0.71457
P03363	P11831	0.23254
P03363	Q8TDR4	0.22504
P03363	Q6ZNG9	0.24806
P03363	Q8WYN3	0.54964
P03363	P29373	0.27109
P03363	Q96D03	0.22969
P03363	Q9P0W5	0.66638
P03363	O75394	0.21263
P03363	O15226	0.8115
P03363	P54136	0.56811
P03363	Q8TF39	0.268
P03363	Q9UPY8	0.80134
P03363	Q9NSC5	0.47705
P03363	Q9BS31	0.22259
P03363	Q4L180	0.3983
P03363	Q9NY61	0.64304
P03363	O75208	0.026987
P03363	Q969H4	0.31643
P03363	Q9Y3V2	0.92347
P03363	Q9H4I8	0.24704
P03363	Q13129	0.5599
P03363	Q8TC71	0.43623
P03363	Q9NX46	0.32461
P03363	Q9HCK8	0.33941
P03363	Q5JWR5	0.14787
P03363	Q8N972	0.22259
P03363	Q9UN37	0.37697
P03363	Q8IYM1	0.24247
P03363	P84101	0.30086
P03363	Q3LI60	0.27762
P03363	Q96MA1	0.31718
P03363	P30050	0.42896
P03363	P46937	0.53533
P03363	Q9H0A8	0.7338
P03363	Q7L266	0.33581
P03363	Q15293	0.33053
P03363	Q96A56	0.66694
P03363	Q5T752	0.23311
P03363	Q14872	0.52543
P03363	Q8N3S3	0.32756
P03363	Q9HBQ8	0.042292
P03363	Q16576	0.42351
P03363	P56693	0.49625
P03363	P27816	0.53996
P03363	Q8IV36	0.46465
P03363	Q9NNX9	0.22583
P03363	Q53HC0	0.80091
P03363	Q9H0W8	0.39142
P03363	Q8WUQ7	0.37398

P03363	Q13426	0.26346
P03363	Q717R9	0.32795
P03363	P28702	0.29444
P03363	Q8IWE2	0.24431
P03363	O60925	0.18144
P03363	Q9BPY8	0.36235
P03363	Q8IYB3	0.51074
P03363	Q86XK3	0.68407
P03363	P60014	0.2934
P03363	Q7Z699	0.33709
P03363	Q9BYD5	0.21723
P03363	Q9BYR5	0.2934
P03363	Q13416	0.46231
P03363	Q9P0K7	0.27299
P03363	Q6P1M3	0.8156
P03363	P35325	0.22177
P03363	P82912	0.1927
P03363	Q5SYE7	0.032049
P03363	Q9NUL7	0.22885
P03363	P60329	0.2934
P03363	Q96FX2	0.74445
P03363	Q9NWA0	0.82851
P03363	Q8N7B1	0.043481
P03363	Q9Y3C8	0.48888
P03363	Q96R06	0.64985
P03363	Q9BYN7	0.27378
P03363	O43790	0.8697
P03363	P20264	0.24711
P03363	Q8NBC4	0.24431
P03363	P28069	0.52262
P03363	Q8IXQ5	0.40887
P03363	Q8N5X7	0.26992
P03363	Q5T6F0	0.40243
P03363	P61960	0.41113
P03363	Q96N58	0.22259
P03363	Q7Z4W3	0.27762
P03363	Q9ULZ0	0.4314
P03363	Q9UI36	0.52779
P03363	O60583	0.57505
P03363	O96019	0.52589
P03363	Q5T124	0.29593
P03363	Q53EZ4	0.50864
P03363	Q9H4T2	0.268
P03363	P59998	0.46393
P03363	Q49AN0	0.26972
P03363	Q6PI26	0.89836
P03363	Q9UDW3	0.47296
P03363	P60006	0.061076
P03363	P14859	0.94584
P03363	Q9P127	0.22504
P03363	Q8IUC1	0.42225
P03363	P28358	0.3466
P03363	Q9Y4X5	0.46926
P03363	Q68CZ6	0.4726
P03363	Q68D10	0.11203
P03363	O76013	0.89041
P03363	O43809	0.56693
P03363	Q2VPK5	0.67538
P03363	P62829	0.55259
P03363	Q9BUE0	0.82851
P03363	Q13616	0.35757
P03363	Q9ULZ2	0.65184
P03363	Q9NVX2	0.29739
P03363	Q8TF09	0.37563
P03363	Q14119	0.31074
P03363	Q53T94	0.26789
P03363	O43684	0.31759
P03363	O95067	0.31835
P03363	Q92622	0.18255
P03363	Q8NB91	0.29751
P03363	Q9BXF9	0.26324
P03363	Q6ZNG2	0.52563
P03363	Q16829	0.2428
P03363	O96020	0.64535
P03363	Q8N393	0.22259
P03363	A1A580	0.27762

P03363	Q12798	0.66176
P03363	P63165	0.66291
P03363	Q00444	0.44984
P03363	Q96HT8	0.20852
P03363	P56915	0.32408
P03363	P38159	0.66016
P03363	Q12906	0.84401
P03363	Q9NZ32	0.23974
P03363	P18583	0.62069
P03363	Q9NSI6	0.64894
P03363	Q8N5P1	0.2936
P03363	P57076	0.27961
P03363	Q5MJ10	0.01487
P03363	P52737	0.73638
P03363	Q6ULP2	0.53098
P03363	Q96MC4	0.11297
P03363	P13647	0.86201
P03363	Q00653	0.75738
P03363	Q14999	0.77037
P03363	Q15544	0.62617
P03363	Q92599	0.41076
P03363	Q9BZD3	0.10174
P03363	Q96QA5	0.27678
P03363	Q96T76	0.62917
P03363	Q5VWJ9	0.24351
P03363	Q9Y5J1	0.25488
P03363	Q96AE4	0.56465
P03363	P11308	0.79075
P03363	Q9BY77	0.69105
P03363	P08107	0.40894
P03363	Q96IR2	0.22259
P03363	Q9UPG8	0.42837
P03363	Q9BSM1	0.60454
P03363	Q8N8Z8	0.22259
P03363	A8MZ59	0.55275
P03363	Q8N157	0.22801
P03363	Q8TF20	0.22259
P03363	Q03111	1.119
P03363	Q6PIF2	0.32732
P03363	O94927	0.4726
P03363	Q92841	0.57857
P03363	Q9ULD5	0.23615
P03363	O96002	0.11203
P03363	Q6DKK2	0.72037
P03363	P26368	0.68828
P03363	P26641	0.4444
P03363	Q9NRG4	0.48819
P03363	Q3SYG4	0.47757
P03363	Q9H974	0.0644
P03363	P42772	0.55312
P03363	Q9BPZ3	0.65229
P03363	Q8WUM4	0.79557
P03363	Q0D2I5	0.27762
P03363	Q5XKL5	0.29721
P03363	Q659A1	0.22504
P03363	Q8IX15	0.57774
P03363	O95238	0.4984
P03363	Q9H0R1	0.2221
P03363	Q9H7N4	0.3261
P03363	Q8WW33	0.13038
P03363	P81877	0.6488
P03363	Q13415	0.2793
P03363	P62877	0.32778
P03363	Q9GZU2	0.45752
P03363	Q14532	0.69887
P03363	Q9GZZ0	0.39652
P03363	Q9NR09	0.32745
P03363	Q96FA3	0.21998
P03363	O75030	0.48406
P03363	Q96KN3	0.49161
P03363	Q9Y5B9	0.22708
P03363	P08235	0.32091
P03363	A6NNV3	0.0070677
P03363	Q9BZP3	0.11203
P03363	Q86WG3	0.49522
P03363	Q86VN1	0.41511

11097	P03363	P63172	0.54912
11098	P03363	P82663	0.49416
11099	P03363	Q17RP2	0.40039
11100	P03363	Q14240	0.23712
11101	P03363	P46063	0.32259
11102	P03363	Q9GZT3	0.30241
11103	P03363	Q15149	0.29248
11104	P03363	O75635	0.18824
11105	P03363	Q9H425	0.24431
11106	P03363	Q6K0P9	0.33619
11107	P03363	P46782	0.28822
11108	P03363	Q8N0T1	0.0086443
11109	P03363	Q9P2S5	0.3171
11110	P03363	Q8IY31	0.57998
11111	P03363	Q96BZ8	0.11203
11112	P03363	Q6ZW54	0.074527
11113	P03363	Q96PU5	0.28634
11114	P03363	Q8TAD1	0.4314
11115	P03363	Q9UPU9	0.14013
11116	P03363	Q92945	0.65944
11117	P03363	P35268	0.41414
11118	P03363	Q96BN2	0.3793
11119	P03363	Q9NRX2	0.19275
11120	P03363	Q9UHY8	0.44508
11121	P03363	Q96HJ5	0.32523
11122	P03363	Q86V25	0.22123
11123	P03363	Q99627	0.47926
11124	P03363	P51504	0.24904
11125	P03363	P17480	0.75546
11126	P03363	Q9P1Y5	0.28234
11127	P03363	Q92902	0.25554
11128	P03363	P34932	0.25526
11129	P03363	Q9Y250	0.64604
11130	P03363	Q9NZ81	0.4684
11131	P03363	Q6P1J9	0.50312
11132	P03363	Q9BQ15	0.72926
11133	P03363	Q8IYB7	0.22518
11134	P03363	Q9UI08	0.38396
11135	P03363	Q9BQS6	0.89306
11136	P03363	P21506	0.22259
11137	P03363	Q6ZN32	0.51853
11138	P03363	Q14241	0.29991
11139	P03363	Q99698	0.25554
11140	P03363	Q8WW59	0.24079
11141	P03363	O75113	0.76346
11142	P03363	Q8N5F7	0.98185
11143	P03363	P24278	0.80384
11144	P03363	O75800	0.48488
11145	P03363	Q9Y3C4	0.4225
11146	P03363	Q9HBT7	0.16475
11147	P03363	Q12894	0.56551
11148	P03363	Q86TA1	0.412
11149	P03363	Q8IW36	0.20893
11150	P03363	Q12874	0.56425
11151	P03363	Q9UHV2	0.58342
11152	P03363	Q8IYN2	0.40525
11153	P03363	Q15717	0.3629
11154	P03363	Q9NSK0	0.46429
11155	P03363	Q8IY81	0.66179
11156	P03363	Q8WUN7	0.49651
11157	P03363	Q9Y2M5	0.71336
11158	P03363	Q9GZV5	0.70713
11159	P03363	Q99426	0.52756
11160	P03363	Q9BXW6	0.40437
11161	P03363	Q14814	0.48835
11162	P03363	Q8N6T7	0.33344
11163	P03363	Q13156	0.26132
11164	P03363	Q9UPN6	0.26998
11165	P03363	O95433	0.53316
11166	P03363	Q9NR28	0.62667
11167	P03363	A6NJT0	0.27589
11168	P03363	P29692	0.37027
11169	P03363	Q9NZC4	0.22479
11170	P03363	P20962	0.24602
11171	P03363	P61571	0.55152
11172	P03363	P54278	0.31322

P03363	P49450	0.22902
P03363	Q8TEB1	0.56144
P03363	Q6XZF7	0.42513
P03363	Q8N1I0	0.37078
P03363	Q6VB84	0.61062
P03363	P78545	0.69055
P03363	P31943	0.81135
P03363	Q9P255	0.22259
P03363	Q13506	0.31719
P03363	Q9NZI8	0.34705
P03363	Q08999	0.96354
P03363	Q9BRG1	0.6809
P03363	Q86UU5	0.074044
P03363	Q9BTE6	0.096938
P03363	Q86WP2	0.52295
P03363	O43172	0.76006
P03363	P05423	0.15972
P03363	Q9NYV6	0.27605
P03363	Q13480	0.23095
P03363	Q9P0J0	0.30229
P03363	Q9NR22	0.35192
P03363	Q14549	0.51582
P03363	Q9UK99	0.59082
P03363	Q9NWV8	0.66031
P03363	Q9BX46	0.28073
P03363	Q32MQ0	0.14777
P03363	P0CG13	0.24693
P03363	Q9BW66	0.74837
P03363	P17039	0.22259
P03363	Q5UE93	0.43138
P03363	P62324	0.63653
P03363	Q6P161	0.22496
P03363	O95721	0.38663
P03363	Q8IXT2	0.26821
P03363	Q6PF04	0.22259
P03363	Q8TAF7	0.22259
P03363	B7ZBB8	0.15493
P03363	Q9H267	0.35841
P03363	P04259	0.47869
P03363	Q76G19	0.23169
P03363	Q96H40	0.22259
P03363	Q14CN4	0.48919
P03363	Q15035	0.25211
P03363	Q9H2G4	0.42108
P03363	P31749	0.15315
P03363	Q92830	0.38536
P03363	Q6T4R5	0.17199
P03363	O15318	0.11633
P03363	Q5VIY5	0.22259
P03363	Q9UDV6	0.60224
P03363	Q9UKK3	0.68565
P03363	Q5JNZ3	0.22259
P03363	Q9NZI6	0.49765
P03363	Q86WZ6	0.22259
P03363	Q14511	0.60301
P03363	Q9BQ87	0.40525
P03363	Q8NB16	0.26356
P03363	Q96BY2	0.62128
P03363	Q7Z2E3	0.28348
P03363	Q3LI59	0.27762
P03363	Q96C28	0.22259
P03363	Q13077	0.41034
P03363	Q96E14	0.41993
P03363	P17032	0.24904
P03363	Q5T1B1	0.14412
P03363	P17096	0.26684
P03363	Q9P1Z2	0.64061
P03363	Q9H808	0.62588
P03363	Q8IYJ2	0.058413
P03363	Q8NB12	0.31648
P03363	Q9ULR0	0.40296
P03363	O14979	0.53548
P03363	Q9Y6G3	0.19217
P03363	P54274	0.33359
P03363	Q9C0C2	0.27161
P03363	C9JLW8	0.016606

P03363	Q5TI25	0.24431
P03363	Q8IZ20	0.22259
P03363	Q9BXK1	0.23523
P03363	Q5SWL7	0.10142
P03363	Q14677	0.48599
P03363	Q66PJ3	0.72935
P03363	Q9BWT7	0.45387
P03363	Q96NM4	0.43182
P03363	Q9H609	0.22259
P03363	Q13432	0.58787
P03363	Q8NEY3	0.21346
P03363	Q13588	0.24874
P03363	Q96KP1	0.40586
P03363	P55209	0.60352
P03363	P63124	0.099877
P03363	Q13489	0.23965
P03363	Q8NEK8	0.0035228
P03363	Q9UJU3	0.22259
P03363	O15453	0.11203
P03363	P14316	0.76247
P03363	Q96EK7	0.34757
P03363	P51003	0.20068
P03363	Q14527	0.38762
P03363	P32242	0.33211
P03363	Q9H3P2	0.77008
P03363	Q9UKT5	0.54396
P03363	Q9UHR5	0.89911
P03363	P18846	0.57283
P03363	Q9BVW5	0.67344
P03363	Q9H0C8	0.42123
P03363	Q01094	0.5611
P03363	Q96QE5	0.73645
P03363	Q8WYA1	0.48735
P03363	Q8NA77	0.22504
P03363	Q9UIF9	0.40808
P03363	Q96JY0	0.16472
P03363	Q8N6Y0	0.68643
P03363	Q3T8J9	0.64238
P03363	P35226	0.38228
P03363	P16333	0.43959
P03363	Q86WI3	0.52902
P03363	Q05DH4	0.065239
P03363	O75901	0.35789
P03363	Q8IZD9	0.47663
P03363	O60826	0.11203
P03363	Q9Y5R5	0.29458
P03363	Q6IQ19	0.076765
P03363	Q6PI48	0.58987
P03363	Q9NQS7	0.58105
P03363	Q8TAE8	0.7254
P03363	Q8NDF8	0.3187
P03363	Q5T1N1	0.24431
P03363	Q92552	0.68391
P03363	Q9BYC8	0.3019
P03363	O75444	0.58734
P03363	Q8N1G1	0.18666
P03363	P35527	1.0007
P03363	A6NDX5	0.22259
P03363	O00148	0.66406
P03363	Q9NXT0	0.22259
P03363	Q9UKV0	0.26446
P03363	Q9Y3D8	0.2817
P03363	P06746	0.41962
P03363	P43487	0.3315
P03363	Q9NVC6	0.22577
P03363	P27797	0.16611
P03363	P14652	0.19159
P03363	O14709	0.268
P03363	O75505	0.55275
P03363	Q6NX45	0.22259
P03363	Q9BSH5	0.4099
P03363	Q9NP08	0.375
P03363	Q99741	0.30597
P03363	Q9HC62	0.41709
P03363	Q9HBL8	0.26606
P03363	P61024	0.49464

P03363	Q13472	0.25813
P03363	P04731	0.15112
P03363	Q6ZNB5	0.20567
P03363	Q5VSL9	0.85425
P03363	P20042	0.39855
P03363	P0CG29	0.42083
P03363	Q8IY51	0.40039
P03363	P61925	0.35153
P03363	Q5JVS0	0.63973
P03363	P11532	0.29899
P03363	Q86VY4	0.5449
P03363	Q969G3	0.71292
P03363	O95678	0.68108
P03363	O00522	0.30335
P03363	I6L9F6	0.41155
P03363	O00327	0.33812
P03363	Q8N0Y7	0.083337
P03363	O43186	0.7455
P03363	Q9UBB4	0.57799
P03363	Q7Z3C6	0.26795
P03363	Q9H4Z2	0.63436
P03363	P46781	0.61784
P03363	Q6L8G9	0.2934
P03363	Q9H668	0.46393
P03363	A6NE82	0.22927
P03363	Q9BWH6	0.24301
P03363	P51814	0.24904
P03363	Q02410	0.48053
P03363	Q8NC69	0.21694
P03363	O94763	0.62608
P03363	P47813	0.13095
P03363	Q96RT8	0.45782
P03363	Q7Z4H9	0.22504
P03363	O75534	0.57125
P03363	P50914	0.65577
P03363	Q6PCD5	0.23534
P03363	Q71F56	0.37738
P03363	Q9BSK1	0.22259
P03363	Q92837	0.24396
P03363	Q6ZNE5	0.47547
P03363	Q9UIF8	0.6288
P03363	Q9UNE7	0.26366
P03363	O15525	0.44404
P03363	Q9BVV6	0.11277
P03363	Q9H2W6	0.25735
P03363	Q9NVX0	0.61815
P03363	Q8ND23	0.24431
P03363	Q6H8Q1	0.23825
P03363	O60688	0.19397
P03363	Q9UBS4	0.23859
P03363	Q86UW9	0.76637
P03363	Q15637	0.35924
P03363	O14645	0.2446
P03363	P38646	0.56027
P03363	P31260	0.55603
P03363	Q13123	0.54406
P03363	H3BTG2	0.0053627
P03363	Q32P44	0.34776
P03363	P19474	0.47789
P03363	Q9BSW2	0.7235
P03363	Q9NP64	0.35933
P03363	Q96DE0	0.19761
P03363	Q9HCE1	0.61927
P03363	Q5VWX1	0.67115
P03363	Q14134	0.59644
P03363	Q9NQG5	0.86713
P03363	Q7L5N1	0.92823
P03363	Q5TCQ9	0.40391
P03363	Q96LT9	0.24492
P03363	Q17RG1	0.21694
P03363	Q13310	0.77257
P03363	Q16670	0.23235
P03363	P52736	0.65117
P03363	O75155	0.61616
P03363	Q9Y5J7	0.14446
P03363	Q9NZR1	0.23426

P03363	Q86T82	0.25891
P03363	A6NMT0	0.40177
P03363	P28290	0.062739
P03363	Q5T6M2	0.0066151
P03363	Q8WVY7	0.27775
P03363	Q9P0U1	0.202
P03363	Q8NE18	0.094198
P03363	Q96ST3	0.51436
P03363	Q9HBD4	0.39255
P03363	O60318	0.24746
P03363	O76075	0.21428
P03363	Q9BPU9	0.2763
P03363	P0C1Z6	0.98096
P03363	Q6A163	0.88908
P03363	Q6TGC4	0.29467
P03363	Q9UHQ1	0.17275
P03363	Q7Z5Q5	0.25024
P03363	P07998	0.2405
P03363	Q5VYV7	0.53508
P03363	Q99732	0.21901
P03363	Q13395	0.2962
P03363	P22528	0.29708
P03363	Q5CZA5	0.22259
P03363	Q9H939	0.25665
P03363	Q8N7M2	0.22259
P03363	P78396	0.57502
P03363	Q9UH90	0.30704
P03363	O00512	0.81126
P03363	Q7Z7C8	0.48883
P03363	P08670	0.34789
P03363	Q5T230	0.89155
P03363	Q06416	0.51853
P03363	Q96AQ1	0.0079666
P03363	Q96AQ6	0.75093
P03363	Q92843	0.40398
P03363	O43541	0.48322
P03363	Q9UK41	0.50017
P03363	Q14749	0.54323
P03363	O43815	0.33268
P03363	Q9UGR2	0.48055
P03363	Q9Y6Q3	0.16808
P03363	Q7RTU0	0.43142
P03363	Q96EB1	0.89686
P03363	P17022	0.268
P03363	O60870	0.17498
P03363	Q96FX7	0.19261
P03363	O95379	0.24003
P03363	Q9BZV1	0.99442
P03363	Q8WZ82	0.21103
P03363	O14633	0.12558
P03363	Q6B0B8	0.40039
P03363	Q14966	0.3115
P03363	Q96E16	0.17461
P03363	O15056	0.24567
P03363	P17661	0.63425
P03363	Q15814	0.21923
P03363	Q8N5A5	0.74446
P03363	Q9NPI1	0.68399
P03363	O75381	0.18391
P03363	Q8TEX9	0.55047
P03363	Q8NFI3	0.22667
P03363	P17036	0.48218
P03363	P02766	0.22692
P03363	Q9BW11	0.41504
P03363	Q8TD86	0.39052
P03363	Q9NPE3	0.69429
P03363	Q13243	0.80333
P03363	Q9ULH7	0.83512
P03363	A6NMX2	0.26182
P03363	Q14320	0.17735
P03363	Q63HK3	0.36165
P03363	Q92870	0.59929
P03363	Q86VX9	0.12352
P03363	Q15004	0.65004
P03363	Q9NSP4	0.19999
P03363	P17861	0.56539

P03363	Q9HBG6	0.34362
P03363	Q4G112	0.51853
P03363	Q14919	0.42661
P03363	Q9H147	0.22504
P03363	Q9H211	0.56092
P03363	Q5VVH5	0.60207
P03363	Q9Y3M9	0.22259
P03363	Q8WVM7	0.5108
P03363	Q9P0M9	0.19909
P03363	P56537	0.98218
P03363	Q96RN5	0.94634
P03363	Q8N3R9	0.27855
P03363	Q8IYD8	0.46389
P03363	O60477	0.64338
P03363	O95931	0.72934
P03363	P78318	0.51032
P03363	Q8WXD5	0.82639
P03363	Q5MJ70	0.18365
P03363	Q86WG5	0.29059
P03363	Q9BYR8	0.42225
P03363	Q9Y2R9	0.19514
P03363	Q86UP8	0.34087
P03363	O96006	0.26902
P03363	Q86V81	0.82032
P03363	P54277	0.16968
P03363	Q8NFG4	0.74558
P03363	Q9BTW9	0.16995
P03363	Q9Y2N7	0.52726
P03363	P41134	0.36117
P03363	Q9BQA5	0.41287
P03363	Q99504	0.40695
P03363	P37837	0.11626
P03363	Q9UHV7	0.20989
P03363	P52744	0.24904
P03363	P22087	0.60885
P03363	P11171	0.46074
P03363	Q7Z3B3	0.3562
P03363	Q3KP66	0.090729
P03363	Q96KG9	0.34216
P03363	Q9UNS1	0.42135
P03363	Q96SK3	0.22259
P03363	P05413	0.4057
P03363	Q8IZ40	0.58877
P03363	Q2M218	0.22259
P03363	A0JLT2	0.37738
P03363	Q9UHQ7	0.071702
P03363	Q5SQN1	0.5492
P03363	O75586	0.50834
P03363	P10412	0.36021
P03363	P20265	0.60065
P03363	Q9H040	0.52834
P03363	Q9HAU5	0.76925
P03363	Q96C03	0.12094
P03363	Q15796	0.28988
P03363	O95983	1.0203
P03363	Q15723	1.0136
P03363	P63010	0.25055
P03363	P07910	0.71205
P03363	P31268	0.4288
P03363	Q96ST8	0.24408
P03363	Q9H9L3	0.51067
P03363	Q6XD76	0.39705
P03363	O75674	0.37445
P03363	Q15270	0.48609
P03363	O75935	0.53887
P03363	P57081	0.87845
P03363	Q9NWB1	0.69376
P03363	Q9UKL3	0.80242
P03363	Q8NAV1	0.32159
P03363	Q9Y3E7	0.54538
P03363	Q8IZU0	0.29721
P03363	Q5TAP6	0.049953
P03363	Q9NUW8	0.55833
P03363	Q9NQ48	0.48958
P03363	Q8N108	0.95791
P03363	Q9NPJ8	0.40588

P03363	Q08AG7	0.75438
P03363	P63220	0.36768
P03363	P57721	0.57544
P03363	P84022	0.16941
P03363	Q9BRQ6	0.37558
P03363	Q05084	0.30862
P03363	Q3SY00	0.031525
P03363	Q9HBM1	0.45189
P03363	Q5T7B8	0.47577
P03363	Q5UIP0	0.28073
P03363	A0AVK6	0.40803
P03363	Q9Y215	0.26418
P03363	Q7L5Y6	0.22504
P03363	Q15847	0.11203
P03363	Q01167	0.31333
P03363	Q7LBR1	0.71737
P03363	P17987	0.20985
P03363	Q7L0Y3	0.69278
P03363	Q96C00	0.25368
P03363	Q96KE9	0.26454
P03363	Q86WZ0	0.032462
P03363	Q8WXC3	0.64926
P03363	Q9H9E1	0.39383
P03363	A6NCL1	0.29088
P03363	P04406	0.25025
P03363	Q9NZU7	0.32263
P03363	P31314	0.75369
P03363	Q6PCB5	0.22504
P03363	Q8N5J4	0.62924
P03363	P14136	0.64676
P03363	O15265	0.79346
P03363	Q13620	0.58413
P03363	Q7L2H7	0.69123
P03363	Q9UKT8	0.59118
P03363	P98170	0.26333
P03363	Q7Z6R9	0.35245
P03363	Q7Z460	0.24055
P03363	Q9Y2L1	0.34087
P03363	Q9H6F5	0.32511
P03363	Q6U7Q0	0.40237
P03363	Q96PE6	0.22259
P03363	Q16656	0.4671
P03363	Q8N2M8	0.274
P03363	Q9Y4K1	0.095959
P03363	P41161	0.38908
P03363	Q6PEX3	0.27762
P03363	Q8N895	0.56235
P03363	Q5TAG4	0.24431
P03363	Q8N163	0.73695
P03363	O14613	0.25403
P03363	O75934	0.75964
P03363	Q92782	0.37674
P03363	Q9BQ83	0.39973
P03363	P49750	0.89851
P03363	P48634	0.92347
P03363	Q9Y6Z5	0.11203
P03363	O00204	0.36986
P03363	Q9BYR2	0.2934
P03363	Q9UKT4	0.42028
P03363	O43768	0.23577
P03363	O95163	0.58459
P03363	Q68CJ9	0.71234
P03363	Q5T619	0.22259
P03363	O94916	0.41489
P03363	Q8N3L3	0.70383
P03363	A8MVA2	0.2934
P03363	Q86UD0	0.49651
P03363	Q8TCY9	0.3478
P03363	Q9NZI7	0.42206
P03363	Q9BRH9	0.22259
P03363	Q6ICG8	0.071287
P03363	Q9HCC6	0.28032
P03363	Q8IVC4	0.22259
P03363	Q8WW01	0.32896
P03363	O95271	0.25867
P03363	P31942	0.41191

P03363	Q7RTT3	0.39008
P03363	Q7Z465	0.63937
P03363	Q9H1L0	0.00248
P03363	Q99619	0.75321
P03363	Q9UKY1	0.52619
P03363	Q8NI08	0.73807
P03363	P47928	0.49309
P03363	Q9Y3D9	0.34462
P03363	O75564	0.4261
P03363	P10244	0.5401
P03363	Q9BPX3	0.74209
P03363	Q8TF68	0.23246
P03363	Q9NZI5	0.4611
P03363	Q9BT92	0.52816
P03363	Q6ZRV2	0.073127
P03363	Q6ZN57	0.22259
P03363	O96004	0.34521
P03363	P61313	0.61631
P03363	Q9H1A4	0.41779
P03363	Q8IZW8	0.64715
P03363	Q96HA1	0.045852
P03363	Q9H2Z4	0.429
P03363	P26358	0.26093
P03363	Q5JR98	0.53508
P03363	A6NLJ0	0.28558
P03363	Q9UHD8	0.38882
P03363	P24522	0.48776
P03363	Q9Y618	0.75212
P03363	Q8IXF0	0.33708
P03363	Q92556	0.30691
P03363	Q8NBR6	0.11203
P03363	Q92499	0.42211
P03363	P49336	0.26361
P03363	Q9UI95	0.78445
P03363	Q00056	0.36024
P03363	P31948	0.80249
P03363	P39019	0.4863
P03363	Q96AY2	0.49253
P03363	P49792	0.38546
P03363	Q9H9Z2	0.57841
P03363	Q9BTX9	0.14001
P03363	Q3LI64	0.32806
P03363	Q5VVS0	0.070767
P03363	O43290	0.8287
P03363	Q8N841	0.388
P03363	Q96I34	0.27257
P03363	Q96N77	0.44662
P03363	Q14011	0.50279
P03363	Q9H788	0.90491
P03363	Q9NYZ3	0.30866
P03363	Q86SG2	0.37252
P03363	Q6ZUI0	0.24431
P03363	P35638	0.5441
P03363	Q86U86	0.80348
P03363	Q15542	0.63526
P03363	Q12929	0.27012
P03363	Q96PM5	0.47576
P03363	P13985	0.11203
P03363	Q92624	0.92878
P03363	Q15742	0.28433
P03363	Q9H0E2	0.34443
P03363	Q9Y3B6	0.22979
P03363	Q9H9L7	0.2977
P03363	Q6P1W5	0.53508
P03363	Q969H0	0.46091
P03363	Q12796	0.89393
P03363	Q9NX20	0.36981
P03363	O94805	0.45924
P03363	Q8TDY2	0.6987
P03363	Q9UIF7	0.19724
P03363	P10914	0.26559
P03363	P62256	0.31119
P03363	Q86UT5	0.24986
P03363	Q9C0C6	0.020717
P03363	O14929	0.62701
P03363	Q5QJ74	0.29593

P03363	Q674X7	0.47976
P03363	Q13573	0.59104
P03363	Q6QNY1	0.59359
P03363	Q6ZNG0	0.22259
P03363	Q8TCG1	0.21828
P03363	P52298	0.44819
P03363	Q96IR3	0.02369
P03363	P26196	0.53484
P03363	Q9NRN7	0.38717
P03363	Q2V2M9	0.42326
P03363	Q13838	0.43706
P03363	Q86XN6	0.22259
P03363	O60732	0.53508
P03363	Q9C035	0.4846
P03363	O43148	0.7064
P03363	Q96PQ7	0.27402
P03363	Q9Y2G7	0.22259
P03363	P35658	0.57718
P03363	O15143	0.26488
P03363	Q6ZN08	0.22259
P03363	Q6ZVN8	0.21794
P03363	P54840	0.10901
P03363	P54253	0.74876
P03363	Q6P9B9	0.32557
P03363	Q9UHX1	0.85121
P03363	Q96I76	0.17736
P03363	Q9BQY9	0.69103
P03363	Q14137	0.70594
P03363	A6NIY4	0.0070677
P03363	O95171	0.23254
P03363	Q92826	0.31355
P03363	Q86XG9	0.24431
P03363	O75167	0.43971
P03363	Q02040	0.54979
P03363	O00566	0.54688
P03363	Q96IT1	0.55174
P03363	O75323	0.25879
P03363	P57075	0.43577
P03363	Q9BT04	0.20462
P03363	Q92618	0.22259
P03363	Q9Y615	0.57453
P03363	Q96NS1	0.29721
P03363	O75153	0.24431
P03363	O00479	0.25389
P03363	P08621	0.7591
P03363	Q6ISB3	0.68056
P03363	Q9BYK8	0.26055
P03363	Q03188	0.21389
P03363	Q2NL82	0.30585
P03363	P55265	0.35208
P03363	Q5T200	0.42886
P03363	Q8NG31	0.31138
P03363	Q53G59	0.68046
P03363	O00470	0.64321
P03363	P46779	0.72485
P03363	Q6P2H3	0.597
P03363	Q96EK5	0.41206
P03363	Q96MY7	0.081768
P03363	Q9NSB2	0.77457
P03363	Q6IN85	0.48938
P03363	P49789	0.48829
P03363	Q08378	0.33305
P03363	Q14106	0.32633
P03363	Q9NPA8	0.26086
P03363	Q5TAB7	0.061488
P03363	Q5T653	0.21263
P03363	Q02543	0.42896
P03363	Q8WXU2	0.76997
P03363	P0CG34	0.26541
P03363	P46777	0.71398
P03363	Q05925	0.2677
P03363	Q9H840	0.82585
P03363	Q96G21	0.82618
P03363	Q9NRR4	0.30094
P03363	Q8TAA5	0.1978
P03363	Q15583	0.35787

P03363	Q9H9Q4	0.52549
P03363	Q9NYF8	0.92881
P03363	Q9BUK0	0.034826
P03363	Q06732	0.24904
P03363	P59047	0.261
P03363	P62987	0.049544
P03363	Q9UNS2	0.73971
P03363	Q3LI58	0.27762
P03363	Q5VWM6	0.10142
P03363	P57055	0.22179
P03363	P35398	0.34882
P03363	Q8WUW1	0.30174
P03363	Q9BQW3	0.17892
P03363	Q9Y2Z0	0.43495
P03363	Q13257	0.45897
P03363	Q9XRX5	0.53508
P03363	Q8N983	0.31668
P03363	Q9H0M5	0.22259
P03363	O75925	0.36781
P03363	P0C860	0.36704
P03363	A6NJ64	0.22504
P03363	Q86UU0	0.45758
P03363	Q14209	0.78201
P03363	Q9Y6P5	0.17036
P03363	Q7Z7H8	0.21901
P03363	O95243	0.58423
P03363	Q92600	0.87487
P03363	Q5QJE6	0.4684
P03363	Q5XKR4	0.27772
P03363	O95198	0.67026
P03363	Q8NCD3	0.53406
P03363	Q9Y283	0.50274
P03363	Q9H2J4	0.73078
P03363	P51114	0.25648
P03363	Q00059	0.56641
P03363	P43243	0.39446
P03363	Q14974	0.59164
P03363	Q92664	0.25777
P03363	Q8NEP9	0.22259
P03363	Q15173	0.39394
P03363	Q96S95	0.4239
P03363	Q96C74	0.54542
P03363	P17025	0.22259
P03363	P23528	0.4481
P03363	Q9BWT1	0.38642
P03363	Q969T4	0.14574
P03363	Q7Z3I7	0.22259
P03363	Q3LHN2	0.27762
P03363	P50549	0.80772
P03363	Q92966	0.49756
P03363	Q33E94	0.72595
P03363	Q9H582	0.22259
P03363	Q9UK13	0.22259
P03363	Q8WX93	0.57142
P03363	O95363	0.049142
P03363	Q9ULE6	0.77561
P03363	P55198	0.64433
P03363	P46108	0.34081
P03363	Q14592	0.22259
P03363	Q8ND90	0.75168
P03363	P15407	0.55222
P03363	Q9Y4F5	0.34776
P03363	Q96CX3	0.22259
P03363	Q9UPS6	0.2806
P03363	Q8WTW3	0.48225
P03363	Q8N0Z3	0.45369
P03363	P51508	0.24904
P03363	O60783	0.22724
P03363	Q6ZU52	0.53508
P03363	Q6WBX8	0.49219
P03363	P22415	0.41931
P03363	Q6ZWJ1	0.35374
P03363	Q8N184	0.22259
P03363	Q9H503	0.52533
P03363	P20290	0.935
P03363	P61758	0.70658

P03363	Q00287	0.20089
P03363	A6NFQ7	0.51853
P03363	O94868	0.53508
P03363	Q3KRA6	0.036373
P03363	Q9H8T0	0.36693
P03363	P63279	0.49811
P03363	Q9NVH2	0.41297
P03363	P54821	0.35308
P03363	Q96NK8	0.25557
P03363	Q6ZSB9	0.22259
P03363	Q9NVR7	0.23873
P03363	Q9H008	0.28402
P03363	Q86XZ4	0.24431
P03363	Q8N7K0	0.22259
P03363	A9YTQ3	0.47432
P03363	Q9P206	0.017352
P03363	P0CB33	0.22259
P03363	Q96EZ8	0.62402
P03363	Q9Y3T9	0.78887
P03363	Q9NVF7	0.0041725
P03363	Q9BUY5	0.22259
P03363	Q86XW9	0.1526
P03363	Q9H7R0	0.22259
P03363	Q9Y512	0.25803
P03363	Q9BV57	0.24146
P03363	Q7Z6G3	0.65694
P03363	Q9UKA1	0.43978
P03363	Q14591	0.22259
P03363	Q6UUV9	1.0224
P03363	Q6NZI2	0.61979
P03363	Q9HCE5	0.181
P03363	Q12905	0.85759
P03363	Q5TC12	0.4931
P03363	Q96LI5	0.39848
P03363	Q13547	0.35455
P03363	Q9BWK5	0.24431
P03363	O75807	0.37629
P03363	P60510	0.60177
P03363	Q86UQ0	0.25099
P03363	P23759	0.3282
P03363	Q14584	0.22259
P03363	Q9Y376	0.3912
P03363	Q9NXK8	0.71731
P03363	Q9NUQ6	0.49651
P03363	Q9Y2G2	0.54237
P03363	Q9BS34	0.22259
P03363	Q8NE22	0.0558
P03363	Q7Z4T9	0.72349
P03363	Q13868	0.54367
P03363	Q00994	0.59855
P03363	O43379	0.41589
P03363	Q8N8L2	0.22259
P03363	Q9NP87	0.16899
P03363	Q9BYQ2	0.2934
P03363	Q4G0Z9	0.027938
P03363	Q92750	0.54358
P03363	O43865	0.22837
P03363	Q96SL8	0.29641
P03363	Q9P2K5	0.25142
P03363	Q9ULV3	0.41329
P03363	Q9UM82	0.20119
P03363	Q9BY27	0.22504
P03363	P25398	0.44263
P03363	Q9BZE9	0.43501
P03363	Q9Y3Z3	0.29896
P03363	Q9BYR0	0.2934
P03363	O43542	0.62304
P03363	O75694	0.33849
P03363	Q14674	0.32319
P03363	Q9UDY2	0.70595
P03363	Q9H3F6	0.57454
P03363	Q96T37	0.46888
P03363	Q9Y4P8	0.69589
P03363	Q96NB1	0.33038
P03363	Q9NP92	0.33492
P03363	Q9NUX5	0.23566

P03363	Q8WYR4	0.46602
P03363	Q8NCU7	0.22504
P03363	Q9UKK6	0.77042
P03363	P49790	0.48128
P03363	Q96EH3	0.24308
P03363	Q96CS2	0.46589
P03363	Q9P270	0.63055
P03363	Q9BWD3	0.014834
P03363	O43396	0.26327
P03363	A8MUV8	0.22259
P03363	Q13884	0.53443
P03363	P08729	1.0179
P03363	Q9HB31	0.35028
P03363	B5ME19	0.25857
P03363	P78560	0.5775
P03363	Q9Y2A0	0.23115
P03363	Q8TCB0	0.2675
P03363	Q8TBY8	0.24431
P03363	Q9H8Y8	0.38615
P03363	P22626	0.84386
P03363	O00189	0.2517
P03363	P19838	0.39647
P03363	Q9BQ65	0.25994
P03363	Q96FZ2	0.01261
P03363	Q76L83	0.3602
P03363	Q16626	0.43971
P03363	Q8IX06	0.31703
P03363	Q7Z3K3	0.52918
P03363	Q86UN6	0.16256
P03363	P54257	0.27523
P03363	Q9Y6H1	0.034826
P03363	Q8TEV9	0.29721
P03363	P42025	0.54147
P03363	O95071	0.26628
P03363	Q7L9L4	0.64089
P03363	O76031	0.1314
P03363	Q5T0J7	0.18182
P03363	Q99814	0.36347
P03363	Q8WVZ9	0.56434
P03363	P09001	0.19838
P03363	Q71F23	0.37225
P03363	Q8IWL3	0.4839
P03363	Q6NSW7	0.51853
P03363	Q99871	0.3839
P03363	Q9Y5L0	0.91007
P03363	Q6ZW61	0.33499
P03363	A6NKU9	0.0070677
P03363	A1A5D9	0.014743
P03363	Q13409	0.45033
P03363	Q14129	0.16602
P03363	P82675	0.2119
P03363	P61572	0.55152
P03363	Q9HB19	0.56019
P03363	O14964	0.39384
P03363	P83876	0.78843
P03363	Q8N0Z6	0.47303
P03363	Q6PJP8	0.38887
P03363	Q6ZMW2	0.22259
P03363	Q8IY92	0.39952
P03363	Q93074	0.183
P03363	O00186	0.54342
P03363	O43345	0.22259
P03363	Q6RFH8	0.83979
P03363	P41219	0.57067
P03363	Q96SW2	0.61519
P03363	Q96S55	0.20318
P03363	Q9UI30	0.50862
P03363	A8MV65	0.40525
P03363	Q9UBS8	0.36751
P03363	P41220	0.26781
P03363	Q495M9	0.38263
P03363	Q8TAK5	0.44697
P03363	Q96SZ4	0.30749
P03363	Q86UQ8	0.40525
P03363	Q9UJA5	0.22076
P03363	P29966	0.066515

P03363	Q8IY22	0.35675
P03363	Q58A44	0.24431
P03363	Q96EY5	0.3759
P03363	Q86XK2	0.55714
P03363	Q02833	0.36286
P03363	P09016	0.37923
P03363	Q9Y692	0.56136
P03363	Q9BYG5	0.50038
P03363	P51808	0.30789
P03363	P53999	0.90494
P03363	Q6ZU80	0.34008
P03363	O00422	0.97467
P03363	Q29RF7	0.66352
P03363	Q92793	0.11303
P03363	Q86Y25	0.22259
P03363	P0C5W0	0.24431
P03363	Q9BTY7	0.11203
P03363	Q99618	0.48956
P03363	P05114	0.35468
P03363	Q5TAX3	0.50478
P03363	Q16633	0.49895
P03363	O60812	0.24924
P03363	A6NEY3	0.019317
P03363	Q9H4P4	0.25167
P03363	Q5JNZ5	0.21422
P03363	P50402	0.15913
P03363	B4DX44	0.22259
P03363	O76095	0.12847
P03363	P57071	0.22259
P03363	Q9Y5N5	0.53953
P03363	O75154	0.5706
P03363	O75971	0.50698
P03363	P82979	0.53751
P03363	Q9BRS8	0.35289
P03363	O15066	0.31899
P03363	Q15365	0.98789
P03363	P0CW71	0.15599
P03363	Q9C0H6	0.41814
P03363	P54725	0.52677
P03363	Q9BXU7	0.27877
P03363	Q8IV03	0.0803
P03363	O95295	0.30046
P03363	Q4LEZ3	0.033323
P03363	Q7Z614	0.50547
P03363	Q8N7A1	0.24431
P03363	Q15651	0.58732
P03363	Q9NS23	0.28667
P03363	Q6ZNG1	0.22259
P03363	Q8TF65	0.24431
P03363	Q01954	0.28329
P03363	Q8IWX8	0.21985
P03363	O95848	0.50863
P03363	Q15697	0.4482
P03363	Q8IUC0	0.27762
P03363	Q15185	0.47311
P03363	P31276	0.70409
P03363	Q16533	0.45861
P03363	Q9H1B7	0.12844
P03363	Q9NZD4	0.16129
P03363	A6NI87	0.0036666
P03363	D6RGH6	0.88438
P03363	P14317	0.28713
P03363	Q6ZN79	0.22259
P03363	Q9Y5Z4	0.23887
P03363	Q6ZPB5	0.14276
P03363	O95125	0.32797
P03363	Q9H1K1	0.6491
P03363	O14974	0.4007
P03363	Q68CQ4	0.17603
P03363	P61574	0.55152
P03363	O75448	0.17119
P03363	Q68CZ2	0.52193
P03363	P40855	0.21267
P03363	Q6ZN28	0.57825
P03363	P05060	0.1282
P03363	P26599	0.49801

P03363	Q8N823	0.22259
P03363	P07476	0.24146
P03363	Q9H0H0	0.23754
P03363	Q7RTT4	0.39008
P03363	Q3LI67	0.27762
P03363	O14530	0.50056
P03363	Q5XUX1	0.53508
P03363	Q96NL8	0.24431
P03363	O75382	0.34145
P03363	P62487	0.20921
P03363	P28289	0.27132
P03363	Q15386	0.54692
P03363	Q701N4	0.2934
P03363	Q9NUG6	0.19359
P03363	O60936	0.69707
P03363	Q6ZSS3	0.22259
P03363	Q96QH2	0.40274
P03363	Q9UPV0	1.0193
P03363	P82933	0.56481
P03363	Q8NC26	0.22259
P03363	P57740	0.4346
P03363	P50222	0.81345
P03363	P51668	0.36543
P03363	Q9NX53	0.53508
P03363	Q92614	0.45799
P03363	Q86V97	0.56434
P03363	Q9BRA2	0.62805
P03363	Q7Z7M0	0.25777
P03363	Q9UI56	0.11265
P03363	O60869	0.61801
P03363	Q9NRL3	0.56491
P03363	Q9BYG3	0.59314
P03363	Q0VAK6	0.27981
P03363	Q5M9Q1	0.10113
P03363	Q92748	0.5883
P03363	P60413	0.2934
P03363	Q96HA8	0.80846
P03363	Q6PI47	0.21694
P03363	Q9H3J6	0.078783
P03363	Q9UBE0	0.27007
P03363	P24385	0.25302
P03363	Q9UPQ9	0.13348
P03363	Q9UI32	0.22851
P03363	Q13469	0.8528
P03363	P49770	0.28599
P03363	Q9BXF6	0.51855
P03363	Q52M58	0.01229
P03363	Q9UBQ0	0.098776
P03363	Q9GZN1	0.29593
P03363	Q8WTW4	0.56352
P03363	Q99638	0.40053
P03363	Q15361	0.52843
P03363	P21675	0.20523
P03363	Q9NR48	0.23885
P03363	P60228	0.74873
P03363	Q13829	0.64246
P03363	Q14585	0.31124
P03363	O14646	0.75147
P03363	P33076	0.39518
P03363	Q9UQK1	0.39074
P03363	Q14CW9	0.49538
P03363	P52740	0.24904
P03363	Q92988	0.82126
P03363	Q5JPI3	0.044835
P03363	Q8IUD6	0.45207
P03363	Q14103	0.77746
P03363	Q6I9Y2	0.83256
P03363	Q13023	0.5311
P03363	Q9UIC8	0.4315
P03363	Q8IWI9	0.40835
P03363	P51523	0.27378
P03363	O96028	0.25839
P03363	Q5VIR6	0.45107
P03363	Q6FIF0	0.29766
P03363	P43357	0.53508
P03363	Q9BWX5	0.23548

P03363	Q8WVF5	0.21694
P03363	P23193	0.63941
P03363	Q9HBL0	0.64192
P03363	Q9UMR2	0.3566
P03363	Q14587	0.24904
P03363	Q9H4B6	0.58274
P03363	Q96M91	0.11203
P03363	Q14CZ0	0.14757
P03363	P83731	0.42896
P03363	P62851	0.47802
P03363	Q2TAK8	0.669
P03363	Q9P2J3	0.23328
P03363	P62280	0.32668
P03363	Q8IW40	0.14536
P03363	Q01201	0.96392
P03363	Q3LI54	0.27762
P03363	Q0PNE2	0.25736
P03363	P56270	0.57707
P03363	Q5T280	0.11203
P03363	O14862	0.38968
P03363	O95782	0.24736
P03363	Q969P5	0.45137
P03363	Q92466	0.61033
P03363	Q9BTX1	0.10384
P03363	Q9NPA5	0.22259
P03363	P35712	0.46009
P03363	Q9UKS6	0.37666
P03363	Q08379	0.39688
P03363	Q99990	0.41169
P03363	Q9NV56	0.32165
P03363	Q9H2G9	0.44931
P03363	Q01844	0.57819
P03363	Q8N9Y4	0.043615
P03363	O94829	0.97697
P03363	O43491	0.30002
P03363	O60393	0.44123
P03363	P42695	0.28166
P03363	Q3LI61	0.27762
P03363	Q96NG5	0.22755
P03363	O75496	0.7062
P03363	Q15628	0.58345
P03363	P55795	0.50613
P03363	Q96C92	0.24431
P03363	Q8N9R8	0.70558
P03363	Q8NEA9	0.48189
P03363	Q13569	0.41717
P03363	A6NIH7	0.35582
P03363	Q96PI1	0.20545
P03363	Q9Y5K8	0.21508
P03363	P0DM35	0.037977
P03363	Q13875	0.44887
P03363	Q9P2J5	0.44186
P03363	Q01534	0.4875
P03363	O00308	0.33065
P03363	Q9BV20	0.16722
P03363	Q9BUW7	0.11203
P03363	Q9BYG7	0.29721
P03363	Q92844	0.50744
P03363	Q9UQR0	0.41196
P03363	O60682	0.49092
P03363	O43711	0.57916
P03363	Q71UM5	0.51557
P03363	Q92665	0.68642
P03363	Q9HBM6	0.69595
P03363	Q9H583	0.81031
P03363	Q96BT3	0.39909
P03363	Q9BY60	0.34776
P03363	P59037	0.023125
P03363	A6NJ78	0.062282
P03363	P07438	0.15627
P03363	Q9Y651	0.40599
P03363	Q96LT7	0.46197
P03363	Q9Y3U8	0.50863
P03363	Q12923	0.31295
P03363	Q7RTU5	0.4492
P03363	Q00978	0.8348

P03363	P62888	0.47273
P03363	Q9P2K3	1.0151
P03363	Q13438	0.25746
P03363	Q8NI35	0.37896
P03363	Q7Z3Z0	1.0007
P03363	Q86UT8	0.070219
P03363	P10242	0.66836
P03363	Q96RS6	0.4314
P03363	Q8WV92	0.40542
P03363	P49458	0.16375
P03363	Q8N3J2	0.15445
P03363	O14531	0.25586
P03363	Q9UL18	0.87595
P03363	Q9H0I3	0.51115
P03363	P20226	0.59431
P03363	Q5THR3	0.34387
P03363	Q9UHJ3	0.43003
P03363	Q92681	0.24801
P03363	Q96F24	0.59284
P03363	Q9UM13	0.32272
P03363	O15040	0.53508
P03363	Q16526	0.46425
P03363	Q9H171	0.4182
P03363	Q8WUI4	0.22486
P03363	P49910	0.64392
P03363	Q6AZW8	0.22259
P03363	Q3B8N5	0.29628
P03363	O60239	0.4511
P03363	Q6ZP68	0.073689
P03363	Q9BVN2	0.4649
P03363	Q9UQ13	0.30233
P03363	P52655	0.70936
P03363	Q7Z3Y7	0.65474
P03363	Q8ND56	0.23589
P03363	Q9NV79	0.22121
P03363	Q9NY27	0.84618
P03363	Q5JU85	0.25286
P03363	Q5TB30	0.35941
P03363	P56945	0.35759
P03363	P17014	0.20871
P03363	Q9UPA5	0.30845
P03363	Q9NX58	0.17708
P03363	P41214	0.2808
P03363	Q9UK76	0.22504
P03363	Q96GE5	0.22259
P03363	Q5VWK0	0.24431
P03363	Q9Y2Y4	0.38119
P03363	Q9Y237	0.36637
P03363	Q96CG3	0.5243
P03363	Q04725	0.70207
P03363	Q7RTU4	0.43142
P03363	Q9BYS1	0.33409
P03363	Q8IWJ2	0.59425
P03363	P63241	0.34344
P03363	Q5H9L2	0.89393
P03363	O75362	0.62378
P03363	Q9BYQ0	0.2934
P03363	Q92670	0.27054
P03363	Q15013	0.88674
P03363	Q15172	0.4087
P03363	Q8IW35	1.0452
P03363	O75956	0.021196
P03363	Q5HYI7	0.023789
P03363	Q8TDN4	0.48562
P03363	Q8WVM0	0.4758
P03363	Q9UL58	0.268
P03363	P43351	0.63576
P03363	Q13485	0.29511
P03363	O75175	1.0107
P03363	Q9NXR7	0.4789
P03363	Q8NA19	0.30439
P03363	Q460N5	0.37207
P03363	Q9BTT4	0.49306
P03363	Q9NZQ3	0.69416
P03363	P12035	0.67214
P03363	Q96HI0	0.4342

12313	P03363	Q15072	0.42508
12314	P03363	Q8N726	0.33343
12315	P03363	P62310	0.76438
12316	P03363	Q7Z5V6	0.54021
12317	P03363	O95076	0.29537
12318	P03363	O95402	0.78581
12319	P03363	P10074	0.46642
12320	P03363	Q96RK0	0.8985
12321	P03363	Q92804	0.46805
12322	P03363	Q9UBW7	0.2386
12323	P03363	P60842	0.19996
12324	P03363	P27469	0.50459
12325	P03363	Q86XP3	0.55398
12326	P03363	Q92625	0.36338
12327	P03363	Q2NKJ3	0.42938
12328	P03363	Q6ZVQ6	0.14324
12329	P03363	O15126	0.28745
12330	P03363	Q9UIF3	0.32276
12331	P03363	Q9NQT5	0.31593
12332	P03363	Q9H0F6	0.21856
12333	P03363	O15182	0.38948
12334	P03363	P35606	0.34688
12335	P03363	Q12968	0.49693
12336	P03363	Q05195	0.53035
12337	P03363	P26651	0.50315
12338	P03363	Q9ULV5	0.61831
12339	P03363	P17482	0.8726
12340	P03363	O95865	0.40526
12341	P03363	Q9NPD8	0.16808
12342	P03363	P52951	0.24794
12343	P03363	P49335	0.35962
12344	P03363	Q9UIV1	0.9484
12345	P03363	O75969	0.33657
12346	P03363	Q8TD23	0.48719
12347	P03363	O60806	0.50965
12348	P03363	Q9H0A6	0.38916
12349	P03363	Q9Y6R6	0.22259
12350	P03363	Q86TB9	0.69351
12351	P03363	P51959	0.47101
12352	P03363	Q96RT7	0.45782
12353	P03363	O95714	0.31204
12354	P03363	Q76KD6	0.35651
12355	P03363	P23409	0.18783
12356	P03363	Q03468	0.30282
12357	P03363	Q96RL7	0.39162
12358	P03363	Q9UKM9	0.37106
12359	P03363	Q9Y4A5	0.51575
12360	P03363	Q8NEZ2	0.47197
12361	P03363	P50749	0.8152
12362	P03363	P63272	0.58817
12363	P03363	O15083	0.767
12364	P03363	Q9UJN7	0.22259
12365	P03363	P25685	0.39037
12366	P03363	Q9UJP4	0.36163
12367	P03363	Q969Q0	0.38572
12368	P03363	Q70YC4	0.06635
12369	P03363	O43402	0.40514
12370	P03363	O95319	0.38409
12371	P03363	Q9BW92	0.12031
12372	P03363	Q5VXH4	0.10142
12373	P03363	Q8WUH2	0.69578
12374	P03363	P10515	0.27213
12375	P03363	P50750	0.22939
12376	P03363	Q16890	0.51761
12377	P03363	Q6PI77	0.4314
12378	P03363	O00167	0.4342
12379	P03363	P78367	0.34532
12380	P03363	A6NI79	0.004542
12381	P03363	Q96GV9	0.62752
12382	P03363	Q08945	0.85312
12383	P03363	Q9NQE9	0.33537
12384	P03363	Q9GZZ1	0.60152
12385	P03363	P13196	0.18391
12386	P03363	O00570	0.8366
12387	P03363	Q14186	0.78593
12388	P03363	P98182	0.19314

P03363	P31269	0.75772
P03363	Q8IX12	0.89636
P03363	Q6ZS27	0.22259
P03363	Q63HK5	0.23928
P03363	Q6ZN01	0.62829
P03363	Q8TC76	0.47966
P03363	Q92616	0.31446
P03363	O15131	0.93497
P03363	Q5T8A7	0.6655
P03363	O60921	0.60678
P03363	Q9UBL0	0.41678
P03363	Q5TA31	0.48916
P03363	Q6PIJ6	0.4314
P03363	Q9NVM9	0.90094
P03363	Q15269	0.34251
P03363	O14662	0.45977
P03363	Q6ZQN3	0.074184
P03363	Q9Y5V3	0.5181
P03363	Q5HY98	0.22259
P03363	Q6IQ16	0.27676
P03363	P12980	0.69667
P03363	Q7Z6J0	0.39958
P03363	Q04837	0.53767
P03363	Q8IUZ0	0.34776
P03363	O60225	0.39008
P03363	Q6ISU1	0.14526
P03363	A8K8V0	0.22259
P03363	Q6P9F0	0.35262
P03363	Q09666	0.72229
P03363	O76042	0.10081
P03363	Q9Y676	0.20437
P03363	P19484	0.53828
P03363	Q86WH2	0.50767
P03363	Q9UQR1	0.68399
P03363	Q2QGD7	0.50181
P03363	Q9Y3B9	0.28674
P03363	Q99687	0.67172
P03363	P61981	0.5116
P03363	Q8TC94	0.29593
P03363	Q8IYN0	0.22259
P03363	O00401	0.41334
P03363	Q8IY42	0.06377
P03363	Q8TD26	0.34857
P03363	A6NK89	0.26133
P03363	Q15257	0.29122
P03363	Q68CL5	0.34776
P03363	P84550	0.26183
P03363	Q92541	0.76626
P03363	Q8TB69	0.22259
P03363	Q9H8Y1	0.08571
P03363	Q07820	0.25185
P03363	Q15599	0.45117
P03363	Q16385	0.84976
P03363	Q9NRI5	0.29758
P03363	Q9UH77	0.79491
P03363	Q8N0Z8	0.06171
P03363	Q96JL9	0.22259
P03363	Q70YC5	0.52064
P03363	Q96P15	0.18824
P03363	Q07955	0.76673
P03363	O00625	0.45101
P03363	Q9C056	0.23338
P03363	O43159	0.61258
P03363	Q8TAD8	1.0852
P03363	Q2VPB7	0.60824
P03363	Q6P3S1	0.22564
P03363	P53804	0.46
P03363	Q9NYT6	0.22259
P03363	O95551	0.51829
P03363	Q96JJ3	0.40838
P03363	Q13114	0.24073
P03363	Q13136	0.55314
P03363	O43660	0.68062
P03363	Q5BJF2	0.22246
P03363	Q9BYQ3	0.2934
P03363	Q8IZQ1	0.55659

P03363	O14737	0.45851
P03363	Q9UIL1	0.57437
P03363	Q5VTL8	0.32159
P03363	P17020	0.22259
P03363	Q96NW4	0.28324
P03363	Q9HCI5	0.62782
P03363	P63261	0.20454
P03363	P07315	0.75368
P03363	Q8N5Q1	0.062103
P03363	O43463	0.46679
P03363	Q8WWW0	0.54249
P03363	P49591	0.089672
P03363	Q14188	0.51268
P03363	Q92733	0.70925
P03363	O95336	0.22961
P03363	P62244	0.38923
P03363	Q8WUJ3	0.24431
P03363	Q8WV07	0.11203
P03363	P51816	0.18328
P03363	Q9UGJ1	0.40977
P03363	P62277	0.6463
P03363	O75531	0.7423
P03363	O60506	0.71028
P03363	Q9NPJ6	0.51717
P03363	Q3MIS6	0.22259
P03363	Q9Y483	0.23888
P03363	Q8N3X1	0.29721
P03363	Q9BR84	0.22259
P03363	P60660	0.34508
P03363	Q9H6Z4	0.98955
P03363	Q9HCM9	0.39171
P03363	Q6TFL4	0.099712
P03363	P14672	0.29355
P03363	A5PLL1	0.4314
P03363	O94901	0.29825
P03363	P15884	0.56657
P03363	P0CI01	0.0070677
P03363	O43583	0.5384
P03363	P62906	0.32344
P03363	P41162	0.55069
P03363	Q96E39	0.28697
P03363	Q5T9G4	0.03308
P03363	Q92540	0.78922
P03363	O75398	0.30749
P03363	Q01081	0.55564
P03363	Q8TF47	0.13014
P03363	O94842	0.71614
P03363	P56211	0.20098
P03363	Q15025	0.54917
P03363	Q9NQY0	0.52246
P03363	Q5VZF2	0.36191
P03363	O95644	0.69067
P03363	Q8N635	0.28771
P03363	A6NP11	0.22259
P03363	Q7Z5H3	0.26886
P03363	Q8TCJ0	0.57351
P03363	Q9Y285	0.43016
P03363	Q14671	0.34392
P03363	Q3SY52	0.22259
P03363	Q96A72	0.31301
P03363	A6NIX2	0.68819
P03363	Q9BTE1	0.35815
P03363	P78371	0.59894
P03363	Q8N954	0.18106
P03363	Q7Z5W3	0.26003
P03363	P25440	0.50012
P03363	Q9UMS6	0.73011
P03363	Q96T88	0.27894
P03363	Q5BKX8	0.29913
P03363	O60294	0.50547
P03363	Q9C0F1	0.40866
P03363	A6NJR5	0.0070677
P03363	O43361	0.22259
P03363	A6NLW8	0.49158
P03363	Q96NA2	0.13367
P03363	Q8IUR6	0.9194

P03363	Q9UK45	0.74542
P03363	Q8NFI4	0.24431
P03363	Q96CA5	0.34766
P03363	Q969H6	0.67389
P03363	Q8NE71	0.38278
P03363	Q9NYL9	0.24205
P03363	Q9H7X3	0.22259
P03363	Q9UKJ5	0.28361
P03363	Q9BSG1	0.22259
P03363	Q9P267	0.225
P03363	Q9H2L5	0.44261
P03363	Q09FC8	0.40375
P03363	Q9UK12	0.22259
P03363	O60810	0.10142
P03363	Q9BQY4	0.99479
P03363	Q86UL8	0.26618
P03363	Q9H0A9	0.14142
P03363	Q9UBF2	0.31552
P03363	Q15714	0.75131
P03363	Q8IVG9	0.28675
P03363	Q96SN7	0.15709
P03363	P35232	0.40944
P03363	Q8IYX8	0.35721
P03363	Q15973	0.22259
P03363	P62266	0.59224
P03363	Q9BYX4	0.24806
P03363	Q9BZI1	0.48292
P03363	Q96QS3	0.22216
P03363	Q9BVP2	0.36688
P03363	P78406	0.44082
P03363	Q9BZI7	0.62634
P03363	Q86UB2	0.4314
P03363	O94993	0.4347
P03363	P61978	0.86794
P03363	Q86XF7	0.22259
P03363	P62945	0.42896
P03363	Q8IUE0	0.51853
P03363	P05412	0.31618
P03363	Q96MN9	0.56241
P03363	Q3LI73	0.27762
P03363	Q8N612	0.49078
P03363	Q6IQ32	0.44194
P03363	Q96PV6	0.53508
P03363	Q9Y536	0.21453
P03363	Q6PD74	0.33766
P03363	P17017	0.22259
P03363	Q2VWA4	0.41305
P03363	A6NLX3	0.010789
P03363	Q9H3R5	0.37269
P03363	Q9P0R6	0.24431
P03363	Q9NTI5	0.64019
P03363	Q92834	0.43627
P03363	Q96K58	0.22259
P03363	P40424	0.55274
P03363	Q9BZL4	0.7338
P03363	Q9H1Q7	0.031479
P03363	Q15054	0.31633
P03363	Q96AV8	0.42523
P03363	Q96G23	0.32208
P03363	O94906	0.44142
P03363	Q9BT49	0.26761
P03363	Q9H116	0.38321
P03363	Q9BRX2	0.25364
P03363	P53992	0.57256
P03363	P78345	0.69177
P03363	O14618	0.30554
P03363	Q96DX5	0.91138
P03363	Q969M7	0.20987
P03363	O60304	0.268
P03363	Q9Y5Y2	0.44657
P03363	Q8TAU3	0.22259
P03363	O15055	0.38336
P03363	Q8IWT3	0.24371
P03363	P0CAP2	0.1315
P03363	Q9Y4L5	0.52237
P03363	Q96AJ1	0.71827

P03363	Q9H3C7	0.16934
P03363	P49207	0.36325
P03363	Q7Z401	0.41049
P03363	C9JBD0	0.24352
P03363	P62241	0.41763
P03363	P78386	0.8697
P03363	Q9HCG8	0.55843
P03363	Q04741	0.2805
P03363	Q5D1E8	0.37769
P03363	Q969L4	0.6894
P03363	A6NCI8	0.039743
P03363	Q13247	0.37397
P03363	Q9BU61	0.58137
P03363	B1AK76	0.01759
P03363	A2RU54	0.2553
P03363	Q5H9K5	0.14309
P03363	Q15428	0.1968
P03363	O75843	0.40738
P03363	Q8IWN7	0.42615
P03363	Q9BVS5	0.43988
P03363	Q00613	0.74975
P03363	Q86W56	0.4782
P03363	Q6PJG6	0.66092
P03363	Q13398	0.22259
P03363	Q9UKT9	0.5062
P03363	Q96LM6	0.22504
P03363	Q53GI3	0.268
P03363	Q9UMX2	0.25995
P03363	Q9NRR3	0.57601
P03363	Q13642	0.31863
P03363	O75386	0.24483
P03363	O15405	0.73803
P03363	P04733	0.15112
P03363	Q9Y3E5	0.41303
P03363	Q96J94	0.3644
P03363	Q8WWH5	0.10262
P03363	Q7Z4G4	0.20167
P03363	Q96CJ1	0.79963
P03363	Q5SXM2	0.55321
P03363	Q7Z2F6	0.24352
P03363	O60675	0.53218
P03363	Q86VP6	0.72009
P03363	O75771	0.35575
P03363	Q8WV22	0.29586
P03363	Q86UD4	0.22259
P03363	Q14934	0.43984
P03363	Q2NL98	0.24431
P03363	Q12873	0.48465
P03363	B2RXF5	0.48261
P03363	Q7L590	0.71305
P03363	Q9C002	0.21533
P03363	Q9UKF7	0.28709
P03363	P80723	0.37714
P03363	O43900	0.30217
P03363	Q9H6R4	0.16652
P03363	Q9HAJ7	0.35822
P03363	Q8WU39	0.19888
P03363	Q8IYL3	0.22504
P03363	P05455	0.23785
P03363	Q9BQC6	0.31668
P03363	P42568	0.75154
P03363	P62424	0.61255
P03363	P28370	0.42638
P03363	Q147U1	0.22259
P03363	P0C7M4	0.55275
P03363	Q9NR11	0.22259
P03363	P0C7H8	0.2934
P03363	O60930	0.2671
P03363	Q96G27	0.099585
P03363	Q9BZW7	0.51788
P03363	Q99581	0.23127
P03363	Q9UI26	0.51245
P03363	P58317	0.22259
P03363	O95359	0.3305
P03363	O43516	0.46209
P03363	Q04864	0.92162

P03363	Q6ZU15	0.46585
P03363	O95886	0.20686
P03363	O60524	0.47529
P03363	Q9H3K2	0.19865
P03363	P19532	0.62113
P03363	Q9BY42	0.11203
P03363	Q6DN90	0.24257
P03363	O95487	0.42611
P03363	Q8WVB3	0.27247
P03363	Q99816	0.23686
P03363	Q9BZL1	0.26643
P03363	Q9UFM2	0.22504
P03363	Q9NUQ3	0.48883
P03363	P55769	0.81889
P03363	P67870	0.22932
P03363	Q15058	0.63857
P03363	O43325	0.21533
P03363	O95373	0.7861
P03363	P19013	0.75224
P03363	Q5T7W7	0.11203
P03363	Q9UKD2	0.22133
P03363	Q9UJW3	0.56806
P03363	Q9H568	0.29593
P03363	Q9H511	0.22927
P03363	Q15059	0.90513
P03363	Q9Y6F6	0.23726
P03363	P52948	0.47957
P03363	Q5SZQ8	0.28629
P03363	Q06609	0.3721
P03363	O14519	0.4024
P03363	Q13596	0.3043
P03363	Q9HB96	0.74351
P03363	Q6P1N0	0.52113
P03363	Q8WVK2	0.41806
P03363	Q8N7F7	0.24431
P03363	Q8N3U4	0.43798
P03363	Q6AW86	0.22259
P03363	Q9UKL0	0.83738
P03363	Q9C004	0.23788
P03363	Q8IYX0	0.22259
P03363	Q7L3T8	0.071322
P03363	O60244	0.20989
P03363	Q8IZE3	0.2968
P03363	Q6VN20	0.43002
P03363	Q9ULC4	0.44183
P03363	O14777	0.34744
P03363	O75427	0.58649
P03363	Q9NZP6	0.024881
P03363	Q9NQR1	0.50815
P03363	Q5VZB9	0.26821
P03363	O96000	0.30838
P03363	Q9H213	0.23743
P03363	Q9Y296	0.27876
P03363	Q96IU2	0.096262
P03363	Q6L8G4	0.2934
P03363	Q27J81	0.27268
P03363	P0CG12	0.22504
P03363	Q9ULJ6	0.39728
P03363	P47974	0.49687
P03363	Q8TEA8	0.24584
P03363	Q5JRK9	0.085053
P03363	Q86XR8	0.76681
P03363	Q9NTG7	0.26064
P03363	P60866	0.42203
P03363	Q04917	0.32515
P03363	P61579	0.55152
P03363	A6NDE4	0.51018
P03363	Q6P280	0.22259
P03363	O60759	0.55729
P03363	O15127	0.49033
P03363	Q8NDP4	0.22259
P03363	Q96GX9	0.34533
P03363	O15259	0.27142
P03363	Q8NFJ8	0.34062
P03363	Q9Y262	0.72494
P03363	Q9Y244	0.27538

P03363	P61247	0.71156
P03363	Q14739	0.22881
P03363	O43745	0.68315
P03363	Q6NVH7	0.58111
P03363	P24534	0.55237
P03363	P61077	0.23776
P03363	Q9BYD9	0.45837
P03363	Q8IWV8	0.21751
P03363	Q96Q89	0.33378
P03363	Q9H446	0.53508
P03363	Q9UKY7	0.30665
P03363	Q5HYJ3	0.26782
P03363	O95997	0.66287
P03363	O75486	0.2853
P03363	Q7L945	0.22259
P03363	Q9H204	0.98273
P03363	O60573	0.58503
P03363	Q96CM3	0.44973
P03363	P55210	0.23354
P03363	Q9NVW2	0.37543
P03363	Q12789	0.69239
P03363	Q5EBM4	0.22259
P03363	O75146	0.32732
P03363	Q8TEQ6	0.74111
P03363	Q9UHN1	0.31143
P03363	Q9Y6B2	0.90497
P03363	Q16612	0.38228
P03363	Q9Y4P1	0.37377
P03363	Q9NQ92	0.77935
P03363	O14776	0.90325
P03363	Q9Y224	1.1094
P03363	Q96QG7	0.52966
P03363	O60333	0.29903
P03363	Q14157	0.45007
P03363	Q5HYW3	0.011573
P03363	Q5T3J3	0.83137
P03363	Q4VC44	0.40225
P03363	Q9H0E3	0.39856
P03363	Q13144	0.39774
P03363	Q9BWW4	0.37873
P03363	P58005	0.20341
P03363	Q9NQZ3	0.49367
P03363	O43918	0.56516
P03363	Q9Y3A2	0.76005
P03363	Q96DN6	0.28998
P03363	Q9UNA4	0.37734
P03363	P61956	0.69241
P03363	P09629	0.92482
P03363	Q8TC21	0.22259
P03363	Q3BBW0	0.24431
P03363	A8MXY4	0.22259
P03363	Q9H8W4	0.31671
P03363	Q93034	0.55543
P03363	Q9P1A6	0.26594
P03363	Q7L2E3	0.50781
P03363	Q96HQ2	0.0038652
P03363	O00165	0.54533
P03363	P55895	0.26818
P03363	P16989	0.55082
P03363	Q9P015	0.3187
P03363	Q5VWI1	0.031634
P03363	Q8NB50	0.18593
P03363	Q9NVP1	0.40607
P03363	Q96EN9	0.53508
P03363	Q9BQI9	0.51838
P03363	Q9BZG8	0.39566
P03363	Q9UFF9	0.5574
P03363	Q15031	0.062031
P03363	Q2M3C7	0.70423
P03363	Q52M93	0.18593
P03363	O14508	0.39251
P03363	Q9Y3D3	0.6142
P03363	P02489	0.29342
P03363	Q00839	0.81952
P03363	Q15573	0.43222
P03363	Q8N9K5	0.22259

12845	P03363	O43617	0.28542
12846	P03363	Q9Y543	0.36143
12847	P03363	Q96LR2	0.21483
12848	P03363	Q8N729	0.079775
12849	P03363	P61962	0.83225
12850	P03363	Q12834	0.45194
12851	P03363	P55197	0.97102
12852	P03363	P40426	0.41162
12853	P03363	Q9UHA3	0.32292
12854	P03363	Q9Y6Z2	0.11203
12855	P03363	Q66K41	0.033479
12856	P03363	Q14008	0.58936
12857	P03363	Q96N95	0.43055
12858	P03363	P09874	0.34208
12859	P03363	P35452	0.26023
12860	P03363	Q9Y664	0.1993
12861	P03363	Q13084	0.90434
12862	P03363	Q96FT9	0.29611
12863	P03363	Q13330	0.49663
12864	P03363	P62308	0.68761
12865	P03363	Q8TDH9	0.3085
12866	P03363	Q9NQ31	0.69149
12867	P03363	Q8WXB4	0.44662
12868	P03363	Q96SQ7	0.26493
12869	P03363	Q9NP71	0.30256
12870	P03363	Q9NRJ4	0.4612
12871	P03363	Q96A49	0.22504
12872	P03363	O95343	0.3194
12873	P03363	Q7Z4G1	0.88629
12874	P03363	Q9UQN3	0.3447
12875	P03363	Q14687	0.53508
12876	P03363	Q02548	0.44099
12877	P03363	Q8IYA7	0.37366
12878	P03363	Q9UJM3	0.39471
12879	P03363	P78316	0.17411
12880	P03363	P52741	0.30118
12881	P03363	O14972	0.22067
12882	P03363	Q6PK04	0.04946
12883	P03363	P62899	0.36325
12884	P03363	P41970	0.74566
12885	P03363	Q9NR33	0.42841
12886	P03363	Q8N699	0.22504
12887	P03363	Q9P013	0.75599
12888	P03363	Q16637	0.79874
12889	P03363	Q9GZU7	0.43159
12890	P03363	Q8WTR7	0.626
12891	P03363	Q14207	0.65126
12892	P03363	Q12765	0.36006
12893	P03363	Q9HCG1	0.22314
12894	P03363	Q9UKF6	0.50214
12895	P03363	Q96MG7	0.76997
12896	P03363	Q86XI8	0.53508
12897	P03363	Q5VWP3	0.29948
12898	P03363	Q9Y2Y0	0.73892
12899	P03363	Q8NHY2	0.77911
12900	P03363	P62857	0.29099
12901	P03363	Q9Y3A3	0.52622
12902	P03363	Q9NZD8	0.50271
12903	P03363	O14490	0.28911
12904	P03363	P13646	0.92986
12905	P03363	Q9NS25	0.41309
12906	P03363	P24855	0.19997
12907	P03363	Q658L1	0.04776
12908	P03363	Q13363	0.29899
12909	P03363	Q9Y2Q1	0.22259
12910	P03363	Q8WW38	0.39042
12911	P03363	Q9Y534	0.75432
12912	P03363	Q86SE8	0.21962
12913	P03363	Q9Y448	0.67547
12914	P03363	P84103	0.74707
12915	P03363	Q5T3I0	0.18106
12916	P03363	Q06587	0.57159
12917	P03363	Q9GZR1	0.4395
12918	P03363	Q8IVM0	0.7338
12919	P03363	Q9P086	0.82851
12920	P03363	Q99728	0.34661

P03363	Q3BBV0	0.24431
P03363	O43482	0.39489
P03363	Q499Z4	0.22259
P03363	O15504	0.23055
P03363	Q99700	0.59003
P03363	O95273	0.93062
P03363	Q9P278	0.67696
P03363	Q13404	0.7045
P03363	Q96FF9	0.57945
P03363	Q9BY31	0.22259
P03363	Q9UMS4	0.77227
P03363	P30047	0.46806
P03363	Q8NA72	0.43147
P03363	O94822	0.32272
P03363	Q2TAL8	0.11203
P03363	Q8NFD5	0.67729
P03363	Q96NJ3	0.22259
P03363	Q14498	0.85175
P03363	O95416	0.52849
P03363	Q96S59	0.81715
P03363	Q9HAT8	0.60875
P03363	Q8NFZ5	0.68494
P03363	P42773	0.31133
P03363	A7MD48	0.19954
P03363	Q99614	0.45209
P03363	Q13216	0.37994
P03363	O75478	0.38437
P03363	P16383	0.44678
P03363	Q68DY9	0.22259
P03363	Q02878	0.49207
P03363	P52743	0.24904
P03363	Q9BV47	0.37643
P03363	Q8TAW3	0.22259
P03363	Q9UBG7	0.24729
P03363	Q96AC1	0.30391
P03363	Q9H910	0.28624
P03363	Q9BXT5	0.084222
P03363	Q9NPI8	0.73918
P03363	Q96GM5	0.59296
P03363	O43364	0.29966
P03363	Q9ULK4	0.82362
P03363	Q9H7P6	0.011299
P03363	Q03828	0.44461
P03363	O60662	0.27871
P03363	Q8TEC5	0.29112
P03363	A6NHP3	0.0070677
P03363	Q9H1H9	0.38731
P03363	P08047	0.39108
P03363	Q53TQ3	0.45161
P03363	P52594	0.40715
P03363	Q5JSZ5	0.53508
P03363	O60248	0.69944
P03363	O76015	0.88908
P03363	O15084	0.74904
P03363	Q8N4C6	0.4239
P03363	Q96ES7	0.23155
P03363	Q8IVF1	0.019164
P03363	Q9HCH5	0.42242
P03363	Q6ZW49	0.351
P03363	Q96KN1	0.57804
P03363	Q8WTZ3	0.3286
P03363	P16452	0.32473
P03363	Q9Y2X8	0.2284
P03363	P78346	0.2171
P03363	Q9NQ29	0.08554
P03363	P82909	0.32206
P03363	Q9UMQ3	0.72461
P03363	Q92796	0.3526
P03363	Q99633	0.35202
P03363	P62837	0.43505
P03363	Q9NQP4	0.75596
P03363	Q9Y6X3	0.71418
P03363	Q9NPP4	0.23566
P03363	Q68E01	0.57064
P03363	P31483	0.63144
P03363	Q8N8E3	0.38184

P03363	Q9HB09	0.044835
P03363	O76041	0.38624
P03363	Q5TEC3	0.23615
P03363	O14512	0.57066
P03363	O15297	0.38496
P03363	Q7Z6J9	0.31286
P03363	Q86Y26	0.4314
P03363	P11802	0.32709
P03363	A8MVS1	0.22259
P03363	Q8IYX7	0.053015
P03363	P46013	0.27959
P03363	Q9H2U1	0.55144
P03363	H3BNL8	0.037194
P03363	O00472	0.43114
P03363	Q9NVD7	0.32871
P03363	Q9UKJ3	0.32728
P03363	Q9NV88	0.66095
P03363	Q6IS14	0.26226
P03363	Q9HB07	0.25151
P03363	Q9UI46	0.2645
P03363	Q9H8S9	0.45
P03363	Q5HYK7	0.79351
P03363	Q53GT1	0.19518
P03363	Q96PT3	0.52563
P03363	Q96FN9	0.25667
P03363	Q6P2P2	0.23591
P03363	Q6PCE3	0.093399
P03363	Q99081	0.45032
P03363	Q969U6	0.47011
P03363	Q6P1L5	0.12937
P03363	Q9H0M0	0.24824
P03363	O00192	0.42505
P03363	Q9HC78	0.31813
P03363	Q8WXE0	0.26643
P03363	Q8N5R6	0.53508
P03363	O00161	0.58595
P03363	Q9NY43	0.4782
P03363	Q9BXH1	0.23261
P03363	Q8TAU0	0.26483
P03363	Q96M11	0.41674
P03363	P55060	0.85184
P03363	Q9NX63	0.71974
P03363	P51513	0.27739
P03363	Q9UKT7	0.61675
P03363	P12036	0.42252
P03363	P82094	1.054
P03363	Q2M3X9	0.22259
P03363	P15622	0.22259
P03363	O43156	0.53508
P03363	Q13541	0.62233
P03363	Q9NP97	0.72876
P03363	O75821	0.70424
P03363	Q1W6H9	0.36026
P03363	Q0VG06	0.56075
P03363	A4GXA9	0.2719
P03363	Q86YW9	0.35717
P03363	O43663	0.68937
P03363	Q86YF9	0.37882
P03363	Q9H501	0.51136
P03363	Q96N03	0.53508
P03363	P51116	0.75943
P03363	Q9H009	0.52433
P03363	Q7Z6B0	0.29865
P03363	Q562F6	0.48426
P03363	Q8IX03	0.7082
P03363	Q9NP79	0.30348
P03363	P22674	0.29314
P03363	Q9H6Y2	0.49095
P03363	Q8N5C8	0.21456
P03363	O75152	0.42886
P03363	O60290	0.30921
P03363	Q9UKN8	0.45793
P03363	Q9NS69	0.19479
P03363	Q93083	0.15627
P03363	Q6NVV1	0.32513
P03363	Q15560	0.71459

P03363	Q6W2J9	0.40674
P03363	Q86VW0	0.35565
P03363	Q5T1J5	0.034826
P03363	Q9H2C0	0.80731
P03363	Q8WZ19	0.23565
P03363	Q9NRY5	0.065007
P03363	Q6KB66	0.42225
P03363	Q96KR7	0.14324
P03363	Q14676	0.54093
P03363	Q9Y6D6	0.44932
P03363	Q8IWS0	0.22834
P03363	O43390	0.91192
P03363	Q9ULM3	0.79052
P03363	P07814	0.30997
P03363	Q9BU02	0.19179
P03363	P78344	0.44614
P03363	Q9NPA3	0.34242
P03363	Q9BQ13	0.21694
P03363	Q9BUZ4	0.24901
P03363	Q9BTP6	0.084117
P03363	P48380	0.37128
P03363	Q9H6J7	0.29721
P03363	Q96KR1	0.58479
P03363	Q9Y238	0.23377
P03363	O75626	0.36786
P03363	O00716	0.72482
P03363	Q03701	0.49774
P03363	Q8NFJ9	0.1645
P03363	Q01546	0.75224
P03363	P0C7X2	0.23615
P03363	Q13761	0.70976
P03363	Q9NZT2	0.26513
P03363	P52742	0.26255
P03363	Q07020	0.47273
P03363	Q6ZRF7	0.22259
P03363	Q9NXR1	0.26162
P03363	P0C6E6	0.21422
P03363	Q9BYR4	0.2934
P03363	Q8N6W0	0.36052
P03363	Q9Y6Y1	0.62874
P03363	Q9H9D4	0.60224
P03363	Q6P3V2	0.18593
P03363	Q9Y248	0.63593
P03363	Q2M3W8	0.22259
P03363	Q5MCW4	0.22259
P03363	Q9NXB0	0.524
P03363	Q7Z7J5	0.54625
P03363	P39023	0.57487
P03363	Q15935	0.43658
P03363	Q8IUC2	0.27762
P03363	Q96PU4	0.38047
P03363	O95521	0.10142
P03363	Q96EI5	0.40525
P03363	Q96NY9	0.41488
P03363	Q8WXF1	0.9479
P03363	A6NFI3	0.22259
P03363	Q9Y6R0	0.34162
P03363	O43298	0.22259
P03363	Q01105	0.54131
P03363	Q9UKC9	0.52838
P03363	Q9BWM5	0.22259
P03363	Q9C0D2	0.38184
P03363	O14604	0.25193
P03363	O43261	0.53508
P03363	P82932	0.39573
P03363	P61581	0.22504
P03363	P62995	0.51319
P03363	Q9NUP9	0.21936
P03363	Q14493	0.75879
P03363	Q14141	0.24031
P03363	Q96TA1	0.49024
P03363	Q8TDM0	0.24431
P03363	O95429	0.22725
P03363	Q8WTP9	0.085053
P03363	Q5CZC0	0.098446
P03363	Q9NQ39	0.23375

P03363	P23258	0.47001
P03363	O00139	0.33072
P03363	Q9NPC6	0.49541
P03363	P0CL83	0.22504
P03363	Q9BQ48	0.19909
P03363	P37198	0.47683
P03363	Q86TS9	0.31668
P03363	Q96A00	0.19151
P03363	Q8WVP5	0.072815
P03363	Q93073	0.054582
P03363	O43829	0.67709
P03363	Q8N1W2	0.22259
P03363	Q01658	0.67537
P03363	Q96C86	0.51308
P03363	O43255	0.44265
P03363	Q8TE76	0.25603
P03363	Q9BQI5	0.21856
P03363	Q9UBD5	0.51986
P03363	Q9BWU0	0.72594
P03363	O60284	0.27705
P03363	Q107X0	0.4314
P03363	O15225	0.11203
P03363	P51531	0.41063
P03363	Q9NZM4	0.53508
P03363	Q6L8H4	0.2934
P03363	O95475	0.40942
P03363	Q5XUX0	0.062023
P03363	P30153	0.31477
P03363	Q9BR61	0.35778
P03363	Q9Y4B6	0.93214
P03363	Q8N8J6	0.22259
P03363	P60410	0.2934
P03363	Q8N587	0.22259
P03363	Q8TF46	0.33423
P03363	P18754	0.72173
P03363	Q92922	0.66176
P03363	P14678	0.76728
P03363	P43355	0.92943
P03363	Q96JB8	0.22798
P03363	Q9H329	0.3437
P03363	Q8WTS6	0.44832
P03363	Q9NQV6	0.22259
P03363	P43359	0.11203
P03363	Q96L03	0.27393
P03363	Q86WJ1	0.48182
P03363	Q16560	0.24492
P03363	Q99719	0.24477
P03363	Q96QA6	0.29721
P03363	Q9NWS1	0.92718
P03363	O14556	0.30488
P03363	Q9P219	0.50754
P03363	Q15543	0.74835
P03363	O60299	0.3514
P03363	Q12980	0.11203
P03363	Q9UL36	0.23315
P03363	Q8N1N4	0.77889
P03363	P53803	0.098796
P03363	P17544	0.80704
P03363	Q9P2R6	0.64952
P03363	P52435	0.49287
P03363	Q9UBZ9	0.40573
P03363	Q00577	0.51258
P03363	Q9NWK9	0.35256
P03363	P04264	0.50788
P03363	Q6UUV7	0.64653
P03363	Q6NZ67	0.78554
P03363	Q8IYI8	0.22259
P03363	P30307	0.3953
P03363	Q96EY1	0.23213
P03363	P36639	0.22095
P03363	P09630	0.39137
P03363	P48553	0.25092
P03363	Q96D98	0.22782
P03363	P49006	0.078291
P03363	P17040	0.38048
P03363	Q13287	0.6671

P03363	O94892	0.22259
P03363	Q8TBC3	0.21694
P03363	Q7Z5K2	0.52565
P03363	Q2TAZ0	0.53508
P03363	Q9H8H0	0.29721
P03363	Q0VGE8	0.22259
P03363	Q9HCE3	0.22259
P03363	O60828	0.67652
P03363	Q6ZS17	0.24431
P03363	Q49MI3	0.3087
P03363	Q9NZJ0	0.66204
P03363	Q8N3F8	0.52874
P03363	Q15572	0.45238
P03363	Q676U5	0.69217
P03363	Q96BR9	0.23615
P03363	Q8N2W9	0.7911
P03363	P30281	0.65084
P03363	Q2KHM9	0.3171
P03363	O75943	0.27622
P03363	Q99811	0.30564
P03363	Q05481	0.24904
P03363	Q9P0W2	0.65997
P03363	P26371	0.32636
P03363	P62854	0.54204
P03363	P35573	0.28017
P03363	Q96CB8	0.4254
P03363	O94874	0.74841
P03363	Q86SQ7	0.8101
P03363	P0CJ78	0.22259
P03363	O15060	0.22259
P03363	Q86W54	0.4211
P03363	O95810	0.31677
P03363	Q8N6I1	0.45159
P03363	Q96PM9	0.44662
P03363	Q7Z5L9	0.47747
P03363	Q6Q759	0.50006
P03363	Q68CP9	0.45575
P03363	O95503	0.82559
P03363	Q9BYQ4	0.2934
P03363	Q8N1E6	0.60465
P03363	P09661	0.77494
P03363	Q9Y399	0.24078
P03363	Q9Y6R9	0.12015
P03363	Q96JB5	0.50614
P03363	P62826	0.27618
P03363	Q08211	0.45628
P03363	Q6NYC8	0.27474
P03363	Q8TF05	0.34607
P03363	O95625	0.26857
P03363	P52434	0.19607
P03363	Q92800	0.40679
P03363	A8MU46	0.29974
P03363	P63302	0.19041
P03363	O14641	0.32983
P03363	P05204	0.58429
P03363	Q9H9H4	0.075639
P03363	Q8NHV9	0.31812
P03363	O43592	0.46664
P03363	Q9NS91	0.43031
P03363	Q9UBC3	0.24345
P03363	P52292	0.75293
P03363	Q16534	0.35945
P03363	Q9P2F9	0.22259
P03363	O15235	0.62222
P03363	Q8IVN3	0.24985
P03363	Q9H1I8	0.22927
P03363	P48595	0.33089
P03363	P49901	0.093349
P03363	P41212	0.87033
P03363	Q86V20	0.040739
P03363	P19429	0.17467
P03363	Q96K75	0.22259
P03363	Q8N1F7	0.3503
P03363	Q9NRD1	0.38844
P03363	Q9UI43	0.17834
P03363	Q9NR12	0.22726

P03363	Q9UJY5	0.42207
P03363	Q9UQB3	0.61252
P03363	P09017	0.32102
P03363	Q9UJU5	0.35214
P03363	Q9P287	0.62948
P03363	Q9H9T3	0.52902
P03363	O15522	0.29325
P03363	Q5FWF6	0.22259
P03363	Q9UJG1	0.39625
P03363	Q9UFN0	0.77561
P03363	Q8N7W2	0.53508
P03363	O43734	0.30887
P03363	P09497	0.42771
P03363	Q8NF64	0.68096
P03363	O43752	0.1242
P03363	P39880	0.64442
P03363	P62917	0.46425
P03363	Q13813	0.29173
P03363	Q5XKE5	0.65787
P03363	Q99590	0.48559
P03363	Q53H80	0.33522
P03363	Q9UK73	0.6976
P03363	Q7Z2W9	0.2119
P03363	Q9ULD2	0.31132
P03363	Q7Z794	0.90158
P03363	Q9NS73	0.76076
P03363	Q9H9S0	0.40597
P03363	Q9ULX9	0.51783
P03363	P55884	0.51787
P03363	Q13137	0.84776
P03363	Q9Y2B4	0.41006
P03363	Q96N16	0.51531
P03363	Q07687	0.64458
P03363	O43715	0.63234
P03363	Q0VDF9	0.50689
P03363	O15042	0.66586
P03363	Q96LB3	0.6548
P03363	Q9UIH9	0.29867
P03363	Q6P2C8	0.64203
P03363	Q8IY18	0.31609
P03363	O43708	0.31204
P03363	O43237	0.24633
P03363	Q9BT81	0.47576
P03363	Q9UJT2	0.70814
P03363	Q9NYW8	0.17268
P03363	Q9H6S0	0.26854
P03363	A6NJG6	0.51853
P03363	Q92621	0.65948
P03363	Q8HWS3	0.37777
P03363	O95251	0.46651
P03363	Q99459	1.0535
P03363	Q13352	0.23791
P03363	P78337	0.51121
P03363	P51815	0.268
P03363	Q8TD16	0.44017
P03363	P15172	0.24598
P03363	Q8WV99	0.17191
P03363	Q7Z4H7	0.63714
P03363	Q14764	1.0618
P03363	Q9UID6	0.47546
P03363	Q96MH2	0.99868
P03363	Q96CS4	0.22259
P03363	Q99666	0.28553
P03363	Q3MJ62	0.268
P03363	Q05516	0.41153
P03363	Q5MIZ7	0.54637
P03363	Q12837	0.51688
P03363	A6NJL1	0.268
P03363	Q96HA7	0.72035
P03363	Q7Z417	0.87684
P03363	Q9P2B4	0.50461
P03363	O43513	0.79537
P03363	Q9BUJ2	1.0421
P03363	P51398	0.83776
P03363	O15534	0.31525
P03363	Q8WXF8	0.65788

13377	P03363	P33240	0.62659
13378	P03363	Q16658	0.34407
13379	P03363	O75688	0.25599
13380	P03363	Q8N987	0.39052
13381	P03363	Q00765	0.20987
13382	P03363	Q3ZCX4	0.22259
13383	P03363	Q8TBZ5	0.22259
13384	P03363	Q15561	0.70373
13385	P03363	A8MSI8	0.12911
13386	P03363	Q8NFH5	0.26529
13387	P03363	O60518	0.9934
13388	P03363	A8MT65	0.22259
13389	P03363	P60953	0.17567
13390	P03363	F5GYI3	0.02512
13391	P03363	Q9Y675	0.24993
13392	P03363	Q9BVG4	0.11203
13393	P03363	Q8N9W8	0.49651
13394	P03363	Q05BV3	0.34776
13395	P03363	P50219	0.25404
13396	P03363	Q13106	0.24904
13397	P03363	Q8TDG2	0.29593
13398	P03363	Q9GZX5	0.61933
13399	P03363	A6NLF2	0.19772
13400	P03363	Q6ICB0	0.20296
13401	P03363	O00541	0.33864
13402	P03363	Q9Y5N6	0.54462
13403	P03363	Q99717	0.35932
13404	P03363	Q9BYC9	0.6324
13405	P03363	Q8TF50	0.22259
13406	P03363	P08238	0.28927
13407	P03363	O60238	0.14493
13408	P03363	Q9UBC1	0.55395
13409	P03363	Q15527	0.11203
13410	P03363	Q9H098	0.0016695
13411	P03363	Q96G25	0.81214
13412	P03363	Q9UQ35	0.36183
13413	P03363	Q5T2W1	0.54182
13414	P03363	P17038	0.22259
13415	P03363	Q99865	0.12371
13416	P03363	O96018	0.52835
13417	P03363	P0CJ79	0.22259
13418	P03363	Q5H9L4	0.26712
13419	P03363	Q6P9G9	0.268
13420	P03363	O60716	0.36172
13421	P03363	P56555	0.12476
13422	P03363	Q8N319	0.1747
13423	P03363	P25208	0.66066
13424	P03363	P43356	0.78388
13425	P03363	P53675	0.30447
13426	P03363	Q9BRT9	0.38324
13427	P03363	Q6R2W3	0.55123
13428	P03363	K7EIQ3	0.070921
13429	P03363	Q9BZE7	0.11203
13430	P03363	P48643	0.20189
13431	P03363	O00170	0.48148
13432	P03363	Q8WWL2	0.27306
13433	P03363	Q8IUD2	0.7241
13434	P03363	Q06520	0.27446
13435	P03363	Q8TBN0	0.4622
13436	P03363	O43598	0.26315
13437	P03363	Q9UPW6	0.65322
13438	P03363	O00257	0.84381
13439	P03363	Q9P2Q2	0.26982
13440	P03363	Q66K64	0.10575
13441	P03363	Q5T890	0.33561
13442	P03363	Q15047	0.49371
13443	P03363	P24666	0.32124
13444	P03363	Q15154	0.4928
13445	P03363	Q12857	0.38777
13446	P03363	Q96NI8	0.22259
13447	P03363	Q5TB80	0.57001
13448	P03363	Q96NB3	0.34423
13449	P03363	Q8TF32	0.22259
13450	P03363	Q6NSI3	0.22504
13451	P03363	Q9BXX3	0.49858
13452	P03363	Q15181	0.20621

P03363	Q6ZMS7	0.42681
P03363	Q9BYH8	0.34409
P03363	Q9Y2T7	0.47616
P03363	Q9P0T4	0.22259
P03363	Q9UBD0	1.1447
P03363	Q8N7H5	0.64243
P03363	Q96B67	0.58976
P03363	Q969F9	0.21578
P03363	P46976	0.27747
P03363	Q5VWQ0	0.22504
P03363	Q6ZTQ3	0.38875
P03363	Q9NRC8	0.80769
P03363	Q8WYQ5	0.58753
P03363	P60002	0.27176
P03363	Q6NUP7	0.62814
P03363	Q8N653	0.28329
P03363	Q7L5D6	0.28064
P03363	O75525	0.69963
P03363	Q15643	0.83748
P03363	P18124	0.36127
P03363	Q9H4I2	0.63426
P03363	Q9BTA9	0.69637
P03363	Q58WW2	0.34775
P03363	Q9NWX5	0.27846
P03363	P40121	0.36894
P03363	Q9H3H9	0.40525
P03363	Q969S2	0.24863
P03363	Q8TF74	0.72001
P03363	Q9P2M7	0.51141
P03363	Q8TDP1	0.19753
P03363	Q8NEJ9	0.20394
P03363	Q9NWB6	0.40525
P03363	P51825	0.9464
P03363	Q9NV06	0.40953
P03363	P27986	0.17125
P03363	P40429	0.43077
P03363	Q96MF7	0.30832
P03363	O95166	0.3038
P03363	Q5H9R7	0.8598
P03363	Q12926	0.59156
P03363	A6NNA2	0.026912
P03363	Q9NP66	0.87177
P03363	Q76FK4	0.67725
P03363	Q5T160	0.064482
P03363	Q00536	0.1203
P03363	Q32M78	0.22259
P03363	Q9BV97	0.24352
P03363	Q86Y97	0.41908
P03363	P67775	0.32433
P03363	P10589	0.3223
P03363	Q92817	0.3367
P03363	Q6P1X5	0.64855
P03363	Q969S3	0.60428
P03363	P15408	0.56795
P03363	Q6RFH5	0.30305
P03363	Q6IQ23	0.42436
P03363	Q9Y6G5	0.53508
P03363	P27635	0.302
P03363	Q9H6T0	0.37848
P03363	Q6QNY0	0.40867
P03363	P63208	0.38146
P03363	Q96E11	0.07112
P03363	O15198	0.31421
P03363	Q8NBZ0	0.87386
P03363	Q9BWT6	0.28707
P03363	P63129	0.098653
P03363	Q8TBE0	0.7005
P03363	P06730	0.42114
P03363	P62263	0.25136
P03363	Q96NL3	0.22259
P03363	Q9Y473	0.39402
P03363	P13056	0.48822
P03363	Q8TD31	0.19742
P03363	Q8NCY6	0.22504
P03363	Q96A73	0.04356
P03363	Q96N21	0.26764

P03363	Q96FJ2	0.51708
P03363	Q9BTD8	0.33526
P03363	Q5SRH9	0.11203
P03363	P61582	0.22504
P03363	Q8WYH8	0.29448
P03363	Q9GZN7	0.15119
P03363	Q5VWM5	0.10142
P03363	Q99594	0.90448
P03363	Q14678	0.43592
P03363	Q70EL1	0.51026
P03363	Q8NCF5	0.60774
P03363	Q9NTM9	0.27113
P03363	O43324	0.91013
P03363	Q17R26	0.24352
P03363	Q9BWC9	0.22504
P03363	Q5VWG9	0.55039
P03363	Q9ULQ0	0.25955
P03363	Q5PSV4	0.37593
P03363	Q8IZU3	0.30733
P03363	Q9Y247	0.22504
P03363	Q93008	0.27395
P03363	P15259	0.16402
P03363	Q86XL3	0.25136
P03363	Q5JSJ4	0.10096
P03363	Q8TCE6	0.12059
P03363	Q96LS8	0.006384
P03363	Q2M2I5	1.0007
P03363	Q8NEM1	0.27378
P03363	Q5HYR2	0.22927
P03363	Q15329	0.91866
P03363	Q9H0E9	0.21474
P03363	Q8IZT6	0.28947
P03363	O95149	0.42815
P03363	Q9UMX3	0.48456
P03363	Q9ULW6	0.17795
P03363	Q9NUP1	0.62353
P03363	Q99933	0.64344
P03363	Q9UL49	0.2311
P03363	Q99583	0.46421
P03363	P55735	0.38237
P03363	Q14683	0.28377
P03363	Q3KNW1	0.23148
P03363	Q9NPC8	0.30758
P03363	Q9BYQ8	0.2934
P03363	Q9NXV6	0.4894
P03363	Q6MZT1	0.15359
P03363	Q14203	0.731
P03363	P78347	0.70612
P03363	Q9UJW9	0.7479
P03363	Q9H069	0.24431
P03363	Q9Y5Q3	0.75653
P03363	Q5T0F9	0.43172
P03363	Q9UBW8	0.47926
P03363	Q9Y5K6	0.38782
P03363	Q8N9B5	0.69743
P03363	Q13233	0.18382
P03363	P04792	0.29143
P03363	Q15696	0.55348
P03363	Q9BVC5	0.065472
P03363	Q01664	0.39145
P03363	Q8IXH6	0.83727
P03363	A6NP61	0.24431
P03363	Q96HR8	0.87117
P03363	Q8WVJ9	0.61395
P03363	Q12982	0.40824
P03363	Q99767	0.35665
P03363	Q15006	0.28768
P03363	Q99747	0.48069
P03363	P52746	0.23615
P03363	Q9ULX6	0.49591
P03363	Q9Y5Q8	0.62918
P03363	Q9BVI4	0.42405
P03363	Q86SH2	0.12519
P03363	O75182	0.84655
P03363	Q9HCJ3	0.33526
P03363	Q8IV53	0.22564

P03363	A6NNA5	0.38788
P03363	Q05215	0.26008
P03363	P55327	0.42882
P03363	Q2M2Z5	0.78468
P03363	Q9UII4	0.39935
P03363	A6NDB9	0.22055
P03363	Q9BYE7	0.56246
P03363	O43236	0.39301
P03363	Q9BSJ2	0.9002
P03363	Q86TP1	0.68082
P03363	Q8IZL2	0.54669
P03363	P47914	0.31082
P03363	Q9Y2S7	0.25668
P03363	Q9BRD0	0.11208
P03363	P15036	0.82576
P03363	Q9NPI6	0.74315
P03363	Q9UII5	0.23615
P03363	Q9HD26	0.58672
P03363	O95833	0.27115
P03363	Q15007	0.41824
P03363	Q9NSN8	0.27961
P03363	A6NG73	0.43424
P03363	P0C2Y1	0.24431
P03363	Q9Y2J2	0.53933
P03363	P42166	0.29819
P03363	Q5TYX0	0.10142
P03363	Q9BZE0	0.29534
P03363	Q8TF63	0.22504
P03363	Q5VZE5	0.33478
P03363	Q9UKG1	0.35575
P03363	Q9Y291	0.32206
P03363	Q70IA6	0.39271
P03363	Q9BTV5	0.49632
P03363	Q92993	0.41148
P03363	P02794	0.33155
P03363	P38935	0.36762
P03363	P62701	0.31253
P03363	P11166	0.2353
P03363	Q96FA7	0.099667
P03363	Q9Y6J9	0.6506
P03363	Q1ED39	0.29721
P03363	Q9NWD9	0.4314
P03363	P0CF97	0.18106
P03363	Q96B42	0.16939
P03363	Q9Y2S0	0.19483
P03363	Q01860	0.58063
P03363	Q9Y5S9	0.77535
P03363	Q6ZRQ5	0.60256
P03363	Q8WWX9	0.38699
P03363	O94817	0.6208
P03363	Q9H074	0.53714
P03363	Q8NCE0	0.46774
P03363	Q9P2Y4	0.3932
P03363	Q9BXJ9	0.61626
P03363	Q9ULC6	0.13873
P03363	Q9NSB4	0.62301
P03363	Q9H5Z6	0.53508
P03363	Q8N831	0.15989
P03363	Q6R327	0.23355
P03363	Q15003	0.68401
P03363	Q5MJ07	0.01487
P03363	P31275	0.38788
P03363	O14813	0.41395
P03363	Q9H814	0.37252
P03363	Q08ER8	0.22259
P03363	Q9BUL9	0.76681
P03363	Q14315	0.39258
P03363	P35663	0.22859
P03363	Q16143	0.26552
P03363	Q9BYN8	0.19094
P03363	Q12824	0.52055
P03363	Q8N3X6	0.49104
P03363	P42771	0.35067
P03363	Q53HV7	0.53099
P03363	Q86TG7	0.69497
P03363	Q8WVV9	0.63544

P03363	Q9BV73	0.55748
P03363	Q03060	0.46404
P03363	Q4V348	0.22259
P03363	Q9UJL9	0.24904
P03363	Q9Y5L5	0.10386
P03363	O14627	0.33705
P03363	Q86X55	0.33071
P03363	Q96FS4	0.22821
P03363	Q9H3U1	0.24425
P03363	O94855	0.1923
P03363	Q8IZH2	0.27258
P03363	Q9BSK0	0.051703
P03363	P38432	0.63626
P03363	Q9BQ70	1.0964
P03363	Q49A33	0.22259
P03363	P23297	0.54214
P03363	Q9NV72	0.22259
P03363	Q8WUX9	0.51119
P03363	Q96N38	0.22259
P03363	Q15942	0.3202
P03363	Q9H469	0.51687
P03363	Q9BXG8	0.62874
P03363	Q6PJ61	0.53508
P03363	Q5T749	0.24431
P03363	Q96LM1	0.029897
P03363	O00291	0.47405
P03363	Q658K8	0.10602
P03363	Q06730	0.24904
P03363	O60516	0.41516
P03363	Q15276	0.23226
P03363	Q9UIE0	0.22259
P03363	Q14151	0.98254
P03363	Q03052	0.38455
P03363	Q693B1	0.21411
P03363	Q7Z7E8	0.2603
P03363	Q9H8G1	0.22259
P03363	Q9H944	0.81188
P03363	O15145	0.51674
P03363	Q99551	0.53453
P03363	Q14153	0.11203
P03363	O14576	0.54499
P03363	O60711	0.34167
P03363	Q9NQR4	0.29079
P03363	Q12996	0.35346
P03363	Q9H1Y0	0.43753
P03363	Q92905	0.44644
P03363	Q8N1A0	0.68396
P03363	P58107	0.4029
P03363	Q02446	0.22685
P03363	Q10567	0.4047
P03363	Q96MP8	0.25324
P03363	Q9H1B4	0.60402
P03363	Q9BW27	0.45035
P03363	Q8NEM0	0.31179
P03363	Q9Y6F1	0.37163
P03363	Q9UJW8	0.22259
P03363	Q9NQC8	0.33416
P03363	Q2TAY7	0.4314
P03363	O75419	0.66883
P03363	O43257	0.412
P03363	P68104	0.66236
P03363	Q5H9F3	0.49766
P03363	Q9H2H8	0.54488
P03363	Q5VYK3	0.14949
P03363	Q9BX26	0.31127
P03363	Q9Y573	0.23634
P03363	Q12840	0.53918
P03363	O95190	0.14608
P03363	Q8TC44	0.27533
P03363	Q9BWG6	0.20839
P03363	Q9UIA9	0.52563
P03363	O14628	0.22259
P03363	O43251	0.6143
P03363	Q8NBF1	0.35396
P03363	Q6P4R8	0.84298
P03363	Q15061	0.29721

P03363	A6NN14	0.22259
P03363	O14503	0.6052
P03363	A5PL33	0.11203
P03363	A8MUQ0	0.01942
P03363	Q8TD47	0.25149
P03363	Q9UNP9	0.70716
P03363	Q96FW1	0.23208
P03363	O14686	0.29541
P03363	Q10587	0.47884
P03363	Q96MN5	0.24232
P03363	O75528	0.46046
P03363	Q9NZM5	0.16103
P03363	Q8WW24	0.33022
P03363	P11142	0.27299
P03363	P55854	0.68874
P03363	Q9NRY2	0.69845
P03363	Q9HBU1	0.2298
P03363	Q13465	0.29551
P03363	P58557	0.086474
P03363	A6NJ46	0.49158
P03363	Q68DY1	0.22259
P03363	Q12888	0.56502
P03363	Q15906	0.86005
P03363	Q9BYD6	0.20041
P03363	Q8IXT5	0.42442
P03363	Q92585	0.6335
P03363	P10243	0.44683
P03363	P61573	0.55152
P03363	Q92698	0.42
P03363	P07737	0.67822
P03363	O14639	0.5245
P03363	Q96CW6	0.52433
P03363	Q13278	0.018951
P03363	Q13094	0.6011
P03363	O75822	0.68795
P03363	Q49AG3	0.13635
P03363	Q5T447	0.513
P03363	P22670	0.77823
P03363	Q99873	0.54284
P03363	Q86TJ2	0.28524
P03363	Q96B01	0.64218
P03363	Q9NX40	0.4412
P03363	Q9NNW5	0.60619
P03363	Q8WXE1	0.65448
P03363	Q12815	0.63398
P03363	Q7Z3V5	0.22259
P03363	Q9Y222	0.81976
P03363	O75177	0.032636
P03363	P61576	0.55152
P03363	Q8N7Q3	0.22259
P03363	A8MT69	0.46399
P03363	Q96CN9	0.50545
P03363	O43791	0.75806
P03363	Q9BZJ0	0.48014
P03363	O95789	0.12425
P03363	Q8TBZ8	0.22259
P03363	Q15555	0.33478
P03363	P61604	0.62214
P03363	Q96JE9	0.39761
P03363	O75494	0.79425
P03363	Q15650	0.39446
P03363	P10073	0.25836
P03363	Q8WY54	0.56992
P03363	Q8NF99	0.43055
P03363	Q13515	0.70788
P03363	Q14135	0.40525
P03363	O75290	0.22259
P03363	Q9HD42	0.61978
P03363	A0AVT1	0.41251
P03363	Q96E09	0.0041725
P03363	Q9BRA0	0.4314
P03363	Q7Z5D8	0.55275
P03363	Q15038	0.92781
P03363	P62269	0.46744
P03363	A1L190	0.16621
P03363	Q3YEC7	0.38317

P03363	Q15102	0.38322
P03363	Q9Y315	0.09767
P03363	Q9NRX1	0.29739
P03363	Q04743	0.49367
P03363	B2RD01	0.27018
P03363	Q6IEG0	0.45402
P03363	A8MX34	0.27762
P03363	Q9Y2G4	0.37246
P03363	Q6ZUV0	0.2016
P03363	Q9HD34	0.21533
P03363	O75569	0.29179
P03363	Q9Y421	0.33514
P03363	Q9NQ94	0.38656
P03363	P51610	0.42671
P03363	P67809	0.67419
P03363	P10916	0.28796
P03363	P05937	0.3385
P03363	Q9NUK0	0.21542
P03363	O15511	0.55333
P03363	O14595	0.15746
P03363	A1L443	0.051971
P03363	Q8NA31	0.23109
P03363	Q8IU60	0.58358
P03363	Q96DI7	0.8044
P03363	Q9BUN5	0.55913
P03363	Q69YN4	0.274
P03363	O94776	0.69857
P03363	Q04695	0.93272
P03363	Q9NR83	0.42101
P03363	Q86YC2	0.65618
P03363	Q8N9W5	0.23743
P03363	O15535	0.268
P03363	Q6ZT77	0.22259
P03363	Q9NZH4	0.32962
P03363	Q9Y5A7	0.71597
P03363	Q9NY74	0.49651
P03363	A8MWA4	0.22259
P03363	Q13098	0.34641
P03363	O15397	0.86251
P03363	Q96AX9	0.33828
P03363	Q9UPT5	0.54118
P03363	O43719	0.32426
P03363	Q8N3C0	0.47575
P03363	Q96KN7	0.27379
P03363	Q9P2A4	0.45594
P03363	O75467	0.22259
P03363	Q13424	0.40247
P03363	Q13542	0.40792
P03363	Q13166	0.11203
P03363	Q6PJT7	0.83465
P03363	Q96QF0	0.65358
P03363	O15160	0.18179
P03363	Q9UHD9	0.78869
P03363	Q9H0H3	0.24431
P03363	P52747	0.6445
P03363	Q9NP77	0.43424
P03363	Q9Y221	0.73071
P03363	P56181	0.13823
P03363	O75928	0.45276
P03363	O60508	0.49788
P03363	P68036	0.57691
P03363	Q6PD62	0.54878
P03363	Q8TAP9	1.0723
P03363	P28324	1.2136
P03363	Q8N344	0.56356
P03363	Q13118	0.42794
P03363	Q8N8A2	0.53508
P03363	Q96SQ5	0.22259
P03363	Q9UH99	0.20414
P03363	P60371	0.2934
P03363	P23511	0.96171
P03363	Q6UB98	0.49651
P03363	P17035	0.22259
P03363	P49368	0.35114
P03363	Q68DX3	0.5469
P03363	Q96AA8	0.096513

P03363	Q96CW1	0.27956
P03363	Q9BTT0	0.40587
P03363	O60216	0.41548
P03363	Q09161	0.59112
P03363	Q6UVJ0	0.79883
P03363	Q8WUU5	0.28924
P03363	O43502	0.36889
P03363	Q96MW7	0.40039
P03363	O95831	0.47278
P03363	Q15833	0.33681
P03363	Q6W0C5	0.2477
P03363	Q8TDN6	0.34102
P03363	Q8TED0	0.44861
P03363	O43299	0.98559
P03363	O75665	0.58954
P03363	Q8N7J2	0.12705
P03363	Q8N0Y2	0.268
P03363	Q6TAR4	0.16157
P03363	Q05BW5	0.39755
P03363	Q8N4W9	0.22259
P03363	P46783	0.85675
P03363	A6NM28	0.22259
P03363	Q99689	0.26142
P03363	Q96CW5	0.76847
P03363	Q92785	0.4411
P03363	Q69YQ0	0.39803
P03363	Q9P016	0.22504
P03363	Q9UBB5	0.89944
P03363	Q02577	0.63356
P03363	Q96G03	0.10754
P03363	Q96BD6	0.27846
P03363	Q92754	0.44511
P03363	A6NHQ2	0.35733
P03363	Q9NWU2	0.22504
P03363	Q7RTU3	0.43142
P03363	A6NFD8	0.35123
P03363	Q3LI63	0.27762
P03363	Q9UI15	0.25602
P03363	Q9UIS9	0.51842
P03363	Q16594	0.40905
P03363	Q15319	0.31465
P03363	Q6VUC0	0.53054
P03363	Q96SN8	0.39128
P03363	Q8N9N8	0.22978
P03363	Q6PK81	0.22259
P03363	Q08AD1	0.83885
P03363	Q9C0H9	0.20957
P03363	Q8IVT2	0.030013
P03363	Q9GZY8	0.22099
P03363	Q3KQU3	0.30182
P03363	Q99613	0.68795
P03363	Q8TEL6	0.58519
P03363	Q9BSC4	0.29721
P03363	Q13401	0.26537
P03363	Q86VM9	0.20896
P03363	Q9H5Q4	0.073178
P03363	Q04637	0.463
P03363	Q9H1J1	0.53477
P03363	Q9H5J8	0.8985
P03363	Q6PI98	0.43091
P03363	Q9UFB7	0.22259
P03363	Q9HAZ2	0.32234
P03363	O00303	0.36721
P03363	Q9UQC2	0.31148
P03363	Q9BXD5	0.11131
P03363	O15315	0.35572
P03363	Q9H9H5	0.27265
P03363	P01100	0.31771
P03363	Q96H86	0.22259
P03363	Q96M61	0.53508
P03363	Q9UGC7	0.24702
P03363	P52294	0.72787
P03363	Q8TA94	0.22259
P03363	Q5JVG8	0.27378
P03363	Q9UBX0	0.35667
P03363	Q5TA45	0.40402

P03363	Q03938	0.25023
P03363	P42285	0.40028
P03363	Q7RTT6	0.39008
P03363	O15234	0.73699
P03363	O14757	0.30829
P03363	Q6P1L8	0.33395
P03363	Q16600	0.25101
P03363	O43913	0.40629
P03363	Q9H2A3	0.29962
P03363	Q15032	0.18106
P03363	Q8IXT1	0.32208
P03363	O00534	0.29721
P03363	Q8N137	0.77238
P03363	Q9Y314	0.80886
P03363	P49640	0.5343
P03363	P53370	0.42069
P03363	Q8N8U2	0.57152
P03363	Q86TU6	0.04981
P03363	Q8N812	0.025469
P03363	Q9NX95	0.41379
P03363	Q12816	0.48215
P03363	Q8N960	0.23757
P03363	Q15819	0.6248
P03363	Q5T5Y3	0.4097
P03363	Q9UPM8	0.32323
P03363	Q14192	0.27255
P03363	Q6DKI1	0.23375
P03363	Q96CF2	0.26785
P03363	Q6QHK4	0.29768
P03363	Q4G0F5	0.27629
P03363	P11161	0.36896
P03363	Q96Q35	0.27491
P03363	Q8NDT2	0.75825
P03363	P53990	0.54448
P03363	Q8N3Y1	0.45445
P03363	Q9BYQ5	0.2934
P03363	Q9Y2P7	0.52009
P03363	O00482	0.26447
P03363	Q8TDQ7	0.22167
P03363	A5D8V6	0.20881
P03363	Q5SW79	0.87339
P03363	P52756	0.39924
P03363	Q9BRP4	0.5264
P03363	Q9Y281	0.73075
P03363	Q9BXY8	0.40665
P03363	Q9UJC3	0.40039
P03363	P05813	0.31329
P03363	Q53GL7	0.53012
P03363	Q9H7R5	0.22259
P03363	Q02078	0.30521
P03363	Q96JB2	0.60271
P03363	Q9Y3C7	0.71555
P03410	Q3ZCQ2	1.6653
P03410	Q9BRP7	0.43532
P03410	Q96RL1	0.7601
P03410	Q6MZT1	1.5766
P03410	Q9NSD4	0.79187
P03410	Q32P41	0.44444
P03410	O60711	0.52089
P03410	Q8N9H9	1.6653
P03410	Q6ZUU3	1.6653
P03410	P78358	1.6157
P03410	Q92546	1.6713
P03410	Q3B820	1.4264
P03410	Q9UDY4	0.90548
P03410	Q96LL4	1.4883
P03410	Q15650	0.72057
P03410	Q96EG3	0.56657
P03410	Q86UW9	0.72009
P03410	Q96MF0	0.35216
P03410	Q9HAW0	0.52152
P03410	P84022	0.84973
P03410	Q8WVR3	1.4883
P03410	A6NL05	0.85205
P03410	Q92830	0.77857
P03410	Q8N4H5	1.2194

P03410	Q9Y2P7	0.77238
P03410	Q96QF7	0.6088
P03410	Q8N3J3	0.76134
P03410	Q96AJ1	1.9234
P03410	Q9H354	1.4411
P03410	Q96LI5	0.5418
P03410	Q14202	0.91496
P03410	O15511	0.55592
P03410	Q03188	0.93611
P03410	P15559	0.1864
P03410	O75192	1.0406
P03410	Q8WUQ7	0.13247
P03410	Q400G9	0.81031
P03410	Q8N9Q2	0.6018
P03410	O75505	0.2823
P03410	P30039	0.44555
P03410	Q99567	0.90084
P03410	O60287	1.892
P03410	Q9BWH6	0.97135
P03410	Q8N3F0	1.0259
P03410	Q14687	0.6999
P03410	Q96LI6	0.39336
P03410	Q9HCG1	0.80974
P03410	A1L3X0	0.86335
P03410	Q00610	1.0178
P03410	Q9UJT2	1.6197
P03410	Q8WV99	0.55966
P03410	Q9Y305	1.2495
P03410	Q96AQ6	1.3301
P03410	P0C875	0.11805
P03410	Q9BVN2	0.83898
P03410	Q6PD74	0.85503
P03410	Q9H3H9	1.2936
P03410	P54803	0.37394
P03410	Q7RTT6	0.78181
P03410	Q99594	1.0689
P03410	Q9UKT4	1.0387
P03410	Q8IWV8	0.3815
P03410	Q96NI8	0.94589
P03410	Q9BY65	0.88539
P03410	Q99700	0.94016
P03410	O14813	0.33989
P03410	Q9P1A6	0.54979
P03410	P69849	0.63371
P03410	Q6IED9	0.27727
P03410	Q9BS18	1.1327
P03410	Q8TDI8	0.66146
P03410	P26447	0.48591
P03410	Q96M02	0.57832
P03410	A8K8V0	0.7107
P03410	Q96HP8	1.616
P03410	Q6AW86	0.67458
P03410	Q7L8W6	0.8348
P03410	Q9HC07	0.48975
P03410	Q08AI8	1.4883
P03410	Q9BRT3	0.40927
P03410	Q9Y6G9	0.26727
P03410	P78385	0.88374
P03410	Q9NQG5	1.1818
P03410	Q5CZA5	0.89343
P03410	O14786	0.11616
P03410	A6NHY6	0.74904
P03410	Q2M3D2	0.40336
P03410	Q71F56	0.48253
P03410	P83876	1.0688
P03410	Q2KHR2	0.38817
P03410	Q14397	0.74379
P03410	P49750	0.96915
P03410	Q9H4K7	0.98316
P03410	O95684	1.0561
P03410	Q9H0Y0	0.25541
P03410	Q30KP8	0.98563
P03410	Q9NQG7	0.49866
P03410	P57086	0.6523
P03410	Q9H0M5	0.6829
P03410	P13645	0.96632

P03410	Q86UP9	1.2023
P03410	Q0PNE2	0.86985
P03410	Q99933	0.93393
P03410	O75648	0.97394
P03410	Q9BVC5	1.1526
P03410	Q8IXH6	1.2066
P03410	Q99743	0.23397
P03410	Q8TAG6	0.89222
P03410	Q92540	1.0004
P03410	Q3KRA9	0.54924
P03410	Q92831	0.90658
P03410	Q96BY7	0.32852
P03410	Q7L592	0.81526
P03410	Q96LZ7	0.22565
P03410	Q9BZZ5	0.52042
P03410	Q13838	0.63849
P03410	Q00613	0.78473
P03410	P20749	0.27142
P03410	Q96M15	0.34492
P03410	Q8NC96	0.78505
P03410	Q96M11	1.85
P03410	Q8IV20	0.79056
P03410	Q9Y2Q9	1.0797
P03410	Q9Y2W6	0.27425
P03410	A6NGH7	0.72994
P03410	Q9Y5J3	0.22822
P03410	Q8NHY2	0.96889
P03410	Q9BQK8	0.18871
P03410	Q5JTH9	0.68261
P03410	O95168	0.42389
P03410	Q8WTT2	0.52183
P03410	Q9BWE0	0.43787
P03410	Q9UKV0	0.38319
P03410	Q9NUP1	0.88781
P03410	P09496	0.37863
P03410	Q8WV41	0.52619
P03410	Q99848	0.59147
P03410	Q9NQ90	0.73338
P03410	Q8IVS8	0.14558
P03410	Q9UHF7	0.45683
P03410	Q9UBW5	0.83836
P03410	Q14CM0	0.30418
P03410	Q86VV8	0.87395
P03410	Q8IX90	0.89034
P03410	P62258	1.0163
P03410	Q68DN1	0.89246
P03410	Q96FW1	0.13782
P03410	O75665	0.97716
P03410	Q53S58	1.0913
P03410	Q9Y2M2	0.78525
P03410	Q9BSM1	0.99002
P03410	P40426	0.2953
P03410	Q4G163	0.19124
P03410	Q96QT6	0.92188
P03410	Q15726	0.52888
P03410	Q8N377	1.6653
P03410	Q5SNT6	1.3129
P03410	Q7Z460	0.57658
P03410	Q9HC24	0.23142
P03410	Q8NBC4	1.6653
P03410	P0DI82	0.51688
P03410	Q92562	0.53453
P03410	Q04206	0.8605
P03410	O60583	1.2158
P03410	A8K554	0.29261
P03410	Q9Y2W1	1.0862
P03410	Q13536	1.6653
P03410	Q08945	1.0479
P03410	Q9NXF1	0.3416
P03410	O43674	0.84059
P03410	P08048	0.32429
P03410	Q5T870	1.2381
P03410	Q9H875	0.67578
P03410	O75949	1.0913
P03410	P62714	0.91955
P03410	A8MZF0	1.4883

P03410	O14771	1.0622
P03410	Q9Y6Q5	0.70597
P03410	P46976	0.37877
P03410	O43399	0.84625
P03410	Q5JY77	0.95766
P03410	Q8NAS9	0.35226
P03410	Q8NBR0	1.15
P03410	Q7L4P6	1.0537
P03410	O14569	0.7603
P03410	Q9UQC2	0.47564
P03410	P28069	0.52679
P03410	Q9UI54	1.6653
P03410	A0PJX8	1.0913
P03410	Q92917	0.91095
P03410	Q5SRD1	0.56619
P03410	Q9BZI1	0.23668
P03410	P62861	2.0352
P03410	Q709C8	1.0189
P03410	Q86Y91	0.35664
P03410	Q14204	0.60988
P03410	Q9Y2L8	0.91229
P03410	Q96DP5	1.3142
P03410	Q9UH77	0.86996
P03410	O95292	0.32041
P03410	P84157	1.0913
P03410	P49321	1.1509
P03410	Q96T21	1.2624
P03410	Q96TC7	1.464
P03410	Q53SF7	0.3227
P03410	A4FU28	0.66737
P03410	Q9BV68	0.50973
P03410	Q8N6U2	1.0381
P03410	Q5TA50	0.52554
P03410	Q96M66	0.51493
P03410	B4DYI2	0.29038
P03410	Q96LP4	0.54498
P03410	Q9HC78	0.28202
P03410	A6NF36	1.0965
P03410	Q5TA45	0.33522
P03410	Q9H3M9	0.59117
P03410	Q9ULU4	0.74393
P03410	Q8N1W2	0.546
P03410	Q96JB3	0.31748
P03410	Q5HYJ3	1.1164
P03410	P13994	0.72872
P03410	O60224	0.35308
P03410	Q86V20	1.4696
P03410	Q9HCH5	0.82011
P03410	E9PQR5	0.13356
P03410	P05386	1.3686
P03410	Q8IWF9	0.33195
P03410	Q96DY7	0.85133
P03410	Q7L5A3	1.1241
P03410	Q9NVN8	0.42532
P03410	Q96E09	1.465
P03410	Q6ZNG0	0.74636
P03410	Q9BUK0	1.7325
P03410	O14901	0.21728
P03410	Q5SZD1	0.89888
P03410	Q86UT5	0.34586
P03410	O00472	0.62801
P03410	Q3SY00	1.3604
P03410	Q6ZNA1	0.78793
P03410	A8MWD9	0.20788
P03410	Q9P2R7	0.63158
P03410	O15392	0.24443
P03410	Q9NS86	0.51178
P03410	P35712	0.58902
P03410	Q9H305	0.22734
P03410	Q6UUV7	2.0967
P03410	P17031	0.85712
P03410	P59942	1.3648
P03410	A6NJ78	1.414
P03410	Q8NHR9	0.29401
P03410	Q9HC47	1.6653
P03410	P22670	0.83668

P03410	Q9NZM5	0.77089
P03410	Q9Y2Q5	0.42193
P03410	Q96QG7	0.51301
P03410	Q29RF7	1.1692
P03410	Q5HY98	1.0047
P03410	Q5TB30	0.24856
P03410	Q9NPH2	0.58057
P03410	Q9NVR2	1.179
P03410	Q9UPM8	0.69845
P03410	Q9NW81	0.14012
P03410	Q8NDC0	1.3254
P03410	Q86VE9	0.86137
P03410	Q7Z3B4	1.0674
P03410	Q8NHW5	0.50686
P03410	Q5SVZ6	1.3943
P03410	P47944	1.5031
P03410	Q8IXJ6	0.44506
P03410	Q9HD34	0.93512
P03410	Q9NQ89	0.60311
P03410	P23025	0.58476
P03410	A6NMK8	1.1122
P03410	P42771	0.64112
P03410	P61576	0.6793
P03410	Q8IZ63	1.6653
P03410	Q9BXU2	1.0049
P03410	O43309	0.71177
P03410	Q969E8	1.6826
P03410	P02810	1.6653
P03410	Q9UI36	0.21677
P03410	Q6ECI4	0.78354
P03410	P49069	0.47007
P03410	P13646	0.93405
P03410	Q9UI56	1.6653
P03410	Q9BT56	1.207
P03410	Q9H9Q2	0.36002
P03410	Q7L4I2	2.2377
P03410	Q8IU60	0.90743
P03410	Q15714	0.67657
P03410	Q9BSU3	0.53903
P03410	Q9UPP1	0.33887
P03410	Q6UXR6	1.6653
P03410	Q4G0N0	0.88221
P03410	P28070	0.81054
P03410	Q8NEL0	0.23565
P03410	Q9NVT9	0.21689
P03410	O95563	0.75265
P03410	P49959	0.23937
P03410	Q8IYU4	0.14628
P03410	O00203	0.1318
P03410	Q96DS6	1.0913
P03410	A6NJI1	0.89572
P03410	Q96RE9	0.75746
P03410	Q86SE8	0.10611
P03410	Q96BF6	0.55259
P03410	Q8TF63	1.8261
P03410	P23409	0.013282
P03410	Q9H496	0.71684
P03410	Q5T0J3	0.4002
P03410	Q9Y573	0.20429
P03410	P63302	1.1452
P03410	Q96QH2	0.47127
P03410	O95619	0.70105
P03410	Q5JSH3	0.37798
P03410	Q6V9R5	0.97066
P03410	Q9BW27	0.98828
P03410	Q96EK5	0.37361
P03410	P04141	0.30449
P03410	Q9BWJ2	0.67641
P03410	Q5T5S1	0.85193
P03410	Q96KX2	0.064758
P03410	Q5JVG2	0.61459
P03410	Q8N3U1	1.6653
P03410	Q6B0K9	0.83359
P03410	Q8WUZ0	1.4765
P03410	Q71RC2	0.71331
P03410	Q9H6W3	0.68013

P03410	Q9H469	0.28881
P03410	Q96Q11	0.6019
P03410	Q7L775	0.70678
P03410	O96018	0.75993
P03410	Q96NL8	1.8247
P03410	P11831	0.86184
P03410	O60281	0.27871
P03410	Q7L804	0.83848
P03410	B1AJZ1	0.094763
P03410	P53602	0.73646
P03410	H0Y7S4	0.34826
P03410	Q30KR1	0.87928
P03410	Q5H9J7	1.3365
P03410	Q9UNX4	0.4605
P03410	Q8NBF1	0.32566
P03410	O43439	0.90799
P03410	Q9UBK5	1.6741
P03410	Q9NWY4	0.90965
P03410	Q9Y2L1	0.44742
P03410	Q9NS37	0.81015
P03410	O75781	0.31951
P03410	Q5U5X8	1.6186
P03410	Q8N5L8	1.4457
P03410	O15063	1.2124
P03410	P49354	0.42777
P03410	Q14588	0.79465
P03410	Q9H6X4	1.0913
P03410	Q8NA54	0.31002
P03410	Q96PV4	0.065808
P03410	Q2M2E5	1.2126
P03410	Q8WXF8	1.8238
P03410	Q9P2N6	0.78572
P03410	P78537	1.6519
P03410	P0C7M6	0.16458
P03410	Q717R9	1.2427
P03410	Q96HJ5	0.41595
P03410	Q96FX2	1.1194
P03410	Q9Y5L4	0.11097
P03410	Q6ZT52	0.00041981
P03410	P40692	0.23205
P03410	B3EWG4	1.4883
P03410	Q96HD9	0.42649
P03410	Q6NZI2	1.1945
P03410	A6NI86	1.2571
P03410	Q96BR6	0.91714
P03410	Q9Y2S7	1.3757
P03410	Q14159	2.0264
P03410	Q9H981	1.0849
P03410	Q5EBM4	0.36
P03410	Q9HB07	0.47998
P03410	P22087	0.74114
P03410	O75290	0.92179
P03410	A6NDU8	0.70433
P03410	Q8IZS6	0.40593
P03410	Q5VT52	0.49167
P03410	O95782	0.74682
P03410	Q8N4H0	0.17123
P03410	B2RXF5	0.42339
P03410	Q9Y399	1.8787
P03410	B7ZBB8	0.28228
P03410	Q7Z7F7	0.97539
P03410	Q9ULE6	0.92813
P03410	P60371	1.9984
P03410	A4D1U4	0.58542
P03410	O95045	0.59666
P03410	Q9BYN7	0.55314
P03410	A1L4L8	0.83255
P03410	P0CJ87	0.27528
P03410	O71037	0.76316
P03410	P30047	0.77385
P03410	Q9BYZ2	0.69158
P03410	Q17RF5	1.5113
P03410	Q92945	0.62477
P03410	Q9GZP8	1.6653
P03410	Q9BZA5	1.287
P03410	Q96EF9	0.9228

P03410	Q9Y4R7	0.19354
P03410	Q53HR8	0.2247
P03410	Q17R31	0.42815
P03410	Q15274	0.41189
P03410	Q96AV8	0.29157
P03410	Q02543	1.009
P03410	Q8IV32	1.4051
P03410	Q674R7	0.95477
P03410	Q6ZRH9	1.6653
P03410	Q9Y512	0.30999
P03410	Q8IZU0	1.9262
P03410	P18887	0.97644
P03410	Q5JNZ3	0.85796
P03410	Q13227	1.8666
P03410	Q8IZ73	0.41855
P03410	Q9UPQ9	0.70858
P03410	O75934	0.80554
P03410	P23246	0.73983
P03410	Q8TAU3	0.82084
P03410	Q8IYN0	0.86116
P03410	Q0D2K0	1.0846
P03410	Q05877	1.4773
P03410	Q8IZX4	0.24689
P03410	Q8N9E0	1.4883
P03410	Q7Z7B7	1.4445
P03410	P43320	0.40006
P03410	Q15041	0.14054
P03410	Q9HAJ7	1.7105
P03410	Q9NPR9	1.4518
P03410	P82094	1.3823
P03410	Q6IEE7	1.3458
P03410	P61247	1.2501
P03410	O75335	0.44317
P03410	P19838	0.86983
P03410	Q8WVI7	0.22086
P03410	Q96PC3	0.65802
P03410	Q9UHV2	2.2085
P03410	Q68CJ9	0.6317
P03410	Q96P26	0.60157
P03410	P13489	0.73904
P03410	Q494R0	1.6653
P03410	Q8TB03	1.2765
P03410	Q96Q89	0.60414
P03410	O00519	0.61487
P03410	P0CI01	1.0907
P03410	P62241	0.82472
P03410	Q9Y285	1.1511
P03410	O95801	0.30087
P03410	Q71RC9	1.0992
P03410	P17987	0.29434
P03410	Q9BYR7	1.5889
P03410	Q5QGS0	0.19068
P03410	Q6ZTN6	0.84824
P03410	P30084	0.32907
P03410	Q13094	0.7731
P03410	Q96TA1	0.491
P03410	P36956	0.25734
P03410	Q8ND61	1.3781
P03410	A6NNP5	0.14039
P03410	Q86SZ2	1.1902
P03410	Q8WVJ2	0.41335
P03410	Q53G59	0.58824
P03410	O75154	1.0323
P03410	Q9UHR6	1.3243
P03410	P46527	0.50255
P03410	Q9NYW8	0.66484
P03410	Q96CW1	0.8409
P03410	Q86VQ0	0.8051
P03410	P0DMC3	1.6653
P03410	Q5BKY9	1.4883
P03410	Q96IU2	1.8044
P03410	Q5TC12	0.82555
P03410	Q8N715	0.92384
P03410	P0C7V5	0.86747
P03410	Q00839	0.31601
P03410	Q9ULR0	1.354

P03410	Q9H6K1	0.38408
P03410	Q96RL7	1.2844
P03410	Q16589	0.62157
P03410	Q8N960	0.35507
P03410	Q9H4X1	1.3941
P03410	Q92529	0.39946
P03410	P52655	1.6033
P03410	Q14157	1.2671
P03410	Q9UPV7	0.5065
P03410	Q9H213	0.1839
P03410	Q17RM4	0.88262
P03410	P35244	0.77231
P03410	P55854	0.83208
P03410	O75461	0.22622
P03410	P53999	1.19
P03410	P62829	1.6473
P03410	Q99457	0.60635
P03410	P25205	0.39489
P03410	A6NHP3	1.0907
P03410	Q0VFX4	1.0954
P03410	E9PJ23	0.15432
P03410	P49207	1.7586
P03410	A2VDF0	0.55156
P03410	Q9NUX5	0.20888
P03410	P51610	0.56291
P03410	Q9HBM0	1.2184
P03410	A8MU10	1.6653
P03410	Q17RC7	0.80906
P03410	Q9BW72	0.6859
P03410	Q86WB0	2.0376
P03410	Q15427	0.37696
P03410	A6NFA0	1.0913
P03410	Q9HA77	1.1146
P03410	P07498	1.4844
P03410	Q9H040	0.57286
P03410	Q8WXJ9	0.71362
P03410	Q9BQN1	1.4984
P03410	P10768	0.21718
P03410	Q7L0L9	1.6653
P03410	Q13137	1.3737
P03410	Q8WTP8	0.14525
P03410	Q8IX21	0.52303
P03410	Q9UPP5	1.2147
P03410	Q5T4T6	1.6188
P03410	Q12767	1.0913
P03410	Q9BZE9	1.1351
P03410	Q92564	0.31885
P03410	Q96FI4	0.61981
P03410	A1A580	1.9939
P03410	O75794	1.5266
P03410	Q9UKT7	0.2927
P03410	Q6ZVU0	0.29892
P03410	Q8IYA8	1.4883
P03410	P0CL81	0.508
P03410	Q16822	0.44449
P03410	Q5JR59	1.641
P03410	Q8N5Q1	1.9055
P03410	O94763	1.0315
P03410	Q8IWZ3	1.1572
P03410	Q13829	0.68602
P03410	Q9UI95	1.2465
P03410	Q9UHP9	0.32187
P03410	P26373	1.2457
P03410	Q6NYC8	0.85899
P03410	Q96H79	1.0932
P03410	P0C2Y1	0.45252
P03410	A6NKU9	1.0907
P03410	Q96HZ7	1.6653
P03410	P61578	0.83286
P03410	O75690	1.6653
P03410	P60002	1.4361
P03410	Q9UNN4	0.49139
P03410	Q86SZ3	0.34438
P03410	P27986	0.86162
P03410	Q9NZN8	0.64583
P03410	Q9H0C3	1.0848

14593	P03410	Q969F2	0.16453
14594	P03410	P61165	0.94538
14595	P03410	O15480	0.039069
14596	P03410	Q9Y6J0	0.15044
14597	P03410	Q9P219	0.56375
14598	P03410	O14910	1.0937
14599	P03410	Q9NNX9	1.5909
14600	P03410	Q96PT3	0.25012
14601	P03410	Q6ZMW3	0.3132
14602	P03410	Q9UBL0	0.41974
14603	P03410	Q8WYH8	0.44615
14604	P03410	Q9H267	0.54115
14605	P03410	Q5TA78	1.6653
14606	P03410	P56556	0.67399
14607	P03410	P82663	1.3257
14608	P03410	Q9C0B2	0.69128
14609	P03410	Q8IWB1	0.44681
14610	P03410	P48539	0.58258
14611	P03410	P05121	0.19789
14612	P03410	Q6RUI8	1.2987
14613	P03410	Q7Z4B0	1.6653
14614	P03410	O75925	0.46371
14615	P03410	Q14CN4	0.71041
14616	P03410	Q93073	1.3349
14617	P03410	Q96GN5	0.94188
14618	P03410	Q86XT9	0.99712
14619	P03410	O14960	0.93617
14620	P03410	Q8IXK0	0.17094
14621	P03410	P55036	0.96841
14622	P03410	P0C852	1.6653
14623	P03410	Q96RS0	0.4925
14624	P03410	Q6PI47	0.32577
14625	P03410	O43929	0.13106
14626	P03410	Q96KJ9	0.43776
14627	P03410	Q9H2G9	0.45957
14628	P03410	P22732	0.37302
14629	P03410	Q9HB71	0.69496
14630	P03410	O75330	0.80205
14631	P03410	Q9BWJ5	0.79175
14632	P03410	Q9Y4Y9	0.89934
14633	P03410	Q9NQG1	1.0992
14634	P03410	Q8TAB7	1.2626
14635	P03410	Q96KW9	1.3202
14636	P03410	Q5VVM6	0.17971
14637	P03410	Q8TDQ7	0.18407
14638	P03410	I3L273	0.28713
14639	P03410	Q9BU89	0.46369
14640	P03410	Q6ZNA4	0.30066
14641	P03410	Q9Y315	1.2213
14642	P03410	Q9UMS6	0.8702
14643	P03410	Q9HD33	1.6306
14644	P03410	Q96QR1	1.2989
14645	P03410	Q9P0P8	0.37525
14646	P03410	Q12873	0.47674
14647	P03410	Q5SNX5	1.6653
14648	P03410	Q2TBE0	0.83779
14649	P03410	Q99733	0.85122
14650	P03410	Q8NA61	0.18013
14651	P03410	P23490	1.3783
14652	P03410	A4D263	0.54573
14653	P03410	Q15572	1.3426
14654	P03410	P61513	0.80463
14655	P03410	Q9Y6Z5	1.4536
14656	P03410	Q6N075	0.91205
14657	P03410	Q6PH81	0.6346
14658	P03410	P0C7X5	0.89166
14659	P03410	Q96PE3	0.12081
14660	P03410	Q8NEB7	1.1503
14661	P03410	Q8IWC1	0.7994
14662	P03410	Q96ST2	0.49519
14663	P03410	P50479	0.50973
14664	P03410	P02794	0.80963
14665	P03410	Q15121	0.79441
14666	P03410	Q6P9G4	0.93127
14667	P03410	Q9UDW3	0.32462
14668	P03410	A8MUA0	0.53532

P03410	Q8WXD5	0.90847
P03410	Q9HAH1	0.88582
P03410	Q68CL5	0.3132
P03410	A4D161	0.22013
P03410	A8MVJ9	0.91979
P03410	P0CW27	1.0218
P03410	Q02221	0.82617
P03410	O60675	0.43674
P03410	Q6ZN01	0.67312
P03410	Q5T619	0.71942
P03410	Q9UL41	0.89247
P03410	Q9NTG7	0.62907
P03410	O75663	0.74167
P03410	P02533	1.0155
P03410	O00512	0.969
P03410	Q01081	0.46371
P03410	Q5W0B7	1.0031
P03410	P59052	0.73015
P03410	Q9UL16	0.57415
P03410	Q7RTS9	1.7119
P03410	P10599	0.24501
P03410	Q5I0X4	1.4883
P03410	P31350	0.35235
P03410	Q03112	0.21009
P03410	Q9UK39	0.55555
P03410	Q12893	0.80674
P03410	Q9UGB4	1.6653
P03410	Q719I0	0.34927
P03410	Q6ZMY9	1.0051
P03410	P49368	0.26446
P03410	Q96KC9	1.1963
P03410	P0CG48	0.59087
P03410	Q8N9K7	1.6653
P03410	Q6ZV77	1.6653
P03410	O75127	0.22177
P03410	Q8NFP7	0.31411
P03410	Q9BUB7	1.4776
P03410	Q6PGP7	0.93413
P03410	O15205	0.54898
P03410	Q9Y6H5	0.88816
P03410	Q86UF4	0.17228
P03410	Q96LW4	0.31275
P03410	Q9BTE7	0.95321
P03410	Q96NM4	0.41575
P03410	P51606	0.58709
P03410	Q9BZ97	1.6653
P03410	P49901	1.6653
P03410	Q9H8Y1	1.3062
P03410	O95218	0.55436
P03410	Q8WXU2	0.92515
P03410	A0PJW8	0.12285
P03410	Q30KP9	0.85264
P03410	Q53QW1	1.4883
P03410	Q9UJC3	0.22411
P03410	Q8N2N9	0.77288
P03410	P15924	0.57495
P03410	Q8IXF0	0.27524
P03410	Q9BYQ5	1.9984
P03410	O15355	0.88104
P03410	A6NNJ1	0.71133
P03410	P0DMB2	0.90112
P03410	Q5JU67	0.24319
P03410	A8MVP8	0.34795
P03410	Q6V702	0.39603
P03410	O95639	0.54154
P03410	Q96B33	0.7982
P03410	P61550	0.77303
P03410	Q3SY89	0.38
P03410	Q96EY4	0.86554
P03410	P0C7P2	0.93301
P03410	A6NN14	0.79552
P03410	Q68EN5	0.61594
P03410	Q52LA3	0.79076
P03410	Q32M78	0.93665
P03410	E7ETH6	0.71345
P03410	P60608	0.84218

14745	P03410	P61565	1.1647
14746	P03410	Q5SY13	1.6653
14747	P03410	Q9Y2R0	1.093
14748	P03410	Q9Y3C0	0.57389
14749	P03410	Q13394	0.45332
14750	P03410	Q9UNY4	0.37375
14751	P03410	Q9NR50	0.70127
14752	P03410	Q9UGK3	0.8372
14753	P03410	Q9Y2D5	0.44068
14754	P03410	Q9NP64	0.42584
14755	P03410	Q53FD0	1.2996
14756	P03410	P51808	0.48959
14757	P03410	Q9Y2Y9	0.80875
14758	P03410	Q6NSX1	0.98261
14759	P03410	Q9NXR7	0.7359
14760	P03410	Q7Z6G3	0.7262
14761	P03410	Q99832	0.23898
14762	P03410	Q6ZTR5	0.67643
14763	P03410	Q15293	0.59211
14764	P03410	P82909	0.76966
14765	P03410	Q8NCS4	1.0913
14766	P03410	P0DJI8	0.78091
14767	P03410	P29374	0.23516
14768	P03410	Q9Y675	1.8316
14769	P03410	Q9NZH5	0.69617
14770	P03410	Q4QY38	0.87928
14771	P03410	Q14129	1.0381
14772	P03410	Q4G0N4	0.42752
14773	P03410	Q9HCH0	1.9367
14774	P03410	Q9NSP4	0.31287
14775	P03410	Q13106	0.79191
14776	P03410	Q32M84	0.29589
14777	P03410	A8MYR8	0.13857
14778	P03410	P51161	0.22411
14779	P03410	Q8N9T2	1.6653
14780	P03410	Q8N9Z0	0.9607
14781	P03410	A5D6W6	1.1822
14782	P03410	Q96DU7	0.65665
14783	P03410	Q5T6M2	1.3218
14784	P03410	O14893	0.67134
14785	P03410	O15255	1.6653
14786	P03410	O75865	0.96211
14787	P03410	Q8N5J4	0.49711
14788	P03410	A1L170	1.6653
14789	P03410	O95405	0.32477
14790	P03410	Q96AB3	0.91885
14791	P03410	Q9H4M3	0.588
14792	P03410	Q8NDW4	0.81544
14793	P03410	P02808	1.3129
14794	P03410	Q5VTH2	1.0132
14795	P03410	Q96T76	0.97011
14796	P03410	Q6ZW13	1.6653
14797	P03410	Q9NZL3	0.78211
14798	P03410	Q6UXU1	1.6653
14799	P03410	Q9BU02	1.6294
14800	P03410	Q16666	0.56485
14801	P03410	Q5VSY0	0.57842
14802	P03410	Q5UE93	1.0212
14803	P03410	Q8NAA4	0.25908
14804	P03410	Q969W9	0.95154
14805	P03410	Q8NHU2	0.27443
14806	P03410	Q8WY91	0.79167
14807	P03410	Q15058	0.59889
14808	P03410	A6NEQ2	1.6653
14809	P03410	Q9Y2G2	0.67192
14810	P03410	Q7L8L6	0.6541
14811	P03410	Q7Z4U5	1.4445
14812	P03410	O14681	1.3
14813	P03410	A6XGL0	0.68302
14814	P03410	Q9HDB8	0.62824
14815	P03410	Q5HYK7	1.3011
14816	P03410	Q9UFF9	0.43881
14817	P03410	Q96N53	1.6653
14818	P03410	Q99708	0.82298
14819	P03410	Q5T3Y7	0.54971
14820	P03410	Q9UDV7	0.58281

P03410	P17568	0.17949
P03410	Q8IWE5	0.69666
P03410	O75638	2.1063
P03410	Q14593	0.98893
P03410	O43402	1.4965
P03410	Q9UNN5	0.82046
P03410	Q3SYF9	1.6653
P03410	A1L188	1.6264
P03410	Q8WW14	0.69454
P03410	Q96EY9	0.57319
P03410	Q27J81	0.35512
P03410	Q9HBK9	0.23304
P03410	Q6ZVM7	0.051615
P03410	Q9H1M0	0.27236
P03410	Q5T7P2	1.6653
P03410	Q8IWY9	0.76789
P03410	Q9UKH3	1.1199
P03410	P02008	0.801
P03410	P10144	0.3424
P03410	P52594	0.35876
P03410	A4D2B0	1.005
P03410	P35638	0.64365
P03410	Q5GH73	0.63672
P03410	O14920	0.84168
P03410	Q14526	0.37859
P03410	Q9GZR2	0.13563
P03410	P26368	0.63265
P03410	P68104	0.66271
P03410	O95359	0.49049
P03410	Q8WWF3	1.1105
P03410	O95238	0.2185
P03410	Q9H3R5	0.6754
P03410	Q10570	0.99849
P03410	Q6Q759	1.5523
P03410	O14828	0.86361
P03410	Q8N8D7	1.0629
P03410	Q59GR2	0.2823
P03410	Q9H869	1.9546
P03410	Q02833	0.75696
P03410	Q9UK13	0.98524
P03410	Q8N1F1	1.6653
P03410	Q9Y3B8	0.36641
P03410	Q8N8E2	0.16637
P03410	Q9BVI4	0.66394
P03410	O00470	0.5084
P03410	Q96N58	0.88535
P03410	Q5SXM8	0.43009
P03410	Q8N2C7	1.3358
P03410	Q8IWA6	0.52172
P03410	Q2M3W8	1.0409
P03410	O76011	1.0001
P03410	Q14257	0.30163
P03410	Q8N813	0.57909
P03410	A6PVL3	0.64661
P03410	A6NH52	1.3876
P03410	Q86WI0	0.88488
P03410	Q14974	1.3655
P03410	Q9NV12	1.0913
P03410	Q13155	0.63529
P03410	Q5T0L3	1.491
P03410	Q8N4M7	1.7052
P03410	O60293	0.26461
P03410	Q8TCS8	0.4721
P03410	Q8N0X7	0.29524
P03410	P56279	1.695
P03410	Q8WXA3	0.25041
P03410	O14556	0.24877
P03410	P61326	1.0202
P03410	Q8N8Z8	0.67652
P03410	Q9H4L7	0.33285
P03410	P62072	0.4928
P03410	Q9HB75	0.86
P03410	O75683	1.3065
P03410	O60613	0.89556
P03410	Q8N5S3	0.044999
P03410	A9QM74	0.76759

P03410	Q9GZZ1	0.58511
P03410	Q6ZVN7	0.6805
P03410	Q9NUU6	0.68053
P03410	Q8WUX2	0.31547
P03410	Q8IV54	0.60985
P03410	Q8WVC0	1.001
P03410	P19387	0.87939
P03410	Q96K17	0.79439
P03410	Q86YD7	0.69342
P03410	Q9GZR5	0.62505
P03410	A1A4T8	0.22378
P03410	A1A5D9	1.433
P03410	P11137	0.36133
P03410	Q14103	0.59979
P03410	O96006	1.3083
P03410	A6NKD9	1.6212
P03410	P62304	0.81849
P03410	P59797	1.6199
P03410	Q13296	0.53793
P03410	Q96NR7	0.23692
P03410	O95567	1.6653
P03410	P02144	0.36975
P03410	P59022	0.80508
P03410	P20585	0.16925
P03410	Q3SYA9	0.6931
P03410	P62899	1.3249
P03410	Q96RT1	0.45077
P03410	P16415	0.83424
P03410	Q96N22	1.004
P03410	Q08AD1	0.9191
P03410	Q15833	0.54584
P03410	P35813	0.24469
P03410	Q96HT8	2.3056
P03410	Q13772	0.42127
P03410	Q9NSE4	0.84507
P03410	Q8TC05	1.8748
P03410	Q17RA5	1.6653
P03410	P78316	1.598
P03410	Q6P6C2	0.636
P03410	Q8TCF1	0.38332
P03410	Q96LA8	0.71557
P03410	Q13360	0.71265
P03410	Q8N7R0	0.30793
P03410	A6NE21	0.71133
P03410	P06454	1.4377
P03410	Q9NX01	0.32076
P03410	Q8N7G0	0.30793
P03410	Q9H7Y2	1.2162
P03410	Q9C0H6	0.30816
P03410	P80303	0.22498
P03410	P56192	0.87459
P03410	Q96B49	1.6165
P03410	Q9UPY6	0.27504
P03410	Q96DZ9	0.75695
P03410	Q14739	0.36471
P03410	Q15697	0.50183
P03410	Q5VZ72	0.8618
P03410	Q8IYX3	1.4058
P03410	Q9BRP9	0.20722
P03410	Q8NDW8	0.17645
P03410	Q9NX38	0.3488
P03410	Q6P9B6	0.6999
P03410	Q15323	0.89153
P03410	Q0VDG4	0.51138
P03410	Q96BW1	0.44945
P03410	Q6ZTK2	0.24455
P03410	P0C671	0.13891
P03410	O15234	1.6569
P03410	O15042	0.35518
P03410	Q9H825	0.43842
P03410	Q14C87	1.1594
P03410	Q7Z6I5	1.5232
P03410	Q9BZL4	0.83836
P03410	Q3LHN0	1.9066
P03410	Q8N0S6	0.67869
P03410	Q6ZRS4	1.3104

P03410	Q6L8Q7	0.40592
P03410	Q9BZL1	0.56087
P03410	Q9Y2G0	0.79662
P03410	Q14353	0.18485
P03410	O43633	0.46675
P03410	Q96T23	0.57172
P03410	Q9P015	1.2694
P03410	Q9H147	1.4311
P03410	P49184	0.51372
P03410	Q8IX12	1.0743
P03410	Q4VC05	1.4988
P03410	Q5T7R7	1.0913
P03410	Q96GC9	0.63773
P03410	Q13889	0.26536
P03410	Q14061	1.2278
P03410	O15172	0.61382
P03410	Q6A163	0.88723
P03410	Q8NCG7	1.2968
P03410	Q6PCB5	0.73589
P03410	Q8WXX7	1.4474
P03410	Q8TF08	1.0078
P03410	Q6ZV65	0.61842
P03410	Q8WYK2	0.37328
P03410	P22531	1.6653
P03410	O00505	1.2956
P03410	O75373	0.81868
P03410	P62736	0.24431
P03410	Q9NV56	1.3355
P03410	Q8IXP5	1.4883
P03410	Q96PK6	0.74209
P03410	Q8NEE0	1.6653
P03410	Q8TD31	1.3634
P03410	P13804	0.09967
P03410	Q99496	0.28112
P03410	Q96A04	1.7311
P03410	A1L4M7	0.078888
P03410	P48634	1.0663
P03410	Q9H5J8	1.5503
P03410	Q9P2T0	0.92315
P03410	Q14525	0.89153
P03410	Q9UI08	0.3599
P03410	A6PVY3	0.91041
P03410	O95780	1.0456
P03410	A8K0R7	1.0151
P03410	Q5VUA4	0.4411
P03410	Q86VQ1	0.60267
P03410	Q8N3J5	0.044314
P03410	A8K830	1.6653
P03410	P0CG30	0.30366
P03410	Q5RGN0	1.289
P03410	A6NL28	0.22746
P03410	Q8N5J2	1.8333
P03410	Q9NRL3	0.72585
P03410	B1ATL7	0.60462
P03410	P0C854	1.6653
P03410	Q5VWM4	0.16455
P03410	Q86VM9	0.27021
P03410	O43684	0.88695
P03410	A6NF01	1.4336
P03410	Q69YW0	0.38254
P03410	Q765I0	1.0305
P03410	Q8IYK2	1.5147
P03410	Q9UKX2	0.28865
P03410	P0CJ79	0.85175
P03410	Q9P000	0.53492
P03410	Q8N7Q3	0.78964
P03410	Q8N511	0.77232
P03410	Q7Z614	0.75291
P03410	Q6PEY1	1.5675
P03410	Q16658	0.40914
P03410	Q2T9F4	0.44202
P03410	Q6W349	1.7021
P03410	Q96P11	0.23202
P03410	Q9HCM9	0.35652
P03410	Q14929	0.79749
P03410	Q96HY6	0.68732

P03410	Q9NZ43	0.12755
P03410	Q9BY41	0.35229
P03410	P17482	1.0161
P03410	Q7Z6J9	1.0797
P03410	Q8TF68	0.34093
P03410	Q05BW5	0.43826
P03410	P06744	0.8875
P03410	Q9BXU0	0.57168
P03410	Q5T5N4	0.505
P03410	P47710	0.82017
P03410	Q96NU1	0.25632
P03410	Q8NE71	0.47764
P03410	Q9Y534	0.40869
P03410	Q9H3H3	0.49649
P03410	P54198	1.0457
P03410	A6NHN6	0.80973
P03410	Q96C34	1.1513
P03410	Q9H0V9	0.40711
P03410	P0CW22	0.39375
P03410	Q969T9	1.6505
P03410	Q5BIV9	1.3058
P03410	Q96H22	0.75254
P03410	O60508	0.43839
P03410	Q86WV1	0.83768
P03410	Q5SWL7	1.0219
P03410	A8MQ03	0.97401
P03410	Q49AS3	1.3612
P03410	P17861	0.88386
P03410	Q15329	1.1562
P03410	Q15038	1.9891
P03410	Q6W2J9	0.33187
P03410	Q9UJK0	1.6739
P03410	P59282	0.66076
P03410	Q9P0M9	1.662
P03410	P59861	0.87928
P03410	P01858	1.6653
P03410	Q9UEY8	0.32763
P03410	Q8NFI3	0.59389
P03410	O75293	0.67742
P03410	P07315	0.80389
P03410	Q86WS4	1.6653
P03410	P12036	0.7491
P03410	Q14749	0.61675
P03410	Q9Y2L5	0.40443
P03410	Q7L266	0.43315
P03410	Q8NBN3	0.87668
P03410	P78346	1.0744
P03410	Q9Y239	0.24128
P03410	Q9Y3B1	0.20886
P03410	Q9NQR7	1.6653
P03410	Q9BTE6	1.1442
P03410	Q9UPT5	0.88479
P03410	Q17RN3	0.8269
P03410	Q9NRD1	0.12221
P03410	Q6PCE3	0.91504
P03410	Q9UKV8	0.78335
P03410	Q8N3C0	0.24254
P03410	Q5VSG8	0.61939
P03410	Q9Y5Q9	0.30191
P03410	B3EWG3	1.4883
P03410	O75698	1.6653
P03410	Q15633	0.9658
P03410	P0CH99	1.1006
P03410	Q9Y2S6	1.6653
P03410	Q9H1C7	1.0913
P03410	P17040	0.74761
P03410	Q99497	0.11108
P03410	Q8NFG4	1.354
P03410	Q9H974	1.9791
P03410	Q8TD10	0.20917
P03410	Q8N9P6	1.6653
P03410	Q07866	0.95045
P03410	Q8NCU4	0.95044
P03410	O14599	1.6653
P03410	O95628	0.30652
P03410	P48643	0.19074

P03410	Q96NB1	0.42123
P03410	Q8TCN5	0.10142
P03410	Q15050	1.0724
P03410	Q7Z7L8	1.7857
P03410	Q96D15	0.57887
P03410	Q13409	0.8747
P03410	P04066	0.45659
P03410	Q8NG41	0.70327
P03410	P61925	0.24268
P03410	Q9UBC9	0.79049
P03410	Q9UHB9	0.48065
P03410	P62328	1.8163
P03410	P30622	0.51523
P03410	Q8IYN6	1.465
P03410	O95503	1.0306
P03410	Q8NHS1	0.87187
P03410	Q9NRI7	1.6653
P03410	Q96QC0	1.4118
P03410	Q16851	0.27495
P03410	C9JH25	1.0913
P03410	Q96H86	0.77496
P03410	Q8WWY3	0.4713
P03410	Q99766	0.94222
P03410	A8MX80	1.0341
P03410	P15621	0.97247
P03410	Q9BYQ2	1.9984
P03410	Q8WY50	1.6653
P03410	Q8TDG2	0.23477
P03410	Q6PJ61	1.3908
P03410	A6ZKI3	0.93458
P03410	Q5GH77	1.1084
P03410	Q8TC90	1.5218
P03410	Q8N1N4	0.8445
P03410	Q9NX08	0.6999
P03410	P08397	0.5653
P03410	Q9H6D7	0.81017
P03410	P18615	0.97781
P03410	P35754	0.31596
P03410	P02100	0.81619
P03410	Q9NPA5	0.33939
P03410	Q9BZ98	0.32566
P03410	Q8IV03	1.8055
P03410	Q8TEA8	1.5292
P03410	O94915	0.38071
P03410	Q5VXH5	0.16455
P03410	Q96M27	0.14694
P03410	Q8WYR1	0.4302
P03410	Q9Y614	0.23236
P03410	Q6QNY1	0.94845
P03410	Q53G26	0.88198
P03410	A6NGB9	0.54408
P03410	Q9BXS5	1.1578
P03410	P37275	0.61433
P03410	Q15637	0.27399
P03410	Q93062	0.79424
P03410	Q8N6Q3	0.62689
P03410	Q9NX77	0.76651
P03410	Q96KF7	1.0913
P03410	Q53R41	0.2112
P03410	Q96L92	0.92808
P03410	Q9HCE5	0.38209
P03410	Q8TEP8	1.1976
P03410	Q2TAC2	0.040425
P03410	Q8NI08	0.96682
P03410	P62244	1.364
P03410	Q9P2B7	1.4883
P03410	P36954	0.20146
P03410	Q13190	0.27453
P03410	Q8IZE3	0.24386
P03410	Q8N660	2.3694
P03410	Q99442	0.62154
P03410	Q9NVI1	0.98716
P03410	Q5TAB7	1.1098
P03410	P30279	0.93158
P03410	Q93015	1.4431
P03410	Q6ZVC0	1.4996

P03410	P13682	0.80547
P03410	Q8TAZ6	1.0218
P03410	Q8N688	0.73641
P03410	Q9H7H0	1.0021
P03410	P41162	0.20834
P03410	O43504	0.40881
P03410	P0C880	1.6653
P03410	Q9BT43	0.34816
P03410	Q96JM3	0.46478
P03410	Q9H0U9	0.54058
P03410	P12018	0.74569
P03410	Q9BYQ9	1.9984
P03410	P35080	0.77053
P03410	Q9H4T2	0.53429
P03410	Q9HAI6	0.97927
P03410	Q9BU70	0.44524
P03410	Q9NZ81	1.8834
P03410	Q9H3P2	1.4092
P03410	Q8N587	0.95607
P03410	Q5T7N8	0.78129
P03410	Q8WUN7	0.49184
P03410	Q9UL59	0.74466
P03410	Q96AB6	0.23238
P03410	Q9NPJ4	2.3089
P03410	A8MV24	0.75601
P03410	O00268	0.96617
P03410	Q69384	1.1647
P03410	Q96AY2	1.1599
P03410	Q6PRD7	1.6653
P03410	Q12983	0.311
P03410	Q8NEP4	1.4883
P03410	Q8NFZ5	0.97203
P03410	Q15047	0.46641
P03410	A6PW82	1.3071
P03410	Q9NWT6	0.31349
P03410	Q9H816	0.68476
P03410	Q8N8Y2	0.13015
P03410	Q86WR7	1.5141
P03410	Q3V5L5	0.64904
P03410	O43903	0.23201
P03410	Q6PI98	0.80969
P03410	Q8N4J0	1.5656
P03410	P36508	0.41637
P03410	Q5VWG9	0.79815
P03410	Q12996	0.24888
P03410	Q9P2Y5	0.67393
P03410	P58317	0.815
P03410	A1A4G5	1.6653
P03410	Q9UJ41	0.37672
P03410	Q9BQQ3	0.55074
P03410	O94964	1.2494
P03410	Q2M3G4	0.2529
P03410	Q5MIZ7	0.6468
P03410	Q9UBF9	0.39612
P03410	Q6ZS46	1.7008
P03410	Q96C28	0.8602
P03410	Q8TBZ6	0.56812
P03410	Q3KNV8	0.9101
P03410	Q8WTP9	1.879
P03410	Q8IYH5	0.53109
P03410	Q8N5H3	1.4883
P03410	Q9UKA4	0.29208
P03410	Q9BWD3	1.3085
P03410	Q8N7M2	0.96545
P03410	Q8N6I4	1.0913
P03410	Q96N38	1.0111
P03410	Q13072	1.0723
P03410	Q15388	0.40298
P03410	P53680	0.40491
P03410	P50995	1.0497
P03410	Q9BRH9	0.82588
P03410	P10916	0.28203
P03410	Q96N03	0.6999
P03410	O00442	0.6626
P03410	Q15029	0.39046
P03410	Q96S99	0.18531

P03410	Q5VUG0	0.51441
P03410	Q969H9	1.6653
P03410	O60841	0.60822
P03410	Q9BQS6	1.018
P03410	Q86SY8	1.6653
P03410	Q5TG92	1.2048
P03410	Q9H5P4	0.69162
P03410	Q52LR7	0.48407
P03410	Q9UJ04	0.37927
P03410	Q96G75	0.16544
P03410	Q9BPX1	0.34464
P03410	Q96SW2	0.60084
P03410	Q86XF7	1.0598
P03410	Q9HBQ8	1.594
P03410	Q13495	1.7282
P03410	Q8NFJ9	0.50538
P03410	O75787	0.45805
P03410	Q8N0T1	1.2581
P03410	Q15696	0.29969
P03410	Q8N7P3	0.64164
P03410	Q8N616	1.6653
P03410	Q9Y3A0	0.52963
P03410	Q6ZRZ4	1.8399
P03410	P49915	0.69581
P03410	Q16520	0.9338
P03410	Q9H1Y0	0.80342
P03410	Q9NYY8	0.53766
P03410	Q3MJ62	0.62675
P03410	Q8N6L1	0.86881
P03410	P37837	1.1572
P03410	P98169	0.2893
P03410	Q5VUB5	1.0285
P03410	Q96G30	0.82066
P03410	Q8TC76	2.3386
P03410	O75943	0.31307
P03410	Q15014	1.1962
P03410	Q9NP97	1.0254
P03410	A6NDX5	0.7891
P03410	Q8TAB5	1.4883
P03410	Q8N7N1	0.032704
P03410	Q13561	1.0928
P03410	Q8N729	0.73366
P03410	Q5T5M9	0.20917
P03410	Q9UNK9	0.33766
P03410	Q9H5Q3	0.67155
P03410	P47756	0.26252
P03410	Q9ULD0	0.43976
P03410	Q8N3V7	1.2102
P03410	Q86Z20	1.4804
P03410	Q8N1Y9	1.6653
P03410	P27816	0.80944
P03410	E5RJ46	1.6653
P03410	Q8NBT3	1.0913
P03410	P62913	1.3147
P03410	Q6ZVT0	0.088381
P03410	Q7L5D6	1.4965
P03410	Q96CB8	0.67054
P03410	P17661	0.81625
P03410	Q86TY3	1.0913
P03410	P26998	1.2726
P03410	Q8TEL6	0.7587
P03410	Q8WTZ3	0.92147
P03410	Q8WTR7	0.72741
P03410	Q701N2	1.6653
P03410	Q6UXP9	0.22981
P03410	Q9BRV3	0.28344
P03410	Q96JB2	1.0773
P03410	Q96CS4	0.78404
P03410	Q14CZ0	2.0397
P03410	Q8WVJ9	0.48134
P03410	Q92804	0.20186
P03410	P36871	0.096746
P03410	P61978	0.87726
P03410	Q6ZVN6	0.47834
P03410	O60610	0.30593
P03410	O43597	0.14039

P03410	Q9BY89	0.40714
P03410	Q5T953	1.6468
P03410	Q9Y3A4	0.71264
P03410	Q9BYQ6	1.9984
P03410	P0C7T5	0.71318
P03410	P78560	0.89703
P03410	Q6ZU67	0.51397
P03410	Q8TAC9	0.86361
P03410	O60535	0.86704
P03410	Q96NF6	1.0723
P03410	Q9UK99	0.33697
P03410	Q9HC36	0.11904
P03410	Q8IV48	0.17755
P03410	Q9H7P6	1.738
P03410	Q9UDV6	0.6439
P03410	Q9UP83	1.5062
P03410	Q8NBJ7	0.60048
P03410	O75822	1.1167
P03410	O15327	0.081297
P03410	Q9BU76	1.1731
P03410	P07438	0.83308
P03410	Q9H1A4	1.0872
P03410	Q8NFF5	0.77602
P03410	Q8WZ55	1.028
P03410	Q9H902	0.36984
P03410	Q96NB3	0.47186
P03410	Q9UPW5	0.20932
P03410	Q9H115	0.28958
P03410	O14929	1.0148
P03410	Q9H0B6	0.95827
P03410	Q04864	0.98902
P03410	P61583	1.6763
P03410	A6NM66	0.50136
P03410	Q5SSG8	1.1653
P03410	Q86XT2	0.45231
P03410	Q9GZY8	0.53425
P03410	Q8WYQ3	1.6264
P03410	Q9NWB1	0.33761
P03410	Q86T03	1.4569
P03410	Q6PJE2	0.1152
P03410	H3BNL1	0.89561
P03410	Q5BKU9	0.23031
P03410	Q96DB5	0.77425
P03410	Q2KJ03	0.4757
P03410	Q9HAZ2	0.37055
P03410	Q99676	0.78354
P03410	Q9BYQ8	1.9984
P03410	Q96F05	1.001
P03410	Q9BQ15	1.0864
P03410	Q92994	0.37847
P03410	O43612	0.7241
P03410	Q9BVC6	1.1514
P03410	Q9Y2I6	0.51745
P03410	Q6ZRH4	2.4261
P03410	O00139	0.51114
P03410	I3L3R5	1.6653
P03410	Q8TCC3	0.49843
P03410	Q9BYU5	0.84455
P03410	Q9P2D3	1.0123
P03410	Q9NPA0	0.79002
P03410	Q9NQ60	0.87407
P03410	Q8TE04	0.89332
P03410	O43303	1.2375
P03410	Q9NVV9	1.2804
P03410	Q9UGY1	0.54188
P03410	Q9UKX7	1.4032
P03410	O60896	0.19139
P03410	Q15773	1.3291
P03410	O60885	0.82633
P03410	Q16540	0.44122
P03410	Q9GZM3	0.26848
P03410	P15822	0.85015
P03410	Q8NA19	0.29677
P03410	Q3BBV1	2.3694
P03410	Q5SWL8	0.38376
P03410	Q9Y3F1	1.6653

P03410	Q3C1V1	1.4883
P03410	Q15010	0.39311
P03410	P55822	0.86291
P03410	O95990	0.63273
P03410	P02729	1.6653
P03410	Q9Y2Z0	0.50424
P03410	Q6UXV1	0.65923
P03410	Q96B86	0.85292
P03410	Q9H7R0	0.91401
P03410	P60468	0.62368
P03410	Q96K80	0.5219
P03410	Q7Z5B4	0.96234
P03410	Q9H8G2	1.5224
P03410	O96002	1.6653
P03410	O15151	0.37083
P03410	Q05048	0.90639
P03410	Q3LI60	1.6653
P03410	Q96FM1	0.79299
P03410	P49257	0.34484
P03410	P50502	0.79061
P03410	P38646	0.57084
P03410	Q9UI25	1.6653
P03410	P52738	0.72414
P03410	Q9Y2G9	1.0847
P03410	O95178	1.3025
P03410	Q14181	0.94877
P03410	Q8TBQ9	1.0378
P03410	O14896	0.28026
P03410	Q5T2W1	0.76729
P03410	Q96MB7	0.86177
P03410	O96004	0.35414
P03410	Q658Y4	1.0762
P03410	Q96M91	1.7161
P03410	A6NCS6	0.94764
P03410	Q9BSD3	0.53424
P03410	P31274	0.10955
P03410	Q9HBT8	0.74683
P03410	Q9Y2J0	0.27846
P03410	Q96A57	1.0913
P03410	Q00534	0.85559
P03410	Q16543	0.5628
P03410	Q9BQE9	1.5004
P03410	Q49AM1	0.63971
P03410	Q92698	0.33441
P03410	Q96B23	1.1296
P03410	Q86VP3	1.066
P03410	Q9NSA3	0.97242
P03410	Q15543	0.96536
P03410	Q9BWW4	1.1221
P03410	P25786	0.87265
P03410	Q16629	0.63071
P03410	Q13416	0.85468
P03410	Q66GS9	0.73254
P03410	Q9Y4R8	0.55327
P03410	P19419	1.3419
P03410	P20963	0.20197
P03410	Q9NR46	0.50565
P03410	Q3B7T3	0.64555
P03410	Q15973	0.67495
P03410	Q8N365	1.4883
P03410	P52740	0.65413
P03410	Q969Q6	0.49883
P03410	Q6UE05	1.0913
P03410	Q6PIZ9	0.90719
P03410	Q15652	0.41874
P03410	P35325	1.6653
P03410	P60328	0.53228
P03410	O95473	0.78861
P03410	Q8WU43	0.52536
P03410	Q9H9H4	1.3059
P03410	Q6NXP2	1.6336
P03410	Q9H840	0.90799
P03410	Q04726	1.0627
P03410	Q9NZF1	0.38834
P03410	Q6UX68	0.97104
P03410	Q96PY0	0.78737

P03410	A6NFY7	1.4792
P03410	P42772	0.95387
P03410	Q6P1L5	2.1063
P03410	P36542	0.48635
P03410	O15061	1.2197
P03410	Q99618	1.7432
P03410	Q8IYD8	0.41427
P03410	P12755	0.26969
P03410	Q9NW97	1.0913
P03410	Q6ZP82	0.6999
P03410	P60059	0.50214
P03410	Q8IYM0	0.24331
P03410	Q8N319	2.273
P03410	Q14684	0.80234
P03410	P0C866	0.26267
P03410	Q9H379	1.7569
P03410	Q8N910	0.27664
P03410	P78345	1.8345
P03410	Q9NXR1	0.45367
P03410	A8MVM7	1.6653
P03410	Q96FV2	1.1687
P03410	B3EWF7	1.6653
P03410	P29728	0.25262
P03410	Q96CP7	1.0913
P03410	Q9NVH6	0.7563
P03410	Q9H6K4	0.96244
P03410	Q9BZK3	0.56945
P03410	Q5JSQ8	0.34702
P03410	Q9Y4P9	0.56121
P03410	Q02083	0.3674
P03410	Q9BYT5	0.58088
P03410	Q6UX71	0.068298
P03410	Q15554	0.59493
P03410	Q9NYA3	0.61239
P03410	Q96G25	0.96855
P03410	Q9NV72	0.97478
P03410	Q15398	1.168
P03410	Q6PD62	0.65737
P03410	P31276	0.46104
P03410	Q02878	1.6855
P03410	Q8N7H1	0.10491
P03410	Q6IPX3	1.3164
P03410	P82970	0.95761
P03410	Q9NYK6	0.78456
P03410	Q8TEW8	0.26507
P03410	Q71RG6	1.6653
P03410	Q8NDH3	0.75002
P03410	Q9BXM0	0.77108
P03410	Q14549	0.32945
P03410	Q9P2R6	0.88795
P03410	P07741	0.86231
P03410	Q8N3X6	1.2763
P03410	Q76KX8	0.99306
P03410	O76003	0.32469
P03410	Q9UN79	0.84021
P03410	Q5T0B9	0.10461
P03410	Q9NWV8	0.85054
P03410	Q66K89	0.59436
P03410	Q08209	0.41487
P03410	Q9NX20	1.4557
P03410	Q9UL03	1.3822
P03410	A4D1B5	0.32371
P03410	Q15008	0.39544
P03410	Q8N865	0.60459
P03410	Q9HC77	1.1995
P03410	O75879	0.96664
P03410	Q99666	0.29442
P03410	Q9HAA7	0.31122
P03410	Q8TF09	1.0749
P03410	P63000	0.86861
P03410	Q5K130	0.70496
P03410	P17676	0.86593
P03410	Q13596	0.59117
P03410	P23511	1.0591
P03410	Q9BPZ3	0.88053
P03410	Q8IWE4	0.50682

P03410	Q92901	0.8783
P03410	Q7Z699	0.38598
P03410	Q8IZD9	0.31978
P03410	Q96KS0	0.24653
P03410	Q9Y6I3	0.18535
P03410	Q9H410	1.0047
P03410	Q96SZ4	0.68122
P03410	Q92900	0.45137
P03410	Q6ZN84	0.52485
P03410	Q8IUB9	1.6653
P03410	Q9BQ50	0.2384
P03410	Q9H7T9	1.1894
P03410	Q8NA57	1.5021
P03410	Q8WUB8	0.41999
P03410	Q580R0	0.25572
P03410	Q96AP0	0.52672
P03410	Q8N1N5	0.70821
P03410	P11166	0.55614
P03410	P22626	0.50625
P03410	Q9P016	1.1568
P03410	Q8IY42	1.5982
P03410	Q5T230	1.855
P03410	P49006	1.7584
P03410	Q16401	1.1541
P03410	Q5TDH0	0.27885
P03410	Q99996	0.96358
P03410	Q9Y2J4	0.44132
P03410	Q9NPD7	1.111
P03410	O76087	0.508
P03410	Q8IUC2	1.7887
P03410	Q9P0U4	0.23904
P03410	Q9GZL8	1.3756
P03410	P98182	0.6124
P03410	Q96LK0	0.45408
P03410	Q6ICL3	0.26159
P03410	O60895	0.18635
P03410	Q107X0	0.84432
P03410	Q1HG43	1.0805
P03410	O75380	0.0048409
P03410	Q8TD86	0.34414
P03410	Q9UI17	0.27046
P03410	Q9BXF9	0.40166
P03410	Q6ZUL3	1.6653
P03410	Q96LQ0	1.2113
P03410	Q9NRR5	0.7643
P03410	Q8TDD2	0.31472
P03410	Q6PGQ1	1.1769
P03410	Q9BQA9	0.27209
P03410	Q86TL2	0.99628
P03410	Q96ER9	0.45401
P03410	Q6ZWC4	1.0579
P03410	Q8N228	0.41575
P03410	Q8N726	0.849
P03410	Q6P3X8	1.3309
P03410	Q8WWB5	1.3651
P03410	O94855	0.64668
P03410	Q9BYV9	0.3558
P03410	Q5SXJ2	2.3694
P03410	Q8N159	0.49322
P03410	Q9BUT9	1.9652
P03410	Q96N11	0.80932
P03410	Q59EK9	0.72684
P03410	Q8N7B7	0.34276
P03410	Q9Y615	0.6605
P03410	O60806	0.39165
P03410	O75153	0.25148
P03410	O43678	1.0337
P03410	Q9P2X7	1.6653
P03410	Q96LK8	0.71076
P03410	P57077	1.0112
P03410	O96005	0.64556
P03410	Q5VYS4	0.31844
P03410	Q15233	0.52875
P03410	P54840	0.86524
P03410	Q8IY22	0.22065
P03410	Q8WVP5	1.626

P03410	Q8NG31	1.1392
P03410	Q9Y4P1	0.75491
P03410	P08238	0.47171
P03410	Q9UHU1	1.6653
P03410	Q9GZM8	0.17264
P03410	Q7Z5L0	0.92287
P03410	P0CJ75	1.4876
P03410	Q8IUB3	0.80669
P03410	Q8N680	0.10142
P03410	Q8N699	1.3203
P03410	P80297	0.83277
P03410	Q9NRF8	0.68083
P03410	Q9H329	0.31872
P03410	P11940	0.34797
P03410	P49589	1.0003
P03410	Q56VL3	1.1553
P03410	Q5T440	0.54984
P03410	P61568	0.65575
P03410	Q9H9D4	0.73955
P03410	Q504T8	0.5033
P03410	A6NGG3	1.6653
P03410	Q96SQ5	0.82084
P03410	Q96CP2	0.26697
P03410	A4GXA9	1.1455
P03410	Q15390	0.71733
P03410	Q5ST30	0.72956
P03410	Q96D09	0.23502
P03410	Q9NP98	1.1686
P03410	Q9UMQ3	0.75669
P03410	O94916	0.1711
P03410	P36639	0.36647
P03410	Q9NWW5	0.3631
P03410	Q9H4I2	0.53752
P03410	Q96JL9	0.83729
P03410	P18847	1.1497
P03410	Q8TDW5	0.24331
P03410	Q96N19	0.67663
P03410	Q9H6Z9	0.46485
P03410	Q9UI66	1.6653
P03410	Q9UJG1	1.3924
P03410	Q8N9R6	0.59251
P03410	P01106	0.38996
P03410	Q96PZ2	0.76712
P03410	Q9NZ52	0.97916
P03410	Q02447	0.56428
P03410	Q8NFP0	1.217
P03410	Q15527	0.64058
P03410	Q9UJ55	0.2347
P03410	Q9H607	0.63705
P03410	P16989	0.18141
P03410	Q8IZ21	0.57664
P03410	Q9NVP1	0.11218
P03410	F2Z333	1.0913
P03410	Q5T1N1	1.6465
P03410	Q9GZZ8	1.6653
P03410	O60551	0.51091
P03410	Q9BUJ2	1.2006
P03410	Q6PJT7	0.99776
P03410	Q5VZT2	0.67718
P03410	Q5H9B9	0.50578
P03410	Q8N4B1	0.42412
P03410	Q969X5	0.39597
P03410	Q8N661	1.1598
P03410	A6NGQ2	0.90395
P03410	Q9NVZ3	0.64276
P03410	P49427	0.44581
P03410	P63165	1.0266
P03410	Q5GH72	1.1702
P03410	Q5T4H9	1.6653
P03410	O95251	0.68851
P03410	Q08752	0.51764
P03410	Q92750	0.63486
P03410	B5ME19	0.46717
P03410	Q9Y2Z2	1.6429
P03410	Q96NJ1	1.6653
P03410	Q969J3	0.031951

P03410	Q96N68	1.6653
P03410	Q9C0D7	0.70642
P03410	Q13015	0.9647
P03410	O15372	0.86947
P03410	P07992	0.14252
P03410	P15880	1.2326
P03410	Q9H3K6	0.80253
P03410	Q15154	1.1201
P03410	Q96C11	0.40354
P03410	Q5CZC0	2.0654
P03410	Q99814	0.24787
P03410	Q9BTL3	1.5769
P03410	P25705	0.17437
P03410	Q9NV06	0.34567
P03410	Q8N1V2	0.76548
P03410	Q8NI27	1.1794
P03410	Q9NXW9	0.66131
P03410	Q9UI46	0.34897
P03410	P0DMB1	1.2368
P03410	O00267	1.1511
P03410	Q52MB2	1.4367
P03410	A1L3X4	1.6653
P03410	P17947	0.881
P03410	Q9Y5B9	0.32163
P03410	P0C860	0.43433
P03410	P62306	0.36164
P03410	Q16600	0.7557
P03410	P63272	0.9664
P03410	P60510	1.038
P03410	Q53HL2	1.0759
P03410	Q8N7Y1	1.6653
P03410	Q86W33	1.0992
P03410	Q9H5J4	0.86335
P03410	Q12968	0.50118
P03410	Q6ZS72	0.8493
P03410	Q9NP79	0.57928
P03410	P46777	1.3528
P03410	O95125	0.73036
P03410	Q9H606	1.1381
P03410	Q9BTV7	0.29155
P03410	Q06330	0.50244
P03410	O15315	0.20578
P03410	O60481	0.24427
P03410	P56378	1.1049
P03410	Q702N8	0.61236
P03410	O76042	1.0076
P03410	P46776	1.2786
P03410	Q6ZR03	0.97282
P03410	Q99502	0.078262
P03410	Q6UXV3	1.6653
P03410	P04264	0.72175
P03410	O14746	1.0439
P03410	Q9BXP5	1.1077
P03410	A2RUQ5	0.50889
P03410	Q96MT0	0.09736
P03410	Q9NZ32	0.22107
P03410	Q9HBH1	1.3262
P03410	O75409	1.1228
P03410	Q99575	0.85479
P03410	Q99549	0.57968
P03410	Q17RQ9	0.75648
P03410	Q7Z392	0.82325
P03410	Q06730	0.59461
P03410	Q5VSD8	0.72567
P03410	Q8NG48	0.18095
P03410	P98171	0.24532
P03410	Q96IR3	1.7067
P03410	Q9NUS5	0.84772
P03410	A6NGN4	0.44804
P03410	P62841	1.1141
P03410	P19429	0.18583
P03410	Q3LI54	1.6653
P03410	Q9BSB4	0.5304
P03410	Q96GE9	0.78116
P03410	P61567	0.94016
P03410	Q96BT1	0.11141

P03410	Q86X60	0.90046
P03410	P82675	1.4181
P03410	P52294	1.3605
P03410	Q08E93	1.6653
P03410	Q13057	0.56984
P03410	Q04837	0.83448
P03410	Q92670	0.61398
P03410	Q86U86	1.0888
P03410	Q5JNZ5	0.85912
P03410	Q5JUK9	0.51383
P03410	O15060	0.37181
P03410	Q13445	0.090673
P03410	Q96GM5	0.94885
P03410	Q9BSG1	0.87552
P03410	O14948	0.38434
P03410	Q6ZWJ1	0.31852
P03410	Q70EL1	0.67234
P03410	Q569K4	0.28241
P03410	Q9NWD9	0.77162
P03410	P54105	1.4195
P03410	Q9P217	0.25864
P03410	O15259	0.39162
P03410	P0C7Q5	1.1631
P03410	Q8NFH4	1.1317
P03410	O75251	0.49782
P03410	Q17RP2	0.11139
P03410	O60682	0.28848
P03410	Q8N1W0	1.6653
P03410	Q8N8C0	0.74317
P03410	H3BNL8	1.4409
P03410	P17812	0.62555
P03410	Q8WV60	0.48337
P03410	P30050	1.009
P03410	Q01860	0.54121
P03410	B7ZAQ6	0.86829
P03410	Q8N344	0.71946
P03410	Q8N9P0	0.92452
P03410	Q96F24	0.73973
P03410	P0CG23	0.62281
P03410	Q6AZW8	0.71108
P03410	Q9NR28	1.0739
P03410	P78318	1.3325
P03410	Q9BZD3	0.69628
P03410	Q96K30	1.1139
P03410	Q4L180	0.29921
P03410	Q86UW1	1.0785
P03410	Q5TYW8	0.22356
P03410	A6NC98	0.17347
P03410	O43736	0.69585
P03410	Q6ZMW2	0.75901
P03410	O75351	0.40027
P03410	Q6P3V2	0.6269
P03410	Q32P44	0.3132
P03410	P05413	0.47132
P03410	Q13077	0.59066
P03410	P24928	0.69868
P03410	Q16548	0.88202
P03410	Q9NRP2	1.5413
P03410	Q2M2W7	1.4883
P03410	O14548	0.15302
P03410	Q9NQ39	0.32994
P03410	Q6IPU0	1.3008
P03410	Q8N1I8	1.6653
P03410	Q902F9	0.82741
P03410	P53992	1.2356
P03410	Q8IVD9	0.8639
P03410	Q9BUW7	1.4883
P03410	Q9NS93	1.0003
P03410	P41218	0.54188
P03410	Q63ZY6	0.25335
P03410	Q96B42	0.83053
P03410	Q9Y2V7	0.35585
P03410	Q8N7X4	0.10775
P03410	Q9H1H9	0.91525
P03410	Q6ZMZ3	0.40445
P03410	Q6PF05	0.67321

P03410	O14497	0.63023
P03410	Q96PE7	0.19368
P03410	O14656	0.22634
P03410	A6NLJ0	1.6608
P03410	Q08AE8	0.40708
P03410	Q9BXH1	0.58528
P03410	Q15287	0.87701
P03410	Q53GL7	0.63163
P03410	Q5HYC2	0.31264
P03410	Q96MU5	1.3886
P03410	P15954	0.81885
P03410	Q8IX03	1.0815
P03410	Q9P260	0.27417
P03410	Q9UC06	0.71433
P03410	Q16517	1.1188
P03410	Q8N8G2	0.92657
P03410	Q8NB16	0.23158
P03410	Q8WTX7	1.0258
P03410	P49356	0.22025
P03410	Q8TCJ0	1.6171
P03410	Q9P299	0.59646
P03410	Q9UII5	0.77805
P03410	Q8N1B3	0.90855
P03410	B3EWG6	1.4883
P03410	Q8N8A8	0.68737
P03410	Q6IEG0	0.65992
P03410	A6NFI3	0.69737
P03410	Q15776	0.65578
P03410	Q8N6F8	0.042244
P03410	P60900	0.67235
P03410	Q8N8J7	0.72796
P03410	Q8NB50	0.81411
P03410	Q495Y7	0.24594
P03410	Q2NL98	1.5976
P03410	Q9UM07	0.76428
P03410	Q8WV44	0.24744
P03410	Q0JRZ9	0.31497
P03410	Q14264	0.61204
P03410	Q8WV07	0.63805
P03410	O96019	1.0085
P03410	Q9Y3C8	0.64371
P03410	P16118	0.2157
P03410	P0CW20	0.43173
P03410	A1L162	1.1749
P03410	Q8N292	0.96448
P03410	Q3SYG4	0.68671
P03410	Q2NL68	1.4883
P03410	Q8TDZ2	0.3052
P03410	Q6ZRP0	1.4883
P03410	P54274	0.56081
P03410	Q9UKD1	0.62603
P03410	P54845	0.34319
P03410	O43427	0.47959
P03410	P62310	0.71644
P03410	O75674	0.63971
P03410	P04733	0.80043
P03410	A6NCN8	1.4883
P03410	Q6TFL3	0.58949
P03410	Q96SA4	0.86137
P03410	P46020	0.49088
P03410	Q9UHD4	1.1026
P03410	O60811	0.10172
P03410	P55060	1.1746
P03410	Q5T3J3	1.1012
P03410	Q13011	0.13262
P03410	Q9UBP6	1.5947
P03410	Q9C0G0	0.23716
P03410	Q8WY22	1.3585
P03410	Q9BYR5	1.9984
P03410	A8MVW0	1.0285
P03410	P12035	0.892
P03410	Q9NY30	0.60299
P03410	O75419	1.1564
P03410	P25791	0.54942
P03410	O96020	1.2925
P03410	O75382	0.52435

P03410	Q96BN8	0.36541
P03410	Q9Y6Q3	0.67613
P03410	A6NC62	0.44221
P03410	Q15172	0.43604
P03410	Q9P2B4	0.65805
P03410	Q96S59	1.043
P03410	Q9ULH7	0.8542
P03410	O43747	0.96634
P03410	Q04917	0.48919
P03410	Q6UWT2	0.95938
P03410	P10073	0.86204
P03410	Q8IUE0	0.30793
P03410	Q92902	0.21402
P03410	Q8IYI0	0.78314
P03410	O95299	0.091218
P03410	P41220	0.3727
P03410	Q9UL18	1.1302
P03410	A9YTQ3	0.31758
P03410	Q495M9	0.58456
P03410	Q0D2J5	0.67869
P03410	P05388	1.5215
P03410	Q8NC69	0.32577
P03410	Q9BXF3	0.89154
P03410	Q9H0P7	0.58008
P03410	Q9ULK2	0.99972
P03410	Q96FT9	0.32977
P03410	Q6UXD1	1.0913
P03410	O76041	0.3686
P03410	P46013	0.60376
P03410	Q86UB2	1.2764
P03410	Q12962	1.2361
P03410	Q4KMX7	0.35337
P03410	Q9BW92	0.7115
P03410	Q9BQA5	0.76958
P03410	Q9Y316	0.80629
P03410	Q96G21	1.1399
P03410	Q8N983	0.78893
P03410	Q9UBT6	0.33408
P03410	Q9H2M3	0.1382
P03410	Q96MC2	0.39121
P03410	Q9BTP6	1.0837
P03410	Q6ZW61	0.39625
P03410	Q5U5X0	1.5911
P03410	Q5H9J9	0.5404
P03410	P21675	0.32436
P03410	Q9BXW9	1.0458
P03410	Q9GZU2	0.69118
P03410	Q9Y4I5	0.52394
P03410	Q8IXS2	0.28981
P03410	Q6QNY0	1.3541
P03410	Q9BYE7	0.61517
P03410	Q14997	0.65997
P03410	Q8IWF6	0.97346
P03410	Q13620	0.76257
P03410	O75031	1.223
P03410	O43516	0.51962
P03410	Q9BYG7	0.36709
P03410	P41212	0.69006
P03410	Q3ZCX4	0.83596
P03410	P16157	0.41861
P03410	A8MUX0	0.89429
P03410	Q03989	0.38374
P03410	Q9UQE7	0.24841
P03410	Q7Z7A1	1.5367
P03410	P09629	0.74143
P03410	Q14149	0.21803
P03410	O15235	1.1934
P03410	Q9Y5J7	0.51539
P03410	O15111	0.86614
P03410	Q9ULZ2	0.97937
P03410	Q6UX46	1.0597
P03410	Q8N1L1	1.6653
P03410	Q8NFV5	1.0907
P03410	P29083	1.2676
P03410	Q9NQZ2	0.38228
P03410	P06730	0.77526

P03410	Q96IL0	0.50222
P03410	Q8N5U0	1.6653
P03410	Q5SY85	1.3881
P03410	Q6NZY4	0.57507
P03410	Q9NVV5	0.899
P03410	Q96K31	1.1734
P03410	Q6ZN03	0.65094
P03410	Q9UHD9	0.99639
P03410	Q10571	1.0543
P03410	O95867	0.78494
P03410	P15941	0.37593
P03410	O75334	0.82568
P03410	Q14686	1.3181
P03410	P0CG35	1.2621
P03410	Q70J99	0.3049
P03410	Q9Y2B2	1.3624
P03410	Q14938	0.20768
P03410	A2RUB1	1.4883
P03410	Q8TAW3	0.81318
P03410	Q8WTW4	0.57619
P03410	H7BZ55	0.014642
P03410	Q9NQ87	0.35773
P03410	Q9ULM6	0.84085
P03410	Q9BU23	1.1144
P03410	Q9NQX6	0.8415
P03410	Q17RH7	1.6653
P03410	Q6ZQY7	1.5078
P03410	Q9H3E2	0.31388
P03410	Q12765	1.0127
P03410	Q8N4W9	0.79465
P03410	Q9NWF9	0.71965
P03410	Q8IUZ0	0.3132
P03410	Q96BT3	0.66857
P03410	O00198	1.5526
P03410	Q8N4C9	1.0913
P03410	Q9BT09	0.329
P03410	Q13098	0.32552
P03410	Q8NCR6	0.80692
P03410	P18848	1.1106
P03410	Q8N0X2	0.3845
P03410	Q0VFZ6	1.4883
P03410	Q1ZZU3	0.93128
P03410	Q8N138	0.61153
P03410	Q567V2	0.49581
P03410	Q69YN2	0.396
P03410	A7E2U8	0.70026
P03410	Q6ISU1	0.677
P03410	P42575	0.34318
P03410	Q8NAX2	1.4883
P03410	P25685	0.32461
P03410	Q9UPR3	1.0333
P03410	Q9UQ80	0.93301
P03410	Q8IVJ8	1.4391
P03410	Q5T871	1.6653
P03410	Q86SX3	1.8247
P03410	Q8N3J2	0.37536
P03410	Q9H0A0	0.68929
P03410	Q8NDY4	1.6653
P03410	P36402	0.44373
P03410	Q5H9L2	1.1794
P03410	Q6UWW9	0.70288
P03410	Q5TAX3	0.71877
P03410	Q9NQ35	1.1788
P03410	P51970	0.12508
P03410	P0DI80	1.0029
P03410	P22528	1.0668
P03410	O75427	0.86318
P03410	A6NIY4	1.0907
P03410	Q92614	0.29095
P03410	Q9UHK0	0.45838
P03410	Q96KR1	0.66409
P03410	A3KN83	1.3611
P03410	P35998	0.47844
P03410	P50222	0.46372
P03410	Q5VWM1	0.62283
P03410	O60502	0.14816

P03410	Q8TAQ5	0.79465
P03410	Q6P2C0	0.66607
P03410	Q14242	1.1851
P03410	Q6YHU6	0.11156
P03410	A5A3E0	0.23109
P03410	Q96RY7	0.053425
P03410	Q15750	0.87426
P03410	Q86Y97	0.3482
P03410	Q49B96	0.77301
P03410	Q9UJ78	0.37759
P03410	O75843	1.0744
P03410	Q9Y3M2	0.90455
P03410	O95777	0.81336
P03410	Q9NVM9	1.2985
P03410	A8MXZ1	0.13951
P03410	P0C2S0	0.96395
P03410	Q6NXT1	0.45927
P03410	Q6T4R5	0.73414
P03410	Q96GX2	1.1594
P03410	Q96A33	0.38444
P03410	P51965	0.4251
P03410	O43920	1.2113
P03410	Q8N6I1	1.3174
P03410	Q6AWC8	1.3597
P03410	Q96FA3	0.87342
P03410	P06396	0.82816
P03410	Q8NEX6	0.89879
P03410	Q9NPI6	1.0308
P03410	Q7Z7B8	0.73641
P03410	Q05682	0.2945
P03410	Q3LI70	1.6653
P03410	Q96AQ7	0.95426
P03410	Q9H1A7	0.26848
P03410	P61421	0.18179
P03410	Q9NVD3	0.07018
P03410	Q14692	0.54593
P03410	Q9BQM9	1.0029
P03410	Q9H1E3	1.5238
P03410	P0CG34	1.2621
P03410	Q8N6M5	0.48736
P03410	Q7Z3Z0	1.0001
P03410	Q96S95	1.2466
P03410	A1L168	1.4883
P03410	Q13526	0.64818
P03410	P46781	1.2398
P03410	Q68D91	0.10995
P03410	A8MTA8	0.46415
P03410	Q15393	0.14305
P03410	Q12959	0.98743
P03410	P54277	0.14347
P03410	Q9UI09	0.1603
P03410	P86481	1.6653
P03410	Q7Z739	0.9812
P03410	Q32Q52	0.71412
P03410	Q96M43	0.65891
P03410	Q9ULV0	0.22368
P03410	P50221	0.38688
P03410	Q15173	0.53955
P03410	Q9Y3C1	0.32112
P03410	Q8IYT2	0.87583
P03410	Q86U90	1.2732
P03410	Q9BUL8	0.95728
P03410	Q9Y2N7	0.45563
P03410	Q13503	0.76004
P03410	Q9Y536	0.1777
P03410	Q6NSI8	0.90597
P03410	O15266	0.40545
P03410	P60412	1.9984
P03410	Q9C0D0	0.62535
P03410	Q0IIN9	1.1125
P03410	Q14019	0.46734
P03410	Q9Y2D1	0.99797
P03410	Q13609	0.21536
P03410	Q9P1Y5	1.0348
P03410	Q9UBI1	0.6999
P03410	A0PG75	0.13955

P03410	Q8WUU5	0.25665
P03410	Q9UH99	0.35444
P03410	Q9H857	0.82263
P03410	O75446	1.752
P03410	Q5T4T1	0.78097
P03410	Q12815	0.73038
P03410	Q9Y255	0.51635
P03410	Q6PJG2	0.83412
P03410	Q2M3A8	0.73916
P03410	Q99460	0.6072
P03410	Q9UGP4	0.4184
P03410	P48553	0.30529
P03410	Q52LJ0	0.66632
P03410	Q8N9M1	0.6542
P03410	Q0VF96	0.27972
P03410	Q16655	1.5299
P03410	P18124	0.99049
P03410	Q14153	1.2127
P03410	Q9H1F0	0.77619
P03410	Q96B45	0.18078
P03410	O60749	0.47783
P03410	O76027	0.02554
P03410	O43542	0.579
P03410	Q4LEZ3	1.6298
P03410	Q8TD17	0.75838
P03410	Q13505	1.431
P03410	Q2VPJ9	1.4883
P03410	Q8IV36	0.8986
P03410	Q9NS73	0.89881
P03410	O60282	0.38415
P03410	Q8NHU6	0.28898
P03410	A8MUU9	0.45106
P03410	O15069	0.54734
P03410	Q8N3J9	0.74326
P03410	Q9BVG4	0.60889
P03410	Q2T9K0	0.9489
P03410	Q9NWW0	0.34399
P03410	Q7Z7N9	0.98082
P03410	Q9NSK7	1.1852
P03410	Q9BXW7	0.24652
P03410	Q6UVJ0	1.2095
P03410	Q3KP31	0.94919
P03410	Q9Y2B4	0.53496
P03410	O00400	0.73352
P03410	Q9BYK8	0.37024
P03410	P49588	0.30881
P03410	A6NNH2	0.12769
P03410	Q9Y224	1.5835
P03410	Q9Y5S9	0.67953
P03410	Q9BR09	0.25726
P03410	Q969E1	1.0339
P03410	Q7L190	1.5508
P03410	P13667	0.43994
P03410	Q16831	0.31141
P03410	Q96QU8	1.1982
P03410	Q96N77	0.62157
P03410	Q8N6T7	0.72532
P03410	Q9P0V3	0.63011
P03410	Q5VW36	0.16654
P03410	P60520	0.46677
P03410	Q96Q45	0.82848
P03410	Q8WW34	0.63587
P03410	Q6PG37	1.0232
P03410	Q7Z5K2	0.81966
P03410	Q8NCN2	0.2267
P03410	Q9Y3C4	0.90083
P03410	Q6PI26	1.5078
P03410	P00374	0.2008
P03410	O75528	0.6475
P03410	O94905	0.45871
P03410	Q06265	0.30953
P03410	Q8N8U3	0.44889
P03410	Q5JXX7	1.0691
P03410	Q9UJX4	0.98786
P03410	P14651	0.18795
P03410	P04053	0.19005

P03410	Q96L16	0.084763
P03410	Q9H579	0.097446
P03410	Q9BUU2	0.20599
P03410	Q7KZ85	0.51779
P03410	P0C6T2	0.7621
P03410	P54136	1.1302
P03410	Q8NB91	0.47243
P03410	Q96A35	1.3245
P03410	Q53GA4	0.19863
P03410	Q9NTK5	1.1396
P03410	P10242	0.92961
P03410	Q8IYB9	0.81404
P03410	Q8WWA1	1.0913
P03410	Q9NPF4	0.36893
P03410	P53567	0.53566
P03410	C9JLR9	1.2834
P03410	Q8N5N4	0.96443
P03410	Q9UIF8	0.8153
P03410	Q147U7	0.72853
P03410	Q9NYG5	0.86822
P03410	Q9Y228	0.068298
P03410	Q9NPF5	1.0498
P03410	Q4G0G5	0.74176
P03410	Q96DX5	1.088
P03410	O43639	0.55832
P03410	Q8WV15	1.0913
P03410	Q9BWT1	1.584
P03410	Q86YW9	0.45739
P03410	Q9BWX1	0.3184
P03410	Q9NR11	0.68927
P03410	A6NEA5	0.83701
P03410	Q9H1H1	0.65282
P03410	Q6ZQX7	0.036031
P03410	Q5SRE5	0.89906
P03410	P54802	0.68395
P03410	Q8N3X1	0.54319
P03410	Q9Y605	2.2462
P03410	Q8N6F7	0.97837
P03410	Q5T6V5	0.93562
P03410	P05141	0.26861
P03410	Q69YI7	1.7589
P03410	O96000	0.81371
P03410	A1L020	0.26003
P03410	Q9NXH3	1.0879
P03410	O15405	1.0064
P03410	O14646	0.51397
P03410	Q9H8S9	0.66853
P03410	Q9BZP3	0.72658
P03410	O43759	0.90984
P03410	O60299	0.67935
P03410	Q96BP3	0.20771
P03410	Q9BYE4	1.6653
P03410	O14810	0.5354
P03410	Q92688	0.34399
P03410	Q7Z4S6	0.34531
P03410	Q8NFH8	0.7367
P03410	Q9H2K0	1.1107
P03410	Q9HBL7	1.1105
P03410	O75152	0.5219
P03410	H3BR10	1.6653
P03410	Q9H0W9	0.77228
P03410	P24522	0.76114
P03410	Q9NTM9	0.25524
P03410	Q9UL33	0.61893
P03410	Q8NA70	0.16338
P03410	P48147	0.67095
P03410	Q9UHL9	0.69214
P03410	Q14493	1.0337
P03410	Q6NUI1	1.3557
P03410	Q15013	1.1327
P03410	O00165	1.1538
P03410	Q30KQ1	0.87928
P03410	Q19AV6	1.9595
P03410	P35789	0.90214
P03410	P14859	1.1389
P03410	Q96LC9	0.84797

P03410	Q8NCQ2	1.2436
P03410	Q8IYW2	1.6653
P03410	O95751	0.86625
P03410	O75582	0.28392
P03410	Q4AC94	0.25756
P03410	Q8N5I9	1.4883
P03410	P0C841	1.6653
P03410	P48444	0.74592
P03410	Q96P70	1.2762
P03410	Q9NVC6	0.59766
P03410	Q9GZU7	0.66586
P03410	P27348	0.55889
P03410	Q76FK4	0.3166
P03410	P10124	0.95282
P03410	A6NEW6	0.6566
P03410	O43561	0.29152
P03410	Q6IB77	0.55374
P03410	Q6ZSN1	0.91891
P03410	Q99717	0.41977
P03410	Q9NVK5	0.40492
P03410	Q8WXI9	0.76216
P03410	P20042	0.63452
P03410	Q9H0G5	0.79509
P03410	P52948	1.2269
P03410	Q5JSZ5	1.4047
P03410	Q12800	0.23677
P03410	Q8NF99	0.69684
P03410	Q9H649	0.23292
P03410	Q14814	0.37704
P03410	Q7Z4L9	0.28755
P03410	Q7Z4T9	1.0177
P03410	Q16352	0.86974
P03410	P57723	0.16083
P03410	P02686	0.64727
P03410	P78545	0.55398
P03410	Q99608	0.26816
P03410	Q6ZRR9	0.39083
P03410	Q96CB9	0.96335
P03410	P35527	1.0001
P03410	Q96AW1	0.83916
P03410	Q8IYX0	1.0067
P03410	Q9UH64	0.7135
P03410	Q96RP9	1.0302
P03410	Q96MK2	0.29951
P03410	Q8N6V4	0.5111
P03410	Q86V87	0.83059
P03410	B5MD39	0.085776
P03410	Q9HAU0	0.48727
P03410	Q6NSJ2	1.6045
P03410	Q86WW8	0.94546
P03410	Q9UL49	1.1621
P03410	Q9Y3I1	0.85007
P03410	Q9H7E9	1.4883
P03410	Q8TBB1	0.57663
P03410	Q5VZF2	0.20449
P03410	P41238	0.10794
P03410	Q8N9N5	0.68102
P03410	Q16719	0.27624
P03410	Q8IVF1	1.4622
P03410	Q7L2R6	0.96412
P03410	Q6P9F0	0.62782
P03410	P11532	0.60408
P03410	Q6P1N9	0.60747
P03410	Q8WZA9	1.4883
P03410	Q16222	0.17511
P03410	Q14410	0.87046
P03410	O00233	0.86116
P03410	Q9P2F5	0.67541
P03410	Q9C0C2	0.49081
P03410	Q99614	0.53164
P03410	B9EJG8	0.75535
P03410	Q8WWL7	1.146
P03410	O76075	0.3507
P03410	O60756	1.6653
P03410	Q7L1V2	1.4967
P03410	Q8N998	0.45518

P03410	O75030	0.42376
P03410	Q96K62	0.53003
P03410	Q9NZC7	0.37395
P03410	Q96P63	0.25437
P03410	Q8TAM2	0.47766
P03410	Q8WWK9	0.6388
P03410	Q96PQ6	1.0175
P03410	Q8IWT0	0.26593
P03410	O15504	0.65938
P03410	Q8IXQ5	0.25815
P03410	Q96HG1	1.6653
P03410	Q14008	1.2036
P03410	Q6P444	1.3102
P03410	P35637	0.40119
P03410	Q05BU3	1.6653
P03410	Q8TEE9	1.4571
P03410	Q53TQ3	0.64817
P03410	Q9BWH2	0.1757
P03410	Q5TCQ9	0.93922
P03410	Q7Z5G4	0.1687
P03410	Q86WZ6	0.68442
P03410	Q96SN8	0.74077
P03410	Q9UPG8	0.33286
P03410	P61604	1.3561
P03410	Q9NRQ2	0.47014
P03410	Q9BSE2	1.0913
P03410	P15884	0.53384
P03410	O14862	0.59209
P03410	O75792	1.6645
P03410	Q8N6N3	0.87287
P03410	Q6ZN11	0.72473
P03410	Q15257	0.94039
P03410	Q8TD35	1.4883
P03410	Q9BV29	0.2208
P03410	Q9H8U3	0.32044
P03410	Q9NYT6	0.6829
P03410	Q7Z5L9	1.2718
P03410	Q9UJV8	0.10622
P03410	Q75VX8	1.5415
P03410	Q14653	0.65317
P03410	Q9BZE7	1.4883
P03410	P51946	0.69495
P03410	P11844	1.3539
P03410	Q9Y6X9	0.2349
P03410	Q5TZJ5	1.4013
P03410	Q96AN2	0.6062
P03410	Q8N1B4	1.0521
P03410	O75444	0.36108
P03410	Q8IYT3	0.027411
P03410	A6NNX1	0.43421
P03410	Q9BPZ7	1.0058
P03410	P68543	0.5483
P03410	Q8N3L3	1.2202
P03410	Q14207	0.8428
P03410	Q9H773	1.0193
P03410	Q5VSL9	1.356
P03410	Q5VVC0	0.5928
P03410	P32929	0.33674
P03410	P51861	1.0228
P03410	Q92610	0.14227
P03410	Q9NTX9	1.0744
P03410	Q8NBB4	0.61733
P03410	Q6P1Q0	0.16303
P03410	Q9NRR3	0.77651
P03410	Q86Y79	1.4975
P03410	Q8TEK3	0.48891
P03410	Q9NWU2	0.20917
P03410	Q9BYR0	1.9984
P03410	Q96Q06	1.4352
P03410	O43187	0.29
P03410	Q9Y5Y5	0.55303
P03410	Q8WY54	0.83229
P03410	Q9Y3P4	1.1052
P03410	Q3LI83	2.1124
P03410	Q8N1A6	0.065434
P03410	P28324	1.3623

16493	P03410	Q8WZA0	0.33117
16494	P03410	Q9UNA4	0.39191
16495	P03410	P19013	0.93738
16496	P03410	Q9BVW5	1.1029
16497	P03410	Q5RKV6	0.13996
16498	P03410	Q12857	0.23948
16499	P03410	Q2T9L4	0.65133
16500	P03410	Q5H9I0	0.33183
16501	P03410	Q8TF50	0.77149
16502	P03410	P57055	1.001
16503	P03410	P49406	1.1457
16504	P03410	Q16254	0.90249
16505	P03410	O75167	0.56973
16506	P03410	P17017	0.66045
16507	P03410	Q8NBT0	0.30502
16508	P03410	Q8TEQ6	0.78524
16509	P03410	Q9UM13	0.9681
16510	P03410	Q496H8	1.2423
16511	P03410	Q5JUX0	0.26652
16512	P03410	P57060	0.7544
16513	P03410	Q8TF45	0.66065
16514	P03410	Q6F5E7	0.38564
16515	P03410	P10645	0.67306
16516	P03410	O94811	1.0055
16517	P03410	Q16612	0.57157
16518	P03410	Q86V59	0.82274
16519	P03410	Q8TCY9	1.0174
16520	P03410	Q15365	1.0744
16521	P03410	Q9NPB0	1.0017
16522	P03410	Q9Y5U4	0.27822
16523	P03410	Q9NRN9	0.57023
16524	P03410	P53367	0.15567
16525	P03410	Q92558	0.74927
16526	P03410	Q6ZRM9	1.3824
16527	P03410	Q8WUE5	0.3445
16528	P03410	Q08AF8	0.93642
16529	P03410	Q16236	0.51089
16530	P03410	Q96KN7	0.34187
16531	P03410	A6NP61	1.1579
16532	P03410	Q9BQE6	1.3642
16533	P03410	O75474	1.7179
16534	P03410	Q5VV11	0.2232
16535	P03410	Q8WXF5	0.9994
16536	P03410	Q5T5A8	1.6653
16537	P03410	D6RGH6	1.2671
16538	P03410	Q8IWA0	0.36709
16539	P03410	Q9BTE3	0.82434
16540	P03410	Q14210	0.57968
16541	P03410	Q9NPC3	0.068285
16542	P03410	Q9H9K5	0.79444
16543	P03410	Q3C1V8	0.54546
16544	P03410	P07902	0.97267
16545	P03410	Q8TF46	0.37675
16546	P03410	O15519	0.52788
16547	P03410	Q9UKK9	0.74717
16548	P03410	Q9P287	0.87165
16549	P03410	Q10567	1.0029
16550	P03410	Q12830	0.77497
16551	P03410	Q9H841	1.0846
16552	P03410	O14713	1.6418
16553	P03410	P30040	0.14125
16554	P03410	Q9BY42	1.1233
16555	P03410	Q8TEA1	0.56813
16556	P03410	Q8NEG2	1.0771
16557	P03410	Q5T750	0.63747
16558	P03410	Q8NFT6	0.30027
16559	P03410	Q9BV81	1.0583
16560	P03410	Q8WVZ9	0.54253
16561	P03410	Q9NZW5	0.44034
16562	P03410	P14625	0.2371
16563	P03410	Q2M1V0	0.30793
16564	P03410	Q96HH4	0.83382
16565	P03410	Q5VIY5	0.96412
16566	P03410	Q15468	0.94078
16567	P03410	Q969S3	0.85336
16568	P03410	Q9BZE3	0.46674

P03410	O95954	0.65043
P03410	Q9Y312	0.50977
P03410	Q9BRA0	0.34399
P03410	Q6WBX8	0.81676
P03410	Q9H7T3	1.3157
P03410	Q96ND8	1.038
P03410	O95816	0.62931
P03410	Q14978	1.2719
P03410	P11168	0.30155
P03410	P23381	0.61419
P03410	Q9NUB4	1.6653
P03410	Q9H0I3	0.59946
P03410	P46199	0.91911
P03410	Q6NSH3	1.0088
P03410	Q9NPJ6	1.0146
P03410	Q5JYT7	0.25643
P03410	P04424	0.20822
P03410	Q701N4	1.6653
P03410	Q9GZN1	0.23477
P03410	Q5BKU6	0.44351
P03410	O00501	0.55833
P03410	Q5T8I3	0.85705
P03410	Q9BTC8	0.56106
P03410	P0CL85	0.45056
P03410	Q04446	0.59731
P03410	Q9P2S5	0.32809
P03410	Q13490	0.30968
P03410	Q6IEE8	0.69139
P03410	O60870	0.94642
P03410	Q92665	1.2242
P03410	Q8IYI8	0.84198
P03410	Q96FZ2	1.7028
P03410	A6NGR9	1.0935
P03410	O75324	1.1749
P03410	Q5W0A0	0.52132
P03410	Q9UPQ4	0.23772
P03410	Q5T699	0.17304
P03410	Q8WZ19	0.043784
P03410	Q5JRC9	1.2691
P03410	Q9UNL4	0.52364
P03410	Q00059	0.8426
P03410	Q8TAT8	1.1422
P03410	Q969E2	0.8202
P03410	Q9UEU5	0.67218
P03410	Q8IW36	0.88144
P03410	P62136	0.57639
P03410	O94868	0.6999
P03410	Q92905	0.62977
P03410	Q86X83	0.82654
P03410	Q9BYD2	1.7377
P03410	Q8IWV1	0.68585
P03410	P35606	0.71606
P03410	P24278	0.68641
P03410	Q7Z6B0	1.0913
P03410	Q53EQ6	0.76506
P03410	Q86UA6	1.6889
P03410	Q8NCR3	0.46557
P03410	O75143	1.8748
P03410	Q9HCJ0	0.62684
P03410	O95931	0.91143
P03410	Q5JQC9	0.50708
P03410	Q9BQ13	0.32577
P03410	Q9NXK8	0.84102
P03410	Q9Y333	0.76454
P03410	Q8WV28	0.36143
P03410	Q9UBI9	1.4354
P03410	Q5VZQ5	1.2619
P03410	Q2M218	0.82417
P03410	Q14533	1.329
P03410	O60716	0.41173
P03410	P06733	0.56726
P03410	Q8TAA5	1.0396
P03410	Q9Y543	0.26176
P03410	Q96B18	0.46891
P03410	Q6P2D0	0.76665
P03410	Q9Y2H0	0.24203

P03410	Q5SQN1	0.63061
P03410	Q9P0W2	0.55824
P03410	A6NEV1	1.3289
P03410	Q8WXE1	1.0035
P03410	Q9UMS5	1.4113
P03410	Q9BUX1	0.95667
P03410	O00232	0.60013
P03410	Q4G112	0.30793
P03410	O43679	0.41848
P03410	Q5T8I9	0.40226
P03410	Q9NRA8	1.2977
P03410	Q5T215	0.6145
P03410	Q9BSK0	1.4953
P03410	O75182	1.1521
P03410	P42695	0.51865
P03410	Q8WTS6	0.38764
P03410	Q96PV6	0.97166
P03410	P0CF75	0.56656
P03410	Q6PJG6	1.3248
P03410	Q15513	1.6653
P03410	A0AVF1	0.28197
P03410	P58107	0.30536
P03410	P36405	0.34328
P03410	O15182	0.6714
P03410	Q9BYX4	0.31295
P03410	Q5JRM2	1.1653
P03410	Q13867	0.36976
P03410	Q14CW9	1.0222
P03410	Q08ER8	0.89343
P03410	Q8N339	0.19611
P03410	Q9NV79	0.18038
P03410	O15198	0.4362
P03410	Q8TF39	1.0409
P03410	Q9Y6R9	1.658
P03410	P13985	1.6653
P03410	Q9NRX3	1.2747
P03410	Q5FWF6	0.91808
P03410	Q9NXZ1	0.41837
P03410	Q9H6J7	0.64244
P03410	Q99627	0.4974
P03410	Q9HAB3	1.0772
P03410	A8MS94	0.65699
P03410	Q9P2K5	0.22349
P03410	Q9NY61	0.94965
P03410	Q8N307	1.0867
P03410	P12694	0.053256
P03410	Q6NXN4	0.8698
P03410	Q14457	0.81958
P03410	Q9NPJ3	0.24898
P03410	Q9UK11	0.89291
P03410	Q75L30	1.6653
P03410	Q5BKY1	0.41169
P03410	Q9ULG1	0.35758
P03410	Q17R26	0.70451
P03410	E7EW31	1.6653
P03410	Q8NHP7	0.48044
P03410	A8MT65	0.97734
P03410	P62269	1.1903
P03410	Q9HCI7	0.46639
P03410	Q9Y586	0.31897
P03410	Q6ZVD7	0.37111
P03410	Q8N1X5	1.6653
P03410	Q86WG3	0.54734
P03410	P59090	1.6653
P03410	Q13425	1.0054
P03410	P28289	0.33651
P03410	Q92572	0.8158
P03410	A6NCN2	0.86808
P03410	P63172	1.1077
P03410	Q5JQC4	1.6653
P03410	O95273	1.1536
P03410	Q8IVI9	0.42076
P03410	P07919	0.32504
P03410	Q8NG50	0.29422
P03410	Q4G0A6	1.5332
P03410	O15544	0.0528

P03410	O15145	0.48094
P03410	A6NC51	0.75535
P03410	Q92545	1.0913
P03410	Q9H9L4	0.7221
P03410	P23610	1.6653
P03410	Q5VYK3	0.47282
P03410	Q8TDY2	0.83213
P03410	Q9BTX9	1.6849
P03410	Q6H8Q1	0.39108
P03410	Q9NSK0	0.36592
P03410	A6NHQ2	0.30626
P03410	Q9BQ52	0.50848
P03410	O96011	0.38137
P03410	Q8NDV7	0.43059
P03410	Q01534	0.43746
P03410	P20700	0.81811
P03410	Q96RS6	0.34399
P03410	O95050	0.06765
P03410	Q9NZY2	1.6653
P03410	Q5JTB6	1.0723
P03410	Q7Z465	0.95377
P03410	Q9UBU8	0.75956
P03410	Q8N4S0	1.4883
P03410	Q92845	0.41584
P03410	O00273	0.45406
P03410	Q8IV33	1.1305
P03410	O43918	0.87258
P03410	O00401	0.61634
P03410	Q7Z4G4	0.32816
P03410	Q5TD94	0.63308
P03410	O43325	1.4003
P03410	P35573	0.45649
P03410	O00159	0.1671
P03410	P55273	0.2967
P03410	Q9H4B0	1.1029
P03410	Q8N9T8	1.9303
P03410	A6NHJ4	1.0268
P03410	Q8IYA6	1.6361
P03410	Q8N5W8	0.95938
P03410	Q68DU8	0.25004
P03410	Q9BXD5	1.1494
P03410	Q9ULJ3	0.66168
P03410	Q5HYM0	0.27658
P03410	P50748	0.97172
P03410	O75969	0.64883
P03410	Q13515	0.74132
P03410	O97980	1.1653
P03410	Q13956	0.84351
P03410	Q5C9Z4	0.79437
P03410	Q6XLA1	0.034477
P03410	Q3LI62	1.6653
P03410	Q2M238	0.31905
P03410	P33947	0.64571
P03410	Q14657	1.5883
P03410	Q8N7X2	1.6653
P03410	A6NCI8	1.461
P03410	Q8WWR9	1.0332
P03410	Q9NVM4	0.16796
P03410	Q9Y6F6	0.80953
P03410	Q9BX70	0.89382
P03410	P0CB33	0.95014
P03410	Q8WW27	0.4304
P03410	Q9BYI3	1.4747
P03410	P20809	0.98812
P03410	Q9NXT0	0.67571
P03410	Q9NRY5	1.6349
P03410	Q9NYP9	0.65218
P03410	Q99732	0.63137
P03410	P60409	1.9984
P03410	P60370	1.9984
P03410	Q8NDZ0	0.43175
P03410	P31249	0.45931
P03410	Q9BWG4	0.12952
P03410	Q53RY4	1.0913
P03410	Q9BYQ7	1.7937
P03410	Q9BU19	0.10142

P03410	Q6ZMR3	0.66514
P03410	Q86SE9	1.0443
P03410	Q9H5X1	0.33473
P03410	Q9H813	0.80118
P03410	Q8N0U4	0.77029
P03410	Q9Y6X2	0.64093
P03410	Q96JM2	0.30084
P03410	Q96BY2	0.78244
P03410	Q15847	1.6653
P03410	Q9Y692	0.48369
P03410	Q7L1Q6	0.50635
P03410	Q9ULD9	1.5656
P03410	Q86YG4	0.8036
P03410	P34949	0.7248
P03410	Q9UFM2	0.3682
P03410	Q9H478	0.68629
P03410	Q14765	0.26221
P03410	Q6ICG8	1.9166
P03410	Q13145	0.42718
P03410	Q5SNT2	0.60438
P03410	F8WFD2	0.70956
P03410	P57075	0.39534
P03410	Q86Y27	1.0723
P03410	Q07020	1.3689
P03410	Q9Y4M8	0.24874
P03410	Q86X55	0.8938
P03410	Q5T0W9	0.31506
P03410	Q460N3	0.31893
P03410	Q13868	0.56789
P03410	O60813	0.64157
P03410	P59646	0.90175
P03410	Q07021	1.2034
P03410	Q9UHD1	0.30238
P03410	Q9NX00	0.57992
P03410	Q8NHZ8	1.6254
P03410	Q8NCU1	0.92031
P03410	O00148	0.63849
P03410	Q99445	0.74303
P03410	Q9NS23	0.45449
P03410	Q96LX8	0.67607
P03410	P49459	0.47809
P03410	Q08378	0.55884
P03410	Q9NR56	0.53556
P03410	P43355	1.1216
P03410	P57721	0.62413
P03410	O95685	0.55009
P03410	P16278	0.19768
P03410	Q9NWZ5	0.25685
P03410	Q9UIH9	0.30419
P03410	Q86XU0	0.83124
P03410	P63220	0.77267
P03410	Q7L3T8	1.1534
P03410	O14639	0.9014
P03410	Q8N812	1.4309
P03410	P62380	0.19641
P03410	Q2TAK8	0.8863
P03410	Q86WR0	0.66275
P03410	O60645	0.82771
P03410	Q5M9Q1	1.3344
P03410	Q6UW49	1.0039
P03410	Q2M389	0.65616
P03410	Q04724	0.75085
P03410	Q8N1A0	0.722
P03410	Q9P2W9	0.62548
P03410	Q53HM4	0.3747
P03410	Q5JVX7	1.0995
P03410	O43776	1.0793
P03410	Q8IZ13	0.39624
P03410	P55769	1.0207
P03410	Q6ZUC2	1.1137
P03410	Q2VY69	0.83577
P03410	Q9H4Z2	0.68989
P03410	Q6N021	0.73424
P03410	Q9H3L0	1.5301
P03410	Q6IQ21	0.80917
P03410	Q9H5L9	0.56022

P03410	Q8IZT9	1.9262
P03410	E9PJI5	1.0659
P03410	Q8N6G1	1.6653
P03410	Q9H8W2	0.70325
P03410	Q6ZTY8	0.25826
P03410	Q9NQG6	0.15505
P03410	Q9P2G4	1.4883
P03410	Q9NZI5	0.39673
P03410	P62910	0.91415
P03410	P43359	1.2552
P03410	O95678	0.76165
P03410	Q92764	0.78893
P03410	Q9HCI6	0.76231
P03410	O95456	0.78804
P03410	A8MXK9	1.1295
P03410	P05787	1.837
P03410	O95379	0.48671
P03410	Q5H9F3	0.54513
P03410	Q9ULR5	0.77415
P03410	Q9UBB5	1.2797
P03410	Q96GC6	0.97903
P03410	P12525	0.31642
P03410	Q9GZY4	0.60631
P03410	Q14689	0.36607
P03410	Q494V2	0.11896
P03410	Q6NSW7	0.30793
P03410	P59998	0.48941
P03410	Q9NS64	1.1679
P03410	B1AMM8	0.72268
P03410	Q9H939	0.2883
P03410	Q16143	0.44511
P03410	Q5W5X9	0.81817
P03410	O75928	0.64196
P03410	A2RU30	1.3104
P03410	Q9P2X8	0.6532
P03410	A6NC78	0.47976
P03410	P82650	0.53602
P03410	Q9HCK1	0.94868
P03410	Q13501	0.45962
P03410	Q9H8T0	0.69111
P03410	Q9BZX4	0.41509
P03410	P25686	0.27881
P03410	P62847	1.0869
P03410	Q59GN2	0.86055
P03410	A2RRL7	0.7694
P03410	Q9NPL8	0.94433
P03410	Q9HA58	1.6653
P03410	Q96S07	1.6653
P03410	P20674	0.14213
P03410	A8K010	0.87666
P03410	O43294	0.14919
P03410	P09912	0.25091
P03410	P22674	0.62052
P03410	P0C5J1	0.34014
P03410	Q96HI0	0.52738
P03410	Q9NPC7	0.69482
P03410	Q9BXN6	0.34399
P03410	Q3SY84	0.76952
P03410	P62906	0.9243
P03410	Q96T37	0.5064
P03410	O95067	0.80732
P03410	Q5T754	1.6653
P03410	P0CJ70	1.4876
P03410	P51826	0.50263
P03410	P0DM63	1.0659
P03410	Q86UQ0	0.57767
P03410	P0CB43	0.56375
P03410	Q8N2U0	0.8809
P03410	A8MWC7	0.01077
P03410	O14908	0.33002
P03410	Q7L2K0	0.47889
P03410	O43830	0.73824
P03410	Q05D60	0.28476
P03410	Q8NEY8	1.452
P03410	Q9BRU2	0.60643
P03410	Q8N7M0	0.62751

P03410	Q9NYH9	0.22649
P03410	Q9HCM7	1.5125
P03410	Q9C0F3	0.71128
P03410	Q9Y6Y0	0.82702
P03410	Q9UKT5	0.36407
P03410	P11142	0.29549
P03410	O95865	0.5487
P03410	Q4G0Z9	0.75539
P03410	Q9HD47	0.51526
P03410	Q96P16	0.23474
P03410	P84090	0.64286
P03410	O75694	0.85413
P03410	Q9C0D2	1.4655
P03410	Q9P0J6	1.6155
P03410	O14662	0.78389
P03410	Q93050	0.20836
P03410	Q7L2H7	0.86673
P03410	P39687	1.3343
P03410	Q9HD36	0.6146
P03410	Q8NAB5	1.6653
P03410	Q2M1K9	0.37497
P03410	Q9NZV7	0.97856
P03410	P78414	0.2823
P03410	P59074	0.19924
P03410	Q8N2B8	0.84055
P03410	O00399	0.30844
P03410	P35321	1.0668
P03410	P57071	0.5128
P03410	Q8IYS2	1.0913
P03410	Q9NRX1	0.29734
P03410	Q8TAA3	0.54062
P03410	Q8N0U6	1.6653
P03410	Q6ZYL4	1.4152
P03410	Q92843	0.74162
P03410	Q96CW6	1.5137
P03410	O00204	0.33927
P03410	Q92783	0.97031
P03410	P45381	0.31063
P03410	P85299	0.077818
P03410	Q6PCB6	0.12864
P03410	P41134	0.36415
P03410	Q9BY43	0.67907
P03410	Q86TJ2	0.48473
P03410	P11172	0.86054
P03410	P31749	0.85409
P03410	Q0VGE8	0.65458
P03410	P17096	0.90016
P03410	Q9H963	0.82125
P03410	Q86UN6	0.56066
P03410	Q8IXQ4	1.4883
P03410	P16452	0.41378
P03410	Q96N95	0.35193
P03410	P58511	1.0913
P03410	P09105	0.83359
P03410	Q99767	0.59748
P03410	O14579	0.79483
P03410	Q9Y5A7	0.77719
P03410	Q6EBC2	0.77249
P03410	Q69YQ0	0.62041
P03410	Q96QK1	1.0582
P03410	Q5T7N7	1.6653
P03410	A6NES4	0.62749
P03410	Q9UBX2	0.27528
P03410	O75420	0.62164
P03410	Q9H7H1	1.0154
P03410	P0C7N4	0.99267
P03410	Q96N23	1.6225
P03410	Q9H8V8	1.6653
P03410	Q99440	0.81767
P03410	Q8N114	1.1058
P03410	P35579	0.59808
P03410	Q6NSZ9	0.52498
P03410	P52815	0.99623
P03410	Q9NZQ3	0.94767
P03410	H3BSY2	0.14694
P03410	Q9Y2K1	0.10142

P03410	Q96DM3	0.64718
P03410	Q53TS8	0.79346
P03410	Q9Y5Z9	0.70324
P03410	Q9NP92	1.6627
P03410	Q5TEV5	1.1405
P03410	Q96N35	1.6653
P03410	Q96GX8	1.4883
P03410	Q5T7P3	1.6653
P03410	P04792	0.43562
P03410	P59047	0.28375
P03410	Q9HAW4	0.62406
P03410	Q7L9L4	0.77376
P03410	Q6L8G5	1.6653
P03410	Q9Y3Q8	1.1649
P03410	Q6ZUG5	0.96856
P03410	A2A288	0.85857
P03410	Q969F1	1.0878
P03410	Q5DJT8	0.37182
P03410	Q9UKI2	0.32962
P03410	P23396	0.93111
P03410	Q9H3M7	0.7808
P03410	O94777	1.044
P03410	Q15124	0.12998
P03410	Q9UK76	1.645
P03410	Q9BUV8	0.19189
P03410	Q7RTS1	0.2539
P03410	O75161	2.12
P03410	Q14232	0.32337
P03410	P10412	0.16413
P03410	Q8TCU4	0.53621
P03410	Q9NYP8	0.50393
P03410	Q8TCZ7	1.1695
P03410	Q15003	1.2813
P03410	Q96LJ8	1.3374
P03410	Q63HN1	0.71274
P03410	Q3MHD2	0.73141
P03410	O75437	0.9221
P03410	Q96DN6	1.4619
P03410	Q96Q80	0.44732
P03410	A6NJV1	1.2147
P03410	Q8NDY3	0.30588
P03410	Q6ZSB9	0.80359
P03410	O00213	0.37465
P03410	Q8TBR5	0.49919
P03410	Q8N3S3	0.41708
P03410	Q92934	0.18005
P03410	Q68DH5	1.3563
P03410	Q5XKR9	1.3654
P03410	P55895	0.94911
P03410	Q99687	0.59411
P03410	Q9NXV6	1.3513
P03410	O43299	1.4565
P03410	Q5T5W8	1.1467
P03410	Q9HBX3	1.6653
P03410	Q8N2W9	0.87434
P03410	Q9UL58	1.0283
P03410	Q96B67	0.58931
P03410	P78406	0.65568
P03410	P06746	0.47328
P03410	Q69YU5	0.95938
P03410	Q6P1M3	1.2113
P03410	O14709	0.79465
P03410	P30419	0.53878
P03410	Q9BUA3	1.2834
P03410	Q969S9	1.0704
P03410	Q9H668	0.35195
P03410	Q9BYR2	1.9984
P03410	Q8N6F1	0.14287
P03410	A6NFD8	0.26279
P03410	P45973	0.94984
P03410	Q9UNZ5	1.4883
P03410	P18621	0.97975
P03410	P48556	0.67204
P03410	Q99633	0.14118
P03410	Q562F6	1.0548
P03410	Q9H972	0.95938

P03410	Q6RVD6	1.6653
P03410	P30876	0.54899
P03410	P63167	1.4146
P03410	Q03060	0.57306
P03410	Q9NWA0	2.0609
P03410	Q12798	1.0001
P03410	Q86WQ0	0.7568
P03410	P26599	0.27659
P03410	Q96K75	0.75596
P03410	Q96SN7	0.45689
P03410	Q9Y2B5	0.58806
P03410	Q9P209	1.2054
P03410	Q9UFV3	0.28967
P03410	Q9Y2V2	0.7026
P03410	O76015	0.88723
P03410	Q9NP77	0.32982
P03410	Q5T8R8	0.11242
P03410	P20132	0.16349
P03410	Q8N0V1	1.223
P03410	P0CJ85	0.27528
P03410	Q6PKG0	0.6999
P03410	P42768	0.62193
P03410	Q92785	0.65206
P03410	Q86V97	0.54253
P03410	Q8TB68	1.0297
P03410	Q96CG3	0.70742
P03410	A8MYZ0	1.0367
P03410	Q7L5Y6	0.20917
P03410	Q6ZUT6	0.90371
P03410	Q9H8Y5	0.26328
P03410	P49721	0.74834
P03410	Q8N720	0.77051
P03410	Q2PZI1	0.88883
P03410	Q96C57	0.98341
P03410	O95167	0.67883
P03410	Q9Y287	0.80036
P03410	Q96HE9	0.26411
P03410	Q92674	0.47859
P03410	Q8NDH6	0.61533
P03410	P62266	1.1668
P03410	Q15653	1.2056
P03410	Q9NS69	0.36968
P03410	Q8N7B6	0.79466
P03410	Q96A00	1.0879
P03410	O95260	0.1147
P03410	O60268	0.5477
P03410	O95190	1.2715
P03410	P54252	0.41435
P03410	Q8N1E6	0.4896
P03410	Q9HD87	0.71182
P03410	Q9Y3X0	1.168
P03410	Q7L3B6	0.51425
P03410	Q9UEE9	1.0757
P03410	Q9NWU5	1.4739
P03410	Q6NVY1	0.1345
P03410	O94929	0.29282
P03410	Q5T292	1.0946
P03410	Q9H6S1	0.18234
P03410	P40855	0.79635
P03410	P56385	0.86334
P03410	O60832	0.79468
P03410	Q96S19	0.46681
P03410	Q9NUP9	0.62406
P03410	P52741	0.74365
P03410	A8MWP4	0.70723
P03410	P50570	0.39378
P03410	Q659A1	0.90967
P03410	Q92889	0.36279
P03410	P19532	0.5967
P03410	Q9BPZ2	1.0729
P03410	Q8NBA8	0.72692
P03410	Q9HC38	0.41276
P03410	P07900	0.46176
P03410	Q00587	0.56046
P03410	Q8N9N2	0.49931
P03410	Q5VTR2	0.36235

P03410	P02766	0.04114
P03410	Q8N0U2	1.6653
P03410	Q8N443	0.80291
P03410	Q6DCA0	0.35117
P03410	P31358	1.3712
P03410	Q9H063	0.86351
P03410	Q9BQA1	1.0464
P03410	Q93075	1.0913
P03410	Q08999	1.2601
P03410	P10243	0.64615
P03410	Q5THR3	0.46163
P03410	A8MU93	1.4883
P03410	Q9Y468	0.8472
P03410	A6NJG6	0.30793
P03410	A6NNA5	0.15036
P03410	Q9H211	0.91693
P03410	Q96EK6	0.83855
P03410	Q53FT3	0.77549
P03410	Q96S55	0.61744
P03410	Q6ZV60	1.6653
P03410	Q59F38	0.22612
P03410	Q7Z7A3	0.82652
P03410	Q6ZSR3	1.6653
P03410	Q9NPP4	0.28816
P03410	P16333	0.71338
P03410	A6NDV4	0.2885
P03410	Q5D862	0.095248
P03410	Q9BRT6	0.776
P03410	Q1HG44	1.3169
P03410	Q9BTL4	1.1718
P03410	Q9UJW0	0.72838
P03410	Q8TAD1	0.59063
P03410	Q7Z794	0.93858
P03410	P20226	0.83912
P03410	P82979	0.45162
P03410	O75953	0.87141
P03410	Q8TDN6	0.42808
P03410	Q96MU7	0.9812
P03410	Q0VDD7	1.9711
P03410	Q9P013	0.92202
P03410	Q96N16	1.4898
P03410	Q9H6L5	0.94236
P03410	O43583	1.73
P03410	Q15366	0.9666
P03410	Q9BWG6	0.89228
P03410	Q96J86	1.0913
P03410	P0DKV0	0.25623
P03410	Q96AN5	0.81715
P03410	Q9GZR1	0.36562
P03410	Q5SZD4	1.1275
P03410	Q8N137	1.2123
P03410	Q15370	1.0596
P03410	Q92844	0.63019
P03410	P62249	1.269
P03410	Q86YP4	0.71129
P03410	O60239	0.49437
P03410	Q86TP1	1.0277
P03410	Q7Z4M0	0.50186
P03410	Q6IPR1	0.71035
P03410	Q6ZQP7	1.6653
P03410	Q8NI35	0.5915
P03410	O15225	1.6653
P03410	P13640	0.97224
P03410	Q01105	0.75991
P03410	Q9UK59	0.13416
P03410	O95948	0.30796
P03410	Q9HAT2	0.97111
P03410	Q86VS8	0.69552
P03410	A5PLN7	1.4883
P03410	Q6P1M9	0.6784
P03410	Q86VE0	1.7014
P03410	Q5T751	1.6653
P03410	P10828	0.46983
P03410	Q8IXS0	1.4883
P03410	Q9C000	0.33224
P03410	P48668	0.79891

P03410	O60732	0.6999
P03410	Q6NX45	0.7597
P03410	P27544	0.73217
P03410	Q9UHA2	0.52786
P03410	A6NCC3	0.62012
P03410	Q6PEX7	0.88877
P03410	Q9Y5Q8	0.84563
P03410	Q5TA31	0.22235
P03410	O00321	0.35954
P03410	O60524	1.5936
P03410	A6H8Y1	0.8595
P03410	Q92791	0.28431
P03410	Q3B7T1	1.1948
P03410	Q15651	1.3768
P03410	Q6ZS62	1.218
P03410	Q9NSC5	0.74876
P03410	P0C7X3	0.80944
P03410	Q9NRG0	0.26854
P03410	Q5TA79	1.6653
P03410	Q9H175	1.0653
P03410	Q15004	1.0744
P03410	Q15906	0.97447
P03410	Q5TKA1	0.59312
P03410	Q9Y2B1	1.0913
P03410	Q9Y6A5	0.16267
P03410	Q96M63	0.40375
P03410	Q9UJL9	0.75781
P03410	P63151	0.57015
P03410	A4QMS7	0.37877
P03410	P21953	0.24962
P03410	P0C7M3	1.1653
P03410	Q8TC20	0.1081
P03410	Q9UF83	0.65157
P03410	Q9UBU6	0.97881
P03410	Q9UIF3	0.57152
P03410	Q13794	0.89949
P03410	Q9NSJ1	0.86213
P03410	Q9Y283	0.4101
P03410	Q6PF04	0.79263
P03410	P62308	0.81849
P03410	Q9BT49	1.0572
P03410	P50749	0.89663
P03410	Q9Y6J3	1.6653
P03410	P14678	0.86186
P03410	Q15814	0.47722
P03410	Q8IYB1	0.53966
P03410	Q96Q83	0.63632
P03410	P63173	1.2562
P03410	P61581	1.7423
P03410	Q5T6F2	1.6144
P03410	Q8N814	1.4065
P03410	A6ND36	0.12419
P03410	O00291	0.63192
P03410	A8MQT2	0.066261
P03410	Q3SXP7	0.6452
P03410	Q9GZP9	0.44732
P03410	Q96FJ8	1.6653
P03410	P62316	0.86567
P03410	Q9UNF1	0.0070625
P03410	Q9Y3T9	1.1771
P03410	P50914	1.2309
P03410	A5PLN9	0.9903
P03410	Q96F63	1.4883
P03410	O75764	0.23577
P03410	Q969Q0	0.49891
P03410	Q12980	1.3993
P03410	P31949	0.48414
P03410	O15037	0.80101
P03410	Q96DV4	0.57901
P03410	O94892	0.73
P03410	Q32MZ4	1.1477
P03410	Q9HAU5	0.85696
P03410	Q9UBB9	0.22401
P03410	Q13835	0.37509
P03410	B1AH88	1.6653
P03410	P39748	0.27094

P03410	Q8N336	0.57167
P03410	A8MXV6	0.65961
P03410	Q9NYZ3	1.9316
P03410	Q8WUH2	0.97907
P03410	P06748	0.59486
P03410	Q9Y3F4	0.99905
P03410	P47897	0.70671
P03410	Q8TCX1	0.024806
P03410	A6NKX1	1.6653
P03410	Q9HCE0	0.44777
P03410	O75368	0.56593
P03410	P60022	0.80017
P03410	Q9UBK9	1.1536
P03410	Q24JP5	0.72863
P03410	Q969I3	0.86773
P03410	O75381	0.89355
P03410	P48378	0.41575
P03410	Q6PIJ6	1.5729
P03410	P06400	0.87382
P03410	Q6PF06	1.2136
P03410	Q5VWX1	0.74868
P03410	Q9BXY8	1.0951
P03410	Q9ULJ6	0.45786
P03410	Q16633	0.65543
P03410	Q5ZEY9	0.92021
P03410	Q6DKK2	1.3838
P03410	Q8TBC3	0.32577
P03410	Q86VN1	0.60018
P03410	Q14137	1.0821
P03410	A8MV72	0.93328
P03410	Q99865	1.0829
P03410	Q96IZ7	1.8934
P03410	Q8TF01	1.6158
P03410	Q6UUV9	1.5402
P03410	P62280	1.2837
P03410	Q9Y366	0.38626
P03410	O60809	0.74583
P03410	Q6NVH7	1.439
P03410	Q9UBS3	0.69303
P03410	O15040	0.6999
P03410	Q9BSI4	1.1436
P03410	Q9NQ40	1.0772
P03410	Q6ZQT7	1.6653
P03410	Q969X0	0.066434
P03410	P06132	1.1055
P03410	Q9Y3M9	0.62099
P03410	Q6P4F2	0.092151
P03410	Q5F1R6	0.21841
P03410	O14530	0.49477
P03410	Q5JR98	0.99799
P03410	A6NM28	0.70851
P03410	Q9BTD3	1.0913
P03410	Q9UJY1	0.55598
P03410	Q9H116	0.82427
P03410	Q03923	1.0223
P03410	Q9Y5Y2	0.33483
P03410	Q0VG73	1.6653
P03410	O15049	1.1911
P03410	Q96CT7	0.2096
P03410	Q6PK81	0.81024
P03410	Q96EH5	1.5427
P03410	Q9NU23	0.79402
P03410	P19484	0.4625
P03410	Q9H9T3	0.56685
P03410	Q96M95	0.18819
P03410	Q9UIY3	0.36413
P03410	Q9H3H1	1.2091
P03410	P56282	0.52958
P03410	A6NMP4	0.54655
P03410	A6NH11	1.1087
P03410	Q15018	0.45903
P03410	Q9H1X1	0.74063
P03410	P55157	0.38089
P03410	A6NJ69	1.0723
P03410	Q8IVT2	1.4357
P03410	A6NGB0	1.0913

P03410	Q9NYF3	0.83743
P03410	Q9HC44	1.9744
P03410	Q9Y697	0.63491
P03410	Q9BQC6	1.9496
P03410	Q53H82	0.21735
P03410	O60260	0.29031
P03410	Q9NQ32	1.0129
P03410	Q5VYM1	1.0913
P03410	A6NJ08	0.37086
P03410	Q9ULM3	0.99888
P03410	A8MZG2	1.4883
P03410	Q96H12	1.2806
P03410	O75376	0.38731
P03410	Q8IWV7	0.5606
P03410	Q6P2I3	0.596
P03410	Q9P243	0.30908
P03410	Q9BWK5	1.6468
P03410	Q9P055	0.86518
P03410	Q9UNH5	0.73719
P03410	F8W1W9	0.4749
P03410	Q96LY2	1.4946
P03410	Q86UQ8	0.67552
P03410	Q9ULL5	1.6653
P03410	P31314	0.58198
P03410	Q9BQB4	0.36228
P03410	Q0P140	0.73414
P03410	P57738	1.0992
P03410	Q96CD0	0.85383
P03410	Q9HCL3	1.0202
P03410	Q9NV44	0.86476
P03410	P0DKL9	1.5155
P03410	Q6UWF5	1.6653
P03410	Q9NYK5	0.77631
P03410	O15239	0.71696
P03410	P49716	0.39957
P03410	Q6L9T8	1.0087
P03410	Q15025	0.71641
P03410	O00110	0.20557
P03410	Q68DY1	1.0122
P03410	Q04323	0.43313
P03410	Q9UPX6	1.0913
P03410	Q9P2A4	0.39754
P03410	A8MTB1	1.6653
P03410	Q9Y6U3	0.076836
P03410	Q8TAX7	1.6653
P03410	Q53FN2	0.9535
P03410	P0C7I6	1.287
P03410	P05060	0.7014
P03410	Q9BUV0	1.4883
P03410	Q9H0A8	1.2337
P03410	Q06547	1.0818
P03410	Q9NSU2	0.17716
P03410	Q9HB21	0.30851
P03410	P68036	0.4436
P03410	O14490	0.51846
P03410	Q8NEJ9	0.70932
P03410	Q96ES7	0.33895
P03410	Q70YC4	1.0766
P03410	Q9P253	0.45101
P03410	Q8N0W5	0.96474
P03410	Q9H7X2	1.0913
P03410	Q9UBF1	1.0247
P03410	Q8TAV0	1.6158
P03410	Q32NC0	0.72217
P03410	Q86VI4	0.49943
P03410	Q8WZ71	1.1282
P03410	P14868	1.2148
P03410	Q6UXQ8	1.6653
P03410	Q9H7X3	0.7207
P03410	Q5JT18	1.6653
P03410	A2A368	0.14589
P03410	Q9BYN8	1.8888
P03410	Q7Z713	0.34399
P03410	Q6DKI1	0.32994
P03410	Q9BTP7	1.2671
P03410	Q8IVG9	1.2391

P03410	Q9H0S4	0.16299
P03410	Q6P988	0.73584
P03410	Q6GMV2	0.54619
P03410	A2RU49	0.38121
P03410	Q9BRA2	0.67374
P03410	Q5SVJ3	0.46096
P03410	Q9GZL7	0.92032
P03410	Q9BVC4	0.64339
P03410	Q9H987	0.22746
P03410	Q6ZR85	1.6653
P03410	P61088	0.87157
P03410	Q9H808	0.71114
P03410	Q6YFQ2	0.15652
P03410	Q86VY9	0.6506
P03410	Q15059	1.1181
P03410	Q96N21	1.2545
P03410	O43432	0.037359
P03410	Q13233	0.8169
P03410	Q8WVH0	0.40446
P03410	Q96R06	1.1024
P03410	Q14160	0.9251
P03410	Q14442	0.25994
P03410	Q96KQ4	1.109
P03410	Q3KP66	1.9117
P03410	Q96SD1	0.25421
P03410	Q9P1G2	1.2464
P03410	Q5JST6	1.0891
P03410	O14972	0.6124
P03410	Q99684	0.46927
P03410	P01215	0.70445
P03410	Q9H3Q1	0.38207
P03410	P43351	0.88252
P03410	Q9H6Q4	0.61944
P03410	O60664	0.99544
P03410	Q9H9G7	0.99596
P03410	Q6L8G8	1.6653
P03410	Q15327	0.48698
P03410	Q8NDZ6	0.7168
P03410	Q7Z3B0	1.0913
P03410	Q49AJ0	0.42506
P03410	O95997	0.83887
P03410	P32519	1.1117
P03410	Q9H2U2	0.43253
P03410	Q9BYH8	0.47263
P03410	Q8N7U7	0.66689
P03410	Q96HA1	1.4495
P03410	A6NEN9	0.15313
P03410	Q9UIE0	0.80131
P03410	Q6A162	0.78893
P03410	P23193	1.0872
P03410	Q96JE9	0.47689
P03410	Q9NXP7	0.52722
P03410	P30793	0.21418
P03410	A3QJZ6	0.1568
P03410	P19623	0.32786
P03410	Q53GI3	0.71499
P03410	Q96PS1	0.89048
P03410	Q1AE95	0.79088
P03410	Q9UKI3	0.44305
P03410	Q96NU7	0.75529
P03410	Q03468	0.36037
P03410	Q5VWM6	1.3613
P03410	Q8N7Q2	1.6653
P03410	P30042	0.40104
P03410	Q99829	0.38296
P03410	Q8NB42	1.0264
P03410	Q9Y4P8	1.0367
P03410	Q6XQN6	0.32468
P03410	P55265	0.38821
P03410	Q8WWC4	1.1379
P03410	Q86X76	0.18627
P03410	O60304	0.69672
P03410	Q9BQ83	0.81602
P03410	A6NEH8	0.89394
P03410	Q9BT78	1.1584
P03410	A5LHX3	0.53245

P03410	Q6U7Q0	0.74466
P03410	P15923	0.5101
P03410	Q9HBV2	1.0913
P03410	A5YM69	0.87102
P03410	P22090	0.55816
P03410	Q8N1V8	0.76081
P03410	P26651	0.84783
P03410	Q8IV31	0.777
P03410	O00411	0.82595
P03410	Q6UB98	0.49184
P03410	Q9Y2V0	0.35545
P03410	P05062	0.22209
P03410	Q09FC8	0.80544
P03410	Q06732	0.59461
P03410	Q9Y3E0	0.50315
P03410	Q9UMY4	0.44123
P03410	Q6ZN19	0.66065
P03410	Q8N808	1.1631
P03410	Q9BVV6	1.6653
P03410	P13639	0.32577
P03410	Q6NUQ1	0.88935
P03410	P0CW26	0.55606
P03410	Q6ZU15	0.33506
P03410	Q99607	1.0411
P03410	Q4VC44	0.97898
P03410	Q14DG7	0.87365
P03410	P17025	0.80606
P03410	Q03052	0.30956
P03410	P52788	0.25489
P03410	Q8N2H4	1.8136
P03410	Q3LI72	1.6653
P03410	Q9Y617	0.79003
P03410	Q13045	1.1384
P03410	Q8WWM7	0.8855
P03410	Q6ZMV5	0.86077
P03410	P0CJ90	0.27528
P03410	Q17RG1	0.32577
P03410	Q9BYD6	1.5139
P03410	P02538	1.1258
P03410	Q52M93	0.63446
P03410	O94850	0.87296
P03410	A6NNL0	1.3673
P03410	Q6ZVX7	0.92118
P03410	Q9Y530	0.70929
P03410	Q8NHR7	0.40073
P03410	P61313	1.2786
P03410	Q9NP85	0.32251
P03410	B1AK76	1.4544
P03410	Q69383	1.1462
P03410	Q6NZY7	0.38207
P03410	Q8WYQ5	0.75145
P03410	Q9NXX6	0.55117
P03410	Q9UIV1	1.0641
P03410	Q5JTJ3	0.4075
P03410	Q9Y3C7	0.99391
P03410	A6NMN3	0.78976
P03410	Q13113	0.68286
P03410	O00193	1.757
P03410	Q99615	0.97471
P03410	Q9BXS6	1.4071
P03410	Q5MJ68	0.58696
P03410	Q9NSB4	0.69023
P03410	Q8TD16	0.58607
P03410	P62979	0.72365
P03410	Q5XUX0	0.94144
P03410	Q1ED39	1.757
P03410	Q96EB1	1.1033
P03410	Q8TBK2	0.70168
P03410	Q9BY14	0.42963
P03410	P54725	0.86083
P03410	Q9UPW6	0.65473
P03410	A6NDP7	0.69362
P03410	Q9Y6M9	0.042124
P03410	Q6ZSV7	0.60541
P03410	P28715	0.31251
P03410	Q99613	0.90431

P03410	Q8TAT5	0.55004
P03410	Q8NG57	0.63451
P03410	Q96MZ4	0.061799
P03410	P86791	0.60221
P03410	Q9NPG3	1.0527
P03410	C9JXX5	0.95938
P03410	Q8TB33	0.74904
P03410	Q9H4A6	0.8709
P03410	Q9UKF7	0.076697
P03410	Q9NV92	0.57573
P03410	P38117	0.54149
P03410	Q9NQW6	0.51301
P03410	Q9H322	1.5696
P03410	Q9NWS9	0.24502
P03410	Q6NS38	0.68305
P03410	Q9BSV6	0.6079
P03410	P43364	1.0663
P03410	Q12840	0.46876
P03410	Q8TAP9	1.9329
P03410	O43159	0.76327
P03410	P47813	1.5459
P03410	Q9NVV4	0.43132
P03410	Q8NEF3	0.5561
P03410	Q8TAD8	1.3792
P03410	Q86VX2	1.3459
P03410	Q96LT6	0.69563
P03410	P86479	1.6653
P03410	Q9NVM1	1.3818
P03410	P23786	0.04383
P03410	Q53FE4	0.40822
P03410	P78549	0.34458
P03410	Q9H7J1	0.87827
P03410	Q969H4	0.44455
P03410	O95817	0.59973
P03410	O60397	0.46884
P03410	Q8TCH9	1.0163
P03410	P35227	0.55266
P03410	P62508	0.41298
P03410	P0CF97	0.8849
P03410	Q96MH7	0.20888
P03410	Q6P597	0.41192
P03410	P0CJ71	1.4876
P03410	Q4LE39	0.74912
P03410	Q8NDZ9	1.6653
P03410	P23327	1.5556
P03410	Q96KN1	0.68391
P03410	Q96S52	0.35154
P03410	C9JFL3	1.6653
P03410	Q92748	1.674
P03410	Q8IYX8	0.32471
P03410	Q8NEM1	1.0122
P03410	Q9NVE4	0.54247
P03410	Q9Y3D5	1.5971
P03410	Q8TDP1	1.159
P03410	Q8TF30	0.16757
P03410	P09467	0.2582
P03410	Q9H9J2	0.42547
P03410	P02549	0.3258
P03410	Q8TEX9	0.68714
P03410	Q9UBX3	0.31887
P03410	Q6P0N0	1.1067
P03410	Q8N6C7	0.15287
P03410	O75146	0.37209
P03410	Q14596	0.22939
P03410	Q6ZUV0	0.18836
P03410	Q5VUD6	0.8763
P03410	Q9Y5R8	0.32637
P03410	P0C1Z6	1.4435
P03410	Q86WP2	0.86628
P03410	Q30KQ6	0.87928
P03410	P52657	1.2495
P03410	E5RQL4	0.67509
P03410	Q8NCD3	0.85902
P03410	Q92466	0.66082
P03410	A6NP81	0.26797
P03410	Q06190	0.35582

P03410	C9JQI7	0.71116
P03410	Q9BYV8	1.0447
P03410	Q9NVN3	0.3101
P03410	Q8WTU0	0.58896
P03410	P56747	0.44906
P03410	P51523	0.61902
P03410	Q8IWS0	0.49071
P03410	Q14188	0.67578
P03410	A6NGC4	1.0913
P03410	Q96ST3	0.66265
P03410	F5H4B4	1.5219
P03410	Q8TDM0	0.13077
P03410	Q9Y4X0	0.55972
P03410	O00461	1.0194
P03410	Q8IYS4	0.012308
P03410	Q05084	0.41189
P03410	Q8NFD5	0.86497
P03410	Q3LI61	1.6653
P03410	Q9H171	0.46722
P03410	Q8NC60	0.080306
P03410	Q16617	1.0029
P03410	Q9NR22	0.48022
P03410	Q9H374	0.54655
P03410	O43761	0.94894
P03410	Q6ZN55	0.71696
P03410	O15514	0.32685
P03410	Q9UJW9	2.3231
P03410	A6NFK2	0.38823
P03410	Q9BRT9	0.65102
P03410	P02812	0.93955
P03410	Q6ZT77	0.67857
P03410	Q5TH74	0.99777
P03410	Q7Z2F6	0.79625
P03410	Q8IYE1	0.11759
P03410	P0DM35	1.4683
P03410	Q6DHV5	0.64295
P03410	Q13541	0.92434
P03410	P07195	0.69158
P03410	Q9Y295	0.59345
P03410	O60216	0.95374
P03410	Q7Z7F0	0.034663
P03410	O14617	0.36833
P03410	Q15306	0.56407
P03410	Q13568	0.78071
P03410	Q6U949	1.6653
P03410	Q8IYL2	0.99258
P03410	P50613	0.17188
P03410	P33241	0.69996
P03410	Q96HQ0	0.79946
P03410	O75177	1.0407
P03410	Q9H1J1	1.4762
P03410	O94817	0.83859
P03410	Q68CZ6	0.81017
P03410	P14866	0.57706
P03410	Q5HYW3	1.4025
P03410	A0MZ66	0.9728
P03410	O15055	0.23774
P03410	Q9UHA4	0.43374
P03410	Q9BQE4	0.91617
P03410	A8MU46	0.23355
P03410	Q9P2N5	0.36961
P03410	Q9H204	1.3512
P03410	Q03933	0.50252
P03410	Q96BW9	0.85224
P03410	Q5H9R7	1.5434
P03410	Q14152	1.2306
P03410	K7EIQ3	1.54
P03410	Q96EK4	1.673
P03410	Q14674	0.32258
P03410	Q5VZB9	0.19656
P03410	Q9H9A5	0.20797
P03410	P61163	0.21475
P03410	Q5D0E6	1.2452
P03410	Q5H9K5	0.25933
P03410	Q6ZRH3	0.76596
P03410	Q9UJY5	0.95911

P03410	Q14258	0.6584
P03410	Q9BYD3	1.8908
P03410	Q96SC8	0.18733
P03410	Q96T49	0.45542
P03410	Q5T742	1.6653
P03410	Q9Y587	0.42276
P03410	Q5TAP6	0.96881
P03410	Q96EH3	0.51112
P03410	Q9NRR4	0.53101
P03410	P41214	0.9481
P03410	O60688	0.36773
P03410	Q99436	1.0314
P03410	Q86UQ5	1.6653
P03410	Q5JT78	0.23883
P03410	Q9Y6B7	0.66244
P03410	Q92832	0.25793
P03410	P28838	1.1121
P03410	Q8NAV1	1.4814
P03410	O00712	0.30034
P03410	P56278	0.37474
P03410	O15318	1.4071
P03410	Q6UXQ6	1.6653
P03410	O95521	0.70932
P03410	Q7LBC6	0.20901
P03410	Q3LI68	1.7978
P03410	P09012	0.45881
P03410	Q13895	0.92375
P03410	P59091	1.0268
P03410	Q7Z3Z2	0.4954
P03410	P62701	0.60347
P03410	Q9NS71	0.88356
P03410	Q92585	1.0125
P03410	Q9BQC3	1.5523
P03410	Q9Y6X4	1.7604
P03410	A5D8V7	0.085551
P03410	Q9H7M6	0.5569
P03410	Q13065	0.26031
P03410	Q96I85	1.6653
P03410	Q01831	0.85182
P03410	A8MWS5	0.12996
P03410	Q15819	0.57656
P03410	Q9UKW6	0.35387
P03410	Q96T58	0.63216
P03410	Q8WW38	0.46682
P03410	P36551	0.5235
P03410	Q9UBV8	0.46375
P03410	Q3MJ40	0.20311
P03410	Q9Y535	0.41003
P03410	A8MXZ3	1.6653
P03410	Q92541	0.98043
P03410	Q16585	0.9042
P03410	O95760	0.37402
P03410	Q01201	0.92611
P03410	Q8TAX9	0.34857
P03410	P25490	0.2003
P03410	Q537H7	0.63816
P03410	P01100	0.5948
P03410	Q96CS2	0.82264
P03410	P18846	0.97953
P03410	Q0D2K3	1.231
P03410	Q96LW1	0.99815
P03410	Q53HV7	0.90061
P03410	O94925	0.56382
P03410	Q8N609	0.67545
P03410	Q02410	0.71589
P03410	P52746	0.51659
P03410	Q5QJE6	1.6338
P03410	Q00577	0.26204
P03410	Q8WVV9	0.29764
P03410	Q9H1K1	0.94858
P03410	Q86VP1	0.82179
P03410	P49770	0.34608
P03410	Q96MP5	0.54647
P03410	Q9BV87	0.81108
P03410	Q5TA81	1.6653
P03410	Q9UFE4	0.38122

P03410	Q86WC6	0.55998
P03410	Q8N9W5	0.29249
P03410	P0C867	1.2861
P03410	P61244	0.7983
P03410	P0C879	1.6653
P03410	Q6NVV7	0.43519
P03410	P41236	1.2137
P03410	Q7RTU4	0.44898
P03410	Q0ZLH3	0.64335
P03410	P41002	1.0932
P03410	Q56P42	0.34399
P03410	Q9BV20	0.71477
P03410	Q9H0C8	0.54945
P03410	O14933	0.42429
P03410	A8MTZ0	1.4607
P03410	P07320	1.4001
P03410	Q9BZI7	1.086
P03410	Q15424	0.78813
P03410	Q9H910	1.645
P03410	Q8NFW9	0.40637
P03410	Q9H009	0.54734
P03410	A6NNC1	1.2342
P03410	Q8IXM7	0.62596
P03410	Q6P2D8	0.29129
P03410	O15226	0.88702
P03410	O15258	0.72221
P03410	Q07666	0.45187
P03410	A6NK58	0.52398
P03410	Q5T1J6	0.3943
P03410	Q8WXG1	0.18515
P03410	Q15560	0.94504
P03410	Q7Z4V5	0.15765
P03410	Q9Y6F1	0.40319
P03410	Q7Z434	0.37026
P03410	Q9H7B2	1.2313
P03410	Q9HCC9	0.62556
P03410	Q9H8X3	0.16142
P03410	Q5EE01	1.066
P03410	Q9NRY6	0.4419
P03410	Q6IV72	0.67818
P03410	Q8N912	1.3285
P03410	P17036	0.70939
P03410	Q6IPM2	0.1757
P03410	Q9NPC6	1.6965
P03410	Q6UXQ4	1.6653
P03410	Q96FV9	0.80949
P03410	Q5T0J7	0.94526
P03410	Q8N490	0.6871
P03410	P07196	0.90636
P03410	Q9BTV5	0.72316
P03410	O00757	0.17306
P03410	Q9H1A3	0.6999
P03410	P53990	0.85462
P03410	Q5QP74	1.6653
P03410	Q8TC21	0.75121
P03410	Q9BZL3	1.1007
P03410	Q13884	1.0234
P03410	Q8IXQ9	0.55452
P03410	P60014	1.7937
P03410	Q8N3R3	0.49283
P03410	Q9NXE4	0.4885
P03410	Q12904	0.17442
P03410	Q6P387	0.9976
P03410	Q8N6M3	1.0157
P03410	Q96HQ2	1.7028
P03410	O15273	0.14201
P03410	O60826	1.2657
P03410	Q9NX53	0.6999
P03410	P38432	1.5493
P03410	A6NFQ7	0.30793
P03410	Q8N1K5	0.84709
P03410	Q96NS8	0.45287
P03410	P15408	0.53011
P03410	Q9H1I8	0.78432
P03410	Q86T23	0.17249
P03410	Q53ET0	2.0967

P03410	Q86SG4	0.14238
P03410	P06703	0.2441
P03410	Q96LV5	0.31671
P03410	Q9UL45	0.26527
P03410	Q8N2C9	0.6577
P03410	O60303	0.52604
P03410	P0CL80	0.508
P03410	P55957	0.82178
P03410	Q9HBL8	0.13602
P03410	H3BQB6	1.5946
P03410	Q5VYP0	1.0269
P03410	A8K0S8	0.2823
P03410	Q9NTW7	0.98837
P03410	Q8WXE9	0.79736
P03410	Q9BVV8	0.88922
P03410	Q2M385	0.80955
P03410	O60663	0.41362
P03410	Q96G23	0.21761
P03410	O75069	0.33166
P03410	P51508	0.82417
P03410	Q3MIS6	0.98524
P03410	Q9H707	0.81641
P03410	Q9H9R9	0.22861
P03410	P23508	0.53339
P03410	Q8WU76	0.1621
P03410	A8MUI8	0.73891
P03410	P33992	0.35737
P03410	P0C7V6	1.2614
P03410	F8WBI6	0.14694
P03410	Q6PII5	0.65159
P03410	Q7Z401	0.39495
P03410	Q9H7C4	0.68581
P03410	Q8ND83	1.8112
P03410	Q9BRT2	0.54535
P03410	A6NHX0	1.3563
P03410	Q5VU69	0.96422
P03410	Q96SR6	0.82417
P03410	Q5TEC3	0.78521
P03410	Q9Y2X3	0.77343
P03410	Q8IVU9	0.27185
P03410	Q9UMZ2	0.27806
P03410	Q9NP66	0.84708
P03410	Q9BYR4	1.9984
P03410	Q8N9R0	1.6653
P03410	Q13158	0.64287
P03410	Q6UWF9	0.79568
P03410	Q96QD9	1.0676
P03410	Q9UQ35	1.0784
P03410	B4DXR9	0.99633
P03410	O43264	0.82453
P03410	Q9NUG6	0.14771
P03410	Q6ZP98	1.1863
P03410	Q8NEP9	0.92179
P03410	Q9UJJ7	0.63983
P03410	Q14693	0.27668
P03410	P23919	0.87227
P03410	Q6UWD8	0.65315
P03410	Q6VMQ6	1.5572
P03410	M0R1G4	0.062582
P03410	O15231	0.15515
P03410	Q9BZV1	1.1401
P03410	Q8TCG1	0.096673
P03410	Q8N1F7	0.8551
P03410	Q8WUW1	0.50232
P03410	Q156A1	1.6653
P03410	P62750	0.98685
P03410	O75569	0.35504
P03410	Q8N112	0.96496
P03410	P42765	0.20318
P03410	P81408	1.0975
P03410	P0CG29	0.30366
P03410	P13073	0.78062
P03410	O75486	1.019
P03410	Q96A22	0.68816
P03410	O95171	0.31636
P03410	Q96GT9	1.2681

P03410	Q92575	1.1213
P03410	Q9BQ66	1.9984
P03410	A6NIH7	0.79853
P03410	Q8WUI4	0.40267
P03410	Q5JUW0	0.87221
P03410	O00559	0.48712
P03410	P17544	0.9359
P03410	Q6PP77	1.2132
P03410	Q9NZ42	0.73376
P03410	Q96MF7	0.56334
P03410	P0C7T8	1.0913
P03410	Q5SVQ8	0.88499
P03410	Q16670	0.6841
P03410	P35659	0.6684
P03410	Q9NSD9	1.1997
P03410	Q9H074	0.52909
P03410	Q2M329	0.60792
P03410	Q9C005	0.62928
P03410	O95249	0.014043
P03410	Q8IZT6	0.47914
P03410	Q8NAP1	1.4883
P03410	P46926	0.087604
P03410	P50452	0.74091
P03410	Q6ZV50	0.41575
P03410	Q96EY7	0.1972
P03410	Q969W3	1.7716
P03410	Q16576	0.33111
P03410	O60381	0.24596
P03410	Q6ZRN7	1.6653
P03410	O60930	0.73641
P03410	Q96BJ3	0.957
P03410	O60783	1.3376
P03410	Q9BY32	0.84651
P03410	P50549	0.77266
P03410	Q8WXI4	0.098298
P03410	Q7Z7K0	0.46284
P03410	H0YKK7	1.5003
P03410	Q5T089	0.0039504
P03410	O14841	0.81206
P03410	Q96JB5	0.59297
P03410	Q9GZS3	1.0663
P03410	Q9BWF2	0.36107
P03410	Q86UW6	0.35971
P03410	Q6ZR93	1.6653
P03410	Q9Y5P3	0.56835
P03410	Q5EBL2	0.65248
P03410	Q7Z7K6	1.2052
P03410	P25815	0.70001
P03410	P82664	1.7659
P03410	Q9HAP6	0.38174
P03410	P49454	0.62279
P03410	Q15700	0.40919
P03410	Q9H8W3	0.80585
P03410	Q2M2I3	0.062703
P03410	Q8N6S5	1.1813
P03410	Q8NEW7	0.93824
P03410	Q9NQY0	0.52525
P03410	Q9NXJ5	0.7708
P03410	A6NN79	0.85968
P03410	Q96M42	0.58484
P03410	Q9H8M2	0.19609
P03410	Q9P1Z9	0.86734
P03410	O75503	0.57206
P03410	Q2TAZ0	0.6999
P03410	P22234	0.49121
P03410	P17010	0.36309
P03410	Q969K4	0.28073
P03410	Q8N1G0	0.2267
P03410	Q8N6Y0	1.0134
P03410	Q5HYL7	1.1653
P03410	Q96I34	0.46939
P03410	Q9Y5K6	0.4484
P03410	Q96E11	1.715
P03410	Q9GZV5	0.99275
P03410	Q9UH36	0.80829
P03410	Q969U7	0.90149

P03410	Q8N7L0	0.46295
P03410	Q8N8K9	0.3976
P03410	Q9H0E3	1.5338
P03410	Q8N442	0.60782
P03410	O60759	0.66849
P03410	Q5JXB2	0.3533
P03410	Q9UQR1	0.75339
P03410	P11473	0.36299
P03410	P0CZ25	0.62862
P03410	Q9NRP4	0.16416
P03410	Q9BQJ4	1.3329
P03410	Q659C4	0.94114
P03410	Q9BSE4	0.75272
P03410	Q6NW29	0.62614
P03410	Q9UQL6	0.44046
P03410	Q02556	0.33564
P03410	Q96MG7	0.83653
P03410	P51825	1.069
P03410	O95295	0.56823
P03410	Q96D70	1.6471
P03410	O95347	0.4395
P03410	Q6ZWB6	0.25004
P03410	Q8NHQ8	1.3276
P03410	A6NKC0	0.71133
P03410	P51003	0.40883
P03410	Q6XD76	0.31824
P03410	Q8IVG5	0.069293
P03410	Q9H2F5	0.60148
P03410	Q8NHX4	1.3572
P03410	O75363	0.92806
P03410	P43366	0.042291
P03410	Q9NVR5	1.467
P03410	Q96QE5	1.0723
P03410	O95219	0.27154
P03410	Q5JPF3	0.16362
P03410	Q9HBZ2	0.24779
P03410	Q9Y592	1.7348
P03410	Q5RL73	0.43332
P03410	Q9NX55	0.60065
P03410	Q96A49	1.5941
P03410	Q9H7N4	1.591
P03410	Q6ZMK1	0.15938
P03410	P78396	1.1282
P03410	Q9BRU9	0.69127
P03410	Q9GZV8	0.17454
P03410	I3L0S3	1.6271
P03410	Q9UI30	0.76097
P03410	Q9P2Z0	0.71531
P03410	A5D8V6	0.63199
P03410	Q9BW11	0.46522
P03410	Q15006	0.48825
P03410	Q9UJ72	0.22773
P03410	Q9NUQ9	0.083991
P03410	O14965	0.30163
P03410	P43490	0.6455
P03410	Q96EA4	0.94348
P03410	Q9Y603	1.1064
P03410	Q15654	0.92477
P03410	O75688	0.41342
P03410	Q9BZK8	1.6653
P03410	Q9H2X8	0.97393
P03410	Q8TC56	1.9055
P03410	Q13148	0.37028
P03410	Q9P2J5	0.67283
P03410	Q14587	0.65413
P03410	Q9UBQ0	0.97375
P03410	Q8WWB3	0.11361
P03410	Q5SRI9	0.97453
P03410	Q96EL1	1.429
P03410	Q96PE6	0.6823
P03410	Q15928	1.0336
P03410	Q5TAG4	0.95923
P03410	Q8N7H5	0.94981
P03410	Q9BRR0	0.64978
P03410	Q6ZRC1	1.6653
P03410	P08621	0.47843

P03410	P41567	1.4907
P03410	A8MV65	0.96876
P03410	Q05BQ5	0.25175
P03410	Q6ZV56	1.6653
P03410	Q9UNH7	0.44265
P03410	Q9NZJ9	0.32773
P03410	O95243	0.79474
P03410	Q8NDY6	0.58505
P03410	Q9HD26	1.2429
P03410	Q92925	0.62989
P03410	Q8IX19	0.82606
P03410	Q96NL0	0.5948
P03410	P55884	0.35076
P03410	O14925	0.54181
P03410	P41219	0.66988
P03410	Q6ZNG2	0.25012
P03410	A6NJ46	0.27528
P03410	Q5VTQ0	0.42546
P03410	P08865	0.95771
P03410	Q8TAF7	0.99815
P03410	Q969V6	1.39
P03410	Q96L21	0.85317
P03410	Q8N2H9	0.58989
P03410	Q6RFH5	0.25989
P03410	Q8N8L2	0.68881
P03410	Q9Y5M1	0.78672
P03410	A7XYQ1	0.7579
P03410	Q9NRX4	0.2915
P03410	K7EJ46	1.6653
P03410	Q92896	0.8182
P03410	P08779	1.2234
P03410	P49591	0.95868
P03410	Q9UHT4	1.6653
P03410	O43809	0.86167
P03410	Q96NG8	0.78499
P03410	O43708	0.41969
P03410	Q86T29	0.66065
P03410	Q96CM3	0.783
P03410	P37802	0.65045
P03410	Q7Z2E3	0.51786
P03410	Q9ULD5	0.61208
P03410	O95644	0.65482
P03410	Q8N9L7	0.72592
P03410	A8MXJ8	0.13951
P03410	Q96FC7	0.31479
P03410	Q9BQY4	0.91039
P03410	P58340	0.32089
P03410	Q9GZT9	0.2249
P03410	Q8NCF5	0.60453
P03410	Q7RTU0	0.44898
P03410	Q96HB5	0.76904
P03410	Q8N6K4	1.6653
P03410	Q9H4D5	0.9951
P03410	Q96IF1	0.46191
P03410	Q99595	0.27364
P03410	Q8WWM9	0.53232
P03410	Q8N987	0.34414
P03410	Q9ULC6	1.4202
P03410	Q96GX1	0.47229
P03410	O75952	0.56948
P03410	A4D0V7	1.4366
P03410	O95166	0.52224
P03410	Q8TBK6	0.93451
P03410	Q9HD64	0.72206
P03410	O15162	0.49333
P03410	Q02930	0.74696
P03410	Q9P1Z2	0.95006
P03410	Q6RFH8	0.79357
P03410	Q96MA1	0.2145
P03410	P50750	0.28169
P03410	P56381	0.53616
P03410	Q9Y2T3	0.46153
P03410	O95789	1.0468
P03410	Q96LP6	0.13811
P03410	Q96SI1	0.32577
P03410	Q96MN9	0.65208

P03410	P11171	0.86526
P03410	Q13356	0.19963
P03410	P10072	0.81334
P03410	P20930	0.23157
P03410	P40306	0.70857
P03410	P0CJ74	1.4876
P03410	Q8N888	1.0793
P03410	Q9P127	0.86602
P03410	Q9NX58	0.52503
P03410	Q8WV48	1.0913
P03410	Q9H0V1	1.0612
P03410	P0C838	0.99629
P03410	Q96FF7	1.6653
P03410	Q8N111	0.80098
P03410	P52209	0.4266
P03410	Q96NL6	0.59303
P03410	P40123	0.28797
P03410	Q96FG2	0.59182
P03410	Q8N831	0.59269
P03410	Q9UBF6	0.65338
P03410	Q0VDF9	0.42045
P03410	P61981	0.73411
P03410	Q96GD0	0.16404
P03410	Q13336	1.0345
P03410	Q5VWQ0	2.1784
P03410	Q9UII2	0.97437
P03410	Q6ZSN7	1.2928
P03410	Q00994	1.4249
P03410	Q9P2J3	0.24616
P03410	Q93099	0.40518
P03410	Q92979	0.44156
P03410	Q9Y5Q3	0.70505
P03410	Q9H1Z4	0.49184
P03410	P10746	0.57532
P03410	Q5SWH9	1.0913
P03410	Q92800	0.47264
P03410	P26358	0.29496
P03410	P27540	0.27991
P03410	Q6IPR3	0.52345
P03410	O15535	0.57148
P03410	A6NNV3	1.0907
P03410	O60384	0.967
P03410	P67809	0.52366
P03410	A8MX19	0.13085
P03410	Q6GPH4	0.43664
P03410	Q5T5Y3	0.73957
P03410	Q8WYA6	0.91102
P03410	Q86WI3	0.60263
P03410	Q5SRH9	1.5131
P03410	Q53HI1	1.1628
P03410	Q96D03	0.46495
P03410	Q9NYV6	0.57856
P03410	Q86WV5	0.73197
P03410	Q99081	0.28473
P03410	Q6ZUF6	1.1327
P03410	Q9UNX3	1.6046
P03410	Q5T2T1	0.52641
P03410	Q63HM2	0.67427
P03410	Q9Y2G4	0.24495
P03410	O43805	0.82366
P03410	Q99816	0.52481
P03410	O14598	1.4395
P03410	Q9GZY1	1.0952
P03410	Q6ZQR2	1.7643
P03410	Q9UBP8	0.97973
P03410	Q9ULC4	0.48697
P03410	P58505	0.92353
P03410	O00625	0.90708
P03410	Q9NR09	0.40808
P03410	Q96KT7	1.1631
P03410	Q9UM54	0.11455
P03410	Q8N8Z3	0.52862
P03410	P33316	0.74831
P03410	Q9H790	0.44399
P03410	Q15599	0.66395
P03410	Q5K131	0.52932

P03410	Q6ZN06	0.95938
P03410	Q9UHT9	1.6653
P03410	A8MQB3	0.49035
P03410	Q9UHB7	1.2457
P03410	Q96BV0	0.59395
P03410	Q14590	0.6829
P03410	O95158	0.87371
P03410	O75151	0.48575
P03410	Q9UHJ9	1.0193
P03410	Q96B36	1.6774
P03410	Q15628	0.81071
P03410	Q9HC16	0.20325
P03410	Q9H2H8	0.33677
P03410	Q902F8	1.1446
P03410	A6NCW3	1.2555
P03410	Q06323	1.3685
P03410	Q9UK10	0.79465
P03410	Q9BRT7	1.6653
P03410	Q5TZK3	0.2149
P03410	Q9BUG6	0.52321
P03410	O95425	0.085959
P03410	P62277	1.2546
P03410	O15371	0.86949
P03410	Q8IZH2	0.29331
P03410	Q7L8J4	0.77627
P03410	Q5T0F9	0.55712
P03410	O60563	1.5585
P03410	Q8NEK8	1.414
P03410	Q13127	0.39916
P03410	Q8N1N2	1.4135
P03410	Q3ZCV2	1.4883
P03410	Q8WXB4	0.67112
P03410	Q8NHV4	1.0774
P03410	Q96MW5	0.38858
P03410	Q8NI28	0.75446
P03410	O60308	0.83152
P03410	Q9NX24	0.25964
P03410	P0CG42	1.6653
P03410	Q86YS3	0.39181
P03410	P17022	0.46379
P03410	Q96GE5	0.94919
P03410	O60333	0.26312
P03410	Q5SXM1	0.88569
P03410	Q9NWD8	0.67444
P03410	O43318	0.84996
P03410	Q86TU6	1.8796
P03410	Q8NEH6	1.4517
P03410	O00716	0.9755
P03410	Q9UKT9	0.39189
P03410	Q9H1L0	1.7243
P03410	A6NKF7	1.0913
P03410	Q0VAF6	0.13502
P03410	Q15032	0.46946
P03410	O15525	0.40117
P03410	Q7Z5Q5	0.38414
P03410	Q16829	0.52002
P03410	Q4VXA5	0.85912
P03410	P25800	0.29844
P03410	Q8N4B4	0.31319
P03410	P08651	0.39144
P03410	P60673	0.48082
P03410	Q92624	1.3796
P03410	Q9Y4H4	1.4726
P03410	O75970	0.4156
P03410	Q9HCS7	0.50089
P03410	Q7Z5V6	0.86197
P03410	Q86WZ0	1.3768
P03410	Q6ZMS7	0.74848
P03410	Q9NTU4	1.3117
P03410	Q8N0Z3	0.87824
P03410	P00480	0.49118
P03410	Q9Y2X9	0.55127
P03410	P22695	0.11679
P03410	A2IDD5	0.9548
P03410	O94906	0.32494
P03410	Q8IZL2	0.9995

P03410	Q13136	0.5178
P03410	O95848	1.3672
P03410	Q93100	0.82983
P03410	Q15583	0.32143
P03410	Q7L5N1	1.3176
P03410	Q9GZU3	1.6533
P03410	P0CW21	1.6653
P03410	Q6ZMS4	0.75415
P03410	Q08AN1	0.79465
P03410	Q5VWP2	0.4146
P03410	Q9BUK6	0.306
P03410	Q70IA8	0.18173
P03410	Q9Y676	1.4249
P03410	Q8NE86	0.64139
P03410	P36578	1.2011
P03410	Q9UGP5	0.2934
P03410	Q9UMX0	1.0238
P03410	B7ZAP0	0.0064086
P03410	Q8N1G4	0.62496
P03410	Q9BYD1	1.7652
P03410	Q8N201	0.68895
P03410	O43715	0.99196
P03410	Q8WU49	1.6653
P03410	Q86X02	0.44597
P03410	P41161	0.25951
P03410	P61573	1.1462
P03410	Q8IYE0	1.4251
P03410	P17029	0.84739
P03410	Q7L3S4	0.77956
P03410	Q96M85	1.6653
P03410	Q8IVP9	0.63916
P03410	Q9UBZ9	0.48531
P03410	P22557	0.21335
P03410	Q86TL0	0.55652
P03410	Q9UMR2	0.30975
P03410	P18077	0.72038
P03410	A6NIX2	1.0319
P03410	O75600	0.11663
P03410	Q86XI6	0.30851
P03410	Q9BT04	1.118
P03410	Q8WYR4	0.43881
P03410	O95571	0.65159
P03410	Q92988	0.50842
P03410	Q3MIV0	1.6653
P03410	Q8N614	0.70848
P03410	Q9BSF4	0.52164
P03410	Q5MJ10	1.5851
P03410	Q9BYD9	0.42594
P03410	Q9NS25	1.6831
P03410	Q7Z7J5	1.2102
P03410	Q9UNQ2	0.9588
P03410	Q9UJA5	1.604
P03410	Q0P670	1.5437
P03410	P12956	0.31552
P03410	Q6VN20	0.48403
P03410	A0ZSE6	0.9149
P03410	Q92556	0.52541
P03410	Q96LM1	1.2185
P03410	Q9C0B9	0.24486
P03410	O76031	1.043
P03410	Q96MC4	1.9477
P03410	O95866	1.1982
P03410	Q8N999	1.0924
P03410	Q96AT1	1.4883
P03410	Q96A59	1.2078
P03410	Q9NPA8	0.48411
P03410	Q9C035	0.56963
P03410	Q9UNE7	0.23018
P03410	Q8N823	0.79465
P03410	Q5T7W7	1.4883
P03410	Q12986	0.21125
P03410	Q8N766	0.7443
P03410	P24385	0.88552
P03410	Q9Y3E7	1.062
P03410	Q9Y3P8	0.97135
P03410	Q9UDY2	1.2863

P03410	Q8IZN7	0.92904
P03410	Q92628	0.8309
P03410	Q99990	0.67767
P03410	Q9Y2W2	1.5564
P03410	Q5VWM5	1.1303
P03410	Q8WWY6	0.54613
P03410	P0DI81	1.1051
P03410	Q9BTV4	0.85342
P03410	P07910	0.3269
P03410	Q9Y4W2	1.5928
P03410	Q9H3F6	0.39138
P03410	A8MUQ0	1.3804
P03410	Q7Z340	0.75755
P03410	Q9UBZ4	0.036616
P03410	Q8TB69	0.96412
P03410	Q86UD1	0.51339
P03410	Q5H9L4	0.33768
P03410	P42166	0.51669
P03410	Q13492	0.28663
P03410	P08579	0.30045
P03410	Q96IY1	0.99342
P03410	P55199	1.1084
P03410	Q96JK9	1.0005
P03410	Q8WZ33	0.88066
P03410	Q9Y6Z4	0.76299
P03410	Q07687	0.40973
P03410	Q6ZRS2	0.36515
P03410	Q96H40	0.92169
P03410	Q86UT6	0.36123
P03410	O95551	0.71399
P03410	P41227	0.41987
P03410	Q9BW61	1.1536
P03410	Q99471	0.9321
P03410	P35548	0.87103
P03410	Q13618	0.4296
P03410	Q9P0M2	0.21976
P03410	P0CJ76	1.4876
P03410	Q15436	1.0652
P03410	Q5VT33	0.78403
P03410	Q9BXI3	0.41616
P03410	Q8NEK5	0.82883
P03410	Q8IZD4	0.98285
P03410	A5PL33	1.0654
P03410	Q7RTT5	0.38119
P03410	Q5GH76	1.1702
P03410	Q8IZW8	0.85052
P03410	Q93052	0.51414
P03410	O14734	0.72324
P03410	Q6R2W3	0.56406
P03410	Q3KQV3	0.81355
P03410	Q9P031	1.6162
P03410	O14715	0.20843
P03410	Q9NPB8	0.062492
P03410	Q8WV24	1.0917
P03410	Q6ZVS7	0.053959
P03410	O00154	0.015886
P03410	Q96MP8	0.42699
P03410	Q9BWT6	0.14926
P03410	Q86V42	1.056
P03410	P17098	1.0155
P03410	Q8WUX9	0.6478
P03410	Q96LL3	1.297
P03410	Q92793	0.34151
P03410	O95905	0.79594
P03410	C9JLW8	0.97389
P03410	P25398	0.81758
P03410	Q9NQR4	0.32899
P03410	P63208	0.79472
P03410	Q9BVK6	0.36156
P03410	Q14966	0.2755
P03410	Q9NWQ4	1.3622
P03410	Q15486	0.83308
P03410	Q13472	0.055772
P03410	P59991	1.8484
P03410	P08133	1.0371
P03410	P53674	0.51352

P03410	Q969S8	0.86863
P03410	Q6B0B8	0.80441
P03410	Q16537	0.62959
P03410	O00567	0.9701
P03410	Q8TBZ5	0.83916
P03410	Q13510	0.66314
P03410	P38935	0.31455
P03410	Q6P1R3	0.77205
P03410	O95707	1.587
P03410	P29474	0.87542
P03410	Q8IYF1	0.56341
P03410	Q8N3F8	0.74226
P03410	H0YL14	1.4551
P03410	Q8NA96	1.1653
P03410	Q9H0H0	0.43228
P03410	P0C843	0.77013
P03410	P35251	0.74454
P03410	P49756	0.4575
P03410	Q9BR11	0.30472
P03410	C9J6K1	0.86271
P03410	Q8TEV8	1.6653
P03410	Q86Y01	0.357
P03410	O14576	0.68396
P03410	Q8N205	1.0045
P03410	Q8N9Y4	1.3862
P03410	Q9H6F2	0.66965
P03410	O60936	1.7285
P03410	Q2KHM9	0.73201
P03410	Q719H9	0.68638
P03410	Q8IYT1	0.85747
P03410	Q8NEZ2	1.0419
P03410	Q6ZVF9	1.0264
P03410	Q15573	0.92412
P03410	Q9H9H5	1.6134
P03410	Q86YF9	0.26238
P03410	O60763	0.77225
P03410	Q6ZTR7	0.16187
P03410	Q9Y3B6	1.4005
P03410	Q96B26	0.62639
P03410	Q96JC9	1.0364
P03410	Q14677	0.71392
P03410	Q5T6S3	0.36494
P03410	Q63HM1	0.71422
P03410	Q8N0U7	0.90509
P03410	Q9NRD5	0.2719
P03410	Q8TDX5	0.36255
P03410	Q13200	0.88024
P03410	Q99828	0.39012
P03410	Q96KR4	0.39217
P03410	Q8NDN9	0.59228
P03410	Q9BWM5	0.81377
P03410	B6SEH8	0.64028
P03410	Q9Y5L0	1.1904
P03410	O60810	1.0571
P03410	P43307	0.63287
P03410	Q8NCT3	1.5891
P03410	P49790	1.1617
P03410	Q8IY85	0.39612
P03410	Q6NUN9	0.72582
P03410	Q8IX07	0.28638
P03410	Q05519	0.9699
P03410	Q9UJ68	0.41053
P03410	Q9P2D6	0.17758
P03410	Q8N8A2	0.6999
P03410	O43818	0.24723
P03410	Q92552	1.1461
P03410	Q8IXH7	1.3177
P03410	Q8TE85	0.24982
P03410	Q8IY57	0.9455
P03410	Q9ULK4	1.2708
P03410	P41223	0.17863
P03410	P48775	0.50581
P03410	B1ANY3	0.77616
P03410	Q9H8Y8	0.77637
P03410	Q96DR8	0.9256
P03410	A6NJY4	1.6653

P03410	Q9Y6X1	0.41543
P03410	Q53QV2	0.81206
P03410	Q13287	0.61024
P03410	Q9Y2F5	0.32464
P03410	Q9BXV9	1.6653
P03410	Q9BT92	1.0528
P03410	Q9Y448	1.071
P03410	Q9H8M1	0.52423
P03410	Q9Y234	0.60574
P03410	A8MWA4	0.98395
P03410	P09972	0.66718
P03410	Q3KNT7	0.35754
P03410	Q9H814	1.7104
P03410	Q9NRX5	0.55074
P03410	Q495D7	0.57134
P03410	O95402	1.0753
P03410	Q9BSJ6	0.84617
P03410	Q14764	1.4282
P03410	Q7Z7H3	0.34786
P03410	H3BMG3	1.2869
P03410	Q6ZSA8	1.6653
P03410	Q6N069	0.24586
P03410	Q9Y2Q1	0.98093
P03410	O75150	0.25684
P03410	Q3KNS6	0.70364
P03410	Q9NXE8	1.0349
P03410	Q6ZS49	1.6653
P03410	Q9UQN3	0.72751
P03410	P48382	0.91742
P03410	Q8WX92	1.4734
P03410	P51814	0.59861
P03410	P63098	0.22805
P03410	Q6IQ32	0.28172
P03410	O94986	1.4919
P03410	Q9Y6P5	0.70469
P03410	P10074	0.63529
P03410	O14531	0.2534
P03410	Q15561	0.79171
P03410	Q5XG85	1.6653
P03410	P50552	0.28895
P03410	Q9Y3D2	0.49742
P03410	P62937	0.62271
P03410	Q14315	0.60687
P03410	O60269	0.6081
P03410	Q96F86	0.84487
P03410	P03886	0.38326
P03410	Q96MG8	0.14701
P03410	Q53H64	0.57718
P03410	Q9UKV3	0.66568
P03410	Q9GZY6	0.66052
P03410	Q6P2H8	1.2245
P03410	Q2M243	0.16032
P03410	Q5H9S7	1.2538
P03410	P10244	0.63753
P03410	P25787	0.92998
P03410	Q7Z7G2	0.86666
P03410	Q14980	1.2895
P03410	P61927	1.2706
P03410	P52701	0.087944
P03410	Q6IQ19	1.0684
P03410	Q8NHY5	1.4367
P03410	Q99619	0.50055
P03410	Q53F19	1.4563
P03410	Q96KN4	0.20978
P03410	P15036	0.79217
P03410	Q9NUN5	0.68093
P03410	Q1W209	1.6653
P03410	Q96DT0	0.2656
P03410	Q08AM6	0.4267
P03410	O00570	0.79738
P03410	Q969G3	1.1708
P03410	Q6ZNH5	0.80188
P03410	Q8TCE6	1.6077
P03410	Q9UL40	0.77638
P03410	Q8IUD2	1.1217
P03410	Q8WVK2	1.3513

P03410	Q9HC62	0.58607
P03410	P49789	0.59998
P03410	Q8IU81	0.20863
P03410	O15297	0.65996
P03410	Q2M3C7	0.80557
P03410	Q8WXW3	1.2908
P03410	P84098	1.0096
P03410	Q9NQV7	0.79278
P03410	P29084	1.6055
P03410	Q8N485	0.53775
P03410	A9UHW6	0.89546
P03410	Q7Z6I8	0.092558
P03410	Q09028	0.60898
P03410	Q9UI72	1.6653
P03410	Q4U2R6	1.4261
P03410	O00483	0.31682
P03410	P68871	0.98234
P03410	Q9UM82	1.6745
P03410	O15084	1.0885
P03410	Q9BTK6	0.68172
P03410	Q96BN2	1.4176
P03410	P61571	0.89339
P03410	Q9NW08	0.14142
P03410	Q96P15	0.19809
P03410	Q15369	1.4183
P03410	Q9BV97	0.66822
P03410	Q99623	1.2029
P03410	Q5VX52	0.34433
P03410	P0CW24	0.38853
P03410	Q8WZ26	0.6634
P03410	Q6ZRT6	1.4883
P03410	Q6NUJ2	1.0913
P03410	P62854	1.2189
P03410	Q8TAP6	1.4102
P03410	Q96AA8	0.71164
P03410	P61236	0.58798
P03410	O14523	0.96804
P03410	Q8TEB1	0.51567
P03410	Q6ZMV9	0.34531
P03410	Q6UWT4	0.95938
P03410	Q9P1T7	1.2143
P03410	Q9UER7	1.3112
P03410	P33552	0.87864
P03410	O95453	0.67318
P03410	Q2TAY7	0.34399
P03410	P17026	0.66742
P03410	Q9HAT0	0.25424
P03410	Q9NZJ4	0.18996
P03410	P14635	0.44479
P03410	Q4VX62	0.66088
P03410	P0CG43	1.6653
P03410	Q6P2E9	1.0467
P03410	Q6UWI2	1.2671
P03410	Q5VZ66	0.72989
P03410	Q9NWS0	0.58595
P03410	Q8NHV5	0.15421
P03410	A6NL82	0.90091
P03410	Q69YN4	0.67519
P03410	Q01538	0.36781
P03410	Q8IUC1	0.71041
P03410	Q96KR7	0.76037
P03410	A8MZ59	0.2823
P03410	P07951	0.033227
P03410	O75396	0.66007
P03410	O95999	0.3175
P03410	P51397	1.1643
P03410	Q96BY9	0.32265
P03410	F2Z3M2	1.6653
P03410	P61570	1.1647
P03410	Q8NAJ2	0.30819
P03410	Q9UKS6	0.48305
P03410	P15502	1.0012
P03410	Q92782	0.57539
P03410	I3L1E1	0.95928
P03410	Q8IXM2	0.27311
P03410	Q8NF67	0.90864

P03410	P49773	0.076205
P03410	Q8TAT2	0.77064
P03410	Q9BX26	1.749
P03410	Q9NTJ4	0.32717
P03410	Q9BS31	0.64747
P03410	Q99459	0.93726
P03410	Q15555	0.36411
P03410	Q9NQ55	0.61669
P03410	Q9Y608	0.46744
P03410	Q9UM11	1.2975
P03410	Q8NFC6	1.5331
P03410	Q14511	0.9975
P03410	O75400	1.0061
P03410	O95298	1.2759
P03410	Q16649	0.20401
P03410	P12270	0.79365
P03410	Q8WWQ0	0.71769
P03410	P21399	0.46199
P03410	Q9HBM6	0.72949
P03410	P38936	0.32885
P03410	Q9ULE4	0.90822
P03410	Q5MJ09	0.51657
P03410	Q6ICB0	1.3348
P03410	Q6EKJ0	0.22671
P03410	Q9UN36	0.36858
P03410	Q9UIL8	0.36494
P03410	Q6P2H3	0.83781
P03410	Q92769	0.38722
P03410	Q9HCE3	0.10142
P03410	O43513	1.2205
P03410	Q9NWS6	1.0302
P03410	Q96FX8	1.2551
P03410	Q02386	0.66023
P03410	C9JDP6	0.64164
P03410	Q8NEA6	0.24335
P03410	P12882	0.25332
P03410	Q9Y4A5	0.76754
P03410	Q9BPY3	0.40016
P03410	Q96KG9	0.32145
P03410	Q9NRL2	0.96912
P03410	Q9H4K1	1.0034
P03410	Q9Y376	0.31567
P03410	Q15691	0.92112
P03410	Q9BWX5	0.26495
P03410	Q68CZ2	0.73803
P03410	O94805	0.6915
P03410	P58512	1.4669
P03410	P0CG31	0.75111
P03410	P43243	0.26114
P03410	P0DJ07	1.1965
P03410	Q8NEV8	0.30879
P03410	Q9BU61	0.98757
P03410	P04731	0.83308
P03410	Q499Z3	0.71071
P03410	Q5T3I0	0.20674
P03410	Q96JM7	0.37631
P03410	Q13042	1.5455
P03410	O43172	0.6895
P03410	Q96E40	0.16794
P03410	Q9UK12	0.81145
P03410	Q96GY3	0.95273
P03410	Q9NVR7	0.53954
P03410	Q8N9Z9	0.95539
P03410	Q9H0R8	0.56661
P03410	Q6ZVN8	0.52961
P03410	P52737	0.79497
P03410	Q9BW85	1.5317
P03410	Q9NSG2	1.2047
P03410	P48454	0.27842
P03410	P26038	0.31592
P03410	P0CG13	0.30063
P03410	A0PK05	0.78375
P03410	Q969M3	0.44427
P03410	Q96NT3	0.72838
P03410	Q96I27	0.71807
P03410	Q7Z398	0.76183

P03410	Q5TGP6	0.12523
P03410	P35908	0.68428
P03410	Q9BSH3	0.36374
P03410	Q13342	0.54581
P03410	O43236	0.40738
P03410	O95996	0.59067
P03410	Q68D10	1.4883
P03410	Q9Y6B2	0.8933
P03410	Q15437	0.78628
P03410	Q9UIL1	1.038
P03410	Q8WXK3	0.25857
P03410	Q5VTU8	0.48502
P03410	O75915	0.84732
P03410	P52435	0.67527
P03410	Q9P1J3	1.6653
P03410	Q9NRJ2	1.6653
P03410	P45985	0.24504
P03410	Q86T75	1.289
P03410	Q5SQS8	1.344
P03410	Q96EL3	0.49843
P03410	Q8NA58	0.76206
P03410	O14508	0.34854
P03410	Q8N5A5	0.72086
P03410	O75438	0.35317
P03410	Q9NPI0	0.87881
P03410	Q92797	1.0213
P03410	Q8N2Y8	0.22961
P03410	Q13309	0.67045
P03410	Q9NUU7	0.3254
P03410	Q5TGS1	0.52912
P03410	Q13522	0.40628
P03410	Q17RB0	0.97842
P03410	Q6ZUJ8	0.25365
P03410	Q96G27	1.0975
P03410	P43362	0.2347
P03410	P62826	0.7585
P03410	Q9H489	0.53274
P03410	Q9C010	1.4108
P03410	Q7Z2X4	0.30261
P03410	O60225	0.35782
P03410	Q6PEV8	0.15039
P03410	A6NCD4	0.86954
P03410	P62273	1.619
P03410	O00629	0.75029
P03410	A6NFC5	1.1445
P03410	P26639	0.76571
P03410	Q96E16	1.2321
P03410	Q8IY92	0.5773
P03410	P59051	1.0799
P03410	O95602	0.58115
P03410	Q9UPZ3	1.0299
P03410	Q8TAG9	0.19462
P03410	Q86XN7	1.4883
P03410	Q8NEQ5	0.66098
P03410	O60684	0.58714
P03410	Q8NBD8	1.154
P03410	P17535	0.94697
P03410	Q9NRX2	1.23
P03410	Q9UDW1	0.39761
P03410	Q496A3	0.616
P03410	O94913	0.47462
P03410	Q13278	1.4864
P03410	Q96GM8	0.49394
P03410	O14558	0.95729
P03410	Q8N972	0.79465
P03410	Q96B70	0.50384
P03410	I6L899	0.2002
P03410	Q5JTZ5	0.26947
P03410	Q3ZCQ8	0.2571
P03410	O43261	0.6999
P03410	Q8IUR0	1.5692
P03410	Q49AG3	1.0618
P03410	A6NLC8	1.4469
P03410	Q4W5G0	0.70588
P03410	Q96HH6	1.0913
P03410	Q9H492	0.46119

18925	P03410	Q9UHQ1	1.1243
18926	P03410	O43502	0.26421
18927	P03410	Q9P0R6	0.64384
18928	P03410	P62140	0.73592
18929	P03410	P35663	0.60562
18930	P03410	P07738	0.96581
18931	P03410	Q9BQ49	1.0913
18932	P03410	P09132	0.44689
18933	P03410	C9JC47	1.6653
18934	P03410	O43491	0.51481
18935	P03410	Q9Y294	1.0544
18936	P03410	O94842	0.80528
18937	P03410	Q9BYC8	1.3965
18938	P03410	P02489	0.36572
18939	P03410	Q9BS40	0.49241
18940	P03410	Q7Z3I7	0.78003
18941	P03410	Q6ZN79	0.88895
18942	P03410	O00264	0.073859
18943	P03410	Q96N76	0.41754
18944	P03410	Q15170	2.0626
18945	P03410	P15170	0.33604
18946	P03410	Q9P2D1	0.18629
18947	P03410	O60739	0.6164
18948	P03410	Q9NPE6	0.24758
18949	P03410	Q6P9G9	0.70056
18950	P03410	E9PQ53	1.3979
18951	P03410	P50990	0.097111
18952	P03410	P0C6E6	0.39375
18953	P03410	Q8WYN3	0.97715
18954	P03410	Q96CX2	0.25004
18955	P03410	Q8N163	0.84404
18956	P03410	Q8IU53	1.4849
18957	P03410	Q96DE9	0.31121
18958	P03410	Q5T280	1.7826
18959	P03410	Q9UGK8	0.79193
18960	P03410	Q9Y2G1	0.34424
18961	P03410	Q2VWA4	0.10041
18962	P03410	O75323	0.91229
18963	P03410	P18545	0.6307
18964	P03410	P0CG08	0.86829
18965	P03410	Q6PDB4	1.0114
18966	P03410	Q9UPV9	0.91397
18967	P03410	Q969H0	0.67477
18968	P03410	Q969W8	0.91156
18969	P03410	P51959	0.59922
18970	P03410	Q96CM4	0.31355
18971	P03410	P17028	0.70249
18972	P03410	Q8N883	0.73633
18973	P03410	Q96G28	0.63309
18974	P03410	Q8WWZ8	0.24073
18975	P03410	Q49A33	0.98227
18976	P03410	Q8NCK3	0.7682
18977	P03410	Q7L4S7	0.72156
18978	P03410	A6NI87	0.90573
18979	P03410	Q9NYU1	0.40331
18980	P03410	Q9NQM4	0.21587
18981	P03410	Q9BUN8	0.47491
18982	P03410	Q86YV9	1.5915
18983	P03410	Q8N4P2	0.3808
18984	P03410	Q6SJ96	0.33706
18985	P03410	Q9BW30	0.51121
18986	P03410	Q9BXM8	1.6653
18987	P03410	Q8N5N7	1.2448
18988	P03410	Q5T1C6	1.1824
18989	P03410	Q6P5X5	1.3579
18990	P03410	Q6UN15	1.8819
18991	P03410	P56181	1.3932
18992	P03410	P53370	0.45183
18993	P03410	Q86X53	0.36456
18994	P03410	Q9BPU9	0.43241
18995	P03410	O15287	1.1881
18996	P03410	Q9UKN5	0.25782
18997	P03410	Q6L8G9	1.6653
18998	P03410	Q3B7S5	1.6653
18999	P03410	P61586	0.3747
19000	P03410	P17019	1.0122

P03410	A6NLW8	0.27528
P03410	Q9BQI6	0.39549
P03410	Q5SZL2	0.57417
P03410	Q07955	0.61539
P03410	Q15942	0.48297
P03410	P52597	0.44027
P03410	Q96C00	0.3566
P03410	P53805	0.57697
P03410	O95424	1.0102
P03410	Q9BWC9	1.6911
P03410	Q9H4G8	0.66015
P03410	A4D2H0	0.094755
P03410	Q8IZM0	0.89104
P03410	Q8NAP3	0.3043
P03410	Q3LI59	1.6653
P03410	Q9UNY5	0.79031
P03410	A6NIN4	1.6653
P03410	Q03924	0.7518
P03410	Q5TA82	1.6653
P03410	C9JVW0	1.6653
P03410	Q96RQ1	0.20035
P03410	O75132	0.28581
P03410	Q8TAE6	1.0879
P03410	Q6NUR6	1.2302
P03410	Q9BZ68	0.10086
P03410	Q7Z5L2	0.76737
P03410	P35658	1.1708
P03410	Q5VWN6	0.57234
P03410	Q96GE4	0.54722
P03410	P08107	0.53363
P03410	Q9Y3S2	0.75928
P03410	P52298	0.62417
P03410	Q9H9Y2	0.39424
P03410	P62081	1.1988
P03410	Q9Y3C5	0.39359
P03410	B2RXF0	0.48219
P03410	Q13395	0.14994
P03410	Q16514	0.74395
P03410	Q96JN2	0.9877
P03410	P28347	0.94682
P03410	P0CL82	0.508
P03410	Q9HA82	0.63139
P03410	Q9NV29	0.97067
P03410	Q16637	0.97209
P03410	P46783	1.4523
P03410	Q9H9F9	1.1697
P03410	Q7Z6J0	0.24798
P03410	O75608	0.22444
P03410	Q86XK2	0.49701
P03410	Q9NPI8	1.4292
P03410	P78395	1.1776
P03410	Q9BXL7	0.24032
P03410	Q6PJP8	0.76554
P03410	Q9H0I2	0.10748
P03410	Q5SZB4	1.0328
P03410	Q7Z353	0.2823
P03410	Q32MQ0	1.0532
P03410	Q8N357	0.72253
P03410	Q9H6F5	0.57933
P03410	P60368	1.1232
P03410	Q8IY31	0.94343
P03410	Q9BSH5	0.70164
P03410	Q86UY8	0.18748
P03410	Q6ZSB3	0.12409
P03410	P25063	1.6653
P03410	Q68DY9	0.85321
P03410	Q5VT40	0.94825
P03410	P61574	1.1462
P03410	Q0VD67	0.30412
P03410	P11686	0.084724
P03410	Q96GL9	0.7782
P03410	Q9BRJ7	1.1725
P03410	Q03252	0.5945
P03410	P17030	0.78811
P03410	O75534	0.43869
P03410	O75190	0.74666

P03410	Q9BS34	0.68818
P03410	P04818	0.27616
P03410	Q9BUL9	1.5656
P03410	P62495	0.74828
P03410	Q9H094	0.15079
P03410	Q8IXQ6	0.87264
P03410	Q99439	0.27798
P03410	Q8NC54	1.6503
P03410	Q9NYL9	0.21344
P03410	Q96ED9	0.84724
P03410	P52743	0.90046
P03410	Q9P0L1	0.8261
P03410	A6NGG8	0.90581
P03410	P27469	0.84367
P03410	P30304	0.77525
P03410	Q9BPX5	0.21769
P03410	Q3LHN1	1.6653
P03410	Q9UBS8	0.40176
P03410	A6NCI5	1.7191
P03410	P24941	0.83473
P03410	Q8TBZ9	1.6684
P03410	P00156	0.82715
P03410	Q96I59	0.97231
P03410	Q8TAI1	1.3774
P03410	Q9P2I0	1.0052
P03410	O75525	0.70767
P03410	Q96I23	0.58423
P03410	Q07157	0.91456
P03410	P08697	0.22524
P03410	O14513	0.92154
P03410	Q8NEZ5	0.60575
P03410	Q6IN84	0.62379
P03410	Q8TD47	0.45192
P03410	Q9UK28	1.1582
P03410	P31943	0.38337
P03410	Q96QU4	1.8316
P03410	Q8N8E1	1.1862
P03410	Q8TB05	1.4883
P03410	Q969P5	0.17249
P03410	P17275	0.85287
P03410	P62256	0.40985
P03410	Q9UKZ1	1.5859
P03410	Q99638	0.76218
P03410	Q8TBY8	1.1358
P03410	O00170	0.65266
P03410	Q8IY50	0.96368
P03410	Q6L8H2	1.6653
P03410	Q13129	0.74555
P03410	Q5SW24	0.46891
P03410	Q00597	0.99689
P03410	O15527	0.56065
P03410	Q9NQR1	0.57625
P03410	Q92499	0.30858
P03410	Q9NWB7	0.88004
P03410	Q12772	0.23752
P03410	Q8IZS5	0.8956
P03410	Q9BYP9	1.9984
P03410	A6NLX4	1.0913
P03410	Q969E4	0.54613
P03410	C9J302	0.8204
P03410	O15155	0.51035
P03410	P62314	0.89985
P03410	Q9BYJ9	0.88339
P03410	Q68G75	0.49347
P03410	Q13330	0.4019
P03410	Q3KRA6	1.8909
P03410	Q2KHT3	1.4353
P03410	Q9UMX3	0.74117
P03410	Q9ULV3	0.47566
P03410	Q75NE6	2.0731
P03410	Q969G5	0.7377
P03410	Q16621	0.31461
P03410	Q9BTW9	0.47157
P03410	P60896	1.6653
P03410	Q9BSU1	1.0188
P03410	Q9Y253	0.25192

P03410	Q16762	0.42993
P03410	Q7Z591	1.7798
P03410	Q96JX3	0.53671
P03410	P0CW71	1.6653
P03410	O00463	0.37285
P03410	P60369	1.9984
P03410	Q86WC4	1.5232
P03410	Q5VU36	1.0269
P03410	Q86SQ9	0.23906
P03410	Q92990	0.61399
P03410	P49848	0.65209
P03410	Q8WZ82	0.096948
P03410	P52736	0.6971
P03410	Q5T681	1.4895
P03410	O43660	0.31092
P03410	Q92973	1.3067
P03410	Q9UPA5	0.49769
P03410	Q9BUH8	0.79738
P03410	P07954	0.20725
P03410	Q9BW60	0.86335
P03410	Q86UR1	0.30829
P03410	A6NGD5	0.57102
P03410	Q9UGR2	0.71172
P03410	Q8N655	1.6653
P03410	B2CW77	1.6653
P03410	Q9NYJ8	0.88304
P03410	Q7Z745	1.0105
P03410	Q9HD42	1.0746
P03410	P0CI00	1.093
P03410	Q9UHJ3	0.51441
P03410	Q7RTU9	0.3193
P03410	Q96L03	0.23126
P03410	A8MV81	0.99677
P03410	Q6NT04	0.58789
P03410	O14561	0.67201
P03410	Q96D71	0.30953
P03410	Q96BN6	0.57197
P03410	Q9UBC1	0.73881
P03410	O95429	0.55909
P03410	Q96LT7	1.2395
P03410	O14787	1.2524
P03410	Q5JXC2	0.32364
P03410	A6NGY1	1.5377
P03410	P83369	0.95129
P03410	P0CJ89	0.27528
P03410	Q7Z2W4	0.60114
P03410	Q9ULD2	0.7268
P03410	Q14558	0.2615
P03410	Q96I36	1.1637
P03410	Q969D9	0.77674
P03410	O75800	0.64825
P03410	Q86XF0	0.32623
P03410	Q9UKY0	0.34442
P03410	Q8IVN3	1.6658
P03410	Q5T5J6	1.0312
P03410	Q9ULG3	1.6653
P03410	Q9Y5Z7	0.63037
P03410	Q8TDC0	0.87815
P03410	Q9BRX2	0.3575
P03410	Q8TAK5	0.62448
P03410	Q9NWV4	1.1417
P03410	P25208	0.70711
P03410	Q6NVV1	0.77524
P03410	Q8N187	1.3223
P03410	Q6ZR62	0.22127
P03410	Q96BZ8	1.4883
P03410	Q9Y2T2	1.0019
P03410	Q8N9S7	0.78333
P03410	Q9BXX3	0.27664
P03410	Q9NP61	0.10013
P03410	Q9HBF5	1.4094
P03410	P62324	0.76299
P03410	P14927	0.16084
P03410	Q86UY6	0.364
P03410	Q96AH0	0.83956
P03410	Q9UHZ2	1.6653

P03410	Q9UGN5	0.29173
P03410	P51815	0.57594
P03410	Q6ZW54	1.6327
P03410	P60602	1.0371
P03410	Q86YA3	1.746
P03410	O96007	1.2991
P03410	P00338	0.69158
P03410	P60329	0.53228
P03410	Q9NXR8	0.23713
P03410	P0CJ68	1.4876
P03410	Q9H9L7	0.41305
P03410	P04553	1.6653
P03410	O14613	0.38117
P03410	A1L429	0.508
P03410	Q5TZA2	1.0274
P03410	Q13371	0.53771
P03410	Q9UN37	0.64014
P03410	O95163	0.59964
P03410	A6NK53	0.84221
P03410	O95271	0.6782
P03410	Q14D33	0.80531
P03410	Q9BQW3	0.15485
P03410	Q96A65	0.34371
P03410	Q5D1E8	0.98922
P03410	Q5T8A7	1.7091
P03410	Q9BR77	0.72855
P03410	Q9Y678	0.83283
P03410	Q8NFH5	0.78283
P03410	P51587	0.17316
P03410	Q15056	0.48873
P03410	O14604	1.3071
P03410	A0PJW6	1.2071
P03410	Q96M19	1.6653
P03410	O43482	0.77488
P03410	Q14872	0.69271
P03410	Q8N5W9	0.8007
P03410	P11802	0.88014
P03410	O75362	0.77062
P03410	Q86SH4	1.6653
P03410	Q9UJW8	0.70757
P03410	Q96K83	0.65426
P03410	Q9Y2J2	0.75367
P03410	Q86UE4	1.0499
P03410	O14633	1.6653
P03410	Q86Y26	1.2725
P03410	Q9BSR8	0.91008
P03410	P56645	0.2465
P03410	Q9BTE1	0.3429
P03410	P0CG39	0.23109
P03410	Q9H5L6	0.77657
P03410	Q6P1K2	1.1576
P03410	Q16775	0.65159
P03410	Q9BW04	0.28715
P03410	Q6ISB3	0.62845
P03410	Q9NRR1	0.9328
P03410	Q8N0Z8	1.3742
P03410	Q96BU1	0.85654
P03410	P78317	0.28112
P03410	Q9BSF8	0.49184
P03410	Q9UMS0	1.2234
P03410	Q9BRJ2	0.78159
P03410	A1A4V9	0.5745
P03410	Q9H6R4	0.72933
P03410	Q8WY36	0.41575
P03410	Q674X7	0.60865
P03410	Q9UPU9	0.62609
P03410	P05937	0.28026
P03410	Q96BR5	0.15917
P03410	Q9UHF0	0.88122
P03410	Q6IPW1	0.90492
P03410	Q5JX71	1.1653
P03410	Q5HYK9	0.77289
P03410	Q8WWM1	1.2709
P03410	Q9BWU0	0.73767
P03410	Q8N8M0	0.70423
P03410	O94829	1.3202

P03410	Q9HA92	0.59814
P03410	O75531	1.1009
P03410	Q8N8R7	0.98145
P03410	O43734	1.4883
P03410	Q8IVM7	1.6653
P03410	P43363	0.044177
P03410	Q16873	0.38059
P03410	Q6UW32	0.70628
P03410	P02511	0.36911
P03410	P55198	0.97091
P03410	Q66K66	0.95154
P03410	O15382	0.20626
P03410	O43310	0.68955
P03410	O75956	0.62218
P03410	Q5VZL5	1.3743
P03410	Q8IXS8	1.4066
P03410	Q58FF3	0.30901
P03410	O95198	0.88966
P03410	Q15024	0.63293
P03410	Q96NS1	0.36709
P03410	A8MZ97	1.2394
P03410	O15379	0.56278
P03410	B2RV13	1.0975
P03410	P56524	0.32251
P03410	P22830	0.30782
P03410	Q9NZ09	0.25201
P03410	Q9BXW4	0.55195
P03410	Q6EEV6	0.21002
P03410	Q9Y6N9	0.53336
P03410	Q9BYG3	0.31783
P03410	Q9BTK2	0.98322
P03410	Q9UL12	0.24891
P03410	Q9HBH7	1.1086
P03410	Q6AI08	0.85748
P03410	Q9Y520	0.74604
P03410	Q5VU43	1.3212
P03410	Q13111	1.901
P03410	Q99704	0.23697
P03410	P61758	0.77029
P03410	P53701	1.1346
P03410	P17014	0.75447
P03410	Q6TAR4	0.46638
P03410	P23434	0.6035
P03410	Q96CD2	0.19879
P03410	Q6URK8	1.0586
P03410	P48728	0.60504
P03410	Q12905	0.91841
P03410	Q16613	0.51207
P03410	Q8IXJ9	0.73348
P03410	Q2TB18	0.046596
P03410	C9JN71	0.80184
P03410	Q86VY4	0.62264
P03410	Q9NQX7	0.5088
P03410	Q9UBB6	0.82193
P03410	Q96CV9	0.79361
P03410	O14836	0.79879
P03410	Q641Q2	1.2477
P03410	Q9HCE1	0.70553
P03410	P0CG24	0.71093
P03410	Q92837	1.3123
P03410	P46939	0.8574
P03410	Q92796	0.48624
P03410	Q9NZ63	0.88139
P03410	P33240	0.58195
P03410	P56555	1.3773
P03410	Q8IZU3	0.34657
P03410	Q8IWL3	0.59994
P03410	Q9UND3	0.61572
P03410	Q9NY74	1.8629
P03410	Q9NWT8	1.0163
P03410	P49366	0.55426
P03410	Q16082	1.0294
P03410	P30566	0.076591
P03410	P02795	0.9907
P03410	Q96A26	0.3817
P03410	Q9Y3A3	0.83026

P03410	Q9NVX0	1.2129
P03410	Q68EA5	0.96545
P03410	Q96SE7	0.78793
P03410	Q5JVF3	0.41762
P03410	Q9UIG4	0.95938
P03410	Q9H706	0.6999
P03410	Q8N157	0.41354
P03410	Q5JVS0	0.81301
P03410	Q99550	0.74388
P03410	Q8N1G2	0.37881
P03410	Q15629	0.68561
P03410	Q9NV88	0.85092
P03410	Q9UBU7	0.34898
P03410	Q9NZM4	1.7978
P03410	A1A4F0	0.14807
P03410	O75818	0.43383
P03410	Q7Z4G1	2.0374
P03410	Q9BPX7	0.88973
P03410	Q8N535	0.96727
P03410	Q5VT98	0.21632
P03410	Q9UNS2	0.85902
P03410	Q8IWE2	1.4763
P03410	Q5VUM1	0.57969
P03410	Q96NY9	0.85464
P03410	Q9H5I1	0.56229
P03410	Q12988	0.49339
P03410	Q9UGC7	1.2628
P03410	Q9BYQ3	1.9984
P03410	Q9UPP2	0.33808
P03410	Q6NUP7	0.73212
P03410	Q9UHL3	0.5957
P03410	Q5VUE5	1.0682
P03410	Q9NTI5	0.95877
P03410	P53803	1.2345
P03410	P02545	0.985
P03410	Q13569	1.0947
P03410	Q14532	0.70244
P03410	Q8NA42	0.83606
P03410	Q08722	0.46219
P03410	Q9H321	1.4395
P03410	Q9Y3U8	1.0346
P03410	P43487	0.53806
P03410	Q9P0N5	0.95894
P03410	P28062	0.55374
P03410	Q13033	0.93646
P03410	Q86UT8	1.7604
P03410	Q9Y3V2	1.0663
P03410	Q8NE18	1.7149
P03410	P0DL12	0.93991
P03410	A6NMA1	0.78421
P03410	Q93083	0.83308
P03410	P79522	0.81366
P03410	Q14CS0	0.53674
P03410	P16083	0.29361
P03410	Q9P2X3	0.3681
P03410	Q9H3C7	1.4603
P03410	Q9H6R7	0.34061
P03410	Q9NWM3	0.48529
P03410	Q9UHX1	0.65752
P03410	Q6P280	1.0521
P03410	Q15797	0.29727
P03410	Q9BXQ6	1.6653
P03410	P62952	1.2353
P03410	Q52LG2	1.1302
P03410	O43615	0.42065
P03410	Q3LI73	1.9939
P03410	Q96KR6	1.1338
P03410	Q71F23	0.63523
P03410	Q9ULP0	0.36161
P03410	Q92922	1.0049
P03410	Q6P1X5	0.99431
P03410	Q96AZ6	0.26481
P03410	P09497	0.74339
P03410	Q6PJW8	0.18641
P03410	Q96KP6	0.16235
P03410	Q14789	0.69104

P03410	P45877	0.22741
P03410	Q9H5V9	1.2616
P03410	C9J3V5	1.0143
P03410	Q9BRQ4	0.99563
P03410	Q6ZUB0	1.0913
P03410	Q2NKX9	1.4883
P03410	P17024	0.81277
P03410	Q02383	0.77602
P03410	Q9BYC2	0.10122
P03410	Q96HJ9	0.41785
P03410	O95486	0.64668
P03410	Q6P4R8	1.075
P03410	Q5VV41	0.36046
P03410	Q6UX65	0.75535
P03410	Q8N5C8	0.82932
P03410	Q01826	0.80263
P03410	Q9C075	0.79493
P03410	P42704	1.148
P03410	Q14140	2.0362
P03410	Q6ZNR8	1.6653
P03410	Q9H609	0.10555
P03410	Q9BXT5	0.99872
P03410	Q9ULE0	0.24399
P03410	Q13112	1.3637
P03410	Q8TEM1	0.54239
P03410	P31040	0.65666
P03410	Q13084	1.4681
P03410	Q96BJ8	0.080131
P03410	Q9Y6X0	0.10622
P03410	Q9UKC9	0.69598
P03410	O15127	1.0685
P03410	Q969G9	0.29107
P03410	Q8N7K0	0.79465
P03410	F5GYI3	1.802
P03410	Q86U70	0.35625
P03410	Q5T5D7	0.64731
P03410	Q9UF72	1.3231
P03410	Q96MT4	1.6063
P03410	Q5HYI7	1.6237
P03410	Q5VT28	1.4596
P03410	Q9H160	0.3602
P03410	Q8IUI8	0.26067
P03410	Q9NP71	0.32674
P03410	O43395	0.80832
P03410	Q15049	0.14453
P03410	Q14847	0.30771
P03410	A6NN06	1.6653
P03410	Q8NA56	0.014907
P03410	Q14119	0.24382
P03410	Q8WYA0	1.0417
P03410	P26678	1.2728
P03410	P49788	0.8634
P03410	P43268	0.58516
P03410	O75909	1.2189
P03410	A6NEF3	1.594
P03410	Q8N1D5	0.54506
P03410	Q5U3C3	1.0913
P03410	Q15428	1.0372
P03410	Q2YD98	1.0515
P03410	Q8IX06	0.085922
P03410	Q9NUC0	0.61757
P03410	Q6ZS27	0.75478
P03410	A6NK89	1.266
P03410	P0CG33	0.4493
P03410	O75145	0.71254
P03410	Q9HBL0	0.8399
P03410	Q15326	1.0583
P03410	Q14602	0.32902
P03410	P31269	0.48712
P03410	Q96S65	1.6793
P03410	O15534	0.27754
P03410	P03928	0.45607
P03410	Q9NZG7	1.0124
P03410	Q8WV92	0.76187
P03410	Q8WXC6	0.81324
P03410	P16455	0.5868

P03410	Q08495	0.3778
P03410	Q96JG6	0.28725
P03410	Q8NHW6	1.1508
P03410	P51665	0.66381
P03410	O75204	1.1092
P03410	O95983	1.3189
P03410	Q9C0I3	1.5263
P03410	Q8N801	0.15808
P03410	Q5TFE4	0.29108
P03410	Q3BBV0	0.62379
P03410	E9PAV3	0.71712
P03410	Q6P656	0.22771
P03410	P62877	0.44499
P03410	P19447	0.14712
P03410	Q8N9U0	0.20917
P03410	Q8IW19	0.34925
P03410	Q9BUH6	1.1125
P03410	Q9Y4X5	0.19561
P03410	Q12796	2.3311
P03410	Q7Z7C7	0.94854
P03410	Q3I5F7	0.20625
P03410	P63146	0.47809
P03410	Q9P215	0.58357
P03410	Q9Y613	0.8835
P03410	A6QL64	0.77288
P03410	Q15363	0.30088
P03410	Q9H0C5	0.7983
P03410	P11387	0.87694
P03410	A0AUZ9	0.72494
P03410	O60927	1.302
P03410	P17020	0.65335
P03410	Q9BXF6	0.91991
P03410	Q14683	0.53721
P03410	Q9Y2Y6	1.1019
P03410	Q99909	0.47959
P03410	P05408	1.0959
P03410	Q5T2E6	0.97411
P03410	P41250	0.63401
P03410	O14907	0.63397
P03410	O60232	1.539
P03410	Q9H246	0.75802
P03410	P42167	0.614
P03410	Q01658	0.5085
P03410	Q14197	0.25415
P03410	P09661	0.74611
P03410	Q9Y284	1.3759
P03410	P47928	0.57099
P03410	P48380	0.49177
P03410	Q6P1L8	1.8139
P03410	P22415	0.37035
P03410	Q9BV44	0.46335
P03410	Q8WXF1	0.93488
P03410	Q9ULX6	0.78496
P03410	Q5T7V8	2.3991
P03410	Q8N0X4	0.34575
P03410	Q9Y291	0.90306
P03410	Q8N4K4	0.65043
P03410	Q1RN00	1.6653
P03410	O43379	0.39139
P03410	Q13485	0.84756
P03410	P0CW19	0.43173
P03410	P12883	0.19684
P03410	Q9UI12	0.27644
P03410	Q6ZRK6	0.026761
P03410	P13807	0.37846
P03410	O15067	0.85399
P03410	Q13156	0.65499
P03410	Q3BBW0	0.49912
P03410	Q9NZT2	0.23249
P03410	Q6XXX2	0.41026
P03410	Q8NC74	0.61032
P03410	Q8NEG4	0.84146
P03410	Q96I24	0.4417
P03410	Q9H2U6	0.92468
P03410	O43765	0.92437
P03410	Q3YBR2	0.67546

P03410	Q15036	0.37022
P03410	Q8TAP8	1.4123
P03410	O15078	0.84489
P03410	Q9HCN2	1.6653
P03410	Q8IYS8	0.98327
P03410	Q9NUW8	0.7616
P03410	Q9Y3D7	0.37372
P03410	Q96LB4	0.43472
P03410	P10914	0.36489
P03410	Q96GS4	1.9727
P03410	A6NEQ9	1.6653
P03410	Q96DE0	1.708
P03410	Q9NY12	0.88095
P03410	Q9BUY5	0.95151
P03410	Q8IW70	1.0913
P03410	Q9H8K7	0.84571
P03410	Q96IP4	0.95458
P03410	Q6ZW35	0.22374
P03410	Q9P2G1	0.50973
P03410	P49419	0.52921
P03410	Q06250	1.6653
P03410	Q6UWN0	0.58341
P03410	Q96A44	0.63637
P03410	Q5H9R4	1.4129
P03410	Q9Y6I8	0.60238
P03410	Q8N2G8	0.22809
P03410	O75506	0.18913
P03410	Q96IR2	0.81411
P03410	Q5T013	0.24826
P03410	Q9ULX9	0.43171
P03410	Q6L8H4	1.6653
P03410	O94864	0.63626
P03410	Q9Y3B9	0.76419
P03410	Q9H098	1.5179
P03410	O60356	1.3816
P03410	Q99583	0.36663
P03410	P61580	1.7423
P03410	Q15185	0.51194
P03410	Q8N8D0	1.6653
P03410	Q15459	0.62584
P03410	Q66K80	1.6653
P03410	Q96B54	1.57
P03410	Q96G01	0.41375
P03410	Q8NEY3	1.1155
P03410	A7E2F4	0.066261
P03410	Q68CQ4	0.27334
P03410	A6NF83	1.071
P03410	Q96HJ3	1.4883
P03410	Q13952	0.68308
P03410	P22736	0.3074
P03410	Q6IN85	0.65434
P03410	P55809	0.025504
P03410	Q5SWW7	1.6653
P03410	Q8N7W2	0.81853
P03410	Q96IZ0	0.89853
P03410	Q7L2E3	0.46787
P03410	Q04727	0.60516
P03410	Q8IWL8	1.9897
P03410	Q8WXC7	0.86902
P03410	Q8IZ26	0.81355
P03410	Q9H299	0.36617
P03410	Q14186	1.1197
P03410	Q9H7L9	0.75522
P03410	Q9BYE3	1.6653
P03410	Q9BUP3	0.29019
P03410	O75489	0.62567
P03410	A6NDK9	0.3132
P03410	P24539	0.40159
P03410	P42898	0.41552
P03410	Q8NEV9	0.98858
P03410	Q12912	1.3126
P03410	Q8N0Y7	0.92904
P03410	Q8IXT1	0.76442
P03410	Q5SWA1	1.3211
P03410	Q9Y371	0.28941
P03410	Q8N3I7	0.93995

P03410	Q96EK7	0.33708
P03410	Q9P2K1	0.63931
P03410	O75533	0.40854
P03410	Q676U5	1.0091
P03410	H0Y354	1.0087
P03410	O95989	0.87375
P03410	Q14106	0.60126
P03410	P13693	0.1775
P03410	Q969J2	0.68469
P03410	Q96DE5	1.9365
P03410	Q9Y2S0	0.27259
P03410	Q8NDT2	0.91688
P03410	Q9H444	1.0984
P03410	Q9HCK5	0.99596
P03410	Q86V81	0.83348
P03410	P07311	1.1782
P03410	Q9BZD4	0.85714
P03410	Q9H9A7	0.81112
P03410	Q8TAF3	0.91518
P03410	Q8TBE0	0.7953
P03410	Q9UKJ5	0.42668
P03410	P81172	0.96348
P03410	Q9NWF4	1.0772
P03410	O60784	0.71062
P03410	O14974	0.54614
P03410	Q9H8Q6	1.7181
P03410	Q8N9R8	1.1868
P03410	Q96MX3	0.72744
P03410	Q9P1F3	1.1904
P03410	P62875	0.75835
P03410	Q9H7R5	0.81411
P03410	O14776	1.2238
P03410	P05423	0.53013
P03410	Q2VPK5	1.0539
P03410	O60447	0.29475
P03410	Q9GZS1	0.47239
P03410	Q8WVK7	0.83375
P03410	Q15054	0.92921
P03410	Q8IYN2	1.5618
P03410	Q9BY31	0.62791
P03410	Q9UPM9	0.18547
P03410	Q9UN42	0.2401
P03410	Q9HCK8	0.28739
P03410	Q8TC84	0.49184
P03410	Q15849	0.68559
P03410	Q9BVC3	0.85786
P03410	Q6XZF7	0.49504
P03410	Q9NZZ3	1.2571
P03410	Q9Y2X8	0.53543
P03410	Q8NE65	0.86984
P03410	Q96N28	0.23773
P03410	O95415	1.1653
P03410	Q16378	1.6653
P03410	Q5T1B0	0.099693
P03410	P31483	0.43272
P03410	Q69YH5	1.4184
P03410	P78386	0.91465
P03410	Q76L83	0.69739
P03410	Q9ULX3	1.0715
P03410	Q3C1V9	0.56707
P03410	Q9P266	1.4179
P03410	Q86VP6	0.64977
P03410	P28702	0.36929
P03410	Q92834	0.69212
P03410	Q12789	0.95908
P03410	Q9H201	0.36287
P03410	Q56UQ5	0.028138
P03410	Q9Y6D6	0.80124
P03410	Q9BRB3	0.83565
P03410	O94830	0.08532
P03410	Q9Y4E1	0.99187
P03410	Q96MY1	1.285
P03410	Q13064	0.31691
P03410	P19338	0.68443
P03410	Q9Y5V0	1.414
P03410	P55327	0.56719

P03410	Q13023	0.81244
P03410	O43924	0.44939
P03410	Q9H089	0.34479
P03410	Q9Y6H1	1.6853
P03410	Q9P1U0	0.18726
P03410	Q9NRF9	0.20972
P03410	Q147U1	0.83595
P03410	Q9H1K6	0.19778
P03410	Q9H1K0	0.16404
P03410	Q96M83	0.45197
P03410	P52272	0.23493
P03410	Q9GZP4	0.099149
P03410	Q6K0P9	0.45017
P03410	Q8IYJ3	0.49477
P03410	Q9UM63	0.43972
P03410	Q8N1G1	0.31201
P03410	P04156	0.81343
P03410	Q8N9S9	0.48798
P03410	P43357	0.6999
P03410	P08708	0.63171
P03410	P09874	0.56356
P03410	P0C7W9	0.18282
P03410	Q9NTK1	1.4336
P03410	Q53EL6	1.1801
P03410	Q5HY64	0.19655
P03410	Q86Y39	1.2475
P03410	Q9BRR8	1.3289
P03410	Q16526	0.37294
P03410	Q8NC38	0.38258
P03410	Q9BTC0	0.28588
P03410	P15259	0.92904
P03410	P86434	0.40084
P03410	O95876	0.52498
P03410	Q12933	0.52114
P03410	Q9BSA9	1.0913
P03410	Q8TC71	0.6449
P03410	Q8WZB0	0.07912
P03410	Q9H0E2	0.33788
P03410	Q9Y2R9	1.8713
P03410	Q9BVV2	0.17293
P03410	Q8N5P1	0.28605
P03410	A6NKN8	1.6655
P03410	P36952	0.19809
P03410	Q9H788	1.1308
P03410	Q8WYJ6	0.41113
P03410	P46782	0.9404
P03410	Q9ULU8	0.32395
P03410	Q5PRF9	0.65956
P03410	Q13066	0.508
P03410	P16220	0.9305
P03410	Q96EN9	1.2795
P03410	Q15466	0.48947
P03410	Q9NVU7	1.0178
P03410	P32969	1.1682
P03410	Q96SK3	0.78354
P03410	Q15834	1.4139
P03410	P56693	0.49868
P03410	Q00403	1.0032
P03410	Q658K8	1.0771
P03410	P0CAP2	0.61884
P03410	P21506	0.85712
P03410	Q9NUQ3	0.62859
P03410	Q5TBE3	0.77429
P03410	Q96C23	0.057075
P03410	P02728	1.6653
P03410	O95831	0.71264
P03410	Q9BVA1	0.12768
P03410	Q96ST8	1.5205
P03410	Q7RTV3	0.21317
P03410	Q9BXL5	0.45765
P03410	Q9NV64	1.0758
P03410	O14818	0.95721
P03410	Q86Y37	0.23047
P03410	Q4W4Y0	0.21555
P03410	Q9BYQ4	1.9984
P03410	P12757	0.43608

P03410	O96017	0.8354
P03410	O43296	0.7974
P03410	Q9H1B7	1.2718
P03410	A8MXV4	1.2137
P03410	Q5EBM0	1.2577
P03410	H3BTG2	1.2588
P03410	P53618	1.0852
P03410	Q2M2I5	1.0001
P03410	Q9UBU9	0.61968
P03410	Q6P1L6	0.7485
P03410	Q96PI1	1.5415
P03410	Q9H777	0.70307
P03410	Q3SY05	0.25672
P03410	P84101	1.8026
P03410	Q9NT62	0.40453
P03410	A6NFU8	0.87472
P03410	P18583	0.43141
P03410	O75436	1.0027
P03410	P36873	0.68122
P03410	P86452	0.5839
P03410	Q8N4C6	0.99776
P03410	Q9HBM1	0.86866
P03410	Q5XG87	0.15306
P03410	Q9UKD2	0.32328
P03410	Q9BQ61	1.4883
P03410	Q5RGS2	1.4013
P03410	Q86SQ7	1.3609
P03410	Q96S94	0.42123
P03410	O95744	0.12378
P03410	A8MVW5	1.1293
P03410	P61923	0.66521
P03410	Q9BTT4	0.74761
P03410	Q9Y6D5	0.34831
P03410	Q9Y6X8	0.56498
P03410	Q8WWX9	0.62692
P03410	Q9BZE2	1.5823
P03410	Q6ZUI0	0.5323
P03410	Q5TGY1	0.63468
P03410	P17032	0.79703
P03410	A6NGS2	0.95877
P03410	P53041	0.56422
P03410	Q7Z7J9	0.75253
P03410	Q5TYM5	0.75636
P03410	Q8WWB7	1.0145
P03410	O15014	1.5081
P03410	Q8N6K0	1.0913
P03410	Q6PGQ7	2.0038
P03410	Q5XUX1	0.6999
P03410	Q8IYW4	0.30289
P03410	Q2VYF4	0.70563
P03410	Q9H0R6	0.89482
P03410	Q96EE3	0.70702
P03410	O14757	0.84086
P03410	Q15046	1.3441
P03410	P30154	0.67137
P03410	O60671	0.96489
P03410	Q6PII3	1.0776
P03410	Q6IQ49	0.52374
P03410	Q92734	0.41442
P03410	P17027	0.66065
P03410	Q9H5Q4	0.95609
P03410	P47974	0.2178
P03410	O75170	1.2108
P03410	Q9H7E2	0.65574
P03410	Q8TAY7	2.3386
P03410	Q5VXH4	0.67757
P03410	Q9H173	0.24644
P03410	Q9BR76	0.70629
P03410	Q9NSI2	1.4883
P03410	Q9NQV6	0.10142
P03410	Q9Y6G3	0.61591
P03410	P51668	0.48162
P03410	Q9HAT8	0.91747
P03410	Q96JE7	1.3841
P03410	Q6NVV0	1.2236
P03410	P0CG20	0.72807

P03410	Q8NI29	0.55816
P03410	Q52LD8	0.8062
P03410	O14602	0.76444
P03410	Q3SY52	0.744
P03410	P0CJ72	1.4876
P03410	Q14585	0.68833
P03410	Q9BRG1	1.2646
P03410	Q86XI8	0.90265
P03410	A8MUV8	1.0058
P03410	Q9P0W5	1.4674
P03410	P15515	1.0127
P03410	A6NHR8	1.6653
P03410	Q6UWH6	0.62829
P03410	Q8NFJ8	0.5354
P03410	Q9UL15	0.20044
P03410	Q6UWF3	1.4002
P03410	Q9UJ98	0.83466
P03410	Q53H80	0.6116
P03410	Q5VWI1	1.3352
P03410	P46937	0.78233
P03410	P78412	0.2823
P03410	Q9NZD2	0.8043
P03410	Q8N196	0.52638
P03410	P20290	1.2273
P03410	Q13542	0.79039
P03410	Q06455	0.55148
P03410	Q68D20	0.27658
P03410	Q6P3S1	0.096873
P03410	Q6P1J9	0.68508
P03410	Q9H4Z3	0.22904
P03410	O43364	0.15271
P03410	Q9Y5P2	0.23754
P03410	P31939	0.76851
P03410	Q7Z5M5	0.75414
P03410	Q86XG9	0.3881
P03410	Q9BQ48	1.6536
P03410	O60443	0.49319
P03410	Q8WU39	0.83862
P03410	Q6P2C8	0.92438
P03410	Q9NQ50	1.7302
P03410	Q8IXM3	1.0766
P03410	Q14118	0.21254
P03410	Q9GZX5	0.80611
P03410	Q13118	0.37372
P03410	Q96FQ7	0.34557
P03410	P68133	0.24431
P03410	Q6ZS17	0.70981
P03410	Q15699	0.3321
P03410	O75410	0.98238
P03410	Q8N2G4	0.45712
P03410	Q86WX3	1.3403
P03410	Q9NTI7	1.429
P03410	P47224	0.59781
P03410	Q6P1X6	1.5089
P03410	P81274	0.67331
P03410	P49910	0.70955
P03410	A8MVA2	1.9984
P03410	P46100	0.59793
P03410	Q86XE5	1.356
P03410	Q8IUW3	1.2333
P03410	Q9UJX5	1.0131
P03410	Q6NSI3	1.6547
P03410	Q8N9V7	1.124
P03410	Q8WTW3	1.3468
P03410	Q49AN0	0.12488
P03410	P60842	0.38568
P03410	Q86Y56	0.057612
P03410	A2AJT9	0.70042
P03410	P21912	0.20356
P03410	P35269	1.058
P03410	Q6UWQ7	0.92929
P03410	Q92618	0.46225
P03410	Q8N9H6	1.0932
P03410	Q14320	0.29128
P03410	Q9NRN7	0.80081
P03410	Q9Y4G6	0.45333

P03410	Q9Y6J9	0.81871
P03410	Q8WWW0	0.67069
P03410	P61962	0.61061
P03410	P55771	0.82039
P03410	Q5T6F0	0.33445
P03410	P42568	0.992
P03410	O43324	1.2634
P03410	P0DJH9	0.28632
P03410	Q86XN6	0.80654
P03410	Q13185	0.84092
P03410	Q8N5D0	0.70101
P03410	Q6P050	1.0828
P03410	Q5VV52	0.69851
P03410	O14832	0.59697
P03410	Q96GJ1	0.51466
P03410	Q5BJF6	0.7694
P03410	Q8NEM7	0.42776
P03410	Q9Y244	0.5856
P03410	Q96RM1	1.6653
P03410	Q9HB96	1.6653
P03410	Q5T5A4	0.95652
P03410	P21673	0.40727
P03410	A6NDN3	0.39823
P03410	P51786	0.81473
P03410	Q9GZX7	0.31381
P03410	Q9NY87	1.6618
P03410	O75935	1.0776
P03410	O75175	1.2905
P03410	Q8N612	1.3242
P03410	Q9H1B4	0.34116
P03410	O75807	0.9908
P03410	Q96A98	0.85606
P03410	Q13469	0.96378
P03410	B1APH4	0.83595
P03410	Q6UWZ7	0.62046
P03410	Q9BUN5	0.80381
P03410	A0AUK8	0.48307
P03410	Q9UPY8	1.2482
P03410	Q5BJE1	0.64176
P03410	Q15910	0.42818
P03410	Q9BYT9	0.92935
P03410	Q14093	1.2218
P03410	Q9NZ20	0.59838
P03410	P0C5W0	0.38853
P03410	P82673	0.44539
P03410	Q9NRS6	0.21436
P03410	P41182	0.1763
P03410	Q6UW10	1.0723
P03410	Q8N3U4	0.9221
P03410	O14990	1.0127
P03410	Q13769	0.65626
P03410	O60921	0.97019
P03410	P43365	0.15517
P03410	P0CJ92	0.43504
P03410	Q9Y375	0.86783
P03410	Q99504	0.39031
P03410	O95670	0.26922
P03410	Q9Y6C7	1.6653
P03410	Q9UHD8	0.25227
P03410	P61572	1.1462
P03410	Q14011	0.3292
P03410	Q6ZPA2	1.6653
P03410	Q5W111	0.28696
P03410	Q8IYB3	1.122
P03410	P51532	0.38165
P03410	O00303	0.30315
P03410	Q9NZU7	0.57914
P03410	P0CG00	0.54521
P03410	Q8IVE0	0.39445
P03410	Q53EZ4	1.0076
P03410	Q5T653	1.5829
P03410	O15015	0.68989
P03410	Q9HBT7	0.85194
P03410	Q6ZU80	0.42068
P03410	P38919	0.25107
P03410	P0CH98	0.35337

P03410	Q68D51	0.051672
P03410	Q96BW5	0.71925
P03410	P59089	0.23979
P03410	Q09MP3	1.2868
P03410	O60256	0.31439
P03410	P49754	0.62461
P03410	Q16594	0.62066
P03410	Q9BQG0	0.46972
P03410	Q9NPI7	0.38942
P03410	Q6VEQ5	0.81442
P03410	B4DJY2	0.6834
P03410	Q96FZ7	0.30873
P03410	Q5JQF7	1.6653
P03410	Q15853	0.52023
P03410	Q9NQ92	0.97602
P03410	Q9NQT4	0.38501
P03410	Q7Z2Z1	1.5711
P03410	Q7RTS7	0.71041
P03410	Q9ULQ0	1.6685
P03410	P0DKX0	0.93715
P03410	Q6ICG7	1.6653
P03410	P87889	0.085143
P03410	Q86WH2	0.54998
P03410	Q6ZSR6	0.55512
P03410	Q8NCY6	0.20917
P03410	P61218	0.45568
P03410	Q5VWT5	1.1236
P03410	Q13398	0.96662
P03410	P82912	1.5291
P03410	P04259	0.7024
P03410	Q5JVG8	0.97401
P03410	Q6PUV4	0.75127
P03410	Q9BSF0	0.72649
P03410	Q16531	0.70286
P03410	Q9BT22	0.98402
P03410	P86480	1.6653
P03410	Q2V2M9	0.49388
P03410	Q9NYF0	0.49165
P03410	Q96LI9	0.85967
P03410	Q5VSR9	1.5851
P03410	Q9NRW3	0.34944
P03410	O75346	0.94731
P03410	Q8NCA5	1.0321
P03410	O95810	0.51495
P03410	O95785	0.30744
P03410	P48788	0.72969
P03410	Q9H693	0.92211
P03410	Q5T036	0.93671
P03410	P13349	0.29624
P03410	Q9H008	0.70645
P03410	P48230	0.67855
P03410	P61566	0.77894
P03410	Q5JTD0	1.8557
P03410	Q8IZP0	0.40699
P03410	Q8N945	0.21192
P03410	O00534	0.607
P03410	Q6ZRF7	0.56084
P03410	Q9H0R1	0.48628
P03410	Q96N06	0.97209
P03410	Q6ZNM6	1.2549
P03410	Q9NR33	0.22636
P03410	Q96GX9	0.43819
P03410	Q13610	0.26546
P03410	Q5BKZ1	0.96876
P03410	Q96S06	1.1144
P03410	Q66K74	0.2771
P03410	Q8N5I3	0.252
P03410	Q9UJZ1	0.20913
P03410	O60296	0.67889
P03410	Q13426	0.42743
P03410	Q4LE28	1.0271
P03410	Q3LI76	1.7519
P03410	Q5TGY3	0.37597
P03410	Q6NSW5	1.4093
P03410	Q13642	0.25216
P03410	Q6ICB4	0.42412

P03410	A2A2V5	1.0913
P03410	Q8TBR4	0.83887
P03410	Q96KT6	1.6653
P03410	P0C7Q6	1.1631
P03410	P48059	0.44608
P03410	O60504	0.87275
P03410	Q2KHR3	0.18992
P03410	Q9BQ89	1.9749
P03410	Q9Y281	0.84246
P03410	Q9BSJ2	1.4623
P03410	P05412	0.73184
P03410	O00522	0.49407
P03410	D6RB28	1.5003
P03410	Q96HA9	1.0798
P03410	Q96FB5	1.0111
P03410	Q8IYB7	0.28448
P03410	Q96IM9	0.22979
P03410	Q5T6L9	1.1653
P03410	Q8IUR6	1.0398
P03410	Q9Y5U8	0.93283
P03410	Q86X95	1.6166
P03410	A8MSI8	1.8647
P03410	Q8N3T6	0.74592
P03410	Q92616	0.39208
P03410	O43541	0.63416
P03410	O75964	0.42204
P03410	Q8IVV7	0.51659
P03410	Q96MR9	0.92459
P03410	Q8N488	0.50034
P03410	P04406	0.30127
P03410	Q99598	0.3626
P03410	Q9BZ23	0.96617
P03410	P49336	0.27042
P03410	Q6ZT83	0.77419
P03410	P50453	0.62885
P03410	Q86XZ4	0.87011
P03410	P39880	0.43972
P03410	Q6PK04	1.334
P03410	P49639	0.22873
P03410	Q8WWL2	0.29642
P03410	Q96KH6	1.3023
P03410	O94927	0.81017
P03410	Q7L590	1.197
P03410	Q6RW13	1.3101
P03410	Q9BQI5	0.68036
P03410	Q3T8J9	0.82581
P03410	Q9NQ33	0.5093
P03410	Q04695	0.95379
P03410	Q495Y8	1.0907
P03410	Q5XKE5	0.75104
P03410	P20248	1.2412
P03410	Q9H7Z3	0.42378
P03410	P04844	0.80183
P03410	Q96GU1	0.69691
P03410	P99999	0.68139
P03410	Q0VD86	0.49184
P03410	Q6ZU52	0.6999
P03410	P86790	0.60221
P03410	P50402	0.70722
P03410	Q8IWN6	1.3633
P03410	Q00765	0.16406
P03410	O15360	1.0095
P03410	Q7Z2V1	1.6653
P03410	P20962	1.5216
P03410	A2RRD8	0.84515
P03410	Q9UNW9	0.15008
P03410	Q5HYN5	0.41446
P03410	Q9BRQ3	1.4619
P03410	Q9Y2Y1	0.15074
P03410	Q9UK61	0.91515
P03410	O95400	1.0687
P03410	Q9NX63	1.0754
P03410	Q5T686	0.87539
P03410	Q8N8B7	0.13575
P03410	Q8WWH5	1.2635
P03410	O76013	0.89153

P03410	Q10586	0.39516
P03410	Q12888	0.69329
P03410	Q9Y334	0.76554
P03410	Q6XYB7	0.30793
P03410	Q9Y3Y2	1.7714
P03410	Q96F45	0.20557
P03410	O60294	0.59649
P03410	P31948	1.1672
P03410	P53677	0.68002
P03410	O43196	0.87569
P03410	Q96HR3	0.8202
P03410	Q14409	0.87046
P03410	Q8IVF2	0.20917
P03410	Q9NW07	0.84019
P03410	Q9BTT0	0.31129
P03410	Q15438	0.35224
P03410	P49711	0.25454
P03410	Q69YW2	0.82637
P03410	P23526	0.15451
P03410	Q9UHB4	0.67729
P03410	A6NDR6	0.2823
P03410	Q5TBC7	0.38997
P03410	Q7Z4H9	0.86705
P03410	Q5JTC6	1.4625
P03410	Q9Y6R6	0.79465
P03410	Q9BZW7	0.33846
P03410	Q75MW2	1.0189
P03410	Q96C90	1.0173
P03410	Q6UX04	0.19834
P03410	B2RUZ4	1.7276
P03410	A8MT70	0.88504
P03410	P18669	1.0057
P03410	Q15051	0.98848
P03410	Q96CJ1	1.2681
P03410	P02042	0.81619
P03410	Q5SRD0	0.59409
P03410	O00231	0.76493
P03410	Q8IYX7	1.314
P03410	Q9UKY7	1.6342
P03410	O15488	0.14631
P03410	Q8N6Q8	0.77934
P03410	Q9NQ29	1.0774
P03410	Q86W56	0.73896
P03410	Q6GV28	1.0448
P03410	Q6ZTB9	0.82426
P03410	Q96G03	1.0184
P03410	Q9UGL9	1.6653
P03410	Q9Y222	0.81774
P03410	O43768	0.36323
P03410	Q8NFU3	0.35638
P03410	P08670	0.48204
P03410	Q9H6B1	0.77834
P03410	P05813	0.64324
P03410	Q8TBN0	0.59709
P03410	Q8N988	0.95629
P03410	Q86SG2	0.3327
P03410	Q9Y4K1	1.2726
P03410	Q86WJ1	0.29577
P03410	Q14678	0.57173
P03410	Q9NUL3	0.87648
P03410	Q6FI81	0.56799
P03410	Q7Z4W3	1.6653
P03410	Q8ND04	0.72542
P03410	P09234	0.69272
P03410	Q99576	0.49906
P03410	O43151	0.90025
P03410	Q8N1Q8	1.0135
P03410	Q96MR7	1.7296
P03410	Q9UNM6	0.5761
P03410	Q8NA92	0.9828
P03410	O75558	1.0616
P03410	Q16625	0.31301
P03410	B9A014	0.36028
P03410	P98168	0.63733
P03410	P53611	0.10315
P03410	Q9H3J6	1.5945

P03410	A6NI79	1.594
P03410	Q5MCW4	0.66013
P03410	P54257	0.1777
P03410	Q9Y263	0.36046
P03410	P30281	1.0284
P03410	Q9UKL3	1.5802
P03410	Q9UK73	0.75718
P03410	Q0D2H9	0.71749
P03410	Q12834	1.0367
P03410	Q86V71	0.81404
P03410	Q9NQZ5	0.38118
P03410	Q6R327	0.27258
P03410	Q6PI97	0.39468
P03410	Q8NBT2	0.89001
P03410	O95447	0.60497
P03410	P09669	0.68988
P03410	O95232	0.60708
P03410	Q92547	0.80047
P03410	Q569G3	1.4883
P03410	Q8ND99	0.90013
P03410	A6NIJ5	0.18282
P03410	Q96T51	0.38267
P03410	Q96ME1	0.33295
P03410	Q6TGC4	0.6804
P03410	Q5JPH6	0.94019
P03410	A6NFL8	1.594
P03410	P80217	0.87267
P03410	O15105	0.32518
P03410	P78559	1.0935
P03410	Q9HA90	1.4883
P03410	Q8WW18	1.4883
P03410	Q6URH7	0.051752
P03410	P54368	0.2531
P03410	O95625	0.59267
P03410	Q8NEM2	0.66984
P03410	O60828	0.74173
P03410	Q96CN7	0.45946
P03410	Q00G26	0.0095964
P03410	Q8WW12	1.5871
P03410	P56182	1.3107
P03410	O95476	0.40084
P03410	P09565	1.352
P03410	Q86SH2	0.33627
P03410	Q16836	0.31168
P03410	O43422	0.70112
P03410	Q5VW32	0.17601
P03410	P30260	1.2912
P03410	P42226	0.67385
P03410	P46779	1.3686
P03410	Q9NVV2	1.6653
P03410	Q504U0	0.24044
P03410	P51114	0.20893
P03410	F7VJQ1	1.6653
P03410	A6NHU5	1.1779
P03410	P10636	0.19351
P03410	Q86US8	0.39414
P03410	O14628	0.95344
P03410	Q9HAN9	0.6449
P03410	Q96AQ9	0.52783
P03410	Q6EEV4	1.6653
P03410	Q5VZE5	0.39197
P03410	Q7Z6R9	0.2562
P03410	Q09666	0.94422
P03410	Q6ZQQ2	0.68301
P03410	Q9HCU5	0.23039
P03410	Q9BTD1	0.64757
P03410	Q3KQU3	1.0152
P03410	Q8NA72	0.62802
P03410	P11388	0.30471
P03410	P24390	0.93248
P03410	O15305	0.29228
P03410	P63104	1.0576
P03410	Q9NYP3	1.0027
P03410	Q9UBC3	0.27363
P03410	O94901	0.38431
P03410	Q15596	0.72317

P03410	Q13216	0.42751
P03410	Q9P086	1.0723
P03410	O15117	1.1994
P03410	Q5U649	0.56732
P03410	Q66K64	0.41023
P03410	Q8TCD5	0.39724
P03410	Q86TA1	0.57776
P03410	Q9Y5J6	0.68397
P03410	Q6ZV70	0.44977
P03410	P27635	0.88618
P03410	P61201	0.63582
P03410	Q8N108	1.1141
P03410	Q9NQP4	1.0405
P03410	P51948	0.48509
P03410	Q8NI51	0.52878
P03410	Q9NVS9	0.89231
P03410	Q13875	0.96048
P03410	Q9BQ69	0.76677
P03410	O15160	0.27865
P03410	Q5TEA3	0.68988
P03410	O60884	0.69534
P03410	P54826	0.52152
P03410	Q8IV56	1.1713
P03410	Q96JN0	1.3825
P03410	Q6ZTI6	1.0412
P03410	Q9UIC8	0.47383
P03410	Q6UWK7	1.6653
P03410	Q4G0U5	0.37289
P03410	Q8N8I6	1.6653
P03410	Q96KA5	0.61505
P03410	Q8IW35	1.5161
P03410	Q96RU7	0.36677
P03410	Q7Z7K2	0.66403
P03410	Q96EL2	0.61222
P03410	A0JLT2	1.4736
P03410	P51531	0.64729
P03410	Q8IYL3	1.6608
P03410	Q5JPI3	1.5224
P03410	Q6P047	0.97539
P03410	Q70EK8	0.84166
P03410	Q86UD0	0.72698
P03410	Q9HAP2	0.68873
P03410	Q9UKA2	0.51025
P03410	Q5JWF8	0.15343
P03410	Q7Z4H3	0.47358
P03410	H3BU77	1.2502
P03410	Q9UN81	0.78267
P03410	E9PIF3	0.091657
P03410	P24863	1.2361
P03410	Q9Y5B0	0.74633
P03410	Q6RSH7	0.54741
P03410	P59894	1.0174
P03410	Q99547	1.4361
P03410	A6NK06	0.9057
P03410	P49450	0.3655
P03410	Q7Z3E2	1.3304
P03410	Q9P2W6	1.6653
P03410	Q6IAA8	0.28527
P03410	A8MPS7	0.23867
P03410	Q9NZE8	1.5601
P03410	Q9P1Q0	0.76993
P03410	Q96CN0	1.6653
P03410	Q9Y6K5	0.3123
P03410	Q99643	0.49086
P03410	P28749	1.3793
P03410	Q9BW71	1.7008
P03410	Q8N8P6	0.8951
P03410	Q8TE02	1.4886
P03410	Q5T6C5	0.99972
P03410	Q9H7C9	0.18704
P03410	Q8N8F7	1.0913
P03410	Q8N1L9	0.92962
P03410	Q6UWP8	0.73644
P03410	Q9NWN3	0.72644
P03410	Q92636	0.57406
P03410	Q9NSI6	0.70003

20445	P03410	Q96IV0	0.65469
20446	P03410	Q9Y3E5	0.76681
20447	P03410	Q7Z3D6	0.058407
20448	P03410	Q9H568	0.23477
20449	P03410	P25789	1.0054
20450	P03410	Q9UKI9	1.1131
20451	P03410	Q96JN8	0.50771
20452	P03410	Q5FBB7	1.4376
20453	P03410	Q9NQ76	0.53387
20454	P03410	Q15544	1.421
20455	P03410	Q96S66	0.76126
20456	P03410	Q9H8N7	0.16095
20457	P03410	Q9Y606	1.5147
20458	P03410	Q9BT30	1.0143
20459	P03410	Q6VUC0	0.42484
20460	P03410	Q9Y3E1	0.43876
20461	P03410	P24666	0.11404
20462	P03410	P51854	0.29468
20463	P03410	P49720	0.7856
20464	P03410	P12980	0.88875
20465	P03410	Q9NUJ3	0.59727
20466	P03410	P0CJ88	0.27528
20467	P03410	Q86TX2	0.2809
20468	P03410	P63010	0.70352
20469	P03410	O75157	0.55393
20470	P03410	Q8IYD1	0.30223
20471	P03410	A6NN90	0.64029
20472	P03410	P58005	0.49927
20473	P03410	Q6ZTZ1	0.75508
20474	P03410	Q8TC94	0.23477
20475	P03410	Q96IT1	0.63738
20476	P03410	P57772	1.2258
20477	P03410	B4DX44	1.0058
20478	P03410	Q9P2H0	0.6999
20479	P03410	Q15643	1.3403
20480	P03410	Q86UY5	0.4095
20481	P03410	Q9UMY1	1.8848
20482	P03410	Q08J23	0.75891
20483	P03410	Q8IWD4	1.4883
20484	P03410	Q7Z5W3	1.1412
20485	P03410	Q9NPJ8	0.61963
20486	P03410	Q8NDA8	0.98374
20487	P03410	Q13901	1.0089
20488	P03410	Q6ZSG2	0.86592
20489	P03410	Q9H2T7	0.71856
20490	P03410	Q6ZW34	1.6653
20491	P03410	Q9ULM2	0.66931
20492	P03410	Q13888	0.53293
20493	P03410	Q8N7R1	1.0081
20494	P03410	P50539	0.32954
20495	P03410	O60318	0.84695
20496	P03410	Q2M3R5	0.99099
20497	P03410	Q15102	0.25454
20498	P03410	Q4G0F5	0.44515
20499	P03410	Q8NA47	1.4883
20500	P03410	Q8N402	0.2919
20501	P03410	Q8WYQ9	0.48976
20502	P03410	Q96QF0	0.80335
20503	P03410	Q9BTY7	0.56375
20504	P03410	O42043	0.91525
20505	P03410	Q86TG7	0.58815
20506	P03410	Q9Y6Y9	0.4136
20507	P03410	P55347	0.3769
20508	P03410	P60953	0.86143
20509	P03410	Q8N9G6	1.0986
20510	P03410	Q9Y6Z2	1.6653
20511	P03410	Q9BYG8	0.30396
20512	P03410	P42025	0.58211
20513	P03410	O60244	0.58395
20514	P03410	Q8TDR4	0.75634
20515	P03410	Q502W7	0.93668
20516	P03410	Q9UJC5	0.77229
20517	P03410	Q8IUE1	0.30793
20518	P03410	O00479	1.5441
20519	P03410	Q5TI25	2.3694
20520	P03410	P03897	0.22566

P03410	Q01130	0.8947
P03410	P09913	0.76802
P03410	Q96D46	1.3482
P03410	Q9ULL0	0.85706
P03410	A8MYV0	0.31374
P03410	P78543	0.84541
P03410	O43677	1.0318
P03410	P13929	0.090738
P03410	Q0VGL1	0.36832
P03410	P46087	0.81762
P03410	O60573	0.76203
P03410	Q7Z624	0.45391
P03410	A6NG73	0.40828
P03410	Q9Y2Y4	0.38605
P03410	Q9C093	0.31404
P03410	P0CZ20	0.55606
P03410	P40926	0.19264
P03410	Q9BZS9	1.6653
P03410	O75347	1.8555
P03410	Q9UHV7	0.58395
P03410	Q8N328	0.52041
P03410	C9JDV5	0.1196
P03410	Q14738	0.92883
P03410	Q9H8W4	0.42123
P03410	Q15742	0.3104
P03410	P35580	0.2103
P03410	O75477	0.88775
P03410	Q8IXQ3	0.5852
P03410	Q9BVP2	0.35561
P03410	Q9NRI5	0.55268
P03410	Q969U6	0.2744
P03410	O15091	0.22218
P03410	Q9Y5J9	0.96796
P03410	Q6ZU35	1.4883
P03410	Q8WV37	1.0058
P03410	Q9BQ95	1.2613
P03410	Q5VY09	1.6468
P03410	Q13616	0.85429
P03410	Q9H0K4	1.4247
P03410	Q8NC26	0.92854
P03410	P32243	0.70968
P03410	Q8IVK1	1.6653
P03410	Q9NX31	0.046431
P03410	P0CJ73	1.4876
P03410	Q13951	0.73419
P03410	Q0VDE8	1.2697
P03410	Q8NCU7	1.6974
P03410	Q969L4	0.9119
P03410	Q8WVM0	1.002
P03410	P28290	0.8142
P03410	A4D1E9	1.4713
P03410	P15516	1.0472
P03410	Q9Y4Z0	0.83083
P03410	O96033	0.38572
P03410	P11474	0.32587
P03410	A4D997	1.5115
P03410	P45984	0.33288
P03410	Q49A88	1.0455
P03410	P50542	0.30689
P03410	Q14746	0.79212
P03410	Q92664	0.31995
P03410	O75821	0.59742
P03410	Q9BZ01	0.56978
P03410	Q96FH0	0.86134
P03410	Q9Y5V3	1.0392
P03410	Q8TBP5	1.0913
P03410	Q96C01	0.31479
P03410	Q6ZTI0	1.6653
P03410	P0CJ86	0.27528
P03410	Q14699	0.72512
P03410	Q9Y6G5	0.6999
P03410	Q9HD40	0.61333
P03410	A8MWP6	1.6653
P03410	Q96RK0	1.0254
P03410	Q14681	0.32577
P03410	Q8WVX3	1.0913

P03410	Q6NVV3	1.0846
P03410	P0CJ69	1.4876
P03410	P30153	0.64348
P03410	Q96DZ7	0.91619
P03410	A6NJL1	0.46682
P03410	Q96FX7	0.52089
P03410	Q4G0X4	0.32577
P03410	A4UGR9	0.35443
P03410	P57081	1.1796
P03410	Q9UC07	0.79881
P03410	O95868	1.0602
P03410	Q9UJX3	1.0325
P03410	A6NLI3	0.20243
P03410	Q9Y248	1.0275
P03410	Q9ULW3	0.75001
P03410	P0CG12	2.1127
P03410	O14593	0.61251
P03410	Q9UKF6	0.79938
P03410	Q96M61	0.6999
P03410	Q6ZU69	1.1006
P03410	Q96RT8	0.72993
P03410	Q9HBF4	0.43703
P03410	Q8IXW0	0.77092
P03410	Q9BZF9	0.535
P03410	Q5T1B1	2.1304
P03410	Q8TC44	0.34176
P03410	P52434	0.40771
P03410	Q86VG3	1.1886
P03410	Q5VTE6	0.15173
P03410	Q9HA38	0.25854
P03410	Q8IW40	0.50868
P03410	Q9BQQ7	0.47911
P03410	Q8NCN5	0.24003
P03410	Q8TCI5	0.71409
P03410	Q8TEF2	1.3387
P03410	Q9H6T0	0.35313
P03410	P20073	0.53873
P03410	P23769	0.34136
P03410	Q8IZA0	0.0592
P03410	Q6ZNB5	0.42319
P03410	Q2NL67	0.32721
P03410	Q12799	0.11071
P03410	Q13761	0.67845
P03410	O95169	0.51228
P03410	P31941	0.39595
P03410	Q96JI7	0.79349
P03410	P63135	0.11835
P03410	Q6I9Y2	1.438
P03410	B7Z1M9	0.11374
P03410	A4FU69	0.28407
P03410	Q6ZP80	1.0913
P03410	Q9HAH7	1.3038
P03410	Q9UI43	1.444
P03410	Q2LD37	0.3533
P03410	O60346	0.26792
P03410	O94854	0.12345
P03410	F5H4A9	1.0913
P03410	Q5T7N2	0.39625
P03410	Q5XKP0	0.65097
P03410	Q05DH4	1.6962
P03410	Q9NPD8	0.45095
P03410	Q96EZ8	0.77433
P03410	O43189	0.41833
P03410	Q14586	0.6829
P03410	Q9NWW7	1.5859
P03410	Q15940	0.90727
P03410	Q96MC9	1.9869
P03410	P46108	0.55182
P03410	Q3B726	0.35464
P03410	Q9BTA9	0.95495
P03410	Q00005	0.88281
P03410	Q96MC5	0.00065626
P03410	P52630	0.62894
P03410	Q7L8S5	0.54772
P03410	Q8NCQ7	0.54579
P03410	Q3L8U1	0.70318

P03410	Q96C10	0.23974
P03410	P23258	0.56175
P03410	Q8NHS9	0.56184
P03410	Q8NFU4	1.6653
P03410	P62987	1.1948
P03410	Q330K2	0.63636
P03410	Q14201	0.16477
P03410	Q9UIS9	0.31582
P03410	Q8N9B5	1.0442
P03410	Q8ND56	1.2356
P03410	Q96KP1	0.64622
P03410	Q86Y28	1.0723
P03410	Q9ULW0	1.8578
P03410	Q9HB03	0.86335
P03410	P69208	1.6653
P03410	Q9BPW8	0.96226
P03410	Q9NPG2	0.34399
P03410	Q9H467	1.195
P03410	Q96PS6	1.0895
P03410	O75081	1.2054
P03410	P60413	1.9984
P03410	Q9UGM6	0.32264
P03410	Q9UBS4	0.61157
P03410	Q6WQI6	0.44669
P03410	P41252	0.79834
P03410	Q66K41	1.2915
P03410	Q96LS8	1.0967
P03410	Q9NQE9	0.44051
P03410	Q9BY44	0.61161
P03410	P56945	0.89379
P03410	Q5T749	1.5479
P03410	Q9P0S9	1.0946
P03410	Q9BSY4	0.86871
P03410	Q8NHW3	0.22147
P03410	Q5TCM9	1.6653
P03410	Q14166	0.26872
P03410	Q9Y3D9	0.82744
P03410	Q9UNS1	0.52289
P03410	A0PJX2	0.64771
P03410	Q96EZ4	1.6653
P03410	P53779	0.22985
P03410	O94776	1.0212
P03410	Q9HCS4	0.12816
P03410	P27695	0.37092
P03410	Q8NCA9	0.52846
P03410	B4DS77	0.74687
P03410	A0A183	1.6653
P03410	Q6ZTQ3	0.52583
P03410	Q7Z2W9	0.49961
P03410	Q96D96	0.88775
P03410	Q6BDS2	0.65073
P03410	Q96Q05	0.73085
P03410	O00425	0.49465
P03410	Q13547	0.87911
P03410	A6NLE4	1.0913
P03410	Q9Y6D0	1.1722
P03410	Q8IXS6	0.43892
P03410	O00762	0.59208
P03410	Q9HCI5	0.82061
P03410	Q9P206	1.462
P03410	Q9H0A9	2.1879
P03410	P68363	0.31103
P03410	Q01664	0.56543
P03410	Q15397	0.29436
P03410	Q494X1	0.59242
P03410	Q9C0F0	0.51683
P03410	Q6XCG6	1.6653
P03410	O75496	1.0949
P03410	A6NMC2	0.16455
P03410	Q9P2F9	0.65886
P03410	P0C7W0	1.4883
P03410	Q16864	0.19957
P03410	Q2M2Z5	1.4376
P03410	Q9Y471	0.3723
P03410	Q9NVH2	0.83476
P03410	Q96CS3	0.80511

P03410 Q96A56 1.4629
P03410 Q9BZR6 0.24744
P03410 Q86TB9 1.1985
P03410 Q9UJN7 0.68956
P03410 Q6ZMY3 0.31228
P03410 Q6ZSS7 0.72976
P03410 Q5TA77 1.6653
P03410 G3V211 1.8079
P03410 Q8WUM0 0.84061
P03410 Q6ZPB5 1.6653
P03410 Q96G04 0.16925
P03410 Q01094 0.68669
P03410 Q9NPE3 1.1541
P03410 O75717 0.53891
P03410 Q9Y426 0.41817
P03410 P35680 0.22877
P03410 P04279 0.25894
P03410 Q96M60 0.72188
P03410 Q5TB80 1.0535
P03410 Q9NWK9 0.99832
P03410 O60869 0.41141
P03410 Q96PV7 1.5141
P03410 Q8NBJ4 0.71496
P03410 Q7L2J0 0.28308
P03410 Q9BY77 0.39144
P03410 Q96RK1 0.65101
P03410 Q9Y2Z4 0.95851
P03410 A6NEJ1 0.35308
P03410 Q9H0P0 0.55339
P03410 P23297 0.65
P03410 Q9BZG8 0.40946
P03410 Q3Y452 1.6653
P03410 P57088 0.93989
P03410 Q7RTV0 0.31774
P03410 Q70IA6 0.41206
P03410 Q969N2 0.42327
P03410 O00167 0.491
P03410 Q14151 0.69216
P03410 Q9Y5U9 1.3374
P03410 Q6UXP7 0.49335
P03410 Q8TCB0 0.22221
P03410 P28698 0.76936
P03410 Q7Z7L9 0.80173
P03410 Q13619 0.60936
P03410 P49116 0.41432
P03410 Q9Y5Z4 0.31479
P03410 Q9NV96 0.18599
P03410 B2RD01 0.20899
P03410 Q86TS9 0.75992
P03410 A6NNA2 1.2142
P03410 Q8NB12 0.24861
P03410 Q8WVB3 0.20746
P03410 P17035 0.66045
P03410 Q6ZUT4 1.4703
P03410 Q8WUB2 0.74434
P03410 P57678 0.92634
P03410 A8MUZ8 1.0149
P03410 Q99698 0.37867
P03410 Q8N6V9 0.14714
P03410 Q96ES6 0.96838
P03410 O00287 1.6987
P03410 Q9BVJ6 0.99979
P03410 O00541 0.63009
P03410 Q8WTQ4 0.58393
P03410 O43790 0.91465
P03410 Q8IYJ0 0.8198
P03410 Q6NSI4 0.31948
P03410 Q53T94 0.4414
P03410 Q9UMS4 0.48912
P03410 P54922 1.0717
P03410 Q8TAQ2 1.2433
P03410 Q96BQ5 1.3053
P03410 K7EIZ4 1.6653
P03410 Q8IY18 0.44934
P03410 O43181 0.11683
P03410 Q8NAA6 0.1245

P03410	Q6P6B1	1.4883
P03410	Q969W0	0.79781
P03410	O75920	1.8909
P03410	O15481	0.15414
P03410	P13010	0.31191
P03410	Q9BUR4	0.94494
P03410	Q8WXC3	0.77302
P03410	O95671	0.93406
P03410	Q4G0S7	0.048766
P03410	Q6X4T0	0.63567
P03410	Q8NBR6	2.2078
P03410	Q9NZ38	0.60678
P03410	O14978	0.67474
P03410	P24534	0.86197
P03410	O43543	0.18664
P03410	Q99932	1.6653
P03410	Q12982	0.34114
P03410	Q8WW43	0.34794
P03410	Q9Y5K5	0.23792
P03410	Q96EU6	0.73366
P03410	Q8N4L1	1.0913
P03410	Q9UI47	0.5898
P03410	P59923	0.79465
P03410	Q496Y0	0.24877
P03410	Q86VX9	1.4597
P03410	O60925	0.36989
P03410	Q8WW33	0.95793
P03410	O75112	0.50274
P03410	P63279	0.61479
P03410	Q9BQI9	1.2702
P03410	Q3ZLR7	1.2614
P03410	Q9H8E8	0.86564
P03410	Q9H943	0.34725
P03410	P13984	0.51972
P03410	Q96IS3	0.26295
P03410	A6NDZ8	0.38071
P03410	P62857	1.219
P03410	Q8NA77	1.1273
P03410	Q0D2I5	0.46908
P03410	Q13465	0.23246
P03410	P49593	0.29605
P03410	Q5R3I4	0.36462
P03410	P54259	1.2905
P03410	O95522	0.15707
P03410	Q96MF4	1.3669
P03410	P26640	0.7316
P03410	Q8N5X7	0.23683
P03410	Q6Y7W6	0.62885
P03410	P10589	0.33186
P03410	Q6UXB1	0.87557
P03410	P0CL84	0.45056
P03410	P78371	0.55193
P03410	Q9NWS1	1.0605
P03410	Q14695	1.1402
P03410	Q9Y3C6	0.22157
P03410	Q56P03	1.233
P03410	Q9Y2H8	0.82386
P03410	Q63HQ0	1.6653
P03410	Q86VD1	0.66498
P03410	Q9BYR8	1.9066
P03410	O95793	0.56437
P03410	Q7Z7G8	0.1866
P03410	A7MD48	1.1372
P03410	Q6ZNB6	0.49838
P03410	Q5BLP8	0.95938
P03410	P14649	0.4316
P03410	Q8N9N8	0.28601
P03410	Q8N8G6	0.90599
P03410	P31689	0.66321
P03410	Q8WTY6	0.43867
P03410	A6NHQ4	1.2992
P03410	Q92966	1.3125
P03410	Q12768	0.57582
P03410	O75901	0.81184
P03410	Q96QA6	0.36709
P03410	Q3SY46	1.3983

P03410	Q9HBG6	0.26147
P03410	Q9BYC9	1.6633
P03410	P20933	0.46015
P03410	O75064	0.13432
P03410	Q5SQH8	0.43105
P03410	Q8N8W4	0.26149
P03410	Q6ULP2	0.9246
P03410	Q9NVF7	1.465
P03410	P56270	0.65635
P03410	P01358	1.6653
P03410	Q8N104	0.87928
P03410	Q9H8M7	1.0674
P03410	Q02577	0.53562
P03410	P26371	1.6653
P03410	Q00LT1	1.1529
P03410	Q5T0D9	0.72692
P03410	Q9NZ72	0.24615
P03410	P17677	0.29152
P03410	O95411	1.6653
P03410	Q9P2J8	0.78354
P03410	Q10587	0.26954
P03410	P61024	1.0772
P03410	Q8IUG1	1.2843
P03410	Q99456	1.0001
P03410	O14521	1.0344
P03410	Q5T655	1.6653
P03410	Q08AG7	1.1492
P03410	Q12972	0.61907
P03410	Q01546	0.83067
P03410	P61582	2.0038
P03410	Q9BY60	0.3132
P03410	Q13428	1.924
P03410	Q9Y6Y1	0.51107
P03410	Q86TE4	0.76568
P03410	Q9BQP7	0.49385
P03410	Q96HV5	1.0394
P03410	Q1MSJ5	2.2487
P03410	P0C7U9	1.1653
P03410	Q9HB09	1.8956
P03410	Q02078	0.48319
P03410	Q9UIF9	0.63479
P03410	Q04118	1.6653
P03410	Q5TC04	0.92569
P03410	Q92542	0.54116
P03410	Q5VYV7	0.6999
P03410	P11441	0.75314
P03410	Q5J5C9	0.73641
P03410	Q96BP2	1.3668
P03410	Q9Y2J8	1.0671
P03410	Q9GZN8	0.67771
P03410	Q69YZ2	0.96134
P03410	Q9BRX5	0.27618
P03410	Q6P2S7	0.24079
P03410	Q6UWH4	1.1688
P03410	Q6P589	0.74847
P03410	Q6PIF2	0.46984
P03410	Q7Z6J8	0.75406
P03410	Q96B97	0.58777
P03410	Q8N268	0.11189
P03410	Q8TEW0	0.41041
P03410	P83916	0.93328
P03410	Q8TE69	0.6999
P03410	Q86XK3	1.7054
P03410	Q3LIE5	0.71001
P03410	Q96GW9	1.0445
P03410	Q5TYW1	0.65413
P03410	O95886	0.77537
P03410	Q8IZU1	1.1496
P03410	Q16718	0.15831
P03410	Q92686	0.61247
P03410	Q6X4W1	1.9095
P03410	O43815	0.50147
P03410	Q9UHQ7	0.94184
P03410	Q9H2C0	0.73232
P03410	P35900	1.2722
P03410	Q8IUC6	0.49763

P03410	Q5T9Z0	0.83021
P03410	Q9Y296	0.51038
P03410	Q8WVY7	0.38484
P03410	O75113	1.1305
P03410	Q15542	0.95119
P03410	Q14CZ7	0.30076
P03410	Q494U1	0.41701
P03410	A8MTW9	1.6653
P03410	A8MT69	0.53976
P03410	Q9UNW1	0.41455
P03410	O43752	0.79543
P03410	Q8N684	0.57839
P03410	A6NK39	0.42281
P03410	Q9NUQ6	0.49184
P03410	P55209	0.79541
P03410	Q9NPB6	0.88426
P03410	Q9Y5A9	0.84029
P03410	Q0VD83	0.87318
P03410	Q16534	0.54311
P03410	P23284	0.53304
P03410	Q86V25	0.41938
P03410	A8MQ14	0.78951
P03410	P63241	0.64182
P03410	Q8WU58	0.72808
P03410	O95397	1.2748
P03410	Q16890	0.67233
P03410	Q6IBW4	0.7657
P03410	Q5SV97	1.3622
P03410	O15350	0.23658
P03410	Q96NG5	0.90928
P03410	A6NGK3	0.72331
P03410	A6NJ64	0.64742
P03410	Q9UBW8	0.4974
P03410	Q8N5Z5	0.25044
P03410	Q9H799	1.5787
P03410	Q7Z3K3	0.51198
P03410	Q8N8Q9	1.0846
P03410	Q49AQ9	0.46283
P03410	P07199	0.96468
P03410	Q96HR8	1.9313
P03410	Q9UHV9	0.98886
P03410	Q8IYG6	0.0065311
P03410	Q13347	0.90431
P03410	Q9UNP9	0.27027
P03410	Q5T5F5	1.6653
P03410	P41970	0.8024
P03410	Q13068	0.26031
P03410	Q2QGD7	0.57189
P03410	Q6ZSS3	0.62537
P03410	A4D250	0.32679
P03410	P80723	1.4047
P03410	Q8N140	0.94417
P03410	Q6TDU7	0.77969
P03410	O94818	1.757
P03410	Q16186	0.6992
P03410	Q9UBY8	0.26093
P03410	Q9NZI6	0.44919
P03410	P62487	0.66087
P03410	Q9H1D9	0.68421
P03410	Q9H6P5	0.63558
P03410	Q9H9Z2	0.73884
P03410	Q8IXR9	0.35337
P03410	P62263	0.80348
P03410	A4D0Y5	1.6653
P03410	Q96E14	1.2317
P03410	Q7Z422	0.14274
P03410	Q9UPV0	1.8244
P03410	P10163	1.6653
P03410	Q6ZN08	0.93658
P03410	Q5W1A9	0.57481
P03410	F5H284	0.22741
P03410	Q8WVM7	1.0651
P03410	P22532	1.6653
P03410	P07316	0.10294
P03410	Q9BZK7	0.87387
P03410	P41091	0.29955

P03410	P63316	0.2756
P03410	Q5TG08	1.5219
P03410	Q8N8U2	0.69471
P03410	Q8IZQ5	0.41515
P03410	P24310	0.65031
P03410	A6NH13	0.92022
P03410	O95159	0.76078
P03410	H3BPM6	0.018954
P03410	Q99590	0.63469
P03410	Q3LI66	1.6653
P03410	Q9H091	0.49344
P03410	Q15631	0.80813
P03410	Q9Y383	1.0374
P03410	P14136	0.74327
P03410	Q96LX7	0.25839
P03410	O95922	0.039274
P03410	A8CG34	0.73923
P03410	O14519	0.66803
P03410	Q96GV9	0.80299
P03410	A6NKL6	1.0913
P03410	Q5VT03	1.3673
P03410	Q9H8G1	0.98893
P03410	Q5SNV9	0.92638
P03410	Q07627	1.2381
P03410	Q7KZF4	0.98422
P03410	Q99962	0.44531
P03410	Q9GZN2	0.47625
P03410	P05204	1.4992
P03410	Q9Y2P0	0.8384
P03410	Q30KQ9	0.84486
P03410	Q86WV6	0.25932
P03410	O75306	0.75554
P03410	Q9P0U1	0.99708
P03410	Q14498	0.73214
P03410	Q8NEU8	0.60386
P03410	A6NJR5	1.0907
P03410	Q9UKK3	0.77455
P03410	Q969M7	0.31298
P03410	Q8ND90	0.84562
P03410	Q8IYB0	1.6653
P03410	Q5JSJ4	1.8663
P03410	Q8N7C4	1.6653
P03410	Q9NQ48	0.61414
P03410	Q13907	0.49014
P03410	Q6ZRI6	1.0689
P03410	Q86XD5	0.50931
P03410	Q969S2	0.49845
P03410	A8K5M9	1.6435
P03410	P09917	0.26281
P03410	P62424	1.3754
P03410	Q7Z3Y8	0.88723
P03410	Q6ZS92	0.76694
P03410	Q8N8X6	0.15204
P03410	Q16204	0.28346
P03410	O43812	0.31534
P03410	A8MXT2	0.19519
P03410	Q8IWP9	1.9877
P03410	O15027	1.0091
P03410	Q587I9	1.3623
P03410	Q9UQB3	0.55316
P03410	O60220	0.95186
P03410	Q9H1Q7	1.4587
P03410	Q9Y462	0.68657
P03410	Q7L273	0.32577
P03410	Q13362	0.28004
P03410	Q6MZP7	0.71596
P03410	O94874	0.89517
P03410	Q86V48	1.7271
P03410	Q3SXZ3	0.7873
P03410	O75052	0.3191
P03410	P63261	0.24431
P03410	Q9H425	1.0786
P03410	Q9Y250	0.9418
P03410	Q86UE3	0.78793
P03410	Q32MH5	0.92601
P03410	Q6ZNE5	0.64088

P03410	Q9Y2D4	0.17046
P03410	P33176	0.24123
P03410	Q9NVD7	0.52314
P03410	O60543	0.49618
P03410	Q9H5Y0	1.1435
P03410	Q5RHP9	0.91384
P03410	Q8WVF5	0.32577
P03410	Q9NYZ1	1.3876
P03410	Q12816	0.40169
P03410	Q8NAE3	1.2155
P03410	Q8WV74	0.056785
P03410	Q7RTU5	0.27116
P03410	P08047	0.82621
P03410	Q8WXQ3	1.9485
P03410	P60606	0.96395
P03410	Q06587	0.48708
P03410	Q8NFR7	1.3798
P03410	Q96LP2	0.28516
P03410	P49005	0.63795
P03410	P27694	0.65028
P03410	Q96G79	0.68062
P03410	P62888	0.96158
P03410	A8MQ11	0.45008
P03410	P46695	0.15327
P03410	P50548	0.30273
P03410	Q9H9C1	0.54371
P03410	Q9Y3Y4	0.71683
P03410	A6NML5	1.0913
P03410	Q8TB45	0.32299
P03410	P13533	0.19684
P03410	P0C7H8	0.67361
P03410	Q96AQ1	1.4946
P03410	P00403	0.65997
P03410	O00115	0.59008
P03410	O60506	0.449
P03410	Q96FC9	0.26974
P03410	Q9P255	0.88814
P03410	Q8IXB3	0.89437
P03410	Q8IUC0	0.94971
P03410	Q5PR19	0.44794
P03410	P07864	0.69158
P03410	Q6ICC9	0.73265
P03410	A6NLF2	0.63451
P03410	Q13487	1.7977
P03410	Q8IY81	0.93229
P03410	Q12872	0.98479
P03410	Q9H900	0.73867
P03410	P61254	1.3028
P03410	Q15011	0.98164
P03410	Q09161	0.87583
P03410	O60248	0.51398
P03410	Q9BRP4	0.88248
P03410	O15446	0.68467
P03410	Q9NXB0	1.5026
P03410	Q8IXR5	0.74018
P03410	Q6UYE1	1.3002
P03410	Q6ZSJ8	1.4883
P03410	Q6L8H1	1.6653
P03410	Q9H2W6	0.75616
P03410	Q8N769	0.44013
P03410	Q96QA5	0.64006
P03410	P55197	1.0828
P03410	P17039	0.66065
P03410	Q9BYA6	1.6653
P03410	Q14139	0.54101
P03410	Q9NZL4	0.88134
P03410	Q6NV74	1.4883
P03410	Q8NCQ5	0.22943
P03410	Q9Y4C2	1.163
P03410	C9JBD0	0.9567
P03410	O15265	1.2567
P03410	Q92597	0.74977
P03410	Q01082	0.62286
P03410	P10109	0.20345
P03410	Q96JZ2	0.50436
P03410	P23760	0.75322

P03410	Q9Y266	1.4353
P03410	Q9Y657	0.42034
P03410	O43711	0.30188
P03410	O60291	0.22805
P03410	Q99856	0.6678
P03410	Q9UBD0	1.0049
P03410	O14653	0.42157
P03410	Q9NV70	0.65981
P03410	Q9BYP8	1.6653
P03410	Q9H0N5	1.1621
P03410	Q9NUR3	0.68021
P03410	Q8N0Z2	0.52405
P03410	Q8IZ40	0.57022
P03410	Q00653	1.1876
P03410	Q96T59	1.1653
P03410	O95873	1.4883
P03410	Q8IWB4	1.0269
P03410	Q8NCX0	0.7996
P03410	P46778	0.8783
P03410	Q9H7D7	0.49184
P03410	Q86U28	0.32184
P03410	P21980	0.30891
P03410	Q9BRQ6	1.983
P03410	Q9BV86	0.38162
P03410	Q9UPX8	0.49909
P03410	Q8NC56	0.70891
P03410	Q6ZRV3	1.6653
P03410	P43360	0.54253
P03410	Q9BVM4	0.94487
P03410	A6NKH3	0.77445
P03410	Q13617	0.84038
P03410	Q92600	1.2562
P03410	P52758	0.51748
P03410	Q3YBM2	1.7019
P03410	P51816	0.73113
P03410	P28072	0.80329
P03410	Q9UBW7	1.2096
P03410	Q6ZVW7	0.16595
P03410	A6NGB7	1.0913
P03410	O43676	0.99844
P03410	Q8IWZ6	0.26968
P03410	O14949	0.1488
P03410	O95229	0.90999
P03410	P54278	0.46795
P03410	Q86XW9	0.29877
P03410	O43148	1.1329
P03410	O15144	0.61163
P03410	Q96RE7	0.9667
P03410	Q99417	1.4681
P03410	Q14247	0.43105
P03410	Q68DL7	1.6653
P03410	Q8N0Z6	0.45627
P03410	Q8N3Y1	0.5744
P03410	Q9UJU3	0.64079
P03410	Q9P2M7	0.82126
P03410	Q8NFW8	0.58469
P03410	Q92567	0.90839
P03410	Q9BVK8	0.94893
P03410	A6NM45	0.64164
P03410	A6NMD2	0.31483
P03410	Q5H9T9	1.271
P03410	P83111	0.29437
P03410	O43687	0.93665
P03410	P62945	1.6653
P03410	Q8ND07	1.3719
P03410	Q658T7	0.36301
P03410	A6NJJ6	1.4883
P03410	Q9UFW8	0.37602
P03410	Q86YD1	0.97874
P03410	A8MVS5	0.65173
P03410	Q96A72	0.13493
P03410	O95900	1.3035
P03410	Q494X3	0.96545
P03410	Q9H081	0.86498
P03410	Q9UI32	0.47324
P03410	Q86Y46	0.71041

P03410	Q460N5	0.31993
P03410	P0C864	0.12692
P03410	Q9BRD0	1.0435
P03410	Q8IVY1	1.0992
P03410	Q5MJ70	1.0907
P03410	P56277	1.6853
P03410	P05387	0.8783
P03410	Q969R8	0.12624
P03410	Q8TBC5	0.28882
P03410	Q6Q795	1.6653
P03410	Q8N7J2	1.5275
P03410	O95336	0.28487
P03410	Q6NZ67	1.2292
P03410	O75352	1.0724
P03410	Q9P2M1	0.83836
P03410	P07814	0.83607
P03410	Q711Q0	1.6653
P03410	A6NK75	0.85017
P03410	Q9Y3R4	0.42833
P03410	Q8N9K5	0.86892
P03410	Q9NYF8	1.1182
P03410	Q8NET4	1.227
P03410	Q1RMZ1	0.93126
P03410	Q86UL8	0.4506
P03410	Q7RTP0	1.0547
P03410	O95201	0.73326
P03410	P61960	0.78174
P03410	A6NP11	1.0344
P03410	Q16656	0.67506
P03410	Q9BTM9	1.2893
P03410	Q9Y324	1.3729
P03410	Q8TF05	0.40888
P03410	Q9NPD3	0.22942
P03410	Q14676	0.81198
P03410	Q9NVU0	0.83257
P03410	Q8NDM7	0.79245
P03410	Q9P267	0.45826
P03410	P13051	1.9284
P03410	Q9H7S9	0.74778
P03410	A8MTY0	0.97478
P03410	Q9BSC4	0.36709
P03410	Q8TE76	0.45876
P03410	Q8IXY8	0.20053
P03410	Q6UXB3	0.52515
P03410	Q8TF20	0.77805
P03410	Q6ZVK1	1.0913
P03410	Q9NUG4	0.4182
P03410	Q86TN4	0.49663
P03410	Q96NR2	0.39437
P03410	Q96F83	0.7879
P03410	Q96F07	0.75565
P03410	Q6ZQT0	0.23161
P03410	A1A519	1.4661
P03410	Q9UJP4	0.32401
P03410	Q9NX46	0.47221
P03410	Q9HAV7	1.0396
P03410	Q9H0H9	0.0041282
P03410	P49753	0.22306
P03410	Q5VWK0	0.21953
P03410	P48200	0.56181
P03410	Q86VR2	1.0011
P03410	Q00987	0.21283
P03410	Q6ZP65	0.36828
P03410	Q6UXU6	1.6737
P03410	P35326	1.5188
P03410	Q8IZL8	1.7147
P03410	P60604	0.53543
P03410	Q5JXM2	0.85152
P03410	Q8N1D0	0.21055
P03410	O00631	1.3016
P03410	Q13601	0.5704
P03410	Q9NX65	0.71271
P03410	Q96FF9	1.2108
P03410	P42766	1.0003
P03410	Q5U623	0.97082
P03410	Q0VF49	1.6653

P03410	Q16384	0.86512
P03410	Q6KB66	0.71041
P03410	Q8TCE9	0.18375
P03410	O15442	0.18927
P03410	Q969F9	1.8526
P03410	Q9NQZ8	0.80606
P03410	P56589	0.13583
P03410	P14854	0.18174
P03410	A6NCJ1	0.41375
P03410	Q5BN46	0.36308
P03410	P35241	0.3332
P03410	Q9UJW3	0.63494
P03410	Q9H7U1	1.7701
P03410	P30414	0.20053
P03410	Q01469	0.39138
P03410	Q02952	1.3031
P03410	Q53GL0	0.12933
P03410	Q13228	1.4192
P03410	P0C5K6	1.6653
P03410	P15927	0.61985
P03410	O14512	0.38468
P03410	Q9H2K2	0.3463
P03410	Q6ZN68	0.68951
P03410	O15453	0.94419
P03410	Q569K6	0.36566
P03410	Q68CP9	0.50782
P03410	Q13404	1.1325
P03410	O75626	0.20023
P03410	Q5TID7	0.97355
P03410	Q96DF8	1.3349
P03410	Q9Y5L5	1.8762
P03410	Q9Y2X0	0.55052
P03410	Q96DX8	0.232
P03410	Q96EY5	1.1774
P03410	O95561	1.2516
P03410	O75340	0.56035
P03410	P49918	0.76772
P03410	Q14CX5	1.0913
P03410	Q8TF32	1.0344
P03410	O76095	0.75424
P03410	Q5VZK9	0.23712
P03410	Q86UD4	0.69928
P03410	Q5THK1	0.13223
P03410	Q14691	0.62528
P03410	P0CAT3	0.23586
P03410	Q96Q35	0.93904
P03410	P12524	0.31642
P03410	Q6J272	1.5909
P03410	Q63HK3	0.74461
P03410	Q06416	0.30793
P03410	Q9BZQ8	1.3954
P03410	O75475	1.3973
P03410	A7E2V4	1.819
P03410	Q06787	1.0983
P03410	Q9H3K2	0.22079
P03410	Q1A5X7	0.051403
P03410	Q9ULJ1	0.27892
P03410	P40925	0.62227
P03410	Q15937	0.73882
P03410	Q92629	0.35364
P03410	Q8NI32	0.58108
P03410	O75478	0.38587
P03410	Q96AQ2	1.0913
P03410	P58004	0.76115
P03410	Q96BK5	0.63858
P03410	Q6P9A3	0.75755
P03410	O95478	0.93819
P03410	Q8WW35	0.6999
P03410	Q5T200	1.1558
P03410	O43829	0.72248
P03410	A8MYP8	0.97182
P03410	Q9C099	1.2136
P03410	P17152	1.0823
P03410	P78347	0.42625
P03410	Q5VXU3	0.69813
P03410	O43463	0.93544

P03410	Q6ZRP5	1.6653
P03410	Q6ZN17	1.1352
P03410	Q9NX94	0.8599
P03410	Q99807	0.28322
P03410	Q15035	0.44735
P03410	Q53SZ7	1.4883
P03410	Q9HB19	0.84948
P03410	O00410	1.4472
P03410	Q9NQC8	0.183
P03410	Q14194	0.56899
P03410	Q9Y3A2	0.98848
P03410	O75223	0.38657
P03410	Q15070	0.61182
P03410	Q96FA7	1.8195
P03410	O96001	1.4734
P03410	Q6NVU6	0.4286
P03410	Q6P2Q9	0.61333
P03410	Q9NV31	1.1431
P03410	Q8WU03	0.92954
P03410	Q9NVA1	0.75083
P03410	Q96RY5	1.2213
P03410	P60006	1.5796
P03410	Q9Y4A0	0.098279
P03410	Q6ZQN3	1.7727
P03410	Q9H8H0	0.36709
P03410	O14744	0.90413
P03410	P80294	0.83308
P03410	P0C7X0	0.18282
P03410	Q9H4B6	0.71023
P03410	Q66LE6	0.61554
P03410	Q8N9K3	1.4883
P03410	Q63HK5	0.28089
P03410	Q8N8H1	0.89197
P03410	Q9HAY2	0.2347
P03410	O75123	0.75546
P03410	O95073	1.1794
P03410	Q8N8J6	0.51034
P03410	Q7RTT4	0.35308
P03410	P59780	0.40393
P03410	Q9P021	0.5078
P03410	Q6P087	0.3973
P03410	Q9BZR8	0.7226
P03410	Q86TA4	0.56865
P03410	P26641	0.7105
P03410	A6NL46	1.1861
P03410	A6NIE6	0.92581
P03410	Q8N693	0.33306
P03410	Q8NE31	0.2998
P03410	Q92805	0.25235
P03410	Q3ZCT1	0.76363
P03410	P35250	0.17332
P03410	Q8N782	0.99836
P03410	A6NLX3	0.63366
P03410	A4D0T7	0.98472
P03410	Q96L11	0.76686
P03410	Q93074	0.53794
P03410	Q9NX18	1.1608
P03410	O15116	0.88983
P03410	Q99622	0.4581
P03410	Q8N5M1	0.8072
P03410	Q9UI26	0.61101
P03410	P0DJG4	1.3184
P03410	Q9NRM1	0.95531
P03410	Q96DM1	1.3674
P03410	Q5TCZ1	0.47368
P03410	P83436	0.89552
P03410	P59990	1.9984
P03410	Q8IXW5	0.96701
P03410	Q9NUD5	0.18656
P03410	Q9H0K6	0.33286
P03410	Q96LW9	0.65456
P03410	O43745	1.0121
P03410	P32189	0.7713
P03410	H3BN30	1.6653
P03410	Q9BQ70	1.1754
P03410	Q6ZQV5	0.79465

P03410	Q86VI1	0.39523
P03410	Q9BXS4	0.50277
P03410	A6NDE8	0.508
P03410	P09001	1.3893
P03410	Q96CW5	1.296
P03410	Q8TF47	0.99815
P03410	P18754	1.2863
P03410	Q9ULV5	0.3744
P03410	A6NC05	0.21289
P03410	O00422	1.1693
P03410	Q9NWB6	1.4883
P03410	Q96DZ1	0.55354
P03410	P03901	0.70239
P03410	Q9NSB2	0.82869
P03410	Q6P575	0.59546
P03410	Q5TBB1	1.6907
P03410	Q5TAQ9	0.51567
P03410	Q6P4D5	1.5181
P03410	P84103	0.70018
P03410	Q5UIP0	1.5908
P03410	Q96M34	0.39672
P03410	Q5FWE3	1.0913
P03410	P15407	0.64859
P03410	Q12901	0.92885
P03410	Q629K1	0.94906
P03410	Q9NRC8	1.1236
P03410	P39019	1.1277
P03410	E9PGG2	0.2823
P03410	Q9H8V3	0.29546
P03410	P62312	0.89016
P03410	P47914	1.9853
P03410	Q9UH92	0.62927
P03410	Q8NE00	1.0935
P03410	O43791	0.69603
P03410	O00151	0.3918
P03410	Q5TA76	1.6653
P03410	Q14584	0.75519
P03410	Q8WVE6	1.1767
P03410	Q14146	1.6653
P03410	Q8N141	1.0409
P03410	Q9Y4A8	0.51127
P03410	Q8WU20	0.4284
P03410	Q9BYR3	1.9984
P03410	Q6ZR54	1.6653
P03410	Q9UQF2	0.55679
P03410	Q6P3W6	1.1275
P03410	P01009	0.28671
P03410	Q8N300	0.77005
P03410	Q13123	1.6965
P03410	P86478	1.6653
P03410	Q9NP87	0.33026
P03410	Q9Y2B0	0.3524
P03410	Q969H6	1.0882
P03410	O00257	0.88522
P03410	Q86XA9	0.62062
P03410	Q13105	0.73269
P03410	A1E959	0.87201
P03410	Q9UQ84	0.25766
P03410	Q5VIR6	1.2921
P03410	Q96C03	1.0938
P03410	P81277	0.59391
P03410	Q49AA0	0.97448
P03410	Q9C0H9	0.42525
P03410	Q6PGN9	1.4207
P03410	Q8IUI4	0.60145
P03410	Q9XRX5	0.81587
P03410	A5YKK6	1.3262
P03410	Q6NT46	0.508
P03410	Q6ZN57	0.69998
P03410	Q03701	0.45445
P03410	A6NKF1	1.2626
P03410	Q9H4W6	0.18756
P03410	Q5HYW2	1.462
P03410	Q9UL01	0.74631
P03410	Q9P270	0.82137
P03410	P08729	1.7329

P03410	C9J069	0.13512
P03410	Q9C0B1	0.31837
P03410	Q99871	0.80444
P03410	Q96BD8	0.84645
P03410	A6NFQ2	1.3107
P03410	Q8NAQ8	2.127
P03410	Q9P0J0	0.59436
P03410	Q9P0I2	0.71965
P03410	Q8TD26	0.17285
P03410	O43731	0.64272
P03410	P56750	0.53505
P03410	P0C7W8	0.55466
P03410	Q86U44	0.24115
P03410	O75691	0.96133
P03410	Q6UXP8	1.6653
P03410	Q96A09	0.9649
P03410	P14316	0.84711
P03410	P82933	1.2812
P03410	Q96BD5	0.55398
P03410	Q6NZ36	0.30005
P03410	Q8N815	0.10204
P03410	Q5T0Z8	1.4883
P03410	Q6ZN28	0.91467
P03410	O75360	0.55727
P03410	Q9BZ67	0.10086
P03410	Q8TDR0	1.8222
P03410	O95257	0.6831
P03410	A2RU37	0.63128
P03410	Q8TD23	1.0889
P03410	A8MSY0	0.40711
P03410	Q9Y2M0	0.88069
P03410	Q9NP81	0.88682
P03410	Q01518	0.18792
P03410	P20810	1.2323
P03410	Q7Z4Q2	1.4154
P03410	Q5SQI0	0.4805
P03410	Q13326	0.49183
P03410	Q9UKL0	1.3185
P03410	Q9UQR0	0.20579
P03410	Q96PQ5	0.42821
P03410	Q15929	0.88
P03410	Q32M88	0.17864
P03410	P43358	0.54253
P03410	P17041	0.70233
P03410	Q14209	0.99932
P03410	Q502X0	0.0039504
P03410	A1L4Q6	0.55701
P03410	Q96LZ2	0.096866
P03410	Q9Y693	0.93757
P03410	P23528	0.7389
P03410	Q16630	0.37673
P03410	Q5JUQ0	0.65688
P03410	Q15283	0.22406
P03410	Q9BQ65	0.77493
P03410	O75564	0.61042
P03410	Q9Y6K9	1.076
P03410	Q9GZV4	0.81614
P03410	P03923	0.78406
P03410	Q86U42	0.78283
P03410	Q5GJ75	0.066391
P03410	Q9NP84	0.63042
P03410	O75618	1.0922
P03410	Q6STE5	0.6343
P03410	Q9NZI7	0.20287
P03410	Q6ZS52	1.6653
P03410	Q5VZR2	1.8193
P03410	P27105	0.13933
P03410	Q9Y5N6	0.87326
P03410	Q6GMR7	1.0665
P03410	Q96SY0	0.21003
P03410	A6NK44	0.20498
P03410	Q2VPB7	0.98862
P03410	O95153	0.87956
P03410	Q9H0W8	1.1169
P03410	B1ANH7	0.17542
P03410	P40429	1.0721

P03410	Q04637	0.66515
P03410	Q9NXG0	0.39593
P03410	P55808	1.7008
P03410	Q14781	0.77625
P03410	Q96P71	0.69139
P03410	Q9P032	0.86652
P03410	O75529	0.57431
P03410	Q9NW38	0.83761
P03410	P40222	1.0179
P03410	O60516	0.55938
P03410	Q8IU99	1.0913
P03410	Q6ZVE7	0.89307
P03410	Q76N32	0.48513
P03410	Q538Z0	1.6653
P03410	Q9NX40	0.82766
P03410	Q8N3Z6	0.38493
P03410	Q5MNV8	0.75573
P03410	Q6PEW1	0.21526
P03410	O14524	1.244
P03410	Q86X51	0.79802
P03410	P25963	0.89229
P03410	Q8N183	0.26191
P03410	Q9Y2T7	0.27326
P03410	P57740	0.93836
P03410	Q96KW2	1.1151
P03410	Q8NEG7	0.71266
P03410	Q5T4I8	0.0013396
P03410	P55735	0.82391
P03410	P54253	0.89544
P03410	O14618	0.41982
P03410	Q8NEQ6	0.78305
P03410	Q16891	0.38563
P03410	P0C7V9	1.414
P03410	Q96HW7	0.87298
P03410	Q9HD15	0.66288
P03410	Q9BV90	0.27196
P03410	Q13424	0.71534
P03410	O15370	0.44962
P03410	P17707	0.20676
P03410	Q15744	0.30596
P03410	Q8WW22	0.70309
P03410	Q5JTV8	0.61541
P03410	Q15361	0.85921
P03410	P49458	1.5729
P03410	Q96MU6	0.71894
P03410	Q14582	0.3903
P03410	O94993	0.25711
P03410	P30305	0.55281
P03410	Q9Y3Z3	0.25389
P03410	O00423	0.86429
P03410	Q14696	1.0224
P03410	P60411	1.9984
P03410	P16930	0.24435
P03410	Q9H944	1.1159
P03410	Q9Y618	1.0766
P03410	Q96DY2	0.41301
P03410	Q0VDD5	1.6653
P03410	Q02080	0.37765
P03410	A1L190	1.688
P03410	Q8NE22	1.8629
P03410	Q92985	0.51996
P03410	Q8NCE0	0.99546
P03410	O60829	1.242
P03410	Q96N20	0.7133
P03410	Q9H5N1	0.9886
P03410	P20265	0.6048
P03410	Q6DD87	0.65198
P03410	Q8NBZ9	1.0294
P03410	O43290	0.86474
P03410	O00178	0.76998
P03410	O14879	0.74746
P03410	A6NKF2	0.7397
P03410	Q9NY33	0.53229
P03410	O60765	0.85876
P03410	O00186	0.87753
P03410	Q6ZUJ4	1.1003

P03410	Q8TCD1	1.2873
P03410	P69892	0.81619
P03410	Q96GI7	1.4883
P03410	Q8IZ16	0.69017
P03410	Q3LI81	1.9066
P03410	Q96QR8	0.56524
P03410	P39023	1.116
P03410	Q16626	1.1295
P03410	Q86YE8	0.79465
P03410	P55795	0.16337
P03410	P53675	0.53956
P03410	Q86YL5	0.53486
P03410	Q7RTU3	0.44898
P03410	Q9H583	1.2869
P03410	O14603	0.30118
P03410	Q7Z6K1	0.98195
P03410	Q6MZM9	1.6653
P03410	A8MUL3	1.6653
P03410	Q8NHZ7	0.8377
P03410	Q8TDB4	0.58222
P03410	Q9Y2P8	0.78098
P03410	Q96JP2	0.23178
P03410	Q14671	0.351
P03410	Q13439	1.4156
P03410	Q9UJT9	0.44726
P03410	P02792	0.07098
P03410	O94952	0.95306
P03410	Q8NDH2	0.18963
P03410	Q8NFQ8	1.06
P03410	Q9UK58	0.42123
P03410	O60264	0.26802
P03410	Q8NEF9	0.67906
P03410	Q8TEV9	1.088
P03410	O15260	0.32379
P03410	Q71F78	0.9249
P03410	Q9P2D0	0.36087
P03410	Q9H320	1.5796
P03410	P25788	1.0054
P03410	Q12837	0.60381
P03410	Q8NDP4	0.78104
P03410	Q12981	0.3431
P03410	O95721	0.55162
P03410	Q9BRR3	0.74224
P03410	Q8TA94	1.0016
P03410	Q6ZRV2	1.661
P03410	Q96L14	0.8074
P03410	Q13367	0.70912
P03410	Q8WY41	0.66394
P03410	Q6ZNK6	1.794
P03410	P67870	0.17101
P03410	Q9NWR8	1.0717
P03410	Q8IY51	0.11807
P03410	Q96LR7	1.3389
P03410	Q9H6K5	1.4883
P03410	Q99935	0.84459
P03410	Q96MF6	0.90048
P03410	Q9BPX3	1.0421
P03410	P11308	0.39231
P03410	E2RYF6	1.6653
P03410	Q8N398	0.90765
P03410	Q14CX7	0.71742
P03410	Q96RT7	0.72993
P03410	P60410	1.9066
P03410	Q96LM5	0.084742
P03410	Q5VVS0	1.6731
P03410	P0C7V4	0.13951
P03410	Q86UU5	1.1636
P03410	Q6ZUA9	1.0412
P03410	Q9NVX2	0.40561
P03410	Q15149	0.4003
P03410	Q9NSC2	0.27198
P03410	Q96LB3	0.81032
P03410	Q9UBB4	1.3896
P03410	Q9Y4F5	0.3132
P03410	Q86UC2	1.0711
P03410	O94983	1.303

P03410	Q9BYS1	1.2381
P03410	O95433	0.72928
P03410	Q9UHY8	0.66524
P03410	Q8TAL5	1.2666
P03410	Q04760	0.17707
P03410	Q8IYF3	0.71106
P03410	A2RTX5	0.76394
P03410	Q9P2L0	0.30029
P03410	Q96NA2	1.5713
P03410	Q9BVS5	0.53925
P03410	Q4V328	1.3304
P03410	Q8N371	0.25122
P03410	Q9Y314	1.0779
P03410	O43493	1.1978
P03410	P13805	0.15239
P03410	Q8N9U9	0.69112
P03410	Q99543	0.41666
P03410	Q5TG53	1.6653
P03410	O60831	1.5556
P03410	Q6ZUX7	0.88488
P03410	A6NFT4	0.27556
P03410	Q9NY43	0.41478
P03410	Q14507	0.9686
P03410	Q9Y664	0.72049
P03410	Q6ZTY9	0.85402
P03410	Q2NKJ3	0.97956
P03410	Q9BR84	1.0268
P03410	P35232	0.65261
P03410	Q9Y238	0.35665
P03410	Q9UID6	0.47086
P03410	P0CW23	0.9344
P03410	Q2TAL8	1.272
P03410	Q9Y6K1	0.38475
P03410	Q9Y5N5	0.58259
P03410	Q03936	0.94346
P03410	O75425	0.94999
P03410	O43257	0.64738
P03410	Q5VTL8	1.4945
P03410	Q13765	1.2949
P03410	Q5T319	1.6653
P03410	Q86XI2	1.2449
P03410	Q96C86	0.8795
P03410	O75940	0.78274
P03410	A6NMV5	0.24589
P03410	A8MYA2	0.4308
P03410	O43617	0.58984
P03410	P39656	0.70553
P03410	Q9BUE0	1.2612
P03410	P18146	0.80929
P03410	P29692	0.59554
P03410	Q96PG8	1.6653
P03410	Q9H633	0.66183
P03410	A1L443	1.8193
P03410	Q49A92	0.52662
P03410	Q9NZH4	1.0107
P03410	Q7Z2X7	1.4365
P03410	Q9H4H8	0.72223
P03410	Q96GG9	0.54985
P03410	Q9BQD3	0.75906
P03410	Q9BX69	0.22756
P03410	D6RF30	0.2977
P03410	O60477	0.76342
P03410	Q9NYB0	1.4134
P03410	B9ZVM9	1.8172
P03410	Q5SW79	1.2857
P03410	Q96K58	0.75894
P03410	P53804	0.46349
P03410	Q8N5U6	0.94049
P03410	Q7L0X2	0.75911
P03410	P60660	0.46127
P03410	Q8IX15	0.44901
P03410	O76021	0.7306
P03410	Q5T035	1.6329
P03410	Q96SK2	0.64038
P03410	P62166	0.4072
P03410	Q9NP74	0.26546

P03410	Q8N841	0.22854
P03410	Q5T6X4	1.4742
P03410	Q9H0R3	0.95723
P03410	P31150	0.17674
P03410	P17021	0.64448
P03410	Q6PI77	1.0268
P03410	P57076	0.60386
P03410	Q6P161	0.49843
P03410	Q569H4	1.4883
P03410	Q68CZ1	0.35557
P03410	P55789	0.39153
P03410	Q8WUD4	0.377
P03410	O75164	0.92822
P03410	Q86W50	0.83413
P03410	A8MZA4	0.75591
P03410	A6PVC2	0.2131
P03410	P15622	0.85653
P03410	Q4KMZ1	0.56477
P03410	P62633	0.3727
P03410	Q86UB9	1.4081
P03410	P52744	0.75611
P03410	P13647	1.1375
P03410	B3KS81	0.55147
P03410	P51522	0.75477
P03410	Q658L1	1.6511
P03410	Q4KN68	0.16385
P03410	P17023	0.71552
P03410	Q7Z6Z6	0.54729
P03410	Q9Y5M6	1.6653
P03410	O75820	0.66045
P03410	Q12769	1.2264
P03410	Q9GZQ3	1.5402
P03410	Q8N895	0.69209
P03410	Q8WUA4	0.30191
P03410	Q01850	1.0107
P03410	Q3LI64	1.6653
P03410	Q9H5F2	0.96209
P03410	Q7Z3C6	0.76285
P03410	Q15181	0.27282
P03410	Q16740	0.84368
P03410	Q9Y262	1.0652
P03410	O14645	0.3784
P03410	Q5MJ08	0.77592
P03410	Q6ZTU2	1.4449
P03410	O95613	1.1614
P03410	Q5VTD9	0.22437
P03410	P0C853	1.6653
P03410	Q12824	0.94877
P03410	P52292	1.2233
P03410	Q9Y2A0	1.0503
P03410	Q69YL0	1.6653
P03410	Q86W67	0.23056
P03410	Q8N6M0	0.34317
P03410	Q9ULZ0	0.34399
P03410	Q13432	0.81604
P03410	P69905	0.66878
P03410	Q96LR2	1.9365
P03410	Q96E29	1.0174
P03410	Q9HAV4	1.387
P03410	Q9Y2G5	0.62814
P03410	Q8NDZ2	1.0289
P03410	P35226	0.4944
P03410	O43913	0.47503
P03410	P40937	0.14937
P03410	Q99426	0.33
P03410	Q9C0F1	0.6494
P03410	Q9H6X5	0.19294
P03410	Q96H20	0.95804
P03410	Q96JC4	0.86338
P03410	Q9H501	1.7408
P03410	Q96MT3	0.85946
P03410	Q495Z4	1.0172
P03410	P20292	0.33119
P03410	Q9H488	0.0718
P03410	Q96EI5	1.1127
P03410	Q9Y247	0.23724

P03410	Q8TBZ8	0.76976
P03410	Q8N9A8	0.97209
P03410	Q8N7Z2	0.75591
P03410	Q8NAV2	0.8048
P03410	Q96D05	1.5748
P03410	O95202	0.53304
P03410	Q9H019	1.3102
P03410	Q96JS3	0.49009
P03410	Q9BT81	0.31201
P03410	Q14865	0.34887
P03410	Q13243	0.78749
P03410	Q13449	0.23516
P03410	Q6S9Z5	1.3361
P03410	Q9NX70	1.1518
P03410	Q6ZRU5	1.6653
P03410	Q6P2I7	0.62401
P03410	Q63ZY3	1.0986
P03410	Q8NA03	0.86553
P03410	Q8N6N6	0.14568
P03410	A6H8Z2	0.65109
P03410	Q9Y2Y0	1.2804
P03410	P82914	1.4495
P03410	O75570	1.2642
P03410	Q8N0V3	1.7167
P03410	Q96NJ3	0.76878
P03410	Q6NXR4	0.49127
P03410	A6NKF9	0.76462
P03410	Q5VTA0	0.62283
P03410	Q8N584	1.3223
P03410	Q8N852	0.75274
P03410	Q6L8G4	1.6653
P03410	Q9BU68	1.1435
P03410	Q8NDD1	0.27147
P03410	Q8TES7	1.0552
P03410	O95416	0.44592
P03410	Q05481	0.79552
P03410	A6NMX2	0.21144
P03410	Q96B01	1.5143
P03410	A6NEM1	1.5003
P03410	Q9HAB8	1.0742
P03410	Q6ZVQ6	2.0012
P03410	Q8IXU6	0.63604
P03410	Q9BWT7	0.44756
P03410	Q96GA3	1.1689
P03410	P62917	1.3937
P03410	Q9P242	1.4996
P03410	Q68DX3	0.92534
P03410	O43592	0.56513
P03410	Q2NL82	0.87064
P03410	P16383	1.7978
P03410	Q9BYR6	1.9066
P03410	P78352	0.92755
P03410	P52565	0.18221
P03410	P62851	1.7358
P03410	Q8N859	1.0068
P03410	Q9BX82	0.97452
P03410	A1L3Z2	0.22406
P03410	Q12874	0.43662
P03410	Q9HBD4	0.90233
P03410	Q5BKY6	1.6653
P03410	Q5VVP1	1.9538
P03410	O15503	0.43296
P03410	Q9Y3T6	0.92474
P03410	P51811	0.25054
P03410	Q9UM00	1.8322
P03410	Q7Z7H8	0.61341
P03410	Q6IA86	0.51756
P03410	A6NI56	0.051542
P03410	Q9UHA3	0.47761
P03410	P67775	0.92215
P03410	Q14566	0.25348
P03410	Q6ICG6	0.5005
P03410	Q14145	1.2923
P03410	Q7L945	0.78091
P03410	A0JNW5	1.1352
P03410	Q9Y3D0	1.2046

P03410	H3BRN8	0.25538
P03410	Q9H2L5	1.308
P03410	Q6ZMI0	0.28418
P03410	Q33E94	1.1291
P03410	Q8NFD4	0.74556
P03410	Q14154	0.8864
P03410	Q7Z388	0.68951
P03410	Q8IWN7	0.53969
P03410	P82921	1.582
P03410	Q92539	0.49814
P03410	Q9NVS2	1.6004
P03410	Q14592	0.84951
P03410	Q9GZU0	1.3669
P03410	Q13427	0.4438
P03410	Q96MW7	0.6932
P03410	Q8IWF7	0.15917
P03410	P40121	0.54989
P03410	Q86X59	0.058243
P03410	P83731	1.009
P03410	Q9C0C6	1.1683
P03410	P48552	0.69355
P03410	Q13625	0.50992
P03410	Q9Y5K8	0.2199
P03410	P56537	1.46
P03410	Q5SQ13	0.45171
P03410	Q6UXR8	1.6653
P03410	Q6P9A1	0.89709
P03410	P61966	0.59124
P03410	Q9UKN8	0.7568
P03410	O00255	0.32069
P03410	Q96MD2	0.34138
P03410	P78364	0.056237
P03410	Q9GZS0	0.32205
P03410	Q96JF6	0.66816
P03410	Q86V15	0.6473
P03410	O43823	0.65038
P03410	Q6ZS94	1.6653
P03410	O14867	0.37137
P03410	Q9NQX5	0.97136
P03410	P40424	0.42159
P03410	Q96CX3	0.78894
P03410	Q8IZY5	0.4687
P03410	Q8TED0	0.39481
P03410	A8MXY4	0.79552
P03410	Q5SYE7	0.99621
P03410	Q9H490	0.81273
P03410	O95394	1.273
P03410	Q6NXS1	1.4617
P03410	Q8IVP5	0.054461
P03410	Q9H4R4	0.19498
P03410	P82930	1.7924
P03410	Q7Z3U7	0.31557
P03410	Q9H2G4	0.64322
P03410	Q9UIA9	0.81283
P03410	P78424	0.2507
P03410	Q6ZU65	0.639
P03410	Q8N7R7	0.18379
P03410	P13535	0.19684
P03410	Q96NS5	0.25857
P03410	A8MUN3	1.6653
P03410	Q12887	0.74957
P03410	Q6VY07	1.1546
P03410	Q4VC31	0.1757
P03410	Q4ADV7	0.75421
P03410	P40305	1.0917
P03410	O94772	0.13812
P03410	P38159	0.71813
P03410	Q04743	0.31618
P03410	Q6JBY9	1.0034
P03410	A6NJB7	1.7603
P03410	Q9H4I3	0.093185
P03410	P0C7V0	1.6653
P03410	O15126	0.73702
P03410	P0C2W7	1.6653
P03410	Q9H0E9	0.18228
P03410	Q15796	0.84874

P03410	P55212	0.52169
P03410	A6NGU7	1.0029
P03410	P10075	0.70885
P03410	Q13166	1.6653
P03410	Q96I76	0.24444
P03410	Q8IY63	0.58031
P03410	P0C7X2	0.92552
P03410	Q6P582	1.3605
P03410	Q9UJQ4	0.17593
P03410	A6NJ88	1.2986
P03410	Q8IWZ8	0.28308
P03410	Q8TF40	1.3067
P03410	P22061	1.0562
P03410	P60228	1.1356
P03410	Q96CF2	0.48873
P03410	A2RUT3	0.972
P03410	A5PLL1	0.34399
P03410	Q8TA86	1.2159
P03410	Q96LM6	0.20917
P03410	Q13813	0.39958
P03410	P0C7Q3	0.29902
P03410	Q15532	1.1335
P03410	P35237	0.45007
P03410	P61579	1.1462
P03410	Q6UWS5	0.67765
P03410	Q9P258	0.75255
P03410	Q8N1I0	0.39163
P03410	Q5U4N7	1.6653
P03410	Q9UD71	1.3279
P03410	Q8WV19	0.61118
P03410	A6NNW6	1.3176
P03410	Q9UL36	0.6599
P03410	O95562	0.40382
P03410	Q9Y3A5	0.86309
P03410	P27352	0.91414
P03410	Q9Y237	0.33391
P03410	Q5SXM2	0.66095
P03410	A5D8W1	0.1242
P03410	P23921	0.15793
P03410	O75459	0.52028
P03410	Q5BJF2	0.8544
P03410	Q05932	0.53943
P03410	Q9BSH4	1.6943
P03410	Q1W6H9	1.7693
P03410	A6NLC5	0.79041
P03410	Q8IXM6	1.2515
P03410	E9PI22	1.2368
P03410	Q9BWN1	1.4883
P03410	Q9NRG9	0.90416
P03410	Q5T5B0	1.6653
P03410	P41208	0.78451
P03410	Q14839	0.46557
P03410	P08235	0.28743
P03410	Q8N4C0	0.67307
P03410	P62993	0.2937
P03410	P60331	1.9984
P03410	Q9UHN1	0.36384
P03410	Q8N8F4	0.59849
P03410	P15336	0.89217
P03410	Q9UEG4	0.85618
P03410	Q9NQX1	0.63142
P03410	Q9BXW3	1.6653
P03410	P11161	0.37317
P03410	Q9NXF7	0.5172
P03410	Q9Y4B6	1.0474
P03410	Q9UHI6	0.49357
P03410	Q92993	0.72733
P03410	Q8WUM4	1.4685
P03410	Q15723	0.98652
P03410	Q9H6Z4	1.384
P03410	A8MPV3	0.22827
P03410	Q5JRK9	1.879
P03410	Q9BXL8	0.44969
P03410	A8MW95	0.24357
P03410	Q5JS54	1.2322
P03410	Q9BRX8	0.22086

P03410	P19388	0.75145
P03410	P28066	0.91596
P03410	P59036	1.6653
P03410	Q8N8D9	0.75233
P03410	O43447	0.6121
P03410	P81877	0.58269
P03410	Q14244	1.2247
P03410	Q6UW78	0.95938
P03410	Q68DK2	1.064
P03410	Q9NR55	0.34363
P03410	Q3MIT2	0.33552
P03410	O60422	0.24545
P03410	Q15562	0.73616
P03410	Q92817	0.28389
P03410	Q8TDN4	1.3634
P03410	Q8WZA8	1.6653
P03410	Q9BV57	0.53855
P03410	O75635	0.25305
P03410	Q5VWP3	1.7495
P03410	P50440	0.72695
P03410	Q96IX5	0.46968
P03410	Q9Y5J1	0.27328
P03410	Q969L2	0.4281
P03410	Q8IV53	0.096873
P03410	Q7Z6K5	0.29881
P03410	Q9H269	0.53313
P03410	Q9NUP7	1.2464
P03410	Q9NP50	1.367
P03410	Q9NZB2	0.98452
P03410	Q3KNT9	1.1255
P03410	Q9HCM1	1.2543
P03410	Q8N2I2	0.80632
P03410	Q96PT4	0.25012
P03410	Q15493	0.090614
P03410	O15131	1.34
P03410	Q5T0U0	0.074171
P03410	Q9NR00	0.70319
P03410	Q9C086	2.0934
P03410	P35268	1.189
P03410	Q08211	0.27224
P03410	Q9BSN7	1.4048
P03410	A8MX34	0.82177
P03410	O14609	1.2808
P03410	P24864	0.71408
P03410	P0CJ78	0.66136
P03410	P12004	0.27694
P03410	P0C7U1	0.084981
P03410	Q14761	1.4547
P03410	B1AL88	1.0913
P03410	P56211	0.63237
P03410	Q8WZ69	0.1404
P03410	A8MVS1	0.95078
P03410	Q9NR77	0.94214
P03410	Q96HM7	1.2927
P03410	Q9H511	0.38071
P03410	Q9BZW4	1.1698
P03410	Q8WYP5	0.65733
P03410	P0C7Q2	0.28158
P03410	Q9NWS8	0.94643
P03410	Q8N5Y2	0.13653
P03410	P63313	1.2621
P03410	Q14241	0.63661
P03410	Q5BKX5	1.4883
P03410	Q96LM9	0.17311
P03410	Q64ET8	1.5698
P03410	Q8TB72	0.81525
P03410	Q9Y2R5	1.126
P03410	Q68DQ2	0.12197
P03410	Q03393	0.24951
P03410	P61803	0.92574
P03410	Q8TDY3	0.23477
P03410	Q86YH6	0.18129
P03410	O43681	0.42328
P03410	P40763	0.28766
P03410	Q86XR2	0.23867
P03410	Q8WUY8	1.01

P03410	Q5VZ46	1.0679
P03410	Q6IA69	0.59514
P03410	P32780	0.81648
P03410	Q8WXS3	1.9993
P03410	P0CG38	0.24431
P03410	Q9BTE0	0.16565
P03410	Q8TDH9	0.47267
P03410	P61956	0.94132
P03410	B1AL46	1.3673
P03410	O75122	0.47513
P03410	Q9NU63	0.62279
P03410	I6L9F6	0.65009
P03410	Q8NFH3	1.1317
P03410	A6NNF4	0.79552
P03410	Q68DI1	0.67541
P03410	P09914	0.6265
P03410	Q8NCE8	0.99565
P03410	Q15053	0.52558
P03410	Q9UBD5	0.83809
P03410	Q8TAD7	1.4883
P03410	P54098	0.76597
P03410	Q8N123	1.1392
P03410	Q8TBF2	0.49953
P03410	Q969V4	0.32837
P03410	Q9NWX6	0.79212
P03410	Q504Q3	0.14482
P03410	O43361	0.6323
P03410	Q96AE4	0.46724
P03410	Q8WW59	0.26078
P03410	Q9BZQ4	0.40724
P03410	Q9UMX2	0.25601
P03410	O76014	0.88723
P03410	P14621	0.16042
P03410	A6NDB9	1.184
P03410	Q8NDQ6	0.76374
P03410	Q969Z0	0.82024
P03410	Q9NZ45	0.7033
P03410	Q9Y2D9	0.72404
P03410	A8MU76	1.0965
P03410	Q5EBL4	0.68948
P03410	Q9UKY1	0.28447
P03410	P61353	0.91415
P03410	B4DU55	0.89343
P03410	Q96GY0	1.3776
P03410	Q9BRP1	1.1279
P03410	Q9NX09	0.48621
P03410	P04732	0.80043
P03410	C9JI98	1.0913
P03410	P13196	0.19646
P03410	O75817	1.1364
P03410	Q9NVL8	0.19497
P03410	Q68E01	1.2088
P03410	Q9UK45	0.84686
P03410	Q9UN30	0.24371
P03410	P07737	0.99006
P03410	Q58F21	0.32176
P03410	Q96SL8	0.65654
P03410	Q86YC2	0.82963
P03410	Q9UMX1	1.3649
P03410	P50135	0.078297
P03410	Q7L5Y9	0.48222
P03410	Q52M58	1.8247
P03410	P42566	0.37811
P03410	Q9UKJ3	1.3152
P03410	P0C7T4	1.6653
P03410	P42773	0.51059
P03410	Q9UGU0	0.35705
P03410	P18850	0.66709
P03410	O95164	1.0505
P03410	P56715	0.36138
P03410	Q9ULW6	0.51552
P03410	Q75QN2	1.5645
P03410	O00471	0.49824
P03410	Q13310	0.53017
P03410	Q9NRT5	1.1623
P03410	Q5T753	1.6653

P03410	Q9HCD5	1.1794
P03410	Q8WXH0	0.39008
P03410	Q9BRV8	0.81199
P03410	Q86X67	0.13271
P03410	O75880	0.22271
P03410	Q8NFM7	0.95673
P03410	Q93034	0.80373
P03410	O95363	1.0343
P03410	Q15517	1.0365
P03410	O75179	0.50359
P03410	Q8N635	0.15091
P03410	Q5XKK7	0.5134
P03410	Q9P2K3	1.3086
P03410	Q8NFR9	1.1256
P03410	Q6W0C5	0.48998
P03410	Q9P2X0	0.19655
P03410	Q494R4	0.013354
P03410	P08727	1.0001
P03410	P63162	0.90302
P03410	O75391	1.0722
P03410	A8MTY7	1.9984
P03410	A6NEL3	0.12972
P03410	Q96HA7	0.86419
P03410	Q99551	0.73502
P03410	O75971	1.7604
P03410	O94992	2.1024
P03410	Q9P1C3	1.0553
P03410	Q5J8X5	1.0913
P03410	Q9H095	0.56752
P03410	B4DZS4	0.6374
P03410	Q9NYJ1	1.7803
P03410	Q4V348	0.65413
P03410	Q9UBY9	1.125
P03410	O75467	0.76893
P03410	Q6UX82	0.53937
P03410	P0C5K7	0.13951
P03410	P62879	0.81791
P03410	P46109	0.32066
P03410	Q9UI14	0.14259
P03410	P10515	0.43936
P03410	Q3KP22	0.43829
P03410	Q96M29	0.87211
P03410	P22914	0.3743
P03410	Q8IXX5	0.79088
P03410	O60907	0.77663
P03410	Q6ICL7	0.70807
P03410	Q969R5	0.32618
P03410	P62891	1.4119
P03410	Q2TAM9	1.8193
P03410	P05783	1.5647
P03410	Q9BV73	0.98688
P03410	Q96MY7	1.9534
P03410	Q76KD6	0.83742
P03410	Q8TAT6	0.51753
P03410	P33076	0.53348
P03410	O00161	1.1445
P03410	P61575	1.1462
P03410	P43356	1.0701
P03410	Q96JJ3	0.5356
P03410	Q9Y5A6	0.66309
P03410	Q8N6T0	1.2441
P03410	Q96G61	0.34631
P03410	Q6PI48	1.28
P03410	Q8N668	0.8854
P03410	Q9UG56	0.72812
P03410	A6NN73	0.71504
P03410	A6NNH0	1.3563
P03410	Q13438	0.19378
P03410	Q6ZN32	0.30793
P03410	P28065	0.98244
P03410	Q86U38	0.38771
P03410	E9PB15	0.40292
P03410	Q8ND76	0.78859
P03410	Q15042	0.69717
P03410	Q9Y221	1.0399
P03410	Q96KT0	0.98322

P03410	Q9BVL2	0.87917
P03410	Q96B96	0.95154
P03410	Q9NRJ3	0.88121
P03410	P58557	1.6858
P03410	Q9BV47	0.44822
P03410	Q5T160	1.1545
P03410	Q92681	0.5953
P03410	Q7RTX8	0.044788
P03410	Q7LBR1	1.2457
P03410	Q14934	0.30406
P03410	Q6AZY7	0.74793
P03410	Q5T1J5	1.6853
P03410	Q9BQY9	0.76463
P03410	Q92890	0.86321
P03410	A1A5B4	0.92935
P03410	Q9BWQ6	0.7513
P03410	O14802	0.35498
P03410	Q8N8D1	1.8323
P03410	Q5JWR5	0.38787
P03410	Q4LDR2	0.96395
P03410	Q9BXU3	1.1058
P03410	Q9NQS1	1.2991
P03410	O43242	0.62103
P03410	Q5T9C2	1.4883
P03410	P27815	0.36817
P03410	E9PQX1	0.49868
P03410	Q6ZMV8	1.0173
P03410	Q8WVT3	0.12951
P03410	Q9BT25	1.0037
P03410	Q9NQA3	0.43138
P03410	B3EWG5	1.4883
P03410	Q96DG6	0.34674
P03410	Q12906	1.0923
P03410	Q9Y5B6	1.2191
P03410	Q9UPR6	0.52978
P03410	O94973	0.67136
P03410	Q9H9E3	0.93796
P03410	Q9Y2K5	0.61162
P03410	Q9BRK5	0.23443
P03410	Q96EF6	0.88333
P03410	P35611	0.1792
P03410	O43865	0.43055
P03410	Q6AI39	1.3918
P03410	Q9Y421	0.36202
P03410	P04198	0.79462
P03410	Q00978	0.79415
P03410	Q30KQ7	0.87928
P03410	Q5HYK3	0.9978
P03410	P47874	0.50871
P03410	Q96HS1	0.52338
P03410	Q14591	0.64587
P03410	Q8NF64	1.242
P03410	Q9BRS8	0.62236
P03410	O43345	0.77805
P03410	Q9P1D8	1.6653
P03410	Q9H0A6	0.58631
P03410	Q8WU90	0.95178
P03410	Q6BDI9	0.8359
P03410	Q86UU0	1.3666
P03410	Q8IXT2	0.15686
P03410	P56377	0.69547
P03410	Q9NRR8	1.2004
P03410	O94903	0.96793
P03410	Q969X6	1.1211
P03410	Q9HCZ1	0.71705
P03410	Q9H079	0.81748
P03410	O75448	0.60744
P03410	Q6PH85	0.12422
P03410	P0DI83	1.168
P03410	Q96EV8	0.29829
P03410	Q3LHN2	1.6653
P03410	Q96RN5	1.3009
P03410	O75896	1.4921
P03410	Q96M53	1.9188
P03410	P30307	0.88835
P03410	Q5TYX0	0.67757

P03410	Q9NPA3	1.7336
P03410	Q8N4Q1	0.37886
P03410	Q6ZNG1	0.81411
P03410	O95177	0.51921
P03410	P18859	0.15693
P03410	K9M1U5	1.6653
P03410	Q8N6L7	1.3271
P03410	Q92733	1.6653
P03410	Q8IWD5	0.98964
P03410	Q5VVH5	0.66707
P03410	Q9BYR9	0.67361
P03410	Q8WX93	0.65578
P03410	Q7Z3B3	0.43038
P03410	Q6ZV80	0.60764
P03410	Q6NTF7	0.21418
P03410	P04280	0.92241
P03410	Q9C002	0.43842
P03410	Q12929	0.35931
P03410	Q86XL3	0.5246
P03410	Q96T17	1.0054
P03410	O95568	0.074364
P03410	Q6ZW49	0.39462
P03410	O00743	0.83887
P03410	Q99750	0.87169
P03410	Q14192	0.54526
P03410	Q9Y5R4	0.27036
P03410	O60888	1.1061
P03410	Q9UJX6	0.67742
P03410	Q9BV19	1.0263
P03410	Q9P278	1.3816
P03410	Q9UFG5	0.6999
P03410	Q9Y2G7	1.0676
P03410	Q96EP1	0.36686
P03410	Q9BQ87	0.54613
P03410	Q96EY1	0.60413
P03410	O15397	1.1291
P03410	Q49MG5	1.616
P03410	O43365	0.33473
P03410	O43167	0.84197
P03410	Q96D98	1.4336
P03410	A2RUB6	0.33445
P03410	Q587J8	0.1935
P03410	Q9UID3	0.24129
P03410	O75348	0.31469
P03410	Q96A73	1.4476
P03410	Q8NHY0	0.555
P03410	P49247	1.3317
P03410	Q9UKK6	0.95222
P03410	Q16533	0.81172
P03410	A6NC42	0.78081
P03410	Q2WGJ8	1.7637
P03410	P60372	1.9984
P03410	Q9Y215	0.31652
P03410	A2RUG3	1.2052
P03410	P15976	0.26444
P03410	Q15012	0.94801
P03410	P11277	0.49396
P03410	Q6S8J3	0.23109
P03410	Q6P9B9	1.2973
P03410	Q9UET6	1.0876
P03410	P0CE67	1.6653
P03410	P05114	0.5542
P03410	Q9NZL9	0.16097
P03410	Q99747	0.9904
P03410	Q9UKG1	0.54328
P03410	P59103	1.2602
P03410	Q14236	0.86438
P03410	O60437	1.1368
P03410	Q93045	0.38186
P03410	Q9UFN0	0.92813
P03410	Q96PM9	0.3238
P03410	P29372	1.2707
P03410	P30405	0.22741
P03410	Q9H2D6	0.25029
P03410	Q92543	0.34827
P03410	Q8N573	0.13822

P03410	Q9BTX1	0.91955
P03410	P22103	1.6653
P03410	P30740	0.26959
P03410	P51580	0.6869
P03410	A6NEY8	0.55629
P03410	O14770	0.51709
P03410	Q6IE81	1.0407
P03410	Q5XKL5	0.36709
P03410	Q6P1K8	0.45857
P03410	P29966	2.0787
P03410	Q7LG56	0.13852
P03410	Q9GZU8	0.67831
P03410	Q86TC9	0.52365
P03410	A6NE82	0.38071
P03410	Q15072	0.71849
P03410	O95471	0.15894
P03410	Q86UP8	0.30478
P03410	O75312	0.80461
P03410	Q9Y5U2	1.4355
P03410	A6NEY3	1.594
P03410	P0CG47	0.60541
P03410	Q05BV3	0.3132
P03410	Q96IT6	1.6653
P03410	Q9N2J8	0.71368
P03410	Q8TF74	0.89895
P03410	Q9BZE4	0.37573
P03410	Q9BX66	0.56147
P03410	Q9BYD5	0.17388
P03410	Q0VAK6	0.22052
P03410	Q8N5T8	1.5759
P03410	Q06830	0.26137
P03410	Q9H4A5	1.5438
P03410	Q8IYM2	0.50774
P03410	Q5JSS6	0.66533
P03410	Q70YC5	0.87199
P03410	Q13442	1.5044
P03410	Q07352	0.761
P03410	Q9BZA0	0.03112
P03410	P34897	0.27685
P03410	Q9NUA8	0.72587
P03410	P86496	1.6653
P03410	Q86TJ5	0.79659
P03410	P36543	0.40503
P03410	A6NFF2	0.37831
P03410	Q3BBV2	1.289
P03410	Q5VWM3	0.16422
P03410	O14683	1.0122
P03410	Q9BQ90	1.1293
P03410	Q92759	0.69419
P03410	Q9Y2A4	0.9961
P03410	Q8IWJ2	0.98846
P03410	Q92615	0.97127
P03410	A2RRP1	0.46154
P03410	Q9UJM3	0.59185
P03410	Q9H6V9	0.90652
P03410	Q5JQS6	0.94898
P03410	Q6P1N0	0.53118
P03410	Q9UL42	1.1055
P03410	Q96SZ5	0.38363
P03410	O60518	1.2414
P03410	Q9BXY0	0.36709
P03410	Q9H9E1	0.3902
P03410	Q9BRQ5	0.31678
P03410	Q7Z4H7	1.0428
P03410	Q86TS7	1.6653
P03410	Q9UGJ1	0.65497
P03410	P52742	0.76906
P03410	Q92621	1.3554
P03410	Q7Z3T8	0.28114
P03410	P24588	0.21864
P03410	Q96PP4	0.40435
P03410	Q3LI67	1.6653
P03410	O60934	0.26237
P03410	O00458	1.2877
P03410	Q9BVW6	1.6653
P03410	Q4G0N7	1.0749

P03410	Q9UJW7	0.6829
P03410	Q9NRY2	1.3839
P03410	O00124	0.59898
P03410	Q5QFB9	1.3692
P03410	P24855	0.22922
P03410	Q9C0E2	1.1472
P03410	Q8IYI6	0.87605
P03410	P59037	1.5752
P03410	Q6NT89	1.67
P03410	Q6P587	0.16314
P03410	Q86V85	0.75362
P03410	P34896	0.36406
P03410	A2BFH1	0.22741
P03410	Q9H9L3	0.57508
P03410	Q30KQ8	0.87928
P03410	Q5T5P2	0.47842
P03410	Q70Z53	1.2338
P03410	Q7L9B9	0.55863
P03410	Q5JRA6	0.23659
P03410	Q9BUI4	0.31997
P03410	P29459	0.46541
P03410	Q8N7S6	1.6653
P03410	O60238	0.61398
P03410	Q6ZR52	0.79552
P03410	Q9NUD7	0.15354
P03410	Q502W6	0.18199
P03410	O43598	0.55422
P03410	Q96NT1	1.1873
P03410	P81534	1.0067
P03410	Q9Y6Q2	0.65793
P03410	A6NCL1	0.41744
P03410	P17038	0.79552
P03410	P0C7M4	0.2823
P03410	A6NDY2	0.74411
P03410	Q9BSQ5	0.60127
P03410	O75884	1.7438
P03410	Q06546	1.2018
P03410	O00221	0.33736
P03410	Q9ULN7	1.4297
P03410	Q96IU4	0.46912
P03410	Q9Y2M5	0.87162
P03410	O15263	1.0066
P03410	P62753	1.0886
P03410	Q9BZQ2	0.66899
P03410	O14737	0.65142
P03410	P78344	0.64224
P03410	O43237	0.22066
P03410	O60290	0.96695
P03410	Q7RTT3	0.37833
P03410	Q13352	0.46517
P03410	O95822	0.35538
P03410	Q5TC84	0.71223
P03410	Q6EMB2	0.18824
P03410	Q49AR2	0.8029
P03410	A4QN01	0.41466
P03410	Q6P5R6	0.54581
P03410	O14829	0.45562
P03410	O75886	1.4395
P03410	Q15022	0.67854
P03410	Q96KF2	1.6653
P03410	Q8NEG0	0.50372
P03410	Q9GZW5	0.70458
P03410	Q9NZP6	1.4336
P03410	O15194	0.16212
P03410	Q6ZVL8	1.6653
P03410	P17097	0.626
P03410	Q9C073	1.4667
P03410	A6NME0	0.62739
P03410	A0AVK6	0.29891
P03410	Q96I25	0.30489
P03410	Q8N3Z3	0.87997
P03410	Q9UJY4	0.98958
P03410	Q9UBQ5	1.0971
P03410	P23588	0.46323
P03410	Q15545	0.83915
P03410	Q9NPI1	0.80101

P03410	P17405	0.32757
P03410	Q8TBZ0	0.41912
P03410	H3BQW9	1.1237
P03410	Q9HB58	0.34429
P03410	I3L1I5	0.65174
P03410	Q5JTW2	0.7191
P03410	O60493	0.38472
P03410	Q8N8A4	0.27319
P03410	P56134	0.58768
P03410	Q32M92	0.22963
P03410	P46060	0.32749
P03410	Q14135	0.54613
P03410	O95070	0.43428
P03410	Q8NC51	0.90147
P03410	A8K8P3	1.2295
P03410	Q8N6M9	0.18606
P03410	O94888	0.68381
P03410	Q8NA31	0.82068
P03410	Q6IQ23	0.59859
P03410	Q6IPT2	1.9055
P03410	Q8TAF5	1.6653
P03410	Q3LI63	1.6653
P03410	Q9UI10	0.48853
P03410	Q9BXW6	0.3591
P03410	O75208	0.95091
P03410	Q9BYG4	0.96675
P03410	Q9NY27	1.6339
P03410	P17980	0.52941
P03410	Q5TIA1	0.12185
P03410	Q9H446	0.6999
P03410	Q5TFG8	0.76532
P03410	Q5HYR2	1.3763
P03410	Q13405	1.1191
P03410	Q8N3R9	0.39906
P03410	Q7Z5D8	1.0068
P03410	Q7Z3Y9	1.0001
P03410	Q8IYJ2	1.6077
P03410	A8K0Z3	0.32624
P03410	Q86TU7	0.27953
P03410	Q5W0V3	1.2824
P03410	Q8NB25	0.35216
P03410	Q5T7B8	0.34531
P03410	Q9UPN9	0.31696
P03410	O95994	0.13248
P03410	O43593	0.12793
P03410	P0CL83	1.0182
P03410	Q8WWR8	0.30058
P03410	P60709	0.24431
P03410	Q71UM5	0.88777
P03410	Q99873	0.65978
P03410	Q8NHY6	0.84049
P03410	Q9UIF7	0.51165
P03410	Q7L2Z9	0.45065
P03410	Q9NX95	0.8285
P03410	Q5VZV4	0.91931
P03410	Q9H5H4	0.76436
P03410	Q8N944	1.5275
P03410	Q0VG06	0.89588
P03410	Q9BVT8	1.4584
P03410	Q9BYQ0	1.9984
P03410	Q9H2J4	1.0701
P03410	Q7Z3V5	0.78793
P03410	O00566	1.1279
P03410	P49411	0.84144
P03410	P31946	0.61076
P03410	Q9H6Y2	0.28878
P03410	Q9BQ67	0.49184
P03410	Q8N4B5	1.4567
P03410	Q9BVL4	1.8288
P03410	Q5T9G4	1.5627
P03410	Q6ZNG9	0.15559
P03410	P53672	1.3539
P03410	Q96PB1	1.0984
P03410	Q9NS26	1.3238
P03410	Q6NUN7	1.6653
P03410	E0CX11	1.6653

P03410	Q8NA69	0.79917
P03410	O75832	0.65782
P03410	Q9HC52	0.78119
P03410	Q9UHC1	0.60231
P03410	Q8N7B1	1.0719
P03410	O14980	1.2917
P03410	P20618	1.0314
P03410	Q8NEA5	0.65542
P03410	Q9H761	0.96997
P03410	P78524	0.051672
P03410	O95391	0.34578
P03410	Q01543	0.36839
P03410	O43156	1.0743
P03410	Q9NX04	0.61094
P03410	Q86W11	0.6702
P03410	O43505	0.93885
P03410	Q13114	0.43561
P03410	P78337	0.27362
P03410	Q9BVA0	0.33607
P03410	P49902	0.088294
P03410	Q9P0K7	0.38538
P03410	Q8N8E3	0.56173
P03410	P40337	0.7402
P03410	P49642	0.50624
P03410	Q9HCU8	1.0644
P03410	Q92754	0.46107
P03410	P14324	0.23025
P03410	Q9H9Q4	0.6189
P03410	Q5SZJ8	0.13501
P03410	Q6MZN7	1.6653
P03410	Q86Y25	0.81906
P03410	Q96FN9	0.877
P03410	Q499Z4	0.80936
P03410	Q9NRQ5	1.6653
P03410	Q8IVM0	0.83836
P03410	P28370	0.44228
P03410	Q6ZTC4	1.6653
P03410	Q96KD3	1.9055
P03410	Q8IW50	1.4883
P03410	Q9Y4U1	0.51341
P03410	P22033	1.0003
P03410	P50991	0.33921
P03410	O75390	0.18749
P03410	Q3LI77	0.46908
P03410	Q9HB65	0.56643
P03410	Q9HBE1	0.55581
P03410	O76009	0.88723
P03410	Q9Y5P8	0.98025
P03410	Q8N393	0.81273
P03410	P48595	0.25305
P03410	Q13506	0.39317
P03410	P61077	0.53543
P03410	Q0P6D6	0.19775
P03410	Q2M3X9	0.82417
P03410	Q03111	1.1722
P03410	P07197	1.2446
P03410	P10606	0.27069
P03410	O14777	0.74555
P03410	Q9Y4J8	0.30618
P03410	Q4G0G2	1.1197
P03410	O96015	0.18613
P03410	Q8NEL9	0.097892
P03410	Q9UPN7	1.1342
P03410	Q13573	0.67263
P03410	O95373	1.3485
P03410	Q14203	1.3045
P03410	Q9BZE1	1.009
P03410	C9JE40	1.1194
P03410	Q9NNW5	0.79318
P03410	Q6B9Z1	1.0868
P03410	O95487	1.0642
P03410	P62837	0.53543
P03410	Q5QJ74	0.23477
P03410	P05814	1.3964
P03410	O95156	1.1068
P03410	Q96LD1	0.57553

P03410	Q5MJ07	1.5851
P03410	O60284	0.14332
P03410	Q5VV17	0.23523
P03410	Q9BX40	1.1703
P03410	Q05195	0.32774
P03410	Q1T7F1	0.54271
P03410	P49792	0.94507
P03410	Q9NX05	0.771
P03410	Q8NFR3	0.67623
P03410	Q06609	0.48907
P03410	P0C7T7	1.6653
P03410	Q15061	0.36709
P03410	Q96MW1	1.4883
P03410	Q9GZN7	1.0471
P03410	Q8NEM0	0.31479
P03410	Q15276	0.62758
P03410	Q01892	0.26519
P03410	Q8NBV4	0.98316
P03410	Q9BYG5	0.85202
P03410	O43819	0.41662
P03410	A7MCY6	1.1015
P03410	Q7Z5Y7	0.32577
P03410	Q8NHG7	1.3877
P03410	Q96MH2	1.2184
P03410	Q3SXR2	1.2658
P03410	Q8N184	0.68671
P03410	Q14240	0.48093
P03410	Q5T752	1.6653
P03410	Q7Z4Y8	0.36834
P03410	Q12894	0.61598
P03410	Q8HWS3	0.17943
P03410	Q14249	0.12251
P03410	Q9BSY9	0.56365
P03410	O75586	0.67422
P03410	Q8IZQ1	0.99732
P03410	P43897	1.9953
P03410	Q9HBU9	0.84707
P03410	Q9UL46	0.6925
P03410	Q13323	0.40236
P03410	Q02241	0.81019
P03410	P55345	0.41528
P03410	Q9BU64	0.61202
P03410	Q5TEZ4	1.6653
P03410	Q53S33	1.4703
P03410	Q9NQ31	1.2575
P03410	A6NJQ4	0.6566
P03410	P28074	0.88827
P03410	P0C221	0.18227
P03410	Q9BQ08	1.0699
P03410	P14921	0.89095
P03410	P10909	0.047769
P03410	O95059	0.75727
P03410	Q9BVM2	0.56932
P03410	Q15021	0.92904
P03410	Q2TAP0	0.6587
P03410	Q4KMQ1	1.4181
P03410	O43663	1.0617
P03410	Q7Z417	1.0018
P03410	Q8WYA1	0.26092
P03410	Q8N2Z9	0.64189
P03410	P61289	1.0914
P03410	O75530	0.66038
P03410	Q9NV39	1.6481
P03410	Q6PF18	0.2215
P03410	P55210	0.23771
P03410	Q8N128	1.6653
P03410	Q96RJ6	0.46522
P03410	A6NGY3	0.44154
P03410	Q9H9Y6	0.59027
P03410	Q9Y473	0.79126
P03410	O75791	1.0938
P03410	Q6PB30	1.675
P03410	Q9ULW8	0.75553
P03410	Q6ZP68	1.7919
P03410	Q9Y5X2	0.39503
P03410	Q8WVI0	1.1965

P03410	Q8N0S2	0.14863
P03410	O75394	1.0615
P03410	Q9NYU2	0.38775
P03410	O15083	0.90676
P03410	P12074	0.60877
P03410	P52747	0.83357
P03410	P0C025	1.2441
P03410	Q96DC7	1.7039
P03410	Q96NJ6	0.76936
P03410	Q5MNZ9	0.23052
P03410	Q9H503	0.67504
P03410	Q9UHR5	1.3414
P03410	P83881	0.98486
P03410	Q9H665	0.59272
P03410	P21695	0.21613
P03410	Q8IVC4	0.80657
P03410	Q9NQ88	0.45586
P03410	P17480	1.0259
P03410	Q9HCX3	0.66571
P03410	A8MTZ7	0.37547
P03410	Q9H0U6	0.97273
P03410	Q9Y6M1	0.39793
P03410	Q13435	1.1443
P03410	Q5VXD3	0.19886
P03410	Q8N9W8	1.9055
P03410	Q14690	0.59798
P03410	Q8WUY3	0.50071
P03410	Q8N1P7	0.40164
P03410	Q969T7	0.15166
P03410	Q86UK5	0.52718
P03410	Q68DK7	1.2419
P03410	Q12788	0.23584
P03410	P60866	1.2925
P03410	O43390	0.55171
P03410	Q8WTZ4	0.11325
P03410	P51398	1.4811
P03410	Q04725	0.8562
P03410	Q9UBF2	0.62725
P03410	O95149	0.6512
P03410	A4D1E1	0.97809
P03410	P01011	0.21139
P03410	Q9H0F6	0.41624
P03410	Q96AX1	0.40908
P03410	Q7Z589	1.6148
P03410	Q8IV50	0.18395
P03410	E9PKD4	0.14598
P03410	A6NHS1	1.0305
P03410	Q9UQK1	0.57887
P03410	Q9BW66	1.3201
P03410	P51504	0.77383
P03410	A2RTY3	0.57739
P03410	B7Z6K7	0.68283
P03410	Q9H307	1.4016
P03410	O95182	0.53398
P03410	Q9UJ94	1.6653
P03410	A6NGA9	0.87187
P03410	Q7Z6M4	1.4517
P03410	Q8N9F8	0.83665
P03410	P69891	0.81619
P03410	Q86WG5	0.33798
P03410	Q6ZVT6	0.45507
P03410	Q3LI58	1.6653
P03410	O75494	0.58073
P03410	P13056	0.61719
P03410	A4D174	0.21624
P03410	Q8WW01	0.73597
P03410	O75431	0.21269
P03410	Q5PSV4	0.48948
P03410	Q15007	0.88868
P03410	Q6P499	1.0846
P03410	O75155	0.87623
P03410	Q5VT06	0.8539
P03410	Q9NRG4	0.58731
P03410	Q13021	0.40115
P03410	Q3MIW9	0.77766
P03410	Q9H6Q3	0.38715

P03410	Q8NBZ0	1.9071
P03410	O95049	0.83082
P03410	Q96NL3	0.85653
P03410	Q13257	0.95804
P03410	Q99569	0.66553
P03410	Q3MIR4	0.0592
P03410	Q14331	0.8185
P03410	Q9NQS7	1.3531
P03410	Q9NZJ0	0.79948
P03410	Q13415	0.31159
P03410	Q8N4U5	0.22946
P03410	P13232	0.065134
P03410	O75607	0.89706
P03410	C9J3I9	1.0363
P03410	O14641	0.61499
P03410	Q8N0W7	0.83104
P03410	Q9H6U6	1.0239
P03410	Q96GK7	0.77695
P03410	Q13144	0.52201
P03410	O43900	0.37924
P03410	Q9H5Z6	1.4883
P03410	Q9Y597	0.32577
P03410	Q9UBG7	0.12181
P03410	Q9BUE6	0.15687
P03410	Q15528	1.1884
P03410	Q96DB2	0.24163
P03410	Q13316	1.0318
P03410	Q08379	0.74329
P03410	P07476	1.5535
P03410	P42677	1.1427
P03410	Q9BRL7	0.92069
P03410	Q6MZQ0	0.3474
P03410	Q9Y3D3	1.2073
P03410	Q9HCG8	0.29064
P03410	O15304	1.2593
P03410	Q8NHU0	0.37182
P03410	P61970	0.6849
P03410	Q9BXJ9	0.47362
P03410	Q9Y3B2	0.76191
P03410	Q58A44	1.6653
P03410	P54920	0.71845
P03410	Q1A5X6	1.4883
P03410	Q96LW7	1.3469
P03410	Q8IWY8	0.74816
P03410	Q96IK1	0.4097
P03410	Q9BXG8	0.51107
P03410	Q99741	0.68802
P03410	Q99689	0.54415
P03410	P20396	1.0655
P03410	P61457	0.91383
P03410	Q6IS14	0.49272
P03410	Q8N2M8	1.479
P03410	Q8IZC7	0.59155
P03410	Q9NZD4	1.3236
P03410	P10155	1.243
P03410	Q8N0Y2	0.53744
P03410	Q16587	0.72858
P03410	Q6PJI1	0.25743
P03410	Q96GC5	1.1191
P03410	O15499	0.20614
P03410	P50616	0.59326
P03410	Q7Z3Y7	0.74827
P03410	Q5TAA0	0.16864
P03410	Q08AG5	1.0612
P03410	Q96MG2	0.9117
P03410	Q7L0Y3	1.1609
P03410	Q9UFB7	0.76365
P03410	Q8WUF5	0.84007
P03410	Q7Z309	0.42449
P03410	Q9BRP8	1.5249
P03410	Q5T124	0.26902
P03410	Q8WTV1	1.3528
P03410	Q9NZV5	0.76667
P03410	Q9H0W7	0.073
P03410	Q15935	0.80479
P03410	P22735	0.23914

P03410	Q96BM0	1.05
P03410	Q53GS7	1.1709
P03410	Q03938	0.89555
P03410	Q6NW34	1.1653
P03410	Q99966	0.16214
P03410	Q86YI8	0.7804
P03410	Q16385	0.98678
P03410	Q6NX49	0.68529
P03410	Q86VW0	0.56561
P03410	O75616	0.68791
P03410	Q16635	0.50578
P03410	Q8NF91	0.76517
P03410	Q9NNW7	0.36996
P03410	Q9NXV2	1.5329
P03410	Q5H9M0	1.4995
P03410	P05198	0.83154
P03410	P27797	0.83569
P03410	Q8NCQ3	1.6653
P03410	P25440	0.42091
P03410	P14406	0.63257
P03410	Q9BSK1	0.70444
P03410	Q9NWZ8	0.87788
P03410	Q9BXS1	0.64369
P03410	Q71SY5	1.1007
P03410	Q02040	1.0931
P03410	Q16559	0.23876
P03410	Q9H2U1	0.29094
P03410	P54727	0.78219
P03410	Q9BTA0	1.4883
P03410	Q8IUC3	1.7887
P03410	Q8NBF4	0.017924
P03410	Q8TD06	0.31787
P03410	Q9H714	0.67886
P03410	P53801	1.4534
P03410	Q15048	0.0052673
P03410	P0CJ77	1.4876
P03410	O14529	0.35837
P03410	Q7Z7F1	0.43268
P03410	Q8N5R6	0.8406
P03410	Q9NRH1	0.62046
P03410	Q6ZRQ5	0.82817
P03410	P0C5Y4	1.2381
P03410	A6NDD8	1.289
P03410	Q96HE8	0.79384
P03410	Q9Y3B7	1.8132
P03410	Q9NSQ0	0.57606
P03410	P62318	0.85746
P03410	Q86WU2	0.24446
P03410	Q92870	0.7775
P14075	Q9Y2Z4	0.40331
P14075	P60953	0.34615
P14075	Q6L8G5	0.63821
P14075	P60331	0.78356
P14075	Q04727	0.4978
P14075	O43674	0.61283
P14075	P43351	0.3676
P14075	Q5BIV9	0.71971
P14075	Q8N3J3	0.45789
P14075	A6NJB7	0.59774
P14075	P11169	0.24848
P14075	Q96GX8	0.94025
P14075	Q8N490	0.33948
P14075	Q96QA6	0.097001
P14075	Q5T319	0.63821
P14075	P49321	0.62535
P14075	P53673	0.4432
P14075	P0CG20	0.2916
P14075	Q9NVU0	0.30728
P14075	Q9H307	0.68199
P14075	Q14146	0.63821
P14075	Q16633	0.3299
P14075	Q9BQI5	0.25248
P14075	K7EIQ3	0.69807
P14075	Q14457	0.33128
P14075	Q9H974	0.87529
P14075	Q86TU6	0.78299

P14075	Q8WWB3	0.062646
P14075	Q9Y6R9	0.86566
P14075	A6NH13	0.3214
P14075	Q9H6Z4	0.84571
P14075	Q9NQ33	0.26096
P14075	Q5VWM1	0.33501
P14075	P28069	0.46079
P14075	P42704	0.69626
P14075	Q8N196	0.50855
P14075	Q9HAY2	0.02175
P14075	Q6L8G4	0.63821
P14075	Q96EB1	0.8575
P14075	Q8IYN6	0.8608
P14075	Q3L8U1	0.62011
P14075	Q5VWX1	0.50571
P14075	Q0D2J5	0.21195
P14075	Q00059	0.66782
P14075	O95453	0.30002
P14075	Q9P2Y5	0.22
P14075	Q8N0Z8	0.54386
P14075	Q8WZA9	0.94025
P14075	Q13278	0.57763
P14075	Q92764	0.62407
P14075	Q9NZL3	0.44945
P14075	Q9Y2M2	0.42677
P14075	Q69383	0.589
P14075	Q8TB05	0.94025
P14075	Q92797	0.50381
P14075	A1L170	0.63821
P14075	Q9C0F3	0.12672
P14075	Q9P0V3	0.47168
P14075	P13051	0.91006
P14075	P52630	0.46295
P14075	P0C838	0.52542
P14075	Q4G0A6	0.82564
P14075	P48668	0.22406
P14075	Q9P000	0.25196
P14075	B1AJZ1	0.0073782
P14075	Q96MT8	0.70621
P14075	Q9NQG7	0.14398
P14075	Q9BSU1	0.49793
P14075	O75953	0.45785
P14075	Q5VSD8	0.34021
P14075	H0Y354	0.43864
P14075	Q5VTR2	0.50457
P14075	Q969F1	0.52887
P14075	Q8NCT3	0.94288
P14075	Q86UU0	0.78342
P14075	Q86WW8	0.47604
P14075	Q6P2E9	0.71817
P14075	Q96IP4	0.52552
P14075	P53804	0.47131
P14075	A6NFC5	0.58806
P14075	Q14781	0.55159
P14075	Q9H246	0.29763
P14075	P59990	0.78356
P14075	Q494X3	0.30057
P14075	Q8WXC6	0.46749
P14075	A8MT69	0.2372
P14075	Q9BY41	0.23775
P14075	Q9Y5P8	0.37386
P14075	P11387	0.46055
P14075	A8MYA2	0.27688
P14075	Q9UI26	0.15562
P14075	Q9UN30	0.12072
P14075	Q6P4R8	0.78412
P14075	Q9BZZ5	0.15662
P14075	A1A4V9	0.19565
P14075	P17480	0.76479
P14075	Q5BKX5	0.94025
P14075	P17029	0.24572
P14075	Q9UBW5	0.31745
P14075	O76014	0.70708
P14075	P49336	0.48027
P14075	Q9P2W6	0.63821
P14075	O95561	0.56893

P14075	Q6ZVN7	0.34062
P14075	Q8N6K4	0.63821
P14075	Q96RL7	0.6898
P14075	P06733	0.62724
P14075	P05204	0.52983
P14075	Q7Z3Z0	0.80046
P14075	Q96BY2	0.28506
P14075	Q68DY1	0.2938
P14075	Q2TAZ0	0.1668
P14075	Q9NVH6	0.32307
P14075	Q8N0U6	0.63821
P14075	P60604	0.19221
P14075	Q96F07	0.27332
P14075	Q6ZR03	0.28496
P14075	Q9NTW7	0.28182
P14075	P78560	0.33407
P14075	Q92545	0.76323
P14075	Q99871	0.30651
P14075	Q6PI97	0.45007
P14075	Q15059	0.98657
P14075	O15297	0.35567
P14075	Q6ZV77	0.63821
P14075	Q3SYG4	0.36769
P14075	Q6ZTI0	0.63821
P14075	Q9BTM9	0.68115
P14075	Q9P0U1	0.47429
P14075	Q9BQE4	0.40794
P14075	Q9UBS3	0.38908
P14075	Q9P1G2	0.45695
P14075	P29474	0.38259
P14075	A6NFT4	0.26322
P14075	P82979	0.14848
P14075	Q5H9L2	0.79563
P14075	P68871	0.3284
P14075	Q49AS3	0.48488
P14075	E9PI22	0.65129
P14075	P42766	0.54689
P14075	Q96AQ6	0.67534
P14075	Q7Z2E3	0.2593
P14075	P54136	0.57345
P14075	P78537	0.6991
P14075	Q6TFL3	0.14968
P14075	Q15257	0.28209
P14075	Q9UI66	0.63821
P14075	Q8NI51	0.4334
P14075	Q5SWL7	0.50336
P14075	P52292	0.66519
P14075	Q9Y5U2	0.90432
P14075	O14530	0.24124
P14075	Q9HAB8	0.59031
P14075	O43617	0.20877
P14075	Q96M11	0.94876
P14075	Q5JQF7	0.63821
P14075	A8MX34	0.49576
P14075	Q8NB91	0.2161
P14075	A6NLC8	0.81665
P14075	Q96FQ7	0.11439
P14075	H3BU77	0.46379
P14075	Q5H9M0	0.79841
P14075	Q99933	0.51804
P14075	Q9Y6J9	0.60448
P14075	Q96ST3	0.58189
P14075	Q9HBH1	0.56624
P14075	P51003	0.1449
P14075	Q9NSC2	0.2035
P14075	Q8TEV8	0.63821
P14075	Q1W209	0.63821
P14075	P22626	0.42497
P14075	O00566	0.58419
P14075	Q52LJ0	0.60059
P14075	Q96PI1	0.90865
P14075	O00213	0.25006
P14075	P08729	1.0616
P14075	Q16576	0.31899
P14075	Q14511	0.49027
P14075	Q92585	0.75404

P14075	Q15572	0.67745
P14075	A8MV65	0.63967
P14075	O15131	0.76685
P14075	A6NLJ0	1.0292
P14075	Q6ZNM6	0.62405
P14075	Q8TEQ6	0.54295
P14075	O14925	0.22989
P14075	Q9NVA1	0.44327
P14075	Q6IPU0	0.85167
P14075	Q15366	0.6477
P14075	Q12789	0.57691
P14075	P84101	1.0669
P14075	P61575	0.589
P14075	P43355	0.79804
P14075	Q96IU2	0.94459
P14075	Q96EZ4	0.63821
P14075	Q9BS18	0.62224
P14075	Q7L1V2	0.73083
P14075	A6NEH8	0.24216
P14075	Q52LD8	0.61594
P14075	O14646	0.45593
P14075	Q9UHL9	0.40974
P14075	Q9UK73	0.5162
P14075	P35869	0.27055
P14075	Q8IZU1	0.52558
P14075	Q3T8J9	0.38766
P14075	Q9BRP1	0.59532
P14075	Q9BUP3	0.14059
P14075	Q8NC74	0.27214
P14075	Q15819	0.41139
P14075	Q9H7H0	0.49088
P14075	Q6NUR6	0.60388
P14075	Q09666	0.45383
P14075	O60663	0.37128
P14075	P49207	0.63103
P14075	P40925	0.29894
P14075	Q8N1G2	0.16359
P14075	Q5T7P2	0.63821
P14075	Q9H425	0.47857
P14075	B3EWG5	0.94025
P14075	Q8TB33	0.15748
P14075	Q9Y6X4	1.1439
P14075	Q96GX9	0.20394
P14075	O15234	0.77358
P14075	Q8TAD1	0.43632
P14075	P09132	0.10466
P14075	Q9NQE9	0.053464
P14075	Q8TAQ5	0.2118
P14075	Q9P266	0.71234
P14075	Q8TB72	0.33442
P14075	Q9H6R7	0.1474
P14075	Q9BQS6	0.54344
P14075	P63165	0.64365
P14075	Q9BWN1	0.94025
P14075	Q56VL3	0.69389
P14075	Q9HB96	0.63821
P14075	O43156	0.65951
P14075	Q8N6T7	0.40256
P14075	Q8IXS0	0.94025
P14075	P51815	0.14594
P14075	Q9UGC7	0.51551
P14075	Q13505	0.64016
P14075	Q969W3	0.64762
P14075	Q9BUA3	0.71853
P14075	A6NJI1	0.68888
P14075	Q92990	0.29294
P14075	A6NHQ4	0.88236
P14075	P62857	0.40577
P14075	Q96CS3	0.33097
P14075	Q96S65	0.89467
P14075	P62304	0.521
P14075	P09497	0.23249
P14075	Q9GZV5	0.7949
P14075	P51825	0.7714
P14075	Q8NDM7	0.3781
P14075	Q9NTU4	0.74711

P14075	P52815	0.37416
P14075	Q5W0V3	0.68538
P14075	P0CG34	0.49457
P14075	P46781	0.78039
P14075	O95049	0.24992
P14075	Q96QR8	0.35229
P14075	Q15643	0.5604
P14075	Q969S3	0.47367
P14075	Q96GM8	0.19151
P14075	Q9BRJ2	0.21714
P14075	Q9C0E2	0.6423
P14075	Q8TAT6	0.18071
P14075	P61580	0.65433
P14075	H3BR10	0.63821
P14075	P63220	0.35782
P14075	Q15929	0.25516
P14075	Q5T5N4	0.20453
P14075	P53801	0.73364
P14075	Q96NR2	0.15164
P14075	Q99607	0.81303
P14075	Q8IXW5	0.51167
P14075	Q5HYK3	0.45041
P14075	Q14129	0.37166
P14075	Q16543	0.21074
P14075	Q6V9R5	0.30288
P14075	P0C7H8	0.49168
P14075	Q68DX3	0.2663
P14075	Q3KNV8	0.65799
P14075	A6NM66	0.2179
P14075	Q96GA3	0.55491
P14075	P54105	0.6983
P14075	Q7RTT5	0.12652
P14075	Q8ND83	0.93223
P14075	Q9UJY4	0.2736
P14075	Q96N21	0.65863
P14075	Q02410	0.32958
P14075	Q9NZQ3	0.49337
P14075	O75564	0.23985
P14075	Q9ULX3	0.5545
P14075	Q14746	0.20122
P14075	Q96HJ5	0.21617
P14075	P54253	0.70476
P14075	Q9BTK2	0.27476
P14075	Q05519	0.54111
P14075	Q9H788	0.67868
P14075	Q9H5J4	0.40775
P14075	O76021	0.23148
P14075	Q8WUB2	0.33058
P14075	Q9BSY4	0.48129
P14075	A8MWP4	0.27115
P14075	P32969	0.58612
P14075	A6NFY7	0.70357
P14075	P61960	0.31428
P14075	Q5T3Y7	0.074952
P14075	Q9NXV6	0.76248
P14075	Q9HAV7	0.39522
P14075	O43303	0.52394
P14075	Q16352	0.65268
P14075	Q8N6V4	0.32313
P14075	Q96E14	0.62693
P14075	A6NDU8	0.47307
P14075	P0DJG4	0.84631
P14075	Q9UHL3	0.25426
P14075	Q7Z2V1	0.63821
P14075	Q8TDR4	0.25641
P14075	Q8NEJ9	0.39451
P14075	A6NDD8	0.455
P14075	Q9H4X1	0.63637
P14075	Q9UGY1	0.13296
P14075	Q4KN68	0.28851
P14075	B4DYI2	0.094299
P14075	Q6TDU7	0.31935
P14075	Q5T0Z8	0.94025
P14075	Q9NP97	0.32727
P14075	Q14602	0.15038
P14075	Q5MNV8	0.4108

P14075	Q8IYA8	0.94025
P14075	Q5TA82	0.63821
P14075	Q7L592	0.44831
P14075	O43402	0.75836
P14075	P14866	0.37348
P14075	Q06250	0.63821
P14075	O60239	0.16812
P14075	Q96EL2	0.20824
P14075	Q9UK45	0.51929
P14075	Q6XCG6	0.63821
P14075	Q5SZD4	0.50589
P14075	Q7Z3Y9	0.80046
P14075	P35321	0.35546
P14075	Q86WG3	0.2259
P14075	Q8NE31	0.073086
P14075	Q9BYD1	0.63111
P14075	Q9BRV8	0.24391
P14075	P06454	0.626
P14075	Q9H5Q4	0.51608
P14075	Q9BYD6	0.52518
P14075	Q12962	0.86379
P14075	Q9H2G9	0.24296
P14075	B4DZS4	0.32924
P14075	Q6XLA1	0.008971
P14075	Q86SY8	0.63821
P14075	Q9BYV8	0.40034
P14075	Q09161	0.58293
P14075	I3L273	0.33358
P14075	Q9H5Z6	0.94025
P14075	Q12983	0.026812
P14075	O94811	0.42858
P14075	P35813	0.081222
P14075	Q02577	0.41162
P14075	Q9H583	0.72238
P14075	P62316	0.54936
P14075	P59022	0.49323
P14075	O95232	0.29947
P14075	P31689	0.26121
P14075	P0CB43	0.33592
P14075	Q5T1J6	0.10016
P14075	Q96MT3	0.52328
P14075	Q9ULC6	0.78762
P14075	Q96JB2	0.56494
P14075	Q8N616	0.63821
P14075	Q8TAD8	0.97899
P14075	P60006	0.72395
P14075	Q9GZY1	0.64373
P14075	Q96EY5	0.47733
P14075	P56181	0.72296
P14075	Q86U70	0.17054
P14075	Q53SZ7	0.94025
P14075	Q9H814	0.70865
P14075	A4QN01	0.11113
P14075	Q9NRX2	0.41264
P14075	A6NNA2	0.79777
P14075	Q96PM5	0.29113
P14075	Q53FT3	0.38549
P14075	Q16719	0.070377
P14075	P62495	0.41268
P14075	Q6PGQ1	0.45992
P14075	O94777	0.39535
P14075	Q6RVD6	0.63821
P14075	Q6Q759	0.63414
P14075	P55036	0.42319
P14075	Q9BYD2	0.78361
P14075	Q6RFH8	0.61677
P14075	Q8WXF1	0.7775
P14075	Q9HAI6	0.57505
P14075	Q08AF8	0.40982
P14075	P62952	0.58214
P14075	P25788	0.48239
P14075	Q7Z3Y7	0.52023
P14075	Q8N814	0.70051
P14075	Q9Y5B6	0.64112
P14075	Q5XG85	0.63821
P14075	B1AH88	0.63821

P14075	Q8ND61	0.78015
P14075	Q14764	0.78817
P14075	Q8TAV0	1.0059
P14075	P11532	0.24507
P14075	Q8NG48	0.064034
P14075	P54098	0.28289
P14075	Q14690	0.34017
P14075	Q9BSV6	0.22471
P14075	Q00005	0.22943
P14075	P42677	0.60427
P14075	O75391	0.5586
P14075	P13984	0.39456
P14075	Q658Y4	0.66101
P14075	Q99608	0.042971
P14075	Q9Y266	0.6542
P14075	Q8N8R7	0.4614
P14075	Q86X95	0.8897
P14075	Q13155	0.4049
P14075	Q9Y5A6	0.48168
P14075	P19338	0.37907
P14075	Q9BY65	0.45737
P14075	Q9GZT8	0.76042
P14075	Q99932	0.63821
P14075	Q9Y6K1	0.25404
P14075	Q969J2	0.14215
P14075	A6NMA1	0.27691
P14075	Q9Y375	0.38186
P14075	P62913	0.79736
P14075	Q9UHD4	0.50756
P14075	Q9GZP4	0.075614
P14075	Q9H175	0.80214
P14075	Q86SE9	0.75358
P14075	Q08752	0.20569
P14075	Q9Y5J9	0.35889
P14075	P35659	0.42726
P14075	Q8N9R6	0.54858
P14075	Q9H211	0.47015
P14075	Q2NKX9	0.94025
P14075	Q9NPJ6	0.74375
P14075	Q8N9N5	0.37973
P14075	Q92900	0.3582
P14075	O15111	0.44973
P14075	P82921	0.72617
P14075	P22532	0.63821
P14075	O75698	0.63821
P14075	Q9NQR4	0.20681
P14075	A8MTY0	0.29359
P14075	P22090	0.20339
P14075	Q99504	0.25141
P14075	Q9Y2J2	0.26573
P14075	Q86XD5	0.31912
P14075	Q6NZ67	0.50987
P14075	Q9UBY9	0.68878
P14075	Q7L5A3	0.61924
P14075	Q96N23	0.60487
P14075	Q8WZ26	0.22458
P14075	Q96GE5	0.2763
P14075	Q9BVC6	0.66466
P14075	Q3LI59	0.63821
P14075	O60841	0.2388
P14075	Q14264	0.27724
P14075	Q9P0M9	0.59136
P14075	Q6L8H2	0.63821
P14075	Q9H7H1	0.38606
P14075	Q6ZN32	0.11658
P14075	Q9NYK5	0.28734
P14075	Q9H7U1	1.0364
P14075	Q00613	0.68448
P14075	Q9GZU2	0.29226
P14075	Q96JE7	0.58747
P14075	Q5SVZ6	0.70207
P14075	Q01850	0.64365
P14075	Q86UC2	0.44488
P14075	Q6ZNE5	0.19597
P14075	P23497	0.64407
P14075	Q6ZTZ1	0.32845

P14075	P55327	0.15253
P14075	Q9BW60	0.40775
P14075	Q3LI54	0.63821
P14075	P55957	0.29833
P14075	P59052	0.39796
P14075	Q92681	0.21735
P14075	P23246	0.57366
P14075	Q92759	0.52313
P14075	P41227	0.21017
P14075	C9J069	0.040248
P14075	Q9HAP2	0.32458
P14075	Q32M84	0.10677
P14075	Q8N187	0.56873
P14075	Q9ULL5	0.63821
P14075	Q9UHR6	0.65251
P14075	Q9Y3C8	0.14292
P14075	Q9H2F5	0.33686
P14075	Q96KS0	0.049218
P14075	Q99990	0.32173
P14075	P12956	0.11131
P14075	P56537	0.90223
P14075	Q14207	0.77173
P14075	P50548	0.1789
P14075	Q13042	0.93887
P14075	Q8NBJ4	0.42008
P14075	Q7Z2Z1	0.70643
P14075	P11142	0.31373
P14075	Q9BWH6	0.6074
P14075	Q9Y3F4	0.55991
P14075	P20042	0.26771
P14075	Q4V326	0.079883
P14075	Q86SZ3	0.18668
P14075	O00534	0.23851
P14075	Q96IV0	0.29817
P14075	Q9NVI1	0.44901
P14075	Q96B49	0.8303
P14075	Q9BYQ2	0.78356
P14075	P0DMB2	0.46262
P14075	Q96D46	0.72978
P14075	Q9BPY3	0.2397
P14075	Q6L8G8	0.63821
P14075	Q02878	0.63717
P14075	Q8IXT1	0.22967
P14075	Q8WXD5	0.62462
P14075	Q6P1X6	0.90539
P14075	Q16533	0.46517
P14075	Q8N3Z6	0.064392
P14075	Q9UBC9	0.3509
P14075	Q9BRU2	0.26009
P14075	Q96FX8	0.54479
P14075	Q5SRI9	0.71216
P14075	O00273	0.075974
P14075	B4DXR9	0.28702
P14075	P09496	0.1163
P14075	P24863	0.86352
P14075	Q9GZT9	0.10047
P14075	Q15102	0.07777
P14075	Q86V48	0.89537
P14075	Q8N141	0.31207
P14075	P31350	0.19742
P14075	B1ATL7	0.36209
P14075	Q13105	0.32112
P14075	P28066	0.47523
P14075	P58557	0.95017
P14075	Q9BY89	0.21492
P14075	P07320	0.72901
P14075	P82912	0.52506
P14075	Q8WV07	0.29148
P14075	P55198	0.63466
P14075	O15360	0.51258
P14075	Q04206	0.45926
P14075	A6NHU5	0.43301
P14075	Q96GS4	1.0749
P14075	Q8IXP5	0.94025
P14075	Q9BSW2	0.50609
P14075	Q9NUJ3	0.46467

P14075	Q8IUC3	1.0403
P14075	Q5T0L3	0.83393
P14075	Q156A1	0.63821
P14075	O43809	0.51092
P14075	Q9Y2V0	0.24358
P14075	Q63ZY3	0.66332
P14075	P21675	0.34161
P14075	P58512	0.61032
P14075	Q9H1H9	0.33736
P14075	Q8IVM0	0.31745
P14075	Q8NEY3	0.49185
P14075	Q6ZN08	0.27123
P14075	Q6ZP98	0.46362
P14075	P17482	0.82933
P14075	O14681	0.63203
P14075	Q86YD1	0.64598
P14075	Q8IXR5	0.40244
P14075	B9A014	0.28267
P14075	Q96KQ4	0.63481
P14075	Q9H4I2	0.558
P14075	Q9UKJ3	0.53482
P14075	Q3KRA6	1.0791
P14075	Q6P9F0	0.21887
P14075	Q9H322	0.69734
P14075	A6NIE6	0.39396
P14075	Q9P2I0	0.6188
P14075	Q9NRQ5	0.63821
P14075	Q9P0W5	0.60952
P14075	Q96EH3	0.25205
P14075	Q6P1M9	0.26239
P14075	Q9NWB6	0.94025
P14075	A4QMS7	0.23508
P14075	Q5RHP9	0.39673
P14075	Q96LP4	0.21923
P14075	Q969R5	0.11228
P14075	Q9Y5R5	0.15761
P14075	Q14442	0.19434
P14075	Q969E8	0.92816
P14075	O95067	0.30592
P14075	Q75QN2	0.75559
P14075	Q15750	0.28889
P14075	Q15528	0.95395
P14075	Q8IWV1	0.32472
P14075	Q9BUK0	0.90115
P14075	A1L4Q6	0.16575
P14075	O15265	0.77973
P14075	Q9UM63	0.098535
P14075	Q8N5L8	0.75301
P14075	Q8IYW4	0.023025
P14075	Q9BUL8	0.39232
P14075	P06132	0.4431
P14075	Q569G3	0.94025
P14075	Q495Y7	0.045435
P14075	Q96L21	0.31227
P14075	Q9Y2S7	0.79854
P14075	Q8N3V7	0.56776
P14075	Q7Z591	1.0947
P14075	Q9BSF8	0.11945
P14075	Q96H12	0.78141
P14075	Q969Z0	0.37795
P14075	P09913	0.33595
P14075	Q9NX63	0.49245
P14075	P0CAP2	0.38257
P14075	C9JLR9	0.71853
P14075	P51523	0.071849
P14075	P78337	0.2673
P14075	P20810	0.44662
P14075	Q9BQ83	0.48227
P14075	Q96DE0	0.82122
P14075	O00512	0.54229
P14075	P24941	0.48998
P14075	Q5VWI1	0.79226
P14075	Q9H1I8	0.36816
P14075	Q8WU39	0.40535
P14075	P81274	0.3495
P14075	Q8NEE0	0.63821

P14075	Q13573	0.49357
P14075	J3KSC0	0.0026735
P14075	O14603	0.055327
P14075	O60645	0.30682
P14075	Q8WWQ0	0.41685
P14075	Q9Y6H5	0.53745
P14075	Q8N9A8	0.543
P14075	Q9H2C0	0.47824
P14075	Q9GZS1	0.1198
P14075	Q5TGS1	0.24395
P14075	Q8N5H3	0.94025
P14075	Q1W6H9	0.91459
P14075	E9PQX1	0.20371
P14075	Q96HJ3	0.94025
P14075	Q9HBD4	0.40643
P14075	P40424	0.4857
P14075	Q9BPX7	0.62242
P14075	Q9NPC6	0.70639
P14075	Q9BYR0	0.78356
P14075	Q14526	0.41751
P14075	Q9H8W2	0.27351
P14075	Q99865	0.61132
P14075	Q969S8	0.59012
P14075	Q5VZR2	1.0753
P14075	Q8N9W8	1.1182
P14075	Q8N5W9	0.58672
P14075	P51816	0.41631
P14075	P49902	0.15275
P14075	Q8N7X2	0.63821
P14075	A6NEQ2	0.63821
P14075	Q9UBZ9	0.25924
P14075	Q13951	0.49259
P14075	P16989	0.13163
P14075	P52737	0.40586
P14075	O15069	0.10449
P14075	P0C7X2	0.38596
P14075	A2RU37	0.27235
P14075	Q8NHY5	0.6945
P14075	Q9UMS0	0.65276
P14075	Q8N0V3	0.90198
P14075	A1L4L8	0.40582
P14075	Q7Z5V6	0.50401
P14075	Q99687	0.28185
P14075	Q86X60	0.41791
P14075	Q2M2W7	0.94025
P14075	P05783	1.046
P14075	Q9Y3D9	0.16984
P14075	P49006	0.64261
P14075	Q5JUQ0	0.55957
P14075	O75030	0.41538
P14075	Q13067	0.079883
P14075	Q9NV72	0.29359
P14075	Q14683	0.30907
P14075	Q96KR1	0.26905
P14075	P41250	0.22793
P14075	Q8N9E0	0.94025
P14075	Q8IXR9	0.19947
P14075	Q9BSB4	0.18194
P14075	Q13884	0.22686
P14075	Q9HAJ7	0.92333
P14075	Q96GM5	0.63021
P14075	Q5MJ09	0.30202
P14075	Q5D0E6	0.63823
P14075	D6RB28	0.66953
P14075	O96018	0.26831
P14075	Q9H0A9	1.1155
P14075	O60248	0.34844
P14075	Q96N11	0.70128
P14075	Q9UBU8	0.51404
P14075	Q96BY7	0.1178
P14075	Q92966	0.5471
P14075	Q8N813	0.15887
P14075	P62277	0.7914
P14075	P25440	0.32341
P14075	Q96LT6	0.40406
P14075	Q96QT6	0.70192

P14075	P55265	0.24512
P14075	Q9UQR1	0.61182
P14075	Q8NEM1	0.2938
P14075	Q96IM9	0.10219
P14075	Q9NQ32	0.6778
P14075	P53990	0.40002
P14075	O95684	0.53555
P14075	Q9H706	0.1668
P14075	Q08999	1.0658
P14075	P08727	0.80046
P14075	P06744	0.44457
P14075	O97980	0.41629
P14075	Q6P582	0.64529
P14075	Q7Z6J9	0.58774
P14075	P0C880	0.63821
P14075	Q9H8Q6	0.63939
P14075	Q96E09	0.8608
P14075	H0Y7S4	0.10476
P14075	Q9BQN1	0.74487
P14075	Q6NVV7	0.52478
P14075	Q8TD31	0.60534
P14075	Q6ZW61	0.034149
P14075	Q8N5J4	0.26526
P14075	Q15006	0.032464
P14075	Q96SY0	0.0065646
P14075	Q9NX70	0.79367
P14075	Q9NXZ1	0.11619
P14075	Q8N1B4	0.33566
P14075	B3EWG6	0.94025
P14075	Q8N371	0.15051
P14075	P52657	0.88878
P14075	Q9UHT4	0.63821
P14075	Q8WWB5	0.66335
P14075	Q0IIN9	0.3557
P14075	Q8IXJ9	0.54504
P14075	Q5T036	0.38061
P14075	Q17RA5	0.63821
P14075	Q96MT4	0.81829
P14075	Q6EEV6	0.033983
P14075	Q9H1L0	0.65526
P14075	Q6DHV5	0.16016
P14075	Q9Y6D9	0.64542
P14075	Q92844	0.2321
P14075	Q15051	0.36344
P14075	A8MTA8	0.30488
P14075	O14746	0.42529
P14075	P08621	0.28377
P14075	Q9NZ38	0.17505
P14075	O15372	0.42218
P14075	O75607	0.47046
P14075	O75323	0.47282
P14075	O60888	0.41355
P14075	Q3SYF9	0.63821
P14075	P23025	0.18394
P14075	Q6ZS46	0.93918
P14075	Q9BXQ6	0.63821
P14075	Q96JS3	0.1371
P14075	Q9NUL3	0.38096
P14075	Q9BYQ7	1.0211
P14075	Q8WU90	0.60099
P14075	Q9UK76	0.81714
P14075	Q9NVL1	0.014284
P14075	Q8WXE1	0.43207
P14075	Q5SWW7	0.63821
P14075	Q02241	0.46526
P14075	Q2TAK8	0.36693
P14075	Q5T4T6	0.77873
P14075	Q96LW7	0.69199
P14075	Q96GC5	0.4211
P14075	Q9UBF1	0.52695
P14075	Q3LI61	0.63821
P14075	Q7L2H7	0.36435
P14075	O43663	0.64493
P14075	Q4VX62	0.41744
P14075	Q9UI54	0.63821
P14075	F2Z3M2	0.63821

P14075	Q9NR46	0.22021
P14075	Q9Y2Q9	0.45911
P14075	Q9Y295	0.31414
P14075	Q9BVT8	0.82247
P14075	O94973	0.23894
P14075	P23381	0.23214
P14075	O95424	0.71944
P14075	Q5PR19	0.17132
P14075	O15083	0.34697
P14075	P0C875	0.088228
P14075	Q52LR7	0.16942
P14075	Q8IXQ6	0.53775
P14075	P62072	0.14262
P14075	Q6ZTY8	0.074477
P14075	Q8NHZ7	0.29476
P14075	O75347	0.78284
P14075	P23284	0.20142
P14075	Q8NFR7	0.84611
P14075	Q9BU68	0.73388
P14075	P68036	0.61168
P14075	Q8NC26	0.26464
P14075	Q8N2H4	0.78049
P14075	Q13495	1.038
P14075	Q658K8	0.44005
P14075	Q8WZ69	0.25441
P14075	Q9UPR3	0.66239
P14075	Q9NPI1	0.80791
P14075	Q5TG53	0.63821
P14075	P05412	0.62726
P14075	Q16385	0.56798
P14075	P62851	0.6785
P14075	Q5T6V5	0.53707
P14075	Q9BXL5	0.20683
P14075	Q9H6X5	0.041407
P14075	Q15542	0.7286
P14075	Q07021	0.56709
P14075	Q8N488	0.33785
P14075	Q9H3H1	0.42261
P14075	Q92979	0.18514
P14075	Q04725	0.64787
P14075	Q96MC9	0.99635
P14075	Q9Y2G0	0.42533
P14075	Q6PRD7	0.63821
P14075	P04733	0.28594
P14075	Q13542	0.40394
P14075	P18615	0.79738
P14075	Q9NTX7	0.26925
P14075	Q8N0W5	0.32825
P14075	Q96NJ6	0.1238
P14075	Q86WI3	0.2859
P14075	Q5MJ70	0.53769
P14075	P11802	0.62371
P14075	P42226	0.55597
P14075	O95503	0.6738
P14075	Q5BKZ1	0.42458
P14075	P54922	0.68799
P14075	Q96A26	0.24069
P14075	P40425	0.4202
P14075	Q06323	0.52434
P14075	Q2NL82	0.44922
P14075	O94874	0.54708
P14075	Q12800	0.21097
P14075	Q5VZE5	0.24345
P14075	O96000	0.5329
P14075	O75832	0.31384
P14075	Q96FA7	0.79359
P14075	Q9C075	0.62642
P14075	K9M1U5	0.63821
P14075	Q14938	0.036706
P14075	P11474	0.26794
P14075	Q9HB75	0.39046
P14075	Q6NUP7	0.26534
P14075	Q96J86	0.76323
P14075	Q93034	0.46522
P14075	Q8NFG4	0.74899
P14075	Q9H9E3	0.27809

P14075	Q9Y2D1	0.92854
P14075	Q6ZVL8	0.63821
P14075	Q2M3W8	0.31207
P14075	Q9H081	0.46073
P14075	P68104	0.53926
P14075	Q92734	0.033865
P14075	P01358	0.63821
P14075	Q9BRA2	0.2604
P14075	Q96CW1	0.31134
P14075	Q01543	0.1906
P14075	Q9UMX3	0.36409
P14075	Q9H6R4	0.37906
P14075	O75558	0.32299
P14075	Q9UQR0	0.096658
P14075	Q8NDF8	0.24397
P14075	Q53H64	0.13122
P14075	Q5VT06	0.31889
P14075	Q8NDD1	0.076154
P14075	Q9NWZ5	0.085298
P14075	Q9UM07	0.43602
P14075	Q15759	0.25235
P14075	Q9HCK5	0.4624
P14075	Q9BQA5	0.62247
P14075	Q14103	0.63907
P14075	Q59GN2	0.2931
P14075	Q6ZN01	0.36943
P14075	Q9BPZ2	0.64073
P14075	O75886	0.72414
P14075	O75648	0.57886
P14075	A6NGS2	0.57869
P14075	O60809	0.4843
P14075	Q07627	0.78379
P14075	Q9ULM6	0.76784
P14075	Q15928	0.31812
P14075	Q6ZVS7	0.13677
P14075	Q86V81	0.60787
P14075	Q9H6F5	0.15854
P14075	P57772	0.51273
P14075	P62324	0.53048
P14075	O75475	0.96677
P14075	Q8WYR1	0.076478
P14075	Q9BVC5	0.65631
P14075	Q9H079	0.41759
P14075	A6NG73	0.19029
P14075	Q2NL68	0.94025
P14075	Q86VS3	0.047905
P14075	O95182	0.28219
P14075	P61024	0.5264
P14075	P0DM35	0.61343
P14075	P35325	0.63821
P14075	Q9NYV6	0.35839
P14075	Q4LE39	0.78473
P14075	O00186	0.48359
P14075	Q6P6B1	0.94025
P14075	Q9NR22	0.19732
P14075	P05060	0.34509
P14075	Q13243	0.58708
P14075	Q16656	0.3168
P14075	Q8IY57	0.83561
P14075	Q9C0F0	0.28197
P14075	Q13166	0.63821
P14075	Q9UEU5	0.15388
P14075	Q14160	0.41994
P14075	Q9NX08	0.1668
P14075	Q9UI30	0.27896
P14075	A4D161	0.2661
P14075	A6NKX1	0.63821
P14075	Q14593	0.30184
P14075	P62837	0.38919
P14075	Q3LI66	0.63821
P14075	Q96P70	0.72362
P14075	P23588	0.15722
P14075	P08865	0.51556
P14075	Q5U5X8	0.99622
P14075	Q9BQA1	0.70732
P14075	P14859	0.91563

P14075	Q13618	0.27705
P14075	Q6ZUT1	0.63821
P14075	Q8N4B5	0.90467
P14075	Q5SZJ8	0.089095
P14075	A6NCJ1	0.24575
P14075	O60828	0.50747
P14075	Q6ZUT6	0.66194
P14075	P61576	0.23178
P14075	P60411	0.78356
P14075	Q5VYM1	0.76323
P14075	P22670	0.54891
P14075	Q9Y4C2	0.80726
P14075	Q5VU43	0.61008
P14075	Q9UM82	0.80393
P14075	Q92831	0.60514
P14075	Q16186	0.31754
P14075	P0CI00	0.43974
P14075	A6NHJ4	0.33283
P14075	Q93015	0.79846
P14075	Q7Z4H7	0.6011
P14075	Q9UKX2	0.10299
P14075	A6PVY3	0.25454
P14075	P80297	0.30195
P14075	P11831	0.41708
P14075	C9J6K1	0.57291
P14075	Q562F6	0.51577
P14075	O60481	0.30069
P14075	Q13769	0.40194
P14075	O75410	0.56765
P14075	P52747	0.59231
P14075	Q9BZD3	0.27999
P14075	Q8TAD7	0.94025
P14075	A6NN06	0.63821
P14075	Q96BW9	0.46055
P14075	P11172	0.31747
P14075	O95295	0.25777
P14075	Q8TAA3	0.17908
P14075	A1L443	1.0753
P14075	A6NF83	0.56442
P14075	Q8IV20	0.55317
P14075	Q96MR7	0.87186
P14075	Q8N5D0	0.26263
P14075	A6NCD4	0.32932
P14075	A6NEN9	0.023262
P14075	Q13522	0.057511
P14075	P0C866	0.11385
P14075	O43236	0.25348
P14075	Q9Y3C4	0.4918
P14075	O43395	0.58591
P14075	P62328	0.75046
P14075	Q9BWW4	0.66352
P14075	O43677	0.57816
P14075	Q15554	0.30613
P14075	Q9NYP3	0.72139
P14075	Q6ZST8	0.0159
P14075	Q5TG92	0.52419
P14075	Q15906	0.62244
P14075	Q8N1L9	0.63615
P14075	P61970	0.22584
P14075	Q8N612	0.7903
P14075	Q9NPJ4	1.2096
P14075	P10828	0.38764
P14075	Q9UKZ1	0.96867
P14075	Q96C90	0.47474
P14075	Q9BXJ9	0.50918
P14075	Q96QC0	0.82998
P14075	Q8NEF3	0.21834
P14075	Q9BSI4	0.33936
P14075	Q6P050	0.59526
P14075	Q13296	0.34912
P14075	P49756	0.29207
P14075	Q8N4B4	0.083567
P14075	Q96BZ8	0.94025
P14075	Q13506	0.19422
P14075	Q5TD94	0.23479
P14075	Q9P2H0	0.33171

P14075	Q07157	0.41446
P14075	Q8NA42	0.14434
P14075	Q99615	0.54599
P14075	P78545	0.63651
P14075	Q86UE4	0.41789
P14075	A6NJR5	0.53769
P14075	O60308	0.34839
P14075	Q14588	0.2118
P14075	Q99619	0.50366
P14075	Q8N9P6	0.63821
P14075	Q13064	0.10937
P14075	A6NGY1	0.68261
P14075	P78395	0.67348
P14075	A6NGG3	0.63821
P14075	Q96Q83	0.33236
P14075	Q9NRJ2	0.63821
P14075	Q49MG5	0.75766
P14075	A6NP61	0.63255
P14075	Q92782	0.36251
P14075	O76095	0.24162
P14075	Q6ZVC0	0.6106
P14075	Q9H2T7	0.21275
P14075	Q8N6T0	0.3752
P14075	Q6ZUT4	0.54705
P14075	Q8N9R8	0.56349
P14075	A6NHY6	0.15748
P14075	O14787	0.70074
P14075	Q9UKT4	0.66892
P14075	Q8WXF5	0.50859
P14075	Q86XF7	0.36328
P14075	Q6ZVQ6	0.9999
P14075	Q9BRT7	0.63821
P14075	Q15697	0.28011
P14075	O75791	0.68547
P14075	Q8NB50	0.22707
P14075	Q9NPD3	0.18534
P14075	Q6EEV4	0.63821
P14075	Q8TC05	0.76127
P14075	O43752	0.21412
P14075	Q10571	0.42008
P14075	P60900	0.30442
P14075	P17861	0.42949
P14075	Q15014	0.87304
P14075	O60732	0.1668
P14075	O60765	0.18055
P14075	Q8N9P0	0.35585
P14075	Q7L0Y3	0.68031
P14075	Q8NBB4	0.14747
P14075	Q08945	0.8172
P14075	P49427	0.36945
P14075	Q86XE5	0.53312
P14075	P34896	0.083289
P14075	Q92845	0.050097
P14075	Q9NT62	0.27157
P14075	Q9NQS7	0.60238
P14075	O95397	0.6834
P14075	Q5T749	0.93733
P14075	Q9Y6X3	0.74592
P14075	Q8NBA8	0.46089
P14075	Q15562	0.63425
P14075	Q6ZNG1	0.22707
P14075	Q7Z465	0.39554
P14075	Q9HD34	0.46303
P14075	A1L3X4	0.63821
P14075	Q9Y224	1.1048
P14075	O00470	0.44899
P14075	P27695	0.32257
P14075	Q9Y2A4	0.30068
P14075	Q8N140	0.59535
P14075	O43427	0.25412
P14075	Q8IWZ3	0.69844
P14075	Q9UFG5	0.39241
P14075	P51532	0.33786
P14075	Q8IXQ4	0.94025
P14075	P62244	0.47929
P14075	Q96HP8	0.95192

P14075	Q68E01	0.42113
P14075	Q6U7Q0	0.12954
P14075	P28347	0.76954
P14075	Q8WVM7	0.55807
P14075	O75143	0.96811
P14075	Q96HT8	1.1974
P14075	A6NEM1	0.66953
P14075	Q6ZS49	0.63821
P14075	Q8WTT2	0.23511
P14075	O60516	0.20884
P14075	P51959	0.36837
P14075	Q9Y3Y2	1.0326
P14075	Q9H7T9	0.45354
P14075	P30876	0.45925
P14075	Q9H0S4	0.021822
P14075	Q9BRP8	0.81265
P14075	P49406	0.37802
P14075	P40222	0.46758
P14075	Q15532	0.73327
P14075	Q9UMS5	0.86655
P14075	Q8IWD4	0.94025
P14075	A8MYZ0	0.58271
P14075	P53567	0.49768
P14075	Q9BQC6	0.71027
P14075	Q96PG8	0.63821
P14075	Q9Y2R5	0.47713
P14075	Q9GZW5	0.45626
P14075	Q12901	0.23554
P14075	Q5BN46	0.3115
P14075	Q9UNY4	0.33152
P14075	Q8TD23	0.45366
P14075	O15127	0.37333
P14075	Q2LD37	0.20013
P14075	A6NK89	0.53976
P14075	Q96GY0	0.80893
P14075	Q9H1Y0	0.3016
P14075	Q9UHR5	0.93707
P14075	P40855	0.32755
P14075	Q07666	0.31705
P14075	Q8NAE3	0.39536
P14075	Q9NX20	0.48896
P14075	Q3LI81	1.0514
P14075	Q53FD0	0.78008
P14075	A6NCN8	0.94025
P14075	P61579	0.589
P14075	P14635	0.18458
P14075	Q9GZP8	0.63821
P14075	Q9GZU8	0.32705
P14075	Q8N699	0.81722
P14075	Q96E11	0.77832
P14075	Q96HR3	0.61322
P14075	Q86VG3	0.64223
P14075	Q76L83	0.41695
P14075	Q6PJT7	0.66778
P14075	P62879	0.29199
P14075	P61326	0.75649
P14075	P25208	0.42791
P14075	Q7LBR1	0.61902
P14075	O15162	0.1639
P14075	Q12815	0.36597
P14075	P38432	0.63017
P14075	Q56P03	0.5678
P14075	Q9H2L5	0.54134
P14075	Q8NHS9	0.28241
P14075	P0CB33	0.28156
P14075	Q8N5I9	0.94025
P14075	Q9UM11	0.83486
P14075	Q86WX3	0.80821
P14075	O43598	0.24449
P14075	A6NNH0	0.76395
P14075	O00233	0.45875
P14075	P08708	0.2332
P14075	P52736	0.42046
P14075	Q4G0G2	0.3059
P14075	Q96MR9	0.25355
P14075	Q9P2G4	0.94025

P14075	Q9UHQ7	0.51551
P14075	Q9P2X8	0.30742
P14075	O43541	0.42624
P14075	P07199	0.53219
P14075	P47944	0.55106
P14075	Q15937	0.13498
P14075	Q6ZTU2	0.72639
P14075	A8MVJ9	0.48581
P14075	Q96SZ4	0.084617
P14075	Q9NQM4	0.005535
P14075	Q9BT78	0.7127
P14075	Q5MIZ7	0.2151
P14075	Q6ZU67	0.3676
P14075	Q9H444	0.44196
P14075	Q8NCQ2	0.46388
P14075	Q9H9G7	0.4624
P14075	Q8IYB0	0.63821
P14075	Q9BXM0	0.49265
P14075	Q9UKN5	0.066988
P14075	Q8N1B3	0.43901
P14075	Q9BY42	0.527
P14075	Q9C086	1.1042
P14075	Q01831	0.3875
P14075	Q9NWV8	0.36308
P14075	A8CG34	0.40877
P14075	Q9UHP9	0.1942
P14075	Q8N5F7	1.02
P14075	Q9NSD9	0.63522
P14075	O14713	0.58143
P14075	P0CW23	0.35516
P14075	Q86YC2	0.37917
P14075	Q96LL4	0.94025
P14075	Q96KH6	0.55297
P14075	Q02083	0.26097
P14075	Q2NKJ3	0.336
P14075	Q53TS8	0.27223
P14075	Q14686	0.71482
P14075	P0C841	0.63821
P14075	Q0D2K3	0.78689
P14075	Q96AQ9	0.26592
P14075	Q9H7B2	0.686
P14075	Q96R06	0.64689
P14075	Q14493	0.60878
P14075	Q9BUT9	1.1254
P14075	O75582	0.24908
P14075	Q8IYW2	0.63821
P14075	Q9ULL0	0.56193
P14075	Q9UHX1	0.49221
P14075	P61088	0.48813
P14075	Q01201	0.72389
P14075	Q92674	0.16565
P14075	Q6IN84	0.23178
P14075	Q9UL46	0.25426
P14075	Q6ZS94	0.63821
P14075	Q5BKY6	0.63821
P14075	Q08E93	0.63821
P14075	Q01658	0.22066
P14075	P57678	0.66507
P14075	Q96GV9	0.28379
P14075	Q96N16	0.5701
P14075	O96002	0.63821
P14075	Q9BV57	0.36728
P14075	Q9Y6Z5	0.72764
P14075	P53672	0.70051
P14075	Q8IVE0	0.01114
P14075	Q8N715	0.40568
P14075	Q6L8H4	0.63821
P14075	O76087	0.45588
P14075	Q9BVN2	0.27902
P14075	O95391	0.17079
P14075	P80723	0.55155
P14075	Q5HY64	0.13457
P14075	Q8NAS9	0.15481
P14075	E5RJ46	0.63821
P14075	P51808	0.099736
P14075	Q8WVK7	0.38776

P14075	Q9H1A3	0.19916
P14075	O75444	0.24378
P14075	Q96MH2	0.94679
P14075	Q6ZU65	0.2882
P14075	Q2M3D2	0.16481
P14075	Q9Y4Z0	0.4705
P14075	P78406	0.31283
P14075	Q17RH7	0.63821
P14075	O94888	0.22759
P14075	Q70YC4	0.5725
P14075	Q5T8A7	0.7274
P14075	Q96IR3	0.70627
P14075	P26196	0.33622
P14075	Q8IVS8	0.037129
P14075	Q8IXQ3	0.28211
P14075	Q9H7R5	0.22707
P14075	Q3LI76	0.70941
P14075	Q53T94	0.24105
P14075	P57740	0.56936
P14075	Q9H116	0.15245
P14075	Q08AG7	0.51304
P14075	P17275	0.70702
P14075	Q9Y5N6	0.34658
P14075	Q9NVR2	0.66555
P14075	Q96KP1	0.22249
P14075	Q9Y4A8	0.31044
P14075	Q9H6J7	0.34852
P14075	Q00403	0.76117
P14075	P30281	0.53622
P14075	Q6ZYL4	0.78708
P14075	K7EIZ4	0.63821
P14075	P30279	0.44411
P14075	Q8WUX9	0.20428
P14075	Q7L2R6	0.27773
P14075	Q96C03	0.57578
P14075	Q96DM3	0.225
P14075	P82094	0.90424
P14075	Q9UET6	0.48052
P14075	A6NDZ8	0.030285
P14075	Q13901	0.85934
P14075	Q6ZSN7	0.42835
P14075	Q5EE01	0.54667
P14075	P43487	0.088624
P14075	Q8WUM4	0.76561
P14075	Q14C87	0.705
P14075	Q8WTY6	0.064004
P14075	Q15459	0.47234
P14075	Q9UHJ3	0.1228
P14075	P53675	0.17038
P14075	A4D997	0.82178
P14075	Q6ZRI6	0.62498
P14075	Q6P9B6	0.1668
P14075	Q96JE9	0.15104
P14075	Q8N7L0	0.26215
P14075	Q15388	0.14074
P14075	Q13425	0.201
P14075	P61572	0.589
P14075	P57086	0.33527
P14075	Q8TE85	0.093429
P14075	Q8WUE5	0.17571
P14075	Q12816	0.08609
P14075	Q5VZQ5	0.56438
P14075	Q96HW7	0.40543
P14075	Q71F56	0.24931
P14075	O75362	0.54868
P14075	P32189	0.28499
P14075	Q6UXR8	0.63821
P14075	Q9NVL8	0.2707
P14075	P55212	0.2848
P14075	Q6X4W1	0.98138
P14075	O75446	1.0442
P14075	Q06546	0.9726
P14075	P0C1Z6	0.8408
P14075	O94818	1.074
P14075	Q6NUN9	0.50812
P14075	Q5QP74	0.63821

P14075	Q6ZVF9	0.59739
P14075	P49720	0.26471
P14075	Q8TCZ7	0.47655
P14075	Q9UKT7	0.24312
P14075	P61254	0.51033
P14075	O60563	1.1739
P14075	A8MSI8	1.0107
P14075	Q8NAV2	0.53579
P14075	Q9Y252	0.27113
P14075	Q96DY7	0.3469
P14075	A4D1E9	0.776
P14075	Q3LI68	0.85226
P14075	O14776	0.8463
P14075	Q9Y2T2	0.35672
P14075	Q9Y3Y4	0.37452
P14075	Q5VTH2	0.49405
P14075	P43268	0.23554
P14075	Q5VT40	0.70918
P14075	Q9NVV4	0.41596
P14075	Q9P2R6	0.81949
P14075	Q9Y5B0	0.33773
P14075	Q6P4D5	0.90484
P14075	P86479	0.63821
P14075	Q8WWR8	0.07912
P14075	Q86XI2	0.54195
P14075	Q8WYP5	0.47295
P14075	P46778	0.38311
P14075	P29083	0.88411
P14075	Q86SQ7	0.61546
P14075	Q96LV5	0.09127
P14075	O75971	1.1439
P14075	A8MVW5	0.45516
P14075	Q6UN15	0.90539
P14075	P23769	0.26778
P14075	P08133	0.41841
P14075	Q9NRL2	0.74014
P14075	Q9UL16	0.25079
P14075	P61244	0.65792
P14075	P49459	0.16118
P14075	Q9UKL0	0.89777
P14075	Q9NSI6	0.6037
P14075	Q9UID6	0.32141
P14075	Q8N365	0.94025
P14075	P50613	0.15891
P14075	Q8WW12	0.74465
P14075	Q13287	0.35088
P14075	Q9UBF2	0.10613
P14075	Q6ZV56	0.63821
P14075	Q9Y2G7	0.33777
P14075	P59923	0.2118
P14075	Q86TS7	0.63821
P14075	P61604	0.55836
P14075	Q99457	0.34187
P14075	Q9BRQ3	0.83225
P14075	Q8IUW3	0.552
P14075	Q9NX18	0.70599
P14075	Q16822	0.16326
P14075	Q96T58	0.51242
P14075	Q5T750	0.30695
P14075	P59037	0.65279
P14075	Q6NUQ1	0.35214
P14075	Q2KJ03	0.17658
P14075	O75394	0.50018
P14075	Q495Y8	0.53769
P14075	Q8NHV4	0.39395
P14075	Q6PH81	0.55787
P14075	P41218	0.30049
P14075	Q7Z460	0.24206
P14075	Q9P1D8	0.63821
P14075	Q68D10	0.94025
P14075	Q8IUC1	0.31219
P14075	Q9BX26	0.88482
P14075	Q8N2B8	0.22782
P14075	P19419	0.99837
P14075	H3BMG3	0.4407
P14075	Q3ZCQ2	0.63821

P14075	Q5H9F3	0.37218
P14075	P41212	0.58689
P14075	Q9Y312	0.26928
P14075	Q9H467	0.72315
P14075	Q9NSB4	0.48911
P14075	P26651	0.44287
P14075	Q8IV36	0.27729
P14075	Q12905	0.83992
P14075	Q8TCE6	0.99766
P14075	Q14241	0.28948
P14075	Q9H446	0.1668
P14075	Q9GZL7	0.51425
P14075	Q92600	0.85246
P14075	Q52LA3	0.55313
P14075	Q6P9A1	0.24912
P14075	Q6P1M3	0.65729
P14075	Q9H019	0.81172
P14075	Q9Y6Z4	0.16405
P14075	P18847	0.94154
P14075	Q8WVC0	0.75365
P14075	P78559	0.3121
P14075	Q01860	0.50318
P14075	Q9BYU5	0.43615
P14075	Q9UPP1	0.31544
P14075	Q9UBD5	0.32816
P14075	Q9BRX2	0.18454
P14075	H3BN30	0.63821
P14075	P36639	0.11394
P14075	A1A5D9	0.68819
P14075	Q9Y6F6	0.24907
P14075	Q5H9J7	0.6992
P14075	Q6ZW49	0.2232
P14075	Q9HCM7	0.69266
P14075	Q14139	0.38268
P14075	Q86TU7	0.12512
P14075	P28698	0.4992
P14075	Q5T7N7	0.63821
P14075	Q9ULU4	0.30061
P14075	Q6PI26	0.77654
P14075	Q96BR5	0.025572
P14075	Q6ZSJ8	0.94025
P14075	Q9NY61	0.61425
P14075	Q96G28	0.19421
P14075	Q8NCX0	0.28286
P14075	O95177	0.19524
P14075	Q9Y468	0.71776
P14075	Q9NS64	0.7393
P14075	Q9UI25	0.63821
P14075	Q96M34	0.15487
P14075	P61201	0.34381
P14075	Q8N782	0.2846
P14075	P09234	0.35616
P14075	Q9BQ15	0.55694
P14075	P38159	0.47278
P14075	Q9H7R0	0.26082
P14075	P13985	0.63821
P14075	O14598	0.89699
P14075	Q8TAA5	0.39522
P14075	Q3LHN1	0.63821
P14075	Q9NX46	0.31407
P14075	Q75MW2	0.49693
P14075	P38935	0.31016
P14075	Q6ZSR9	0.023467
P14075	Q9HBX3	0.63821
P14075	Q96SL8	0.080621
P14075	P60510	0.55807
P14075	Q86UW6	0.12834
P14075	Q96M91	0.92896
P14075	Q6ICG8	1.0799
P14075	A5PLN9	0.65916
P14075	O95886	0.70937
P14075	Q6ZQR2	0.80198
P14075	Q96GU1	0.35213
P14075	Q9UGP4	0.31554
P14075	O15037	0.36462
P14075	Q14839	0.36782

P14075	Q6ZS92	0.25823
P14075	Q9UBD0	0.85108
P14075	Q8N6L1	0.62109
P14075	Q03933	0.49752
P14075	A8K554	0.051629
P14075	Q9BX40	0.59986
P14075	Q9HC44	1.2608
P14075	Q9UHT9	0.63821
P14075	Q9NPI8	0.61201
P14075	Q6NSH3	0.49813
P14075	P48380	0.33335
P14075	P34949	0.35538
P14075	Q5TAB7	0.58995
P14075	Q9BQD3	0.18389
P14075	Q9UH64	0.21723
P14075	P59090	0.63821
P14075	P42768	0.12831
P14075	Q96KT6	0.63821
P14075	Q8IZM0	0.33898
P14075	Q92945	0.52477
P14075	Q53GS7	0.41936
P14075	Q5GJ75	0.0031367
P14075	Q5TG08	0.83777
P14075	P51610	0.30974
P14075	P62633	0.31576
P14075	Q13561	0.44947
P14075	Q9Y2Y0	0.73213
P14075	Q33E94	0.61509
P14075	Q86SZ2	0.49499
P14075	Q5JTJ3	0.16981
P14075	O15527	0.2933
P14075	Q14153	0.73401
P14075	Q9BSU3	0.24483
P14075	Q9HBT7	0.18018
P14075	Q92973	0.78332
P14075	Q8IY31	0.29249
P14075	Q6ZU52	0.22045
P14075	Q15652	0.2875
P14075	O75419	0.65396
P14075	Q9NTI7	0.90131
P14075	Q93100	0.30788
P14075	Q86WB0	0.99021
P14075	O15015	0.39205
P14075	P35548	0.45448
P14075	P20700	0.19332
P14075	Q96C28	0.27983
P14075	P11161	0.31021
P14075	Q969H9	0.63821
P14075	Q8NC51	0.48148
P14075	Q12837	0.33374
P14075	Q15561	0.69234
P14075	Q6ZQV5	0.2118
P14075	H0UI37	0.25405
P14075	Q7Z3B3	0.32225
P14075	Q9NYP8	0.44152
P14075	Q5VV17	0.064158
P14075	P61573	0.589
P14075	Q9NTX9	0.38163
P14075	Q9NVN8	0.17744
P14075	Q53HV7	0.35759
P14075	Q6DCA0	0.17539
P14075	P15172	0.2416
P14075	A4D2B0	0.63106
P14075	Q15428	0.39384
P14075	Q6PGP7	0.48277
P14075	Q96ND8	0.3005
P14075	Q6P1X5	0.65017
P14075	O00541	0.29851
P14075	P49792	0.63219
P14075	Q5VXH4	0.2982
P14075	Q969T9	0.87844
P14075	Q9Y2D8	0.38331
P14075	P17096	0.37621
P14075	Q92783	0.34279
P14075	Q49A88	0.55434
P14075	Q15327	0.2371

P14075	Q9NWN3	0.29485
P14075	Q9Y606	0.76998
P14075	Q9Y4P1	0.35818
P14075	P56524	0.21228
P14075	Q96MH7	0.059526
P14075	Q9H8V8	0.63821
P14075	Q96PU4	0.24131
P14075	P05813	0.31048
P14075	Q5JT18	0.63821
P14075	A5PLN7	0.94025
P14075	Q00994	0.75094
P14075	Q2M329	0.39103
P14075	O60813	0.28377
P14075	Q9BV20	0.28082
P14075	Q9UK11	0.23581
P14075	Q3LI60	0.63821
P14075	Q8N1F7	0.39819
P14075	Q9NTM9	0.027251
P14075	Q96HQ2	0.92508
P14075	O43918	0.74097
P14075	Q9NP79	0.015904
P14075	P17036	0.24165
P14075	Q92917	0.66601
P14075	Q99747	0.31774
P14075	O00422	0.97042
P14075	P51965	0.30957
P14075	Q01844	0.41741
P14075	Q96IZ7	0.9523
P14075	A8K8P3	0.49059
P14075	O14709	0.2118
P14075	Q8NDZ9	0.63821
P14075	P05388	0.9333
P14075	Q96IY1	0.64831
P14075	Q08050	0.32541
P14075	Q9NSB2	0.62301
P14075	Q15714	0.60287
P14075	Q49AJ0	0.25585
P14075	Q13257	0.50877
P14075	Q53G59	0.36399
P14075	Q9H9Z2	0.53732
P14075	H3BQW9	0.6611
P14075	Q5T681	0.83544
P14075	Q8TF01	1.0059
P14075	O43186	0.57798
P14075	Q68CZ6	0.32838
P14075	P0CW21	0.63821
P14075	Q5PSV4	0.16676
P14075	P18146	0.50432
P14075	Q13015	0.55637
P14075	O60930	0.11949
P14075	Q13442	0.57373
P14075	Q2VPJ9	0.94025
P14075	P41252	0.46193
P14075	Q5VWN6	0.19175
P14075	Q9UJW3	0.40918
P14075	Q9BXR0	0.94409
P14075	Q9NYW8	0.12763
P14075	Q00534	0.46937
P14075	A8K5M9	0.81159
P14075	Q9HC77	0.45995
P14075	P19484	0.31253
P14075	Q9NRP2	0.75196
P14075	Q9HCE1	0.49809
P14075	Q9NQG5	0.82645
P14075	P26641	0.22842
P14075	Q9NRT5	0.40716
P14075	P78344	0.21212
P14075	Q8IZQ1	0.38418
P14075	Q8NE18	1.0009
P14075	A6NN90	0.38936
P14075	O95257	0.2591
P14075	P18669	0.55532
P14075	Q96EZ8	0.44529
P14075	Q9Y3D3	0.58858
P14075	P0C7T5	0.43125
P14075	Q8N159	0.23016

P14075	Q9H761	0.29335
P14075	Q15047	0.45186
P14075	Q7L4I2	1.2359
P14075	O75312	0.28294
P14075	P02810	0.63821
P14075	Q16384	0.39489
P14075	Q9P1T7	0.98115
P14075	O60675	0.22573
P14075	Q9BZK3	0.24811
P14075	O95521	0.36986
P14075	O95625	0.067017
P14075	A6NER3	0.079883
P14075	P06746	0.24382
P14075	Q96TC7	0.63083
P14075	A1L429	0.45588
P14075	Q99576	0.29258
P14075	Q14320	0.21912
P14075	P29084	0.94701
P14075	Q86VX9	0.72338
P14075	Q96T21	0.5623
P14075	Q969U7	0.37862
P14075	P80217	0.36639
P14075	Q8N3U1	0.63821
P14075	O95619	0.59117
P14075	A1L190	0.81328
P14075	Q96RK0	0.68205
P14075	Q16520	0.70259
P14075	O95931	0.57698
P14075	Q9NZ52	0.25406
P14075	Q3LI73	0.78906
P14075	Q6ECI4	0.19334
P14075	Q9Y6J3	0.63821
P14075	Q6IA86	0.35266
P14075	Q86VE0	0.95874
P14075	Q70EL1	0.30278
P14075	Q86X02	0.1853
P14075	Q9HD33	0.69348
P14075	Q9Y2N7	0.32444
P14075	Q8N336	0.31174
P14075	Q8TE76	0.078671
P14075	P0CF75	0.19965
P14075	O43790	0.70038
P14075	P42771	0.41785
P14075	P41220	0.25232
P14075	Q6ZRM9	0.66314
P14075	Q5T5A8	0.63821
P14075	Q7LBC6	0.021314
P14075	B2RV13	0.55755
P14075	P20265	0.49382
P14075	Q9UBQ0	0.32751
P14075	P33076	0.33116
P14075	Q5TZK3	0.040278
P14075	Q9HCH0	0.98719
P14075	Q5T870	0.72916
P14075	Q6RSH7	0.22583
P14075	Q8WWX9	0.19414
P14075	F7VJQ1	0.63821
P14075	P0C2W7	0.63821
P14075	Q9UH92	0.61766
P14075	Q9BW61	0.56727
P14075	Q53H80	0.30627
P14075	P83881	0.49283
P14075	P17661	0.28304
P14075	P04156	0.31096
P14075	Q5VV11	0.16221
P14075	Q96LI9	0.43263
P14075	O00629	0.26492
P14075	Q9NPJ8	0.2275
P14075	Q5UE93	0.42549
P14075	Q8TDC0	0.43925
P14075	Q86WS4	0.63821
P14075	O14734	0.41379
P14075	P82650	0.13041
P14075	Q9H374	0.29603
P14075	Q16613	0.19918
P14075	Q92922	0.89023

P14075	P54920	0.2117
P14075	Q16537	0.12467
P14075	Q8WTX7	0.69586
P14075	O15226	0.58315
P14075	Q8N268	0.058695
P14075	Q86VN1	0.23581
P14075	Q8NDN9	0.41146
P14075	Q676U5	0.42455
P14075	Q9BWK5	0.98003
P14075	P14136	0.60184
P14075	A6NME0	0.34001
P14075	Q9BYG4	0.43699
P14075	Q13620	0.52852
P14075	Q99613	0.44281
P14075	P19388	0.4328
P14075	Q3MIS6	0.29291
P14075	O75363	0.41785
P14075	Q7KZ85	0.27839
P14075	Q92870	0.47417
P14075	Q9H321	0.89699
P14075	Q9Y603	0.94184
P14075	Q9HCH5	0.34293
P14075	Q13515	0.90592
P14075	O95400	0.52621
P14075	P49750	0.88422
P14075	P48634	0.55947
P14075	Q9BZK8	0.63821
P14075	Q9P086	0.80648
P14075	O94776	0.76878
P14075	B2CW77	0.63821
P14075	Q6MZT1	0.68144
P14075	Q01130	0.55543
P14075	O75925	0.38281
P14075	Q5TBB1	0.74061
P14075	Q5VZL5	0.70403
P14075	Q9BRQ4	0.32208
P14075	Q86V42	0.74242
P14075	Q9BXS6	0.56934
P14075	O60232	0.81451
P14075	Q9HAH1	0.24135
P14075	Q6ZRH4	1.0495
P14075	Q9Y244	0.23153
P14075	P10244	0.35059
P14075	Q96MY1	0.79149
P14075	Q8N0V1	0.39925
P14075	P61218	0.1659
P14075	Q5T753	0.63821
P14075	Q5HYK7	0.81116
P14075	Q9NQR1	0.50973
P14075	Q6L8G9	0.63821
P14075	Q6ZRQ5	0.33602
P14075	A6NMK8	0.67019
P14075	Q6ZN03	0.21077
P14075	Q5DJT8	0.15736
P14075	Q9Y2V2	0.27962
P14075	Q09MP3	0.57095
P14075	P17019	0.2938
P14075	Q8N128	0.63821
P14075	P0C5Y4	0.78379
P14075	Q96RK1	0.39475
P14075	Q6L8H1	0.63821
P14075	P35638	0.57906
P14075	Q9Y3F1	0.63821
P14075	Q9NP50	0.8627
P14075	Q719H9	0.44577
P14075	Q8TDY2	0.63031
P14075	P13073	0.30766
P14075	Q96PY0	0.28748
P14075	P50750	0.36746
P14075	P15822	0.59407
P14075	Q8IYX7	0.95729
P14075	Q15070	0.23197
P14075	Q8TD35	0.94025
P14075	Q16548	0.34185
P14075	O15117	0.6328
P14075	P19367	0.1965

24929	P14075	P41236	0.75956
24930	P14075	Q5VZV4	0.56584
24931	P14075	Q6AWC8	0.73023
24932	P14075	Q49AR2	0.63362
24933	P14075	A6NL46	0.63038
24934	P14075	O75821	0.35351
24935	P14075	O95218	0.41487
24936	P14075	A7E2U8	0.35674
24937	P14075	Q9Y4K1	0.65175
24938	P14075	O95777	0.41529
24939	P14075	Q7Z7F1	0.0074033
24940	P14075	O60583	0.75127
24941	P14075	P42568	0.64816
24942	P14075	Q9UIF7	0.13311
24943	P14075	Q92994	0.15885
24944	P14075	Q9UN81	0.38778
24945	P14075	H3BNL1	0.40897
24946	P14075	Q7L5Y9	0.19304
24947	P14075	Q53EQ6	0.37896
24948	P14075	Q9Y2Z2	0.86548
24949	P14075	Q96KT0	0.27476
24950	P14075	P31249	0.34283
24951	P14075	Q9Y2A0	0.42494
24952	P14075	Q9BT04	0.36123
24953	P14075	Q96FN9	0.48553
24954	P14075	A8MUI8	0.43821
24955	P14075	Q9UNP9	0.27182
24956	P14075	A8MT65	0.25328
24957	P14075	Q6ISB3	0.3936
24958	P14075	O00287	0.61425
24959	P14075	Q8TEA8	0.81979
24960	P14075	Q96NA2	0.78891
24961	P14075	P47928	0.30232
24962	P14075	O95363	0.44767
24963	P14075	Q5T653	0.57634
24964	P14075	P46777	0.90651
24965	P14075	Q9H840	0.62438
24966	P14075	O95997	0.68588
24967	P14075	Q9Y250	0.7391
24968	P14075	Q7Z4G1	1.1566
24969	P14075	P0C7W0	0.94025
24970	P14075	Q8N9Z2	0.84554
24971	P14075	Q02080	0.22249
24972	P14075	O60671	0.36574
24973	P14075	A6PW82	0.47064
24974	P14075	Q8NA69	0.27242
24975	P14075	Q9GZQ3	0.85723
24976	P14075	Q9ULX9	0.25153
24977	P14075	O75170	0.59982
24978	P14075	Q6ZS72	0.48817
24979	P14075	Q86VQ0	0.1928
24980	P14075	Q8WZA8	0.63821
24981	P14075	P22732	0.47499
24982	P14075	Q96PS1	0.6406
24983	P14075	Q5T440	0.27295
24984	P14075	Q96H22	0.51352
24985	P14075	Q96EF9	0.51137
24986	P14075	Q96G03	0.50128
24987	P14075	Q9NX53	0.1668
24988	P14075	Q5SRH9	0.8321
24989	P14075	O43172	0.51544
24990	P14075	Q5H9J9	0.4382
24991	P14075	Q6P1L8	0.7383
24992	P14075	P19012	0.80046
24993	P14075	Q13510	0.54162
24994	P14075	Q9NQ31	0.47065
24995	P14075	Q96QA5	0.39977
24996	P14075	Q1T7F1	0.20116
24997	P14075	Q93062	0.64132
24998	P14075	Q8TF40	0.5836
24999	P14075	Q9H825	0.14495
25000	P14075	Q15910	0.32146
25001	P14075	Q5JS54	0.71048
25002	P14075	Q6NV74	0.94025
25003	P14075	Q96IT1	0.44189
25004	P14075	Q9HB03	0.40775

P14075	Q86TA1	0.14228
P14075	P11844	0.70051
P14075	Q9UKX7	0.81795
P14075	P29692	0.15807
P14075	Q96EY4	0.50916
P14075	Q9HD26	0.68559
P14075	O95433	0.32774
P14075	O15453	0.44013
P14075	O43684	0.47946
P14075	O60296	0.38217
P14075	Q9GZV4	0.41834
P14075	P57076	0.4436
P14075	Q96A04	0.73048
P14075	Q9BTP7	0.63438
P14075	A6NL82	0.64102
P14075	Q4W4Y0	0.2985
P14075	P0CL81	0.45588
P14075	Q9Y3U8	0.57095
P14075	Q9BVG4	0.32131
P14075	P04792	0.25246
P14075	Q53ET0	1.1714
P14075	Q96SN8	0.24433
P14075	Q9NVF7	0.8608
P14075	Q96LM1	0.61964
P14075	P04280	0.70558
P14075	Q6UVJ0	0.57357
P14075	Q8N1Q8	0.44137
P14075	P56182	0.53883
P14075	P02728	0.63821
P14075	P78347	0.45062
P14075	Q92988	0.38762
P14075	O43167	0.39205
P14075	Q8N9H6	0.28265
P14075	Q9NUQ6	0.11945
P14075	P31943	0.35146
P14075	P40926	0.17722
P14075	Q8NEP4	0.94025
P14075	Q5SWA1	0.46203
P14075	P62249	0.47832
P14075	Q8IVG9	0.45022
P14075	Q9BYQ8	0.78356
P14075	Q9BZG8	0.17374
P14075	Q8TF09	0.41524
P14075	Q8NHV5	0.15877
P14075	Q9H7E9	0.94025
P14075	P63162	0.419
P14075	Q7Z7C8	0.32582
P14075	Q92890	0.47291
P14075	O14933	0.13004
P14075	P59051	0.37169
P14075	Q9BYP9	0.78356
P14075	Q16082	0.60379
P14075	A6NLX3	0.32941
P14075	Q96CT7	0.15766
P14075	Q12906	0.85928
P14075	Q92800	0.34835
P14075	Q13216	0.24422
P14075	P23490	0.56784
P14075	Q9H2W6	0.3418
P14075	Q8WUY3	0.11724
P14075	Q99543	0.25247
P14075	Q75NE6	1.0062
P14075	Q13601	0.17716
P14075	O15397	0.59793
P14075	Q86UF4	0.21375
P14075	Q5VUE5	0.37558
P14075	A6NFL8	0.71412
P14075	Q9H7N4	0.71076
P14075	Q9P242	0.6106
P14075	Q8WY50	0.63821
P14075	Q86U90	0.50384
P14075	P39687	1.0796
P14075	O75175	1.0079
P14075	Q6PK04	0.80319
P14075	Q05DH4	0.95406
P14075	Q5TAX3	0.48697

P14075	Q8NCN5	0.074286
P14075	Q9H074	0.23874
P14075	O00625	0.40069
P14075	Q5SXM2	0.42766
P14075	Q9NYK6	0.54402
P14075	Q8N5N7	0.65854
P14075	Q9BSJ6	0.33335
P14075	Q15544	0.86031
P14075	Q9H091	0.2858
P14075	P49005	0.34268
P14075	Q8WUF5	0.61688
P14075	Q7Z614	0.25113
P14075	Q3LI58	0.63821
P14075	F8W1W9	0.20092
P14075	P78371	0.33257
P14075	Q6P444	0.81172
P14075	Q8IUD2	0.60791
P14075	P45973	0.48918
P14075	O95159	0.40377
P14075	Q93075	0.57894
P14075	Q9HAW0	0.24595
P14075	Q02040	0.5432
P14075	Q86UB2	0.72124
P14075	Q9NNX9	0.96999
P14075	Q6IPT2	1.1182
P14075	Q53G26	0.35989
P14075	Q9UK41	0.447
P14075	P0C853	0.63821
P14075	Q9UFW8	0.155
P14075	O15027	0.42474
P14075	Q0VD67	0.019594
P14075	Q14157	0.64801
P14075	Q8TAF7	0.28409
P14075	C9J3I9	0.45142
P14075	P0DL12	0.40859
P14075	Q99717	0.25517
P14075	Q8N769	0.084911
P14075	Q9P2X3	0.18986
P14075	Q13200	0.34505
P14075	P13645	0.77038
P14075	P08397	0.20355
P14075	O75448	0.34135
P14075	P12035	0.54544
P14075	Q5VWP3	0.91018
P14075	Q9UJY1	0.30769
P14075	Q9BXU2	0.27696
P14075	Q96BU1	0.48442
P14075	A8MXV4	0.47666
P14075	F5H4B4	0.83777
P14075	Q9HA58	0.63821
P14075	Q5TZA2	0.52358
P14075	A0JNW5	0.57632
P14075	P0DJH9	0.022662
P14075	P48556	0.24186
P14075	A1A580	0.78906
P14075	P19387	0.32256
P14075	Q5H9R7	0.8243
P14075	P06730	0.34871
P14075	Q2KHT3	0.90422
P14075	Q9NX05	0.35454
P14075	Q12798	0.46103
P14075	P01009	0.13314
P14075	P62140	0.56315
P14075	Q8N5M1	0.29228
P14075	O00167	0.37813
P14075	Q9Y4A5	0.62155
P14075	Q2NL98	0.8181
P14075	Q9UKV0	0.29475
P14075	A2AJT9	0.4283
P14075	O00716	0.87253
P14075	P62306	0.16265
P14075	Q9ULE4	0.28008
P14075	P56693	0.48567
P14075	Q8IY81	0.52227
P14075	Q9H9C1	0.43862
P14075	Q9H040	0.2357

P14075	A3KN83	0.86576
P14075	Q16401	0.53057
P14075	Q9NPI6	0.65599
P14075	P62081	0.75519
P14075	Q9NQR7	0.63821
P14075	P86481	0.63821
P14075	Q96FW1	0.12174
P14075	Q5TBC7	0.15381
P14075	O75494	0.48129
P14075	Q7Z6J8	0.54735
P14075	Q96JB5	0.20395
P14075	Q9UK61	0.62266
P14075	Q86VI4	0.42335
P14075	Q7RTU5	0.28657
P14075	Q9BW66	0.77701
P14075	Q00978	0.67184
P14075	Q8WW38	0.3599
P14075	Q8N668	0.5618
P14075	Q12980	0.83634
P14075	P0CW24	0.1745
P14075	Q96DY2	0.15212
P14075	Q8IX12	0.80164
P14075	P20226	0.71928
P14075	Q5TCQ9	0.37905
P14075	Q9BT49	0.55001
P14075	P15336	0.67994
P14075	P04198	0.61684
P14075	O95045	0.25968
P14075	Q15629	0.30277
P14075	Q9NRX3	0.74879
P14075	Q9H9J2	0.21221
P14075	Q13111	1.0393
P14075	A6NMN3	0.40888
P14075	Q9GZY8	0.23913
P14075	Q9UHV2	1.3021
P14075	Q15653	0.6727
P14075	Q8NEU8	0.25909
P14075	P63241	0.30624
P14075	Q69YL0	0.63821
P14075	Q96BQ5	0.59938
P14075	Q8N2W9	0.80963
P14075	Q8NHQ1	0.92796
P14075	Q9Y2X8	0.19221
P14075	O15061	0.54102
P14075	O14929	0.52616
P14075	A0AUZ9	0.4549
P14075	Q9NRI7	0.63821
P14075	Q96AQ1	0.72258
P14075	P48595	0.1105
P14075	Q9UL58	0.40635
P14075	Q9NXR7	0.19519
P14075	P62308	0.521
P14075	P15559	0.066893
P14075	Q8IYB1	0.30464
P14075	O43734	0.94025
P14075	Q5T1C6	0.53004
P14075	P62750	0.61236
P14075	Q3KP22	0.31313
P14075	A6NCN2	0.65232
P14075	O15126	0.1059
P14075	A6XGL0	0.33219
P14075	O00567	0.58869
P14075	P18621	0.3873
P14075	A8MZG2	0.94025
P14075	Q9UBB5	0.8746
P14075	Q5VVH5	0.35212
P14075	Q8WWY6	0.19547
P14075	Q92754	0.39189
P14075	Q9ULQ0	0.88258
P14075	Q9UHB4	0.39729
P14075	P13489	0.26715
P14075	Q9NZI7	0.1437
P14075	Q96LI5	0.36623
P14075	Q8IU60	0.61306
P14075	P15927	0.25179
P14075	Q5SQS8	0.49119

P14075	Q6ZTR5	0.44227
P14075	Q96BD8	0.39487
P14075	Q969Q0	0.12743
P14075	Q9NZD8	0.38826
P14075	Q9Y613	0.31796
P14075	Q96BN6	0.44125
P14075	Q9UI95	0.96469
P14075	Q68DN1	0.64837
P14075	Q15560	0.77498
P14075	Q6NUI1	0.50348
P14075	P35658	0.811
P14075	Q6ZU35	0.94025
P14075	A6NKU9	0.53769
P14075	Q9Y3D5	0.55505
P14075	Q86TL2	0.68398
P14075	Q9NZN8	0.49364
P14075	Q9NWS8	0.4565
P14075	P0C5K6	0.63821
P14075	P63279	0.60239
P14075	P31040	0.48166
P14075	Q9BYA6	0.63821
P14075	Q6PIJ6	0.81808
P14075	O96020	0.6921
P14075	A6NJ88	0.7048
P14075	Q9BRR0	0.14156
P14075	Q9Y6D6	0.37276
P14075	P10242	0.71564
P14075	Q16666	0.44428
P14075	P12270	0.47328
P14075	O43390	0.46611
P14075	Q9H6Y2	0.30057
P14075	O94983	1.002
P14075	O94903	0.38452
P14075	Q15796	0.45037
P14075	Q96BN2	0.72756
P14075	Q9NYJ1	0.94346
P14075	Q9NZY2	0.63821
P14075	Q01105	0.53308
P14075	P58505	0.46631
P14075	O95801	0.13564
P14075	Q02543	0.49535
P14075	Q08AN1	0.2118
P14075	Q96N95	0.086077
P14075	Q9UJG1	0.67678
P14075	Q53HC0	0.99151
P14075	P0DM63	0.67697
P14075	Q9UI10	0.19153
P14075	Q5MJ68	0.30571
P14075	A8MVA2	0.78356
P14075	P46976	0.28792
P14075	Q9Y3I1	0.60115
P14075	Q9NV44	0.24324
P14075	Q86W67	0.035852
P14075	Q5T0D9	0.45056
P14075	P78385	0.67347
P14075	Q9BYQ3	0.78356
P14075	Q9NWA0	1.2517
P14075	Q6P5X5	0.60136
P14075	Q9Y2J8	0.60448
P14075	Q5TYM5	0.35444
P14075	Q9NQ87	0.31389
P14075	Q7Z6I5	0.62644
P14075	Q8WWC4	0.64556
P14075	Q9HAV4	0.88809
P14075	Q9UMY1	1.0264
P14075	Q86UD1	0.37734
P14075	Q96KF2	0.63821
P14075	Q9H2U6	0.35033
P14075	Q6NXR4	0.36683
P14075	P0CH99	0.45305
P14075	Q96A00	0.45019
P14075	Q9UDY4	0.56219
P14075	Q8IVF1	0.71985
P14075	P63172	0.56221
P14075	P54840	0.32613
P14075	Q6NSW5	0.90658

P14075	Q8WWH5	0.5865
P14075	Q8NHY6	0.22775
P14075	P08238	0.44916
P14075	Q6ZVK8	0.16893
P14075	O15304	0.68178
P14075	Q03936	0.27718
P14075	P28070	0.28158
P14075	Q9BYC9	0.58789
P14075	Q5JY77	0.57349
P14075	A8MVP8	0.061133
P14075	Q8TBK2	0.35382
P14075	Q9NZB2	0.54362
P14075	Q7Z5W3	0.59114
P14075	A6NF01	0.71739
P14075	Q9UHA2	0.21084
P14075	Q9UQ35	0.64831
P14075	Q86V87	0.36967
P14075	O43920	0.54823
P14075	Q13148	0.42776
P14075	A8MXK9	0.48682
P14075	Q15010	0.074578
P14075	Q9P2X7	0.63821
P14075	Q9BXD5	0.48959
P14075	Q96ST8	0.73612
P14075	E7EW31	0.63821
P14075	P15923	0.42348
P14075	Q6ZMV8	0.31444
P14075	P63313	0.49457
P14075	Q9UD71	0.73731
P14075	O75794	0.8294
P14075	P18124	0.36949
P14075	Q9UNX3	0.65058
P14075	Q66PJ3	0.5778
P14075	Q96AP0	0.10495
P14075	Q9BYJ9	0.56264
P14075	P15408	0.38203
P14075	O94906	0.19898
P14075	Q5JST6	0.66876
P14075	P53992	0.65956
P14075	O60294	0.21847
P14075	Q8N319	1.1383
P14075	P20073	0.19718
P14075	P63302	0.4331
P14075	P25398	0.45256
P14075	P58004	0.46741
P14075	Q9HCG8	0.11136
P14075	A6NCI8	0.76197
P14075	P0CG43	0.63821
P14075	Q96LC9	0.47654
P14075	P50402	0.34092
P14075	Q9BT92	0.33446
P14075	Q14691	0.2155
P14075	Q4V328	0.84195
P14075	Q6PJG6	0.57558
P14075	Q13398	0.2886
P14075	Q9Y6H1	0.87053
P14075	Q53GI3	0.14795
P14075	Q86W11	0.12192
P14075	Q96JI7	0.3865
P14075	Q9Y3E5	0.38545
P14075	Q6NSI8	0.44121
P14075	Q9BQ48	0.81752
P14075	Q5BJE1	0.32108
P14075	O75901	0.41442
P14075	Q8N7Q3	0.2122
P14075	Q9NZ63	0.49071
P14075	A8MTB1	0.63821
P14075	Q6NXS1	0.87443
P14075	Q5JTD0	0.93278
P14075	Q8NEG0	0.28028
P14075	Q9P016	0.63632
P14075	Q9H2G4	0.48519
P14075	Q9NYF8	0.89517
P14075	Q14585	0.14625
P14075	Q7Z3K6	0.29481
P14075	Q6ZSR6	0.18567

P14075	Q9UBB4	0.72646
P14075	Q12872	0.62151
P14075	Q5JVX7	0.67543
P14075	Q86YW9	0.22989
P14075	Q13057	0.2898
P14075	Q14209	0.86926
P14075	Q8IY63	0.26129
P14075	Q9NVU7	0.58688
P14075	Q8IYI0	0.37634
P14075	Q13952	0.50221
P14075	P25963	0.43081
P14075	Q9ULJ3	0.39559
P14075	Q17RY6	0.57247
P14075	C9J302	0.64724
P14075	Q9BYE7	0.56066
P14075	Q96CB9	0.48307
P14075	Q9BRU9	0.36436
P14075	O43365	0.28091
P14075	O75306	0.34276
P14075	O14777	0.38318
P14075	Q9NWD9	0.35916
P14075	Q9NZP6	0.71739
P14075	Q92621	0.81266
P14075	P43358	0.39464
P14075	P61981	0.30327
P14075	A8MYX2	0.017615
P14075	O75182	0.89553
P14075	P38646	0.40891
P14075	Q13547	0.5713
P14075	A6NNV3	0.53769
P14075	Q9BXV9	0.63821
P14075	A6NHX0	0.76395
P14075	Q9UJX2	0.40006
P14075	I3L1I5	0.32393
P14075	Q9ULR0	0.7354
P14075	Q5VVS0	0.73697
P14075	Q6K0P9	0.1006
P14075	Q9BT25	0.40911
P14075	Q6W349	0.64769
P14075	Q04323	0.17216
P14075	P61077	0.33202
P14075	Q13642	0.26638
P14075	Q5TBE3	0.27693
P14075	A1L168	0.94025
P14075	Q99856	0.40423
P14075	O00570	0.63291
P14075	Q9BXY0	0.099562
P14075	Q496A3	0.40546
P14075	Q05195	0.35209
P14075	Q16594	0.40955
P14075	Q8IV33	0.69551
P14075	P54198	0.86027
P14075	O94763	0.60294
P14075	P55854	0.32737
P14075	P49721	0.27455
P14075	Q96GW9	0.40413
P14075	A8MVM7	0.63821
P14075	Q8TCH9	0.43328
P14075	O95571	0.30626
P14075	Q96GE4	0.16524
P14075	Q8N9G6	0.55134
P14075	P26358	0.26352
P14075	Q5W1A9	0.17547
P14075	Q9BYQ9	0.78356
P14075	Q8NEZ2	0.3987
P14075	O15511	0.045069
P14075	Q6SJ96	0.094341
P14075	P07195	0.32299
P14075	Q9Y5M6	0.63821
P14075	Q9H0A0	0.48909
P14075	Q8N8H1	0.26226
P14075	A6NF36	0.53493
P14075	Q9BQI9	0.63787
P14075	Q6PEX3	0.45447
P14075	Q8IYN2	0.9329
P14075	Q86Y46	0.39857

25461	P14075	Q6A163	0.70708
25462	P14075	Q96AQ2	0.76323
25463	P14075	Q9UQK1	0.20881
25464	P14075	Q8NA31	0.35313
25465	P14075	A2RUB6	0.11425
25466	P14075	Q9BZE1	0.5173
25467	P14075	P20962	0.62036
25468	P14075	A8MZF0	0.94025
25469	P14075	Q13084	0.74772
25470	P14075	Q8N0Z2	0.13252
25471	P14075	Q96IZ0	0.52334
25472	P14075	P13056	0.46703
25473	P14075	Q9Y2L1	0.3447
25474	P14075	Q8ND90	0.49786
25475	P14075	Q8IVD9	0.38573
25476	P14075	O75638	1.2136
25477	P14075	P55735	0.35851
25478	P14075	P01106	0.31329
25479	P14075	Q6PCE3	0.44801
25480	P14075	Q8N8E1	0.49045
25481	P14075	Q9UBI9	0.73677
25482	P14075	O14561	0.28348
25483	P14075	Q92750	0.44127
25484	P14075	Q96EL1	0.90131
25485	P14075	Q8IWT0	0.14797
25486	P14075	P39880	0.37897
25487	P14075	Q9NSI2	0.94025
25488	P14075	Q9BR46	0.69628
25489	P14075	Q8WVJ9	0.48493
25490	P14075	Q86XI8	0.37331
25491	P14075	P43359	0.64711
25492	P14075	Q9BYI3	0.71815
25493	P14075	A9UHW6	0.5282
25494	P14075	Q8N2C9	0.15845
25495	P14075	P47813	0.80853
25496	P14075	A6QL64	0.42835
25497	P14075	Q8WU03	0.44716
25498	P14075	P78316	0.86927
25499	P14075	Q9H478	0.21732
25500	P14075	O00471	0.058576
25501	P14075	Q92993	0.72836
25502	P14075	Q15185	0.27733
25503	P14075	Q9UKV3	0.40663
25504	P14075	O95900	0.77918
25505	P14075	Q96H20	0.63722
25506	P14075	Q9NRI5	0.18718
25507	P14075	Q8N6N3	0.55449
25508	P14075	Q8N163	0.49598
25509	P14075	A6H8Y1	0.43466
25510	P14075	Q9HA77	0.43149
25511	P14075	P0C879	0.63821
25512	P14075	Q96FF9	0.59963
25513	P14075	Q86Y79	0.6389
25514	P14075	A6H8Z2	0.4937
25515	P14075	Q70EK8	0.56735
25516	P14075	Q8NFZ5	0.62672
25517	P14075	P53805	0.42528
25518	P14075	P36873	0.52145
25519	P14075	Q9Y618	0.8438
25520	P14075	O14497	0.58459
25521	P14075	Q9UI43	0.61979
25522	P14075	Q8TAL5	0.47308
25523	P14075	Q96KW2	0.58453
25524	P14075	B3EWF7	0.63821
25525	P14075	Q96HR8	0.87102
25526	P14075	Q9NP81	0.37995
25527	P14075	Q96GI7	0.94025
25528	P14075	O95229	0.53556
25529	P14075	O75290	0.2718
25530	P14075	Q5VWG9	0.56068
25531	P14075	Q13868	0.38965
25532	P14075	Q9UHV9	0.55068
25533	P14075	Q15029	0.21794
25534	P14075	O75081	0.865
25535	P14075	Q8TEP8	0.49613
25536	P14075	P61574	0.589

P14075	O95402	0.87263
P14075	Q07687	0.34755
P14075	P05198	0.44758
P14075	Q8WV24	0.40809
P14075	P22103	0.63821
P14075	O95347	0.25266
P14075	Q86Y26	0.78213
P14075	O75400	0.68175
P14075	O14503	0.67128
P14075	O75132	0.075083
P14075	Q6JBY9	0.53945
P14075	Q5JSJ4	1.1042
P14075	Q9UPV9	0.52079
P14075	Q6IPW1	0.41792
P14075	A8MU93	0.94025
P14075	Q9BVW5	0.70152
P14075	Q96A09	0.50797
P14075	O15371	0.42219
P14075	A7E2V4	1.106
P14075	O76031	0.57356
P14075	P49366	0.22692
P14075	Q9Y255	0.24038
P14075	P56279	0.67704
P14075	Q8TEL6	0.26075
P14075	P62312	0.57169
P14075	Q9Y2Q1	0.28992
P14075	P17040	0.3919
P14075	Q9Y248	0.46992
P14075	Q8N1I8	0.63821
P14075	P82909	0.31371
P14075	Q86V97	0.39464
P14075	Q9Y3B7	0.77418
P14075	P54252	0.3268
P14075	Q9BYR9	0.49168
P14075	Q7Z392	0.47968
P14075	Q6PF06	0.70187
P14075	P31269	0.44115
P14075	Q96LJ8	0.8371
P14075	O00231	0.29204
P14075	P07737	0.63382
P14075	Q8N5C8	0.26097
P14075	O75376	0.31391
P14075	Q5SQ13	0.046528
P14075	Q9C010	0.6114
P14075	Q92748	0.85129
P14075	Q9Y316	0.36542
P14075	A6NEQ9	0.63821
P14075	Q01826	0.54789
P14075	Q5JR59	0.75582
P14075	Q4G112	0.11658
P14075	P46527	0.21953
P14075	Q96D70	1.0023
P14075	Q92785	0.32033
P14075	Q9Y4P8	0.52438
P14075	Q5TEA3	0.55421
P14075	Q32P41	0.18019
P14075	Q9HCN2	0.63821
P14075	Q504T8	0.16891
P14075	Q16670	0.11191
P14075	Q96AQ7	0.44478
P14075	Q8IYX3	0.89363
P14075	Q9BV19	0.67075
P14075	P15515	0.37094
P14075	Q8IV03	1.0128
P14075	Q5T6C5	0.48825
P14075	Q8WV37	0.28249
P14075	Q8TEE9	0.84255
P14075	Q9HAZ2	0.24056
P14075	Q96TA1	0.096957
P14075	Q16637	0.63691
P14075	Q8WW33	0.46771
P14075	Q0VFZ6	0.94025
P14075	Q8IZ16	0.26921
P14075	P27540	0.29639
P14075	Q9BZ01	0.31426
P14075	Q6UWZ7	0.27578

P14075	P51398	0.85912
P14075	Q5HYI7	0.74769
P14075	Q05877	0.76903
P14075	O43255	0.24298
P14075	O75381	0.30763
P14075	Q8NCU4	0.24599
P14075	Q96MF0	0.26557
P14075	Q69YH5	0.75612
P14075	A6NJ64	0.35621
P14075	Q9Y3E7	0.52809
P14075	Q15390	0.54807
P14075	P0C671	0.12542
P14075	A1L188	0.87504
P14075	Q9Y2B4	0.12823
P14075	Q5VUG0	0.1228
P14075	Q00587	0.18572
P14075	Q9HCM9	0.35128
P14075	H0YKK7	0.66953
P14075	Q8NCU1	0.38453
P14075	Q92624	0.51476
P14075	Q9BUN5	0.24424
P14075	A8MZA4	0.36542
P14075	P23528	0.54779
P14075	Q9H7C4	0.20078
P14075	O95298	0.54966
P14075	Q9H714	0.31443
P14075	O60269	0.56642
P14075	Q96A44	0.30187
P14075	P62136	0.46451
P14075	Q9BVV6	0.63821
P14075	Q7Z4U5	0.81189
P14075	P55347	0.28459
P14075	Q9Y2R9	0.71259
P14075	Q86Z20	0.78594
P14075	Q9NW38	0.40008
P14075	Q8WTW4	0.23679
P14075	O75586	0.515
P14075	Q8IXM7	0.48907
P14075	Q3YBR2	0.33615
P14075	Q9H0E3	0.70823
P14075	Q71F78	0.35662
P14075	C9J3V5	0.56972
P14075	Q9C073	0.80583
P14075	O75940	0.44461
P14075	Q4LEZ3	0.736
P14075	Q8NI27	0.57773
P14075	O00461	0.47807
P14075	Q9UPN7	0.67833
P14075	P78396	0.65319
P14075	Q8IVU9	0.17021
P14075	Q9BSH5	0.18271
P14075	Q9UJX6	0.35911
P14075	Q76KD6	0.34431
P14075	Q7RTV3	0.19614
P14075	Q9H9H4	0.51296
P14075	O43592	0.12467
P14075	Q9BTX1	0.58741
P14075	Q8N5A5	0.6163
P14075	O00505	0.66305
P14075	A6NHR8	0.63821
P14075	Q96B23	0.69556
P14075	Q13875	0.32906
P14075	Q6ZUG5	0.31389
P14075	Q9UQB3	0.37117
P14075	Q99459	0.94122
P14075	C9JBD0	0.36365
P14075	Q9BTT4	0.48467
P14075	Q8IVK1	0.63821
P14075	Q13405	0.4211
P14075	O43516	0.060347
P14075	Q8N2Z9	0.30313
P14075	Q02556	0.23215
P14075	Q13137	0.71812
P14075	Q9Y676	0.63887
P14075	O00743	0.49196
P14075	Q96BJ3	0.48193

P14075	Q5HYC2	0.035575
P14075	P12757	0.20749
P14075	Q9H8S9	0.22592
P14075	Q9BYR6	1.0514
P14075	Q8TEV9	0.49599
P14075	P41219	0.46091
P14075	Q9NPD8	0.13309
P14075	P62487	0.24929
P14075	Q6ICB0	0.85156
P14075	Q9UFN0	0.38432
P14075	Q9BYR8	1.0514
P14075	Q9Y2M0	0.37379
P14075	Q9NUQ3	0.35508
P14075	Q03468	0.25014
P14075	Q9Y5P3	0.41736
P14075	Q9H4A5	0.64232
P14075	P0CW27	0.35926
P14075	P0CL80	0.45588
P14075	Q6P435	0.18558
P14075	Q5T7N2	0.10711
P14075	Q9ULD5	0.10499
P14075	Q8NHZ8	0.88202
P14075	P35711	0.71013
P14075	Q96QD9	0.5305
P14075	O15225	0.63821
P14075	Q9BWJ2	0.27732
P14075	O43583	0.89307
P14075	Q86UU5	0.61859
P14075	P41162	0.23395
P14075	P57077	0.46601
P14075	Q5T9G4	0.76943
P14075	Q8WV99	0.2404
P14075	Q96DI7	0.46061
P14075	Q8IUI4	0.18934
P14075	Q9H0C8	0.19352
P14075	Q92530	0.49745
P14075	Q15847	0.63821
P14075	Q9NV39	0.60703
P14075	Q8N1F1	0.63821
P14075	A1L3X0	0.40775
P14075	Q5XKK7	0.16522
P14075	P07438	0.30226
P14075	O00257	0.74948
P14075	P55209	0.4873
P14075	Q96DX5	0.76416
P14075	O96004	0.34936
P14075	Q96NI8	0.25704
P14075	Q9UKT5	0.25945
P14075	Q96MD2	0.24266
P14075	Q9Y285	0.55658
P14075	Q96HA8	0.79957
P14075	P16333	0.39254
P14075	Q9BU89	0.14863
P14075	Q13568	0.65406
P14075	Q6ZPA2	0.63821
P14075	P17098	0.29968
P14075	Q96EF6	0.4455
P14075	Q96MN9	0.38989
P14075	Q9BTE3	0.46531
P14075	O00291	0.23843
P14075	O43678	0.4604
P14075	Q8WU49	0.63821
P14075	Q6ICG7	0.63821
P14075	Q8TAI1	0.48874
P14075	A6NI86	0.60332
P14075	Q8NA96	0.41629
P14075	Q96BK5	0.3204
P14075	Q52M58	0.70371
P14075	Q13485	0.46037
P14075	P35232	0.33603
P14075	Q5T871	0.63821
P14075	O94992	1.1267
P14075	Q9UHK0	0.1809
P14075	A8MUZ8	0.34491
P14075	Q9NXV2	0.84521
P14075	Q96QR1	0.85349

P14075	Q99638	0.36204
P14075	Q8NA77	0.55098
P14075	O60870	0.47503
P14075	O00255	0.10019
P14075	Q9H777	0.42468
P14075	Q96AA8	0.32934
P14075	Q502W7	0.61279
P14075	Q8N7H5	0.68429
P14075	P60368	0.45591
P14075	Q9UPV0	1.1101
P14075	Q86WV1	0.32371
P14075	P09629	0.71226
P14075	P69208	0.63821
P14075	Q8N8I6	0.63821
P14075	P63208	0.39296
P14075	P17152	0.70107
P14075	Q6P047	0.45747
P14075	Q93074	0.35402
P14075	Q9BVC3	0.37979
P14075	O75817	0.70981
P14075	O75969	0.29831
P14075	Q9BVV2	0.11413
P14075	Q7Z4W3	0.63821
P14075	Q5TKA1	0.20805
P14075	P60371	0.78356
P14075	P61582	0.89322
P14075	O94864	0.36766
P14075	Q9ULN7	0.71723
P14075	P07900	0.45459
P14075	Q9NQT4	0.29639
P14075	Q6P1J9	0.47678
P14075	Q9NX04	0.42582
P14075	O75691	0.54622
P14075	Q9C0B2	0.33923
P14075	Q9P206	0.93609
P14075	Q96SW2	0.33517
P14075	Q8TAF3	0.37153
P14075	Q9H3R5	0.29147
P14075	Q5MJ10	0.88201
P14075	Q9Y6E7	0.28574
P14075	Q3LI64	0.63821
P14075	Q8WTR7	0.44801
P14075	Q9NP71	0.32571
P14075	A6NEY3	0.71412
P14075	Q8N535	0.36259
P14075	Q13156	0.19899
P14075	Q9NR28	0.54678
P14075	Q9H6K1	0.16122
P14075	Q6PJ61	0.85575
P14075	Q92499	0.29853
P14075	P11940	0.27979
P14075	P24278	0.61836
P14075	Q8TAX7	0.63821
P14075	Q9UFV3	0.053239
P14075	P10155	0.48861
P14075	A4D1E1	0.46672
P14075	Q14186	1.0451
P14075	Q96HA1	0.75131
P14075	Q9UII2	0.37035
P14075	A5PL33	0.81
P14075	Q6NZI2	0.74978
P14075	A6NMP4	0.29603
P14075	Q86XN6	0.2168
P14075	Q9Y238	0.26743
P14075	A6NHP3	0.53769
P14075	Q09028	0.47743
P14075	Q9Y315	0.48099
P14075	Q3SYA9	0.33853
P14075	O60831	0.72604
P14075	Q9BPZ7	0.51151
P14075	Q10586	0.22552
P14075	Q9NPF5	0.69054
P14075	Q969Q6	0.094409
P14075	P15036	0.54347
P14075	Q9Y2J4	0.13623
P14075	Q9UNZ5	0.94025

P14075	Q9ULW0	0.74248
P14075	Q86TA4	0.21569
P14075	Q5TEC3	0.14883
P14075	Q96HI0	0.37799
P14075	Q14999	0.64454
P14075	O14980	0.79458
P14075	P60228	0.67286
P14075	Q9NTK5	0.53678
P14075	Q86VM9	0.1522
P14075	P63173	0.57968
P14075	Q96MB7	0.55903
P14075	Q7Z7L8	0.88464
P14075	Q86WR7	0.92041
P14075	Q15365	0.68953
P14075	Q9BRT6	0.49501
P14075	Q9H204	0.78879
P14075	Q5T5A4	0.62555
P14075	Q6ZVX7	0.47604
P14075	Q96B36	0.65677
P14075	Q2M2I3	0.093036
P14075	Q8N999	0.79097
P14075	Q00839	0.26692
P14075	O14920	0.3884
P14075	Q86XA9	0.33398
P14075	Q8IYI8	0.23053
P14075	Q15853	0.45782
P14075	P55822	0.50388
P14075	Q8N5R6	0.37778
P14075	Q6F5E7	0.2246
P14075	Q9H9Q4	0.20084
P14075	Q8WW18	0.94025
P14075	Q8N1N5	0.27589
P14075	Q8N5U6	0.77047
P14075	Q9NQ89	0.21127
P14075	Q9BQ70	1.0273
P14075	P49116	0.45552
P14075	Q9NWS1	0.60675
P14075	Q12857	0.13416
P14075	O95628	0.49535
P14075	P16118	0.059968
P14075	Q9UKL3	0.89702
P14075	Q9UJ98	0.35629
P14075	Q16655	0.53872
P14075	Q86VP3	0.3942
P14075	A8MTY7	0.78356
P14075	P12036	0.32592
P14075	Q9NWF9	0.53377
P14075	P58005	0.30277
P14075	P50995	0.6136
P14075	Q68DK2	0.58167
P14075	Q9NWU5	0.64706
P14075	Q9NRY5	0.7971
P14075	O96019	0.73385
P14075	Q15323	0.70903
P14075	Q9HAW4	0.32669
P14075	Q8WW01	0.46314
P14075	Q9BW27	0.55424
P14075	Q6T4R5	0.29416
P14075	P46100	0.3706
P14075	P35244	0.34149
P14075	P54274	0.27775
P14075	Q9BXL8	0.2419
P14075	P30050	0.49535
P14075	O75865	0.45808
P14075	Q9HCL3	0.28807
P14075	Q92541	0.72873
P14075	Q6ZVD7	0.35278
P14075	Q9H606	0.45914
P14075	O43493	0.63581
P14075	Q8NBZ0	0.9324
P14075	P0DKV0	0.21272
P14075	Q9HCC9	0.17246
P14075	Q9H7Y2	0.43766
P14075	A2RRP1	0.118
P14075	O00189	0.094461
P14075	P52294	0.75349

P14075	Q6IE81	0.71347
P14075	P56378	0.61809
P14075	Q96D05	0.96877
P14075	Q9H2K0	0.40421
P14075	Q04726	0.75722
P14075	O14770	0.28309
P14075	Q8IWS0	0.2729
P14075	Q9UHI6	0.21451
P14075	A6NGU7	0.35107
P14075	P23327	0.58648
P14075	A6NCS6	0.37212
P14075	P25791	0.24972
P14075	Q8N9K3	0.94025
P14075	Q06587	0.59897
P14075	Q5VIY5	0.27773
P14075	P31749	0.38022
P14075	Q9Y6Y0	0.23784
P14075	O95487	0.43206
P14075	Q9H5I1	0.44662
P14075	Q9H4B6	0.34891
P14075	P07902	0.41518
P14075	Q9Y6G5	0.23554
P14075	Q9NXB0	0.50295
P14075	Q3B726	0.054527
P14075	Q2M3C7	0.30068
P14075	Q9H910	0.81714
P14075	P57721	0.20649
P14075	P02545	0.58109
P14075	Q19AV6	0.84912
P14075	O95810	0.22772
P14075	O75478	0.16888
P14075	Q8TAB5	0.94025
P14075	Q5JT78	0.30904
P14075	Q8ND07	0.50837
P14075	Q8TAE6	0.45019
P14075	P0CG48	0.27172
P14075	Q5VXD3	0.048221
P14075	Q15631	0.45197
P14075	Q9BYQ5	0.78356
P14075	Q96I23	0.13749
P14075	Q8WXS3	0.98542
P14075	Q96JC9	0.73062
P14075	Q9UPY8	0.70489
P14075	Q8N7U7	0.39001
P14075	Q04695	0.74795
P14075	O15235	0.46341
P14075	K7EJ46	0.63821
P14075	Q8IUC0	0.43324
P14075	Q9BTE6	0.59992
P14075	P04406	0.28286
P14075	A8MUA0	0.2376
P14075	Q5T655	0.63821
P14075	Q96EN9	0.72211
P14075	Q86US8	0.29353
P14075	Q8N9T2	0.63821
P14075	Q8IZD4	0.595
P14075	Q9BYR3	0.78356
P14075	Q66GS9	0.070043
P14075	Q9BQG0	0.38348
P14075	Q9Y221	0.55046
P14075	Q02952	0.40767
P14075	Q9NY43	0.25252
P14075	Q9C0F1	0.087371
P14075	P35908	0.47755
P14075	Q5VUA4	0.23115
P14075	Q5T7N8	0.22856
P14075	Q6I9Y2	0.64551
P14075	Q92539	0.30339
P14075	Q96DE5	0.92673
P14075	A2RUB1	0.94025
P14075	Q6ZS17	0.33313
P14075	P62979	0.2116
P14075	Q5T752	0.63821
P14075	Q9ULV5	0.21875
P14075	P28324	1.1064
P14075	Q96KC9	0.45762

25993	P14075	P49419	0.37172
25994	P14075	Q12986	0.072161
25995	P14075	Q6ZNA4	0.4212
25996	P14075	Q8N108	0.84816
25997	P14075	Q9Y692	0.48781
25998	P14075	Q96MF6	0.52792
25999	P14075	O75920	1.0791
26000	P14075	Q13435	0.60858
26001	P14075	P43897	0.81602
26002	P14075	Q6IQ49	0.22837
26003	P14075	Q99996	0.31831
26004	P14075	O00762	0.40178
26005	P14075	Q6ZNR8	0.63821
26006	P14075	Q5T699	0.033413
26007	P14075	Q96S95	0.53948
26008	P14075	Q86UY5	0.181
26009	P14075	Q8N7J2	0.82301
26010	P14075	Q9Y253	0.05997
26011	P14075	Q6ZUF6	0.26951
26012	P14075	O00401	0.41938
26013	P14075	Q9H8T0	0.28398
26014	P14075	Q5JPI3	0.90615
26015	P14075	Q17RM4	0.58215
26016	P14075	Q9NY27	0.78293
26017	P14075	Q96M60	0.56488
26018	P14075	O15078	0.38763
26019	P14075	Q96A49	0.61166
26020	P14075	Q8NCE0	0.382
26021	P14075	P0C7X3	0.40378
26022	P14075	Q6ZVN8	0.26223
26023	P14075	Q8N344	0.29481
26024	P14075	P57081	0.63456
26025	P14075	Q13310	0.2729
26026	P14075	Q9Y399	0.73211
26027	P14075	Q92670	0.16167
26028	P14075	P59797	0.61946
26029	P14075	Q96CG3	0.29412
26030	P14075	O60573	0.31482
26031	P14075	Q9H5J8	0.96753
26032	P14075	Q9Y2P8	0.42303
26033	P14075	O14964	0.36216
26034	P14075	Q15326	0.92206
26035	P14075	P61236	0.27189
26036	P14075	O75618	0.88774
26037	P14075	Q9H9H5	0.54045
26038	P14075	Q86XN7	0.94025
26039	P14075	Q14CW9	0.6362
26040	P14075	Q9NPE3	0.64046
26041	P14075	Q8TA94	0.31311
26042	P14075	Q6ZTN6	0.48582
26043	P14075	Q5JU67	0.03075
26044	P14075	P83731	0.49535
26045	P14075	Q08379	0.11649
26046	P14075	Q8NC38	0.090643
26047	P14075	O75556	0.29237
26048	P14075	Q9H8G2	0.90615
26049	P14075	Q9H6K4	0.58861
26050	P14075	O14604	0.51575
26051	P14075	Q107X0	0.59777
26052	P14075	Q9BU64	0.3547
26053	P14075	Q8N8D1	0.69126
26054	P14075	Q9BU61	0.40067
26055	P14075	P51397	0.68026
26056	P14075	Q9BYQ4	0.78356
26057	P14075	Q96EA4	0.47865
26058	P14075	O60756	0.63821
26059	P14075	P49458	0.6215
26060	P14075	Q15013	0.58225
26061	P14075	Q5SW79	0.52568
26062	P14075	Q9BXY8	0.4678
26063	P14075	P84098	0.38311
26064	P14075	Q9Y2W2	0.72939
26065	P14075	Q9NX55	0.29666
26066	P14075	Q9Y3C7	0.73677
26067	P14075	Q96BW1	0.16677
26068	P14075	Q8TDR0	0.68599

P14075	Q8NAP1	0.94025
P14075	Q8N4U5	0.10299
P14075	O00139	0.24681
P14075	P60602	0.51691
P14075	Q5BJF6	0.28164
P14075	O43505	0.53711
P14075	Q9NQX1	0.33997
P14075	Q9NZV7	0.33849
P14075	Q8WUD4	0.24684
P14075	Q86V20	0.73725
P14075	P32519	1.0104
P14075	Q9UP83	0.90273
P14075	A4D250	0.091626
P14075	Q4LE28	0.51806
P14075	Q9Y512	0.074531
P14075	Q8IXQ9	0.2129
P14075	Q86VP1	0.19477
P14075	P49754	0.27442
P14075	Q9UNN5	0.41708
P14075	A6NEY8	0.31238
P14075	Q6IN85	0.16905
P14075	O14948	0.21686
P14075	Q96NS8	0.29819
P14075	Q9C0I3	0.82824
P14075	Q6ZUL3	0.63821
P14075	P59036	0.63821
P14075	O60613	0.42923
P14075	O00170	0.43858
P14075	Q8TAY7	1.2058
P14075	Q96GG9	0.24793
P14075	Q3YBM2	0.75144
P14075	Q8NEF9	0.39175
P14075	P14406	0.52052
P14075	P49918	0.49547
P14075	Q14653	0.41071
P14075	A6NGD5	0.14392
P14075	P62318	0.59235
P14075	Q96S59	0.56496
P14075	Q9Y5A7	0.45161
P14075	Q8WXW3	0.61938
P14075	Q9BZ98	0.036759
P14075	Q9UPW6	0.52299
P14075	P28062	0.15077
P14075	Q06455	0.5031
P14075	Q9UGL9	0.63821
P14075	Q8N983	0.3008
P14075	Q86T75	0.455
P14075	Q96RM1	0.63821
P14075	Q96HA7	0.4926
P14075	Q96FJ2	0.33082
P14075	Q96M53	0.94474
P14075	Q9UGK3	0.42667
P14075	P83369	0.49375
P14075	Q2KHM9	0.3621
P14075	Q96D98	0.74783
P14075	Q6P1R3	0.35942
P14075	Q5JQC4	0.63821
P14075	Q92546	0.96574
P14075	Q8N1K5	0.46495
P14075	Q15370	0.82592
P14075	Q8NCF5	0.257
P14075	O15116	0.56711
P14075	Q9NVM1	0.87155
P14075	O14641	0.47021
P14075	Q06547	0.84001
P14075	Q01664	0.38423
P14075	Q7Z3Y8	0.70708
P14075	Q7Z6K1	0.49089
P14075	Q7L3T8	0.49952
P14075	Q8NDZ0	0.11256
P14075	O43768	0.029362
P14075	O00411	0.3939
P14075	Q9HBH7	0.45839
P14075	Q5VSL9	0.69209
P14075	Q9UBC1	0.28293
P14075	P99999	0.44997

P14075	Q12874	0.32955
P14075	Q14532	0.55056
P14075	O60287	0.9966
P14075	P26371	0.63821
P14075	Q8TBZ0	0.026295
P14075	Q13490	0.29607
P14075	Q86SX3	1.1716
P14075	Q8TCC3	0.15452
P14075	Q9P258	0.32428
P14075	O75683	0.48545
P14075	Q14106	0.25138
P14075	Q2TAL8	0.73919
P14075	O76013	0.70903
P14075	P22674	0.16711
P14075	O95983	0.92085
P14075	P51116	0.37914
P14075	Q53TQ3	0.37441
P14075	O60739	0.27865
P14075	P55769	0.65348
P14075	B2RUZ4	0.98812
P14075	Q9UHD9	0.53294
P14075	P28074	0.38808
P14075	O75935	0.61351
P14075	O14744	0.72312
P14075	O95865	0.21008
P14075	P54845	0.11975
P14075	Q14232	0.079623
P14075	Q9UHB7	1.0522
P14075	Q6ZTC4	0.63821
P14075	Q5VY09	0.98003
P14075	Q9HAF1	0.73244
P14075	P23193	0.92383
P14075	Q52MB2	0.67938
P14075	Q17RC7	0.5225
P14075	Q5T0W9	0.14797
P14075	Q8TES7	0.37934
P14075	Q58F21	0.15004
P14075	A6NK39	0.13209
P14075	Q9BSH4	1.0016
P14075	P08779	0.7693
P14075	P22033	0.41913
P14075	Q96HB5	0.42705
P14075	Q9NZH5	0.34269
P14075	Q96JM7	0.22447
P14075	Q8IWE5	0.22009
P14075	P29966	0.74258
P14075	O75161	0.79378
P14075	Q6ZT83	0.25192
P14075	Q8IX21	0.43121
P14075	A8MU10	0.63821
P14075	Q9NR56	0.30118
P14075	Q05481	0.20657
P14075	A6NES4	0.38275
P14075	Q96LA8	0.48808
P14075	Q96LB3	0.32655
P14075	A6NH11	0.62505
P14075	Q15437	0.11814
P14075	O75208	0.54354
P14075	Q9C0C6	0.78532
P14075	Q9NUC0	0.4044
P14075	Q2VPK5	0.47373
P14075	P33240	0.37946
P14075	Q8NHG7	0.79071
P14075	Q9HD40	0.31488
P14075	Q9BZQ8	0.59777
P14075	Q96RE7	0.66502
P14075	Q9UJX5	0.52601
P14075	Q9UHV7	0.39358
P14075	P56192	0.31844
P14075	Q9UNY5	0.31491
P14075	O75528	0.54533
P14075	Q96DF8	0.8246
P14075	Q8NA47	0.94025
P14075	Q7Z6B0	0.33317
P14075	Q96NL0	0.251
P14075	Q12830	0.70532

P14075	Q8N0T1	0.52497
P14075	P52746	0.018679
P14075	Q93083	0.30226
P14075	Q96PV7	0.92041
P14075	Q8N7B1	0.56549
P14075	Q12933	0.47554
P14075	A5D8W1	0.040252
P14075	P26678	0.46314
P14075	P61247	0.85171
P14075	Q8N4W9	0.2118
P14075	Q9NRL3	0.25577
P14075	Q495Z4	0.40532
P14075	Q96BP2	0.8499
P14075	O60783	0.51419
P14075	Q9P1J3	0.63821
P14075	Q9P0L1	0.18226
P14075	Q99909	0.17003
P14075	Q9H4I3	0.044635
P14075	Q9BUY5	0.26869
P14075	O14910	0.48326
P14075	Q99873	0.40061
P14075	O14519	0.28501
P14075	P62753	0.70555
P14075	Q8N228	0.073272
P14075	Q9NXH3	0.45019
P14075	Q86XR8	0.64015
P14075	Q9H0N5	0.6457
P14075	Q9NP98	0.55296
P14075	Q9Y6B2	0.76799
P14075	A8K830	0.63821
P14075	Q5HYR2	0.84601
P14075	P46782	0.33404
P14075	Q14676	0.40023
P14075	Q6IQ19	0.49027
P14075	Q96M42	0.1478
P14075	Q9NTG7	0.28032
P14075	P50221	0.4937
P14075	Q32M78	0.25506
P14075	P62891	0.49305
P14075	Q13469	0.81845
P14075	Q9H0A8	0.64522
P14075	Q9BWD3	0.65902
P14075	Q7L266	0.27278
P14075	O96017	0.54675
P14075	Q96N22	0.31321
P14075	Q5T751	0.63821
P14075	Q8IXS8	0.73255
P14075	O60299	0.18552
P14075	Q15650	0.52908
P14075	P13640	0.38669
P14075	Q9UJM3	0.25034
P14075	Q96PK6	0.56411
P14075	P22531	0.63821
P14075	Q8N0U7	0.55833
P14075	Q5TEZ4	0.63821
P14075	Q5T5J6	0.64221
P14075	Q5T1N1	1.0433
P14075	Q9Y2T7	0.075014
P14075	Q9UHC1	0.26268
P14075	O95373	0.71773
P14075	Q96B45	0.083005
P14075	P62861	0.8238
P14075	Q6W2J9	0.27366
P14075	Q9H943	0.15822
P14075	Q8WTP9	1.1259
P14075	A6NIN4	0.63821
P14075	Q9HB07	0.25622
P14075	Q13889	0.17389
P14075	Q58A44	0.63821
P14075	O15350	0.22465
P14075	P52742	0.18341
P14075	Q16254	0.80743
P14075	Q9H8E8	0.44481
P14075	Q8NCA5	0.63165
P14075	Q9UL49	0.54288
P14075	Q8N7M2	0.30057

P14075	Q13526	0.40586
P14075	Q99684	0.22965
P14075	Q14684	0.38392
P14075	P04264	0.18692
P14075	Q9UBK9	0.73755
P14075	Q13066	0.45588
P14075	Q6PI98	0.41256
P14075	Q8N693	0.2727
P14075	Q9BYN7	0.048821
P14075	Q8N3J9	0.12173
P14075	A6NEV1	0.69847
P14075	Q15038	1.066
P14075	Q92769	0.27146
P14075	P0CL82	0.45588
P14075	Q9UHJ9	0.50858
P14075	Q7L804	0.38086
P14075	Q9HCG1	0.23304
P14075	Q01892	0.20165
P14075	Q12904	0.073534
P14075	Q6AI08	0.45209
P14075	A6NGH7	0.31016
P14075	Q86U86	0.76916
P14075	Q6ZV65	0.29059
P14075	P0CW71	0.63821
P14075	Q0VDD5	0.63821
P14075	Q2YD98	0.3943
P14075	Q96A35	0.63295
P14075	O95456	0.26513
P14075	O14862	0.30873
P14075	P25205	0.21969
P14075	Q7Z5K2	0.46092
P14075	A6NNL0	0.72275
P14075	O75145	0.25515
P14075	P28065	0.44968
P14075	P0C860	0.1397
P14075	P13646	0.84455
P14075	P48378	0.22921
P14075	Q9BV29	0.14717
P14075	Q6NZY4	0.43599
P14075	O95990	0.43183
P14075	Q8NC54	0.88741
P14075	Q9C005	0.42237
P14075	P61571	0.41774
P14075	O60518	0.69697
P14075	Q9H8W3	0.43285
P14075	Q9H8G1	0.30184
P14075	Q8N1L1	0.63821
P14075	Q9BUJ2	0.90159
P14075	Q9BYR5	0.78356
P14075	Q63HM1	0.26027
P14075	Q6ZN17	0.8843
P14075	Q9P1F3	0.57845
P14075	Q9NZF1	0.157
P14075	Q9UPQ9	0.37369
P14075	Q96RY5	0.73692
P14075	P37802	0.20936
P14075	P18077	0.30461
P14075	O14965	0.24225
P14075	Q3C1V1	0.94025
P14075	Q05048	0.54949
P14075	Q8N7B6	0.21625
P14075	O75525	0.62552
P14075	Q05BU3	0.63821
P14075	Q96NB1	0.13763
P14075	Q96M66	0.18632
P14075	Q9H963	0.24855
P14075	P11441	0.33676
P14075	A8MQ03	0.39347
P14075	Q96K30	0.51932
P14075	Q9NVS9	0.39668
P14075	P82664	0.84967
P14075	Q7Z309	0.47411
P14075	Q8NCR3	0.21329
P14075	P78543	0.45785
P14075	Q9Y5B9	0.085568
P14075	Q9NPA3	0.91989

26373	P14075	Q9NSA3	0.49265
26374	P14075	Q9Y5N5	0.19647
26375	P14075	Q9NQS1	0.52502
26376	P14075	Q69YN4	0.41977
26377	P14075	Q5HYW3	0.65019
26378	P14075	Q99623	0.77482
26379	P14075	Q8TEM1	0.2089
26380	P14075	Q9BQ65	0.48955
26381	P14075	Q8N7R1	0.49026
26382	P14075	Q9Y5Q3	0.64368
26383	P14075	O95780	0.31244
26384	P14075	Q7RTS9	0.87311
26385	P14075	Q9NVH2	0.4411
26386	P14075	Q15834	0.82568
26387	P14075	P49848	0.54584
26388	P14075	Q9BZP3	0.23756
26389	P14075	Q15424	0.58052
26390	P14075	Q9NV31	0.61102
26391	P14075	Q8IZS5	0.6087
26392	P14075	Q68CP9	0.30872
26393	P14075	P46779	0.83108
26394	P14075	Q6UW32	0.47672
26395	P14075	Q5XKE5	0.52312
26396	P14075	Q8NDQ6	0.1983
26397	P14075	Q96AB3	0.49037
26398	P14075	Q86X55	0.50814
26399	P14075	Q6IPX3	0.71383
26400	P14075	P35712	0.36276
26401	P14075	Q92665	0.53398
26402	P14075	P62917	0.59151
26403	P14075	Q9BXT5	0.42111
26404	P14075	Q2TAP0	0.46003
26405	P14075	P53618	0.49657
26406	P14075	A5YM69	0.23692
26407	P14075	O95831	0.4711
26408	P14075	Q9HCU9	0.84768
26409	P14075	A6NEF3	0.71412
26410	P14075	Q13185	0.61997
26411	P14075	P60370	0.78356
26412	P14075	Q9UBB9	0.23589
26413	P14075	Q6ZP68	0.74887
26414	P14075	Q6MZP7	0.36433
26415	P14075	Q3B7T1	0.82917
26416	P14075	Q7L8W6	0.55312
26417	P14075	Q8WXC3	0.38336
26418	P14075	Q15637	0.22598
26419	P14075	Q6ZVT6	0.29054
26420	P14075	Q9ULX6	0.35176
26421	P14075	Q9NSC5	0.31519
26422	P14075	Q3KQU3	0.49936
26423	P14075	Q9Y2D9	0.39205
26424	P14075	Q86SG4	0.009084
26425	P14075	Q96LM9	0.061456
26426	P14075	Q9BUW7	0.94025
26427	P14075	Q9NWT8	0.52302
26428	P14075	Q9BQ52	0.28087
26429	P14075	Q5T686	0.38848
26430	P14075	O14523	0.68914
26431	P14075	Q9UQN3	0.2346
26432	P14075	Q9H2J4	0.4136
26433	P14075	P0CI01	0.53769
26434	P14075	O43679	0.12111
26435	P14075	P39023	0.68268
26436	P14075	Q3KP66	0.98706
26437	P14075	P86452	0.25931
26438	P14075	Q8TE69	0.1668
26439	P14075	Q9NQ29	0.52501
26440	P14075	Q9H1D9	0.31845
26441	P14075	Q9BVM2	0.41096
26442	P14075	P55771	0.59989
26443	P14075	Q6VMQ6	0.89013
26444	P14075	Q6QNY1	0.50307
26445	P14075	Q9HB09	0.96006
26446	P14075	Q9BW85	0.91878
26447	P14075	Q96E29	0.46529
26448	P14075	Q9Y3X0	0.70218

P14075	Q9UND3	0.20858
P14075	Q9NZE8	0.71767
P14075	Q9BQ66	0.78356
P14075	A2RTX5	0.30111
P14075	Q6PD62	0.44218
P14075	Q49AM1	0.21026
P14075	Q9H9D4	0.3617
P14075	P41214	0.46601
P14075	Q96NY9	0.45566
P14075	Q0VG73	0.63821
P14075	Q6R327	0.033757
P14075	Q15287	0.71913
P14075	Q3ZCV2	0.94025
P14075	Q04637	0.30689
P14075	Q9UIS9	0.34267
P14075	P46939	0.32312
P14075	Q5TCM9	0.63821
P14075	P51504	0.1745
P14075	A1L162	0.42481
P14075	Q6Q795	0.63821
P14075	Q7Z5L9	0.68681
P14075	Q9H7Z3	0.2684
P14075	Q96N06	0.61962
P14075	Q9H944	0.88349
P14075	P52744	0.17201
P14075	Q9BYT5	0.42224
P14075	Q9HC62	0.31179
P14075	Q9BTK6	0.24046
P14075	O60543	0.20654
P14075	Q3SY46	0.70449
P14075	Q9HCK8	0.21616
P14075	P50502	0.37205
P14075	Q701N2	0.63821
P14075	Q9UNQ2	0.43608
P14075	O15014	0.7795
P14075	Q9UPN9	0.31725
P14075	Q86WH2	0.016985
P14075	Q8WXE9	0.327
P14075	Q8WWL7	0.69486
P14075	Q9BYG8	0.14817
P14075	Q9UN79	0.68845
P14075	P50914	0.74903
P14075	Q5TGP6	0.35732
P14075	Q15007	0.47573
P14075	O15370	0.23243
P14075	O00472	0.33296
P14075	Q5JVS0	0.54996
P14075	Q13765	0.78923
P14075	Q99732	0.34646
P14075	Q8IUR0	0.72771
P14075	Q8NE22	0.99467
P14075	Q16890	0.26245
P14075	P24928	0.66013
P14075	Q8NAP3	0.15666
P14075	Q86WV5	0.45027
P14075	Q8TE04	0.47723
P14075	Q05BQ5	0.04253
P14075	Q15691	0.44742
P14075	P61956	0.43665
P14075	A2RUQ5	0.1322
P14075	Q9UHF7	0.32061
P14075	P67870	0.14371
P14075	P50616	0.20701
P14075	Q9H6K5	0.94025
P14075	Q6NVV1	0.27332
P14075	Q1A5X6	0.94025
P14075	P62269	0.48985
P14075	P62847	0.41024
P14075	Q6PG37	0.308
P14075	Q330K2	0.36714
P14075	Q9BW92	0.34641
P14075	Q5TA79	0.63821
P14075	Q8N655	0.63821
P14075	A4D0Y5	0.63821
P14075	Q99733	0.5089
P14075	Q14696	0.47736

P14075	Q8IW19	0.1378
P14075	O75251	0.12061
P14075	Q63HQ0	0.63821
P14075	Q8N6N6	0.096162
P14075	Q3LI72	0.63821
P14075	P62910	0.4006
P14075	Q9H320	0.99246
P14075	Q9BXH1	0.21388
P14075	Q8TCJ0	0.79786
P14075	Q9Y421	0.24093
P14075	O43776	0.47166
P14075	Q96C11	0.17383
P14075	P0DI81	0.7535
P14075	Q53FE4	0.182
P14075	O60238	0.17673
P14075	O43189	0.27035
P14075	O95671	0.47891
P14075	P0CG12	1.1051
P14075	Q5SY13	0.63821
P14075	P62273	0.60336
P14075	Q8N6I1	0.75342
P14075	Q9BV73	0.41414
P14075	Q7Z7A3	0.42839
P14075	Q86TB9	0.55702
P14075	Q9NUL5	0.28665
P14075	Q96HA9	0.50685
P14075	Q5T7V8	1.3037
P14075	Q9ULM3	0.54938
P14075	Q15025	0.29174
P14075	Q9BVM4	0.66303
P14075	H3BNL8	0.69256
P14075	O95782	0.22651
P14075	Q96ST2	0.29867
P14075	Q96MU7	0.53716
P14075	Q9NYP9	0.30915
P14075	P27797	0.34638
P14075	Q14154	0.29562
P14075	Q9H5F2	0.52238
P14075	Q96IT6	0.63821
P14075	O43318	0.42025
P14075	Q3LI63	0.63821
P14075	P0C7T7	0.63821
P14075	Q08AI8	0.94025
P14075	O75150	0.3266
P14075	Q8IVV7	0.26331
P14075	Q15723	0.97999
P14075	Q99848	0.21098
P14075	Q9NS25	0.92895
P14075	Q96S07	0.63821
P14075	O75663	0.25995
P14075	P51531	0.50379
P14075	Q5T8I9	0.032127
P14075	Q9BTA9	0.69069
P14075	Q9HAT8	0.36899
P14075	P86480	0.63821
P14075	Q6ZQN3	0.74168
P14075	Q5TB80	0.58135
P14075	P30047	0.41211
P14075	Q9BTD3	0.76323
P14075	Q8IY42	1.0932
P14075	Q14738	0.4796
P14075	Q5T0J7	0.6278
P14075	Q16836	0.34785
P14075	Q9Y535	0.033137
P14075	Q9H808	0.31176
P14075	P04259	0.22406
P14075	Q6P2H3	0.25173
P14075	Q8WXC7	0.31812
P14075	Q13069	0.079883
P14075	Q9Y6D0	0.71741
P14075	Q6ZRV3	0.63821
P14075	Q9Y608	0.26634
P14075	Q96LS8	0.45184
P14075	Q9P015	0.31111
P14075	Q9NZM5	0.40191
P14075	Q14934	0.2798

26601	P14075	Q9BXN6	0.25576
26602	P14075	Q6ZRT6	0.94025
26603	P14075	Q5T5Y3	0.28778
26604	P14075	Q96AJ1	1.1609
26605	P14075	Q8WYQ3	0.87504
26606	P14075	Q6UUV9	1.1049
26607	P14075	Q15573	0.45023
26608	P14075	Q8NEY8	0.65763
26609	P14075	P02794	0.5652
26610	P14075	Q8IYX0	0.30419
26611	P14075	Q9UKT9	0.34846
26612	P14075	Q8TC56	1.1182
26613	P14075	Q8IZT9	0.83666
26614	P14075	Q13158	0.25336
26615	P14075	Q92837	0.82737
26616	P14075	P0CG00	0.065088
26617	P14075	P35269	0.85762
26618	P14075	Q8N5N4	0.55562
26619	P14075	Q5T7W7	0.94025
26620	P14075	Q96N53	0.63821
26621	P14075	Q9NPA8	0.22164
26622	P14075	P27986	0.35683
26623	P14075	B9ZVM9	0.77798
26624	P14075	Q8N5U0	0.63821
26625	P14075	O15198	0.25698
26626	P14075	P11473	0.24448
26627	P14075	Q14CS0	0.12242
26628	P14075	Q99594	0.91047
26629	P14075	Q8IZU0	0.83666
26630	P14075	P60410	1.0514
26631	P14075	Q14657	1.0068
26632	P14075	Q5VWM5	0.51701
26633	P14075	Q14677	0.22346
26634	P14075	Q9BUN8	0.223
26635	P14075	Q5TID7	0.4227
26636	P14075	Q659A1	0.31489
26637	P14075	Q6NXP2	0.96178
26638	P14075	Q5TH74	0.47439
26639	P14075	P86478	0.63821
26640	P14075	Q8N443	0.53937
26641	P14075	Q6ZSA8	0.63821
26642	P14075	A6NFQ2	0.82659
26643	P14075	Q86VP6	0.53372
26644	P14075	O60244	0.39358
26645	P14075	Q96EE3	0.39009
26646	P14075	Q9Y6X8	0.53068
26647	P14075	Q96CJ1	0.93618
26648	P14075	Q3MHD2	0.38538
26649	P14075	Q9Y3B9	0.46901
26650	P14075	Q9H008	0.22499
26651	P14075	Q96MF4	0.71296
26652	P14075	Q6P2Q9	0.40791
26653	P14075	Q9BQE6	0.54537
26654	P14075	P14324	0.10623
26655	P14075	Q3SY00	0.7534
26656	P14075	A8MUX0	0.57913
26657	P14075	O14558	0.50268
26658	P14075	Q5RKV6	0.062262
26659	P14075	Q7Z7H3	0.2552
26660	P14075	Q9NZ81	1.1204
26661	P14075	P49842	0.70226
26662	P14075	Q86TS9	0.21705
26663	P14075	Q96GT9	0.90785
26664	P14075	O75928	0.55074
26665	P14075	Q8IWJ2	0.45549
26666	P14075	Q9HC52	0.59783
26667	P14075	Q9ULW3	0.67036
26668	P14075	Q5VX52	0.059261
26669	P14075	Q92925	0.40621
26670	P14075	Q86YV9	0.79813
26671	P14075	P37198	0.70223
26672	P14075	P40429	0.49673
26673	P14075	P54727	0.4705
26674	P14075	O75807	0.28231
26675	P14075	Q9UN36	0.36355
26676	P14075	Q9H6W3	0.25057

P14075	Q6NYC8	0.38535
P14075	Q9NQ50	0.87505
P14075	P59091	0.34253
P14075	Q96Q35	0.37828
P14075	Q6IV72	0.1193
P14075	P0C025	0.56067
P14075	Q96BT1	0.037151
P14075	Q8N587	0.26731
P14075	A6NNW6	0.48155
P14075	F8WFD2	0.27544
P14075	Q5VWQ0	1.1172
P14075	Q8N137	0.62294
P14075	Q8N895	0.30058
P14075	Q9H816	0.23652
P14075	Q96FA3	0.34174
P14075	P82663	0.57474
P14075	Q96IR2	0.22707
P14075	P26368	0.38796
P14075	Q7Z417	0.51882
P14075	Q9BZL1	0.34705
P14075	P23396	0.53921
P14075	Q9C099	0.63532
P14075	P48382	0.69203
P14075	Q9BRD0	0.54064
P14075	Q8IWF9	0.38345
P14075	Q6ZUU3	0.63821
P14075	Q9H095	0.30581
P14075	O14907	0.24783
P14075	Q5TYX0	0.2982
P14075	P20248	0.79525
P14075	P53999	0.98547
P14075	O60220	0.34863
P14075	P19474	0.27673
P14075	P57060	0.46915
P14075	O15255	0.63821
P14075	Q8NDX9	0.60984
P14075	Q5VVC0	0.31067
P14075	Q8WXF8	0.96456
P14075	P22087	0.45233
P14075	P62241	0.38457
P14075	P35251	0.41499
P14075	Q9Y6K5	0.05805
P14075	Q8N584	0.71876
P14075	Q9UK39	0.30277
P14075	P52655	0.87542
P14075	Q7Z4H9	0.46956
P14075	P25786	0.43914
P14075	Q8NET4	0.45399
P14075	Q9Y291	0.43358
P14075	Q8TAP6	0.58298
P14075	Q9UJJ7	0.21639
P14075	Q01081	0.40307
P14075	Q15306	0.55864
P14075	P56555	0.6934
P14075	Q16531	0.37503
P14075	Q8WVR3	0.94025
P14075	Q8IXM6	0.66346
P14075	Q96NJ1	0.63821
P14075	Q8TF74	0.33429
P14075	Q92575	0.60086
P14075	Q711Q0	0.63821
P14075	A6NDE8	0.45588
P14075	P07864	0.32299
P14075	Q96GN5	0.56091
P14075	Q5SNX5	0.63821
P14075	O75474	1.0515
P14075	O43745	0.47425
P14075	O00410	0.85473
P14075	P0C7T4	0.63821
P14075	Q969X6	1.0329
P14075	Q9Y2G5	0.34198
P14075	Q8N5S3	0.26819
P14075	Q6AI39	0.91344
P14075	Q658L1	0.82868
P14075	Q99456	0.80046
P14075	Q5SRE5	0.42425

P14075	P15880	0.72412
P14075	Q5SV97	0.85113
P14075	Q96FH0	0.49119
P14075	Q6QNY0	0.54284
P14075	Q15173	0.15245
P14075	O15405	0.70668
P14075	P17947	0.53178
P14075	Q9UIF9	0.50176
P14075	Q9UKD1	0.50783
P14075	Q96RS0	0.41012
P14075	Q6RUI8	0.62867
P14075	Q8NE71	0.2197
P14075	P78345	0.85283
P14075	Q32NC0	0.52579
P14075	P62826	0.5054
P14075	Q9UKC9	0.31334
P14075	O14818	0.45704
P14075	Q15054	0.40821
P14075	Q99750	0.41376
P14075	Q14CX7	0.31736
P14075	Q14687	0.1668
P14075	A6NIX2	0.75949
P14075	Q8TBE0	0.5084
P14075	P11308	0.41605
P14075	Q8IYJ2	0.69053
P14075	Q9H8Y8	0.1482
P14075	Q9BSF4	0.47147
P14075	Q9NZZ3	0.62406
P14075	Q5SNV9	0.33245
P14075	Q8TE02	0.90336
P14075	Q6ZMV5	0.36255
P14075	Q9P2M1	0.31745
P14075	O95336	0.04456
P14075	P46087	0.53233
P14075	Q16514	0.64862
P14075	O95251	0.63624
P14075	Q6R2W3	0.2543
P14075	P86434	0.070635
P14075	P82930	0.91004
P14075	Q96BT3	0.29531
P14075	Q9NQP4	0.59299
P14075	Q96CS2	0.33094
P14075	P49770	0.11101
P14075	Q5QJE6	0.94955
P14075	Q5THK1	0.16891
P14075	Q9NUP1	0.4215
P14075	O14613	0.052977
P14075	Q5JR98	0.41004
P14075	P0CG42	0.63821
P14075	P82970	0.44612
P14075	Q8TAQ2	1.1845
P14075	Q9NR77	0.44537
P14075	Q8N9M1	0.28921
P14075	P78318	0.63972
P14075	P62987	0.41742
P14075	P0DMC3	0.63821
P14075	O14628	0.26779
P14075	Q9Y4P9	0.35159
P14075	Q0VF49	0.63821
P14075	Q14151	0.51347
P14075	B3EWG4	0.94025
P14075	P78346	0.49556
P14075	O76015	0.70708
P14075	P67809	0.47023
P14075	O43257	0.30389
P14075	O14490	0.46604
P14075	Q8IYD1	0.18807
P14075	Q8IX15	0.28947
P14075	Q13309	0.38792
P14075	Q8WUY8	0.70967
P14075	Q8IYK2	0.89928
P14075	O60907	0.57097
P14075	O60290	0.36407
P14075	Q06787	0.64068
P14075	Q96K31	0.67811
P14075	Q9BPU9	0.1003

P14075	Q8WXQ3	0.97015
P14075	A8MQB3	0.12021
P14075	P63104	0.71855
P14075	Q9P1Z2	0.55575
P14075	P06396	0.18832
P14075	Q9UH77	0.33388
P14075	Q8IZ63	0.63821
P14075	Q9BR84	0.33283
P14075	P0C7V0	0.63821
P14075	Q99417	1.151
P14075	Q8NB42	0.30624
P14075	Q9Y3P8	0.55445
P14075	P60412	0.78356
P14075	Q9C035	0.35674
P14075	Q8IYB9	0.21527
P14075	Q8TCI5	0.37598
P14075	A2RU30	0.7363
P14075	Q9HB19	0.29954
P14075	Q9Y3S2	0.29071
P14075	O43747	0.44917
P14075	Q13114	0.23734
P14075	Q9NWT6	0.28842
P14075	A8MXZ3	0.63821
P14075	Q96LR7	0.64624
P14075	Q05D60	0.013653
P14075	Q9UIA9	0.25244
P14075	Q6ZR54	0.63821
P14075	Q1HG44	0.76733
P14075	O14602	0.30134
P14075	Q8N865	0.25616
P14075	A0JLT2	0.95001
P14075	O00458	0.67695
P14075	Q9BRS8	0.28334
P14075	P13994	0.28631
P14075	Q9HD47	0.12137
P14075	Q5W0A0	0.30266
P14075	P53041	0.35295
P14075	P21127	0.36636
P14075	P35579	0.55765
P14075	P08697	0.090477
P14075	P78358	0.95806
P14075	Q96DC7	0.78327
P14075	Q96KX1	0.068553
P14075	Q9P031	0.99645
P14075	Q99551	0.49491
P14075	Q9BZA5	0.59445
P14075	Q9H063	0.42981
P14075	Q01534	0.23578
P14075	P14868	0.56002
P14075	P01858	0.63821
P14075	Q9Y5L5	1.0211
P14075	Q8NEZ5	0.48866
P14075	Q9HAU5	0.57266
P14075	Q9NWW7	0.96867
P14075	Q9BZQ2	0.36033
P14075	Q9HBQ8	0.71412
P14075	Q8TAT8	0.38078
P14075	Q5JUK9	0.34451
P14075	P06748	0.29207
P14075	Q5MJ08	0.30868
P14075	Q6PJG2	0.45794
P14075	P82914	0.61828
P14075	Q8N8A8	0.59424
P14075	Q86YI8	0.33367
P14075	Q3ZCT1	0.14871
P14075	Q5T160	0.59948
P14075	Q8TBK6	0.54985
P14075	Q8IW50	0.94025
P14075	Q15545	0.72977
P14075	Q96BD5	0.2455
P14075	P23511	0.86355
P14075	Q6PF05	0.23361
P14075	O60303	0.36658
P14075	Q96LQ0	0.53383
P14075	Q5TA76	0.63821
P14075	Q96DB5	0.3246

P14075	P61457	0.78196
P14075	A6ND36	0.015696
P14075	Q52LG2	0.43451
P14075	Q9H3M7	0.57553
P14075	P57055	0.47254
P14075	Q7L1Q6	0.16628
P14075	Q13227	0.88372
P14075	Q8WVZ9	0.39464
P14075	O15442	0.11311
P14075	Q9BSF0	0.28219
P14075	P35573	0.30864
P14075	Q8N9U9	0.26393
P14075	P28072	0.2964
P14075	Q5T035	0.59825
P14075	Q9NWV4	0.69265
P14075	Q86VX2	0.70598
P14075	Q9P2F5	0.39889
P14075	P55197	1.0537
P14075	Q9UJK0	0.97908
P14075	P78317	0.45463
P14075	A6NEA5	0.27516
P14075	Q5FBB7	0.58759
P14075	P0CG35	0.49457
P14075	P18848	0.81675
P14075	O75360	0.28149
P14075	Q9P2J5	0.25341
P14075	O43711	0.22878
P14075	Q5VZT2	0.15467
P14075	Q969F9	0.84309
P14075	Q9UBP6	0.84663
P14075	Q53EL6	0.7158
P14075	P80294	0.46119
P14075	Q9NWQ4	0.86088
P14075	Q9NRR4	0.30512
P14075	Q9UMQ3	0.44155
P14075	Q9H147	0.49027
P14075	Q99549	0.46349
P14075	Q96B26	0.50891
P14075	Q13123	0.8566
P14075	A6NKF2	0.33062
P14075	Q6ZSV7	0.15192
P14075	O14737	0.35973
P14075	Q9H8H0	0.17936
P14075	P51580	0.18635
P14075	Q9BTA0	0.94025
P14075	Q9UMS4	0.41661
P14075	B3EWG3	0.94025
P14075	Q96JK9	0.69611
P14075	Q9ULW8	0.40915
P14075	Q86UT8	1.1439
P14075	Q969V6	1.0558
P14075	Q6ZSN1	0.54871
P14075	P09661	0.45549
P14075	O60356	0.80509
P14075	Q96LP2	0.070363
P14075	Q9UIG4	0.73547
P14075	Q6ZVN6	0.2034
P14075	Q15436	0.3937
P14075	Q8IYB3	0.54511
P14075	Q8NAB5	0.63821
P14075	Q92796	0.20297
P14075	Q9Y5M1	0.31541
P14075	Q01094	0.62848
P14075	Q4V321	0.079883
P14075	Q5XKR9	0.91245
P14075	O14639	0.28571
P14075	Q6ZN79	0.28942
P14075	Q9UKV8	0.60319
P14075	Q9BTL3	0.63721
P14075	Q5RGN0	0.455
P14075	P15502	0.40048
P14075	O14978	0.21808
P14075	P28370	0.27924
P14075	Q9BWJ5	0.33329
P14075	Q8TA86	0.52105
P14075	Q96LY2	0.72258

P14075	Q8NEQ6	0.16681
P14075	P61586	1.0001
P14075	Q9NZM4	1.0553
P14075	Q5D1E8	0.52531
P14075	Q9NRR5	0.39086
P14075	Q14258	0.72809
P14075	Q13536	0.63821
P14075	Q9NU23	0.4651
P14075	Q5VU69	0.66421
P14075	Q9BUX1	0.5045
P14075	P62841	0.46093
P14075	Q15773	0.88418
P14075	Q96AH0	0.34548
P14075	H0YL14	0.58154
P14075	Q9Y333	0.49091
P14075	Q6PB30	0.69844
P14075	P10515	0.24735
P14075	Q5VTE6	0.020626
P14075	Q8IYS4	0.064867
P14075	O96007	0.68574
P14075	Q86X83	0.44051
P14075	P36578	0.76792
P14075	Q5JNZ5	0.33115
P14075	A0AUK8	0.19993
P14075	Q9BUR4	0.56535
P14075	Q8N4H0	0.38083
P14075	Q9BVS5	0.18618
P14075	P22061	0.40874
P14075	Q13487	1.2523
P14075	Q8NHY2	0.71553
P14075	P08651	0.10543
P14075	Q5VT33	0.25759
P14075	Q9H0U9	0.27969
P14075	P43364	0.55947
P14075	P61978	0.74749
P14075	O15105	0.19166
P14075	Q9Y3A3	0.31251
P14075	P14921	0.60432
P14075	P0CW26	0.28911
P14075	Q8NAV1	0.87308
P14075	O95999	0.093946
P14075	P62266	0.71048
P14075	Q9BQ95	0.77137
P14075	Q9GZS3	0.85774
P14075	P50452	0.37761
P14075	Q9H4H8	0.21261
P14075	Q14397	0.48039
P14075	Q9H4R4	0.0080065
P14075	Q99496	0.28868
P14075	P36402	0.37945
P14075	P48728	0.17209
P14075	Q92572	0.25661
P14075	Q9Y2L8	0.35147
P14075	P30304	0.3727
P14075	Q9GZL8	0.49705
P14075	Q8N831	0.33384
P14075	Q3MIV0	0.63821
P14075	P06400	0.50919
P14075	Q13794	0.38625
P14075	Q8N801	0.031357
P14075	Q8N485	0.17163
P14075	P83916	0.50876
P14075	Q07352	0.66288
P14075	P33316	0.41306
P14075	O43542	0.30374
P14075	Q8N402	0.0054577
P14075	A8MWA4	0.38483
P14075	Q7Z422	0.20644
P14075	Q9BRT9	0.38643
P14075	Q494R0	0.63821
P14075	Q5M9Q1	0.72201
P14075	Q8N9Z0	0.26879
P14075	Q96EU6	0.32
P14075	Q5VSR9	0.88201
P14075	O14593	0.43088
P14075	Q9NWZ8	0.58104

P14075	P02511	0.21985
P14075	Q66K41	0.54693
P14075	Q9BSG1	0.23704
P14075	Q8NBR6	1.2358
P14075	Q3LI67	0.63821
P14075	Q5VWT5	0.54983
P14075	E9PB15	0.11311
P14075	Q9UBI1	0.1668
P14075	P82932	0.23783
P14075	Q9BZ23	0.60255
P14075	P50453	0.40718
P14075	Q9NVR5	0.68945
P14075	Q5TC12	0.31993
P14075	Q9NWB7	0.39036
P14075	Q8N7Y1	0.63821
P14075	Q9Y5Q8	0.43554
P14075	A8MTZ7	0.21366
P14075	P0C7P2	0.28438
P14075	Q9H0P7	0.22583
P14075	Q6IQ32	0.35268
P14075	Q8N6G1	0.63821
P14075	Q15935	0.22477
P14075	Q494V2	0.18051
P14075	Q9UIL1	0.36264
P14075	Q8IVT2	0.86866
P14075	P0C867	0.43947
P14075	Q13625	0.28966
P14075	Q69YW0	0.35419
P14075	Q9H6U6	0.52644
P14075	Q99766	0.51761
P14075	O15287	0.623
P14075	Q9H410	0.5989
P14075	Q96AV8	0.20687
P14075	Q8NEL0	0.096144
P14075	Q6ZQT7	0.63821
P14075	Q8WU58	0.49328
P14075	O95149	0.25249
P14075	Q9NV29	0.62601
P14075	Q13867	0.12873
P14075	Q15053	0.1524
P14075	A0PJX2	0.41618
P14075	Q15654	0.57212
P14075	Q03112	0.15085
P14075	Q02078	0.30731
P14075	Q13426	0.15347
P14075	P55789	0.091278
P14075	Q15633	0.60568
P14075	Q5U649	0.23425
P14075	Q9UBB6	0.41786
P14075	Q9Y4W2	0.82098
P14075	Q8NDH2	0.029727
P14075	Q9Y3R5	0.46883
P14075	Q5TA81	0.63821
P14075	O94986	0.80035
P14075	Q9NP66	0.75338
P14075	P52743	0.23429
P14075	Q8N4L8	0.23535
P14075	P62714	0.41756
P14075	Q5TI25	1.0145
P14075	Q9BTD1	0.20194
P14075	Q5JTC6	0.81782
P14075	Q9UK28	0.72793
P14075	B1AMM8	0.24609
P14075	Q8N988	0.34111
P14075	Q9BZE2	0.80363
P14075	P61758	0.35409
P14075	O60832	0.4935
P14075	Q6ZN28	0.60732
P14075	P24539	0.1252
P14075	Q9Y262	0.56261
P14075	A8MUV8	0.28249
P14075	O43913	0.27382
P14075	A8MVS1	0.32244
P14075	P27348	0.19417
P14075	Q9UPZ3	0.44634
P14075	Q9NRR3	0.29123

P14075	Q8IXH6	0.82345
P14075	A6NGQ2	0.31936
P14075	Q8IV56	0.85155
P14075	P41208	0.36431
P14075	P17028	0.50392
P14075	Q9H9Y2	0.025203
P14075	P27694	0.36152
P14075	Q12824	0.80995
P14075	Q9NTK1	0.68952
P14075	Q7L0X2	0.48992
P14075	Q6ZUA9	0.64233
P14075	P14316	0.6874
P14075	Q6UWF3	0.54696
P14075	P0DKL9	0.74444
P14075	P50549	0.69326
P14075	Q15628	0.3684
P14075	Q13228	0.78166
P14075	P26998	0.65175
P14075	O14896	0.30465
P14075	Q9NXX6	0.13588
P14075	Q15742	0.18237
P14075	P43356	0.67054
P14075	Q06330	0.39658
P14075	Q9NX40	0.26588
P14075	Q15233	0.44501
P14075	Q9Y2G9	0.64817
P14075	Q9Y324	0.77973
P14075	Q9UIF8	0.57965
P14075	P0C7M3	0.41629
P14075	P67775	0.45886
P14075	Q8NEM7	0.14882
P14075	Q9H2U2	0.238
P14075	Q8NEG4	0.43563
P14075	Q5VIR6	0.47909
P14075	Q8N4M7	0.90788
P14075	Q5H9L4	0.18315
P14075	Q6ZR85	0.63821
P14075	Q5T2W1	0.27497
P14075	Q6ICG6	0.41123
P14075	Q8WY41	0.2842
P14075	P60328	0.17004
P14075	Q9H9Y6	0.49609
P14075	O94805	0.55123
P14075	P0C7V6	0.54549
P14075	O95073	0.79563
P14075	Q5VWP2	0.27983
P14075	A8MX80	0.51179
P14075	O94829	0.75093
P14075	Q96G21	0.61496
P14075	Q13347	0.44281
P14075	P08670	0.10218
P14075	P60896	0.63821
P14075	P22528	0.35546
P14075	Q9BVL4	0.97036
P14075	Q13094	0.33414
P14075	Q5BKU6	0.17117
P14075	A8MYP8	0.74097
P14075	P30153	0.2989
P14075	P51946	0.48539
P14075	Q96FZ2	0.7675
P14075	E9PQ53	0.65415
P14075	Q8IYA6	0.73454
P14075	Q9BQY9	0.27791
P14075	P53370	0.27623
P14075	Q9C002	0.1514
P14075	Q3C1V8	0.39708
P14075	Q9NS26	0.91631
P14075	P63000	0.35269
P14075	Q8N7Z2	0.36542
P14075	Q8N1V8	0.22537
P14075	Q96A22	0.45185
P14075	O60826	0.5817
P14075	Q9NUP7	0.67342
P14075	Q6NVU6	0.27079
P14075	Q14997	0.48236
P14075	Q9BQ69	0.27782

P14075	Q9UQ80	0.8544
P14075	Q9H3J6	0.91546
P14075	Q7Z2W4	0.26036
P14075	Q8IYL3	1.0292
P14075	Q6NSI4	0.26844
P14075	Q8WVI0	0.78884
P14075	Q969E4	0.21325
P14075	Q9BX70	0.34768
P14075	H3BTG2	0.56675
P14075	Q96RU7	0.30587
P14075	P52594	0.26061
P14075	Q9HAA7	0.061461
P14075	Q8IUC6	0.093042
P14075	Q6ZVH6	0.035295
P14075	O75436	0.42111
P14075	P61513	0.38314
P14075	Q9ULD0	0.16037
P14075	O75822	0.68574
P14075	O60477	0.37929
P14075	Q8N2N9	0.42835
P14075	Q8NEM2	0.50133
P14075	Q15154	0.40968
P14075	Q9NV56	0.72379
P14075	Q9NZI6	0.30061
P14075	O14879	0.51645
P14075	Q8IX90	0.40584
P14075	Q6PCB5	0.39752
P14075	P28749	1.0231
P14075	P32780	0.55006
P14075	Q9GZR5	0.29572
P14075	O00161	0.62814
P14075	O95163	0.51443
P14075	Q96PV6	0.41358
P14075	P09012	0.25914
P14075	Q9Y6P5	0.31389
P14075	O75896	0.68355
P14075	P55199	0.78301
P14075	O15379	0.40605
P14075	Q8ND99	0.36191
P14075	Q9UK10	0.2118
P14075	Q1RN00	0.63821
P14075	P78386	0.70038
P14075	Q6ZRP5	0.63821
P14075	Q8WWM7	0.39699
P14075	Q66K89	0.65026
P14075	Q68CJ9	0.44894
P14075	Q6ZMS7	0.17594
P14075	O43504	0.19466
P14075	P61313	0.72715
P14075	P98168	0.48913
P14075	Q8NF99	0.13223
P14075	Q9P1Q0	0.269
P14075	Q7Z7K6	0.52554
P14075	Q8TC71	0.30708
P14075	Q9Y3C0	0.18353
P14075	Q9H9L3	0.21939
P14075	P27635	0.36773
P14075	A6NKF1	0.68578
P14075	Q9ULK4	1.0144
P14075	Q8TEK3	0.19405
P14075	Q5VYV7	0.1668
P14075	P01100	0.4089
P14075	P02812	0.59508
P14075	Q9BRQ6	0.92739
P14075	Q8TDB4	0.22267
P14075	A8K8V0	0.24018
P14075	C9JC47	0.63821
P14075	Q8IYL2	0.52238
P14075	Q8NFH3	0.74228
P14075	O60921	0.54578
P14075	P17010	0.084994
P14075	Q96DM1	0.61563
P14075	A7MD48	0.73002
P14075	Q9ULE6	0.38432
P14075	Q86Y97	0.28151
P14075	Q8NI29	0.26079

P14075	Q14134	0.40861
P14075	Q9GZU7	0.29175
P14075	O00268	0.83526
P14075	P14678	0.58821
P14075	Q14789	0.2567
P14075	Q96MC4	0.81412
P14075	Q8NDT2	0.6864
P14075	Q9BRJ6	0.61711
P14075	A6NK44	0.036782
P14075	B1AL46	0.72275
P14075	Q96LX7	0.024215
P14075	Q9UNL4	0.22766
P14075	Q02447	0.34602
P14075	Q9HD64	0.21489
P14075	Q8IWC1	0.29733
P14075	Q6ZWC4	0.36956
P14075	P0C7V9	0.86595
P14075	Q96A73	0.86895
P14075	Q9UNN4	0.20958
P14075	P0DJ07	0.78884
P14075	O15525	0.19127
P14075	O95602	0.42171
P14075	O75792	0.79225
P14075	Q86YG4	0.48126
P14075	Q9BYQ0	0.78356
P14075	Q5U4N7	0.63821
P14075	Q9Y222	0.7218
P14075	O95059	0.41724
P14075	Q9BQ89	0.99111
P14075	Q9BZE7	0.94025
P14075	Q8NDY4	0.63821
P14075	Q5PRF9	0.34964
P14075	Q9UEE9	0.56241
P14075	Q9UJX4	0.51501
P14075	O43447	0.4168
P14075	Q9NSG2	0.57186
P14075	Q6B0B8	0.39689
P14075	Q8IWP9	0.96625
P14075	O75190	0.52682
P14075	Q3BBV2	0.455
P14075	Q6ZRS4	0.7363
P14075	Q9H633	0.19873
P14075	P63010	0.21045
P14075	Q5T1B1	0.91576
P14075	Q5H9B9	0.29235
P14075	P60369	0.78356
P14075	Q9H009	0.15804
P14075	Q9H900	0.31244
P14075	Q5EBL4	0.22683
P14075	Q53EZ4	0.42969
P14075	Q8NF67	0.57736
P14075	Q9UHQ1	0.55643
P14075	Q6UXP9	0.073276
P14075	Q0VDE8	0.59765
P14075	Q8WY54	0.53107
P14075	Q9Y534	0.13189
P14075	O94925	0.29495
P14075	Q9BZE4	0.1772
P14075	P62888	0.48349
P14075	Q8TDH9	0.085214
P14075	O76009	0.70708
P14075	Q3BBV1	1.0145
P14075	O43310	0.28379
P14075	Q13439	0.56939
P14075	Q6P1L5	1.2136
P14075	Q8TCS8	0.13476
P14075	Q86YE8	0.2118
P14075	O94952	0.64941
P14075	Q9H4M3	0.11282
P14075	Q96Q06	0.53449
P14075	Q9HCS7	0.26169
P14075	Q5HYN5	0.20293
P14075	Q5T5F5	0.63821
P14075	Q96QU8	0.75112
P14075	Q9Y4R8	0.22617
P14075	P23508	0.20328

P14075	Q96BR6	0.26816
P14075	Q9BRK4	0.32004
P14075	P02795	0.41477
P14075	Q96H79	0.49406
P14075	Q9UHN1	0.052572
P14075	Q4KMZ1	0.26664
P14075	Q5HY98	0.26969
P14075	O60885	0.46831
P14075	Q53HL2	0.67058
P14075	P00338	0.32299
P14075	P20290	0.8435
P14075	Q9UHY8	0.22525
P14075	Q9Y2X9	0.44825
P14075	P15621	0.28266
P14075	O95751	0.45927
P14075	O94817	0.31618
P14075	Q9UFF9	0.29692
P14075	Q9H9T3	0.59427
P14075	Q9H7E2	0.18883
P14075	Q05084	0.17836
P14075	Q29RF7	0.74862
P14075	Q6ZV50	0.073272
P14075	Q9UK58	0.16372
P14075	A6NE82	0.030285
P14075	Q7L2K0	0.21061
P14075	Q9Y3T9	0.81422
P14075	O15063	0.45435
P14075	Q9HAH7	0.85055
P14075	Q9ULZ2	0.49589
P14075	Q9Y678	0.3122
P14075	Q5T1J5	0.87053
P14075	Q6ZPB5	0.63821
P14075	Q9P2T0	0.50109
P14075	Q15513	0.63821
P14075	Q5H9S7	0.73839
P14075	Q6P2C8	0.74735
P14075	Q9H693	0.51711
P14075	Q49AA0	0.26988
P14075	Q9NRD1	0.050819
P14075	Q9H0U6	0.35582
P14075	Q9UKY7	0.94894
P14075	Q9H3H3	0.27954
P14075	O43159	0.66304
P14075	Q9P1A6	0.137
P14075	Q92901	0.38311
P14075	P24522	0.36696
P14075	A2IDD5	0.45258
P14075	A4D263	0.38686
P14075	Q13342	0.41477
P14075	P20809	0.36992
P14075	Q7L190	0.77412
P14075	Q96EV8	0.11719
P14075	Q9NZI5	0.25118
P14075	Q5T9C2	0.94025
P14075	Q9H5L9	0.18644
P14075	Q9NV92	0.12533
P14075	P0CG47	0.27823
P14075	Q5SYE7	0.66542
P14075	Q8NEK8	0.86595
P14075	Q9UNS2	0.42101
P14075	Q9H098	0.94799
P14075	O95394	0.57725
P14075	Q9BXF6	0.38603
P14075	Q9H1E3	0.76615
P14075	P62937	0.45262
P14075	Q7Z4T9	0.45364
P14075	P98182	0.056799
P14075	Q9BRR8	0.72435
P14075	P0C7V5	0.22798
P14075	Q6PGN9	0.62615
P14075	P07197	0.66016
P14075	Q8NAJ2	0.027144
P14075	Q13330	0.25779
P14075	Q96B01	0.57446
P14075	P58511	0.76323
P14075	A9QM74	0.27061

P14075	Q6ZRH3	0.37498
P14075	Q5T6M2	0.85677
P14075	P61583	0.66896
P14075	Q6P2I7	0.18724
P14075	Q8IWL8	0.83317
P14075	O95478	0.48791
P14075	P42772	0.58894
P14075	O15504	0.31888
P14075	Q9BSQ5	0.26604
P14075	Q494X1	0.17209
P14075	Q9BXG8	0.22712
P14075	Q9ULG3	0.63821
P14075	Q8N6M0	0.10757
P14075	Q8NCD3	0.34757
P14075	Q5T230	1.043
P14075	Q8IXM3	0.46453
P14075	Q9UL42	0.57654
P14075	A6NEL3	0.042213
P14075	Q9NVX0	0.68544
P14075	Q9H7P6	0.75985
P14075	P12980	0.58625
P14075	P09972	0.37476
P14075	Q6P387	0.39937
P14075	P49711	0.13498
P14075	Q9Y383	0.46142
P14075	P49789	0.24993
P14075	Q96LR2	0.92375
P14075	P25787	0.46127
P14075	Q13428	0.93619
P14075	Q96LW9	0.11557
P14075	Q9BZK7	0.71351
P14075	P15259	0.50609
P14075	Q9P2D6	0.050567
P14075	Q96H40	0.28445
P14075	Q96T23	0.48513
P14075	Q00577	0.39835
P14075	Q99547	0.70398
P14075	Q8TBY8	0.41375
P14075	Q32MQ0	0.44828
P14075	P26639	0.31923
P14075	Q9BWC9	1.0184
P14075	Q5C9Z4	0.48053
P14075	A6NGY3	0.34057
P14075	Q96QF0	0.34856
P14075	O00712	0.080964
P14075	Q96N20	0.11815
P14075	Q8WZ33	0.52502
P14075	Q92793	0.31292
P14075	Q9UDY2	0.59211
P14075	Q7RTS7	0.23689
P14075	Q9BYE4	0.63821
P14075	P08107	0.38424
P14075	Q9BYE3	0.63821
P14075	Q96LI6	0.25802
P14075	P28702	0.26666
P14075	P15884	0.45264
P14075	Q9BTY7	0.33592
P14075	Q69YI7	0.94197
P14075	Q8N7W2	0.46184
P14075	Q96MK2	0.25765
P14075	O14832	0.41074
P14075	Q7L5N1	0.77003
P14075	P54725	0.3493
P14075	Q717R9	0.56424
P14075	O60925	0.12972
P14075	Q9NVC6	0.42932
P14075	Q9H6V9	0.45108
P14075	Q9UNS1	0.41276
P14075	E9PJ23	0.025923
P14075	Q13416	0.34229
P14075	P0CL83	0.45967
P14075	A8MUL3	0.63821
P14075	Q8WYA6	0.52564
P14075	Q9UKW6	0.27014
P14075	Q92841	0.47154
P14075	A6NJ08	0.030285

P14075	Q8N6M3	0.5651
P14075	P50748	0.5426
P14075	Q5SZD1	0.32465
P14075	Q9Y5S9	0.59226
P14075	O95447	0.15403
P14075	P35268	0.51122
P14075	P78352	0.41926
P14075	Q9UI36	0.31977
P14075	Q86YA3	0.92208
P14075	Q9BVI4	0.35384
P14075	Q13772	0.15379
P14075	Q5TA78	0.63821
P14075	Q86WR0	0.43671
P14075	Q9BYN8	0.92056
P14075	Q8TC90	0.90896
P14075	P0C843	0.37873
P14075	Q14695	0.46496
P14075	O43463	0.60668
P14075	Q6ULP2	0.49883
P14075	O95376	0.23609
P14075	P13647	0.63715
P14075	Q8TF63	0.6364
P14075	Q8N0U4	0.40571
P14075	P0CZ25	0.32866
P14075	Q9UH99	0.065692
P14075	Q6UUV7	1.1714
P14075	Q14674	0.14354
P14075	Q8N8P6	0.31752
P14075	Q16526	0.23693
P14075	Q9Y3B6	0.70595
P14075	Q5R3I4	0.26896
P14075	Q8TF47	0.28409
P14075	P38117	0.25804
P14075	P18754	0.83802
P14075	Q13404	0.77988
P14075	Q14137	0.60882
P14075	Q9NWM3	0.25521
P14075	Q5U5X0	0.79949
P14075	P16383	0.85226
P14075	Q8N4C0	0.2228
P14075	Q15468	0.42874
P14075	Q9NR09	0.28303
P14075	Q96PQ6	0.32094
P14075	Q9UGK8	0.44438
P14075	O75843	0.40756
P14075	Q15699	0.31828
P14075	P35080	0.19009
P14075	Q6ZR93	0.63821
P14075	O95243	0.42565
P14075	Q96KN1	0.22673
P14075	P49247	0.55775
P14075	Q96C86	0.58927
P14075	Q8TAC9	0.31211
P14075	Q6ZW54	0.92676
P14075	Q8IUG1	0.81551
P14075	P46937	0.61607
P14075	Q6P9G9	0.11305
P14075	Q8ND76	0.38174
P14075	Q2T9L4	0.37832
P14075	Q96HZ7	0.63821
P14075	Q53QW1	0.94025
P14075	Q8N8D9	0.22202
P14075	Q13127	0.35437
P14075	Q9UBS8	0.48333
P14075	O75113	0.60994
P14075	Q86UD0	0.35269
P14075	Q5CZC0	1.0153
P14075	Q8IW36	0.28368
P14075	P35226	0.41368
P14075	Q9UF83	0.27185
P14075	Q96PY6	0.26661
P14075	Q9Y294	0.73443
P14075	Q8IWA6	0.37633
P14075	Q3LHN2	0.63821
P14075	Q9H3M9	0.36872
P14075	Q9HD42	0.73346

P14075	Q9NR50	0.33026
P14075	Q8N5J2	1.0048
P14075	P31314	0.43238
P14075	Q9UKN8	0.45968
P14075	Q32MZ4	0.8333
P14075	O75192	0.46979
P14075	Q96B67	0.14837
P14075	Q3MJ62	0.10668
P14075	Q9BYZ2	0.32299
P14075	A6NIH7	0.27782
P14075	Q8N398	0.32487
P14075	Q9BRG1	0.74081
P14075	Q9Y2P7	0.25605
P14075	Q5SY85	0.48498
P14075	Q96RN5	1.0418
P14075	Q9BZS9	0.63821
P14075	P48552	0.48956
P14075	Q6ZSG2	0.59415
P14075	Q99590	0.38222
P14075	Q14533	0.70038
P14075	Q9UDV6	0.3617
P14075	O75486	0.49901
P14075	Q96HG1	0.63821
P14075	P61581	0.65433
P14075	Q13503	0.45634
P14075	Q8TF39	0.31207
P14075	Q5BKY9	0.94025
P14075	Q9UHB9	0.13838
P14075	Q6ICC9	0.45104
P14075	Q969I3	0.36669
P14075	Q7L5D6	0.78768
P14075	Q9NXF7	0.19062
P14075	P60409	0.78356
P14075	Q96CN0	0.63821
P14075	Q6TGC4	0.38586
P14075	O43439	0.70908
P14075	P31276	0.41428
P14075	Q8WWK9	0.26363
P14075	P62945	0.63821
P14075	E9PJI5	0.67697
P14075	Q3LI70	0.63821
P14075	Q99567	0.54894
P14075	O43715	0.61947
P14075	Q05516	0.52347
P14075	Q8N6Y0	0.38695
P14075	Q96CV9	0.30502
P14075	Q6ZRP0	0.94025
P14075	Q9NP64	0.046073
P14075	O43325	0.78056
P14075	P56377	0.12861
P14075	P35637	0.25922
P14075	Q6PI48	0.57509
P14075	Q4VC05	0.83555
P14075	Q8N9V7	0.52936
P14075	Q9Y5L0	0.69192
P14075	Q9HBM0	0.55609
P14075	A6NKD9	0.94766
P14075	Q9H0C5	0.35261
P14075	O60524	0.84128
P14075	Q15022	0.43419
P14075	O60711	0.2555
P14075	O75884	0.99402
P14075	Q6ZRH9	0.63821
P14075	Q9NRC8	0.94914
P14075	Q9Y2S6	0.63821
P14075	Q9BYG5	0.41654
P14075	P04731	0.30226
P14075	Q9Y5J7	0.11634
P14075	Q8WUH2	0.43141
P14075	Q6NW29	0.42739
P14075	Q9BZF9	0.37662
P14075	P46776	0.72715
P14075	I6L9F6	0.056889
P14075	Q9UG56	0.20649
P14075	Q15466	0.41869
P14075	Q8N998	0.099575

P14075	P19838	0.55066
P14075	P0CZ20	0.28911
P14075	Q96F63	0.94025
P14075	Q9H9F9	0.85732
P14075	Q8NHR7	0.16132
P14075	Q96AY2	0.63042
P14075	B1ANH7	0.030093
P14075	Q96HY6	0.32038
P14075	Q5SXJ2	1.0145
P14075	Q9BYR4	0.78356
P14075	Q86XK2	0.47966
P14075	P60014	1.0211
P14075	O60504	0.38345
P14075	Q7Z7J5	0.7988
P14075	Q9UQL6	0.33004
P14075	O95873	0.94025
P14075	Q3KNT7	0.10425
P14075	Q12769	0.78318
P14075	Q6ZS52	0.63821
P14075	P60002	0.85228
P14075	Q03060	0.19283
P14075	Q04724	0.6551
P14075	Q9H8K7	0.47403
P14075	Q9H3C7	1.022
P14075	Q8NBC4	0.63821
P14075	Q2VPB7	0.47631
P14075	Q53QV2	0.35613
P14075	Q6ZN06	0.28445
P14075	Q99741	0.39368
P14075	P11168	0.20732
P14075	P07196	0.54521
P14075	Q96F24	0.44871
P14075	Q15011	0.63315
P14075	Q8IUF8	0.18723
P14075	Q96GC6	0.85282
P14075	Q96EH5	0.53684
P14075	P09001	0.46738
P14075	P10074	0.16061
P14075	O94927	0.32838
P14075	Q7RTT6	0.37226
P14075	Q86UA6	0.85283
P14075	Q9H0W8	0.4582
P14075	Q9HCD5	0.79563
P14075	Q9Y3Q8	1.0044
P14075	P27469	0.56985
P14075	Q14749	0.38716
P14075	Q96FX7	0.17082
P14075	Q9NQ48	0.12647
P14075	Q3Y452	0.63821
P14075	Q8NFD5	0.62335
P14075	Q8IYI6	0.40827
P14075	Q9GZZ8	0.63821
P14075	P48788	0.19778
P14075	Q9UJA5	0.87023
P14075	Q8IYS8	0.47573
P14075	Q9NY74	0.99467
P14075	Q9UL40	0.40862
P14075	O95273	0.68254
P14075	P83876	0.73141
P14075	A2RTY3	0.1312
P14075	Q12888	0.47997
P14075	Q13129	0.47987
P14075	Q96EI5	0.56374
P14075	Q09FC8	0.16686
P14075	Q9BYD3	0.72005
P14075	Q8NFC6	0.8161
P14075	Q8N377	0.63821
P14075	Q9UL18	0.74231
P14075	Q96T59	0.41629
P14075	Q8TBR4	0.30467
P14075	O60810	0.44462
P14075	Q8NA03	0.62891
P14075	Q9H981	0.78119
P14075	P54259	0.66314
P14075	Q9BZW7	0.20797
P14075	Q8IWE2	0.82308

P14075	Q14678	0.3233
P14075	O75665	0.43032
P14075	Q15024	0.46092
P14075	P56270	0.37206
P14075	Q99502	0.027493
P14075	Q9Y2H0	0.23671
P14075	P35789	0.24999
P14075	O15266	0.35951
P14075	Q00653	0.90917
P14075	Q8IU53	0.56554
P14075	Q6IC83	0.066774
P14075	Q9P260	0.24968
P14075	Q9H379	0.67365
P14075	Q6ZSR3	0.63821
P14075	Q13427	0.20514
P14075	O60304	0.13387
P14075	A8MUQ0	0.74791
P14075	Q8WW14	0.18927
P14075	Q96N38	0.28795
P14075	Q9HBM1	0.46352
P14075	P16220	0.66491
P14075	Q5UIP0	0.74341
P14075	Q0VD86	0.11945
P14075	Q93073	0.6126
P14075	Q5T0J3	0.24794
P14075	Q15361	0.51853
P14075	P31946	0.38879
P14075	Q53FN2	0.045219
P14075	Q92558	0.1575
P14075	P40305	0.58798
P14075	O43791	0.36452
P14075	Q32M92	0.00033455
P14075	P17022	0.088158
P14075	Q5MJ07	0.88201
P14075	Q8NDA8	0.58658
P14075	O75934	0.47359
P14075	Q92597	0.25557
P14075	Q9ULE0	0.057317
P14075	Q6ZRC1	0.63821
P14075	Q9HBF5	0.73711
P14075	Q5JXC2	0.25426
P14075	P84103	0.43188
P14075	Q96I59	0.41523
P14075	Q5JQS6	0.33667
P14075	P54278	0.17177
P14075	P20618	0.47769
P14075	Q9BQC3	0.7718
P14075	Q7RTT3	0.049323
P14075	Q96HE9	0.11378
P14075	Q8NE00	0.75184
P14075	Q9HCU8	0.46416
P14075	Q5SQH8	0.34642
P14075	A8MUU9	0.11715
P14075	Q9H1A4	0.49416
P14075	Q7KZF4	0.5742
P14075	Q5VWM6	0.83358
P14075	P56211	0.32858
P14075	G3V211	0.75421
P14075	Q96M85	0.63821
P14075	O76042	0.50855
P14075	Q969W0	0.43049
P14075	Q8N448	0.24944
P14075	Q86VS8	0.23067
P14075	Q6P1K2	0.86928
P14075	Q99700	0.54587
P14075	Q8NCR6	0.45483
P14075	Q9Y6K9	0.61734
P14075	P13807	0.21003
P14075	P56385	0.30705
P14075	F5GYI3	0.9288
P14075	Q969W8	0.25517
P14075	Q569K6	0.046082
P14075	Q9GZZ1	0.28076
P14075	P24385	0.39817
P14075	Q9BXP5	0.82064
P14075	A6NLC5	0.61887

P14075	O43261	0.17224
P14075	Q6P1W5	0.46868
P14075	Q9NS71	0.63238
P14075	Q49A33	0.27331
P14075	Q13363	0.31544
P14075	O94855	0.1654
P14075	P62256	0.11125
P14075	P63146	0.16118
P14075	Q8WUZ0	0.82681
P14075	Q7Z794	0.72566
P14075	Q6ZV80	0.2813
P14075	Q9UKI9	0.99687
P14075	O43148	0.72694
P14075	Q8WVK2	0.85021
P14075	Q8NHX4	0.84807
P14075	Q9H4K1	0.66429
P14075	P0DI83	0.46369
P14075	Q86W56	0.33308
P14075	Q9BXS5	0.46153
P14075	O75154	0.5128
P14075	O95678	0.53972
P14075	Q6NUN7	0.63821
P14075	Q9HB65	0.3402
P14075	Q7Z745	0.35465
P14075	Q5TA31	0.30561
P14075	Q9H3P2	0.85132
P14075	P56277	0.86523
P14075	Q9Y473	0.13982
P14075	P52758	0.21757
P14075	Q6P9B9	0.61175
P14075	Q9BQE9	0.896
P14075	O76011	0.80046
P14075	Q9ULJ6	0.27776
P14075	A6NJJ6	0.94025
P14075	Q8TB69	0.27773
P14075	Q8N1D5	0.33525
P14075	Q9ULK2	0.48825
P14075	Q8IWN6	0.45146
P14075	P10124	0.40275
P14075	Q9NPB6	0.43672
P14075	Q9Y6Z2	0.63821
P14075	Q96LW1	0.28409
P14075	P13010	0.20384
P14075	P61962	0.47179
P14075	Q9NRA8	0.71859
P14075	Q03923	0.31975
P14075	Q9UBU9	0.28899
P14075	P35900	0.80046
P14075	P46783	1.0088
P14075	Q75L30	0.63821
P14075	Q9BXW3	0.63821
P14075	P41970	0.65558
P14075	Q7Z7H8	0.091521
P14075	Q8WVM0	0.41095
P14075	Q12894	0.34215
P14075	Q96GX2	0.75906
P14075	Q15329	0.97588
P14075	P48200	0.19927
P14075	Q9P287	0.44431
P14075	Q8IVN3	0.73981
P14075	Q14974	0.75038
P14075	O95613	0.45845
P14075	O75529	0.39315
P14075	O95685	0.23444
P14075	P0C852	0.63821
P14075	Q9BYR7	0.91867
P14075	Q5TA77	0.63821
P14075	Q9P209	0.49555
P14075	Q14203	0.62011
P14075	Q9GZN7	0.53989
P14075	Q96DZ1	0.13728
P14075	P81877	0.30497
P14075	O95125	0.13203
P14075	P56282	0.24219
P14075	P22415	0.35483
P14075	Q8NFI3	0.36773

P14075	Q96M29	0.42716
P14075	Q96NS1	0.15812
P14075	Q4U2R6	0.68898
P14075	Q5VZ46	0.37083
P14075	Q9UBS4	0.21941
P14075	P62701	0.29185
P14075	Q86WP2	0.54598
P14075	Q92985	0.37021
P14075	Q9UMS6	0.34709
P14075	Q0VG06	0.31138
P14075	P0C5J1	0.049779
P14075	Q9H607	0.24482
P14075	Q8NBZ9	0.44967
P14075	Q96DE9	0.04993
P14075	P07741	0.35148
P14075	A6NDB9	0.63116
P14075	P84022	0.37919
P14075	Q9BVL2	0.43872
P14075	Q86V71	0.21527
P14075	Q8NFP0	0.7202
P14075	Q6NVH7	0.52696
P14075	Q5HYJ3	0.54061
P14075	P59998	0.14134
P14075	Q13144	0.33322
P14075	Q15003	0.6329
P14075	Q96JG6	0.10058
P14075	Q96AT1	0.94025
P14075	P21980	0.14167
P14075	Q5XKP0	0.31005
P14075	P60372	0.78356
P14075	Q6ZUJ4	0.79138
P14075	Q9HAN9	0.20588
P14075	Q8N3X6	0.62135
P14075	Q8NI08	0.56609
P14075	Q96E40	0.019759
P14075	Q9H3F6	0.24788
P14075	Q9H3H9	0.7023
P14075	Q9NP92	0.85025
P14075	Q13070	0.079883
P14075	Q53HI1	0.49328
P14075	Q96AN2	0.30215
P14075	Q9BYP8	0.63821
P14075	P25815	0.29241
P14075	P49790	0.77638
P14075	Q9NVV5	0.62263
P14075	Q86VY4	0.30246
P14075	Q9UER7	0.68243
P14075	Q6ZQP7	0.63821
P14075	Q99816	0.28392
P14075	O60437	0.46214
P14075	Q96DN6	0.74462
P14075	P15407	0.45471
P14075	Q13352	0.24109
P14075	Q8IZL8	0.9411
P14075	Q16621	0.38174
P14075	Q9UL33	0.24845
P14075	Q9BYR2	0.78356
P14075	Q9H4B0	0.6929
P14075	Q5JRC9	0.78694
P14075	Q9Y3M2	0.57583
P14075	Q96S55	0.27999
P14075	Q9H1B4	0.23695
P14075	Q9H7S9	0.53442
P14075	Q9BZV1	0.8729
P14075	P23760	0.61384
P14075	Q9BYC8	0.51798
P14075	Q6J272	0.85481
P14075	Q07866	0.29704
P14075	Q9H0G5	0.40519
P14075	Q496H8	0.58096
P14075	O43309	0.1558
P14075	P62875	0.2924
P14075	Q14008	0.6109
P14075	Q02833	0.46054
P14075	O14599	0.63821
P14075	Q12972	0.62573

P14075	Q9H5V9	0.87411
P14075	Q99583	0.24278
P14075	P49589	0.38017
P14075	Q9P2M7	0.26775
P14075	O00479	0.63115
P14075	Q5VTQ0	0.19586
P14075	Q6V702	0.16586
P14075	Q14525	0.70903
P14075	Q6RW13	0.5653
P14075	P62899	0.44977
P14075	Q99618	1.0342
P14075	P52597	0.39889
P14075	O75534	0.15538
P14075	Q8N3S3	0.095447
P14075	Q8N720	0.70518
P14075	Q8N2M8	0.94059
P14075	Q7L9L4	0.46714
P14075	Q15050	0.55141
P14075	Q9Y2M5	0.55532
P14075	Q8NAX2	0.94025
P14075	Q8N4C6	0.39895
P14075	O00425	0.26392
P14075	Q9BRJ7	0.57597
P14075	Q502W6	0.11532
P14075	P17676	0.58407
P14075	O43324	0.81388
P14075	P37275	0.64771
P14075	P0DKX0	0.24892
P14075	P01215	0.45771
P14075	Q0VFX4	0.50179
P14075	P59991	0.93909
P14075	Q9BZL4	0.31745
P14075	Q8N4S0	0.94025
P14075	P30793	0.033565
P14075	Q5VT03	0.72275
P14075	P41002	0.70331
P14075	Q8NDZ2	0.37212
P14075	Q5T7P3	0.63821
P14075	Q14CN4	0.39857
P14075	Q9P2D3	0.54261
P14075	Q96MF7	0.33532
P14075	A6NCW3	0.52011
P14075	Q5JPH6	0.34954
P14075	Q96KN4	0.032039
P14075	Q8WUM0	0.50316
P14075	Q8WUI4	0.22588
P14075	Q6ZRN7	0.63821
P14075	Q9C0C2	0.21408
P14075	P07476	0.5819
P14075	A8MSY0	0.17062
P14075	Q8TAB7	0.52683
P14075	Q16236	0.37668
P14075	O60936	0.75037
P14075	Q9NWY4	0.48167
P14075	P82933	0.44272
P14075	Q64ET8	0.67892
P14075	Q537H7	0.49703
P14075	Q13360	0.2042
P14075	Q13569	0.3594
P14075	O60869	0.48542
P14075	Q08J23	0.47773
P14075	Q6ZVU0	0.080742
P14075	Q86X53	0.3775
P14075	A6NJV1	0.75174
P14075	P59103	0.46885
P14075	Q9H0B6	0.33771
P14075	P47914	0.83754
P14075	Q6P3X8	0.53685
P14075	Q9NYZ3	0.64912
P14075	O14512	0.33688
P14075	A8MPV3	0.052971
P14075	Q8TDP1	0.57225
P14075	Q9Y6C7	0.63821
P14075	O75489	0.12945
P14075	Q13068	0.27499
P14075	Q96S19	0.16092

P14075	Q8WYH8	0.32765
P14075	Q8NAQ8	0.97086
P14075	Q8IXH7	0.93682
P14075	Q9C0H9	0.22133
P14075	Q6FI81	0.15832
P14075	Q13829	0.51517
P14075	Q9BUH6	0.63401
P14075	O75330	0.16615
P14075	Q9H501	1.1048
P14075	P23610	0.63821
P14075	O95153	0.45253
P14075	Q9UNM6	0.085308
P14075	P21673	0.17147
P14075	Q9Y605	1.2323
P14075	Q7Z2X7	0.90347
P14075	Q9UHU1	0.63821
P14075	Q96JN0	0.70228
P14075	Q6NT89	1.0629
P14075	Q9BXF3	0.38685
P14075	Q6L9T8	0.43864
P14075	Q8WTZ3	0.28331
P14075	A6NK53	0.17629
P14075	Q7Z589	0.79441
P14075	O14893	0.42948
P14075	Q5HYW2	0.93609
P14075	Q86UQ8	0.19547
P14075	Q5T5B0	0.63821
P14075	P07738	0.52942
P14075	Q92466	0.37352
P14075	P62906	0.43312
P14075	Q2M1K9	0.15978
P14075	O43264	0.37077
P14075	Q6STE5	0.63782
P14075	Q9UJ94	0.63821
P14075	Q8IV32	0.84554
P14075	Q8TAP9	1.0726
P14075	Q9H2H8	0.14372
P14075	Q13616	0.39304
P14075	O75496	0.65885
P14075	O94842	0.39331
P14075	Q71SY5	0.80368
P14075	Q15121	0.27798
P14075	Q96K17	0.44417
P14075	Q9Y287	0.25837
P14075	Q9NZJ0	0.46678
P14075	Q1ED39	1.074
P14075	O60716	0.28319
P14075	Q96CD0	0.55958
P14075	Q14331	0.38928
P14075	Q00597	0.49231
P14075	Q6ZNK6	1.0575
P14075	P62314	0.62088
P14075	O95271	0.39028
P14075	P28290	0.48722
P14075	Q8N9L7	0.22473
P14075	Q8N328	0.14386
P14075	Q13112	1.0919
P14075	Q86WZ0	0.46922
P14075	Q8IZL2	0.61793
P14075	Q9H0I3	0.31658
P14075	Q9NVV2	0.63821
P14075	Q9UI72	0.63821
P14075	Q86Y01	0.3628
P14075	Q1ZZU3	0.39132
P14075	O15040	0.1668
P14075	Q5T3J3	0.74704
P14075	Q9BRP4	0.25575
P14075	Q6PII3	0.65761
P14075	Q9BUB7	0.60076
P14075	O75800	0.23613
P14075	Q5TC04	0.28117
P14075	P41567	0.71356
P14075	O95848	0.63845
P14075	O43823	0.25586
P14075	Q13233	0.37975
P14075	Q9BVJ6	0.53608

P14075	Q9UPA5	0.08854
P14075	P04732	0.28594
P14075	Q6UYE1	0.86718
P14075	P38919	0.14204
P14075	Q9UK13	0.29291
P14075	Q15046	0.57543
P14075	Q8IZY5	0.14549
P14075	Q9BYS1	0.78379
P14075	Q8N684	0.32421
P14075	Q9UM13	0.44572
P14075	Q9NX95	0.31578
P14075	Q9UJW0	0.30262
P14075	Q2M238	0.056147
P14075	Q5JYT7	0.037403
P14075	P10914	0.27111
P14075	Q7Z7F7	0.50361
P14075	Q96G25	0.83155
P14075	Q6XQN6	0.045954
P14075	Q9BUL9	0.97916
P14075	Q8WUQ7	0.042906
P14075	Q8NEG2	0.41175
P14075	Q9H4D5	0.35883
P14075	Q8TBB1	0.51142
P14075	Q8TDN6	0.12802
P14075	Q14181	0.36134
P14075	Q5JSZ5	0.86894
P14075	Q6ZTI6	0.74099
P14075	Q14980	0.77533
P14075	Q9Y4Y9	0.51576
P14075	O94921	0.22444
P14075	Q6ZW13	0.63821
P14075	Q96NG5	0.30146
P14075	Q1RMZ1	0.56636
P14075	Q6IEG0	0.41447
P14075	Q96FV9	0.68149
P14075	Q6ZRV2	0.84788
P14075	Q9Y592	0.89371
P14075	Q8NFD4	0.23896
P14075	Q9UBQ5	0.70638
P14075	Q9UIV1	0.91432
P14075	Q8N9K7	0.63821
P14075	Q9UQ88	0.12529
P14075	O75155	0.52483
P14075	Q9BW71	0.93918
P14075	P59942	0.54235
P14075	Q9BU02	0.73128
P14075	P40337	0.56071
P14075	O75909	0.85254
P14075	P14625	0.10895
P14075	Q6BDS2	0.41457
P14075	Q1MSJ5	0.88582
P14075	P17544	0.7483
P14075	Q5VTA0	0.33501
P14075	Q2KHR2	0.14646
P14075	A1A519	0.7862
P14075	Q8N6C7	0.049674
P14075	O43290	0.68951
P14075	Q9BQM9	0.37807
P14075	Q96M83	0.10592
P14075	Q03188	0.36274
P14075	Q8N1N4	0.62841
P14075	P30260	0.79986
P14075	Q8TAF5	0.63821
P14075	Q6PI77	0.59544
P14075	P61289	0.65687
P14075	P05386	0.83108
P14075	Q9UKF6	0.52969
P14075	Q9Y3A2	0.62937
P14075	Q14061	0.45625
P14075	Q9NVE4	0.30764
P14075	Q8N8F4	0.12996
P14075	Q9NY12	0.30668
P14075	A8MTZ0	0.51834
P14075	Q8IUC2	1.0403
P14075	Q7Z3K3	0.27719
P14075	Q3SXR2	0.31842

28197	P14075	Q96ES7	0.10368
28198	P14075	A1A4F0	0.030182
28199	P14075	Q8NG31	0.55517
28200	P14075	Q7L590	0.74964
28201	P14075	Q9UJX3	0.46355
28202	P14075	O60927	0.63447
28203	P14075	Q99081	0.34951
28204	P14075	Q92905	0.48979
28205	P14075	Q96KD3	1.1182
28206	P14075	O43482	0.35164
28207	P14075	Q9UII5	0.19195
28208	P14075	Q9NVM9	0.83518
28209	P14075	O00267	0.86242
28210	P14075	Q8TDN4	0.60369
28211	P14075	Q9Y5V3	0.5984
28212	P14075	Q8WXX7	0.56678
28213	P14075	Q04837	0.29877
28214	P14075	Q9BSD3	0.1893
28215	P14075	Q9H1B7	0.68681
28216	P14075	Q5EBM0	0.59634
28217	P14075	Q4G0N7	0.59127
28218	P14075	Q8WVV9	0.041936
28219	P14075	Q2QGD7	0.51283
28220	P14075	Q5K130	0.33082
28221	P14075	Q8N8E3	0.18663
28222	P14075	Q99622	0.13611
28223	P14075	P07311	0.68515
28224	P14075	Q9Y2W1	0.62119
28225	P14075	Q9NNW5	0.36237
28226	P14075	Q8NA57	0.83513
28227	P14075	Q13838	0.093144
28228	P14075	Q9H160	0.30114
28229	P14075	Q7L8J4	0.38519
28230	P14075	Q86U42	0.53946
28231	P14075	Q9Y296	0.11583
28232	P14075	Q6ZMY9	0.28491
28233	P14075	Q9NTI5	0.62668
28234	P14075	Q9P270	0.26596
28235	P14075	Q68DK7	0.67067
28236	P14075	Q8IXW0	0.30783
28237	P14075	Q70Z53	0.76604
28238	P14075	A6NC62	0.096061
28239	P14075	Q9UL03	0.78716
28240	P14075	P55895	0.41836
28241	P14075	O95707	0.76291
28242	P14075	Q9BTE7	0.46489
28243	P14075	Q9NYF0	0.17057
28244	P14075	P0DMB1	0.65129
28245	P14075	Q6NVV0	0.75874
28246	P14075	P40306	0.23061
28247	P14075	P02538	0.5808
28248	P14075	Q9NQ55	0.38794
28249	P14075	O43399	0.36869
28250	P14075	P86496	0.63821
28251	P14075	P07315	0.32863
28252	P14075	P17535	0.72717
28253	P14075	Q9BX82	0.28313
28254	P14075	Q14188	0.5583
28255	P14075	O14633	0.63821
28256	P14075	Q96EK6	0.23299
28257	P14075	P61353	0.4006
28258	P14075	P0CE67	0.63821
28259	P14075	P51668	0.43503
28260	P14075	Q9NYG5	0.49821
28261	P14075	O60260	0.27859
28262	P14075	Q969H0	0.49575
28263	P14075	P14672	0.54871
28264	P14075	Q8N660	1.0145
28265	P14075	P31948	0.64947
28266	P14075	P49454	0.42034
28267	P14075	P37837	0.54905
28268	P14075	Q5T8R8	0.10036
28269	P14075	O15446	0.20727
28270	P14075	B4DX44	0.3121
28271	P14075	Q6X4T0	0.15919
28272	P14075	Q9Y4M8	0.078022

P14075	Q9H9A7	0.3518
P14075	Q9NZD4	0.45382
P14075	Q8N859	0.28029
P14075	Q8N9Y4	0.7067
P14075	P29372	0.56693
P14075	P39019	0.62034
P14075	Q5W5X9	0.47304
P14075	Q5JSQ8	0.099695
P14075	Q5SZB4	0.34274
P14075	Q9BXM8	0.63821
P14075	Q6NSI3	1.0154
P14075	P28838	0.6616
P14075	Q92830	0.55645
P14075	A6NFK2	0.21819
P14075	Q9GZX5	0.58898
P14075	Q9NXW9	0.24107
P14075	Q9P032	0.29696
P14075	Q9H9L7	0.17135
P14075	O15205	0.33427
P14075	Q9NX58	0.25431
P14075	Q13617	0.411
P14075	Q6P0N0	0.66395
P14075	Q6PJP8	0.36547
P14075	Q13432	0.28207
P14075	O95178	0.66538
P14075	Q86V15	0.30772
P14075	Q9BSJ2	0.73739
P14075	Q15369	1.0539
P14075	D6RGH6	0.97549
P14075	Q99708	0.45985
P14075	Q86V59	0.32013
P14075	Q92552	0.46751
P14075	P60413	0.78356
P14075	P62424	0.74
P14075	O43513	1.0006
P14075	P49901	0.63821
P14075	Q5FWF6	0.28987
P14075	Q99436	0.47769
P14075	Q92540	0.63783
P14075	P49910	0.50266
P14075	Q9NYV4	0.049103
P14075	Q96N77	0.19576
P14075	Q96IK1	0.053558
P14075	P42167	0.24126
P14075	P43360	0.39464
P14075	Q16626	0.48682
P14075	Q14159	0.98848
P14075	P60608	0.41324
P14075	Q8TAP8	0.64135
P14075	Q92547	0.26808
P14075	O75694	0.42839
P14075	P35527	0.80046
P14075	Q9NRG9	0.65908
P14075	Q8IXS2	0.14679
P14075	Q12765	0.65669
P14075	Q12873	0.38325
P14075	Q9NXE8	0.65177
P14075	Q9UHZ2	0.63821
P14075	O75151	0.43849
P14075	A6NI87	0.55767
P14075	Q9NSE4	0.31993
P14075	H3BRN8	0.025654
P14075	Q9BPZ3	0.43825
P14075	Q96G27	0.7527
P14075	Q9UKJ5	0.26065
P14075	P09467	0.12878
P14075	Q13033	0.45038
P14075	Q3LI62	0.63821
P14075	Q01546	0.60884
P14075	Q96ME1	0.0969
P14075	Q16629	0.45766
P14075	A5YKK6	0.7659
P14075	Q9UNH5	0.32734
P14075	O60225	0.10346
P14075	P52434	0.17958
P14075	Q06265	0.15992

P14075	Q9GZU0	0.71296
P14075	Q96NL8	0.70371
P14075	Q96EK4	0.93454
P14075	Q9BSM1	0.77687
P14075	Q96AE4	0.34412
P14075	Q8WWM1	0.82672
P14075	Q5W111	0.020857
P14075	Q9ULC4	0.25603
P14075	O15049	0.56085
P14075	Q9BPX3	0.61283
P14075	O75427	0.30737
P14075	P32243	0.65127
P14075	P46013	0.18924
P14075	Q96MC2	0.25236
P14075	Q641Q2	0.6299
P14075	Q8N0Y2	0.1488
P14075	Q5JRK9	1.1259
P14075	Q32Q52	0.23864
P14075	Q9H6D7	0.32838
P14075	Q8WXI9	0.30718
P14075	O15355	0.58165
P14075	Q2M3A8	0.29301
P14075	Q63HK3	0.29246
P14075	Q9NRR8	0.49387
P14075	Q9C0D2	0.69661
P14075	Q99627	0.18172
P14075	Q9NUU6	0.64509
P14075	Q96Q89	0.27282
P14075	Q86XK3	0.79656
P14075	O95486	0.1654
P14075	Q9H4A6	0.35893
P14075	Q8WYN3	0.41114
P14075	Q8IUR6	0.7543
P14075	Q96CM3	0.3399
P14075	A6ZKI3	0.47267
P14075	B3KS81	0.15488
P14075	P27816	0.21386
P14075	Q7Z3B4	0.59189
P14075	Q9P2B7	0.94025
P14075	P30307	0.44229
P14075	Q96FX2	0.62066
P14075	Q14814	0.40792
P14075	Q03111	0.9661
P14075	Q9BY44	0.24886
P14075	Q9ULV3	0.085347
P14075	A6NCL2	0.02177
P14075	Q6WBX8	0.24728
P14075	Q9BWT1	0.81687
P14075	P84090	0.49989
P14075	Q8N8G6	0.21733
P14075	Q6ZUC2	0.37143
P14075	Q86YF9	0.073537
P14075	Q96BN8	0.35901
P14075	Q8N9B5	0.48259
P14075	Q9UBW8	0.14526
P14075	O00165	0.45931
P14075	Q6P1N0	0.23025
P14075	Q15031	0.42445
P14075	Q9UMX1	0.5348
P14075	Q96RL1	0.50747
P14075	P51861	0.33993
P14075	P50440	0.27281
P14075	Q6UWI2	0.48976
P14075	Q7Z7A1	0.7888
P14075	Q9P0J0	0.24454
P14075	O75818	0.061121
P14075	Q9NRG4	0.51566
P14075	P43357	0.1668
P14075	Q86X51	0.24041
P14075	Q9Y2X3	0.44885
P14075	P24534	0.37167
P14075	Q71F23	0.31743
P14075	B1AK76	0.81406
P14075	Q9Y5J6	0.23313
P14075	Q9H3K6	0.45887
P14075	Q86XZ4	0.36207

P14075	Q96LT7	0.65263
P14075	Q99471	0.65124
P14075	Q6P1K8	0.15655
P14075	Q8NFH4	0.74228
P14075	Q8WXB4	0.12672
P14075	Q9NS37	0.64587
P14075	Q9ULR5	0.2846
P14075	Q9XRX5	0.33705
P14075	P54826	0.1922
P14075	Q5TA50	0.28232
P14075	Q9UKK6	0.46151
P14075	Q9UQF2	0.27664
P14075	O14972	0.21616
P14075	Q9P013	0.62503
P14075	Q12796	1.2352
P14075	A8MWP6	0.63821
P14075	P62829	0.8288
P14075	Q8WTV1	0.72991
P14075	Q9ULD9	0.78999
P14075	Q9Y6X2	0.54936
P14075	Q504U0	0.24573
P14075	O75368	0.33408
P14075	P62280	0.54778
P14075	Q16851	0.2473
P14075	Q10567	0.4048
P14075	Q96C57	0.71417
P14075	Q92567	0.65422
P14075	Q9P1Y5	0.48832
P14075	Q9Y448	0.54541
P14075	O75461	0.13662
P14075	Q6ZQY7	0.53427
P14075	P02533	0.80046
P14075	Q5VTL8	0.94592
P14075	Q9NVV9	0.79362
P14075	O60216	0.55893
P14075	Q96NT1	0.82122
P14075	Q86WQ0	0.3119
P14075	Q8NFH8	0.20595
P14075	Q9P278	0.69108
P14075	Q8TAG6	0.60459
P14075	Q6P3W6	0.48727
P14075	P63167	0.94058
P14075	Q96QE5	0.66358
P14075	I3L0S3	0.63121
P14075	Q96CN9	0.29119
P14075	Q6A162	0.62407
P14075	Q8TCY9	0.50334
P14075	Q8IWL3	0.38447
P14075	P63272	0.78776
P14075	Q92733	0.63821
P14075	Q12968	0.45125
P14075	P02808	0.52302
P14075	Q8NE65	0.26405
P14075	P49591	0.41447
P14075	P42025	0.1461
P14075	A8MXT2	0.0024234
P14075	Q9NS73	0.48729
P14075	C9JFL3	0.63821
P14075	A6NGR9	0.75184
P14075	Q8WY22	0.80341
P14075	Q15170	1.3033
P14075	Q6PGQ7	1.0444
P14075	Q9Y3B2	0.54335
P14075	Q15004	0.56663
P14075	O15084	0.50313
P14075	Q9HB71	0.34483
P14075	Q9NRY2	0.65099
P14075	Q9UBW7	0.65842
P14075	P19532	0.46963
P14075	Q8N9Z9	0.42932
P14075	Q70YC5	0.3723
P14075	Q9BXW9	0.54308
P14075	Q53S33	0.87205
P14075	Q9Y4H4	0.49203
P14075	Q8IY92	0.19978
P14075	Q96C74	0.1368

P14075	Q0VDD7	1.0468
P14075	Q6NT76	0.24624
P14075	Q9P0J6	0.55862
P14075	Q9H0F6	0.072988
P14075	Q96MG7	0.51318
P14075	Q14872	0.63102
P14075	O60829	0.78504
P14075	P83436	0.25922
P14075	C9JLW8	0.56271
P14075	Q6KB66	0.28852
P14075	Q9NV88	0.3608
P14075	Q68EA5	0.30057
P14075	Q8N9R0	0.63821
P14075	P55060	0.65876
P14075	Q96MW1	0.94025
P14075	Q9BPW8	0.51558
P14075	Q8NBT2	0.47096
P14075	Q96F86	0.42698
P14075	Q9NZL4	0.38826
P14075	Q9BZD4	0.5349
P14075	Q7Z5D8	0.34234
P14075	P18583	0.23587
P14075	O75570	0.51646
P14075	Q15776	0.24641
P14075	Q10570	0.59078
P14075	Q14192	0.5256
P14075	Q9NZH4	0.53275
P14075	A0AVK6	0.2637
P14075	Q13541	0.55486
P14075	A6NI79	0.75061
P14075	Q9NPI7	0.15513
P14075	Q8TAE8	0.70789
P14075	Q9HD15	0.36293
P14075	Q8HWS3	0.16579
P14075	Q17RN3	0.51178
P14075	P35237	0.1327
P14075	O75437	0.26696
P14075	Q9BYK8	0.27681
P14075	Q9NS69	0.081256
P14075	Q9ULW6	0.25402
P14075	Q8ND04	0.48924
P14075	Q96A56	0.77198
P14075	O14802	0.072694
P14075	Q9Y305	0.52998
P14075	Q9UBF6	0.48463
P14075	Q495D7	0.085154
P14075	Q9NQ92	0.66876
P14075	Q9UI56	0.63821
P14075	A6NJ78	0.86595
P14075	Q9Y462	0.39201
P14075	Q07020	0.55712
P14075	Q8IWF6	0.50906
P14075	Q8N5T8	0.59539
P14075	Q9Y2F5	0.075517
P14075	O75477	0.39786
P14075	Q3SY84	0.54544
P14075	Q8N1X5	0.63821
P14075	Q9H0W9	0.39601
P14075	Q7Z6K5	0.14756
P14075	Q02930	0.56517
P14075	Q96FF7	0.63821
P14075	P10073	0.22972
P14075	O95411	0.63821
P14075	C9JE40	0.58209
P14075	O00148	0.1599
P14075	Q8WWY3	0.2278
P14075	O94850	0.48497
P14075	Q8NCE8	0.55079
P14075	Q9Y4E1	0.52568
P14075	Q8NHU0	0.15736
P14075	Q5JVG8	0.27372
P14075	A7MCY6	0.49256
P14075	Q96NU7	0.2725
P14075	Q8NFH5	0.38809
P14075	P62508	0.33984
P14075	Q5VT28	0.57471

P14075	Q8NDC0	0.79783
P14075	O43829	0.39345
P14075	Q9Y281	0.21294
P14075	Q8WXU2	0.50325
P14075	Q9BWU0	0.2928
P14075	Q5T6F2	0.79864
P14075	O14757	0.43158
P14075	Q8N8Z3	0.063746
P14075	Q5XUX0	0.67487
P14075	Q9H171	0.13547
P14075	Q9BTC8	0.39335
P14075	Q96E16	0.59931
P14075	Q96M63	0.10081
P14075	Q8N3X1	0.16679
P14075	Q9NUS5	0.2762
P14075	P62263	0.32522
P14075	Q8NEP9	0.2718
P14075	Q0P140	0.54739
P14075	O14556	0.29723
P14075	Q5U623	0.50965
P14075	P0C7I6	0.59659
P14075	Q5T280	0.97394
P14075	Q12834	0.60316
P14075	Q96CW5	0.58999
P14075	Q9NUW8	0.43989
P14075	O14513	0.3611
P14075	P22392	0.20977
P14075	Q14978	0.704
P14075	Q9Y3V2	0.55947
P14075	Q96EY1	0.27751
P14075	A4D174	0.090536
P14075	Q969L4	0.4873
P14075	O96006	0.64678
P14075	Q0P641	0.024697
P14075	Q8N661	0.75144
P14075	O15318	0.79117
P14075	Q9P2K3	0.88369
P14075	Q9Y2B5	0.32713
P14075	Q08AG5	0.43767
P14075	Q9UBL0	0.22318
P14075	Q8WU43	0.0902
P14075	Q00610	0.61561
P14075	P50222	0.44098
P14075	Q96QU4	0.76082
P14075	Q96DP5	0.47958
P14075	P19013	0.60884
P14075	Q9P0W2	0.50666
P14075	Q6MZN7	0.63821
P14075	P11171	0.25534
P14075	P62854	0.61345
P14075	Q8NFV5	0.53769
P14075	Q96CB8	0.28213
P14075	Q6ZTY9	0.34215
P14075	Q8IYE0	0.63147
P14075	A0A183	0.63821
P14075	Q6VY07	0.3376
P14075	Q8N6F7	0.32417
P14075	P60866	0.48566
P14075	Q8IWY8	0.12197
P14075	Q0PNE2	0.5206
P14075	Q8IZ40	0.38482
P14075	C9JN71	0.18615
P14075	Q569H4	0.94025
P14075	Q2M2Z5	0.64889
P14075	Q8N0Y7	0.50609
P14075	Q14145	0.96268
P14075	Q9H7L9	0.46336
P14075	Q9UJY5	0.32552
P14075	Q8N7M0	0.20837
P14075	Q8NF64	0.97699
P14075	Q8NEH6	0.79921
P14075	P09565	0.48035
P14075	Q8ND56	0.65962
P14075	O95644	0.57261
P14075	Q8N9H9	0.63821
P14075	A8MV24	0.30403

P14075	P82675	0.47204
P14075	Q9BQY4	0.64001
P14075	Q15651	0.71508
P14075	Q2TAM9	1.0753
P14075	Q96FV2	0.58341
P14075	O95201	0.39205
P14075	Q76KX8	0.3048
P14075	Q9Y2Y9	0.54713
P14075	Q9UJT2	0.57314
P14075	Q06609	0.26727
P14075	Q8WTQ4	0.23412
P14075	Q13501	0.26256
P14075	Q5SVJ3	0.27226
P14075	Q701N4	0.63821
P14075	Q8N8G2	0.57276
P14075	Q15181	0.067245
P14075	A6NIY4	0.53769
P14075	Q8N944	0.82301
P14075	Q8N726	0.44178
P14075	Q96T17	0.40055
P14075	P26373	0.71018
P14075	P51826	0.23271
P14075	P07814	0.30327
P14075	Q9H7T3	0.47839
P14075	P50749	0.5281
P14075	Q13367	0.26011
P14075	Q8TB03	0.64017
P14075	Q15021	0.60878
P14075	P81408	0.7527
P14075	P09874	0.48966
P14075	Q5JTZ5	0.27794
P14075	Q9BUV0	0.94025
P14075	Q9Y2X0	0.36423
P14075	Q8N823	0.2118
P14075	Q9BTX9	0.65143
P14075	A6NLI3	0.062068
P14075	P62877	0.33197
P14075	Q15527	0.51836
P14075	O75956	0.34753
P14075	Q7Z3C6	0.25742
P14075	Q8IYT1	0.39362
P14075	Q9HCI5	0.42527
P14075	O43615	0.11289
P14075	Q8NHU2	0.12281
P14075	Q8N972	0.2118
P14075	Q8IYF3	0.30354
P14075	Q7Z7C7	0.3347
P14075	P0DI82	0.17671
P14075	Q8TBN0	0.1389
P14075	Q96NB3	0.26605
P14075	O00198	0.60346
P14075	Q16775	0.30626
P14075	Q14498	0.61618
P14075	O60664	0.2766
P14075	Q96HM7	0.66139
P14075	P35227	0.52285
P14075	O60784	0.15708
P14075	Q96T76	0.662
P14075	O95567	0.63821
P14075	Q5T8I3	0.6496
P14075	O00193	1.074
P14075	Q13045	0.76394
P14075	Q9NRH1	0.33685
P14075	B1ANY3	0.5577
P14075	Q3ZLR7	0.54549
P14075	P61578	0.45541
P14075	P05387	0.38311
P14075	O95429	0.17251
P14075	Q6DKK2	0.6845
P14075	O75346	0.25665
P14075	Q96MY7	0.99153
P14075	Q9GZN8	0.45783
P14075	P33552	0.35453
P14075	Q6VUC0	0.21924
P14075	Q9H1K1	0.56759
P14075	O75164	0.74494

P14075	Q9H875	0.36066
P14075	O43676	0.55791
P14075	Q99575	0.48668
P14075	Q9UM00	0.84599
P14075	Q7Z739	0.53716
P14075	Q15596	0.48625
P14075	Q03252	0.25322
P14075	Q9BWG6	0.38085
P14075	Q86UW9	0.61649
P14075	Q9BTL4	0.66602
P14075	P20749	0.25855
P14075	Q8TC76	1.2058
P14075	Q53GL7	0.3282
P14075	A6NP11	0.31228
P14075	Q9H8Y1	0.78019
P14075	O94913	0.13499
P14075	Q4KMQ1	0.68828
P14075	Q9H3L0	0.93187
P14075	P56945	0.37783
P14075	Q4VC44	0.58427
P14075	Q9Y6G3	0.14172
P14075	Q96P71	0.24351
P14075	Q9NRN7	0.28341
P14075	Q14152	0.82125
P14075	P18887	0.3963
P14075	Q8N7K0	0.2118
P14075	Q96GY3	0.60102
P14075	Q9NYF3	0.60037
P14075	O43765	0.42702
P14075	Q32MH5	0.56973
P14075	O95905	0.51549
P14075	Q2M2I5	0.80046
P14075	Q96ED9	0.28138
P14075	A6NKH3	0.32368
P14075	Q6MZQ0	0.35701
P14075	Q8N3L3	0.53095
P14075	O75690	0.63821
P14075	Q8NA72	0.058739
P14075	Q7Z6M4	0.57896
P14075	Q68EN5	0.3247
P14075	Q5T5S1	0.37205
P14075	Q9UJW9	1.2384
P14075	Q8N0Z3	0.36924
P14075	Q96FJ8	0.63821
P14075	Q5T0F9	0.18125
P14075	A8MU76	0.54875
P14075	Q2KHR3	0.043139
P14075	O14990	0.64925
P14075	Q8WTW3	0.58033
P14075	P23297	0.31285
P14075	Q9BUE0	0.84633
P14075	Q9UPP5	0.75174
P14075	Q6P280	0.33703
P14075	A6NKN8	0.96685
P14075	P62310	0.47978
P14075	Q6P656	0.15022
P14075	Q8N7B7	0.14264
P14075	Q96EP1	0.41472
P14075	O95190	0.62712
P14075	Q96NT3	0.35734
P14075	Q9BVP2	0.14349
P14075	B1APH4	0.23684
P14075	Q68DL7	0.63821
P14075	Q9BY77	0.21626
P14075	Q86YP4	0.63879
P14075	Q6PD74	0.41451
P14075	O60535	0.24348
P14075	Q9NSQ0	0.26322
P14075	Q66K80	0.63821
P14075	Q9NVS2	0.71424
P14075	Q9GZR1	0.23528
P14075	Q8IVM7	0.63821
P14075	Q8N8X9	0.20592
P14075	O95989	0.28692
P14075	A6NHS1	0.65093
P14075	Q8TF32	0.31228

P14075	P18846	0.63447
P14075	Q9Y5K5	0.1725
P14075	Q63HN1	0.48314
P14075	Q9H7J1	0.46414
P14075	Q9P2R7	0.3009
P14075	Q14244	0.58704
P14075	Q9UN37	0.32278
P14075	Q9ULH7	0.68993
P14075	Q6S9Z5	0.71351
P14075	Q07955	0.44556
P14075	Q14135	0.19547
P14075	P52948	0.70774
P14075	O75157	0.48009
P14075	Q75VX8	1.0319
P14075	P51948	0.36569
P14075	Q8WTS6	0.30884
P14075	E9PAV3	0.31079
P14075	Q9UMX0	0.51106
P14075	O75530	0.43437
P14075	Q9BZE9	0.55735
P14075	Q8N812	0.43721
P14075	Q9H503	0.22264
P14075	Q9NXK8	0.37695
P14075	Q6U949	0.63821
P14075	P62258	0.57796
P14075	Q9BYQ6	0.78356
P14075	Q86TG7	0.29009
P14075	Q96L92	0.48375
P14075	Q9UGB4	0.63821
P14075	Q9BY27	0.32697
P14075	Q9Y6R6	0.2118
P14075	Q04864	0.85778
P14075	Q5T953	0.98003
P14075	A1A4G5	0.63821
P14075	Q15072	0.13307
P14075	P48539	0.46205
P14075	Q9BSK0	0.85698
P14075	Q9NY87	0.82289
P14075	Q9Y2G2	0.26668
P14075	O75177	0.64704
P14075	P55808	0.93918
P14075	Q8N6U2	0.31618
P14075	Q15276	0.23447
P14075	O75334	0.32542
P14075	A4GXA9	0.64924
P14075	Q8N3U4	0.48005
P14075	P52298	0.43776
P14075	Q8WWR9	0.79845
P14075	O00321	0.16138
P14075	Q8TAK5	0.24671
P14075	Q96C34	0.50317
P14075	Q6UXP7	0.15942
P14075	P18850	0.38769
P14075	Q9HA90	0.94025
P14075	Q6ZV60	0.63821
P14075	Q9Y2K5	0.31665
P14075	Q96LK8	0.16665
P14075	Q5T200	0.65413
P14075	Q3SY05	0.13569
P14075	A2A288	0.41634
P14075	Q8TBZ9	0.88187
P14075	Q3MJ40	0.13808
P14075	Q5T4H9	0.63821
P14075	Q96EK5	0.13098
P14075	Q7L4P6	0.56946
P14075	O75152	0.20455
P14075	Q8IX03	0.73049
P14075	Q9UJC5	0.4588
P14075	Q5H9I0	0.22463
P14075	Q9H6Z9	0.29335
P14075	O43299	0.8756
P14075	Q8IW35	0.82645
P14075	Q5BJF2	0.4504
P14075	Q8IWV8	0.14158
P14075	Q96CW6	0.85637
P14075	O75531	0.50359

P14075	Q9UKK3	0.42491
P14075	Q9NYB0	0.64456
P14075	Q8WX92	1.1625
P14075	Q5VYK3	0.12263
P14075	Q8IZX4	0.23099
P14075	A8MV72	0.53375
P14075	Q6GMV2	0.25209
P14075	Q9BQ90	0.4948
P14075	Q9UL41	0.49713
P14075	Q17RB0	0.49014
P14075	Q9BU76	0.90542
P14075	Q9Y4B6	0.5966
P14075	Q3C1V9	0.26466
P14075	P58340	0.27539
P14075	Q9Y697	0.40956
P14075	O95551	0.48589
P14075	P05787	1.0818
P14075	Q9H5Y0	0.56988
P14075	P52435	0.40938
P14075	Q3KP31	0.2763
P14075	O14771	0.46087
P14075	P10243	0.25954
P14075	Q96F83	0.30753
P14075	Q9H8M7	0.53501
P14075	A4D1U4	0.3796
P14075	Q5I0X4	0.94025
P14075	Q8N888	0.65442
P14075	O75031	0.39925
P14075	A6NGK3	0.50458
P14075	Q9Y3D0	0.70685
P14075	Q8IUB9	0.63821
P14075	Q9BUG6	0.14433
P14075	Q8N4J0	0.8714
P14075	Q9H668	0.021488
P14075	P53803	0.53073
P14075	Q15543	0.79809
P14075	Q8WYQ5	0.46294
P14075	Q7Z3E2	0.84195
P14075	Q9UIC8	0.18887
P14075	Q9HBE1	0.08421
P14075	P52741	0.10726
P14075	P25063	0.63821
P14075	Q8N9W5	0.27819
P14075	Q9UPT5	0.33615
P14075	Q6XXX2	0.047706
P14075	Q538Z0	0.63821
P14075	Q6NT46	0.45588
P14075	Q13023	0.37428
P14075	Q2T9F4	0.15501
P14075	Q14693	0.18961
P14075	Q8WVP5	0.89587
P14075	Q9Y675	0.76082
P14075	P15924	0.22698
P14075	Q3LI83	0.84358
P14075	Q9NWK9	0.53928
P14075	Q7L8S5	0.25321
P14075	Q14140	1.2688
P14075	Q9UC07	0.18107
P14075	Q6ZN84	0.3483
P14075	I3L3R5	0.63821
P14075	Q9UNK9	0.14325
P14075	P25789	0.48239
P14075	Q7Z4Q2	0.68445
P14075	Q86TP1	0.52789
P14075	Q8TBR5	0.18133
P14075	Q96M15	0.043605
P14075	Q13761	0.6078
P14075	Q3LHN0	1.0514
P14075	P24864	0.45881
P14075	Q9H869	1.0792
P14075	O75167	0.10962
P14075	P10599	0.269
P14075	Q99689	0.14367
P14075	Q969H6	0.62526
P14075	Q8N9T8	1.1422
P14075	Q71UM5	0.36106

P14075	Q96PP4	0.37914
P14075	Q9H6T0	0.14984
P14075	Q49B96	0.37179
P14075	Q9HBM6	0.46726
P14075	Q08AD1	0.34415
P14075	Q96QK1	0.38775
P14075	P05114	0.32618
P14075	Q8N8K9	0.15669
P14075	Q9BZI7	0.56865
P14075	P36543	0.084738
P14075	O14579	0.29675
P14075	Q9H1J1	0.7589
P14075	Q5TAP6	0.65111
P14075	Q16534	0.23441
P14075	P45984	0.25614
P14075	Q96M61	0.1668
P14075	Q9HCM1	0.89407
P14075	Q14CZ0	1.1739
P14075	A4D0V7	0.74595
P14075	Q03938	0.29644
P14075	Q8TD19	0.25459
P14075	Q3B820	0.84358
P14075	Q9UIY3	0.29794
P14075	Q9Y314	0.65943
P14075	Q13895	0.4515
P14075	P69905	0.21852
P14075	Q9NUD7	0.02377
P14075	Q9NPG3	0.58938
P14075	Q9NYJ8	0.27222
P14075	Q9BQ61	0.94025
P14075	Q8N5Q1	1.1182
P14075	Q5T754	0.63821
P14075	P61927	0.411
P14075	Q7RTV0	0.28589
P14075	Q13888	0.42488
P14075	Q9Y3A5	0.48976
P14075	Q9Y5U8	0.45274
P14075	P08047	0.57709
P14075	Q8NCU7	1.0195
P14075	Q8N3F0	0.6285
P0C214	P41970	0.83566
P0C214	P0C2W7	0.086276
P0C214	Q8N5I9	0.24937
P0C214	Q9Y536	0.66154
P0C214	Q9NWB7	0.46867
P0C214	Q9HD33	0.68023
P0C214	P62861	1.3088
P0C214	P80217	0.76809
P0C214	Q9BQ67	0.35391
P0C214	Q9Y5A9	0.46237
P0C214	P43351	0.53357
P0C214	P22681	0.44491
P0C214	Q9BSH5	0.43767
P0C214	A6NP61	0.43883
P0C214	Q9Y316	0.53655
P0C214	O00198	0.34319
P0C214	Q9Y3Q8	1.2112
P0C214	Q6ZRN7	0.086276
P0C214	Q9Y2B5	0.11609
P0C214	Q9P1F3	0.52933
P0C214	P40424	0.57258
P0C214	Q8IW19	0.47263
P0C214	Q8WWR8	0.17154
P0C214	Q9BY77	0.41689
P0C214	Q7Z3E2	0.15134
P0C214	P27469	0.77114
P0C214	Q96BN6	0.060302
P0C214	Q86UD4	0.22841
P0C214	P50440	0.0096668
P0C214	P06132	0.59558
P0C214	Q05086	0.61844
P0C214	Q16795	0.17322
P0C214	Q14457	0.42616
P0C214	P61236	0.16769
P0C214	P41250	0.47671
P0C214	Q9Y6R9	0.30221

P0C214	Q9H7E9	0.24937
P0C214	O14933	0.81891
P0C214	A6NDX5	0.37134
P0C214	Q9H147	0.083812
P0C214	Q9UGP4	0.34962
P0C214	Q6ZN55	0.29588
P0C214	Q6X4W1	0.89219
P0C214	Q04446	0.22793
P0C214	Q9H7C4	0.39304
P0C214	Q9BPZ7	0.56272
P0C214	Q92993	1.0045
P0C214	P60520	0.24638
P0C214	Q96BW9	0.34568
P0C214	Q5BKZ1	0.57723
P0C214	Q02833	0.44676
P0C214	Q6NX49	0.22763
P0C214	Q6PDB4	0.49464
P0C214	Q12996	0.19159
P0C214	Q8TAG6	0.047621
P0C214	O00470	0.45318
P0C214	Q9BZI7	0.80125
P0C214	O75683	0.3769
P0C214	Q8N616	0.086276
P0C214	Q8NCT3	0.39893
P0C214	Q13310	0.45338
P0C214	P84098	0.86977
P0C214	O95551	0.73003
P0C214	Q96B02	0.43148
P0C214	Q9NQG7	0.29213
P0C214	A8MPV3	0.072472
P0C214	Q9BSU1	0.4882
P0C214	O00170	0.79955
P0C214	O00268	1.0617
P0C214	P29474	0.52724
P0C214	Q9UKW6	0.35156
P0C214	O94829	0.90472
P0C214	Q9NZL4	0.33549
P0C214	Q96LL4	0.2675
P0C214	Q9UJX6	0.60201
P0C214	Q14997	0.24254
P0C214	Q96CW1	0.56335
P0C214	Q8TEX9	0.31926
P0C214	Q92618	0.17436
P0C214	Q6NZY4	0.58023
P0C214	O43768	0.05135
P0C214	B2RV13	0.53982
P0C214	Q15833	0.31101
P0C214	Q96S94	0.31702
P0C214	P55822	0.59487
P0C214	Q9Y2M2	0.32232
P0C214	Q6ZTC4	0.086276
P0C214	Q9UI26	0.25812
P0C214	Q14410	0.58072
P0C214	Q6NUQ1	0.41213
P0C214	Q9BVJ6	0.76722
P0C214	Q9NRR3	0.40332
P0C214	P53567	0.59995
P0C214	Q14CN4	0.34216
P0C214	Q5SZL2	0.29706
P0C214	Q9UBW5	0.45292
P0C214	Q8N1V2	0.3743
P0C214	Q06732	0.19269
P0C214	Q8NCU1	0.034403
P0C214	Q9UM00	1.1233
P0C214	Q3LI64	0.21795
P0C214	Q96A35	0.60091
P0C214	Q9BZL4	0.45292
P0C214	Q8NEK5	0.30414
P0C214	O75444	0.29835
P0C214	O95243	0.67454
P0C214	Q8TB72	0.49531
P0C214	Q9H081	0.70879
P0C214	O15078	0.55969
P0C214	Q6ICB4	0.12739
P0C214	Q9NQZ8	0.28926
P0C214	Q6RFH5	0.28606

P0C214	Q9HA82	0.28631
P0C214	Q07866	0.60572
P0C214	Q14526	0.45005
P0C214	Q8N163	0.66659
P0C214	P35080	0.33738
P0C214	Q9Y3E1	0.38879
P0C214	Q03052	0.24412
P0C214	P82979	0.35477
P0C214	Q9UKA1	0.2671
P0C214	P61247	1.3687
P0C214	P51665	0.4302
P0C214	Q9BPX3	0.80148
P0C214	Q8IY18	0.3217
P0C214	P60228	1.051
P0C214	Q9BTM9	0.89975
P0C214	P49406	0.51314
P0C214	B3EWG6	0.24937
P0C214	Q9NRP2	0.61231
P0C214	Q8TAI1	0.022733
P0C214	P18850	0.55201
P0C214	P53990	0.45014
P0C214	Q96HJ3	0.24937
P0C214	Q9BWW4	0.59458
P0C214	Q96P26	0.14583
P0C214	O15198	0.50374
P0C214	Q15742	0.3131
P0C214	O43684	0.71048
P0C214	Q5VU69	0.22498
P0C214	Q9BW11	0.29396
P0C214	Q8NFW9	0.28853
P0C214	Q9Y6P5	0.43066
P0C214	Q16637	1.0072
P0C214	Q5MIZ7	0.37536
P0C214	Q9Y5L0	0.87747
P0C214	Q96J02	0.4187
P0C214	Q8NI51	0.5141
P0C214	Q5H9R7	1.1614
P0C214	Q9UI08	0.21797
P0C214	Q9UHZ2	0.086276
P0C214	P02794	0.98278
P0C214	P54198	0.94998
P0C214	P61923	0.32617
P0C214	P08697	0.85505
P0C214	Q5JQF7	0.086276
P0C214	Q96GM8	0.35092
P0C214	Q03989	0.28774
P0C214	Q9HAJ7	0.98806
P0C214	Q9Y6X2	0.71423
P0C214	Q6IQ32	0.36931
P0C214	H3BU77	0.3362
P0C214	O14490	0.27756
P0C214	D6RGH6	1.1673
P0C214	Q8IY42	0.79876
P0C214	Q9Y2W2	0.67038
P0C214	A6NIE6	0.56712
P0C214	Q7Z3K3	0.40675
P0C214	A6NCW3	0.53828
P0C214	P51114	0.014757
P0C214	Q96C28	0.39613
P0C214	Q9UHB7	1.3515
P0C214	Q9HBH1	0.62943
P0C214	Q96B67	0.25112
P0C214	Q9Y399	0.9174
P0C214	Q96BK5	0.52218
P0C214	O15287	0.90254
P0C214	Q96FV9	0.76691
P0C214	O76011	0.58756
P0C214	Q9Y6X4	0.84223
P0C214	Q9GZZ9	0.19711
P0C214	P30084	0.405
P0C214	P62312	0.95657
P0C214	O00213	0.33321
P0C214	Q8N5N7	0.47684
P0C214	P08729	1.3356
P0C214	Q9BUH8	0.33709
P0C214	Q8NHW5	0.27701

P0C214	Q92585	0.91574
P0C214	Q96T66	0.29901
P0C214	Q8IWJ2	0.54945
P0C214	Q05BW5	0.10665
P0C214	P35754	0.57477
P0C214	Q14527	0.39627
P0C214	Q8N371	0.32832
P0C214	O75608	0.43666
P0C214	P55735	0.56882
P0C214	Q96MU6	0.25117
P0C214	Q8IYB1	0.12874
P0C214	Q9P255	0.37249
P0C214	Q9NQR7	0.086276
P0C214	Q6B0B8	0.34167
P0C214	O75865	0.64403
P0C214	Q9Y3T9	0.92832
P0C214	Q9H9Q4	0.36028
P0C214	Q15007	0.37377
P0C214	Q9BUK0	0.78076
P0C214	Q3LI81	0.6322
P0C214	Q8ND07	0.85883
P0C214	Q8ND76	0.4883
P0C214	Q32M92	0.055577
P0C214	O94927	0.37012
P0C214	P20248	0.94664
P0C214	Q96NL8	0.27067
P0C214	Q9H825	0.17526
P0C214	A8MQ11	0.2972
P0C214	O14813	0.20519
P0C214	Q9UHL9	0.26807
P0C214	Q17RB0	0.17928
P0C214	O95671	0.5976
P0C214	Q3MIT2	0.30179
P0C214	P82930	0.57625
P0C214	P51946	0.52977
P0C214	P24863	1.0391
P0C214	Q8IVV7	0.15553
P0C214	Q8WTX7	0.19098
P0C214	P48728	0.089107
P0C214	P49354	0.33544
P0C214	Q8WX92	1.4696
P0C214	Q9H832	0.85583
P0C214	Q8IX04	0.28316
P0C214	Q8TAP9	1.2581
P0C214	Q9UKN5	0.31779
P0C214	Q96FX7	0.37753
P0C214	P49419	0.93156
P0C214	Q8N9A8	0.27409
P0C214	Q9UDY4	0.95057
P0C214	Q14532	0.38807
P0C214	O15083	0.43551
P0C214	Q96CJ1	1.1288
P0C214	O15234	0.97002
P0C214	Q58F21	0.24157
P0C214	Q9P278	0.76403
P0C214	Q13619	0.36567
P0C214	O75306	0.42396
P0C214	Q96RE9	0.26948
P0C214	P35226	0.52263
P0C214	Q58FG0	0.8863
P0C214	Q13907	0.37342
P0C214	Q9H5Z6	0.2675
P0C214	Q9UBB6	0.37249
P0C214	Q13617	0.7745
P0C214	Q8NA19	0.24078
P0C214	A9QM74	0.5537
P0C214	Q13761	0.67793
P0C214	Q96KH6	0.20081
P0C214	Q9NZT2	0.023023
P0C214	Q9NZN8	0.91198
P0C214	O15116	0.9542
P0C214	Q8IX03	1.1126
P0C214	Q8N1G1	0.23781
P0C214	Q96M61	0.2675
P0C214	Q13405	0.68937
P0C214	Q969S3	0.70039

P0C214	Q9UGC7	0.27126
P0C214	Q14739	0.28956
P0C214	Q5TEZ4	0.086276
P0C214	Q969W3	0.58117
P0C214	Q17RN3	0.12146
P0C214	P26368	0.5254
P0C214	Q9Y3D5	0.54478
P0C214	A6NCL1	0.13382
P0C214	Q96NS1	0.16769
P0C214	P0DI83	0.35283
P0C214	P09417	0.1646
P0C214	Q8IWV8	0.33188
P0C214	Q96S65	0.89872
P0C214	P60709	0.10604
P0C214	Q13257	0.72411
P0C214	Q15327	0.46509
P0C214	Q56P42	0.12649
P0C214	Q96QA6	0.16769
P0C214	Q86V59	0.28996
P0C214	Q8WTP9	0.80497
P0C214	Q86XI2	0.876
P0C214	Q8NAX2	0.24937
P0C214	Q5JTD0	0.93134
P0C214	Q7Z2F6	0.41504
P0C214	A8MUV8	0.48694
P0C214	A6H8Y1	0.61356
P0C214	Q05195	0.51295
P0C214	Q9NWK9	0.50182
P0C214	O75818	0.2805
P0C214	Q9NWW7	0.45005
P0C214	P41220	0.50727
P0C214	Q9H6T0	0.16429
P0C214	Q15059	1.1239
P0C214	P0CG43	0.086276
P0C214	O75935	0.82408
P0C214	Q9Y3I1	0.90072
P0C214	P08294	0.23094
P0C214	O14910	0.46813
P0C214	O43296	0.30224
P0C214	P49006	0.77848
P0C214	Q9Y615	0.22353
P0C214	Q6ZQT0	0.035153
P0C214	Q969F9	0.72059
P0C214	Q6ZN17	1.166
P0C214	Q9UHA2	0.21221
P0C214	P59036	0.086276
P0C214	P07315	0.5283
P0C214	Q16543	0.51688
P0C214	Q9HBK9	0.26451
P0C214	Q8WV28	0.23792
P0C214	Q14264	0.35694
P0C214	Q96GA3	0.83325
P0C214	P35237	0.78945
P0C214	Q9Y617	0.18141
P0C214	Q5XUX1	0.2675
P0C214	P62316	0.86673
P0C214	Q2M2I5	0.58756
P0C214	Q9NS37	0.75851
P0C214	Q9ULX3	0.73115
P0C214	Q96PQ5	0.31698
P0C214	Q14746	0.23069
P0C214	O60346	0.23914
P0C214	Q16254	0.91568
P0C214	O75161	0.79218
P0C214	Q9BU02	0.63896
P0C214	Q9BYD2	0.65728
P0C214	P49589	0.59731
P0C214	Q9BSY4	0.49121
P0C214	P52209	0.25295
P0C214	A6ZKI3	0.037258
P0C214	Q9NXB9	0.18079
P0C214	A7E2Y1	0.89031
P0C214	Q16864	0.041016
P0C214	O00411	0.58296
P0C214	O00534	0.28903
P0C214	Q8TBZ8	0.30731

P0C214	Q96QE5	0.90707
P0C214	P25789	0.80504
P0C214	Q8IZC7	0.18328
P0C214	Q9H633	0.43118
P0C214	Q96LV5	0.032037
P0C214	O75467	0.25256
P0C214	Q9HCS7	0.42757
P0C214	Q6ZN11	0.28997
P0C214	O14529	0.23676
P0C214	Q5JQC4	0.086276
P0C214	Q96A49	1.0901
P0C214	Q9H4X1	0.76162
P0C214	Q8N9V7	0.18802
P0C214	O75691	0.72324
P0C214	Q6TDU7	0.16831
P0C214	Q96LW9	0.25728
P0C214	A0PG75	0.072794
P0C214	Q86TA1	0.24065
P0C214	P60014	0.5539
P0C214	Q9UNZ5	0.24937
P0C214	Q9UID6	0.43062
P0C214	P20290	1.083
P0C214	P11308	0.40685
P0C214	O15117	0.64132
P0C214	Q00653	1.188
P0C214	P27695	0.56236
P0C214	Q9BXY0	0.16769
P0C214	Q96LI9	0.075677
P0C214	Q8IXQ6	0.92806
P0C214	Q8N573	0.14574
P0C214	P05937	0.27333
P0C214	Q9BUL9	1.0716
P0C214	Q14159	0.99264
P0C214	P32243	0.73208
P0C214	O60239	0.36513
P0C214	Q96EL2	0.36621
P0C214	P09917	0.32454
P0C214	P49754	0.39069
P0C214	Q8N228	0.242
P0C214	P35321	0.32578
P0C214	Q5T035	0.11107
P0C214	Q05932	0.29432
P0C214	Q9BYD1	0.84108
P0C214	O43365	0.33513
P0C214	Q6ZTI0	0.086276
P0C214	Q68DX3	0.33652
P0C214	Q9NVH2	0.1981
P0C214	P35813	0.35275
P0C214	Q6ZNA1	0.37033
P0C214	Q00597	0.80726
P0C214	Q9ULR0	0.9001
P0C214	Q9UGY1	0.20534
P0C214	P83369	0.64813
P0C214	P38432	0.66832
P0C214	Q9H9J2	0.3993
P0C214	P63000	0.50445
P0C214	Q09161	0.82825
P0C214	Q75MW2	0.67518
P0C214	Q16537	0.24755
P0C214	O75874	0.20064
P0C214	Q9P0T4	0.47613
P0C214	O43805	0.53033
P0C214	Q8N377	0.086276
P0C214	Q7Z6J0	0.36493
P0C214	P35789	0.43494
P0C214	Q16763	0.92173
P0C214	Q96NT3	0.14522
P0C214	O75486	0.8041
P0C214	P12035	0.44022
P0C214	Q8NDQ6	0.35056
P0C214	Q9UJK0	0.69155
P0C214	Q9H5I1	0.4967
P0C214	Q96PM5	0.52843
P0C214	Q13887	0.25691
P0C214	Q9NSE4	0.42675
P0C214	Q9H0A6	0.28844

P0C214	Q59F38	0.09337
P0C214	Q96C86	1.1084
P0C214	P35227	0.6041
P0C214	O95817	0.51725
P0C214	O14786	0.38641
P0C214	P49639	0.19275
P0C214	O43678	0.60373
P0C214	P26196	0.68172
P0C214	Q9BQS6	0.78784
P0C214	P14625	0.8256
P0C214	Q8IX12	0.92762
P0C214	Q9BQ65	0.10652
P0C214	P15880	1.1747
P0C214	Q6IS14	0.22733
P0C214	Q8ND56	0.55079
P0C214	Q96T58	0.66293
P0C214	P55036	0.72371
P0C214	Q5XKP0	0.46184
P0C214	O76013	0.51477
P0C214	Q96HI0	0.48359
P0C214	P17032	0.28667
P0C214	P61244	0.78256
P0C214	Q12894	0.39711
P0C214	Q9ULE6	0.74795
P0C214	O14595	0.1896
P0C214	Q06609	0.41319
P0C214	Q5H9M0	0.93308
P0C214	O75663	0.38021
P0C214	P62081	1.2121
P0C214	P42766	1.0598
P0C214	Q9H040	0.39826
P0C214	P11413	0.15753
P0C214	Q12904	0.35796
P0C214	Q14593	0.4838
P0C214	B1AH88	0.086276
P0C214	Q15697	0.41118
P0C214	Q8WW12	0.58979
P0C214	Q9NV31	0.84485
P0C214	A6NGD5	0.22448
P0C214	A8MVS1	0.48315
P0C214	Q6U949	0.086276
P0C214	Q7L8S5	0.37727
P0C214	Q9Y5Z7	0.32801
P0C214	Q9HCZ1	0.27075
P0C214	Q9UI10	0.43653
P0C214	P30307	0.78514
P0C214	P19474	0.45316
P0C214	Q14CX7	0.42462
P0C214	P84101	0.75796
P0C214	Q9H606	0.07667
P0C214	Q02930	0.69066
P0C214	Q04727	0.55649
P0C214	Q9H875	0.31171
P0C214	Q6P0N0	0.93317
P0C214	P56270	0.51272
P0C214	Q7L3T8	0.33602
P0C214	Q9Y2S7	0.66839
P0C214	Q7Z4Q2	0.80089
P0C214	P62913	1.2981
P0C214	O43829	0.52796
P0C214	Q92546	0.81442
P0C214	Q9HAU4	0.52804
P0C214	Q29RF7	0.94319
P0C214	P48788	0.61356
P0C214	Q9UK99	0.39219
P0C214	P29966	0.81427
P0C214	Q5HYW3	0.33636
P0C214	Q9NVH6	0.33741
P0C214	O15111	0.54262
P0C214	Q969Z0	0.42597
P0C214	Q15022	0.53566
P0C214	P68133	0.10604
P0C214	O95810	0.30566
P0C214	A0AVK6	0.29381
P0C214	P22090	0.586
P0C214	Q969G5	0.44153

P0C214	Q8IUC1	0.16924
P0C214	Q8WXF8	1.0814
P0C214	Q9NPP4	0.25268
P0C214	Q00613	0.78198
P0C214	Q9H3H3	0.067003
P0C214	Q9BSJ2	1.0983
P0C214	Q6NZ67	0.57701
P0C214	P35869	0.31809
P0C214	Q7L5A3	0.4476
P0C214	Q9Y5V3	0.67422
P0C214	P51508	0.3048
P0C214	Q13469	1.131
P0C214	Q15024	0.87089
P0C214	Q7Z398	0.26542
P0C214	O95363	0.56206
P0C214	Q15154	0.55287
P0C214	B9ZVM9	1.1928
P0C214	P22695	0.10746
P0C214	Q06787	0.81438
P0C214	Q9UIA9	0.46163
P0C214	Q8IWN6	0.13362
P0C214	Q8TAY7	1.3916
P0C214	Q9NZY2	0.086276
P0C214	Q13573	0.61319
P0C214	Q96MC4	0.65403
P0C214	O95045	0.38549
P0C214	Q9UGB4	0.086276
P0C214	Q9NWN3	0.36372
P0C214	P53672	0.65733
P0C214	Q99643	0.35424
P0C214	Q8NCN5	0.31705
P0C214	O60663	0.37105
P0C214	Q96M53	1.0978
P0C214	Q8N0Z2	0.24219
P0C214	Q14409	0.58072
P0C214	Q9NRL2	0.91961
P0C214	Q9BVC4	0.42312
P0C214	Q5JVS0	0.99479
P0C214	P42166	0.15838
P0C214	P04818	0.19652
P0C214	Q32M84	0.14741
P0C214	Q8WUX2	0.27044
P0C214	Q14331	0.51198
P0C214	Q99832	0.16168
P0C214	P24522	0.47492
P0C214	O14787	0.84428
P0C214	Q9P299	0.080403
P0C214	O43172	0.68299
P0C214	Q8WUX9	0.48809
P0C214	P16989	0.16959
P0C214	O95260	0.063476
P0C214	O15061	0.54813
P0C214	Q8N335	0.20341
P0C214	Q14207	0.85255
P0C214	Q9C002	0.18383
P0C214	P51610	0.40738
P0C214	P60369	0.43378
P0C214	P11474	0.24379
P0C214	O14681	0.40999
P0C214	Q9H4A6	0.48128
P0C214	Q96M95	0.070287
P0C214	Q9H6Y2	0.44497
P0C214	Q9UGM6	0.069137
P0C214	O75528	0.69428
P0C214	Q9NPE3	0.68813
P0C214	Q58FF7	0.49678
P0C214	O60841	0.3278
P0C214	Q9BU76	0.43827
P0C214	A8MQ14	0.29081
P0C214	Q8NEF3	0.3475
P0C214	Q15047	0.6366
P0C214	Q13330	0.34035
P0C214	Q8NFH5	0.58641
P0C214	Q5T752	0.086276
P0C214	Q96CF2	0.146
P0C214	O14746	0.58345

P0C214	Q8IZP0	0.29899
P0C214	Q8IY31	0.39164
P0C214	Q9H788	0.93554
P0C214	Q86V87	0.41473
P0C214	Q8N2H4	1.1044
P0C214	Q71SY5	0.97172
P0C214	Q96BT3	0.48483
P0C214	P52743	0.39773
P0C214	Q99728	0.29377
P0C214	Q13620	0.75087
P0C214	P35637	0.43023
P0C214	O75896	0.24882
P0C214	P55060	0.73489
P0C214	Q8WY50	0.086276
P0C214	Q8N6F7	0.37404
P0C214	O94868	0.2675
P0C214	Q9BRU2	0.45356
P0C214	A6NF83	0.55397
P0C214	Q13156	0.35739
P0C214	O95238	0.080499
P0C214	O95153	0.65701
P0C214	Q13952	0.60666
P0C214	Q08AD1	0.35925
P0C214	Q9BQ83	0.54619
P0C214	Q9Y5L5	0.93784
P0C214	Q8TBZ0	0.27704
P0C214	Q9Y6K9	0.95733
P0C214	Q9BT49	0.54815
P0C214	Q9BU89	0.36654
P0C214	P31350	0.51661
P0C214	Q8TD31	0.45369
P0C214	Q8IV54	0.37495
P0C214	Q5TYX0	0.402
P0C214	Q17R26	0.35498
P0C214	O94913	0.27156
P0C214	P82912	0.50373
P0C214	Q06546	1.0429
P0C214	Q92901	0.86977
P0C214	Q9Y5Q8	0.61983
P0C214	P0C7V0	0.086276
P0C214	Q8WUY3	0.26085
P0C214	Q96P70	0.87382
P0C214	Q5HYN5	0.22704
P0C214	P62140	0.80138
P0C214	O95456	0.44945
P0C214	A6NK39	0.25676
P0C214	Q13363	0.47716
P0C214	P28062	0.40103
P0C214	Q14008	0.77212
P0C214	Q9UK55	0.85505
P0C214	Q4VC44	0.16987
P0C214	Q6ZU35	0.24937
P0C214	Q5UIP0	0.8131
P0C214	Q53QW1	0.24937
P0C214	P05783	1.1509
P0C214	Q9Y6J9	0.72238
P0C214	P62249	0.85445
P0C214	Q6RVD6	0.086276
P0C214	Q9Y3D9	0.41291
P0C214	O75461	0.11465
P0C214	P13489	0.38968
P0C214	Q8N6T0	0.010672
P0C214	Q9P0L1	0.2988
P0C214	Q9UKX2	0.89031
P0C214	Q0VFX4	0.2635
P0C214	Q86YD1	0.63582
P0C214	Q9H0A8	0.48513
P0C214	P35527	0.58756
P0C214	A6NFL8	0.49852
P0C214	Q8N8F4	0.14839
P0C214	P17010	0.25573
P0C214	P61956	0.65288
P0C214	O43257	0.44843
P0C214	O95633	0.27116
P0C214	Q3LHN0	0.6322
P0C214	O95478	0.40314

P0C214	Q63HK3	0.34481
P0C214	Q9Y2G9	0.62332
P0C214	Q9ULD5	0.23038
P0C214	P55347	0.3198
P0C214	Q9Y376	0.20674
P0C214	Q6P2E9	1.1785
P0C214	Q99747	0.42413
P0C214	A8K8V0	0.24946
P0C214	F8WFD2	0.13188
P0C214	P49368	0.26453
P0C214	P23919	0.39245
P0C214	Q15388	0.18724
P0C214	Q6NXS1	0.68669
P0C214	Q86WR0	0.037552
P0C214	Q495Z4	0.27196
P0C214	Q9UHF7	0.34568
P0C214	P49901	0.086276
P0C214	Q9H4I8	0.27316
P0C214	P09914	0.47805
P0C214	O75934	0.66077
P0C214	Q8N9N2	0.31448
P0C214	P00480	0.30825
P0C214	O95198	0.32036
P0C214	Q8IYT1	0.32585
P0C214	Q969E8	1.1057
P0C214	Q1MSJ5	0.92214
P0C214	Q8WV44	0.36895
P0C214	Q6AWC8	0.13764
P0C214	Q9Y4A8	0.38157
P0C214	O94986	1.1951
P0C214	P62899	0.86977
P0C214	O75764	0.11999
P0C214	O95453	0.63718
P0C214	Q17RA5	0.086276
P0C214	Q8IV03	0.85366
P0C214	Q9BV47	0.42756
P0C214	Q15651	0.71738
P0C214	O43830	0.22082
P0C214	A5LHX3	0.44255
P0C214	Q96S55	0.30457
P0C214	Q7Z591	0.85227
P0C214	Q9BSF8	0.35391
P0C214	Q13547	0.77379
P0C214	A8K830	0.086276
P0C214	Q12988	0.39692
P0C214	Q9BS34	0.20002
P0C214	Q9Y2A4	0.4876
P0C214	Q14865	0.25184
P0C214	Q86Y46	0.34216
P0C214	Q9BS18	0.35101
P0C214	Q8TEA8	0.91447
P0C214	Q8WWW0	0.24574
P0C214	P60002	0.68831
P0C214	Q8NFI3	0.10738
P0C214	Q6PEW1	0.069529
P0C214	A7E2U8	0.55347
P0C214	P78337	0.32964
P0C214	Q6NUI1	0.37122
P0C214	Q96KD3	0.907
P0C214	Q9HAN9	0.49203
P0C214	Q7Z3Y8	0.5102
P0C214	Q9HAH7	0.32226
P0C214	Q12815	0.23856
P0C214	Q9HCD5	1.0428
P0C214	Q6IPW1	0.43494
P0C214	Q8NEE0	0.086276
P0C214	P0CI00	0.5624
P0C214	P49959	0.1603
P0C214	P35558	0.58613
P0C214	P16157	0.23817
P0C214	Q8NCR6	0.2723
P0C214	P02538	0.57271
P0C214	Q12824	0.90821
P0C214	Q86VG3	0.22321
P0C214	O00762	0.66402
P0C214	Q66PJ3	0.57695

P0C214	Q5SNX5	0.086276
P0C214	P04264	0.20757
P0C214	Q9UPX8	0.29151
P0C214	P09913	0.62802
P0C214	Q8TE04	0.60862
P0C214	Q9H2L5	0.5203
P0C214	Q14872	0.79
P0C214	Q53EQ6	0.31376
P0C214	P11177	0.19907
P0C214	Q8IXK0	0.13893
P0C214	Q6ZR54	0.086276
P0C214	Q96DV4	0.22263
P0C214	O00148	0.35223
P0C214	Q9HAB8	0.44729
P0C214	Q8N7Q3	0.37857
P0C214	Q13490	0.51641
P0C214	Q13772	0.36732
P0C214	Q86SE9	0.97022
P0C214	P31249	0.39784
P0C214	Q6XCG6	0.086276
P0C214	P38159	0.60754
P0C214	Q9H816	0.42744
P0C214	Q92624	0.67383
P0C214	Q6ZRS4	0.25438
P0C214	Q16775	0.44639
P0C214	Q8IYN0	0.36263
P0C214	Q5QP74	0.086276
P0C214	P56556	0.41917
P0C214	P49902	0.26606
P0C214	Q14929	0.25251
P0C214	Q9UKF6	0.69101
P0C214	Q8NHY5	1.0288
P0C214	Q96KR1	0.29373
P0C214	Q96C34	0.64272
P0C214	Q6IEG0	0.31423
P0C214	Q8NF64	1.3134
P0C214	P28838	0.34249
P0C214	A8MVA2	0.43378
P0C214	P17023	0.25564
P0C214	Q9NRR8	0.49591
P0C214	Q9BX82	0.48724
P0C214	Q9BR77	0.27061
P0C214	P12757	0.26208
P0C214	O75909	0.95913
P0C214	A8MV72	0.012015
P0C214	Q53HL2	0.91805
P0C214	C9JE40	0.8012
P0C214	Q8NDD1	0.0062454
P0C214	Q5SWL7	0.58313
P0C214	Q00005	0.38259
P0C214	Q6R2W3	0.38236
P0C214	P62854	1.0548
P0C214	Q9NWD9	0.41726
P0C214	Q6PJ61	0.96206
P0C214	P78318	0.88955
P0C214	Q9Y2D9	0.56957
P0C214	O00321	0.20656
P0C214	Q6RW13	0.66383
P0C214	P07864	1.0002
P0C214	Q6VMQ6	0.94906
P0C214	P56945	0.54264
P0C214	Q9Y4C2	0.2489
P0C214	Q9H201	0.20521
P0C214	Q13505	0.78974
P0C214	Q14591	0.22636
P0C214	P30876	0.56203
P0C214	Q16740	0.32252
P0C214	Q96JN0	0.42574
P0C214	O75351	0.2885
P0C214	Q9Y5A6	0.65582
P0C214	Q86W67	0.060555
P0C214	P53992	0.96757
P0C214	O43264	0.49313
P0C214	Q8NI35	0.24774
P0C214	Q5T9C2	0.24937
P0C214	P28065	0.76068

P0C214	O15194	0.074564
P0C214	P39019	1.0557
P0C214	O96033	0.20122
P0C214	Q6ZN57	0.22955
P0C214	P62263	0.72831
P0C214	Q9HAV7	0.49939
P0C214	Q8NDF8	0.29572
P0C214	Q9P2B7	0.24937
P0C214	Q8N5D0	0.56789
P0C214	Q8IWP9	0.81347
P0C214	P51814	0.20378
P0C214	O75347	1.1235
P0C214	P02042	0.3641
P0C214	Q5T5D7	0.20437
P0C214	O95684	0.85563
P0C214	Q9H706	0.2675
P0C214	Q08999	1.2333
P0C214	Q86Y79	0.49873
P0C214	Q9BRP8	1.0545
P0C214	P60842	0.30637
P0C214	Q8N7X2	0.086276
P0C214	P19838	0.66343
P0C214	Q05481	0.38021
P0C214	Q6UUV7	1.6697
P0C214	P46087	0.75649
P0C214	Q6ZP68	0.62598
P0C214	P82675	0.52012
P0C214	P14921	0.70892
P0C214	P0C843	0.020304
P0C214	Q15628	0.59177
P0C214	A6NHJ4	0.49186
P0C214	A1L190	0.69814
P0C214	O94842	0.58949
P0C214	Q96SQ5	0.30462
P0C214	Q9H9E3	0.31755
P0C214	P57721	0.70133
P0C214	Q86XF0	0.1818
P0C214	P49770	0.35447
P0C214	Q9Y3F1	0.086276
P0C214	Q9UI12	0.056885
P0C214	Q8WUF5	0.74194
P0C214	Q6ZU67	0.3852
P0C214	Q92664	0.10599
P0C214	Q96MT4	0.53987
P0C214	Q0VG73	0.086276
P0C214	Q6ZRH4	1.1176
P0C214	Q15486	0.40142
P0C214	Q9Y3C4	0.53551
P0C214	Q9H079	0.33626
P0C214	Q9NRI5	0.27882
P0C214	Q9Y262	0.74286
P0C214	P0CB33	0.44927
P0C214	Q86XA9	0.26755
P0C214	P41227	0.37556
P0C214	Q9UL59	0.31247
P0C214	Q8IVF1	0.91547
P0C214	Q15013	0.76378
P0C214	Q3SYF9	0.086276
P0C214	Q8N402	0.074324
P0C214	P15259	1.128
P0C214	P30153	0.56538
P0C214	P78371	0.76281
P0C214	O75925	0.49633
P0C214	Q7Z4H3	0.28632
P0C214	P52744	0.30614
P0C214	P41002	0.87346
P0C214	Q9UJW0	0.4826
P0C214	Q9BSB4	0.2423
P0C214	Q8N584	0.55956
P0C214	Q8WXE1	0.62091
P0C214	Q96IV0	0.45469
P0C214	Q9UPV9	0.77336
P0C214	Q02241	0.61714
P0C214	Q6ZNH5	0.28971
P0C214	Q75NE6	1.0342
P0C214	P13646	0.55013

P0C214	Q9BRV8	0.57768
P0C214	Q96JI7	0.64328
P0C214	Q9Y6C7	0.086276
P0C214	Q9NQ35	0.59129
P0C214	Q15573	0.40209
P0C214	Q6ZUG5	0.020415
P0C214	Q9H9Y6	0.67702
P0C214	Q96IZ0	0.64959
P0C214	Q5SXJ2	1.2293
P0C214	Q9Y2J4	0.34989
P0C214	Q3KNT7	0.2308
P0C214	O14497	0.67251
P0C214	Q6AI08	0.48229
P0C214	Q7Z5D8	0.40657
P0C214	Q9NXZ1	0.16769
P0C214	Q52LR7	0.36197
P0C214	Q9NXX6	0.3376
P0C214	P0C6E6	0.13509
P0C214	Q9H0U6	0.42926
P0C214	P62993	0.25958
P0C214	P02533	0.86902
P0C214	Q5T5J6	0.81315
P0C214	P59780	0.07925
P0C214	Q6BDS2	0.20101
P0C214	Q5JVG8	0.46355
P0C214	Q96BY2	0.40263
P0C214	Q8N699	0.1459
P0C214	Q9UJJ7	0.43032
P0C214	Q9BU68	0.20005
P0C214	A6NJV1	0.17012
P0C214	Q8IWY8	0.30132
P0C214	Q8NC26	0.41325
P0C214	P49789	0.69453
P0C214	Q5SYE7	0.33359
P0C214	Q9UJY5	0.39473
P0C214	Q9P0V3	0.56008
P0C214	Q0VF49	0.086276
P0C214	Q5TG53	0.086276
P0C214	Q9BY44	0.35213
P0C214	Q96I59	0.73781
P0C214	O75586	0.62749
P0C214	Q9NYK5	0.6721
P0C214	Q7Z3Y7	0.38714
P0C214	Q8N5A5	0.68783
P0C214	O14829	0.30127
P0C214	P15336	0.78122
P0C214	Q9UFW8	0.34832
P0C214	Q96MC9	0.93983
P0C214	Q969Q6	0.20171
P0C214	Q969S8	0.72963
P0C214	Q9BUI4	0.16748
P0C214	Q9P086	0.98887
P0C214	Q96M91	0.56431
P0C214	O75390	0.37778
P0C214	P78543	0.55792
P0C214	Q7Z6G3	0.37391
P0C214	Q9Y473	0.3031
P0C214	Q06547	0.9729
P0C214	Q13568	1.0042
P0C214	Q86Y56	0.016653
P0C214	Q6V702	0.093126
P0C214	Q9NRR5	0.82926
P0C214	P52657	1.1526
P0C214	Q92889	0.19799
P0C214	Q96LW1	0.47199
P0C214	P54922	0.46105
P0C214	P13010	0.3707
P0C214	Q8IZL2	0.81561
P0C214	P06746	0.40937
P0C214	P02489	0.26511
P0C214	Q9BYQ5	0.43378
P0C214	Q9NS23	0.23376
P0C214	Q8IUC6	0.23121
P0C214	Q9P016	0.13404
P0C214	O43913	0.37394
P0C214	Q15051	0.46852

P0C214	A6NP11	0.50756
P0C214	Q5SVZ6	0.71154
P0C214	C9J302	0.14467
P0C214	Q16540	0.28646
P0C214	Q99698	0.24147
P0C214	Q9BUT9	0.93811
P0C214	P54253	0.88897
P0C214	Q6P1X6	0.47099
P0C214	Q9NQ55	0.32844
P0C214	P54098	0.437
P0C214	Q15003	0.79975
P0C214	Q9UH90	0.34482
P0C214	Q9NQ89	0.22796
P0C214	O14879	0.8085
P0C214	Q969H6	0.89618
P0C214	Q6ZS17	0.4149
P0C214	I3L1I5	0.33801
P0C214	Q9UHQ7	0.51847
P0C214	Q9H9F9	1.1324
P0C214	Q96I27	0.22501
P0C214	Q7L0Y3	0.98738
P0C214	Q9BRA2	0.58224
P0C214	Q9UIL8	0.25884
P0C214	Q96G75	0.079678
P0C214	Q96S95	0.40139
P0C214	Q9NP81	0.52517
P0C214	Q8WXC7	0.42083
P0C214	Q9BYP8	0.086276
P0C214	Q6UX04	0.65778
P0C214	Q16186	0.54822
P0C214	P21675	0.38579
P0C214	Q7L266	0.25538
P0C214	O15535	0.21155
P0C214	O00159	0.073424
P0C214	P53803	0.78265
P0C214	Q9BRK4	0.64956
P0C214	Q9H8S9	0.54429
P0C214	Q9H175	0.92208
P0C214	Q9BXS1	0.41641
P0C214	P84103	0.57542
P0C214	Q15361	0.69493
P0C214	A2BFH1	0.77514
P0C214	Q96QR8	0.33811
P0C214	Q8TD17	0.22145
P0C214	Q6PG37	0.50814
P0C214	P12081	0.02571
P0C214	P46379	0.25015
P0C214	O43159	0.85111
P0C214	Q8N0Z6	0.22269
P0C214	Q6L8H1	0.086276
P0C214	Q5I0G3	0.40746
P0C214	Q6PJG6	0.63046
P0C214	O60716	0.51615
P0C214	Q7Z699	0.2397
P0C214	Q9NPI6	1.1088
P0C214	Q99865	0.14921
P0C214	Q96GK7	0.48674
P0C214	Q96ED9	0.28421
P0C214	Q9NVU0	0.41567
P0C214	Q53TQ3	0.50282
P0C214	P07476	0.21182
P0C214	Q9BTX9	0.63452
P0C214	Q86V81	0.91689
P0C214	P43360	0.41816
P0C214	A6NHQ4	0.1985
P0C214	Q96LI6	0.36475
P0C214	P57772	0.70618
P0C214	Q9UJ94	0.086276
P0C214	Q2NL68	0.24937
P0C214	Q6P161	0.12426
P0C214	P22735	0.1359
P0C214	Q9NTW7	0.46536
P0C214	P02511	0.47097
P0C214	Q8TF45	0.25925
P0C214	Q04917	0.23645
P0C214	O76015	0.5102

30021	P0C214	P0C7V9	0.37656
30022	P0C214	Q9BSI4	0.10549
30023	P0C214	O60759	0.32417
30024	P0C214	P48643	0.13685
30025	P0C214	Q9Y2K3	0.89031
30026	P0C214	O60826	0.7296
30027	P0C214	O94811	0.35929
30028	P0C214	Q9UBB4	1.0936
30029	P0C214	Q08379	0.22202
30030	P0C214	O43186	0.73974
30031	P0C214	P48552	0.62283
30032	P0C214	Q9BWC9	0.70657
30033	P0C214	A8MZF0	0.24937
30034	P0C214	B3EWG5	0.24937
30035	P0C214	Q9BSM1	0.99405
30036	P0C214	P86479	0.086276
30037	P0C214	Q96A44	0.46359
30038	P0C214	Q9NWS8	0.64205
30039	P0C214	Q8NG57	0.31331
30040	P0C214	P62380	0.11343
30041	P0C214	Q9Y676	0.61883
30042	P0C214	Q16656	0.3444
30043	P0C214	A8MTY7	0.43378
30044	P0C214	Q9UKJ5	0.27919
30045	P0C214	Q14164	0.26145
30046	P0C214	Q9C0F0	0.34036
30047	P0C214	P61758	0.61963
30048	P0C214	O75820	0.26437
30049	P0C214	Q9UPT5	0.49502
30050	P0C214	Q96F24	0.65072
30051	P0C214	Q9BPZ2	0.1626
30052	P0C214	Q6DKK2	0.85131
30053	P0C214	Q5VTL8	0.49481
30054	P0C214	Q14203	0.8963
30055	P0C214	P53396	0.24387
30056	P0C214	Q9BWM5	0.30522
30057	P0C214	Q8TAQ5	0.3804
30058	P0C214	Q9Y281	0.46018
30059	P0C214	Q92934	0.070743
30060	P0C214	P31314	0.44717
30061	P0C214	Q9UDW3	0.25982
30062	P0C214	P23381	0.50782
30063	P0C214	Q96N21	0.38524
30064	P0C214	Q9BYR7	0.92503
30065	P0C214	Q9UNX4	0.28317
30066	P0C214	Q86TS9	0.31815
30067	P0C214	P52742	0.34248
30068	P0C214	Q13625	0.38016
30069	P0C214	Q9Y5S9	0.85649
30070	P0C214	Q5TB30	0.33549
30071	P0C214	P07741	0.39871
30072	P0C214	Q6ZTU2	0.5536
30073	P0C214	Q9BZK3	0.53566
30074	P0C214	Q8NCR3	0.17761
30075	P0C214	O94888	0.33185
30076	P0C214	Q96DB2	0.23087
30077	P0C214	Q8IU53	0.16193
30078	P0C214	A6NFY7	0.5047
30079	P0C214	P61571	0.36767
30080	P0C214	Q96FJ8	0.086276
30081	P0C214	P01011	0.8218
30082	P0C214	O60543	0.36354
30083	P0C214	P51003	0.22844
30084	P0C214	Q93015	0.43723
30085	P0C214	P27348	0.28134
30086	P0C214	Q7Z4H7	0.73887
30087	P0C214	Q8NET4	0.50063
30088	P0C214	Q8TC56	0.907
30089	P0C214	O14645	0.14089
30090	P0C214	G3V211	0.5062
30091	P0C214	P62273	0.92915
30092	P0C214	P81274	0.51145
30093	P0C214	Q13769	0.53429
30094	P0C214	Q14587	0.24611
30095	P0C214	Q8WTW4	0.23352
30096	P0C214	Q5TCQ9	0.53213

30097	P0C214	O60739	0.2439
30098	P0C214	Q12873	0.56405
30099	P0C214	P15407	0.62436
30100	P0C214	O95295	0.47055
30101	P0C214	Q9NPC7	0.20138
30102	P0C214	Q9NP92	0.8541
30103	P0C214	A4D0Y5	0.086276
30104	P0C214	Q86U90	0.61031
30105	P0C214	P51825	0.92592
30106	P0C214	Q9BYR5	0.43378
30107	P0C214	P52655	1.1378
30108	P0C214	A6NCD4	0.1624
30109	P0C214	Q5VWN6	0.13677
30110	P0C214	P06748	0.41808
30111	P0C214	O60508	0.29893
30112	P0C214	Q9P209	0.77758
30113	P0C214	Q96GT9	0.32396
30114	P0C214	O95628	0.91542
30115	P0C214	Q96LC9	0.58124
30116	P0C214	Q6ZUT1	0.086276
30117	P0C214	Q13829	0.71584
30118	P0C214	Q8WYN3	0.48016
30119	P0C214	Q9Y3C0	0.2517
30120	P0C214	Q6K0P9	0.18039
30121	P0C214	P35269	1.0799
30122	P0C214	Q15554	0.41032
30123	P0C214	P50750	0.312
30124	P0C214	Q96LT6	0.17118
30125	P0C214	Q86UE3	0.37033
30126	P0C214	P60371	0.43378
30127	P0C214	Q659A1	0.079957
30128	P0C214	P26599	0.31298
30129	P0C214	Q8NHQ1	1.3814
30130	P0C214	Q9UIH9	0.31943
30131	P0C214	P58505	0.17054
30132	P0C214	P23396	0.92076
30133	P0C214	P27815	0.43999
30134	P0C214	Q92900	0.62181
30135	P0C214	Q6ZQR2	0.9143
30136	P0C214	O00458	0.7619
30137	P0C214	Q9Y255	0.09056
30138	P0C214	Q9H1E3	0.53076
30139	P0C214	Q7L590	0.9859
30140	P0C214	P18077	0.75023
30141	P0C214	Q9UI25	0.086276
30142	P0C214	Q9HD15	0.45833
30143	P0C214	Q8TBC5	0.067802
30144	P0C214	P19484	0.44689
30145	P0C214	P25208	0.53526
30146	P0C214	Q2TAK8	0.58259
30147	P0C214	Q99081	0.3787
30148	P0C214	Q00987	0.31125
30149	P0C214	O15235	0.73233
30150	P0C214	A6NJR5	0.28575
30151	P0C214	O95567	0.086276
30152	P0C214	Q96FX2	0.86903
30153	P0C214	Q9GZU2	0.45087
30154	P0C214	Q9GZV4	0.71056
30155	P0C214	O15037	0.17626
30156	P0C214	Q8NDP4	0.30705
30157	P0C214	O00267	0.97958
30158	P0C214	Q3LI54	0.086276
30159	P0C214	Q96CX3	0.32018
30160	P0C214	P33316	0.702
30161	P0C214	Q96NJ3	0.27255
30162	P0C214	Q9UHT9	0.086276
30163	P0C214	Q8N693	0.34369
30164	P0C214	Q16222	0.36887
30165	P0C214	P52738	0.26578
30166	P0C214	P50613	0.21354
30167	P0C214	Q9Y4E1	0.35898
30168	P0C214	P43487	0.36849
30169	P0C214	Q92841	0.6965
30170	P0C214	P05388	1.5805
30171	P0C214	P28331	0.188
30172	P0C214	Q6ZUT4	0.26672

P0C214	O95400	0.74605
P0C214	Q96G04	0.069093
P0C214	A8MU93	0.24937
P0C214	A8MSI8	1.0139
P0C214	P60410	0.6322
P0C214	Q6L8G4	0.086276
P0C214	Q3T8J9	0.57316
P0C214	Q9NTK1	0.54594
P0C214	Q6NXR4	0.04166
P0C214	P55212	0.48642
P0C214	Q8TEM1	0.17016
P0C214	Q9H3M7	0.80797
P0C214	Q13309	0.70546
P0C214	O75190	0.86176
P0C214	Q8TAD8	1.2548
P0C214	Q9H814	0.47665
P0C214	Q7KZ85	0.43529
P0C214	Q8TC05	0.46462
P0C214	Q9BU61	0.69929
P0C214	Q13112	1.4717
P0C214	O43920	0.75168
P0C214	Q9H171	0.2696
P0C214	P40121	0.39842
P0C214	Q92796	0.26968
P0C214	Q15014	1.1553
P0C214	Q5MJ07	0.46567
P0C214	A6NHP3	0.28575
P0C214	P49427	0.85585
P0C214	O15372	0.79591
P0C214	Q9Y2L1	0.66363
P0C214	P33076	0.4123
P0C214	Q5VT03	0.39603
P0C214	Q9Y2X0	0.48556
P0C214	Q09666	0.65215
P0C214	Q0VFZ6	0.24937
P0C214	Q9H6J7	0.16769
P0C214	Q96RK0	0.90001
P0C214	Q5VWT5	0.41817
P0C214	Q9H246	0.2193
P0C214	P29084	1.1704
P0C214	Q5T0Z8	0.24937
P0C214	Q5JVG2	0.21855
P0C214	Q7Z465	0.68406
P0C214	P0CG31	0.29774
P0C214	Q8N8H1	0.46577
P0C214	P49590	0.055572
P0C214	P12694	0.2372
P0C214	P23025	0.19847
P0C214	Q2NL82	0.46222
P0C214	A6NCI8	0.80356
P0C214	Q7Z6R9	0.24424
P0C214	Q9BW71	0.57163
P0C214	Q9NR56	0.41397
P0C214	Q96QK1	0.6366
P0C214	P07814	0.55922
P0C214	Q96K62	0.27834
P0C214	P62847	0.7392
P0C214	O75618	1.0596
P0C214	Q96BR6	0.40332
P0C214	Q9HBE1	0.18816
P0C214	P29622	0.96136
P0C214	Q96C90	0.67014
P0C214	Q8TAC9	0.47471
P0C214	Q8NCA9	0.18456
P0C214	Q8TAT8	0.0036186
P0C214	Q9BYD3	0.73176
P0C214	Q8WTT2	0.34352
P0C214	Q8NEH6	0.80476
P0C214	P23588	0.41622
P0C214	Q9HBF5	0.55347
P0C214	P42226	0.7832
P0C214	Q6ZW54	0.96495
P0C214	Q5H9I0	0.3453
P0C214	Q96GC5	0.68937
P0C214	Q9UM54	0.31733
P0C214	P43358	0.41816

P0C214	P42898	0.18698
P0C214	Q7L2R6	0.44782
P0C214	Q460N5	0.41142
P0C214	P51959	0.45004
P0C214	Q7RTU4	0.25688
P0C214	Q9Y2S6	0.086276
P0C214	P15884	0.48509
P0C214	O76021	0.53502
P0C214	Q6ZMV5	0.38023
P0C214	Q13158	0.44609
P0C214	Q6ZRP5	0.086276
P0C214	Q5SW79	0.67949
P0C214	Q9BYA6	0.086276
P0C214	Q96EI5	0.75796
P0C214	Q8NHW3	0.15678
P0C214	Q7Z794	0.54908
P0C214	Q9H0S4	0.16164
P0C214	Q9BQ61	0.24937
P0C214	Q96MF6	0.45028
P0C214	P36952	0.78972
P0C214	Q5JS54	0.20558
P0C214	Q9NS56	0.27185
P0C214	O75478	0.26803
P0C214	Q8TD23	0.5619
P0C214	Q538Z0	0.086276
P0C214	O14818	0.76226
P0C214	O60907	0.72309
P0C214	O14579	0.57465
P0C214	Q9NV39	0.25186
P0C214	Q9UJP4	0.21649
P0C214	B1AL46	0.39603
P0C214	Q9P2F9	0.22643
P0C214	Q96M85	0.086276
P0C214	O15350	0.27346
P0C214	Q8TBN0	0.21579
P0C214	P13349	0.23151
P0C214	P40855	0.33636
P0C214	Q9C035	0.63635
P0C214	Q9NP79	0.22499
P0C214	Q9NX20	0.44456
P0C214	Q8N587	0.44914
P0C214	P15924	0.23748
P0C214	Q53FD0	0.50333
P0C214	A6NGN4	0.28595
P0C214	P14678	0.88416
P0C214	Q99856	0.61144
P0C214	Q9NY27	0.81775
P0C214	Q14764	0.9427
P0C214	Q8TAU3	0.30462
P0C214	Q96EA4	0.66963
P0C214	Q8WWM1	0.43185
P0C214	Q9BUA3	0.028204
P0C214	P86496	0.086276
P0C214	Q9C0C2	0.24762
P0C214	O00167	0.66455
P0C214	Q8N2W9	1.0035
P0C214	Q14119	0.19264
P0C214	Q9BYR8	0.6322
P0C214	Q14CW9	0.83329
P0C214	Q03112	0.17412
P0C214	Q9UJN7	0.25959
P0C214	Q86UB2	0.46036
P0C214	Q9Y2D8	0.53321
P0C214	Q9BTW9	0.26811
P0C214	P17019	0.49815
P0C214	P60412	0.43378
P0C214	Q9BWH6	0.58277
P0C214	Q8WWX9	0.51521
P0C214	Q13015	0.47824
P0C214	Q6PH85	0.0027648
P0C214	Q9BQ87	0.39389
P0C214	Q8N0Y2	0.21909
P0C214	Q9P1Y5	0.61168
P0C214	Q9Y285	0.66461
P0C214	Q8IVP9	0.17344
P0C214	Q9UC06	0.2912

P0C214	O60927	0.74413
P0C214	P62829	1.3176
P0C214	P83916	0.60402
P0C214	Q6ZV60	0.086276
P0C214	C9JN71	0.33203
P0C214	O75157	0.37294
P0C214	Q504Q3	0.38884
P0C214	O75530	0.57202
P0C214	P61580	0.37639
P0C214	Q96BW1	0.19776
P0C214	Q02080	0.35413
P0C214	A6NGH7	0.15258
P0C214	Q13023	0.54093
P0C214	O75807	0.51146
P0C214	O75496	0.90344
P0C214	Q9H444	0.67645
P0C214	Q9Y2V2	0.46934
P0C214	Q53HC0	0.71663
P0C214	P41252	0.90389
P0C214	Q96SN8	0.42625
P0C214	P07954	0.27316
P0C214	Q15029	0.38426
P0C214	P26373	1.2111
P0C214	Q8N1B3	0.52979
P0C214	Q5T230	1.448
P0C214	Q14765	0.29286
P0C214	Q9BZE3	0.32752
P0C214	Q9C086	1.4268
P0C214	P0CG30	0.29858
P0C214	O60220	0.49725
P0C214	Q9BQ66	0.43378
P0C214	Q9HB09	0.89831
P0C214	Q495M9	0.33188
P0C214	O00541	0.35725
P0C214	Q8N5F7	1.245
P0C214	Q96SD1	0.11392
P0C214	Q86WW8	0.37663
P0C214	Q9BYQ2	0.43378
P0C214	Q68DY1	0.49815
P0C214	Q495Y8	0.28575
P0C214	Q9UDY8	0.33479
P0C214	Q8NDY6	0.39843
P0C214	Q4LEZ3	0.92572
P0C214	Q6ZSA8	0.086276
P0C214	Q86UW6	0.21915
P0C214	Q8TF09	0.45551
P0C214	Q5TAA0	0.021619
P0C214	Q16621	0.43533
P0C214	Q96R06	0.81127
P0C214	Q5MCW4	0.25059
P0C214	Q8N183	0.29742
P0C214	P62306	0.41319
P0C214	Q9Y2T2	0.40315
P0C214	Q8NA72	0.11376
P0C214	Q13867	0.43888
P0C214	P01100	0.49211
P0C214	Q7Z4G1	1.2595
P0C214	Q9H227	0.23355
P0C214	Q969L4	0.63275
P0C214	Q16384	0.2686
P0C214	Q14152	1.2231
P0C214	Q15599	0.31638
P0C214	Q5W111	0.1545
P0C214	Q96GV9	0.29058
P0C214	Q9HCM7	0.84135
P0C214	Q9P2N5	0.19497
P0C214	Q9BV57	0.82436
P0C214	Q9BXS6	0.537
P0C214	Q99676	0.36207
P0C214	P11844	0.65733
P0C214	Q9HCI6	0.47696
P0C214	Q6NT89	0.59295
P0C214	Q9NPA3	1.2057
P0C214	Q3LI83	0.51364
P0C214	Q8NCK3	0.33303
P0C214	O43261	0.2675

30401	P0C214	Q86TS7	0.086276
30402	P0C214	A6QL64	0.2654
30403	P0C214	Q9NR55	0.2181
30404	P0C214	Q14145	1.267
30405	P0C214	Q96D46	0.7903
30406	P0C214	Q6PF06	0.92931
30407	P0C214	P41236	0.29577
30408	P0C214	Q93074	0.45437
30409	P0C214	O75698	0.086276
30410	P0C214	A6NJ78	0.37656
30411	P0C214	P0CW01	0.69286
30412	P0C214	O60829	0.26319
30413	P0C214	P11055	0.85592
30414	P0C214	P78406	0.53001
30415	P0C214	O95299	0.04047
30416	P0C214	Q96BP2	1.2308
30417	P0C214	Q92560	0.17908
30418	P0C214	A6NH11	0.27027
30419	P0C214	P52435	0.50364
30420	P0C214	A4QMS7	0.15979
30421	P0C214	Q86WI3	0.60916
30422	P0C214	P05204	0.28171
30423	P0C214	Q9BSQ5	0.1795
30424	P0C214	Q5VTE6	0.076574
30425	P0C214	Q8IXQ3	0.48672
30426	P0C214	P55265	0.40908
30427	P0C214	P17568	0.077953
30428	P0C214	O95678	0.43311
30429	P0C214	O15266	0.34608
30430	P0C214	P57740	0.8195
30431	P0C214	P17275	0.82061
30432	P0C214	Q5JTZ9	0.17573
30433	P0C214	Q8N823	0.3804
30434	P0C214	Q9NVM4	0.2947
30435	P0C214	Q92750	0.56338
30436	P0C214	Q8N5L8	1.134
30437	P0C214	Q96EU6	0.3397
30438	P0C214	Q9UNY5	0.42258
30439	P0C214	Q6ZRH9	0.086276
30440	P0C214	P62166	0.27277
30441	P0C214	Q8N814	0.72928
30442	P0C214	O00189	0.37202
30443	P0C214	Q5EE01	0.53507
30444	P0C214	Q8NEP9	0.44452
30445	P0C214	Q8NA77	0.71592
30446	P0C214	Q13367	0.37437
30447	P0C214	Q9HD42	0.82012
30448	P0C214	Q9Y4P1	0.24814
30449	P0C214	Q08AN1	0.3804
30450	P0C214	Q8N7Y1	0.086276
30451	P0C214	P11802	0.66449
30452	P0C214	Q9Y6K5	0.27557
30453	P0C214	P17022	0.17565
30454	P0C214	Q7Z417	0.74845
30455	P0C214	P53370	0.56787
30456	P0C214	Q12800	0.28267
30457	P0C214	A8K8P3	0.78551
30458	P0C214	Q9HBD4	0.5105
30459	P0C214	Q9H7H0	0.70491
30460	P0C214	A6NHQ2	0.21519
30461	P0C214	P63313	0.72737
30462	P0C214	P27635	0.87273
30463	P0C214	Q69YN4	0.19759
30464	P0C214	Q9ULK2	0.74718
30465	P0C214	Q6NT04	0.32238
30466	P0C214	Q5VTH2	0.71913
30467	P0C214	Q99567	0.87474
30468	P0C214	O15056	0.24072
30469	P0C214	Q9Y305	0.71928
30470	P0C214	A6NCN8	0.24937
30471	P0C214	O75362	0.64081
30472	P0C214	O14929	0.72161
30473	P0C214	P57678	1.0501
30474	P0C214	Q3BBV2	0.51029
30475	P0C214	Q86WS4	0.086276
30476	P0C214	Q13111	1.1121

P0C214	P32519	1.2372
P0C214	Q6ZRH3	0.02249
P0C214	Q9NPJ4	1.619
P0C214	P08133	0.62935
P0C214	Q5T7N7	0.086276
P0C214	Q5VZQ5	0.34703
P0C214	P31150	0.19906
P0C214	Q9C099	0.84614
P0C214	Q9NUA8	0.24858
P0C214	P61572	0.39132
P0C214	Q5SRH9	0.64735
P0C214	Q9Y6H1	0.75974
P0C214	Q9H6B1	0.43512
P0C214	I3L0S3	0.10358
P0C214	P53618	0.71295
P0C214	O14513	0.37354
P0C214	P34932	0.4293
P0C214	Q8TC20	0.0047744
P0C214	Q9BQC3	1.0084
P0C214	O60224	0.17331
P0C214	O43242	0.37699
P0C214	Q9UPP5	0.17012
P0C214	Q13526	0.53532
P0C214	Q9Y3B2	0.98343
P0C214	Q8N9P6	0.086276
P0C214	Q9BQG0	0.61948
P0C214	Q6ZT83	0.14432
P0C214	Q53HV7	0.58539
P0C214	Q6P4D5	0.41177
P0C214	P47813	1.1223
P0C214	Q8WYP5	0.61323
P0C214	P46778	0.86977
P0C214	Q9P0M2	0.21728
P0C214	Q8N5J2	1.1916
P0C214	O94921	0.17504
P0C214	P50748	0.72842
P0C214	A8MVW5	0.49923
P0C214	P30405	0.77514
P0C214	Q99743	0.51557
P0C214	Q15233	0.60956
P0C214	P23769	0.29993
P0C214	Q9BRX2	0.31856
P0C214	P41219	0.37466
P0C214	Q5T6S3	0.25884
P0C214	Q92793	0.43346
P0C214	Q8N9E0	0.24937
P0C214	Q16587	0.23738
P0C214	P50548	0.12318
P0C214	O60888	0.27846
P0C214	Q5TH74	0.24104
P0C214	Q96FA3	0.64025
P0C214	Q9Y6N9	0.36196
P0C214	P51580	0.16583
P0C214	Q8WYR4	0.41814
P0C214	Q9BTE6	0.61173
P0C214	Q9UJX5	0.85073
P0C214	Q8NA47	0.24937
P0C214	Q7Z7K2	0.20212
P0C214	Q8TBZ9	1.151
P0C214	P61604	0.94241
P0C214	Q5BKY9	0.24937
P0C214	Q99457	0.98315
P0C214	Q9Y5J3	0.25721
P0C214	Q96HG1	0.086276
P0C214	Q504T8	0.16769
P0C214	Q8N5X7	0.15496
P0C214	Q9UBY9	1.0572
P0C214	Q969W8	0.42978
P0C214	Q15637	0.35465
P0C214	Q96NY9	0.64461
P0C214	Q9BXU2	0.10625
P0C214	Q00994	0.90054
P0C214	Q8NHV4	0.66105
P0C214	Q96KS0	0.22798
P0C214	Q86TX2	0.28311
P0C214	P35711	0.82798

P0C214	Q8IZL8	0.77311
P0C214	Q9UIF8	0.75803
P0C214	O43865	0.19587
P0C214	P19419	1.1966
P0C214	Q9HBH7	0.49377
P0C214	Q6ZTI6	0.59314
P0C214	P56693	0.55492
P0C214	Q6ZP82	0.2675
P0C214	Q92797	0.64217
P0C214	Q5VYV7	0.2675
P0C214	Q86V20	0.7813
P0C214	P67809	0.54355
P0C214	Q8N7M0	0.28482
P0C214	Q499Z4	0.36523
P0C214	Q9NNW7	0.38708
P0C214	Q6P5R6	0.46387
P0C214	P61024	0.57531
P0C214	A6NMX2	0.086411
P0C214	Q5T200	0.86928
P0C214	Q9H1L0	0.25298
P0C214	Q8IV36	0.42159
P0C214	Q8IYS8	0.076506
P0C214	Q7Z7A1	1.0741
P0C214	Q99613	0.8284
P0C214	Q9UJ98	0.43733
P0C214	P15408	0.48161
P0C214	Q6PGP7	0.69929
P0C214	Q9BUE0	0.98887
P0C214	Q16352	0.50462
P0C214	P61586	0.37861
P0C214	Q9BWN1	0.24937
P0C214	Q96GE5	0.46805
P0C214	Q9GZL7	0.68654
P0C214	Q9NVR5	0.60202
P0C214	Q6P1M3	0.74349
P0C214	Q4U2R6	0.84108
P0C214	O15446	0.24028
P0C214	Q9Y295	0.29929
P0C214	P53999	1.2011
P0C214	Q8N9N5	0.2548
P0C214	P46937	0.81079
P0C214	P31483	0.28798
P0C214	P40261	0.18224
P0C214	A1L188	0.37228
P0C214	Q86WX3	1.0691
P0C214	Q6PI26	1.1996
P0C214	Q9NX70	1.0224
P0C214	O95625	0.18766
P0C214	P79522	0.58083
P0C214	Q13033	0.53625
P0C214	P61201	0.47496
P0C214	Q9NWV8	0.55017
P0C214	Q86XU0	0.30697
P0C214	Q9BW85	0.86463
P0C214	O60684	0.25408
P0C214	A6NL46	0.65621
P0C214	P61457	1.1839
P0C214	Q05048	0.71345
P0C214	Q7Z4T9	0.69752
P0C214	P42695	0.2446
P0C214	Q9H2J4	0.57428
P0C214	Q8NFJ8	0.35715
P0C214	Q9BSH3	0.27367
P0C214	P35606	0.32691
P0C214	Q9NXF7	0.25402
P0C214	Q5JNZ5	0.63692
P0C214	Q9HBX3	0.086276
P0C214	Q9Y375	0.61004
P0C214	Q8N8G2	0.83156
P0C214	Q9BXW4	0.11332
P0C214	Q96QT6	0.84859
P0C214	Q99708	0.55872
P0C214	Q7Z5L9	0.9375
P0C214	Q9UND3	0.15833
P0C214	Q8TF20	0.37211
P0C214	Q5VYK3	0.4

P0C214	Q9UBZ9	0.40656
P0C214	P63208	0.71247
P0C214	A5PLN9	0.14824
P0C214	O43167	0.56957
P0C214	Q96GU1	0.11884
P0C214	P0CW21	0.086276
P0C214	Q9NSB2	0.47593
P0C214	Q6P2Q9	0.54168
P0C214	Q8NE22	0.64942
P0C214	P04792	0.5119
P0C214	Q6P9A1	0.41035
P0C214	Q16082	1.0784
P0C214	Q86Y37	0.13425
P0C214	Q8WU49	0.086276
P0C214	P81877	0.34419
P0C214	P84022	0.46043
P0C214	P48594	0.92397
P0C214	Q9H1A7	0.13654
P0C214	Q9NWU5	0.54771
P0C214	Q8WW35	0.2675
P0C214	Q86VW0	0.18057
P0C214	Q4G0A6	1.0377
P0C214	Q969H9	0.086276
P0C214	P14649	0.29298
P0C214	O00186	0.777
P0C214	O75437	0.4434
P0C214	Q9UMS6	0.5793
P0C214	P23246	0.75015
P0C214	Q6ZRS2	0.36071
P0C214	Q6ZYL4	0.77282
P0C214	Q12906	1.2124
P0C214	Q9BRX8	0.10261
P0C214	Q9ULP0	0.1228
P0C214	Q6PK81	0.26609
P0C214	Q9H4G8	0.44813
P0C214	Q6P1L6	0.24918
P0C214	Q9Y512	0.069639
P0C214	P48444	0.3669
P0C214	O00567	0.83219
P0C214	Q15172	0.21147
P0C214	Q9UL58	0.60167
P0C214	Q5VV17	0.17374
P0C214	Q9BVW5	0.91895
P0C214	Q9BYV8	0.55734
P0C214	Q8N9K7	0.086276
P0C214	Q8WW24	0.39532
P0C214	Q9BRQ3	0.65924
P0C214	O14602	0.43736
P0C214	P29401	0.16655
P0C214	P54725	0.58072
P0C214	Q9BYQ8	0.43378
P0C214	Q5TBC7	0.26771
P0C214	Q9BQD3	0.31888
P0C214	Q5SQS8	0.070215
P0C214	Q8IXM3	0.56202
P0C214	Q9UKN8	0.50781
P0C214	Q5T749	0.37631
P0C214	P17096	0.53242
P0C214	Q5U623	0.85059
P0C214	Q86WB0	1.1605
P0C214	Q9BWJ5	0.54133
P0C214	Q96EH5	0.60578
P0C214	Q8N6F8	0.0042177
P0C214	P29728	0.084579
P0C214	Q8IWZ8	0.26346
P0C214	Q8NFG4	0.68203
P0C214	P34897	0.22569
P0C214	Q6IQ49	0.39346
P0C214	O43156	0.40847
P0C214	P61962	0.66714
P0C214	O95125	0.29867
P0C214	Q14689	0.2927
P0C214	P61573	0.39132
P0C214	Q9NWA0	1.2889
P0C214	Q9NZI5	0.44546
P0C214	Q96DP5	0.40853

P0C214	Q5TYW1	0.24611
P0C214	Q9H1D9	0.48407
P0C214	Q9NTX7	0.64211
P0C214	Q3SY84	0.44022
P0C214	P15172	0.27688
P0C214	Q6PII3	0.060194
P0C214	Q15428	0.25165
P0C214	Q9BSU3	0.46316
P0C214	Q6PF04	0.28202
P0C214	P78345	0.85867
P0C214	Q76L83	0.47663
P0C214	Q9BYH8	0.29156
P0C214	Q86WZ6	0.26904
P0C214	Q15397	0.15439
P0C214	Q5PR19	0.23594
P0C214	P17947	0.61473
P0C214	P46527	0.34799
P0C214	Q6PII5	0.44639
P0C214	Q969T9	0.91873
P0C214	Q9GZR5	0.68356
P0C214	Q9Y244	0.42707
P0C214	Q9P2R6	0.94251
P0C214	Q9H808	0.49536
P0C214	Q9H3H9	0.90731
P0C214	Q6NZ36	0.17985
P0C214	Q9BX66	0.31092
P0C214	P56524	0.28762
P0C214	Q96PU4	0.36674
P0C214	Q16533	0.51016
P0C214	O95229	0.77729
P0C214	Q96F07	0.3845
P0C214	Q9GZV5	1.066
P0C214	Q9UK11	0.3951
P0C214	Q7Z7C8	0.45767
P0C214	Q8N1F7	0.57626
P0C214	Q6W2J9	0.36427
P0C214	Q86YE8	0.3804
P0C214	P55957	0.42812
P0C214	Q96MW1	0.24937
P0C214	Q99623	1.0844
P0C214	P23258	0.31254
P0C214	Q9NR11	0.22197
P0C214	Q96CS4	0.24682
P0C214	Q8NBR6	1.4967
P0C214	Q9NUS5	0.45967
P0C214	Q9Y6Y1	0.43561
P0C214	Q9BW61	0.60035
P0C214	Q9Y6K1	0.30976
P0C214	Q5TBB1	0.85488
P0C214	O14709	0.3804
P0C214	P17029	0.43208
P0C214	A4D1U4	0.10335
P0C214	O14907	0.37183
P0C214	Q9H501	1.1533
P0C214	O00425	0.58953
P0C214	O75884	1.0884
P0C214	Q6NT76	0.36403
P0C214	Q8IYX3	0.64779
P0C214	Q99576	0.37515
P0C214	H3BQW9	0.076415
P0C214	Q5T681	0.53221
P0C214	O75821	0.76555
P0C214	Q3LI66	0.086276
P0C214	Q9H6V9	0.14201
P0C214	O75694	0.59078
P0C214	P42704	0.90103
P0C214	P60660	0.37617
P0C214	Q14678	0.46777
P0C214	P12277	0.09917
P0C214	Q5HYW2	0.38939
P0C214	Q6ICG8	1.354
P0C214	Q5JXB2	0.68236
P0C214	P17544	0.94644
P0C214	P41218	0.54737
P0C214	Q12986	0.13244
P0C214	O43715	0.63264

P0C214	Q5T6C5	0.74718
P0C214	Q96NA2	1.0494
P0C214	P11831	0.50308
P0C214	Q9Y2Y9	0.68564
P0C214	Q96BD5	0.32391
P0C214	Q00403	0.99261
P0C214	Q15906	0.75224
P0C214	Q96AQ9	0.32933
P0C214	Q96SR6	0.3048
P0C214	Q5JUW0	0.43784
P0C214	Q8NI29	0.35888
P0C214	Q8TCI5	0.52975
P0C214	Q8NAP3	0.30406
P0C214	Q9UJM3	0.37353
P0C214	Q9HC77	0.72932
P0C214	Q9UK13	0.48849
P0C214	Q96HR3	0.77393
P0C214	Q9UPU9	0.3579
P0C214	Q96ST2	0.12533
P0C214	P61513	0.85748
P0C214	Q641Q2	0.71414
P0C214	Q9UL40	0.60161
P0C214	Q5JTW2	0.31925
P0C214	Q9H0F6	0.1637
P0C214	A8MXT2	0.048798
P0C214	Q99417	1.4991
P0C214	P78344	0.54113
P0C214	A1L3Z2	0.092734
P0C214	Q13515	0.4805
P0C214	P11172	0.49565
P0C214	Q58FG1	0.85371
P0C214	O60318	0.68612
P0C214	Q9UNN4	0.33106
P0C214	Q9H095	0.18967
P0C214	Q5H9F3	0.61791
P0C214	Q9BXM0	0.64324
P0C214	Q99990	0.52075
P0C214	Q53EL6	1.0884
P0C214	P17026	0.26221
P0C214	Q96MN9	0.5523
P0C214	Q8IUD6	0.49152
P0C214	O14530	0.30198
P0C214	Q8NCF5	0.45436
P0C214	Q6ICC9	0.18129
P0C214	Q3LI70	0.086276
P0C214	Q96SK3	0.36207
P0C214	Q6TFL3	0.26817
P0C214	Q04206	0.59287
P0C214	Q8TAB5	0.24937
P0C214	Q86VX9	0.94747
P0C214	Q9UNQ2	0.45694
P0C214	Q96AH0	0.60677
P0C214	Q9NPC6	0.67933
P0C214	Q6PK04	0.92744
P0C214	Q9NW07	0.29044
P0C214	O60516	0.20994
P0C214	O14639	0.46609
P0C214	Q9NRI7	0.086276
P0C214	Q9NVC6	0.54759
P0C214	Q8N1E6	0.35254
P0C214	Q9Y315	0.36919
P0C214	C9JFL3	0.086276
P0C214	Q7Z2W9	0.11747
P0C214	Q9BVI4	0.36603
P0C214	P29692	0.45242
P0C214	Q16520	0.8217
P0C214	O95931	0.70796
P0C214	Q9UNL4	0.24408
P0C214	Q9UHU1	0.086276
P0C214	Q5HY98	0.43572
P0C214	Q3L8U1	0.90741
P0C214	P48735	0.36921
P0C214	Q2M1K9	0.18771
P0C214	Q7Z589	0.69748
P0C214	Q63HK5	0.26189
P0C214	P0DKX0	0.44604

P0C214	Q5RGN0	0.51029
P0C214	P83436	0.29425
P0C214	Q7L2K0	0.082505
P0C214	Q9NSI2	0.24937
P0C214	Q7L2E3	0.38557
P0C214	Q53S33	0.69639
P0C214	Q8TCD5	0.14851
P0C214	O43402	0.68113
P0C214	Q6STE5	0.74523
P0C214	Q96GX9	0.44769
P0C214	Q12962	1.0451
P0C214	Q9H307	0.81129
P0C214	Q9H3L0	0.4292
P0C214	P69905	0.29612
P0C214	Q66LE6	0.38105
P0C214	Q8NCE8	0.35221
P0C214	O75123	0.27035
P0C214	O60535	0.37038
P0C214	Q96EE3	0.57683
P0C214	Q53H80	0.35354
P0C214	P83881	1.0069
P0C214	Q2M3W8	0.50227
P0C214	Q15121	0.34094
P0C214	Q9NP98	0.3766
P0C214	Q9Y3B6	0.73626
P0C214	Q15056	0.52538
P0C214	O14964	0.51922
P0C214	P63279	0.95701
P0C214	Q06455	0.59962
P0C214	P05787	1.3053
P0C214	Q9NSG2	0.28533
P0C214	Q9Y5Q3	0.7422
P0C214	Q16613	0.19615
P0C214	Q6ZTZ1	0.30254
P0C214	Q9UHK0	0.35939
P0C214	Q96G21	0.84614
P0C214	Q6XQN6	0.36146
P0C214	P60953	0.43984
P0C214	P42224	0.23761
P0C214	Q676U5	0.48338
P0C214	Q9HAT8	0.65292
P0C214	Q02386	0.2717
P0C214	O75607	0.68938
P0C214	P14136	0.42785
P0C214	O75394	0.53548
P0C214	Q15973	0.2007
P0C214	Q6ZRI6	0.18738
P0C214	O00423	0.33752
P0C214	Q12816	0.27301
P0C214	P49459	0.91374
P0C214	Q13404	1.1206
P0C214	P48556	0.54378
P0C214	Q3MJ62	0.2507
P0C214	O14656	0.28049
P0C214	Q86WD7	0.88936
P0C214	Q9HCH5	0.53703
P0C214	O75208	0.39794
P0C214	Q9H777	0.38657
P0C214	Q96MT8	0.95523
P0C214	P42568	0.84549
P0C214	P37275	0.69456
P0C214	Q68CJ9	0.67704
P0C214	Q96S19	0.025122
P0C214	Q8WVR3	0.24937
P0C214	Q9Y2S0	0.1585
P0C214	P28347	0.89544
P0C214	Q3KP31	0.46805
P0C214	Q7Z739	0.46322
P0C214	Q96A73	1.0632
P0C214	Q6NZI2	1.2071
P0C214	P10914	0.39484
P0C214	O60232	0.97261
P0C214	Q14204	0.33053
P0C214	Q96FH0	0.21105
P0C214	Q8NI08	0.80979
P0C214	Q6PJT7	0.89877

30933	P0C214	Q92734	0.091404
30934	P0C214	Q96MY1	0.62472
30935	P0C214	P78537	0.97573
30936	P0C214	P61218	0.2029
30937	P0C214	Q5HYK7	1.2812
30938	P0C214	Q70IA6	0.39765
30939	P0C214	Q5T5S1	0.60526
30940	P0C214	Q9P1A6	0.14636
30941	P0C214	Q8N8C0	0.33766
30942	P0C214	Q9NZ81	0.93949
30943	P0C214	Q8TBR4	0.41631
30944	P0C214	Q8N5Q1	0.907
30945	P0C214	Q9HAV4	1.2386
30946	P0C214	Q16666	0.52251
30947	P0C214	Q96NM4	0.242
30948	P0C214	Q9NPJ8	0.38275
30949	P0C214	Q13427	0.69288
30950	P0C214	Q8N128	0.086276
30951	P0C214	Q15928	0.50859
30952	P0C214	Q9UI32	0.46921
30953	P0C214	Q5VVS0	0.95859
30954	P0C214	P0C7W0	0.24937
30955	P0C214	Q8N448	0.75722
30956	P0C214	A6H8Z2	0.15822
30957	P0C214	Q8N5H3	0.24937
30958	P0C214	P17482	0.98819
30959	P0C214	O60299	0.30795
30960	P0C214	O43493	0.53748
30961	P0C214	Q17RC7	0.41178
30962	P0C214	Q8TDY2	0.91964
30963	P0C214	Q8IXW5	0.65864
30964	P0C214	O43745	0.6522
30965	P0C214	O95721	0.24622
30966	P0C214	Q8NHY2	1.0913
30967	P0C214	Q8WWB5	0.64222
30968	P0C214	P49792	0.91022
30969	P0C214	P35270	0.24586
30970	P0C214	Q5SXM2	0.53443
30971	P0C214	Q8IYW2	0.086276
30972	P0C214	O75381	0.24388
30973	P0C214	A8MYA2	0.067192
30974	P0C214	O75886	1.0515
30975	P0C214	Q96BN2	0.77665
30976	P0C214	Q9ULJ6	0.47098
30977	P0C214	Q9ULG1	0.24582
30978	P0C214	O95503	0.83199
30979	P0C214	Q9GZZ1	0.38322
30980	P0C214	O96018	0.35583
30981	P0C214	Q86TJ2	0.3055
30982	P0C214	Q86YV9	0.80588
30983	P0C214	Q8TDH9	0.14522
30984	P0C214	Q9UPG8	0.1622
30985	P0C214	Q96K58	0.23938
30986	P0C214	Q7L8W6	0.48256
30987	P0C214	Q9BYX4	0.34679
30988	P0C214	P54920	0.3857
30989	P0C214	Q5JVF3	0.3641
30990	P0C214	O43395	0.79068
30991	P0C214	Q05DH4	0.7199
30992	P0C214	Q9Y2Z2	0.92021
30993	P0C214	Q8NG27	0.23741
30994	P0C214	Q13618	0.4111
30995	P0C214	P57075	0.38497
30996	P0C214	Q9Y5B6	0.76615
30997	P0C214	P22087	0.63201
30998	P0C214	A0MZ66	0.6129
30999	P0C214	P23284	0.65781
31000	P0C214	O15127	0.47822
31001	P0C214	A8MT65	0.45792
31002	P0C214	Q9Y3C5	0.35592
31003	P0C214	Q6ISB3	0.6434
31004	P0C214	Q9UKI9	1.1671
31005	P0C214	Q9BUL8	0.67837
31006	P0C214	Q9UKX7	1.0112
31007	P0C214	Q08378	0.27173
31008	P0C214	Q9BWD3	0.16623

P0C214	Q9BYQ9	0.43378
P0C214	Q5T653	0.54353
P0C214	P40306	0.51422
P0C214	Q93034	0.8311
P0C214	Q3ZCQ2	0.086276
P0C214	Q9H869	1.3951
P0C214	Q49AM1	0.44086
P0C214	Q96HQ2	0.92792
P0C214	Q9BTA0	0.24937
P0C214	Q96JC4	0.35746
P0C214	Q86W56	0.78558
P0C214	P62701	0.68068
P0C214	Q9HA92	0.48254
P0C214	Q99608	0.17134
P0C214	Q9GZQ3	1.0619
P0C214	Q9BZK8	0.086276
P0C214	Q71UM5	0.86958
P0C214	P36543	0.28827
P0C214	Q6IPU0	0.24794
P0C214	P49207	0.89116
P0C214	Q06330	0.4863
P0C214	Q99873	0.66953
P0C214	P11940	0.54638
P0C214	P98168	0.65832
P0C214	P05813	0.19081
P0C214	Q9NYK6	0.37781
P0C214	Q12905	1.1009
P0C214	P35251	0.52965
P0C214	Q99497	0.18325
P0C214	Q969T4	0.79024
P0C214	Q7Z3I7	0.26874
P0C214	Q15910	0.38814
P0C214	P14635	0.2499
P0C214	Q15370	1.2248
P0C214	Q14232	0.22281
P0C214	P0CW26	0.16767
P0C214	P63220	0.76824
P0C214	Q9Y296	0.1947
P0C214	Q15257	0.49741
P0C214	Q96HZ7	0.086276
P0C214	Q99607	0.97771
P0C214	O60296	0.55482
P0C214	Q96IL0	0.17552
P0C214	A6NJJ6	0.24937
P0C214	Q0VDD7	1.0809
P0C214	Q7Z7F1	0.2436
P0C214	Q32MZ4	1.0281
P0C214	A2RU30	0.25438
P0C214	Q5JWF8	0.041938
P0C214	O00716	0.97278
P0C214	Q8NBT2	0.73405
P0C214	Q8IZ26	0.3115
P0C214	Q92529	0.32272
P0C214	Q10570	0.75988
P0C214	H3BNL1	0.19015
P0C214	Q15050	0.76213
P0C214	O75030	0.47179
P0C214	Q9NSI6	0.83245
P0C214	Q8N726	0.51863
P0C214	Q9NVF7	0.3662
P0C214	Q3BBV0	0.42949
P0C214	Q9UBS3	0.71286
P0C214	Q9Y2A0	0.20991
P0C214	B3EWG3	0.24937
P0C214	P78395	0.65692
P0C214	Q6WBX8	0.34094
P0C214	Q6ZV77	0.086276
P0C214	Q8N9F8	0.33108
P0C214	Q8N668	0.75466
P0C214	Q9Y6Q2	0.25454
P0C214	Q6ZUA9	0.21138
P0C214	Q5RKV6	0.35397
P0C214	P48200	0.56075
P0C214	Q9Y613	0.51813
P0C214	Q9NUQ6	0.35391
P0C214	Q9NRD1	0.21063

P0C214	Q9Y2M0	0.3016
P0C214	Q5SWA1	0.67395
P0C214	O43617	0.38421
P0C214	P17024	0.31548
P0C214	Q9Y232	0.21984
P0C214	Q9Y2Q1	0.47209
P0C214	Q8NC74	0.34205
P0C214	Q9BZS9	0.086276
P0C214	Q6ZUL3	0.086276
P0C214	Q6ZR52	0.38021
P0C214	Q9NXV2	1.4022
P0C214	O75340	0.43798
P0C214	P20585	0.030835
P0C214	P68402	0.099086
P0C214	P26641	0.61424
P0C214	Q8NCU7	0.65252
P0C214	P69208	0.098385
P0C214	P06396	0.40971
P0C214	Q3LI76	0.50329
P0C214	Q7L9L4	0.83762
P0C214	Q5T870	0.70223
P0C214	Q9NQ29	0.81084
P0C214	O14771	0.58933
P0C214	Q9H6Q4	0.48571
P0C214	Q96CT7	0.054683
P0C214	Q9BXQ6	0.086276
P0C214	Q96DN6	0.23587
P0C214	Q12901	0.40317
P0C214	Q92540	1.0915
P0C214	Q96NG5	0.40737
P0C214	O60813	0.36259
P0C214	Q8WVZ9	0.41816
P0C214	O14599	0.41813
P0C214	Q8N819	0.40985
P0C214	Q8NFU3	0.12809
P0C214	O60437	0.69749
P0C214	Q96N77	0.3338
P0C214	Q9NSC5	0.37717
P0C214	P20700	0.2073
P0C214	O75164	1.093
P0C214	Q9H910	0.78406
P0C214	Q86SQ9	0.10075
P0C214	P61575	0.39132
P0C214	P55198	0.80901
P0C214	P40939	0.36045
P0C214	O60477	0.43754
P0C214	P05413	0.23094
P0C214	P55345	0.40618
P0C214	Q8NCQ2	0.48343
P0C214	Q8N5J4	0.36351
P0C214	Q9NX18	0.87889
P0C214	Q9H6R4	0.39322
P0C214	P24385	0.51418
P0C214	Q6ZU52	0.2675
P0C214	P51808	0.18397
P0C214	Q86WZ0	0.159
P0C214	Q96SE7	0.37033
P0C214	O75151	0.64715
P0C214	P60411	0.43378
P0C214	O00139	0.29899
P0C214	Q9NVM9	1.0327
P0C214	Q04724	0.82698
P0C214	Q9BUR4	0.80799
P0C214	Q9UQN3	0.50704
P0C214	Q13875	0.59529
P0C214	Q14676	0.52841
P0C214	P08865	0.93163
P0C214	Q96JG6	0.018603
P0C214	Q9H091	0.17354
P0C214	Q9BTT4	0.61448
P0C214	P49005	0.49437
P0C214	Q14999	1.1197
P0C214	Q7Z614	0.29301
P0C214	Q9Y2Q9	0.54217
P0C214	Q96CW5	0.78446
P0C214	Q9H0A9	1.0231

P0C214	P05121	0.78952
P0C214	Q8WXD5	0.99045
P0C214	Q14194	0.45535
P0C214	Q9UBU7	0.026052
P0C214	Q6ZMS7	0.37171
P0C214	Q15691	0.54855
P0C214	Q9NZI8	0.26202
P0C214	P32969	1.1125
P0C214	Q5VZR2	0.72088
P0C214	Q6IPT2	0.907
P0C214	Q9NNX9	0.70144
P0C214	Q9UK41	0.69211
P0C214	Q3KRA6	0.9744
P0C214	Q13884	0.30716
P0C214	Q86WP2	0.37729
P0C214	Q5JPH6	0.42695
P0C214	Q9H4L7	0.32241
P0C214	Q5U5X8	0.52883
P0C214	Q7Z2X4	0.23431
P0C214	P35908	0.38324
P0C214	Q02543	0.97266
P0C214	Q8NEQ6	0.11647
P0C214	Q6ZN19	0.25925
P0C214	Q13200	0.60989
P0C214	Q9UL41	0.53311
P0C214	P07199	0.669
P0C214	Q9UK61	0.46209
P0C214	O43776	0.81413
P0C214	Q9Y3R4	0.19396
P0C214	Q13901	1.0659
P0C214	Q92733	0.75083
P0C214	P62266	1.1544
P0C214	Q14129	0.51831
P0C214	P17038	0.38021
P0C214	Q8NEK8	0.37656
P0C214	Q9UJX2	0.67236
P0C214	F5H4B4	0.54789
P0C214	A6NNH0	0.63857
P0C214	Q6ZMR3	0.96724
P0C214	Q9Y297	0.41858
P0C214	Q9Y468	0.81485
P0C214	Q9UC07	0.30617
P0C214	Q68E01	0.54302
P0C214	Q8NEY3	0.31433
P0C214	Q9Y3D8	0.21653
P0C214	Q2KHT3	0.23452
P0C214	Q9HAU5	1.0073
P0C214	Q9NXK8	0.56048
P0C214	Q14686	0.82256
P0C214	P30414	0.69592
P0C214	Q8IXP5	0.24937
P0C214	A8MZG2	0.24937
P0C214	O94864	0.46776
P0C214	Q9NYP3	0.37563
P0C214	Q9Y6I3	0.052121
P0C214	Q99807	0.2924
P0C214	P31948	0.88552
P0C214	Q99962	0.31397
P0C214	A6NGG3	0.086276
P0C214	O95149	0.4992
P0C214	Q8NEJ9	0.29361
P0C214	Q15545	0.84241
P0C214	Q96EK7	0.24855
P0C214	P38935	0.42249
P0C214	Q9HAW4	0.28615
P0C214	Q96HT8	1.28
P0C214	Q8N7B7	0.22927
P0C214	A8MSY0	0.23803
P0C214	Q8WV60	0.25451
P0C214	O95396	0.23505
P0C214	Q8N2H9	0.32414
P0C214	Q7L3B6	0.25373
P0C214	Q494X3	0.47643
P0C214	P0CG35	0.72737
P0C214	Q99547	0.70904
P0C214	Q14938	0.12315

P0C214	Q9BXT5	0.39984
P0C214	O75420	0.48436
P0C214	Q96MR9	0.43328
P0C214	Q9Y2P0	0.29953
P0C214	Q9H0R1	0.10687
P0C214	O60244	0.51898
P0C214	Q7RTU5	0.38179
P0C214	Q00978	0.96962
P0C214	Q86Z20	1.0729
P0C214	Q8N612	0.23781
P0C214	Q6EEV4	0.086276
P0C214	Q15170	1.3647
P0C214	Q9GZU7	0.53839
P0C214	Q9H379	0.30078
P0C214	Q8N5T8	0.18531
P0C214	Q96K75	0.28618
P0C214	Q8N6Q8	0.44451
P0C214	Q1W209	0.086276
P0C214	Q9BYR0	0.43378
P0C214	Q9BQN1	0.67458
P0C214	P40926	0.75212
P0C214	P16083	0.17757
P0C214	P09001	0.59874
P0C214	P62877	0.67169
P0C214	Q2VPB7	0.6712
P0C214	Q5MJ68	0.15857
P0C214	Q6ZQP7	0.086276
P0C214	P32189	0.34829
P0C214	Q9NPB8	0.13242
P0C214	O96006	0.61822
P0C214	O75330	0.072932
P0C214	O60551	0.2092
P0C214	Q569H4	0.24937
P0C214	Q9UII5	0.37211
P0C214	Q49B96	0.6344
P0C214	O00308	0.299
P0C214	Q6P1K2	1.1361
P0C214	Q5T4H9	0.086276
P0C214	Q15653	1.0113
P0C214	Q4V348	0.24611
P0C214	Q9NY12	0.35369
P0C214	P22557	0.29461
P0C214	P59090	0.086276
P0C214	O43463	0.72156
P0C214	O75794	1.2143
P0C214	Q9BT81	0.26441
P0C214	Q7KZF4	0.96346
P0C214	P63241	0.50103
P0C214	Q9UKG1	0.35374
P0C214	P46060	0.26181
P0C214	Q9UNS2	0.6335
P0C214	Q96SZ4	0.22695
P0C214	A6NLF2	0.31331
P0C214	Q9H063	0.4638
P0C214	Q15031	0.29999
P0C214	P62308	0.80392
P0C214	Q6IV72	0.27217
P0C214	Q562F6	0.78356
P0C214	Q96LT7	0.55173
P0C214	Q9NY61	0.77078
P0C214	Q96GX8	0.24937
P0C214	Q9BXR0	1.3921
P0C214	O14556	0.80401
P0C214	P51854	0.14233
P0C214	Q9H489	0.85642
P0C214	Q8N141	0.50227
P0C214	Q3KP22	0.089033
P0C214	Q86UY8	0.17404
P0C214	Q15436	0.6379
P0C214	Q96TC7	0.46291
P0C214	Q9NWT8	0.84438
P0C214	Q9Y4Z0	0.8311
P0C214	Q8TBE0	0.67207
P0C214	P51786	0.2833
P0C214	Q5VVH5	0.58787
P0C214	Q9BY31	0.20165

P0C214	Q3B7T1	0.39389
P0C214	Q13094	0.7069
P0C214	Q96T17	0.55877
P0C214	Q658Y4	0.39128
P0C214	Q8TAQ2	1.2801
P0C214	Q75VX8	0.74501
P0C214	Q9Y266	0.86844
P0C214	Q96Q05	0.33859
P0C214	Q69YH5	0.72707
P0C214	Q8IWE5	0.3113
P0C214	Q9UET6	0.86401
P0C214	P35573	0.71008
P0C214	Q9UK45	0.89284
P0C214	Q9C0C6	0.64982
P0C214	Q969Q0	0.095792
P0C214	F8W1W9	0.043236
P0C214	Q01844	0.64756
P0C214	Q9HCU8	0.46207
P0C214	Q8NDH6	0.39171
P0C214	Q9UI95	1.1515
P0C214	Q15560	0.96142
P0C214	Q9H2F5	0.48541
P0C214	O43318	0.47939
P0C214	Q96GI7	0.24937
P0C214	Q9UHR6	0.98757
P0C214	P14868	0.98867
P0C214	P13196	0.47245
P0C214	O43148	0.92061
P0C214	Q6ZSJ8	0.24937
P0C214	P52565	0.13808
P0C214	Q96BV0	0.16136
P0C214	P13640	0.56842
P0C214	Q7RTX8	9.3936e-005
P0C214	Q6ZRQ5	0.53551
P0C214	P29508	0.96136
P0C214	P08579	0.28881
P0C214	Q6P1K8	0.35545
P0C214	P38936	0.233
P0C214	O60287	0.96041
P0C214	A6NEJ1	0.17331
P0C214	Q6PIJ6	0.5958
P0C214	Q8NAV1	0.70342
P0C214	Q5SVQ8	0.35429
P0C214	Q6ZS94	0.086276
P0C214	Q6IA86	0.54942
P0C214	O76042	0.46927
P0C214	O43390	0.63499
P0C214	Q92541	0.88848
P0C214	P36542	0.18202
P0C214	Q13542	0.57944
P0C214	P60331	0.43378
P0C214	Q16594	0.51174
P0C214	Q8N883	0.26062
P0C214	O00232	0.36835
P0C214	Q9Y5N6	0.48445
P0C214	Q5T447	0.38666
P0C214	Q5T655	0.086276
P0C214	Q99750	0.37406
P0C214	Q8N6I1	0.64762
P0C214	Q96MT3	0.8875
P0C214	Q15366	1.0133
P0C214	Q9Y2S2	0.079191
P0C214	Q96FF9	0.76515
P0C214	O15131	1.0779
P0C214	O95232	0.42145
P0C214	O95347	0.33998
P0C214	Q8NDC0	0.13629
P0C214	Q3SXZ3	0.30182
P0C214	Q9GZX5	0.78655
P0C214	O60810	0.69314
P0C214	Q8WW22	0.97466
P0C214	P05423	0.177
P0C214	Q8IWL3	0.60647
P0C214	Q7L592	0.4323
P0C214	Q16890	0.38258
P0C214	Q9H1A3	0.26948

P0C214	P82664	1.0017
P0C214	Q9BYQ3	0.43378
P0C214	Q8N684	0.45735
P0C214	Q8NCX0	0.33472
P0C214	Q9NXT0	0.25251
P0C214	Q6P5X5	0.72991
P0C214	Q7Z6M4	0.44484
P0C214	Q9UKD1	0.73823
P0C214	Q13064	0.19885
P0C214	P78316	1.0604
P0C214	P51523	0.20866
P0C214	P49591	0.55377
P0C214	Q7Z6I5	0.76405
P0C214	Q86UE4	0.49292
P0C214	O95049	0.389
P0C214	Q9P2T0	0.29541
P0C214	Q6ZQY7	0.37777
P0C214	P82914	0.71396
P0C214	P0CV99	0.69286
P0C214	Q9H4I2	0.65925
P0C214	Q9BYD9	0.095747
P0C214	Q8IZU0	0.60323
P0C214	Q86VP3	0.59786
P0C214	Q8IUR0	0.92167
P0C214	Q5XKE5	0.42688
P0C214	Q147U1	0.3168
P0C214	Q12789	0.77767
P0C214	Q8N3F0	0.21779
P0C214	Q86XZ4	0.44203
P0C214	Q9HCE1	0.85944
P0C214	Q8WXG1	0.089621
P0C214	Q96BQ5	0.55913
P0C214	Q9BSK1	0.23356
P0C214	P51965	0.82252
P0C214	Q9H3C7	0.68258
P0C214	O60524	1.056
P0C214	P17676	0.68498
P0C214	O43639	0.45849
P0C214	Q96EN9	0.30252
P0C214	Q9BTE1	0.18177
P0C214	P24278	0.80252
P0C214	Q96T88	0.24021
P0C214	P17031	0.34363
P0C214	Q9UBS4	0.77743
P0C214	P15170	0.66928
P0C214	Q9P242	0.78573
P0C214	Q9UD71	0.89278
P0C214	Q5SWW7	0.086276
P0C214	O75792	0.97602
P0C214	Q5PSV4	0.359
P0C214	Q9BZQ2	0.54279
P0C214	Q9BPZ3	0.4756
P0C214	A8MV65	0.61461
P0C214	P43355	1.0247
P0C214	Q01105	0.92207
P0C214	Q9BXD5	0.56488
P0C214	Q9UKT7	0.45991
P0C214	P46976	0.66221
P0C214	Q9HCX3	0.23454
P0C214	Q99700	0.7552
P0C214	Q96AT1	0.24937
P0C214	Q16718	0.01924
P0C214	Q13057	0.2186
P0C214	Q16630	0.29385
P0C214	P55789	0.15201
P0C214	P78317	0.62333
P0C214	Q15542	0.93003
P0C214	Q16385	0.81218
P0C214	P62277	1.3005
P0C214	P14651	0.047469
P0C214	O94906	0.31677
P0C214	Q86UT8	0.84223
P0C214	Q92562	0.28119
P0C214	Q9H503	0.21051
P0C214	Q8N336	0.16714
P0C214	P48147	0.24366

P0C214	Q9H0M0	0.36125
P0C214	Q99615	0.83605
P0C214	A0PJX2	0.48763
P0C214	Q5I0X4	0.24937
P0C214	Q9UIV1	1.327
P0C214	Q3LI60	0.086276
P0C214	Q9Y248	0.64298
P0C214	Q03933	0.63006
P0C214	Q9Y3Z3	0.1586
P0C214	Q96RT7	0.27841
P0C214	Q9UGJ1	0.1994
P0C214	Q5VTD9	0.16919
P0C214	Q7Z2W4	0.42133
P0C214	O95619	0.70453
P0C214	O75843	0.53015
P0C214	Q13398	0.43651
P0C214	P25205	0.32901
P0C214	Q53GI3	0.29263
P0C214	Q8NF67	0.21396
P0C214	P05062	0.35674
P0C214	A8CG34	0.22124
P0C214	Q9Y3E5	0.65763
P0C214	O94874	0.85034
P0C214	Q09028	0.54881
P0C214	Q5JR59	0.99982
P0C214	P0C879	0.086276
P0C214	Q8TF46	0.26371
P0C214	Q6ZSG2	0.088984
P0C214	Q9HBT8	0.25299
P0C214	Q8NEG0	0.007562
P0C214	P55854	0.45883
P0C214	Q9BR46	0.48404
P0C214	P86452	0.39917
P0C214	Q9NWS0	0.39274
P0C214	Q8N7U7	0.30483
P0C214	Q96C11	0.28531
P0C214	Q8TDN6	0.30115
P0C214	Q93008	0.44942
P0C214	O60732	0.2675
P0C214	Q96JZ2	0.54427
P0C214	Q6PCE3	0.62358
P0C214	Q9Y4Y9	0.89957
P0C214	Q8N365	0.24937
P0C214	Q9UI30	0.35517
P0C214	Q5M9Q1	0.91393
P0C214	A8MTZ7	0.12874
P0C214	Q99909	0.17331
P0C214	O14776	1.0264
P0C214	Q01082	0.47635
P0C214	O43248	0.2349
P0C214	Q8IYI0	0.041094
P0C214	Q9BSF0	0.33507
P0C214	Q8N9N8	0.20832
P0C214	P08779	0.61981
P0C214	Q6ZVX7	0.58901
P0C214	P27816	0.3152
P0C214	Q9C0I3	0.85525
P0C214	Q86UC2	0.35413
P0C214	Q8NA31	0.62725
P0C214	Q9UK58	0.31702
P0C214	Q5VWM6	0.73225
P0C214	Q8NE18	1.2768
P0C214	P23490	0.66191
P0C214	Q9Y2G5	0.18365
P0C214	Q9NZP6	0.91744
P0C214	P20810	0.16103
P0C214	Q01534	0.68814
P0C214	Q8N983	0.54328
P0C214	Q9NWF9	0.78845
P0C214	Q5VZB9	0.068143
P0C214	Q6ZNM6	0.58553
P0C214	Q68CZ2	0.28749
P0C214	Q9UMS4	0.57638
P0C214	Q06830	0.40417
P0C214	Q6UYE1	0.80116
P0C214	P50914	1.2566

P0C214	Q9HB03	0.9999
P0C214	O95257	0.26971
P0C214	Q8IWE4	0.31901
P0C214	Q5VSL9	0.75774
P0C214	Q5TAQ9	0.25402
P0C214	P61077	1.0001
P0C214	Q9BZV1	1.0203
P0C214	O60290	0.53162
P0C214	Q96IZ7	1.0576
P0C214	Q86W50	0.48706
P0C214	Q9NUQ3	0.65808
P0C214	P49716	0.26665
P0C214	P78386	0.53378
P0C214	Q1RMZ1	0.23275
P0C214	Q9H089	0.22125
P0C214	Q9NWQ4	0.18973
P0C214	Q96DC7	1.168
P0C214	Q6ZW49	0.37607
P0C214	Q8WTV1	0.77682
P0C214	Q10587	0.20601
P0C214	Q9UPQ9	0.44757
P0C214	P32929	0.34673
P0C214	Q8NAE3	0.15056
P0C214	Q8N5U6	0.9957
P0C214	Q9BXW3	0.086276
P0C214	P62072	0.19607
P0C214	A8MVM7	0.086276
P0C214	O95571	0.69606
P0C214	Q07955	0.58056
P0C214	Q5T6F2	0.70773
P0C214	P10244	0.47271
P0C214	Q8N344	0.47392
P0C214	Q5VTA0	0.36894
P0C214	P11166	0.57008
P0C214	P49450	0.23893
P0C214	P54368	0.057821
P0C214	P53701	0.45284
P0C214	P50749	0.66582
P0C214	P18615	1.0761
P0C214	Q9H0W9	0.61831
P0C214	Q7Z5V6	0.29952
P0C214	E9PAV3	0.53901
P0C214	Q96BF6	0.31209
P0C214	Q9BQI9	0.62503
P0C214	Q9BY27	0.46777
P0C214	P17030	0.27117
P0C214	Q10586	0.30727
P0C214	Q9UH99	0.13541
P0C214	Q7L804	0.49951
P0C214	Q9H098	0.47683
P0C214	Q96QU8	0.93058
P0C214	Q9Y534	0.27362
P0C214	Q9NZC7	0.55894
P0C214	Q13794	0.57147
P0C214	O95359	0.24586
P0C214	Q6VUC0	0.33775
P0C214	Q9BT92	0.51152
P0C214	Q9BWG6	0.13248
P0C214	Q13428	0.86117
P0C214	Q9Y250	0.84989
P0C214	Q6QNY0	0.70421
P0C214	A7MCY6	0.58714
P0C214	E9PB15	0.046414
P0C214	O75564	0.35016
P0C214	Q03936	0.46545
P0C214	Q5QJE6	0.8248
P0C214	O14561	0.37539
P0C214	Q8TBF2	0.31124
P0C214	Q6ZNK6	0.77451
P0C214	Q96CN9	0.43634
P0C214	Q96NL3	0.32443
P0C214	Q99798	0.16285
P0C214	P0C1Z6	1.048
P0C214	O60828	0.75495
P0C214	Q8TF68	0.095757
P0C214	Q701N2	0.086276

P0C214	Q9Y5P3	0.37134
P0C214	O14974	0.28369
P0C214	Q9H944	1.0186
P0C214	P60510	0.79389
P0C214	Q9UKJ3	0.70309
P0C214	Q9BYK8	0.47916
P0C214	Q5T2W1	0.29107
P0C214	Q9NR46	0.38819
P0C214	Q96CS2	0.36456
P0C214	Q14315	0.30044
P0C214	Q9NZI7	0.19846
P0C214	P80297	0.39508
P0C214	P57086	0.44629
P0C214	P22061	0.48968
P0C214	Q5SQN1	0.28546
P0C214	Q9Y5K5	0.44134
P0C214	Q2VWA4	0.08379
P0C214	Q9BSV6	0.34808
P0C214	Q9UK39	0.38387
P0C214	Q9BTE3	0.6417
P0C214	O75800	0.33839
P0C214	P48382	0.80578
P0C214	P0CB43	0.012852
P0C214	Q9H4R4	0.049355
P0C214	Q9BRU9	0.45151
P0C214	Q9HBZ2	0.26766
P0C214	P20265	0.56656
P0C214	Q96LM1	0.32623
P0C214	Q9P2R7	0.53453
P0C214	B3EWF7	0.086276
P0C214	Q13216	0.43088
P0C214	Q14980	1.0131
P0C214	Q7RTT4	0.17331
P0C214	Q5VWG9	0.7059
P0C214	Q2NL98	0.67135
P0C214	Q53QV2	0.37961
P0C214	Q52M58	0.27067
P0C214	Q02078	0.30804
P0C214	Q9ULL0	0.21184
P0C214	Q8WUN7	0.35391
P0C214	Q8TEP8	0.7999
P0C214	O15481	0.011836
P0C214	O75223	0.079485
P0C214	Q96FA7	0.69995
P0C214	O75830	0.96136
P0C214	Q9BW66	0.79069
P0C214	Q86XN6	0.37855
P0C214	Q658L1	0.51235
P0C214	Q9UBX3	0.38415
P0C214	Q53FT3	0.29392
P0C214	Q86Y26	0.49983
P0C214	O14503	0.78183
P0C214	P11532	0.44053
P0C214	O95059	0.28385
P0C214	Q9UPV0	1.521
P0C214	Q9HCE7	0.23016
P0C214	Q9C0F1	0.13056
P0C214	A4D997	0.16667
P0C214	Q8NHU0	0.19717
P0C214	Q92665	0.76286
P0C214	Q9H2H8	0.7118
P0C214	Q13501	0.43183
P0C214	O76031	0.5296
P0C214	Q8IZH2	0.32895
P0C214	P49366	0.52586
P0C214	A6NES4	0.0089829
P0C214	Q7RTT5	0.17331
P0C214	Q15561	0.80827
P0C214	Q9H8Q6	0.56305
P0C214	Q969F1	0.62253
P0C214	O15519	0.23423
P0C214	P82094	1.1667
P0C214	Q9BZE9	0.65022
P0C214	Q9BT78	0.999
P0C214	Q86V97	0.41816
P0C214	P46779	1.371

P0C214	Q9H0B6	0.5921
P0C214	Q07352	0.95883
P0C214	P62714	0.75714
P0C214	O95989	0.44549
P0C214	Q5TAB7	0.62088
P0C214	O60921	0.72112
P0C214	Q8N5C8	0.51528
P0C214	Q8TDN4	0.8069
P0C214	O60825	0.35115
P0C214	P55795	0.16029
P0C214	Q9NYP7	0.17897
P0C214	Q8N3C0	0.38152
P0C214	Q8N2Z9	0.48239
P0C214	A6NEQ9	0.086276
P0C214	Q8TAD7	0.24937
P0C214	P10746	0.28818
P0C214	Q16548	0.45556
P0C214	P08397	0.23293
P0C214	O95201	0.56957
P0C214	Q6PGN9	0.76428
P0C214	Q9BY42	0.41397
P0C214	Q9P0R6	0.28951
P0C214	Q8TC21	0.26163
P0C214	Q969E4	0.39389
P0C214	Q96D70	0.53651
P0C214	A6NMK8	0.23055
P0C214	Q9NX05	0.37248
P0C214	Q9UJ68	0.16036
P0C214	P23786	0.17719
P0C214	Q6P3W6	0.50532
P0C214	O95707	0.94567
P0C214	Q8N1A0	0.47439
P0C214	P28698	0.64714
P0C214	O75525	0.8226
P0C214	Q00059	0.88237
P0C214	Q15633	0.94028
P0C214	P78352	0.49376
P0C214	Q8IXW0	0.43438
P0C214	Q9NWS9	0.054703
P0C214	Q96ST8	0.88046
P0C214	P15121	0.59032
P0C214	Q70EK8	0.5502
P0C214	Q14258	1.1824
P0C214	Q9GZS1	0.32027
P0C214	Q9Y3C7	0.86837
P0C214	Q9H0N5	1.0214
P0C214	P50747	0.18494
P0C214	Q6PJP8	0.58883
P0C214	Q14197	0.082977
P0C214	Q9BY43	0.38623
P0C214	Q9UJ04	0.65767
P0C214	Q03188	0.26574
P0C214	Q92922	0.99249
P0C214	Q9ULE4	0.40212
P0C214	Q8IXF0	0.2763
P0C214	Q9BWU0	0.5206
P0C214	Q7L4I2	1.5753
P0C214	Q9H3H1	0.44088
P0C214	Q8NFR7	0.26461
P0C214	Q6ZW13	0.086276
P0C214	O43303	0.85151
P0C214	Q9H299	0.15826
P0C214	P15622	0.32443
P0C214	Q86UD0	0.43557
P0C214	Q6ECI4	0.36207
P0C214	Q5VUG0	0.28639
P0C214	Q9BZ01	0.25786
P0C214	A6NEF3	0.49852
P0C214	Q15102	0.31496
P0C214	Q8IW35	1.0727
P0C214	O95397	0.7
P0C214	Q9UM07	0.58567
P0C214	Q9Y333	0.79467
P0C214	O43299	1.0911
P0C214	A6NNA2	0.60967
P0C214	Q9BXH1	0.40266

P0C214	O75312	0.42947
P0C214	Q6ZSS3	0.21588
P0C214	Q9NSA3	0.74772
P0C214	Q9BRR0	0.24903
P0C214	Q14696	0.25402
P0C214	Q5TG08	0.54789
P0C214	Q9H7P6	0.58195
P0C214	Q59GN2	0.33359
P0C214	Q9HB19	0.42017
P0C214	Q6ZN08	0.44278
P0C214	Q8N859	0.50853
P0C214	Q9NWY4	0.18463
P0C214	Q8IV32	0.10061
P0C214	P63010	0.43366
P0C214	Q99460	0.36592
P0C214	D6RB28	0.48478
P0C214	Q9H1K1	1.0355
P0C214	A6NI86	0.64941
P0C214	Q13129	0.68518
P0C214	O95298	0.31685
P0C214	O15049	0.85067
P0C214	Q96NJ1	0.086276
P0C214	P52298	0.66769
P0C214	Q9UK76	0.78406
P0C214	Q8N8U2	0.44816
P0C214	Q13642	0.46105
P0C214	Q9GZT8	1.0197
P0C214	P13056	0.4401
P0C214	Q9Y3X0	0.39895
P0C214	P48668	0.25397
P0C214	Q9UIS9	0.42099
P0C214	Q9H9A7	0.54312
P0C214	Q10567	0.5889
P0C214	P52272	0.30317
P0C214	Q76KD6	0.28434
P0C214	P28066	0.75435
P0C214	A6NFQ2	0.10906
P0C214	Q9Y421	0.3032
P0C214	Q99966	0.22334
P0C214	Q9HC78	0.11201
P0C214	Q969P5	0.10974
P0C214	Q96N23	0.25246
P0C214	Q9NZJ4	0.15507
P0C214	A6NKX1	0.086276
P0C214	Q9NPG2	0.23272
P0C214	Q9Y237	0.40702
P0C214	Q8TE85	0.22002
P0C214	Q9UQB3	0.48416
P0C214	P28715	0.24773
P0C214	O00303	0.40309
P0C214	Q96MB7	0.24623
P0C214	P10243	0.45378
P0C214	Q12968	0.7288
P0C214	Q01094	0.62832
P0C214	O75832	0.5213
P0C214	Q14814	0.48112
P0C214	Q9H467	1.0022
P0C214	Q96GX2	0.5369
P0C214	Q8WUE5	0.12778
P0C214	H3BN30	0.086276
P0C214	Q9ULQ0	0.89614
P0C214	O75446	1.0452
P0C214	O43516	0.16196
P0C214	Q7LBR1	0.80255
P0C214	Q99614	0.25893
P0C214	Q8NCD3	0.44894
P0C214	P0DJG4	0.53898
P0C214	Q86VE0	0.81683
P0C214	Q13137	1.0386
P0C214	Q9Y2P8	0.52746
P0C214	Q9H7D7	0.35391
P0C214	Q9Y6Z5	0.53008
P0C214	Q9Y2N7	0.62955
P0C214	Q5T9G4	0.86255
P0C214	P45877	0.77514
P0C214	Q05BU3	0.086276

P0C214	Q9UHY8	0.25255
P0C214	Q9HC16	0.24104
P0C214	O75969	0.44509
P0C214	Q8N3U1	0.086276
P0C214	Q9UHQ1	0.76741
P0C214	P11168	0.095236
P0C214	B1APH4	0.38062
P0C214	Q9UL36	0.27688
P0C214	P18848	0.95416
P0C214	P00374	0.13432
P0C214	Q6P9F0	0.26011
P0C214	Q92966	0.4412
P0C214	P62253	0.80576
P0C214	O95685	0.14802
P0C214	P62487	0.40566
P0C214	O75122	0.2187
P0C214	O43482	0.52726
P0C214	O00273	0.33598
P0C214	Q9ULJ3	0.55998
P0C214	O14925	0.18528
P0C214	Q92681	0.34102
P0C214	P13929	0.37846
P0C214	Q96RN5	1.2391
P0C214	Q16633	0.50381
P0C214	Q86WH2	0.097721
P0C214	P78358	0.45292
P0C214	Q8N6G1	0.086276
P0C214	Q9NRX3	0.64599
P0C214	Q9UHV9	0.89845
P0C214	Q6P435	0.33737
P0C214	Q8N6K4	0.086276
P0C214	Q86WV5	0.50542
P0C214	P18545	0.27444
P0C214	P40429	0.96028
P0C214	Q96HW7	0.54729
P0C214	Q9P0J6	0.55115
P0C214	Q9ULM6	1.2067
P0C214	Q9Y3C6	0.7118
P0C214	Q99575	0.70386
P0C214	Q03393	0.23815
P0C214	O14867	0.35877
P0C214	Q14566	0.1817
P0C214	Q674X7	0.31558
P0C214	Q5VSR9	0.46567
P0C214	Q8WVK2	0.39398
P0C214	P17535	0.8271
P0C214	Q92597	0.5098
P0C214	A6NIX2	0.94363
P0C214	Q9UI17	0.11908
P0C214	Q13506	0.36859
P0C214	P62314	0.95805
P0C214	P41091	0.20726
P0C214	Q99732	0.19016
P0C214	Q96DX5	1.1421
P0C214	Q3KNV8	0.85785
P0C214	Q8TF63	0.4422
P0C214	Q53GL7	0.53245
P0C214	A2A288	0.23636
P0C214	P98170	0.32805
P0C214	Q8N655	0.086276
P0C214	O43734	0.24937
P0C214	P30793	0.27452
P0C214	A8MWS5	0.027571
P0C214	O00479	0.38875
P0C214	Q7Z7J5	0.50721
P0C214	Q96KW2	0.61951
P0C214	Q86US8	0.6676
P0C214	Q96G03	0.62745
P0C214	Q6ICG7	0.086276
P0C214	Q86VS8	0.26595
P0C214	P07910	0.29319
P0C214	Q71F78	0.17488
P0C214	Q14493	0.91816
P0C214	Q8IYB3	0.44767
P0C214	Q9NX53	0.2675
P0C214	Q9Y6R6	0.3804

P0C214	Q49AA0	0.46445
P0C214	Q8N3S3	0.27569
P0C214	Q92754	0.45241
P0C214	Q92572	0.28657
P0C214	Q92698	0.21281
P0C214	Q9NRY5	0.7859
P0C214	Q15834	1.0154
P0C214	Q969G3	0.95813
P0C214	O60870	0.79865
P0C214	Q92674	0.42941
P0C214	P47914	1.1278
P0C214	Q9H1B4	0.33488
P0C214	Q8TAT6	0.5626
P0C214	O43439	0.82635
P0C214	Q8TF40	0.83638
P0C214	Q9UMS0	1.1751
P0C214	Q15819	0.65313
P0C214	P53675	0.23642
P0C214	P37837	0.58253
P0C214	Q00534	0.488
P0C214	Q8TCC3	0.088153
P0C214	O43809	0.69184
P0C214	Q8N8I6	0.086276
P0C214	O00712	0.16344
P0C214	Q9NPB6	0.64293
P0C214	P09467	0.53181
P0C214	Q8N9R0	0.086276
P0C214	O75817	0.85867
P0C214	Q8NE71	0.36056
P0C214	Q15528	1.1754
P0C214	Q9HB96	0.75471
P0C214	Q8WY36	0.242
P0C214	Q13045	0.98457
P0C214	Q9UHX1	0.6153
P0C214	Q14533	0.64234
P0C214	P0CG34	0.72737
P0C214	Q9NTM9	0.21776
P0C214	Q5BKX5	0.24937
P0C214	Q9NX04	0.12396
P0C214	Q13233	0.44297
P0C214	Q9Y2T7	0.13545
P0C214	Q92844	0.5426
P0C214	P49790	1.0748
P0C214	P35580	0.79029
P0C214	O75635	0.92397
P0C214	B3EWG4	0.24937
P0C214	Q8IXR9	0.014912
P0C214	Q5MJ10	0.46567
P0C214	Q13011	0.11336
P0C214	Q2TAM9	0.72088
P0C214	Q96EY5	0.36006
P0C214	O43681	0.20652
P0C214	Q7Z340	0.2834
P0C214	Q96Q89	0.35453
P0C214	Q12798	0.54982
P0C214	Q9BUP3	0.27801
P0C214	Q9UKA2	0.46131
P0C214	Q6ZNG1	0.38935
P0C214	A6NF01	0.91744
P0C214	Q9BYQ4	0.43378
P0C214	Q8IVI9	0.24275
P0C214	A6NNW6	0.25592
P0C214	P18583	0.32536
P0C214	Q96AT9	0.10981
P0C214	Q8IXJ9	0.65515
P0C214	Q15696	0.22266
P0C214	Q14186	1.1233
P0C214	Q9UII2	0.3722
P0C214	Q96EZ4	0.086276
P0C214	Q9H8Y8	0.33912
P0C214	P63173	0.74586
P0C214	P50213	0.36996
P0C214	P56211	0.31513
P0C214	Q9UIF9	0.62049
P0C214	Q9Y3V2	0.79434
P0C214	Q8IYH5	0.34015

P0C214	P13639	0.26858
P0C214	Q12837	0.40216
P0C214	P55895	0.64784
P0C214	Q6PI98	0.59548
P0C214	P49116	0.43538
P0C214	Q01201	1.0054
P0C214	A6NEQ2	0.086276
P0C214	P21127	0.13913
P0C214	Q9BRR8	0.95536
P0C214	Q6R327	0.13569
P0C214	A4D1E9	1.0277
P0C214	Q6NVV0	0.21808
P0C214	Q16881	0.31814
P0C214	Q9UHR5	1.1504
P0C214	Q92985	0.56445
P0C214	O14980	1.0715
P0C214	Q07687	0.27929
P0C214	Q8NF91	0.41576
P0C214	Q9H1A4	0.79143
P0C214	Q9H6Z9	0.60969
P0C214	P47928	0.38525
P0C214	Q8N831	0.99971
P0C214	Q6PGQ1	0.28671
P0C214	P37198	0.8857
P0C214	Q9H5P4	0.35415
P0C214	Q9HAF1	0.97362
P0C214	Q99622	0.03893
P0C214	Q9NPD3	0.49059
P0C214	P22033	0.43563
P0C214	Q00536	0.19271
P0C214	P61960	0.1156
P0C214	P49750	1.0633
P0C214	Q8WW18	0.24937
P0C214	Q9ULC6	0.78554
P0C214	P36871	0.40644
P0C214	Q3LI58	0.086276
P0C214	Q9BQ70	1.2921
P0C214	Q9P1G2	0.13455
P0C214	A0JLT2	0.87283
P0C214	Q8IZQ1	0.5198
P0C214	Q9BWT1	0.63845
P0C214	Q53ET0	1.6697
P0C214	A6NJ88	0.73332
P0C214	P41134	0.23252
P0C214	Q9UKL3	1.1465
P0C214	Q8TF74	0.33545
P0C214	Q8IYB0	0.086276
P0C214	Q96IT1	0.56958
P0C214	P08621	0.39732
P0C214	Q3MHD2	0.57472
P0C214	Q9Y224	1.4745
P0C214	O75143	1.0375
P0C214	P10124	0.11785
P0C214	Q6ZR85	0.086276
P0C214	H0YKK7	0.48478
P0C214	Q13487	1.2391
P0C214	Q96PS1	0.29049
P0C214	Q2T9F4	0.072545
P0C214	O60765	0.32997
P0C214	Q15124	0.033126
P0C214	Q13426	0.35313
P0C214	P68871	0.26239
P0C214	Q13084	1.0442
P0C214	Q9BW27	0.77719
P0C214	Q15173	0.38209
P0C214	P15923	0.47982
P0C214	Q06730	0.19269
P0C214	Q9UJU3	0.22811
P0C214	Q86UQ8	0.39389
P0C214	Q5T319	0.086276
P0C214	Q8N184	0.22977
P0C214	P56192	0.37873
P0C214	Q71F56	0.38743
P0C214	Q9Y6H5	0.76013
P0C214	P46783	1.5586
P0C214	Q9UGU0	0.23348

P0C214	Q8N8D1	0.43609
P0C214	Q86Y25	0.31387
P0C214	Q13425	0.29742
P0C214	Q9HBL8	0.099501
P0C214	Q03252	0.23127
P0C214	Q6ZTY9	0.070546
P0C214	Q6NS38	0.50418
P0C214	P52294	1.0413
P0C214	Q16831	0.014036
P0C214	B4DXR9	0.5084
P0C214	Q96QU4	0.39389
P0C214	P14618	0.45048
P0C214	O14613	0.1375
P0C214	Q04726	0.94979
P0C214	Q3LI72	0.086276
P0C214	P57060	0.72659
P0C214	Q9UBF2	0.22879
P0C214	Q8IWS0	0.47654
P0C214	P51948	0.38741
P0C214	P25791	0.31334
P0C214	Q8WXF1	1.0169
P0C214	O75132	0.17062
P0C214	Q6ZVF9	0.31356
P0C214	P69892	0.3641
P0C214	Q9Y6Y0	0.41558
P0C214	Q5JY77	0.45292
P0C214	Q9Y697	0.90092
P0C214	Q6P6B1	0.24937
P0C214	O95416	0.28328
P0C214	O95487	0.66828
P0C214	Q8N9Z2	0.10061
P0C214	P99999	0.83707
P0C214	Q8N319	1.1361
P0C214	Q5VU43	0.83199
P0C214	Q9H4B6	0.65874
P0C214	Q53G59	0.41283
P0C214	P12074	0.10088
P0C214	Q9NXB0	0.71119
P0C214	P51398	1.1597
P0C214	Q14695	0.53664
P0C214	Q96MH2	1.1907
P0C214	Q96PV7	0.33967
P0C214	O75373	0.3186
P0C214	Q2M3C7	0.43199
P0C214	Q5HYR2	0.48846
P0C214	Q8TDB4	0.45177
P0C214	Q8IYU4	0.01136
P0C214	Q8WWC4	0.6804
P0C214	Q8WTZ3	0.51532
P0C214	Q8NEF9	0.65691
P0C214	P61086	0.81164
P0C214	Q9NZD4	0.31661
P0C214	Q14103	0.98536
P0C214	Q9NVV9	0.8539
P0C214	P28370	0.4318
P0C214	P62310	0.8299
P0C214	Q1A5X6	0.24937
P0C214	Q8N1Q8	0.54764
P0C214	Q9UER7	0.93564
P0C214	O94776	0.82888
P0C214	Q04695	0.5582
P0C214	O96017	0.51576
P0C214	Q68DL7	0.086276
P0C214	Q12980	0.46024
P0C214	O14531	0.27125
P0C214	Q14978	0.76981
P0C214	Q96BD8	0.54859
P0C214	Q96DM1	0.54955
P0C214	Q92905	0.67954
P0C214	Q6TGC4	0.16665
P0C214	Q9BYR3	0.43378
P0C214	Q66GS9	0.35321
P0C214	Q14687	0.2675
P0C214	Q9UHT4	0.086276
P0C214	Q9Y221	0.78884
P0C214	Q6ICB0	0.5964

P0C214	Q96FV2	0.63341
P0C214	Q14749	0.69988
P0C214	Q92539	0.77275
P0C214	O43542	0.57658
P0C214	Q96AY2	0.71699
P0C214	Q7Z5W3	0.69175
P0C214	Q96KC9	0.5266
P0C214	Q2VPK5	0.69192
P0C214	Q9Y2H8	0.308
P0C214	Q15929	0.47837
P0C214	P49257	0.20014
P0C214	F5H284	0.77514
P0C214	P0CW24	0.1016
P0C214	A1L168	0.24937
P0C214	Q8IXT1	0.13103
P0C214	P43897	1.0755
P0C214	Q9UL42	0.77759
P0C214	Q08AM6	0.21101
P0C214	Q9NQR4	0.35124
P0C214	Q07666	0.39649
P0C214	Q8N4W9	0.3804
P0C214	Q9BQ48	0.69281
P0C214	A8MXY4	0.38021
P0C214	O60756	0.086276
P0C214	Q8TDR0	0.51273
P0C214	Q14151	0.71493
P0C214	Q96ME1	0.14478
P0C214	A1L3X0	0.9999
P0C214	Q9H8T0	0.34683
P0C214	P49720	0.57213
P0C214	P63165	0.83585
P0C214	Q9UJY4	0.36629
P0C214	Q9NZD8	0.8246
P0C214	P82663	0.95947
P0C214	Q86SX3	1.2405
P0C214	Q6UXP7	0.19654
P0C214	O95071	0.35618
P0C214	P49336	0.31139
P0C214	Q96PE6	0.22476
P0C214	P28074	0.67951
P0C214	Q3ZCX4	0.32628
P0C214	Q96M63	0.16801
P0C214	O43837	0.19359
P0C214	Q9H6U6	0.74897
P0C214	O00165	0.74454
P0C214	Q68DI1	0.18003
P0C214	Q9P0W2	0.58109
P0C214	Q8N3L3	0.63311
P0C214	O60225	0.17331
P0C214	Q7Z6E9	0.38765
P0C214	Q9HBM1	0.71266
P0C214	Q8IYX8	0.1813
P0C214	P59797	0.77906
P0C214	O95865	0.46062
P0C214	P21953	0.26948
P0C214	Q8N488	0.36179
P0C214	Q9HB07	0.28565
P0C214	Q9H5H4	0.30495
P0C214	Q6ZR03	0.040838
P0C214	Q02556	0.42879
P0C214	P34896	0.26602
P0C214	Q9H9H5	0.3253
P0C214	Q06587	0.79989
P0C214	P17861	0.51955
P0C214	Q8N9T2	0.086276
P0C214	Q8N9R8	0.51525
P0C214	Q00577	0.51347
P0C214	Q9H0A0	0.37176
P0C214	P26639	0.65617
P0C214	Q9Y2Z0	0.50268
P0C214	Q6P280	0.49708
P0C214	Q9HCC9	0.44211
P0C214	Q5VUA4	0.4662
P0C214	Q16600	0.31013
P0C214	P17014	0.26248
P0C214	P56277	0.53705

P0C214	Q9Y2X8	1.0001
P0C214	Q9NR50	0.64197
P0C214	Q14244	0.68304
P0C214	Q8WYR1	0.35514
P0C214	Q6RFH8	0.68558
P0C214	O15453	0.54417
P0C214	O15126	0.25478
P0C214	Q9Y4B6	0.90852
P0C214	Q9NTG7	0.51724
P0C214	Q9NZF1	0.083641
P0C214	C9J3I9	0.292
P0C214	Q8WXQ3	0.83978
P0C214	O95983	1.1043
P0C214	Q9BTL4	0.15301
P0C214	Q8N9K5	0.32792
P0C214	P55769	0.84992
P0C214	Q7Z2V1	0.086276
P0C214	Q99933	0.87286
P0C214	P82921	0.57295
P0C214	Q6RUI8	0.09521
P0C214	Q99459	1.0939
P0C214	Q53GS7	0.78409
P0C214	Q8NF99	0.27602
P0C214	P62280	0.80437
P0C214	Q9UHD4	0.73159
P0C214	Q14137	0.78404
P0C214	Q9P0J0	0.43829
P0C214	Q9BPU9	0.15674
P0C214	Q9UJW7	0.27328
P0C214	Q96F83	0.58353
P0C214	P0CG42	0.086276
P0C214	Q8TBZ5	0.31075
P0C214	Q9HBM6	0.57117
P0C214	P61326	1.0616
P0C214	Q9UNN5	0.65828
P0C214	Q5XG85	0.086276
P0C214	Q49A33	0.45025
P0C214	P09104	0.068334
P0C214	O14948	0.30761
P0C214	A5YKK6	1.1794
P0C214	Q9Y3M2	0.24311
P0C214	Q99549	0.64513
P0C214	Q9P2G4	0.24937
P0C214	O15162	0.27629
P0C214	Q8N2I2	0.32928
P0C214	Q14139	0.65756
P0C214	Q9HCH0	0.96081
P0C214	O14604	0.75331
P0C214	O75290	0.44452
P0C214	P49918	0.59486
P0C214	P49356	0.047482
P0C214	Q5HYC2	0.069564
P0C214	Q16851	0.57773
P0C214	Q9BV19	0.019474
P0C214	Q8TEQ6	0.87388
P0C214	Q9BZE2	1.2832
P0C214	Q9UNX3	1.2318
P0C214	Q86U70	0.17927
P0C214	Q96S59	0.99641
P0C214	Q8WXW3	0.73126
P0C214	Q8NAQ8	1.1012
P0C214	Q92979	0.38504
P0C214	Q9Y543	0.25442
P0C214	O14832	0.13158
P0C214	P52788	0.086363
P0C214	P18887	0.57762
P0C214	P13535	0.75898
P0C214	Q8IWZ3	0.97786
P0C214	Q96JF6	0.26157
P0C214	Q9BRS8	0.53148
P0C214	Q9BRG1	1.132
P0C214	Q2KJ03	0.20944
P0C214	Q9BZQ8	0.59087
P0C214	Q96D98	0.63965
P0C214	Q13356	0.65916
P0C214	Q9GZW5	0.52081

P0C214	P0CG48	0.39957
P0C214	Q96DE9	0.022936
P0C214	P53677	0.086155
P0C214	P46100	0.52614
P0C214	Q86TC9	0.36735
P0C214	Q9UL46	0.5756
P0C214	Q9NWW0	0.12649
P0C214	Q9Y324	0.87996
P0C214	P38919	0.3714
P0C214	Q96RJ6	0.29396
P0C214	Q9NX08	0.2675
P0C214	O14641	0.68583
P0C214	Q9UJT2	0.3824
P0C214	Q63HM1	0.45642
P0C214	O75570	0.27476
P0C214	Q9BUX1	0.81568
P0C214	Q9UKK6	0.59849
P0C214	Q9UHA3	0.24199
P0C214	A6NDZ8	0.086276
P0C214	P61576	0.52497
P0C214	Q08E93	0.086276
P0C214	Q9BQK8	0.086627
P0C214	O75901	0.59185
P0C214	P0C7T5	0.40717
P0C214	Q8IVM7	0.086276
P0C214	Q9NWM0	0.26168
P0C214	Q6P9A3	0.2834
P0C214	Q6AW86	0.19987
P0C214	O43513	1.1329
P0C214	Q9Y3U8	1.0997
P0C214	Q9UHC1	0.39429
P0C214	Q9H321	0.57136
P0C214	Q9P258	0.60363
P0C214	Q86XR8	0.94766
P0C214	Q9BQ52	0.2598
P0C214	P60409	0.43378
P0C214	P50402	0.19811
P0C214	Q9UNA4	0.25785
P0C214	Q9NSK7	0.73715
P0C214	Q86VP1	0.46648
P0C214	Q3LI62	0.086276
P0C214	Q9BYN8	0.78465
P0C214	O15488	0.24938
P0C214	Q9UKL0	1.1364
P0C214	Q6P050	0.61871
P0C214	Q96L92	0.74625
P0C214	Q5VZE5	0.24511
P0C214	Q8TDR4	0.51912
P0C214	P56377	0.31911
P0C214	Q6P587	0.19481
P0C214	Q9Y2H0	0.13406
P0C214	P26358	0.31533
P0C214	Q8NHS4	0.024185
P0C214	O43812	0.16797
P0C214	Q6ZS46	0.45901
P0C214	P78396	0.89181
P0C214	Q9NYP9	0.33435
P0C214	Q8TCZ7	0.28363
P0C214	Q6ZMW2	0.26428
P0C214	Q15386	0.4942
P0C214	Q9UNY4	0.4918
P0C214	P25440	0.46691
P0C214	Q5ZEY9	0.57641
P0C214	Q15398	0.93536
P0C214	Q8TF47	0.47199
P0C214	P51826	0.33701
P0C214	Q96KF2	0.086276
P0C214	Q6MZT1	0.3594
P0C214	O95336	0.45925
P0C214	Q6NSI3	0.57744
P0C214	Q92994	0.30174
P0C214	Q9H074	0.55734
P0C214	Q52MB2	0.97215
P0C214	Q6P1L8	0.80978
P0C214	Q9Y4R8	0.25003
P0C214	Q6DD87	0.25423

P0C214	Q9BVL4	1.2159
P0C214	P04732	0.37691
P0C214	Q9NZH5	0.42405
P0C214	Q96JM7	0.36513
P0C214	O43189	0.3567
P0C214	Q12982	0.32043
P0C214	Q6P1J9	0.54427
P0C214	Q6ZNE5	0.35932
P0C214	Q86TU6	0.58851
P0C214	P30050	0.97266
P0C214	Q8TCY9	0.15908
P0C214	O95376	0.46582
P0C214	Q08J23	0.6249
P0C214	Q9NRX1	0.1273
P0C214	Q9BT25	0.36456
P0C214	Q15437	0.24217
P0C214	Q5VY09	0.41881
P0C214	P14653	0.23444
P0C214	P59990	0.43378
P0C214	Q9Y6B2	0.86293
P0C214	P51504	0.24105
P0C214	Q6MZP7	0.46638
P0C214	O00422	1.2352
P0C214	Q7Z2E3	0.43113
P0C214	Q96N95	0.2319
P0C214	Q659C4	0.71354
P0C214	Q8WWQ0	0.60209
P0C214	Q9NRG9	0.94911
P0C214	O95999	0.19354
P0C214	P78385	0.51338
P0C214	E7EW31	0.086276
P0C214	Q8TF39	0.50227
P0C214	Q9BTP6	0.61393
P0C214	A6NLC8	1.1422
P0C214	O43615	0.24278
P0C214	Q68D10	0.24937
P0C214	Q86XE5	0.62026
P0C214	Q12933	0.55435
P0C214	P30281	0.63134
P0C214	Q9NVW2	0.36077
P0C214	Q9NVE4	0.009953
P0C214	P78347	0.51798
P0C214	Q9XRX5	0.5881
P0C214	Q8IZ40	0.4761
P0C214	O75145	0.36608
P0C214	Q9NZM4	0.89875
P0C214	P24539	0.28573
P0C214	O43791	0.55583
P0C214	Q9BX40	0.6527
P0C214	Q96MF7	0.45874
P0C214	P46939	0.51957
P0C214	Q6NSW5	0.97462
P0C214	Q86YP4	0.75439
P0C214	Q96SW2	0.51678
P0C214	Q6P2D0	0.24987
P0C214	P57081	0.84065
P0C214	Q13485	0.55872
P0C214	A8MUU9	0.19558
P0C214	Q9H6W3	0.49376
P0C214	P62753	1.143
P0C214	A8MV23	0.88945
P0C214	P62910	0.88548
P0C214	Q9Y2M5	0.8459
P0C214	Q9Y4P8	0.88383
P0C214	Q6UXR8	0.086276
P0C214	Q96LJ8	0.28315
P0C214	Q8ND04	0.74653
P0C214	Q13144	0.64546
P0C214	Q96B36	0.83413
P0C214	A6XGL0	0.54838
P0C214	Q9Y2G0	0.10244
P0C214	Q9UIF7	0.42042
P0C214	Q6PKG0	0.5625
P0C214	P62891	0.86977
P0C214	Q5JR98	0.55193
P0C214	Q9C0F3	0.3338

P0C214	Q4W5G0	0.30991
P0C214	Q56VL3	0.30515
P0C214	Q96LW7	0.47944
P0C214	O00154	0.12417
P0C214	Q9H7X3	0.27785
P0C214	Q5JTJ3	0.14014
P0C214	Q96N22	0.49442
P0C214	Q8NEU8	0.30745
P0C214	H0UI37	0.25201
P0C214	P61574	0.39132
P0C214	A6NE82	0.086276
P0C214	Q8NHZ8	1.2017
P0C214	Q9UQC2	0.28399
P0C214	Q9UI54	0.086276
P0C214	P22531	0.37988
P0C214	P0C841	0.086276
P0C214	Q9BZQ4	0.21067
P0C214	Q9NVP1	0.1915
P0C214	Q9H1K6	0.015845
P0C214	Q5BKY6	0.086276
P0C214	Q15369	1.5991
P0C214	Q8NBJ7	0.3156
P0C214	Q6L9T8	0.38992
P0C214	A9UHW6	0.6666
P0C214	P55197	1.2877
P0C214	Q9BWD1	0.35932
P0C214	Q9NUL3	0.63665
P0C214	Q9HCK1	0.55061
P0C214	Q6VY07	0.64589
P0C214	Q16658	0.15774
P0C214	Q93073	0.88098
P0C214	Q9NXE8	0.18174
P0C214	Q86XL3	0.22216
P0C214	P35658	1.1125
P0C214	Q9NYT6	0.27328
P0C214	Q96DM3	0.16532
P0C214	Q03924	0.25652
P0C214	Q9H8M7	0.79196
P0C214	Q92917	0.83846
P0C214	Q15572	0.8098
P0C214	Q969X6	1.2587
P0C214	Q86YW9	0.35832
P0C214	Q9NX63	0.73816
P0C214	Q5FWF6	0.47154
P0C214	O43310	0.45248
P0C214	Q6NW29	0.14187
P0C214	Q96DF8	0.93067
P0C214	Q8NEG4	0.33915
P0C214	Q08209	0.44765
P0C214	Q96MP8	0.23659
P0C214	P18847	1.0913
P0C214	Q9HD36	0.51734
P0C214	Q9Y242	0.21761
P0C214	Q9NYW8	0.25528
P0C214	Q96QA5	0.18643
P0C214	Q92556	0.28027
P0C214	Q08AG5	0.49158
P0C214	Q9BYE7	0.66866
P0C214	H3BR10	0.086276
P0C214	Q8N1N4	0.48861
P0C214	Q92769	0.32455
P0C214	Q12834	0.73852
P0C214	Q6UWZ7	0.39658
P0C214	Q6ZV65	0.42412
P0C214	P0CW71	0.086276
P0C214	Q8NEY8	0.67041
P0C214	Q86TJ5	0.27573
P0C214	Q9C073	0.53087
P0C214	Q8TEV8	0.086276
P0C214	Q96K31	0.088201
P0C214	Q93100	0.31572
P0C214	A8MUA0	0.22102
P0C214	O15105	0.29711
P0C214	Q14671	0.23385
P0C214	P55199	0.99346
P0C214	P60608	0.42865

P0C214	O60583	0.97467
P0C214	Q9ULW0	0.68923
P0C214	Q13243	0.76067
P0C214	Q9NZJ0	0.72961
P0C214	P0DMB2	0.47185
P0C214	Q9C005	0.59728
P0C214	Q14240	0.35167
P0C214	Q9H8G1	0.4838
P0C214	Q8N1L1	0.086276
P0C214	Q8IYL2	0.30768
P0C214	Q15468	0.17667
P0C214	P08670	0.14426
P0C214	Q86UP8	0.07874
P0C214	Q3LI67	0.086276
P0C214	P63151	0.54163
P0C214	Q8N6M9	0.092547
P0C214	Q9UPR3	1.1303
P0C214	Q9UH92	0.8561
P0C214	O94817	0.43971
P0C214	Q9UII4	0.52698
P0C214	Q9UBU8	0.68808
P0C214	Q8TAF3	0.60491
P0C214	Q8TD47	0.059237
P0C214	Q7L2H7	0.52441
P0C214	Q13522	0.15955
P0C214	Q5T0L3	1.1526
P0C214	Q13352	0.4324
P0C214	Q8N398	0.62513
P0C214	O43592	0.23888
P0C214	Q6GMV2	0.24139
P0C214	Q15427	0.32826
P0C214	P05543	0.85337
P0C214	Q5SW24	0.18476
P0C214	Q8N108	1.0706
P0C214	P63272	0.87661
P0C214	Q15544	1.0982
P0C214	P20749	0.36341
P0C214	Q9HAZ2	0.28646
P0C214	Q96AQ1	0.46157
P0C214	P83876	0.87357
P0C214	Q9H8X3	0.11647
P0C214	Q14691	0.35182
P0C214	Q53TS8	0.35248
P0C214	Q9P2I0	0.81636
P0C214	Q8N7M2	0.47643
P0C214	F5GYI3	0.83956
P0C214	P17028	0.61459
P0C214	Q9P2J8	0.36207
P0C214	O95780	0.52994
P0C214	P0CH99	0.56767
P0C214	Q7RTS9	0.84346
P0C214	Q99590	0.53845
P0C214	P98169	0.1226
P0C214	Q9UMX3	0.5209
P0C214	Q8N3X6	0.39103
P0C214	P49848	0.62685
P0C214	O43663	0.76155
P0C214	Q86WU2	0.2912
P0C214	Q15643	0.77327
P0C214	Q15329	1.1328
P0C214	Q8N490	0.56141
P0C214	Q8TAE8	1.0095
P0C214	Q8WZA8	0.086276
P0C214	P50222	0.47948
P0C214	Q53EZ4	0.55287
P0C214	Q9BUK6	0.13252
P0C214	Q6ZRV2	0.54636
P0C214	P0CL83	0.5567
P0C214	P28324	1.2626
P0C214	Q3MIS6	0.48849
P0C214	Q6P4R8	1.0423
P0C214	P54577	0.097249
P0C214	Q9GZY1	0.75882
P0C214	Q9UMS5	0.6044
P0C214	Q6P1N9	0.14098
P0C214	P16383	0.78534

P0C214	Q9UKV0	0.39097
P0C214	Q9HCU9	1.0349
P0C214	Q6Q795	0.086276
P0C214	P52597	0.54206
P0C214	Q8WW38	0.48189
P0C214	Q96G23	0.22158
P0C214	O95067	0.56103
P0C214	O00257	0.92826
P0C214	Q9Y5J9	0.51271
P0C214	Q8WU03	0.61009
P0C214	Q15652	0.54348
P0C214	Q8WXC3	0.71927
P0C214	Q9UL45	0.19436
P0C214	P82909	0.33048
P0C214	Q9H0E2	0.26572
P0C214	O60356	0.78098
P0C214	Q9NVI1	0.55104
P0C214	B4DU55	0.36913
P0C214	Q9NQG5	1.0891
P0C214	Q9BUW7	0.24937
P0C214	Q9UF83	0.09009
P0C214	Q9BZE7	0.24937
P0C214	P15927	0.38459
P0C214	Q9H8H0	0.16769
P0C214	Q8NDW8	0.12047
P0C214	Q9H160	0.40381
P0C214	Q8TCS8	0.22468
P0C214	Q9H2G9	0.31818
P0C214	Q96A09	0.53384
P0C214	Q9NQX6	0.31277
P0C214	Q8IVM0	0.45292
P0C214	Q5T8I9	0.23774
P0C214	Q96LK0	0.13923
P0C214	O43679	0.16536
P0C214	Q96MG7	0.75044
P0C214	O75152	0.36483
P0C214	Q12872	0.75888
P0C214	Q96RK1	0.64028
P0C214	P25788	0.80504
P0C214	Q3B726	0.1779
P0C214	Q9UNS1	0.4393
P0C214	P05546	0.92489
P0C214	P0CW23	0.34056
P0C214	O43502	0.30429
P0C214	Q96E29	0.4175
P0C214	P51815	0.23111
P0C214	Q9NPA8	0.337
P0C214	Q6ZS27	0.30372
P0C214	Q86XN7	0.24937
P0C214	Q9ULW8	0.35572
P0C214	P62136	0.76937
P0C214	Q9NZE8	0.5902
P0C214	O14896	0.52071
P0C214	P02100	0.3641
P0C214	A2RTX5	0.6462
P0C214	P51522	0.29177
P0C214	P26998	0.63292
P0C214	A6NFF2	0.28016
P0C214	Q8N393	0.27688
P0C214	Q9H9G7	0.77466
P0C214	O43823	0.14904
P0C214	P62495	0.78293
P0C214	Q6ZN28	0.60837
P0C214	Q8NDM7	0.41893
P0C214	Q96TA1	0.35642
P0C214	P24941	0.55321
P0C214	O15382	0.19682
P0C214	Q14157	0.26923
P0C214	O15060	0.10637
P0C214	O75334	0.44893
P0C214	P40337	0.94418
P0C214	Q86TP1	0.73946
P0C214	Q9NXG0	0.058823
P0C214	Q05519	0.68138
P0C214	P0DL12	0.029386
P0C214	P53804	0.82808

P0C214	Q9BRJ6	0.38521
P0C214	A6NCN2	0.50374
P0C214	Q9BUG6	0.20198
P0C214	Q9Y603	1.0981
P0C214	Q5W0A0	0.22181
P0C214	Q9UBS8	0.69228
P0C214	P49321	0.61984
P0C214	Q8WZ19	0.062476
P0C214	P19623	0.20013
P0C214	Q9Y5N5	0.26165
P0C214	Q15714	0.7487
P0C214	O14519	0.13753
P0C214	I3L3R5	0.086276
P0C214	Q99689	0.18776
P0C214	Q8TDC0	0.41241
P0C214	Q7RTV0	0.39038
P0C214	Q2M238	0.028619
P0C214	Q76FK4	0.38816
P0C214	Q9HCK8	0.35227
P0C214	Q0VG06	0.36754
P0C214	P0C7V6	0.82922
P0C214	Q15654	0.7055
P0C214	Q8WVY7	0.27037
P0C214	Q6ZMY9	0.49442
P0C214	O95278	0.33927
P0C214	O15014	1.0105
P0C214	Q96IM9	0.087625
P0C214	P02795	0.62734
P0C214	Q9GZT9	0.38692
P0C214	O95900	0.60201
P0C214	E9PKD4	0.030304
P0C214	Q9UN79	0.78384
P0C214	Q9BQQ3	0.090327
P0C214	Q13765	1.0401
P0C214	Q96PC3	0.29607
P0C214	Q9UIL1	0.69023
P0C214	Q92925	0.50962
P0C214	Q3SYG4	0.30863
P0C214	P38646	0.72307
P0C214	P58557	0.82995
P0C214	Q8WXI9	0.41939
P0C214	Q9ULD2	0.28916
P0C214	P42574	0.24109
P0C214	Q9UI43	0.70784
P0C214	Q05BQ5	0.17355
P0C214	P17480	0.98161
P0C214	Q8N7H5	0.90526
P0C214	Q6ZS52	0.086276
P0C214	Q9BY65	0.091496
P0C214	E7ETH6	0.30272
P0C214	Q99618	0.93313
P0C214	Q8IXQ4	0.24937
P0C214	Q04323	0.29466
P0C214	Q14019	0.16863
P0C214	Q9Y6G3	0.23359
P0C214	P50616	0.32981
P0C214	Q9H6K5	0.24937
P0C214	P36639	0.4373
P0C214	P62269	0.93216
P0C214	P19367	0.57747
P0C214	Q13185	0.74369
P0C214	Q9NVU7	0.35426
P0C214	P63162	0.58486
P0C214	Q16576	0.36325
P0C214	P0C880	0.086276
P0C214	O95886	0.49546
P0C214	Q9BVC3	0.45439
P0C214	Q2QGD7	0.66011
P0C214	O15226	0.81227
P0C214	O75376	0.34123
P0C214	Q9BZZ5	0.35381
P0C214	Q96AB3	0.8061
P0C214	Q70Z53	0.59726
P0C214	Q49AN0	0.15892
P0C214	Q9Y4W2	0.77226
P0C214	Q9Y6X9	0.28491

P0C214	Q96N58	0.32351
P0C214	P30043	0.25939
P0C214	A8MZA4	0.37738
P0C214	P0DI81	1.0612
P0C214	Q9UNM6	0.35179
P0C214	Q99550	0.2475
P0C214	Q9BYR2	0.43378
P0C214	Q96M11	0.69033
P0C214	P17020	0.23797
P0C214	Q8TE69	0.2675
P0C214	P19429	0.05525
P0C214	Q96KT6	0.086276
P0C214	Q9ULD0	0.55106
P0C214	P0CW00	0.69286
P0C214	Q9BRT7	0.086276
P0C214	Q15025	0.48785
P0C214	Q9ULX9	0.3337
P0C214	P04733	0.37691
P0C214	Q76KX8	0.50034
P0C214	P67775	0.72514
P0C214	A6NEV1	0.41187
P0C214	Q5UE93	0.61634
P0C214	Q6ZN01	0.54531
P0C214	Q86TB9	0.93199
P0C214	Q8NFH3	0.97357
P0C214	Q96MR7	1.1597
P0C214	P10809	0.22074
P0C214	Q9BUJ2	1.1245
P0C214	Q15459	0.63881
P0C214	P0C7T7	0.086276
P0C214	Q08AI8	0.24937
P0C214	Q9NRJ2	0.086276
P0C214	Q99848	0.21813
P0C214	Q9BZW7	0.24633
P0C214	O00231	0.61566
P0C214	P41567	0.87517
P0C214	O60264	0.22187
P0C214	Q9BPX1	0.45612
P0C214	P19532	0.67715
P0C214	Q15796	0.55508
P0C214	P48634	0.79434
P0C214	Q9NZ52	0.34155
P0C214	Q6NVH7	0.48272
P0C214	P42771	0.54051
P0C214	Q56P03	0.47093
P0C214	Q9H019	0.32651
P0C214	Q9NSC2	0.26183
P0C214	Q96I24	0.41501
P0C214	Q9Y5J6	0.36952
P0C214	Q9UHI6	0.43781
P0C214	Q9H0G5	0.56828
P0C214	Q8TE76	0.14739
P0C214	Q69YI7	0.81041
P0C214	P61579	0.39132
P0C214	Q969J2	0.25668
P0C214	Q9P0W5	0.53306
P0C214	Q9ULM2	0.19723
P0C214	P51816	0.38179
P0C214	P12882	0.89031
P0C214	Q96T21	0.60665
P0C214	P11441	0.62536
P0C214	Q9H9L3	0.45919
P0C214	Q14192	0.66464
P0C214	P62324	0.65354
P0C214	O15318	0.5551
P0C214	O00204	0.62852
P0C214	Q6UUV9	1.5188
P0C214	O60930	0.33473
P0C214	O75717	0.30282
P0C214	Q6L8G9	0.086276
P0C214	P0C5Y4	0.081911
P0C214	P41223	0.09131
P0C214	Q6P2H3	0.44208
P0C214	P62318	0.89139
P0C214	Q9NRF8	0.33618
P0C214	O43593	0.1174

P0C214	O15525	0.26634
P0C214	Q86V71	0.39888
P0C214	Q8NDV7	0.2895
P0C214	Q9UMY1	0.8529
P0C214	Q96CS3	0.55353
P0C214	Q9Y3M9	0.21809
P0C214	P22674	0.25109
P0C214	Q7Z6B0	0.51616
P0C214	P17027	0.25925
P0C214	Q9BRP1	0.81409
P0C214	Q86TU7	0.25096
P0C214	Q9BYQ0	0.43378
P0C214	P25963	0.61404
P0C214	Q5VSD8	0.00049291
P0C214	Q86UQ0	0.27943
P0C214	A8MQ03	0.67894
P0C214	Q96KN1	0.35932
P0C214	Q8IY85	0.28537
P0C214	A6NLX3	0.10536
P0C214	Q01543	0.29355
P0C214	O60381	0.16353
P0C214	O43598	0.19551
P0C214	O95411	0.086276
P0C214	Q96RL7	0.91327
P0C214	P59998	0.17995
P0C214	Q96GC6	1.0332
P0C214	Q99687	0.3352
P0C214	Q99594	1.0319
P0C214	E5RJ46	0.086276
P0C214	P41182	0.1413
P0C214	Q9Y6X8	0.59208
P0C214	P35244	0.50116
P0C214	O95429	0.31074
P0C214	Q96K30	0.67982
P0C214	Q8N0U6	0.086276
P0C214	O60711	0.35812
P0C214	Q6P9G9	0.23921
P0C214	O15360	0.70005
P0C214	Q03923	0.52248
P0C214	O60294	0.31329
P0C214	P14324	0.52869
P0C214	Q3SY00	0.61615
P0C214	Q5SV97	0.41108
P0C214	Q9H1B7	0.9375
P0C214	P07311	0.36285
P0C214	Q9BXJ9	0.6323
P0C214	Q5VZL5	0.78761
P0C214	P07093	0.85514
P0C214	Q96FF7	0.086276
P0C214	Q8TAV0	0.53941
P0C214	Q99504	0.48251
P0C214	Q9NRR4	0.49796
P0C214	Q9Y5B9	0.27318
P0C214	Q06323	1.1025
P0C214	Q5VX52	0.086185
P0C214	Q9UMX2	0.35692
P0C214	Q86SG2	0.23549
P0C214	Q9NP71	0.50949
P0C214	Q9UQR0	0.17965
P0C214	Q8N972	0.3804
P0C214	Q9P206	0.38939
P0C214	P59037	0.35211
P0C214	Q9NS73	0.6955
P0C214	Q9NQ50	0.55152
P0C214	Q9H3J6	0.66547
P0C214	P11388	0.1662
P0C214	Q9BQY4	0.72354
P0C214	Q9BVT8	0.21352
P0C214	Q9HCN2	0.086276
P0C214	P17098	0.48478
P0C214	Q8IV48	0.032814
P0C214	Q6ZT77	0.34811
P0C214	Q9H446	0.2675
P0C214	Q8NI27	0.45307
P0C214	P21506	0.34363
P0C214	Q8IY81	0.72951

P0C214	P46781	1.2594
P0C214	Q93009	0.38099
P0C214	Q9Y608	0.31299
P0C214	P61578	0.35559
P0C214	Q96KQ4	0.85509
P0C214	O43541	0.648
P0C214	Q6ZUU3	0.086276
P0C214	Q96P71	0.50392
P0C214	Q5SY13	0.086276
P0C214	Q13868	0.77531
P0C214	P30305	0.36614
P0C214	Q9Y291	0.32421
P0C214	Q3SYA9	0.26303
P0C214	Q7L1Q6	0.33495
P0C214	Q86X83	0.31698
P0C214	Q5VVC0	0.20454
P0C214	Q8IYB9	0.39888
P0C214	Q5T5Y3	0.32395
P0C214	Q8TD16	0.15813
P0C214	Q3LI63	0.086276
P0C214	Q69YL0	0.086276
P0C214	O15379	0.50392
P0C214	Q9UN30	0.20918
P0C214	P25786	0.68368
P0C214	Q8TAP6	0.90358
P0C214	Q8NEZ2	0.58758
P0C214	P06733	0.99403
P0C214	Q01081	0.52298
P0C214	Q96RL1	0.68581
P0C214	Q5BJE1	0.20816
P0C214	P60372	0.43378
P0C214	Q9NZU7	0.33704
P0C214	Q9HB75	0.56561
P0C214	Q01892	0.26437
P0C214	Q96PK6	0.73985
P0C214	Q0PNE2	0.6967
P0C214	C9J6K1	0.15595
P0C214	Q9Y2R9	0.89012
P0C214	Q96DB5	0.46336
P0C214	Q6P1X5	0.81819
P0C214	O15205	0.51248
P0C214	Q9P1T7	1.3609
P0C214	A4GXA9	0.72268
P0C214	Q96RT8	0.27841
P0C214	Q96JK9	0.91321
P0C214	Q8WW14	0.17012
P0C214	P43268	0.37639
P0C214	O75533	0.22879
P0C214	Q6AI39	0.34291
P0C214	Q96B01	0.4585
P0C214	Q96KR7	0.21557
P0C214	P19012	0.58756
P0C214	Q99619	0.59086
P0C214	O14508	0.3583
P0C214	Q53G26	0.70167
P0C214	Q15004	0.85109
P0C214	Q9Y2D1	1.0656
P0C214	Q96RS0	0.76948
P0C214	Q96MY7	0.88594
P0C214	Q96CM4	0.18195
P0C214	Q5T4T6	0.89936
P0C214	Q9Y2G2	0.44567
P0C214	Q9UHY7	0.3744
P0C214	Q8N6T7	0.59621
P0C214	Q8TC76	1.3916
P0C214	Q96DI7	0.6329
P0C214	Q9P1D8	0.086276
P0C214	O75167	0.13415
P0C214	Q969U7	0.52781
P0C214	Q9UBW7	0.70238
P0C214	Q8IVD9	0.35289
P0C214	H3BTG2	0.36066
P0C214	Q9H322	0.45745
P0C214	O75475	1.4309
P0C214	Q14690	0.73547
P0C214	Q16829	0.31349

P0C214	Q5XKK7	0.091777
P0C214	O43324	1.2876
P0C214	Q9NRF9	0.06667
P0C214	Q9NX95	0.30869
P0C214	Q9Y5U2	0.2347
P0C214	P13073	0.1999
P0C214	Q3LHN2	0.086276
P0C214	Q9NYV6	0.54205
P0C214	Q5T440	0.082247
P0C214	Q9UNH5	0.5993
P0C214	Q8TE02	1.1782
P0C214	Q8TAA5	0.49939
P0C214	Q9P2M1	0.45292
P0C214	P01008	0.88945
P0C214	P33240	0.53033
P0C214	Q16514	0.74487
P0C214	Q9UKC9	0.55612
P0C214	Q9BXF6	0.48768
P0C214	O95251	0.8289
P0C214	Q96EV8	0.28489
P0C214	Q9H211	0.71816
P0C214	Q2M3D2	0.23707
P0C214	Q4LE28	0.70372
P0C214	P25063	0.086276
P0C214	Q9Y3F4	0.92514
P0C214	Q8IXH6	1.2435
P0C214	Q7RTU3	0.25688
P0C214	Q8WYA1	0.26709
P0C214	P17035	0.26437
P0C214	Q9HA77	0.57449
P0C214	P62736	0.10604
P0C214	P82970	0.56739
P0C214	Q13616	0.61705
P0C214	Q2M2W7	0.24937
P0C214	Q9NR77	0.30831
P0C214	Q9HCM9	0.61615
P0C214	Q17R31	0.23495
P0C214	O14618	0.54518
P0C214	Q8TBB1	0.75447
P0C214	A3KN83	0.78325
P0C214	Q8NDN9	0.55976
P0C214	P0DMC3	0.086276
P0C214	Q5EBL4	0.52769
P0C214	Q8WVP5	0.73452
P0C214	P0C7X5	0.33639
P0C214	Q7Z7K6	0.39311
P0C214	Q9NW08	0.090663
P0C214	Q9Y3B9	0.38699
P0C214	Q6NVY1	0.14712
P0C214	Q66K80	0.086276
P0C214	O95202	0.18935
P0C214	P04259	0.25397
P0C214	Q9P2B4	0.23817
P0C214	Q8IYD1	0.59363
P0C214	Q8IX07	0.28951
P0C214	Q96CV9	0.41465
P0C214	Q58FF3	0.99507
P0C214	Q9ULX6	0.49779
P0C214	Q58FF6	0.8863
P0C214	P78559	0.63761
P0C214	Q96CN0	0.086276
P0C214	I6L9F6	0.15831
P0C214	Q8WTP8	0.099752
P0C214	Q9H607	0.43421
P0C214	Q8WXU2	0.71941
P0C214	A6NIN4	0.086276
P0C214	Q6ZVC0	0.78573
P0C214	P48378	0.242
P0C214	P38117	0.25913
P0C214	Q9UJL9	0.24964
P0C214	Q8N5M1	0.48453
P0C214	O43752	0.083834
P0C214	Q3ZCV2	0.24937
P0C214	Q8TES7	0.46846
P0C214	Q86X51	0.11224
P0C214	Q9BRT3	0.11128

33061	P0C214	Q9H4B0	0.26979
33062	P0C214	A2RUB1	0.24937
33063	P0C214	Q9NV56	0.6156
33064	P0C214	P49588	0.17539
33065	P0C214	Q9H9Z2	0.66934
33066	P0C214	O00193	0.73154
33067	P0C214	P12755	0.13003
33068	P0C214	Q6U7Q0	0.31247
33069	P0C214	A4D1E1	0.39345
33070	P0C214	P62879	0.47369
33071	P0C214	A6NKH3	0.50471
33072	P0C214	O94850	0.2103
33073	P0C214	Q13127	0.48619
33074	P0C214	A8MU10	0.086276
33075	P0C214	Q9ULZ2	0.76617
33076	P0C214	Q9C075	0.44878
33077	P0C214	P0DKL9	0.63546
33078	P0C214	Q96GW9	0.43891
33079	P0C214	Q14934	0.48123
33080	P0C214	Q9NX55	0.41348
33081	P0C214	Q4LE39	0.96843
33082	P0C214	Q9UBF1	0.74972
33083	P0C214	Q8NDW4	0.2817
33084	P0C214	Q9UG56	0.32034
33085	P0C214	P13994	0.38384
33086	P0C214	P23921	0.22202
33087	P0C214	Q9BSG1	0.38137
33088	P0C214	P53041	0.73417
33089	P0C214	Q8IU60	1.0385
33090	P0C214	Q6P1M9	0.27253
33091	P0C214	Q01850	0.84761
33092	P0C214	O75940	0.57763
33093	P0C214	Q9BYC9	0.74818
33094	P0C214	O75155	0.78447
33095	P0C214	Q7Z4U5	0.90801
33096	P0C214	Q8N1B4	0.3943
33097	P0C214	Q8WV99	0.17284
33098	P0C214	Q9NXW9	0.27869
33099	P0C214	Q15650	0.71783
33100	P0C214	Q96LR7	0.60714
33101	P0C214	P62875	0.30567
33102	P0C214	P28069	0.60416
33103	P0C214	Q8TB05	0.24937
33104	P0C214	P30740	0.96136
33105	P0C214	Q9NSQ0	0.45677
33106	P0C214	Q2VY69	0.35336
33107	P0C214	O60763	0.3319
33108	P0C214	Q96P63	0.92484
33109	P0C214	Q8WUW1	0.2436
33110	P0C214	Q9Y252	0.45847
33111	P0C214	A8MU76	0.6505
33112	P0C214	O75346	0.44074
33113	P0C214	Q86YI8	0.4013
33114	P0C214	A2RRD8	0.32699
33115	P0C214	Q5W1A9	0.52643
33116	P0C214	Q96BP3	0.68301
33117	P0C214	Q8NDZ9	0.086276
33118	P0C214	Q4G0N7	0.42506
33119	P0C214	P43359	0.84363
33120	P0C214	Q6ZNG0	0.29348
33121	P0C214	Q5RHP9	0.29767
33122	P0C214	P13984	0.59207
33123	P0C214	Q15072	0.21906
33124	P0C214	P20226	0.89918
33125	P0C214	Q93075	0.65392
33126	P0C214	Q969H0	0.70874
33127	P0C214	A6NJB7	0.81462
33128	P0C214	Q96JS3	0.2637
33129	P0C214	P13682	0.33929
33130	P0C214	Q12959	0.83302
33131	P0C214	Q9H2G4	0.65596
33132	P0C214	Q99871	0.39644
33133	P0C214	Q9BTC8	0.49724
33134	P0C214	Q96D03	0.26939
33135	P0C214	Q9UHV2	1.4859
33136	P0C214	P61254	1.219

P0C214	Q96AQ6	0.55892
P0C214	Q5SZD4	0.48517
P0C214	Q15424	0.76988
P0C214	Q86W11	0.22221
P0C214	Q1W6H9	1.1281
P0C214	Q13227	1.0011
P0C214	Q12874	0.46832
P0C214	Q8N443	0.13274
P0C214	Q6NSZ9	0.17892
P0C214	Q14511	0.55715
P0C214	P28072	0.61611
P0C214	A6NL82	0.029641
P0C214	O95777	0.57804
P0C214	Q75QN2	0.54729
P0C214	Q92890	0.90679
P0C214	Q3YBR2	0.41251
P0C214	A8MTA8	0.078024
P0C214	Q86V42	0.28965
P0C214	A8MWA4	0.46621
P0C214	Q14160	0.46978
P0C214	O75534	0.11145
P0C214	Q14653	0.60616
P0C214	Q5TC12	0.43922
P0C214	Q8IZX4	0.28016
P0C214	Q86Y97	0.42516
P0C214	Q9NVK5	0.22364
P0C214	Q8N4B4	0.26516
P0C214	O00505	1.024
P0C214	Q9UI36	0.36065
P0C214	Q9Y4X0	0.33461
P0C214	Q9UBP6	1.0762
P0C214	Q6P2C8	0.94853
P0C214	Q8IXQ5	0.31982
P0C214	Q5SXM1	0.37755
P0C214	Q9P2Y5	0.34574
P0C214	O75494	0.62892
P0C214	P09972	0.76774
P0C214	O75531	0.96435
P0C214	Q96ND8	0.51762
P0C214	Q5TA31	0.56978
P0C214	Q9H7B2	0.95185
P0C214	P54727	0.65065
P0C214	O75529	0.52768
P0C214	Q6NXT1	0.30992
P0C214	Q9BSJ6	0.54635
P0C214	Q8IVT2	0.58821
P0C214	Q32MH5	0.23755
P0C214	Q9H2K2	0.35441
P0C214	O75943	0.20224
P0C214	Q9BSD3	0.17731
P0C214	Q86UU0	0.97844
P0C214	Q9UDY2	0.87332
P0C214	Q6ZNA4	0.63005
P0C214	Q14397	0.78708
P0C214	Q96D15	0.29312
P0C214	Q9H4T2	0.18742
P0C214	P25815	0.52999
P0C214	P32780	0.67521
P0C214	Q9Y4A5	0.75689
P0C214	Q7Z7A3	0.80015
P0C214	Q8TED0	0.298
P0C214	Q5JTH9	0.3548
P0C214	Q9UQF2	0.54982
P0C214	Q9UKV8	0.88366
P0C214	P41208	0.61173
P0C214	O75360	0.29152
P0C214	P60413	0.43378
P0C214	Q7L5D6	0.93611
P0C214	Q9H5X1	0.17854
P0C214	A6NJ08	0.064098
P0C214	P17040	0.62728
P0C214	Q96NB3	0.3826
P0C214	P51116	0.54062
P0C214	O95639	0.39073
P0C214	Q9H0E9	0.33017
P0C214	Q5TAP6	0.35516

P0C214	Q6GPH4	0.46674
P0C214	P16455	0.21681
P0C214	Q8N7W2	0.43958
P0C214	Q6V9R5	0.42705
P0C214	P62841	0.9238
P0C214	Q9P0J1	0.13574
P0C214	Q8N9Z9	0.50158
P0C214	O00512	0.75022
P0C214	Q6PB30	0.46641
P0C214	Q8TAA3	0.4743
P0C214	Q8WVC0	0.97947
P0C214	Q9H840	0.99018
P0C214	O96007	0.7898
P0C214	Q9Y2X9	0.53258
P0C214	P11387	0.60677
P0C214	Q14140	1.4757
P0C214	P12883	0.75898
P0C214	Q9BTE7	0.60234
P0C214	P58317	0.27826
P0C214	Q9UDV6	0.51321
P0C214	Q96C00	0.33732
P0C214	P10074	0.31788
P0C214	Q92845	0.26055
P0C214	Q15287	1.0845
P0C214	Q8WXS3	0.69946
P0C214	Q13536	0.086276
P0C214	P24928	0.77244
P0C214	Q86YC2	0.54164
P0C214	Q9P1J3	0.086276
P0C214	O43918	0.95958
P0C214	P0C5K6	0.086276
P0C214	O94903	0.47486
P0C214	Q68EA5	0.47643
P0C214	Q99496	0.45512
P0C214	A8MTZ0	0.1603
P0C214	P36402	0.42047
P0C214	Q9Y3E7	0.64729
P0C214	P62244	0.98797
P0C214	Q8N535	0.088172
P0C214	Q8NAB5	0.086276
P0C214	Q9NP64	0.21669
P0C214	Q9HD26	0.77018
P0C214	Q8TAK5	0.42006
P0C214	Q9C0D0	0.39152
P0C214	P59022	0.3707
P0C214	P52747	0.73937
P0C214	Q8IYX0	0.48999
P0C214	Q9NRX2	0.44761
P0C214	Q9BRT2	0.37704
P0C214	O75582	0.24024
P0C214	P26651	0.67734
P0C214	O60784	0.36937
P0C214	Q9BQI5	0.10759
P0C214	P10242	0.8584
P0C214	Q494R0	0.086276
P0C214	Q9UHV7	0.51898
P0C214	Q8N9Z0	0.44153
P0C214	P62987	0.79237
P0C214	P09622	0.28034
P0C214	Q6XD76	0.25897
P0C214	O14593	0.61102
P0C214	Q9NPI8	0.76317
P0C214	Q14135	0.39389
P0C214	Q8N0U4	0.21899
P0C214	Q9NV72	0.46031
P0C214	P57077	0.888
P0C214	Q9ULV5	0.27177
P0C214	P16333	0.72469
P0C214	Q9BXF3	0.56161
P0C214	P86480	0.086276
P0C214	P62826	0.78322
P0C214	Q6ZU65	0.23697
P0C214	Q6IN85	0.32771
P0C214	A8MYR8	0.0043805
P0C214	Q5VIY5	0.44782
P0C214	Q6P3X8	0.15946

P0C214	Q5W5X9	0.14652
P0C214	Q9BZ23	0.48441
P0C214	Q12983	0.11502
P0C214	P50453	0.86267
P0C214	Q5THR3	0.33132
P0C214	Q8N9K3	0.24937
P0C214	Q8WUB8	0.33863
P0C214	Q5XKR9	0.41022
P0C214	Q9Y462	0.5484
P0C214	A2RUQ5	0.040743
P0C214	P08727	0.58756
P0C214	Q9UEG4	0.31744
P0C214	Q6ZSR6	0.21846
P0C214	Q9H410	0.87927
P0C214	Q15935	0.37571
P0C214	P22670	0.76033
P0C214	Q9H9T3	0.81439
P0C214	P39880	0.54376
P0C214	P42773	0.33019
P0C214	Q6NUP7	0.38579
P0C214	P59991	0.51868
P0C214	O60806	0.37581
P0C214	Q9UBD0	1.0077
P0C214	Q96LR2	0.81251
P0C214	Q2TAZ0	0.27384
P0C214	P62633	0.67853
P0C214	Q15750	0.52154
P0C214	Q9UKT9	0.41654
P0C214	P36508	0.084997
P0C214	Q92558	0.34547
P0C214	P43243	0.16507
P0C214	Q7L945	0.29427
P0C214	O60671	0.48918
P0C214	P54687	0.026156
P0C214	Q15532	0.96198
P0C214	O14598	0.57136
P0C214	Q07343	0.23506
P0C214	Q7Z7F7	0.67358
P0C214	Q6ZRC1	0.086276
P0C214	Q04637	0.65365
P0C214	O60832	0.68238
P0C214	A6NHR8	0.086276
P0C214	A6NEY8	0.093148
P0C214	O43345	0.37211
P0C214	P31943	0.48963
P0C214	O60563	1.4294
P0C214	Q9BYQ7	0.5539
P0C214	Q9H4P4	0.34154
P0C214	Q96A04	0.62777
P0C214	Q9Y294	0.9473
P0C214	P23497	0.83367
P0C214	Q96LY2	0.46157
P0C214	Q8IUR6	0.8769
P0C214	O96000	0.48484
P0C214	Q5TBE3	0.31838
P0C214	O94992	1.2742
P0C214	Q92843	0.52171
P0C214	Q6IE81	0.95387
P0C214	P26447	0.3001
P0C214	Q9BX26	1.0938
P0C214	Q99816	0.36203
P0C214	O75665	0.69877
P0C214	O15392	0.24338
P0C214	Q9Y2R5	0.49263
P0C214	Q32P41	0.46131
P0C214	Q2M2Z5	0.50871
P0C214	Q8IWD4	0.24937
P0C214	Q5T8A7	0.68461
P0C214	Q9Y6G5	0.2675
P0C214	Q9NXV6	0.9208
P0C214	O43747	0.63335
P0C214	Q92499	0.41382
P0C214	Q6IPR1	0.3532
P0C214	P43356	0.85082
P0C214	Q96NL0	0.065242
P0C214	Q96E11	0.6058

P0C214	O75920	0.9744
P0C214	P05412	0.79058
P0C214	P62304	0.80392
P0C214	Q9H8M2	0.10184
P0C214	Q9H1Y0	0.45363
P0C214	Q8N9B5	0.68432
P0C214	Q6P444	0.32651
P0C214	Q8WU90	0.83217
P0C214	Q9UMQ3	0.48029
P0C214	Q9Y2G7	0.54106
P0C214	Q07020	1.0005
P0C214	Q8WY41	0.3771
P0C214	Q8N5R6	0.4662
P0C214	Q9H6D7	0.37012
P0C214	P17041	0.21141
P0C214	O95476	0.26252
P0C214	Q8NB50	0.38935
P0C214	O95073	1.0428
P0C214	Q99456	0.58756
P0C214	Q9UPY8	0.85326
P0C214	Q96I25	0.30011
P0C214	A8MX80	0.64099
P0C214	Q14669	0.46506
P0C214	Q9NYB0	0.69582
P0C214	Q5JU67	0.040911
P0C214	Q96HQ0	0.29646
P0C214	Q9BY89	0.020693
P0C214	Q8WVJ9	0.48352
P0C214	Q5T5A4	0.31123
P0C214	O00442	0.44231
P0C214	Q96CG3	0.27276
P0C214	Q96B23	0.81535
P0C214	Q9H3R5	0.55279
P0C214	Q86WV1	0.49754
P0C214	Q9NV92	0.055995
P0C214	Q96E09	0.3662
P0C214	Q6NX45	0.30702
P0C214	E9PQ53	0.63248
P0C214	P49711	0.16868
P0C214	Q86YA3	0.59896
P0C214	Q6PF05	0.36663
P0C214	Q9NQE9	0.49557
P0C214	Q15323	0.51477
P0C214	Q5MNV8	0.28354
P0C214	Q14781	0.63654
P0C214	Q9NV88	0.54729
P0C214	Q9HD47	0.23677
P0C214	Q8N7Z2	0.37738
P0C214	K7EIQ3	0.79811
P0C214	Q49A88	0.27024
P0C214	Q8NFZ5	0.87411
P0C214	Q01826	0.58294
P0C214	Q7Z5K2	0.68035
P0C214	Q13106	0.23468
P0C214	P0C853	0.086276
P0C214	Q9NUP7	0.87683
P0C214	Q9BQ69	0.49304
P0C214	Q02447	0.45465
P0C214	Q709C8	0.57595
P0C214	Q99766	0.51027
P0C214	Q12857	0.27602
P0C214	O15297	0.59744
P0C214	Q69383	0.39132
P0C214	Q8N2G8	0.23317
P0C214	Q9BX70	0.48486
P0C214	Q9NZM5	0.44348
P0C214	O75410	0.64976
P0C214	Q96RU7	0.45509
P0C214	P42677	0.98345
P0C214	P12036	0.38243
P0C214	P62945	0.97266
P0C214	Q9HBL0	0.24053
P0C214	Q9Y2J8	0.54022
P0C214	Q8N8E1	0.20727
P0C214	O75436	0.62502
P0C214	O15160	0.12842

P0C214	Q9H2U1	0.32166
P0C214	O60291	0.57788
P0C214	Q9UFV3	0.033817
P0C214	O95182	0.079041
P0C214	Q9P000	0.24456
P0C214	Q96IR3	0.54568
P0C214	O75822	0.8284
P0C214	Q9NVD3	0.045664
P0C214	Q6NVV1	0.67347
P0C214	Q9BXW9	0.68317
P0C214	Q8IXR5	0.053772
P0C214	O75474	0.87479
P0C214	Q14738	0.67984
P0C214	Q9NZI6	0.44388
P0C214	Q5VIR6	0.74498
P0C214	P06454	0.45843
P0C214	Q8IX90	0.49546
P0C214	P28749	1.2273
P0C214	Q7Z7L8	0.96197
P0C214	A6NEY3	0.49852
P0C214	O95273	0.84805
P0C214	P09012	0.37897
P0C214	Q14683	0.37808
P0C214	Q92831	0.74541
P0C214	O75419	0.79388
P0C214	Q8N8Z8	0.27596
P0C214	Q9Y535	0.22129
P0C214	P42025	0.39308
P0C214	O95793	0.24773
P0C214	A8MTB1	0.086276
P0C214	Q53H82	0.18546
P0C214	P17021	0.25339
P0C214	Q8N9Y4	0.92562
P0C214	Q5VT40	0.0082096
P0C214	Q9P0M9	0.52504
P0C214	Q6A162	0.44438
P0C214	O75448	0.46602
P0C214	Q719H9	0.6512
P0C214	P18146	0.57844
P0C214	Q8N3U4	0.56999
P0C214	Q16534	0.19934
P0C214	P46776	1.2375
P0C214	P05198	0.80304
P0C214	O43504	0.18781
P0C214	O94973	0.42066
P0C214	Q9NUU7	0.034031
P0C214	Q96NT1	1.1026
P0C214	Q8TEE9	1.0506
P0C214	Q9P1Q0	0.28478
P0C214	Q9HC44	1.2873
P0C214	Q15631	0.62458
P0C214	Q96JL9	0.30874
P0C214	Q9H204	1.0798
P0C214	P0C867	0.11824
P0C214	P0C7X2	0.5413
P0C214	K9M1U5	0.086276
P0C214	Q8WTR7	0.56686
P0C214	Q8N187	0.5916
P0C214	Q9UL12	0.098104
P0C214	Q6ZVN8	0.21759
P0C214	Q8TEL6	0.41567
P0C214	Q9H4A5	0.9562
P0C214	Q8IXQ9	0.425
P0C214	Q9NS25	0.51904
P0C214	Q8IU81	0.26741
P0C214	Q96IU4	0.40636
P0C214	P07327	0.20793
P0C214	Q9P2M7	0.38406
P0C214	A6PW82	0.47991
P0C214	P0CG00	0.1961
P0C214	Q9H9E1	0.25458
P0C214	O15164	0.29774
P0C214	Q96G01	0.20196
P0C214	P58340	0.30689
P0C214	P15621	0.48541
P0C214	Q99436	0.79871

P0C214	Q13126	0.22518
P0C214	Q8IWC1	0.50827
P0C214	Q0D2J5	0.20334
P0C214	P0CJ78	0.2154
P0C214	Q13114	0.4559
P0C214	O43447	0.71554
P0C214	Q9GZM3	0.13654
P0C214	O95602	0.58036
P0C214	Q9Y6Q5	0.32439
P0C214	Q02577	0.51259
P0C214	Q66K41	0.84301
P0C214	Q15773	0.73775
P0C214	Q9ULN7	0.73395
P0C214	Q8WVM7	0.64167
P0C214	Q9NPI1	0.92568
P0C214	Q2VPJ9	0.24937
P0C214	Q9BVN2	0.45737
P0C214	Q8NG31	0.6456
P0C214	P24864	0.61261
P0C214	Q5PRF9	0.59004
P0C214	Q8N196	0.54042
P0C214	P30279	0.69011
P0C214	Q9UJX4	0.84553
P0C214	Q9NZ63	0.35391
P0C214	Q9BZA5	0.69161
P0C214	P04156	0.43564
P0C214	Q8IVC4	0.28452
P0C214	P20962	0.26936
P0C214	P10909	0.20397
P0C214	Q8N3J5	0.33472
P0C214	A0JNW5	0.39623
P0C214	Q8N2N9	0.2654
P0C214	Q6ZRP0	0.24937
P0C214	P63172	0.7201
P0C214	Q96MX3	0.28348
P0C214	Q01831	0.54984
P0C214	Q9H9Q2	0.17821
P0C214	Q14584	0.28722
P0C214	Q9H009	0.36249
P0C214	P05120	0.96136
P0C214	Q9UBQ0	0.48492
P0C214	O75391	0.63212
P0C214	Q96T23	0.62292
P0C214	Q15629	0.32243
P0C214	O75489	0.3365
P0C214	Q5T7V8	1.5025
P0C214	Q3SY52	0.21984
P0C214	Q8WY54	0.89464
P0C214	Q16822	0.25585
P0C214	O14576	0.34084
P0C214	Q9NQX1	0.40468
P0C214	Q9P013	0.45853
P0C214	O94925	0.70937
P0C214	P27797	0.4823
P0C214	Q8TC84	0.35391
P0C214	P07438	0.39612
P0C214	Q15853	0.54331
P0C214	Q5VZ66	0.35824
P0C214	A6NK75	0.34824
P0C214	Q96CB8	0.43122
P0C214	P0DMB1	0.26625
P0C214	Q86U86	0.8966
P0C214	Q9C0D7	0.42931
P0C214	O15504	0.59018
P0C214	Q3KQU3	0.61269
P0C214	Q8IYA8	0.24937
P0C214	Q6ZN06	0.45138
P0C214	Q9H1I8	0.36007
P0C214	Q13439	0.90077
P0C214	Q99574	0.88947
P0C214	Q6P1L5	1.0139
P0C214	Q9NQ48	0.19586
P0C214	Q6UB98	0.35391
P0C214	O94952	0.42549
P0C214	Q9H7E2	0.34443
P0C214	P31949	0.32709

P0C214	Q9Y312	0.091507
P0C214	Q03060	0.52776
P0C214	Q7Z401	0.22663
P0C214	Q8IWE2	0.49223
P0C214	Q7Z460	0.34361
P0C214	Q9BVC6	0.19331
P0C214	Q9GZR2	0.21122
P0C214	P57071	0.14964
P0C214	Q6ZRV3	0.086276
P0C214	Q15759	0.33133
P0C214	Q96H79	0.6144
P0C214	Q9NPC3	0.16311
P0C214	O60885	0.66225
P0C214	Q8N7B6	0.59464
P0C214	Q8IVN3	0.71798
P0C214	Q6IQ21	0.40859
P0C214	Q9UM82	0.80665
P0C214	Q9P031	1.0428
P0C214	Q8N0T1	0.53577
P0C214	P34949	0.14413
P0C214	Q9BUH6	0.19108
P0C214	Q9UFN0	0.74795
P0C214	Q6ZSN1	0.13871
P0C214	Q96JB3	0.073138
P0C214	O95751	0.50209
P0C214	P40425	0.51924
P0C214	Q9BV73	0.56883
P0C214	P07902	0.533
P0C214	Q96EY4	0.34484
P0C214	Q6ZV50	0.242
P0C214	Q16526	0.37801
P0C214	Q6P589	0.50014
P0C214	Q8IYJ2	0.36021
P0C214	Q9UHB4	0.85387
P0C214	Q9UKD2	0.14212
P0C214	Q04725	0.79823
P0C214	Q5T1J5	0.75974
P0C214	Q14592	0.3116
P0C214	Q156A1	0.086276
P0C214	P68543	0.26781
P0C214	Q13895	0.71328
P0C214	O60238	0.28581
P0C214	P17036	0.27059
P0C214	O43309	0.25555
P0C214	Q9BYG5	0.62295
P0C214	P54840	0.585
P0C214	P39748	0.28328
P0C214	P09497	0.32443
P0C214	Q6PF18	0.083408
P0C214	P61981	0.58197
P0C214	Q9BQC6	0.77055
P0C214	Q9HCI5	0.6102
P0C214	P17039	0.25925
P0C214	Q02410	0.39461
P0C214	Q2V2M9	0.2235
P0C214	O60506	0.40216
P0C214	A2IDD5	0.43955
P0C214	Q3ZCQ8	0.082079
P0C214	Q6J272	0.68201
P0C214	Q1ZZU3	0.61065
P0C214	Q9NX65	0.25006
P0C214	Q86SY8	0.086276
P0C214	Q6PEX3	0.19225
P0C214	Q99440	0.32966
P0C214	Q6ZTN6	0.096911
P0C214	P0CG47	0.40707
P0C214	P52815	0.64719
P0C214	Q8N1I0	0.27762
P0C214	Q8N4M7	0.69839
P0C214	O95394	0.98672
P0C214	Q9Y5J7	0.20935
P0C214	P04053	0.20376
P0C214	P60370	0.43378
P0C214	Q107X0	0.37948
P0C214	Q8TAF7	0.47199
P0C214	P98182	0.24118

P0C214	P0C7V5	0.36588
P0C214	Q9UDV7	0.25203
P0C214	P25787	0.73413
P0C214	O14521	0.33686
P0C214	Q9NYZ3	0.26785
P0C214	P55327	0.33348
P0C214	Q99814	0.33962
P0C214	P09086	0.23659
P0C214	Q86TG7	0.37712
P0C214	P61583	0.49709
P0C214	Q9BTL3	0.51487
P0C214	P62241	0.85239
P0C214	Q99471	0.88113
P0C214	Q96IU2	0.87705
P0C214	O76009	0.5102
P0C214	P36873	0.82274
P0C214	Q86XI8	0.53004
P0C214	Q86XK2	0.81636
P0C214	P46777	1.4465
P0C214	P82932	0.47259
P0C214	Q9NZZ3	0.92422
P0C214	Q14154	0.4753
P0C214	P60673	0.11573
P0C214	P21980	0.28109
P0C214	Q8TEA1	0.5372
P0C214	Q6P387	0.35391
P0C214	Q8NB42	0.49679
P0C214	Q9NZQ3	0.656
P0C214	Q460N3	0.30706
P0C214	Q9UKK3	0.54991
P0C214	Q01658	0.28533
P0C214	Q6PJG2	0.67589
P0C214	Q9BZK7	0.86112
P0C214	Q9HCG8	0.15326
P0C214	Q12765	0.21681
P0C214	Q8NAP1	0.24937
P0C214	Q9H900	0.51187
P0C214	Q96H40	0.50851
P0C214	Q15010	0.48251
P0C214	A6NKF1	0.74013
P0C214	Q96I99	0.41969
P0C214	Q92830	0.71186
P0C214	Q15006	0.36193
P0C214	Q9H0U9	0.89424
P0C214	Q13956	0.56758
P0C214	Q96FJ2	0.77617
P0C214	Q58FF8	0.99507
P0C214	Q9GZR1	0.38815
P0C214	O14893	0.78947
P0C214	Q6ZRT6	0.24937
P0C214	Q6NXP2	0.75769
P0C214	Q9BSW2	0.71253
P0C214	Q9H469	0.2627
P0C214	Q52M93	0.22841
P0C214	Q5FBB7	0.68952
P0C214	O14734	0.35552
P0C214	Q96GN5	0.86356
P0C214	O14558	0.74433
P0C214	Q9UP83	1.2351
P0C214	Q8IXJ6	0.28155
P0C214	Q53SZ7	0.24937
P0C214	Q9BRD0	0.62937
P0C214	P61582	0.79382
P0C214	Q12796	1.43
P0C214	Q15054	0.61668
P0C214	A1A4G5	0.086276
P0C214	Q13615	0.23774
P0C214	Q9NQ31	0.62755
P0C214	Q96ST3	0.68028
P0C214	Q96LQ0	0.46952
P0C214	A5PLN7	0.24937
P0C214	P23526	0.3058
P0C214	Q8NEG2	0.35192
P0C214	Q8IYX7	0.57704
P0C214	Q9UBL0	0.24413
P0C214	Q96HB5	0.19033

P0C214	Q75L30	0.086276
P0C214	O60925	0.037262
P0C214	P55884	0.41809
P0C214	Q96N06	0.30206
P0C214	P0C860	0.30774
P0C214	Q13416	0.45245
P0C214	P09629	0.79789
P0C214	Q96NU7	0.1987
P0C214	Q8N8L2	0.22593
P0C214	Q86Y13	0.37241
P0C214	A6NNF4	0.38021
P0C214	P10599	0.56097
P0C214	Q9H9Y2	0.20342
P0C214	Q9HB58	0.248
P0C214	P02792	0.19676
P0C214	Q9BTV5	0.31057
P0C214	P60006	0.31033
P0C214	Q8N0Y7	1.128
P0C214	Q5DJT8	0.19717
P0C214	Q96IT6	0.086276
P0C214	Q59GR2	0.086281
P0C214	A6NG73	0.22305
P0C214	Q8N4S0	0.24937
P0C214	O75396	0.14369
P0C214	Q9Y2Y0	0.95835
P0C214	Q8IYA6	0.50029
P0C214	Q6NUR6	0.57378
P0C214	Q96SL8	0.2298
P0C214	Q8WW33	0.46486
P0C214	Q8IUI4	0.20131
P0C214	Q9NUP1	0.72531
P0C214	Q9Y5B0	0.56063
P0C214	P24534	0.75105
P0C214	Q9Y6M1	0.49792
P0C214	P0CG12	0.99949
P0C214	Q9BTD1	0.21386
P0C214	P11161	0.42026
P0C214	Q9NVR2	0.92769
P0C214	O00399	0.15703
P0C214	Q3ZLR7	0.82922
P0C214	Q2NKX9	0.24937
P0C214	Q13888	0.49337
P0C214	Q9BQ95	1.1318
P0C214	Q6ZRF7	0.19865
P0C214	Q9HAY2	0.071673
P0C214	Q96Q06	0.6385
P0C214	O75150	0.54618
P0C214	Q86X02	0.30705
P0C214	Q9UJW8	0.22359
P0C214	P83731	0.97266
P0C214	Q96JM3	0.32627
P0C214	Q9Y6X3	0.87223
P0C214	Q6ULP2	0.63782
P0C214	P13647	0.90353
P0C214	Q15038	1.2759
P0C214	Q8IW50	0.24937
P0C214	A8MWP6	0.086276
P0C214	Q8WZA9	0.24937
P0C214	Q9NQS7	0.81933
P0C214	A6NKN8	0.76896
P0C214	Q8IXM2	0.17121
P0C214	P42765	0.25069
P0C214	Q9Y6D6	0.61115
P0C214	Q9NZH4	0.87884
P0C214	Q9GZX7	0.26206
P0C214	P09661	0.60157
P0C214	Q5VWP3	0.58342
P0C214	Q9UKZ1	0.45005
P0C214	Q969M7	0.58719
P0C214	Q5SNT6	0.66607
P0C214	Q9UHJ9	0.37191
P0C214	Q96L21	0.68931
P0C214	O75251	0.33116
P0C214	P45973	0.7101
P0C214	Q9UMR2	0.082082
P0C214	Q9UQ35	0.37275

P0C214	Q8IYN6	0.3662
P0C214	Q6ZMV8	0.48577
P0C214	Q5VTQ0	0.17233
P0C214	Q5T619	0.30008
P0C214	Q92973	1.2214
P0C214	Q96AE4	0.49652
P0C214	H0Y354	0.38992
P0C214	Q9NR09	0.36631
P0C214	Q8IYE1	0.053963
P0C214	O15265	1.0319
P0C214	Q96PQ6	0.48657
P0C214	Q7Z406	0.69922
P0C214	Q9UGK8	0.6542
P0C214	O43290	1.0049
P0C214	Q7RTT3	0.17331
P0C214	Q16143	0.3927
P0C214	Q9ULE0	0.091717
P0C214	Q6ZR93	0.086276
P0C214	A1L4L8	0.4125
P0C214	Q9UI09	0.070267
P0C214	A1L443	0.72088
P0C214	Q9UFF9	0.66635
P0C214	P35268	0.88053
P0C214	O15305	0.26421
P0C214	Q12888	0.67522
P0C214	Q9NSD4	0.31357
P0C214	Q9BYJ9	0.55796
P0C214	Q9UJX3	0.75025
P0C214	Q86VY4	0.50415
P0C214	P23511	1.0642
P0C214	Q9UKY7	0.56233
P0C214	Q7Z6Z7	0.43158
P0C214	Q8N4J0	0.50031
P0C214	O75113	0.85053
P0C214	Q68CZ6	0.37012
P0C214	Q7RTS7	0.26387
P0C214	Q8NHY6	0.39171
P0C214	Q8IW36	0.46372
P0C214	Q9NP97	0.39635
P0C214	P45984	0.35917
P0C214	Q9HCK5	0.77466
P0C214	Q8N7J2	0.89019
P0C214	Q14684	0.4878
P0C214	Q8WWY6	0.39389
P0C214	Q8TF01	0.53941
P0C214	Q5JSJ4	1.0959
P0C214	Q9ULW3	0.83291
P0C214	Q3LIE5	0.52579
P0C214	Q8N8K9	0.06839
P0C214	Q14585	0.2817
P0C214	Q5T160	1.0723
P0C214	P80294	0.66345
P0C214	Q96DZ1	0.27989
P0C214	Q6Y7W6	0.48252
P0C214	Q8NC51	0.72853
P0C214	A6NIH7	0.27271
P0C214	Q5BJF6	0.3726
P0C214	P78549	0.27508
P0C214	Q8N7B1	0.49032
P0C214	Q03111	1.2388
P0C214	Q99717	0.4478
P0C214	P47897	0.4361
P0C214	O60664	0.39961
P0C214	P49593	0.28002
P0C214	Q86WV6	0.074115
P0C214	Q4KN68	0.24284
P0C214	O14978	0.37861
P0C214	P56182	0.75131
P0C214	O75956	0.11463
P0C214	P15036	0.6608
P0C214	P17987	0.26979
P0C214	Q9BYR4	0.43378
P0C214	P16415	0.35861
P0C214	Q6ZQV5	0.3804
P0C214	Q9HC62	0.39405
P0C214	Q8TAB7	0.51977

33897	P0C214	Q9UIE0	0.28997
33898	P0C214	Q2M218	0.3048
33899	P0C214	Q9UHB9	0.51262
33900	P0C214	Q0D2K3	0.6528
33901	P0C214	O95155	0.27231
33902	P0C214	Q711Q0	0.086276
33903	P0C214	Q5T1B1	0.866
33904	P0C214	Q9UI56	0.086276
33905	P0C214	Q969I3	0.52792
33906	P0C214	Q8IXS2	0.047977
33907	P0C214	Q9BW92	0.57851
33908	P0C214	A6NK58	0.28219
33909	P0C214	Q9NR48	0.26481
33910	P0C214	Q8N0Z3	0.41835
33911	P0C214	Q8N1G2	0.33067
33912	P0C214	Q53FN2	0.37462
33913	P0C214	Q8WWK9	0.22913
33914	P0C214	O15144	0.1299
33915	P0C214	Q9HCE5	0.18808
33916	P0C214	P78560	0.52187
33917	P0C214	A8MVJ9	0.30033
33918	P0C214	Q96AJ1	1.0229
33919	P0C214	Q9BW60	0.9999
33920	P0C214	Q8N6Y0	0.5159
33921	P0C214	Q5T1N1	0.68629
33922	P0C214	P62424	1.203
33923	P0C214	P29372	0.38038
33924	P0C214	P52434	0.27719
33925	P0C214	O43325	0.96582
33926	P0C214	Q08945	1.1115
33927	P0C214	P46013	0.14105
33928	P0C214	Q969V6	1.4006
33929	P0C214	Q8IXH7	1.1808
33930	P0C214	Q6PI48	0.79471
33931	P0C214	O14862	0.56155
33932	P0C214	Q8N7K0	0.3804
33933	P0C214	Q04864	1.2118
33934	P0C214	Q9HCG1	0.38886
33935	P0C214	P80723	0.1826
33936	P0C214	Q8WXE9	0.48082
33937	P0C214	P51861	0.10702
33938	P0C214	Q08211	0.32001
33939	P0C214	Q9NSP4	0.13336
33940	P0C214	Q6PD74	0.19238
33941	P0C214	Q9NZV7	0.57377
33942	P0C214	O95905	0.75523
33943	P0C214	P61966	0.26698
33944	P0C214	Q9NS26	0.43141
33945	P0C214	Q8IYN2	1.1271
33946	P0C214	P04731	0.39612
33947	P0C214	Q5TEC3	0.31024
33948	P0C214	O60293	0.080137
33949	P0C214	O14972	0.33957
33950	P0C214	Q9BZF9	0.67138
33951	P0C214	Q8IY63	0.47091
33952	P0C214	Q8IYW4	0.15062
33953	P0C214	P30419	0.34909
33954	P0C214	P60866	0.88422
33955	P0C214	A6NI87	0.50529
33956	P0C214	P40763	0.32555
33957	P0C214	P0CZ20	0.16767
33958	P0C214	O00401	0.73048
33959	P0C214	Q9H9D4	0.51321
33960	P0C214	A8MUZ8	0.49972
33961	P0C214	O60260	0.58967
33962	P0C214	Q8WZ33	0.82382
33963	P0C214	Q8WU39	0.24933
33964	P0C214	Q9UQ84	0.21716
33965	P0C214	Q8WVM0	0.57741
33966	P0C214	Q9H2W6	0.29728
33967	P0C214	O60504	0.62137
33968	P0C214	Q0VGE8	0.25018
33969	P0C214	Q96IP4	0.52367
33970	P0C214	O96004	0.37455
33971	P0C214	Q12972	0.80624
33972	P0C214	Q8WYA6	0.67674

P0C214	Q9H668	0.19446
P0C214	C9JBD0	0.54414
P0C214	Q6VN20	0.42327
P0C214	Q9Y5A7	0.71298
P0C214	Q96BT7	0.46992
P0C214	Q9UIV8	0.72749
P0C214	Q5TG92	0.1367
P0C214	Q86SQ7	0.92082
P0C214	O76083	0.25757
P0C214	P13533	0.75898
P0C214	Q9H5J8	0.90001
P0C214	Q9BYC8	0.87415
P0C214	Q8N6M0	0.26013
P0C214	Q08ER8	0.36913
P0C214	O00472	0.55977
P0C214	O95218	0.51588
P0C214	O95190	0.77645
P0C214	Q7RTT6	0.22639
P0C214	Q8N4Q1	0.041166
P0C214	P62857	0.70631
P0C214	Q9Y675	0.39389
P0C214	Q9UHJ3	0.28639
P0C214	Q9NWB6	0.39389
P0C214	Q7RTU0	0.25688
P0C214	P27540	0.35413
P0C214	P49910	0.65862
P0C214	Q8IYB7	0.076374
P0C214	A2A368	0.019596
P0C214	Q5VT06	0.50552
P0C214	Q9UJA5	0.86333
P0C214	P09496	0.33182
P0C214	Q9BR84	0.49186
P0C214	Q9GZN7	0.21691
P0C214	Q86V15	0.37778
P0C214	Q8WWM7	0.62701
P0C214	O95997	0.90513
P0C214	P52292	1.001
P0C214	Q9H2K0	0.3468
P0C214	Q8N0V3	0.56001
P0C214	Q15274	0.097524
P0C214	P12270	0.6247
P0C214	O00743	0.85609
P0C214	Q14602	0.27801
P0C214	Q9NWZ5	0.12733
P0C214	Q5MJ70	0.28575
P0C214	Q9NRH1	0.24733
P0C214	Q8TB69	0.44782
P0C214	O60884	0.96549
P0C214	Q9NVN8	0.46564
P0C214	Q96GD0	0.13483
P0C214	P50221	0.47943
P0C214	Q6GYQ0	0.20884
P0C214	Q8NA03	0.14126
P0C214	P27694	0.4952
P0C214	Q99543	0.58019
P0C214	Q9NUW8	0.6285
P0C214	Q8NDT2	0.93649
P0C214	Q9NXR7	0.31845
P0C214	Q9HA58	0.086276
P0C214	Q9H0B3	0.30112
P0C214	Q68DK2	0.66508
P0C214	Q7L8J4	0.14927
P0C214	O75971	0.84223
P0C214	Q6KB66	0.23837
P0C214	Q9UK73	0.73148
P0C214	Q5D0E6	1.0107
P0C214	A6NKD9	0.94604
P0C214	O15069	0.20112
P0C214	Q8WXF5	0.49301
P0C214	P52758	0.53837
P0C214	Q71F23	0.67257
P0C214	Q96N03	0.2675
P0C214	Q9NPJ3	0.062823
P0C214	O95848	0.43378
P0C214	Q9NWV4	0.61671
P0C214	Q9H707	0.28531

P0C214	Q92600	1.3113
P0C214	Q9HD40	0.56404
P0C214	Q6P1R3	0.59757
P0C214	Q96N38	0.47128
P0C214	Q9H8V8	0.086276
P0C214	Q9HBT7	0.34442
P0C214	Q68CP9	0.44846
P0C214	Q8WWL7	0.8534
P0C214	Q9HAW0	0.40164
P0C214	Q9UPW6	0.58435
P0C214	P08151	0.32768
P0C214	Q9BTA9	0.88855
P0C214	P16118	0.25574
P0C214	Q92804	0.19205
P0C214	Q96SY0	0.020266
P0C214	A4D2B0	0.41379
P0C214	Q96B49	0.30985
P0C214	Q9UFB7	0.27102
P0C214	Q5U5X0	0.60065
P0C214	P07197	0.60517
P0C214	Q6P1N0	0.14168
P0C214	O15040	0.2675
P0C214	Q9UKV3	0.70183
P0C214	Q13435	0.73695
P0C214	Q92614	0.055196
P0C214	Q8IXS0	0.24937
P0C214	Q9NYJ1	1.0119
P0C214	O14727	0.29522
P0C214	O94916	0.12024
P0C214	P23508	0.32816
P0C214	Q3B820	0.30618
P0C214	Q9BRJ7	0.50589
P0C214	Q712K3	0.82252
P0C214	Q9HA90	0.24937
P0C214	Q96H20	0.93314
P0C214	Q86X55	0.76531
P0C214	Q14974	1.0028
P0C214	Q00839	0.34975
P0C214	P50570	0.33082
P0C214	P49458	0.69236
P0C214	O00625	0.63383
P0C214	Q7Z3Z0	0.58756
P0C214	O75688	0.5004
P0C214	Q9H0I3	0.39064
P0C214	P54278	0.32562
P0C214	A6NM28	0.21148
P0C214	Q8NA57	0.22683
P0C214	P48380	0.33881
P0C214	Q96A56	0.99489
P0C214	Q86V48	0.60038
P0C214	Q5T1C6	0.67768
P0C214	Q9UIC8	0.30552
P0C214	Q16698	0.4198
P0C214	Q9ULU4	0.41257
P0C214	Q52LA3	0.21564
P0C214	Q9H008	0.62309
P0C214	Q96PV6	0.66871
P0C214	Q5TKA1	0.32511
P0C214	O95782	0.42098
P0C214	Q93083	0.39612
P0C214	Q9BV90	0.054611
P0C214	P09234	0.50737
P0C214	P35998	0.46218
P0C214	P60900	0.54202
P0C214	Q9HD34	0.72382
P0C214	Q86UD1	0.16266
P0C214	Q569K6	0.14592
P0C214	A5D8V6	0.12906
P0C214	Q53T94	0.35578
P0C214	P78545	0.70536
P0C214	P61313	1.2375
P0C214	Q15181	0.5141
P0C214	Q99684	0.30684
P0C214	Q9Y623	0.89031
P0C214	P62256	0.73939
P0C214	P08708	0.63412

P0C214	Q5T215	0.29512
P0C214	Q6ZNR8	0.086276
P0C214	A6NDD8	0.51029
P0C214	Q6H8Q1	0.30057
P0C214	P04424	0.019079
P0C214	Q5H9L4	0.20645
P0C214	Q9Y4L5	0.52003
P0C214	Q05084	0.39934
P0C214	Q9H3P2	1.0297
P0C214	P50549	0.84925
P0C214	Q8IY92	0.34028
P0C214	Q9HCL3	0.49249
P0C214	Q9ULH7	0.84471
P0C214	Q08752	0.38976
P0C214	O14770	0.2773
P0C214	Q6NUN9	0.68141
P0C214	Q9NWS1	0.87145
P0C214	A6NNV3	0.28575
P0C214	Q9P243	0.38293
P0C214	Q9NVD7	0.24338
P0C214	Q92988	0.35875
P0C214	E9PI22	0.26625
P0C214	Q86VX2	0.55172
P0C214	O60645	0.27848
P0C214	Q96LX7	0.13616
P0C214	Q96MU7	0.6466
P0C214	C9JC47	0.086276
P0C214	Q9P015	0.36099
P0C214	Q9H320	0.58589
P0C214	P22392	0.36166
P0C214	Q9Y5P8	0.61346
P0C214	Q9P0U1	0.026927
P0C214	Q9P2D3	0.84633
P0C214	Q9BSN8	0.46879
P0C214	P52594	0.39215
P0C214	P41162	0.20115
P0C214	Q92945	0.87102
P0C214	Q96LS8	0.039873
P0C214	Q8NAJ2	0.028151
P0C214	Q8NEP4	0.24937
P0C214	Q14011	0.27112
P0C214	Q9NNW5	0.3899
P0C214	Q9H2T7	0.45878
P0C214	Q6NUN7	0.086276
P0C214	Q13342	0.52812
P0C214	Q9UN37	0.45057
P0C214	Q13278	0.29289
P0C214	Q92575	0.95616
P0C214	Q9Y3A3	0.40733
P0C214	Q9Y3D0	0.92139
P0C214	Q8N865	0.17987
P0C214	Q92547	0.40837
P0C214	P60174	0.50715
P0C214	Q7Z2X7	0.23416
P0C214	Q9P287	0.59087
P0C214	Q9BZE4	0.36797
P0C214	O95613	0.66877
P0C214	O75558	0.39219
P0C214	Q8IUF8	0.42793
P0C214	Q8WV24	0.57007
P0C214	Q9BUY5	0.41895
P0C214	Q86UA6	0.59002
P0C214	O00566	0.809
P0C214	P56555	0.47457
P0C214	Q8TC71	0.37432
P0C214	Q9HB65	0.54131
P0C214	Q8N3J9	0.29462
P0C214	B5ME19	0.31422
P0C214	Q9HBQ8	0.49852
P0C214	Q92636	0.24223
P0C214	P21673	0.42011
P0C214	Q9BQA5	0.7492
P0C214	P20042	0.60232
P0C214	Q9HCJ0	0.37187
P0C214	P0C5J1	0.21724
P0C214	P08651	0.18251

P0C214	Q5CZC0	0.9131
P0C214	Q96M83	0.047906
P0C214	A6NFI3	0.2567
P0C214	Q9BZG8	0.19561
P0C214	Q15776	0.43247
P0C214	P02545	0.64272
P0C214	Q13601	0.40118
P0C214	P22415	0.42816
P0C214	Q9BYN7	0.17348
P0C214	A6NEM1	0.48478
P0C214	Q8IYF1	0.24806
P0C214	Q9H9C1	0.4529
P0C214	Q9UM63	0.18441
P0C214	P0CG20	0.076114
P0C214	P19387	0.64095
P0C214	Q96EK6	0.36964
P0C214	Q8N782	0.43239
P0C214	Q9NP87	0.16513
P0C214	Q9UQ80	1.0425
P0C214	Q16612	0.22832
P0C214	A2RTY3	0.30773
P0C214	P05387	0.86977
P0C214	P50991	0.40261
P0C214	Q86WG3	0.25974
P0C214	O14646	0.63859
P0C214	P25398	0.90921
P0C214	Q9P1Z2	0.71131
P0C214	Q6IN84	0.23997
P0C214	Q99996	0.54452
P0C214	Q9H3F6	0.52568
P0C214	Q96AN2	0.095412
P0C214	Q96QD9	0.65643
P0C214	Q96H12	0.60135
P0C214	Q9NRC8	1.1932
P0C214	Q13148	0.53171
P0C214	Q9UBB5	1.0442
P0C214	Q96LB3	0.52515
P0C214	Q8NFH4	0.97357
P0C214	P22626	0.58362
P0C214	P10828	0.35304
P0C214	Q7RTV3	0.33161
P0C214	Q8NHQ8	0.74321
P0C214	Q5H9J7	0.85403
P0C214	P23193	1.1525
P0C214	Q9HAI6	0.47018
P0C214	Q8N1W2	0.21165
P0C214	Q70EL1	0.38153
P0C214	P35520	0.25143
P0C214	Q15596	0.62658
P0C214	Q6ZVL8	0.086276
P0C214	Q9H7R0	0.43344
P0C214	Q13503	0.62916
P0C214	Q8WYH8	0.45393
P0C214	Q15797	0.21031
P0C214	Q5T7W7	0.24937
P0C214	O43708	0.67107
P0C214	Q5T280	1.1308
P0C214	O00233	0.74614
P0C214	Q86XK3	0.61065
P0C214	O75791	1.0687
P0C214	Q9UM11	1.1668
P0C214	Q8IXY8	0.69592
P0C214	P19388	0.57589
P0C214	Q96FQ6	0.41816
P0C214	Q9BVL2	0.63318
P0C214	Q8IYL3	0.53881
P0C214	Q9NY74	0.64942
P0C214	P52736	0.54639
P0C214	O15527	0.49974
P0C214	B3KS81	0.24287
P0C214	A8K554	0.04885
P0C214	Q9H0W8	0.48666
P0C214	Q8N4C6	0.38694
P0C214	Q6ZRM9	0.36484
P0C214	Q6L8G5	0.086276
P0C214	Q8N3V7	0.47869

P0C214	Q9UBQ5	1.1694
P0C214	Q8NDZ0	0.13374
P0C214	Q9H2C0	0.55562
P0C214	P67870	0.31549
P0C214	O95644	0.81365
P0C214	Q96P15	0.78972
P0C214	Q13409	0.39224
P0C214	Q8NFH8	0.35117
P0C214	Q15543	0.91711
P0C214	Q9Y651	0.24449
P0C214	Q8TD35	0.24937
P0C214	Q8IVE0	0.075175
P0C214	Q8WUI4	0.31744
P0C214	Q5C9Z4	0.68187
P0C214	Q6QNY1	0.66538
P0C214	P07738	1.1711
P0C214	Q8TA86	0.59053
P0C214	P40692	0.056254
P0C214	Q9BQY9	0.50131
P0C214	P51668	0.81042
P0C214	P16220	0.83463
P0C214	C9JLW8	0.60203
P0C214	Q5TD94	0.2222
P0C214	O43251	0.39696
P0C214	Q13360	0.2224
P0C214	Q96NJ6	0.25699
P0C214	Q13569	0.42904
P0C214	O60869	0.5756
P0C214	Q5VWI1	1.0442
P0C214	Q8NFW8	0.11525
P0C214	P36551	0.21379
P0C214	P14373	0.28034
P0C214	O14757	0.45674
P0C214	O14512	0.50347
P0C214	Q92783	0.53195
P0C214	Q9H4D5	0.61202
P0C214	Q8IV33	0.052525
P0C214	Q8TDP1	0.50411
P0C214	O95948	0.26001
P0C214	Q13228	1.1452
P0C214	Q9NSD9	0.86786
P0C214	Q9P2K3	1.1097
P0C214	Q9UJG1	0.74174
P0C214	Q99627	0.38964
P0C214	O94763	0.82016
P0C214	P35232	0.47377
P0C214	Q8WTS6	0.45773
P0C214	Q3KQV3	0.30056
P0C214	Q9H5V9	0.21939
P0C214	Q14146	0.16769
P0C214	P23610	0.086276
P0C214	Q9NRN7	0.62429
P0C214	Q9BYZ2	1.0002
P0C214	Q8NB25	0.22041
P0C214	Q9Y605	1.32
P0C214	H0Y7S4	0.17424
P0C214	Q9NQ92	0.87307
P0C214	O43633	0.30631
P0C214	O00463	0.29081
P0C214	P26640	0.27612
P0C214	Q9BZE1	0.31815
P0C214	P17017	0.26437
P0C214	Q8NHZ7	0.42069
P0C214	Q96LI5	0.70614
P0C214	A6NHX0	0.63857
P0C214	Q9NQR1	0.58974
P0C214	Q92466	0.59924
P0C214	O95391	0.12811
P0C214	Q9P219	0.24212
P0C214	O75674	0.32935
P0C214	Q8N0S6	0.10605
P0C214	P55210	0.24819
P0C214	Q08AG7	0.55226
P0C214	Q96FN9	0.47161
P0C214	O14628	0.44727
P0C214	Q9Y3B7	1.183

34353	P0C214	Q9BQE4	0.3807
34354	P0C214	Q9H9H4	0.65641
34355	P0C214	P53805	0.53845
34356	P0C214	Q9Y3Y4	0.53429
34357	P0C214	Q9BV97	0.31995
34358	P0C214	Q9H974	1.3718
34359	P0C214	P46108	0.50771
34360	P0C214	Q9NXR1	0.2821
34361	P0C214	Q5F1R6	0.14055
34362	P0C214	Q5VWM5	0.81925
34363	P0C214	P05114	0.3519
34364	P0C214	Q15046	0.85313
34365	P0C214	Q9BWK5	0.41881
34366	P0C214	Q8NFD5	0.82876
34367	P0C214	P17812	0.3312
34368	P0C214	Q9H0C8	0.6432
34369	P0C214	P69891	0.3641
34370	P0C214	Q5HYI7	0.95494
34371	P0C214	P30047	0.76042
34372	P0C214	Q3ZCT1	0.25449
34373	P0C214	Q9Y3R5	0.55431
34374	P0C214	Q6L8Q7	0.35958
34375	P0C214	Q9NQP4	1.1153
34376	P0C214	Q9H3K6	0.38958
34377	P0C214	O95271	0.4807
34378	P0C214	Q15293	0.30055
34379	P0C214	Q92800	0.45887
34380	P0C214	Q9NVV2	0.086276
34381	P0C214	Q96C74	0.23585
34382	P0C214	Q86Y01	0.68057
34383	P0C214	Q5JQS6	0.11688
34384	P0C214	Q5T3J3	0.98876
34385	P0C214	Q8NFV5	0.28575
34386	P0C214	Q8NA42	0.32046
34387	P0C214	Q7LG56	0.37894
34388	P0C214	A6NKF2	0.57314
34389	P0C214	P22830	0.082794
34390	P0C214	Q86YF9	0.15972
34391	P0C214	Q15562	0.75596
34392	P0C214	Q6FI81	0.36676
34393	P0C214	Q9Y606	1.2348
34394	P0C214	Q96G28	0.26852
34395	P0C214	F2Z3M2	0.086276
34396	P0C214	Q8TBY8	0.66758
34397	P0C214	Q99638	0.53935
34398	P0C214	Q96H86	0.29809
34399	P0C214	Q9BZL1	0.39346
34400	P0C214	Q9BYS1	0.12997
34401	P0C214	Q8N9H9	0.086276
34402	P0C214	Q8N159	0.2515
34403	P0C214	Q6P047	0.26803
34404	P0C214	O60315	0.2406
34405	P0C214	P0C7X3	0.32642
34406	P0C214	Q5SWL8	0.27649
34407	P0C214	Q9BWX1	0.22491
34408	P0C214	Q5VWQ0	1.026
34409	P0C214	P48454	0.16926
34410	P0C214	Q9Y4X5	0.56995
34411	P0C214	P61927	0.88621
34412	P0C214	Q96G25	0.98533
34413	P0C214	Q8N895	0.418
34414	P0C214	Q8N3F8	0.30669
34415	P0C214	P31946	0.61588
34416	P0C214	Q02040	0.23584
34417	P0C214	Q9Y3D2	0.21251
34418	P0C214	P62750	1.0892
34419	P0C214	Q9Y383	0.6544
34420	P0C214	Q63HQ0	0.092351
34421	P0C214	Q7Z3Y9	0.58756
34422	P0C214	O95521	0.30244
34423	P0C214	Q14568	0.99507
34424	P0C214	Q96IY1	0.95151
34425	P0C214	A6NGY1	0.73438
34426	P0C214	Q9ULK4	1.1657
34427	P0C214	O15370	0.35159
34428	P0C214	P51531	0.60383

P0C214	Q8N5U0	0.086276
P0C214	P62979	0.49628
P0C214	Q9NR28	0.88425
P0C214	A4D0V7	0.53574
P0C214	Q07157	0.61324
P0C214	Q14CZ0	1.3453
P0C214	Q32M78	0.44702
P0C214	O94818	0.73154
P0C214	Q5T5F5	0.086276
P0C214	P52630	0.801
P0C214	P82673	0.060152
P0C214	P59942	0.326
P0C214	O95561	0.70381
P0C214	Q5VTR2	0.76361
P0C214	Q16762	0.28129
P0C214	Q96AQ7	0.65368
P0C214	O94805	0.66732
P0C214	Q9UBW8	0.38964
P0C214	Q96S07	0.086276
P0C214	Q9UI72	0.086276
P0C214	Q8N5N4	0.23926
P0C214	Q7Z6K1	0.59482
P0C214	Q96K17	0.53349
P0C214	Q19AV6	1.129
P0C214	Q6ZPA2	0.086276
P0C214	P42768	0.3659
P0C214	Q5VXH4	0.402
P0C214	Q5VWX1	0.67813
P0C214	Q8NB12	0.21654
P0C214	Q3BBV1	1.2293
P0C214	P30260	1.1311
P0C214	Q8TDB6	0.42826
P0C214	Q6PI77	0.38622
P0C214	Q9BRQ6	1.0674
P0C214	P05386	1.371
P0C214	P49454	0.52394
P0C214	Q14061	0.51617
P0C214	Q06265	0.26578
P0C214	P48163	0.23099
P0C214	Q8NDY4	0.086276
P0C214	Q6ZN32	0.18216
P0C214	Q96JE7	0.95654
P0C214	P21912	0.47081
P0C214	Q8IUD2	0.64885
P0C214	Q9ULG3	0.086276
P0C214	Q5VZK9	0.30279
P0C214	P09132	0.52578
P0C214	P0CJ79	0.31307
P0C214	Q9UNP9	0.41667
P0C214	Q96NG8	0.30833
P0C214	Q6ZQN3	0.5165
P0C214	Q5JRC9	0.26194
P0C214	Q01546	0.48019
P0C214	P21399	0.47054
P0C214	Q14657	1.2574
P0C214	Q14106	0.35581
P0C214	P53680	0.15238
P0C214	Q9UPN7	0.94164
P0C214	Q9Y678	0.5134
P0C214	P08107	0.7759
P0C214	P54845	0.15357
P0C214	Q04837	0.46138
P0C214	Q9Y3A5	0.59568
P0C214	Q96JC9	0.95714
P0C214	Q15326	1.139
P0C214	Q5EBM0	0.64097
P0C214	Q9UQR1	0.69685
P0C214	Q6P3V2	0.21705
P0C214	A6NN90	0.041555
P0C214	P62851	1.0316
P0C214	Q8IYW5	0.25447
P0C214	Q8IX15	0.39856
P0C214	Q8TCE6	0.96481
P0C214	Q6ZSB9	0.25987
P0C214	Q9Y2W1	0.83275
P0C214	Q8TAF5	0.086276

P0C214	Q13123	0.52798
P0C214	Q96RE7	0.97825
P0C214	Q5D1E8	0.44924
P0C214	Q3MIV0	0.086276
P0C214	Q92748	1.116
P0C214	Q96EB1	1.0872
P0C214	O15499	0.080357
P0C214	Q8NDA8	0.291
P0C214	Q969R5	0.094082
P0C214	P10515	0.55021
P0C214	Q86U42	0.74503
P0C214	Q9UBK9	0.91012
P0C214	Q99733	0.79371
P0C214	Q9NVX0	0.982
P0C214	Q9P270	0.36609
P0C214	P63261	0.10604
P0C214	Q6AZW8	0.28423
P0C214	P61970	0.41599
P0C214	P0C852	0.086276
P0C214	Q9UL03	0.65124
P0C214	Q8IUB9	0.086276
P0C214	Q96EZ8	0.66561
P0C214	Q9H902	0.074155
P0C214	P19013	0.48019
P0C214	P50452	0.96136
P0C214	Q8TCJ0	1.0171
P0C214	Q14590	0.27328
P0C214	Q9H5F2	0.31831
P0C214	Q5SRE5	0.59971
P0C214	Q1ED39	0.73154
P0C214	P40222	0.50478
P0C214	Q9NVV4	0.66801
P0C214	Q6P582	0.63949
P0C214	Q9NWZ8	0.79329
P0C214	P06730	0.6776
P0C214	O43255	0.47682
P0C214	P19338	0.51964
P0C214	Q5ST30	0.29762
P0C214	P0CE67	0.086276
P0C214	Q9NT62	0.51256
P0C214	Q9NZL3	0.60856
P0C214	P18754	1.0027
P0C214	P14672	0.57271
P0C214	Q8N660	1.2293
P0C214	Q96JJ3	0.21071
P0C214	Q96Q83	0.61861
P0C214	O60308	0.56639
P0C214	P20073	0.52658
P0C214	Q9Y5Y2	0.17775
P0C214	Q9UJ78	0.2378
P0C214	Q96JB2	0.71187
P0C214	Q9NTI5	0.73965
P0C214	Q6X4T0	0.23053
P0C214	Q86XF7	0.59266
P0C214	Q9UNE7	0.40852
P0C214	Q9BYQ6	0.43378
P0C214	Q9NQ87	0.37412
P0C214	Q9BYG3	0.27723
P0C214	A1L170	0.086276
P0C214	O43236	0.33347
P0C214	P98171	0.33185
P0C214	Q9Y618	1.0048
P0C214	Q8IUC3	0.52687
P0C214	Q96CA5	0.28393
P0C214	O15524	0.23763
P0C214	Q15185	0.65159
P0C214	Q13838	0.18558
P0C214	Q8NFF5	0.35668
P0C214	Q6P9B6	0.2675
P0C214	Q7L1V2	0.85823
P0C214	P62508	0.33715
P0C214	Q8IZT6	0.27936
P0C214	Q9NRG4	0.74098
P0C214	Q5TI25	1.2293
P0C214	Q9P032	0.49352
P0C214	Q9Y448	0.70423

P0C214	Q8IZ73	0.22666
P0C214	O76064	0.29751
P0C214	A8MYZ0	0.29668
P0C214	P30304	0.66014
P0C214	Q96FZ2	0.98722
P0C214	Q96EK4	1.2067
P0C214	Q96EG3	0.21487
P0C214	A6NN06	0.086276
P0C214	P33552	0.52759
P0C214	Q8WUH2	0.58328
P0C214	Q9H963	0.43256
P0C214	Q9UBI1	0.2675
P0C214	P50995	0.7626
P0C214	Q5SNV9	0.24382
P0C214	Q92782	0.55057
P0C214	Q96BY7	0.24463
P0C214	Q9NPD8	0.80175
P0C214	Q01664	0.45251
P0C214	P56282	0.3173
P0C214	Q8N3R3	0.23672
P0C214	Q96Q35	0.42134
P0C214	Q13561	0.70344
P0C214	Q99704	0.16228
P0C214	Q9H7N4	0.76033
P0C214	Q5TB80	0.74228
P0C214	Q9UPN9	0.47305
P0C214	P27986	0.47638
P0C214	Q15365	0.91306
P0C214	Q6ZS49	0.086276
P0C214	Q53HM4	0.37861
P0C214	Q6PD62	0.56023
P0C214	Q8TB03	0.29443
P0C214	O43765	0.58409
P0C214	Q8WYQ3	0.37228
P0C214	Q8N7R1	0.56659
P0C214	O75177	0.68232
P0C214	Q8IWL8	0.59004
P0C214	O95373	0.91195
P0C214	Q14498	0.77553
P0C214	Q5T871	0.086276
P0C214	Q9NY87	0.45586
P0C214	A8MXZ3	0.086276
P0C214	P46782	0.71074
P0C214	Q9UI66	0.086276
P0C214	P43490	0.20422
P0C214	O75175	1.5103
P0C214	Q8WV07	0.44053
P0C214	Q9UJY1	0.44566
P0C214	Q9NP66	0.91644
P0C214	Q9BS31	0.1962
P0C214	Q5EBL2	0.24669
P0C214	Q8WYQ5	0.7018
P0C214	Q9C0E2	0.77357
P0C214	O43790	0.53378
P0C214	Q17RY6	0.56722
P0C214	Q96PE7	0.22382
P0C214	Q86WR7	0.33967
P0C214	Q569G3	0.24937
P0C214	Q96CB9	0.82406
P0C214	A8MUL3	0.086276
P0C214	P59282	0.36817
P0C214	Q8IYF3	0.36978
P0C214	Q9BRP4	0.39882
P0C214	Q6NSH3	0.55315
P0C214	P39687	1.4878
P0C214	Q92530	0.79407
P0C214	Q6L8H4	0.086276
P0C214	P26678	0.19666
P0C214	Q9UL33	0.41975
P0C214	Q8IZ63	0.086276
P0C214	Q9ULC4	0.38118
P0C214	Q8IYI6	0.436
P0C214	Q8NE65	0.42468
P0C214	Q5CZA5	0.36913
P0C214	P61081	0.043552
P0C214	O95163	0.61101

P0C214	Q9GZY8	0.20718
P0C214	Q96QF0	0.53902
P0C214	P15822	0.76081
P0C214	Q96HA7	0.71339
P0C214	Q96LX8	0.28195
P0C214	P07320	0.68261
P0C214	Q9NTK5	0.75098
P0C214	Q6A163	0.5102
P0C214	P46020	0.28008
P0C214	Q3LI59	0.086276
P0C214	Q5VVM6	0.14036
P0C214	Q9NYF0	0.19829
P0C214	Q5JRK9	0.80497
P0C214	Q9UJT9	0.34612
P0C214	P78346	0.71492
P0C214	P56537	1.155
P0C214	Q7L190	0.73259
P0C214	Q9UBC9	0.38624
P0C214	O60284	0.030112
P0C214	Q8NG50	0.20723
P0C214	Q9H3K2	0.035737
P0C214	Q9NQ33	0.44481
P0C214	Q9C0D2	0.50735
P0C214	Q8WV37	0.48694
P0C214	Q8N9G6	0.66203
P0C214	Q13287	0.54139
P0C214	Q8N1K5	0.17664
P0C214	Q15699	0.28048
P0C214	Q9UN36	0.57869
P0C214	Q96GS4	0.77019
P0C214	P52746	0.13336
P0C214	Q96CM3	0.57601
P0C214	Q9H0M5	0.27328
P0C214	O15042	0.32876
P0C214	O00757	0.29035
P0C214	Q5HYK3	0.22257
P0C214	Q9H6Z4	1.0268
P0C214	P14859	1.0484
P0C214	Q9NSJ1	0.33466
P0C214	Q7L5Y9	0.25486
P0C214	Q7Z3B4	0.79585
P0C214	Q9UK12	0.29235
P0C214	Q9Y6E7	0.53395
P0C214	Q9H492	0.19282
P0C214	Q9Y2T3	0.21315
P0C214	Q96LR5	0.82252
P0C214	Q9ULV3	0.23177
P0C214	P13807	0.63099
P0C214	P31939	0.46332
P0C214	Q96ES7	0.085513
P0C214	Q9H693	0.72474
P0C214	Q9NYG5	0.80367
P0C214	Q96M29	0.41792
P0C214	Q5JSZ5	0.63719
P0C214	Q8WW01	0.24525
P0C214	Q96E14	0.45974
P0C214	Q5JTC6	0.69549
P0C214	Q96N20	0.2932
P0C214	A6NKU9	0.28575
P0C214	H3BNL8	1.014
P0C214	Q5TID7	0.3716
P0C214	Q9Y3Y2	0.85151
P0C214	P82933	0.64427
P0C214	Q9UMX1	0.6684
P0C214	Q16531	0.58143
P0C214	Q9H7T9	0.55989
P0C214	Q9Y692	0.67311
P0C214	P15559	0.40127
P0C214	Q8ND83	0.81906
P0C214	O14920	0.45388
P0C214	Q15008	0.20541
P0C214	O15304	0.94115
P0C214	Q9BXS5	0.72815
P0C214	P23297	0.47678
P0C214	O00161	0.73139
P0C214	P56279	0.62665

P0C214	Q8WXB4	0.3338
P0C214	Q99583	0.24468
P0C214	Q9ULR5	0.41189
P0C214	A6NK89	0.5294
P0C214	Q9UBC1	0.43978
P0C214	Q6P1W5	0.27899
P0C214	Q86X95	1.0371
P0C214	Q9Y253	0.21032
P0C214	P18124	0.78028
P0C214	Q5VWM1	0.36894
P0C214	Q14209	0.87576
P0C214	Q93062	0.83774
P0C214	Q9H3M9	0.35519
P0C214	Q9UHD9	0.77198
P0C214	Q9Y2P7	0.3005
P0C214	Q9ULD9	1.0498
P0C214	Q6ZVQ6	1.0379
P0C214	P86478	0.086276
P0C214	P59047	0.42985
P0C214	O75368	0.16541
P0C214	Q9NQT4	0.66135
P0C214	P04406	0.66395
P0C214	O75323	0.6159
P0C214	Q14CS0	0.50612
P0C214	Q3LI68	0.78534
P0C214	Q92837	0.77586
P0C214	Q01860	0.6334
P0C214	Q96BZ8	0.24937
P0C214	Q9Y2L8	0.52865
P0C214	O60216	0.62671
P0C214	Q2M3X9	0.3048
P0C214	P26371	0.086276
P0C214	Q86WQ0	0.46466
P0C214	P56181	0.40343
P0C214	Q6UVJ0	0.63845
P0C214	Q86UW9	0.89507
P0C214	Q86U38	0.23018
P0C214	Q8WVV9	0.15201
P0C214	Q6ZQT7	0.086276
P0C214	P82650	0.19627
P0C214	P57076	0.23809
P0C214	Q9NY33	0.21123
P0C214	Q6ZTY8	0.042339
P0C214	O95178	0.28096
P0C214	Q6NV74	0.24937
P0C214	Q96GM5	0.6875
P0C214	O15155	0.036955
P0C214	P49247	0.70324
P0C214	Q8NCU4	0.19057
P0C214	Q9NP77	0.22305
P0C214	Q9UMX0	0.71248
P0C214	Q6PGQ7	1.198
P0C214	Q9BSH4	0.7665
P0C214	Q66K89	0.76818
P0C214	Q96NI8	0.41788
P0C214	Q86VP6	0.72005
P0C214	A8MV24	0.33628
P0C214	O14713	0.35606
P0C214	Q9UBD5	0.46169
P0C214	Q5TGS1	0.37222
P0C214	Q8IYD8	0.27057
P0C214	P01106	0.37804
P0C214	Q96RY5	0.77018
P0C214	Q92785	0.54905
P0C214	A1A4V9	0.17641
P0C214	Q3C1V8	0.52302
P0C214	O43660	0.25592
P0C214	Q9UM13	0.73291
P0C214	Q05639	0.72864
P0C214	O15027	0.61328
P0C214	O95402	0.98938
P0C214	O75400	0.83371
P0C214	P11586	0.13354
P0C214	Q8N9W8	0.907
P0C214	Q8TC90	0.43024
P0C214	Q9NW81	0.019055

34809	P0C214	Q5T6M2	0.57802
34810	P0C214	O43361	0.25678
34811	P0C214	Q6L8G8	0.086276
34812	P0C214	Q96F86	0.78074
34813	P0C214	P00558	0.17616
34814	P0C214	O60675	0.30336
34815	P0C214	Q9HB71	0.51263
34816	P0C214	Q9BZD4	0.76692
34817	P0C214	P14316	0.94613
34818	P0C214	Q8N888	0.70115
34819	P0C214	Q9BQ89	0.85578
34820	P0C214	Q86WJ1	0.37942
34821	P0C214	Q99551	0.73714
34822	P0C214	Q9UL18	1.0786
34823	P0C214	O76014	0.5102
34824	P0C214	Q8NEM1	0.49815
34825	P0C214	A6NI79	0.78682
34826	P0C214	Q2KHR2	0.1604
34827	P0C214	O76075	0.15147
34828	P0C214	P10155	0.42326
34829	P0C214	O75081	1.039
34830	P0C214	Q5TA45	0.19207
34831	P0C214	Q70YC5	0.1894
34832	P0C214	Q92764	0.44438
34833	P0C214	O60518	0.83797
34834	P0C214	Q6ZS92	0.12973
34835	P0C214	O60248	0.39387
34836	P0C214	Q9P2J9	0.18697
34837	P0C214	Q9ULW6	0.81783
34838	P0C214	Q8WUM0	0.73135
34839	P0C214	Q03468	0.36114
34840	P0C214	O14802	0.22983
34841	P0C214	Q96EF6	0.68225
34842	P0C214	A6NK53	0.34099
34843	P0C214	Q9UBF6	0.68998
34844	P0C214	P59052	0.16928
34845	P0C214	Q9H7U1	0.77515
34846	P0C214	P35712	0.39219
34847	P0C214	O96019	0.86192
34848	P0C214	Q13495	1.0366
34849	P0C214	Q3C1V1	0.24937
34850	P0C214	O60304	0.27193
34851	P0C214	Q9ULM3	0.6861
34852	P0C214	Q8IYE0	0.4921
34853	P0C214	P07196	0.52469
34854	P0C214	Q14188	0.62021
34855	P0C214	Q9H7R5	0.38935
34856	P0C214	O95433	0.67963
34857	P0C214	Q8TEV9	0.38792
34858	P0C214	Q7LBC6	0.27492
34859	P0C214	Q9NPJ6	0.90274
34860	P0C214	Q9UH77	0.59108
34861	P0C214	Q9UQL6	0.4864
34862	P0C214	Q7L5N1	1.1297
34863	P0C214	A7E2V4	1.1075
34864	P0C214	Q9UPP1	0.44538
34865	P0C214	Q9NYJ8	0.49528
34866	P0C214	O95486	0.38305
34867	P0C214	Q13105	0.38834
34868	P0C214	P35749	0.89031
34869	P0C214	Q86VN1	0.44507
34870	P0C214	P41161	0.10416
34871	P0C214	P28070	0.57615
34872	P0C214	Q9UBB9	0.30927
34873	P0C214	P13804	0.1443
34874	P0C214	P06400	0.59903
34875	P0C214	Q4VC05	0.19839
34876	P0C214	O43583	0.8367
34877	P0C214	Q8IZU1	0.53036
34878	P0C214	Q9BXY8	0.56284
34879	P0C214	Q9BV86	0.31939
34880	P0C214	O75953	0.92441
34881	P0C214	Q92759	0.60748
34882	P0C214	Q16798	0.33328
34883	P0C214	Q5XUX0	0.35622
34884	P0C214	P35548	0.50891

34885	P0C214	Q7Z2Z1	0.76582
34886	P0C214	Q96T37	0.38304
34887	P0C214	P57055	0.55775
34888	P0C214	P08047	0.72112
34889	P0C214	O00291	0.35682
34890	P0C214	Q96B97	0.33971
34891	P0C214	P0C7I6	0.83125
34892	P0C214	Q0VDD5	0.086276
34893	P0C214	Q09FC8	0.28669
34894	P0C214	K7EJ46	0.086276
34895	P0C214	P49756	0.42896
34896	P0C214	Q14966	0.14786
34897	P0C214	A6NLJ0	0.53881
34898	P0C214	Q8IYI8	0.34605
34899	P0C214	Q96EY1	0.43074
34900	P0C214	Q9UKT4	0.85268
34901	P0C214	Q8TA94	0.47555
34902	P0C214	Q05516	0.6169
34903	P0C214	P42575	0.37079
34904	P0C214	Q9HC52	0.77904
34905	P0C214	P63146	0.91374
34906	P0C214	Q96B26	0.90296
34907	P0C214	Q7Z3K6	0.47392
34908	P0C214	P12956	0.29095
34909	P0C214	Q3LHN1	0.086276
34910	P0C214	Q8HWS3	0.21572
34911	P0C214	Q9GZS3	1.12
34912	P0C214	P11171	0.3732
34913	P0C214	O00255	0.15633
34914	P0C214	P0CI01	0.28575
34915	P0C214	Q12830	0.8393
34916	P0C214	Q8ND90	0.6469
34917	P0C214	Q96PM9	0.3338
34918	P0C214	Q9Y6D9	0.89676
34919	P0C214	P52948	0.99825
34920	P0C214	Q96DG6	0.2058
34921	P0C214	Q8N815	0.0040258
34922	P0C214	Q96JB5	0.26914
34923	P0C214	Q92870	0.64553
34924	P0C214	Q6VVB1	0.50123
34925	P0C214	Q5VTE0	0.95695
34926	P0C214	P12980	0.79946
34927	P0C214	B4DX44	0.49065
34928	P0C214	Q96NB1	0.24274
34929	P0C214	Q16629	0.61013
34930	P0C214	P42772	0.83022
34931	P0C214	Q70YC4	0.25805
34932	P0C214	P52737	0.50904
34933	P0C214	Q92621	1.0383
34934	P0C214	Q3KP66	0.76386
34935	P0C214	P31749	0.46181
34936	P0C214	Q8NCE0	0.53844
34937	P0C214	Q9BYG4	0.66658
34938	P0C214	O95447	0.11921
34939	P0C214	Q9NRY2	0.74038
34940	P0C214	Q14839	0.53135
34941	P0C214	P61581	0.37639
34942	P0C214	Q8N3X1	0.31799
34943	P0C214	Q8N2M8	0.73728
34944	P0C214	Q8TF50	0.30903
34945	P0C214	P61353	0.88548
34946	P0C214	Q9BXP5	1.017
34947	P0C214	Q1RN00	0.086276
34948	P0C214	Q63ZY3	0.80636
34949	P0C214	Q9Y2X3	0.65357
34950	P0C214	Q9H583	1.043
34951	P0C214	Q9Y3B8	0.23108
34952	P0C214	Q9BRA0	0.12649
34953	P0C214	Q9BVS5	0.32106
34954	P0C214	Q8N1L9	0.722
34955	P0C214	P61088	0.76035
34956	P0C214	Q9NR22	0.29505
34957	P0C214	Q8N8R7	0.45292
34958	P0C214	P54252	0.46048
34959	P0C214	Q701N4	0.086276
34960	P0C214	Q5JVX7	0.46482

P0C214	A6NIY4	0.28575
P0C214	Q9BYD6	0.51065
P0C214	Q96EH3	0.16728
P0C214	P14866	0.5049
P0C214	Q96N53	0.086276
P0C214	P0CG24	0.21769
P0C214	O15397	0.72169
P0C214	Q68DY9	0.28571
P0C214	O15405	0.88415
P0C214	P36578	1.3257
P0C214	Q8N8A2	0.2675
P0C214	O60384	0.50652
P0C214	A6NJL1	0.16744
P0C214	Q9NQV7	0.27381
P0C214	Q17RH7	0.086276
P0C214	Q5VZF2	0.13979
P0C214	Q13155	0.68293
P0C214	Q5JUK9	0.034609
P0C214	Q9NY43	0.31825
P0C214	O60936	0.89984
P0C214	Q8N1I8	0.086276
P0C214	Q15021	0.7874
P0C214	P30154	0.25227
P0C214	P35638	0.68749
P0C214	P20618	0.79871
P0C214	Q4G0F5	0.22528
P0C214	Q9BUV0	0.24937
P0C214	Q5HYK9	0.35008
P0C214	P13051	1.1636
P0C214	Q3LI61	0.086276
P0C214	Q8N812	0.050468
P0C214	Q9BVG4	0.30917
P0C214	Q9NV06	0.23606
P0C214	Q5TZA2	0.52811
P0C214	Q96HA8	1.0883
P0C214	Q9HAH1	0.38993
P0C214	P62328	1.0459
P0C214	Q14588	0.3804
P0C214	P29083	1.1183
P0C214	Q6EEV6	0.11408
P0C214	Q8N8E3	0.22381
P0C214	P31276	0.40585
P0C214	Q14677	0.52453
P0C214	Q2TAL8	0.8592
P0C214	O00287	0.22107
P0C214	Q33E94	0.79523
P0C214	Q06520	0.35067
P0C214	Q6ZVK8	0.43837
P0C214	Q9UGK3	0.66653
P0C214	Q0VD86	0.35391
P0C214	Q49MG5	0.79446
P0C214	Q6IPX3	0.76306
P0C214	Q9Y5R5	0.13859
P0C214	Q15466	0.46017
P0C214	Q8IUW3	0.73186
P0C214	Q96T76	0.86536
P0C214	Q9BU64	0.30428
P0C214	P07195	1.0002
P0C214	Q6L8H2	0.086276
P0C214	Q96JN8	0.26982
P0C214	O60613	0.52503
P0C214	B1ANY3	0.045761
P0C214	Q96QC0	0.85674
P0C214	Q96PU5	0.40479
P0C214	Q9BRQ4	0.039288
P0C214	P04198	0.75262
P0C214	A6NNL0	0.39603
P0C214	Q9BQ15	0.5807
P0C214	Q9H8E8	0.62823
P0C214	Q7Z3V5	0.37033
P0C214	P36957	0.21972
P0C214	P80303	0.29411
P0C214	Q66K74	0.19923
P0C214	Q8NBZ0	1.2037
P0C214	P0DI82	0.34572
P0C214	A8MTY0	0.46031

P0C214	P51553	0.39563
P0C214	Q9Y6J0	0.10946
P0C214	Q9UBU9	0.60026
P0C214	Q12769	1.0576
P0C214	Q9NW38	0.65653
P0C214	Q9UN81	0.23599
P0C214	Q9UK10	0.3804
P0C214	Q86SZ2	0.70507
P0C214	P54274	0.34341
P0C214	O95873	0.24937
P0C214	Q8TDD2	0.26644
P0C214	O00629	0.5405
P0C214	Q96EF9	0.025247
P0C214	Q6NSI8	0.72849
P0C214	Q7Z4H9	0.40259
P0C214	P0CV98	0.69286
P0C214	Q9UGL9	0.086276
P0C214	Q8N944	0.89019
P0C214	Q7Z745	0.45172
P0C214	Q5T953	0.41881
P0C214	Q16719	0.53883
P0C214	O75154	0.65705
P0C214	Q8TEB1	0.25402
P0C214	Q9H7S9	0.72848
P0C214	Q8IWV7	0.33505
P0C214	Q8IZT9	0.60323
P0C214	Q9NPF5	0.8302
P0C214	Q13432	0.3279
P0C214	Q8TAW3	0.30069
P0C214	Q9UJW9	1.4211
P0C214	Q9Y2Z4	0.32373
P0C214	P09874	0.59145
P0C214	P49721	0.57107
P0C214	Q5JNZ3	0.36568
P0C214	Q5T0F9	0.32658
P0C214	Q9NX46	0.36247
P0C214	O15015	0.56957
P0C214	Q8WTW3	0.69685
P0C214	Q6ZN79	0.42302
P0C214	Q9BR76	0.23574
P0C214	P58004	0.092801
P0C214	Q5VT28	0.033188
P0C214	Q9BRJ2	0.36156
P0C214	P17025	0.28926
P0C214	Q96CD0	0.2675
P0C214	Q96EP1	0.64336
P0C214	P51532	0.39256
P0C214	P47974	0.194
P0C214	A1A580	0.39837
P0C214	Q9HAP2	0.63034
P0C214	Q64ET8	0.47428
P0C214	Q6UN15	1.0398
P0C214	Q9BVP2	0.3075
P0C214	Q8TBK2	0.51061
P0C214	Q9BQ90	0.80878
P0C214	Q99741	0.52308
P0C214	Q9H981	1.0055
P0C214	A1A5D9	0.45563
P0C214	Q8IUC2	0.52687
P0C214	Q16401	0.80987
P0C214	Q92552	0.69067
P0C214	Q9BXV9	0.086276
P0C214	Q15937	0.31537
P0C214	Q9UQK1	0.35407
P0C214	Q8N8X9	0.18819
P0C214	A6NHS1	0.12567
P0C214	Q9Y3D3	0.69558
P0C214	P18846	0.75675
P0C214	Q86T75	0.51029
P0C214	Q8WVK7	0.54854
P0C214	O15371	0.79592
P0C214	Q7Z3C6	0.10248
P0C214	Q7Z6J9	0.7215
P0C214	Q9P2W9	0.068198
P0C214	B1AK76	0.93393
P0C214	Q5T7N2	0.25309

P0C214	Q9NYF8	1.1468
P0C214	Q9Y4K1	0.63292
P0C214	Q9NSB4	0.38419
P0C214	P0DM35	0.69409
P0C214	P30566	0.087289
P0C214	P50502	0.66138
P0C214	Q0VDF9	0.44255
P0C214	O94993	0.14039
P0C214	P86481	0.086276
P0C214	P43364	0.79434
P0C214	Q6I9Y2	0.766
P0C214	Q9NWM3	0.090278
P0C214	Q8N3Z6	0.46382
P0C214	Q8N140	0.8463
P0C214	P23760	0.67312
P0C214	P18621	0.86057
P0C214	Q9UPZ3	0.71268
P0C214	Q8NHR7	0.24424
P0C214	Q6SJ96	0.18905
P0C214	Q17RM4	0.12352
P0C214	Q8N1X5	0.086276
P0C214	Q9NTI7	0.22737
P0C214	Q13541	0.92175
P0C214	A2AJT9	0.6136
P0C214	P0C838	0.26818
P0C214	Q8IZD2	0.26786
P0C214	Q9P1U0	0.082848
P0C214	P54259	0.83533
P0C214	P10073	0.39344
P0C214	Q8N6U2	0.28966
P0C214	Q9BTP7	0.8667
P0C214	Q86XD5	0.030246
P0C214	P36404	0.24677
P0C214	Q05877	0.52681
P0C214	Q8WW59	0.24079
P0C214	P52740	0.24611
P0C214	O96020	0.96219
P0C214	Q5EBM4	0.13091
P0C214	P55072	0.28502
P0C214	Q9H857	0.62076
P0C214	P63167	1.3679
P0C214	Q13951	0.58909
P0C214	Q9H2P0	0.22077
P0C214	Q96LA8	0.68372
P0C214	Q9H5Q4	0.62028
P0C214	Q9H0E3	0.52382
P0C214	Q9H0P0	0.35109
P0C214	Q8IY57	1.1019
P0C214	Q86T29	0.25925
P0C214	P18054	0.30229
P0C214	Q9BYR6	0.6322
P0C214	Q15723	1.1579
P0C214	O75182	1.1313
P0C214	Q96DE0	0.7047
P0C214	O00471	0.22108
P0C214	Q01130	0.69396
P0C214	Q9UJC5	0.52015
P0C214	P17980	0.4214
P0C214	A5D8V7	0.0016201
P0C214	Q8NBB4	0.29585
P0C214	O60783	0.55594
P0C214	O94892	0.2499
P0C214	O00570	0.77933
P0C214	Q6ZV56	0.086276
P0C214	Q9UJW3	0.70755
P0C214	Q16670	0.23889
P0C214	Q32MQ0	0.42743
P0C214	Q96CW6	0.56494
P0C214	Q13415	0.35275
P0C214	Q14181	0.56737
P0C214	Q5TAX3	0.65147
P0C214	P13645	0.56529
P0C214	Q9BXG8	0.43561
P0C214	Q8WWY3	0.33639
P0C214	Q8NE31	0.078699
P0C214	P43357	0.2675

P0C214	Q658K8	0.45829
P0C214	Q6W349	0.24108
P0C214	A6NME0	0.047773
P0C214	O75928	0.74882
P0C214	Q9H4M3	0.20121
P0C214	Q8TEK3	0.29571
P0C214	Q7L3S4	0.30122
P0C214	Q86YG4	0.5761
P0C214	Q9BV20	0.22595
P0C214	Q13347	0.8284
P0C214	Q9BPW8	0.45234
P0C214	K7EIZ4	0.086276
P0C214	Q68DK7	0.5989
P0C214	P62906	0.92893
P0C214	O96028	0.26345
P0C214	P0C7M6	0.060206
P0C214	Q6RSH7	0.46226
P0C214	O75638	1.0139
P0C214	Q9BYP9	0.43378
P0C214	Q5W0V3	0.66142
P0C214	Q16236	0.64074
P0C214	Q92670	0.2331
P0C214	O94855	0.38305
P0C214	Q8IZD4	1.0907
P0C214	Q9H7L9	0.56618
P0C214	Q96HR8	1.167
P0C214	O75170	0.82705
P0C214	P48595	0.92397
P0C214	Q330K2	0.25672
P0C214	P13667	0.33737
P0C214	O94983	1.228
P0C214	Q9H1Z4	0.35391
P0C214	Q9UKT5	0.52047
P0C214	O60573	0.63408
P0C214	A6NKD2	0.69286
P0C214	P11142	0.62486
P0C214	Q96E16	0.61668
P0C214	Q9NVN3	0.074523
P0C214	P52741	0.2289
P0C214	Q9NX40	0.46318
P0C214	Q9NS39	0.24578
P0C214	O15084	0.6866
P0C214	Q9UKT8	0.33409
P0C214	Q96F63	0.24937
P0C214	Q9Y592	0.50132
P0C214	Q3LI73	0.39837
P0C214	Q14693	0.46935
P0C214	A8MUQ0	0.8399
P0C214	Q6Q759	0.50185
P0C214	P60604	1.0001
P0C214	Q96IR2	0.38935
P0C214	Q16773	0.27839
P0C214	Q9NRA8	1.0593
P0C214	Q96GG9	0.35026
P0C214	O43399	0.54427
P0C214	Q9UHN1	0.14485
P0C214	Q8N988	0.43475
P0C214	Q6IB77	0.28275
P0C214	Q9UNK9	0.24543
P0C214	O15511	0.12637
P0C214	Q96DE5	0.87198
P0C214	Q14790	0.27072
P0C214	Q9H116	0.27612
P0C214	Q9C000	0.365
P0C214	P0C7T4	0.086276
P0C214	Q96NN9	0.30568
P0C214	Q96PI1	0.38078
P0C214	P55771	0.79826
P0C214	P35900	0.6674
P0C214	Q13042	1.1962
P0C214	O14777	0.59584
P0C214	Q9H7J1	0.2661
P0C214	Q9BY41	0.36813
P0C214	Q2NKJ3	0.42084
P0C214	Q8N9T8	1.1831
P0C214	Q7Z4W3	0.086276

35265	P0C214	Q02878	1.2069
35266	P0C214	Q8TF32	0.50756
35267	P0C214	Q8N8J6	0.15486
35268	P0C214	P31269	0.47447
35269	P0C214	Q9P2J5	0.50318
35270	P0C214	Q16589	0.22865
35271	P0C214	Q9H9L7	0.22197
35272	P0C214	Q15306	0.8414
35273	P0C214	P59923	0.3804
35274	P0C214	Q9H0C5	0.48074
35275	P0C214	Q5H9L2	1.0428
35276	P0C214	Q9NWT6	0.58393
35277	P0C214	Q9H714	0.40832
35278	P0C214	Q14241	0.34304
35279	P0C214	Q9NPG3	0.70205
35280	P0C214	Q86VM9	0.09871
35281	P0C214	O14737	0.268
35282	P0C214	Q9H1J1	1.2315
35283	P0C214	Q9NRL3	0.34365
35284	P0C214	Q96MW7	0.27837
35285	P0C214	P21695	0.34998
35286	P0C214	P25685	0.46384
35287	P0C214	P41212	0.65651
35288	P0C214	P16278	0.21674
35289	P0C214	O75427	0.42038
35290	P0C214	Q9BRT9	0.44262
35291	P0C214	Q96AV8	0.20486
35292	P0C214	Q9NZL9	0.40083
35293	P0C214	Q9HCM1	0.26865
35294	P0C214	P0CG29	0.29858
35295	P0C214	Q3I5F7	0.1505
35296	P0C214	Q9BZR8	0.43141
35297	P0C214	Q03938	0.51306
35298	P0C214	P17661	0.4509
35299	P0C214	Q4G112	0.18216
35300	P0C214	O60481	0.33449
35301	P0C214	Q9NVS2	0.62561
35302	P0C214	Q03701	0.2973
35303	P0C214	Q9Y314	1.1352
35304	P0C214	Q9Y222	0.86552
35305	P0C214	Q14525	0.51477
35306	P0C214	Q99502	0.12995
35307	P0C214	Q9Y3A2	0.92069
35308	P0C214	Q3KNS6	0.22926
35309	P0C214	Q8N720	0.90465
35310	P0C214	A8K5M9	0.71934
35311	P0C214	Q8IYK2	0.30734
35312	P0C214	Q96N16	0.9322
35313	P0C214	Q8NHM5	0.27376
35314	P0C214	P41214	0.62821
35315	P0C214	A6NN14	0.38021
35316	P0C214	Q8N137	0.71836
35317	P0C214	Q9BRH9	0.30964
35318	P0C214	Q9UGR2	0.39864
35319	P0C214	Q14674	0.26913
35320	P0C214	Q8WUM4	0.97897
35321	P0C214	Q96HA1	0.57849
35322	P03383	A6NN06	0.33834
35323	P03383	Q96BW9	0.35954
35324	P03383	Q969W8	0.27462
35325	P03383	Q5JQC4	0.33834
35326	P03383	Q5VUE5	0.17241
35327	P03383	Q6NSI4	0.079255
35328	P03383	Q14244	0.5153
35329	P03383	Q96LX8	0.21369
35330	P03383	P43351	0.3676
35331	P03383	Q6P3W6	0.38692
35332	P03383	Q9UD71	0.67767
35333	P03383	Q9UQR1	0.61182
35334	P03383	Q17RY6	0.52165
35335	P03383	Q8N3J3	0.19143
35336	P03383	Q7Z7A1	0.7888
35337	P03383	Q5BKU6	0.031284
35338	P03383	P35227	0.52285
35339	P03383	O75323	0.35152
35340	P03383	Q96MH2	0.94679

P03383	Q86UA6	0.57753
P03383	P08107	0.38424
P03383	Q9Y618	0.8438
P03383	Q7Z7J5	0.67519
P03383	O95678	0.51989
P03383	Q14683	0.30907
P03383	Q8NEF3	0.16565
P03383	Q6AI39	0.68802
P03383	O14628	0.29029
P03383	Q9BVL2	0.43872
P03383	O95402	0.87263
P03383	Q0VDD7	0.93007
P03383	Q9BRK4	0.34086
P03383	P78345	0.64395
P03383	Q86U86	0.76916
P03383	Q9UNS1	0.41276
P03383	Q13573	0.49357
P03383	Q14457	0.33128
P03383	Q92681	0.19856
P03383	Q8IXJ9	0.54504
P03383	Q9H1B4	0.23695
P03383	Q9Y6R9	0.56422
P03383	Q7Z2E3	0.2593
P03383	P02545	0.58109
P03383	O96018	0.26831
P03383	A1A4G5	0.33834
P03383	Q96EE3	0.39009
P03383	P23490	0.56784
P03383	A8MVA2	0.5733
P03383	Q06330	0.39658
P03383	O15504	0.31888
P03383	Q8N9W8	0.87506
P03383	O14519	0.13334
P03383	Q13761	0.6078
P03383	P59037	0.45203
P03383	O43167	0.39205
P03383	Q9NYV4	0.021814
P03383	Q5VSL9	0.5724
P03383	P42766	0.54689
P03383	Q9BZS9	0.33834
P03383	A6NGH7	0.20342
P03383	P43357	0.1668
P03383	Q9H8V8	0.33834
P03383	Q13257	0.50877
P03383	Q13228	0.78166
P03383	Q96CW1	0.31134
P03383	Q5VYM1	0.37766
P03383	Q15329	0.97588
P03383	Q5TGP6	0.018727
P03383	O14879	0.51645
P03383	Q9BQE6	0.36701
P03383	Q56VL3	0.47239
P03383	Q15723	0.97999
P03383	Q6IPT2	0.87506
P03383	Q9NQG7	0.14398
P03383	Q9BSU1	0.43443
P03383	P15924	0.22698
P03383	Q9P2D3	0.63377
P03383	O75167	0.059351
P03383	P41002	0.70331
P03383	P10244	0.35059
P03383	Q5T953	0.67106
P03383	Q16612	0.088023
P03383	O76087	0.17372
P03383	A6NG73	0.078213
P03383	Q8N3F0	0.37725
P03383	Q9Y3X0	0.45373
P03383	P30307	0.44229
P03383	Q0VFZ6	0.60341
P03383	Q8N1X5	0.33834
P03383	Q8WUM0	0.50316
P03383	Q9NVV4	0.41596
P03383	Q9H7R0	0.28794
P03383	Q02577	0.41162
P03383	Q8TAG6	0.31585
P03383	Q9UI26	0.15562

P03383	Q9H5V9	0.5605
P03383	Q9Y2Y0	0.73213
P03383	P53803	0.51687
P03383	Q9UBW5	0.31745
P03383	P59091	0.085124
P03383	Q9BZ01	0.22807
P03383	Q9UJY4	0.2736
P03383	Q8NHZ8	0.88202
P03383	A6NH11	0.40074
P03383	Q9NRR4	0.30512
P03383	Q6ZVK8	0.18902
P03383	Q96JG6	0.081923
P03383	Q8N0V1	0.11058
P03383	P51398	0.85912
P03383	Q8WTW4	0.21054
P03383	Q9BYQ7	0.7585
P03383	Q68CJ9	0.44894
P03383	Q7L5D6	0.72394
P03383	P48378	0.21863
P03383	O60927	0.50934
P03383	P36578	0.76792
P03383	Q8N8X9	0.16676
P03383	Q9NQS7	0.60238
P03383	Q9BQG0	0.38348
P03383	Q96GI7	0.60341
P03383	O60716	0.28319
P03383	Q6ZU52	0.1668
P03383	Q09028	0.47743
P03383	Q9Y6J9	0.60448
P03383	Q8NE22	0.73146
P03383	Q9HD47	0.10256
P03383	P13073	0.1147
P03383	Q8WTZ3	0.31327
P03383	Q9BYE4	0.33834
P03383	Q9UKN8	0.38183
P03383	Q8NA47	0.60341
P03383	P27986	0.35683
P03383	P0C860	0.1397
P03383	Q8N2M8	0.79445
P03383	O15198	0.25698
P03383	P02794	0.5652
P03383	Q8IX15	0.28947
P03383	A4QMS7	0.1104
P03383	Q13616	0.39304
P03383	Q99504	0.25141
P03383	Q03188	0.26025
P03383	Q4KN68	0.39984
P03383	O75182	0.89553
P03383	Q8N319	0.99505
P03383	Q8NI51	0.4334
P03383	P62140	0.56315
P03383	O00470	0.44899
P03383	Q719H9	0.44577
P03383	Q9Y253	0.05997
P03383	Q06265	0.12267
P03383	Q6P435	0.19612
P03383	Q8TC76	1.0726
P03383	Q9UHN1	0.033725
P03383	P78358	0.64765
P03383	Q8TB05	0.60341
P03383	H3BU77	0.27914
P03383	Q5FWF6	0.30166
P03383	Q12872	0.61847
P03383	Q7L804	0.38086
P03383	B1AL46	0.5011
P03383	P05388	0.9333
P03383	Q2NL68	0.60341
P03383	Q9HBH1	0.45975
P03383	Q5JVG8	0.29857
P03383	Q8IVU9	0.030538
P03383	P62266	0.71048
P03383	Q9Y285	0.446
P03383	Q6P1R3	0.39321
P03383	O95478	0.35581
P03383	Q14978	0.64911
P03383	A6NNF4	0.2288

P03383	P43359	0.60145
P03383	P60371	0.5733
P03383	O00213	0.25006
P03383	P16989	0.13163
P03383	Q9UBU9	0.28899
P03383	P08729	1.0616
P03383	Q8NHW5	0.067223
P03383	Q9NY61	0.61425
P03383	Q92585	0.75404
P03383	Q8IWJ2	0.45549
P03383	Q15935	0.22477
P03383	P39019	0.62034
P03383	Q96LC9	0.46025
P03383	Q07955	0.44556
P03383	Q15014	0.87304
P03383	Q8N5J2	0.84524
P03383	Q9Y2M2	0.25578
P03383	Q6QNY0	0.48358
P03383	P49454	0.42034
P03383	P50995	0.6136
P03383	O75920	0.82324
P03383	Q9Y3C8	0.14292
P03383	Q13601	0.17716
P03383	P55199	0.78301
P03383	Q12830	0.70532
P03383	Q6RW13	0.45566
P03383	Q9UHL9	0.35564
P03383	Q96EN9	0.44541
P03383	Q8NBB4	0.17074
P03383	O00458	0.55491
P03383	Q9BRJ7	0.42836
P03383	Q05DH4	0.87614
P03383	P19474	0.27673
P03383	Q8N1N4	0.60058
P03383	P10914	0.27111
P03383	Q8N3L3	0.56434
P03383	Q96N23	0.35687
P03383	O75925	0.38281
P03383	Q9NQ31	0.4154
P03383	P34949	0.17216
P03383	Q8TF74	0.24787
P03383	P26373	0.71018
P03383	O14737	0.2801
P03383	Q9NYW8	0.14944
P03383	Q9H7Z3	0.21221
P03383	O15234	0.80806
P03383	Q969F9	0.69872
P03383	Q9Y5J7	0.11634
P03383	P84101	0.811
P03383	Q63HK3	0.29246
P03383	Q9BUK0	0.6964
P03383	Q5TAB7	0.55677
P03383	Q7Z739	0.42872
P03383	P62879	0.29199
P03383	Q6PJG6	0.52478
P03383	Q8IVD9	0.30819
P03383	Q502W6	0.0067479
P03383	Q8N888	0.59132
P03383	Q2M238	0.0031475
P03383	P56945	0.37783
P03383	A6NNV3	0.38945
P03383	Q8TA86	0.45984
P03383	Q9BXW3	0.33834
P03383	P14316	0.6874
P03383	Q969W3	0.54692
P03383	Q15561	0.69234
P03383	Q9Y2L1	0.3447
P03383	Q96S65	0.89028
P03383	Q6P047	0.3175
P03383	Q86Y46	0.39317
P03383	Q8N344	0.29481
P03383	Q5TG92	0.37335
P03383	Q9BWJ2	0.093306
P03383	Q9P1A6	0.137
P03383	E7EW31	0.33834
P03383	Q9BXU2	0.098116

35569	P03383	O15266	0.35951
35570	P03383	P09661	0.45549
35571	P03383	Q9BU76	0.65528
35572	P03383	P56377	0.12861
35573	P03383	Q5BKX5	0.60341
35574	P03383	Q92831	0.60514
35575	P03383	O15126	0.1059
35576	P03383	Q9Y2S7	0.6308
35577	P03383	Q8N0Z3	0.32932
35578	P03383	Q8IXT1	0.072039
35579	P03383	Q9Y3I1	0.60115
35580	P03383	Q86XR8	0.64015
35581	P03383	Q330K2	0.20775
35582	P03383	Q96FJ8	0.33834
35583	P03383	Q5JR59	0.68091
35584	P03383	Q8NBR6	1.0587
35585	P03383	Q96NL0	0.18106
35586	P03383	Q9UBD5	0.32816
35587	P03383	O75794	0.80164
35588	P03383	Q5JVS0	0.54996
35589	P03383	Q92621	0.81266
35590	P03383	Q9H3J6	0.68731
35591	P03383	Q16543	0.21074
35592	P03383	Q8IWF9	0.051817
35593	P03383	Q9BVV6	0.33834
35594	P03383	Q9UHV9	0.55068
35595	P03383	Q96GA3	0.6131
35596	P03383	Q12888	0.47997
35597	P03383	Q96BR6	0.27836
35598	P03383	Q32MZ4	0.8333
35599	P03383	Q9H974	0.82188
35600	P03383	Q6ZTI6	0.66283
35601	P03383	Q6UXR8	0.33834
35602	P03383	Q8N8E1	0.25057
35603	P03383	Q9BZE7	0.60341
35604	P03383	Q99547	0.55896
35605	P03383	Q9ULX3	0.44693
35606	P03383	Q86UU5	0.48946
35607	P03383	Q14746	0.20122
35608	P03383	P60608	0.46353
35609	P03383	Q7Z4W3	0.33834
35610	P03383	P11831	0.41708
35611	P03383	P49792	0.63219
35612	P03383	O14757	0.43158
35613	P03383	B9ZVM9	0.8045
35614	P03383	Q15287	0.71913
35615	P03383	Q8WZ33	0.52502
35616	P03383	Q8N2Z9	0.30313
35617	P03383	K7EIQ3	0.67516
35618	P03383	Q4G0G2	0.077779
35619	P03383	Q8NFH4	0.74228
35620	P03383	Q53EL6	0.7158
35621	P03383	Q9H8X3	0.0057033
35622	P03383	Q15047	0.45186
35623	P03383	Q9H019	0.51712
35624	P03383	Q6W349	0.39604
35625	P03383	Q96T76	0.662
35626	P03383	Q17RB0	0.33511
35627	P03383	Q16352	0.62256
35628	P03383	Q5T6M2	0.67642
35629	P03383	Q5T871	0.33834
35630	P03383	Q8NHR7	0.14018
35631	P03383	Q8N6V4	0.091287
35632	P03383	Q8N535	0.18021
35633	P03383	Q8ND04	0.39513
35634	P03383	Q9BQA1	0.70732
35635	P03383	Q15010	0.15471
35636	P03383	Q9H4X1	0.55549
35637	P03383	Q7Z417	0.51882
35638	P03383	Q06609	0.26727
35639	P03383	O95625	0.08798
35640	P03383	Q9UH92	0.61766
35641	P03383	Q6TDU7	0.16829
35642	P03383	Q5TYX0	0.28378
35643	P03383	Q5TC12	0.26368
35644	P03383	Q6ZV56	0.33834

P03383	P86480	0.33834
P03383	P04733	0.2243
P03383	Q9UBB6	0.33752
P03383	Q96G03	0.39338
P03383	Q96A22	0.23718
P03383	Q7Z460	0.24206
P03383	Q96SK3	0.22485
P03383	Q14839	0.36782
P03383	O75534	0.11033
P03383	Q96JK9	0.69611
P03383	Q96LI9	0.19446
P03383	Q8NFC6	0.59341
P03383	A6NFY7	0.49529
P03383	Q96EL2	0.20824
P03383	P15036	0.54347
P03383	P35321	0.29773
P03383	Q6UVJ0	0.57357
P03383	Q9BYD1	0.58692
P03383	Q0D2K3	0.69429
P03383	Q9Y281	0.21294
P03383	Q86UW9	0.61649
P03383	O75582	0.24908
P03383	Q9BTL4	0.38163
P03383	H3BTG2	0.41986
P03383	P62913	0.79736
P03383	Q6ZN08	0.29071
P03383	Q8NI27	0.52524
P03383	Q05048	0.54949
P03383	Q8WWY6	0.19547
P03383	P80294	0.46119
P03383	Q86XD5	0.17389
P03383	P35080	0.19009
P03383	Q9Y3V2	0.55947
P03383	Q12894	0.34215
P03383	O14497	0.58459
P03383	Q9BX40	0.54274
P03383	P35712	0.36276
P03383	P50549	0.69326
P03383	P62945	0.49535
P03383	O95602	0.42171
P03383	Q01105	0.53308
P03383	O60671	0.29839
P03383	Q9UK58	0.16372
P03383	P06746	0.24382
P03383	Q5VZQ5	0.39512
P03383	Q8IWT0	0.03876
P03383	Q6PJT7	0.66778
P03383	Q9NQR7	0.33834
P03383	Q02080	0.22249
P03383	P55036	0.42319
P03383	Q96N21	0.42098
P03383	P61024	0.5264
P03383	Q13485	0.46037
P03383	Q969Q6	0.075182
P03383	Q9H2U2	0.30173
P03383	Q9UJW0	0.16716
P03383	Q9Y4B6	0.5966
P03383	Q6P1J9	0.47678
P03383	P02533	0.75254
P03383	Q96SZ4	0.12019
P03383	Q56P03	0.37695
P03383	Q9Y6Z5	0.56716
P03383	Q9BYG8	0.021759
P03383	Q12904	0.073534
P03383	Q15369	1.0539
P03383	Q9HBD4	0.40643
P03383	Q9P1J3	0.33834
P03383	Q9Y324	0.66545
P03383	Q8NFH8	0.20595
P03383	P61574	0.43317
P03383	P63272	0.78776
P03383	Q9NQ92	0.66876
P03383	Q9UHV2	1.2488
P03383	Q6IPU0	0.52894
P03383	Q9BSG1	0.25293
P03383	Q14653	0.41071

P03383	Q5SVZ6	0.57792
P03383	Q9BSJ2	0.73739
P03383	O75494	0.48129
P03383	P20290	0.8435
P03383	Q9BZL4	0.31745
P03383	Q9UK10	0.24149
P03383	Q9BVW5	0.70152
P03383	Q86XI2	0.52687
P03383	Q06455	0.5031
P03383	Q6PI97	0.083393
P03383	Q04864	0.85778
P03383	Q96A56	0.78643
P03383	O14862	0.30873
P03383	A6NK75	0.21999
P03383	A6NNW6	0.34097
P03383	Q7Z3C6	0.19473
P03383	Q9NTU4	0.45558
P03383	P0DI83	0.29972
P03383	O95163	0.51443
P03383	P33076	0.33116
P03383	Q96I23	0.13749
P03383	Q64ET8	0.56042
P03383	Q96TC7	0.48855
P03383	O00716	0.87253
P03383	Q86WP2	0.40115
P03383	O60809	0.34634
P03383	Q9BYA6	0.33834
P03383	O75791	0.68547
P03383	Q15366	0.6477
P03383	P08047	0.57709
P03383	Q9UPW6	0.52299
P03383	Q9H6U6	0.52644
P03383	P19013	0.58419
P03383	Q8HWS3	0.16579
P03383	Q569H4	0.60341
P03383	Q9UKL3	0.91729
P03383	Q7L5A3	0.39277
P03383	O96019	0.73385
P03383	P07311	0.42451
P03383	A6NK39	0.12558
P03383	Q9NWT6	0.28842
P03383	Q6P050	0.45471
P03383	Q9NTK1	0.50934
P03383	P51003	0.1295
P03383	Q9P2J5	0.15735
P03383	Q9H3P2	0.85132
P03383	O94925	0.29495
P03383	Q9BSH4	0.78971
P03383	Q9UIA9	0.27412
P03383	Q9NYV6	0.35839
P03383	Q9BTC8	0.39335
P03383	P06748	0.29207
P03383	Q68E01	0.32765
P03383	Q5T749	0.63707
P03383	Q1MSJ5	0.80221
P03383	O43148	0.72694
P03383	P0C7T4	0.33834
P03383	P13010	0.20384
P03383	P62854	0.61345
P03383	Q32M84	0.069626
P03383	Q9H3H3	0.098584
P03383	Q9ULM3	0.54938
P03383	Q14209	0.86926
P03383	P25815	0.29241
P03383	Q96BD8	0.39487
P03383	Q6ZU35	0.60341
P03383	Q6NW29	0.27433
P03383	A6NKU9	0.38945
P03383	Q00610	0.61561
P03383	P29084	0.94701
P03383	P29474	0.38259
P03383	Q969L4	0.4873
P03383	Q96ES7	0.044807
P03383	Q4G0A6	0.77565
P03383	P40926	0.17722
P03383	Q9H9E3	0.27809

P03383	Q9GZW5	0.45626
P03383	O75528	0.54533
P03383	A6NJB7	0.60137
P03383	F8W1W9	0.14207
P03383	O75143	0.8233
P03383	O14933	0.25293
P03383	P82930	0.65601
P03383	O95865	0.21008
P03383	Q9Y305	0.42946
P03383	P55212	0.2848
P03383	A2RTY3	0.15528
P03383	Q99607	0.81303
P03383	Q9BR84	0.35536
P03383	Q5T0Z8	0.60341
P03383	A5PLN9	0.39297
P03383	P53804	0.47131
P03383	O60543	0.18123
P03383	A6NJI1	0.37614
P03383	Q9BPY3	0.086229
P03383	P17096	0.32758
P03383	A8K554	0.024989
P03383	Q9BPZ2	0.42693
P03383	Q5TBE3	0.22214
P03383	Q8TBB1	0.51142
P03383	O75478	0.16888
P03383	P40429	0.49673
P03383	Q9H0F6	0.030294
P03383	Q5JUQ0	0.27367
P03383	P0C7X3	0.2823
P03383	Q9BRU2	0.26009
P03383	Q9H8S9	0.22592
P03383	Q9UKI9	0.99687
P03383	Q9NXX6	0.13588
P03383	Q86SX3	1.0823
P03383	O15397	0.59793
P03383	Q9Y244	0.15616
P03383	Q9Y5L5	0.83646
P03383	P16220	0.66491
P03383	Q9UHZ2	0.33834
P03383	P08727	0.75254
P03383	Q9P206	0.64276
P03383	Q9NZV7	0.37043
P03383	Q9HCU8	0.32381
P03383	Q9BYR3	0.5733
P03383	Q9H7R5	0.25401
P03383	Q96M63	0.057716
P03383	Q5SV97	0.5684
P03383	P82912	0.39604
P03383	Q9BVJ6	0.53608
P03383	P27540	0.29639
P03383	Q9NWS1	0.60675
P03383	A6PW82	0.39629
P03383	O14579	0.33042
P03383	Q96DE5	0.78343
P03383	O00167	0.37813
P03383	Q5SZB4	0.044355
P03383	Q15562	0.63425
P03383	Q9Y2P7	0.25605
P03383	Q8N0W5	0.073244
P03383	Q9BYD2	0.59283
P03383	Q8N1L9	0.63615
P03383	O75251	0.03063
P03383	Q68DK2	0.58167
P03383	P58505	0.3294
P03383	Q8NCF5	0.257
P03383	Q9BQD3	0.18389
P03383	P14868	0.56496
P03383	Q7Z6K1	0.44437
P03383	Q8WW33	0.43519
P03383	A6NJV1	0.46487
P03383	Q9H0S4	0.029572
P03383	O43390	0.46611
P03383	Q9Y2P8	0.41192
P03383	Q15051	0.36344
P03383	Q96S19	0.052759
P03383	A6NDB9	0.33536

P03383	B9A014	0.11208
P03383	P28069	0.46079
P03383	Q02241	0.46526
P03383	P54278	0.17177
P03383	Q8NBT2	0.47096
P03383	A6NNH0	0.52445
P03383	Q9Y6K9	0.61734
P03383	Q9BV19	0.38343
P03383	Q6ZSN7	0.21126
P03383	Q49A33	0.29837
P03383	Q8WXS3	0.73927
P03383	Q93083	0.23866
P03383	Q15544	0.83827
P03383	Q8IYN6	0.58513
P03383	O60756	0.33834
P03383	Q5U649	0.011095
P03383	Q14678	0.3233
P03383	Q92844	0.2321
P03383	Q5T752	0.33834
P03383	Q9H4K1	0.36465
P03383	Q8TF01	0.71733
P03383	Q92572	0.25661
P03383	Q9UQK1	0.20881
P03383	Q9H0C8	0.19352
P03383	Q8N8H1	0.28301
P03383	Q9H8H0	0.050323
P03383	Q96A35	0.49411
P03383	Q8N3U4	0.48005
P03383	Q63HN1	0.19935
P03383	Q8TAD8	0.97899
P03383	Q8IUC1	0.23053
P03383	Q6ZYL4	0.6634
P03383	O94986	0.80035
P03383	Q8IWL8	0.60533
P03383	Q13469	0.81845
P03383	Q9H9Z2	0.53732
P03383	Q52LG2	0.22016
P03383	Q8NBZ9	0.28513
P03383	Q7Z591	0.88263
P03383	P62987	0.43829
P03383	Q9BSF8	0.11945
P03383	Q13515	0.78461
P03383	Q9NWB7	0.39036
P03383	P17544	0.7483
P03383	Q6ZUT1	0.33834
P03383	P56270	0.37206
P03383	Q8N6T7	0.40256
P03383	Q96B67	0.14837
P03383	Q5TI25	0.95616
P03383	Q8IUR6	0.7543
P03383	Q9NPA8	0.22164
P03383	P62891	0.4221
P03383	Q12824	0.80995
P03383	Q9NVN8	0.17744
P03383	P25398	0.45256
P03383	B1AMM8	0.087817
P03383	Q9H4B0	0.52021
P03383	Q9NSB4	0.46889
P03383	Q5BN46	0.087099
P03383	Q16633	0.3299
P03383	P35269	0.85762
P03383	Q8IUC6	0.093042
P03383	Q8NEE0	0.33834
P03383	P27469	0.56985
P03383	Q96JI7	0.27719
P03383	O95397	0.70573
P03383	Q8WV99	0.086122
P03383	Q14008	0.6109
P03383	P0C7T5	0.39679
P03383	Q8N7Y1	0.33834
P03383	Q9H777	0.31808
P03383	P51808	0.099736
P03383	Q86V20	0.59763
P03383	Q7Z6B0	0.36611
P03383	Q86VY4	0.30246
P03383	Q14160	0.41994

P03383	Q8TAK5	0.24671
P03383	Q9NZJ0	0.46678
P03383	Q13216	0.24422
P03383	P32243	0.65127
P03383	Q9NSB2	0.59286
P03383	Q9UP83	0.90273
P03383	Q9H900	0.31244
P03383	Q15691	0.44742
P03383	A6NFL8	0.5567
P03383	Q9HD40	0.31488
P03383	P61572	0.43317
P03383	P62324	0.53048
P03383	Q9Y3C0	0.18353
P03383	Q32MQ0	0.38248
P03383	P83876	0.73141
P03383	P56277	0.61063
P03383	Q8NHY5	0.66864
P03383	Q9NX63	0.49245
P03383	Q96AJ1	0.96594
P03383	A6NF01	0.71685
P03383	Q5H9I0	0.22463
P03383	Q86WZ0	0.26246
P03383	P19338	0.37907
P03383	Q16626	0.1798
P03383	Q13884	0.22686
P03383	P51948	0.36569
P03383	Q02040	0.37365
P03383	O60287	0.77282
P03383	Q13233	0.37975
P03383	Q9H4I2	0.558
P03383	Q13084	0.74772
P03383	P11844	0.58659
P03383	O00472	0.33296
P03383	Q9NP64	0.046073
P03383	P13640	0.38669
P03383	A4D1U4	0.24382
P03383	Q9NYG5	0.49821
P03383	Q9H840	0.62438
P03383	Q9HBE1	0.10931
P03383	Q00653	0.90917
P03383	Q3ZLR7	0.60338
P03383	P0CZ25	0.0052814
P03383	C9JFL3	0.33834
P03383	O75896	0.41602
P03383	Q8WUY3	0.077409
P03383	Q9UL18	0.74231
P03383	Q15459	0.47234
P03383	Q658K8	0.32506
P03383	Q96LR2	0.78859
P03383	O00567	0.58869
P03383	P62280	0.46333
P03383	O60765	0.20784
P03383	P82664	0.76771
P03383	Q9H2J4	0.4136
P03383	P62318	0.59235
P03383	Q9BYR2	0.5733
P03383	Q96IZ7	0.90379
P03383	Q8WTV1	0.67872
P03383	P55735	0.35851
P03383	Q8IYB0	0.33834
P03383	Q13356	0.14348
P03383	Q96QF0	0.34856
P03383	P26371	0.33834
P03383	P0CG20	0.18629
P03383	O95684	0.53555
P03383	Q96DY2	0.045709
P03383	Q9H706	0.1668
P03383	Q08999	1.0658
P03383	A8MVS1	0.33178
P03383	Q5VZE5	0.13755
P03383	H0YKK7	0.52432
P03383	Q07020	0.52506
P03383	Q9H8G1	0.3213
P03383	Q8IYL3	0.72583
P03383	A0PJX2	0.34313
P03383	O94805	0.55123

P03383	Q14129	0.40337
P03383	Q9UNP9	0.27182
P03383	O94829	0.75093
P03383	Q8IU60	0.61306
P03383	Q8IY31	0.29249
P03383	Q8N6I1	0.72988
P03383	Q9BTA9	0.69069
P03383	Q13166	0.33834
P03383	Q14139	0.38268
P03383	Q93075	0.43172
P03383	Q96M60	0.22794
P03383	Q5UIP0	0.68691
P03383	P51965	0.30957
P03383	P25440	0.32341
P03383	O95433	0.27961
P03383	Q6UUV9	1.1049
P03383	P0C7H8	0.23605
P03383	P49458	0.48548
P03383	Q8WWK9	0.20952
P03383	Q86UE4	0.37292
P03383	Q9NTX9	0.11309
P03383	Q9NWS8	0.39185
P03383	Q96MT4	0.59664
P03383	P06454	0.38233
P03383	Q9GZZ1	0.28076
P03383	O14639	0.28571
P03383	A8MWA4	0.36598
P03383	Q9C075	0.58667
P03383	P53618	0.49657
P03383	Q9P2G4	0.60341
P03383	Q96HA9	0.35459
P03383	O43290	0.68951
P03383	O60248	0.34844
P03383	Q9UJX6	0.35911
P03383	Q9H944	0.88349
P03383	P0CW23	0.23459
P03383	O94776	0.76878
P03383	Q3SYF9	0.33834
P03383	Q3T8J9	0.38766
P03383	P40337	0.56071
P03383	P62312	0.57169
P03383	P25789	0.48239
P03383	O15040	0.1668
P03383	P62750	0.61236
P03383	Q8N1I8	0.33834
P03383	Q9NZD8	0.41322
P03383	Q5VTL8	0.68292
P03383	Q8WXE1	0.43207
P03383	P49711	0.13498
P03383	Q9H4D5	0.39853
P03383	Q32MH5	0.31194
P03383	Q0VD86	0.11945
P03383	A8MYP8	0.42146
P03383	Q96M91	0.68468
P03383	P54105	0.59709
P03383	Q8WUX9	0.20428
P03383	Q9BSK0	0.55613
P03383	Q5D0E6	0.56706
P03383	P53370	0.27623
P03383	P17022	0.09884
P03383	P22626	0.42497
P03383	P62072	0.11076
P03383	P0CL83	0.44986
P03383	Q5T5J6	0.55995
P03383	Q9BUX1	0.5045
P03383	I3L3R5	0.33834
P03383	Q9BTA0	0.60341
P03383	Q107X0	0.4565
P03383	Q8N1A0	0.79565
P03383	Q9BUN5	0.24424
P03383	Q14106	0.26787
P03383	P83436	0.25922
P03383	Q86V71	0.24064
P03383	Q8TD19	0.25459
P03383	Q8N163	0.49598
P03383	Q9Y678	0.3122

P03383	P60409	0.5733
P03383	A6H8Z2	0.30181
P03383	Q52LR7	0.16942
P03383	P08670	0.11869
P03383	Q9BRJ2	0.17835
P03383	P60900	0.30442
P03383	Q15596	0.48625
P03383	Q6P4R8	0.78412
P03383	P15172	0.2416
P03383	Q01094	0.62848
P03383	P49789	0.24993
P03383	Q96GE5	0.30771
P03383	Q9BSJ6	0.33335
P03383	Q9H5H4	0.18821
P03383	Q8N128	0.33834
P03383	Q5TG53	0.33834
P03383	Q96HB5	0.27198
P03383	Q8TAF3	0.37153
P03383	Q96SL8	0.10704
P03383	Q15013	0.58225
P03383	P04156	0.27973
P03383	B2RV13	0.42591
P03383	Q6ZRH3	0.26503
P03383	Q96RL7	0.72398
P03383	Q5HYK7	0.81116
P03383	Q8N1B4	0.33566
P03383	P14866	0.37348
P03383	P36639	0.11394
P03383	Q5HY98	0.28894
P03383	Q96MC9	0.76829
P03383	A6NEA5	0.03148
P03383	Q3KP31	0.30771
P03383	Q5VZR2	0.80122
P03383	O15371	0.42219
P03383	Q9NYB0	0.6077
P03383	Q9H869	1.0792
P03383	O15511	0.028469
P03383	P05387	0.38311
P03383	Q9BQ48	0.62313
P03383	Q9BTK6	0.24046
P03383	P14921	0.60432
P03383	Q15937	0.177
P03383	Q15029	0.21794
P03383	P55771	0.59989
P03383	P62249	0.50761
P03383	P54922	0.47345
P03383	Q15072	0.14988
P03383	O75376	0.31391
P03383	Q8WVJ9	0.48493
P03383	P0DMB1	0.37274
P03383	Q8WU58	0.26427
P03383	Q15572	0.66739
P03383	Q9H1K1	0.56759
P03383	Q8N7R1	0.46463
P03383	O75486	0.53787
P03383	Q14CN4	0.39317
P03383	P60331	0.5733
P03383	Q6NZI2	0.74978
P03383	Q96KF2	0.33834
P03383	P02728	0.33834
P03383	Q9BU02	0.52786
P03383	Q9ULK4	1.0144
P03383	Q5VYV7	0.1668
P03383	Q3SXR2	0.067839
P03383	Q14526	0.41751
P03383	P54920	0.2117
P03383	Q96G27	0.38631
P03383	Q96EH3	0.072976
P03383	Q9UQL6	0.33004
P03383	P17029	0.28158
P03383	P17661	0.28304
P03383	Q9NXZ1	0.072501
P03383	Q07687	0.34755
P03383	Q7Z3E2	0.51034
P03383	Q9BRA2	0.2604
P03383	P38646	0.40891

36177	P03383	Q9NVX0	0.68544
36178	P03383	Q6ZS72	0.28574
36179	P03383	Q9H0C5	0.35261
36180	P03383	P58340	0.27539
36181	P03383	Q96F24	0.44871
36182	P03383	Q5BJE1	0.14485
36183	P03383	Q6ZR85	0.33834
36184	P03383	Q4VC05	0.51645
36185	P03383	Q3ZCQ2	0.33834
36186	P03383	Q3MIV0	0.33834
36187	P03383	O14713	0.33997
36188	P03383	Q9Y5Q3	0.64368
36189	P03383	Q7Z4Q2	0.61986
36190	P03383	Q96NT1	0.81027
36191	P03383	P0CG00	0.086869
36192	P03383	Q5TAP6	0.50339
36193	P03383	Q9P2W6	0.33834
36194	P03383	A4D0V7	0.63209
36195	P03383	A8MX34	0.27495
36196	P03383	Q96H20	0.63722
36197	P03383	Q96RL1	0.50747
36198	P03383	P62888	0.48349
36199	P03383	Q96H12	0.67645
36200	P03383	Q96BN6	0.24623
36201	P03383	A6NHQ4	0.54577
36202	P03383	Q9H981	0.78119
36203	P03383	Q8N4H0	0.11295
36204	P03383	Q8IYJ2	0.49962
36205	P03383	Q9H4T2	0.079859
36206	P03383	Q9NWZ5	0.027697
36207	P03383	Q96MD2	0.071182
36208	P03383	O95487	0.43206
36209	P03383	P57772	0.45101
36210	P03383	Q12815	0.2894
36211	P03383	A6NKD9	0.88164
36212	P03383	O00287	0.34246
36213	P03383	Q8NAE3	0.1833
36214	P03383	P62899	0.40319
36215	P03383	Q6ZS94	0.33834
36216	P03383	Q15650	0.52908
36217	P03383	Q7Z4T9	0.45364
36218	P03383	Q9UBB4	0.70582
36219	P03383	Q6NUP7	0.26534
36220	P03383	O15162	0.10979
36221	P03383	Q96JS3	0.1371
36222	P03383	Q5PSV4	0.16676
36223	P03383	Q9UHB4	0.39729
36224	P03383	P19484	0.31253
36225	P03383	Q9UBQ5	0.70638
36226	P03383	P50502	0.37205
36227	P03383	Q9NWK9	0.39625
36228	P03383	Q16656	0.2146
36229	P03383	Q5TID7	0.28688
36230	P03383	O43264	0.37077
36231	P03383	Q9C0F0	0.25509
36232	P03383	Q49A88	0.34899
36233	P03383	P61571	0.3072
36234	P03383	P12270	0.47328
36235	P03383	Q03111	0.9661
36236	P03383	B1ATL7	0.12825
36237	P03383	Q8NA96	0.034227
36238	P03383	Q9BQ61	0.60341
36239	P03383	O14920	0.3884
36240	P03383	P31946	0.38879
36241	P03383	Q9Y5R5	0.15761
36242	P03383	O75792	0.73677
36243	P03383	Q8TDB4	0.21421
36244	P03383	O95503	0.6738
36245	P03383	Q9UPQ9	0.35791
36246	P03383	Q5FBB7	0.49055
36247	P03383	Q969H6	0.62526
36248	P03383	Q9HCL3	0.31749
36249	P03383	P01106	0.31329
36250	P03383	Q9BU61	0.40067
36251	P03383	Q13439	0.6471
36252	P03383	Q9UK73	0.5162

P03383	A6NDE8	0.17372
P03383	Q15173	0.15245
P03383	O43172	0.51544
P03383	Q9Y675	0.49935
P03383	Q8WW38	0.3599
P03383	O75437	0.28415
P03383	Q96FJ2	0.37554
P03383	Q86YP4	0.63879
P03383	Q96CJ1	0.93618
P03383	Q86U70	0.17039
P03383	Q6PD62	0.44218
P03383	Q96MB7	0.3355
P03383	P19367	0.1965
P03383	Q9H633	0.14165
P03383	Q5T2W1	0.27497
P03383	O43257	0.24286
P03383	P60228	0.67286
P03383	Q6ZR54	0.33834
P03383	Q15257	0.2659
P03383	Q06547	0.84001
P03383	Q93015	0.54925
P03383	Q13487	1.1651
P03383	O60613	0.41636
P03383	O95251	0.63624
P03383	P0C852	0.33834
P03383	Q8NDM7	0.37477
P03383	Q8TAV0	0.71733
P03383	Q13769	0.40194
P03383	P61927	0.411
P03383	Q96GN5	0.56091
P03383	Q9UHR5	0.93707
P03383	Q7L590	0.74964
P03383	P40424	0.4857
P03383	O95295	0.25777
P03383	Q7L592	0.33857
P03383	Q6ZNR8	0.33834
P03383	O00534	0.22715
P03383	A8MXY4	0.2288
P03383	Q9GZN8	0.17672
P03383	Q8TC05	0.51935
P03383	Q96MU7	0.48818
P03383	Q9UL33	0.24743
P03383	A6NEN9	0.0027008
P03383	A6NIX2	0.75949
P03383	Q5DJT8	0.069402
P03383	P41220	0.25232
P03383	Q12986	0.072161
P03383	O15304	0.68178
P03383	Q13838	0.093144
P03383	P24534	0.37167
P03383	P78347	0.45062
P03383	Q04727	0.4978
P03383	Q6NT76	0.24624
P03383	Q9H0W8	0.30636
P03383	Q7Z5L9	0.66613
P03383	Q6AWC8	0.43254
P03383	Q6ZTY9	0.18473
P03383	Q9HD26	0.68559
P03383	Q6NVH7	0.34096
P03383	Q15554	0.30613
P03383	Q8TDH9	0.085214
P03383	Q9UKD1	0.50783
P03383	Q9UJW3	0.40918
P03383	P00338	0.38442
P03383	Q96GY3	0.31789
P03383	Q9H7E2	0.18883
P03383	Q96NI8	0.28926
P03383	O00541	0.28415
P03383	Q5XKE5	0.50629
P03383	Q9HCS7	0.26169
P03383	O15527	0.2933
P03383	Q6ZUA9	0.36908
P03383	Q9H379	0.43004
P03383	Q9NRI5	0.18718
P03383	A8CG34	0.33372
P03383	Q3YBM2	0.5469

P03383	A6NKH3	0.27174
P03383	O15037	0.27014
P03383	Q9UPN9	0.31725
P03383	P41227	0.21017
P03383	Q8NE71	0.2197
P03383	Q99608	0.032897
P03383	Q9UI95	0.96469
P03383	P53990	0.40002
P03383	Q9ULR0	0.66178
P03383	Q13888	0.42488
P03383	Q8IWV8	0.14158
P03383	Q9P2H0	0.1668
P03383	Q9BW60	0.41347
P03383	Q14181	0.35271
P03383	Q6ZT83	0.080212
P03383	O96006	0.52569
P03383	A6NJR5	0.38945
P03383	Q9BXQ6	0.33834
P03383	Q9NRX3	0.55604
P03383	P46777	0.90651
P03383	Q96FH0	0.31354
P03383	P0C7M3	0.034227
P03383	Q86WG3	0.2259
P03383	P49116	0.45552
P03383	Q9P2R6	0.81949
P03383	Q7Z7A3	0.41144
P03383	Q96DZ1	0.10352
P03383	Q9Y5S9	0.59226
P03383	Q92670	0.1717
P03383	Q99575	0.48668
P03383	Q5JXB2	0.19426
P03383	Q9BYZ2	0.38442
P03383	Q9Y5B6	0.54757
P03383	P23511	0.86355
P03383	P05204	0.26701
P03383	O75362	0.54868
P03383	Q9UL49	0.31887
P03383	Q6ZUT4	0.33629
P03383	Q5VZT2	0.001272
P03383	P51815	0.15789
P03383	Q9HAW0	0.24595
P03383	Q96C34	0.41523
P03383	P14859	0.91563
P03383	Q8IWA6	0.23861
P03383	Q8TAC9	0.30848
P03383	P23193	0.92383
P03383	Q9NTK5	0.43647
P03383	Q13875	0.24985
P03383	P46779	0.83108
P03383	B3EWG5	0.60341
P03383	Q8N668	0.5618
P03383	Q9UKW6	0.27014
P03383	Q9ULN7	0.58421
P03383	P49427	0.36945
P03383	P23297	0.31285
P03383	Q9HAY2	0.013612
P03383	Q53FE4	0.024361
P03383	Q19AV6	0.76361
P03383	Q8IWN6	0.23641
P03383	A6NGG3	0.33834
P03383	Q8N7M2	0.32673
P03383	Q86YW9	0.22989
P03383	Q5JTZ5	0.078901
P03383	Q9BYP8	0.33834
P03383	P52948	0.70774
P03383	P41567	0.60077
P03383	Q96C90	0.49589
P03383	Q9BRR0	0.15978
P03383	Q9GZN7	0.32178
P03383	P49207	0.54942
P03383	Q7Z465	0.39554
P03383	Q13360	0.19829
P03383	Q6DHV5	0.04737
P03383	O00570	0.63291
P03383	Q8WXE9	0.327
P03383	A6NLC8	0.82598

36405	P03383	Q9NP66	0.75338
36406	P03383	P61956	0.43665
36407	P03383	P07902	0.31814
36408	P03383	Q86VG3	0.44286
36409	P03383	Q9H8W3	0.28426
36410	P03383	P27694	0.36152
36411	P03383	Q9ULQ0	0.78362
36412	P03383	Q3SYA9	0.33037
36413	P03383	A8MTY0	0.31091
36414	P03383	Q9P0J6	0.40518
36415	P03383	Q9Y4A5	0.62155
36416	P03383	O60244	0.39358
36417	P03383	Q5JSZ5	0.69687
36418	P03383	Q9BWW4	0.61839
36419	P03383	P12036	0.31459
36420	P03383	A6NMN3	0.15305
36421	P03383	Q9NYJ1	0.74004
36422	P03383	Q8WTT2	0.23189
36423	P03383	Q7Z3K6	0.29481
36424	P03383	Q9H7P6	0.65778
36425	P03383	P31749	0.38022
36426	P03383	Q96BD5	0.2455
36427	P03383	Q8TDR0	0.51674
36428	P03383	Q969F1	0.45519
36429	P03383	P55795	0.017956
36430	P03383	Q9GZR5	0.27533
36431	P03383	Q5SXJ2	0.95616
36432	P03383	P31040	0.48166
36433	P03383	Q9NRG4	0.51566
36434	P03383	Q96FX2	0.62066
36435	P03383	Q53HV7	0.35759
36436	P03383	Q5VTQ0	0.073768
36437	P03383	Q9P258	0.32428
36438	P03383	Q9P1G2	0.21097
36439	P03383	Q9H7U1	0.79193
36440	P03383	P36952	0.18062
36441	P03383	O43236	0.25348
36442	P03383	G3V211	0.5348
36443	P03383	Q9H6Y2	0.30057
36444	P03383	Q5VYK3	0.18547
36445	P03383	Q8TD23	0.44163
36446	P03383	Q3LI70	0.33834
36447	P03383	Q05481	0.2288
36448	P03383	Q9BPZ7	0.41201
36449	P03383	O60907	0.57097
36450	P03383	Q9ULE6	0.38432
36451	P03383	Q53TQ3	0.26935
36452	P03383	Q96M85	0.33834
36453	P03383	P62316	0.54936
36454	P03383	Q99456	0.75254
36455	P03383	Q13023	0.37428
36456	P03383	Q9NX20	0.33546
36457	P03383	P42025	0.1461
36458	P03383	Q53FD0	0.5752
36459	P03383	O43791	0.36452
36460	P03383	Q9Y295	0.19206
36461	P03383	Q5JPH6	0.25617
36462	P03383	Q9Y2Y9	0.54713
36463	P03383	O15111	0.44973
36464	P03383	P55198	0.63466
36465	P03383	P07864	0.38442
36466	P03383	Q9BXS6	0.46067
36467	P03383	P34896	0.075194
36468	P03383	Q6ZPA2	0.33834
36469	P03383	Q9BUA3	0.37104
36470	P03383	Q96BZ8	0.60341
36471	P03383	Q3ZCV2	0.60341
36472	P03383	P63172	0.56221
36473	P03383	O96000	0.2889
36474	P03383	Q9BXW9	0.54308
36475	P03383	P48382	0.69203
36476	P03383	O75360	0.28149
36477	P03383	A9UHW6	0.46058
36478	P03383	Q9Y535	0.057169
36479	P03383	Q8IXR5	0.21792
36480	P03383	Q8N1Q8	0.34162

P03383	Q14203	0.62011
P03383	Q9BZL1	0.30164
P03383	Q96A73	0.72236
P03383	A1L4L8	0.30872
P03383	Q96H40	0.30584
P03383	Q8N9K3	0.60341
P03383	A8MUU9	0.095415
P03383	Q2M3W8	0.34707
P03383	Q6NZ67	0.48044
P03383	P22674	0.16711
P03383	Q93034	0.46522
P03383	Q7Z3Z0	0.75254
P03383	Q5VXD3	0.018444
P03383	Q9Y2X8	0.3359
P03383	Q9NUC0	0.153
P03383	Q8N4W9	0.24149
P03383	Q9BYQ9	0.5733
P03383	Q96SN8	0.24433
P03383	Q8IVM0	0.31745
P03383	Q00005	0.22943
P03383	Q8N1B3	0.43901
P03383	P56537	0.90223
P03383	Q9UJM3	0.25034
P03383	Q9C086	1.0482
P03383	Q6ZTZ1	0.25245
P03383	O43463	0.60668
P03383	Q99700	0.54587
P03383	Q8N377	0.33834
P03383	Q9UMS6	0.34709
P03383	A8MSI8	0.88257
P03383	O75410	0.56765
P03383	O43734	0.60341
P03383	Q8N5F7	1.02
P03383	Q96LX7	0.037576
P03383	Q9UHU1	0.33834
P03383	P49589	0.25929
P03383	P17482	0.82933
P03383	O60290	0.36381
P03383	Q6ZSR6	0.098428
P03383	P83731	0.49535
P03383	Q9BTX9	0.55213
P03383	O94864	0.29053
P03383	Q5JT78	0.022329
P03383	Q3LI60	0.33834
P03383	Q9H2U6	0.23457
P03383	P62424	0.74
P03383	Q9Y3E7	0.52809
P03383	Q9BUN8	0.15571
P03383	Q6ZN06	0.31056
P03383	Q9H9Y6	0.49609
P03383	A4GXA9	0.51267
P03383	Q6ECI4	0.22485
P03383	Q6ZUT6	0.26594
P03383	P53999	0.98547
P03383	Q9HAH7	0.55102
P03383	Q7Z4H7	0.6011
P03383	Q9NV56	0.62673
P03383	P40306	0.23061
P03383	Q96DE0	0.62439
P03383	Q9UJ98	0.3465
P03383	Q15038	1.0585
P03383	Q9H2W6	0.25393
P03383	Q5VVM6	0.0052024
P03383	P31948	0.64947
P03383	Q96AQ6	0.54191
P03383	Q5T160	0.61098
P03383	A8MUA0	0.2302
P03383	Q8IYB1	0.2071
P03383	Q9UMQ3	0.44155
P03383	P78406	0.31283
P03383	O95644	0.57261
P03383	Q9UFN0	0.38432
P03383	Q6P3V2	0.10773
P03383	P0CAP2	0.24217
P03383	Q9Y4C2	0.55062
P03383	Q2VPJ9	0.60341

P03383	Q8IXQ3	0.26631
P03383	Q71F78	0.26367
P03383	Q9BRP4	0.25575
P03383	Q96LJ8	0.53077
P03383	O75618	0.88774
P03383	Q96GG9	0.22983
P03383	Q6ZQP7	0.33834
P03383	Q8N4M7	0.77454
P03383	P57740	0.56936
P03383	Q9H8Q6	0.47239
P03383	A8MV24	0.23146
P03383	O95886	0.70207
P03383	Q9UI54	0.33834
P03383	Q9H7L9	0.46336
P03383	Q9NRP2	0.54123
P03383	Q8IYW4	0.023122
P03383	Q92905	0.48979
P03383	P49406	0.30622
P03383	Q8N0V3	0.64039
P03383	Q3SY46	0.45194
P03383	Q9UHC1	0.26268
P03383	Q9NUQ3	0.35508
P03383	Q5EE01	0.51663
P03383	Q9UI10	0.19153
P03383	Q9Y4P1	0.32814
P03383	Q5JY77	0.42898
P03383	H3BN30	0.33834
P03383	A6NJ08	0.0088503
P03383	O00233	0.45875
P03383	Q86UT8	0.91463
P03383	Q96SW2	0.33517
P03383	Q9BQ65	0.33056
P03383	Q9H8G2	0.5885
P03383	Q8NHZ7	0.29315
P03383	Q96M61	0.1668
P03383	I3L1I5	0.26907
P03383	Q86U90	0.39677
P03383	A6NLX3	0.20459
P03383	Q5RGN0	0.34297
P03383	O14972	0.21828
P03383	Q9Y3B6	0.54326
P03383	O43678	0.39878
P03383	Q9P000	0.15659
P03383	Q96G28	0.13804
P03383	Q8WWL7	0.69486
P03383	Q4LE28	0.51806
P03383	Q9BZK8	0.33834
P03383	O94811	0.37291
P03383	P18847	0.94154
P03383	Q92966	0.38137
P03383	Q15031	0.25815
P03383	Q9NR56	0.30118
P03383	O60299	0.19718
P03383	Q99689	0.10209
P03383	Q9BZF9	0.37662
P03383	Q8NEU8	0.25909
P03383	Q15004	0.56663
P03383	Q10567	0.4048
P03383	Q8NFH5	0.38809
P03383	Q96P70	0.72362
P03383	Q99576	0.29258
P03383	Q96CW5	0.58999
P03383	Q9BYR4	0.5733
P03383	Q5SQH8	0.03858
P03383	Q9BUR4	0.56535
P03383	O14925	0.21086
P03383	A6NCW3	0.44499
P03383	O43303	0.52394
P03383	Q5SRH9	0.57418
P03383	Q5RHP9	0.22798
P03383	P25205	0.21969
P03383	P60953	0.34615
P03383	Q9H5J4	0.41347
P03383	P18124	0.36949
P03383	Q9UBW8	0.14526
P03383	P82933	0.38972

P03383	A8MWP6	0.33834
P03383	Q6P4D5	0.71616
P03383	Q9UK13	0.32573
P03383	Q86X55	0.50814
P03383	Q7Z6J9	0.53223
P03383	P17861	0.42949
P03383	Q9UBC1	0.28293
P03383	P51508	0.17681
P03383	Q8WYP5	0.47295
P03383	P46778	0.38311
P03383	O95218	0.41487
P03383	Q3LHN2	0.33834
P03383	Q9NZZ3	0.62406
P03383	A8MVW5	0.45516
P03383	Q96KQ4	0.63481
P03383	O43920	0.48907
P03383	Q9BRQ6	0.85069
P03383	P41208	0.36431
P03383	P46013	0.12822
P03383	Q8IZL2	0.61793
P03383	Q96CN0	0.33834
P03383	Q01826	0.54789
P03383	Q8NEG2	0.27707
P03383	P22670	0.54891
P03383	A3KN83	0.73701
P03383	Q8NEY3	0.35531
P03383	Q5H9L4	0.20107
P03383	P20749	0.25855
P03383	Q96BP3	0.15257
P03383	P61604	0.55836
P03383	Q99457	0.42712
P03383	Q96FZ2	0.75514
P03383	Q9BYN7	0.077529
P03383	Q8IYL2	0.33865
P03383	Q5JTD0	0.78434
P03383	Q9NUU6	0.28313
P03383	Q6VY07	0.31703
P03383	Q8WY50	0.33834
P03383	Q9NS73	0.48729
P03383	Q53TS8	0.16652
P03383	O00257	0.74948
P03383	Q8IW19	0.1378
P03383	Q6J272	0.66879
P03383	Q8NHV4	0.39395
P03383	Q9UF83	0.19776
P03383	O43615	0.11289
P03383	Q8N3J9	0.16372
P03383	P62328	0.69335
P03383	Q13490	0.29607
P03383	Q96MY7	0.8473
P03383	Q16836	0.34785
P03383	P19419	0.99837
P03383	Q6ZN17	0.8843
P03383	P52744	0.19314
P03383	P18669	0.53186
P03383	Q96M34	0.024067
P03383	Q9BTE3	0.46531
P03383	Q9BWT1	0.65396
P03383	Q14686	0.71323
P03383	Q9BSF0	0.24104
P03383	Q6STE5	0.63782
P03383	Q8IV36	0.27729
P03383	P28370	0.27924
P03383	B4DX44	0.33034
P03383	P55957	0.23746
P03383	Q96FV9	0.68149
P03383	Q9UBS8	0.48333
P03383	Q75VX8	0.80888
P03383	Q8NEP9	0.29751
P03383	Q8N2N9	0.28525
P03383	Q9H2H8	0.16461
P03383	Q6ZQR2	0.75492
P03383	Q5CZC0	0.8457
P03383	Q6P1M3	0.65729
P03383	P08697	0.2068
P03383	Q96CG3	0.1727

P03383	Q06787	0.64068
P03383	Q9HAV7	0.30962
P03383	Q12972	0.62573
P03383	Q92901	0.38311
P03383	Q96BY7	0.12901
P03383	Q10570	0.59078
P03383	P62753	0.70555
P03383	P02511	0.21985
P03383	Q9UH99	0.053022
P03383	P52655	0.90538
P03383	Q6PI26	0.77654
P03383	Q96B01	0.35757
P03383	O75489	0.057326
P03383	O00186	0.48359
P03383	Q86WV1	0.32371
P03383	P62304	0.521
P03383	P25063	0.33834
P03383	O95177	0.19708
P03383	P30304	0.3727
P03383	Q9NX95	0.2591
P03383	Q9H9Q4	0.20084
P03383	P61201	0.34381
P03383	P48380	0.3329
P03383	Q9NX55	0.33805
P03383	Q96ME1	0.048887
P03383	Q9H9C1	0.43862
P03383	Q15714	0.54694
P03383	Q6ZS49	0.33834
P03383	Q6MZT1	0.46489
P03383	P62937	0.45262
P03383	Q9BQC6	0.57653
P03383	Q96LI6	0.25802
P03383	Q9BV73	0.41414
P03383	Q9UBP6	0.78333
P03383	P62714	0.41756
P03383	P0CE67	0.33834
P03383	Q9P2R7	0.24203
P03383	Q5CZA5	0.2372
P03383	Q8TF20	0.21702
P03383	P23769	0.26778
P03383	Q6A162	0.58359
P03383	Q8NEK8	0.59219
P03383	Q5R3I4	0.1052
P03383	P50222	0.44098
P03383	Q5D1E8	0.40665
P03383	O43399	0.36869
P03383	Q6ZR03	0.11308
P03383	P22392	0.20977
P03383	Q9H503	0.13703
P03383	Q92797	0.50381
P03383	P69905	0.18426
P03383	P13807	0.21003
P03383	P09467	0.12878
P03383	Q9H825	0.061199
P03383	Q6MZP7	0.36433
P03383	Q9NYP9	0.2669
P03383	Q00059	0.66782
P03383	Q9NP81	0.27091
P03383	Q8WYQ5	0.46294
P03383	Q76KX8	0.33683
P03383	Q8TBY8	0.41274
P03383	Q9Y262	0.56261
P03383	Q9BRQ3	0.5578
P03383	O75474	0.88927
P03383	Q92841	0.47154
P03383	Q9H204	0.78879
P03383	A6NCD4	0.20841
P03383	Q9GZT8	0.74675
P03383	Q9NVM9	0.83518
P03383	Q96AQ7	0.45562
P03383	P49006	0.5551
P03383	H0Y354	0.27608
P03383	Q9BYR6	0.81527
P03383	P61326	0.75649
P03383	Q9BQS6	0.54344
P03383	Q6ZRV3	0.33834

P03383	Q9GZV4	0.41834
P03383	Q86WH2	0.016985
P03383	Q2M2I5	0.75254
P03383	Q9HCM1	0.62509
P03383	Q9UHI6	0.21451
P03383	A6NCJ1	0.067006
P03383	Q9UMY1	0.78956
P03383	Q9Y6X4	0.91463
P03383	Q9UMX3	0.36409
P03383	Q9NWT8	0.52302
P03383	P42677	0.60427
P03383	A8MUX0	0.39509
P03383	Q8N443	0.30053
P03383	Q96IZ0	0.52334
P03383	Q0PNE2	0.43907
P03383	Q86WB0	0.90759
P03383	Q96LS8	0.20661
P03383	P37275	0.64771
P03383	Q9BZV1	0.8729
P03383	Q8NDQ6	0.22425
P03383	O75886	0.72414
P03383	Q9Y3Q8	1.0044
P03383	Q9BQ95	0.77137
P03383	Q8WTQ4	0.014525
P03383	Q86X83	0.34832
P03383	Q7Z4U5	0.74733
P03383	Q96MT3	0.52328
P03383	P61573	0.43317
P03383	Q5SNV9	0.21633
P03383	P09972	0.37476
P03383	Q9NWU5	0.48445
P03383	Q9NQ87	0.31389
P03383	P31276	0.41428
P03383	A8MTZ0	0.25726
P03383	Q96N77	0.19192
P03383	O60810	0.44567
P03383	O95831	0.4711
P03383	Q9HC52	0.59783
P03383	P60368	0.23779
P03383	Q6PII3	0.37959
P03383	Q9UL58	0.42296
P03383	Q9H2F5	0.33686
P03383	Q70Z53	0.5491
P03383	Q8WXD5	0.62462
P03383	Q96B23	0.61774
P03383	Q969T9	0.71639
P03383	Q9ULD5	0.12698
P03383	Q9UMS5	0.6867
P03383	P0DL12	0.18124
P03383	O75030	0.41538
P03383	Q8IYT1	0.35017
P03383	Q9ULU4	0.30061
P03383	Q9BZE9	0.46446
P03383	P80297	0.23827
P03383	Q8WWM7	0.3757
P03383	P0DMB2	0.36961
P03383	Q96PU4	0.24131
P03383	Q5JT18	0.33834
P03383	Q06587	0.59897
P03383	Q86V59	0.19876
P03383	Q8N1F7	0.39819
P03383	Q15542	0.7286
P03383	Q9P2F5	0.24307
P03383	Q9UFG5	0.1668
P03383	Q9BZW7	0.20797
P03383	Q9H9L7	0.12396
P03383	B3KS81	0.16526
P03383	P24522	0.36696
P03383	Q6RFH8	0.61677
P03383	O94983	1.002
P03383	O00193	0.80264
P03383	Q96BK5	0.3204
P03383	O14709	0.24149
P03383	Q8ND83	0.77489
P03383	Q15742	0.18237
P03383	Q9Y2S6	0.33834

P03383	P48200	0.19927
P03383	Q6ZRH9	0.33834
P03383	Q5U5X0	0.65386
P03383	Q96KH6	0.36187
P03383	P63000	0.35269
P03383	H3BQW9	0.36947
P03383	Q5T681	0.66417
P03383	P61247	0.85171
P03383	Q9UEU5	0.15388
P03383	Q5T036	0.11528
P03383	O00712	0.080964
P03383	Q9H6V9	0.23371
P03383	P40925	0.32848
P03383	A6NP61	0.5008
P03383	Q92733	0.56939
P03383	D6RGH6	0.97549
P03383	Q96A26	0.12191
P03383	Q9BYC9	0.47531
P03383	Q569G3	0.60341
P03383	Q96GX8	0.60341
P03383	Q6VMQ6	0.78527
P03383	Q8NEP4	0.60341
P03383	P52630	0.46295
P03383	P35638	0.57906
P03383	Q9HC77	0.45253
P03383	Q8IXW5	0.45782
P03383	O00762	0.40178
P03383	Q2M2W7	0.60341
P03383	O75461	0.13662
P03383	O43365	0.28091
P03383	Q06250	0.33834
P03383	Q63HM1	0.15893
P03383	Q9UNQ2	0.36918
P03383	Q5TD94	0.14074
P03383	O60318	0.45099
P03383	Q9HBM6	0.46726
P03383	Q96JB2	0.56494
P03383	Q9BXM0	0.49265
P03383	Q9NS26	0.62663
P03383	P15923	0.42348
P03383	Q6P2Q9	0.40791
P03383	Q8IZL8	0.69234
P03383	Q3MJ62	0.13447
P03383	A8MQ03	0.41323
P03383	Q15468	0.25475
P03383	Q9ULW3	0.67036
P03383	Q9NQ55	0.21665
P03383	Q9BWU0	0.2928
P03383	Q6NVV0	0.47044
P03383	Q9H2G9	0.24296
P03383	P68871	0.16142
P03383	Q6ZSG2	0.28836
P03383	Q5XKK7	0.088594
P03383	Q5H9L2	0.79563
P03383	Q9NS25	0.66241
P03383	Q8IUC3	0.76208
P03383	P53805	0.42528
P03383	Q5JSJ4	0.94404
P03383	Q6PJP8	0.36547
P03383	Q14146	0.33834
P03383	Q9BXV9	0.33834
P03383	Q9BZZ5	0.15662
P03383	Q8NCE0	0.38328
P03383	P61582	0.77258
P03383	Q86VX9	0.79916
P03383	O95347	0.25266
P03383	P0CL81	0.17372
P03383	P38919	0.14204
P03383	Q12798	0.46103
P03383	Q96KR1	0.30486
P03383	Q16520	0.70259
P03383	O95931	0.57698
P03383	Q9UBU8	0.51404
P03383	Q9Y4Y9	0.51576
P03383	Q8WXF5	0.41881
P03383	Q86Y97	0.28151

P03383	Q86UD1	0.24802
P03383	Q86TG7	0.29009
P03383	Q5JNZ5	0.363
P03383	Q1RN00	0.33834
P03383	Q6L8G5	0.33834
P03383	Q05BU3	0.33834
P03383	O75155	0.52483
P03383	P28838	0.42245
P03383	O14490	0.45403
P03383	Q12901	0.2662
P03383	Q14738	0.4796
P03383	Q8N4J0	0.57862
P03383	Q6ZRM9	0.49037
P03383	Q5VY09	0.67106
P03383	O95400	0.53005
P03383	Q00536	0.68884
P03383	Q9C0F3	0.12684
P03383	A4D1E1	0.31015
P03383	P49721	0.27455
P03383	Q06323	0.61001
P03383	O60936	0.68818
P03383	Q3KQU3	0.46689
P03383	Q15532	0.73327
P03383	Q8NEZ2	0.3987
P03383	P83881	0.49283
P03383	Q15121	0.27798
P03383	Q2TAZ0	0.1668
P03383	Q6ZNM6	0.4997
P03383	Q8IXP5	0.60341
P03383	Q9NTX7	0.26925
P03383	O43402	0.56557
P03383	Q8TAB7	0.47353
P03383	O00170	0.43858
P03383	Q9UK39	0.30277
P03383	Q8NDF8	0.24397
P03383	P35789	0.2708
P03383	Q9NZM5	0.31075
P03383	Q16613	0.16013
P03383	Q6ZMV5	0.3037
P03383	P37837	0.37012
P03383	Q99816	0.28392
P03383	Q9NX18	0.67703
P03383	Q96G25	0.83155
P03383	P63313	0.45586
P03383	Q86VP1	0.19477
P03383	P14136	0.52684
P03383	Q9NXE8	0.41307
P03383	Q8N6K4	0.33834
P03383	Q9BZE4	0.1772
P03383	Q96P71	0.24351
P03383	Q9H0B6	0.33771
P03383	Q9UNL4	0.22766
P03383	Q6IPX3	0.59137
P03383	Q9UFF9	0.29692
P03383	Q12874	0.32955
P03383	O95486	0.1654
P03383	Q8TB72	0.33442
P03383	O14646	0.45593
P03383	P0C671	0.014048
P03383	Q9HCH5	0.34293
P03383	Q6ZNA4	0.4212
P03383	Q5TKA1	0.20805
P03383	Q8NA31	0.43439
P03383	Q5MJ10	0.61942
P03383	Q5T8I9	0.070767
P03383	P13645	0.72432
P03383	Q32NC0	0.34182
P03383	Q86YA3	0.67549
P03383	O95059	0.25196
P03383	A6NEY3	0.5567
P03383	P59991	0.72344
P03383	Q5SWW7	0.33834
P03383	O60232	0.70062
P03383	P52434	0.17958
P03383	Q96MY1	0.60498
P03383	Q3BBV2	0.34297

P03383	P24385	0.39817
P03383	Q6PJG2	0.45794
P03383	P61218	0.12454
P03383	Q9Y2G0	0.2485
P03383	A8MSY0	0.094808
P03383	Q9Y6X3	0.74592
P03383	Q9NWN3	0.20112
P03383	Q01850	0.64365
P03383	P26368	0.38796
P03383	Q13243	0.58708
P03383	Q9HCK5	0.4624
P03383	Q8N1N5	0.24722
P03383	Q04323	0.17216
P03383	Q96HJ3	0.60341
P03383	Q8NA42	0.17925
P03383	Q6PK04	0.75992
P03383	O14503	0.67128
P03383	P35573	0.30864
P03383	Q8TDY2	0.63031
P03383	Q6P9G9	0.13995
P03383	P17019	0.32025
P03383	P09001	0.38653
P03383	Q8N8A8	0.28521
P03383	Q8WW14	0.12385
P03383	Q5T0J3	0.066153
P03383	P63162	0.419
P03383	O00165	0.45883
P03383	Q9H5Q4	0.50456
P03383	Q96M29	0.33927
P03383	Q9UKT4	0.66892
P03383	Q9BRP1	0.56999
P03383	Q86WR7	0.59867
P03383	Q8IYI6	0.40155
P03383	Q9H1Q7	0.65475
P03383	Q6NSW5	0.83081
P03383	O43598	0.11298
P03383	Q96FA7	0.66251
P03383	Q8TCS8	0.12686
P03383	Q96QT6	0.70192
P03383	P30153	0.2989
P03383	Q6IQ21	0.23434
P03383	Q14814	0.40792
P03383	Q8WWB5	0.6086
P03383	Q9Y2Z2	0.7176
P03383	Q9BRX2	0.18454
P03383	P26641	0.22842
P03383	Q8NI29	0.14951
P03383	Q5T5A4	0.35878
P03383	Q96N20	0.14779
P03383	Q9Y2Q1	0.311
P03383	Q01860	0.50318
P03383	Q8NA57	0.54492
P03383	Q9Y2B5	0.19401
P03383	Q9BYD3	0.59079
P03383	Q86XK2	0.47966
P03383	Q6ISB3	0.3936
P03383	Q8IYW2	0.33834
P03383	P78317	0.45463
P03383	P47914	0.7479
P03383	Q8IZX4	0.23099
P03383	Q08945	0.8172
P03383	O76011	0.75254
P03383	Q5T653	0.43403
P03383	Q8WYH8	0.32765
P03383	Q9GZL8	0.24095
P03383	P04792	0.25246
P03383	Q8TEV8	0.33834
P03383	Q9C0C2	0.17812
P03383	Q13536	0.33834
P03383	Q96IU2	0.80307
P03383	Q9GZQ3	0.78235
P03383	Q96BN2	0.61543
P03383	Q5HYC2	0.035851
P03383	O60841	0.073454
P03383	Q15797	0.11504
P03383	Q96FF7	0.33834

P03383	Q5W0V3	0.63733
P03383	Q9P0M9	0.434
P03383	Q71UM5	0.36106
P03383	Q8TAD1	0.12615
P03383	P42226	0.55597
P03383	Q15773	0.80511
P03383	Q8IVV7	0.23537
P03383	Q96H22	0.26437
P03383	O75638	1.0032
P03383	Q86X53	0.19721
P03383	Q9P013	0.4475
P03383	Q14997	0.34172
P03383	O60826	0.49497
P03383	P69208	0.33834
P03383	Q9Y2R9	0.65677
P03383	Q9H1A4	0.49416
P03383	P10599	0.269
P03383	P67775	0.45886
P03383	P61978	0.74749
P03383	Q9BYE7	0.56066
P03383	Q8WXF1	0.7775
P03383	Q9Y296	0.11583
P03383	Q9H2C0	0.47824
P03383	Q96JB5	0.20395
P03383	Q9Y6D6	0.37276
P03383	Q96A09	0.4388
P03383	Q9UN36	0.36355
P03383	A2RUQ5	0.082563
P03383	Q9NRN7	0.28269
P03383	Q9NT62	0.27157
P03383	O75691	0.54622
P03383	Q9NVF7	0.58513
P03383	Q7Z7H3	0.0546
P03383	Q96T21	0.48002
P03383	Q8NAB5	0.33834
P03383	Q96N95	0.086077
P03383	P11387	0.46055
P03383	P26196	0.33622
P03383	Q9P2K3	0.88369
P03383	P86481	0.33834
P03383	O75177	0.59109
P03383	Q75QN2	0.49915
P03383	Q03923	0.34049
P03383	Q96LM1	0.41221
P03383	B4DZS4	0.11576
P03383	Q96RK1	0.39475
P03383	Q9NUQ6	0.11945
P03383	Q8N988	0.32881
P03383	Q7Z3Y8	0.66284
P03383	Q8WYN3	0.40328
P03383	Q14331	0.34388
P03383	P18846	0.63447
P03383	Q5SWA1	0.46372
P03383	Q9NVE4	0.13332
P03383	O43189	0.27035
P03383	O76014	0.66284
P03383	O15405	0.70668
P03383	Q96B36	0.50844
P03383	Q8N999	0.42086
P03383	P11940	0.27979
P03383	P51816	0.38786
P03383	Q14152	0.82125
P03383	Q9GZS1	0.1198
P03383	Q9BVS5	0.17863
P03383	P31943	0.35146
P03383	P63208	0.39296
P03383	Q6ZP68	0.57381
P03383	Q9NPE3	0.49875
P03383	Q495Z4	0.19554
P03383	O00505	0.66305
P03383	P62917	0.63174
P03383	Q92993	0.72836
P03383	Q86V15	0.30117
P03383	Q8N9G6	0.56973
P03383	Q9UI43	0.50668
P03383	Q9UQF2	0.27664

P03383	Q9ULR5	0.2846
P03383	Q9NP92	0.69228
P03383	Q9NWV4	0.50558
P03383	O75400	0.68175
P03383	Q3MIS6	0.32573
P03383	Q99459	0.94122
P03383	O75151	0.43849
P03383	Q96SE7	0.23295
P03383	Q1W209	0.33834
P03383	Q58A44	0.33834
P03383	A6NIN4	0.33834
P03383	Q969V6	1.0558
P03383	P49321	0.61021
P03383	P61575	0.43317
P03383	Q9BYT5	0.15594
P03383	P49005	0.34268
P03383	Q7Z614	0.25113
P03383	Q86XF7	0.39973
P03383	Q9Y592	0.62396
P03383	Q05516	0.52347
P03383	Q96DM3	0.069471
P03383	Q96K30	0.51932
P03383	A6QL64	0.28525
P03383	P82914	0.5175
P03383	P28698	0.4992
P03383	Q9GZX5	0.58898
P03383	P49247	0.46088
P03383	Q9Y4R8	0.14749
P03383	Q13405	0.4211
P03383	P12980	0.58625
P03383	Q8N693	0.2727
P03383	Q8N402	0.027281
P03383	P62273	0.51119
P03383	Q8TB69	0.29921
P03383	A6NIE6	0.36825
P03383	Q07352	0.66288
P03383	Q86V81	0.60787
P03383	Q8TEM1	0.15551
P03383	Q86V87	0.32142
P03383	O94888	0.22759
P03383	Q86VE0	0.86884
P03383	Q15306	0.55864
P03383	Q5U623	0.5513
P03383	P0CG12	0.91651
P03383	P0C7X2	0.38684
P03383	Q6NVV7	0.15634
P03383	O15297	0.35567
P03383	Q8NDZ2	0.16291
P03383	O14734	0.36864
P03383	Q9P0L1	0.20528
P03383	Q9NPJ6	0.74375
P03383	O60220	0.33471
P03383	Q96K17	0.47518
P03383	P53675	0.17038
P03383	Q15651	0.66382
P03383	P17038	0.2288
P03383	Q9Y4E1	0.34908
P03383	O60573	0.31482
P03383	P07900	0.45459
P03383	Q2KHT3	0.57772
P03383	Q6ZW49	0.2232
P03383	Q6P9B6	0.1668
P03383	Q92782	0.36251
P03383	Q9NUS5	0.2762
P03383	Q7Z7K6	0.38795
P03383	Q5VV17	0.023322
P03383	Q8IYE0	0.57342
P03383	Q6PGQ1	0.33684
P03383	Q14690	0.34017
P03383	Q5JR98	0.39103
P03383	Q96T23	0.48513
P03383	P49754	0.27442
P03383	Q96DX5	0.76416
P03383	Q96S55	0.21327
P03383	Q15847	0.33834
P03383	P05783	1.046

P03383	O14990	0.4616
P03383	B1AH88	0.33834
P03383	Q86TU7	0.12512
P03383	Q8N655	0.33834
P03383	Q9H6R4	0.34438
P03383	Q13352	0.24109
P03383	Q13435	0.51868
P03383	Q96PS1	0.41538
P03383	Q15050	0.52164
P03383	O43493	0.50117
P03383	Q7RTU5	0.28657
P03383	Q92597	0.25557
P03383	Q00978	0.67184
P03383	Q9BZA5	0.52191
P03383	Q13330	0.25779
P03383	Q5T200	0.6202
P03383	Q92546	0.71084
P03383	Q5SY85	0.18846
P03383	P61457	0.78196
P03383	Q969X6	1.0329
P03383	Q53GS7	0.45144
P03383	Q5JQS6	0.22395
P03383	P30740	0.24939
P03383	P15621	0.31671
P03383	Q00534	0.46937
P03383	A6NK53	0.20961
P03383	Q96KS0	0.049218
P03383	Q15631	0.45197
P03383	Q8N865	0.13476
P03383	Q15653	0.6727
P03383	Q86YE8	0.24149
P03383	Q9UNX3	0.69662
P03383	Q9BZE1	0.31874
P03383	Q8NFV5	0.38945
P03383	Q8WV37	0.31046
P03383	O15061	0.54102
P03383	Q9HAH1	0.26656
P03383	Q7L0Y3	0.68031
P03383	Q08752	0.20569
P03383	P63241	0.30624
P03383	A6NF83	0.46548
P03383	Q96L21	0.33605
P03383	Q3LI76	0.57432
P03383	Q9ULW8	0.25286
P03383	Q8N2W9	0.80963
P03383	Q96EA4	0.47865
P03383	Q9Y2W2	0.59296
P03383	P35813	0.081222
P03383	P20618	0.47769
P03383	O43324	0.81388
P03383	O95567	0.33834
P03383	Q6NZY4	0.43599
P03383	Q9H1Y0	0.32604
P03383	Q9NQP4	0.59299
P03383	P51504	0.18444
P03383	Q04724	0.6551
P03383	Q8N587	0.3011
P03383	Q96PI1	0.61945
P03383	Q9H7N4	0.63339
P03383	P15408	0.38203
P03383	Q9H6W3	0.28134
P03383	P78337	0.2673
P03383	Q9H321	0.66758
P03383	Q9UHD4	0.51266
P03383	Q9BUH6	0.36337
P03383	Q8NAV1	0.65788
P03383	Q5VVH5	0.35212
P03383	Q6ZR52	0.2288
P03383	O00189	0.13687
P03383	Q96NT3	0.21382
P03383	Q8TAI1	0.21185
P03383	Q96EK4	0.93454
P03383	Q3LI73	0.56255
P03383	P09132	0.10466
P03383	Q13064	0.10937
P03383	O60216	0.55893

P03383	Q969Q0	0.08152
P03383	Q8NAV2	0.22192
P03383	Q9UKT7	0.24312
P03383	Q9Y5Q8	0.43554
P03383	Q8N9R6	0.22617
P03383	Q8TE85	0.093429
P03383	Q15560	0.77498
P03383	Q8TD31	0.46193
P03383	Q13158	0.25336
P03383	Q96MF7	0.33532
P03383	O43156	0.48625
P03383	Q6ZSJ8	0.60341
P03383	Q5VWQ0	0.95004
P03383	Q495Y8	0.38945
P03383	Q92665	0.53398
P03383	Q6P1K8	0.15655
P03383	P78537	0.69407
P03383	Q13625	0.28966
P03383	Q13042	0.93887
P03383	Q6PIJ6	0.61158
P03383	A1A580	0.56255
P03383	P31689	0.36356
P03383	O75683	0.35845
P03383	Q9H1D9	0.31845
P03383	Q93074	0.35402
P03383	P02795	0.3404
P03383	Q96LV5	0.03132
P03383	Q9Y248	0.46992
P03383	Q9BYP9	0.5733
P03383	P07737	0.63382
P03383	Q8N5U6	0.77047
P03383	Q5XG85	0.33834
P03383	Q6P1W5	0.36078
P03383	P59923	0.24149
P03383	P17098	0.33436
P03383	Q13155	0.4049
P03383	Q8NCU4	0.084955
P03383	Q9UKF6	0.52969
P03383	Q9BYR7	0.7963
P03383	Q9H175	0.80214
P03383	Q96LR7	0.54842
P03383	Q9NUP1	0.4215
P03383	Q01831	0.3875
P03383	Q96KP1	0.22249
P03383	O00321	0.16138
P03383	O15117	0.50336
P03383	Q9NRX2	0.29849
P03383	P55209	0.4873
P03383	O00267	0.86242
P03383	Q9NQX1	0.33997
P03383	Q9BYQ3	0.5733
P03383	Q6P5X5	0.48525
P03383	Q6L8G9	0.33834
P03383	P38432	0.50932
P03383	Q13432	0.27384
P03383	Q5TYM5	0.15914
P03383	Q5SYE7	0.45597
P03383	Q9Y2A0	0.23318
P03383	Q7Z6I5	0.60689
P03383	Q14684	0.34203
P03383	Q96GX2	0.64555
P03383	Q6X4W1	0.79373
P03383	Q9H2L5	0.44518
P03383	Q6ZQY7	0.30824
P03383	P14635	0.18458
P03383	P0C853	0.33834
P03383	Q15022	0.43419
P03383	P59052	0.21884
P03383	Q9H693	0.5298
P03383	Q9UHT9	0.33834
P03383	B4DU55	0.2372
P03383	Q12983	0.026812
P03383	Q9P278	0.58795
P03383	Q8N5U0	0.33834
P03383	Q9BZE2	0.80152
P03383	P53677	0.033782

P03383	Q9Y3D0	0.70685
P03383	Q6NSZ9	0.088669
P03383	Q9BRS8	0.27809
P03383	Q6PI98	0.40379
P03383	A6NEH8	0.046454
P03383	Q3KP66	0.79785
P03383	Q8TDC0	0.28435
P03383	P0CI00	0.43604
P03383	Q6NUR6	0.48593
P03383	Q32P41	0.18019
P03383	P30050	0.49535
P03383	Q86UU0	0.83827
P03383	Q5VT06	0.31889
P03383	Q5VUG0	0.1228
P03383	Q9Y3F1	0.33834
P03383	Q9Y3B2	0.54335
P03383	Q9NWQ4	0.54249
P03383	Q9Y692	0.48781
P03383	Q9BXM8	0.33834
P03383	Q96MT8	0.70621
P03383	Q17RM4	0.33496
P03383	Q16385	0.56798
P03383	P11802	0.62371
P03383	P06400	0.50919
P03383	P46939	0.32312
P03383	O14744	0.72312
P03383	O43318	0.42025
P03383	O75190	0.52682
P03383	F5H4B4	0.59139
P03383	P18146	0.50432
P03383	Q08AG7	0.45866
P03383	Q7L1V2	0.70883
P03383	Q6RSH7	0.21988
P03383	Q9NX08	0.1668
P03383	O94906	0.19898
P03383	O75306	0.25446
P03383	Q13522	0.057511
P03383	P41162	0.23395
P03383	Q9BTY7	0.17715
P03383	P48147	0.31542
P03383	P10155	0.28038
P03383	Q92558	0.1575
P03383	Q96EV8	0.11719
P03383	Q52M58	0.43574
P03383	Q9NTW7	0.31457
P03383	Q9UIL1	0.36264
P03383	Q15436	0.3937
P03383	Q6L8G4	0.33834
P03383	Q8N9H9	0.33834
P03383	A6ZKI3	0.27506
P03383	P84098	0.38311
P03383	P31350	0.19742
P03383	Q4V328	0.51034
P03383	P49750	0.88422
P03383	Q13398	0.31353
P03383	Q66K89	0.65026
P03383	Q53GI3	0.1769
P03383	Q9UK11	0.25101
P03383	Q8WW12	0.51673
P03383	O15525	0.19127
P03383	Q8NAQ8	1.1544
P03383	Q9Y3E5	0.29843
P03383	Q9H9F9	0.85732
P03383	Q96BT3	0.29531
P03383	A6NJ78	0.59219
P03383	Q9UBB5	0.8746
P03383	Q9Y697	0.40956
P03383	Q13618	0.27705
P03383	Q8IZU1	0.47691
P03383	P78396	0.65319
P03383	Q9Y2T7	0.075014
P03383	Q8NEG0	0.10277
P03383	P38159	0.47278
P03383	Q9NP71	0.32571
P03383	A2A288	0.28208
P03383	A6NJJ6	0.60341

P03383	O95178	0.42264
P03383	Q49AA0	0.30761
P03383	Q9NQ29	0.55324
P03383	Q96D46	0.6757
P03383	Q14525	0.66522
P03383	P78386	0.66532
P03383	A8MUQ0	0.6925
P03383	E5RJ46	0.33834
P03383	Q9Y6G3	0.05576
P03383	Q12769	0.78318
P03383	Q6IQ32	0.35268
P03383	A8MV72	0.27727
P03383	Q8N2H4	0.82018
P03383	Q9NYF3	0.32241
P03383	Q8TAF7	0.31865
P03383	Q8WUD4	0.062356
P03383	Q96R06	0.64689
P03383	A4D161	0.055158
P03383	O75663	0.25995
P03383	A8MZA4	0.32695
P03383	Q8N612	0.60978
P03383	Q9BQ89	0.76967
P03383	O75394	0.38425
P03383	Q8IWE4	0.22547
P03383	Q537H7	0.17814
P03383	Q9Y3A2	0.62937
P03383	Q6ZV60	0.33834
P03383	Q9NZP6	0.71685
P03383	Q6ZWC4	0.11527
P03383	O60535	0.24348
P03383	Q96DB5	0.2776
P03383	Q01664	0.38423
P03383	Q9NX46	0.19736
P03383	O95049	0.24992
P03383	Q5T4H9	0.33834
P03383	Q9NVU0	0.23883
P03383	Q9BY44	0.24886
P03383	O60921	0.54578
P03383	Q96CN9	0.303
P03383	Q9UK76	0.60274
P03383	P31249	0.34283
P03383	A9QM74	0.27061
P03383	Q6ZPB5	0.33834
P03383	Q8N7M0	0.19257
P03383	Q9BV57	0.36728
P03383	Q9C0F1	0.041184
P03383	Q92870	0.47417
P03383	Q9NSQ0	0.26288
P03383	P61077	0.3359
P03383	Q5BKY9	0.60341
P03383	Q15654	0.57212
P03383	O96002	0.33834
P03383	Q8TES7	0.37934
P03383	Q96B49	0.63601
P03383	Q96N53	0.33834
P03383	Q9HCH0	0.9661
P03383	Q9NZY2	0.33834
P03383	Q6PCE3	0.33313
P03383	Q86X02	0.1999
P03383	P21127	0.36636
P03383	Q6UYE1	0.67868
P03383	A6NKF1	0.68578
P03383	Q8IYI8	0.24216
P03383	Q96HR3	0.61322
P03383	Q9BQ52	0.20607
P03383	P60014	0.7585
P03383	Q5W0A0	0.15929
P03383	Q8TCH9	0.12087
P03383	Q7Z7L8	0.7036
P03383	Q14872	0.63102
P03383	Q969Z0	0.39666
P03383	Q9NTI7	0.57392
P03383	O14602	0.25537
P03383	O75164	0.74494
P03383	Q96IR3	0.50694
P03383	Q9H1H9	0.33736

P03383	Q69YN4	0.27841
P03383	Q658L1	0.59283
P03383	Q00403	0.76117
P03383	P36873	0.52145
P03383	Q96G21	0.61496
P03383	Q13542	0.35475
P03383	Q8NDZ9	0.33834
P03383	Q9BQI9	0.6172
P03383	P62826	0.5054
P03383	Q49AS3	0.13592
P03383	P55347	0.28459
P03383	Q9BSU3	0.24483
P03383	Q9HAJ7	0.84446
P03383	A8K8P3	0.52817
P03383	Q07157	0.41446
P03383	P32969	0.58612
P03383	Q99618	0.81447
P03383	Q96LL4	0.60341
P03383	O43913	0.27382
P03383	Q6PGP7	0.48277
P03383	Q9BW71	0.64499
P03383	Q9UKJ3	0.47389
P03383	A6NI87	0.46078
P03383	P11168	0.23327
P03383	Q14533	0.66532
P03383	Q8ND99	0.012509
P03383	Q9BYN8	0.72117
P03383	Q9Y2L8	0.37583
P03383	Q8N6M0	0.076041
P03383	Q8WTW3	0.53753
P03383	P30279	0.44411
P03383	Q8NBC4	0.33834
P03383	Q9NVS2	0.54418
P03383	Q09161	0.58293
P03383	O95628	0.49535
P03383	Q9BTD1	0.14691
P03383	P0C7V9	0.59219
P03383	O43447	0.4168
P03383	Q6PD74	0.27213
P03383	Q9BXL5	0.20683
P03383	Q6ZRC1	0.33834
P03383	P41218	0.30049
P03383	Q16629	0.45766
P03383	Q9ULD0	0.16037
P03383	P01358	0.33834
P03383	Q6ZUG5	0.12609
P03383	Q6ZVL8	0.33834
P03383	Q8NHG7	0.48924
P03383	P17014	0.17144
P03383	Q14588	0.24149
P03383	P33552	0.37959
P03383	P83916	0.50876
P03383	Q9HB65	0.3402
P03383	Q7Z7F1	0.067937
P03383	P25208	0.42791
P03383	Q14CW9	0.6362
P03383	Q8IXQ6	0.53775
P03383	Q9NRJ2	0.33834
P03383	Q8N228	0.073272
P03383	Q8N9L7	0.055996
P03383	B3EWF7	0.33834
P03383	Q7Z3V5	0.23295
P03383	Q9NQG5	0.82645
P03383	Q86WR0	0.18037
P03383	Q9Y6C7	0.33834
P03383	P59036	0.33834
P03383	Q5VWG9	0.56068
P03383	P13985	0.33834
P03383	H3BNL1	0.223
P03383	Q5TAX3	0.48697
P03383	Q8TEP8	0.49613
P03383	Q96E09	0.58513
P03383	Q9H0E9	0.17681
P03383	Q9NV39	0.33261
P03383	Q6WBX8	0.24728
P03383	P20042	0.26771

P03383	Q86Y26	0.53361
P03383	Q9BZQ8	0.43039
P03383	Q71F23	0.31743
P03383	Q96LW9	0.14737
P03383	P61581	0.5351
P03383	Q9Y3T9	0.81422
P03383	Q9H0A8	0.49238
P03383	P20226	0.71928
P03383	Q8TAF5	0.33834
P03383	P49366	0.22692
P03383	Q8N365	0.60341
P03383	Q9NYK6	0.42497
P03383	A6NJ64	0.27451
P03383	Q8TE76	0.078671
P03383	Q96A04	0.68207
P03383	P09913	0.33311
P03383	Q9ULK2	0.48392
P03383	O43159	0.66304
P03383	Q8IUF8	0.23162
P03383	A8MUV8	0.31046
P03383	A6NKX1	0.33834
P03383	A8K8V0	0.22149
P03383	Q8IVN3	0.64611
P03383	Q9UBS4	0.29819
P03383	Q96EB1	0.8575
P03383	Q9HBF5	0.62314
P03383	Q8TF47	0.31865
P03383	Q8N5C8	0.26097
P03383	Q33E94	0.61509
P03383	Q5VU43	0.61008
P03383	Q6ZVD7	0.095456
P03383	Q8IWP9	0.81539
P03383	P62136	0.46451
P03383	Q99567	0.54894
P03383	A6NEQ9	0.33834
P03383	Q9UBW7	0.57722
P03383	O95201	0.39205
P03383	Q9NXF7	0.14284
P03383	Q96D70	0.71373
P03383	Q9UPR3	0.66239
P03383	O95707	0.72937
P03383	P82675	0.37909
P03383	Q5SWL7	0.44316
P03383	Q14687	0.1668
P03383	Q8NCT3	0.64342
P03383	P61088	0.48813
P03383	Q8IXH6	0.82345
P03383	Q8N9E0	0.60341
P03383	Q8NFG4	0.47549
P03383	O75800	0.23613
P03383	Q12834	0.60316
P03383	Q8IYX7	0.75608
P03383	Q8IZT9	0.61185
P03383	Q13895	0.4515
P03383	Q4KMZ1	0.048304
P03383	P52758	0.21757
P03383	Q9HC44	1.1511
P03383	O75901	0.3802
P03383	Q6IPW1	0.36194
P03383	P35527	0.75254
P03383	Q9HAZ2	0.24056
P03383	A6NLC5	0.24946
P03383	Q6P1N0	0.17733
P03383	A6NEF3	0.5567
P03383	Q9BWD3	0.44385
P03383	Q16719	0.070377
P03383	Q5VWX1	0.50571
P03383	Q96GX9	0.20394
P03383	O14770	0.28309
P03383	Q9Y5P8	0.37386
P03383	P56282	0.22758
P03383	Q96L92	0.48375
P03383	Q66PJ3	0.39841
P03383	Q9NZH4	0.55015
P03383	Q6W2J9	0.27366
P03383	Q8N485	0.072429

P03383	Q9Y6Z2	0.33834
P03383	Q7Z589	0.74898
P03383	Q9UQR0	0.096658
P03383	P57077	0.46601
P03383	Q4G112	0.11658
P03383	Q13495	0.93169
P03383	Q9H9A7	0.3518
P03383	Q13287	0.35088
P03383	Q08E93	0.33834
P03383	Q5JPI3	0.5885
P03383	P08651	0.088024
P03383	Q76KD6	0.24027
P03383	O75832	0.31384
P03383	Q9HCG1	0.25769
P03383	O75427	0.30737
P03383	P52657	0.88878
P03383	Q53H80	0.22324
P03383	Q3BBV1	0.95616
P03383	O14530	0.24124
P03383	A1A4V9	0.15604
P03383	Q6PGN9	0.5903
P03383	P52746	0.047743
P03383	Q15006	0.032464
P03383	Q504T8	0.13465
P03383	A6NJ88	0.59022
P03383	Q9UQB3	0.37117
P03383	Q9UBC9	0.3509
P03383	Q14934	0.2798
P03383	P43360	0.39464
P03383	Q9H6J7	0.20788
P03383	P08779	0.72283
P03383	Q5VWT5	0.3937
P03383	Q9UI66	0.33834
P03383	O43516	0.060347
P03383	Q8TCI5	0.37598
P03383	Q8IYF3	0.30354
P03383	Q13137	0.71812
P03383	Q13506	0.19422
P03383	Q0VF49	0.33834
P03383	Q499Z4	0.21286
P03383	Q8N831	0.42364
P03383	Q96RN5	1.0418
P03383	A6NDX5	0.23283
P03383	Q7L2R6	0.29921
P03383	Q99733	0.5089
P03383	O60308	0.36695
P03383	O75448	0.34135
P03383	Q96N06	0.45388
P03383	P62277	0.7914
P03383	Q96GM5	0.63021
P03383	Q8N8G2	0.57276
P03383	Q75NE6	0.93016
P03383	Q9BTE6	0.48531
P03383	Q6RUI8	0.37989
P03383	O00401	0.41938
P03383	O96004	0.34936
P03383	Q8WTS6	0.30884
P03383	Q92552	0.46751
P03383	P62487	0.2499
P03383	O15069	0.10449
P03383	O75529	0.39315
P03383	Q99871	0.30651
P03383	Q6ZSN1	0.29008
P03383	Q14397	0.48039
P03383	Q7L266	0.12118
P03383	P07738	0.52163
P03383	Q86WQ0	0.3119
P03383	Q8NFR7	0.55416
P03383	Q15643	0.5604
P03383	Q5TZA2	0.52358
P03383	P46100	0.3706
P03383	Q6ZV77	0.33834
P03383	Q05084	0.17836
P03383	Q9Y613	0.31796
P03383	O75570	0.3023
P03383	Q13144	0.33322

P03383	Q86TS7	0.33834
P03383	Q8WTP9	0.8942
P03383	Q9H0A9	0.94531
P03383	Q5SY13	0.33834
P03383	Q15326	0.92206
P03383	Q9Y5A7	0.45161
P03383	P29372	0.33973
P03383	A6NFQ2	0.48647
P03383	Q8N9U9	0.056707
P03383	P78560	0.33407
P03383	Q9BYD6	0.39651
P03383	Q16822	0.049138
P03383	P11473	0.24448
P03383	P18077	0.30461
P03383	Q8IUR0	0.68231
P03383	O15105	0.19166
P03383	Q96NB1	0.11192
P03383	P0DM63	0.50049
P03383	Q15003	0.54458
P03383	Q8N684	0.32421
P03383	Q9HD34	0.41203
P03383	Q9H6Q4	0.27335
P03383	Q6ICG7	0.33834
P03383	O14558	0.50268
P03383	O43395	0.58591
P03383	P51610	0.30974
P03383	Q6ZRQ5	0.33602
P03383	Q12796	1.235
P03383	Q14C87	0.44686
P03383	P01009	0.26512
P03383	Q1A5X6	0.60341
P03383	Q96E16	0.48696
P03383	Q6ZS46	0.64499
P03383	Q9BSM1	0.77687
P03383	Q6P3X8	0.30823
P03383	O60870	0.51016
P03383	Q6P1L8	0.65678
P03383	O94913	0.13499
P03383	Q9NQ89	0.21458
P03383	P23588	0.19825
P03383	Q17RH7	0.33834
P03383	Q6PF05	0.24529
P03383	P06730	0.34871
P03383	P63173	0.45875
P03383	Q9Y3D9	0.16984
P03383	Q8IYB3	0.48669
P03383	Q8N8I6	0.33834
P03383	Q5VUA4	0.23115
P03383	P41161	0.013187
P03383	P11474	0.26794
P03383	Q9NR50	0.33026
P03383	Q9UBS3	0.38908
P03383	O75817	0.59897
P03383	Q16533	0.38015
P03383	Q6ZNA1	0.23295
P03383	Q99549	0.46349
P03383	Q496A3	0.15899
P03383	Q86UD0	0.33711
P03383	Q16851	0.2473
P03383	Q03112	0.15085
P03383	P49336	0.48027
P03383	O14978	0.21808
P03383	Q96NS8	0.094778
P03383	Q9NX04	0.26022
P03383	Q6TFL3	0.18306
P03383	O00291	0.23843
P03383	O95243	0.42565
P03383	Q86TA1	0.14228
P03383	Q9H8M7	0.53501
P03383	Q9HAN9	0.23402
P03383	Q2NL82	0.33677
P03383	Q9NSA3	0.49265
P03383	Q13952	0.50221
P03383	Q16594	0.40955
P03383	Q5TB80	0.58135
P03383	Q01892	0.20165

P03383	Q8WU49	0.33834
P03383	P01008	0.22058
P03383	O15015	0.39205
P03383	Q12873	0.38325
P03383	Q5W111	0.016307
P03383	Q92985	0.37021
P03383	P86478	0.33834
P03383	Q9C0B2	0.17117
P03383	O60296	0.38217
P03383	Q14186	1.0451
P03383	Q96EZ4	0.33834
P03383	Q9UII2	0.30026
P03383	Q13428	0.81935
P03383	Q9BSV6	0.18903
P03383	Q96CS2	0.33094
P03383	Q8NHX4	0.5308
P03383	Q6P2C8	0.74735
P03383	P60006	0.48631
P03383	Q6PH81	0.22249
P03383	Q9Y606	0.76709
P03383	Q2M2Z5	0.44549
P03383	Q8IYX0	0.3204
P03383	Q9UET6	0.47258
P03383	P0C843	0.21302
P03383	Q5W5X9	0.29678
P03383	Q9H816	0.23652
P03383	Q8N1K5	0.37572
P03383	P06744	0.4751
P03383	P54252	0.3268
P03383	Q9GZP8	0.33834
P03383	P27816	0.21386
P03383	Q15629	0.27984
P03383	Q7L2K0	0.045303
P03383	Q53HL2	0.67058
P03383	A8MXT2	4.9042e-005
P03383	Q8IXH7	0.93682
P03383	Q9H3H9	0.6782
P03383	Q68DK7	0.55012
P03383	Q9Y316	0.33206
P03383	Q16637	0.63691
P03383	O94763	0.60294
P03383	P0C867	0.15988
P03383	Q13427	0.20514
P03383	Q8NB50	0.25401
P03383	P59998	0.094996
P03383	Q16621	0.38174
P03383	Q8NAP1	0.60341
P03383	O94927	0.32838
P03383	Q9BQ66	0.5733
P03383	P78543	0.45785
P03383	Q96T17	0.41995
P03383	Q96RM1	0.33834
P03383	Q9NVI1	0.42473
P03383	O95777	0.41529
P03383	Q5U4N7	0.33834
P03383	Q7L9L4	0.46714
P03383	Q8WW18	0.60341
P03383	P54845	0.13359
P03383	P15927	0.25179
P03383	O60783	0.4007
P03383	Q9BQ70	1.0273
P03383	Q6NXP2	0.75233
P03383	B3EWG4	0.60341
P03383	Q9Y3R5	0.44747
P03383	Q13185	0.61997
P03383	P62081	0.75519
P03383	P41219	0.44705
P03383	Q9P260	0.0039245
P03383	P86452	0.25315
P03383	Q96PG8	0.33834
P03383	O75607	0.47046
P03383	A7MD48	0.48773
P03383	Q8WUZ0	0.52012
P03383	Q5T6F2	0.74962
P03383	O95229	0.53556
P03383	Q9BVC6	0.40061

P03383	Q9H963	0.27909
P03383	Q1ED39	0.80264
P03383	Q9BW27	0.55424
P03383	P45973	0.48918
P03383	Q96MR9	0.28623
P03383	P49639	0.17743
P03383	Q8IZS5	0.31126
P03383	Q9UHX1	0.49221
P03383	P55060	0.65876
P03383	P84022	0.37919
P03383	Q9BU64	0.26217
P03383	O75956	0.19022
P03383	Q96AB3	0.40163
P03383	Q8TEL6	0.26075
P03383	P98168	0.48913
P03383	B4DXR9	0.31563
P03383	Q5HY64	0.019547
P03383	Q9UHQ1	0.50701
P03383	Q9NV44	0.014553
P03383	Q8IYX3	0.71415
P03383	Q92994	0.15885
P03383	Q9H160	0.30114
P03383	Q04726	0.75722
P03383	Q1RMZ1	0.37483
P03383	Q8IWS0	0.2729
P03383	Q8N895	0.30058
P03383	Q86SE9	0.75358
P03383	O75081	0.865
P03383	P25791	0.24972
P03383	P52292	0.66519
P03383	A6NDD8	0.34297
P03383	B1AK76	0.74678
P03383	P52747	0.59231
P03383	Q9Y6Y0	0.23784
P03383	Q8IXS0	0.60341
P03383	P46976	0.28792
P03383	P19387	0.35386
P03383	Q5H9J9	0.1699
P03383	Q66K80	0.33834
P03383	Q9NXB0	0.54995
P03383	P33240	0.37946
P03383	Q9Y2Q9	0.35355
P03383	Q9HB71	0.34483
P03383	Q2M3C7	0.30068
P03383	Q15928	0.35013
P03383	P11308	0.41605
P03383	Q9HD15	0.36293
P03383	P81274	0.3495
P03383	P62314	0.62088
P03383	Q8IWE2	0.5911
P03383	Q9H489	0.32581
P03383	Q96PK6	0.56411
P03383	Q6ZTC4	0.33834
P03383	O60238	0.17849
P03383	Q9UJK0	0.73592
P03383	Q9Y4K1	0.54734
P03383	Q8WZA8	0.33834
P03383	Q9NVV9	0.79362
P03383	Q9H583	0.72238
P03383	P62310	0.47978
P03383	Q9BY65	0.21793
P03383	P05787	1.0818
P03383	Q9H808	0.31176
P03383	Q04695	0.70649
P03383	Q15361	0.51853
P03383	Q86WS4	0.33834
P03383	P18583	0.23587
P03383	A6NEM1	0.52432
P03383	O60563	1.1739
P03383	A6NGD5	0.15179
P03383	Q96ND8	0.32972
P03383	Q9UDV6	0.3617
P03383	A4D0Y5	0.33834
P03383	Q9H7B2	0.62437
P03383	Q99747	0.31774
P03383	B1ANY3	0.26752

P03383	Q9BT78	0.7127
P03383	Q66GS9	0.070043
P03383	Q9C035	0.35674
P03383	Q7Z2Z1	0.64538
P03383	Q9Y221	0.55046
P03383	Q8WVC0	0.75365
P03383	Q00994	0.69539
P03383	P0C841	0.33834
P03383	Q53EQ6	0.32213
P03383	P61580	0.5351
P03383	Q13111	1.0167
P03383	Q9NXK8	0.37695
P03383	Q6NV74	0.60341
P03383	P84103	0.43188
P03383	Q9UID6	0.32141
P03383	O75558	0.32299
P03383	Q9BTM9	0.58685
P03383	A6NJL1	0.084369
P03383	P06132	0.39824
P03383	Q6ZN28	0.4839
P03383	Q8NEM1	0.32025
P03383	Q96BQ5	0.44531
P03383	Q8WUE5	0.16657
P03383	Q96A49	0.68833
P03383	Q9NV72	0.31091
P03383	Q9NTG7	0.28032
P03383	Q96N16	0.62616
P03383	Q86UY5	0.064539
P03383	Q8N4B4	0.053608
P03383	Q5QJE6	0.79773
P03383	Q9H8T0	0.28398
P03383	Q5JNZ3	0.2283
P03383	Q9Y2R5	0.37779
P03383	Q96N38	0.31271
P03383	Q86SQ7	0.61546
P03383	Q8IVE0	0.018533
P03383	Q9Y2J4	0.13623
P03383	Q92945	0.52477
P03383	Q9ULG3	0.33834
P03383	Q9UK41	0.447
P03383	Q96AQ9	0.25258
P03383	O97980	0.034227
P03383	Q7LBR1	0.61902
P03383	Q15652	0.2875
P03383	Q12857	0.13416
P03383	P17010	0.084994
P03383	P50613	0.15891
P03383	Q75L30	0.33834
P03383	A8MVM7	0.33834
P03383	Q9UHK0	0.18971
P03383	Q8NHU0	0.069402
P03383	Q9BXY8	0.39095
P03383	P13984	0.39456
P03383	Q96KN1	0.22673
P03383	Q9UNY5	0.31738
P03383	Q8IYA6	0.57502
P03383	Q9H9H5	0.35518
P03383	Q32M78	0.28869
P03383	Q8N9N5	0.27636
P03383	Q96FN9	0.33484
P03383	Q96MK2	0.081085
P03383	Q9UI72	0.33834
P03383	Q86UC2	0.30443
P03383	Q9P031	0.81631
P03383	P39687	1.0796
P03383	Q96MF4	0.36489
P03383	O60260	0.27859
P03383	P42772	0.58894
P03383	Q8TAY7	1.0726
P03383	P23381	0.22013
P03383	Q6P9F0	0.19357
P03383	Q86VQ0	0.1928
P03383	P20962	0.33415
P03383	Q99551	0.49491
P03383	Q9H6T0	0.10107
P03383	Q49B96	0.29801

P03383	Q969G3	1.0006
P03383	Q8NDZ0	0.097553
P03383	Q5MJ07	0.61942
P03383	Q9BXJ9	0.50918
P03383	Q9H2T7	0.24576
P03383	Q96TA1	0.030285
P03383	O00273	0.059795
P03383	Q5BKY6	0.33834
P03383	O75843	0.40756
P03383	Q86W56	0.33308
P03383	Q9Y6B2	0.76799
P03383	Q5T655	0.33834
P03383	Q8NF64	0.97699
P03383	K7EJ46	0.33834
P03383	P63104	0.71855
P03383	Q9H4R4	0.01399
P03383	Q8NCE8	0.4177
P03383	P20700	0.19332
P03383	Q5H9J7	0.64999
P03383	Q9HAU5	0.57266
P03383	P35548	0.45448
P03383	Q9UNN5	0.41708
P03383	A6NGQ2	0.27962
P03383	O14948	0.21686
P03383	Q9UM13	0.44572
P03383	Q99873	0.40061
P03383	O75157	0.4046
P03383	Q14674	0.14354
P03383	Q96MN9	0.38989
P03383	Q02447	0.34602
P03383	Q9H063	0.32604
P03383	A8MU93	0.60341
P03383	B3EWG3	0.60341
P03383	P60604	0.3359
P03383	O75347	0.72807
P03383	Q9Y3C7	0.73677
P03383	P43487	0.14402
P03383	A6NNA2	0.63671
P03383	O14556	0.29723
P03383	O43633	0.17708
P03383	Q9NNX9	0.74489
P03383	Q6ZVN8	0.12842
P03383	Q6VUC0	0.21924
P03383	P60412	0.5733
P03383	Q5JSQ8	0.037193
P03383	P80217	0.36639
P03383	Q96MC2	0.010675
P03383	P54098	0.28289
P03383	Q9HCM7	0.64528
P03383	Q9ULL0	0.37195
P03383	Q8TEQ6	0.54295
P03383	Q8IZ16	0.081599
P03383	Q96S59	0.56496
P03383	Q8WXW3	0.57582
P03383	P07195	0.38442
P03383	Q96HI0	0.37799
P03383	Q96EL1	0.57392
P03383	Q8N3Z6	0.12898
P03383	Q9Y6K1	0.25404
P03383	Q8WWC4	0.45612
P03383	Q7RTT5	0.10215
P03383	A8MTY7	0.5733
P03383	Q13105	0.32112
P03383	Q6ZMR3	0.36755
P03383	Q8WVZ9	0.39464
P03383	O14893	0.42948
P03383	P28324	1.1064
P03383	Q9Y5J6	0.23619
P03383	Q9H1I8	0.30774
P03383	Q96A44	0.30187
P03383	Q96D98	0.68968
P03383	Q9Y2T2	0.35672
P03383	A6NLJ0	0.72583
P03383	Q8N488	0.33785
P03383	Q9H040	0.2357
P03383	Q9H0W9	0.39601

P03383	Q5TH74	0.30138
P03383	Q99990	0.32173
P03383	Q71F56	0.24931
P03383	P61586	0.75858
P03383	Q8NCX0	0.27856
P03383	O95125	0.16948
P03383	Q9H147	0.23662
P03383	Q9Y3B7	0.80436
P03383	Q92973	0.78332
P03383	Q9BPX7	0.31692
P03383	O14641	0.47021
P03383	C9JC47	0.33834
P03383	Q6UUV7	1.1517
P03383	Q9NV31	0.61102
P03383	Q96QA6	0.033288
P03383	Q9H0I3	0.17169
P03383	Q13951	0.49259
P03383	Q8N159	0.17023
P03383	Q9H8E8	0.44481
P03383	Q9BQE4	0.31483
P03383	P48595	0.23418
P03383	Q9HA90	0.60341
P03383	Q8IZ63	0.33834
P03383	Q9Y6H1	0.67327
P03383	Q9NVV5	0.36296
P03383	Q12816	0.084371
P03383	O14896	0.30465
P03383	O14929	0.52616
P03383	Q9UHD9	0.53294
P03383	Q9Y5N6	0.34658
P03383	Q13541	0.55486
P03383	Q6ZNK6	0.8446
P03383	A6NFK2	0.068039
P03383	Q9ULV5	0.21875
P03383	Q9NZQ3	0.49337
P03383	Q08AN1	0.24149
P03383	Q6ZTY8	0.0071301
P03383	P28347	0.76954
P03383	P63010	0.21045
P03383	P35637	0.25922
P03383	P51946	0.48539
P03383	Q9BSW2	0.50609
P03383	O43768	0.029362
P03383	O60732	0.1668
P03383	O15265	0.77973
P03383	Q7L8W6	0.37589
P03383	Q9UDY4	0.56219
P03383	Q15819	0.41139
P03383	P24928	0.66013
P03383	Q9H7Y2	0.093929
P03383	Q9BRT7	0.33834
P03383	P61313	0.72715
P03383	Q9UKV3	0.40663
P03383	P60372	0.5733
P03383	Q53QV2	0.20496
P03383	Q13068	0.11815
P03383	Q53G59	0.36399
P03383	Q13015	0.41969
P03383	Q13123	0.62364
P03383	P59990	0.5733
P03383	Q9NZH5	0.31304
P03383	Q96JM7	0.22447
P03383	P48539	0.21316
P03383	Q14192	0.5256
P03383	P46527	0.21953
P03383	Q03468	0.25014
P03383	Q8WXC7	0.33142
P03383	O14980	0.79458
P03383	Q9NPF5	0.69054
P03383	Q9HAW4	0.24033
P03383	P08865	0.51556
P03383	Q8IU53	0.33779
P03383	O43747	0.44917
P03383	Q5TA31	0.30561
P03383	Q15437	0.1396
P03383	P42568	0.64816

P03383	Q8N8F4	0.10316
P03383	Q01130	0.55543
P03383	Q8WVV9	0.041936
P03383	Q9GZU2	0.29226
P03383	P51959	0.36837
P03383	O15287	0.623
P03383	Q8IUC0	0.23416
P03383	Q96F07	0.27332
P03383	Q5JTJ3	0.051745
P03383	Q9HAV4	0.88809
P03383	Q9BZK3	0.30745
P03383	P60410	0.81527
P03383	Q6ZV65	0.31187
P03383	Q9BQA5	0.62247
P03383	P17040	0.3919
P03383	Q96BW1	0.086196
P03383	P0DM35	0.529
P03383	Q9GZU7	0.29175
P03383	O94855	0.1654
P03383	Q01546	0.58419
P03383	O75290	0.29751
P03383	Q96I59	0.4042
P03383	P51861	0.15095
P03383	Q9NPD3	0.18534
P03383	Q15528	0.95395
P03383	Q12933	0.47554
P03383	O43684	0.47946
P03383	Q969E8	0.80519
P03383	Q05195	0.35209
P03383	Q8NCA5	0.33039
P03383	P82909	0.30581
P03383	A1L3X0	0.41347
P03383	Q9BQE9	0.5908
P03383	P52742	0.21483
P03383	Q9H607	0.24274
P03383	Q8TC56	0.87506
P03383	O00471	0.058576
P03383	Q9Y383	0.46684
P03383	Q8IV03	0.83788
P03383	O15078	0.38763
P03383	P82663	0.46869
P03383	Q9NWY4	0.31757
P03383	P24941	0.48998
P03383	Q9BUT9	0.92766
P03383	O94818	0.80264
P03383	Q96MW1	0.60341
P03383	Q13568	0.65406
P03383	O60269	0.20864
P03383	Q9BVI4	0.33042
P03383	Q9UJY5	0.32552
P03383	Q8NAX2	0.60341
P03383	P62851	0.60908
P03383	Q8N9H6	0.039412
P03383	Q9H0P7	0.0038799
P03383	Q8IYK2	0.58597
P03383	Q9UPP5	0.46487
P03383	P55854	0.32737
P03383	Q7Z794	0.6878
P03383	P17032	0.18442
P03383	Q8IUW3	0.56391
P03383	Q6NUN7	0.33834
P03383	P0CH99	0.44628
P03383	Q9NSG2	0.32643
P03383	Q96N22	0.32458
P03383	Q99687	0.28185
P03383	Q8N859	0.31314
P03383	Q9NR22	0.19732
P03383	Q13426	0.15347
P03383	P62495	0.41268
P03383	Q9BYR8	0.81527
P03383	Q8N7B7	0.065873
P03383	Q6P1X6	0.63405
P03383	Q9Y294	0.73443
P03383	O75884	0.85965
P03383	A6NCS6	0.12952
P03383	P22531	0.34656

38305	P03383	O43261	0.1668
38306	P03383	A8MUL3	0.33834
38307	P03383	Q86US8	0.29353
38308	P03383	Q9BYR5	0.5733
38309	P03383	Q9Y2G7	0.3713
38310	P03383	P17036	0.24165
38311	P03383	Q9BYG5	0.41654
38312	P03383	P24278	0.61836
38313	P03383	P18754	0.83802
38314	P03383	Q9ULE0	0.031365
38315	P03383	Q9NQT4	0.29639
38316	P03383	Q9H943	0.017
38317	P03383	Q13404	0.77988
38318	P03383	Q15388	0.15212
38319	P03383	Q96LW1	0.31865
38320	P03383	Q13066	0.17372
38321	P03383	Q6PG37	0.34108
38322	P03383	Q9BXF6	0.38603
38323	P03383	Q96HT8	1.0626
38324	P03383	Q12765	0.41223
38325	P03383	Q6ZTR5	0.17844
38326	P03383	Q6ZSA8	0.33834
38327	P03383	Q8NF67	0.37798
38328	P03383	O43345	0.21702
38329	P03383	Q9BX82	0.31686
38330	P03383	Q8IY81	0.52227
38331	P03383	O60294	0.21847
38332	P03383	A8MVJ9	0.32866
38333	P03383	Q8N3S3	0.085327
38334	P03383	O43617	0.2123
38335	P03383	Q7KZF4	0.5742
38336	P03383	Q4VX62	0.25987
38337	P03383	P0CW24	0.090604
38338	P03383	Q92769	0.27146
38339	P03383	P0CL82	0.17372
38340	P03383	Q9H444	0.44196
38341	P03383	Q6ICB0	0.60011
38342	P03383	Q9ULZ2	0.49589
38343	P03383	Q6TGC4	0.20799
38344	P03383	Q52M93	0.11565
38345	P03383	Q9UBF6	0.48463
38346	P03383	Q8N8R7	0.31745
38347	P03383	P0CW71	0.33834
38348	P03383	Q76L83	0.36282
38349	P03383	P51825	0.7714
38350	P03383	Q9H171	0.14275
38351	P03383	Q9BYQ8	0.5733
38352	P03383	Q96AY2	0.52689
38353	P03383	O75865	0.41075
38354	P03383	Q3LI54	0.33834
38355	P03383	O95801	0.26879
38356	P03383	Q68CP9	0.30872
38357	P03383	Q9ULE4	0.28147
38358	P03383	Q16537	0.12467
38359	P03383	Q13342	0.41477
38360	P03383	Q92830	0.55645
38361	P03383	P47813	0.80256
38362	P03383	Q8NCQ2	0.37327
38363	P03383	Q969H9	0.33834
38364	P03383	O43715	0.49747
38365	P03383	Q9NRR3	0.29123
38366	P03383	Q6ZN32	0.11658
38367	P03383	A6NHJ4	0.35536
38368	P03383	Q9NP97	0.32727
38369	P03383	Q9C005	0.42237
38370	P03383	Q9BY27	0.35851
38371	P03383	P53672	0.58659
38372	P03383	Q86UB2	0.51534
38373	P03383	Q8N1L1	0.33834
38374	P03383	O00625	0.34345
38375	P03383	Q9UPV9	0.52079
38376	P03383	Q70YC4	0.37105
38377	P03383	Q3C1V9	0.067916
38378	P03383	Q6PF06	0.64619
38379	P03383	Q99717	0.25517
38380	P03383	P12035	0.52727

P03383	P43358	0.39464
P03383	Q99590	0.38222
P03383	Q9UKT5	0.25945
P03383	Q9Y5M1	0.17876
P03383	Q9NXV2	0.84521
P03383	Q8TBZ9	0.80758
P03383	P60370	0.5733
P03383	Q6ZRT6	0.60341
P03383	Q6PGQ7	0.98189
P03383	O60663	0.37128
P03383	Q8TAT6	0.18071
P03383	Q9UNH5	0.32734
P03383	Q9UGP4	0.31554
P03383	P17676	0.58407
P03383	Q6ZRN7	0.33834
P03383	P09874	0.48966
P03383	Q9XRX5	0.331
P03383	Q7Z422	0.012871
P03383	Q9H322	0.50808
P03383	Q93073	0.6134
P03383	Q9NPI6	0.65599
P03383	Q14511	0.49027
P03383	P23025	0.1741
P03383	Q9BZD4	0.5349
P03383	Q14493	0.60878
P03383	P49901	0.33834
P03383	Q6EEV6	0.047771
P03383	Q04725	0.64787
P03383	Q9P2J8	0.22485
P03383	O95780	0.34017
P03383	Q7RTS9	0.71058
P03383	P49848	0.54584
P03383	O00422	0.97042
P03383	O60304	0.16017
P03383	Q3LHN1	0.33834
P03383	O60711	0.2555
P03383	Q86VP3	0.34738
P03383	P46782	0.33652
P03383	E9PJI5	0.50049
P03383	Q5T0J7	0.28813
P03383	Q9P1Y5	0.44528
P03383	Q9UKX2	0.22419
P03383	Q6ZUL3	0.33834
P03383	Q15046	0.49854
P03383	Q9Y2N7	0.32444
P03383	Q9HCU9	0.84768
P03383	Q9Y5N5	0.19647
P03383	P59051	0.077725
P03383	Q17RN3	0.26192
P03383	Q8IXS2	0.10452
P03383	Q9BSI4	0.15335
P03383	Q13867	0.12873
P03383	P0DI82	0.17585
P03383	P78395	0.54941
P03383	Q14103	0.63907
P03383	Q8WXC3	0.38336
P03383	Q9BRQ4	0.12905
P03383	Q8IZQ1	0.38418
P03383	Q96DE9	0.0034526
P03383	Q9UM11	0.83486
P03383	Q9Y5V3	0.5984
P03383	Q16548	0.34185
P03383	P07197	0.60855
P03383	Q9BUW7	0.60341
P03383	Q7Z3K3	0.27719
P03383	Q9H9L3	0.21939
P03383	Q9NYF8	0.89517
P03383	Q6SJ96	0.094341
P03383	P54725	0.3493
P03383	O43679	0.10996
P03383	O43711	0.22878
P03383	Q14696	0.29032
P03383	Q9H501	1.0262
P03383	Q9UMX0	0.51106
P03383	Q5QP74	0.33834
P03383	A2RUB6	0.026922

P03383	Q8N6F7	0.13976
P03383	Q5JXC2	0.026721
P03383	Q6ZNG1	0.25401
P03383	Q9HBH7	0.3609
P03383	Q99081	0.34951
P03383	P0C7V6	0.60338
P03383	Q9ULH7	0.68993
P03383	Q96E29	0.3962
P03383	Q9Y3B9	0.30359
P03383	Q9HD33	0.57229
P03383	Q8N4S0	0.60341
P03383	Q8ND07	0.54713
P03383	Q8N7L0	0.091739
P03383	Q9NZE8	0.53403
P03383	Q9UM07	0.42743
P03383	Q8NEF9	0.39175
P03383	I3L0S3	0.34646
P03383	A2RTX5	0.26646
P03383	Q6ZTN6	0.27322
P03383	Q8N3X6	0.51199
P03383	Q96CS3	0.33097
P03383	A2AJT9	0.40206
P03383	Q9UJT2	0.34727
P03383	Q99417	1.151
P03383	Q9H6Z9	0.29335
P03383	Q96QD9	0.5305
P03383	O95376	0.23609
P03383	Q14159	0.83364
P03383	Q9NX70	0.79367
P03383	Q53GL7	0.3282
P03383	P07476	0.38136
P03383	Q2TAK8	0.36693
P03383	Q9NSC2	0.2035
P03383	Q5JUK9	0.21911
P03383	Q15466	0.41869
P03383	Q16254	0.80743
P03383	Q8N398	0.36393
P03383	Q5MJ68	0.16377
P03383	A6NKF2	0.33062
P03383	Q5HYI7	0.73558
P03383	Q09MP3	0.34414
P03383	P57081	0.63456
P03383	O75381	0.25639
P03383	K9M1U5	0.33834
P03383	Q14CZ0	1.0299
P03383	P41214	0.43794
P03383	P55145	0.31721
P03383	A5PL33	0.55938
P03383	Q96LY2	0.57292
P03383	Q08J23	0.47773
P03383	Q8N7J2	0.6722
P03383	Q9H9H4	0.4919
P03383	A6NGS2	0.2932
P03383	O15014	0.77702
P03383	Q14657	0.95856
P03383	O43541	0.42624
P03383	P60002	0.66149
P03383	Q6DKK2	0.6845
P03383	Q9H2K0	0.24465
P03383	Q9UN79	0.68845
P03383	A6NF36	0.3503
P03383	A5YKK6	0.7659
P03383	P52743	0.2567
P03383	Q6IQ49	0.21138
P03383	Q13765	0.78923
P03383	O75909	0.85254
P03383	Q9UL46	0.25243
P03383	Q9UH77	0.33388
P03383	O14746	0.39177
P03383	Q8IUB9	0.33834
P03383	Q8N1D5	0.15646
P03383	Q6NXS1	0.69184
P03383	P78316	0.78507
P03383	P49756	0.29207
P03383	Q05BQ5	0.04253
P03383	P63165	0.64365

P03383	Q5SZD1	0.023403
P03383	O95149	0.19276
P03383	P0DKL9	0.5828
P03383	P15407	0.45471
P03383	Q6P9B9	0.28942
P03383	Q9H6K5	0.60341
P03383	Q9HB03	0.41347
P03383	P62269	0.49042
P03383	Q9Y2K5	0.23073
P03383	Q8NF99	0.16408
P03383	Q9Y6X2	0.54936
P03383	O95190	0.55572
P03383	O60225	0.079455
P03383	Q8WXQ3	0.83891
P03383	Q96LT6	0.22347
P03383	C9J3I9	0.38038
P03383	Q99732	0.19792
P03383	Q9P015	0.1606
P03383	Q68CZ6	0.32838
P03383	P57721	0.20649
P03383	Q8IV20	0.29543
P03383	P28702	0.26666
P03383	Q9HCM9	0.35128
P03383	P0DI81	0.7535
P03383	Q6UWF3	0.36323
P03383	Q9NY87	0.57852
P03383	P68104	0.53926
P03383	Q9UNM6	0.085308
P03383	Q9ULJ3	0.39559
P03383	Q5T7N2	0.091723
P03383	Q9H3K6	0.35317
P03383	Q9GZY1	0.65021
P03383	Q15025	0.29174
P03383	Q49AR2	0.31993
P03383	P63220	0.35782
P03383	O95411	0.33834
P03383	Q9HCD5	0.79563
P03383	Q6IV72	0.1508
P03383	Q9BS18	0.40269
P03383	Q9BQI5	0.16211
P03383	Q96MF0	0.11716
P03383	P50750	0.36746
P03383	Q6P1M9	0.17996
P03383	P0C7T7	0.33834
P03383	Q08AI8	0.60341
P03383	Q8N4B5	0.57747
P03383	P05198	0.44758
P03383	Q99848	0.15555
P03383	Q8N5A5	0.6163
P03383	Q9NPB6	0.43672
P03383	Q9BZQ2	0.29028
P03383	Q96BP2	0.8499
P03383	P04406	0.28286
P03383	Q7L190	0.64766
P03383	Q9P2M7	0.26775
P03383	Q99471	0.65124
P03383	Q9UIY3	0.095238
P03383	Q86VP6	0.53372
P03383	P02538	0.5808
P03383	Q9H467	0.72315
P03383	D6RB28	0.52432
P03383	Q9UHQ7	0.43355
P03383	Q9Y5U2	0.57785
P03383	O75971	0.91463
P03383	Q12837	0.33374
P03383	Q5ZEY9	0.48598
P03383	Q12968	0.45125
P03383	Q02543	0.49535
P03383	P62244	0.47642
P03383	P0CG35	0.45586
P03383	A8MTB1	0.33834
P03383	Q15154	0.33178
P03383	Q9UFW8	0.155
P03383	O43745	0.47425
P03383	Q9NZ81	0.9346
P03383	Q01201	0.72389

38609	P03383	Q5VSR9	0.61942
38610	P03383	Q70YC5	0.13971
38611	P03383	O95067	0.30592
38612	P03383	Q96PM5	0.29113
38613	P03383	Q8TCJ0	0.74263
38614	P03383	P29966	0.62444
38615	P03383	P35711	0.71013
38616	P03383	Q6NXR4	0.16118
38617	P03383	Q676U5	0.42455
38618	P03383	Q15697	0.28011
38619	P03383	Q9UGY1	0.12505
38620	P03383	P14678	0.58821
38621	P03383	Q02878	0.66087
38622	P03383	Q8N7K0	0.24149
38623	P03383	Q01543	0.1906
38624	P03383	Q9H7T3	0.12096
38625	P03383	Q86UE3	0.23295
38626	P03383	Q9NSI6	0.6037
38627	P03383	Q8NBZ0	0.90397
38628	P03383	Q8ND56	0.55511
38629	P03383	Q9Y2Z4	0.26198
38630	P03383	Q68DY1	0.32025
38631	P03383	Q9Y6X8	0.53068
38632	P03383	Q7Z2V1	0.33834
38633	P03383	O95363	0.37014
38634	P03383	P0CW27	0.018055
38635	P03383	Q9NQ48	0.12647
38636	P03383	P14324	0.10623
38637	P03383	Q3SY00	0.62841
38638	P03383	Q9BT92	0.33446
38639	P03383	Q9NY12	0.22737
38640	P03383	Q6ZRP0	0.60341
38641	P03383	Q8IXR9	0.10045
38642	P03383	Q9UKN5	0.066988
38643	P03383	P0CW21	0.33834
38644	P03383	Q96N11	0.34587
38645	P03383	Q8IZ40	0.38482
38646	P03383	Q9Y333	0.49091
38647	P03383	Q5VX52	0.019872
38648	P03383	P51531	0.50379
38649	P03383	P14625	0.20262
38650	P03383	Q7L2H7	0.36435
38651	P03383	Q9BVL4	0.82218
38652	P03383	O75818	0.061121
38653	P03383	A0AUK8	0.10717
38654	P03383	Q156A1	0.33834
38655	P03383	Q99615	0.54599
38656	P03383	Q9UNN4	0.20958
38657	P03383	P42768	0.12831
38658	P03383	P35244	0.34149
38659	P03383	P15822	0.59407
38660	P03383	Q8WVK7	0.38776
38661	P03383	O15379	0.40605
38662	P03383	P53801	0.42574
38663	P03383	O00268	0.83526
38664	P03383	P60411	0.5733
38665	P03383	Q9Y5P3	0.32297
38666	P03383	Q9HD64	0.023529
38667	P03383	O15350	0.22465
38668	P03383	A0JNW5	0.53349
38669	P03383	P07196	0.52671
38670	P03383	Q9UIV1	0.91432
38671	P03383	Q6ZUU3	0.33834
38672	P03383	Q13278	0.34871
38673	P03383	C9JBD0	0.36886
38674	P03383	Q9UGK3	0.42667
38675	P03383	P11532	0.24507
38676	P03383	Q8WW24	0.17817
38677	P03383	Q9Y6E7	0.28574
38678	P03383	Q8IXM7	0.24929
38679	P03383	Q5VTH2	0.45383
38680	P03383	Q9NQ33	0.26096
38681	P03383	Q9UER7	0.7258
38682	P03383	A6NDU8	0.15103
38683	P03383	Q5VVC0	0.2007
38684	P03383	Q96Q89	0.27282

P03383	O60828	0.50747
P03383	Q5JTC6	0.65271
P03383	P25786	0.43914
P03383	O75698	0.33834
P03383	Q99460	0.1055
P03383	Q6ZV50	0.073272
P03383	Q01081	0.40307
P03383	P20810	0.16572
P03383	O00479	0.43416
P03383	P55895	0.41682
P03383	A8MT69	0.2372
P03383	Q9BRT9	0.30017
P03383	O95782	0.22651
P03383	P62837	0.38919
P03383	Q0VFX4	0.35784
P03383	P07814	0.2144
P03383	C9J3V5	0.32123
P03383	Q9BYV8	0.40034
P03383	Q3LI63	0.33834
P03383	Q8N1V8	0.025816
P03383	Q75MW2	0.45257
P03383	A6NNL0	0.5011
P03383	O94842	0.39331
P03383	A6NI86	0.52225
P03383	Q15759	0.25235
P03383	Q86V97	0.39464
P03383	P49770	0.11101
P03383	Q8N8K9	0.026573
P03383	Q9BTX1	0.43961
P03383	Q96RS0	0.41012
P03383	Q8IZU0	0.61185
P03383	Q8N6T0	0.12265
P03383	O96020	0.6921
P03383	A6XGL0	0.27902
P03383	Q9UI25	0.33834
P03383	H0Y7S4	0.089818
P03383	Q96DN6	0.45082
P03383	Q9BT25	0.33094
P03383	Q96PV7	0.59867
P03383	Q68EN5	0.11292
P03383	Q8WU39	0.26769
P03383	Q8N9Y4	0.72845
P03383	Q5VIY5	0.29921
P03383	Q9NX40	0.26588
P03383	Q68D10	0.60341
P03383	Q15185	0.27733
P03383	Q49AJ0	0.12503
P03383	Q8N699	0.49772
P03383	Q9UGC7	0.30099
P03383	P99999	0.44997
P03383	Q8TE02	0.90336
P03383	Q9P2M1	0.31745
P03383	A5PLN7	0.60341
P03383	Q16514	0.64862
P03383	Q7Z2X7	0.57687
P03383	Q9UIF9	0.50176
P03383	Q9NZL4	0.30695
P03383	Q86TB9	0.56068
P03383	Q9BRR8	0.66731
P03383	O14513	0.30893
P03383	Q5BJF2	0.1819
P03383	Q9Y3F4	0.55991
P03383	Q52LA3	0.44013
P03383	Q562F6	0.51577
P03383	Q9Y3D5	0.42407
P03383	P22033	0.29932
P03383	Q8NDA8	0.37817
P03383	P10074	0.16061
P03383	P82970	0.4306
P03383	Q86VS8	0.23067
P03383	Q9NR77	0.31354
P03383	Q00613	0.68448
P03383	Q07627	0.52964
P03383	Q9UMS0	0.65276
P03383	Q8TEE9	0.81598
P03383	Q9C0E2	0.6423

P03383	Q8N9V7	0.33466
P03383	Q9Y3M2	0.37923
P03383	Q9H7J1	0.32188
P03383	Q8IX21	0.17159
P03383	P0DMC3	0.33834
P03383	Q8N448	0.40792
P03383	O00255	0.10637
P03383	Q9NYZ3	0.38517
P03383	P26678	0.30292
P03383	Q2TAL8	0.64496
P03383	P10073	0.25729
P03383	Q8IYD1	0.19644
P03383	A8MYA2	0.14933
P03383	Q96F63	0.60341
P03383	A6NCI8	0.68034
P03383	Q5H9R7	0.76548
P03383	E9PAV3	0.36041
P03383	A8MWP4	0.02634
P03383	Q9HB07	0.14231
P03383	O75475	0.96677
P03383	Q86YV9	0.62559
P03383	A6NES4	0.1852
P03383	Q12800	0.21097
P03383	P11142	0.31373
P03383	Q9UBF2	0.10613
P03383	P40222	0.39468
P03383	Q569K6	0.050766
P03383	O60813	0.26448
P03383	Q16666	0.44428
P03383	P0C1Z6	0.8408
P03383	Q96HA8	0.73105
P03383	Q6XCG6	0.33834
P03383	Q6U7Q0	0.17247
P03383	Q7Z5D8	0.24968
P03383	Q9H788	0.67868
P03383	Q86WX3	0.80821
P03383	Q9BRG1	0.74081
P03383	P25963	0.43081
P03383	Q9BVG4	0.18737
P03383	Q96GW9	0.22684
P03383	Q8N9K7	0.33834
P03383	Q96C28	0.27882
P03383	P13994	0.28631
P03383	Q7L8J4	0.23172
P03383	Q92600	0.85246
P03383	P53041	0.35295
P03383	A8MUZ8	0.35121
P03383	Q9NRY5	0.68478
P03383	Q9Y5K5	0.1725
P03383	Q53QW1	0.60341
P03383	O75935	0.61351
P03383	Q03936	0.29806
P03383	Q6ZS52	0.33834
P03383	Q9NPG3	0.58938
P03383	Q86TS9	0.099869
P03383	Q9Y375	0.38186
P03383	Q9NX05	0.29797
P03383	Q07666	0.31705
P03383	Q5T319	0.33834
P03383	O15453	0.48981
P03383	A6NL82	0.35039
P03383	Q9P0W5	0.51431
P03383	Q86V48	0.65901
P03383	Q8IWF6	0.31346
P03383	Q8N0Y7	0.49853
P03383	A8MU76	0.56454
P03383	Q86YI8	0.33367
P03383	P82921	0.53195
P03383	P82979	0.14848
P03383	Q5XKR9	0.64516
P03383	Q96RE7	0.66502
P03383	Q9NZM4	0.89349
P03383	Q8TDN4	0.59602
P03383	P78344	0.21888
P03383	P22061	0.30864
P03383	Q8N140	0.59535

38837	P03383	P61962	0.47179
38838	P03383	Q8IVF1	0.67157
38839	P03383	Q86TU6	0.61687
38840	P03383	Q9HCN2	0.33834
38841	P03383	Q9Y2M0	0.24593
38842	P03383	Q9Y462	0.39201
38843	P03383	Q07866	0.29704
38844	P03383	P28072	0.2964
38845	P03383	A8K5M9	0.63163
38846	P03383	O43255	0.24298
38847	P03383	Q15929	0.28672
38848	P03383	A7E2U8	0.31496
38849	P03383	Q96B26	0.50891
38850	P03383	Q9NRY2	0.49
38851	P03383	Q8ND76	0.38174
38852	P03383	Q9H095	0.22042
38853	P03383	O15084	0.50313
38854	P03383	Q8N0Z2	0.13252
38855	P03383	A1L443	0.80122
38856	P03383	Q9H7S9	0.53442
38857	P03383	P54727	0.4705
38858	P03383	Q9BY77	0.21626
38859	P03383	O14787	0.70074
38860	P03383	Q8NE65	0.28409
38861	P03383	Q9HBM1	0.46352
38862	P03383	Q96HQ2	0.79184
38863	P03383	Q13094	0.33414
38864	P03383	Q96EI5	0.57633
38865	P03383	Q9UBZ9	0.25924
38866	P03383	Q9C0I3	0.76631
38867	P03383	P62910	0.4006
38868	P03383	Q7L5N1	0.77003
38869	P03383	A1L162	0.074582
38870	P03383	O15370	0.23243
38871	P03383	Q96GM8	0.18467
38872	P03383	Q8N8C0	0.2132
38873	P03383	Q15365	0.68953
38874	P03383	Q9NV88	0.3608
38875	P03383	Q6P1X5	0.65017
38876	P03383	P27635	0.36773
38877	P03383	O15355	0.58165
38878	P03383	O95810	0.19964
38879	P03383	A8MXK9	0.1798
38880	P03383	O43829	0.39345
38881	P03383	Q8N4L8	0.024786
38882	P03383	Q14974	0.75038
38883	P03383	Q9BXR0	0.86318
38884	P03383	O94921	0.22444
38885	P03383	Q9UKV8	0.60319
38886	P03383	Q92759	0.52313
38887	P03383	P50749	0.5281
38888	P03383	O14776	0.8463
38889	P03383	P48788	0.19778
38890	P03383	Q13200	0.34505
38891	P03383	O94850	0.19052
38892	P03383	Q9ULM6	0.76784
38893	P03383	Q12905	0.83992
38894	P03383	Q8WVM0	0.41095
38895	P03383	Q15054	0.40821
38896	P03383	Q5BJF6	0.28164
38897	P03383	Q9UKY7	0.68859
38898	P03383	Q8IWL3	0.38447
38899	P03383	Q9H0U9	0.38701
38900	P03383	Q86XN7	0.60341
38901	P03383	Q12980	0.55996
38902	P03383	Q92925	0.40621
38903	P03383	Q5VSD8	0.12912
38904	P03383	Q6ZU67	0.32714
38905	P03383	P22532	0.33834
38906	P03383	A6NKN8	0.7048
38907	P03383	Q13569	0.27749
38908	P03383	Q6IEG0	0.28212
38909	P03383	Q96ED9	0.28138
38910	P03383	O00512	0.54229
38911	P03383	Q6PB30	0.54188
38912	P03383	A4D997	0.49427

P03383	O96007	0.54696
P03383	Q96LB3	0.32655
P03383	Q9H074	0.23874
P03383	P12883	0.17113
P03383	Q9P0W2	0.50666
P03383	Q9BYQ0	0.5733
P03383	Q9UJG1	0.55561
P03383	Q6ZW54	0.74611
P03383	Q9BYQ5	0.5733
P03383	Q96IT1	0.44189
P03383	P31269	0.44115
P03383	Q96IM9	0.088354
P03383	Q9Y312	0.16389
P03383	O43299	0.8756
P03383	Q6P1L5	1.0032
P03383	Q9BQN1	0.61131
P03383	Q8IVT2	0.68155
P03383	Q3LI72	0.33834
P03383	Q9Y6K5	0.023755
P03383	Q3LI62	0.33834
P03383	Q9H4H8	0.21261
P03383	Q6IN85	0.18293
P03383	Q8WWB3	0.02676
P03383	Q9Y266	0.57784
P03383	Q8IY63	0.26129
P03383	Q3ZCT1	0.17115
P03383	Q99496	0.28868
P03383	Q9H8W2	0.091024
P03383	P36402	0.37945
P03383	Q8TAQ2	1.1845
P03383	Q9Y2J8	0.45638
P03383	Q3SY05	0.059202
P03383	Q8N5J4	0.26526
P03383	P58004	0.25823
P03383	Q8IXM3	0.42103
P03383	Q15573	0.34054
P03383	Q9Y3A5	0.48976
P03383	Q02833	0.37246
P03383	Q68DL7	0.33834
P03383	P35232	0.33603
P03383	P0CG42	0.33834
P03383	Q96E14	0.47326
P03383	Q494R0	0.33834
P03383	Q7Z4H9	0.34923
P03383	Q8N9Z0	0.29224
P03383	Q9UBF1	0.52695
P03383	O14593	0.43088
P03383	Q3SY84	0.52727
P03383	Q8TDR4	0.25085
P03383	Q96NL8	0.43574
P03383	Q6L9T8	0.27608
P03383	Q96HR8	0.88286
P03383	O95999	0.093946
P03383	Q9H4A6	0.33732
P03383	Q99543	0.25247
P03383	Q9HD42	0.73346
P03383	Q6K0P9	0.10263
P03383	Q9BZ23	0.46115
P03383	P50453	0.40718
P03383	Q5TCQ9	0.37905
P03383	Q9UHJ3	0.1228
P03383	P28065	0.44968
P03383	Q13501	0.26256
P03383	Q9Y4H4	0.2416
P03383	E9PB15	0.0042209
P03383	P0CI01	0.38945
P03383	P04264	0.19973
P03383	P48668	0.24286
P03383	Q8N972	0.24149
P03383	Q9C0C6	0.65221
P03383	Q5VVS0	0.67676
P03383	C9JLW8	0.44588
P03383	Q16775	0.22145
P03383	Q9P016	0.37222
P03383	Q69YW0	0.093457
P03383	O43918	0.74097

P03383	Q9HB96	0.57356
P03383	Q9HA77	0.28305
P03383	Q9H410	0.5989
P03383	Q9P0U1	0.32612
P03383	Q5T7W7	0.60341
P03383	O15027	0.42307
P03383	Q5VWI1	0.80294
P03383	Q8N5R6	0.33122
P03383	P51116	0.37914
P03383	Q96FX7	0.18355
P03383	O60784	0.15708
P03383	Q5T870	0.5587
P03383	Q5MIZ7	0.23902
P03383	P22528	0.26317
P03383	Q29RF7	0.74862
P03383	Q9P1F3	0.52931
P03383	Q8IZD4	0.595
P03383	O60829	0.5376
P03383	Q8IX12	0.80164
P03383	Q5VXH4	0.28378
P03383	Q96NS1	0.10471
P03383	P41212	0.58689
P03383	Q9NWV8	0.36308
P03383	Q9BSH5	0.18271
P03383	Q16236	0.37668
P03383	Q9BW85	0.79054
P03383	Q8TAA5	0.30962
P03383	O94992	1.1343
P03383	Q8TF32	0.33501
P03383	P07741	0.21298
P03383	Q68EA5	0.32673
P03383	Q5SRE5	0.42425
P03383	P50402	0.23574
P03383	Q15170	1.2006
P03383	Q96KT6	0.33834
P03383	Q5T8A7	0.51715
P03383	P57076	0.25301
P03383	P43897	0.76286
P03383	Q969H0	0.49575
P03383	Q58F21	0.15004
P03383	Q9NQR4	0.20681
P03383	P42771	0.41785
P03383	Q96JN0	0.57939
P03383	Q96T58	0.51242
P03383	P62857	0.35554
P03383	Q9H832	0.26971
P03383	Q99684	0.22965
P03383	Q9NY74	0.73146
P03383	Q9Y2G2	0.26668
P03383	Q8WVK2	0.59398
P03383	Q8WUF5	0.61688
P03383	Q8WY41	0.25604
P03383	P04732	0.2243
P03383	P28066	0.47523
P03383	P31314	0.43238
P03383	O15131	0.76685
P03383	O95751	0.34413
P03383	Q502W7	0.2968
P03383	Q5VWP2	0.057688
P03383	A8MX80	0.53208
P03383	Q6PJ61	0.72639
P03383	Q13547	0.5713
P03383	P26651	0.44287
P03383	Q15853	0.45782
P03383	O43809	0.51092
P03383	Q14207	0.77173
P03383	Q92783	0.34279
P03383	P35580	0.18378
P03383	P52298	0.43776
P03383	Q9ULX6	0.3111
P03383	Q9UI56	0.33834
P03383	Q8IXQ9	0.20816
P03383	Q99613	0.44281
P03383	Q5VTR2	0.50457
P03383	P78371	0.33257
P03383	E9PQ53	0.5252

P03383	Q8N812	0.19828
P03383	Q9P2T0	0.36611
P03383	Q9H098	0.66204
P03383	Q6ZQN3	0.54278
P03383	P10124	0.27303
P03383	Q8N7Z2	0.32695
P03383	P52737	0.40586
P03383	P50616	0.21797
P03383	Q15323	0.66522
P03383	Q8WUQ7	0.042906
P03383	Q96IR2	0.25401
P03383	O14965	0.24225
P03383	Q9NUP7	0.61226
P03383	Q96C57	0.33532
P03383	Q9BQ69	0.27782
P03383	Q12906	0.85928
P03383	Q99933	0.51804
P03383	Q99583	0.24278
P03383	Q14264	0.34957
P03383	Q3SYG4	0.20599
P03383	A8MXZ3	0.33834
P03383	Q9Y3C4	0.41635
P03383	Q9UGL9	0.33834
P03383	Q9BX70	0.34768
P03383	Q9UHB7	1.0522
P03383	Q8IYI0	0.22035
P03383	Q96RU7	0.30587
P03383	Q8IV33	0.39065
P03383	P82932	0.23783
P03383	Q8TCY9	0.24999
P03383	Q15628	0.3684
P03383	O75436	0.42111
P03383	P17152	0.37345
P03383	Q9H081	0.46073
P03383	A6NN14	0.2288
P03383	Q52MB2	0.6604
P03383	Q5I0X4	0.60341
P03383	Q9BY41	0.23775
P03383	O75822	0.55465
P03383	O76042	0.39704
P03383	Q96PV6	0.46167
P03383	O75446	0.93217
P03383	Q8WYQ3	0.57124
P03383	Q6ZMS7	0.20364
P03383	Q9NZI6	0.30061
P03383	Q8IX90	0.40584
P03383	Q5JQF7	0.33834
P03383	P28749	1.0231
P03383	Q09666	0.45383
P03383	P35908	0.4615
P03383	P46087	0.53233
P03383	Q92748	0.81329
P03383	P09012	0.25914
P03383	Q9UKL0	0.89777
P03383	Q15007	0.43222
P03383	Q9UIC8	0.18887
P03383	Q5JRC9	0.4877
P03383	P35658	0.811
P03383	P62906	0.43312
P03383	Q9BRU9	0.28158
P03383	Q9UKC9	0.31334
P03383	Q5SVQ8	0.22267
P03383	Q96FF9	0.59963
P03383	Q16670	0.14422
P03383	A6NP11	0.33501
P03383	Q7L8S5	0.19313
P03383	O43504	0.026914
P03383	P13489	0.26715
P03383	Q9BWG6	0.26493
P03383	Q9Y4Z0	0.4705
P03383	Q96IY1	0.64831
P03383	Q8IY92	0.19978
P03383	Q9P1Q0	0.269
P03383	Q8N616	0.33834
P03383	P46783	1.0088
P03383	Q6ZS17	0.29798

P03383	Q8ND90	0.49786
P03383	Q14980	0.77533
P03383	Q9BV20	0.14274
P03383	Q5H9F3	0.37218
P03383	Q9BRV8	0.24391
P03383	Q3L8U1	0.62011
P03383	Q9H307	0.65403
P03383	Q71SY5	0.80368
P03383	Q9NP50	0.54395
P03383	Q16082	0.60379
P03383	Q9NSC5	0.31519
P03383	Q9H7H0	0.49088
P03383	Q9NZN8	0.49364
P03383	Q8IX03	0.73049
P03383	P54253	0.70476
P03383	Q96IP4	0.44958
P03383	O75525	0.62552
P03383	Q5SNX5	0.33834
P03383	Q99708	0.45985
P03383	A2RUB1	0.60341
P03383	Q9Y3U8	0.57095
P03383	O95232	0.29947
P03383	P10242	0.71564
P03383	P67870	0.14371
P03383	P23497	0.64407
P03383	Q96HY6	0.13806
P03383	Q9H116	0.16326
P03383	Q96ST8	0.77507
P03383	Q5T5Y3	0.22747
P03383	Q9UKJ5	0.26065
P03383	Q9Y252	0.27113
P03383	Q8IWC1	0.29733
P03383	P60866	0.48492
P03383	Q7L4I2	1.14
P03383	A1L429	0.17372
P03383	Q04637	0.30689
P03383	Q8N3U1	0.33834
P03383	A6NL46	0.61919
P03383	P52815	0.37416
P03383	O75419	0.65396
P03383	Q9UK45	0.51929
P03383	Q5RKV6	0.041133
P03383	Q13617	0.411
P03383	Q9BVT8	0.63008
P03383	Q6P582	0.51933
P03383	Q9P086	0.80648
P03383	Q8TE69	0.1668
P03383	Q96C86	0.58927
P03383	Q86WI3	0.2859
P03383	Q9BYQ4	0.5733
P03383	Q15181	0.067245
P03383	Q9P2Y5	0.19572
P03383	Q96GC6	0.85282
P03383	Q15327	0.2371
P03383	Q9HCI5	0.42527
P03383	Q9H9D4	0.3617
P03383	Q5EBL4	0.20122
P03383	Q99638	0.36204
P03383	Q96EK5	0.13098
P03383	Q9UJX4	0.51501
P03383	O15535	0.093676
P03383	Q9UKK3	0.42491
P03383	P37802	0.26911
P03383	O95238	0.093715
P03383	P61254	0.58611
P03383	O15127	0.37333
P03383	Q9HCK8	0.21616
P03383	P0DKX0	0.27416
P03383	Q8WZA9	0.60341
P03383	Q14188	0.5583
P03383	Q8TF63	0.44746
P03383	Q9H009	0.19735
P03383	Q7Z6M4	0.44417
P03383	Q9UPV0	1.1101
P03383	Q9H2G4	0.48519
P03383	P62633	0.31576

39217	P03383	Q494X3	0.32673
39218	P03383	Q16401	0.59805
39219	P03383	O14633	0.33834
39220	P03383	Q8TAP6	0.58298
39221	P03383	Q8WY54	0.53107
39222	P03383	P01100	0.4089
39223	P03383	Q3KP22	0.14005
39224	P03383	Q9H3L0	0.62879
39225	P03383	P04198	0.61684
39226	P03383	Q6ZRI6	0.35258
39227	P03383	Q86XN6	0.24483
39228	P03383	Q9UC06	0.17256
39229	P03383	Q4LE39	0.78473
39230	P03383	Q5T7V8	1.1857
39231	P03383	P43268	0.23554
39232	P03383	Q8WXF8	0.96255
39233	P03383	Q8NCD3	0.32508
39234	P03383	Q9UGB4	0.33834
39235	P03383	Q2KHR2	0.14678
39236	P03383	P11161	0.31021
39237	P03383	Q8N9R0	0.33834
39238	P03383	Q9UQ80	0.8544
39239	P03383	Q05519	0.50389
39240	P03383	O95983	0.92085
39241	P03383	A8MZF0	0.60341
39242	P03383	Q9P1D8	0.33834
39243	P03383	Q8NA77	0.51555
39244	P03383	P05114	0.33615
39245	P03383	Q9H7C4	0.20078
39246	P03383	O43765	0.42702
39247	P03383	P62979	0.20176
39248	P03383	Q86Y79	0.47859
39249	P03383	Q0VDD5	0.33834
39250	P03383	Q8N0Y2	0.14388
39251	P03383	Q96H79	0.40071
39252	P03383	A6NEV1	0.43652
39253	P03383	Q6RVD6	0.33834
39254	P03383	Q5T280	0.81192
39255	P03383	O60885	0.46831
39256	P03383	Q96Q35	0.2841
39257	P03383	P10515	0.24735
39258	P03383	Q8NG31	0.42866
39259	P03383	Q92750	0.44127
39260	P03383	Q96P63	0.23476
39261	P03383	Q92754	0.39189
39262	P03383	O00231	0.29204
39263	P03383	Q96HA1	0.66909
39264	P03383	A7E2V4	0.96614
39265	P03383	Q9UKV0	0.29475
39266	P03383	Q96CD0	0.38267
39267	P03383	Q14153	0.40492
39268	P03383	O43186	0.57798
39269	P03383	Q00597	0.49231
39270	P03383	P61981	0.30327
39271	P03383	Q14145	0.96268
39272	P03383	P39880	0.37897
39273	P03383	Q8N108	0.84816
39274	P03383	Q5T1J5	0.67327
39275	P03383	P23760	0.61384
39276	P03383	P61579	0.43317
39277	P03383	Q9UIF8	0.57965
39278	P03383	Q5HYK9	0.22259
39279	P03383	Q86XK3	0.56269
39280	P03383	Q9NRL3	0.25577
39281	P03383	Q5TEZ4	0.33834
39282	P03383	Q9HBX3	0.33834
39283	P03383	A2IDD5	0.38446
39284	P03383	A4D263	0.16655
39285	P03383	A6NHU5	0.13506
39286	P03383	Q96AT1	0.60341
39287	P03383	Q9NY27	0.68362
39288	P03383	Q15906	0.62244
39289	P03383	Q9H7E9	0.60341
39290	P03383	Q9HAI6	0.45603
39291	P03383	Q9NYF0	0.13727
39292	P03383	Q9NQ32	0.42486

P03383	Q96DP5	0.32423
P03383	P0CG47	0.27823
P03383	Q3LI81	0.81527
P03383	Q9UHA2	0.20459
P03383	Q9NZI7	0.1437
P03383	O95394	0.55605
P03383	Q96QK1	0.38775
P03383	A6H8Y1	0.43466
P03383	Q8N715	0.037095
P03383	Q96QC0	0.734
P03383	Q8N782	0.31389
P03383	Q9BSY4	0.33299
P03383	P49918	0.49547
P03383	P0C7V5	0.24415
P03383	Q99750	0.39987
P03383	O60524	0.75313
P03383	Q6P6B1	0.60341
P03383	Q5MJ08	0.037487
P03383	Q9BUJ2	0.90159
P03383	Q96HW7	0.3608
P03383	Q8N3X1	0.17267
P03383	P62875	0.21466
P03383	Q9H6Z4	0.84571
P03383	Q96PY6	0.26661
P03383	P58557	0.71751
P03383	P0CB43	0.17715
P03383	Q5T9G4	0.74659
P03383	Q9BYR9	0.23605
P03383	Q8N4U5	0.01334
P03383	Q9Y4P8	0.52438
P03383	Q96RK0	0.68205
P03383	Q5TEC3	0.185
P03383	Q15796	0.45037
P03383	Q99594	0.91047
P03383	Q9NYP3	0.52454
P03383	Q53SZ7	0.60341
P03383	P62241	0.38457
P03383	P21675	0.34161
P03383	Q9P209	0.49555
P03383	Q9H0N5	0.66646
P03383	Q96MG7	0.51318
P03383	Q15059	0.98657
P03383	Q3LI61	0.33834
P03383	Q96NB3	0.26605
P03383	Q9BZK7	0.71351
P03383	P56555	0.50968
P03383	P56524	0.21228
P03383	Q9BUL8	0.39232
P03383	Q6ZVN6	0.0075334
P03383	Q9BUP3	0.14059
P03383	Q96CB8	0.28213
P03383	Q96JE7	0.63447
P03383	Q9Y6G5	0.1668
P03383	A6NGY3	0.13371
P03383	C9JE40	0.58209
P03383	Q8WU03	0.38754
P03383	Q92541	0.72873
P03383	Q86YF9	0.056738
P03383	Q13367	0.26011
P03383	Q9UHY8	0.17098
P03383	Q96FA3	0.31129
P03383	Q6P280	0.3549
P03383	Q9NRH1	0.2503
P03383	Q9UPN7	0.67833
P03383	A1L190	0.7148
P03383	Q8TC71	0.15245
P03383	Q7Z7F7	0.51384
P03383	Q9H5Y0	0.37892
P03383	Q9NWM3	0.12111
P03383	Q9BVC5	0.39716
P03383	H3BNL8	0.71002
P03383	Q6ZMY9	0.3136
P03383	P46781	0.78039
P03383	Q8N584	0.4957
P03383	Q14232	0.054278
P03383	Q9NUW8	0.40409

P03383	O75208	0.40882
P03383	P35237	0.26449
P03383	Q717R9	0.27302
P03383	Q8WWY3	0.2278
P03383	O60925	0.013876
P03383	Q53FT3	0.28086
P03383	Q86TP1	0.52789
P03383	P41970	0.65558
P03383	Q13416	0.34229
P03383	Q6Q759	0.48806
P03383	Q86W67	0.015086
P03383	O43309	0.17727
P03383	Q96GU1	0.2309
P03383	Q9NRG9	0.65908
P03383	P0C866	0.0011014
P03383	Q9BYJ9	0.52267
P03383	P48634	0.55947
P03383	Q96EH5	0.4722
P03383	P32519	1.0104
P03383	Q9UQ35	0.48386
P03383	Q13347	0.44281
P03383	Q6NSI8	0.40149
P03383	Q00577	0.39835
P03383	Q9HBT7	0.21449
P03383	Q96NJ6	0.15714
P03383	Q5HYN5	0.098536
P03383	P68036	0.61168
P03383	Q8NAP3	0.15666
P03383	A8MT65	0.28416
P03383	Q5VZV4	0.24248
P03383	Q15527	0.19114
P03383	Q8NEG4	0.32956
P03383	Q09FC8	0.19011
P03383	E9PI22	0.37274
P03383	Q14677	0.22346
P03383	Q6P1K2	0.86928
P03383	Q5JS54	0.43101
P03383	O95153	0.45253
P03383	Q96AE4	0.34412
P03383	Q8N9A8	0.36433
P03383	Q6ULP2	0.34344
P03383	P13647	0.63715
P03383	Q8WWQ0	0.41685
P03383	Q03252	0.25322
P03383	Q9UJX5	0.52601
P03383	P62877	0.33197
P03383	Q17RC7	0.48787
P03383	Q9P0V3	0.47168
P03383	Q9Y4A8	0.31044
P03383	Q5SQS8	0.24635
P03383	P23508	0.17481
P03383	Q9P255	0.22586
P03383	Q99909	0.13592
P03383	O75161	0.71865
P03383	Q2NKX9	0.60341
P03383	Q8IWY8	0.16661
P03383	P52435	0.40938
P03383	Q5VT03	0.5011
P03383	Q9H9J2	0.25343
P03383	Q9HB09	0.78652
P03383	Q15910	0.32146
P03383	Q63ZY3	0.66332
P03383	Q8N9T2	0.33834
P03383	Q9NR09	0.28303
P03383	Q13309	0.38792
P03383	Q9UND3	0.13958
P03383	Q9UFV3	0.026251
P03383	Q96E11	0.57964
P03383	Q9GZL7	0.51425
P03383	Q9UGK8	0.44438
P03383	Q5T230	1.0147
P03383	Q9UII5	0.21702
P03383	Q08AD1	0.28837
P03383	P22103	0.33834
P03383	P32189	0.13278
P03383	Q6NSH3	0.37918

P03383	P51522	0.18615
P03383	Q9H3M7	0.57553
P03383	Q8N9B5	0.48259
P03383	Q9NRL2	0.74014
P03383	P82650	0.041862
P03383	P23284	0.20142
P03383	Q04206	0.45926
P03383	A8MZG2	0.60341
P03383	Q7RTV3	0.19614
P03383	Q9Y2X3	0.44885
P03383	Q9HC62	0.31179
P03383	Q8IZM0	0.13626
P03383	P39023	0.68268
P03383	P53992	0.65956
P03383	Q10586	0.22552
P03383	Q8TF40	0.48661
P03383	O95997	0.68588
P03383	P78318	0.6148
P03383	O75113	0.60994
P03383	Q07021	0.52426
P03383	P49910	0.50266
P03383	P55265	0.24512
P03383	Q9Y2D9	0.39205
P03383	Q9H910	0.60274
P03383	Q8IW36	0.29344
P03383	Q96QU8	0.75112
P03383	O43776	0.4574
P03383	Q8N9Z2	0.50098
P03383	P17535	0.72717
P03383	F5GYI3	0.69867
P03383	Q8WWR8	0.05437
P03383	Q6L8H1	0.33834
P03383	Q8WTR7	0.44801
P03383	O15360	0.51258
P03383	Q9H606	0.22955
P03383	Q8N0U6	0.33834
P03383	Q9BQ15	0.37707
P03383	Q2T9L4	0.19224
P03383	P78545	0.63651
P03383	Q6ZRH4	1.0364
P03383	P11169	0.25174
P03383	P56182	0.45419
P03383	A6NIH7	0.27233
P03383	P57678	0.66507
P03383	Q9NQ50	0.62883
P03383	Q6NUN9	0.50812
P03383	Q8IWD4	0.60341
P03383	Q99865	0.40873
P03383	O43583	0.71662
P03383	P08397	0.16632
P03383	O15318	0.59734
P03383	Q9NRR5	0.39086
P03383	Q3LI66	0.33834
P03383	Q9BU89	0.074609
P03383	Q9NY43	0.25252
P03383	P55769	0.65348
P03383	Q8IY57	0.83561
P03383	Q8NCU1	0.16243
P03383	O60930	0.071947
P03383	Q5T5N4	0.090165
P03383	F8WFD2	0.22014
P03383	Q68DX3	0.2663
P03383	Q8WXI9	0.33862
P03383	Q9Y448	0.54541
P03383	Q3B820	0.55921
P03383	P49459	0.29496
P03383	Q9UHB9	0.15677
P03383	P45984	0.25614
P03383	P82094	0.90424
P03383	Q5VWM6	0.71279
P03383	P51532	0.33786
P03383	O95671	0.35781
P03383	Q96SR6	0.17681
P03383	Q92567	0.31531
P03383	Q14602	0.15038
P03383	Q9BW66	0.61252

P03383	Q8N137	0.62294
P03383	Q86X60	0.21591
P03383	Q8WYR1	0.10013
P03383	P42704	0.69626
P03383	Q96GT9	0.64111
P03383	Q9BTL3	0.43479
P03383	Q5SVJ3	0.11806
P03383	Q9P2I0	0.6188
P03383	O60645	0.30682
P03383	Q96S95	0.35731
P03383	Q8N5T8	0.34583
P03383	A8MUI8	0.30294
P03383	Q6ZU65	0.2453
P03383	Q9NWB6	0.60341
P03383	Q8N6Y0	0.38695
P03383	Q6EEV4	0.33834
P03383	Q9UBI1	0.1668
P03383	O43325	0.64944
P03383	Q9UPZ3	0.37045
P03383	Q96IT6	0.33834
P03383	Q02556	0.23215
P03383	Q6PI48	0.46193
P03383	A6NGY1	0.57958
P03383	Q86SY8	0.33834
P03383	O00198	0.43239
P03383	Q8WUM4	0.76561
P03383	Q9Y222	0.7218
P03383	O43752	0.13627
P03383	P04731	0.23866
P03383	Q6AI08	0.40514
P03383	Q8WUY8	0.41154
P03383	Q9UKZ1	0.65596
P03383	Q9Y5B0	0.33773
P03383	O76031	0.43026
P03383	A0JLT2	0.93294
P03383	P80723	0.32172
P03383	Q8N9P6	0.33834
P03383	Q96QU4	0.49935
P03383	P0CZ20	0.18349
P03383	Q96F83	0.33291
P03383	P53567	0.49768
P03383	Q53S33	0.65701
P03383	Q9UKT9	0.34846
P03383	Q9H6D7	0.32838
P03383	Q6ZVC0	0.52986
P03383	Q5UE93	0.39242
P03383	A6NEQ2	0.33834
P03383	O60504	0.38345
P03383	Q9NPI1	0.80791
P03383	O76015	0.66284
P03383	O60518	0.69697
P03383	Q5T5F5	0.33834
P03383	Q9UJY1	0.30769
P03383	P50452	0.37761
P03383	Q8N5S3	0.0067398
P03383	O14771	0.46597
P03383	Q86YC2	0.37917
P03383	P0DJG4	0.64266
P03383	Q8NDT2	0.6864
P03383	O14907	0.24783
P03383	Q8TCC3	0.11199
P03383	P78385	0.6399
P03383	Q8TAB5	0.60341
P03383	Q5SW79	0.52568
P03383	Q9NRA8	0.71859
P03383	Q15370	0.82592
P03383	P52736	0.42046
P03383	Q7RTT6	0.29227
P03383	Q9Y2M5	0.55532
P03383	Q96RY5	0.65414
P03383	Q6ZQT7	0.33834
P03383	A6NHP3	0.38945
P03383	P0CW26	0.18349
P03383	Q96EU6	0.28289
P03383	Q9H3M9	0.32342
P03383	Q02410	0.32958

39597	P03383	Q96NJ1	0.33834
39598	P03383	Q96MR7	0.95681
39599	P03383	Q96KD3	0.87506
39600	P03383	Q92979	0.18514
39601	P03383	Q99996	0.31835
39602	P03383	Q8N7W2	0.43631
39603	P03383	Q15233	0.44501
39604	P03383	Q9UJA5	0.75168
39605	P03383	A6NHX0	0.52445
39606	P03383	Q9UM63	0.10894
39607	P03383	Q8N9P0	0.079313
39608	P03383	P12757	0.20749
39609	P03383	Q9UBY9	0.68878
39610	P03383	Q13227	0.79551
39611	P03383	Q2TAM9	0.80122
39612	P03383	Q13772	0.15379
39613	P03383	Q9BR46	0.5087
39614	P03383	P0CG48	0.27172
39615	P03383	P54840	0.29116
39616	P03383	Q12962	0.86379
39617	P03383	O60675	0.22573
39618	P03383	O95373	0.71773
39619	P03383	Q6ZUC2	0.085584
39620	P03383	O43310	0.28379
39621	P03383	P35579	0.55765
39622	P03383	Q969E4	0.19547
39623	P03383	Q8NA03	0.38694
39624	P03383	A8MU10	0.33834
39625	P03383	Q9NPD8	0.25572
39626	P03383	Q9H320	0.7165
39627	P03383	P33316	0.41306
39628	P03383	Q96ST3	0.58189
39629	P03383	Q3LI64	0.33834
39630	P03383	Q6ZSR3	0.33834
39631	P03383	A6NGK3	0.21377
39632	P03383	Q13310	0.2729
39633	P03383	O43542	0.30374
39634	P03383	P38935	0.31016
39635	P03383	Q9Y5A6	0.48168
39636	P03383	Q6L8H2	0.33834
39637	P03383	Q96EF9	0.25158
39638	P03383	Q6Q795	0.33834
39639	P03383	P07199	0.48297
39640	P03383	P43356	0.67054
39641	P03383	H0UI37	0.078684
39642	P03383	Q8NDC0	0.51506
39643	P03383	O75807	0.21431
39644	P03383	P62952	0.2467
39645	P03383	Q9BTT4	0.48467
39646	P03383	Q5MJ09	0.1039
39647	P03383	Q6ZN84	0.19012
39648	P03383	Q9Y2G9	0.54468
39649	P03383	Q92845	0.050097
39650	P03383	A8MYZ0	0.45898
39651	P03383	Q9UK61	0.4612
39652	P03383	Q8NDD1	0.0058186
39653	P03383	O75635	0.23418
39654	P03383	O94817	0.31618
39655	P03383	O00411	0.34286
39656	P03383	Q14691	0.22301
39657	P03383	P21980	0.14167
39658	P03383	Q8TBE0	0.5084
39659	P03383	P28074	0.38808
39660	P03383	Q5M9Q1	0.76137
39661	P03383	Q92796	0.20297
39662	P03383	Q6ZW13	0.33834
39663	P03383	Q14258	0.72809
39664	P03383	O95456	0.26513
39665	P03383	Q6R2W3	0.2543
39666	P03383	Q96HA7	0.4926
39667	P03383	Q9BXP5	0.82064
39668	P03383	Q9HAP2	0.32458
39669	P03383	Q8IYN0	0.21305
39670	P03383	Q5T9C2	0.60341
39671	P03383	Q2T9F4	0.089617
39672	P03383	Q86XA9	0.29995

P03383	Q3LI67	0.33834
P03383	Q14151	0.51347
P03383	P62841	0.50687
P03383	Q9HA58	0.33834
P03383	Q96DF8	0.8466
P03383	Q92785	0.32033
P03383	Q8N0T1	0.38321
P03383	P78559	0.3121
P03383	Q96M53	0.98701
P03383	P50221	0.4937
P03383	Q9H1B7	0.66613
P03383	P18887	0.3963
P03383	Q9UM00	0.87216
P03383	Q96F86	0.42698
P03383	O00425	0.26392
P03383	Q9Y6H5	0.53745
P03383	Q8N3V7	0.45087
P03383	Q69383	0.43317
P03383	O14681	0.44173
P03383	P62308	0.521
P03383	Q9NR46	0.22021
P03383	P50548	0.1789
P03383	Q8TBN0	0.1389
P03383	Q8TF09	0.37653
P03383	Q8N7U7	0.3704
P03383	P62256	0.22644
P03383	P27695	0.32257
P03383	Q6NUQ1	0.35214
P03383	P61236	0.16286
P03383	O95551	0.48589
P03383	A8MTA8	0.1877
P03383	P0CG43	0.33834
P03383	Q99676	0.22485
P03383	Q92530	0.47952
P03383	P63146	0.29496
P03383	B1APH4	0.25856
P03383	C9JN71	0.21181
P03383	P57086	0.33527
P03383	Q96JC9	0.73062
P03383	Q9UL42	0.57654
P03383	Q96LP2	0.0016313
P03383	Q9UC07	0.19712
P03383	P23246	0.57366
P03383	P0DKV0	0.05412
P03383	Q9HAF1	0.73244
P03383	P07320	0.61117
P03383	P51397	0.43563
P03383	Q86VX2	0.59004
P03383	Q8NA72	0.067357
P03383	P51786	0.1696
P03383	P19388	0.4328
P03383	P23528	0.54779
P03383	P49720	0.26471
P03383	P59797	0.50117
P03383	Q01534	0.29298
P03383	Q969J2	0.16398
P03383	Q5TGS1	0.24395
P03383	Q8N371	0.15051
P03383	Q8WTX7	0.44915
P03383	Q6ZVQ6	0.88532
P03383	Q16531	0.37503
P03383	Q14140	1.2027
P03383	Q13148	0.42776
P03383	Q9ULW0	0.63067
P03383	Q9UHR6	0.59324
P03383	Q9UBB9	0.23589
P03383	Q6NVV1	0.266
P03383	Q8TAQ5	0.24149
P03383	H3BR10	0.33834
P03383	Q3LI59	0.33834
P03383	P48552	0.48956
P03383	Q8WU90	0.60099
P03383	Q8N5H3	0.60341
P03383	Q9UHV7	0.39358
P03383	Q96CB9	0.48676
P03383	Q96Q83	0.33236

P03383	Q9P287	0.44431
P03383	Q8TAA3	0.17908
P03383	O95613	0.45845
P03383	Q3MHD2	0.33225
P03383	Q15424	0.58052
P03383	Q13561	0.44947
P03383	A6NN90	0.2413
P03383	P61289	0.65687
P03383	Q8TAL5	0.16618
P03383	O76009	0.66284
P03383	Q9C099	0.63532
P03383	Q8IY42	0.8882
P03383	O60831	0.62817
P03383	O14777	0.38318
P03383	Q5U5X8	0.70465
P03383	P55197	1.0537
P03383	Q8TAD7	0.60341
P03383	Q9BSD3	0.077499
P03383	Q9Y2X9	0.44825
P03383	Q6P0N0	0.66671
P03383	Q96CV9	0.30502
P03383	P22090	0.20339
P03383	Q96KW2	0.57575
P03383	P29083	0.88411
P03383	P24863	0.86352
P03383	Q96GS4	0.85276
P03383	Q5HYJ3	0.24176
P03383	O75152	0.20165
P03383	Q96C03	0.31806
P03383	A1L188	0.57124
P03383	Q96AQ1	0.57292
P03383	Q9P1T7	0.98115
P03383	Q969R5	0.065552
P03383	Q8TAE8	0.70789
P03383	Q13156	0.19831
P03383	P0C5J1	0.035146
P03383	Q8NFD5	0.62335
P03383	Q70EK8	0.46224
P03383	Q96EP1	0.41472
P03383	Q8N187	0.4555
P03383	Q13033	0.45038
P03383	Q8NFP0	0.38993
P03383	Q68DN1	0.33889
P03383	P61970	0.22584
P03383	Q9NVR5	0.53329
P03383	A1L168	0.60341
P03383	Q5T1B1	0.80124
P03383	Q9P242	0.52986
P03383	O75132	0.02568
P03383	Q13045	0.76394
P03383	O94952	0.49926
P03383	Q9H7T9	0.45354
P03383	Q92922	0.89023
P03383	Q00587	0.036885
P03383	Q66K41	0.63353
P03383	Q9H3F6	0.24788
P03383	O43592	0.12467
P03383	O43663	0.64493
P03383	Q5JU67	0.018214
P03383	Q8TEK3	0.19405
P03383	Q96AN2	0.2119
P03383	Q49MG5	0.66114
P03383	Q92990	0.16186
P03383	Q9BYR0	0.5733
P03383	P0CF75	0.025095
P03383	Q12789	0.57691
P03383	O95447	0.10784
P03383	P15336	0.67994
P03383	Q13794	0.36699
P03383	P10828	0.38764
P03383	Q9NXV6	0.72795
P03383	Q8TBK2	0.35382
P03383	Q9H1A3	0.1668
P03383	Q9NZI5	0.25118
P03383	Q9NUL5	0.1608
P03383	Q92540	0.63783

P03383	Q9NRD1	0.050819
P03383	Q92837	0.70389
P03383	P41250	0.20077
P03383	P16383	0.68909
P03383	Q8IW50	0.60341
P03383	Q92499	0.29853
P03383	P36543	0.082084
P03383	Q96HZ7	0.33834
P03383	Q99856	0.40423
P03383	Q9H446	0.1668
P03383	A1L170	0.33834
P03383	Q9NS37	0.64587
P03383	P47928	0.30232
P03383	A6NH13	0.14464
P03383	P37198	0.70223
P03383	Q9UKX7	0.81795
P03383	P18848	0.81675
P03383	Q8N4C6	0.28996
P03383	Q9UJX2	0.40006
P03383	P62701	0.29185
P03383	P11441	0.33676
P03383	Q8N5M1	0.29228
P03383	P01215	0.26568
P03383	Q9NVC6	0.42932
P03383	Q2M1K9	0.15978
P03383	Q86X51	0.11818
P03383	P18850	0.38769
P03383	A1A5D9	0.56415
P03383	P62829	0.91388
P03383	P56211	0.2556
P03383	O76095	0.085457
P03383	O60481	0.30069
P03383	Q9UI30	0.28127
P03383	Q7Z5W3	0.52716
P03383	Q8WXU2	0.50325
P03383	Q8WUI4	0.22588
P03383	Q8NCR3	0.16762
P03383	P11172	0.26135
P03383	P62508	0.33984
P03383	Q01844	0.41741
P03383	P0C2W7	0.33834
P03383	Q92547	0.26808
P03383	O15116	0.56711
P03383	Q6UXP7	0.10116
P03383	O95873	0.60341
P03383	O00161	0.62814
P03383	Q8IUC2	0.76208
P03383	P61583	0.61783
P03383	O60869	0.48542
P03383	Q14676	0.40023
P03383	P41236	0.48557
P03383	Q8IXW0	0.26755
P03383	Q9H0G5	0.40519
P03383	P86479	0.33834
P03383	Q86X95	0.86137
P03383	O14512	0.33688
P03383	Q9UNS2	0.42101
P03383	Q96T59	0.034227
P03383	Q8TDP1	0.3969
P03383	Q9H1E3	0.56493
P03383	Q6P2H3	0.25173
P03383	Q9Y534	0.13189
P03383	Q6NSI3	0.72771
P03383	Q9UNZ5	0.60341
P03383	Q9NZL3	0.44945
P03383	P23610	0.33834
P03383	O95619	0.59117
P03383	Q9BXG8	0.22712
P03383	Q6P387	0.23895
P03383	Q96EF6	0.40407
P03383	Q8N196	0.50855
P03383	Q8NHY2	0.71553
P03383	Q96BJ3	0.46736
P03383	P83369	0.49375
P03383	Q8N8E3	0.14243
P03383	Q5T035	0.30869

P03383	Q6IE81	0.71347
P03383	Q5TEA3	0.2382
P03383	P13646	0.72519
P03383	Q15776	0.24641
P03383	Q9BXH1	0.16385
P03383	Q5T0L3	0.80426
P03383	Q92466	0.37352
P03383	P0C7W0	0.60341
P03383	Q96DM1	0.54439
P03383	Q9Y676	0.50728
P03383	Q9BQC3	0.68193
P03383	P30876	0.45925
P03383	Q6ZRV2	0.64385
P03383	Q9NPC6	0.59651
P03383	O75586	0.515
P03383	Q9BY89	0.12716
P03383	P29692	0.14503
P03383	Q99622	0.10044
P03383	Q5SXM1	0.23242
P03383	Q7Z309	0.11605
P03383	Q6ZTI0	0.33834
P03383	O95900	0.57942
P03383	Q9BXS5	0.46153
P03383	P62263	0.32522
P03383	Q5H9B9	0.10099
P03383	Q3YBR2	0.33615
P03383	Q8NET4	0.38451
P03383	Q5VWM5	0.52376
P03383	Q9H4M3	0.11282
P03383	P28062	0.15077
P03383	P62847	0.34538
P03383	P04259	0.24286
P03383	Q9BUL9	0.72257
P03383	P09629	0.71226
P03383	Q9NW38	0.36461
P03383	Q9P0J0	0.24454
P03383	Q711Q0	0.33834
P03383	Q6ZRS4	0.45556
P03383	O95271	0.39028
P03383	C9J6K1	0.351
P03383	Q9BY42	0.45623
P03383	Q9Y2A4	0.33127
P03383	Q02930	0.56517
P03383	P24864	0.45881
P03383	Q9NVV2	0.33834
P03383	P60896	0.33834
P03383	Q08ER8	0.2372
P03383	Q86Y01	0.3628
P03383	Q15545	0.72977
P03383	Q5T3J3	0.74704
P03383	Q9NTI5	0.62668
P03383	Q9BXF3	0.38685
P03383	Q7L1Q6	0.16628
P03383	P19012	0.75254
P03383	Q9UHT4	0.33834
P03383	Q6ZN01	0.36943
P03383	Q9BYS1	0.52964
P03383	Q8IYA8	0.60341
P03383	P35268	0.42577
P03383	Q9NWZ8	0.58104
P03383	Q969U7	0.37862
P03383	Q7LBC6	0.021314
P03383	Q9H8Y8	0.1482
P03383	Q96IV0	0.29817
P03383	Q8TC90	0.62063
P03383	P30281	0.53622
P03383	Q6A163	0.66284
P03383	Q8NEL0	0.054273
P03383	Q4U2R6	0.58692
P03383	P41252	0.46193
P03383	O15049	0.60222
P03383	Q8IUD2	0.60791
P03383	Q9Y2D8	0.38331
P03383	Q9H5J8	0.72651
P03383	P13051	0.8419
P03383	P0C7P2	0.077152

P03383	Q2VPK5	0.47373
P03383	Q13642	0.26638
P03383	Q9Y2D1	0.92854
P03383	Q9UI36	0.31977
P03383	Q9UJC5	0.40469
P03383	Q53ET0	1.1517
P03383	O95989	0.22572
P03383	Q658Y4	0.45972
P03383	P59942	0.35729
P03383	Q8N7B6	0.2481
P03383	Q9BV29	0.0053471
P03383	Q13829	0.51517
P03383	P09497	0.23249
P03383	Q9HAB8	0.36005
P03383	Q9Y2V2	0.32069
P03383	Q9NYP8	0.2149
P03383	Q9UBQ0	0.3071
P03383	P54136	0.57141
P03383	Q53HM4	0.75858
P03383	P10243	0.25954
P03383	Q15699	0.31828
P03383	Q86W11	0.14286
P03383	P30260	0.79986
P03383	Q8NEZ5	0.48866
P03383	Q6PI77	0.43647
P03383	P05386	0.83108
P03383	A6NIY4	0.38945
P03383	Q86WV5	0.43069
P03383	Q92624	0.51476
P03383	Q9ULC6	0.6469
P03383	Q08AG5	0.429
P03383	O15083	0.34697
P03383	Q9BU68	0.48239
P03383	Q8N490	0.2646
P03383	Q9NUL3	0.38096
P03383	Q8TDN6	0.12802
P03383	Q6ICG6	0.13747
P03383	Q92674	0.16565
P03383	P50914	0.74903
P03383	Q5SXM2	0.42766
P03383	Q2QGD7	0.51283
P03383	Q9HCE1	0.49809
P03383	Q9UPT5	0.33615
P03383	P17275	0.70702
P03383	Q04837	0.29877
P03383	Q9UM82	0.77742
P03383	O15235	0.46341
P03383	Q7RTS7	0.25546
P03383	Q8NDY4	0.33834
P03383	Q5EBM0	0.49695
P03383	P20265	0.49382
P03383	Q9UN81	0.28638
P03383	Q9NYJ8	0.27222
P03383	O95273	0.68254
P03383	Q9BWK5	0.67106
P03383	Q9Y2W1	0.70149
P03383	A6NMK8	0.53188
P03383	Q9BRJ6	0.41832
P03383	O95453	0.30002
P03383	Q86U42	0.53946
P03383	Q9UBK9	0.73755
P03383	O75444	0.24378
P03383	Q9Y291	0.31007
P03383	Q9P270	0.26596
P03383	Q92764	0.58359
P03383	Q6ZVF9	0.43912
P03383	Q96Q06	0.48545
P03383	Q4G0N7	0.46758
P03383	Q9UL03	0.55394
P03383	Q96EZ8	0.44529
P03383	Q3C1V1	0.60341
P03383	Q6ZRP5	0.33834
P03383	Q4LEZ3	0.77425
P03383	P0CL80	0.17372
P03383	Q9GZS3	0.85774
P03383	O75145	0.25515

P03383	O60437	0.46214
P03383	O15225	0.33834
P03383	Q8N6N3	0.24489
P03383	O95571	0.29682
P03383	P17480	0.76479
P03383	Q5T4T6	0.78981
P03383	Q9UL41	0.41465
P03383	Q9BTE7	0.41033
P03383	P14672	0.54871
P03383	Q8N660	0.95616
P03383	Q9Y4P9	0.17688
P03383	P61576	0.18741
P03383	Q14585	0.17124
P03383	P51523	0.10563
P03383	Q96AH0	0.29923
P03383	Q6X4T0	0.085921
P03383	Q9Y3A3	0.31251
P03383	Q8N5L8	0.69525
P03383	Q96HM7	0.44198
P03383	Q8TCE6	0.86291
P03383	Q6PEX3	0.30943
P03383	O95561	0.53065
P03383	P16333	0.39254
P03383	Q8N7X2	0.33834
P03383	P06733	0.62724
P03383	Q0IIN9	0.055387
P03383	O60664	0.2766
P03383	O75150	0.3266
P03383	Q5T0W9	0.020767
P03383	Q8N720	0.70518
P03383	O94973	0.23894
P03383	Q8IUI4	0.1539
P03383	Q8N9Z9	0.42932
P03383	P48556	0.24186
P03383	Q9NWF9	0.53377
P03383	Q9H3R5	0.29147
P03383	Q9P032	0.29696
P03383	Q14135	0.19547
P03383	O95073	0.79563
P03383	P63167	0.94058
P03383	Q6NUI1	0.33433
P03383	Q8NC51	0.48148
P03383	Q2M3D2	0.17048
P03383	P61244	0.65792
P03383	Q9GZY8	0.18472
P03383	O00743	0.49196
P03383	P19838	0.55066
P03383	Q3KNT7	0.094537
P03383	P07438	0.23866
P03383	Q8NBA8	0.20328
P03383	Q96AV8	0.20687
P03383	Q9Y5L0	0.69192
P03383	O00629	0.26492
P03383	Q5T7N7	0.33834
P03383	Q9Y421	0.24093
P03383	Q9NRI7	0.33834
P03383	Q96QR8	0.32564
P03383	A8MV65	0.58845
P03383	P35869	0.27055
P03383	Q6ZN79	0.28972
P03383	Q9BWC9	0.74742
P03383	Q9H3C7	0.83227
P03383	Q8IWZ3	0.69844
P03383	Q9Y5J9	0.34955
P03383	Q9ULJ6	0.27776
P03383	P05813	0.28704
P03383	Q8N9R8	0.51743
P03383	Q8N7Q3	0.23279
P03383	Q8N141	0.34707
P03383	Q8TA94	0.32002
P03383	Q9NPI8	0.61201
P03383	Q9BRT6	0.30048
P03383	O14964	0.36216
P03383	Q14CS0	0.078793
P03383	P56693	0.48567
P03383	Q1ZZU3	0.39132

P03383	P22087	0.45233
P03383	Q9C0H9	0.092425
P03383	Q9NSD9	0.55166
P03383	O14818	0.45704
P03383	Q5TBB1	0.57757
P03383	P17028	0.50392
P03383	P49591	0.29513
P03383	Q8WUB2	0.15218
P03383	Q9BVC3	0.37979
P03383	P67809	0.47023
P03383	Q8N8D1	0.48736
P03383	O75175	1.0079
P03383	Q9H079	0.28591
P03383	Q8WV24	0.40809
P03383	Q8NCR6	0.27922
P03383	Q9H875	0.30582
P03383	Q9UBD0	0.85108
P03383	Q96CT7	0.10045
P03383	Q16534	0.24365
P03383	K7EIZ4	0.33834
P03383	A2RU30	0.45556
P03383	Q9UL40	0.40862
P03383	Q15637	0.22598
P03383	Q9HBQ8	0.5567
P03383	P0C880	0.33834
P03383	Q49AM1	0.22075
P03383	O14599	0.33834
P03383	Q14137	0.60882
P03383	Q6KB66	0.28291
P03383	A6NCN2	0.622
P03383	P0C7V0	0.33834
P03383	P60369	0.5733
P03383	P07315	0.32863
P03383	Q9ULC4	0.25603
P03383	O15205	0.33427
P03383	Q99627	0.14526
P03383	Q5VZ46	0.11631
P03383	Q9NNW5	0.36237
P03383	Q9Y399	0.66904
P03383	Q9HCG8	0.071642
P03383	Q9Y3Y2	0.84736
P03383	Q9UJX3	0.46355
P03383	P20073	0.19718
P03383	P46937	0.61607
P03383	Q63HQ0	0.33834
P03383	P61578	0.33986
P03383	Q5JRK9	0.8942
P03383	Q9Y603	0.94184
P03383	O15446	0.063531
P03383	F2Z3M2	0.33834
P03383	Q9C0D2	0.50284
P03383	P0C838	0.40411
P03383	A8K830	0.33834
P03383	Q9NX53	0.1668
P03383	O75665	0.43032
P03383	Q14241	0.28613
P03383	P61758	0.35409
P03383	Q8IYS8	0.30521
P03383	Q8WXX7	0.29903
P03383	Q96CM3	0.27181
P03383	Q96PQ6	0.3432
P03383	Q9NSJ1	0.2137
P03383	Q7Z3B4	0.59189
P03383	Q9NR28	0.54678
P03383	Q8NC74	0.24254
P03383	Q8NAJ2	0.048061
P03383	P0C5Y4	0.52964
P03383	A6NGU7	0.085404
P03383	Q5VTE6	0.011966
P03383	Q6ZVN7	0.047161
P03383	Q6UN15	0.79589
P03383	P84090	0.33439
P03383	P56279	0.49934
P03383	Q8WYA6	0.52564
P03383	Q9Y608	0.26634
P03383	P62258	0.57796

P03383	P09234	0.35616
P03383	P13056	0.46703
P03383	Q01658	0.22066
P03383	Q9GZV5	0.7949
P03383	Q02952	0.14657
P03383	Q9BUY5	0.30703
P03383	Q9BT04	0.25186
P03383	Q9ULX9	0.25153
P03383	P78346	0.4462
P03383	Q9UMX1	0.5766
P03383	Q8NG48	0.022385
P03383	Q86VN1	0.23581
P03383	Q5T1C6	0.41228
P03383	Q6ZUJ4	0.43143
P03383	Q6P9A1	0.27634
P03383	Q9HB19	0.29954
P03383	Q6IA86	0.35266
P03383	O60583	0.75127
P03383	Q9UN37	0.32278
P03383	P54198	0.86027
P03383	Q9UHF7	0.32061
P03383	Q17RA5	0.33834
P03383	Q8N8P6	0.036386
P03383	Q8WXB4	0.14384
P03383	O75564	0.20431
P03383	Q6U949	0.33834
P03383	O43439	0.70908
P03383	Q9BYU5	0.28322
P03383	Q15834	0.82568
P03383	Q9BW61	0.45738
P03383	Q92900	0.3582
P03383	Q6ZR93	0.33834
P03383	Q9NVH2	0.19157
P03383	Q86Z20	0.76454
P03383	Q06546	0.9726
P03383	Q86V42	0.53184
P03383	P62861	0.77961
P03383	O75368	0.18084
P03383	O75334	0.32542
P03383	Q9H5I1	0.44662
P03383	Q7Z5V6	0.23858
P03383	Q7L0X2	0.20916
P03383	Q3LI68	0.68909
P03383	P56181	0.44714
P03383	Q9BWN1	0.60341
P03383	Q9NWA0	1.2342
P03383	O14604	0.47502
P03383	Q3C1V8	0.39708
P03383	Q7L3T8	0.30705
P03383	P08133	0.41841
P03383	Q9H091	0.18718
P03383	Q9H714	0.2377
P03383	P08238	0.44916
P03383	O75391	0.47817
P03383	O75953	0.45785
P03383	O75969	0.26527
P03383	P59022	0.36474
P03383	A6NME0	0.20361
P03383	Q14532	0.51386
P03383	Q92917	0.66601
P03383	P30047	0.41211
P03383	Q13868	0.38965
P03383	P24539	0.1252
P03383	A6NEY8	0.15789
P03383	Q00839	0.26692
P03383	Q92988	0.38762
P03383	A6NHR8	0.33834
P03383	Q9BRD0	0.45385
P03383	P78352	0.45
P03383	Q99436	0.47769
P03383	Q5T1N1	0.80145
P03383	P43364	0.55947
P03383	Q9UPP1	0.31544
P03383	Q2NL98	0.68038
P03383	Q3KNV8	0.65799
P03383	Q8NC26	0.27721

P03383	Q9NZ63	0.25487
P03383	Q5MNV8	0.27322
P03383	Q86UQ8	0.19547
P03383	Q16186	0.31754
P03383	Q8IUG1	0.55334
P03383	P18621	0.3873
P03383	Q9P2B7	0.60341
P03383	P52294	0.75349
P03383	P52594	0.26061
P03383	O94874	0.54708
P03383	Q16890	0.26245
P03383	Q5PRF9	0.34964
P03383	Q13112	1.0919
P03383	O15372	0.42218
P03383	Q9BVP2	0.14349
P03383	O60516	0.14018
P03383	Q92539	0.30339
P03383	Q92890	0.47291
P03383	Q9Y5B9	0.085568
P03383	P08621	0.28377
P03383	Q8TBR4	0.2797
P03383	Q2NKJ3	0.24593
P03383	Q3LHN0	0.81527
P03383	Q9H0A0	0.40968
P03383	Q8WWM1	0.57601
P03383	Q96LI5	0.36623
P03383	Q9NQR1	0.50973
P03383	Q6V9R5	0.30957
P03383	Q9Y3Y4	0.37452
P03383	P21673	0.12322
P03383	Q6I9Y2	0.59899
P03383	A6NI79	0.61902
P03383	O75346	0.28211
P03383	Q701N2	0.33834
P03383	Q9BT49	0.49456
P03383	Q96DC7	0.84226
P03383	Q13114	0.23734
P03383	Q96C74	0.1368
P03383	Q96HG1	0.33834
P03383	Q9BYK8	0.27681
P03383	P49790	0.77638
P03383	Q5JVX7	0.55335
P03383	Q86T75	0.34297
P03383	O14802	0.077041
P03383	P46776	0.72715
P03383	O95990	0.18414
P03383	Q9UNY4	0.33152
P03383	O75170	0.51242
P03383	Q8NE31	0.019715
P03383	Q03060	0.29081
P03383	Q9Y2X0	0.36423
P03383	Q96GC5	0.4211
P03383	O75821	0.35351
P03383	Q6L8H4	0.33834
P03383	Q2YD98	0.12777
P03383	Q3LI58	0.33834
P03383	Q14764	0.78817
P03383	Q9BPZ3	0.34312
P03383	Q6ZTU2	0.59553
P03383	P57060	0.45777
P03383	P26358	0.26352
P03383	Q96EY5	0.39343
P03383	P32780	0.55006
P03383	Q8NFZ5	0.62672
P03383	O75694	0.42839
P03383	Q14154	0.29562
P03383	Q8NHS9	0.15019
P03383	Q8WVP5	0.74227
P03383	Q7Z3Y9	0.75254
P03383	Q9NQS1	0.21242
P03383	A6NK89	0.41636
P03383	Q9H211	0.47015
P03383	Q8IV54	0.76835
P03383	Q9NPA3	0.88605
P03383	Q53EZ4	0.42969
P03383	Q8NDN9	0.41146

P03383	P52597	0.39889
P03383	Q9NXR7	0.19519
P03383	Q9UPY8	0.70489
P03383	P60413	0.5733
P03383	Q9Y2H0	0.22666
P03383	Q5W1A9	0.29393
P03383	P15880	0.72412
P03383	Q53HC0	0.7977
P03383	Q5XUX0	0.4668
P03383	O60832	0.4935
P03383	P0C5K6	0.33834
P03383	P23396	0.53921
P03383	Q16384	0.323
P03383	Q05877	0.57894
P03383	Q8N5N7	0.4543
P03383	Q0P140	0.19547
P03383	Q9UBL0	0.20448
P03383	P0C7I6	0.54338
P03383	Q8IWE5	0.22009
P03383	Q5T8I3	0.33705
P03383	Q14749	0.38716
P03383	Q8N983	0.29487
P03383	Q96EY1	0.27751
P03383	P19532	0.46963
P03383	Q8N5D0	0.26263
P03383	Q8N5Q1	0.87506
P03383	Q96K31	0.38845
P03383	P15259	0.49853
P03383	B2CW77	0.33834
P03383	Q969I3	0.32527
P03383	O95391	0.11021
P03383	O75312	0.22144
P03383	Q53FN2	0.14523
P03383	O95424	0.44228
P03383	Q6ICC9	0.32778
P03383	Q9H4B6	0.34891
P03383	Q8NHY6	0.25927
P03383	Q9H246	0.21235
P03383	H3BMG3	0.16197
P03383	P16415	0.22428
P03383	Q3B7T1	0.57625
P03383	Q3LI83	0.66338
P03383	Q9Y3D3	0.43447
P03383	Q9Y6R6	0.24149
P03383	O75154	0.5128
P03383	Q9BQ90	0.4948
P03383	Q4VC44	0.29426
P03383	Q9BQ83	0.42206
P03383	Q9NWW7	0.65596
P03383	Q15024	0.46092
P03383	P17947	0.53178
P03383	Q8N5N4	0.35339
P03383	Q9BTP7	0.63438
P03383	Q5HYR2	0.62578
P03383	Q9UN30	0.12072
P03383	Q9NPJ4	1.237
P03383	Q59GR2	0.053806
P03383	Q6UWZ7	0.23542
P03383	Q9Y4W2	0.65114
P03383	Q5VT40	0.41221
P03383	O14910	0.48326
P03383	Q5VWN6	0.075959
P03383	Q8WVR3	0.60341
P03383	P63279	0.60239
P03383	Q69YH5	0.6495
P03383	P20248	0.79525
P03383	O95521	0.26217
P03383	I6L9F6	0.086838
P03383	Q9H9T3	0.59427
P03383	Q6ZS92	0.151
P03383	Q701N4	0.33834
P03383	Q86UW6	0.098585
P03383	Q3KRA6	0.82324
P03383	Q9BYQ2	0.5733
P03383	P18615	0.79738
P03383	Q96LA8	0.48808

P03383	Q9Y2V0	0.088446
P03383	O43482	0.31204
P03383	O95429	0.12642
P03383	Q9Y473	0.17739
P03383	Q8IVG9	0.13904
P03383	A0AVK6	0.2637
P03383	Q9BPX3	0.61283
P03383	Q9BPW8	0.37403
P03383	Q8N6G1	0.33834
P03383	P60510	0.55807
P03383	P15502	0.25979
P03383	Q7Z4G1	1.0239
P03383	Q8N7H5	0.68429
P03383	Q86XI8	0.38298
P03383	P28070	0.28158
P03383	Q15021	0.60878
P03383	O15226	0.58315
P03383	Q9BUG6	0.1454
P03383	Q2M329	0.18862
P03383	Q9BUV0	0.60341
P03383	Q9UHL3	0.083517
P03383	P51826	0.17522
P03383	P81877	0.27942
P03383	Q9Y615	0.07375
P03383	Q9BUE0	0.84937
P03383	Q5T5S1	0.29034
P03383	Q6ZP98	0.17879
P03383	A7MCY6	0.38891
P03383	P61513	0.38314
P03383	Q9BSF4	0.2089
P03383	P98182	0.094431
P03383	Q9ULD9	0.78579
P03383	O75934	0.47359
P03383	Q9H9G7	0.4624
P03383	Q59GN2	0.22152
P03383	Q9P1Z2	0.55575
P03383	P40425	0.4202
P03383	Q8TCZ7	0.30898
P03383	Q6L8G8	0.33834
P03383	O14598	0.66758
P03383	Q9Y605	1.0963
P03383	Q9UMS4	0.41661
P03383	Q8WXC6	0.1709
P03383	Q13363	0.31544
P03383	Q96QA5	0.2505
P03383	O95257	0.22134
P03383	Q9NPJ8	0.22619
P03383	Q16576	0.31899
P03383	Q8WW01	0.26231
P03383	P49419	0.37172
P03383	Q6ZMV8	0.33306
P03383	Q8IVM7	0.33834
P03383	Q7Z3Y7	0.4922
P03383	O60477	0.37929
P03383	Q92793	0.31292
P03383	Q5VU69	0.40297
P03383	Q13901	0.85934
P03383	Q96ST2	0.27084
P03383	Q8N823	0.24149
P03383	O75531	0.50359
P03383	A6NGR9	0.38389
P03383	P40855	0.30349
P03383	Q96GV9	0.28379
P03383	Q7RTV0	0.28589
P03383	Q96LT7	0.43861
P03383	O15063	0.17059
P03383	Q96QE5	0.66358
P03383	Q9UQN3	0.27881
P03383	Q86SZ2	0.51344
P03383	Q8TF39	0.34707
P03383	Q13526	0.40586
P03383	P54274	0.27775
P03383	Q9BRP8	0.73991
P03383	Q9BZG8	0.10311
P03383	O00566	0.5726
P03383	P35325	0.33834

P03383	Q02078	0.30731
P03383	P61960	0.088472
P03383	P59090	0.33834
P03383	Q6ZNE5	0.19597
P03383	Q6FI81	0.15832
P03383	Q96NA2	0.78804
P03383	C9J302	0.38282
P03383	Q9Y224	1.1048
P03383	Q0VG06	0.15273
P03383	Q8NFH3	0.74228
P03383	Q8NHQ1	0.92796
P03383	O00410	0.85473
P03383	P35226	0.41368
P03383	Q99623	0.77482
P03383	Q4W4Y0	0.040186
P03383	A2RU37	0.1006
P03383	Q9BQY9	0.27791
P03383	Q8N1G2	0.16359
P03383	Q92800	0.34835
P03383	Q9GZR1	0.23528
P03383	Q969S8	0.59012
P03383	Q9UJW9	1.2242
P03383	Q2KJ03	0.15382
P03383	Q5T0F9	0.18125
P03383	P35900	0.75254
P03383	Q2VPB7	0.53313
P03383	P14651	0.079085
P03383	Q9NRC8	0.94914
P03383	Q2VY69	0.21802
P03383	Q8WX92	1.1625
P03383	O75940	0.44461
P03383	Q5VT28	0.24723
P03383	Q6NT89	0.78152
P03383	Q9BYC8	0.49054
P03383	Q86YD1	0.61892
P03383	P54259	0.69345
P03383	Q92575	0.60086
P03383	Q5T6V5	0.23045
P03383	P51668	0.43503
P03383	Q9BWH6	0.47227
P03383	Q8TD35	0.60341
P03383	Q5T6C5	0.48392
P03383	O43790	0.66532
P03383	Q8TAT8	0.094744
P03383	Q9H6R7	0.029377
P03383	Q53G26	0.35989
P03383	Q9UDY2	0.59211
P03383	A6NHS1	0.38808
P03383	P13682	0.19989
P03383	Q9NVR2	0.66555
P03383	Q8NEQ6	0.14092
P03383	Q14999	0.64454
P03383	Q99619	0.50366
P03383	Q9UIS9	0.34267
P03383	Q8NI08	0.56609
P03383	Q08379	0.11649
P03383	Q9Y468	0.71776
P03383	O75496	0.65885
P03383	P57055	0.4885
P03383	Q96PP4	0.12627
P03383	Q14157	0.50646
P03383	Q99741	0.39368
P03383	Q7Z5K2	0.46092
P03383	P12956	0.11131
P03383	A6NFT4	0.026209
P03383	Q69YL0	0.33834
P03383	Q9NZF1	0.095817
P03383	Q9BWJ5	0.34648
P03383	Q8N6U2	0.19399
P03383	Q13127	0.35437
P03383	Q15276	0.11138
P03383	P86496	0.33834
P03383	Q15633	0.60568
P03383	Q9ULV3	0.085347
P03383	O75530	0.43437
P03383	Q5VIR6	0.56009

40585	P03383	Q8WWR9	0.44518
40586	P03383	Q3B726	0.033171
40587	P03383	Q9NWD9	0.31004
40588	P03383	Q13425	0.201
40589	P03383	Q6ZQV5	0.24149
40590	P03383	Q8TEA8	0.66314
40591	P03383	O00148	0.1599
40592	P03383	Q9ULW6	0.32897
40593	P03383	Q8N814	0.58007
40594	P03383	Q9BVN2	0.27902
40595	P03383	Q9UKK6	0.46151
40596	P03383	Q96M11	0.69408
40597	P03383	Q8TAP9	1.0726
40598	P03383	P43355	0.79804
40599	P03383	A6NCN8	0.60341
40600	P03383	Q1W6H9	0.82495
40601	P03383	Q96NG5	0.29714
40602	P03383	Q5TC04	0.0065078
40603	P03383	Q9H814	0.62051
40604	P03383	P0C879	0.33834
40605	P03383	Q53T94	0.24105
40606	P03383	Q14593	0.3213
40607	P03383	Q8IV32	0.50098
40608	P03383	Q8N726	0.44178
40609	P03383	Q8N1F1	0.33834
40610	P03383	Q9H4A5	0.65546
40611	P03383	Q96S07	0.33834
40612	P03383	P25787	0.46127
40613	P03383	Q9NVU7	0.51028
40614	P03383	Q9P0T4	0.22614
40615	P03383	Q96BN8	0.14638
40616	P03383	Q96CW6	0.7392
40617	P03383	A4D1E9	0.67382
40618	P03383	Q9H1L0	0.40361
40619	P03383	Q8WWX9	0.19414
40620	P03383	Q5C9Z4	0.48053
40621	P03383	Q9BSQ5	0.13673
40622	P03383	P62306	0.16265
40623	P03383	Q6ZUF6	0.024215
40624	P03383	Q5TG08	0.59139
40625	P03383	Q6ICG8	0.96018
40626	P03383	P22415	0.35483
40627	P03383	Q6PRD7	0.33834
40628	P03383	Q6BDS2	0.18949
40629	P03383	O76021	0.21301
40630	P03383	Q969S3	0.47367
40631	P03383	Q96BY2	0.28506
40632	P03383	Q9HB75	0.39046
40633	P03383	Q9UJ94	0.33834
40634	P03383	Q14695	0.34419
40635	P03383	P27797	0.34638
40636	P03383	Q9H0U6	0.28972
40637	P03383	O76013	0.66522
40638	P03383	Q8TEV9	0.35738
40639	P03383	Q9UBI9	0.58216
40640	P03383	O96017	0.54675
40641	P03383	O95905	0.51549
40642	P03383	Q8NEH6	0.71611
40643	P03383	Q5PR19	0.20649
40644	P03383	P08708	0.2332
40645	P03383	Q8TB03	0.42059
40646	P03383	P06396	0.18832
40647	P03383	Q8IYN2	0.88219
40648	P03383	Q9HAT8	0.35059
40649	P03383	Q6NT46	0.17372
40650	P03383	B3EWG6	0.60341
40651	P03383	Q13503	0.45634
40652	P03383	A8MPV3	0.041445
40653	P03383	P52741	0.13841
40654	P03383	Q6P444	0.51712
40655	P03383	Q8NB42	0.33974
40656	P03383	P22732	0.49099
40657	P03383	Q9HCC9	0.17246
40658	P03383	Q14693	0.18961
40659	P03383	Q6ZVT6	0.028692
40660	P03383	Q8WUH2	0.43141

40661	P03383	Q5BKZ1	0.38344
40662	P03383	Q9Y6D9	0.64542
40663	P03383	P0CG34	0.45586
40664	P03383	A8MTZ7	0.11288
40665	P03383	P35251	0.37025
40666	P03383	Q96EY4	0.35359
40667	P03383	Q7Z2W4	0.26036
40668	P03383	Q9UNK9	0.12333
40669	P03383	Q6QNY1	0.50307
40670	P03383	O95848	0.37768
40671	P03383	Q9BSB4	0.16174
40672	P03383	Q5SZD4	0.36008
40673	P03383	P25788	0.48239
40674	P03383	Q96LW7	0.53237
40675	P03383	Q9H0E3	0.53499
40676	P03383	P0CB33	0.29965
40677	P03383	Q8IW35	0.82645
40678	P03383	Q15750	0.28889
40679	P03383	Q5HYW2	0.64276
40680	P03383	Q9BQY4	0.64001
40681	P03383	Q9BPX1	0.037316
40682	P03383	Q9Y3P8	0.33854
40683	P03383	Q13620	0.52852
40684	P03383	Q9NP98	0.40695
40685	P03383	Q3MJ40	0.011001
40686	P03383	Q5MJ70	0.38945
40687	P03383	Q9BX26	0.96283
40688	P03383	Q8N9T8	0.99314
40689	P03383	Q9H008	0.19277
40690	P03383	Q8NEY8	0.5278
40691	P03383	Q6V702	0.095248
40692	P03383	O75928	0.55074
40693	P03383	Q9NZ52	0.25406
40694	P03383	Q9BZI7	0.53402
40695	P03383	Q9NZD4	0.34818
40696	P03383	Q5HYW3	0.55535
40697	P03383	Q9NSI2	0.60341
40698	P03383	Q15543	0.79809
40699	P03383	Q8WV07	0.3024
40700	P03383	Q0VG73	0.33834
40701	P03383	Q9Y250	0.7391
40702	P03383	Q9BYQ6	0.5733
40703	P03383	Q9H1J1	0.88747
40704	P03383	P50748	0.5426
40705	P03383	Q96NY9	0.39182
40706	P03383	Q14781	0.55159
40707	P03383	Q14498	0.61618
40708	P03383	Q8NCU7	0.75185
40709	P03383	Q9BW92	0.25837
40710	P03383	Q8NE18	0.96124
40711	P03383	Q8WVM7	0.55807
40712	P03383	P61353	0.4006
40713	P03383	Q6P2E9	0.71817
40714	P03383	O60356	0.69051
40715	P03383	Q9H5F2	0.42331
40716	P03383	Q03938	0.31143
40717	P03383	Q93062	0.64132
40718	P03383	Q8IXQ4	0.60341
40719	P03383	Q538Z0	0.33834
40720	P03383	Q03933	0.49752
40721	P03383	Q13505	0.6163
40722	P03383	P05412	0.62726
40723	P03383	Q7Z745	0.25846
40724	P03383	Q9Y314	0.65943
40725	P03383	Q96DI7	0.46061
40726	P03383	Q16526	0.23693
40727	P03383	Q9BYG4	0.43699
40728	P03383	Q8N9M1	0.16327
40729	P03383	Q8IYB9	0.24064
40730	P03383	Q70EL1	0.26939
40731	P03383	Q96MC4	0.62518
40732	P03383	Q9C073	0.65824
40733	P03383	Q5H9M0	0.70277
40734	P03383	Q7KZ85	0.27839
40735	P03383	O60888	0.25416
40736	P03383	Q8N5I9	0.60341

40737	P03383	Q69YI7	0.87175
40738	P03383	P27348	0.19417
40739	P03383	Q9H5Z6	0.60341
40740	P03383	Q13129	0.47987
40741	P14079	Q9Y3C0	0.41839
40742	P14079	O15255	1.1715
40743	P14079	P31751	0.16487
40744	P14079	Q96AQ9	0.73088
40745	P14079	Q6ZQY7	1.1125
40746	P14079	Q969J2	0.56913
40747	P14079	A6NNH0	1.4681
40748	P14079	Q5VV42	1.0005
40749	P14079	Q9H6X5	0.31805
40750	P14079	P04083	0.43219
40751	P14079	Q96DC7	1.6464
40752	P14079	Q9H0U9	0.51138
40753	P14079	P0DM35	1.3448
40754	P14079	P36543	0.39776
40755	P14079	Q96M91	1.8372
40756	P14079	Q6ZRN7	1.1715
40757	P14079	Q14CX7	0.84768
40758	P14079	O14776	1.1822
40759	P14079	Q5SRI9	1.3902
40760	P14079	Q63HN1	1.1198
40761	P14079	Q96LP4	0.73938
40762	P14079	Q5TC84	0.7178
40763	P14079	Q5EBL4	0.73509
40764	P14079	P02808	1.1392
40765	P14079	Q5TAQ9	0.37638
40766	P14079	Q13228	1.3362
40767	P14079	Q9UQL6	0.46344
40768	P14079	O60671	0.96695
40769	P14079	Q15629	0.79113
40770	P14079	Q9NQ39	0.23329
40771	P14079	P51854	0.47389
40772	P14079	Q6P1M9	0.81288
40773	P14079	Q96JN0	1.4954
40774	P14079	A8MUM7	0.12848
40775	P14079	Q9Y2S0	0.29856
40776	P14079	Q15637	0.25535
40777	P14079	Q8IV31	0.97018
40778	P14079	Q16655	1.2149
40779	P14079	O94923	0.49546
40780	P14079	Q8NDH6	0.73756
40781	P14079	Q7L2K0	0.59878
40782	P14079	F5H4A9	1.2485
40783	P14079	Q5VZR2	1.8285
40784	P14079	P0CH98	0.42881
40785	P14079	Q15916	0.1949
40786	P14079	Q70EL1	0.69532
40787	P14079	Q9NZ81	1.8162
40788	P14079	Q8NFH5	0.66106
40789	P14079	O00625	0.97985
40790	P14079	P28838	1.3197
40791	P14079	Q8N6M3	1.0833
40792	P14079	Q96SC8	0.29941
40793	P14079	Q3T8J9	0.82292
40794	P14079	Q9BWM5	0.60552
40795	P14079	Q9BXW6	0.33207
40796	P14079	Q9BRP4	0.81334
40797	P14079	P98168	0.74722
40798	P14079	P59894	0.80039
40799	P14079	O43809	0.85007
40800	P14079	Q5JTD0	1.8485
40801	P14079	O75031	1.0577
40802	P14079	Q6P4F2	0.24347
40803	P14079	Q8TCE6	1.6459
40804	P14079	Q8N4C0	0.91852
40805	P14079	Q9UH36	0.93836
40806	P14079	C9J302	1.1223
40807	P14079	O60225	0.42957
40808	P14079	Q49AS3	1.3238
40809	P14079	Q494U1	0.5073
40810	P14079	P01215	0.90315
40811	P14079	Q9NY59	0.42209
40812	P14079	Q2TAM9	1.8285

P14079	A0A183	1.1715
P14079	Q96QK1	0.98148
P14079	P07199	1.1986
P14079	Q6VY07	0.92802
P14079	A0JNW5	1.2177
P14079	Q8IYX0	1.0042
P14079	Q7Z7L8	1.8351
P14079	Q96NU1	0.4352
P14079	Q8N7L0	0.86339
P14079	Q8NHY6	0.81594
P14079	Q9Y3U8	1.0283
P14079	A8MZ97	1.6986
P14079	Q8IWY9	1.0075
P14079	Q6P1N9	0.79526
P14079	Q9NRJ2	1.1715
P14079	Q9NQG7	0.39655
P14079	Q9BQC6	1.4778
P14079	Q5XUX0	1.0485
P14079	Q5TGZ0	0.83596
P14079	Q9NV72	1.0073
P14079	Q9H9H4	1.269
P14079	O15442	0.48908
P14079	A7E2V4	1.9641
P14079	Q02952	1.0846
P14079	P56181	1.239
P14079	P27635	0.87172
P14079	Q92664	0.42258
P14079	A6NH13	1.0855
P14079	Q9BQD7	0.82293
P14079	Q32M92	0.23551
P14079	Q8IXM3	1.0197
P14079	Q15691	0.89408
P14079	Q8N9N8	0.29399
P14079	Q14585	0.57154
P14079	Q6AW86	0.51329
P14079	P48552	0.73066
P14079	B3KS81	0.45711
P14079	Q6NUQ1	0.7997
P14079	O95373	1.3001
P14079	P15622	0.62309
P14079	Q6R327	0.33379
P14079	P52738	0.54251
P14079	Q8N377	1.1715
P14079	Q96BQ5	1.416
P14079	Q96EK7	0.28299
P14079	P22695	0.12658
P14079	Q3SXP7	1.095
P14079	Q4VC31	0.033046
P14079	Q96C00	0.42501
P14079	Q7Z5D8	1.2576
P14079	Q9UKA4	0.38977
P14079	Q5SXM2	0.72564
P14079	O43292	0.53737
P14079	Q8NEQ5	0.85625
P14079	Q14980	1.3058
P14079	P0CG08	1.0766
P14079	Q5XUX1	0.56221
P14079	Q9Y5Y5	0.71972
P14079	Q8IYW4	0.30078
P14079	Q13952	0.77627
P14079	O60284	0.22779
P14079	Q7Z4L0	0.67613
P14079	Q9H1X1	0.82265
P14079	Q9UHU1	1.1715
P14079	Q96M15	0.39576
P14079	Q8WYA0	1.2527
P14079	Q6PF06	1.2773
P14079	Q5TBE3	0.82065
P14079	A8MPS7	0.3767
P14079	Q9UJN7	0.57015
P14079	P52815	0.96821
P14079	Q9BYG7	0.27853
P14079	O15194	0.44063
P14079	Q9H3L0	1.3222
P14079	A6NF36	1.0589
P14079	P84098	0.86734

40889	P14079	P46779	1.3548
40890	P14079	Q9UQR1	0.83709
40891	P14079	P28065	1.1615
40892	P14079	Q8N9K3	1.3222
40893	P14079	Q9GZN8	0.79115
40894	P14079	O15379	0.59534
40895	P14079	Q8NBV4	1.3093
40896	P14079	O75886	1.3933
40897	P14079	Q6ZNR8	1.1715
40898	P14079	Q0D2J5	0.69509
40899	P14079	Q8NDD1	0.47656
40900	P14079	A6NFL8	1.4427
40901	P14079	Q9HBX3	1.1715
40902	P14079	Q5RI15	0.74516
40903	P14079	Q96CS4	0.5913
40904	P14079	Q96E40	0.38943
40905	P14079	Q658L1	1.501
40906	P14079	Q8IWP9	1.6301
40907	P14079	Q8NA92	1.2179
40908	P14079	A7XYQ1	0.98473
40909	P14079	Q9BZP3	0.92288
40910	P14079	P07320	1.3894
40911	P14079	Q9UPW6	0.68145
40912	P14079	Q9UGB4	1.1715
40913	P14079	Q8NI29	0.76269
40914	P14079	Q9UK12	0.63029
40915	P14079	Q9H204	1.3275
40916	P14079	Q6P0N0	1.2835
40917	P14079	Q8TF09	1.049
40918	P14079	Q9BXU3	1.1938
40919	P14079	A6NKH3	0.95372
40920	P14079	Q7L2H7	0.76803
40921	P14079	Q9H1C7	1.2485
40922	P14079	Q9UHB4	0.63776
40923	P14079	Q9UDX5	0.57616
40924	P14079	Q86XF0	0.35153
40925	P14079	Q2M2Z5	1.5377
40926	P14079	Q13492	0.28001
40927	P14079	A6NMK8	1.3357
40928	P14079	Q9BQ08	1.0741
40929	P14079	Q04837	0.76111
40930	P14079	Q9BYD6	1.1168
40931	P14079	Q14525	0.89111
40932	P14079	Q9NSD4	0.66428
40933	P14079	Q9BW72	0.83563
40934	P14079	Q15560	1.0059
40935	P14079	Q9NUR3	0.90496
40936	P14079	O75556	0.70904
40937	P14079	Q9Y296	0.39562
40938	P14079	Q9H8S5	0.3842
40939	P14079	E9PIF3	0.30565
40940	P14079	Q96CW1	0.68662
40941	P14079	Q8WUH1	0.37459
40942	P14079	O95070	0.70185
40943	P14079	Q8N944	1.6794
40944	P14079	Q99436	1.2175
40945	P14079	Q96A56	1.4257
40946	P14079	Q8TD35	1.3222
40947	P14079	P00374	0.21736
40948	P14079	Q8N402	0.27494
40949	P14079	H0UI37	0.85048
40950	P14079	P15259	0.86177
40951	P14079	O95159	1.099
40952	P14079	Q9Y5B9	0.30282
40953	P14079	Q8N2W9	0.90686
40954	P14079	P07316	0.12389
40955	P14079	Q32MQ0	1.1263
40956	P14079	H3BPM6	0.25199
40957	P14079	Q8N1Y9	1.1715
40958	P14079	Q86VS3	0.45687
40959	P14079	Q8N9N2	0.39914
40960	P14079	Q6ZWC4	0.86514
40961	P14079	Q7Z2T5	0.46995
40962	P14079	Q7L8J4	0.9745
40963	P14079	O43516	0.46823
40964	P14079	Q8IXQ4	1.3222

P14079	Q96KP6	0.15225
P14079	Q9HBT8	0.51539
P14079	Q96AB6	0.2333
P14079	P52741	0.53765
P14079	Q9HAK2	0.16611
P14079	Q5THR3	0.37892
P14079	A2VDF0	0.88775
P14079	Q9NVL8	0.63386
P14079	Q9UHY8	0.66549
P14079	O75192	0.99163
P14079	P17707	0.29124
P14079	Q9P0N5	1.1594
P14079	Q9BW11	0.43586
P14079	Q8WWM9	0.471
P14079	O75112	0.5035
P14079	Q12986	0.33329
P14079	O14519	0.886
P14079	Q5VZT2	0.81383
P14079	Q92844	0.66016
P14079	Q6P6B1	1.3222
P14079	A6NNJ1	0.57067
P14079	Q96FX2	1.161
P14079	Q8IZC7	0.61073
P14079	Q3ZCQ8	0.28227
P14079	O14810	0.59511
P14079	Q04725	0.78858
P14079	Q6VN20	0.4612
P14079	P51959	0.48417
P14079	P52565	0.18799
P14079	Q9BXL7	0.24486
P14079	P35711	0.96546
P14079	Q96D03	0.5216
P14079	Q3LIE5	0.892
P14079	Q9H668	0.31154
P14079	Q8IZU0	1.532
P14079	Q7Z4U5	1.5349
P14079	Q9BPZ2	1.3481
P14079	Q9NRQ2	0.48833
P14079	Q99623	1.1241
P14079	P22528	1.0119
P14079	Q9NQX1	0.72452
P14079	P54274	0.56024
P14079	P52758	0.39559
P14079	Q8IUC0	1.1516
P14079	C9JC47	1.1715
P14079	Q5GJ75	0.27108
P14079	Q9BUV8	0.62727
P14079	O43261	0.67208
P14079	P05937	0.28807
P14079	P36542	0.49027
P14079	Q3SY52	0.62154
P14079	Q8TE04	0.90801
P14079	P16452	0.34774
P14079	Q8IYJ2	1.5051
P14079	O43674	1.2573
P14079	A6NMX2	0.17668
P14079	E9PGG2	0.24936
P14079	Q9Y4P9	0.97187
P14079	O43615	0.30925
P14079	Q9Y4G2	0.13146
P14079	Q96ST2	0.72145
P14079	B1ATL7	0.81221
P14079	O00463	0.43789
P14079	Q12929	0.30285
P14079	Q9H320	1.5357
P14079	Q9BZ68	0.025392
P14079	Q9NQW6	0.4981
P14079	Q86YV9	1.5691
P14079	Q14CX5	1.2485
P14079	Q9NR46	0.47374
P14079	Q5K130	0.88918
P14079	A8MUX0	1.1313
P14079	A6NCX1	0.11595
P14079	O75324	1.0428
P14079	A8MUN3	1.1715
P14079	Q92552	1.0019

P14079	Q8N5M1	0.91708
P14079	P62875	0.84581
P14079	Q4G0U5	0.52697
P14079	Q09MP3	1.014
P14079	Q8IV48	0.20094
P14079	P0CW19	0.54513
P14079	Q14CS0	0.48019
P14079	Q8N831	0.55345
P14079	P80297	0.77432
P14079	Q13772	0.49546
P14079	P51504	0.65804
P14079	Q96FV2	1.1957
P14079	Q96G79	0.72618
P14079	A6NC05	0.66693
P14079	Q9H0E3	1.6465
P14079	O00422	1.0835
P14079	Q8NHW6	1.031
P14079	P48454	0.33441
P14079	Q8N9P6	1.1715
P14079	O75161	1.6782
P14079	Q9UHZ2	1.1715
P14079	Q0VF49	1.1715
P14079	Q13362	0.25213
P14079	Q9NVR5	1.3841
P14079	Q8N9Z0	0.9663
P14079	A5PLL7	1.1359
P14079	Q3C1V9	1.0392
P14079	P78346	1.0339
P14079	O60244	0.67177
P14079	O43196	1.0274
P14079	Q8NG48	0.32738
P14079	P11388	0.33994
P14079	Q9H446	0.56221
P14079	Q8NA72	0.59252
P14079	Q3LI67	1.1715
P14079	P48775	0.53275
P14079	O15069	0.45685
P14079	Q8N895	0.68858
P14079	Q12894	0.46041
P14079	Q49B96	0.79923
P14079	Q9Y2J2	0.70726
P14079	P0CJ70	1.3031
P14079	P35579	0.52374
P14079	Q96MU7	1.1366
P14079	Q86V20	1.3863
P14079	Q6ZUT6	1.1354
P14079	Q99459	0.89078
P14079	Q9BXS1	0.57049
P14079	Q9Y333	0.70283
P14079	P41218	0.54682
P14079	Q8N573	0.12814
P14079	Q96IY1	0.9337
P14079	Q9Y285	1.3002
P14079	Q7Z7F7	1.1265
P14079	P13164	0.46117
P14079	Q9NPI1	0.77888
P14079	O95402	1.1194
P14079	Q68DK7	1.5005
P14079	O75223	0.57322
P14079	Q3LI70	1.1715
P14079	Q9BTW9	0.47394
P14079	Q9Y5P2	0.51712
P14079	O60260	0.29068
P14079	Q8NEY3	1.1006
P14079	Q9H857	0.92807
P14079	Q8N9Q2	0.5594
P14079	P17098	1.018
P14079	Q15014	1.1185
P14079	Q9H061	0.81101
P14079	Q9UGV2	0.38162
P14079	P0C879	1.1715
P14079	Q9H7T9	1.1553
P14079	Q01105	0.769
P14079	Q8N159	0.55845
P14079	P48643	0.17096
P14079	Q8IX12	1.0226

P14079	O43447	0.58067
P14079	P08151	0.33613
P14079	Q8IVE0	0.34895
P14079	Q15293	0.47116
P14079	Q6PIY5	0.22596
P14079	Q8N8B7	0.11388
P14079	Q14166	0.42822
P14079	Q9Y5K8	0.32936
P14079	P20042	0.61176
P14079	O94830	0.041402
P14079	P15036	0.8325
P14079	Q03188	0.96724
P14079	Q5JVS0	0.96656
P14079	Q8TC94	0.89007
P14079	O60397	0.78471
P14079	P0CJ74	1.3031
P14079	Q9GZM3	0.23838
P14079	O95166	0.41166
P14079	P59036	1.1715
P14079	P30042	0.72377
P14079	Q5SNV9	0.87733
P14079	Q8N7U7	0.88134
P14079	P0C7V0	1.1715
P14079	P62333	0.42583
P14079	P59052	1.1149
P14079	Q8NA69	0.81719
P14079	Q9BQ95	1.1691
P14079	P13056	0.7453
P14079	P53804	0.40464
P14079	Q9NXE8	1.356
P14079	O43677	1.0705
P14079	A1E959	0.8216
P14079	Q8TEA8	1.4439
P14079	P0C6T2	1.0481
P14079	Q96GG9	0.60775
P14079	O76087	0.99818
P14079	O15164	0.23504
P14079	Q9BQN1	1.4166
P14079	Q8NEM7	0.53886
P14079	Q569G3	1.3222
P14079	Q9Y2X0	0.63179
P14079	Q6ZVX7	1.0333
P14079	Q8IXQ6	0.81644
P14079	Q7L804	0.72066
P14079	Q9BW27	0.94376
P14079	O43513	1.2063
P14079	Q9C010	1.3204
P14079	P13805	0.1876
P14079	O95816	0.56144
P14079	Q8WYK2	0.37431
P14079	P07498	1.4033
P14079	Q9BRX2	0.30835
P14079	P35237	0.43296
P14079	Q96HZ7	1.1715
P14079	P48668	0.96294
P14079	Q96JI7	1.2962
P14079	Q8N4H5	1.0895
P14079	Q9H374	0.73811
P14079	Q6ZS52	1.1715
P14079	P0C864	0.32955
P14079	Q86Y56	0.080836
P14079	P0CJ89	0.25626
P14079	Q9UKT5	0.27782
P14079	Q96M27	0.15484
P14079	O75123	0.52714
P14079	Q9NXP7	0.45857
P14079	Q14004	0.34877
P14079	O15315	0.1968
P14079	Q14789	0.69539
P14079	Q8IWL8	1.7013
P14079	Q8N443	1.087
P14079	Q8N3T6	0.97751
P14079	O14746	1.0055
P14079	Q13416	0.87997
P14079	Q3MIW9	1.2698
P14079	Q3SY00	1.3865

P14079	O14773	0.042998
P14079	Q5XKL5	0.27853
P14079	Q14C87	1.3386
P14079	P02042	0.65657
P14079	Q9UGL9	1.1715
P14079	Q92979	0.45711
P14079	P0C7U1	0.12476
P14079	Q30KQ8	0.91101
P14079	Q6P587	0.40659
P14079	Q6ZVE7	0.95713
P14079	Q9HAZ2	0.48833
P14079	O14867	0.40037
P14079	O95425	0.17769
P14079	Q9H0P0	0.62321
P14079	Q14746	0.63652
P14079	P0CJ88	0.25626
P14079	Q8N9K7	1.1715
P14079	P0CL85	0.46781
P14079	O75467	0.52324
P14079	Q68DY9	0.63518
P14079	Q9NWS1	1.0741
P14079	B1ANH7	0.65518
P14079	Q96NA2	1.6417
P14079	Q9BWK5	1.566
P14079	P25800	0.3426
P14079	A8MZF0	1.3222
P14079	Q8WTP9	1.7294
P14079	Q9GZT9	0.24991
P14079	Q5VU69	1.152
P14079	Q5T2E6	1.3019
P14079	Q15007	1.0081
P14079	O00442	0.74447
P14079	Q6PGP7	0.85169
P14079	P23381	0.57992
P14079	P20585	0.22468
P14079	P55199	1.1282
P14079	Q9UL40	0.8525
P14079	Q5SVJ3	0.97165
P14079	Q9BRT9	0.79212
P14079	Q8NCY6	0.23265
P14079	Q9NQ50	1.3792
P14079	Q96FN9	1.0146
P14079	O95628	0.32427
P14079	P61581	1.7166
P14079	Q9Y5U4	0.38197
P14079	Q8NAJ2	0.36546
P14079	Q86XD5	0.80645
P14079	Q68DX3	0.85142
P14079	P26639	0.84387
P14079	Q8NEB7	0.9829
P14079	Q658T7	0.3152
P14079	P0C7M4	0.24936
P14079	P10243	0.70969
P14079	Q9H7R0	0.94651
P14079	Q8WWX9	0.53016
P14079	Q56A73	0.56501
P14079	Q9H094	0.22747
P14079	P09565	0.94741
P14079	Q9UKV8	0.79383
P14079	Q53S99	0.38401
P14079	Q53FE4	0.6778
P14079	A0AUZ9	1.0404
P14079	Q99743	0.27677
P14079	P59282	0.77614
P14079	Q96HA1	1.6792
P14079	O95873	1.3222
P14079	Q96F85	0.0057309
P14079	Q06730	0.44092
P14079	Q9H295	0.49105
P14079	Q9BQ15	1.2691
P14079	Q9BZL1	0.7837
P14079	Q8N0W5	1.0178
P14079	P26373	1.2212
P14079	Q9NQG1	1.1823
P14079	Q8N4P2	0.63873
P14079	Q53GI3	0.56951

P14079	O75346	0.9499
P14079	O95561	1.2016
P14079	P37802	0.48303
P14079	P60002	1.5351
P14079	Q9Y3E5	0.8605
P14079	P57678	0.91795
P14079	Q86VN1	0.56277
P14079	Q15513	1.1715
P14079	Q6P3S1	0.12095
P14079	Q9UNH7	0.3908
P14079	B1AL88	1.2485
P14079	P16383	1.6908
P14079	P62508	0.58284
P14079	Q6T4R5	0.78774
P14079	A8MZA4	0.89121
P14079	Q5TZA2	0.96552
P14079	Q8WZ33	0.81071
P14079	Q92562	0.44774
P14079	Q9ULD9	1.781
P14079	Q6NSI8	1.0503
P14079	P33076	0.59991
P14079	Q3ZCQ2	1.1715
P14079	Q9GZU0	1.1689
P14079	P09327	0.088626
P14079	Q8IXS6	0.57042
P14079	Q8IYU4	0.24849
P14079	O95415	1.0613
P14079	P86479	1.1715
P14079	O43156	1.2265
P14079	Q9HBT7	0.68829
P14079	Q8N4S0	1.3222
P14079	P60409	1.3698
P14079	P49753	0.23623
P14079	P05114	0.72086
P14079	Q9UL33	0.74375
P14079	P22531	1.1715
P14079	Q8TAT5	0.61766
P14079	Q96HA9	0.99552
P14079	Q6IEE8	1.0684
P14079	Q93083	0.77468
P14079	P57076	0.85644
P14079	A9UHW6	1.1067
P14079	P0CL82	0.99818
P14079	P15502	0.84493
P14079	Q4G112	0.28169
P14079	B3EWF7	1.1715
P14079	Q9Y3B8	0.48217
P14079	Q6ZUJ4	1.2201
P14079	Q99627	0.57354
P14079	O43759	0.97807
P14079	Q86UY5	0.59525
P14079	Q96I25	0.21549
P14079	P09874	0.64919
P14079	Q96JB3	0.21516
P14079	Q8N1B3	0.80199
P14079	Q8WVV9	0.24192
P14079	Q9HAT8	0.97187
P14079	O15225	1.1715
P14079	P78543	0.82071
P14079	Q7Z5L0	0.94645
P14079	Q5HYI7	1.6457
P14079	Q969M3	0.55727
P14079	C9JVW0	1.1715
P14079	P49257	0.34868
P14079	Q5T7N2	0.46109
P14079	Q96DY7	0.94061
P14079	O43181	0.18274
P14079	A4D161	0.65781
P14079	P05813	0.67475
P14079	Q15287	0.8967
P14079	Q9Y4A5	0.69381
P14079	Q9NPH2	0.67527
P14079	Q8IWE2	1.4569
P14079	P16278	0.084402
P14079	Q9NPG2	0.48318
P14079	Q12982	0.30725

P14079	Q9BQK8	0.26903
P14079	A8MYZ0	1.1773
P14079	Q5MCW4	0.53925
P14079	P35900	1.2514
P14079	Q9NQ48	0.48044
P14079	P27216	0.65206
P14079	Q5TIA1	0.36808
P14079	Q4KMZ1	0.95475
P14079	Q9H0S4	0.13736
P14079	Q8N6Q3	0.79631
P14079	O14841	1.0995
P14079	Q5T0L3	1.6329
P14079	Q96RJ6	0.43586
P14079	Q96K30	1.1041
P14079	A8MTY0	1.0073
P14079	P52742	0.73618
P14079	Q9NTX9	0.77499
P14079	Q86XZ4	0.96374
P14079	Q96QH2	0.35123
P14079	Q16890	0.63143
P14079	Q96T23	0.60807
P14079	Q8TD23	1.2643
P14079	Q6PGQ7	1.7845
P14079	Q9P0P8	0.61851
P14079	P61923	0.54358
P14079	Q8TDR4	0.63132
P14079	Q8NHY5	1.3476
P14079	P48556	0.83362
P14079	Q6IQ49	0.58942
P14079	Q8IXH6	1.2325
P14079	Q5HYK9	0.74415
P14079	Q9BVC5	1.1409
P14079	Q6XYB7	0.28169
P14079	Q9Y6G5	0.85111
P14079	Q4U2R6	1.3251
P14079	A1A4V9	0.55177
P14079	Q5T5N4	0.53769
P14079	Q8IXS2	0.47209
P14079	P42357	0.1472
P14079	P57088	0.92113
P14079	Q9BQ90	1.1205
P14079	A6NDN3	0.71479
P14079	E9PJI5	1.3504
P14079	Q9BQS6	0.94018
P14079	P0C874	0.94399
P14079	P35326	1.2047
P14079	O75618	1.143
P14079	O95447	0.72979
P14079	Q96IR2	0.80079
P14079	P55327	0.44376
P14079	Q9UPY8	1.1846
P14079	O76075	0.35669
P14079	Q9BTD3	1.2485
P14079	Q9P2X8	0.93707
P14079	Q08945	1.0986
P14079	O15027	1.0124
P14079	Q8NCA5	1.1911
P14079	Q96LW7	1.5173
P14079	Q9BRQ6	1.881
P14079	Q13506	0.53082
P14079	P51965	0.49344
P14079	P61970	0.61239
P14079	Q9NQ35	1.3448
P14079	Q13442	1.0786
P14079	O15162	0.53829
P14079	O95169	0.74645
P14079	P41227	0.37402
P14079	P09132	0.50763
P14079	Q6XZF7	0.44458
P14079	P69891	0.65657
P14079	P10155	1.2695
P14079	Q96C01	0.55719
P14079	Q5TA31	0.28231
P14079	Q05BQ5	0.30577
P14079	P57723	0.13109
P14079	P27469	0.84264

P14079	Q9UPP2	0.35301
P14079	Q96RS0	0.50917
P14079	Q9H8S9	0.62679
P14079	Q9NVQ4	0.15625
P14079	O75151	0.47904
P14079	Q96HE9	0.50707
P14079	Q8WTR7	0.8622
P14079	Q8NAN2	0.91801
P14079	P31948	1.0892
P14079	Q99807	0.43248
P14079	Q53H80	0.80325
P14079	Q86SZ2	1.2865
P14079	Q9UL36	0.5268
P14079	Q9NV88	0.75135
P14079	Q96CF2	0.41173
P14079	Q9H579	0.20109
P14079	A8MV81	1.0498
P14079	Q9Y6Q5	0.59076
P14079	Q8WXC3	0.76159
P14079	Q9UD71	1.3139
P14079	P60842	0.15808
P14079	Q9H8W4	0.37877
P14079	Q86UY6	0.47127
P14079	Q86UT8	1.8063
P14079	Q96EY5	1.1716
P14079	P12755	0.31378
P14079	Q9Y3B2	0.79466
P14079	Q9UPP5	1.3341
P14079	P55055	0.35388
P14079	Q8WXW3	1.2364
P14079	A6NEJ1	0.31443
P14079	Q5MJ70	1.092
P14079	Q8TBK6	1.2549
P14079	Q8N8L2	0.56276
P14079	P21953	0.20869
P14079	O14602	0.82748
P14079	P0CG31	0.57343
P14079	Q9GZL8	1.2337
P14079	Q96EV2	0.9364
P14079	Q7Z745	0.9485
P14079	Q15036	0.30662
P14079	Q6UB98	0.42364
P14079	Q9NR22	0.39456
P14079	Q9BRP7	0.56797
P14079	P61966	0.49388
P14079	P23396	0.97849
P14079	Q86V97	0.36435
P14079	Q5JUX0	0.46837
P14079	Q9Y4E1	1.2
P14079	Q7L590	1.2535
P14079	O60925	0.57051
P14079	Q5T1R4	0.35952
P14079	P0C5K6	1.1715
P14079	Q9BUK0	1.6151
P14079	Q5VZQ5	1.4808
P14079	H3BMG3	0.92587
P14079	P13196	0.10985
P14079	Q8NEH6	1.4228
P14079	O14613	0.3825
P14079	Q5D1E8	1.3043
P14079	Q9ULG1	0.31063
P14079	Q6ZR03	0.92183
P14079	O00291	0.66182
P14079	O60293	0.22157
P14079	Q96GW9	1.1889
P14079	O95544	0.91121
P14079	O95416	0.41359
P14079	Q08495	0.35996
P14079	Q6ULP2	1.2139
P14079	Q9P2J5	0.77353
P14079	Q3LHN0	1.5617
P14079	O60294	0.4659
P14079	Q05084	0.29232
P14079	P63261	0.99952
P14079	Q15038	2.1029
P14079	Q8N5C8	0.92672

P14079	Q9UIF7	0.49377
P14079	Q8WUN7	0.42364
P14079	Q14242	1.2018
P14079	Q92994	0.47821
P14079	Q8IZD4	0.90557
P14079	Q7Z7K0	0.68154
P14079	Q15056	0.4775
P14079	Q5EE01	1.2058
P14079	Q8N9T2	1.1715
P14079	Q86WV1	0.85187
P14079	Q8N4B5	1.3431
P14079	Q9BTX9	1.4993
P14079	Q9Y614	0.89007
P14079	Q86V25	0.68606
P14079	Q9Y664	0.72704
P14079	Q9H0I3	0.76395
P14079	Q96B67	0.51322
P14079	Q8N307	1.0124
P14079	Q7Z3J2	0.78469
P14079	P11142	0.29537
P14079	Q8N7P3	0.63456
P14079	Q3SY84	0.98252
P14079	Q6PRD7	1.1715
P14079	Q86WP2	1.2469
P14079	Q96KR4	0.66234
P14079	P54922	1.228
P14079	P56134	0.72976
P14079	P10412	0.38825
P14079	A8MUQ0	1.5165
P14079	Q9P2M1	0.72622
P14079	Q8WY41	0.73767
P14079	Q6ZVM7	0.05613
P14079	A8K8V0	0.75186
P14079	Q10587	0.22589
P14079	C9JDP6	0.63456
P14079	Q9HAJ7	1.9201
P14079	Q9Y5Q3	0.67148
P14079	Q9BXS5	1.0724
P14079	Q569K6	0.37266
P14079	P0C880	1.1715
P14079	P55039	0.86784
P14079	O60381	0.18393
P14079	Q6NVV1	0.64056
P14079	Q9BYK8	0.44847
P14079	Q9BTV5	0.68921
P14079	P57077	0.94124
P14079	Q9UDV6	0.72847
P14079	E2RYF6	1.1715
P14079	Q9NWB6	1.3222
P14079	Q58A44	1.1715
P14079	P0CB43	0.83392
P14079	Q8WXB4	0.51635
P14079	Q8N6C7	0.41577
P14079	Q9NPB8	0.10397
P14079	P63279	0.69239
P14079	Q16873	0.45461
P14079	Q15561	0.81859
P14079	Q30KQ9	0.87535
P14079	Q8WWF1	0.88143
P14079	P17677	0.26824
P14079	Q9Y2Y4	0.52332
P14079	Q9BZK7	0.93873
P14079	Q5TGP6	0.63072
P14079	Q86VE9	0.98083
P14079	Q6UW32	0.85692
P14079	P56556	0.71399
P14079	E9PKD4	0.12445
P14079	Q71F56	0.40818
P14079	P60014	1.4903
P14079	P41238	0.14213
P14079	Q8N365	1.3222
P14079	O75146	0.30687
P14079	Q15573	1.2092
P14079	Q8IZX4	0.24398
P14079	Q8WV41	0.41619
P14079	Q12815	1.0178

P14079	P14373	0.25587
P14079	P07954	0.2358
P14079	Q9UKG1	0.58027
P14079	O75934	0.69605
P14079	Q8N655	1.1715
P14079	Q15532	1.2692
P14079	Q9H0A6	0.59058
P14079	Q8N123	1.4748
P14079	Q6UVJ0	1.1557
P14079	Q8N9W7	0.90704
P14079	Q68DL7	1.1715
P14079	Q92686	0.61384
P14079	P17152	1.1879
P14079	O43364	0.15306
P14079	Q9NV31	1.0402
P14079	Q8TB68	1.1717
P14079	Q96LW1	0.9897
P14079	Q9HCJ5	0.34113
P14079	H3BRN8	0.40745
P14079	Q9UC06	0.57224
P14079	Q5JQC9	0.55956
P14079	Q9BVP2	0.30172
P14079	P60468	0.68789
P14079	Q9H8V8	1.1715
P14079	P35869	0.31034
P14079	Q8IZ16	0.91367
P14079	Q8IVK1	1.1715
P14079	Q8N999	1.1486
P14079	P57086	0.84274
P14079	Q96T37	0.47325
P14079	Q8WV24	1.0483
P14079	Q969X6	0.99815
P14079	Q8N8E1	1.3146
P14079	Q3ZAQ7	0.95523
P14079	Q8TEQ6	0.76521
P14079	Q02078	0.79005
P14079	Q8IXB3	1.1369
P14079	Q8NFF5	1.0819
P14079	Q3KQU3	1.1169
P14079	Q8NEV8	0.33125
P14079	Q9Y2G7	1.0764
P14079	Q9Y3D7	0.46528
P14079	Q9H7J1	0.95313
P14079	Q5T124	0.37346
P14079	O95125	0.53302
P14079	P0C7P3	1.2416
P14079	Q99814	0.30944
P14079	P60413	1.3698
P14079	Q8WU39	1.0429
P14079	Q7Z340	0.55626
P14079	A1A4T8	0.5247
P14079	A6NL28	0.2068
P14079	Q9UK13	0.98685
P14079	Q9Y375	0.74179
P14079	O75817	1.3924
P14079	Q9Y4U1	0.92442
P14079	Q9NYV6	0.6093
P14079	Q9UIS9	0.35612
P14079	Q8TBY8	0.96371
P14079	A6NM66	0.86222
P14079	O14828	1.0112
P14079	Q14241	0.75851
P14079	Q5VW32	0.31881
P14079	Q9BXT5	1.1014
P14079	Q8N5D0	0.68102
P14079	Q96P71	0.5988
P14079	Q8NDQ6	0.77193
P14079	Q96LL4	1.3222
P14079	Q96DY2	0.53316
P14079	P86434	0.77823
P14079	Q96B01	1.4144
P14079	P0C5J1	0.30613
P14079	Q3KP22	0.69231
P14079	P0CG48	0.66704
P14079	Q06413	0.30045
P14079	Q1RN00	1.1715

P14079	P19429	0.16965
P14079	P40855	0.90544
P14079	O95793	0.50535
P14079	Q0VDE8	1.4165
P14079	Q9BWX5	0.40622
P14079	Q86WG3	0.41677
P14079	Q9BRT2	0.46164
P14079	Q9H799	1.2549
P14079	Q13609	0.41572
P14079	Q8NDP4	0.69818
P14079	Q6STE5	0.66394
P14079	P08779	1.1852
P14079	Q9C073	1.5622
P14079	Q9NSI6	0.66017
P14079	Q9H3H3	0.75427
P14079	Q9UNA4	0.49052
P14079	Q8N196	0.51722
P14079	P49069	0.63552
P14079	Q6ZT83	0.71401
P14079	Q8TBZ6	0.65786
P14079	P02728	1.1715
P14079	Q6PP77	1.4324
P14079	Q9NXF7	0.71704
P14079	Q86SG4	0.65928
P14079	Q99502	0.12944
P14079	Q8NEG2	1.0466
P14079	Q9C0D0	0.71812
P14079	Q8NB91	0.73511
P14079	O60826	1.3091
P14079	Q00610	0.88852
P14079	Q8IZ21	0.6781
P14079	Q5QGS0	0.43573
P14079	Q6PH81	0.98617
P14079	P63172	1.0265
P14079	Q96N68	1.1715
P14079	Q5TYM5	0.86671
P14079	P54257	0.07946
P14079	Q9NPE3	1.1297
P14079	O60811	0.0080986
P14079	P13073	1.0584
P14079	Q76L83	0.92146
P14079	Q96KP4	0.73984
P14079	Q9NYY8	0.60942
P14079	Q53RY4	1.2485
P14079	Q9NUQ7	0.55033
P14079	Q8IUE1	0.28169
P14079	O75419	1.171
P14079	O15370	0.42902
P14079	Q9UI43	1.305
P14079	Q8N693	0.33196
P14079	P40429	0.9726
P14079	O43482	0.78379
P14079	Q9BU23	1.2107
P14079	Q8N8G2	1.0419
P14079	Q9BYX4	0.41472
P14079	O75400	0.93033
P14079	Q9NWB7	0.96516
P14079	Q14738	0.89663
P14079	O95876	0.56473
P14079	O75879	0.88097
P14079	Q5T1J5	1.571
P14079	Q5VT28	1.2112
P14079	Q5EBM0	1.2111
P14079	Q15596	0.77366
P14079	Q96LJ8	1.245
P14079	Q9BYR8	1.5617
P14079	Q68DK2	1.0429
P14079	Q6ICB0	1.5046
P14079	Q9BTL4	1.243
P14079	Q7L3T8	1.1106
P14079	Q15047	0.38885
P14079	Q8NEE0	1.1715
P14079	P0C7V5	0.8885
P14079	Q8N9R0	1.1715
P14079	Q86V15	0.84563
P14079	P10072	0.59229

P14079	Q08AD1	0.84231
P14079	A6NKL6	1.2485
P14079	Q8N141	1.0323
P14079	Q8TEL6	0.74472
P14079	Q16222	0.1375
P14079	Q8IZU1	1.0534
P14079	O14609	1.5015
P14079	P28715	0.31259
P14079	O75363	0.92052
P14079	Q9ULC4	0.40191
P14079	Q05BU3	1.1715
P14079	Q92600	1.3891
P14079	P35908	0.88485
P14079	P52630	0.7232
P14079	P49593	0.26988
P14079	Q9UL18	1.0951
P14079	Q9BTE6	1.2376
P14079	Q494R0	1.1715
P14079	Q9NRX4	0.48645
P14079	O00303	0.23897
P14079	Q96MF7	0.53004
P14079	O43505	1.0174
P14079	Q8NHR7	0.4649
P14079	O94906	0.28087
P14079	Q96K62	0.57002
P14079	Q9C005	0.53986
P14079	Q9UJW7	0.54706
P14079	Q8NHX4	1.2387
P14079	Q86XR2	0.35469
P14079	Q7Z794	1.0155
P14079	P30307	0.95271
P14079	P59089	0.45231
P14079	Q6ZTQ3	0.48021
P14079	Q14566	0.23491
P14079	Q8N4B4	0.37775
P14079	A6NMA1	0.58408
P14079	Q96H86	0.50353
P14079	Q8N5I9	1.3222
P14079	Q5XG85	1.1715
P14079	Q7Z7N9	1.2446
P14079	Q8N1A6	0.19994
P14079	O43745	0.90835
P14079	Q99996	0.87981
P14079	O94888	0.5471
P14079	A8MUL3	1.1715
P14079	P21506	0.68126
P14079	Q9NT62	0.41298
P14079	P0CZ20	0.86237
P14079	Q96EZ4	1.1715
P14079	Q9H939	0.25016
P14079	Q9NQV6	0.1949
P14079	Q14761	1.5544
P14079	Q96AB3	1.1702
P14079	Q9BZE2	1.5894
P14079	Q107X0	1.1597
P14079	Q13106	0.58453
P14079	Q7Z7K2	0.49873
P14079	Q9BW92	0.85063
P14079	Q5K131	0.77137
P14079	O43716	0.77503
P14079	Q9BUH8	0.66771
P14079	Q8N490	0.70533
P14079	Q9UJY4	0.87676
P14079	A2RUQ5	0.44491
P14079	Q96CB8	0.66734
P14079	O14771	1.2088
P14079	Q5TZJ5	1.6988
P14079	Q9BYQ4	1.3698
P14079	Q9Y6Q3	0.51814
P14079	Q9H9G7	0.95157
P14079	O75072	0.49794
P14079	Q14442	0.80231
P14079	Q86VI1	0.48787
P14079	P20962	1.1914
P14079	Q9Y2X3	0.69706
P14079	Q8IY31	0.87967

P14079	Q6PCB5	1.1238
P14079	Q8IV50	0.2678
P14079	Q9H0R6	0.8855
P14079	Q9UJC3	0.18135
P14079	Q9NYV7	0.50252
P14079	Q92681	0.83528
P14079	Q9GZV5	1.0387
P14079	Q9NWT8	0.91301
P14079	Q9BQE4	1.1093
P14079	Q9NUX5	0.19586
P14079	Q9NS23	0.47237
P14079	Q9BT81	0.32722
P14079	Q96H20	0.89403
P14079	Q06546	1.239
P14079	Q9HC36	0.09247
P14079	Q9H0P7	0.59843
P14079	P14859	1.1592
P14079	P83436	0.74896
P14079	Q96SY0	0.28916
P14079	C9JBD0	1.0571
P14079	P04279	0.56503
P14079	Q96FC7	0.19405
P14079	O15305	0.29029
P14079	Q5VWQ0	1.9447
P14079	P29728	0.341
P14079	O75663	0.6545
P14079	P53041	0.58079
P14079	Q8WZ82	0.16561
P14079	Q9H7C4	0.66835
P14079	P0CG34	1.1711
P14079	P02795	0.95509
P14079	Q49AG3	1.4353
P14079	Q9UNK9	0.42612
P14079	Q5T292	1.223
P14079	Q9Y6J9	0.78137
P14079	Q6IQ23	0.61352
P14079	Q9NPA3	1.7237
P14079	Q8TF50	0.47167
P14079	Q8WV99	0.84628
P14079	Q96MU6	0.55888
P14079	Q9UKT4	1.1181
P14079	Q86U38	0.40311
P14079	Q8N6N6	0.41835
P14079	Q9UID3	0.12946
P14079	Q1HG44	1.5402
P14079	O43187	0.3158
P14079	Q16851	0.21408
P14079	A6NJ64	0.86845
P14079	Q9NRR3	0.72073
P14079	Q9Y2W2	1.3966
P14079	Q96QC0	1.6153
P14079	Q6UXB0	0.82486
P14079	Q9P2G4	1.3222
P14079	Q96NL6	0.54529
P14079	Q9BQ66	1.3698
P14079	Q96AT1	1.3222
P14079	Q8TEP8	1.2206
P14079	A6PW82	1.236
P14079	Q5SWA1	1.0244
P14079	P10073	0.87381
P14079	Q86V42	1.1526
P14079	Q96DX7	0.28018
P14079	P31150	0.12563
P14079	Q96E11	1.3811
P14079	Q14236	0.83653
P14079	Q7Z7L9	0.59666
P14079	O75821	0.5665
P14079	Q9UHT9	1.1715
P14079	O60508	0.38573
P14079	O43768	0.26752
P14079	Q16526	0.37901
P14079	Q8N163	0.7911
P14079	P47224	0.94181
P14079	P35637	0.51105
P14079	Q96PT4	0.2245
P14079	Q8IXY8	0.1778

P14079	Q9UL42	1.0327
P14079	Q5VZF2	0.24368
P14079	A8K0S8	0.24936
P14079	Q969J3	0.14536
P14079	Q9HC44	2.2309
P14079	B1AK76	1.3892
P14079	Q6NT04	0.80396
P14079	Q08752	0.47353
P14079	Q8WXS3	1.9264
P14079	Q9Y2Q5	0.50149
P14079	Q969H0	0.69853
P14079	Q9H8M2	0.36383
P14079	Q7L592	0.88813
P14079	P11532	0.63173
P14079	Q01518	0.38158
P14079	P62324	0.73396
P14079	Q8TC90	1.4784
P14079	Q6P9B6	0.56221
P14079	O14990	1.207
P14079	Q8N535	1.1512
P14079	P53779	0.26368
P14079	Q14693	0.26309
P14079	O60828	0.741
P14079	Q96A04	1.4641
P14079	Q9UBU8	0.67553
P14079	Q9ULV3	0.5185
P14079	P28749	1.4057
P14079	Q9H4K7	1.0731
P14079	Q86X59	0.23765
P14079	Q86XA9	0.742
P14079	Q9Y316	1.0269
P14079	Q5T035	1.5169
P14079	Q9NPI0	0.94686
P14079	P50452	0.66779
P14079	P61201	0.62622
P14079	P78337	0.25261
P14079	D6RGH6	1.1858
P14079	Q6ZVD7	0.61606
P14079	O43422	0.87128
P14079	Q96LA8	0.69561
P14079	P41219	0.88585
P14079	Q5VSG8	0.96159
P14079	Q4LDR2	1.1353
P14079	Q9BYG5	0.79225
P14079	Q5TH74	1.2507
P14079	P13349	0.30705
P14079	Q01534	0.40864
P14079	Q7Z460	0.55256
P14079	Q9UNY5	0.93026
P14079	Q16649	0.22274
P14079	Q96BN6	0.95486
P14079	Q14653	0.73832
P14079	Q9NZM5	0.93273
P14079	Q8NEU8	0.58432
P14079	A1L3X0	0.77014
P14079	P61570	1.0384
P14079	A8MS94	0.93955
P14079	A2A368	0.22377
P14079	Q99633	0.11627
P14079	Q02383	0.96444
P14079	A6NK53	0.6845
P14079	Q9UL59	0.54351
P14079	Q96NY9	0.99331
P14079	Q8N587	0.92278
P14079	P51808	0.37527
P14079	Q9H321	1.445
P14079	P06730	0.74779
P14079	Q8WY36	0.38002
P14079	Q8N1D5	0.84703
P14079	Q8IX90	0.80052
P14079	Q6PIJ6	1.5141
P14079	P59990	1.3698
P14079	Q8IWT0	0.48847
P14079	A8K554	0.37211
P14079	Q6P1R3	0.90321
P14079	Q6ZNA1	0.76643

P14079	Q8IYM2	0.67206
P14079	Q6QNY0	1.0943
P14079	Q96HB5	0.93563
P14079	Q86WV5	0.89225
P14079	Q96A33	0.67268
P14079	Q8TAI1	1.2677
P14079	Q96KW9	1.545
P14079	Q7Z4H9	1.0038
P14079	Q5VSY0	0.53731
P14079	Q13765	1.3112
P14079	Q96JN2	0.90922
P14079	Q92917	0.77713
P14079	Q9UHV2	2.2067
P14079	Q9Y2D5	0.57695
P14079	O15553	0.26879
P14079	P19387	0.7484
P14079	O75691	0.93512
P14079	Q5H9L4	0.48573
P14079	Q9UGK8	0.74716
P14079	Q6ZVN8	0.72783
P14079	Q9Y3A2	0.91905
P14079	Q96BT3	0.7047
P14079	Q8N9F8	0.6406
P14079	P02100	0.65657
P14079	Q5TEV5	1.1002
P14079	Q99704	0.30536
P14079	O75437	0.96899
P14079	O60739	0.81433
P14079	A8MQ03	1.0699
P14079	O95139	0.31524
P14079	A6NIH7	0.77774
P14079	P40222	1.1093
P14079	Q6NUJ2	1.2485
P14079	P52747	0.97966
P14079	Q24JP5	1.0649
P14079	Q9NZJ4	0.19373
P14079	Q6EEV4	1.1715
P14079	Q16548	0.82894
P14079	Q9UI25	1.1715
P14079	F8WBI6	0.082129
P14079	P25705	0.21212
P14079	Q9C035	0.62353
P14079	Q8N9W5	0.76332
P14079	Q5D862	0.017627
P14079	Q8NCQ3	1.1715
P14079	P61421	0.25563
P14079	A5PLL1	0.28036
P14079	Q96FA7	1.7459
P14079	O75928	0.73206
P14079	P09914	0.62351
P14079	Q9UHQ1	1.2216
P14079	Q9Y5B0	0.76158
P14079	Q12887	0.80021
P14079	Q52LR7	0.40154
P14079	P35269	1.3001
P14079	P57721	0.6273
P14079	Q5T751	1.1715
P14079	Q15776	0.54371
P14079	Q56P03	1.1865
P14079	Q14186	1.1612
P14079	Q6ZNG9	0.11264
P14079	Q13118	0.4961
P14079	A3QJZ6	0.097036
P14079	P20809	0.87321
P14079	P61583	1.7501
P14079	P11171	0.8731
P14079	Q9H009	0.53185
P14079	Q9Y255	0.74486
P14079	Q96IU2	1.9492
P14079	Q9Y519	0.80647
P14079	Q96GY0	1.3053
P14079	Q9ULQ0	1.6049
P14079	Q9UHA3	0.38116
P14079	Q9UI10	0.4593
P14079	O75528	0.68178
P14079	P54136	1.114

P14079	Q86UQ0	0.53928
P14079	Q2M1V0	0.28169
P14079	Q6UX01	1.0651
P14079	Q9NRG7	0.80677
P14079	Q7L273	0.18591
P14079	Q8TF05	0.31551
P14079	Q6ZMV9	0.31989
P14079	P43366	0.09333
P14079	Q9GZQ3	1.5397
P14079	P49336	0.27274
P14079	Q8N112	0.95946
P14079	Q8N755	0.91861
P14079	Q6ZNG0	0.5775
P14079	Q9H869	1.8819
P14079	A8MTW9	1.1715
P14079	Q9BZA5	1.3646
P14079	Q3LI60	1.1715
P14079	Q9Y4P8	1.0017
P14079	Q9ULC6	1.4646
P14079	Q96DB2	0.14369
P14079	O15063	1.1789
P14079	Q13148	0.36558
P14079	Q30KR1	0.91101
P14079	Q8IU53	1.3031
P14079	P51946	0.75089
P14079	Q9UDY2	1.2932
P14079	P08048	0.1949
P14079	Q9H2U6	1.2197
P14079	Q9NRG4	0.62518
P14079	P17019	1.0245
P14079	Q96MC2	0.60242
P14079	E9PQR5	0.30744
P14079	A6NL46	1.3045
P14079	P11844	1.342
P14079	Q9P0R6	0.65799
P14079	A6NI79	1.5826
P14079	O95999	0.3544
P14079	Q9P055	1.0304
P14079	Q9Y305	1.1225
P14079	Q8IWV8	0.56167
P14079	Q7L4P6	0.98184
P14079	Q6ZMY9	0.99738
P14079	Q9UKF7	0.24922
P14079	Q8TDZ2	0.37623
P14079	Q15853	0.52962
P14079	Q2NL82	1.0476
P14079	Q6ZN84	0.83414
P14079	A3KN83	1.4154
P14079	P14621	0.59147
P14079	O00321	1.0851
P14079	Q5JVX7	1.3448
P14079	A8MXU0	0.79509
P14079	Q96P70	1.1722
P14079	Q8NFM7	0.94035
P14079	Q9Y3A4	0.83861
P14079	Q8NBR6	2.2522
P14079	P0C221	0.18221
P14079	Q14532	0.70212
P14079	O15297	0.60224
P14079	Q8NEQ6	0.71391
P14079	Q9H2K0	0.87449
P14079	P18621	0.8975
P14079	Q8NDY3	0.55665
P14079	Q96ER9	0.67368
P14079	Q9NVU7	1.0675
P14079	Q9BVN2	0.76693
P14079	Q86WI0	1.089
P14079	O60481	0.40625
P14079	A8MTB1	1.1715
P14079	P12074	0.93065
P14079	Q9H5V9	1.1738
P14079	Q8NAX2	1.3222
P14079	Q13415	0.35264
P14079	Q7Z2W4	0.60073
P14079	Q9HBM1	0.8164
P14079	Q9UHL3	0.78875

P14079	O95487	0.98564
P14079	Q9UM82	1.6847
P14079	Q9Y3X0	1.1621
P14079	Q6ZTR7	0.30187
P14079	Q07866	0.92144
P14079	Q3ZCX4	0.66611
P14079	P68036	0.4218
P14079	Q6ZVK1	1.2485
P14079	Q9BVM2	0.84298
P14079	A8MTZ7	0.66624
P14079	O95684	1.0837
P14079	Q7L2J0	0.27713
P14079	Q7L0X2	1.0551
P14079	Q9P209	1.2138
P14079	Q9Y6K1	0.43784
P14079	Q6ZT77	0.64204
P14079	Q8NBQ7	0.56109
P14079	O15535	0.43752
P14079	O95359	0.43228
P14079	Q6NVV3	1.288
P14079	Q8N8P6	1.0612
P14079	Q8IXJ9	0.73836
P14079	P04066	0.66068
P14079	C9J069	0.41561
P14079	Q5T5W8	1.2159
P14079	P00338	0.60641
P14079	Q16594	0.6586
P14079	Q49AJ0	0.62528
P14079	P22735	0.14639
P14079	Q9UPR6	0.66974
P14079	Q9C0B1	0.50743
P14079	Q96S52	0.69967
P14079	P61576	0.722
P14079	Q9BYR2	1.3698
P14079	Q9GZU2	0.67038
P14079	Q13309	0.67451
P14079	Q6ZS72	0.94472
P14079	Q658K8	1.0629
P14079	A6NIJ5	0.13295
P14079	Q16517	0.96502
P14079	Q6ZW54	1.7187
P14079	Q8IZA3	0.67228
P14079	Q9H4H8	0.64779
P14079	Q2VYF4	0.85052
P14079	O14893	0.68092
P14079	Q9Y6X2	0.64352
P14079	Q9H0A0	0.94511
P14079	P47813	1.5452
P14079	Q92734	0.36397
P14079	Q8N339	0.22441
P14079	Q3MIV0	1.1715
P14079	Q96IV0	0.58554
P14079	P56589	0.33421
P14079	Q9HCG1	0.80718
P14079	Q96A22	1.1507
P14079	P17035	0.52589
P14079	Q8WWL2	0.3662
P14079	P23409	0.081192
P14079	Q14192	0.63959
P14079	Q9Y5N5	0.47676
P14079	Q9UHF0	1.1527
P14079	A6NCL1	0.35299
P14079	Q9BQI9	1.5012
P14079	Q99581	0.68313
P14079	Q92830	0.8197
P14079	Q6NT46	0.99818
P14079	Q9Y2P0	0.59897
P14079	Q9H3M9	0.71083
P14079	P07951	0.04123
P14079	O43736	0.77936
P14079	Q9H0U6	0.91496
P14079	Q8IYK2	1.3214
P14079	Q8N660	1.9823
P14079	Q15173	0.5288
P14079	O75312	0.92205
P14079	Q5TFG8	1.0613

P14079	Q9BT09	0.5292
P14079	A6NCI8	1.3951
P14079	A6ZKI3	1.3539
P14079	Q6ZSN7	0.96683
P14079	Q9H7Y0	0.94831
P14079	Q9H5J8	1.6642
P14079	Q1HG43	1.3179
P14079	Q96DZ9	1.0706
P14079	Q8N0S6	0.86275
P14079	Q7Z4Q2	1.4682
P14079	Q8N865	0.73181
P14079	P83881	0.98326
P14079	A6NKX1	1.1715
P14079	Q96SZ5	0.51989
P14079	Q9BWH6	1.3299
P14079	Q9NVR2	1.3668
P14079	Q6UXV3	1.1715
P14079	P22234	0.76842
P14079	Q9Y2G0	0.95185
P14079	Q9P266	1.1634
P14079	O75626	0.31558
P14079	O75368	0.8004
P14079	Q8IVF1	1.6599
P14079	O60664	0.86191
P14079	Q96PV6	1.0191
P14079	Q969E4	0.73922
P14079	P62072	0.52408
P14079	Q9C0B9	0.18006
P14079	A6NP81	0.28495
P14079	Q9NSI2	1.3222
P14079	P52564	0.24996
P14079	A4UGR9	0.48781
P14079	Q6MZQ0	0.86038
P14079	O15519	0.5452
P14079	P20132	0.041198
P14079	Q5VXH5	0.23582
P14079	Q96P26	0.70913
P14079	Q6P161	0.376
P14079	F7VJQ1	1.1715
P14079	Q711Q0	1.1715
P14079	Q6IPR3	0.80916
P14079	Q8IXH7	1.3135
P14079	Q9Y2Y9	0.92235
P14079	Q96R06	1.0472
P14079	Q8N6L1	1.0688
P14079	Q6P9G9	0.48299
P14079	Q8N7P7	0.46867
P14079	O43597	0.15013
P14079	Q8TA94	1.0284
P14079	Q15700	0.29077
P14079	O43309	0.60694
P14079	Q52MB2	1.4444
P14079	Q99456	0.99997
P14079	Q14938	0.29792
P14079	Q86TN4	0.67801
P14079	Q8ND56	1.2179
P14079	Q9BU76	1.4935
P14079	Q3MIT2	0.44242
P14079	Q96N06	1.084
P14079	Q92614	0.26709
P14079	Q330K2	0.86555
P14079	Q9H4D5	0.98562
P14079	Q15911	0.22908
P14079	Q96QR8	0.73843
P14079	Q8NDC0	1.3459
P14079	Q9BYR9	0.94393
P14079	O75362	0.91716
P14079	Q96M43	0.82478
P14079	Q9NTK1	1.6309
P14079	Q5JXB2	0.44562
P14079	Q96HI0	0.40396
P14079	Q9UF83	0.83787
P14079	Q8TCD5	0.50544
P14079	P17010	0.43648
P14079	Q68CJ9	0.58529
P14079	Q15723	1.0851

P14079	Q9UGP5	0.25585
P14079	Q9HCI6	0.93349
P14079	P20292	0.39129
P14079	Q9Y2N7	0.54053
P14079	B2RD01	0.38266
P14079	Q9Y6A5	0.095896
P14079	Q9Y2Q1	1.0266
P14079	P35398	0.46957
P14079	Q5T6L9	1.0613
P14079	P17812	0.80501
P14079	Q9UKF6	0.80097
P14079	Q6UWF5	1.1715
P14079	Q96GQ5	0.79956
P14079	A4FU69	0.53508
P14079	Q6UX68	1.3388
P14079	Q9BV87	0.84473
P14079	Q9BRL7	0.97088
P14079	Q9NXE4	0.77887
P14079	Q9H0W8	1.4129
P14079	Q8NA34	0.13538
P14079	Q9UKN8	0.86685
P14079	Q8NHU2	0.48655
P14079	Q0D2K0	1.288
P14079	P0CI01	1.092
P14079	Q9UBW8	0.42871
P14079	Q5TAG4	0.88477
P14079	Q01082	0.61605
P14079	Q5T3J3	1.0064
P14079	Q8N9T8	1.8629
P14079	P0CB33	0.98083
P14079	P78396	1.1467
P14079	Q9HB09	1.8408
P14079	P30040	0.059223
P14079	A1A519	1.7332
P14079	O75896	1.1268
P14079	P09912	0.6358
P14079	Q5VX52	0.34135
P14079	Q8N3Z3	0.94396
P14079	Q96CD0	1.1399
P14079	Q9BVM4	0.9671
P14079	A6QL64	1.0803
P14079	O95271	0.64615
P14079	O14896	0.34791
P14079	Q8NFW8	0.65981
P14079	P53567	0.49685
P14079	Q16537	0.58533
P14079	Q86VI4	0.76981
P14079	P0C5Y4	1.1219
P14079	P56750	0.54752
P14079	Q9UKD1	0.69547
P14079	Q9NX95	0.90674
P14079	Q9UJ70	0.48917
P14079	Q8N888	1.2821
P14079	Q96M53	1.8704
P14079	Q9Y5N6	0.90431
P14079	A6NJJ6	1.3222
P14079	Q9BYR6	1.5617
P14079	Q9H9C1	0.65932
P14079	Q9BR84	1.07
P14079	Q15031	0.97261
P14079	Q14103	0.66896
P14079	P23434	0.51168
P14079	Q9UFG5	0.7692
P14079	P67809	0.56575
P14079	Q13505	1.5024
P14079	Q5T6M2	1.5841
P14079	Q8TF01	1.5675
P14079	Q7LFL8	0.075161
P14079	Q8IYD8	0.39328
P14079	P80217	0.806
P14079	Q9UGN5	0.2987
P14079	P0C7H8	0.94393
P14079	P47974	0.29099
P14079	Q6Q795	1.1715
P14079	P0C7N4	1.0429
P14079	Q8N8D1	1.4368

42333	P14079	Q12799	0.028939
42334	P14079	Q4VC05	1.3208
42335	P14079	Q9Y5M6	1.1715
42336	P14079	Q6ZTY9	1.1354
42337	P14079	Q1A5X7	0.11054
42338	P14079	Q8NFD4	0.90722
42339	P14079	Q9BWU0	0.70821
42340	P14079	O95817	0.53436
42341	P14079	Q5VTT2	0.94348
42342	P14079	Q96RQ1	0.29997
42343	P14079	P68104	0.64261
42344	P14079	Q5SW24	0.47276
42345	P14079	P53999	1.1513
42346	P14079	Q9UPN7	1.0595
42347	P14079	Q96FJ2	0.89172
42348	P14079	P50995	1.038
42349	P14079	Q15714	0.82485
42350	P14079	Q6RVD6	1.1715
42351	P14079	Q8TAG9	0.3082
42352	P14079	Q502W6	0.54949
42353	P14079	Q9UKJ5	0.62929
42354	P14079	Q9NWF4	1.2062
42355	P14079	Q8NDN9	0.49523
42356	P14079	Q6GV28	1.1349
42357	P14079	Q9H1L0	1.3598
42358	P14079	Q8TCC3	0.57577
42359	P14079	Q96LI6	0.39223
42360	P14079	A6NDV4	0.14017
42361	P14079	Q96DS6	1.2485
42362	P14079	Q8WWF3	1.3357
42363	P14079	P24864	0.78621
42364	P14079	Q86YH6	0.2216
42365	P14079	Q8IU60	0.86612
42366	P14079	Q5T160	1.1272
42367	P14079	Q2VPB7	0.87242
42368	P14079	P41567	1.3714
42369	P14079	Q8N108	1.1708
42370	P14079	Q96KT6	1.1715
42371	P14079	P62487	0.60253
42372	P14079	Q9NUJ7	0.5661
42373	P14079	Q8IZW8	0.85412
42374	P14079	P10244	0.65854
42375	P14079	Q86VS8	0.63276
42376	P14079	Q9HB07	0.72844
42377	P14079	Q9Y237	0.33327
42378	P14079	Q9BVC6	1.4292
42379	P14079	Q8N782	0.97319
42380	P14079	Q9BY44	0.52452
42381	P14079	Q86VY9	1.0007
42382	P14079	Q99700	0.87687
42383	P14079	O95503	0.95514
42384	P14079	P49842	1.1278
42385	P14079	Q9UJM3	0.52961
42386	P14079	O15258	0.76541
42387	P14079	Q15155	0.97149
42388	P14079	Q9NW08	0.1289
42389	P14079	Q14146	1.1715
42390	P14079	Q9NUA8	0.51237
42391	P14079	Q96CP7	1.2485
42392	P14079	Q96KH6	1.2843
42393	P14079	Q8N9E0	1.3222
42394	P14079	Q9ULX6	0.88269
42395	P14079	Q6F5E7	0.68744
42396	P14079	O43439	0.91536
42397	P14079	Q7Z6K1	1.182
42398	P14079	Q9UHR6	1.4363
42399	P14079	Q6WRX3	0.027675
42400	P14079	Q68DH5	1.4936
42401	P14079	O15304	1.3809
42402	P14079	P43360	0.36435
42403	P14079	O95789	1.2437
42404	P14079	Q8TDY2	0.82542
42405	P14079	Q9UNX3	1.3864
42406	P14079	P82664	1.5725
42407	P14079	P31939	0.67507
42408	P14079	Q9BUE0	1.3926

P14079	Q9UNQ2	0.93114
P14079	Q5SVZ6	1.6391
P14079	O43924	0.36055
P14079	Q9NSK7	1.4847
P14079	Q96MY7	1.6715
P14079	Q4VX62	0.77043
P14079	P86790	0.80083
P14079	Q96CJ1	1.3323
P14079	P46781	1.2432
P14079	Q8NEX6	1.1226
P14079	P36873	0.69438
P14079	Q9NP66	0.79349
P14079	Q0VFX4	1.0286
P14079	Q9NX70	1.0559
P14079	Q5T4T1	1.025
P14079	Q6NUP7	0.63548
P14079	Q9UJY5	0.83465
P14079	Q16658	0.42094
P14079	Q52LA3	1.152
P14079	Q9UFB7	0.59641
P14079	Q7RTT3	0.49064
P14079	P26998	1.2574
P14079	P17022	0.41703
P14079	Q8N1W0	1.1715
P14079	P82979	0.43303
P14079	Q96GE4	0.76753
P14079	Q92966	1.2303
P14079	P49354	0.38653
P14079	P05408	0.98291
P14079	P11473	0.48733
P14079	Q8NDA8	1.4027
P14079	Q9H0M5	0.54706
P14079	Q9Y6I3	0.23461
P14079	Q96G30	0.92676
P14079	P57740	0.85714
P14079	Q3KP31	0.95434
P14079	Q8N9B5	1.0575
P14079	Q96NT3	1.0164
P14079	P17041	0.55842
P14079	O60493	0.30428
P14079	Q14210	0.59903
P14079	Q9UBL3	0.25994
P14079	Q96BN8	0.71631
P14079	Q96CM3	0.81637
P14079	Q13021	0.54171
P14079	Q8IVL6	0.65898
P14079	Q9BW85	1.5305
P14079	P83731	0.96995
P14079	O15453	1.1598
P14079	Q9XRX5	0.79394
P14079	A6NGR9	1.2633
P14079	Q99873	0.64066
P14079	C9JE40	1.0499
P14079	P43357	0.56221
P14079	O75781	0.50667
P14079	Q8TEV8	1.1715
P14079	A2RU30	1.2979
P14079	Q9H9Q4	0.60485
P14079	Q9UBS3	0.62663
P14079	Q8WVR3	1.3222
P14079	Q8N1F7	0.71107
P14079	Q4KN68	0.15711
P14079	P10109	0.28702
P14079	O60290	1.1175
P14079	Q8N393	0.60102
P14079	P41223	0.31565
P14079	Q9H492	0.40057
P14079	P61513	0.83081
P14079	Q96F63	1.3222
P14079	P20396	1.0221
P14079	P59103	0.93007
P14079	P35680	0.2541
P14079	O60682	0.27989
P14079	O75533	0.37271
P14079	O15446	0.88073
P14079	P20265	0.60535

P14079	P40937	0.173
P14079	Q8N8F4	0.64849
P14079	Q8IXT2	0.29941
P14079	Q6IA86	0.51675
P14079	Q96GX1	0.79662
P14079	P0C838	1.0064
P14079	Q15006	0.39633
P14079	Q5TEC3	0.5681
P14079	Q7L5D6	1.6747
P14079	P50479	0.47474
P14079	Q9Y617	0.7317
P14079	Q9NY61	0.98029
P14079	Q6UWS5	0.86323
P14079	Q7Z3Z0	0.99997
P14079	P62847	1.0406
P14079	Q5T0B9	0.1949
P14079	P0CZ25	0.81101
P14079	P13010	0.30948
P14079	Q96ST3	0.67754
P14079	Q8N7N1	0.050966
P14079	Q8WUF5	0.81309
P14079	Q13023	0.77871
P14079	Q6ZQP7	1.1715
P14079	O00193	1.6655
P14079	P56378	1.1272
P14079	Q9NXW9	0.83947
P14079	Q15819	0.53821
P14079	O60543	0.58255
P14079	P15515	0.88262
P14079	Q6IS14	0.39345
P14079	Q86W33	1.1823
P14079	P41091	0.31388
P14079	Q15024	0.52739
P14079	Q99676	0.75812
P14079	Q5T7W7	1.3222
P14079	Q701N2	1.1715
P14079	Q9NRG0	0.44007
P14079	P40306	0.88237
P14079	Q92782	0.61704
P14079	Q6P087	0.39241
P14079	Q96FF7	1.1715
P14079	O76011	0.99997
P14079	Q92558	0.71767
P14079	P55347	0.37147
P14079	Q6W349	1.3323
P14079	Q9UKS6	0.3323
P14079	Q5T742	1.1715
P14079	P09086	0.26326
P14079	P31941	0.30542
P14079	Q8N8W4	0.45174
P14079	Q9BTD1	0.77186
P14079	Q9NQ31	1.273
P14079	P13498	0.31245
P14079	A6NKN8	1.5961
P14079	Q7Z7H3	0.71638
P14079	Q75VX8	1.5373
P14079	Q9NP84	0.95443
P14079	Q9NR12	0.16103
P14079	Q96RK0	0.98143
P14079	Q96CS3	0.69587
P14079	A6NER3	0.33792
P14079	P39656	0.79564
P14079	Q9NZ32	0.92536
P14079	A8MT65	0.96521
P14079	O75582	0.3381
P14079	Q96JK9	1.0237
P14079	Q8NCP5	0.1949
P14079	Q16254	0.91448
P14079	Q71F78	1.0181
P14079	Q9UK45	0.75543
P14079	Q96L14	0.84394
P14079	O95229	0.88917
P14079	Q08AN1	0.77845
P14079	A6NJG6	0.28169
P14079	O14641	0.52079
P14079	P07476	1.2019

P14079	Q6XD76	0.2791
P14079	Q86VX9	1.5384
P14079	Q8NB42	1.0252
P14079	P10828	0.58522
P14079	Q92541	0.99479
P14079	Q16829	0.52653
P14079	Q49AM1	0.70694
P14079	P0CW27	0.75981
P14079	Q9BSU1	1.2168
P14079	Q9BY42	1.2722
P14079	Q9BXU0	0.69749
P14079	Q15053	0.79083
P14079	Q14151	0.6755
P14079	A6NDU8	1.1192
P14079	P62753	1.1043
P14079	Q9BVL4	1.8084
P14079	O43679	0.39526
P14079	Q86Y79	1.1862
P14079	Q6WQI6	0.66064
P14079	Q5VVH5	0.80142
P14079	Q8TE85	0.26702
P14079	Q96JC9	0.9657
P14079	Q9Y5Z4	0.19405
P14079	Q9NPJ8	0.7173
P14079	Q58F21	0.44466
P14079	Q8IVM7	1.1715
P14079	Q9BYH8	0.40073
P14079	Q9UBF6	0.65728
P14079	Q8WVX3	1.2485
P14079	A6NCL2	0.18105
P14079	Q96NU7	0.75964
P14079	Q8NBT2	0.83496
P14079	Q96IR3	1.3298
P14079	Q02083	0.66334
P14079	Q9NVM4	0.16757
P14079	Q13907	0.48508
P14079	A8MXV4	1.2061
P14079	P16083	0.40879
P14079	Q5W0V3	1.5066
P14079	Q5MJ07	1.5871
P14079	Q13620	0.65427
P14079	Q92542	0.6773
P14079	I3L0S3	1.1511
P14079	Q9H4G8	0.83309
P14079	Q6BDI9	1.029
P14079	Q969T7	0.39521
P14079	A6NK89	1.2971
P14079	Q12840	0.42294
P14079	P56537	1.4076
P14079	P82933	1.0084
P14079	Q499Z3	1.1457
P14079	Q9BRU9	0.83891
P14079	Q14410	0.88589
P14079	Q08722	0.64962
P14079	Q14157	1.2871
P14079	Q13323	0.72882
P14079	Q9UJX3	1.0375
P14079	Q2QGD7	0.66216
P14079	Q9BSI4	1.3216
P14079	Q86XT2	0.39477
P14079	Q3B7S5	1.1715
P14079	P08235	0.41817
P14079	Q6YFQ2	0.037428
P14079	Q9BYQ9	1.3698
P14079	Q10570	0.9768
P14079	P98171	0.30249
P14079	Q8IV33	1.0467
P14079	A6NC78	0.5943
P14079	P52434	0.57715
P14079	P02511	0.32211
P14079	Q9UI47	0.56042
P14079	A8MU93	1.3222
P14079	Q8IVG5	0.31741
P14079	Q9NZ42	0.85424
P14079	P48634	1.1122
P14079	Q8IUC6	0.52952

P14079	H3BNL1	1.181
P14079	Q92985	0.61377
P14079	A6NFK2	0.62696
P14079	Q32M78	0.93128
P14079	Q6PDB4	1.0584
P14079	Q9BQ48	1.4448
P14079	Q9UNY4	0.3458
P14079	Q86WT1	0.27301
P14079	P15924	0.55556
P14079	Q96KN4	0.38904
P14079	P17039	0.51784
P14079	Q13404	1.2576
P14079	Q9NRR8	1.2613
P14079	Q9BUW7	1.3222
P14079	Q8IYN2	1.7066
P14079	O60832	0.76493
P14079	Q9HBM0	1.2946
P14079	Q8IYX3	1.4597
P14079	P52298	0.61677
P14079	P04156	0.77254
P14079	Q9UMX0	0.98861
P14079	Q8N0X2	0.34708
P14079	P55036	1.1063
P14079	Q6V702	0.58571
P14079	Q6ZQX7	0.27848
P14079	P35658	1.0771
P14079	Q9H1K0	0.12567
P14079	O94986	1.5399
P14079	Q9UHP9	0.66609
P14079	Q9BPY3	0.89702
P14079	P13985	1.1715
P14079	Q96HY6	0.79795
P14079	O76095	0.81226
P14079	P35606	0.61982
P14079	Q92564	0.31874
P14079	O15231	0.21523
P14079	Q5EBM4	0.27146
P14079	Q86UT5	0.24885
P14079	F5H4B4	1.5912
P14079	Q9NTI7	1.2694
P14079	P40692	0.28054
P14079	Q32MH5	1.1967
P14079	A6NKC0	0.57067
P14079	A6NM45	0.63456
P14079	Q9P0M2	0.22734
P14079	P84157	1.2485
P14079	Q9HCK1	0.96374
P14079	Q9NPI6	0.94005
P14079	Q5JQF7	1.1715
P14079	Q6P988	1.0653
P14079	P47756	0.27258
P14079	Q92804	0.35586
P14079	Q14207	0.83595
P14079	Q9Y314	1.0221
P14079	Q9UBZ4	0.061983
P14079	P43359	1.4995
P14079	Q9UQF2	0.52746
P14079	Q99871	0.71672
P14079	Q3MIR4	0.18288
P14079	A6NEM1	1.3415
P14079	Q9BY43	0.656
P14079	P52209	0.55917
P14079	Q8N2C7	1.5254
P14079	Q9BV20	0.77897
P14079	Q63HQ0	1.1715
P14079	Q5T230	2.0479
P14079	Q7Z739	1.1366
P14079	Q96DF8	1.3968
P14079	Q9BRQ4	1.052
P14079	Q13098	0.26348
P14079	Q8IYJ3	0.32436
P14079	P0CH99	1.1929
P14079	Q92793	0.69945
P14079	O00170	0.61744
P14079	Q7Z5Y7	0.30585
P14079	Q5JTZ5	0.57367

P14079	Q9P0U1	1.0078
P14079	Q05D60	0.36147
P14079	Q6NUN9	0.84305
P14079	Q9HAY2	0.30704
P14079	Q13033	0.92252
P14079	Q9HCE1	0.60781
P14079	P51522	0.58766
P14079	Q496A3	0.83405
P14079	Q8N5T8	1.2255
P14079	P28074	1.0683
P14079	Q30KQ7	0.91101
P14079	Q9ULM2	0.69526
P14079	P27352	0.9562
P14079	Q92545	1.2485
P14079	Q9H521	0.03933
P14079	Q5BN46	0.88669
P14079	Q96ED9	0.7747
P14079	Q7Z388	1.1385
P14079	Q8N9H9	1.1715
P14079	Q99733	0.73466
P14079	Q9HC52	0.7351
P14079	Q9NRL2	0.97252
P14079	Q5JVG8	0.96469
P14079	P12524	0.29305
P14079	Q15172	0.38732
P14079	Q07157	0.88597
P14079	Q9UPM8	0.56649
P14079	Q8WVT3	0.18584
P14079	Q6PK04	1.4147
P14079	Q0VG73	1.1715
P14079	Q8NFR7	1.2417
P14079	Q6WBX8	0.76703
P14079	Q9Y675	1.4627
P14079	Q96I76	0.27735
P14079	Q96LB4	0.43194
P14079	Q5JXM2	1.0967
P14079	Q9UBB5	1.3761
P14079	Q5PSV4	0.42468
P14079	Q9H171	0.49486
P14079	Q9BZK3	0.67171
P14079	Q70IA6	0.40175
P14079	Q6GMV2	0.74886
P14079	Q13618	0.35148
P14079	Q9BT56	1.1875
P14079	Q5GH72	1.3596
P14079	P57772	1.1669
P14079	A6NHJ4	1.07
P14079	Q8NDZ2	0.96935
P14079	Q9BPX7	1.063
P14079	Q9BWC9	1.5875
P14079	Q9BYQ3	1.3698
P14079	Q13166	1.1715
P14079	Q9NZ45	0.78861
P14079	Q15013	1.0954
P14079	P26641	0.68239
P14079	Q8N4H0	0.59278
P14079	O95948	0.30148
P14079	Q5SQH8	0.51999
P14079	P21399	0.42352
P14079	Q99549	0.48218
P14079	P61582	1.7865
P14079	Q9NSB4	0.85949
P14079	A1A4F0	0.35675
P14079	P50135	0.12281
P14079	Q16633	0.64909
P14079	Q9UNS2	0.81255
P14079	Q9Y5R5	0.19688
P14079	Q8N157	0.39687
P14079	P39023	1.1266
P14079	A6NLE4	1.2485
P14079	Q9H1J1	1.3524
P14079	Q9BXJ9	0.4684
P14079	Q8TE76	0.42635
P14079	Q8WX93	0.69097
P14079	C9J3I9	1.0315
P14079	A8MSI8	1.9145

P14079	Q66K74	0.28039
P14079	Q96CT7	0.43999
P14079	Q15528	1.1926
P14079	P48728	0.53448
P14079	O00232	0.72906
P14079	Q99622	0.54599
P14079	P49006	1.4773
P14079	Q12874	0.44491
P14079	Q9BWF2	0.37163
P14079	Q9NX08	0.56221
P14079	Q9BWT6	0.10992
P14079	Q8N3Y1	0.45433
P14079	Q9H0Y0	0.34298
P14079	Q4G0N0	0.89158
P14079	P28370	0.43832
P14079	P62273	1.4589
P14079	Q9NTW7	0.95408
P14079	P61457	0.87863
P14079	Q49AN0	0.12712
P14079	O14604	1.2142
P14079	Q6ZVN7	0.91226
P14079	O75290	0.96033
P14079	Q8NCQ2	1.3772
P14079	Q49A88	1.1401
P14079	Q99607	1.0899
P14079	Q8IYT1	1.1067
P14079	P07311	1.298
P14079	Q96FF9	1.3051
P14079	Q9UI56	1.1715
P14079	Q6P9F0	0.67688
P14079	O43823	0.895
P14079	Q9Y586	0.59822
P14079	A6NI86	1.2306
P14079	Q9P2Y4	0.24294
P14079	P52746	0.28961
P14079	P01225	0.24756
P14079	Q7L2E3	0.33056
P14079	Q6ZN57	0.52984
P14079	Q93052	0.48737
P14079	Q02410	0.57606
P14079	Q9Y468	0.8985
P14079	Q9BTA9	0.95712
P14079	Q96DT0	0.20961
P14079	Q86VG3	1.2723
P14079	Q8IWF7	0.19124
P14079	Q9NVV9	1.3799
P14079	Q14696	0.97916
P14079	Q99829	0.28784
P14079	Q8TB05	1.3222
P14079	Q86WQ0	0.732
P14079	O75792	1.5397
P14079	P14672	0.73088
P14079	P20933	0.66957
P14079	Q86WR0	1.1509
P14079	Q9P016	1.4961
P14079	Q9NPI8	1.2203
P14079	O00165	1.1457
P14079	Q69YU5	0.98371
P14079	A8MSY0	0.52653
P14079	Q13094	0.70726
P14079	Q9Y2R9	1.5019
P14079	Q9BYE4	1.1715
P14079	Q8N3R3	0.63877
P14079	O75564	0.76486
P14079	Q96HJ9	0.76722
P14079	Q9H7H1	0.93329
P14079	A6NNA2	1.4206
P14079	Q69YH5	1.495
P14079	Q9P0W8	0.65242
P14079	Q5T2T1	0.40771
P14079	Q9BYD2	1.4448
P14079	Q8WYQ3	1.3165
P14079	Q13129	0.88416
P14079	D6RB28	1.3415
P14079	Q6ZRH9	1.1715
P14079	Q5ST30	0.73419

P14079	Q9BX73	0.80563
P14079	Q9NYZ3	1.4915
P14079	Q9H4L7	0.29307
P14079	Q9NW81	0.20584
P14079	Q0D2I5	0.38733
P14079	Q96A73	1.5724
P14079	Q96S07	1.1715
P14079	Q9BXQ6	1.1715
P14079	A6NF01	1.4294
P14079	Q75QN2	1.3543
P14079	Q9NYK6	1.0802
P14079	Q92556	0.53639
P14079	O00400	1.0907
P14079	Q969S8	0.86466
P14079	P47944	1.0978
P14079	Q9H1A4	1.0939
P14079	O95429	0.61714
P14079	Q8IUW3	1.3401
P14079	Q9BYC9	1.2139
P14079	Q6ZPB5	1.1715
P14079	Q13243	0.79526
P14079	O00471	0.43582
P14079	Q9BVK6	0.20815
P14079	Q14677	0.58936
P14079	Q10567	0.87114
P14079	Q6ZN03	0.8632
P14079	Q8IVD9	0.89721
P14079	Q5T0U0	0.12918
P14079	Q8IWL3	0.54202
P14079	Q8N1G2	0.33729
P14079	Q2KJ03	0.48688
P14079	Q9BX51	0.13772
P14079	Q8IVG9	1.0761
P14079	Q14CW9	1.2118
P14079	Q63HK3	0.56445
P14079	Q9NZV6	0.3781
P14079	Q9HCK5	0.95157
P14079	Q8IXQ3	0.63452
P14079	Q9UGJ1	0.67425
P14079	A8CG34	0.99979
P14079	Q9H8E8	0.80361
P14079	Q68CZ1	0.33637
P14079	Q15397	0.20375
P14079	Q9H777	0.79325
P14079	P39880	0.51723
P14079	Q2NL68	1.3222
P14079	Q9NUP7	1.3353
P14079	Q15583	0.39164
P14079	O60524	1.4972
P14079	Q9UMX2	0.3138
P14079	A8MZ25	0.17307
P14079	B1AMM8	0.875
P14079	Q6P3X8	1.3375
P14079	Q9Y5S9	0.62732
P14079	Q9H040	0.60908
P14079	Q9GZS1	0.47349
P14079	Q15041	0.26406
P14079	Q13522	0.30425
P14079	P24588	0.25947
P14079	Q7Z3K3	0.58103
P14079	Q96DU7	0.70156
P14079	P13807	0.27639
P14079	O43592	0.50074
P14079	P55771	0.76605
P14079	Q13835	0.35082
P14079	P46109	0.24549
P14079	Q8WUI4	0.43655
P14079	P12036	0.68291
P14079	Q6ZR52	0.78684
P14079	Q13901	0.97749
P14079	Q7Z6G3	0.62006
P14079	Q9NPD8	0.58328
P14079	O43432	0.045908
P14079	O14639	0.974
P14079	Q02241	0.83353
P14079	Q9UJX5	1.0125

42941	P14079	P0C852	1.1715
42942	P14079	P25398	0.81839
42943	P14079	P48382	0.90797
42944	P14079	O96000	1.0374
42945	P14079	O15405	1.0664
42946	P14079	O75475	1.5027
42947	P14079	A6NCJ1	0.96733
42948	P14079	P50991	0.35526
42949	P14079	Q8N910	0.31556
42950	P14079	Q2T9K0	0.95549
42951	P14079	Q16531	0.76901
42952	P14079	Q9NXH3	1.0405
42953	P14079	Q9UKH3	1.0034
42954	P14079	P0C867	1.07
42955	P14079	Q66K80	1.1715
42956	P14079	Q8NG50	0.39061
42957	P14079	Q2M329	1.1403
42958	P14079	Q9BZE9	1.1965
42959	P14079	Q902F9	0.80727
42960	P14079	Q9ULG3	1.1715
42961	P14079	Q8N336	0.81684
42962	P14079	Q9Y6D5	0.30397
42963	P14079	P49005	0.6571
42964	P14079	Q6PJT7	1.0148
42965	P14079	O95167	0.87638
42966	P14079	Q9NP79	0.50253
42967	P14079	Q96GX8	1.3222
42968	P14079	P13489	0.64259
42969	P14079	Q8WV74	0.13921
42970	P14079	Q8NDH3	0.79089
42971	P14079	Q2TAZ0	0.56221
42972	P14079	A6NNH2	0.4243
42973	P14079	Q9UBS4	0.54784
42974	P14079	Q05DH4	1.6004
42975	P14079	Q9ULU8	0.26624
42976	P14079	Q6P6C2	0.8109
42977	P14079	Q86U70	0.45678
42978	P14079	O15198	1.0002
42979	P14079	Q9UKZ1	1.3714
42980	P14079	Q9BUJ2	1.1339
42981	P14079	Q9UL41	1.1987
42982	P14079	P16298	0.14687
42983	P14079	Q9BSW2	0.86098
42984	P14079	O14974	0.55966
42985	P14079	Q9BUT9	1.8779
42986	P14079	P0CJ75	1.3031
42987	P14079	Q7Z422	0.53146
42988	P14079	Q6RW13	1.4172
42989	P14079	A8MXT2	0.24809
42990	P14079	Q8NA47	1.3222
42991	P14079	Q9BYR3	1.3698
42992	P14079	Q6NZY4	0.49573
42993	P14079	Q9BXL8	0.81186
42994	P14079	Q8NEK5	0.59459
42995	P14079	Q6P444	1.1992
42996	P14079	Q63HM1	0.83146
42997	P14079	Q9UII2	0.85704
42998	P14079	Q8NET4	1.2144
42999	P14079	Q9UPU9	0.75918
43000	P14079	Q9NVX0	1.1964
43001	P14079	Q8TBN0	0.53748
43002	P14079	Q8N5A5	0.76853
43003	P14079	Q96Q45	0.88585
43004	P14079	Q7Z3K6	0.71985
43005	P14079	O94901	0.30168
43006	P14079	Q14204	0.57736
43007	P14079	Q9UQR0	0.22401
43008	P14079	Q5SWH9	1.2485
43009	P14079	P48147	0.81974
43010	P14079	Q96HR3	0.88621
43011	P14079	P04080	0.066209
43012	P14079	Q7Z6I5	1.4881
43013	P14079	Q12906	1.1561
43014	P14079	Q2VWA4	0.1063
43015	P14079	Q8IYB9	0.8029
43016	P14079	Q9H707	0.64158

P14079	Q9NUB4	1.1715
P14079	Q495Z4	1.0931
P14079	Q96SR6	0.58948
P14079	Q6ZTN6	1.1646
P14079	Q96PM9	0.43669
P14079	Q15048	0.055376
P14079	Q9BW04	0.95175
P14079	O15514	0.3771
P14079	P62495	0.69372
P14079	Q9C000	0.36747
P14079	Q9BRJ2	0.67129
P14079	Q9NXG0	0.35976
P14079	Q8NHU6	0.27285
P14079	Q96D15	0.44757
P14079	Q6ZS49	1.1715
P14079	Q9H910	1.6397
P14079	Q15329	1.1949
P14079	Q9NP81	0.83172
P14079	Q9NQ29	1.0637
P14079	Q8WVI7	0.36001
P14079	Q16656	1.0213
P14079	P33240	0.56345
P14079	Q96BW9	1.0735
P14079	Q15276	0.83777
P14079	Q6L8H2	1.1715
P14079	Q99576	0.46533
P14079	P31949	0.45643
P14079	Q9BSV6	0.61691
P14079	P54802	0.84279
P14079	Q6ZT21	0.79459
P14079	Q6P1L5	2.0085
P14079	Q9GZL7	0.86736
P14079	P26038	0.3053
P14079	Q99594	1.1158
P14079	Q5JST6	0.97105
P14079	Q9H8X3	0.16445
P14079	Q5JTW2	0.73566
P14079	Q9NX46	0.75116
P14079	Q8N987	0.29425
P14079	Q9H7P6	1.674
P14079	P52737	0.77875
P14079	Q8N5J2	1.8829
P14079	O43913	0.4804
P14079	Q92834	0.63009
P14079	P35789	0.94578
P14079	Q68D20	0.37324
P14079	Q9H1K1	0.94316
P14079	Q8TB33	0.83592
P14079	Q6P3W6	1.236
P14079	Q9NWY4	1.2343
P14079	A0MZ66	1.1072
P14079	Q4G0N4	0.75561
P14079	Q7RTS7	0.97433
P14079	P60604	0.61533
P14079	A6NEL3	0.35845
P14079	Q6ZUU3	1.1715
P14079	P0CJ86	0.25626
P14079	P51815	0.54104
P14079	Q6IBW4	0.73692
P14079	Q9H3J6	1.4447
P14079	Q15699	0.35511
P14079	P54252	0.41508
P14079	Q13330	0.53548
P14079	A6NLJ0	1.6012
P14079	P57054	0.94337
P14079	Q8NEP4	1.3222
P14079	Q99932	1.1715
P14079	Q9Y315	1.1491
P14079	Q24JQ0	0.78375
P14079	Q6ZRM9	1.2464
P14079	Q86VZ6	0.25394
P14079	Q9Y6H5	0.82135
P14079	O94874	0.82023
P14079	Q1T7F1	0.78783
P14079	O75306	0.89958
P14079	Q5JUR7	0.21199

P14079	Q6ZNB5	0.53147
P14079	B3EWG4	1.3222
P14079	O43247	0.068981
P14079	P28347	0.98943
P14079	Q86TB9	1.0695
P14079	Q8NEK8	1.5082
P14079	Q8TAG6	1.1924
P14079	Q9UKY0	0.34626
P14079	Q8N0U7	1.012
P14079	Q3LI63	1.1715
P14079	P17096	0.98345
P14079	O14715	0.13526
P14079	P42566	0.30899
P14079	Q8N9L7	0.70752
P14079	P47897	0.779
P14079	P30876	0.61683
P14079	P0CG42	1.1715
P14079	Q9BUK6	0.3151
P14079	Q14691	0.72478
P14079	Q8IY22	0.1864
P14079	A8MYR8	0.16234
P14079	Q14591	0.45328
P14079	Q96LI5	0.49925
P14079	Q5SWW7	1.1715
P14079	Q15018	0.64332
P14079	P00395	0.34144
P14079	B2CW77	1.1715
P14079	Q03060	0.95697
P14079	Q96GL9	1.1789
P14079	Q9BZD3	0.94785
P14079	P56377	0.57164
P14079	Q8N9S7	0.84839
P14079	Q9BYS1	1.1219
P14079	P11474	0.48913
P14079	Q92922	1.0778
P14079	Q96F24	0.75767
P14079	Q6P5X5	1.3795
P14079	Q9Y2Y0	1.2551
P14079	Q16831	0.49326
P14079	Q8IWD4	1.3222
P14079	Q6ISU1	0.69075
P14079	Q92621	1.1991
P14079	Q6UXU1	1.1715
P14079	Q8WWA1	1.2485
P14079	Q70YC5	1.0991
P14079	A6NL05	1.005
P14079	Q96NB3	0.51682
P14079	O43815	0.43783
P14079	Q6ZN32	0.72221
P14079	Q6ZRF7	0.39965
P14079	Q8TBZ8	0.69402
P14079	O75865	1.0842
P14079	Q9Y2Z2	1.5239
P14079	Q5W0A0	0.70163
P14079	Q70EK8	1.0551
P14079	Q8TDQ7	0.053375
P14079	O95251	0.6797
P14079	P43490	0.69748
P14079	Q04118	1.1715
P14079	Q6Y7W6	0.65293
P14079	P48380	0.98409
P14079	Q6ZQR2	1.5458
P14079	O95363	1.0136
P14079	Q96EP0	0.20558
P14079	Q14320	0.46585
P14079	O95990	1.0831
P14079	Q9BZD4	0.80134
P14079	P52294	1.4023
P14079	Q8N2H9	0.65175
P14079	Q86V87	0.99816
P14079	P00403	0.76541
P14079	Q9H993	0.37056
P14079	Q9Y3C8	0.51437
P14079	Q14264	0.60504
P14079	P51532	0.79012
P14079	Q9H211	0.95809

P14079	Q00597	1.0419
P14079	Q5XG87	0.15525
P14079	Q8NE00	1.2633
P14079	Q8NCA9	0.335
P14079	P09105	0.67533
P14079	O60518	1.1388
P14079	A8MUV8	1.0106
P14079	Q14164	0.25232
P14079	Q8N7B6	0.73941
P14079	Q6NV74	1.3222
P14079	P40123	0.4194
P14079	Q9Y2T2	0.84417
P14079	Q9Y678	0.75494
P14079	Q9BV86	0.33293
P14079	Q6P1X6	1.3103
P14079	P49591	0.92101
P14079	Q9HC24	0.27402
P14079	Q96N76	0.65909
P14079	Q9NP50	1.2152
P14079	Q9UQB3	0.52048
P14079	Q96I36	1.0946
P14079	Q6UWZ7	0.84272
P14079	Q96NM4	0.38002
P14079	Q8WVP5	1.5386
P14079	O95644	0.76684
P14079	Q5VZ66	0.75883
P14079	P21980	0.21313
P14079	Q96S90	0.2718
P14079	Q6VEQ5	0.84005
P14079	Q9BXK5	0.80335
P14079	Q5SWL7	1.1083
P14079	Q9UGK3	0.76661
P14079	P61218	0.59036
P14079	P17024	0.68453
P14079	Q99497	0.091308
P14079	Q8WYR1	0.39037
P14079	Q9P255	0.74045
P14079	Q9UNL4	0.50807
P14079	Q92783	0.9133
P14079	Q14592	0.60546
P14079	Q9BTK6	0.84306
P14079	Q9BRA0	0.28036
P14079	P49788	0.78916
P14079	Q8N0U4	0.81511
P14079	Q9GZX5	0.86826
P14079	Q7Z4G4	0.35527
P14079	Q12980	1.2391
P14079	Q8WXE1	0.99045
P14079	Q15742	0.46925
P14079	Q96K49	0.79282
P14079	O75800	0.6398
P14079	Q3MJ62	0.48579
P14079	C9J3V5	1.0795
P14079	P0C5K7	0.33379
P14079	P43307	0.80429
P14079	Q7Z417	1.1629
P14079	P54278	0.43186
P14079	Q15170	2.1047
P14079	Q9P013	1.2265
P14079	P17568	0.19329
P14079	Q8WTT2	0.59924
P14079	P26368	0.60822
P14079	Q9H5L6	0.77013
P14079	Q2M389	0.74498
P14079	P35475	0.52971
P14079	Q6P2C8	0.93744
P14079	Q9NSJ1	0.68895
P14079	Q5U5X8	1.5594
P14079	Q86U86	1.0804
P14079	Q92540	0.96552
P14079	A6NNL0	1.8041
P14079	P56385	0.77685
P14079	O95405	0.30299
P14079	Q9Y6B7	0.49688
P14079	Q8NFJ9	0.63134
P14079	Q96BW5	0.8897

P14079	Q96NG8	0.6166
P14079	Q01538	0.45954
P14079	Q9Y3C7	0.97429
P14079	Q96MU5	1.2549
P14079	O14646	0.44836
P14079	Q9BSF4	0.74338
P14079	Q5SYE7	1.2838
P14079	Q9NXV6	1.5226
P14079	P58005	0.84857
P14079	Q6ZN79	0.94289
P14079	Q5T0J7	1.1691
P14079	P0C866	0.51483
P14079	P0C7M6	0.17064
P14079	Q96DD7	0.78072
P14079	Q01804	0.2747
P14079	Q5VVC0	0.76785
P14079	P49247	1.1377
P14079	Q96S94	0.35751
P14079	Q6PI77	1.283
P14079	Q14865	0.37582
P14079	Q66GS9	0.76582
P14079	Q8TED0	0.31573
P14079	O60675	0.44679
P14079	Q9BRP9	0.50849
P14079	Q8NEM2	0.92809
P14079	P10606	0.47904
P14079	Q9Y6G3	0.55627
P14079	Q9BTP6	1.3429
P14079	Q5BKU9	0.51526
P14079	Q8NG57	0.80003
P14079	Q629K1	1.0399
P14079	O60688	0.68732
P14079	B5MD39	0.28751
P14079	Q9UDY4	0.78805
P14079	Q9P1F3	1.2801
P14079	P29372	1.2326
P14079	Q2PZI1	1.5121
P14079	Q96B49	1.4739
P14079	Q99684	0.54469
P14079	Q8IV56	1.1456
P14079	Q8N2C9	0.64619
P14079	Q8TEM1	0.72143
P14079	Q6PCB6	0.34355
P14079	O15392	0.28548
P14079	Q86TP1	1.2008
P14079	Q9NTM9	0.3112
P14079	Q92610	0.24729
P14079	P83876	0.97112
P14079	Q6ZRS4	1.2979
P14079	O43598	0.7687
P14079	Q93099	0.48608
P14079	Q7L4I2	2.1207
P14079	Q66K64	0.86207
P14079	Q9BR77	0.76763
P14079	A2RTX5	0.81576
P14079	Q01826	0.87441
P14079	Q96N20	0.52472
P14079	O60907	0.80857
P14079	Q9ULP0	0.47958
P14079	Q9NRX2	0.96926
P14079	Q8IW70	1.2485
P14079	Q13070	0.33792
P14079	A6NJV1	1.3341
P14079	O75420	0.63989
P14079	P13646	0.92977
P14079	P61579	1.355
P14079	Q7Z6K5	0.68728
P14079	Q8TEV9	1.2636
P14079	Q6PEW1	0.19064
P14079	Q9H3P2	1.4894
P14079	Q9H8H0	0.68182
P14079	Q16585	0.9149
P14079	Q8IXF0	0.37081
P14079	Q5JT78	0.69382
P14079	P18077	0.69763
P14079	P49720	0.97585

P14079	P35712	0.52518
P14079	Q8TB03	1.2104
P14079	Q969T9	1.7449
P14079	Q9NXL6	0.76249
P14079	Q9UID6	0.55199
P14079	A4D174	0.52953
P14079	Q7LBR1	1.1214
P14079	O95163	0.62066
P14079	O75529	0.5236
P14079	Q9BU70	0.64125
P14079	Q86Z20	1.4841
P14079	Q9Y291	1.0294
P14079	Q5T0F9	0.50026
P14079	Q93062	0.71903
P14079	Q8WV48	1.2485
P14079	A8K5M9	1.6201
P14079	A4D1E9	1.5514
P14079	Q8NBZ9	0.83085
P14079	Q9ULK4	1.2555
P14079	Q8NE01	0.53036
P14079	O75807	0.69313
P14079	Q13536	1.1715
P14079	Q6ZSR3	1.1715
P14079	Q6ZUJ8	0.32152
P14079	Q86TU7	0.36324
P14079	P0C875	0.56468
P14079	O00487	0.28065
P14079	Q0D2H9	0.84738
P14079	O75570	1.0993
P14079	O95867	0.96241
P14079	H3BN30	1.1715
P14079	Q9H008	0.65524
P14079	Q9UMX1	1.2224
P14079	P09972	0.5915
P14079	A6NE82	0.44102
P14079	Q8WVJ9	0.53667
P14079	Q6IPX3	1.543
P14079	Q9H8T0	0.57128
P14079	O95190	1.2189
P14079	O95931	0.85285
P14079	Q6B9Z1	1.1517
P14079	Q68DI1	0.66003
P14079	Q9NQT4	0.27269
P14079	Q495Y8	1.092
P14079	Q9Y250	0.9211
P14079	Q13428	1.7521
P14079	Q13487	2.0772
P14079	Q52LG2	0.95677
P14079	Q8N9A8	1.2716
P14079	Q16891	0.65831
P14079	Q717R9	1.3143
P14079	Q6ZUF6	1.0905
P14079	Q93100	0.704
P14079	Q9BXS4	0.83033
P14079	Q9BPX5	0.19716
P14079	Q9NWZ5	0.342
P14079	Q9UPA5	0.58904
P14079	Q16186	0.63176
P14079	P62136	0.5688
P14079	P11172	0.81083
P14079	Q86TL0	0.73221
P14079	Q3BBW0	0.51084
P14079	Q3KNT7	0.41165
P14079	Q9UDW1	0.6875
P14079	Q7L5Y6	0.1664
P14079	Q9C0C6	1.3481
P14079	P25787	1.0803
P14079	Q5VTU8	0.49269
P14079	Q658Y4	1.0917
P14079	Q00994	1.5182
P14079	Q92902	0.34412
P14079	Q9P260	0.80964
P14079	P0DI83	1.1422
P14079	Q9UKK9	0.83288
P14079	Q92698	0.28319
P14079	Q9NTJ4	0.44561

P14079	P51587	0.20732
P14079	O60216	0.94366
P14079	Q5GH76	1.3596
P14079	Q6ICG7	1.1715
P14079	Q8N0Y2	0.55847
P14079	Q8N720	0.86517
P14079	P80294	0.83367
P14079	Q1MSJ5	1.7629
P14079	Q8IYT3	0.26344
P14079	Q6UWF3	1.3655
P14079	Q8IUC3	1.497
P14079	O60684	0.50025
P14079	P07438	0.77468
P14079	Q75L30	1.1715
P14079	Q5JRV8	1.1449
P14079	Q8N8X6	0.22668
P14079	Q6NXN4	1.2737
P14079	Q8IV20	1.0931
P14079	Q14966	0.30514
P14079	Q9NXJ5	1.0009
P14079	Q15070	0.62543
P14079	A6NCN2	0.99362
P14079	Q08AI8	1.3222
P14079	P22557	0.074483
P14079	Q6ZNA4	0.40493
P14079	P69208	1.1715
P14079	P29401	0.31202
P14079	Q8N7Z2	0.89121
P14079	Q6ZRI6	1.185
P14079	Q8N769	0.83516
P14079	Q8IX15	0.42798
P14079	Q9NVC6	0.67274
P14079	O75179	0.50099
P14079	P07737	0.96923
P14079	Q99966	0.19704
P14079	Q8WV83	0.82384
P14079	Q96N19	0.97467
P14079	Q13868	0.52815
P14079	Q32NC0	0.97877
P14079	Q8IWZ3	1.074
P14079	P49184	0.71019
P14079	Q8NI27	1.2149
P14079	P54198	0.99196
P14079	Q9H7X3	0.52651
P14079	P59780	0.2355
P14079	P32189	0.87601
P14079	Q9ULN7	1.5833
P14079	Q96JG6	0.43687
P14079	P58304	0.27651
P14079	P0CJ68	1.3031
P14079	Q9HC16	0.22442
P14079	Q9UNX4	0.3761
P14079	Q9H773	1.0021
P14079	O94855	0.61526
P14079	O00189	0.66338
P14079	Q5H9I0	0.3082
P14079	Q9P1Q0	0.6474
P14079	Q9BTV7	0.37606
P14079	Q9UHC1	0.56898
P14079	Q96ST8	1.6547
P14079	Q6IEG0	0.9684
P14079	P51531	0.75882
P14079	P42704	1.1178
P14079	Q96SE7	0.76643
P14079	Q96MF0	0.64425
P14079	Q14209	1.0257
P14079	Q8N8G6	0.94062
P14079	Q96M89	0.13861
P14079	Q8IZ13	0.8753
P14079	Q9UKV0	0.3861
P14079	O43813	0.45058
P14079	Q96DZ7	1.0346
P14079	O75832	0.75652
P14079	O94919	0.97046
P14079	Q96MI9	0.55521
P14079	Q9GZN2	0.55411

P14079	Q9HCH5	0.71118
P14079	Q5VIY5	0.99996
P14079	A6NCD4	0.90661
P14079	O43167	0.77928
P14079	M0R1G4	0.21851
P14079	P14866	0.54303
P14079	Q9BUB7	1.4714
P14079	P14868	1.2294
P14079	Q5T200	1.2419
P14079	Q8N726	0.80782
P14079	Q9Y266	1.364
P14079	Q6P1Q0	0.25792
P14079	Q86U28	0.4127
P14079	A8MZ59	0.24936
P14079	Q05516	0.99655
P14079	Q96QG7	0.40511
P14079	Q4LE39	0.73183
P14079	Q3KP66	1.7957
P14079	Q9HA82	0.68895
P14079	P23246	0.77175
P14079	Q8N8E3	0.69938
P14079	Q16540	0.37584
P14079	Q96KA5	1.0112
P14079	Q587I9	1.2345
P14079	Q8TAK5	0.5385
P14079	O75446	1.929
P14079	O95153	0.72374
P14079	P46783	1.4322
P14079	Q96LW4	0.35518
P14079	P46060	0.33495
P14079	Q86US8	0.46056
P14079	Q16143	0.35228
P14079	Q9HAP2	0.63552
P14079	Q9H9K5	0.73996
P14079	A6NC42	0.96687
P14079	Q12988	0.42367
P14079	Q9P2H0	0.88941
P14079	P07910	0.2461
P14079	Q32Q52	0.7102
P14079	Q9UI14	0.22227
P14079	P0CG23	0.45067
P14079	Q8NI08	0.9496
P14079	Q9UBU9	0.67562
P14079	Q5T3I0	0.20547
P14079	Q9Y5A6	0.82264
P14079	P14927	0.27613
P14079	O60287	1.6757
P14079	Q9UFW8	0.46096
P14079	Q9NPD3	0.13667
P14079	B4DYI2	0.49735
P14079	Q13310	0.44313
P14079	Q96KW2	1.2257
P14079	Q9NRN9	0.64839
P14079	Q8NBZ0	1.6903
P14079	Q9BYQ2	1.3698
P14079	O96004	0.41837
P14079	Q92748	1.6473
P14079	Q8IYL3	1.6012
P14079	Q96PG8	1.1715
P14079	Q86XG9	0.38249
P14079	Q9BXF9	0.33138
P14079	P22736	0.44583
P14079	A6NKD9	1.72
P14079	P52594	0.30341
P14079	Q8TCJ0	1.4931
P14079	P0CG20	0.8917
P14079	Q9HD42	0.98983
P14079	Q13838	0.082615
P14079	Q13573	0.62856
P14079	O75674	0.58471
P14079	Q9GZR2	0.048668
P14079	Q6Q759	1.4707
P14079	O60645	0.68687
P14079	Q8N328	0.76641
P14079	Q8NEG4	0.89733
P14079	Q08E93	1.1715

P14079	Q14D33	1.0632
P14079	Q9P1Z9	0.9124
P14079	P57075	0.36784
P14079	Q6L9T8	1.1532
P14079	Q8NI32	0.75465
P14079	Q8NFU3	0.23838
P14079	Q99856	0.73161
P14079	Q92800	0.41299
P14079	Q9H305	0.19119
P14079	E9PJ23	0.4201
P14079	Q14106	0.58466
P14079	O95067	0.80974
P14079	P78371	0.51582
P14079	Q08378	0.45708
P14079	Q96C23	0.46173
P14079	O60303	0.80894
P14079	Q8IZD9	0.21699
P14079	Q92624	1.3212
P14079	Q9NVM9	1.1999
P14079	O75208	1.1847
P14079	A4QN01	0.64657
P14079	Q76KX8	0.99887
P14079	A6H8Z2	0.8266
P14079	P05198	0.81886
P14079	Q6NZ36	0.28311
P14079	Q8NAB5	1.1715
P14079	Q9P2T0	1.356
P14079	A6NNW6	1.1882
P14079	Q12816	0.41253
P14079	O95922	0.12368
P14079	P31358	1.1139
P14079	Q9H840	0.88413
P14079	Q9NU23	1.0186
P14079	Q96D70	1.6528
P14079	Q9UBF9	0.32155
P14079	Q5SRE7	0.62533
P14079	Q8IZP0	0.42014
P14079	Q9BWE0	0.33127
P14079	P51606	0.5246
P14079	P61254	1.0605
P14079	P26371	1.1715
P14079	Q9NRI5	0.47052
P14079	O60613	0.96726
P14079	O95433	0.96673
P14079	Q6P582	1.3632
P14079	Q6ZUL3	1.1715
P14079	Q02447	0.66539
P14079	O43812	0.30975
P14079	O60268	0.79172
P14079	Q8WW01	0.99028
P14079	Q6K0P9	0.40896
P14079	Q6P9B9	1.3385
P14079	Q8NE86	0.79398
P14079	O94929	0.33346
P14079	Q5T8A7	1.3731
P14079	Q8N3I7	0.94544
P14079	Q9BSA9	1.2485
P14079	Q96EH3	0.76524
P14079	Q13105	0.60915
P14079	Q969P5	0.21276
P14079	O00213	0.37999
P14079	Q8NCQ7	0.75172
P14079	Q96MC4	1.558
P14079	Q7Z4S6	0.31989
P14079	Q8N9V7	1.5263
P14079	Q5MJ68	0.76554
P14079	Q9Y383	1.1069
P14079	Q15750	0.90695
P14079	Q9BUE6	0.20081
P14079	Q13394	0.66081
P14079	Q5JRK9	1.7294
P14079	Q9H503	0.77392
P14079	Q5SZB4	0.82206
P14079	Q8N9S9	0.4892
P14079	O15481	0.23019
P14079	P15976	0.30224

P14079	P08729	1.7444
P14079	Q9BSH4	1.7221
P14079	P50914	1.21
P14079	Q5T5D7	0.67256
P14079	P35232	0.68511
P14079	Q9NVH6	0.76017
P14079	Q9BWG6	1.1358
P14079	P60410	1.5617
P14079	Q9BWT1	1.6581
P14079	P10916	0.25533
P14079	Q8IYB0	1.1715
P14079	A0JLT2	1.5977
P14079	Q8TEE9	1.4836
P14079	Q6P1N0	0.67818
P14079	O60895	0.24754
P14079	Q3MIS6	0.98685
P14079	P68363	0.2946
P14079	Q9BYN7	0.38904
P14079	A6NGY3	0.82059
P14079	Q659C4	0.91879
P14079	Q5VWM3	0.12466
P14079	Q5SQI0	0.54911
P14079	P30084	0.15203
P14079	Q9BV73	0.99643
P14079	Q99442	0.907
P14079	Q14244	1.2344
P14079	Q6ZRS2	0.32378
P14079	Q9UIL1	0.95034
P14079	O43708	0.29226
P14079	Q9BRX8	0.40568
P14079	Q8NEL9	0.20189
P14079	O60346	0.30386
P14079	P06744	0.85028
P14079	Q96G61	0.56413
P14079	Q8TE69	0.56221
P14079	Q7Z2W9	0.43647
P14079	Q9BT22	1.2204
P14079	Q96KX1	0.24242
P14079	Q96GI7	1.3222
P14079	Q9NP85	0.20471
P14079	Q9HCU8	1.0744
P14079	Q96D46	1.4726
P14079	Q9P086	0.9883
P14079	Q9Y5L5	1.743
P14079	Q9C002	0.56818
P14079	Q8WWQ0	0.6998
P14079	Q9GZS3	1.0011
P14079	Q8NC26	0.93222
P14079	Q86YS3	0.23475
P14079	Q92785	0.77591
P14079	Q9H246	0.85015
P14079	Q96C11	0.44737
P14079	Q8IZL2	1.0038
P14079	Q86XK2	0.5155
P14079	Q9BXW3	1.1715
P14079	Q92636	0.50157
P14079	P59037	1.5616
P14079	Q9NY27	1.5413
P14079	Q6NVV0	1.3468
P14079	A1A5B4	1.1778
P14079	Q5T5J6	1.2751
P14079	Q9NR83	0.21799
P14079	A6NH11	1.2086
P14079	Q9NPC6	1.559
P14079	Q5SZJ8	0.47375
P14079	Q9Y5Q9	0.32842
P14079	Q8NG31	1.1993
P14079	G3V211	1.553
P14079	Q9P2J8	0.75812
P14079	P53805	0.58085
P14079	Q96Q80	0.51059
P14079	Q14739	0.28071
P14079	Q6P387	1.0782
P14079	A8MVW5	1.0122
P14079	Q9P0L1	0.70737
P14079	Q9UHV9	0.89957

43701	P14079	P35080	0.76298
43702	P14079	Q32MZ4	1.3755
43703	P14079	Q9C0I3	1.5139
43704	P14079	P19237	0.010469
43705	P14079	Q9BSK0	1.741
43706	P14079	Q5SZD4	0.97827
43707	P14079	Q9UPW5	0.45198
43708	P14079	Q9NRS6	0.11399
43709	P14079	P13804	0.32947
43710	P14079	Q6ZSV7	0.79071
43711	P14079	Q15059	1.0258
43712	P14079	Q86Y39	1.1345
43713	P14079	O14497	0.68722
43714	P14079	Q969X0	0.064522
43715	P14079	Q6ZUT1	1.1715
43716	P14079	P29144	0.19686
43717	P14079	Q96C86	0.77799
43718	P14079	P46199	0.88888
43719	P14079	Q9HCC9	0.6116
43720	P14079	Q6UWK7	1.1715
43721	P14079	Q9UBZ9	0.52647
43722	P14079	P10909	0.1271
43723	P14079	Q9BRB3	0.90835
43724	P14079	Q8TBZ9	1.6989
43725	P14079	Q9H0R3	1.1069
43726	P14079	Q14596	0.22452
43727	P14079	Q9UH64	0.53764
43728	P14079	A6NJY4	1.1715
43729	P14079	Q9UBC1	0.66379
43730	P14079	O75157	0.72881
43731	P14079	Q1W6H9	1.6505
43732	P14079	P56278	0.45678
43733	P14079	O94913	0.45689
43734	P14079	Q5T2P8	0.1776
43735	P14079	Q15050	1.0316
43736	P14079	Q9BWG4	0.22967
43737	P14079	Q8ND76	0.9309
43738	P14079	Q02221	0.73241
43739	P14079	Q9P2D3	1.0385
43740	P14079	Q6L8H4	1.1715
43741	P14079	Q15527	0.9502
43742	P14079	A5LHX3	0.65923
43743	P14079	O75569	0.34121
43744	P14079	Q5VT98	0.22339
43745	P14079	Q86SG2	0.28073
43746	P14079	O15116	0.83104
43747	P14079	Q96M63	0.46897
43748	P14079	Q9BY14	0.69311
43749	P14079	Q96MG8	0.21329
43750	P14079	P86791	0.80083
43751	P14079	Q9NS39	0.055104
43752	P14079	Q96LM9	0.32354
43753	P14079	Q8N344	0.71985
43754	P14079	P53992	1.1942
43755	P14079	A4D2H0	0.10259
43756	P14079	Q13439	1.3598
43757	P14079	Q8TCF1	0.51234
43758	P14079	O95639	0.60733
43759	P14079	Q9BUL8	0.85643
43760	P14079	P04844	0.97025
43761	P14079	Q9H2K2	0.28681
43762	P14079	Q5VUD6	1.2163
43763	P14079	Q96EB1	1.0525
43764	P14079	Q5TA79	1.1715
43765	P14079	Q9UKC9	0.64648
43766	P14079	Q14674	0.27943
43767	P14079	Q9Y303	0.17963
43768	P14079	Q86UQ8	0.63496
43769	P14079	Q5T699	0.61046
43770	P14079	Q96NL0	0.83201
43771	P14079	Q587J8	0.46134
43772	P14079	Q9Y615	0.89007
43773	P14079	Q9Y3F4	0.94613
43774	P14079	Q9ULW0	1.5087
43775	P14079	O60437	1.0513
43776	P14079	Q8WUH6	0.84704

P14079	Q9H8Q6	1.6831
P14079	P20810	0.99226
P14079	Q8NAV2	0.9261
P14079	Q9HAF1	0.98895
P14079	Q4VC44	1.297
P14079	Q6NTE8	0.36355
P14079	Q9UHA4	0.50155
P14079	Q5VY09	1.566
P14079	P69905	0.53063
P14079	Q9H074	0.51819
P14079	Q8N3F8	0.77086
P14079	Q9NY43	0.419
P14079	O60281	0.37848
P14079	Q8WXJ9	1.0423
P14079	P32519	1.1905
P14079	Q13112	1.3877
P14079	Q969E8	1.6789
P14079	Q9Y2J4	0.3248
P14079	Q13216	0.39771
P14079	Q96H12	1.6347
P14079	P62312	0.83144
P14079	Q6X4T0	0.70052
P14079	Q9Y3D0	1.1644
P14079	Q8NA70	0.55769
P14079	Q96CP2	0.65195
P14079	P0DKL9	1.5322
P14079	A1L168	1.3222
P14079	Q15029	0.38767
P14079	Q8N859	0.98344
P14079	P16455	0.67096
P14079	Q9Y5J7	0.4127
P14079	Q9H6F5	0.85408
P14079	Q59EK9	0.9842
P14079	Q13045	1.1348
P14079	Q13889	0.40699
P14079	P50990	0.071366
P14079	Q9Y4F9	0.34592
P14079	Q6UYE1	1.4032
P14079	Q8N1E6	0.40123
P14079	Q6NSI4	0.76853
P14079	Q8NHM5	0.27773
P14079	O95848	1.225
P14079	Q9UK11	0.78948
P14079	Q6ZV65	0.69358
P14079	O60504	0.8812
P14079	Q9BQ65	1.1766
P14079	P81408	1.2303
P14079	Q8N5I3	0.11285
P14079	P57679	0.79923
P14079	Q8N128	1.1715
P14079	Q9BXW9	1.0072
P14079	Q8N9M1	0.79582
P14079	P10746	0.62307
P14079	P16157	0.38856
P14079	Q8TAQ2	1.2834
P14079	Q9Y6Z2	1.1715
P14079	Q8N7M2	1.0057
P14079	Q8N7J2	1.6794
P14079	P53803	1.1396
P14079	O95905	0.95491
P14079	Q8WXF5	1.0104
P14079	Q96K83	0.61785
P14079	Q6IPU0	1.3681
P14079	O95954	0.67229
P14079	Q13472	0.087376
P14079	P19484	0.52387
P14079	Q13111	1.779
P14079	Q9GZU8	0.96939
P14079	Q6PG37	1.0311
P14079	Q7L5Y9	0.61353
P14079	Q8N883	0.52115
P14079	O60732	0.56221
P14079	Q12968	0.61307
P14079	Q03923	1.0696
P14079	Q9Y3A5	0.85961
P14079	Q15025	0.72057

P14079	Q96B97	0.55402
P14079	P62937	0.62826
P14079	Q9NV29	1.0526
P14079	Q6UWW9	1.0014
P14079	Q9BQM9	0.99732
P14079	Q8TF74	0.85926
P14079	P34897	0.26877
P14079	Q86SQ9	0.27371
P14079	A8MTA8	0.76595
P14079	Q9UJ41	0.38879
P14079	Q1A5X6	1.3222
P14079	O60759	0.58466
P14079	Q9NP98	1.3248
P14079	Q9UKX2	0.25261
P14079	Q53R41	0.29361
P14079	Q14847	0.32409
P14079	Q9NQP4	0.98366
P14079	Q9UJK0	1.6736
P14079	O00423	0.79677
P14079	Q15323	0.89111
P14079	Q8NCU4	1.0613
P14079	Q99747	0.82614
P14079	P41220	0.29412
P14079	Q9NVH2	1.0693
P14079	A6H8Y1	0.96775
P14079	Q9BZV1	1.0515
P14079	Q5TC12	0.81304
P14079	Q15124	0.13543
P14079	Q13427	0.47033
P14079	Q7Z7C7	0.78102
P14079	Q96MK2	0.64279
P14079	Q96NI8	0.92549
P14079	Q9Y2B2	1.6276
P14079	Q9NVR7	0.66419
P14079	Q5M9Q1	1.3529
P14079	Q8N0V3	1.5081
P14079	Q9H2P0	0.18683
P14079	Q6ZMS7	0.77037
P14079	Q9H1B4	0.22239
P14079	Q9NZU5	0.25726
P14079	Q8IYI8	0.766
P14079	Q14129	1.0354
P14079	O43900	0.30548
P14079	P62979	0.66459
P14079	O00399	0.28193
P14079	Q5VWP3	1.5594
P14079	Q06732	0.44092
P14079	Q9H900	0.65763
P14079	P46937	0.85047
P14079	Q5SVQ8	0.68319
P14079	Q75MW2	1.1965
P14079	Q10571	1.1224
P14079	O43829	0.83122
P14079	A8K8P3	1.1714
P14079	P18615	0.94191
P14079	Q9Y6J3	1.1715
P14079	Q5HYJ3	1.4627
P14079	Q5T089	0.40977
P14079	Q86YC2	0.82173
P14079	P60022	0.81621
P14079	A6NCW3	1.4194
P14079	Q9UGY1	0.49318
P14079	Q9GZP9	0.51059
P14079	O71037	0.70023
P14079	Q71RG6	1.1715
P14079	Q9H0A8	1.3776
P14079	Q96MR9	0.91481
P14079	Q9UIG4	0.98371
P14079	O14907	0.53101
P14079	Q6ZN55	0.52025
P14079	Q86UD1	0.73326
P14079	P60608	0.79007
P14079	Q56UQ5	0.045291
P14079	Q86YP4	0.73315
P14079	Q6ZNK6	1.6477
P14079	A6NHQ2	0.22433

P14079	Q9BYE3	1.1715
P14079	Q8TCD1	0.99803
P14079	O60869	0.51876
P14079	Q96HR8	1.6353
P14079	O00151	0.41523
P14079	F5GYI3	1.7166
P14079	Q96M29	1.0096
P14079	Q6P2D0	0.57947
P14079	Q96GS4	1.7191
P14079	A0PJX2	0.89539
P14079	O95182	0.84392
P14079	O43715	1.192
P14079	Q32P41	0.35438
P14079	Q32NB8	1.019
P14079	Q6XXX2	0.49812
P14079	Q01546	0.99979
P14079	Q96S99	0.23133
P14079	Q96G27	1.2303
P14079	Q86YL5	0.48973
P14079	A6ND36	0.27984
P14079	A6NKF2	0.81531
P14079	Q13625	0.50585
P14079	Q9UM54	0.098001
P14079	Q9Y2P7	0.61408
P14079	Q9Y3Y2	1.7668
P14079	Q86UK5	0.6607
P14079	Q8N1N5	0.59017
P14079	A6PVY3	0.94424
P14079	O00233	0.99093
P14079	Q15526	0.6834
P14079	Q9NUU6	0.95719
P14079	O94864	0.89436
P14079	O60784	0.63709
P14079	A5PLN7	1.3222
P14079	Q6ZMV5	0.94057
P14079	Q8N5N4	1.2303
P14079	Q4G0I0	0.77839
P14079	Q04917	0.45795
P14079	Q9P2R7	0.68729
P14079	Q9NUQ6	0.42364
P14079	Q8TAP6	1.3822
P14079	P41161	1.0851
P14079	Q6UXQ8	1.1715
P14079	Q96NL3	0.62309
P14079	O60934	0.26668
P14079	P60673	0.50523
P14079	Q96B18	0.47276
P14079	O75190	0.6652
P14079	Q5JS54	1.24
P14079	Q8IXX5	1.1391
P14079	Q8TDN6	0.39237
P14079	Q5T7P2	1.1715
P14079	Q6P2Q9	0.51943
P14079	P35527	0.99997
P14079	Q7Z2X7	1.2734
P14079	Q9UJW0	0.90456
P14079	Q12796	2.1537
P14079	C9J6K1	1.249
P14079	Q8TF46	0.22934
P14079	Q9NX53	0.56221
P14079	Q6ZMI0	0.47971
P14079	Q96JJ3	0.55099
P14079	Q8NFI3	0.94184
P14079	Q9BSD3	0.82042
P14079	Q969I3	0.80477
P14079	Q86XL3	0.40214
P14079	P49918	0.76867
P14079	Q9Y3Q8	1.1047
P14079	Q9BXU2	1.2057
P14079	Q6P2E9	0.96279
P14079	P62841	0.96768
P14079	Q96E29	0.99002
P14079	P31350	0.36248
P14079	Q9H213	0.12669
P14079	Q5RHP9	0.89479
P14079	Q92597	0.70112

P14079	Q8IUD2	0.976
P14079	Q76KD6	0.88636
P14079	Q3LI59	1.1715
P14079	Q8N4J0	1.7729
P14079	O94777	1.0624
P14079	P18124	0.87813
P14079	Q8NCE0	0.96602
P14079	O15527	0.52768
P14079	Q96L92	0.92912
P14079	P61758	0.71861
P14079	Q7RTX8	0.045609
P14079	B4DJY2	0.80886
P14079	Q3SXZ3	0.64025
P14079	Q96RM1	1.1715
P14079	Q15003	1.3818
P14079	A6NN73	0.94914
P14079	Q9UPT5	0.76117
P14079	Q7Z7F0	0.13988
P14079	Q14444	0.38828
P14079	Q8TC20	0.10229
P14079	Q8NEV9	0.84969
P14079	P63165	0.97935
P14079	P15559	0.43313
P14079	Q5R3K3	0.84874
P14079	Q8NE22	1.7829
P14079	Q8NEF3	0.71138
P14079	Q99828	0.36198
P14079	Q76N32	0.45191
P14079	P62380	0.38349
P14079	Q9H3C7	1.7286
P14079	Q6XCG6	1.1715
P14079	Q9UHI6	0.39976
P14079	Q9H706	0.56221
P14079	Q9BTE3	0.74284
P14079	Q96MT4	1.4367
P14079	P57738	1.1823
P14079	Q8N6T0	1.0901
P14079	Q14240	0.17377
P14079	Q96A49	1.4058
P14079	F5H284	0.18124
P14079	Q8N6F8	0.080393
P14079	Q86YA3	1.6718
P14079	A6NGD5	0.53786
P14079	Q5MJ10	1.5871
P14079	Q9Y448	1.0095
P14079	O00115	0.72547
P14079	Q7Z6J8	1.1093
P14079	Q569H4	1.3222
P14079	Q7Z591	1.9627
P14079	Q9H9H5	1.2353
P14079	Q8N9R6	0.97965
P14079	Q7Z4G1	2.0433
P14079	P0C7Q5	1.2643
P14079	O43379	0.3613
P14079	Q9Y371	0.228
P14079	Q9BVC4	0.61906
P14079	P35998	0.63119
P14079	O60711	0.53235
P14079	Q14118	0.25133
P14079	Q96SZ4	0.44187
P14079	Q96RE9	0.53783
P14079	P07919	0.68913
P14079	Q8TEA1	0.54929
P14079	Q5T319	1.1715
P14079	A5YKK6	1.4411
P14079	Q7Z3C6	0.65483
P14079	Q9P217	0.55614
P14079	P78549	0.34831
P14079	Q9NZL9	0.10783
P14079	Q9BQA5	0.93959
P14079	Q9H147	1.2474
P14079	Q8WY91	1.0053
P14079	Q96NG5	0.96114
P14079	Q9H0C3	1.218
P14079	Q14159	1.9029
P14079	Q6UW78	0.98371

P14079	Q8NA31	0.87435
P14079	Q9H3E2	0.46526
P14079	O15155	0.35634
P14079	Q9P215	0.61985
P14079	Q7L3S4	0.51366
P14079	O75461	0.18838
P14079	P31271	0.14603
P14079	P56211	0.75629
P14079	Q9UBW7	1.3567
P14079	Q6ZMK1	0.17701
P14079	A6NHR8	1.1715
P14079	P15923	0.58496
P14079	Q8WWC4	1.2532
P14079	Q15306	0.60977
P14079	Q9NR56	0.54694
P14079	Q8NAS9	0.6576
P14079	Q96M61	0.56221
P14079	Q9NS37	0.78193
P14079	Q14409	0.88589
P14079	K7EJ46	1.1715
P14079	Q9NRX5	0.74001
P14079	Q9Y4H4	1.1527
P14079	Q9BTE0	0.43728
P14079	O75352	1.2722
P14079	Q0P670	1.7191
P14079	Q9HAU4	0.23799
P14079	P48443	0.25311
P14079	Q9BVV6	1.1715
P14079	Q9UHK0	0.52879
P14079	Q13424	0.70828
P14079	Q96HT8	2.1833
P14079	O60870	0.95651
P14079	O43830	0.53511
P14079	P62314	0.8634
P14079	Q9HD64	0.9953
P14079	Q96LX8	0.66944
P14079	Q8IYY4	0.27964
P14079	P54253	0.90763
P14079	Q69YQ0	0.54795
P14079	P14324	0.24032
P14079	Q6UX82	0.91704
P14079	Q13156	0.61476
P14079	Q8IWN6	1.1275
P14079	O94905	0.60901
P14079	P11166	0.37256
P14079	Q14498	0.7153
P14079	Q9NX94	1.0941
P14079	P05413	0.39416
P14079	Q9BYU5	1.1584
P14079	O95707	1.4609
P14079	Q9ULM3	0.93081
P14079	Q496H8	1.12
P14079	Q9HCS7	0.44872
P14079	Q9H9A7	0.69474
P14079	Q9BYR7	1.5936
P14079	P42766	1.0052
P14079	Q9Y4G6	0.44373
P14079	Q06547	1.0213
P14079	Q5VYK3	0.48581
P14079	Q8TDX5	0.43148
P14079	Q9Y2T3	0.53879
P14079	Q5BJF2	1.2205
P14079	Q9C0H9	0.61126
P14079	Q9NUQ3	0.56526
P14079	Q86YG4	0.97848
P14079	Q14258	0.76727
P14079	Q8N7G0	0.28169
P14079	Q15797	0.5408
P14079	Q96HJ3	1.3222
P14079	Q9BQI6	0.33298
P14079	Q6ZRR5	0.89497
P14079	O75608	0.10553
P14079	Q8WW18	1.3222
P14079	P06733	0.56239
P14079	Q16600	0.55492
P14079	Q9H299	0.50824

P14079	Q96BJ3	1.1899
P14079	O43298	0.1949
P14079	P79522	0.91702
P14079	P30793	0.22948
P14079	Q6YHU6	0.25993
P14079	Q9H9Q2	0.29546
P14079	P02489	0.36722
P14079	Q8TBC5	0.24971
P14079	Q969V6	1.4144
P14079	Q15427	0.321
P14079	P33316	0.65589
P14079	O60678	0.23621
P14079	Q96NT0	0.25118
P14079	Q13065	0.5777
P14079	Q03933	0.51906
P14079	Q9NWA0	2.057
P14079	P61077	0.61533
P14079	Q6ZRV3	1.1715
P14079	Q5TA82	1.1715
P14079	Q08211	0.26295
P14079	P25685	0.25982
P14079	Q14765	0.30746
P14079	Q7RTT4	0.31443
P14079	O75155	0.89589
P14079	Q9H9Z2	0.79628
P14079	A5YM69	0.7896
P14079	Q5TAP6	1.1972
P14079	Q9Y2F5	0.88463
P14079	Q04695	1.0011
P14079	Q96BN2	1.3084
P14079	Q8N326	0.88101
P14079	Q16385	0.93972
P14079	Q96DZ1	0.70787
P14079	Q9NYW8	0.5383
P14079	Q9NU63	0.52862
P14079	Q6IE81	1.0678
P14079	Q969N2	0.70065
P14079	P10827	0.24351
P14079	Q9HBV2	1.2485
P14079	Q9H175	1.0756
P14079	Q5T1Q4	0.84328
P14079	Q12837	0.64088
P14079	Q4G0F5	0.48942
P14079	Q9UHX1	0.66392
P14079	Q9UNZ5	1.3222
P14079	Q8NCT3	1.5108
P14079	Q9Y6H1	1.571
P14079	A8MUI8	1.2005
P14079	P57081	1.1625
P14079	Q96NT5	0.67651
P14079	Q9UKK3	0.78568
P14079	O95822	0.4559
P14079	Q96AH0	0.95705
P14079	P62952	1.1848
P14079	F2Z3M2	1.1715
P14079	Q5SNT2	0.92098
P14079	P61289	1.1936
P14079	A6NFF2	0.47338
P14079	Q6PD62	0.61691
P14079	O95453	0.77496
P14079	Q96JM2	0.39971
P14079	Q9HB03	0.77014
P14079	Q96JC4	0.70617
P14079	O14925	0.64638
P14079	Q96N23	1.3057
P14079	Q5H9R4	1.3529
P14079	Q8IY81	0.83501
P14079	Q3MHD2	0.82906
P14079	I3L1E1	0.84303
P14079	Q6ZN06	0.99755
P14079	A2RUB1	1.3222
P14079	Q6ISB3	0.61927
P14079	Q0VD83	1.004
P14079	P02008	0.64778
P14079	Q9BRQ5	0.42266
P14079	Q96AY2	1.2341

P14079	Q9NS69	0.44141
P14079	Q9NWS0	0.70909
P14079	P23025	0.73853
P14079	A8MQT2	0.1307
P14079	O75143	1.7072
P14079	P63151	0.53142
P14079	P29084	1.6054
P14079	Q49A92	0.71812
P14079	P0C7Q6	1.2643
P14079	A6NGN4	0.46494
P14079	A6NFC5	1.2954
P14079	Q7Z465	0.93837
P14079	Q3SYF9	1.1715
P14079	Q8N8A8	1.1755
P14079	Q8N5S3	0.37841
P14079	P0CJ77	1.3031
P14079	Q9H1Y0	0.73969
P14079	Q03989	0.39249
P14079	Q8IVB5	0.48967
P14079	Q86UN6	0.69432
P14079	P23497	1.0002
P14079	P62266	1.1503
P14079	Q9BVV2	0.52084
P14079	P0C7V9	1.5082
P14079	Q9Y4R7	0.31229
P14079	P14649	0.34468
P14079	Q16740	0.84796
P14079	Q9BYP9	1.3698
P14079	Q96BJ8	0.10685
P14079	Q5JSS6	0.98355
P14079	P30304	0.83486
P14079	Q6NUR6	1.3372
P14079	Q86TJ5	0.55217
P14079	Q9C093	0.36672
P14079	Q9NQZ5	0.57079
P14079	Q6P2C0	0.86568
P14079	Q99608	0.34667
P14079	Q5VVM6	0.20167
P14079	P61956	0.84136
P14079	P52744	0.71839
P14079	P57071	0.31932
P14079	Q8IWY8	0.51176
P14079	Q6ZU80	0.39441
P14079	Q14249	0.22309
P14079	Q9NQ90	1.0151
P14079	Q5QFB9	1.0092
P14079	Q05048	0.86149
P14079	Q15652	0.44538
P14079	Q9NQZ8	0.5751
P14079	Q9Y2R5	0.94541
P14079	P60006	1.2213
P14079	Q5T8R8	0.41994
P14079	Q9UI72	1.1715
P14079	Q8NA19	0.23227
P14079	P61550	0.72581
P14079	Q3KQV3	0.64419
P14079	Q9NQ94	0.28459
P14079	Q9H2X8	1.0754
P14079	Q6ZP98	1.0541
P14079	Q96LV5	0.39499
P14079	Q96HA8	1.5033
P14079	P59942	1.2711
P14079	Q8TF40	1.3397
P14079	Q9H307	1.4123
P14079	P17027	0.51784
P14079	Q6ZT52	0.049457
P14079	O95149	0.81211
P14079	Q9NRQ5	1.1715
P14079	A6NGQ2	1.0186
P14079	Q8N1A0	0.69249
P14079	P51610	0.5922
P14079	Q7Z3E2	1.1859
P14079	Q6EKJ0	0.26649
P14079	A8MY62	0.27841
P14079	Q9P2W6	1.1715
P14079	P54845	0.41009

P14079	Q9UL16	0.56417
P14079	Q9NPI7	0.76351
P14079	Q92890	0.83702
P14079	Q5T5F5	1.1715
P14079	O14683	1.1295
P14079	Q3LI66	1.1715
P14079	Q7Z4B0	1.1715
P14079	Q92539	0.436
P14079	P29692	0.58882
P14079	O15371	0.81333
P14079	Q96N53	1.1715
P14079	Q9NYA3	0.72864
P14079	P0CW20	0.54513
P14079	Q9UN81	1.0571
P14079	Q9HCI5	0.69862
P14079	Q7L8L6	0.72738
P14079	Q9BUZ4	0.27148
P14079	Q13136	0.45245
P14079	O15350	0.31808
P14079	P46782	0.8451
P14079	Q96HQ2	1.6816
P14079	Q8IWN7	0.48356
P14079	Q00839	0.27353
P14079	Q71RC9	1.1823
P14079	Q9BY77	0.34837
P14079	Q5XG99	0.93632
P14079	Q8TB72	0.70579
P14079	P62877	0.52443
P14079	Q6PJG2	0.83796
P14079	Q4J6C6	0.54478
P14079	Q15744	0.27386
P14079	Q3Y452	1.1715
P14079	Q9Y2H0	0.22171
P14079	Q96JX3	0.62386
P14079	Q9BXH1	0.54203
P14079	P23327	1.0913
P14079	Q9NWN3	0.86464
P14079	P09629	0.73332
P14079	O94892	0.5223
P14079	Q9NVF7	1.3798
P14079	P62987	1.0384
P14079	O00501	0.57185
P14079	Q16576	0.25211
P14079	Q15032	0.73413
P14079	B9EJG8	0.91469
P14079	Q9H665	1.1206
P14079	Q96JE7	1.3153
P14079	P07196	0.97456
P14079	Q96JG8	0.043255
P14079	Q6L8Q7	0.49212
P14079	Q9Y4P1	0.8741
P14079	Q719H9	0.55294
P14079	Q8IVV7	0.74153
P14079	Q6ZRT6	1.3222
P14079	Q3SYA9	0.86812
P14079	Q03252	0.51891
P14079	Q9P0V3	0.43707
P14079	Q9UIL8	0.37434
P14079	Q5TAA0	0.14963
P14079	Q99612	0.31129
P14079	P22087	0.66443
P14079	Q9BXS6	1.2375
P14079	P42568	0.90662
P14079	P02533	1.0536
P14079	Q8WXU2	0.85597
P14079	Q9H6V9	1.0786
P14079	Q8TCH9	1.1515
P14079	Q96N16	1.3344
P14079	Q6ZSS7	0.86195
P14079	Q16670	0.53459
P14079	Q8N1F1	1.1715
P14079	Q5T870	1.4015
P14079	O94903	1.0085
P14079	Q5JNZ5	0.79675
P14079	Q9BYD5	0.23156
P14079	Q8WTX7	1.2077

P14079	P58511	1.2485
P14079	Q15072	0.57676
P14079	O60315	0.26707
P14079	Q6N021	0.94383
P14079	Q9BTE7	0.98667
P14079	Q9H1A7	0.23838
P14079	Q8IUR0	1.5031
P14079	Q6XLA1	0.32486
P14079	Q9H5N1	1.0409
P14079	Q15572	1.3255
P14079	O43290	0.89931
P14079	P50552	0.27886
P14079	Q9H3K6	0.98123
P14079	Q96L16	0.13422
P14079	Q8IUG1	1.1644
P14079	Q13077	0.62358
P14079	Q8N6M5	0.78164
P14079	Q9H269	0.59263
P14079	Q5FBB7	1.2398
P14079	Q9HCM9	0.36754
P14079	Q0VGL1	0.4952
P14079	P46939	0.89555
P14079	Q6PIF2	0.37075
P14079	Q9NYU1	0.43806
P14079	P33947	0.66737
P14079	P55197	1.1806
P14079	Q9H410	0.96888
P14079	Q7Z4Y8	0.38719
P14079	Q7LG56	0.12279
P14079	Q9BVJ6	0.91584
P14079	Q9NPD7	1.089
P14079	Q8TAP8	1.0838
P14079	Q9Y4I5	0.69139
P14079	Q9HA90	1.3222
P14079	H0Y7S4	0.37503
P14079	P61604	1.0876
P14079	Q96S65	1.6551
P14079	Q8WU49	1.1715
P14079	Q96GD0	0.13011
P14079	P55212	0.54133
P14079	A6NIN4	1.1715
P14079	Q9BTA0	1.3222
P14079	Q49AA0	0.94973
P14079	Q6PJG6	1.2253
P14079	Q9P2X0	0.50934
P14079	Q8TAP4	0.18002
P14079	Q9BQ50	0.4146
P14079	O60783	1.112
P14079	Q92572	0.63271
P14079	Q9H5X1	0.38202
P14079	O00178	0.83011
P14079	A2RUG3	1.418
P14079	Q96NR7	0.40403
P14079	Q9HA77	1.1015
P14079	P07738	0.89643
P14079	Q96LK0	0.39441
P14079	Q7L266	0.67908
P14079	P36402	0.44564
P14079	Q03938	0.98563
P14079	P06280	0.3167
P14079	Q96MN9	0.72987
P14079	Q8IXR9	0.55044
P14079	Q5VTQ0	0.67352
P14079	Q12873	0.50601
P14079	P40121	0.53166
P14079	Q96FA3	0.8877
P14079	Q03111	1.1707
P14079	Q5TBK1	0.035613
P14079	A1L020	0.28856
P14079	Q8TF32	1.0501
P14079	A6NDY2	0.59627
P14079	Q7Z434	0.41064
P14079	P11308	0.42552
P14079	Q9Y6Z5	1.441
P14079	Q8NB25	0.3504
P14079	Q6ZUT4	1.2318

P14079	Q9BV97	0.67917
P14079	Q8IWV7	0.72796
P14079	Q69YN2	0.64603
P14079	O14513	0.93274
P14079	Q9Y613	0.86683
P14079	Q9HCE5	0.43344
P14079	Q9BSB4	0.68557
P14079	Q9GZS0	0.25984
P14079	Q13144	0.50055
P14079	A1XBS5	0.074633
P14079	Q9H5I1	0.55804
P14079	P09913	0.75749
P14079	P55273	0.2963
P14079	Q86VV8	0.78529
P14079	Q96NS8	0.73949
P14079	Q9NZ52	0.86788
P14079	Q96EF6	0.92113
P14079	Q9UHQ7	1.1915
P14079	B3EWG3	1.3222
P14079	O95059	1.0077
P14079	Q5T753	1.1715
P14079	Q96KP1	0.49194
P14079	Q5SQN1	0.48855
P14079	O95718	0.23819
P14079	P50570	0.36503
P14079	Q14CZ7	0.40762
P14079	Q49AR2	0.98686
P14079	Q9UF72	1.3258
P14079	Q14588	0.77845
P14079	A6NP11	1.0501
P14079	Q8WWY3	0.40412
P14079	Q86WH2	0.47946
P14079	Q9BUA3	1.2541
P14079	P0DMB1	1.3036
P14079	B9ZVM9	1.4988
P14079	Q9NPJ3	0.30142
P14079	Q7L9B9	0.60572
P14079	Q96FV9	0.82757
P14079	O96005	0.98364
P14079	P62241	0.8316
P14079	P83369	0.95787
P14079	Q6MZN7	1.1715
P14079	A4GXA9	1.2907
P14079	Q8NEA5	1.0212
P14079	P51665	0.78963
P14079	P55822	0.95323
P14079	O14949	0.31603
P14079	Q01201	0.96113
P14079	Q9Y2S6	1.1715
P14079	Q9H6R7	0.48409
P14079	Q9BVV7	0.49594
P14079	Q4G0P3	0.24567
P14079	Q16613	0.44581
P14079	Q14667	0.91315
P14079	Q6UXB3	0.79381
P14079	Q9BXM8	1.1715
P14079	O15037	1.0666
P14079	Q9HB19	0.78288
P14079	Q5TI25	1.9823
P14079	Q99618	1.6305
P14079	Q5TD94	0.76333
P14079	P0CW71	1.1715
P14079	P41236	1.0885
P14079	Q5T681	1.5158
P14079	Q6NW29	0.79179
P14079	Q9NP97	0.97526
P14079	Q15459	0.50818
P14079	Q9BZL4	0.72622
P14079	Q9H6J7	0.91838
P14079	A6NDB9	1.385
P14079	Q8N960	0.32405
P14079	O60477	0.6465
P14079	Q86V71	0.8029
P14079	O00461	1.2045
P14079	Q9UKX7	1.2856
P14079	Q3KNS6	0.53219

P14079	Q5T4H9	1.1715
P14079	Q9BVI4	0.81672
P14079	Q8N2N9	1.0803
P14079	Q8WVY7	0.27553
P14079	O95671	1.0293
P14079	P07195	0.60641
P14079	Q13541	0.91488
P14079	Q9UHB7	1.1536
P14079	O75167	0.47647
P14079	P0C2Y1	0.49952
P14079	Q9NR09	0.37622
P14079	P02686	0.91228
P14079	Q8IYD1	0.18892
P14079	Q9Y592	1.5792
P14079	Q8N5W8	0.98371
P14079	P35611	0.21245
P14079	Q6FI81	0.53098
P14079	P20618	1.2175
P14079	A6PVL3	0.92959
P14079	Q15363	0.17564
P14079	P78344	0.59165
P14079	Q9NTI5	0.94266
P14079	Q8WU43	0.73407
P14079	O00567	0.88056
P14079	P14854	0.42713
P14079	Q15388	0.54628
P14079	Q9NWD9	0.94672
P14079	Q00987	0.29204
P14079	Q6BEB4	0.22313
P14079	P23588	0.42521
P14079	Q9BUP3	0.38528
P14079	Q5SRH9	1.7006
P14079	P45984	0.35311
P14079	P0CF97	1.273
P14079	Q86XI6	0.35396
P14079	P55198	0.9649
P14079	Q8N292	0.95817
P14079	Q9NPR9	1.7731
P14079	Q9H841	1.288
P14079	Q9UKI2	0.47727
P14079	Q92797	0.98743
P14079	Q52LJ0	0.98433
P14079	O75843	0.96568
P14079	Q9Y421	0.70864
P14079	O75069	0.59555
P14079	Q96RY5	1.5217
P14079	Q68DQ2	0.2639
P14079	Q6ZUS5	0.040449
P14079	P0C7W9	0.13295
P14079	Q6ZUG5	0.95453
P14079	O95198	0.83219
P14079	Q2TAK8	0.806
P14079	Q9BSU3	0.45791
P14079	A4D1E1	1.2076
P14079	Q8TBB1	0.513
P14079	P25815	0.62908
P14079	Q2LD37	0.77064
P14079	Q9BVT8	1.7428
P14079	O95394	1.1793
P14079	Q9Y3D3	1.0485
P14079	Q9BUN8	0.57935
P14079	Q96I34	0.28276
P14079	Q96FH0	1.1631
P14079	Q9BZE4	0.29449
P14079	P32969	1.0531
P14079	Q5HYR2	1.6896
P14079	Q6NXR4	0.80597
P14079	Q5I0X4	1.3222
P14079	P0CL83	0.91322
P14079	Q8NHU0	0.62582
P14079	P63104	1.0496
P14079	Q96ND8	1.0454
P14079	Q3B7T1	1.4937
P14079	A8MQ11	0.5869
P14079	Q6NVH7	1.2735
P14079	Q5VYS4	0.48941

P14079	Q9Y3B1	0.36724
P14079	Q9NRF8	0.81536
P14079	P08651	0.53484
P14079	Q2TAY7	0.28036
P14079	Q9NS71	0.86894
P14079	Q6ZNE5	0.5363
P14079	O75558	0.87411
P14079	Q9BV68	0.47474
P14079	Q96RT1	0.41236
P14079	Q9NYF8	1.1071
P14079	P22090	0.55529
P14079	Q9H808	0.67196
P14079	P46100	0.62901
P14079	Q9NVD3	0.11081
P14079	P62306	0.32606
P14079	Q9UHJ9	1.1646
P14079	Q15012	1.1547
P14079	Q9H813	1.0447
P14079	Q9NWS9	0.24097
P14079	O00534	0.7489
P14079	Q86U44	0.23701
P14079	Q9Y244	0.84802
P14079	Q6NSW5	1.5252
P14079	Q8N4M7	1.5903
P14079	Q8IZ73	0.51974
P14079	Q9P0M9	1.1844
P14079	Q9ULW8	1.052
P14079	Q9BPW8	1.1413
P14079	O95156	1.1843
P14079	Q96HH6	1.2485
P14079	P0CI00	1.1722
P14079	Q5JPE7	0.72609
P14079	Q5SZL2	0.56972
P14079	Q9BRR0	0.5625
P14079	Q08AG7	1.0522
P14079	P51814	0.45498
P14079	O43776	1.0409
P14079	Q7RTU9	0.52936
P14079	P43243	0.30287
P14079	Q9NVI1	1.1205
P14079	A8MUZ8	1.0642
P14079	A2RU49	0.64761
P14079	Q6ZW35	0.596
P14079	Q15061	0.27853
P14079	Q96EI5	1.1389
P14079	Q9NPF4	0.71304
P14079	Q52M58	1.4283
P14079	Q8N104	0.91101
P14079	Q15078	0.1434
P14079	A8K010	0.92514
P14079	P28698	0.81871
P14079	Q5JPH6	1.063
P14079	Q9BRJ7	1.3311
P14079	P62829	1.4392
P14079	O94763	1.1768
P14079	Q9NYG5	0.95074
P14079	Q9UJ55	0.30704
P14079	O75396	0.50227
P14079	Q96GU1	0.89414
P14079	Q99614	0.40602
P14079	Q8TF68	0.25526
P14079	Q5T749	1.4932
P14079	Q5VWI1	1.3201
P14079	Q4G0S7	0.12109
P14079	Q96GE5	0.95434
P14079	P35813	0.29917
P14079	P28069	0.60944
P14079	O75448	0.72924
P14079	Q9BVL2	0.72857
P14079	Q6ZSJ9	1.0234
P14079	Q6PI98	1.0226
P14079	Q96FJ8	1.1715
P14079	Q96BV0	0.51144
P14079	O75431	0.050619
P14079	P51825	0.9706
P14079	Q9NRR5	0.71397

P14079	P14678	0.84518
P14079	Q8IWZ6	0.19332
P14079	O15061	1.2156
P14079	Q8NDY4	1.1715
P14079	Q9Y2V7	0.20729
P14079	Q9BWD3	1.7375
P14079	Q7Z7G8	0.28765
P14079	Q9NNX9	1.5627
P14079	P10911	0.27185
P14079	Q9Y2J8	1.2762
P14079	O60573	0.69872
P14079	Q96MB7	1.1449
P14079	B5ME19	0.50682
P14079	Q8TEW0	0.37859
P14079	O96007	1.4477
P14079	Q12772	0.26867
P14079	Q9NTU4	1.2888
P14079	O75506	0.15891
P14079	O95073	1.0823
P14079	Q9Y6P5	0.90727
P14079	Q17RY6	1.484
P14079	Q9UL58	1.1491
P14079	P60369	1.3698
P14079	A8MX80	1.1299
P14079	Q13769	0.6592
P14079	Q8TAA3	0.91638
P14079	P0C671	0.44627
P14079	O15145	0.4636
P14079	Q9HBL0	0.82262
P14079	Q9UKY1	0.39442
P14079	P31276	0.4231
P14079	Q92990	0.84171
P14079	Q8N815	0.13928
P14079	Q9H0E9	0.11285
P14079	Q5W0B7	1.1542
P14079	P55081	1.1105
P14079	P53673	0.94815
P14079	Q16204	0.32856
P14079	B2RV13	1.2612
P14079	P0CJ90	0.25626
P14079	P33552	0.77421
P14079	Q96PZ0	0.12922
P14079	Q86UB2	1.2809
P14079	Q14061	1.0165
P14079	Q96EL1	1.2694
P14079	Q9H488	0.22705
P14079	Q8IXW5	1.0306
P14079	Q99687	0.59222
P14079	A6NDP7	0.94614
P14079	P0C7X2	1.0371
P14079	O95400	1.1922
P14079	Q8TBR5	0.58032
P14079	Q14149	0.25848
P14079	P62256	0.53416
P14079	P24390	0.9834
P14079	Q8WUX2	0.36883
P14079	P60866	1.0559
P14079	Q5TYX0	0.68944
P14079	Q9BRP1	1.2971
P14079	Q5TA50	0.84036
P14079	Q8N7H1	0.53011
P14079	Q8IYB3	1.2715
P14079	Q12905	0.96198
P14079	P43356	1.031
P14079	Q8TCN5	0.1949
P14079	Q9BUR4	0.88017
P14079	Q3B7I2	0.1824
P14079	C9JFL3	1.1715
P14079	Q8WY22	1.4889
P14079	Q5HY64	0.52163
P14079	Q96JF6	0.52044
P14079	Q9BV19	1.1882
P14079	Q8N0X4	0.54037
P14079	Q96DG6	0.43529
P14079	P43897	1.8224
P14079	Q96RP9	1.0527

P14079	Q9ULD5	0.47863
P14079	O94915	0.26737
P14079	Q9UC07	0.68484
P14079	O75648	1.1759
P14079	Q9ULR0	1.3405
P14079	Q8NBB4	0.60394
P14079	Q6W2J9	0.27232
P14079	Q96HH4	1.3522
P14079	Q9H2G9	0.41667
P14079	P45973	0.99593
P14079	Q6NT76	0.40548
P14079	Q86TC9	0.48381
P14079	Q3SX64	0.09154
P14079	Q9Y3E1	0.37664
P14079	P60370	1.3698
P14079	Q9H9L7	0.58072
P14079	Q6ZVU0	0.55869
P14079	Q3I5F7	0.12114
P14079	A6NHU5	0.85652
P14079	Q9NWK9	1.255
P14079	Q6P1X5	0.98042
P14079	A6NCS6	1.2758
P14079	A8K830	1.1715
P14079	Q12802	0.269
P14079	Q9HD87	0.84093
P14079	Q502X0	0.40977
P14079	Q9NQX5	0.92489
P14079	P19338	0.7119
P14079	Q3SY46	1.2482
P14079	Q96M02	0.53371
P14079	P42226	0.76161
P14079	A6NIE6	0.85556
P14079	Q6X4W1	1.8433
P14079	Q9C075	0.80279
P14079	Q9P1C3	1.1899
P14079	Q06330	0.54534
P14079	O15504	0.59803
P14079	Q9BUH6	1.2831
P14079	Q14695	1.2555
P14079	B4DS77	0.97599
P14079	Q96T59	1.0613
P14079	Q9BR46	1.448
P14079	A4D2B0	1.3155
P14079	Q9UBL0	0.55969
P14079	Q9UIC8	0.50935
P14079	P0C7P2	0.90286
P14079	Q6V9R5	0.987
P14079	Q9UL01	1.0608
P14079	Q6ZVF9	1.2818
P14079	K7EIZ4	1.1715
P14079	Q9ULL5	1.1715
P14079	Q8WU90	0.95226
P14079	P0C7M3	1.0613
P14079	Q69YL0	1.1715
P14079	O00186	0.7869
P14079	Q8WTZ4	0.12175
P14079	O75323	1.0629
P14079	A8K0R7	1.2883
P14079	P80303	0.19054
P14079	Q96NR2	0.688
P14079	Q9HD26	1.0965
P14079	Q6NZY7	0.53442
P14079	A6NJ78	1.5082
P14079	Q9NWZ8	0.80912
P14079	Q9H1Q7	1.3583
P14079	Q9H8M9	1.169
P14079	Q15149	0.39369
P14079	Q8NHS1	1.0004
P14079	Q8N4L1	1.2485
P14079	Q8N3S3	0.39438
P14079	Q0VDF9	0.34958
P14079	Q9H095	0.7243
P14079	O43427	0.77608
P14079	Q9NRL3	0.60903
P14079	Q6ZS46	1.5495
P14079	Q96IK1	0.38817

P14079	O75503	0.60263
P14079	O15511	0.53121
P14079	P14136	0.94033
P14079	O43148	1.1556
P14079	Q96DE9	0.41522
P14079	Q53FD0	1.5129
P14079	Q8N1G1	0.32911
P14079	Q9BQ61	1.3222
P14079	Q8N3L3	1.2496
P14079	P0C025	1.3144
P14079	Q9H9E1	0.36204
P14079	P82094	1.3476
P14079	P45983	0.24729
P14079	Q99741	0.72973
P14079	Q8N0W7	1.0639
P14079	P20674	0.34266
P14079	Q9NUP9	0.49899
P14079	Q9UK73	0.7431
P14079	Q8NAE3	1.1394
P14079	Q9BW61	1.1259
P14079	Q9HB71	0.62657
P14079	P82970	1.0915
P14079	Q9NV12	1.2485
P14079	Q6ICG6	0.83419
P14079	Q3V5L5	0.85429
P14079	Q96KD3	1.8805
P14079	Q8TAW3	0.62703
P14079	Q96NT1	1.3882
P14079	Q92670	0.57881
P14079	P68032	0.99952
P14079	Q8N6I4	1.2485
P14079	Q96PP4	0.92434
P14079	H3BSY2	0.07102
P14079	Q8IXT1	0.77095
P14079	Q9NPC3	0.021896
P14079	O15066	0.33973
P14079	Q8N3U4	0.89599
P14079	Q16629	0.67266
P14079	P50613	0.24652
P14079	Q8IZY5	0.51993
P14079	Q9H6W3	0.7174
P14079	Q5VYP0	1.3668
P14079	Q86UU5	1.2973
P14079	Q9NZC4	0.39793
P14079	Q9H2U2	0.3195
P14079	Q86VX2	1.5462
P14079	Q9Y2T7	0.31313
P14079	A6NN90	0.87359
P14079	Q9H8Y8	0.62123
P14079	Q9H0N5	1.1395
P14079	Q9H981	0.9744
P14079	Q5VUE5	1.2887
P14079	Q99865	1.3384
P14079	Q9H6R4	0.82647
P14079	Q9P2D1	0.15498
P14079	Q5H9J9	1.0898
P14079	Q5T752	1.1715
P14079	Q1W209	1.1715
P14079	P68871	0.76311
P14079	O14633	1.1715
P14079	A6NDZ8	0.44102
P14079	Q8TD26	0.10076
P14079	O95721	0.43633
P14079	P62854	1.1257
P14079	Q8TDY3	0.96178
P14079	Q6PUV4	0.71466
P14079	Q9NZI7	0.16767
P14079	Q8IVJ8	1.2831
P14079	Q8WVZ9	0.36435
P14079	P40337	0.88275
P14079	P27544	0.8101
P14079	Q9BTE1	0.31466
P14079	Q08AE8	0.48468
P14079	A1A4G5	1.1715
P14079	O43865	0.2975
P14079	Q3C1V8	0.54

P14079	Q15631	0.92882
P14079	Q9NSG2	1.182
P14079	Q9Y247	0.4291
P14079	Q6ZRR9	0.45757
P14079	A6NDX4	0.92092
P14079	Q9NR50	0.66897
P14079	Q8N7R1	1.0757
P14079	Q5H9F3	0.43239
P14079	P56715	0.31796
P14079	Q9Y5Z7	0.62451
P14079	Q5BKZ1	1.0705
P14079	O95831	0.65528
P14079	Q9GZX7	0.32685
P14079	Q8NA57	1.7137
P14079	A6PVC2	0.2361
P14079	Q5JWR5	0.60911
P14079	O43189	0.46078
P14079	Q15651	1.5605
P14079	Q13888	0.59917
P14079	Q8N319	1.9946
P14079	Q96CW6	1.7588
P14079	Q5TCM9	1.1715
P14079	Q9Y5U9	1.376
P14079	Q7Z5M5	0.93546
P14079	Q15929	0.90924
P14079	Q07020	1.2846
P14079	Q86X02	0.50286
P14079	Q9BQE9	1.3407
P14079	P78364	0.18156
P14079	Q9UG56	0.72943
P14079	Q00613	0.82136
P14079	P39019	1.0313
P14079	Q86XN7	1.3222
P14079	Q8N6M0	0.40602
P14079	Q01892	0.68463
P14079	Q3LI83	1.4471
P14079	Q9UKL3	1.686
P14079	Q9NZ09	0.33973
P14079	Q7L9L4	0.79088
P14079	O15060	0.30232
P14079	P78559	1.078
P14079	Q9NQX7	0.71384
P14079	Q13084	1.3625
P14079	Q86Y25	0.6138
P14079	Q8IVM0	0.72622
P14079	Q9NQT6	0.071756
P14079	Q8N205	0.90243
P14079	Q14202	1.1727
P14079	Q96LZ2	0.14781
P14079	Q8N357	0.82787
P14079	P18545	0.67049
P14079	Q9NYZ1	1.4268
P14079	A4D997	1.2383
P14079	P17480	1.043
P14079	Q99583	0.40346
P14079	Q8TAB5	1.3222
P14079	Q96KR7	0.9137
P14079	Q9HCM7	1.515
P14079	Q0P6H9	1.0503
P14079	P26599	0.2307
P14079	Q5TFE4	0.34779
P14079	Q6ZN19	0.51784
P14079	Q8IY85	0.33142
P14079	Q8N813	0.62194
P14079	Q9BSQ5	0.78928
P14079	Q15833	0.48634
P14079	Q8IVI9	0.25791
P14079	P36405	0.28417
P14079	Q8TE02	1.4987
P14079	O95571	0.68487
P14079	A6NGA9	1.0004
P14079	Q99543	0.49041
P14079	Q9NSP4	0.54738
P14079	Q9NUG6	0.29625
P14079	Q5VV52	0.52674
P14079	Q8HWS3	0.51885

P14079	A6NLX3	0.80081
P14079	Q9Y6I8	0.81598
P14079	B4DU55	0.72218
P14079	Q96MW7	0.82405
P14079	O95868	1.2124
P14079	Q9NZL3	0.79328
P14079	Q15054	0.9847
P14079	Q9BTT0	0.23819
P14079	Q8WV60	0.44713
P14079	Q86XI8	1.0472
P14079	Q6ZP68	1.5444
P14079	Q8IYB7	0.14651
P14079	Q9Y3A3	0.71248
P14079	P0C854	1.1715
P14079	Q8TDN4	1.2268
P14079	Q8WY54	0.80255
P14079	P55795	0.24397
P14079	Q9NWS8	0.98358
P14079	Q9UBU6	1.1193
P14079	O96033	0.54587
P14079	Q9BXG8	0.50059
P14079	O75152	0.61803
P14079	Q5VYM1	1.2485
P14079	Q13568	0.79764
P14079	O75940	0.7643
P14079	P59091	1.113
P14079	Q9UJW9	2.1908
P14079	P0CF75	0.67683
P14079	Q9BZE1	1.0353
P14079	P61981	0.68167
P14079	A6NDE8	0.99818
P14079	Q00059	0.85382
P14079	Q7KZ85	0.52203
P14079	Q93045	0.25895
P14079	Q9C0C2	0.63477
P14079	Q92629	0.66506
P14079	P12956	0.33182
P14079	Q8IYN6	1.3798
P14079	O75952	0.64274
P14079	Q9ULZ2	0.95768
P14079	H0YKK7	1.3415
P14079	P61572	1.355
P14079	Q9NWU2	0.12251
P14079	P08758	0.56919
P14079	P61236	0.90931
P14079	Q9H4W6	0.36867
P14079	Q96D05	1.6764
P14079	Q9BWJ5	0.85207
P14079	Q9BZR8	0.69338
P14079	P55345	0.46639
P14079	O15117	1.2772
P14079	Q96QT6	0.94011
P14079	Q8WUD4	0.8243
P14079	Q9P0W2	0.50933
P14079	Q70IA8	0.20252
P14079	Q9Y3T9	1.2149
P14079	P06132	0.95455
P14079	Q9ULW3	0.72955
P14079	Q6ZVS7	0.47122
P14079	Q96D98	1.4727
P14079	Q93073	1.1565
P14079	Q96MC9	1.8592
P14079	Q9HAW4	0.78774
P14079	Q9Y534	0.40345
P14079	O94805	0.96175
P14079	Q14390	0.20497
P14079	P54368	0.38938
P14079	O14744	0.92957
P14079	Q99661	0.29013
P14079	Q8N9N5	0.96378
P14079	A0PG75	0.20822
P14079	Q6NXT1	0.35734
P14079	Q96LT7	1.3317
P14079	Q9NQ32	1.2126
P14079	P46976	0.41076
P14079	Q9Y5V3	1.0387

P14079	A1L162	1.0428
P14079	Q537H7	0.79141
P14079	Q6I9Y2	1.365
P14079	A0AVF1	0.36572
P14079	Q96BP2	1.256
P14079	Q9BWJ2	0.7594
P14079	P48230	0.98482
P14079	P61580	1.7166
P14079	Q03924	0.59749
P14079	Q3LI61	1.1715
P14079	Q8NCN2	0.31886
P14079	Q96T21	1.0749
P14079	Q96N22	1.0179
P14079	P54826	0.45368
P14079	Q8N6F1	0.22769
P14079	Q9BUN5	0.69047
P14079	Q9BQW3	0.29586
P14079	A7E2U8	0.76175
P14079	P50542	0.24729
P14079	Q5U3C3	1.2485
P14079	Q9Y6A4	0.48066
P14079	Q7Z699	0.32085
P14079	P63220	0.83236
P14079	Q5HYC2	0.39011
P14079	Q8IV03	1.709
P14079	P61326	0.96739
P14079	P41182	0.23898
P14079	O43663	0.99252
P14079	P31274	0.75378
P14079	Q8WYJ6	0.29185
P14079	P78385	0.98262
P14079	Q5BJF6	0.79943
P14079	Q7Z6E9	0.19372
P14079	Q86UE3	0.76643
P14079	Q9GZY1	1.1764
P14079	Q6ZMR3	0.58195
P14079	O75880	0.29315
P14079	Q9UNF1	0.046188
P14079	O00566	1.1497
P14079	Q8NAP3	0.41224
P14079	Q9P0I2	0.97942
P14079	Q9UL49	1.2574
P14079	Q6NUI1	1.5018
P14079	Q460N3	0.2217
P14079	Q9NV06	0.24751
P14079	P12525	0.29305
P14079	Q8NFR3	0.93734
P14079	Q8N609	0.7313
P14079	Q01543	0.83392
P14079	O60239	0.4009
P14079	P35250	0.20223
P14079	O15263	1.1399
P14079	Q8N5J4	0.48256
P14079	Q8IZH2	0.22273
P14079	Q9NUW8	1.1062
P14079	P0DJG4	1.5851
P14079	Q96IM9	0.35026
P14079	Q8IVU9	0.55942
P14079	Q96GA3	1.2663
P14079	P10074	0.5376
P14079	A6NN06	1.1715
P14079	Q96RL7	1.3294
P14079	P0CJ87	0.25626
P14079	Q5MIZ7	0.61214
P14079	P23919	0.94023
P14079	Q8WW12	1.3332
P14079	Q5TC04	0.75706
P14079	Q15814	0.54636
P14079	Q6ZUA9	1.3848
P14079	Q9NXR7	0.91094
P14079	Q16637	0.98417
P14079	Q0P5P2	0.90696
P14079	Q9UPG8	0.45023
P14079	Q71F23	0.69873
P14079	Q9BZA0	0.23312
P14079	Q5VWM6	1.6075

P14079	A6NDD2	1.0712
P14079	O95801	0.09332
P14079	A5PLN9	1.101
P14079	Q96CX3	0.56288
P14079	Q86Y01	0.42034
P14079	Q8WUM0	0.76637
P14079	Q6MZP7	0.72859
P14079	Q9HAU0	0.35708
P14079	Q14997	1.0328
P14079	Q7Z4M0	0.89884
P14079	Q9H790	0.73546
P14079	Q96AE4	0.45339
P14079	Q14549	0.30496
P14079	O75791	1.0679
P14079	Q86V59	1.0304
P14079	Q9NRF9	0.53556
P14079	Q13642	0.27178
P14079	Q96GC9	0.81438
P14079	Q96Q35	0.86213
P14079	Q9BSH3	0.55309
P14079	Q8NCG7	1.293
P14079	Q96BR5	0.36349
P14079	A6NMV5	0.28074
P14079	Q3ZCT1	0.6038
P14079	Q8IUX1	0.80739
P14079	Q9BXM0	0.63894
P14079	Q7RTV5	0.050638
P14079	P17097	0.43535
P14079	Q8N5U6	1.0923
P14079	Q7Z7J9	0.90726
P14079	Q6ZU35	1.3222
P14079	Q2M385	0.81442
P14079	O14656	0.15543
P14079	P54803	0.58834
P14079	Q7RTS6	0.94685
P14079	Q86UP8	0.3466
P14079	Q5TAX3	0.73893
P14079	Q17RC7	1.0395
P14079	Q96LL3	1.1127
P14079	A6NJ88	1.4096
P14079	Q0P6D6	0.48323
P14079	P49207	1.4205
P14079	Q9P1U0	0.19978
P14079	Q15906	0.95509
P14079	Q16586	0.55426
P14079	A6NEV1	1.2542
P14079	Q9BV29	0.67808
P14079	Q13185	0.77531
P14079	Q8NC60	0.098938
P14079	Q9Y5K5	0.27156
P14079	Q99496	0.29928
P14079	Q96BU1	0.9129
P14079	Q6VMQ6	1.6647
P14079	P19388	0.81968
P14079	P58317	0.62405
P14079	Q5VWX1	0.66843
P14079	Q96EG3	0.4409
P14079	P38117	0.70226
P14079	Q96NJ1	1.1715
P14079	Q495Y7	0.36123
P14079	P22103	1.1715
P14079	P13645	0.97141
P14079	O95926	0.19344
P14079	Q9NSQ0	0.66878
P14079	Q8WYQ9	0.55015
P14079	P49756	0.45254
P14079	Q96L21	0.72151
P14079	P49589	0.99558
P14079	P62244	1.2179
P14079	Q9UBC3	0.31539
P14079	P51668	0.502
P14079	Q9Y3T6	0.92801
P14079	A6NDD8	1.2315
P14079	A6NK75	0.75103
P14079	P14635	0.49197
P14079	Q8NHZ8	1.5354

45221	P14079	Q14749	0.59156
45222	P14079	Q8N614	1.131
45223	P14079	Q9P2F9	0.46223
45224	P14079	Q00403	1.1581
45225	P14079	Q9UMS4	0.49925
45226	P14079	Q7Z3Y9	0.99997
45227	P14079	A6NLI3	0.34873
45228	P14079	Q3C1V1	1.3222
45229	P14079	Q68DN1	1.1943
45230	P14079	Q03936	0.98917
45231	P14079	O96011	0.51649
45232	P14079	Q8ND61	1.5489
45233	P14079	Q8N8Q3	0.20702
45234	P14079	Q14315	0.54236
45235	P14079	Q9BZE3	0.47376
45236	P14079	Q14692	0.77951
45237	P14079	Q9Y287	0.87366
45238	P14079	Q9H7Z3	0.62919
45239	P14079	Q9UK39	0.54004
45240	P14079	Q9H2C0	0.64711
45241	P14079	Q8NHG7	1.375
45242	P14079	P82921	1.2984
45243	P14079	Q9HC62	0.469
45244	P14079	Q147U1	0.61199
45245	P14079	I3L3R5	1.1715
45246	P14079	Q9UPM9	0.19246
45247	P14079	Q86YW9	0.39009
45248	P14079	Q6A162	0.78763
45249	P14079	Q08ER8	0.72218
45250	P14079	Q13875	0.97863
45251	P14079	P60520	0.37067
45252	P14079	P51955	0.24531
45253	P14079	Q6P2H8	1.3091
45254	P14079	P35754	0.23843
45255	P14079	Q8NBR0	1.4416
45256	P14079	Q9UMS0	1.1857
45257	P14079	O15172	0.65795
45258	P14079	Q13356	0.17368
45259	P14079	O95782	0.65716
45260	P14079	O75943	0.2888
45261	P14079	Q9H7N4	1.5448
45262	P14079	Q86WU2	0.087118
45263	P14079	A6NCI5	1.2481
45264	P14079	Q14657	1.8479
45265	P14079	O00257	0.8391
45266	P14079	Q7L0L9	1.1715
45267	P14079	O75586	0.71708
45268	P14079	Q9H7D7	0.42364
45269	P14079	P15516	0.87818
45270	P14079	Q8TA86	1.291
45271	P14079	O75690	1.1715
45272	P14079	Q9NNW7	0.24978
45273	P14079	Q14790	0.35647
45274	P14079	Q99689	0.62791
45275	P14079	Q9NPB0	1.2018
45276	P14079	Q2M243	0.31618
45277	P14079	A6NGH7	0.80962
45278	P14079	Q5T215	0.71032
45279	P14079	O95379	0.64942
45280	P14079	Q14781	0.80275
45281	P14079	P12270	0.70631
45282	P14079	P55209	0.66293
45283	P14079	Q9H6U6	0.94819
45284	P14079	Q9BZ23	1.1264
45285	P14079	Q9NX40	0.68136
45286	P14079	Q9UNN5	0.84116
45287	P14079	Q9UJ98	0.74687
45288	P14079	O94925	0.45483
45289	P14079	Q9UK58	0.35751
45290	P14079	Q14353	0.22335
45291	P14079	Q8NHV4	1.0586
45292	P14079	Q8N661	1.3556
45293	P14079	Q53GS7	1.1123
45294	P14079	Q14DG7	1.1754
45295	P14079	Q5BJE1	0.86779
45296	P14079	Q9BX82	0.97753

P14079	P03928	0.60053
P14079	O75145	0.60174
P14079	P45877	0.18124
P14079	Q8NBJ4	1.0755
P14079	A6NES4	0.95229
P14079	Q9NXT0	0.55972
P14079	Q01860	0.5811
P14079	O95685	0.60785
P14079	O14929	1.0578
P14079	O00512	1.023
P14079	Q8N1N4	0.98699
P14079	Q9H2D6	0.2848
P14079	O75530	0.57509
P14079	Q9Y6C7	1.1715
P14079	Q9H0A3	0.82534
P14079	Q9BSY4	0.95493
P14079	Q9UND3	0.67903
P14079	P63302	1.0039
P14079	Q9H6T0	0.55969
P14079	P30154	0.53791
P14079	Q04760	0.27522
P14079	A7MD48	1.2814
P14079	Q15046	1.3379
P14079	Q9Y2Y6	1.3455
P14079	Q8WTW3	1.4067
P14079	Q8TF39	1.0323
P14079	Q53FT3	1.0059
P14079	Q9BVK8	1.1904
P14079	Q96HA7	0.8125
P14079	O75373	0.69686
P14079	Q9Y3Z3	0.24133
P14079	Q2M3A8	0.71184
P14079	Q6P575	0.56923
P14079	Q15543	1.0149
P14079	Q86UW1	1.1986
P14079	Q9H943	0.55564
P14079	Q6ZYL4	1.5087
P14079	Q66PJ3	1.065
P14079	Q5T036	0.96534
P14079	P28062	0.71967
P14079	O75764	0.28681
P14079	P30408	1.0411
P14079	A8MU10	1.1715
P14079	Q8IZM0	0.968
P14079	Q5VTD9	0.28561
P14079	A6NLF2	0.80003
P14079	A6NI87	1.1145
P14079	P57055	1.08
P14079	Q8N0Z8	1.106
P14079	Q7L8W6	1.1185
P14079	P60900	0.7803
P14079	Q9NP64	0.42003
P14079	Q8NFW9	0.37464
P14079	Q96HD9	0.62934
P14079	P19532	0.68988
P14079	Q9Y5X2	0.28587
P14079	Q13066	0.99818
P14079	P82650	0.65514
P14079	Q69384	1.0384
P14079	B1APH4	0.85237
P14079	Q05481	0.78684
P14079	Q14145	1.2409
P14079	Q15345	0.34598
P14079	A6NGB7	1.2485
P14079	Q8N114	1.132
P14079	Q9UBI9	1.3392
P14079	P42167	0.83206
P14079	Q9H425	1.2653
P14079	A5D8V7	0.11596
P14079	Q5RGS2	1.6988
P14079	Q9P2D6	0.45631
P14079	Q9ULX3	1.1096
P14079	Q5JTV8	0.68961
P14079	Q9BXY0	0.33547
P14079	Q9BYA6	1.1715
P14079	Q9BSF8	0.42364

P14079	P17026	0.47783
P14079	Q7Z3Y8	0.88619
P14079	Q8IX03	0.9903
P14079	Q9BU89	0.62336
P14079	Q96BD5	0.66508
P14079	O00519	0.9038
P14079	P16989	0.18479
P14079	Q8NC51	1.0584
P14079	Q9Y238	0.68281
P14079	A8MTY7	1.3698
P14079	Q02040	1.2762
P14079	Q56P42	0.28036
P14079	Q9UIH9	0.45238
P14079	Q00005	0.84748
P14079	A6NN14	0.78684
P14079	Q9Y6U3	0.27235
P14079	P12980	0.9272
P14079	Q9BXF6	0.80664
P14079	Q9UPN9	0.44425
P14079	Q9UPZ3	0.99242
P14079	Q9H0W9	0.71518
P14079	Q9HCE3	0.1949
P14079	Q9NP74	0.35849
P14079	P11161	0.49685
P14079	P09001	1.1324
P14079	Q5T6V5	1.0403
P14079	Q9BVG4	0.82295
P14079	Q9NWD8	0.99267
P14079	P22392	0.16005
P14079	Q0VDD7	2.2404
P14079	O00482	0.33627
P14079	Q9Y2S7	1.4739
P14079	Q6ICL7	0.80869
P14079	Q08AG5	1.1741
P14079	Q8N5Y2	0.24586
P14079	P0CW21	1.1715
P14079	Q96LP2	0.38694
P14079	Q96A35	1.1692
P14079	Q9H9T3	0.58525
P14079	Q2M3G4	0.27638
P14079	O75425	0.98175
P14079	Q96BY2	0.70274
P14079	Q8N4C6	0.97685
P14079	Q9UJW3	0.65947
P14079	Q9BQ89	1.8375
P14079	Q8NF64	1.2388
P14079	Q969L2	0.57199
P14079	Q9NZC7	0.3179
P14079	Q9NWF9	0.78835
P14079	Q96T51	0.35609
P14079	Q9NX05	0.98744
P14079	P24534	0.78141
P14079	A1L3X4	1.1715
P14079	Q9GZU3	1.6442
P14079	P20930	0.37813
P14079	Q86U90	1.3652
P14079	Q99732	0.89019
P14079	H3BR10	1.1715
P14079	Q32M88	0.56045
P14079	Q9NV56	1.3349
P14079	Q96C34	1.2557
P14079	A6NGS2	1.238
P14079	Q16644	0.25069
P14079	O94829	1.2132
P14079	Q6N075	1.0464
P14079	Q86UC2	1.077
P14079	A6NCC3	0.85082
P14079	Q13956	0.93145
P14079	Q9H761	0.82736
P14079	Q96K75	0.56807
P14079	Q6ZU67	0.82529
P14079	O60318	0.88482
P14079	Q9H6Q4	0.6494
P14079	Q6NSJ2	1.2749
P14079	Q9UEE9	1.2615
P14079	Q8NC96	1.0646

P14079	Q86YE8	0.77845
P14079	P62263	0.76983
P14079	Q8N972	0.77845
P14079	Q9UKV3	0.69341
P14079	Q9Y3C1	0.24848
P14079	Q969R5	0.53184
P14079	Q12962	1.2315
P14079	Q8N5R6	0.9716
P14079	Q9NZQ3	0.92902
P14079	Q9H963	0.81093
P14079	Q6DD87	0.50185
P14079	Q9UER7	1.3594
P14079	Q8ND90	0.813
P14079	Q02543	0.96995
P14079	Q3SY05	0.4211
P14079	Q6ZVH6	0.45051
P14079	P19474	0.27566
P14079	O95521	1.0333
P14079	Q9ULH7	0.79925
P14079	P62424	1.3062
P14079	Q9Y4M8	0.48845
P14079	P60331	1.3698
P14079	Q6UXU6	1.8529
P14079	P55265	0.43572
P14079	Q16719	0.34392
P14079	Q92901	0.86734
P14079	Q8TAC9	1.0112
P14079	P36639	0.37336
P14079	A6NGB9	0.6828
P14079	P17544	1.1167
P14079	Q9Y6Z4	0.7477
P14079	Q9NPF5	1.0618
P14079	Q9NYU2	0.47772
P14079	Q8WYP5	0.67014
P14079	A6NFY7	1.1151
P14079	Q6UWN0	0.78423
P14079	Q9BV81	1.1389
P14079	Q6RFH8	0.78747
P14079	Q9BR11	0.44257
P14079	P62277	1.2662
P14079	O00631	1.16
P14079	Q9NX09	0.55018
P14079	O75427	0.72931
P14079	Q96AQ7	0.91714
P14079	Q9UH90	0.11619
P14079	Q17RH7	1.1715
P14079	A6NML5	1.2485
P14079	O95238	0.80426
P14079	Q9HCJ0	0.68872
P14079	Q92769	0.43128
P14079	A8MQB3	0.73576
P14079	Q86T23	0.18173
P14079	P31040	0.49604
P14079	Q9UBD5	0.8681
P14079	Q8IUH2	0.9891
P14079	Q9UK28	1.2329
P14079	Q9BXR0	1.7865
P14079	O43681	0.43543
P14079	Q96T17	1.0096
P14079	Q96LK8	0.75056
P14079	P56277	1.47
P14079	Q8IZS5	1.2271
P14079	Q96HJ5	0.59183
P14079	Q9BXX3	0.24143
P14079	Q8N2B8	0.99123
P14079	Q9UL45	0.2088
P14079	Q8N9W8	1.8805
P14079	Q96I24	0.49296
P14079	Q2M3R5	1.2951
P14079	O75822	1.2752
P14079	Q5W150	0.11821
P14079	Q9BZI7	1.2214
P14079	A6NKF7	1.2485
P14079	Q9NQA3	0.59718
P14079	Q6UXQ6	1.1715
P14079	Q96IL0	0.61574

P14079	Q9P2Z0	1.0623
P14079	Q9H467	1.1846
P14079	O00716	1.0617
P14079	Q8N8X9	0.67081
P14079	Q2M2I5	0.99997
P14079	Q9NVV2	1.1715
P14079	Q8IY57	1.0615
P14079	Q8N9U9	0.89899
P14079	Q96IF1	0.50574
P14079	Q6ZMV8	1.0592
P14079	A8MV65	1.1698
P14079	Q499Y3	0.2506
P14079	O95347	0.34394
P14079	Q969E2	0.96757
P14079	Q9BW66	1.3996
P14079	Q8IVS8	0.44393
P14079	Q5SXM1	0.74708
P14079	O00410	1.4057
P14079	Q96EN9	1.3553
P14079	O14737	0.97695
P14079	Q9NYH9	0.25824
P14079	Q9H4T2	0.43151
P14079	Q9BZ01	0.75211
P14079	Q969X5	0.3649
P14079	P23528	0.65212
P14079	Q9UM11	1.3244
P14079	Q9H2W6	0.92173
P14079	O14829	0.37625
P14079	Q9UL15	0.29243
P14079	Q86VP3	0.95374
P14079	P53674	0.49183
P14079	P11940	0.2929
P14079	Q92934	0.17174
P14079	Q3SY89	0.56053
P14079	P29374	0.25779
P14079	Q8NCK3	0.58663
P14079	Q9BZQ4	0.48014
P14079	O43711	0.2521
P14079	P0CG43	1.1715
P14079	P62750	0.99345
P14079	O75200	0.81659
P14079	Q8IZU3	0.29489
P14079	P0CAT3	0.23996
P14079	O96018	0.63426
P14079	A6NMN3	1.2214
P14079	Q7Z5Q5	0.61952
P14079	Q96Q89	0.62544
P14079	Q5H9S7	1.4076
P14079	Q8NDM7	0.90986
P14079	Q5VWM5	1.1205
P14079	O43633	0.46163
P14079	Q8WV28	0.30471
P14079	Q9UN42	0.30061
P14079	Q9UBB6	1.0186
P14079	Q8TF20	0.76639
P14079	O14548	0.62161
P14079	Q96EX1	0.79946
P14079	P29966	1.7407
P14079	Q5T6C5	1.0973
P14079	Q9BY32	0.9955
P14079	Q6P2I7	0.67609
P14079	Q8NCU7	1.6087
P14079	Q96MD2	0.64753
P14079	Q9H8W3	1.2295
P14079	P62910	0.90311
P14079	Q86TS9	0.85626
P14079	Q538Z0	1.1715
P14079	O95522	0.15504
P14079	Q8IV53	0.15996
P14079	Q9UBB4	1.407
P14079	Q6XQN6	0.47877
P14079	Q15121	0.71584
P14079	Q8N201	0.87137
P14079	Q96EK5	0.63167
P14079	Q9UHV7	0.67177
P14079	Q96PU9	1.0259

P14079	Q9H583	1.1507
P14079	Q9GZZ1	0.46034
P14079	A6NKW6	0.85881
P14079	Q5VT06	0.84095
P14079	O75915	0.96664
P14079	Q63ZY6	0.33428
P14079	Q6ZQT7	1.1715
P14079	Q5H9T9	1.0155
P14079	P49755	0.17088
P14079	Q9BQA1	1.0204
P14079	Q9P299	0.43366
P14079	Q8IV36	0.72395
P14079	O43676	1.0337
P14079	Q9NX00	0.72411
P14079	P63173	1.1886
P14079	Q4G163	0.16561
P14079	Q8NBT0	0.27372
P14079	Q5VTR2	0.37549
P14079	Q86XJ0	0.80503
P14079	Q9UPP1	0.37708
P14079	Q86XW9	0.36002
P14079	Q5SQS8	1.188
P14079	O94818	1.6655
P14079	P07197	1.248
P14079	Q9Y4Z0	0.76478
P14079	P17032	0.57645
P14079	Q9H6D3	0.95618
P14079	B7ZBB8	0.4603
P14079	P26196	0.55329
P14079	Q8IZQ5	0.78892
P14079	Q5TID7	1.0479
P14079	A6NFT4	0.76001
P14079	P31946	0.59287
P14079	Q9BQY9	0.6537
P14079	Q7RTS1	0.27975
P14079	O75787	0.69314
P14079	Q8N6T7	0.74001
P14079	O60248	0.59198
P14079	Q12912	1.4366
P14079	Q8NI35	0.46824
P14079	Q9Y5P3	0.85942
P14079	Q86WV6	0.088286
P14079	Q9BUI4	0.35187
P14079	O95989	0.72871
P14079	Q86T03	1.6002
P14079	Q5JY77	1.1759
P14079	Q02930	0.91651
P14079	Q08379	0.57496
P14079	Q6ZSN1	0.98894
P14079	Q8NFZ5	1.0315
P14079	Q9H1I8	0.86323
P14079	Q5VTA0	0.88502
P14079	Q8TEK3	0.65182
P14079	Q2KHM9	0.91677
P14079	Q8WUM4	1.4497
P14079	Q9NQ33	0.49163
P14079	Q53FP2	1.0739
P14079	Q99708	0.84588
P14079	Q969U7	0.85985
P14079	O95567	1.1715
P14079	O95049	0.71329
P14079	O75949	1.2485
P14079	O00264	0.14082
P14079	Q5VXU3	0.84086
P14079	Q9H2G4	0.59002
P14079	P43363	0.10847
P14079	Q6ZP80	1.2485
P14079	O95397	1.5333
P14079	Q9Y6X1	0.77793
P14079	A4D250	0.62096
P14079	Q6P4D5	1.4309
P14079	O75409	1.0453
P14079	Q14093	1.0715
P14079	P49458	1.3997
P14079	Q8WVC0	0.92483
P14079	Q9NWT6	0.32871

P14079	P55210	0.24934
P14079	P0C5W0	0.63681
P14079	Q8N823	0.77845
P14079	Q9BQ49	1.2485
P14079	P03973	0.3624
P14079	Q8ND04	1.0448
P14079	P49715	0.23854
P14079	Q8NFU4	1.1715
P14079	P69849	1.0231
P14079	Q96PY0	0.94038
P14079	E0CX11	1.1715
P14079	Q6DKI1	0.23329
P14079	Q96G23	0.13657
P14079	Q8N8J6	0.44884
P14079	Q9BWW4	1.3763
P14079	Q9BQ67	0.42364
P14079	Q96GC5	1.0375
P14079	O75334	0.71038
P14079	O60663	0.46789
P14079	Q9NW97	1.2485
P14079	O14709	0.77845
P14079	Q9BSH5	0.51284
P14079	Q9BZ97	1.1715
P14079	Q8NFH8	0.65078
P14079	O94772	0.35716
P14079	O15266	0.38579
P14079	Q8N6Y0	0.85637
P14079	Q8IYQ7	0.28389
P14079	Q5DJT8	0.62582
P14079	Q14934	0.36514
P14079	Q96QA5	0.71705
P14079	Q96RN5	1.2947
P14079	A1L188	1.3165
P14079	Q5TA81	1.1715
P14079	Q8IUC1	0.72906
P14079	Q9NR11	0.50553
P14079	A8MT66	0.14551
P14079	P23508	0.61978
P14079	Q0VG06	0.97034
P14079	Q15102	0.1791
P14079	Q9BXY8	1.2199
P14079	O60610	0.25774
P14079	O14603	0.50091
P14079	Q07666	0.45448
P14079	O14530	0.31165
P14079	P05423	0.58691
P14079	Q8WXC7	0.9837
P14079	Q9BPZ3	0.85879
P14079	Q5MJ08	0.73098
P14079	Q9UI46	0.27197
P14079	B2RXF0	0.72996
P14079	Q9NZI6	0.44289
P14079	A6NFQ7	0.28169
P14079	Q5JWF8	0.75964
P14079	O43678	1.1063
P14079	P56182	1.3247
P14079	Q96S55	0.70711
P14079	P28289	0.35676
P14079	Q6U7Q0	0.54351
P14079	Q6ZRZ4	1.4968
P14079	Q9BVW6	1.1715
P14079	Q05BV3	0.25965
P14079	Q7L3V2	0.0063456
P14079	Q9BYI3	1.4237
P14079	Q6IB77	0.48859
P14079	P61573	1.355
P14079	Q05519	1.1047
P14079	P60372	1.3698
P14079	Q92764	0.78763
P14079	O75340	0.45542
P14079	Q8N4U5	0.46663
P14079	Q5RKV6	0.20073
P14079	Q92560	0.083849
P14079	P38936	0.36882
P14079	P22914	0.55859
P14079	A8MVM7	1.1715

P14079	Q5GH73	0.88059
P14079	P41252	0.99288
P14079	Q15759	0.23499
P14079	P55808	1.5495
P14079	Q9H1E3	1.6422
P14079	A6NL88	0.85881
P14079	O15205	0.49629
P14079	Q96MH7	0.39154
P14079	Q8N680	0.1949
P14079	Q99426	0.2821
P14079	Q6ZQQ2	1.1718
P14079	Q8WTZ3	0.97414
P14079	O60282	0.35541
P14079	Q53SF7	0.44726
P14079	Q9UM13	0.94638
P14079	Q9H8M1	0.75511
P14079	Q15599	0.5811
P14079	O14832	0.80945
P14079	O60384	1.1388
P14079	Q8NC54	1.7237
P14079	Q0VDG4	0.76695
P14079	P46776	1.2548
P14079	Q8NCR3	0.47989
P14079	Q7Z3Y7	0.77408
P14079	Q9Y6K9	1.2353
P14079	Q6P280	1.0605
P14079	Q5PRF9	0.8663
P14079	Q68EA5	1.0057
P14079	O43345	0.76639
P14079	Q15075	0.2045
P14079	P0C881	0.12268
P14079	Q8N7X2	1.1715
P14079	P08238	0.37052
P14079	Q9BRT3	0.50482
P14079	Q7RTV0	0.38484
P14079	Q641Q2	1.4354
P14079	Q7Z5L9	1.4672
P14079	Q9Y473	0.58331
P14079	Q15726	0.5041
P14079	Q5JSH3	0.29068
P14079	Q9Y6D0	1.0631
P14079	Q9GZN7	1.1722
P14079	Q9BYD3	1.3663
P14079	O60809	1.2246
P14079	Q5T3Y7	0.5671
P14079	Q9HD36	0.55738
P14079	Q7Z4L9	0.28346
P14079	O75477	1.0949
P14079	Q9NUL3	0.80622
P14079	P84101	1.8065
P14079	Q8IWC1	0.79085
P14079	Q9BRP8	1.5411
P14079	Q9Y6K5	0.49461
P14079	Q9H511	0.26737
P14079	Q96RT8	0.73313
P14079	Q6NS38	0.8108
P14079	P09525	0.40375
P14079	P61158	0.028439
P14079	Q68CZ2	0.64219
P14079	Q9BQI5	0.66039
P14079	P0C7U9	1.0613
P14079	O75469	0.28991
P14079	Q6ZRV2	1.5118
P14079	Q969F1	1.1362
P14079	Q9Y6D6	0.76772
P14079	Q9H8G2	1.3459
P14079	Q969M7	0.35218
P14079	Q9NQZ2	0.48602
P14079	Q6ZRX8	0.11952
P14079	P17017	0.52589
P14079	Q9NY12	0.79306
P14079	Q7Z392	0.85098
P14079	P17987	0.27692
P14079	Q9NPG3	1.1001
P14079	O60829	1.3718
P14079	P31269	0.4413

P14079	Q9BRR8	1.3746
P14079	Q99933	0.92972
P14079	O95996	0.45858
P14079	Q92832	0.25766
P14079	Q13278	1.3096
P14079	Q96F86	0.74381
P14079	O60502	0.32385
P14079	P08107	0.4926
P14079	P0CG40	0.10657
P14079	Q12798	0.94464
P14079	P24863	1.257
P14079	Q3KRA9	0.89437
P14079	A5A3E0	0.96316
P14079	Q9BYT9	1.1778
P14079	P25791	0.60085
P14079	P82912	1.1271
P14079	P08621	0.44427
P14079	Q5T9C2	1.3222
P14079	Q96SN7	0.50831
P14079	P45985	0.31811
P14079	I6L899	0.28471
P14079	Q9HAB3	1.2062
P14079	P08397	0.50902
P14079	O76021	0.71123
P14079	P49848	0.70597
P14079	Q9UI26	0.5192
P14079	Q13042	1.5935
P14079	Q9UBI1	0.56221
P14079	Q99567	0.7654
P14079	Q13057	0.77511
P14079	P48681	0.13875
P14079	P51508	0.58948
P14079	A8MZG2	1.3222
P14079	Q6NVV7	0.85905
P14079	Q5T1C6	1.2284
P14079	Q9H2H8	0.21079
P14079	Q5H9M0	1.4965
P14079	O00139	0.5218
P14079	Q16587	0.5585
P14079	Q9H1B7	1.4672
P14079	Q15773	1.5911
P14079	Q6UWF9	1.2175
P14079	P0CJ69	1.3031
P14079	Q6UXQ4	1.1715
P14079	Q9NZ72	0.40316
P14079	O15049	1.0342
P14079	O43765	0.85034
P14079	Q96GT9	1.3702
P14079	Q5HYL7	1.0613
P14079	P0C7X5	0.64981
P14079	Q8WWM1	1.45
P14079	P61571	1.1454
P14079	Q9NVZ3	0.88008
P14079	Q4VXA5	0.99171
P14079	Q8NC56	0.78731
P14079	A6NDK9	0.49605
P14079	Q6P050	1.3018
P14079	Q92945	0.63893
P14079	Q9NRY5	1.8974
P14079	P35321	1.0119
P14079	Q9NWV8	1.0675
P14079	Q2VPK5	0.95238
P14079	O75820	0.52589
P14079	Q6ZS27	0.51682
P14079	O14558	0.87489
P14079	Q2M1K9	0.32182
P14079	Q96A00	1.0405
P14079	Q8N684	0.562
P14079	Q15555	0.30864
P14079	Q9UNM6	0.70091
P14079	Q9UQ35	1.1966
P14079	O15160	0.31145
P14079	A6NHX0	1.4681
P14079	Q9NQ76	0.67406
P14079	Q13867	0.46971
P14079	Q8N6M9	0.27176

P14079	A2RU37	0.81816
P14079	O95243	0.81026
P14079	P16333	0.65633
P14079	P58557	1.6877
P14079	P51970	0.13406
P14079	P56282	0.60822
P14079	Q8NE71	0.46893
P14079	O14734	0.81677
P14079	Q15058	0.60399
P14079	Q9BYG4	0.84627
P14079	Q4ADV7	0.8519
P14079	Q9Y3B6	1.4159
P14079	O60256	0.37508
P14079	Q9NY33	0.59952
P14079	Q96JM7	0.26348
P14079	P27540	0.35056
P14079	Q9BQG0	0.44584
P14079	Q8TCI5	0.60789
P14079	P61960	1.0085
P14079	A6NHQ4	1.4327
P14079	C9JN71	0.69781
P14079	Q8IVN3	1.4296
P14079	P0CG12	1.936
P14079	Q96J86	1.2485
P14079	Q9H4Z2	0.77928
P14079	P13640	0.90692
P14079	A8MVW0	1.245
P14079	Q9NZM4	1.8643
P14079	Q9NUC0	0.80312
P14079	Q8TAY7	2.1949
P14079	Q6ZUV0	0.47365
P14079	O95866	1.3136
P14079	Q96GX9	0.43987
P14079	Q6IAA8	0.38754
P14079	Q86X24	0.48081
P14079	Q8N1P7	0.56124
P14079	Q96DE5	1.6566
P14079	P52435	0.71425
P14079	Q93034	0.83601
P14079	P08708	0.62102
P14079	Q6ZNB6	0.68329
P14079	Q9Y263	0.28589
P14079	Q3B726	0.38785
P14079	Q702N8	0.73043
P14079	Q6NXS1	1.3737
P14079	Q9UIF9	0.67475
P14079	Q8IVT2	1.3499
P14079	Q96JZ2	0.45652
P14079	P63272	1.0579
P14079	O75525	0.6624
P14079	Q6UWT2	0.98371
P14079	O75534	0.56195
P14079	Q400G9	1.0217
P14079	Q9BXF3	0.81098
P14079	Q8WUX9	0.59817
P14079	Q9H0H9	0.15091
P14079	O95178	1.1931
P14079	Q8N1V8	0.76662
P14079	Q5BKY6	1.1715
P14079	Q12830	0.8409
P14079	P63267	0.99952
P14079	Q66K89	0.70835
P14079	P41002	1.0512
P14079	Q6A163	0.88619
P14079	Q9H1M4	0.83271
P14079	Q9BVV8	1.1037
P14079	Q8N2Z9	0.63901
P14079	O95177	0.59394
P14079	P17031	0.68126
P14079	Q7Z6J9	1.1476
P14079	Q9Y520	1.138
P14079	Q5JRA6	0.10792
P14079	Q5QP74	1.1715
P14079	Q6ZS92	0.79499
P14079	Q9UET6	0.90634
P14079	Q9UEG4	0.60348

P14079	Q6IPT2	1.8805
P14079	Q9Y478	0.10932
P14079	Q13257	0.95912
P14079	Q9H8Y1	1.2172
P14079	Q8N6G1	1.1715
P14079	A6NME0	0.77837
P14079	O75251	0.50305
P14079	Q7Z5S9	0.7849
P14079	P62328	1.5892
P14079	Q14974	1.4203
P14079	Q9UI54	1.1715
P14079	O15195	0.084452
P14079	Q8N2U0	1.1601
P14079	Q96AA8	0.85897
P14079	Q8WZA9	1.3222
P14079	Q9HC47	1.1715
P14079	O14561	0.64566
P14079	Q6ZSK4	0.28022
P14079	Q96EA4	0.94267
P14079	Q9NS64	1.3845
P14079	Q8TAM2	0.38681
P14079	Q86UW6	0.47658
P14079	P0DMC3	1.1715
P14079	P18887	0.97031
P14079	P55060	1.1172
P14079	Q8IYS8	1.0549
P14079	Q96I85	1.1715
P14079	Q9P2R6	0.95177
P14079	Q3LI62	1.1715
P14079	O60888	1.0206
P14079	P50549	0.80368
P14079	A8MUA0	0.71632
P14079	Q92973	1.2878
P14079	Q9UKJ3	1.3031
P14079	Q99569	0.5909
P14079	A6NLW8	0.25626
P14079	Q99613	0.84637
P14079	P17029	0.84533
P14079	P34949	1.0045
P14079	Q68CQ4	0.50567
P14079	Q9BYE7	0.66054
P14079	Q9BRS8	0.61714
P14079	Q96SK3	0.75812
P14079	Q5JUK9	0.87986
P14079	Q7L190	1.4904
P14079	P22670	1.0037
P14079	Q9HBH1	1.064
P14079	Q8NFG4	1.6036
P14079	Q9NQR1	0.4705
P14079	Q8NB12	0.31465
P14079	Q8NFP7	0.5433
P14079	P35251	0.93369
P14079	Q562F6	0.93548
P14079	P49459	0.56404
P14079	Q8WUY3	0.51418
P14079	Q0P641	0.34652
P14079	Q9H6Z9	0.51436
P14079	Q9Y5J3	0.23832
P14079	P41250	0.61106
P14079	Q6NSI3	1.5847
P14079	Q9H4B6	0.69755
P14079	A6NC62	0.79041
P14079	Q9BVC3	0.92553
P14079	A6NIX2	1.0681
P14079	Q9HCE0	0.66463
P14079	Q9HC38	0.56644
P14079	Q96QF0	0.78792
P14079	P17275	0.86989
P14079	P17021	0.63301
P14079	P60328	0.4643
P14079	Q5T655	1.1715
P14079	Q9UJZ1	0.13043
P14079	Q8WVK7	0.79555
P14079	P25063	1.1715
P14079	Q6ZTB9	0.58198
P14079	Q66LE6	0.61407

P14079	P16415	0.69298
P14079	Q9ULJ1	0.27102
P14079	Q86UW9	0.71351
P14079	Q9NZT2	0.34337
P14079	P62140	0.73213
P14079	Q9NYT6	0.54706
P14079	Q9H9L3	0.47764
P14079	Q96IT6	1.1715
P14079	Q6ZQT0	0.29306
P14079	P49450	0.40599
P14079	Q8N8M0	0.80482
P14079	E9PI22	1.3036
P14079	Q86UD0	0.84107
P14079	Q6ZST8	0.42526
P14079	Q86WJ1	0.22432
P14079	Q9H379	1.3698
P14079	F8W1W9	0.85075
P14079	P23258	0.56665
P14079	Q99990	0.67082
P14079	Q86V81	0.82685
P14079	Q8N2G8	0.10345
P14079	Q8N485	0.59587
P14079	I3L1I5	0.89553
P14079	Q8WU58	0.7295
P14079	Q9NY87	1.5672
P14079	Q5VZ72	1.1047
P14079	O95164	1.1657
P14079	P15336	1.007
P14079	Q9UI09	0.18323
P14079	Q9UQK1	0.45356
P14079	Q9UL12	0.27733
P14079	Q14593	1.0416
P14079	Q8N5X7	0.18127
P14079	Q96BF6	0.44669
P14079	Q9NRR4	0.52495
P14079	Q8N4B1	0.29473
P14079	Q9UBF2	0.49977
P14079	Q9NZD4	1.3136
P14079	B9A014	0.68497
P14079	Q96AX1	0.28966
P14079	Q86Y46	0.90092
P14079	Q19AV6	1.8943
P14079	Q4LEZ3	1.5542
P14079	Q8IZA0	0.11531
P14079	Q92750	0.70434
P14079	P0CL81	0.99818
P14079	Q6ZPA2	1.1715
P14079	Q15010	0.27541
P14079	A8MX34	1.0199
P14079	P45381	0.40919
P14079	Q9NYP8	0.86236
P14079	Q9HD15	0.81901
P14079	Q8IZ63	1.1715
P14079	Q8N0U6	1.1715
P14079	P46087	0.72645
P14079	Q9ULZ0	0.28036
P14079	P62166	0.31055
P14079	A8MUU9	0.44324
P14079	Q86U42	0.79452
P14079	O95785	0.25518
P14079	Q8WTW4	0.73884
P14079	Q6R2W3	0.50892
P14079	Q96JM3	0.52775
P14079	Q7L8S5	0.68767
P14079	Q99575	0.7507
P14079	Q9P2A4	0.35778
P14079	Q99460	0.73342
P14079	P11686	0.19063
P14079	O94964	1.3221
P14079	Q96NC0	0.19168
P14079	Q8NBT3	1.2485
P14079	Q8IXK0	0.20684
P14079	Q9H0C8	0.48094
P14079	Q9Y5Q8	0.90791
P14079	Q9HBH7	1.2234
P14079	Q8NC38	0.68921

P14079	Q4AC94	0.50486
P14079	P24855	0.41532
P14079	Q86TX2	0.23623
P14079	O94811	1.1938
P14079	Q8IVP9	0.68773
P14079	P0C7W8	0.42916
P14079	Q12981	0.22084
P14079	Q9H720	0.76278
P14079	Q16384	1.0102
P14079	P35325	1.1715
P14079	Q8NFC6	1.426
P14079	Q8N5Q1	1.8805
P14079	P35573	0.56956
P14079	Q96GM5	0.87682
P14079	Q96I59	0.93806
P14079	Q9BV47	0.30407
P14079	Q9Y697	0.53297
P14079	Q7Z3Z2	0.80814
P14079	A2A2V5	1.2485
P14079	Q96IZ7	1.6932
P14079	Q5VT33	1.0561
P14079	Q9UP83	1.4109
P14079	Q15424	0.78231
P14079	Q96LW9	0.50361
P14079	P53370	0.28988
P14079	A6NEW6	0.50122
P14079	Q92665	1.0836
P14079	P78316	1.5094
P14079	O00559	0.70333
P14079	Q9NVV4	0.37993
P14079	Q30KQ1	0.91101
P14079	Q5VSD8	0.91925
P14079	Q8NHW3	0.25031
P14079	Q8N8Q9	1.288
P14079	Q9H1H9	0.84329
P14079	Q8NAP1	1.3222
P14079	P51523	0.42092
P14079	Q92616	0.31467
P14079	Q17RF5	1.6543
P14079	Q8NDL9	0.4298
P14079	Q969F9	1.7573
P14079	P24522	0.72305
P14079	P0C1Z6	1.5552
P14079	Q9NZ43	0.11193
P14079	Q15697	0.56549
P14079	Q8N2I2	0.5613
P14079	O00148	0.11315
P14079	Q96KX2	0.099211
P14079	Q9Y2Q9	1.1544
P14079	P48200	0.53296
P14079	Q8WV07	0.67114
P14079	Q9Y2B5	0.87921
P14079	Q96AC1	0.2129
P14079	P05814	1.283
P14079	Q8NBN3	1.07
P14079	Q8N1H7	0.78134
P14079	Q9UKK6	0.90817
P14079	Q96H22	1.0112
P14079	Q99445	1.0261
P14079	Q9BQE6	1.4565
P14079	Q8N3V7	1.2495
P14079	A6NI56	0.17318
P14079	Q68DU8	0.09989
P14079	Q9NZP6	1.4294
P14079	Q3BBV0	0.51494
P14079	Q96M83	0.60363
P14079	Q96M78	0.16172
P14079	P36578	1.1841
P14079	O00124	0.64648
P14079	Q9UBK9	1.1731
P14079	P28702	0.4803
P14079	Q969S2	0.6449
P14079	P52743	0.87928
P14079	P00156	0.83264
P14079	P24278	0.82022
P14079	P60660	0.36482

P14079	Q13510	0.89222
P14079	Q9P2X3	0.53955
P14079	Q9BTV4	1.2072
P14079	P04198	0.85622
P14079	Q8N5N7	1.3095
P14079	O43310	0.63702
P14079	Q16630	0.32913
P14079	Q9BYM8	0.25919
P14079	A5D8V6	0.5802
P14079	Q9NR28	1.0156
P14079	Q6ZRH3	0.98809
P14079	P27348	0.43432
P14079	P0CJ72	1.3031
P14079	Q9Y3C4	1.1411
P14079	Q9Y3M9	0.43665
P14079	Q9UBF1	0.95823
P14079	Q6ZW49	0.35646
P14079	Q9BY41	0.34641
P14079	Q6ZRC1	1.1715
P14079	A1L429	0.99818
P14079	Q9NRX1	0.21047
P14079	P62308	0.79088
P14079	P17020	0.46843
P14079	P07864	0.60641
P14079	Q96RT7	0.73313
P14079	Q96T49	0.34246
P14079	P06454	1.2158
P14079	O95299	0.11193
P14079	Q9BT73	0.033928
P14079	Q8N9Z2	1.3103
P14079	Q9H6D7	0.74419
P14079	Q8NFD5	0.86989
P14079	P26640	0.75011
P14079	Q8WXH0	0.37918
P14079	O14978	0.50576
P14079	P23490	1.0462
P14079	O60551	0.52303
P14079	Q8TAD8	1.3186
P14079	Q8WZ55	1.0478
P14079	A6NNX1	0.456
P14079	Q8WV37	1.0106
P14079	Q9GZZ8	1.1715
P14079	E9PQ53	1.3799
P14079	Q86TU6	1.5193
P14079	Q9NY30	0.5861
P14079	Q9BQY4	0.87039
P14079	Q9Y4A0	0.22994
P14079	Q9Y3Y4	0.69802
P14079	Q8NHS9	0.84314
P14079	O75175	1.2501
P14079	O75953	0.8209
P14079	Q6ZMY3	0.39257
P14079	Q5VVP1	2.1067
P14079	A4D1N5	0.050586
P14079	Q8NFH3	1.0451
P14079	B4DZS4	0.91737
P14079	Q5T0Z8	1.3222
P14079	P55854	0.72692
P14079	P0CW22	0.35338
P14079	P35663	0.87908
P14079	Q9Y6R6	0.77845
P14079	Q8NDZ9	1.1715
P14079	Q02833	1.0506
P14079	Q9BYD1	1.2954
P14079	Q6TFL3	0.51874
P14079	Q9NYB0	1.3868
P14079	Q9NYP3	1.2876
P14079	Q96IZ0	0.88889
P14079	O60749	0.40371
P14079	P30419	0.61312
P14079	Q8WUB8	0.42855
P14079	Q96N03	0.56221
P14079	Q9HB58	0.48009
P14079	Q96ES6	1.1888
P14079	Q8NA56	0.12101
P14079	A1L4M7	0.53867

P14079	Q4V348	0.50207
P14079	Q96RV3	0.90212
P14079	Q86UA6	1.5804
P14079	Q9UHR5	1.296
P14079	Q00LT1	1.3639
P14079	Q9NRN7	0.85257
P14079	A6NMP4	0.73811
P14079	P61586	1.0002
P14079	O60506	0.41053
P14079	Q9H944	1.1216
P14079	Q92791	0.51659
P14079	Q5VWM4	0.23582
P14079	P07900	0.37972
P14079	O75360	0.56413
P14079	O43294	0.26735
P14079	Q15370	1.0733
P14079	Q6ZMZ3	0.34389
P14079	Q8N6N3	1.2669
P14079	Q8N945	0.28861
P14079	Q3LI81	1.5617
P14079	Q8NBJ7	0.78482
P14079	Q9H8W2	0.78622
P14079	Q9Y4B6	1.0215
P14079	Q6ZW13	1.1715
P14079	Q15468	0.8912
P14079	Q9UK10	0.77845
P14079	Q15696	0.27824
P14079	Q8N8Y2	0.22388
P14079	Q6GPH4	0.48648
P14079	Q9P253	0.38226
P14079	P0C7X3	1.0071
P14079	Q3ZLR7	1.1571
P14079	Q9P243	0.44368
P14079	Q8TAT6	0.60557
P14079	Q9Y2X9	0.6956
P14079	Q7Z6I8	0.1972
P14079	P05204	1.4345
P14079	P0C843	1.0642
P14079	Q8TF45	0.51784
P14079	Q9UJG1	1.5137
P14079	O95045	0.70949
P14079	Q8NDX6	0.25535
P14079	Q9Y5Y2	0.36504
P14079	Q8TC56	1.8805
P14079	Q9Y2V0	0.78852
P14079	Q9Y2M5	0.8291
P14079	Q15788	0.33503
P14079	O43325	1.4554
P14079	Q8TAD7	1.3222
P14079	Q96EE3	0.63251
P14079	Q86XU0	0.61968
P14079	Q5TGS1	0.53112
P14079	O95292	0.21831
P14079	Q969H8	0.56769
P14079	Q8N6U2	0.88733
P14079	Q9UBB9	0.11939
P14079	Q92759	0.78501
P14079	Q2NL98	1.4633
P14079	Q7Z412	0.49805
P14079	Q9Y6Y0	0.87402
P14079	Q9UFF9	0.46117
P14079	Q9UJQ4	0.2776
P14079	Q6PD74	1.0622
P14079	Q8WWM7	0.84985
P14079	P09012	0.40061
P14079	Q8WVE6	1.2491
P14079	Q96P11	0.27012
P14079	O75909	1.1193
P14079	Q93015	1.3566
P14079	P86478	1.1715
P14079	P60709	0.99952
P14079	Q15185	0.49417
P14079	Q5XKK7	0.61804
P14079	Q8WXQ3	1.7722
P14079	Q96LP6	0.55149
P14079	Q96B33	0.80677

P14079	P13639	0.32386
P14079	Q8TB69	0.99996
P14079	Q9Y4W2	1.4808
P14079	A8MVJ9	1.3134
P14079	O76013	0.89111
P14079	Q969R8	0.21636
P14079	Q13360	0.69598
P14079	Q9H8V3	0.30676
P14079	Q8N801	0.3216
P14079	P49910	0.80948
P14079	A9YTQ3	0.36908
P14079	P16930	0.42436
P14079	Q6UWT4	0.98371
P14079	Q53GA4	0.26713
P14079	Q8N841	0.16329
P14079	Q5T1N1	1.7669
P14079	P49356	0.22029
P14079	P09669	0.92323
P14079	Q8WWL7	1.0581
P14079	Q15847	1.1715
P14079	Q9HAW0	0.58565
P14079	Q08J23	0.70295
P14079	P26358	0.31541
P14079	Q5VZB9	0.34305
P14079	Q9UBV8	0.34285
P14079	Q9BUL9	1.6376
P14079	Q9UK99	0.23607
P14079	Q8WTY6	0.49244
P14079	P56192	0.96395
P14079	Q96I45	1.0611
P14079	Q9Y4X0	0.55607
P14079	Q9Y6M1	0.35318
P14079	Q8N4W9	0.77845
P14079	Q96AQ6	1.5244
P14079	Q9HCD5	1.0823
P14079	Q9UNN4	0.49868
P14079	Q01664	0.57174
P14079	Q9HAP6	0.26763
P14079	Q7Z3B3	0.62676
P14079	Q13326	0.59723
P14079	Q6KB66	0.68555
P14079	Q86W11	0.53302
P14079	Q495D7	0.7212
P14079	Q8NEZ2	1.0224
P14079	O43583	1.58
P14079	Q8N8U2	0.57165
P14079	Q96HG1	1.1715
P14079	O76027	0.23093
P14079	P50749	0.83155
P14079	P37198	0.96678
P14079	Q99417	1.4189
P14079	Q96FI4	0.78243
P14079	P51861	1.0928
P14079	Q96B23	1.4247
P14079	Q9HCK8	0.25544
P14079	O14818	1.1252
P14079	Q9UJA5	1.5428
P14079	Q13794	0.78216
P14079	Q9HBF5	1.4218
P14079	Q96M95	0.20635
P14079	Q5TF58	0.010267
P14079	P22033	0.89387
P14079	Q5VIR6	1.1035
P14079	P46695	0.06196
P14079	P59797	1.4417
P14079	Q92837	1.4457
P14079	Q70Z53	1.4174
P14079	Q8WU20	0.49101
P14079	Q9P0J0	0.54655
P14079	Q5T754	1.1715
P14079	Q86WC6	0.44638
P14079	O00570	0.76011
P14079	Q8IVP5	0.43385
P14079	O00221	0.25504
P14079	P26447	0.40345
P14079	Q9NSD9	1.357

P14079	P15822	0.97655
P14079	Q71UM5	0.84666
P14079	Q9Y312	0.73746
P14079	P51786	0.58477
P14079	Q5GH77	1.2967
P14079	Q96MP5	0.96459
P14079	O14653	0.48837
P14079	Q14134	0.63721
P14079	Q14684	0.78842
P14079	A8MXZ3	1.1715
P14079	Q6ZMB5	0.80647
P14079	O76041	0.29068
P14079	Q5JTJ3	0.60441
P14079	Q92889	0.35617
P14079	Q9BQA9	0.78103
P14079	Q9Y512	0.39629
P14079	Q5JVG2	0.43827
P14079	Q9BX69	0.28419
P14079	Q9H0R8	0.49318
P14079	Q3ZCV2	1.3222
P14079	Q71RC2	0.7753
P14079	Q8IX07	0.33682
P14079	Q9BQJ4	1.416
P14079	C9JDV5	0.34773
P14079	Q17RM4	1.1876
P14079	Q6NSX1	1.1393
P14079	Q96PE7	0.078639
P14079	Q12767	1.2485
P14079	Q14153	1.4106
P14079	P31249	0.3817
P14079	Q9Y3V2	0.986
P14079	Q9BPU9	0.57826
P14079	Q8WZ26	0.91231
P14079	Q96HV5	1.2024
P14079	Q9UJW8	0.53909
P14079	O76015	0.88619
P14079	Q14689	0.21852
P14079	Q7RTT5	0.5033
P14079	Q7RTT6	1.0669
P14079	P78386	1.0109
P14079	Q15366	1.0026
P14079	O95983	1.3944
P14079	Q8N1D0	0.41893
P14079	Q9NZY2	1.1715
P14079	A6NEN9	0.28541
P14079	Q68E01	1.1752
P14079	A6NKF1	1.187
P14079	Q9Y5U8	1.0559
P14079	Q8N8I6	1.1715
P14079	A2BFH1	0.18124
P14079	Q9P258	0.74223
P14079	Q96B96	1.1905
P14079	Q9Y248	0.993
P14079	Q9HA92	0.75283
P14079	Q5PR19	0.57015
P14079	Q2M3X9	0.58948
P14079	Q8TBZ5	0.60617
P14079	Q9Y693	1.0892
P14079	Q15834	1.6027
P14079	P58512	1.4076
P14079	Q9BYC8	1.358
P14079	P55789	0.60627
P14079	A4D1B5	0.41503
P14079	Q86UY8	0.18886
P14079	O43903	0.21429
P14079	A6NK06	0.7927
P14079	Q14533	1.2624
P14079	Q17RN3	1.0245
P14079	Q6ZW34	1.1715
P14079	Q9NS26	1.6039
P14079	Q9NZI5	0.38913
P14079	Q96MF6	1.1332
P14079	Q96Q05	0.71092
P14079	Q8WUY8	1.3524
P14079	P09497	0.60453
P14079	Q14247	0.39231

P14079	Q8IUB9	1.1715
P14079	Q9NS25	1.6647
P14079	Q6P1S2	0.73522
P14079	Q9BTL3	1.4185
P14079	Q2TAL8	1.5279
P14079	P56270	0.76103
P14079	Q13409	0.90128
P14079	O60936	1.4994
P14079	P04141	0.3192
P14079	Q5TB30	0.19381
P14079	O00110	0.25535
P14079	O14836	0.79686
P14079	B3EWG6	1.3222
P14079	P27695	0.40389
P14079	Q53HI1	1.2269
P14079	Q8WUB2	0.92288
P14079	Q5JVF3	0.4711
P14079	Q12983	0.31011
P14079	Q16082	0.97903
P14079	Q8NA42	0.62543
P14079	Q8NHQ8	1.3577
P14079	Q9P2X7	1.1715
P14079	Q9BYQ7	1.4903
P14079	Q13561	1.0563
P14079	P51811	0.48458
P14079	Q96BY7	0.42558
P14079	Q9NX18	1.2662
P14079	Q9Y471	0.74886
P14079	Q8IX21	0.67217
P14079	Q0IIN9	0.96477
P14079	P49721	0.9257
P14079	Q92905	0.61873
P14079	Q9UJY1	0.51272
P14079	P62304	0.79088
P14079	Q15428	1.2202
P14079	Q8N268	0.44185
P14079	Q8TCU4	0.48652
P14079	Q9P2K3	1.3016
P14079	Q8N8U3	0.44963
P14079	Q2M238	0.39555
P14079	Q6ZMW2	0.59361
P14079	O43264	0.80601
P14079	Q9BRJ6	1.3436
P14079	O43612	0.57385
P14079	A2RRP1	0.5874
P14079	Q9UHL9	1.0411
P14079	Q5VSR9	1.5871
P14079	P13232	0.23998
P14079	O75335	0.38952
P14079	Q7Z3V5	0.76643
P14079	O96002	1.1715
P14079	P51116	0.80869
P14079	Q13351	0.24015
P14079	Q99909	0.63993
P14079	P10124	0.84379
P14079	P78424	0.24971
P14079	Q96RK1	0.66181
P14079	Q9UBD0	1.0024
P14079	Q9H0K6	0.59132
P14079	Q9P0S9	1.1991
P14079	Q8IWJ2	0.88802
P14079	A2RUB6	0.5633
P14079	Q9NX01	0.22059
P14079	Q5C9Z4	0.72507
P14079	Q156A1	1.1715
P14079	Q96T92	0.32579
P14079	Q9BU02	1.4752
P14079	Q75NE6	1.7896
P14079	Q8N668	0.8728
P14079	Q92618	0.45445
P14079	P81172	0.90887
P14079	Q96H79	1.1469
P14079	Q5TG53	1.1715
P14079	Q8NC74	0.66448
P14079	Q96LX7	0.24257
P14079	Q9H633	0.71899

46589	P14079	Q6P499	1.288
46590	P14079	Q9P2L0	0.24371
46591	P14079	Q4G0N7	1.1772
46592	P14079	Q96FG2	0.77824
46593	P14079	O94992	1.9791
46594	P14079	Q9NY74	1.7829
46595	P14079	Q9NV70	0.77687
46596	P14079	Q15042	0.82616
46597	P14079	Q5JRC9	1.563
46598	P14079	Q9BQ52	0.59042
46599	P14079	O95171	0.24918
46600	P14079	Q14194	0.51502
46601	P14079	Q49MG5	1.5728
46602	P14079	Q6ZVW7	0.40597
46603	P14079	Q9NVK5	0.44274
46604	P14079	Q7Z309	0.62375
46605	P14079	O15235	1.1209
46606	P14079	Q9BR76	0.67612
46607	P14079	Q96KN1	0.54018
46608	P14079	Q9BQD3	0.62279
46609	P14079	Q9UMX3	0.71216
46610	P14079	Q8NEP9	0.96033
46611	P14079	Q96B70	0.631
46612	P14079	Q17RP2	0.07601
46613	P14079	Q86X51	0.84025
46614	P14079	Q6ZRG5	0.13328
46615	P14079	Q68D10	1.3222
46616	P14079	Q96N35	1.1715
46617	P14079	Q6UXR8	1.1715
46618	P14079	Q8N635	0.21069
46619	P14079	Q96LI9	1.0458
46620	P14079	P60059	0.62098
46621	P14079	Q49A33	0.96042
46622	P14079	Q8TAL5	1.1147
46623	P14079	Q9NX58	0.97698
46624	P14079	Q8IWA6	0.78655
46625	P14079	Q9UKL0	1.3743
46626	P14079	Q9Y2Y1	0.2521
46627	P14079	Q9BYV9	0.34085
46628	P14079	B2RUZ4	1.9102
46629	P14079	P63010	0.58823
46630	P14079	A7MCY6	1.1699
46631	P14079	P32929	0.32406
46632	P14079	Q86VD1	0.84372
46633	P14079	Q9Y284	1.5652
46634	P14079	Q7Z7J5	1.6035
46635	P14079	P62906	0.89675
46636	P14079	P68543	0.67857
46637	P14079	Q9H8U3	0.44416
46638	P14079	Q8N8K9	0.75198
46639	P14079	Q9UI32	0.32823
46640	P14079	Q96MA1	0.24718
46641	P14079	Q5VZ52	0.19009
46642	P14079	Q8N1I8	1.1715
46643	P14079	Q5BIV9	1.2495
46644	P14079	P48788	0.75294
46645	P14079	Q99766	1.136
46646	P14079	Q9Y2M0	1.0293
46647	P14079	P09917	0.27611
46648	P14079	Q9H091	0.88953
46649	P14079	A8MVA2	1.3698
46650	P14079	Q969W0	1.1145
46651	P14079	Q15035	0.44496
46652	P14079	Q8NBK3	0.53612
46653	P14079	Q96Q06	1.3686
46654	P14079	Q1ZZU3	0.85797
46655	P14079	Q9HAU5	0.86467
46656	P14079	Q8NDB6	0.73902
46657	P14079	Q9Y2L1	0.35797
46658	P14079	Q9H3Y8	0.6771
46659	P14079	Q9H0I2	0.6489
46660	P14079	Q9NWR8	1.1808
46661	P14079	A1A5D9	1.3485
46662	P14079	Q6ZUC2	1.1308
46663	P14079	Q2VY69	0.6867
46664	P14079	Q5T7P3	1.1715

P14079	Q96B54	1.6662
P14079	O76014	0.88619
P14079	Q9Y281	0.87566
P14079	Q6UWQ7	1.179
P14079	Q6IPW1	0.84062
P14079	Q96AJ1	1.8924
P14079	A8MT69	0.47369
P14079	P22830	0.24916
P14079	Q96PT3	0.2245
P14079	Q14586	0.54706
P14079	Q9NXV2	1.5238
P14079	Q9Y597	0.18591
P14079	O15397	1.0661
P14079	Q96RL1	0.69462
P14079	Q9Y603	1.0917
P14079	Q9NWX6	1.0228
P14079	P60329	0.4643
P14079	Q8N140	1.1533
P14079	Q9H649	0.25428
P14079	Q96NJ3	0.55945
P14079	Q8NA77	1.1016
P14079	Q9HAI6	1.2004
P14079	Q9NUL5	0.73707
P14079	Q8NAM6	0.08829
P14079	Q9NS56	0.26052
P14079	O60264	0.24762
P14079	Q9BQC3	1.5583
P14079	Q96A26	0.59041
P14079	Q8TDH9	0.37268
P14079	Q9HC78	0.38798
P14079	Q5T7N7	1.1715
P14079	Q9NPJ6	1.1048
P14079	Q8IWA0	0.27853
P14079	Q9ULX9	0.45253
P14079	Q15327	0.52229
P14079	Q8TF30	0.17554
P14079	Q7Z5B4	1.1257
P14079	O43365	0.30277
P14079	Q92530	1.1169
P14079	P49790	1.2323
P14079	P0DJ07	1.2916
P14079	Q8NDW8	0.17571
P14079	O15040	0.56221
P14079	O00287	1.4402
P14079	Q9BZL3	1.2438
P14079	P62316	0.81563
P14079	Q9NSC5	0.6154
P14079	Q6ZN28	1.2595
P14079	P47914	1.6642
P14079	Q9H6X4	1.2485
P14079	Q14135	0.46001
P14079	Q96DX5	0.96869
P14079	P50539	0.3262
P14079	P62280	1.2308
P14079	Q96GK7	0.85454
P14079	Q5XKE5	0.97807
P14079	Q66K66	1.1905
P14079	P46926	0.19637
P14079	Q93075	1.1
P14079	Q16378	1.1715
P14079	Q8N7W2	1.1043
P14079	P53677	0.51642
P14079	Q9NPL8	1.3081
P14079	Q13227	1.7877
P14079	Q8IVC4	0.62784
P14079	Q9UI36	0.21828
P14079	Q6UXP8	1.1715
P14079	P11277	0.47428
P14079	Q8IU54	0.6502
P14079	Q86UE4	0.99589
P14079	Q92982	0.92601
P14079	C9JG80	0.0132
P14079	Q9BRD0	1.1869
P14079	Q9C0F3	0.51295
P14079	Q8N7M0	0.68191
P14079	Q969Z0	0.72808

P14079	Q6P5R6	0.49889
P14079	Q8WTV1	1.5608
P14079	Q5SW79	1.2527
P14079	Q9H354	1.1363
P14079	Q86UU0	1.2687
P14079	A8MVS1	0.97958
P14079	Q5RL73	0.90297
P14079	P84090	0.74249
P14079	Q9UMQ3	0.78855
P14079	Q969S9	0.95321
P14079	Q5TA77	1.1715
P14079	O95744	0.17708
P14079	Q2TBE0	1.0173
P14079	Q5JSQ8	0.52068
P14079	Q8N1X5	1.1715
P14079	Q9BQQ3	0.44857
P14079	A6NFQ2	1.3323
P14079	P62310	0.59113
P14079	O95336	0.17151
P14079	Q9UI30	0.85602
P14079	P35638	0.68384
P14079	Q9Y6X0	0.092831
P14079	Q96J02	0.22763
P14079	Q14154	0.76014
P14079	Q9HA38	0.31108
P14079	O95551	0.67931
P14079	Q7Z7B8	0.7885
P14079	Q9NYP9	0.79074
P14079	P21127	0.019892
P14079	Q07955	0.59424
P14079	O15083	0.83
P14079	O95295	0.49368
P14079	Q96ES7	0.48021
P14079	Q92817	0.2619
P14079	Q8IW40	0.62875
P14079	Q8WWY6	0.46001
P14079	Q9Y324	1.4302
P14079	E5RJ46	1.1715
P14079	Q9NX77	0.67632
P14079	Q9NX38	0.54613
P14079	Q8IXQ9	0.59026
P14079	Q9H2F5	0.64266
P14079	O43296	0.67419
P14079	Q9NQ03	0.28348
P14079	Q6H8Q1	0.44301
P14079	P57764	0.20537
P14079	D6RF30	0.32342
P14079	Q8N0X7	0.33756
P14079	Q9Y4F5	0.25965
P14079	Q9C0F0	0.7151
P14079	Q9UDV7	0.5221
P14079	P49902	0.47454
P14079	Q5H9B9	0.76129
P14079	O97980	1.0613
P14079	Q8WZ69	0.69812
P14079	O14593	0.62471
P14079	Q8IZN7	1.2598
P14079	Q4G0G2	1.2808
P14079	Q9NW68	0.11129
P14079	Q8N0Z3	0.83672
P14079	P23297	0.65573
P14079	P49642	0.70213
P14079	Q9UFN0	0.85079
P14079	Q9NP77	0.25698
P14079	P61574	1.355
P14079	Q16621	0.38414
P14079	Q9GZP8	1.1715
P14079	Q9NV79	0.23644
P14079	Q5T0D9	0.96378
P14079	A9QM74	0.74447
P14079	Q92754	0.47927
P14079	P61962	0.51928
P14079	Q9BZQ2	0.86063
P14079	Q03701	0.4328
P14079	A2IDD5	0.98748
P14079	P06748	0.61252

P14079	P01106	0.4548
P14079	Q9BSJ2	1.4884
P14079	Q6ZP65	0.29059
P14079	Q5TZK3	0.41231
P14079	Q4KMQ1	1.3945
P14079	Q8IV32	1.3103
P14079	Q9H5Y0	1.1977
P14079	P62258	0.99134
P14079	Q13316	1.0114
P14079	Q8NDH2	0.46743
P14079	Q9UGC7	1.0983
P14079	Q9H6P5	0.83297
P14079	Q8N699	1.4834
P14079	Q96LR2	1.6693
P14079	O14662	0.63466
P14079	Q9UBC9	0.70807
P14079	Q8TD31	1.5287
P14079	Q9ULU4	0.77514
P14079	O14569	1.0846
P14079	O43303	1.2546
P14079	Q8IX19	0.91911
P14079	P12883	0.029907
P14079	Q5HYK7	1.2276
P14079	Q5VTE6	0.25093
P14079	Q8WWR8	0.37398
P14079	Q9H115	0.1505
P14079	Q9NRT5	0.98554
P14079	Q6ZTC4	1.1715
P14079	Q8NEM0	0.25939
P14079	Q9H0G5	0.84899
P14079	Q15973	0.56012
P14079	Q9Y262	1.007
P14079	P50453	0.58006
P14079	Q13596	0.53389
P14079	Q9Y3A0	0.8541
P14079	Q86U37	0.5118
P14079	Q6SJ96	0.8553
P14079	P61803	1.013
P14079	Q9BZS9	1.1715
P14079	Q9H8Y5	0.28018
P14079	Q9H6Q3	0.27607
P14079	P02794	0.68691
P14079	Q9HBK9	0.15658
P14079	Q96F07	0.68049
P14079	Q9BTC8	0.6807
P14079	Q9C0F1	0.60994
P14079	Q6PI47	0.18591
P14079	O00472	0.55101
P14079	O76003	0.27567
P14079	Q9NUJ3	0.94507
P14079	Q7RTS9	1.6545
P14079	Q86X53	0.89857
P14079	Q9HDB8	0.78217
P14079	Q9NX04	1.0719
P14079	P10620	0.37055
P14079	Q6PCE3	0.88769
P14079	Q9NZ20	0.62318
P14079	O00505	1.3124
P14079	Q9C099	1.1827
P14079	Q9BXD5	1.0285
P14079	Q86YI8	0.82436
P14079	O43502	0.25181
P14079	Q9H9R9	0.27259
P14079	Q8N3R9	0.27052
P14079	O75444	0.36893
P14079	Q69YG0	0.89617
P14079	Q6UX46	1.2766
P14079	Q8WXC6	0.89072
P14079	Q6DKK2	1.2422
P14079	Q15181	0.22589
P14079	Q5R3I4	0.76669
P14079	Q9BYR4	1.3698
P14079	Q13501	0.55826
P14079	Q68D91	0.20182
P14079	Q5VZE5	0.60146
P14079	Q8N300	0.81519

P14079	Q9NSU2	0.29877
P14079	O43402	1.4169
P14079	Q969W3	1.3025
P14079	Q5BKX5	1.3222
P14079	Q14181	0.90529
P14079	Q6MZT1	1.6755
P14079	Q9UIE0	0.61583
P14079	Q9H0W7	0.095786
P14079	Q6P3V2	0.43181
P14079	Q96LS8	1.1168
P14079	P54277	0.20422
P14079	Q6P1L6	0.53333
P14079	Q7Z3G6	0.23989
P14079	Q969L4	0.92302
P14079	Q9H6Z4	1.3066
P14079	Q13137	1.3248
P14079	Q9UH92	0.54754
P14079	B7ZAQ6	1.0766
P14079	Q14CN4	0.90092
P14079	O43929	0.15225
P14079	Q969H4	0.35284
P14079	Q86VK4	0.1949
P14079	Q8N7Q2	1.1715
P14079	Q9NTK5	1.2962
P14079	Q6UXR6	1.1715
P14079	P62899	1.1272
P14079	P56645	0.25887
P14079	Q8NDW4	0.58579
P14079	Q0VGE8	0.55107
P14079	F2Z333	1.2485
P14079	Q6NT89	1.6052
P14079	A6NNV3	1.092
P14079	Q12765	1.2277
P14079	Q96QR1	1.3458
P14079	H3BNL8	1.4707
P14079	Q9H3R5	0.6487
P14079	Q8N4Q1	0.43709
P14079	P52657	1.2857
P14079	P05783	1.5771
P14079	Q9Y2Z4	1.0041
P14079	Q8N715	1.0359
P14079	Q6IN84	0.60587
P14079	Q9H490	0.93748
P14079	Q7L775	1.1992
P14079	Q8NF99	0.54734
P14079	P56693	0.59086
P14079	Q9P1J3	1.1715
P14079	A6NMD2	0.38459
P14079	P58004	1.1371
P14079	Q16236	0.61135
P14079	Q9P2M7	0.72392
P14079	O94973	0.54956
P14079	P98169	0.14748
P14079	Q16533	0.99906
P14079	Q5T2W1	0.62129
P14079	Q9Y692	0.54917
P14079	P17025	0.5751
P14079	Q5SXM8	0.71366
P14079	Q9BWN1	1.3222
P14079	Q13398	1.0082
P14079	A6NGG3	1.1715
P14079	Q96LD1	0.71011
P14079	Q96P16	0.30841
P14079	P61163	0.51854
P14079	Q8TDB4	0.6111
P14079	O94952	1.2769
P14079	Q96KJ9	0.63232
P14079	Q8WVH0	0.45689
P14079	Q00444	0.20401
P14079	Q8IYJ0	1.0077
P14079	A6NEQ2	1.1715
P14079	Q9GZM8	0.16153
P14079	Q8TAE8	1.2109
P14079	Q9Y3R4	0.56955
P14079	Q06190	0.35736
P14079	O43541	1.0002

P14079	Q8N1B4	0.96022
P14079	Q15365	1.0645
P14079	Q00577	0.25729
P14079	Q9NVL1	0.096751
P14079	P59022	1.2299
P14079	P0CL80	0.99818
P14079	O95997	0.80691
P14079	Q9Y3D2	0.6629
P14079	Q2T9F4	0.49817
P14079	Q9P0U4	0.40074
P14079	O14862	0.60936
P14079	Q04726	1.0096
P14079	Q96RE7	0.91919
P14079	Q9H6L5	1.204
P14079	Q5JUQ0	0.82273
P14079	Q6URH7	0.40586
P14079	Q9NP92	1.4515
P14079	A6NH52	1.4268
P14079	Q8WUQ7	0.35942
P14079	Q6ZRU5	1.1715
P14079	Q9HAN9	0.57993
P14079	Q9Y606	1.5284
P14079	Q9UJX6	0.68984
P14079	Q12972	0.60179
P14079	Q494X3	1.0057
P14079	Q9NP87	0.31543
P14079	Q9Y4X4	0.26907
P14079	P0C853	1.1715
P14079	Q9BYN8	1.5042
P14079	Q5SRD0	0.78389
P14079	Q96GE9	1.1025
P14079	Q12800	0.23725
P14079	Q86WB0	1.7921
P14079	Q8TDP1	1.2571
P14079	Q14139	0.43973
P14079	Q86WZ0	1.2281
P14079	Q8N2G4	0.55467
P14079	Q8N2H4	1.6954
P14079	Q7RTU0	0.42542
P14079	Q96FB5	1.2828
P14079	Q8NCU1	1.1333
P14079	Q9P031	1.8208
P14079	Q8WW24	0.4859
P14079	Q96G01	0.34967
P14079	Q0VD86	0.42364
P14079	Q9H0H0	0.76335
P14079	Q9BS34	0.5583
P14079	A6NFA0	1.2485
P14079	Q7RTV3	0.33874
P14079	Q9P0W5	1.211
P14079	Q96A44	0.51053
P14079	P01358	1.1715
P14079	Q13405	1.0375
P14079	Q8TBR4	0.75577
P14079	Q96E09	1.3798
P14079	Q5T5Y3	0.70577
P14079	Q9UFE4	0.48824
P14079	P48444	0.61213
P14079	Q8N8A4	0.45439
P14079	Q6ZV50	0.40094
P14079	P0CJ92	0.47222
P14079	P0DKV0	0.52246
P14079	Q96JE9	0.50405
P14079	Q8WVK2	1.4232
P14079	O95478	0.98764
P14079	Q6L8G5	1.1715
P14079	Q2M2I3	0.42028
P14079	Q86WR6	0.11302
P14079	Q5TEZ4	1.1715
P14079	O75366	0.03519
P14079	Q8WW43	0.44321
P14079	Q96DB5	0.81776
P14079	Q96SL8	0.45054
P14079	O42043	0.89894
P14079	P42224	0.27399
P14079	Q9HCU9	1.3518

47045	P14079	A4D1U4	0.92289
47046	P14079	P00480	0.43592
47047	P14079	Q6P4R8	1.1571
47048	P14079	O75964	0.37657
47049	P14079	Q5VUA4	0.5522
47050	P14079	Q86V85	1.0214
47051	P14079	Q86UD4	0.53698
47052	P14079	O00411	0.89423
47053	P14079	Q96BM0	0.99429
47054	P14079	O75531	1.2091
47055	P14079	Q2M2E5	1.1606
47056	P14079	Q8IUC2	1.497
47057	P14079	Q6S9Z5	1.5994
47058	P14079	Q9UPV7	0.88749
47059	P14079	Q6L8G9	1.1715
47060	P14079	Q8N6L7	1.5998
47061	P14079	Q7Z570	0.41433
47062	P14079	P58505	0.9848
47063	P14079	Q8N1V2	0.66973
47064	P14079	Q5VTH2	1.0108
47065	P14079	Q9BZG8	0.64153
47066	P14079	Q8IWV1	0.93551
47067	P14079	Q9UN37	0.51704
47068	P14079	Q13617	0.82578
47069	P14079	Q6UX71	0.25154
47070	P14079	P02810	1.1715
47071	P14079	Q8TEX9	0.59093
47072	P14079	P0CG35	1.1711
47073	P14079	Q6ZNM6	1.3143
47074	P14079	C9J442	0.86791
47075	P14079	P33992	0.34466
47076	P14079	Q8N0Z6	0.41189
47077	P14079	Q96RU7	0.35709
47078	P14079	Q7Z614	0.66074
47079	P14079	O00488	0.27261
47080	P14079	O60831	1.6215
47081	P14079	Q4QY38	0.91101
47082	P14079	Q96QA6	0.51205
47083	P14079	A8MYP8	1.1675
47084	P14079	P17038	0.78684
47085	P14079	Q6ECI4	0.75812
47086	P14079	Q8N3J9	0.53011
47087	P14079	O43543	0.17588
47088	P14079	Q8IWD5	1.1674
47089	P14079	Q8TBZ0	0.32082
47090	P14079	Q9H4Z3	0.14508
47091	P14079	Q6ZVT0	0.15983
47092	P14079	P10588	0.35299
47093	P14079	Q8N3X6	1.3764
47094	P14079	Q569K4	0.41299
47095	P14079	Q99848	0.63023
47096	P14079	Q6ZRP5	1.1715
47097	P14079	Q9UI17	0.28997
47098	P14079	Q96FZ7	0.21536
47099	P14079	Q8N1G0	0.31886
47100	P14079	Q6PGN9	1.3047
47101	P14079	Q13601	0.4915
47102	P14079	Q5QJE6	1.5743
47103	P14079	Q9BX26	1.5747
47104	P14079	Q3LI72	1.1715
47105	P14079	Q3LI77	0.38733
47106	P14079	Q8IXJ6	0.43022
47107	P14079	Q6IA69	0.8506
47108	P14079	Q8NA58	0.75455
47109	P14079	H3BU77	1.2231
47110	P14079	P63241	0.60259
47111	P14079	Q96JL9	0.58146
47112	P14079	B1AJZ1	0.35534
47113	P14079	Q5VZI3	0.96135
47114	P14079	Q99595	0.3024
47115	P14079	Q13542	0.84446
47116	P14079	Q6AI39	1.4234
47117	P14079	Q9UBW5	0.72622
47118	P14079	Q86WG5	0.26861
47119	P14079	A6NEA5	0.77329
47120	P14079	P35226	0.52277

P14079	Q8NBF4	0.40238
P14079	Q5TB80	0.97846
P14079	Q9UH77	0.86931
P14079	Q9ULD2	0.61049
P14079	P18850	0.69574
P14079	Q4ZIN3	0.92863
P14079	Q8N0Z2	0.515
P14079	Q8IZL8	1.734
P14079	Q8N612	1.2179
P14079	P32243	0.70641
P14079	Q5U649	0.8077
P14079	Q6RUI8	1.0067
P14079	Q96S66	1.0439
P14079	Q86UL8	0.42389
P14079	Q8WX92	1.49
P14079	O60296	0.55606
P14079	P61353	0.90311
P14079	Q9BT78	1.0752
P14079	Q6PJ61	1.4744
P14079	Q53HL2	1.0798
P14079	P14625	0.13259
P14079	Q68CL5	0.25965
P14079	Q00587	0.53442
P14079	Q969V4	0.24436
P14079	P62081	1.2158
P14079	Q86VE0	1.9844
P14079	A6NP61	1.5416
P14079	A8MV24	0.86888
P14079	Q96HP8	1.7635
P14079	Q3KNV8	0.90711
P14079	Q86XF7	1.0317
P14079	Q8NAV1	1.4595
P14079	Q15554	0.59298
P14079	Q7Z6R9	0.27592
P14079	Q9Y5R8	0.22979
P14079	Q9HAC7	0.40397
P14079	Q8WWK9	0.73992
P14079	Q5W0Q7	0.69597
P14079	Q5HY98	0.94687
P14079	Q9H1A3	0.56221
P14079	P62945	1.1715
P14079	A6NDX5	0.76931
P14079	Q96MW5	0.22963
P14079	Q5JXC2	0.85423
P14079	P20700	0.85037
P14079	Q6P9A3	0.55626
P14079	Q92628	1.1914
P14079	Q9UIV1	1.0611
P14079	Q8N3J2	0.41934
P14079	Q96IU4	0.37496
P14079	Q6B0K9	0.67533
P14079	Q6ZR54	1.1715
P14079	P41212	0.67455
P14079	Q6ICG8	1.8858
P14079	Q6ZUX7	1.089
P14079	Q9GZP4	0.41871
P14079	Q9Y2A4	1.0063
P14079	P32780	0.85017
P14079	Q86Y91	0.32532
P14079	Q5SWL8	0.44171
P14079	Q9NUM4	1.1092
P14079	O75081	1.3237
P14079	Q8TAP9	1.7479
P14079	Q86UZ6	0.25535
P14079	Q9NQ60	1.1768
P14079	Q9BSJ6	0.93719
P14079	P50548	0.84161
P14079	Q16762	0.45974
P14079	Q9NYJ1	1.7853
P14079	Q3LI64	1.1715
P14079	Q53HV7	0.94774
P14079	Q8N5P1	0.31955
P14079	Q9Y2K1	0.1949
P14079	Q6PJW8	0.25691
P14079	Q86SQ7	1.3851
P14079	Q9C0B2	0.97185

P14079	Q8N1L9	0.9021
P14079	Q9NQ89	0.7842
P14079	Q8WWZ3	0.59849
P14079	Q5HYN5	0.69828
P14079	Q13064	0.25562
P14079	Q8IXM7	0.89587
P14079	Q04743	0.32405
P14079	O95760	0.47642
P14079	Q16775	0.68487
P14079	Q8N682	0.91469
P14079	Q96EL3	0.37599
P14079	Q6IN85	0.73174
P14079	Q05195	0.31539
P14079	P86480	1.1715
P14079	P49915	0.82607
P14079	Q9BXN6	0.90284
P14079	Q8N7Y1	1.1715
P14079	O75489	0.65364
P14079	Q96A65	0.23284
P14079	Q9BW71	1.5495
P14079	A4D263	1.0065
P14079	Q71SY5	1.1024
P14079	Q96PS6	1.4933
P14079	Q9BWQ6	0.94085
P14079	O43361	0.61922
P14079	P41208	0.8237
P14079	Q9NX20	1.1212
P14079	P46777	1.3499
P14079	Q96DN6	1.466
P14079	Q8NDC4	0.20571
P14079	Q5TD97	0.21928
P14079	Q16626	1.0599
P14079	Q8N488	0.66504
P14079	Q07687	0.38856
P14079	Q8N442	0.75378
P14079	Q7Z3T8	0.29551
P14079	Q4G0A6	1.6293
P14079	P04731	0.77468
P14079	Q8N5W9	1.0043
P14079	P42677	1.0677
P14079	O14713	1.2954
P14079	Q8IYI6	0.94258
P14079	A6NKF9	0.79972
P14079	O14508	0.29526
P14079	O14770	0.52138
P14079	P13929	0.088474
P14079	Q14814	0.69901
P14079	Q96BT1	0.57319
P14079	Q701N4	1.1715
P14079	Q9H9J2	0.42492
P14079	Q8N0T1	1.231
P14079	P60371	1.3698
P14079	Q8N9H6	0.96957
P14079	Q96EK4	1.737
P14079	Q53H64	1.027
P14079	Q9Y5K6	0.4045
P14079	Q9UKR5	1.0014
P14079	P30050	0.96995
P14079	Q5VUM1	0.86374
P14079	Q5SY85	1.0086
P14079	Q6AI08	1.0435
P14079	Q5JTH9	0.74995
P14079	O75920	1.8872
P14079	Q9NRP2	1.5509
P14079	Q902F8	1.0072
P14079	Q86Y26	1.3874
P14079	Q9NYF3	1.2768
P14079	A2AJT9	0.90854
P14079	O14645	0.35766
P14079	Q0D2K3	1.359
P14079	Q9Y2M2	1.0144
P14079	P81877	0.76434
P14079	Q9BUY5	0.93637
P14079	Q9NV44	0.81779
P14079	Q6NZI2	1.1701
P14079	Q0VDD5	1.1715

P14079	Q13067	0.33792
P14079	Q86TG7	0.7057
P14079	Q8TD06	0.64135
P14079	Q5T4I8	0.030869
P14079	Q9H4M3	0.46683
P14079	Q9H7R5	0.80079
P14079	Q53TS8	0.83039
P14079	Q8N9M5	0.57481
P14079	Q9HCX3	0.4662
P14079	Q9Y2G2	0.67892
P14079	Q9H4A5	1.4788
P14079	Q13367	0.58338
P14079	Q12933	0.49506
P14079	Q6PK81	0.62253
P14079	Q9P015	1.0817
P14079	O60813	0.66668
P14079	Q8TBF2	0.70828
P14079	Q13432	0.80475
P14079	Q6ZV77	1.1715
P14079	Q9BS31	0.47343
P14079	Q9GZW5	0.85599
P14079	A6NIY4	1.092
P14079	Q04724	0.73839
P14079	O95424	1.2617
P14079	Q9UJ04	0.33753
P14079	Q3LI54	1.1715
P14079	P17050	0.20718
P14079	Q9Y366	0.35221
P14079	Q8TAZ6	1.2231
P14079	Q5JPF3	0.2366
P14079	C9JLR9	1.2541
P14079	Q96PE3	0.27686
P14079	P20248	1.2785
P14079	O60841	0.65335
P14079	Q9BXI3	0.58523
P14079	Q6ICB4	0.29473
P14079	P61978	0.9111
P14079	Q8TC44	0.30219
P14079	A6NKU9	1.092
P14079	Q14872	0.83337
P14079	Q8NFH4	1.0451
P14079	Q9UL03	1.5664
P14079	P06746	0.51936
P14079	Q8TDD2	0.43309
P14079	Q9Y6N9	0.49723
P14079	Q5T7B8	0.31989
P14079	A1A580	1.37
P14079	Q12789	0.97949
P14079	Q5BJD5	0.79644
P14079	Q8IUE0	0.28169
P14079	Q8WV19	0.86513
P14079	Q9NQS7	1.1689
P14079	Q8N584	1.653
P14079	P35227	0.62761
P14079	P53602	0.84459
P14079	P15927	0.65178
P14079	Q63ZY3	1.0436
P14079	Q7L5N1	1.2924
P14079	Q2NL67	0.31763
P14079	Q9H7E2	0.61895
P14079	P27815	0.3436
P14079	P31943	0.30568
P14079	Q8TES7	1.0311
P14079	Q6ZSS3	0.5908
P14079	Q9BSC4	0.27853
P14079	Q14676	0.77309
P14079	Q14839	0.49367
P14079	Q9Y2X8	0.61533
P14079	P23610	1.1715
P14079	Q2WGJ8	1.4568
P14079	Q9UKI3	0.52561
P14079	Q86UT6	0.26264
P14079	Q9BT43	0.59427
P14079	Q8WWB7	1.1221
P14079	Q8N3F0	1.0703
P14079	O60756	1.1715

47349	P14079	Q9Y3M2	0.97242
47350	P14079	Q6ZVT6	0.81988
47351	P14079	Q93050	0.071365
47352	P14079	A6NFY4	0.81841
47353	P14079	P20749	0.3133
47354	P14079	O75925	0.55938
47355	P14079	P15880	1.244
47356	P14079	P22532	1.1715
47357	P14079	Q9HCM1	1.495
47358	P14079	Q96LC9	1.0982
47359	P14079	Q86W67	0.3419
47360	P14079	Q96RS6	0.28036
47361	P14079	Q99440	0.72369
47362	P14079	P0CJ78	0.43771
47363	P14079	Q6P656	0.58401
47364	P14079	Q01850	0.92717
47365	P14079	Q9NWM3	0.56024
47366	P14079	Q9Y5R4	0.25663
47367	P14079	Q68DY1	1.0245
47368	P14079	Q9H3K2	0.19238
47369	P14079	Q92547	0.82233
47370	P14079	Q5CZC0	1.6821
47371	P14079	Q14764	1.3355
47372	P14079	P59051	0.92794
47373	P14079	A6NFU8	1.2681
47374	P14079	Q6ZTK2	0.33165
47375	P14079	Q6ICC9	1.2101
47376	P14079	Q6ZV80	1.0896
47377	P14079	Q9H9Y2	0.34874
47378	P14079	A4D0V7	1.545
47379	P14079	Q9H4B0	1.2682
47380	P14079	Q8NA54	0.37985
47381	P14079	Q9H0R1	0.3898
47382	P14079	Q8ND83	1.6777
47383	P14079	A8MPV3	0.28719
47384	P14079	Q9P270	0.77824
47385	P14079	Q9NUK0	0.097805
47386	P14079	Q01081	0.48752
47387	P14079	Q9BUN1	0.99149
47388	P14079	Q9BTT4	0.75955
47389	P14079	K7EIQ3	1.7079
47390	P14079	P10515	0.2639
47391	P14079	Q9Y253	0.29773
47392	P14079	P43358	0.36435
47393	P14079	B2RXF5	0.59902
47394	P14079	Q8TC84	0.42364
47395	P14079	O14512	0.23998
47396	P14079	P35659	1.0131
47397	P14079	Q9BYJ9	1.0626
47398	P14079	P62993	0.29114
47399	P14079	O95810	0.6938
47400	P14079	Q05682	0.37576
47401	P14079	Q86TA1	0.50254
47402	P14079	Q9H8N7	0.28775
47403	P14079	B4DX44	1.0418
47404	P14079	P49427	0.51632
47405	P14079	P23193	1.2446
47406	P14079	Q9H875	0.80308
47407	P14079	Q9Y5W3	0.2338
47408	P14079	Q8N6F7	1.037
47409	P14079	Q5TYW1	0.50207
47410	P14079	Q59GN2	0.85698
47411	P14079	A0PJX8	1.2485
47412	P14079	C9JH25	1.2485
47413	P14079	Q8WZ79	0.73073
47414	P14079	Q8IXW0	0.86347
47415	P14079	Q3B820	1.2395
47416	P14079	O15525	0.48275
47417	P14079	O75394	1.0864
47418	P14079	Q96LT6	0.72651
47419	P14079	Q13158	0.686
47420	P14079	O43918	1.0025
47421	P14079	A8K0Z3	0.22727
47422	P14079	Q96PE6	0.56902
47423	P14079	Q99541	0.42649
47424	P14079	Q96AZ6	0.27591

P14079	Q96S95	1.2152
P14079	Q9H0A9	1.983
P14079	Q7Z6Z6	0.66576
P14079	P13984	0.52071
P14079	Q9P1D8	1.1715
P14079	O75818	0.34553
P14079	P49750	0.93386
P14079	Q9BYG3	0.28323
P14079	P30039	0.54005
P14079	Q9ULL0	1.282
P14079	Q6ZVL8	1.1715
P14079	O75698	1.1715
P14079	Q9BPX3	0.96308
P14079	P25440	0.38451
P14079	O00458	1.4355
P14079	Q9BZF9	0.49459
P14079	P47874	0.76953
P14079	Q9BTY7	0.83392
P14079	Q6AWC8	1.1144
P14079	Q8TDR0	1.6182
P14079	Q8N554	0.1949
P14079	Q86WC4	1.3944
P14079	Q07627	1.1219
P14079	Q7Z7H8	0.5435
P14079	Q7L5A3	1.3061
P14079	Q4W5P6	0.94884
P14079	P38432	1.2401
P14079	Q9BSK1	0.55471
P14079	Q14493	1.0702
P14079	P78406	0.59352
P14079	Q8NE31	0.37695
P14079	Q96SZ6	0.50612
P14079	Q8WXE9	0.639
P14079	P30260	1.34
P14079	Q06323	1.14
P14079	A5D6W6	1.255
P14079	Q9H5L9	0.94805
P14079	Q6L8H1	1.1715
P14079	Q6P9G4	1.1755
P14079	Q9UN79	0.80136
P14079	Q9UJJ7	0.60589
P14079	Q7Z6M4	1.1822
P14079	Q8TAX7	1.1715
P14079	P20073	0.54721
P14079	O43639	0.52506
P14079	P0C7Q2	0.48185
P14079	P60602	1.0996
P14079	Q9Y3F1	1.1715
P14079	Q5T7R7	1.2485
P14079	P62891	1.1719
P14079	P10163	1.1715
P14079	O43299	1.6886
P14079	Q9UNW1	0.47791
P14079	O95613	1.1964
P14079	Q5T011	0.82772
P14079	Q9Y5Z9	0.96105
P14079	O75293	0.75297
P14079	Q6PEX7	1.1591
P14079	Q9ULR5	0.66885
P14079	Q86VM9	0.55754
P14079	P38646	0.4814
P14079	Q13503	0.71015
P14079	Q9UKY7	1.5263
P14079	Q8N729	0.78321
P14079	Q7Z4T9	0.8771
P14079	Q9UII5	0.76639
P14079	Q7Z401	0.36034
P14079	Q96CB9	0.86615
P14079	P15407	0.68498
P14079	Q8TB45	0.25735
P14079	Q2KHR2	0.70362
P14079	Q969Q6	0.54546
P14079	Q0VFZ6	1.3222
P14079	Q9Y4R8	0.73803
P14079	Q99487	0.24435
P14079	Q9NVU0	0.96822

P14079	Q96FZ2	1.6109
P14079	Q06455	0.60605
P14079	Q8N9U0	0.12251
P14079	Q8NDZ6	1.0041
P14079	Q9HCG8	0.45885
P14079	Q8IXM2	0.34032
P14079	Q96SD1	0.26629
P14079	Q8TCY5	0.76276
P14079	Q9BQ87	0.46001
P14079	Q9BQP7	0.62271
P14079	P03923	0.88317
P14079	O43747	0.85714
P14079	Q8NCD3	1.0545
P14079	Q96EF9	0.95381
P14079	Q8N8J7	1.0453
P14079	Q96LM1	1.3478
P14079	Q4V326	0.33792
P14079	P10599	0.25512
P14079	Q5HYM0	0.49753
P14079	Q9Y283	0.33296
P14079	Q9C0H6	0.28511
P14079	Q8IYM0	0.59546
P14079	P27694	0.72229
P14079	P59020	0.38345
P14079	Q6RSH7	0.61897
P14079	Q96M42	0.79299
P14079	Q52M75	0.33278
P14079	Q8N5L8	1.528
P14079	P0DJ93	1.0943
P14079	Q96MZ4	0.21702
P14079	O60220	0.96103
P14079	Q15051	0.95184
P14079	Q9UBS8	0.50052
P14079	Q8TD86	0.29425
P14079	Q5T1J6	0.40445
P14079	Q05877	1.528
P14079	Q8N9R8	1.2172
P14079	Q8WZA8	1.1715
P14079	P28290	1.0145
P14079	Q86WX3	1.3055
P14079	Q8NEZ5	0.50971
P14079	O43159	0.68649
P14079	O75410	0.90619
P14079	Q5JPI3	1.3459
P14079	P63167	1.4007
P14079	Q96KR1	0.86763
P14079	O95456	0.67679
P14079	Q00978	0.85637
P14079	Q9H019	1.1992
P14079	Q3YBR2	0.56875
P14079	Q6PI26	1.6894
P14079	Q8N8Z3	0.54286
P14079	Q2M218	0.58948
P14079	Q9H079	0.8672
P14079	Q6GMR7	1.3551
P14079	Q8WWB3	0.25478
P14079	Q86WZ6	0.54153
P14079	P62191	0.44962
P14079	Q8N6Q8	0.90569
P14079	Q9P242	1.4801
P14079	Q9NNW5	0.70613
P14079	Q96PQ6	1.0085
P14079	P60411	1.3698
P14079	O95777	0.7091
P14079	P82909	0.8163
P14079	Q9UGM6	0.4857
P14079	Q8IYN0	0.71522
P14079	Q14331	0.89597
P14079	Q6UWP8	0.92298
P14079	Q6ZU52	0.65732
P14079	Q9H0F6	0.48379
P14079	Q9UBQ0	0.9541
P14079	Q7Z3B4	1.0968
P14079	B4DXR9	1.0055
P14079	Q16878	0.2905
P14079	Q6ZU69	1.2444

P14079	P29459	0.40949
P14079	Q96IS3	0.265
P14079	Q4V328	1.1859
P14079	Q9H7H0	0.86917
P14079	Q96MX3	0.50263
P14079	Q9P1Y5	0.92651
P14079	O75182	1.1438
P14079	Q14602	0.30059
P14079	Q9UM00	1.7083
P14079	Q96GJ1	0.61033
P14079	O43752	0.75051
P14079	Q9UHT4	1.1715
P14079	P82675	1.0749
P14079	Q8TC76	2.1949
P14079	Q6ZMW3	0.25965
P14079	Q8WYQ5	0.81704
P14079	Q9NZV5	0.86701
P14079	Q9H582	0.1949
P14079	Q9H9A5	0.33348
P14079	A0PK05	1.1071
P14079	A8MVS5	0.82656
P14079	Q9NWW7	1.3714
P14079	P00915	0.09112
P14079	O75376	0.43186
P14079	A6NJI1	1.149
P14079	Q9Y605	2.1545
P14079	A8MXY4	0.78684
P14079	O43791	0.60482
P14079	C9JI98	1.2485
P14079	P60510	1.0237
P14079	Q9ULE6	0.85079
P14079	P54259	1.3385
P14079	P31689	0.52933
P14079	Q86XI2	1.2329
P14079	Q9UK76	1.6397
P14079	Q96LY2	1.4698
P14079	O95563	0.93325
P14079	P24310	0.93068
P14079	Q9H4I3	0.42615
P14079	O14529	0.33373
P14079	Q6P047	0.99599
P14079	Q92900	0.48983
P14079	A8MWA4	1.1051
P14079	Q9BT92	0.97696
P14079	A5D8W1	0.38804
P14079	Q6AZW8	0.57976
P14079	O15042	0.23802
P14079	P0C860	0.32777
P14079	Q9NVM1	1.5543
P14079	Q5T750	0.91588
P14079	Q86YF9	0.42019
P14079	Q8TC05	1.4671
P14079	Q5T0J3	0.92739
P14079	Q63HK5	0.32909
P14079	O15260	0.44373
P14079	Q8N9P0	0.97019
P14079	A8MYV0	0.5863
P14079	O60583	1.2949
P14079	Q7Z2F6	0.86015
P14079	O15078	0.86652
P14079	P78317	0.38643
P14079	O75478	0.41524
P14079	Q09666	0.8366
P14079	Q96AN5	0.83643
P14079	Q86UF4	0.81869
P14079	Q96F45	0.25535
P14079	Q06787	1.0337
P14079	O00198	1.34
P14079	Q8IY18	0.36305
P14079	Q8TCB0	0.38012
P14079	Q5T619	0.53756
P14079	Q8N9K5	0.63696
P14079	Q8WW33	1.107
P14079	O14579	0.81721
P14079	Q9Y3E7	0.97471
P14079	Q6NSH3	1.0398

P14079	Q9HCI7	0.58654
P14079	Q96KM6	0.29703
P14079	Q86W56	0.73527
P14079	Q9H972	0.98371
P14079	Q6NXP0	0.45259
P14079	Q495M9	0.51568
P14079	Q3MJ40	0.61872
P14079	Q6EMB2	0.16797
P14079	Q9BW30	0.51322
P14079	Q6TGC4	1.0771
P14079	Q16718	0.2537
P14079	Q92499	0.27658
P14079	Q92896	0.76442
P14079	Q96SQ5	0.61621
P14079	Q6ZRP0	1.3222
P14079	Q9HB21	0.27571
P14079	Q9Y6Y9	0.65133
P14079	Q9NZE8	1.2688
P14079	Q92674	0.45874
P14079	P04053	0.21992
P14079	Q96FC9	0.31672
P14079	Q3LHN2	1.1715
P14079	Q9UKT7	0.26246
P14079	Q9UIF3	0.47258
P14079	Q96B26	0.52268
P14079	Q96CX2	0.25699
P14079	Q96BK5	0.57277
P14079	Q9HCZ1	0.68687
P14079	Q8IYI0	1.0541
P14079	O95232	0.60528
P14079	O15265	1.3286
P14079	Q9Y2H8	0.60224
P14079	P17014	0.57901
P14079	A4QMS7	0.69189
P14079	Q9H5F2	0.95058
P14079	Q8WY50	1.1715
P14079	O00541	0.72102
P14079	Q6PGQ1	1.0619
P14079	Q9UKB5	0.89575
P14079	Q8TBK2	0.62058
P14079	P67870	0.14264
P14079	O95476	0.31656
P14079	Q9P032	0.72777
P14079	Q5T8I3	0.88228
P14079	O60299	0.567
P14079	Q8N6K4	1.1715
P14079	Q99638	0.73731
P14079	Q9BT30	1.1173
P14079	O43687	1.0589
P14079	Q02577	0.51395
P14079	Q8N6S5	1.2918
P14079	Q9HB96	1.1715
P14079	A6NNA5	0.15259
P14079	Q9UI12	0.34264
P14079	Q9HD33	1.3251
P14079	Q6L8G4	1.1715
P14079	Q96C55	0.1949
P14079	Q9NWW0	0.28036
P14079	Q8IWE5	0.61701
P14079	Q9H0B3	0.22519
P14079	Q15928	1.0401
P14079	Q4V321	0.33792
P14079	P62888	0.95912
P14079	Q8IW36	0.97541
P14079	P19623	0.34236
P14079	P46527	0.53936
P14079	Q9P1T7	1.2328
P14079	O43561	0.3714
P14079	P03897	0.37061
P14079	Q9UFV3	0.41797
P14079	Q9BS92	0.20744
P14079	Q502W7	1.0857
P14079	Q9Y6X3	1.2207
P14079	P08865	0.947
P14079	Q14699	0.94946
P14079	Q9BYR5	1.3698

P14079	O14787	1.1507
P14079	O75688	0.34197
P14079	A6NJQ4	0.50122
P14079	Q15486	0.86844
P14079	Q9UIA9	0.78594
P14079	Q5VT52	0.44811
P14079	Q9NSE4	0.92993
P14079	P55735	0.73364
P14079	Q6ZNH5	0.64121
P14079	Q14590	0.54706
P14079	Q8WXF8	1.8009
P14079	Q8NCN5	0.27578
P14079	Q9NVV5	1.215
P14079	Q9Y4C2	1.572
P14079	Q96AV8	0.24141
P14079	Q9NPA5	0.31665
P14079	Q709C8	0.98802
P14079	Q9H8K7	1.097
P14079	Q9HBU9	0.95511
P14079	Q8N5Z5	0.11489
P14079	Q96BY9	0.73316
P14079	P62837	0.61533
P14079	Q14584	0.6355
P14079	Q70YC4	1.356
P14079	Q14011	0.30813
P14079	P41162	0.39136
P14079	Q9UQE7	0.26751
P14079	Q0P140	1.0897
P14079	Q13155	0.64833
P14079	Q8ND07	1.2418
P14079	Q15562	0.76782
P14079	Q7Z7B7	1.4082
P14079	Q8N1K5	1.2536
P14079	Q86T75	1.2315
P14079	Q5JSZ5	1.6357
P14079	O75132	0.54692
P14079	Q8WUW1	0.5375
P14079	Q0ZLH3	0.98547
P14079	Q8N988	1.0415
P14079	Q13069	0.33792
P14079	Q8N511	1.181
P14079	A1L443	1.8285
P14079	Q9UKI9	1.125
P14079	Q5BKY9	1.3222
P14079	Q8N371	0.28706
P14079	A8MXK9	1.0599
P14079	Q9Y3P4	1.3186
P14079	Q5BKY1	0.34053
P14079	O15067	0.86107
P14079	O75607	0.78487
P14079	Q9P2K1	0.65575
P14079	Q2TAA8	0.63289
P14079	Q15493	0.22702
P14079	Q9UPR3	0.99648
P14079	P36508	0.19861
P14079	P15884	0.58656
P14079	P56381	0.56918
P14079	Q1ED39	1.6655
P14079	Q9NRD1	0.25467
P14079	Q9H607	0.6802
P14079	Q9H0K4	1.3497
P14079	Q96AW1	1.0095
P14079	Q8IYF1	0.73891
P14079	Q8WV92	0.63707
P14079	Q8WXX7	1.0321
P14079	P78414	0.24936
P14079	Q5TA45	0.24462
P14079	Q99504	0.37888
P14079	Q6N069	0.22485
P14079	P15954	0.80284
P14079	Q99439	0.28032
P14079	B8ZZ34	0.85881
P14079	Q5VWN6	0.70296
P14079	Q96QU4	1.4627
P14079	Q13515	0.71659
P14079	P55769	0.95127

P14079	Q9NR55	0.33566
P14079	A6NHY6	0.83592
P14079	Q96LR7	1.3492
P14079	Q9H3M7	0.77713
P14079	Q9P127	1.0058
P14079	P78545	0.96817
P14079	O60885	0.84975
P14079	Q8IV54	0.65487
P14079	Q9BSE2	1.2485
P14079	P82673	0.42865
P14079	Q13164	0.22996
P14079	Q4KMX7	0.42881
P14079	P42025	0.55528
P14079	P41134	0.37184
P14079	P59998	0.51342
P14079	Q9BX40	1.3349
P14079	Q53GL7	0.70576
P14079	O14972	0.65935
P14079	Q53H82	0.24685
P14079	Q96M19	1.1715
P14079	Q9BRH9	0.6446
P14079	Q03468	0.35882
P14079	P78395	1.2703
P14079	Q7Z4H7	0.99639
P14079	P49454	0.63791
P14079	Q17RG1	0.18591
P14079	Q9BVS5	0.69481
P14079	Q4G0X4	0.18591
P14079	Q7Z5V6	1.07
P14079	Q96SA4	0.98083
P14079	O95886	0.66785
P14079	Q7Z7G2	0.96679
P14079	Q96B36	1.4131
P14079	Q8IWB4	1.3668
P14079	Q5JU67	0.27365
P14079	Q9NQC8	0.29688
P14079	P18669	0.96654
P14079	Q9BQ70	1.1757
P14079	P53675	0.44178
P14079	P53672	1.342
P14079	Q5SQ13	0.41385
P14079	Q9NZJ0	0.75845
P14079	O75204	1.2525
P14079	Q92543	0.19276
P14079	Q9NZ63	1.1793
P14079	Q9H9E3	0.77803
P14079	Q9BTP7	1.3249
P14079	Q5XKP0	0.86282
P14079	Q6IPR1	0.69293
P14079	Q2M3C7	0.69688
P14079	Q9H8G1	1.0416
P14079	Q8N137	1.1318
P14079	O75154	0.93282
P14079	A5PL33	1.4259
P14079	P51397	1.073
P14079	Q92466	0.67782
P14079	Q96GM8	0.54697
P14079	Q9NVA1	0.8146
P14079	A6NJB7	1.4513
P14079	P15941	0.62212
P14079	Q9NQV7	0.61787
P14079	Q9BUV0	1.3222
P14079	O75884	1.7845
P14079	Q8IYE1	0.10982
P14079	Q3LHN1	1.1715
P14079	Q9NZV7	1.0397
P14079	A8MW95	0.18893
P14079	P38935	0.28933
P14079	Q69YZ2	1.0911
P14079	Q14137	1.0331
P14079	Q96JB5	0.48919
P14079	Q16589	0.51813
P14079	Q86SH2	0.85922
P14079	Q9HBZ2	0.32463
P14079	P12004	0.31204
P14079	Q9Y3C5	0.47703

P14079	P0CG33	0.65403
P14079	P12757	0.4993
P14079	Q9Y3E0	0.63707
P14079	Q9GZR5	0.61366
P14079	Q8IXS0	1.3222
P14079	A6NG73	0.53464
P14079	Q96EV8	0.24444
P14079	P42166	0.66295
P14079	Q15011	1.1862
P14079	O14521	0.95483
P14079	I3L273	0.64251
P14079	P61927	1.1915
P14079	Q9P2B7	1.3222
P14079	P48378	0.81485
P14079	Q9Y5V0	1.0887
P14079	Q9HAH1	0.8897
P14079	Q96DX8	0.44125
P14079	O14948	0.36877
P14079	Q6IC83	0.43269
P14079	Q9NRP0	0.75027
P14079	P52597	0.35545
P14079	O43920	1.105
P14079	Q96MC5	0.18792
P14079	O60516	0.52655
P14079	P62633	0.54488
P14079	O43390	0.47614
P14079	Q14397	0.67702
P14079	P60606	1.1353
P14079	Q5MJ09	0.94021
P14079	Q9P021	0.5684
P14079	Q9Y2A0	1.0097
P14079	Q6UE05	1.2485
P14079	O15015	0.77928
P14079	P04732	0.74382
P14079	Q9UHA2	0.75785
P14079	Q9UBA6	0.0072542
P14079	Q8NCS4	1.2485
P14079	Q96CB5	0.28199
P14079	Q9Y2B4	0.52313
P14079	Q96M66	0.62927
P14079	Q5VUG0	0.4217
P14079	O95670	0.33047
P14079	Q9NRY2	1.3328
P14079	Q7KZF4	0.95706
P14079	A6NGG8	1.1324
P14079	P62917	1.2356
P14079	P09234	0.70688
P14079	Q6ZSR9	0.38618
P14079	Q02386	0.52686
P14079	Q6ZV56	1.1715
P14079	Q5JQS6	0.90873
P14079	Q9BX70	0.78093
P14079	Q9H2L5	1.3389
P14079	Q99598	0.48144
P14079	P81274	0.65749
P14079	Q8N3X1	0.58099
P14079	Q8N2M8	1.5721
P14079	Q6ZV60	1.1715
P14079	Q9BYQ6	1.3698
P14079	P61567	0.89911
P14079	Q6UWH6	0.75507
P14079	O75381	0.92958
P14079	Q9H0V9	0.5884
P14079	O15239	0.85656
P14079	Q676U5	0.85706
P14079	Q96MG7	0.75772
P14079	Q6NVU6	0.77363
P14079	Q9GZY8	0.74641
P14079	Q9BT25	0.84907
P14079	Q9H1Z4	0.42364
P14079	P49321	1.2541
P14079	Q9NS86	0.89463
P14079	P08670	0.67998
P14079	O43493	1.3864
P14079	Q9HAV4	1.3593
P14079	P07902	1.0429

P14079	Q96MP8	0.30217
P14079	Q8IWS0	0.49861
P14079	O00762	0.56632
P14079	Q9H5P4	0.57823
P14079	A6NEH8	0.79147
P14079	O15226	0.88662
P14079	P0DI80	0.99732
P14079	Q96DM1	1.3329
P14079	Q5VSL9	1.2993
P14079	Q5D0E6	1.3835
P14079	O43504	0.57711
P14079	O94817	0.77481
P14079	Q9Y3B7	1.448
P14079	Q6W0C5	0.79124
P14079	O15182	0.61917
P14079	Q8IZ20	0.1949
P14079	Q99615	0.94133
P14079	Q96N38	1.0078
P14079	P21695	0.23566
P14079	Q8NBD8	1.3453
P14079	Q14687	0.56221
P14079	Q9P1A6	0.40933
P14079	Q9P2B4	0.5316
P14079	O95602	0.65753
P14079	Q8N398	1.0259
P14079	Q9HCN2	1.1715
P14079	P0CG00	0.4186
P14079	P61568	0.91326
P14079	O75347	1.6299
P14079	H0YL14	1.2438
P14079	Q96AA3	0.75495
P14079	Q96G21	1.0268
P14079	Q9BRT6	1.2134
P14079	Q6P2H3	0.8317
P14079	Q969H9	1.1715
P14079	Q96EK6	0.79959
P14079	Q8NBL1	0.2531
P14079	Q9HC07	0.70465
P14079	Q15910	0.41492
P14079	P23526	0.06411
P14079	A6NJ08	0.44102
P14079	Q68CP9	0.57407
P14079	Q5SY13	1.1715
P14079	P30305	0.61999
P14079	Q8IUI8	0.24637
P14079	Q12872	1.1231
P14079	Q9BXL5	0.56169
P14079	Q5SRD1	0.66747
P14079	Q6UW49	0.84301
P14079	Q8NHQ1	1.6759
P14079	P49023	0.22044
P14079	Q96Q11	0.72477
P14079	Q5VZ46	0.8317
P14079	P56279	1.4567
P14079	Q9BU64	0.79525
P14079	Q96MT0	0.5188
P14079	Q15008	0.53113
P14079	Q16625	0.27078
P14079	Q9UMY4	0.30859
P14079	Q9NUP1	0.82799
P14079	Q5VWP2	0.77554
P14079	Q5MNV8	1.1154
P14079	Q9NS93	1.0843
P14079	Q9NZF1	0.54531
P14079	Q9NZH4	1.0343
P14079	Q6NZ67	1.1772
P14079	Q00765	0.20445
P14079	Q9Y2R0	1.1307
P14079	Q9H0V1	1.1456
P14079	O75351	0.34578
P14079	O60269	0.94916
P14079	Q9NQ87	0.34239
P14079	Q13761	0.72016
P14079	Q5JR59	1.5282
P14079	Q9UJP4	0.23093
P14079	Q02556	0.38123

P14079	P49773	0.15016
P14079	O60356	1.3652
P14079	Q6UX65	0.91469
P14079	P26678	1.0711
P14079	Q5T7N8	0.58699
P14079	Q9P0J6	1.1765
P14079	Q8WWR9	1.0802
P14079	P24928	0.77418
P14079	Q96TA1	0.48639
P14079	P04264	0.83522
P14079	Q8IU81	0.17242
P14079	Q9H5Q4	0.98288
P14079	Q6PKG0	0.7217
P14079	O75638	2.0085
P14079	P11137	0.37648
P14079	Q6ZN68	1.1385
P14079	Q9NV92	0.57166
P14079	Q86VW0	0.49719
P14079	Q68D51	0.15996
P14079	Q8N7B1	1.1865
P14079	Q03052	0.3383
P14079	Q13829	0.63207
P14079	Q6U949	1.1715
P14079	Q53SZ7	1.3222
P14079	Q14558	0.29872
P14079	Q5BLP8	0.98371
P14079	E9PQX1	0.65653
P14079	Q68CZ6	0.74419
P14079	Q9NSK0	0.32818
P14079	Q9Y2P8	0.88776
P14079	Q96G03	1.0299
P14079	P80723	1.4509
P14079	Q9ULM6	0.81919
P14079	Q13395	0.18527
P14079	P42773	0.53314
P14079	Q8NEA6	0.37626
P14079	O75665	0.995
P14079	Q8N1G4	0.49474
P14079	P61566	0.77952
P14079	Q96MR7	1.6542
P14079	P30153	0.65571
P14079	Q1RMZ1	1.0134
P14079	Q8N9Z9	0.96871
P14079	Q8N184	0.6226
P14079	Q15369	1.4771
P14079	P46778	0.86734
P14079	Q96A09	1.0411
P14079	Q9P2N5	0.45404
P14079	Q13895	0.8551
P14079	Q9NPC7	0.47342
P14079	P62249	1.041
P14079	Q96K58	0.53274
P14079	O95201	0.77928
P14079	A8MWP6	1.1715
P14079	Q9NWB1	0.29393
P14079	Q69YI7	1.9808
P14079	P0C2W7	1.1715
P14079	Q8IY42	1.7396
P14079	Q6ZW61	0.39428
P14079	Q52LD8	1.0449
P14079	Q7Z4V5	0.12814
P14079	Q9Y6F6	1.0148
P14079	Q9H7S9	0.82104
P14079	A6NMC2	0.23582
P14079	Q9HB65	0.5511
P14079	Q3KRA6	1.8872
P14079	Q8IYX7	1.6142
P14079	Q9BRV8	0.77377
P14079	P13928	0.1776
P14079	Q6UXP7	0.58236
P14079	Q9NWW5	0.72955
P14079	Q8NBA8	0.78495
P14079	Q6ZSG2	1.2582
P14079	P0DI82	0.58206
P14079	Q9H7T3	1.1029
P14079	Q12824	0.97423

P14079	P49754	0.55406
P14079	Q6PI97	0.8805
P14079	P43686	0.42227
P14079	Q8IYE0	1.4415
P14079	P48553	0.50402
P14079	P10589	0.51812
P14079	Q5EBL2	0.47388
P14079	Q9BRQ3	1.3548
P14079	A8MWS5	0.094263
P14079	A2A288	1.1916
P14079	Q96MG2	0.9579
P14079	P51816	0.844
P14079	P78345	1.5053
P14079	Q9Y4K1	1.2574
P14079	O15091	0.18283
P14079	Q99471	0.85964
P14079	Q8N4K4	0.98613
P14079	Q96BR6	0.91027
P14079	Q96JB2	0.96024
P14079	Q86WI3	0.61196
P14079	Q8N8D7	1.2144
P14079	Q6PF04	0.58172
P14079	P42575	0.3597
P14079	Q0PNE2	1.0526
P14079	Q12904	0.34908
P14079	Q9NUS5	0.78027
P14079	O14617	0.28225
P14079	Q96CD2	0.28535
P14079	Q13469	1.0596
P14079	Q2V2M9	0.49226
P14079	Q5VVS0	1.4691
P14079	Q8WZB0	0.1848
P14079	P06703	0.28251
P14079	Q9NZZ3	1.1966
P14079	Q9UKA2	0.44847
P14079	P59074	0.28141
P14079	Q8NEL0	0.39442
P14079	A8MWC7	0.40984
P14079	Q96KF2	1.1715
P14079	Q9Y234	0.73817
P14079	Q93074	0.61282
P14079	Q9BUG6	0.53728
P14079	O00425	0.4506
P14079	Q99081	0.30875
P14079	Q96F05	0.96431
P14079	P58107	0.25262
P14079	P51003	0.4016
P14079	Q9C0G0	0.1949
P14079	Q9BU68	1.3629
P14079	P54098	0.73069
P14079	Q969W9	1.1905
P14079	O43790	1.0109
P14079	Q8IW03	0.36274
P14079	Q5FWE3	1.2485
P14079	Q2NKX9	1.3222
P14079	Q13347	0.84637
P14079	Q8N1N2	1.098
P14079	Q01831	0.89757
P14079	O43242	0.74956
P14079	Q86V48	1.7373
P14079	Q6B0B8	0.94313
P14079	Q03393	0.39044
P14079	Q6ZTR5	0.96725
P14079	Q499Z4	0.61432
P14079	Q8TBC3	0.18591
P14079	A2RRL7	0.95768
P14079	Q8NHV5	0.46842
P14079	O14503	0.91914
P14079	Q8WVI0	1.2916
P14079	Q9Y3B9	0.94247
P14079	Q9Y676	1.2292
P14079	Q96A57	1.2485
P14079	P62826	0.81358
P14079	O43399	0.73148
P14079	P25788	1.1793
P14079	Q15154	0.96547

P14079	Q7L945	0.7079
P14079	P17040	0.74724
P14079	O95361	0.31586
P14079	Q9P2N6	0.85702
P14079	Q8TD10	0.12251
P14079	P63208	0.80456
P14079	P82930	1.4302
P14079	Q9H2M3	0.24202
P14079	Q6IQ32	0.35383
P14079	P98182	0.37529
P14079	Q8N7R7	0.29414
P14079	P09467	0.18009
P14079	Q86VY4	0.51106
P14079	Q96DV4	0.48692
P14079	Q6DN90	0.21773
P14079	P43364	0.986
P14079	Q9GZV8	0.14398
P14079	Q15004	0.99932
P14079	Q9UJT9	0.40806
P14079	Q9BRT7	1.1715
P14079	Q7Z353	0.24936
P14079	Q8TAF3	0.92057
P14079	P0DI81	1.0187
P14079	Q12996	0.24687
P14079	P49116	0.54187
P14079	O75436	0.91783
P14079	Q9BQ83	0.92854
P14079	Q12768	0.61386
P14079	Q92796	0.42346
P14079	Q9H7C9	0.096766
P14079	P07315	0.75753
P14079	Q8TD17	0.49238
P14079	Q5VXH4	0.68944
P14079	P09661	0.65844
P14079	Q5J8M3	0.66992
P14079	O76031	1.2054
P14079	Q969Q0	0.49586
P14079	P02144	0.35657
P14079	O00167	0.45324
P14079	Q6UXB1	1.0714
P14079	Q9BXW7	0.51221
P14079	Q7L1Q6	0.42018
P14079	Q9ULE4	0.84924
P14079	Q9UHD1	0.2552
P14079	Q13465	0.15933
P14079	P04733	0.74382
P14079	B3EWG5	1.3222
P14079	O95260	0.07708
P14079	Q8NCF5	0.53807
P14079	Q3SYG4	1.0405
P14079	P61165	1.2098
P14079	Q14197	0.28117
P14079	Q8N187	1.3571
P14079	Q9H3H1	1.2564
P14079	Q8N5H3	1.3222
P14079	Q9BYR0	1.3698
P14079	Q9UKN5	0.44064
P14079	O14802	0.30928
P14079	A0AVK6	0.23824
P14079	Q9NPJ4	2.1195
P14079	O14576	0.62893
P14079	Q8WYN3	1.0932
P14079	Q9NZN8	0.62429
P14079	O00231	0.91737
P14079	Q13616	0.90417
P14079	Q9H7B2	1.2354
P14079	Q9P2G1	0.47474
P14079	O95273	1.4627
P14079	Q5TA76	1.1715
P14079	Q5T4T6	1.5167
P14079	Q9Y2L8	0.97673
P14079	Q5W111	0.40158
P14079	O00479	1.5162
P14079	Q9H2L4	0.86848
P14079	Q01469	0.23959
P14079	Q96B45	0.53275

P14079	P59090	1.1715
P14079	P13994	0.61194
P14079	P05386	1.3548
P14079	Q53S58	1.2485
P14079	Q9H478	0.6572
P14079	Q96N28	0.52805
P14079	Q5U4N7	1.1715
P14079	Q5VUB5	1.245
P14079	O75505	0.24936
P14079	Q5U5X0	1.5744
P14079	Q8IYB1	0.78965
P14079	Q96GX2	1.3048
P14079	P50402	0.82475
P14079	E5RQL4	0.90413
P14079	A6NK39	0.51953
P14079	O15055	0.2505
P14079	Q9BY89	0.59638
P14079	Q5XKR9	1.4417
P14079	P52701	0.071543
P14079	Q9BT49	1.251
P14079	P23284	0.45927
P14079	Q96SW2	0.53413
P14079	Q6PJP8	0.65361
P14079	Q9NV39	1.1263
P14079	Q8NF91	0.7265
P14079	O15318	1.3734
P14079	P28066	1.0649
P14079	Q53GL0	0.31187
P14079	Q86X67	0.1685
P14079	O95600	0.1949
P14079	P05412	0.69047
P14079	P49588	0.3369
P14079	Q9Y5A9	0.90872
P14079	P35268	1.1731
P14079	Q7L4S7	0.9976
P14079	Q9H7E9	1.3222
P14079	Q8IZS6	0.29307
P14079	Q14929	0.57629
P14079	Q5RGN0	1.2315
P14079	O95486	0.5833
P14079	Q0VD67	0.51792
P14079	O95678	0.96275
P14079	Q6UX04	0.17257
P14079	Q15650	0.89143
P14079	Q9BTK2	0.95937
P14079	P83916	0.92361
P14079	Q96SK2	1.0541
P14079	Q29RF7	1.0435
P14079	Q96N77	0.63598
P14079	Q9Y5P8	0.97899
P14079	Q14232	0.3442
P14079	Q13371	0.60581
P14079	Q5T9G4	1.6704
P14079	Q9BRA2	0.58615
P14079	Q15466	0.52952
P14079	Q494X1	0.85319
P14079	Q12901	0.77985
P14079	Q8WUU5	0.2657
P14079	A4D0Y5	1.1715
P14079	Q5T5S1	0.96683
P14079	Q96JY0	0.34297
P14079	Q9NPB6	0.84441
P14079	Q9H6S1	0.040636
P14079	Q96K80	0.48141
P14079	Q8IWF6	1.103
P14079	Q6NX45	0.54513
P14079	Q13569	1.0831
P14079	Q86VP6	0.59636
P14079	Q53G59	0.36217
P14079	P0CW24	0.63681
P14079	Q9BZX4	0.39663
P14079	Q9Y294	0.97527
P14079	Q8TBQ9	1.2188
P14079	Q9H444	1.0092
P14079	Q86XR8	1.2032
P14079	Q9UL46	0.79475

P14079	Q8WW27	0.41182
P14079	Q92845	0.38197
P14079	P04424	0.22289
P14079	B7ZAP0	0.16319
P14079	Q7Z3B0	1.2485
P14079	P13682	0.60953
P14079	P04792	0.44966
P14079	O15382	0.20063
P14079	Q86XN6	0.7875
P14079	Q96KC9	1.156
P14079	C9JQI7	1.0446
P14079	Q96KQ4	1.0437
P14079	Q9BRG1	1.2337
P14079	A6NJL1	0.433
P14079	Q9NXR1	0.44827
P14079	A6NJR5	1.092
P14079	Q6P589	0.59722
P14079	Q9Y6B2	1.0479
P14079	Q9Y2D8	0.90028
P14079	Q96LB3	0.77709
P14079	Q9NWS6	1.2099
P14079	Q5VV17	0.32224
P14079	Q9BS40	0.72292
P14079	O00244	0.34485
P14079	Q5HYW2	1.4755
P14079	Q9GZY4	0.76052
P14079	P50748	0.97641
P14079	O96020	1.3119
P14079	Q9UHD9	0.89246
P14079	Q2TAP0	1.074
P14079	Q9BSG1	0.89445
P14079	Q9HBF4	0.38968
P14079	Q12888	0.76442
P14079	Q9UEY8	0.36953
P14079	Q9GZN1	0.30313
P14079	Q8N1L1	1.1715
P14079	Q8N1W2	0.41707
P14079	Q96KF7	1.2485
P14079	P0C7T4	1.1715
P14079	Q86VQ1	0.69657
P14079	P0DKX0	0.93942
P14079	Q8NEJ9	0.95563
P14079	Q9BPZ7	1.0698
P14079	Q8NCX0	0.82953
P14079	O96015	0.13595
P14079	Q7Z5G4	0.081494
P14079	A1L4L8	0.9941
P14079	A8MVP8	0.57465
P14079	O75330	0.69157
P14079	P17661	0.86222
P14079	Q8WW35	0.56221
P14079	Q8IYS2	1.2485
P14079	Q96B42	1.2793
P14079	Q5U623	1.0343
P14079	Q9H3H9	1.3282
P14079	Q9Y3R5	1.1776
P14079	O15499	0.17194
P14079	Q8IZ40	0.5655
P14079	A8MYA2	0.84926
P14079	O15084	1.0339
P14079	P62913	1.2436
P14079	Q12769	1.1446
P14079	Q9BW60	0.77014
P14079	P0C7Q3	0.20861
P14079	P15621	0.97355
P14079	O95235	0.26822
P14079	Q9HD40	0.63347
P14079	Q13526	0.66011
P14079	O75794	1.4757
P14079	Q8N9Y4	1.4003
P14079	A6NL82	0.91687
P14079	A2VCL2	0.012635
P14079	Q8IXP5	1.3222
P14079	Q96PC3	0.53718
P14079	Q9H609	0.1949
P14079	P41214	0.96826

P14079	Q9Y6X9	0.30533
P14079	P36551	0.51446
P14079	Q5VZV4	1.3564
P14079	Q9UHJ3	0.4217
P14079	Q9P2F5	0.98034
P14079	Q86Y37	0.18775
P14079	P04259	0.96294
P14079	Q9UH99	0.36872
P14079	P10242	1.0047
P14079	P49368	0.20734
P14079	Q9Y4A8	0.51752
P14079	Q6P1J9	0.65062
P14079	Q9UM07	0.85846
P14079	Q9UJU3	0.45921
P14079	P40925	0.53087
P14079	Q7Z2X4	0.26533
P14079	Q99698	0.33817
P14079	Q5T6F0	0.29528
P14079	Q9Y2D4	0.065533
P14079	Q9H0E2	0.32328
P14079	Q16543	0.51074
P14079	P02545	1.0341
P14079	P56524	0.40413
P14079	Q6P1K8	0.48284
P14079	Q6UXV1	0.9137
P14079	Q5F1R6	0.22664
P14079	Q8TCZ7	1.0031
P14079	A8MYX2	0.29906
P14079	Q15361	0.91731
P14079	O14813	0.3514
P14079	Q14671	0.29662
P14079	Q9H7L9	0.60772
P14079	P51398	1.3991
P14079	Q2YD98	0.85599
P14079	Q7RTP0	1.2835
P14079	Q7RTU3	0.42542
P14079	O60930	0.6524
P14079	Q6BDS2	1.0765
P14079	A6NEQ9	1.1715
P14079	Q9UHN1	0.47606
P14079	Q8TF08	1.0799
P14079	Q96S19	0.67085
P14079	Q15021	0.8373
P14079	Q6ZTY8	0.45451
P14079	Q8IXS8	1.5286
P14079	Q8ND99	0.94323
P14079	Q6PI48	1.313
P14079	Q96MY1	1.567
P14079	P50440	0.79588
P14079	Q96S59	1.0009
P14079	Q13127	0.50538
P14079	Q9P000	0.69689
P14079	Q9H7X2	1.2485
P14079	Q9Y6R9	1.3453
P14079	P53801	1.6318
P14079	Q96AQ2	1.2485
P14079	Q3LI73	1.37
P14079	P99999	0.6508
P14079	Q9NVE4	0.7456
P14079	Q92529	0.31669
P14079	Q6ZWJ1	0.25736
P14079	P0DMB2	0.9729
P14079	Q9Y426	0.86782
P14079	Q9GZR1	0.29531
P14079	Q0VDI3	0.93342
P14079	Q8N6K0	1.2485
P14079	Q09028	0.54109
P14079	O75030	0.4973
P14079	O95411	1.1715
P14079	Q13015	1.1588
P14079	P49789	0.50878
P14079	Q16864	0.25856
P14079	Q9Y2B0	0.26545
P14079	Q52M93	0.44666
P14079	Q9H2T7	0.71681
P14079	H0Y354	1.1532

P14079	P49716	0.39819
P14079	Q6ZQV5	0.77845
P14079	Q8N983	0.81151
P14079	Q96QD9	1.0012
P14079	Q96FX8	1.371
P14079	Q9NQM4	0.37406
P14079	Q86UP9	1.4314
P14079	Q06416	0.28169
P14079	Q6IEE7	1.5917
P14079	Q6ZNG1	0.80079
P14079	Q9BXP5	1.1551
P14079	Q99816	0.51931
P14079	Q96DT7	0.25992
P14079	Q96IT1	0.80141
P14079	Q8WYR4	0.41342
P14079	Q9NVS2	1.3164
P14079	Q9BX66	0.5448
P14079	Q9NRC8	1.1295
P14079	Q9UK61	1.153
P14079	Q6ZSJ8	1.3222
P14079	P25490	0.27672
P14079	O43172	0.61022
P14079	Q86XT9	1.2094
P14079	Q9UM63	0.46138
P14079	Q16514	0.77274
P14079	Q86SH4	1.1715
P14079	P49639	0.22592
P14079	P54840	0.72704
P14079	Q9P2W9	0.43891
P14079	Q504U0	0.61075
P14079	P05387	0.86734
P14079	A6NE21	0.57067
P14079	O95619	0.73892
P14079	Q8N5U0	1.1715
P14079	Q14457	0.74447
P14079	Q9Y530	0.92331
P14079	Q6P2S7	0.45533
P14079	P57060	0.88455
P14079	P0DM63	1.3504
P14079	Q5VXD3	0.38652
P14079	Q5T440	0.75391
P14079	Q96QU8	1.06
P14079	Q9UMY1	1.6598
P14079	Q9Y2G5	0.63446
P14079	P52756	0.2354
P14079	Q5TAB7	1.1416
P14079	Q6ZR93	1.1715
P14079	Q9NRH1	0.75101
P14079	Q8IYL2	1.215
P14079	Q6DHV5	0.74624
P14079	O60238	0.51962
P14079	Q16534	0.67324
P14079	Q9BY60	0.25965
P14079	Q96HE8	1.0454
P14079	O43819	0.51556
P14079	Q2M3W8	1.0323
P14079	Q9BYP8	1.1715
P14079	O75494	0.54516
P14079	Q8N814	1.5319
P14079	Q9UKD2	0.28
P14079	Q9UBX2	0.25626
P14079	B1AL46	1.8041
P14079	O15355	0.83255
P14079	Q6UXD1	1.2485
P14079	Q6ZVN6	0.70081
P14079	O14598	1.445
P14079	Q96HM7	1.4207
P14079	Q9Y536	0.13659
P14079	P50616	0.50727
P14079	Q86XE5	1.167
P14079	Q7Z7A3	0.92707
P14079	Q6PEY1	1.5919
P14079	Q9BUX1	1.0162
P14079	Q9UBQ5	1.0646
P14079	Q9H1D9	0.70875
P14079	P61247	1.2837

P14079	Q6ZRQ5	0.77501
P14079	Q8NFP0	1.0653
P14079	Q5JUW0	0.84162
P14079	Q8WV44	0.29839
P14079	Q5T9Z0	1.0479
P14079	Q53T94	0.52936
P14079	Q8WUH2	0.88995
P14079	Q5T6X4	1.455
P14079	Q6EBC2	1.1894
P14079	Q9H4I2	0.65266
P14079	Q8WUE5	0.44258
P14079	Q15233	0.51822
P14079	O75474	1.8186
P14079	Q8NF67	1.1388
P14079	Q7Z5K2	0.79075
P14079	P28360	0.12926
P14079	Q9Y2I6	0.51416
P14079	Q6ZUI0	0.75474
P14079	Q8TBF5	0.87959
P14079	Q9BSY9	0.92372
P14079	Q8N8R7	1.2929
P14079	Q96MW1	1.3222
P14079	Q6ZS62	1.0604
P14079	Q9BZ98	0.41696
P14079	Q8WTU0	0.58349
P14079	Q6L8G8	1.1715
P14079	Q8WW22	0.57749
P14079	Q8IYA6	1.4195
P14079	P52740	0.50207
P14079	Q3KNT9	1.5131
P14079	Q13813	0.41012
P14079	O43313	0.25342
P14079	P24539	0.23288
P14079	Q9NVX2	0.31898
P14079	Q99547	1.3951
P14079	Q96TC7	1.3844
P14079	Q5BKU6	0.58702
P14079	Q8NFQ8	1.0631
P14079	P35244	0.79307
P14079	Q5UIP0	1.4491
P14079	Q9NTG7	0.5543
P14079	Q9Y221	0.93134
P14079	O15105	0.92638
P14079	Q9BZZ5	0.4731
P14079	Q14140	2.1106
P14079	A6NGK3	1.0162
P14079	P78347	0.4492
P14079	Q5JTC6	1.6336
P14079	Q8NFV5	1.092
P14079	Q8N6V4	0.89445
P14079	Q9UDW3	0.34419
P14079	Q7Z3I7	0.56358
P14079	Q96E14	1.4104
P14079	Q69YN4	0.97769
P14079	Q96HQ0	0.59196
P14079	A6NGY1	1.395
P14079	Q5VWM1	0.88502
P14079	Q9BYQ0	1.3698
P14079	P54105	1.3451
P14079	Q9HAH7	1.1817
P14079	P0CW26	0.86237
P14079	Q92575	1.1286
P14079	Q96BD8	0.82119
P14079	P78524	0.15996
P14079	Q8N1I0	0.30564
P14079	P48059	0.40537
P14079	Q8IZT9	1.532
P14079	A6NFI3	0.50383
P14079	Q86VQ0	0.70174
P14079	Q9BVA0	0.23525
P14079	Q06609	0.4668
P14079	Q69YW2	0.96912
P14079	Q8TEF2	1.1885
P14079	O14901	0.35221
P14079	Q92988	0.46462
P14079	Q86TY3	1.2485

P14079	Q8NB50	0.80079
P14079	O75122	0.41983
P14079	Q8N9G6	1.1938
P14079	Q9Y3I1	1.0138
P14079	Q8TBP5	1.2485
P14079	Q8WYA1	0.30398
P14079	Q9Y6X8	0.63663
P14079	Q07021	1.1692
P14079	O00470	0.46739
P14079	Q14683	0.53802
P14079	O00267	1.1843
P14079	Q9NW38	0.97455
P14079	P62318	0.83696
P14079	P0CG47	0.68371
P14079	Q6ZQN3	1.5648
P14079	Q3LI76	1.3568
P14079	A6NCN8	1.3222
P14079	Q32P44	0.25965
P14079	Q96ME1	0.3778
P14079	Q9H489	0.51865
P14079	Q9UN36	0.43896
P14079	P52736	0.67364
P14079	Q9NVP1	0.10046
P14079	Q86SZ3	0.71601
P14079	Q8IY50	1.1271
P14079	Q96FQ7	0.52179
P14079	P56555	1.5466
P14079	P54727	0.78942
P14079	Q17RQ9	0.91808
P14079	Q6S8J3	0.96316
P14079	Q6ZUB0	1.2485
P14079	Q9HAA7	0.49115
P14079	Q9NYK5	0.76971
P14079	Q8N616	1.1715
P14079	O14965	0.33962
P14079	Q9UQ80	0.93404
P14079	Q5T1B0	0.13008
P14079	Q6P9A1	0.92077
P14079	Q15935	0.6303
P14079	Q5TKA1	0.54465
P14079	P81277	0.50785
P14079	Q5JRM2	1.0613
P14079	Q14582	0.38342
P14079	Q9NX65	0.45326
P14079	O95780	1.0602
P14079	Q9Y5L0	1.1151
P14079	Q96Q83	0.74287
P14079	P02549	0.31687
P14079	Q86TS7	1.1715
P14079	Q6UWH4	1.3408
P14079	Q9NXF1	0.66658
P14079	O95257	0.70637
P14079	P62269	1.0666
P14079	Q86VR2	1.2942
P14079	Q9H2J4	1.0518
P14079	Q9ULJ6	0.50195
P14079	Q96MH2	1.1717
P14079	Q86WR7	1.5091
P14079	P21673	0.55294
P14079	Q96PS1	1.1146
P14079	Q8N7S6	1.1715
P14079	Q5TCZ1	0.39444
P14079	O94776	1.1148
P14079	A6NHS1	1.3601
P14079	Q6PH85	0.20994
P14079	Q9H0B6	0.93946
P14079	Q86YD7	0.52751
P14079	P0C7W0	1.3222
P14079	O15151	0.46361
P14079	Q9UJC5	0.95503
P14079	Q8WYA6	0.97385
P14079	Q9H4A6	0.97652
P14079	Q96E16	1.3579
P14079	Q9UNS1	0.45634
P14079	Q6PIZ9	1.0881
P14079	P69892	0.65657

48717	P14079	Q9NRX6	0.60686
48718	P14079	Q9Y4Y9	0.82907
48719	P14079	Q8N0V1	1.0577
48720	P14079	Q9NR77	0.99614
48721	P14079	Q6ZMS4	0.54886
48722	P14079	Q9P1Z2	1.0392
48723	P14079	P61578	1.1515
48724	P14079	Q9UPY6	0.28326
48725	P14079	Q9NVD7	0.53488
48726	P14079	Q96LU7	1.028
48727	P14079	P62701	0.62217
48728	P14079	P0CJ76	1.3031
48729	P14079	E9PAV3	0.7974
48730	P14079	Q9BYZ2	0.60641
48731	P14079	Q9Y573	0.19215
48732	P14079	O75969	0.66724
48733	P14079	Q9BQQ7	0.54081
48734	P14079	Q13287	0.56868
48735	P14079	Q7Z2Z1	1.441
48736	P14079	Q9UJ78	0.46997
48737	P14079	Q17RA5	1.1715
48738	P14079	Q6EEV6	0.22082
48739	P14079	Q9BRK4	0.83379
48740	P14079	Q2NKJ3	1.0156
48741	P14079	Q6UN15	1.7325
48742	P14079	Q92622	0.53802
48743	P14079	P18583	0.42296
48744	P14079	Q9Y239	0.28448
48745	P14079	Q9NRX3	1.2495
48746	P14079	P36954	0.2975
48747	P14079	Q9H7U1	1.6767
48748	P14079	P51826	0.6984
48749	P14079	O96006	1.4929
48750	P14079	Q9NWQ4	1.3098
48751	P14079	Q7Z2E3	0.62573
48752	P14079	P17030	0.60452
48753	P14079	Q8N808	1.2643
48754	P14079	Q5VYV7	0.56221
48755	P14079	Q7Z4W3	1.1715
48756	P14079	Q12857	0.40943
48757	P14079	Q8IYX8	0.34309
48758	P14079	Q9UHB9	0.50433
48759	P14079	Q15274	0.46804
48760	P14079	H3BQW9	1.2292
48761	P14079	Q8IUI4	0.70324
48762	P14079	Q9H5H4	0.5795
48763	P14079	P36956	0.2924
48764	P14079	O43617	0.58443
48765	P14079	Q9ULV5	0.38365
48766	P14079	P36871	0.059046
48767	P14079	Q96LM5	0.031437
48768	P14079	Q53TQ3	0.94572
48769	P14079	O75616	0.87
48770	P14079	A6NGB0	1.2485
48771	P14079	O14879	0.64608
48772	P14079	E9PB15	0.6674
48773	P14079	A8MWP4	0.86626
48774	P14079	Q96GY3	1.0215
48775	P14079	Q9Y5A7	0.8753
48776	P14079	Q9UQC2	0.43364
48777	P14079	Q6QNY1	1.0022
48778	P14079	O43151	1.0447
48779	P14079	Q9Y236	0.30349
48780	P14079	Q4VC39	0.8325
48781	P14079	Q6PII3	1.1705
48782	P14079	Q6URK8	1.0272
48783	P14079	Q9P2Y5	0.71445
48784	P14079	Q01094	0.79105
48785	P14079	Q5NDL2	0.38766
48786	P14079	Q13352	0.61244
48787	P14079	Q16612	0.31255
48788	P14079	Q9UBP8	0.9498
48789	P14079	Q9H902	0.23906
48790	P14079	Q9H5Z6	1.3222
48791	P14079	O00483	0.38303
48792	P14079	O75935	1.0307

48793	P14079	Q86X95	1.7529
48794	P14079	Q9Y5B6	1.4104
48795	P14079	Q5T1B1	1.7345
48796	P14079	Q504Q3	0.10722
48797	P14079	O60443	0.62947
48798	P14079	A6NFD8	0.255
48799	P14079	Q9H568	0.70001
48800	P14079	Q8N4C9	1.2485
48801	P14079	Q969P6	0.78697
48802	P14079	Q8NEG0	0.90331
48803	P14079	Q5T5A4	0.94754
48804	P14079	A6NDR6	0.24936
48805	P14079	Q7L2Z9	0.79627
48806	P14079	Q6DCA0	0.70001
48807	P14079	Q9P2S5	0.2601
48808	P14079	P53701	1.1115
48809	P14079	Q14678	0.49494
48810	P14079	Q9C0D2	1.5293
48811	P14079	Q8N912	1.4726
48812	P14079	Q96MT3	0.86795
48813	P14079	Q96G25	0.95487
48814	P14079	Q9Y224	1.6052
48815	P14079	P86452	0.84777
48816	P14079	Q9Y5J9	0.97706
48817	P14079	P0CG38	0.99952
48818	P14079	Q5HYW3	1.7339
48819	P14079	Q92585	1.0491
48820	P14079	O60765	0.68218
48821	P14079	Q8N228	0.38002
48822	P14079	Q7RTU4	0.42542
48823	P14079	O75486	1.0316
48824	P14079	Q9Y2Z0	0.49088
48825	P14079	O00255	0.39266
48826	P14079	Q96HA4	0.82292
48827	P14079	O76042	1.1521
48828	P14079	Q9NUN5	0.96178
48829	P14079	A6NK58	0.58354
48830	P14079	P83111	0.14665
48831	P14079	P61575	1.355
48832	P14079	Q8N8C0	0.61722
48833	P14079	Q9NQC7	0.18479
48834	P14079	Q9H496	0.91283
48835	P14079	O95900	1.2936
48836	P14079	Q6ZS17	0.74576
48837	P14079	Q2VPJ9	1.3222
48838	P14079	Q96KS0	0.32306
48839	P14079	Q8WUF8	0.97691
48840	P14079	Q8N6I1	1.4032
48841	P14079	P15408	0.53462
48842	P14079	Q580R0	0.4117
48843	P14079	Q9ULK2	1.0973
48844	P14079	Q6PEV8	0.24266
48845	P14079	Q9UBY9	1.028
48846	P14079	P43355	1.0517
48847	P14079	Q3LI68	1.6908
48848	P14079	Q6ZV70	0.74121
48849	P14079	O94868	0.56221
48850	P14079	O14933	0.50939
48851	P14079	Q96C12	0.041588
48852	P14079	P61244	0.79362
48853	P14079	Q9HC77	1.211
48854	P14079	Q5JQC4	1.1715
48855	P14079	A6NLX4	1.2485
48856	P14079	Q6UWD8	0.94074
48857	P14079	O60884	0.58981
48858	P14079	Q9HBQ8	1.4427
48859	P14079	Q17R31	0.49931
48860	P14079	A6NNP5	0.20695
48861	P14079	Q13951	0.73049
48862	P14079	Q9ULE0	0.29482
48863	P14079	Q9BZQ8	1.311
48864	P14079	P63313	1.1711
48865	P14079	Q96CN0	1.1715
48866	P14079	Q8IYW2	1.1715
48867	P14079	Q8N7H5	0.88688
48868	P14079	P51580	0.67068

P14079	A6XGL0	0.87949
P14079	P08727	0.99997
P14079	Q15940	0.88249
P14079	Q8TEB1	0.37638
P14079	Q9NW07	0.55049
P14079	Q9NRA8	1.2586
P14079	P0C7I6	1.3224
P14079	Q9NX31	0.30749
P14079	O75971	1.8063
P14079	Q8TF63	1.3902
P14079	O15480	0.081932
P14079	Q15628	0.85695
P14079	Q96M11	1.783
P14079	Q6TDU7	0.93752
P14079	Q53S33	1.5781
P14079	Q9H3Q1	0.53442
P14079	Q13145	0.59749
P14079	Q96N11	0.96974
P14079	Q9BXV9	1.1715
P14079	O15259	0.40028
P14079	Q96FT9	0.54383
P14079	O14908	0.26554
P14079	Q9H6K1	0.53811
P14079	Q5SRE5	0.98304
P14079	P04818	0.29906
P14079	O14618	0.44614
P14079	Q02080	0.79005
P14079	Q06265	0.37119
P14079	Q9UEU5	0.53923
P14079	Q9NRG9	0.82786
P14079	A2RUT3	1.344
P14079	P0CW23	1.0342
P14079	Q9Y4J8	0.30054
P14079	Q8N7B7	0.58518
P14079	Q96EY4	1.0971
P14079	O14523	1.3259
P14079	Q6ZVC0	1.4801
P14079	Q5HYK3	0.92098
P14079	Q8WW14	0.59037
P14079	Q9H8M7	1.0018
P14079	Q9NSB2	0.94437
P14079	Q96DM3	0.72814
P14079	P51571	0.7016
P14079	Q96KT0	0.95937
P14079	Q30KQ6	0.91101
P14079	Q8IW35	1.5022
P14079	Q9GZV4	0.75544
P14079	P01100	0.63772
P14079	Q4W4Y0	0.93518
P14079	Q9UJ68	0.44542
P14079	Q8NE65	0.91121
P14079	Q96PZ2	1.0953
P14079	Q9P267	0.58195
P14079	P10075	0.52953
P14079	O75153	0.2893
P14079	Q8TDI8	0.94171
P14079	Q9NQ40	1.2062
P14079	H3BTG2	1.2331
P14079	O43395	0.92992
P14079	P61313	1.2548
P14079	Q5TBC7	0.57328
P14079	Q9UBT6	0.38564
P14079	Q9BRU2	0.52899
P14079	P12035	0.98252
P14079	P78412	0.24936
P14079	Q8N812	1.4217
P14079	P63146	0.56404
P14079	O15273	0.33372
P14079	Q9H816	0.64409
P14079	Q8N7Q3	0.80886
P14079	O95625	0.40943
P14079	A8MTZ0	1.2008
P14079	P0DL12	1.0595
P14079	Q5VT03	1.8041
P14079	O95218	0.61067
P14079	Q5T5A8	1.1715

P14079	Q9BQ13	0.18591
P14079	Q96G28	0.60862
P14079	Q719I0	0.58864
P14079	Q9BYT5	0.88786
P14079	Q96FX7	0.50331
P14079	O75683	1.1414
P14079	Q9NP71	0.37373
P14079	Q8WZ71	1.2289
P14079	Q9NZW5	0.2686
P14079	Q9Y376	0.24404
P14079	Q16342	0.23145
P14079	O15234	1.5056
P14079	Q5JSJ4	1.8235
P14079	Q9BXW4	0.43453
P14079	P0CL84	0.46781
P14079	Q9NZB2	1.4239
P14079	P42695	0.42114
P14079	Q96EZ8	0.77469
P14079	O75694	0.70035
P14079	Q5TDH0	0.38319
P14079	O14599	1.1715
P14079	Q14188	0.69755
P14079	Q9HAT2	0.9994
P14079	Q10586	0.41112
P14079	Q96SN8	0.77329
P14079	Q9BY65	1.2746
P14079	Q8IUM7	0.27737
P14079	P23769	0.41158
P14079	O60716	0.3233
P14079	Q5T653	1.2117
P14079	Q9UIG5	0.30168
P14079	Q9H7Y2	1.0738
P14079	Q8WVB3	0.096538
P14079	O14681	1.4529
P14079	P50502	0.71713
P14079	O75717	0.57211
P14079	Q96C03	1.2573
P14079	P14406	0.83234
P14079	P23511	1.1288
P14079	Q96M34	0.63065
P14079	Q8IYS4	0.577
P14079	Q86Y97	0.23361
P14079	Q9BTC0	0.35575
P14079	O95473	0.8439
P14079	Q56VL3	1.1262
P14079	Q8TAF5	1.1715
P14079	Q9NVN3	0.37442
P14079	Q8NEF9	0.61376
P14079	Q96H40	0.97758
P14079	Q9H4R4	0.30003
P14079	Q9P287	0.78489
P14079	Q5UE93	0.9255
P14079	Q9NV96	0.32918
P14079	O15360	1.0132
P14079	Q86X60	0.96022
P14079	Q9HB75	0.85994
P14079	P03901	0.87522
P14079	Q08209	0.40183
P14079	Q6ZU65	0.83977
P14079	P14316	0.89714
P14079	Q9NXX6	0.52703
P14079	O00629	0.7272
P14079	Q8WWW0	0.70331
P14079	O60232	1.7258
P14079	Q9H974	1.77
P14079	Q8NCQ5	0.24772
P14079	P0CG24	0.58926
P14079	O60810	1.0087
P14079	Q8NA61	0.31561
P14079	Q8WTQ4	0.70852
P14079	Q8N8D0	1.1715
P14079	Q7L1V2	1.4935
P14079	Q5T7V8	2.2431
P14079	P68133	0.99952
P14079	P82663	1.2209
P14079	Q3BBV2	1.2315

P14079	Q15438	0.29933
P14079	Q9H300	0.88713
P14079	P53680	0.285
P14079	Q06250	1.1715
P14079	P04280	0.9456
P14079	Q9BZK8	1.1715
P14079	Q86SY8	1.1715
P14079	Q01658	0.50564
P14079	P03886	0.52548
P14079	O43593	0.15936
P14079	Q969S3	0.88076
P14079	Q96AQ1	1.4698
P14079	P25205	0.40918
P14079	Q96G04	0.16974
P14079	Q9NZJ9	0.42934
P14079	Q494R4	0.015
P14079	Q9NR33	0.22047
P14079	P52292	1.2387
P14079	O43734	1.3222
P14079	Q9NQG5	1.1221
P14079	Q14152	1.2128
P14079	Q9NQX6	0.60526
P14079	P78537	1.3442
P14079	Q7L0Y3	1.2327
P14079	Q9BZE0	0.23869
P14079	O75382	0.54243
P14079	Q16401	1.1304
P14079	Q8TD16	0.42765
P14079	Q92615	0.93817
P14079	P0CAP2	0.95944
P14079	Q96CV9	0.70345
P14079	Q9Y5U2	1.2745
P14079	Q13426	0.45686
P14079	P59047	0.25552
P14079	Q5SSG8	1.0613
P14079	Q8TAB7	1.3784
P14079	Q4W5G0	0.83593
P14079	Q9HBE1	0.43665
P14079	O43761	1.0406
P14079	Q9UKW6	0.41417
P14079	Q9UQ84	0.23136
P14079	P10144	0.33581
P14079	Q05932	0.55322
P14079	P48595	0.15934
P14079	Q494V2	0.29766
P14079	Q6P1W5	1.1201
P14079	Q9NXB0	1.2914
P14079	Q96PV7	1.5091
P14079	Q96BP3	0.17474
P14079	Q9UJX4	0.98825
P14079	Q8NET1	1.0712
P14079	Q96M60	1.0841
P14079	Q8IWF9	0.74167
P14079	Q7Z589	1.6808
P14079	Q5H9R7	1.486
P14079	Q9H9D4	0.72847
P14079	Q96T76	1.0157
P14079	Q9NUD7	0.50384
P14079	Q13113	0.95129
P14079	Q96I23	0.425
P14079	Q8N3Z6	0.36455
P14079	Q8WXD5	0.88465
P14079	Q9NQR4	0.182
P14079	Q9ULW6	0.46579
P14079	Q8TD47	0.42121
P14079	Q9Y3S2	0.99113
P14079	Q9BYV8	1.0174
P14079	P53990	1.0291
P14079	Q9Y5L4	0.18193
P14079	Q9BV57	0.48759
P14079	Q5VV11	0.72163
P14079	Q14686	1.3606
P14079	Q8NG41	0.62495
P14079	Q5T0W9	0.62653
P14079	Q2M2W7	1.3222
P14079	Q15542	1.0481

P14079	Q8NDT2	0.93071
P14079	Q9H063	1.0257
P14079	A6NFR6	0.79017
P14079	Q8TAE6	1.0405
P14079	Q5VWG9	0.80351
P14079	P17405	0.40576
P14079	Q5FWF6	0.98476
P14079	Q6MZM9	1.1715
P14079	P0C7T8	1.2485
P14079	Q9UHF7	0.57559
P14079	Q96C10	0.27214
P14079	Q96CG3	0.70329
P14079	P41970	1.0851
P14079	Q14999	1.048
P14079	Q8WY98	0.83696
P14079	O75956	0.86418
P14079	O60921	0.95343
P14079	P49711	0.2757
P14079	Q92567	1.2766
P14079	Q9BV44	0.60174
P14079	O94842	0.79153
P14079	P54920	0.61622
P14079	Q9H9L4	0.86209
P14079	Q9NQR7	1.1715
P14079	B2RC85	0.12268
P14079	Q9NQS1	1.1436
P14079	P17980	0.69287
P14079	Q6NXP2	1.8257
P14079	P78318	1.2612
P14079	O15327	0.29326
P14079	Q8TDG2	0.96178
P14079	Q9Y5M1	0.94864
P14079	Q8N3U1	1.1715
P14079	Q9NXK8	0.73761
P14079	P0CJ71	1.3031
P14079	Q92688	0.28036
P14079	Q15022	0.74446
P14079	O75496	1.1102
P14079	Q9H693	1.0439
P14079	O75901	0.89717
P14079	P0C7T5	0.86422
P14079	Q15326	1.1825
P14079	Q6IQ19	1.0173
P14079	Q8NEY8	1.4341
P14079	Q8WV15	1.2485
P14079	Q9Y2W1	1.1981
P14079	Q96DE0	1.7427
P14079	Q3YBM2	1.6651
P14079	P18754	1.3321
P14079	Q9Y6F1	0.37578
P14079	P86481	1.1715
P14079	Q03154	0.51173
P14079	Q9NPA8	0.58928
P14079	Q96EL2	0.48542
P14079	Q96K31	1.1415
P14079	Q96N95	0.36857
P14079	Q9NZL4	0.89153
P14079	Q8NE18	1.6831
P14079	Q96GN5	1.129
P14079	Q8TC21	0.60926
P14079	Q8NHR9	0.29665
P14079	Q9BZW4	1.4221
P14079	Q9NQ92	0.8653
P14079	O15131	1.3085
P14079	Q96EY1	1.0003
P14079	P78358	1.3978
P14079	O60304	0.54607
P14079	Q6ZPD9	0.85783
P14079	Q69YW0	0.75607
P14079	Q8N111	1.0085
P14079	Q8N0Y7	0.86177
P14079	O14777	0.71284
P14079	Q96PK6	0.69306
P14079	P50222	0.45113
P14079	P07814	0.90646
P14079	P0DJI8	0.9033

49173	P14079	Q9BS18	1.1557
49174	P14079	P17023	0.53698
49175	P14079	O94993	0.25139
49176	P14079	A6NC51	0.91469
49177	P14079	Q8NFJ8	0.47544
49178	P14079	Q6ZN17	1.1761
49179	P14079	Q9C0E2	1.0412
49180	P14079	Q9UHD4	1.0828
49181	P14079	Q7Z624	0.65124
49182	P14079	Q5TEA3	0.92792
49183	P14079	Q9UI66	1.1715
49184	P14079	Q9P2D0	0.31138
49185	P14079	Q86UB9	1.4779
49186	P14079	Q96C90	0.97829
49187	P14079	A1L4Q6	0.6655
49188	P14079	O95202	0.64075
49189	P14079	Q9NUQ9	0.16973
49190	P14079	Q96PI1	1.4673
49191	P14079	Q04727	0.56344
49192	P14079	Q99836	0.26126
49193	P14079	O75177	1.2692
49194	P14079	O00743	0.8056
49195	P14079	Q969W8	0.92268
49196	P14079	Q86TJ2	0.56769
49197	P14079	P07741	0.83577
49198	P14079	Q8WUA4	0.32842
49199	P14079	Q86YD1	1.2708
49200	P14079	Q9Y2V2	0.74468
49201	P14079	Q8WYH8	0.46104
49202	P14079	Q16520	0.91566
49203	P14079	Q9NZH5	0.87682
49204	P14079	Q14203	1.2742
49205	P14079	Q9UJ94	1.1715
49206	P14079	Q9H3F6	0.30734
49207	P14079	A6NK44	0.39752
49208	P14079	O43324	1.2237
49209	P14079	Q86UQ5	1.1715
49210	P14079	Q9Y462	0.8349
49211	P14079	Q8IY63	0.49732
49212	P14079	Q9H5J4	0.77014
49213	P14079	Q8IYF3	0.62499
49214	P14079	P30047	0.73534
49215	P14079	Q8NFT6	0.32605
49216	P14079	Q9BY31	0.4226
49217	P14079	Q9H9F9	1.1207
49218	P14079	Q9UGU0	0.41998
49219	P14079	O95050	0.013212
49220	P14079	Q9BRX5	0.51585
49221	P14079	Q8IY51	0.18624
49222	P14079	Q96B86	0.91194
49223	P14079	Q12893	1.0673
49224	P14079	Q5H9J7	1.4257
49225	P14079	Q9P219	0.41225
49226	P14079	Q8IXM6	1.3508
49227	P14079	Q8WUZ0	1.3152
49228	P14079	O15144	0.58107
49229	P14079	Q96CN9	0.89744
49230	P14079	Q5SV97	1.3928
49231	P14079	Q8IWB1	0.97642
49232	P14079	Q8WW38	0.59341
49233	P14079	Q2KHR3	0.47117
49234	P14079	Q6UWI2	1.3063
49235	P14079	Q5VWK0	0.18717
49236	P14079	Q5VXU9	0.26969
49237	P14079	Q2T9L4	0.79289
49238	P14079	P47928	0.57993
49239	P14079	P48539	0.78118
49240	P14079	Q9UJT2	1.2581
49241	P14079	Q96DP5	1.1415
49242	P14079	P0C2S0	1.1353
49243	P14079	Q8IVY1	1.1823
49244	P14079	Q5JR98	0.99094
49245	P14079	Q9UBM8	0.82214
49246	P14079	P02729	1.1715
49247	P14079	Q9NQE9	0.30614
49248	P14079	O94850	1.2904

P14079	Q8TAX9	0.45181
P14079	P0CG39	0.96316
P14079	Q8IZT6	0.44773
P14079	Q96PB1	1.382
P14079	Q9Y2B1	1.2485
P14079	P62851	1.3924
P14079	P27816	0.73656
P14079	Q5VU36	1.3668
P14079	Q8N7U9	0.47868
P14079	Q9HCL3	1.0178
P14079	Q9H788	1.0563
P14079	Q8IYA8	1.3222
P14079	O43684	0.87238
P14079	Q8N688	0.7885
P14079	Q8TAU3	0.61621
P14079	P07992	0.13874
P14079	Q6P1L8	1.3656
P14079	Q9NVS9	0.70574
P14079	Q32M84	0.44343
P14079	Q49AQ9	0.80567
P14079	P0C6E6	0.35338
P14079	Q9BTM9	1.2887
P14079	Q8TCS8	0.42442
P14079	Q9NZU7	0.54708
P14079	Q9BTX1	1.1942
P14079	Q86WW8	1.0085
P14079	P37275	0.74765
P14079	A6NLC5	1.0773
P14079	Q8NEM1	1.0245
P14079	Q8N7C4	1.1715
P14079	P35241	0.30182
P14079	Q96S06	1.2107
P14079	Q07352	0.87138
P14079	Q9UGP4	0.45652
P14079	Q969G5	0.90632
P14079	Q53QV2	1.0452
P14079	Q7Z5W3	1.0929
P14079	O14490	0.44078
P14079	Q8TC71	0.88532
P14079	Q96FW1	0.5784
P14079	Q9H6K4	1.1856
P14079	Q92925	0.55839
P14079	Q5T013	0.60157
P14079	Q8TDC0	1.2628
P14079	Q6J272	1.6338
P14079	P28072	0.98613
P14079	Q9BSM1	0.98923
P14079	Q96M85	1.1715
P14079	Q04323	0.39913
P14079	P0C7V6	1.1571
P14079	Q99619	0.33959
P14079	P19447	0.17153
P14079	Q3SY56	0.22134
P14079	Q9UMS5	1.6062
P14079	Q8N1Q8	0.98341
P14079	Q9Y535	0.45833
P14079	Q7Z2V1	1.1715
P14079	Q8TAV0	1.5675
P14079	Q8N3J3	1.1154
P14079	Q8NC69	0.18591
P14079	Q6UXP9	0.7104
P14079	Q9BWX1	0.29221
P14079	Q96NS1	0.50693
P14079	O95751	1.3101
P14079	Q9UGR2	0.8057
P14079	Q9BYQ8	1.3698
P14079	O43542	0.5359
P14079	Q8WTS6	0.27252
P14079	Q8WZA0	0.51671
P14079	Q8TAD1	1.0968
P14079	A6NGU7	0.85092
P14079	Q86XK3	1.5029
P14079	O95391	0.52634
P14079	Q9Y657	0.54786
P14079	Q9P0K7	0.31337
P14079	Q15849	0.9101

P14079	Q9UNH5	0.66839
P14079	Q9NWV4	1.3425
P14079	P39687	1.2895
P14079	Q9BU19	0.1949
P14079	Q9H501	1.86
P14079	B7Z6K7	0.53821
P14079	Q8N8F7	1.2485
P14079	Q9UIY3	0.74433
P14079	Q5J8X5	1.2485
P14079	Q96A59	1.1211
P14079	P49411	0.76812
P14079	O60308	0.89162
P14079	Q96AP0	0.70534
P14079	Q9UBU7	0.40821
P14079	Q96EY9	0.75258
P14079	Q96KN7	0.249
P14079	Q6AZY7	0.7893
P14079	P46108	0.50407
P14079	Q3MUY2	1.0803
P14079	P0C7T7	1.1715
P14079	Q09161	0.89826
P14079	P05388	1.5593
P14079	O43805	0.86603
P14079	Q5T2D3	0.16521
P14079	A8MQ14	0.64109
P14079	Q6P1M3	1.0859
P14079	Q9Y2G9	1.2728
P14079	P11441	0.66019
P14079	O60927	1.3662
P14079	P17535	0.99069
P14079	P0C7X0	0.13295
P14079	Q8IUD6	0.22805
P14079	Q14587	0.50207
P14079	Q674X7	0.60051
P14079	Q9Y608	0.44446
P14079	Q8N766	0.90574
P14079	Q53EZ4	0.91616
P14079	Q86WS4	1.1715
P14079	Q6ZSB9	0.48782
P14079	Q04637	0.67817
P14079	Q09FC8	0.63549
P14079	Q9NX78	0.9887
P14079	O75459	1.0766
P14079	Q5JX71	1.0613
P14079	P31483	0.4127
P14079	Q5JYT7	0.29793
P14079	P09496	0.26061
P14079	O14628	0.99076
P14079	P50221	0.34143
P14079	Q9NSC2	0.36055
P14079	P0CJ85	0.25626
P14079	Q9BRR3	0.80054
P14079	Q5SXJ2	1.9823
P14079	Q99550	0.69042
P14079	Q9Y6X4	1.8063
P14079	P22626	0.43549
P14079	Q14019	0.43264
P14079	Q8N7K0	0.77845
P14079	P46020	0.58361
P14079	Q9UFM2	0.92038
P14079	Q15390	0.89576
P14079	Q96MF4	1.1689
P14079	Q8N8E2	0.28627
P14079	Q8NI51	0.6408
P14079	Q7Z7A1	1.5113
P14079	Q9BSF0	0.74122
P14079	Q99767	0.45309
P14079	P07305	0.39419
P14079	Q6IV72	0.53255
P14079	Q14CZ0	2.0415
P14079	Q8NFR9	1.3079
P14079	Q3B7T3	0.95483
P14079	Q8NCR6	1.0796
P14079	Q13619	0.57252
P14079	Q5H9L2	1.0823
P14079	Q9NVT9	0.5743

P14079	Q15643	1.2878
P14079	Q5SRR4	1.0431
P14079	Q8NBF1	0.50773
P14079	Q1AE95	1.1391
P14079	Q9NRP4	0.040861
P14079	O00522	0.4172
P14079	A6NM28	0.57412
P14079	Q5T953	1.566
P14079	Q5VTL8	1.5092
P14079	Q96K17	0.9967
P14079	A6NEY3	1.4427
P14079	Q8N183	0.4383
P14079	Q53QW1	1.3222
P14079	Q9Y587	0.23675
P14079	O15488	0.25288
P14079	Q96N58	0.63215
P14079	O14524	1.0244
P14079	Q8N8A2	0.56221
P14079	Q9H6F2	0.81473
P14079	Q96IP4	1.041
P14079	Q96C74	0.48695
P14079	O76009	0.88619
P14079	Q3SXR2	1.0048
P14079	O60763	0.65928
P14079	P13051	1.7343
P14079	P10914	0.43127
P14079	P22674	0.60056
P14079	Q9H160	0.43867
P14079	O43236	0.34542
P14079	Q2M3D2	0.50714
P14079	Q86X83	1.0047
P14079	P84103	0.69899
P14079	Q9UN30	0.20516
P14079	P0C841	1.1715
P14079	Q9ULJ3	0.73947
P14079	P59861	0.91101
P14079	Q8NHZ7	0.91772
P14079	Q6NUN7	1.1715
P14079	Q7Z398	0.58326
P14079	Q6ZSA8	1.1715
P14079	P29083	1.4132
P14079	Q5SZD1	1.0363
P14079	Q96NJ6	0.55736
P14079	Q5THK1	0.62789
P14079	Q13342	0.67034
P14079	Q86SE9	1.0622
P14079	Q6P597	0.35092
P14079	Q5SNT6	1.3964
P14079	Q9Y3P8	1.1766
P14079	A6NGC4	1.2485
P14079	Q8IWZ8	0.20168
P14079	Q8NAA6	0.38693
P14079	Q9H267	0.43954
P14079	Q8IY92	0.53104
P14079	Q8IYS1	0.37516
P14079	O43257	0.92402
P14079	O95865	0.42125
P14079	Q9HA58	1.1715
P14079	Q8N0U2	1.1715
P14079	P60368	1.1184
P14079	Q9UK59	0.48061
P14079	O15534	0.27529
P14079	Q6NSW7	0.28169
P14079	Q02446	0.17327
P14079	P61925	0.24433
P14079	Q96NF6	0.79957
P14079	Q8NEG7	0.94318
P14079	Q92993	0.78229
P14079	Q8TCY9	1.4147
P14079	Q4G0Z9	1.1602
P14079	P19419	1.4312
P14079	O94927	0.74419
P14079	P0CE67	1.1715
P14079	O60224	0.31443
P14079	Q8TF47	0.9897
P14079	Q14690	0.5416

P14079	P49917	0.088548
P14079	O75170	1.2288
P14079	Q9Y222	0.83457
P14079	Q9BU61	0.83674
P14079	O15014	1.7517
P14079	Q9BYQ5	1.3698
P14079	O94854	0.37444
P14079	Q7Z7K6	1.1257
P14079	Q9H714	0.86906
P14079	Q9H999	0.95045
P14079	Q6ZR85	1.1715
P14079	Q08AF8	0.95714
P14079	Q9H089	0.38984
P14079	Q9HAB8	1.2931
P14079	Q99551	0.90561
P14079	Q8WWH5	1.054
P14079	Q63HM2	1.0148
P14079	Q13114	0.51165
P14079	Q27J81	0.31443
P14079	P55957	0.7724
P14079	Q9UQN3	0.67368
P14079	P21675	0.42258
P14079	Q9UPQ9	0.81704
P14079	Q9Y2K5	0.872
P14079	A6NHP3	1.092
P14079	Q9NPA0	0.972
P14079	P13647	1.0576
P14079	Q8N6G2	0.031863
P14079	Q96KN8	0.012626
P14079	Q8TAT8	0.93818
P14079	O00268	1.1166
P14079	C9JXX5	0.98371
P14079	Q5VU43	1.2821
P14079	Q16666	0.61225
P14079	Q8IUR6	1.2406
P14079	Q9HBM6	0.64795
P14079	Q9BR09	0.55714
P14079	P01858	1.1715
P14079	Q8WVF5	0.18591
P14079	P30622	0.53773
P14079	Q659A1	1.0193
P14079	Q86X10	0.82063
P14079	P39748	0.26791
P14079	P14651	0.15955
P14079	Q5TGY3	0.85254
P14079	P59991	1.4458
P14079	P38159	0.70663
P14079	Q68EN5	0.75145
P14079	Q8IZ26	0.60675
P14079	Q9NS73	0.83522
P14079	Q9UIF8	0.86433
P14079	Q5CZA5	0.72218
P14079	P49901	1.1715
P14079	Q96I27	0.55399
P14079	Q9H116	0.62194
P14079	Q13438	0.086056
P14079	Q96LZ7	0.29703
P14079	Q96L11	1.0694
P14079	Q6P1K2	1.1203
P14079	Q9UKT9	0.45512
P14079	Q9NV64	0.9257
P14079	A6NEF3	1.4427
P14079	P58340	0.29421
P14079	B1AH88	1.1715
P14079	Q64ET8	1.4746
P14079	Q96BW1	0.55137
P14079	Q96CW5	1.3221
P14079	P52948	1.1774
P14079	O75113	1.0336
P14079	P34896	0.35304
P14079	Q9NQ55	0.75934
P14079	P13693	0.15486
P14079	Q9BYG8	0.62864
P14079	A1L170	1.1715
P14079	Q9H606	1.0403
P14079	Q6IQ21	0.75178

P14079	Q6NW34	1.0613
P14079	Q6NX49	0.53332
P14079	Q14978	1.3241
P14079	Q3L8U1	0.6457
P14079	P0CG13	0.59217
P14079	Q9ULD0	0.2653
P14079	Q17RB0	1.2815
P14079	Q99750	0.99421
P14079	Q8NDV7	0.6329
P14079	O95298	1.2593
P14079	Q8N6V9	0.2666
P14079	Q96GC6	1.1189
P14079	Q96T58	0.7015
P14079	O75970	0.34015
P14079	B7Z1M9	0.17796
P14079	Q5M9N0	0.023846
P14079	Q96EH5	1.2526
P14079	Q53EL6	1.1627
P14079	Q9UJL9	0.53095
P14079	Q8NA03	1.1425
P14079	Q7Z713	0.28036
P14079	Q5JNZ3	0.71663
P14079	Q0VAF6	0.48057
P14079	Q9H9Y6	0.65343
P14079	Q9P2I0	0.96424
P14079	Q9UPV0	1.8404
P14079	Q9H7M6	0.71756
P14079	O15372	0.81331
P14079	Q13296	0.8483
P14079	Q5T6F2	1.5951
P14079	Q7LBC6	0.32669
P14079	Q8IU68	0.94171
P14079	Q08999	1.2778
P14079	Q5VZL5	1.6004
P14079	Q8N5C7	0.33635
P14079	A0PJW6	1.2281
P14079	Q9UJV8	0.10002
P14079	Q5TYW8	0.24326
P14079	Q96QF7	0.77901
P14079	Q8IW50	1.3222
P14079	Q96JS3	0.40998
P14079	Q8IYG6	0.022844
P14079	Q96HW7	0.81654
P14079	Q8NDY6	0.57363
P14079	Q3LI58	1.1715
P14079	Q8N8D9	0.69563
P14079	Q8NAQ8	1.9833
P14079	Q7Z5L4	1.0219
P14079	Q9NRI7	1.1715
P14079	P43320	0.53079
P14079	Q6ZN11	0.55341
P14079	E7EW31	1.1715
P14079	Q9BYD9	0.96178
P14079	Q9UBG7	0.16561
P14079	Q9NZD2	0.99061
P14079	Q9NYF0	0.49655
P14079	Q8NCE8	1.059
P14079	Q53F19	1.6203
P14079	P50750	0.35626
P14079	Q86TA4	0.64033
P14079	Q8WXG1	0.2836
P14079	Q7Z5L2	0.92628
P14079	Q5TG92	1.2191
P14079	Q9H081	0.81275
P14079	P20290	1.152
P14079	Q6PB30	1.4359
P14079	Q5T6S3	0.37434
P14079	Q9Y6J0	0.13942
P14079	O00273	0.53131
P14079	P40305	1.1533
P14079	Q86W50	0.8542
P14079	Q9NUG4	0.37637
P14079	P42768	0.62745
P14079	C9JLW8	1.1508
P14079	Q5VWT5	1.2828
P14079	Q53EQ6	0.98962

P14079	A6NF83	1.1925
P14079	Q8TBE0	0.75096
P14079	Q8WVM0	0.92582
P14079	Q30KP9	0.97021
P14079	Q6VUC0	0.37224
P14079	Q16836	0.1092
P14079	Q9HCH0	1.8498
P14079	A8MT70	1.3446
P14079	Q15545	0.84454
P14079	Q13490	0.39996
P14079	P46977	0.42096
P14079	O00712	0.49375
P14079	Q7Z6B0	0.99148
P14079	Q5TG08	1.5912
P14079	Q92870	0.64656
P14079	A6NLC8	1.5655
P14079	Q96NB1	0.42054
P14079	P42771	0.68193
P14079	Q99962	0.37228
P14079	Q9Y295	0.70778
P14079	Q9Y3D5	1.1754
P14079	Q69383	1.355
P14079	Q99457	0.5801
P14079	Q9BWH2	0.15502
P14079	Q9H322	1.3275
P14079	Q8N138	1.0559
P14079	Q96SI1	0.18591
P14079	Q9Y2G1	0.36614
P14079	Q8N7R0	0.28169
P14079	Q9HAV7	0.87526
P14079	A6NHN6	1.1493
P14079	Q5T280	1.7686
P14079	P49366	0.46787
P14079	Q96KR6	1.3607
P14079	Q6ZRH4	1.9689
P14079	Q8NAF0	0.1949
P14079	Q9Y2D9	0.77928
P14079	P10636	0.18943
P14079	Q9UBK5	1.522
P14079	J3KSC0	0.27668
P14079	O00401	0.60638
P14079	Q96KG9	0.28377
P14079	Q9H6E4	0.83655
P14079	P81534	0.95735
P14079	Q9UPX6	1.2485
P14079	Q5T5P2	0.60719
P14079	P42898	0.5127
P14079	Q14008	1.2114
P14079	Q9GZU7	0.6395
P14079	A6NEY8	0.88702
P14079	Q9UI08	0.35537
P14079	Q6ZP82	0.56221
P14079	A2RU67	0.91569
P14079	Q92733	1.1715
P14079	A8MV72	1.2455
P14079	F8WFD2	0.93248
P14079	Q14119	0.34674
P14079	Q9Y2L5	0.43112
P14079	Q5T871	1.1715
P14079	Q96D09	0.35142
P14079	Q9NPP4	0.28783
P14079	P17028	0.80586
P14079	O96001	1.0538
P14079	P04406	0.21361
P14079	Q8N998	0.71406
P14079	Q9BSE4	1.0284
P14079	Q8WWB5	1.4021
P14079	Q5VW36	0.6409
P14079	Q9H201	0.36898
P14079	Q8WVJ2	0.46566
P14079	Q14681	0.18591
P14079	P23760	0.82375
P14079	Q96EU6	0.84735
P14079	Q9C0D7	0.96288
P14079	Q6ZTU2	1.5023
P14079	Q8NBC4	1.1715

49705	P14079	Q96GV9	0.67424
49706	P14079	P0DJH9	0.75084
49707	P14079	Q3BBV1	1.9823
49708	P14079	P62857	0.97776
49709	P14079	Q86TL2	1.1538
49710	P14079	Q9NSN8	0.25447
49711	P14079	Q6ZSB3	0.46525
49712	P14079	Q96PV4	0.30393
49713	P14079	Q9BT04	1.0825
49714	P14079	Q9BYX7	0.86229
49715	P14079	Q9BVW5	1.0454
49716	P14079	Q8TAQ5	0.77845
49717	P14079	Q9UPX8	0.39138
49718	P14079	Q96G75	0.21263
49719	P14079	P05060	0.71989
49720	P14079	P55884	0.29678
49721	P14079	Q8NHY2	1.0798
49722	P14079	Q9P1G2	1.1212
49723	P14079	Q8NA96	1.0613
49724	P14079	P62861	1.6749
49725	P14079	Q9H6K5	1.3222
49726	P14079	Q92843	0.66563
49727	P14079	Q8IWE4	0.62851
49728	P14079	Q96CM4	0.55134
49729	P14079	Q9GZM5	1.0676
49730	P14079	Q8TAF7	0.9897
49731	P14079	Q15942	0.54799
49732	P14079	P0CJ79	0.71663
49733	P14079	E7ETH6	0.5823
49734	P14079	Q8WXI4	0.28777
49735	P14079	Q6ZS94	1.1715
49736	P14079	Q5T686	1.029
49737	P14079	Q5VV41	0.38732
49738	P14079	A1L190	1.5102
49739	P14079	Q96C28	0.8567
49740	P14079	P46013	0.62183
49741	P14079	Q9Y3D9	0.7261
49742	P14079	Q6P2I3	0.70336
49743	P14079	Q6ZTI6	1.2431
49744	P14079	P60412	1.3698
49745	P14079	Q96LQ0	1.073
49746	P14079	Q15398	1.1106
49747	P14079	Q9GZY6	0.69012
49748	P14079	H3BQB6	1.5312
49749	P14079	O94916	0.51423
49750	P14079	Q06587	0.50535
49751	P14079	Q6ZSR6	0.70369
49752	P14079	Q8N5F7	1.3918
49753	P14079	A0ZSE6	1.0693
49754	P14079	P43362	0.30704
49755	P14079	Q5VT40	1.1731
49756	P14079	Q5TA78	1.1715
49757	P14079	Q14201	0.20849
49758	P14079	Q66K41	1.3958
49759	P14079	O43731	0.80489
49760	P14079	P22415	0.38671
49761	P14079	Q9H0C5	0.71001
49762	P14079	P37837	1.247
49763	P14079	P82914	1.2442
49764	P14079	Q8IUZ0	0.25965
49765	P14079	P43351	0.89489
49766	P14079	O15503	0.51487
49767	P14079	Q6ZTZ1	0.90832
49768	P14079	P61024	1.0304
49769	P14079	Q86T29	0.51784
49770	P14079	Q86SX3	1.9267
49771	P14079	Q8N852	0.84909
49772	P14079	Q7L3B6	0.62039
49773	P14079	Q96PQ5	0.43343
49774	P14079	Q15653	1.2753
49775	P14079	Q9BSR8	1.0273
49776	P14079	Q8WXI9	0.8296
49777	P14079	Q13495	1.7083
49778	P14079	Q8NHW5	0.4748
49779	P14079	Q8TAT2	0.82352
49780	P14079	P51948	0.56751

P14079	Q5T5B0	1.1715
P14079	Q6ZTI0	1.1715
P14079	O15544	0.53058
P14079	Q8TAA5	0.87526
P14079	Q8IW19	0.38463
P14079	Q9HD34	1.0041
P14079	Q6NSZ9	0.38376
P14079	P21912	0.082224
P14079	Q8WTP8	0.11287
P14079	Q9UMS6	0.83339
P14079	Q9NXZ1	0.47767
P14079	P58513	0.23646
P14079	Q9NX55	0.64512
P14079	Q96C57	1.1674
P14079	Q13123	1.4533
P14079	Q9H814	1.5648
P14079	Q96LM6	0.51314
P14079	Q7Z4H3	0.35535
P14079	Q17R26	0.70934
P14079	Q5TBB1	1.5049
P14079	Q8IXR5	1.0576
P14079	Q6ICL3	0.81135
P14079	Q9HD47	0.58708
P14079	Q9H6B1	0.98575
P14079	Q96NL8	1.4283
P14079	O75391	1.3421
P14079	P49406	0.94824
P14079	Q96FM1	0.85426
P14079	Q9Y6Q2	0.28158
P14079	Q8N8H1	0.93794
P14079	O94983	1.2996
P14079	Q96F83	0.68697
P14079	P63162	0.80323
P14079	K9M1U5	1.1715
P14079	Q6JBY9	0.97806
P14079	Q96CS2	0.76457
P14079	Q9BZW7	0.27174
Q2Q067	Q3KQU3	0.36757
Q2Q067	Q9BTW9	0.10311
Q2Q067	Q92783	0.5594
Q2Q067	O95363	0.23654
Q2Q067	P19012	0.69995
Q2Q067	Q86W56	0.66187
Q2Q067	P04156	0.31504
Q2Q067	P61580	0.48164
Q2Q067	Q9H714	0.17438
Q2Q067	Q96S95	0.26715
Q2Q067	Q5JS54	0.23277
Q2Q067	Q7Z3C6	0.068786
Q2Q067	Q5JNZ5	0.27303
Q2Q067	Q6P047	0.22687
Q2Q067	E7ETH6	0.10377
Q2Q067	O95376	0.31653
Q2Q067	Q8N5N7	0.28691
Q2Q067	Q68CZ2	0.33339
Q2Q067	Q13562	0.25144
Q2Q067	Q0D2J5	0.11356
Q2Q067	Q5VSL9	0.76954
Q2Q067	Q12901	0.1777
Q2Q067	Q8IXW0	0.22005
Q2Q067	O95411	0.19215
Q2Q067	P05204	0.35292
Q2Q067	Q5MJ07	0.40408
Q2Q067	A8MYZ0	0.30161
Q2Q067	P02792	0.15177
Q2Q067	O60806	0.46742
Q2Q067	Q9NUC0	0.061697
Q2Q067	P51398	1.1807
Q2Q067	P49454	0.55148
Q2Q067	Q9HC78	0.13257
Q2Q067	Q09FC8	0.21153
Q2Q067	Q96EI5	0.42404
Q2Q067	A8MTY7	0.34715
Q2Q067	Q9Y253	0.22824
Q2Q067	Q8WW24	0.1203
Q2Q067	Q9H4I2	0.76059

Q2Q067	Q01844	0.51672
Q2Q067	Q9NZ81	0.69263
Q2Q067	Q9UNP9	0.3075
Q2Q067	Q9UM63	0.16623
Q2Q067	O15014	0.6492
Q2Q067	P23588	0.12363
Q2Q067	Q92900	0.53858
Q2Q067	A6NJJ6	0.29573
Q2Q067	P05198	0.70496
Q2Q067	Q96SQ5	0.12519
Q2Q067	Q96TC7	0.31827
Q2Q067	O15511	0.13168
Q2Q067	Q14974	1.104
Q2Q067	P78346	0.35194
Q2Q067	Q9HD33	0.36818
Q2Q067	Q7L2H7	0.52105
Q2Q067	Q2TAM9	0.54396
Q2Q067	Q13278	0.32659
Q2Q067	Q9BUE0	1.0006
Q2Q067	Q9Y473	0.24041
Q2Q067	Q6ZRS4	0.2567
Q2Q067	Q9BYE7	0.77359
Q2Q067	Q9H583	1.008
Q2Q067	Q66PJ3	0.60884
Q2Q067	O43290	0.93736
Q2Q067	Q13573	0.62304
Q2Q067	Q8TCS8	0.069106
Q2Q067	Q9C0C6	0.43222
Q2Q067	Q8IZ16	0.13934
Q2Q067	Q96F63	0.29573
Q2Q067	P61313	1.0382
Q2Q067	Q9BU02	0.46811
Q2Q067	B1AH88	0.19215
Q2Q067	Q9NQG7	0.26694
Q2Q067	Q9BSU1	0.35334
Q2Q067	P07438	0.18499
Q2Q067	Q14526	0.53132
Q2Q067	Q8NHR7	0.057884
Q2Q067	Q9Y5K5	0.32826
Q2Q067	P30153	0.55294
Q2Q067	Q8WV37	0.38703
Q2Q067	Q9BRT2	0.32308
Q2Q067	Q9NWS1	0.93617
Q2Q067	Q9UNN4	0.41554
Q2Q067	Q8N859	0.22415
Q2Q067	Q12798	0.60905
Q2Q067	O43365	0.89284
Q2Q067	O95602	0.58405
Q2Q067	Q2YD98	0.12878
Q2Q067	Q6PF05	0.17122
Q2Q067	P83369	0.68999
Q2Q067	P52742	0.1371
Q2Q067	Q15054	0.59032
Q2Q067	Q15573	0.44637
Q2Q067	Q9BRH9	0.11603
Q2Q067	Q8NDY4	0.19215
Q2Q067	Q9ULD0	0.32592
Q2Q067	Q9UKI9	1.2939
Q2Q067	Q3KRA6	0.59206
Q2Q067	Q9UI26	0.32176
Q2Q067	Q9Y4R8	0.11677
Q2Q067	Q8NG57	0.24331
Q2Q067	Q86X02	0.074505
Q2Q067	Q96N77	0.29295
Q2Q067	Q9Y2J2	0.32669
Q2Q067	Q5SZL2	0.071228
Q2Q067	Q3MIV0	0.19215
Q2Q067	Q9UBW5	0.47001
Q2Q067	Q9NWB6	0.39593
Q2Q067	Q8N0U6	0.19215
Q2Q067	Q9Y6X3	0.94733
Q2Q067	Q4G0F5	0.054305
Q2Q067	C9JN71	0.13128
Q2Q067	Q8NHY2	1.0329
Q2Q067	Q14151	0.65493
Q2Q067	Q9BZZ5	0.35432

Q2Q067	Q9P2Y5	0.23327
Q2Q067	O14734	0.23345
Q2Q067	Q12905	1.0621
Q2Q067	O43463	0.77783
Q2Q067	Q9UPP1	0.40636
Q2Q067	O95671	0.23493
Q2Q067	Q9NNW5	0.46718
Q2Q067	Q7RTT3	0.12144
Q2Q067	Q96EE3	0.56907
Q2Q067	Q9NWY4	0.20754
Q2Q067	A6NFL8	0.37471
Q2Q067	Q9NZI7	0.2899
Q2Q067	Q8TAF3	0.62517
Q2Q067	Q8IUD2	0.80658
Q2Q067	Q8WZ33	0.81112
Q2Q067	P33240	0.48395
Q2Q067	Q01082	0.43036
Q2Q067	Q9NWK9	0.4745
Q2Q067	Q99460	0.2383
Q2Q067	O15027	0.30734
Q2Q067	P27540	0.43526
Q2Q067	Q3LI58	0.19215
Q2Q067	A4D997	0.2528
Q2Q067	P17544	1.069
Q2Q067	Q9P2D3	0.38872
Q2Q067	O75525	0.75429
Q2Q067	Q9BWC9	0.47289
Q2Q067	P30281	0.70821
Q2Q067	Q13118	0.323
Q2Q067	A6NJV1	0.26085
Q2Q067	Q8WW35	0.28783
Q2Q067	Q9BZL4	0.47001
Q2Q067	P25490	0.24197
Q2Q067	Q33E94	0.91753
Q2Q067	Q6VMQ6	0.99512
Q2Q067	P52434	0.29775
Q2Q067	Q8NCF5	0.47309
Q2Q067	Q3ZCV2	0.29573
Q2Q067	Q9BZK3	0.1814
Q2Q067	Q92750	0.63491
Q2Q067	Q9BPX7	0.062418
Q2Q067	Q6IPW1	0.21605
Q2Q067	O60232	0.5901
Q2Q067	P01100	0.60196
Q2Q067	Q96J02	0.30092
Q2Q067	Q9H3C7	0.51883
Q2Q067	P83876	0.91461
Q2Q067	Q8IXJ6	0.31795
Q2Q067	Q99613	0.69547
Q2Q067	Q96D70	0.44312
Q2Q067	Q9UL45	0.15732
Q2Q067	Q9BQ61	0.29573
Q2Q067	Q03989	0.29397
Q2Q067	P62701	0.50169
Q2Q067	P22392	0.26528
Q2Q067	P46108	0.42959
Q2Q067	Q3KNT7	0.032013
Q2Q067	H3BU77	0.18601
Q2Q067	Q96S07	0.19215
Q2Q067	Q9UN81	0.30205
Q2Q067	Q6PB30	0.42267
Q2Q067	Q9UK13	0.23615
Q2Q067	P35789	0.20369
Q2Q067	Q9HBH1	0.35356
Q2Q067	P53370	0.41559
Q2Q067	Q9UH99	0.081153
Q2Q067	O60524	0.55837
Q2Q067	Q8NCT3	0.37244
Q2Q067	P50402	0.14022
Q2Q067	Q6V9R5	0.21433
Q2Q067	Q8NA42	0.11771
Q2Q067	O00213	0.39095
Q2Q067	Q9HB65	0.51366
Q2Q067	Q9Y6X8	0.69773
Q2Q067	Q6FI81	0.36772
Q2Q067	Q8NHW5	0.09739

Q2Q067	Q92585	0.9681
Q2Q067	O75351	0.27784
Q2Q067	Q9BTV5	0.37446
Q2Q067	Q96HA8	0.94956
Q2Q067	Q92988	0.56881
Q2Q067	P35908	0.24881
Q2Q067	O00425	0.4258
Q2Q067	O75161	0.51717
Q2Q067	Q8N187	0.66047
Q2Q067	Q9H2G9	0.40367
Q2Q067	P82921	0.33904
Q2Q067	Q8WWX9	0.4411
Q2Q067	O95639	0.32893
Q2Q067	Q3SY00	0.41702
Q2Q067	Q9Y3A2	0.84719
Q2Q067	Q96MR7	0.77455
Q2Q067	Q96FV9	0.8617
Q2Q067	Q9ULR5	0.42796
Q2Q067	Q9GZT8	0.81427
Q2Q067	O43167	0.58991
Q2Q067	Q2M218	0.10324
Q2Q067	Q04864	1.232
Q2Q067	P02538	0.67965
Q2Q067	P81877	0.42639
Q2Q067	Q8TA86	0.47525
Q2Q067	Q9UHL9	0.27553
Q2Q067	Q7L0X2	0.061262
Q2Q067	Q9NYP9	0.30323
Q2Q067	P0C853	0.19215
Q2Q067	Q9BYD6	0.2879
Q2Q067	Q9UJN7	0.091745
Q2Q067	Q9NXW9	0.1687
Q2Q067	Q5SWW7	0.19215
Q2Q067	O00411	0.49157
Q2Q067	Q5H9J9	0.058047
Q2Q067	Q96MW7	0.19704
Q2Q067	Q8N831	0.2401
Q2Q067	O75832	0.464
Q2Q067	Q99865	0.32334
Q2Q067	P0C1Z6	1.1991
Q2Q067	Q16236	0.67566
Q2Q067	Q5TAQ9	0.24229
Q2Q067	Q03923	0.25959
Q2Q067	Q6UN15	0.61578
Q2Q067	Q9Y2S7	0.44629
Q2Q067	Q9Y2M0	0.33929
Q2Q067	Q8N5R6	0.32153
Q2Q067	Q14657	0.7317
Q2Q067	O60684	0.32002
Q2Q067	A8MSI8	0.69679
Q2Q067	Q86U42	0.70446
Q2Q067	A8MYA2	0.15705
Q2Q067	P48556	0.39923
Q2Q067	O94973	0.39104
Q2Q067	O15234	0.87169
Q2Q067	Q9Y4A5	0.80781
Q2Q067	Q8WV44	0.29601
Q2Q067	P09917	0.23837
Q2Q067	Q9BYQ7	0.41862
Q2Q067	Q13084	1.0215
Q2Q067	Q9UEG4	0.11466
Q2Q067	Q9H321	0.43298
Q2Q067	P62633	0.60791
Q2Q067	P10746	0.14814
Q2Q067	Q5VVS0	0.53547
Q2Q067	Q9NVH2	0.20549
Q2Q067	Q6ZUA9	0.24272
Q2Q067	Q5TD94	0.094971
Q2Q067	Q9H2U2	0.3933
Q2Q067	Q96N21	0.28161
Q2Q067	P54252	0.43114
Q2Q067	Q8IWL8	0.4678
Q2Q067	Q6P387	0.32989
Q2Q067	Q6NUN7	0.19215
Q2Q067	Q9BUG6	0.076446
Q2Q067	Q969W3	0.36981

Q2Q067	Q9NUL5	0.025585
Q2Q067	C9JE40	0.83476
Q2Q067	Q5R3I4	0.054652
Q2Q067	Q8NCX0	0.11855
Q2Q067	P18754	1.0894
Q2Q067	P49458	0.40228
Q2Q067	Q96S65	0.70154
Q2Q067	Q9BYQ6	0.34715
Q2Q067	P60331	0.34715
Q2Q067	Q96GE4	0.031307
Q2Q067	Q9NX08	0.28783
Q2Q067	Q96H22	0.14794
Q2Q067	Q9BZE1	0.28685
Q2Q067	A6NDX5	0.15529
Q2Q067	Q15013	0.80221
Q2Q067	P47914	0.57329
Q2Q067	P23381	0.2013
Q2Q067	A8MU10	0.19215
Q2Q067	P02545	0.73076
Q2Q067	Q9H3J6	0.39716
Q2Q067	O43364	0.61745
Q2Q067	Q6VN20	0.35239
Q2Q067	Q86VW0	0.19247
Q2Q067	P82933	0.59997
Q2Q067	Q8IU60	0.90724
Q2Q067	P07910	0.24013
Q2Q067	P31749	0.55651
Q2Q067	Q8NEM1	0.24839
Q2Q067	P52948	1.0538
Q2Q067	Q92890	0.80268
Q2Q067	P35754	0.3622
Q2Q067	Q9Y305	0.31752
Q2Q067	Q9NRP2	0.40933
Q2Q067	Q71F23	0.61356
Q2Q067	O15525	0.39254
Q2Q067	Q7LBR1	0.85651
Q2Q067	Q6P2H3	0.50076
Q2Q067	P40306	0.41448
Q2Q067	Q13185	0.77814
Q2Q067	Q6NVH7	0.56614
Q2Q067	Q9NWS8	0.32996
Q2Q067	Q9UC06	0.10905
Q2Q067	Q9BZE4	0.26462
Q2Q067	Q8N535	0.20616
Q2Q067	Q96GA3	0.44044
Q2Q067	Q52M58	0.30292
Q2Q067	Q9BQY9	0.41843
Q2Q067	Q14258	1.0352
Q2Q067	Q86VG3	0.23361
Q2Q067	Q86XA9	0.087098
Q2Q067	P50995	0.7908
Q2Q067	P0DMB2	0.26811
Q2Q067	Q99583	0.41784
Q2Q067	Q2KHR2	0.22597
Q2Q067	Q9ULX3	0.63234
Q2Q067	Q6ZN28	0.57327
Q2Q067	P62312	0.84991
Q2Q067	A8MTB1	0.19215
Q2Q067	Q08AI8	0.29573
Q2Q067	Q93034	0.75541
Q2Q067	O75155	0.76301
Q2Q067	Q8IZL2	0.87367
Q2Q067	P98182	0.042317
Q2Q067	Q14008	0.83619
Q2Q067	Q9BSJ6	0.47129
Q2Q067	Q6UXP7	0.10047
Q2Q067	Q9Y2Y0	0.97635
Q2Q067	P42773	0.31869
Q2Q067	O43920	0.32856
Q2Q067	Q9Y333	0.73413
Q2Q067	Q8IX15	0.51811
Q2Q067	Q8N6V4	0.072688
Q2Q067	Q5U5X0	0.4051
Q2Q067	Q9NRF9	0.034431
Q2Q067	A0AUK8	0.045275
Q2Q067	Q5HYW3	0.41864

Q2Q067	Q9UNS2	0.6289
Q2Q067	Q12769	1.0412
Q2Q067	Q9H4X1	0.41661
Q2Q067	Q12837	0.56038
Q2Q067	Q96SD1	0.0024929
Q2Q067	Q96CB9	0.35209
Q2Q067	Q8WUQ7	0.11351
Q2Q067	Q8N2M8	0.54566
Q2Q067	P46779	1.1616
Q2Q067	O00186	0.73784
Q2Q067	Q9H7X3	0.091251
Q2Q067	Q9H008	0.40325
Q2Q067	O14786	0.33477
Q2Q067	Q9H910	0.45693
Q2Q067	Q6UXP9	0.03377
Q2Q067	Q9BYQ2	0.34715
Q2Q067	Q9BXU2	0.2148
Q2Q067	Q8TF63	0.34114
Q2Q067	Q7L8J4	0.20814
Q2Q067	O14744	0.93696
Q2Q067	Q9BVP2	0.18996
Q2Q067	Q9Y6K5	0.10627
Q2Q067	Q9UPN9	0.4484
Q2Q067	O60239	0.27414
Q2Q067	Q96EL2	0.30752
Q2Q067	Q9Y5Q3	0.87933
Q2Q067	Q9Y3S2	0.21919
Q2Q067	Q8WXF5	0.26153
Q2Q067	Q5T036	0.016216
Q2Q067	P35321	0.37712
Q2Q067	Q9Y6D6	0.60753
Q2Q067	Q9Y6Q5	0.34478
Q2Q067	Q9BYD1	0.82504
Q2Q067	Q03052	0.3639
Q2Q067	O75461	0.26705
Q2Q067	Q9Y613	0.54262
Q2Q067	Q9H246	0.10062
Q2Q067	Q8IUC6	0.24356
Q2Q067	O00410	1.1626
Q2Q067	P56524	0.32728
Q2Q067	Q93009	0.32544
Q2Q067	Q9UK11	0.1732
Q2Q067	Q9BY41	0.37912
Q2Q067	P17029	0.2057
Q2Q067	Q8WXQ3	0.65353
Q2Q067	Q96GX2	0.36712
Q2Q067	Q12796	1.0622
Q2Q067	Q9BW66	0.85206
Q2Q067	P52738	0.079889
Q2Q067	Q9ULX6	0.3226
Q2Q067	Q9NR56	0.40937
Q2Q067	Q96IP4	0.25585
Q2Q067	Q9H0C8	0.36563
Q2Q067	Q96GI7	0.29573
Q2Q067	Q10587	0.36826
Q2Q067	A8MTY0	0.22615
Q2Q067	P0C7X3	0.18322
Q2Q067	O75427	0.4431
Q2Q067	Q6NVV0	0.29201
Q2Q067	A5PL33	0.36577
Q2Q067	Q9HAW0	0.41702
Q2Q067	Q495Y8	0.23617
Q2Q067	O75909	1.0122
Q2Q067	Q3MHD2	0.33951
Q2Q067	Q9Y3C7	0.91133
Q2Q067	Q9NQT4	0.48699
Q2Q067	A0AVK6	0.39033
Q2Q067	Q96NU7	0.065492
Q2Q067	Q15631	0.71082
Q2Q067	Q8N228	0.30214
Q2Q067	P55036	0.59286
Q2Q067	Q9UMS5	0.5332
Q2Q067	Q96D98	0.5152
Q2Q067	Q9H7R0	0.20181
Q2Q067	Q99747	0.48767
Q2Q067	Q96PE6	0.089474

Q2Q067	Q8N1I8	0.19215
Q2Q067	Q8IZD2	0.25129
Q2Q067	Q3LI54	0.19215
Q2Q067	Q4U2R6	0.82504
Q2Q067	A6NFQ7	0.32768
Q2Q067	Q99502	0.12582
Q2Q067	Q9H3H9	0.51012
Q2Q067	Q8IZ26	0.10927
Q2Q067	P07741	0.23436
Q2Q067	Q10567	0.6406
Q2Q067	Q7Z353	0.25814
Q2Q067	P60002	0.54009
Q2Q067	Q9HBZ2	0.32854
Q2Q067	Q9NR28	0.85518
Q2Q067	O75474	0.61002
Q2Q067	Q9BRR0	0.29228
Q2Q067	Q6ZW49	0.38588
Q2Q067	Q96HW7	0.57387
Q2Q067	Q5VUG0	0.3157
Q2Q067	Q96HA1	0.47828
Q2Q067	Q9UPR3	1.0134
Q2Q067	P10827	0.27434
Q2Q067	Q8WWY6	0.39593
Q2Q067	A6NJL1	0.058453
Q2Q067	P04198	0.86056
Q2Q067	Q9Y615	0.28879
Q2Q067	P15884	0.63579
Q2Q067	Q96A49	0.51629
Q2Q067	B1AL46	0.4993
Q2Q067	Q9P219	0.31389
Q2Q067	Q2KJ03	0.077271
Q2Q067	P20700	0.34604
Q2Q067	O75663	0.39655
Q2Q067	P20226	0.97674
Q2Q067	Q9NVM4	0.22247
Q2Q067	Q04726	0.99024
Q2Q067	Q6ZRH9	0.19215
Q2Q067	Q96N03	0.28783
Q2Q067	Q96HR3	0.77088
Q2Q067	Q96KQ4	0.86974
Q2Q067	O15126	0.18082
Q2Q067	O43361	0.12208
Q2Q067	Q9HAU4	0.34925
Q2Q067	Q5D1E8	0.40254
Q2Q067	P0C7T4	0.19215
Q2Q067	O60294	0.27826
Q2Q067	A9UHW6	0.73024
Q2Q067	P10243	0.5304
Q2Q067	Q9Y462	0.60647
Q2Q067	Q7Z4W3	0.19215
Q2Q067	Q9Y324	0.54298
Q2Q067	P55957	0.44251
Q2Q067	P24385	0.59769
Q2Q067	Q99417	1.4642
Q2Q067	Q7Z6J0	0.24785
Q2Q067	P55769	0.86276
Q2Q067	Q7L5A3	0.25564
Q2Q067	Q9Y5V3	0.81168
Q2Q067	Q9NRH1	0.11691
Q2Q067	P59022	0.28782
Q2Q067	P09067	0.26893
Q2Q067	P56377	0.33492
Q2Q067	Q8TCE6	0.58433
Q2Q067	Q7Z4H7	0.80542
Q2Q067	P78559	0.64888
Q2Q067	Q96NI8	0.20344
Q2Q067	Q3BBV2	0.3158
Q2Q067	Q9GZW5	0.5105
Q2Q067	Q92681	0.37464
Q2Q067	Q5JXB2	0.10977
Q2Q067	Q16594	0.60742
Q2Q067	Q6ZUG5	0.10067
Q2Q067	O75340	0.41759
Q2Q067	O00303	0.24485
Q2Q067	Q6ZTY9	0.11671
Q2Q067	Q9Y266	0.49408

Q2Q067	Q9BVL4	0.6609
Q2Q067	P49591	0.17564
Q2Q067	P56182	0.44684
Q2Q067	Q9UGU0	0.3101
Q2Q067	Q9GZP8	0.19215
Q2Q067	Q96NB3	0.38146
Q2Q067	Q32M84	0.018047
Q2Q067	Q96DE9	0.012384
Q2Q067	O75925	0.53475
Q2Q067	O95273	0.88398
Q2Q067	P07195	0.23751
Q2Q067	Q92922	1.0562
Q2Q067	Q6NUN9	0.70422
Q2Q067	Q63HQ0	0.19215
Q2Q067	P63104	1.0277
Q2Q067	Q6ZVL8	0.19215
Q2Q067	Q8IVT2	0.40664
Q2Q067	Q96KN1	0.33672
Q2Q067	P07737	0.87363
Q2Q067	Q8N9R8	0.56447
Q2Q067	Q9UII4	0.30181
Q2Q067	Q9Y3F4	0.84451
Q2Q067	Q711Q0	0.19215
Q2Q067	P39687	1.403
Q2Q067	Q13033	0.66266
Q2Q067	P55895	0.42067
Q2Q067	Q8N9R0	0.19215
Q2Q067	Q9UJL9	0.088569
Q2Q067	Q15398	0.97416
Q2Q067	Q8N1D5	0.037777
Q2Q067	Q9UL41	0.32741
Q2Q067	Q9BPY3	0.037799
Q2Q067	Q96QU8	0.94532
Q2Q067	O60664	0.49615
Q2Q067	Q13875	0.49762
Q2Q067	Q13536	0.19215
Q2Q067	A6NEY8	0.09569
Q2Q067	Q9UQ80	1.0888
Q2Q067	Q3LHN0	0.46381
Q2Q067	Q13356	0.057687
Q2Q067	Q9NS56	0.28734
Q2Q067	Q96RT8	0.30342
Q2Q067	Q70YC5	0.1967
Q2Q067	O43747	0.6781
Q2Q067	O60304	0.087873
Q2Q067	Q9NTK1	0.4489
Q2Q067	Q96PV7	0.33983
Q2Q067	Q9H5J4	0.2866
Q2Q067	Q8IVF1	0.59249
Q2Q067	Q9BRU2	0.46897
Q2Q067	P22087	0.58162
Q2Q067	P55265	0.37419
Q2Q067	P23511	1.1908
Q2Q067	P63172	0.78748
Q2Q067	Q9BU68	0.24142
Q2Q067	Q7Z2Z1	0.64555
Q2Q067	Q9NSB2	0.15836
Q2Q067	H0UI37	0.053977
Q2Q067	Q9Y4P1	0.17069
Q2Q067	Q15010	0.13408
Q2Q067	Q9BYR5	0.34715
Q2Q067	P24278	0.80978
Q2Q067	P49790	1.1348
Q2Q067	P82912	0.2882
Q2Q067	Q3LI63	0.19215
Q2Q067	Q9UD71	0.46617
Q2Q067	Q8NEP9	0.21203
Q2Q067	Q99638	0.53786
Q2Q067	Q9H2H8	0.21345
Q2Q067	P59036	0.19215
Q2Q067	Q59GN2	0.12011
Q2Q067	O95782	0.43539
Q2Q067	Q9P209	0.77967
Q2Q067	O15519	0.23517
Q2Q067	Q53QW1	0.29573
Q2Q067	Q9H063	0.26238

Q2Q067	Q96E14	0.38255
Q2Q067	Q9BSH4	0.56218
Q2Q067	Q9NYK6	0.23304
Q2Q067	Q9Y5A7	0.66696
Q2Q067	Q8WVC0	0.98527
Q2Q067	Q8N1G4	0.22814
Q2Q067	Q05066	0.49594
Q2Q067	Q8NEF9	0.62384
Q2Q067	P07196	0.56096
Q2Q067	Q8WX93	0.24777
Q2Q067	Q6ZQV5	0.16182
Q2Q067	Q9HBE1	0.073018
Q2Q067	Q14315	0.23532
Q2Q067	Q06416	0.32768
Q2Q067	O60296	0.50953
Q2Q067	Q5T6F2	0.47078
Q2Q067	O60841	0.072619
Q2Q067	Q8TAD8	1.2689
Q2Q067	A6NGQ2	0.14982
Q2Q067	Q9NXR8	0.25197
Q2Q067	Q86SY8	0.19215
Q2Q067	P82932	0.46547
Q2Q067	Q9Y6C7	0.19215
Q2Q067	Q86WQ0	0.49421
Q2Q067	Q9H444	0.70817
Q2Q067	Q16589	0.26633
Q2Q067	Q13487	0.88241
Q2Q067	Q63HK5	0.32097
Q2Q067	Q5TAP6	0.31591
Q2Q067	O95391	0.10313
Q2Q067	P0CJ88	0.27517
Q2Q067	Q9BY44	0.34844
Q2Q067	Q6ZVK8	0.26464
Q2Q067	Q9NQW6	0.22355
Q2Q067	Q8NI35	0.24382
Q2Q067	Q9BUP3	0.18682
Q2Q067	E9PI22	0.28994
Q2Q067	Q8TD31	0.37672
Q2Q067	Q9HAV7	0.18761
Q2Q067	Q6ICB0	0.41668
Q2Q067	Q86UA6	0.61659
Q2Q067	Q99081	0.4861
Q2Q067	Q86XZ4	0.21187
Q2Q067	Q8N0Y2	0.10021
Q2Q067	Q9Y3B7	0.85845
Q2Q067	Q9Y2D1	1.1951
Q2Q067	Q96PV6	0.37901
Q2Q067	Q1A5X6	0.29573
Q2Q067	Q92664	0.098905
Q2Q067	C9J3I9	0.20816
Q2Q067	Q5VTD9	0.22242
Q2Q067	P86480	0.19215
Q2Q067	Q96C74	0.23098
Q2Q067	Q9GZL7	0.7062
Q2Q067	Q8IY63	0.42771
Q2Q067	Q15928	0.25761
Q2Q067	Q14160	0.56212
Q2Q067	Q6PGQ1	0.20845
Q2Q067	Q9NPB6	0.67809
Q2Q067	Q9BSF8	0.32989
Q2Q067	Q2VPB7	0.45094
Q2Q067	Q8IY18	0.31658
Q2Q067	Q6ZQR2	0.51861
Q2Q067	Q96K75	0.090033
Q2Q067	O95433	0.53418
Q2Q067	Q9Y3Q8	1.2604
Q2Q067	Q9H7N4	0.57009
Q2Q067	Q9UHB7	1.3326
Q2Q067	Q12888	0.73469
Q2Q067	Q9BYQ8	0.34715
Q2Q067	Q9H175	1.1059
Q2Q067	P51861	0.30462
Q2Q067	O00512	0.78563
Q2Q067	Q96LI5	0.55342
Q2Q067	Q6XD76	0.29177
Q2Q067	Q8NEE0	0.19215

Q2Q067	Q96B97	0.33314
Q2Q067	O00291	0.43039
Q2Q067	Q8WW12	0.59432
Q2Q067	O00470	0.61739
Q2Q067	Q8NCR6	0.15623
Q2Q067	P52272	0.22401
Q2Q067	Q9BUT9	0.60936
Q2Q067	Q9UBX2	0.27517
Q2Q067	O75818	0.23666
Q2Q067	Q6PJP8	0.58221
Q2Q067	Q8WY54	0.79297
Q2Q067	Q86V59	0.16324
Q2Q067	Q92889	0.24821
Q2Q067	Q9UHR5	1.2198
Q2Q067	Q13887	0.25257
Q2Q067	Q7L8W6	0.2896
Q2Q067	Q9Y6G5	0.28783
Q2Q067	Q14980	1.0414
Q2Q067	Q9NVU7	0.38967
Q2Q067	Q92572	0.37613
Q2Q067	Q9P032	0.46005
Q2Q067	Q8NCQ2	0.37491
Q2Q067	Q15293	0.24578
Q2Q067	O00267	1.0433
Q2Q067	P35080	0.34479
Q2Q067	Q02543	0.78122
Q2Q067	Q96RK1	0.6348
Q2Q067	P06400	0.71708
Q2Q067	Q8TA94	0.24343
Q2Q067	Q9HB96	0.80624
Q2Q067	Q8IV32	0.28913
Q2Q067	P61326	1.0595
Q2Q067	O43493	0.35412
Q2Q067	B4DZS4	0.01678
Q2Q067	O60784	0.33773
Q2Q067	Q6NUP7	0.40334
Q2Q067	Q8IVG9	0.081155
Q2Q067	Q6ZN32	0.32768
Q2Q067	Q9H9T3	0.78432
Q2Q067	P30419	0.12854
Q2Q067	Q9NPJ6	0.88576
Q2Q067	O75534	0.081839
Q2Q067	O75494	0.57959
Q2Q067	O00231	0.48061
Q2Q067	Q8WYA1	0.33342
Q2Q067	Q9UMX3	0.55272
Q2Q067	Q8IZH2	0.20517
Q2Q067	Q0D2K3	0.48085
Q2Q067	Q8NCE0	0.35634
Q2Q067	Q7L9L4	0.74681
Q2Q067	Q8IWE5	0.33609
Q2Q067	O43257	0.24537
Q2Q067	Q96I24	0.43219
Q2Q067	Q8WW33	0.26738
Q2Q067	P14866	0.4648
Q2Q067	Q9BXQ6	0.19215
Q2Q067	Q7Z5K2	0.69142
Q2Q067	P29374	0.23394
Q2Q067	Q99547	0.67294
Q2Q067	P26373	1.0132
Q2Q067	O95567	0.19215
Q2Q067	O00233	0.65724
Q2Q067	P42167	0.12377
Q2Q067	Q9NPF5	0.90449
Q2Q067	P29692	0.31326
Q2Q067	Q9UJX3	0.74537
Q2Q067	Q9UJX5	0.80792
Q2Q067	A6NL46	0.41724
Q2Q067	Q6NW29	0.072759
Q2Q067	Q86YE8	0.16182
Q2Q067	P21980	0.20719
Q2Q067	Q9H2K2	0.30117
Q2Q067	Q5SRH9	0.43965
Q2Q067	O43663	0.78883
Q2Q067	P27694	0.54919
Q2Q067	P51825	1.0085

50541	Q2Q067	Q08AN1	0.16182
50542	Q2Q067	Q9C005	0.61146
50543	Q2Q067	P49959	0.19297
50544	Q2Q067	Q6RW13	0.47173
50545	Q2Q067	Q9Y676	0.32798
50546	Q2Q067	Q5TG08	0.42889
50547	Q2Q067	P41220	0.37096
50548	Q2Q067	Q8NEM7	0.27988
50549	Q2Q067	Q5XKK7	0.027736
50550	Q2Q067	Q15459	0.60867
50551	Q2Q067	P49792	0.85947
50552	Q2Q067	Q01094	0.79982
50553	Q2Q067	P58317	0.12151
50554	Q2Q067	O75394	0.23005
50555	Q2Q067	Q8IYF1	0.1975
50556	Q2Q067	Q9UKV0	0.41393
50557	Q2Q067	Q14678	0.4662
50558	Q2Q067	Q8WW38	0.53047
50559	Q2Q067	Q99575	0.6777
50560	Q2Q067	Q9Y4B6	0.8822
50561	Q2Q067	Q13427	0.38384
50562	Q2Q067	O95684	0.8369
50563	Q2Q067	Q9H706	0.28783
50564	Q2Q067	Q08999	1.328
50565	Q2Q067	Q96LR2	0.53194
50566	Q2Q067	Q6ZMY9	0.22722
50567	Q2Q067	Q56VL3	0.23797
50568	Q2Q067	Q96NJ1	0.19215
50569	Q2Q067	P40763	0.35081
50570	Q2Q067	Q9H5Q4	0.34354
50571	Q2Q067	Q8WYH8	0.51055
50572	Q2Q067	P78424	0.24106
50573	Q2Q067	Q8N8Z8	0.090549
50574	Q2Q067	P14921	0.85141
50575	Q2Q067	Q6ZNK6	0.53044
50576	Q2Q067	Q9NXV6	0.52994
50577	Q2Q067	Q8TAV0	0.44409
50578	Q2Q067	Q96A35	0.31215
50579	Q2Q067	Q5VZB9	0.026821
50580	Q2Q067	Q8TF01	0.44409
50581	Q2Q067	Q9UBQ5	1.0282
50582	Q2Q067	P0C7W0	0.29573
50583	Q2Q067	P23610	0.19215
50584	Q2Q067	P62750	0.86707
50585	Q2Q067	Q7Z6I5	0.47585
50586	Q2Q067	Q6ZS27	0.093473
50587	Q2Q067	Q9BV29	0.027765
50588	Q2Q067	Q9Y3E1	0.33063
50589	Q2Q067	Q8WWL7	0.88592
50590	Q2Q067	Q5VUE5	0.21787
50591	Q2Q067	Q96MT4	0.38755
50592	Q2Q067	Q0VDF9	0.26091
50593	Q2Q067	Q9NYB0	0.51342
50594	Q2Q067	Q9P127	0.071225
50595	Q2Q067	Q08209	0.38483
50596	Q2Q067	A6NP11	0.2627
50597	Q2Q067	Q8TAF5	0.19215
50598	Q2Q067	O60732	0.28783
50599	Q2Q067	Q9UL59	0.095324
50600	Q2Q067	Q96LA8	0.68414
50601	Q2Q067	Q8N6N3	0.098878
50602	Q2Q067	O15226	0.79674
50603	Q2Q067	Q9NS26	0.45678
50604	Q2Q067	P61579	0.36503
50605	Q2Q067	Q3SYF9	0.19215
50606	Q2Q067	Q8IZD4	0.95237
50607	Q2Q067	P06454	0.40814
50608	Q2Q067	Q9BZK8	0.19215
50609	Q2Q067	Q8N4W9	0.16182
50610	Q2Q067	Q53EQ6	0.24653
50611	Q2Q067	Q64ET8	0.38375
50612	Q2Q067	A6NGS2	0.31249
50613	Q2Q067	Q13889	0.25609
50614	Q2Q067	Q96NT1	0.57092
50615	Q2Q067	Q9H1L0	0.27594
50616	Q2Q067	Q9BRV8	0.52594

50617	Q2Q067	P60413	0.34715
50618	Q2Q067	P41219	0.091321
50619	Q2Q067	Q6ZQN3	0.3913
50620	Q2Q067	Q13352	0.43741
50621	Q2Q067	P31040	0.6795
50622	Q2Q067	P01106	0.456
50623	Q2Q067	O14579	0.31897
50624	Q2Q067	P47813	0.69178
50625	Q2Q067	Q8N1N5	0.44566
50626	Q2Q067	Q9H2P0	0.20944
50627	Q2Q067	P62258	0.8833
50628	Q2Q067	Q13148	0.5022
50629	Q2Q067	Q7Z6B0	0.25012
50630	Q2Q067	O95257	0.24277
50631	Q2Q067	Q6NS38	0.2331
50632	Q2Q067	A6NN90	0.070821
50633	Q2Q067	P10073	0.19323
50634	Q2Q067	A6NK39	0.17929
50635	Q2Q067	Q5I0X4	0.29573
50636	Q2Q067	Q52LR7	0.39053
50637	Q2Q067	O75145	0.3839
50638	Q2Q067	Q4VX62	0.040805
50639	Q2Q067	Q96JZ2	0.40655
50640	Q2Q067	Q8WUE5	0.039346
50641	Q2Q067	Q9BYG5	0.64098
50642	Q2Q067	Q6PGQ7	0.69013
50643	Q2Q067	Q7Z460	0.37141
50644	Q2Q067	O75940	0.61947
50645	Q2Q067	O14503	0.93756
50646	Q2Q067	Q9HAZ2	0.35867
50647	Q2Q067	Q6ZUJ4	0.18391
50648	Q2Q067	P36402	0.52095
50649	Q2Q067	O43159	0.78443
50650	Q2Q067	Q6ZR85	0.19215
50651	Q2Q067	Q7Z589	0.61115
50652	Q2Q067	Q9P2M7	0.42199
50653	Q2Q067	Q9BWG6	0.24343
50654	Q2Q067	Q12872	0.39866
50655	Q2Q067	Q8IZW8	0.29235
50656	Q2Q067	P63151	0.40284
50657	Q2Q067	Q16666	0.61541
50658	Q2Q067	Q6ZQP7	0.19215
50659	Q2Q067	P46937	0.80691
50660	Q2Q067	Q9UKX2	0.15564
50661	Q2Q067	Q15910	0.43964
50662	Q2Q067	Q8NCU1	0.10104
50663	Q2Q067	Q8N0Y7	0.37579
50664	Q2Q067	Q8IVM0	0.47001
50665	Q2Q067	Q9NR55	0.39056
50666	Q2Q067	O00193	0.54789
50667	Q2Q067	Q9NPJ8	0.11252
50668	Q2Q067	Q16533	0.47122
50669	Q2Q067	O75167	0.14846
50670	Q2Q067	Q8N2N9	0.24591
50671	Q2Q067	O43447	0.58868
50672	Q2Q067	O75151	0.60009
50673	Q2Q067	Q16775	0.15797
50674	Q2Q067	Q9Y3T9	1.0427
50675	Q2Q067	Q14690	0.60477
50676	Q2Q067	O60481	0.41087
50677	Q2Q067	P38432	0.70413
50678	Q2Q067	Q6IE81	1.0884
50679	Q2Q067	Q7KZF4	0.89741
50680	Q2Q067	Q96QA5	0.063067
50681	Q2Q067	Q92636	0.2383
50682	Q2Q067	P62273	0.62974
50683	Q2Q067	P67809	0.61143
50684	Q2Q067	Q9BQD3	0.34344
50685	Q2Q067	Q12982	0.21654
50686	Q2Q067	O95402	1.074
50687	Q2Q067	Q6PD62	0.61136
50688	Q2Q067	Q9BRT7	0.19215
50689	Q2Q067	Q13485	0.66858
50690	Q2Q067	P13929	0.16413
50691	Q2Q067	P09913	0.56698
50692	Q2Q067	Q8TF09	0.20659

Q2Q067	A6NGD5	0.14982
Q2Q067	Q9Y2B5	0.30342
Q2Q067	Q6ZTU2	0.48197
Q2Q067	Q6ZUT1	0.19215
Q2Q067	Q9UHQ7	0.3414
Q2Q067	Q96DX5	1.0354
Q2Q067	Q5SXM1	0.16388
Q2Q067	Q9BRA2	0.48685
Q2Q067	A2RUQ5	0.006992
Q2Q067	Q01543	0.41566
Q2Q067	O75334	0.48165
Q2Q067	Q9Y285	0.34673
Q2Q067	O43734	0.29573
Q2Q067	Q6ZS52	0.19215
Q2Q067	O00287	0.36415
Q2Q067	Q6IV72	0.15254
Q2Q067	A8MVS1	0.2324
Q2Q067	O95886	0.22198
Q2Q067	Q9UNS1	0.52015
Q2Q067	Q96K17	0.25699
Q2Q067	Q9HAJ7	0.72841
Q2Q067	O14639	0.51637
Q2Q067	P10589	0.2603
Q2Q067	Q3LI64	0.19215
Q2Q067	Q8N2I2	0.11047
Q2Q067	Q8N999	0.14222
Q2Q067	Q8N3V7	0.31798
Q2Q067	Q7Z6K1	0.31828
Q2Q067	Q6ECI4	0.14687
Q2Q067	P63313	0.34514
Q2Q067	P62837	0.53228
Q2Q067	Q9UC07	0.11961
Q2Q067	Q9NSC5	0.40811
Q2Q067	O14974	0.28951
Q2Q067	P46087	0.67093
Q2Q067	P35680	0.29203
Q2Q067	Q9NX58	0.14931
Q2Q067	Q05BU3	0.19215
Q2Q067	P53804	0.65686
Q2Q067	P38646	0.52131
Q2Q067	Q8WU58	0.071927
Q2Q067	Q14549	0.3497
Q2Q067	P51665	0.28864
Q2Q067	Q8IWV8	0.19345
Q2Q067	P55735	0.60413
Q2Q067	Q9P0U4	0.25659
Q2Q067	Q8NDD1	0.042204
Q2Q067	Q9H9D4	0.53405
Q2Q067	Q12986	0.19805
Q2Q067	Q9Y2L1	0.52771
Q2Q067	P55795	0.060059
Q2Q067	Q6P6B1	0.29573
Q2Q067	A6NJ64	0.079595
Q2Q067	Q9NR48	0.25973
Q2Q067	Q9Y3C4	0.29856
Q2Q067	Q5VWQ0	0.69023
Q2Q067	Q01130	0.72983
Q2Q067	Q496A3	0.092627
Q2Q067	A6NES4	0.15278
Q2Q067	Q9HCN2	0.19215
Q2Q067	Q5JQC4	0.19215
Q2Q067	Q9UI56	0.19215
Q2Q067	P31274	0.14312
Q2Q067	O95997	0.90099
Q2Q067	P13056	0.62814
Q2Q067	Q14674	0.25361
Q2Q067	Q9UJK0	0.52429
Q2Q067	Q9NPI6	0.97572
Q2Q067	Q9NVN8	0.27062
Q2Q067	Q8N0Z6	0.24265
Q2Q067	Q9H2T7	0.32281
Q2Q067	O75694	0.63148
Q2Q067	Q9HCH0	0.62636
Q2Q067	Q8IXK0	0.18945
Q2Q067	Q9C0F0	0.3175
Q2Q067	Q9P000	0.15956

Q2Q067	Q6IPU0	0.30454
Q2Q067	P31268	0.33074
Q2Q067	Q8WXE9	0.48133
Q2Q067	Q96C57	0.055702
Q2Q067	Q9Y2R9	0.50788
Q2Q067	Q6ZT77	0.10945
Q2Q067	Q9BYP9	0.34715
Q2Q067	Q86TX2	0.12431
Q2Q067	Q8IUE1	0.32768
Q2Q067	Q9H1B4	0.3004
Q2Q067	Q86XL3	0.23482
Q2Q067	Q5VSD8	0.12143
Q2Q067	Q9UJT2	0.43475
Q2Q067	Q9UHF7	0.49599
Q2Q067	O43516	0.18712
Q2Q067	P53675	0.24736
Q2Q067	Q15323	0.16267
Q2Q067	P62318	0.84614
Q2Q067	P62826	0.66768
Q2Q067	O43189	0.44104
Q2Q067	Q99816	0.43689
Q2Q067	Q96ST8	0.56668
Q2Q067	Q8IVN3	0.4066
Q2Q067	Q99741	0.50582
Q2Q067	Q96GY3	0.46738
Q2Q067	P62508	0.46129
Q2Q067	Q9BZQ8	0.32218
Q2Q067	Q6XQN6	0.2015
Q2Q067	Q92797	0.68324
Q2Q067	Q6P1J9	0.62418
Q2Q067	Q9ULD5	0.062315
Q2Q067	Q6AI39	0.33904
Q2Q067	P51508	0.10324
Q2Q067	Q9BQA1	0.9983
Q2Q067	Q9UBB5	1.1786
Q2Q067	Q14CZ0	0.74667
Q2Q067	Q0PNE2	0.33673
Q2Q067	Q14590	0.087195
Q2Q067	Q93015	0.29439
Q2Q067	A6NDD8	0.3158
Q2Q067	Q17RN3	0.10332
Q2Q067	F5H4B4	0.42889
Q2Q067	Q8TF40	0.45661
Q2Q067	Q9BRP4	0.40677
Q2Q067	Q9Y3V2	0.81743
Q2Q067	A5YKK6	1.0576
Q2Q067	Q15386	0.2463
Q2Q067	Q15596	0.62232
Q2Q067	P48380	0.33275
Q2Q067	Q8WVM0	0.53637
Q2Q067	O95295	0.43916
Q2Q067	Q9H2U6	0.10589
Q2Q067	O14813	0.33768
Q2Q067	Q9BSW2	0.75194
Q2Q067	Q08E93	0.19215
Q2Q067	Q69YL0	0.19215
Q2Q067	Q13405	0.64337
Q2Q067	Q9NTW7	0.22405
Q2Q067	Q9Y606	0.66525
Q2Q067	P08779	0.73022
Q2Q067	Q16656	0.38036
Q2Q067	Q9Y2Y9	0.75393
Q2Q067	Q96NG8	0.11072
Q2Q067	Q96GC5	0.64337
Q2Q067	Q9HCK5	0.70956
Q2Q067	Q5TC04	0.013253
Q2Q067	P35712	0.55247
Q2Q067	Q6ZN06	0.22154
Q2Q067	Q9UHL3	0.012028
Q2Q067	Q6NVV1	0.18467
Q2Q067	Q15233	0.57138
Q2Q067	Q13829	0.71763
Q2Q067	O95751	0.56499
Q2Q067	Q6VY07	0.58097
Q2Q067	Q9NXZ1	0.15082
Q2Q067	P41182	0.19432

Q2Q067	Q9Y2X9	0.61743
Q2Q067	P19474	0.37901
Q2Q067	Q15554	0.475
Q2Q067	P54259	0.86885
Q2Q067	Q5W111	0.043034
Q2Q067	Q8WWY3	0.36663
Q2Q067	Q9H5H4	0.10741
Q2Q067	Q99497	0.11091
Q2Q067	I6L9F6	0.11246
Q2Q067	Q86VX2	0.42881
Q2Q067	Q5SQN1	0.28062
Q2Q067	P10828	0.51689
Q2Q067	Q96BZ8	0.29573
Q2Q067	P82930	0.39251
Q2Q067	Q96AE4	0.56102
Q2Q067	Q12988	0.35026
Q2Q067	Q15562	0.84489
Q2Q067	Q9GZS3	1.1095
Q2Q067	P15880	1.0109
Q2Q067	Q96R06	0.86521
Q2Q067	Q8N9K7	0.19215
Q2Q067	P40429	0.7867
Q2Q067	Q8IZU1	0.32741
Q2Q067	Q5MJ09	0.07779
Q2Q067	O60260	0.44894
Q2Q067	Q8NE18	0.72254
Q2Q067	Q9P2H0	0.28783
Q2Q067	Q9BYN8	0.45686
Q2Q067	P30793	0.16239
Q2Q067	A6NJR5	0.23617
Q2Q067	O94818	0.54789
Q2Q067	Q9NUL3	0.61088
Q2Q067	Q9H074	0.46652
Q2Q067	Q460N3	0.2508
Q2Q067	O96019	0.89356
Q2Q067	Q9HC52	0.78769
Q2Q067	Q9H1H9	0.44235
Q2Q067	Q01664	0.59316
Q2Q067	Q9UDY8	0.24277
Q2Q067	Q9BXH1	0.34828
Q2Q067	P60371	0.34715
Q2Q067	Q9BU76	0.40566
Q2Q067	P55197	1.3702
Q2Q067	Q5JTD0	0.96483
Q2Q067	Q9UF83	0.12181
Q2Q067	Q8NBB4	0.10279
Q2Q067	Q06265	0.074678
Q2Q067	Q9NUP1	0.67061
Q2Q067	Q9BUL9	0.97869
Q2Q067	O00570	0.88029
Q2Q067	P28072	0.50319
Q2Q067	Q6A162	0.12452
Q2Q067	Q9GZS1	0.3579
Q2Q067	Q8N3X6	0.48739
Q2Q067	Q7Z4G1	0.81276
Q2Q067	Q6ZUT4	0.31485
Q2Q067	Q8N814	0.45016
Q2Q067	P61024	0.63428
Q2Q067	P0DI83	0.34546
Q2Q067	Q96JI7	0.54765
Q2Q067	P82979	0.35432
Q2Q067	Q9UBB6	0.2024
Q2Q067	Q14159	0.62808
Q2Q067	Q9BRQ6	0.67393
Q2Q067	P09132	0.32235
Q2Q067	Q8IZ63	0.19215
Q2Q067	Q96H40	0.22807
Q2Q067	Q9Y6E7	0.37851
Q2Q067	P49910	0.70866
Q2Q067	Q6P1X6	0.3233
Q2Q067	Q9P0M2	0.10037
Q2Q067	Q8N5U6	1.072
Q2Q067	O14604	0.36209
Q2Q067	Q9ULC6	0.53444
Q2Q067	Q15326	1.2007
Q2Q067	I3L1I5	0.23961

Q2Q067	A6NCD4	0.12554
Q2Q067	Q9NWU5	0.31991
Q2Q067	Q9NZV7	0.26783
Q2Q067	Q96IS3	0.19365
Q2Q067	Q9P299	0.14226
Q2Q067	Q86UQ8	0.39593
Q2Q067	O95243	0.69212
Q2Q067	O95801	0.11363
Q2Q067	P18847	1.2352
Q2Q067	Q9NWN3	0.1566
Q2Q067	Q9UHX1	0.60346
Q2Q067	P49427	0.3813
Q2Q067	Q9HA90	0.29573
Q2Q067	Q14684	0.20074
Q2Q067	P0CJ87	0.27517
Q2Q067	Q8N7X2	0.19215
Q2Q067	Q8TEV9	0.29626
Q2Q067	P51531	0.63605
Q2Q067	Q8WZA9	0.29573
Q2Q067	P60660	0.27881
Q2Q067	P53990	0.48207
Q2Q067	Q5VTL8	0.40188
Q2Q067	Q9NPG3	0.87003
Q2Q067	Q99708	0.6494
Q2Q067	A0PJX2	0.18467
Q2Q067	Q96FJ8	0.19215
Q2Q067	P08729	1.4125
Q2Q067	P10515	0.36792
Q2Q067	Q8N9N5	0.22482
Q2Q067	Q96F86	0.66099
Q2Q067	P18887	0.64626
Q2Q067	Q32MQ0	0.32623
Q2Q067	P49716	0.43022
Q2Q067	Q8WYR4	0.32597
Q2Q067	P41970	0.90765
Q2Q067	P57076	0.14907
Q2Q067	Q8N684	0.43159
Q2Q067	Q8N7L0	0.12804
Q2Q067	O75807	0.44729
Q2Q067	Q9HAF1	1.0414
Q2Q067	Q6P2D0	0.095999
Q2Q067	Q86X83	0.28783
Q2Q067	Q9HCL3	0.2406
Q2Q067	O43805	0.51752
Q2Q067	Q3C1V9	0.15384
Q2Q067	P46782	0.52651
Q2Q067	Q6U7Q0	0.10668
Q2Q067	Q93083	0.18499
Q2Q067	P78385	0.1632
Q2Q067	Q8NAB5	0.19215
Q2Q067	Q14204	0.3083
Q2Q067	Q9NSQ0	0.16808
Q2Q067	P57060	0.30098
Q2Q067	Q8WTT2	0.14455
Q2Q067	Q7Z4Q2	0.41883
Q2Q067	C9J302	0.19051
Q2Q067	Q6ZSN7	0.12388
Q2Q067	Q8IVM7	0.19215
Q2Q067	Q7Z7A3	0.59907
Q2Q067	P0CW21	0.19215
Q2Q067	Q13111	1.0018
Q2Q067	Q96LJ8	0.25736
Q2Q067	Q8WXF8	0.84218
Q2Q067	Q5SW79	0.7589
Q2Q067	A6NIX2	1.0036
Q2Q067	G3V211	0.40883
Q2Q067	Q9UKJ3	0.39207
Q2Q067	O75865	0.30975
Q2Q067	Q9Y5Q8	0.68039
Q2Q067	Q86VY4	0.47962
Q2Q067	P22033	0.20874
Q2Q067	Q9BRJ2	0.31691
Q2Q067	Q8TD23	0.51835
Q2Q067	Q8N137	0.78742
Q2Q067	Q9BW11	0.37545
Q2Q067	A1L429	0.019044

50997	Q2Q067	Q9NQ87	0.49684
50998	Q2Q067	O00762	0.52837
50999	Q2Q067	Q9HAB8	0.28376
51000	Q2Q067	Q9Y244	0.32215
51001	Q2Q067	Q5PSV4	0.35681
51002	Q2Q067	Q6PD74	0.24076
51003	Q2Q067	P13349	0.33049
51004	Q2Q067	Q9UJG1	0.43623
51005	Q2Q067	Q8ND07	0.40143
51006	Q2Q067	Q8IUC1	0.16378
51007	Q2Q067	P0DL12	0.16593
51008	Q2Q067	Q7Z3Z0	0.23725
51009	Q2Q067	Q53FD0	0.45629
51010	Q2Q067	Q9H0F6	0.11885
51011	Q2Q067	Q9Y312	0.079991
51012	Q2Q067	Q9UI30	0.339
51013	Q2Q067	Q96B49	0.3098
51014	Q2Q067	A6NIE6	0.28619
51015	Q2Q067	Q9NV31	0.85286
51016	Q2Q067	Q96B23	0.49034
51017	Q2Q067	Q9Y2X8	0.22096
51018	Q2Q067	P10155	0.41704
51019	Q2Q067	Q96DC7	0.5972
51020	Q2Q067	Q9H6J7	0.15082
51021	Q2Q067	Q96SK3	0.14687
51022	Q2Q067	P61457	1.0532
51023	Q2Q067	Q96IT1	0.64563
51024	Q2Q067	Q147U1	0.11372
51025	Q2Q067	Q15697	0.46776
51026	Q2Q067	O43255	0.34072
51027	Q2Q067	Q9BQS6	0.79385
51028	Q2Q067	Q96LW1	0.22688
51029	Q2Q067	Q96P71	0.47123
51030	Q2Q067	Q9NZL3	0.6383
51031	Q2Q067	Q6VUC0	0.47835
51032	Q2Q067	Q9Y2J8	0.38199
51033	Q2Q067	O96004	0.49229
51034	Q2Q067	Q8N0Z3	0.46631
51035	Q2Q067	Q8WV92	0.23327
51036	Q2Q067	Q92985	0.61347
51037	Q2Q067	O95400	0.73093
51038	Q2Q067	P35268	0.67288
51039	Q2Q067	Q8TBY8	0.26354
51040	Q2Q067	Q9Y6M1	0.34676
51041	Q2Q067	O94927	0.45285
51042	Q2Q067	Q6P1K2	1.1493
51043	Q2Q067	O43390	0.54895
51044	Q2Q067	Q9HBM1	0.69818
51045	Q2Q067	Q15059	1.1367
51046	Q2Q067	Q53SZ7	0.29573
51047	Q2Q067	Q8WXU2	0.71405
51048	Q2Q067	A8MYP8	0.15024
51049	Q2Q067	Q9UL49	0.24991
51050	Q2Q067	Q7Z6R9	0.32732
51051	Q2Q067	Q8N398	0.27203
51052	Q2Q067	Q8HWS3	0.26676
51053	Q2Q067	Q7L2R6	0.22332
51054	Q2Q067	Q5JSQ8	0.029629
51055	Q2Q067	O00167	0.59874
51056	Q2Q067	Q9NZD8	0.81753
51057	Q2Q067	O75794	0.50176
51058	Q2Q067	Q9BXM0	0.61991
51059	Q2Q067	P60409	0.34715
51060	Q2Q067	P49419	0.649
51061	Q2Q067	Q8N1B3	0.57535
51062	Q2Q067	P08151	0.29798
51063	Q2Q067	Q9BZE3	0.46596
51064	Q2Q067	Q969F9	0.59662
51065	Q2Q067	O60437	0.71369
51066	Q2Q067	Q9C086	1.2094
51067	Q2Q067	P15121	0.28053
51068	Q2Q067	Q5UIP0	0.48693
51069	Q2Q067	O60829	0.27828
51070	Q2Q067	Q15653	1.0047
51071	Q2Q067	Q9BW61	0.29409
51072	Q2Q067	P49750	1.0431

Q2Q067	P36639	0.30724
Q2Q067	Q5TC12	0.42044
Q2Q067	O96006	0.45919
Q2Q067	Q01196	0.33705
Q2Q067	Q00613	0.88678
Q2Q067	A1A4V9	0.075963
Q2Q067	Q9H211	0.74533
Q2Q067	O14618	0.45271
Q2Q067	Q8IYI0	0.17721
Q2Q067	O75698	0.19215
Q2Q067	P56181	0.27543
Q2Q067	Q9HCM1	0.40736
Q2Q067	O43439	0.94602
Q2Q067	Q5VYK3	0.11595
Q2Q067	Q96TA1	0.30806
Q2Q067	Q99676	0.14687
Q2Q067	Q9NRR4	0.44199
Q2Q067	Q8N9H9	0.19215
Q2Q067	Q5VZE5	0.18621
Q2Q067	Q6ZP68	0.42662
Q2Q067	Q9UJY4	0.44418
Q2Q067	O75478	0.44071
Q2Q067	Q16082	0.95302
Q2Q067	Q9BRG1	1.1485
Q2Q067	P33316	0.62114
Q2Q067	Q96CM4	0.027173
Q2Q067	Q13435	0.41643
Q2Q067	Q13077	0.22767
Q2Q067	Q17RM4	0.19498
Q2Q067	Q92552	0.68082
Q2Q067	A6NNH0	0.42645
Q2Q067	Q01105	0.84414
Q2Q067	Q86UU0	0.71419
Q2Q067	Q6ZRC1	0.19215
Q2Q067	Q96T88	0.26705
Q2Q067	Q9P0W5	0.37055
Q2Q067	Q9UJW0	0.46824
Q2Q067	Q17RH7	0.19215
Q2Q067	Q13772	0.3324
Q2Q067	P24534	0.61369
Q2Q067	Q0IIN9	0.026247
Q2Q067	O75935	0.841
Q2Q067	P23490	0.7457
Q2Q067	Q49AS3	0.071261
Q2Q067	Q9P206	0.37524
Q2Q067	P78406	0.49064
Q2Q067	Q8N8G6	0.071687
Q2Q067	Q6P3W6	0.32407
Q2Q067	P19388	0.64726
Q2Q067	Q5T8A7	0.68176
Q2Q067	Q96SL8	0.081509
Q2Q067	P08133	0.64843
Q2Q067	P05813	0.081456
Q2Q067	P37275	0.78485
Q2Q067	Q70Z53	0.62556
Q2Q067	Q8IXQ3	0.26691
Q2Q067	O00273	0.28887
Q2Q067	Q8IYX7	0.57176
Q2Q067	Q9BYR9	0.028225
Q2Q067	Q8IWY8	0.091092
Q2Q067	Q6ZUU3	0.19215
Q2Q067	P19367	0.28571
Q2Q067	Q14140	0.94414
Q2Q067	Q7Z7A1	1.0945
Q2Q067	O75081	1.154
Q2Q067	Q8N5H3	0.29573
Q2Q067	Q5SXM2	0.64653
Q2Q067	Q6ZNR8	0.19215
Q2Q067	Q12815	0.25268
Q2Q067	Q96NT3	0.21475
Q2Q067	Q05516	0.78221
Q2Q067	Q8NEK8	0.32016
Q2Q067	Q32MZ4	1.1057
Q2Q067	P20749	0.40075
Q2Q067	Q8NF64	1.2752
Q2Q067	Q05481	0.16589

Q2Q067	Q5EE01	0.55233
Q2Q067	Q02930	0.80635
Q2Q067	Q8N377	0.19215
Q2Q067	Q9HD15	0.55021
Q2Q067	P54274	0.42787
Q2Q067	Q9Y6K1	0.33326
Q2Q067	P0C843	0.22236
Q2Q067	Q96DI7	0.58275
Q2Q067	Q8N888	0.46193
Q2Q067	P52435	0.58166
Q2Q067	A6NNW6	0.23001
Q2Q067	Q9BYJ9	0.3979
Q2Q067	Q12765	0.25535
Q2Q067	P01009	0.14201
Q2Q067	Q9NVW2	0.27915
Q2Q067	O76013	0.16267
Q2Q067	Q8NCK3	0.094185
Q2Q067	Q9BW71	0.59938
Q2Q067	O00743	0.72072
Q2Q067	Q460N5	0.31041
Q2Q067	Q8NDW4	0.11252
Q2Q067	Q9NRN7	0.42659
Q2Q067	A6NKD9	0.6484
Q2Q067	O14929	0.74515
Q2Q067	Q15696	0.19822
Q2Q067	A6NGG3	0.19215
Q2Q067	O00257	0.94276
Q2Q067	Q8NDF8	0.27957
Q2Q067	Q2NL82	0.2207
Q2Q067	Q5VU69	0.16708
Q2Q067	O60543	0.30174
Q2Q067	Q9BSI4	0.20173
Q2Q067	P38117	0.080424
Q2Q067	Q9P1D8	0.19215
Q2Q067	O75820	0.076987
Q2Q067	Q9NZJ0	0.72356
Q2Q067	O43172	0.67307
Q2Q067	Q9NZI6	0.4959
Q2Q067	P49116	0.54835
Q2Q067	Q9UIF7	0.36542
Q2Q067	P22090	0.40762
Q2Q067	Q7Z7J5	0.5181
Q2Q067	P10074	0.30665
Q2Q067	Q9BXW4	0.14432
Q2Q067	Q15046	0.34151
Q2Q067	Q8WWK9	0.20995
Q2Q067	A8CG34	0.26858
Q2Q067	Q9Y5N5	0.24625
Q2Q067	O75489	0.17115
Q2Q067	Q7Z2E3	0.447
Q2Q067	Q9HCD5	1.0622
Q2Q067	Q9NX40	0.42502
Q2Q067	Q3LI68	0.58843
Q2Q067	O60315	0.25286
Q2Q067	Q04206	0.70317
Q2Q067	P36543	0.23471
Q2Q067	A8MV72	0.14505
Q2Q067	Q99551	0.689
Q2Q067	Q9UM82	0.50559
Q2Q067	P49770	0.23477
Q2Q067	Q8IV20	0.12239
Q2Q067	P11387	0.64508
Q2Q067	P86478	0.19215
Q2Q067	Q15181	0.2614
Q2Q067	A5PLN7	0.29573
Q2Q067	Q9UJY5	0.4651
Q2Q067	Q6P4D5	0.34883
Q2Q067	Q96FA7	0.62008
Q2Q067	Q53TS8	0.13687
Q2Q067	Q8WYP5	0.68915
Q2Q067	P46778	0.65612
Q2Q067	Q6ZU52	0.28783
Q2Q067	Q99456	0.20138
Q2Q067	Q9BXL5	0.32507
Q2Q067	Q6AWC8	0.21229
Q2Q067	Q9P0L1	0.14032

Q2Q067	O95336	0.24312
Q2Q067	A8MVW5	0.64088
Q2Q067	Q8N8F4	0.11552
Q2Q067	Q9ULV3	0.22922
Q2Q067	A6NC62	0.073722
Q2Q067	Q9H944	1.0474
Q2Q067	Q3LI67	0.19215
Q2Q067	Q9BR46	0.45148
Q2Q067	P61981	0.51156
Q2Q067	O75896	0.20705
Q2Q067	Q5T752	0.19215
Q2Q067	P42695	0.28165
Q2Q067	Q9BYR0	0.34715
Q2Q067	P12980	0.87666
Q2Q067	Q8N7B6	0.19165
Q2Q067	Q5CZC0	0.56427
Q2Q067	P61604	0.73497
Q2Q067	Q99457	0.24396
Q2Q067	Q9H3R5	0.53
Q2Q067	Q5DJT8	0.070895
Q2Q067	P50502	0.60041
Q2Q067	Q9UK45	0.80491
Q2Q067	Q14119	0.17633
Q2Q067	Q5BKY6	0.19215
Q2Q067	Q9BPZ2	0.34715
Q2Q067	P50548	0.32358
Q2Q067	Q9H4Z2	0.58991
Q2Q067	Q8N319	0.73498
Q2Q067	Q96C86	0.89985
Q2Q067	Q00994	0.6878
Q2Q067	Q8NHV4	0.67001
Q2Q067	P30279	0.70512
Q2Q067	Q92870	0.70196
Q2Q067	P05062	0.25069
Q2Q067	P30084	0.17267
Q2Q067	Q6P9A1	0.19035
Q2Q067	Q13868	0.60425
Q2Q067	Q12933	0.61278
Q2Q067	Q9NWD9	0.26682
Q2Q067	Q6ZNE5	0.33497
Q2Q067	Q96MA1	0.16114
Q2Q067	Q8TAB7	0.43978
Q2Q067	A8MQ03	0.26889
Q2Q067	Q16633	0.60416
Q2Q067	Q9ULV5	0.41665
Q2Q067	O43639	0.38531
Q2Q067	Q9HAN9	0.19872
Q2Q067	Q8TEV8	0.19215
Q2Q067	O94829	0.9899
Q2Q067	Q8IV36	0.43406
Q2Q067	P62140	0.72381
Q2Q067	Q8IXT2	0.024199
Q2Q067	Q9UKY7	0.44099
Q2Q067	Q96PQ6	0.24081
Q2Q067	Q86SE9	1.0134
Q2Q067	Q9Y3B6	0.38138
Q2Q067	Q9UHC1	0.4197
Q2Q067	Q8N782	0.22914
Q2Q067	Q6P1M3	0.83943
Q2Q067	Q9NSD9	0.39523
Q2Q067	Q969S2	0.071613
Q2Q067	B1AMM8	0.10475
Q2Q067	Q5T1C6	0.33137
Q2Q067	P82663	0.45173
Q2Q067	P07954	0.079242
Q2Q067	Q96NL3	0.12037
Q2Q067	Q6NXT1	0.33708
Q2Q067	Q96B36	0.81015
Q2Q067	O60238	0.19067
Q2Q067	Q96B01	0.54081
Q2Q067	Q96HG1	0.19215
Q2Q067	Q4W5G0	0.21381
Q2Q067	P26641	0.45788
Q2Q067	Q13625	0.37536
Q2Q067	Q8WTR7	0.62849
Q2Q067	Q6P1N0	0.23728

Q2Q067	Q9NYJ8	0.52371
Q2Q067	Q13547	0.82322
Q2Q067	Q6PI26	1.1443
Q2Q067	A8MV65	0.39593
Q2Q067	Q6ZSJ8	0.29573
Q2Q067	Q9NP66	1.0069
Q2Q067	Q14511	0.63404
Q2Q067	Q9UP83	1.2689
Q2Q067	Q05195	0.55058
Q2Q067	P08238	0.62824
Q2Q067	Q8TAW3	0.12685
Q2Q067	Q96AV8	0.33531
Q2Q067	Q6ZTZ1	0.1662
Q2Q067	Q5T5F5	0.19215
Q2Q067	P38919	0.23137
Q2Q067	Q9BTT4	0.63393
Q2Q067	Q9BXX3	0.25565
Q2Q067	P63167	1.4147
Q2Q067	P0C867	0.13702
Q2Q067	P51815	0.097938
Q2Q067	P14373	0.23549
Q2Q067	Q8N6F7	0.31193
Q2Q067	Q5VWX1	0.68611
Q2Q067	P0DM35	0.42541
Q2Q067	Q9UJY1	0.41943
Q2Q067	Q14103	0.83961
Q2Q067	Q9UIS9	0.51543
Q2Q067	P78358	0.47001
Q2Q067	O43752	0.067951
Q2Q067	P55345	0.3446
Q2Q067	O60506	0.3481
Q2Q067	A5LHX3	0.30339
Q2Q067	Q96MF4	0.19092
Q2Q067	O00712	0.2268
Q2Q067	Q8NF67	0.25537
Q2Q067	P22061	0.21052
Q2Q067	Q9NZI5	0.4457
Q2Q067	Q8N9H6	0.083116
Q2Q067	Q99990	0.54172
Q2Q067	Q9BYC8	0.44271
Q2Q067	Q93074	0.4745
Q2Q067	Q8TF20	0.15472
Q2Q067	Q6ICC9	0.24396
Q2Q067	Q9UGC7	0.18502
Q2Q067	Q01201	1.0626
Q2Q067	P34949	0.13199
Q2Q067	Q6UUV9	1.4899
Q2Q067	Q9HB58	0.24792
Q2Q067	Q9UBU9	0.49442
Q2Q067	Q6ZR52	0.16589
Q2Q067	Q9BSB4	0.25531
Q2Q067	O00534	0.16164
Q2Q067	Q8IYA8	0.29573
Q2Q067	Q7Z6G3	0.36954
Q2Q067	O60930	0.22588
Q2Q067	A3KN83	0.5167
Q2Q067	P60604	0.22096
Q2Q067	Q3B820	0.28732
Q2Q067	Q5H9J7	0.46546
Q2Q067	Q15528	1.1757
Q2Q067	P0CB43	0.050495
Q2Q067	Q99684	0.3657
Q2Q067	P52741	0.13205
Q2Q067	Q9P1Y5	0.26677
Q2Q067	Q5TKA1	0.35625
Q2Q067	Q9UDV7	0.085876
Q2Q067	Q5FBB7	0.72076
Q2Q067	Q9P1A6	0.16775
Q2Q067	Q8N9P6	0.19215
Q2Q067	P35244	0.55376
Q2Q067	O00566	0.41428
Q2Q067	P49450	0.25741
Q2Q067	O14662	0.35498
Q2Q067	P62888	0.77652
Q2Q067	Q15006	0.27584
Q2Q067	Q53FT3	0.22423

Q2Q067	Q13884	0.42605
Q2Q067	Q14814	0.57726
Q2Q067	P39880	0.6071
Q2Q067	O95347	0.34876
Q2Q067	Q8N9V7	0.24893
Q2Q067	Q93073	0.44152
Q2Q067	Q13257	0.72968
Q2Q067	Q8NFR7	0.26479
Q2Q067	Q86XR8	0.98037
Q2Q067	Q8IUR0	0.49239
Q2Q067	P18850	0.64812
Q2Q067	Q9NV88	0.57387
Q2Q067	Q9UBP6	0.76859
Q2Q067	Q6QNY0	0.46851
Q2Q067	Q8TF68	0.068884
Q2Q067	Q3KQV3	0.11652
Q2Q067	O43617	0.26475
Q2Q067	P50549	0.95391
Q2Q067	P83436	0.3792
Q2Q067	Q92990	0.060339
Q2Q067	P32780	0.72187
Q2Q067	Q99594	1.1492
Q2Q067	Q9H777	0.23511
Q2Q067	Q9BZV1	1.0068
Q2Q067	Q86WB0	0.84294
Q2Q067	Q96AQ6	0.50051
Q2Q067	Q8N2Z9	0.53289
Q2Q067	O94983	1.342
Q2Q067	Q9H322	0.36356
Q2Q067	A6NDE8	0.019044
Q2Q067	Q8WUH2	0.63392
Q2Q067	Q8IUW3	0.40712
Q2Q067	Q8IUE0	0.32768
Q2Q067	Q5JY77	0.47001
Q2Q067	Q9XRX5	0.28783
Q2Q067	Q8N1N4	0.16378
Q2Q067	Q9Y3D9	0.3707
Q2Q067	P61573	0.36503
Q2Q067	O00204	0.43043
Q2Q067	O75208	0.25111
Q2Q067	Q9P255	0.1589
Q2Q067	Q8IZP0	0.2508
Q2Q067	Q96FH0	0.21828
Q2Q067	Q9H6T0	0.087135
Q2Q067	P32969	0.88677
Q2Q067	A6H8Y1	0.70245
Q2Q067	Q09MP3	0.058385
Q2Q067	O76031	0.30997
Q2Q067	Q8NC26	0.18763
Q2Q067	P17483	0.13846
Q2Q067	Q6L8G5	0.19215
Q2Q067	O95999	0.23471
Q2Q067	Q9Y3C0	0.24973
Q2Q067	P14316	0.97948
Q2Q067	Q96EY5	0.23232
Q2Q067	A8MT69	0.43813
Q2Q067	Q6UYE1	0.46973
Q2Q067	O43296	0.1152
Q2Q067	Q86Y79	0.3086
Q2Q067	P58004	0.15724
Q2Q067	A6NKX1	0.19215
Q2Q067	Q6R2W3	0.44069
Q2Q067	Q76L83	0.47583
Q2Q067	Q8NAX2	0.29573
Q2Q067	O94776	1.0289
Q2Q067	Q9H4T2	0.058453
Q2Q067	Q9UBU7	0.077023
Q2Q067	O15453	0.35671
Q2Q067	P56555	0.54172
Q2Q067	O15379	0.5597
Q2Q067	O75486	0.54134
Q2Q067	O15266	0.52908
Q2Q067	P36956	0.23929
Q2Q067	P0CG30	0.2324
Q2Q067	P61970	0.39203
Q2Q067	P62277	1.1246

Q2Q067	Q99567	0.79612
Q2Q067	Q8N1F7	0.60294
Q2Q067	P18615	0.93209
Q2Q067	Q9C0E2	0.85201
Q2Q067	A6NFD8	0.28241
Q2Q067	P60372	0.34715
Q2Q067	Q8IWL3	0.48319
Q2Q067	Q86WS4	0.19215
Q2Q067	O95948	0.37793
Q2Q067	Q8N1B4	0.47716
Q2Q067	P56945	0.57099
Q2Q067	O14709	0.16182
Q2Q067	P0CL81	0.019044
Q2Q067	Q99933	0.82086
Q2Q067	P41134	0.31519
Q2Q067	C9JBD0	0.29933
Q2Q067	P41161	0.20842
Q2Q067	Q08ER8	0.14861
Q2Q067	P0C7M4	0.25814
Q2Q067	Q5T681	0.38114
Q2Q067	Q9UJT9	0.31311
Q2Q067	Q9H1Z4	0.32989
Q2Q067	Q13601	0.38006
Q2Q067	Q86SZ2	0.3221
Q2Q067	O60535	0.51608
Q2Q067	Q9BQ87	0.39593
Q2Q067	Q9UKN8	0.54599
Q2Q067	Q14192	0.65721
Q2Q067	Q5T5D7	0.13247
Q2Q067	P13646	0.18021
Q2Q067	P07900	0.61392
Q2Q067	Q86UC2	0.20504
Q2Q067	Q96EV8	0.2465
Q2Q067	P56537	1.1743
Q2Q067	Q9BWW4	0.47427
Q2Q067	Q13123	0.48874
Q2Q067	Q70EL1	0.33398
Q2Q067	Q9H171	0.16258
Q2Q067	P57723	0.40522
Q2Q067	Q9NQX1	0.51532
Q2Q067	P50616	0.29444
Q2Q067	Q9HC77	0.76588
Q2Q067	Q9UPV0	1.5272
Q2Q067	Q7Z5D8	0.32756
Q2Q067	Q03188	0.23296
Q2Q067	Q9Y5R5	0.15414
Q2Q067	Q9H098	0.40352
Q2Q067	Q8TE76	0.15693
Q2Q067	Q5SY85	0.12635
Q2Q067	P05114	0.23888
Q2Q067	Q92800	0.44805
Q2Q067	Q96M61	0.28783
Q2Q067	Q8IYB1	0.10399
Q2Q067	O60318	0.30747
Q2Q067	Q9BWK5	0.39148
Q2Q067	Q86WR7	0.33983
Q2Q067	Q6NZI2	1.1279
Q2Q067	Q5VYV7	0.28783
Q2Q067	Q96EZ8	0.78764
Q2Q067	Q15633	0.87516
Q2Q067	Q96S55	0.1724
Q2Q067	Q6ZMW2	0.097854
Q2Q067	Q9UNA4	0.29656
Q2Q067	P53672	0.39249
Q2Q067	Q8N5I9	0.29573
Q2Q067	Q9H825	0.10872
Q2Q067	P06748	0.44904
Q2Q067	P30876	0.57966
Q2Q067	Q107X0	0.34746
Q2Q067	O14757	0.545
Q2Q067	A8MUX0	0.11141
Q2Q067	Q9UNH5	0.57225
Q2Q067	P49789	0.54893
Q2Q067	Q9NWV8	0.5884
Q2Q067	Q7RTS7	0.16378
Q2Q067	O95503	0.90908

Q2Q067	Q8WU03	0.30493
Q2Q067	P19387	0.474
Q2Q067	Q8N699	0.27397
Q2Q067	Q86XN7	0.29573
Q2Q067	Q4LE39	0.91834
Q2Q067	Q9NVX0	0.98487
Q2Q067	Q86VX9	0.59741
Q2Q067	Q9UER7	0.86986
Q2Q067	Q6P1R3	0.28243
Q2Q067	Q9C0B2	0.058025
Q2Q067	P62241	0.65617
Q2Q067	O00148	0.23552
Q2Q067	Q86WV1	0.53429
Q2Q067	Q16520	0.99622
Q2Q067	P15976	0.29548
Q2Q067	O95931	0.78662
Q2Q067	P59991	0.40476
Q2Q067	Q9UNL4	0.44852
Q2Q067	Q9BWX5	0.33243
Q2Q067	Q96G21	0.86474
Q2Q067	Q8N5N4	0.19583
Q2Q067	Q86X51	0.093973
Q2Q067	Q6ZRI6	0.19455
Q2Q067	Q8N8U2	0.41458
Q2Q067	Q6L8H1	0.19215
Q2Q067	Q96MY7	0.5595
Q2Q067	Q8N7J2	0.51822
Q2Q067	Q8N8K9	0.042224
Q2Q067	Q9H307	0.64076
Q2Q067	Q8N660	0.72826
Q2Q067	Q9P2B4	0.25434
Q2Q067	P40222	0.3161
Q2Q067	O75347	0.60524
Q2Q067	Q5T3J3	0.99943
Q2Q067	Q9Y6X4	0.63552
Q2Q067	O75586	0.71829
Q2Q067	Q6P582	0.33705
Q2Q067	Q8N196	0.70099
Q2Q067	Q9Y2G9	0.39051
Q2Q067	Q96JC9	0.97359
Q2Q067	Q9H0E3	0.58496
Q2Q067	F5GYI3	0.51184
Q2Q067	Q9H8V8	0.19215
Q2Q067	Q9BXW3	0.19215
Q2Q067	Q66LE6	0.34277
Q2Q067	O60356	0.49079
Q2Q067	Q3KP66	0.52159
Q2Q067	Q2M3W8	0.24938
Q2Q067	Q15542	1.0491
Q2Q067	Q15172	0.209
Q2Q067	Q17RC7	0.19694
Q2Q067	O14686	0.27868
Q2Q067	Q16626	0.056025
Q2Q067	O15535	0.058453
Q2Q067	Q9NXR1	0.3083
Q2Q067	Q6MZP7	0.57419
Q2Q067	P14136	0.11798
Q2Q067	Q9P0M9	0.30628
Q2Q067	Q6SJ96	0.24496
Q2Q067	Q9H8Y8	0.28916
Q2Q067	Q6H8Q1	0.28044
Q2Q067	A6NHQ4	0.29095
Q2Q067	Q69383	0.36503
Q2Q067	O00423	0.37113
Q2Q067	Q13200	0.50051
Q2Q067	Q3LI83	0.39972
Q2Q067	Q9BYQ5	0.34715
Q2Q067	Q8TEL6	0.3881
Q2Q067	Q7RTT5	0.12144
Q2Q067	Q4VC44	0.29551
Q2Q067	A6NCN2	0.16378
Q2Q067	Q6ZP82	0.28783
Q2Q067	Q03933	0.71603
Q2Q067	Q8TDN6	0.28013
Q2Q067	P28356	0.23273
Q2Q067	Q9HCH5	0.54012

Q2Q067	Q6ZU67	0.19686
Q2Q067	P29966	0.52276
Q2Q067	Q13432	0.37123
Q2Q067	Q92754	0.53491
Q2Q067	Q96I23	0.26103
Q2Q067	Q96CN9	0.44998
Q2Q067	O94842	0.64158
Q2Q067	Q9ULN7	0.47117
Q2Q067	Q6L8G9	0.19215
Q2Q067	A8K8P3	0.75366
Q2Q067	Q8N3J3	0.11159
Q2Q067	Q9HC44	0.93086
Q2Q067	Q96ST3	0.76474
Q2Q067	O14628	0.21589
Q2Q067	P58557	0.52167
Q2Q067	Q9NSB4	0.17614
Q2Q067	Q8TBC5	0.058453
Q2Q067	Q15025	0.48873
Q2Q067	Q9UBQ0	0.22655
Q2Q067	Q5T7V8	1.0647
Q2Q067	Q9UL16	0.11333
Q2Q067	P61218	0.238
Q2Q067	Q8IWP9	0.54093
Q2Q067	O75570	0.18602
Q2Q067	Q96RK0	0.97805
Q2Q067	Q8N8G2	0.85361
Q2Q067	Q9Y237	0.30914
Q2Q067	A6ZKI3	0.24822
Q2Q067	P62244	0.75691
Q2Q067	Q86TU6	0.43187
Q2Q067	O15350	0.34389
Q2Q067	P05387	0.65612
Q2Q067	Q5SXJ2	0.72826
Q2Q067	Q6ZSS3	0.10643
Q2Q067	Q8N9K3	0.29573
Q2Q067	Q92759	0.66671
Q2Q067	Q6ZRS2	0.33704
Q2Q067	Q9BTK6	0.43906
Q2Q067	P13073	0.11152
Q2Q067	P35638	0.79814
Q2Q067	Q5T5J6	0.48112
Q2Q067	Q96EY4	0.24029
Q2Q067	A1L443	0.54396
Q2Q067	Q7L804	0.55959
Q2Q067	Q9NY61	0.87278
Q2Q067	Q8TDY2	0.85805
Q2Q067	Q68DL7	0.19215
Q2Q067	O75448	0.49164
Q2Q067	Q96AH0	0.25041
Q2Q067	Q9Y6J0	0.086745
Q2Q067	Q9NVR2	0.92109
Q2Q067	Q9NWM3	0.022228
Q2Q067	Q96GX9	0.35808
Q2Q067	Q92945	0.7916
Q2Q067	P48444	0.38198
Q2Q067	P54727	0.66024
Q2Q067	Q9UFW8	0.36593
Q2Q067	Q9NPI1	0.98674
Q2Q067	Q96PS1	0.24529
Q2Q067	Q9NQ48	0.21534
Q2Q067	Q03252	0.30514
Q2Q067	P41236	0.27377
Q2Q067	P02511	0.38569
Q2Q067	Q8IY42	0.62395
Q2Q067	Q96ST2	0.16697
Q2Q067	Q9Y2Z2	0.47355
Q2Q067	Q9BY42	0.32207
Q2Q067	I3L3R5	0.19215
Q2Q067	P57075	0.29437
Q2Q067	A6NL82	0.14181
Q2Q067	P54198	1.0547
Q2Q067	Q9H5F2	0.15819
Q2Q067	Q6NXS1	0.42296
Q2Q067	Q9UK76	0.45693
Q2Q067	Q8WVR3	0.29573
Q2Q067	Q7L5N1	1.1024

Q2Q067	Q6ISB3	0.65972
Q2Q067	A6NEY3	0.37471
Q2Q067	Q8TDR4	0.1456
Q2Q067	Q08378	0.28439
Q2Q067	Q02447	0.50481
Q2Q067	P25787	0.63799
Q2Q067	Q5T653	0.2979
Q2Q067	Q6ZR93	0.19215
Q2Q067	O15040	0.28783
Q2Q067	Q99909	0.12144
Q2Q067	Q5VYM1	0.094766
Q2Q067	Q6ZV77	0.19215
Q2Q067	P52594	0.40922
Q2Q067	Q86YF9	0.047542
Q2Q067	A8MXZ3	0.19215
Q2Q067	Q9NVS2	0.36613
Q2Q067	Q9NQE9	0.31788
Q2Q067	Q9NSD4	0.12553
Q2Q067	Q8NBR6	0.8215
Q2Q067	Q16534	0.19072
Q2Q067	P37837	0.2728
Q2Q067	Q14152	1.1237
Q2Q067	Q2M1K9	0.26579
Q2Q067	P62861	0.62997
Q2Q067	Q04727	0.6205
Q2Q067	Q6NT46	0.019044
Q2Q067	Q8TDH9	0.15809
Q2Q067	E9PAV3	0.23436
Q2Q067	Q96T58	0.68595
Q2Q067	Q8WY36	0.30214
Q2Q067	P08107	0.57181
Q2Q067	Q9C099	0.90062
Q2Q067	Q8N1L9	0.91996
Q2Q067	Q9P270	0.4152
Q2Q067	O60244	0.5241
Q2Q067	Q9H5P4	0.35833
Q2Q067	A2RUB1	0.29573
Q2Q067	Q5HYR2	0.51158
Q2Q067	Q86YP4	0.81915
Q2Q067	Q5BKX5	0.29573
Q2Q067	Q05877	0.44533
Q2Q067	H0YKK7	0.35087
Q2Q067	Q96SC8	0.09819
Q2Q067	Q9Y3Y4	0.58591
Q2Q067	A6NEV1	0.26898
Q2Q067	Q53HL2	0.90486
Q2Q067	Q13522	0.11603
Q2Q067	P52657	1.2277
Q2Q067	A6NGK3	0.014778
Q2Q067	Q6ZNA4	0.53595
Q2Q067	O14896	0.51883
Q2Q067	Q8IX12	0.94764
Q2Q067	P42766	0.84323
Q2Q067	Q06323	0.38843
Q2Q067	Q9Y534	0.30532
Q2Q067	A6NHQ2	0.14692
Q2Q067	Q8TAA5	0.18761
Q2Q067	P41218	0.58282
Q2Q067	O00401	0.63747
Q2Q067	Q5TH74	0.22525
Q2Q067	Q96SN8	0.46256
Q2Q067	P67775	0.7377
Q2Q067	Q9NVF7	0.41087
Q2Q067	Q96KD3	0.63352
Q2Q067	O43684	0.69341
Q2Q067	Q8NBT2	0.71527
Q2Q067	O75683	0.30487
Q2Q067	Q8NAV1	0.42622
Q2Q067	Q8WUF5	0.83767
Q2Q067	Q96HR8	0.85827
Q2Q067	P17030	0.11003
Q2Q067	Q9Y3R5	0.3434
Q2Q067	P86496	0.19215
Q2Q067	P51610	0.53119
Q2Q067	O60927	0.40566
Q2Q067	Q14934	0.50357

51757	Q2Q067	Q9H981	1.0513
51758	Q2Q067	Q86WP2	0.43952
51759	Q2Q067	Q9NUQ6	0.32989
51760	Q2Q067	Q9ULQ0	0.54306
51761	Q2Q067	A6NNL0	0.4993
51762	Q2Q067	Q504T8	0.15082
51763	Q2Q067	Q7Z3B4	0.84932
51764	Q2Q067	Q96K30	0.77794
51765	Q2Q067	P17031	0.12798
51766	Q2Q067	Q14457	0.50194
51767	Q2Q067	Q8TBK2	0.49261
51768	Q2Q067	Q7Z340	0.089995
51769	Q2Q067	P42704	1.0209
51770	Q2Q067	Q14532	0.095341
51771	Q2Q067	A6NLC8	0.64333
51772	Q2Q067	Q7L0Y3	0.88456
51773	Q2Q067	Q86UE3	0.1529
51774	Q2Q067	Q6ZMV5	0.23054
51775	Q2Q067	Q53TQ3	0.52874
51776	Q2Q067	O14910	0.61417
51777	Q2Q067	P09086	0.32347
51778	Q2Q067	Q5JTC6	0.47488
51779	Q2Q067	P04792	0.39356
51780	Q2Q067	P26447	0.27454
51781	Q2Q067	Q9H668	0.22561
51782	Q2Q067	P63279	0.78863
51783	Q2Q067	Q6ZR03	0.069256
51784	Q2Q067	O75362	0.74678
51785	Q2Q067	Q8N5D0	0.53453
51786	Q2Q067	Q96FX2	0.84723
51787	Q2Q067	Q6P656	0.0013873
51788	Q2Q067	Q96AB3	0.70499
51789	Q2Q067	O00189	0.23587
51790	Q2Q067	Q16670	0.14982
51791	Q2Q067	Q9BVV6	0.19215
51792	Q2Q067	Q96N95	0.24359
51793	Q2Q067	Q8N3U1	0.19215
51794	Q2Q067	Q9Y5A6	0.6937
51795	Q2Q067	Q9UBZ9	0.44017
51796	Q2Q067	B3EWG4	0.29573
51797	Q2Q067	Q92547	0.5473
51798	Q2Q067	A2RU37	0.0024558
51799	Q2Q067	Q9Y3Y2	0.66209
51800	Q2Q067	E9PJI5	0.2076
51801	Q2Q067	Q9H8Q6	0.5038
51802	Q2Q067	Q8TF47	0.22688
51803	Q2Q067	Q86YC2	0.65438
51804	Q2Q067	Q9NPI7	0.011348
51805	Q2Q067	Q13158	0.46394
51806	Q2Q067	Q7Z3Y9	0.20138
51807	Q2Q067	Q9NQ29	0.40207
51808	Q2Q067	O75971	0.63552
51809	Q2Q067	Q86TS7	0.19215
51810	Q2Q067	Q330K2	0.14487
51811	Q2Q067	P05386	1.1616
51812	Q2Q067	Q5T160	0.37733
51813	Q2Q067	O75175	1.3889
51814	Q2Q067	Q9H6R4	0.21385
51815	Q2Q067	Q9Y291	0.29299
51816	Q2Q067	Q8ND90	0.67563
51817	Q2Q067	P17036	0.29645
51818	Q2Q067	Q6P435	0.11005
51819	Q2Q067	P49005	0.53807
51820	Q2Q067	Q7Z614	0.36101
51821	Q2Q067	O15117	0.44503
51822	Q2Q067	P53801	0.3061
51823	Q2Q067	O94993	0.21921
51824	Q2Q067	Q13838	0.13097
51825	Q2Q067	F8W1W9	0.10647
51826	Q2Q067	Q9ULJ3	0.5849
51827	Q2Q067	Q13415	0.25023
51828	Q2Q067	O14776	1.1053
51829	Q2Q067	Q9ULR0	0.45607
51830	Q2Q067	Q86V48	0.463
51831	Q2Q067	Q8TBE0	0.766
51832	Q2Q067	Q6VVB1	0.34427

Q2Q067	D6RGH6	1.1954
Q2Q067	O95990	0.05789
Q2Q067	P86479	0.19215
Q2Q067	P17040	0.61696
Q2Q067	Q96P70	0.9557
Q2Q067	Q15560	1.0054
Q2Q067	Q9P258	0.57237
Q2Q067	P17676	0.85627
Q2Q067	Q2QGD7	0.63202
Q2Q067	Q9H019	0.24596
Q2Q067	Q16531	0.62333
Q2Q067	Q6U949	0.19215
Q2Q067	Q86V97	0.37541
Q2Q067	O95359	0.25184
Q2Q067	Q5T280	0.60492
Q2Q067	O95551	0.70051
Q2Q067	Q9H320	0.4701
Q2Q067	P17482	1.1273
Q2Q067	Q9P086	1.0006
Q2Q067	O00541	0.3554
Q2Q067	P17010	0.2503
Q2Q067	O95625	0.11121
Q2Q067	Q96MD2	0.096693
Q2Q067	P11308	0.53389
Q2Q067	Q92837	0.47558
Q2Q067	Q53H80	0.36625
Q2Q067	P17038	0.16589
Q2Q067	Q56P42	0.1321
Q2Q067	Q9Y3B2	0.81126
Q2Q067	Q8N5Y2	0.010509
Q2Q067	Q49B96	0.18994
Q2Q067	Q7L1Q6	0.30306
Q2Q067	Q07955	0.57369
Q2Q067	Q6PF06	0.40441
Q2Q067	P59091	0.080353
Q2Q067	Q08AD1	0.42372
Q2Q067	Q9P0J6	0.31693
Q2Q067	Q9BWJ5	0.22568
Q2Q067	Q9Y2B4	0.18741
Q2Q067	Q9Y4Z2	0.23396
Q2Q067	Q9NTG7	0.44604
Q2Q067	Q9UK12	0.10888
Q2Q067	Q6I9Y2	0.79296
Q2Q067	Q96LT7	0.32524
Q2Q067	Q8WU90	0.82814
Q2Q067	Q1MSJ5	0.58511
Q2Q067	Q9NPD3	0.33603
Q2Q067	Q96MH2	1.2105
Q2Q067	Q9H7T3	0.1762
Q2Q067	Q8WYN3	0.54101
Q2Q067	Q9UDW3	0.24989
Q2Q067	Q9NVK5	0.091575
Q2Q067	P38935	0.42274
Q2Q067	Q8NDY6	0.48324
Q2Q067	E9PGG2	0.25814
Q2Q067	Q9NYG5	0.77379
Q2Q067	Q86TB9	0.86845
Q2Q067	Q96CB8	0.47713
Q2Q067	Q5BJF2	0.084686
Q2Q067	Q9NYF8	1.202
Q2Q067	P08047	0.82146
Q2Q067	Q8NFH4	0.96831
Q2Q067	Q8NEU8	0.34017
Q2Q067	Q96DP5	0.20614
Q2Q067	Q8TC21	0.11317
Q2Q067	Q5VSR9	0.40408
Q2Q067	Q8N5T8	0.32005
Q2Q067	Q9BRD0	0.32021
Q2Q067	P11473	0.34642
Q2Q067	Q13156	0.15449
Q2Q067	Q7RTU5	0.37479
Q2Q067	Q00978	0.98772
Q2Q067	P15924	0.28022
Q2Q067	O00458	0.4403
Q2Q067	Q8NAV2	0.060335
Q2Q067	Q99471	0.87157

Q2Q067	Q63HM1	0.26003
Q2Q067	Q8NFI3	0.10239
Q2Q067	Q9BQN1	0.39979
Q2Q067	Q8TAB5	0.29573
Q2Q067	Q9UHJ3	0.3157
Q2Q067	Q7Z3Y7	0.26069
Q2Q067	Q8NF91	0.46851
Q2Q067	Q96C28	0.20379
Q2Q067	O75928	0.75671
Q2Q067	P30304	0.59861
Q2Q067	O15355	0.85795
Q2Q067	O14907	0.41824
Q2Q067	Q92905	0.6389
Q2Q067	Q7RTT6	0.25757
Q2Q067	Q9NPE3	0.69397
Q2Q067	Q9BZI7	0.71832
Q2Q067	Q3LI81	0.46381
Q2Q067	Q96N16	0.47595
Q2Q067	Q96RJ6	0.37545
Q2Q067	Q3LI73	0.33745
Q2Q067	O75182	1.1319
Q2Q067	Q13769	0.5654
Q2Q067	Q13794	0.39809
Q2Q067	Q8NDT2	0.84724
Q2Q067	P22557	0.14376
Q2Q067	P59090	0.19215
Q2Q067	P63241	0.50269
Q2Q067	Q13342	0.64253
Q2Q067	Q9NY87	0.38704
Q2Q067	Q16629	0.585
Q2Q067	A6NHU5	0.046436
Q2Q067	Q569G3	0.29573
Q2Q067	Q86V42	0.20882
Q2Q067	Q9H1A4	0.78777
Q2Q067	Q9ULJ6	0.47094
Q2Q067	Q15022	0.60427
Q2Q067	O43829	0.5896
Q2Q067	Q569K6	0.01004
Q2Q067	Q96EK5	0.18336
Q2Q067	P16415	0.14116
Q2Q067	P35573	0.50123
Q2Q067	Q5JVX7	0.36583
Q2Q067	Q9BSM1	1.0132
Q2Q067	P21953	0.032982
Q2Q067	K7EJ46	0.19215
Q2Q067	Q8NHM5	0.25829
Q2Q067	Q5VVH5	0.5795
Q2Q067	Q86Y25	0.11612
Q2Q067	Q9BQ15	0.62943
Q2Q067	Q9H9Y6	0.6483
Q2Q067	Q6P1L8	0.47888
Q2Q067	Q96KP1	0.23167
Q2Q067	Q9Y2D8	0.62948
Q2Q067	Q01892	0.37629
Q2Q067	Q9BV47	0.30694
Q2Q067	P0CG00	0.14982
Q2Q067	A8MV24	0.20432
Q2Q067	Q14790	0.2561
Q2Q067	Q4G0A6	0.61435
Q2Q067	Q969Q0	0.076547
Q2Q067	P60370	0.34715
Q2Q067	A6NP61	0.43237
Q2Q067	Q9Y262	0.74056
Q2Q067	O95848	0.39895
Q2Q067	Q8N5F7	1.304
Q2Q067	Q96BW1	0.1321
Q2Q067	Q96HB5	0.11635
Q2Q067	Q5VWI1	0.61845
Q2Q067	O95793	0.21619
Q2Q067	Q96JS3	0.3271
Q2Q067	Q8N3S3	0.30002
Q2Q067	P13196	0.24356
Q2Q067	Q495D7	0.0050941
Q2Q067	Q92973	1.1537
Q2Q067	A6NKF1	0.83843
Q2Q067	P30154	0.27198

Q2Q067	P12270	0.62677
Q2Q067	Q7Z6Z7	0.26428
Q2Q067	Q92558	0.32937
Q2Q067	Q6L8H4	0.19215
Q2Q067	Q6PIJ6	0.41713
Q2Q067	O75400	0.85533
Q2Q067	Q14749	0.58985
Q2Q067	P09017	0.13176
Q2Q067	Q8WXC6	0.05478
Q2Q067	Q8N184	0.12227
Q2Q067	Q9NZC7	0.39266
Q2Q067	Q96NJ6	0.10402
Q2Q067	Q8NAP1	0.29573
Q2Q067	O14829	0.23249
Q2Q067	Q96CS3	0.50666
Q2Q067	P51965	0.46506
Q2Q067	P22670	0.82305
Q2Q067	Q14240	0.12388
Q2Q067	O60563	1.5404
Q2Q067	Q9UNX3	0.58372
Q2Q067	Q6PEX3	0.173
Q2Q067	Q9ULE4	0.16834
Q2Q067	H3BR10	0.19215
Q2Q067	Q9UIC8	0.24264
Q2Q067	Q5BJF6	0.4207
Q2Q067	Q504Q3	0.19513
Q2Q067	Q9HB09	0.54165
Q2Q067	Q6ZPA2	0.19215
Q2Q067	Q8WUB8	0.33944
Q2Q067	Q9ULG3	0.19215
Q2Q067	O95416	0.39341
Q2Q067	Q5JQF7	0.19215
Q2Q067	Q9Y3I1	0.84401
Q2Q067	Q495Z4	0.15603
Q2Q067	Q9BRA0	0.1321
Q2Q067	Q6P1W5	0.28783
Q2Q067	A8MUL3	0.19215
Q2Q067	Q8TCI5	0.54999
Q2Q067	Q9BYQ3	0.34715
Q2Q067	Q9UHA3	0.23499
Q2Q067	Q8IY31	0.43461
Q2Q067	Q6P5X5	0.38205
Q2Q067	Q9BQC3	0.49955
Q2Q067	Q9GZR1	0.34325
Q2Q067	Q8WYQ5	0.63894
Q2Q067	Q5TYM5	0.067904
Q2Q067	P0DKL9	0.34521
Q2Q067	Q8TAD7	0.29573
Q2Q067	Q8WUN7	0.32989
Q2Q067	O15084	0.74487
Q2Q067	P13640	0.58416
Q2Q067	Q9NVI1	0.64465
Q2Q067	P05413	0.22369
Q2Q067	Q9BWD3	0.38894
Q2Q067	Q00597	0.81225
Q2Q067	O43542	0.51424
Q2Q067	Q13129	0.71922
Q2Q067	Q6BDS2	0.21707
Q2Q067	Q9H7S9	0.70338
Q2Q067	Q2M2Z5	0.60634
Q2Q067	Q8N726	0.6017
Q2Q067	O95394	0.42751
Q2Q067	Q86UE4	0.34996
Q2Q067	P11831	0.62072
Q2Q067	P60174	0.22071
Q2Q067	Q96FZ2	0.55312
Q2Q067	Q6ZUT6	0.010056
Q2Q067	O95198	0.36902
Q2Q067	P20265	0.67286
Q2Q067	O75531	0.95974
Q2Q067	Q5JTW2	0.29868
Q2Q067	Q7Z3V5	0.1529
Q2Q067	Q9NR22	0.29276
Q2Q067	P60673	0.13329
Q2Q067	P30050	0.78122
Q2Q067	Q8WUY3	0.13288

52061	Q2Q067	Q6ZRN7	0.19215
52062	Q2Q067	Q9H267	0.20356
52063	Q2Q067	P15172	0.34264
52064	Q2Q067	Q6IPT2	0.63352
52065	Q2Q067	Q06609	0.4263
52066	Q2Q067	P0C7T5	0.22651
52067	Q2Q067	P63010	0.4232
52068	Q2Q067	Q9H147	0.26255
52069	Q2Q067	Q3SXZ3	0.1161
52070	Q2Q067	Q86VP6	0.69972
52071	Q2Q067	Q9BV57	0.61675
52072	Q2Q067	A1A580	0.33745
52073	Q2Q067	O43248	0.30805
52074	Q2Q067	Q9Y2M2	0.18873
52075	Q2Q067	O94906	0.29907
52076	Q2Q067	Q6NT76	0.48135
52077	Q2Q067	P0CL83	0.26834
52078	Q2Q067	Q5TAX3	0.63196
52079	Q2Q067	Q156A1	0.19215
52080	Q2Q067	Q8NEQ6	0.092004
52081	Q2Q067	A6NHS1	0.24407
52082	Q2Q067	A6NEH8	0.059362
52083	Q2Q067	P57721	0.59986
52084	Q2Q067	Q9H9Q4	0.44685
52085	Q2Q067	Q14CW9	0.84061
52086	Q2Q067	Q96PC3	0.3056
52087	Q2Q067	P28702	0.3661
52088	Q2Q067	O43148	0.88239
52089	Q2Q067	Q9UFB7	0.10004
52090	Q2Q067	Q96I59	0.23394
52091	Q2Q067	Q6IQ21	0.14332
52092	Q2Q067	Q6ZS49	0.19215
52093	Q2Q067	Q00577	0.53588
52094	Q2Q067	A6NIY4	0.23617
52095	Q2Q067	O15049	0.45056
52096	Q2Q067	P48552	0.64919
52097	Q2Q067	P54105	0.87701
52098	Q2Q067	Q13398	0.23851
52099	Q2Q067	Q53GI3	0.095696
52100	Q2Q067	Q9ULW6	0.19253
52101	Q2Q067	Q96T17	0.2792
52102	Q2Q067	Q8IU81	0.1973
52103	Q2Q067	Q9Y3E5	0.53996
52104	Q2Q067	Q9BSD3	0.19165
52105	Q2Q067	Q3BBV1	0.72826
52106	Q2Q067	P12035	0.33029
52107	Q2Q067	Q96PT3	0.20973
52108	Q2Q067	Q96MR9	0.19951
52109	Q2Q067	P48378	0.30214
52110	Q2Q067	Q86TP1	0.74515
52111	Q2Q067	P80217	0.70076
52112	Q2Q067	Q9BZE9	0.45326
52113	Q2Q067	Q05DH4	0.51319
52114	Q2Q067	Q6PJT7	0.89072
52115	Q2Q067	P36871	0.16427
52116	Q2Q067	Q15561	0.89884
52117	Q2Q067	O96000	0.38297
52118	Q2Q067	Q8NEG0	0.11635
52119	Q2Q067	Q8NBZ0	1.2195
52120	Q2Q067	P31943	0.40418
52121	Q2Q067	Q14134	0.52019
52122	Q2Q067	Q96BV0	0.088474
52123	Q2Q067	O75446	0.82922
52124	Q2Q067	Q8IZL8	0.77769
52125	Q2Q067	Q5EBL4	0.37598
52126	Q2Q067	Q96LW7	0.35829
52127	Q2Q067	A8MTA8	0.041351
52128	Q2Q067	Q9UNK9	0.10793
52129	Q2Q067	Q96BF6	0.34443
52130	Q2Q067	Q9UBB4	0.47725
52131	Q2Q067	Q5H9M0	0.54367
52132	Q2Q067	O43776	0.27004
52133	Q2Q067	P11388	0.21876
52134	Q2Q067	Q9NP97	0.51119
52135	Q2Q067	Q9UJW3	0.62971
52136	Q2Q067	P28065	0.67176

Q2Q067	P35227	0.7238
Q2Q067	Q92979	0.3144
Q2Q067	P54725	0.60679
Q2Q067	Q6ZRV3	0.19215
Q2Q067	A8MT65	0.21334
Q2Q067	Q9P2B7	0.29573
Q2Q067	A6NK53	0.12921
Q2Q067	Q99873	0.58684
Q2Q067	Q12962	1.1094
Q2Q067	Q9H788	0.93512
Q2Q067	Q9Y295	0.083135
Q2Q067	Q9NZP6	0.53604
Q2Q067	Q14669	0.30105
Q2Q067	Q8WZA8	0.19215
Q2Q067	P37802	0.31983
Q2Q067	Q9Y5L0	0.93565
Q2Q067	Q8TF74	0.37525
Q2Q067	Q8N612	0.30099
Q2Q067	P11166	0.56565
Q2Q067	Q86TJ2	0.38905
Q2Q067	Q9H693	0.31641
Q2Q067	Q96GT9	0.34764
Q2Q067	Q9NX18	0.76163
Q2Q067	P28069	0.70744
Q2Q067	P32189	0.075902
Q2Q067	Q9BUY5	0.22248
Q2Q067	Q86UL8	0.25899
Q2Q067	Q96GU1	0.12303
Q2Q067	Q09028	0.59745
Q2Q067	O14818	0.67138
Q2Q067	O60663	0.5435
Q2Q067	Q15629	0.17994
Q2Q067	P61077	0.49702
Q2Q067	Q68EN5	0.022543
Q2Q067	P07902	0.28639
Q2Q067	P22674	0.32574
Q2Q067	O14972	0.12028
Q2Q067	Q5VY09	0.39148
Q2Q067	Q9UKW6	0.46358
Q2Q067	Q8IYA6	0.34279
Q2Q067	P14635	0.28317
Q2Q067	Q9P2T0	0.32695
Q2Q067	Q86X53	0.015065
Q2Q067	Q96QC0	0.69279
Q2Q067	Q8N9Y4	0.5618
Q2Q067	Q8TCH9	0.014743
Q2Q067	P0CE67	0.19215
Q2Q067	O43583	0.47232
Q2Q067	P08727	0.52331
Q2Q067	P58005	0.18393
Q2Q067	P49756	0.36378
Q2Q067	Q8IXM3	0.37574
Q2Q067	Q3C1V1	0.29573
Q2Q067	Q9BWN1	0.29573
Q2Q067	Q9BYD9	0.11065
Q2Q067	A6NGU7	0.029617
Q2Q067	Q9UPP5	0.26085
Q2Q067	O95049	0.43202
Q2Q067	Q15424	0.73604
Q2Q067	Q8N9P0	0.022434
Q2Q067	P78545	0.80701
Q2Q067	B2RXF5	0.26381
Q2Q067	Q9BQI9	0.49528
Q2Q067	Q7KZ85	0.43973
Q2Q067	P23528	0.7641
Q2Q067	P26196	0.52569
Q2Q067	O00170	0.67949
Q2Q067	Q8N3J9	0.092234
Q2Q067	Q8N6T7	0.60063
Q2Q067	Q9Y2P0	0.10893
Q2Q067	P60369	0.34715
Q2Q067	A6NCW3	0.48486
Q2Q067	Q9Y3D3	0.64964
Q2Q067	Q6ZRH3	0.11916
Q2Q067	P09629	0.95676
Q2Q067	Q92575	0.91112

52213	Q2Q067	O15069	0.26705
52214	Q2Q067	P63165	0.94553
52215	Q2Q067	Q9NS37	0.9139
52216	Q2Q067	Q9UHD9	0.76126
52217	Q2Q067	Q9BQG0	0.65151
52218	Q2Q067	O43156	0.28783
52219	Q2Q067	Q9Y2H8	0.10687
52220	Q2Q067	Q9NQ55	0.30746
52221	Q2Q067	O15304	1.0183
52222	Q2Q067	Q9BRP8	0.88626
52223	Q2Q067	Q5MNV8	0.21946
52224	Q2Q067	Q70YC4	0.38139
52225	Q2Q067	Q9UQR0	0.26021
52226	Q2Q067	Q8IV33	0.18863
52227	Q2Q067	Q96AQ7	0.54799
52228	Q2Q067	P52743	0.19186
52229	Q2Q067	Q9NYZ3	0.29936
52230	Q2Q067	Q99871	0.44212
52231	Q2Q067	Q5HYW2	0.37524
52232	Q2Q067	Q96QF0	0.55901
52233	Q2Q067	Q9Y2R5	0.23001
52234	Q2Q067	Q99627	0.35354
52235	Q2Q067	P58340	0.36825
52236	Q2Q067	Q96GN5	0.80438
52237	Q2Q067	Q9BUX1	0.76376
52238	Q2Q067	Q8WY50	0.19215
52239	Q2Q067	Q499Z4	0.12624
52240	Q2Q067	Q8N9A8	0.24698
52241	Q2Q067	Q10570	0.80815
52242	Q2Q067	Q04323	0.28937
52243	Q2Q067	P54845	0.32624
52244	Q2Q067	Q9Y248	0.69799
52245	Q2Q067	Q12996	0.1657
52246	Q2Q067	P18077	0.54068
52247	Q2Q067	O75886	1.1179
52248	Q2Q067	Q9NZQ3	0.68275
52249	Q2Q067	Q8IXH6	1.1569
52250	Q2Q067	Q9UND3	0.22989
52251	Q2Q067	Q8N8A2	0.28783
52252	Q2Q067	Q9UBS4	0.18138
52253	Q2Q067	P42568	0.88643
52254	Q2Q067	Q9NXE8	0.23818
52255	Q2Q067	Q15937	0.09883
52256	Q2Q067	Q9BV90	0.017889
52257	Q2Q067	Q9Y2A0	0.12175
52258	Q2Q067	Q9Y4A8	0.55723
52259	Q2Q067	Q96A04	0.47507
52260	Q2Q067	B3EWF7	0.19215
52261	Q2Q067	Q9BXG8	0.4808
52262	Q2Q067	Q9BTX9	0.46063
52263	Q2Q067	P62891	0.65612
52264	Q2Q067	Q9ULK4	1.2089
52265	Q2Q067	O95873	0.29573
52266	Q2Q067	Q68DK2	0.74516
52267	Q2Q067	Q00444	0.30046
52268	Q2Q067	A6NN06	0.19215
52269	Q2Q067	Q96AQ9	0.18349
52270	Q2Q067	Q8TEP8	0.79058
52271	Q2Q067	Q86XI2	0.44202
52272	Q2Q067	Q6ZVD7	0.24679
52273	Q2Q067	Q9H6Y2	0.35417
52274	Q2Q067	Q7Z2W4	0.40411
52275	Q2Q067	P27695	0.49683
52276	Q2Q067	Q15723	1.2601
52277	Q2Q067	Q7Z3E2	0.22303
52278	Q2Q067	Q9UBC3	0.23372
52279	Q2Q067	Q5T871	0.19215
52280	Q2Q067	P62314	0.88999
52281	Q2Q067	Q86Y26	0.34427
52282	Q2Q067	P08621	0.3612
52283	Q2Q067	Q8IXQ5	0.24902
52284	Q2Q067	Q15744	0.34751
52285	Q2Q067	A4D1U4	0.09293
52286	Q2Q067	Q9GZR2	0.15787
52287	Q2Q067	Q8N7G0	0.32768
52288	Q2Q067	Q9H3K6	0.25156

Q2Q067	Q8IX03	1.0564
Q2Q067	Q08050	0.43526
Q2Q067	Q9UHK0	0.30672
Q2Q067	Q8IXP5	0.29573
Q2Q067	P31948	0.92039
Q2Q067	Q13541	0.83371
Q2Q067	Q5VZV4	0.17662
Q2Q067	Q68EA5	0.2352
Q2Q067	Q5TID7	0.29924
Q2Q067	Q9Y2K5	0.090139
Q2Q067	O75323	0.24511
Q2Q067	Q5T1B1	0.57378
Q2Q067	O43399	0.54549
Q2Q067	P11142	0.43896
Q2Q067	Q9NZF1	0.041328
Q2Q067	P0CI01	0.23617
Q2Q067	Q96DB5	0.23408
Q2Q067	O75381	0.15633
Q2Q067	O75607	0.66024
Q2Q067	Q9NQS7	0.84216
Q2Q067	Q14203	0.93183
Q2Q067	O15405	1.0076
Q2Q067	P61978	1.0113
Q2Q067	A6NDB9	0.17589
Q2Q067	O43823	0.18694
Q2Q067	Q99569	0.25429
Q2Q067	Q16630	0.21378
Q2Q067	Q5T0J3	0.02475
Q2Q067	Q8N5C8	0.50869
Q2Q067	Q8WUB2	0.15683
Q2Q067	Q96B67	0.24659
Q2Q067	Q9BQ48	0.41104
Q2Q067	Q9Y5B6	0.49189
Q2Q067	Q9BW60	0.27937
Q2Q067	O95163	0.64551
Q2Q067	P62324	0.69074
Q2Q067	A6NEQ9	0.19215
Q2Q067	Q9C075	0.12751
Q2Q067	Q9UKL0	1.2555
Q2Q067	Q8IZT9	0.46789
Q2Q067	O60711	0.38633
Q2Q067	Q9BQ67	0.32989
Q2Q067	Q14765	0.3453
Q2Q067	Q9HAU5	0.91985
Q2Q067	Q9Y5Z7	0.37558
Q2Q067	Q3LI70	0.19215
Q2Q067	Q9HBL0	0.32175
Q2Q067	Q1ED39	0.54789
Q2Q067	O95521	0.23465
Q2Q067	P22528	0.18454
Q2Q067	Q8IVV7	0.16238
Q2Q067	A8K830	0.19215
Q2Q067	K7EIQ3	0.59911
Q2Q067	O95178	0.20969
Q2Q067	Q06830	0.2735
Q2Q067	Q8N6K4	0.19215
Q2Q067	Q5TEZ4	0.19215
Q2Q067	Q9H3L0	0.32481
Q2Q067	Q13287	0.49256
Q2Q067	Q96IF1	0.35608
Q2Q067	P29084	1.2414
Q2Q067	Q13112	1.4257
Q2Q067	Q96CW1	0.5269
Q2Q067	Q9UJX2	0.65145
Q2Q067	Q8WVY7	0.20797
Q2Q067	Q68DY1	0.24839
Q2Q067	Q5TB30	0.2064
Q2Q067	Q8IXW5	0.52959
Q2Q067	Q7RTV3	0.31762
Q2Q067	Q12972	0.78554
Q2Q067	Q5JR98	0.28783
Q2Q067	Q14137	0.81691
Q2Q067	Q6ICB4	0.1425
Q2Q067	P27797	0.5449
Q2Q067	P12004	0.20035
Q2Q067	Q9UQL6	0.4749

Q2Q067	Q9H7C4	0.37499
Q2Q067	Q8N5P1	0.23388
Q2Q067	Q07021	0.82388
Q2Q067	Q6ZSR6	0.13792
Q2Q067	Q8TAK5	0.48352
Q2Q067	Q00059	0.89131
Q2Q067	P0CW26	0.1398
Q2Q067	Q9Y3M2	0.2699
Q2Q067	Q8ND83	0.52119
Q2Q067	O43524	0.37516
Q2Q067	Q14181	0.25532
Q2Q067	Q8TDC0	0.42718
Q2Q067	Q16851	0.36745
Q2Q067	P53999	1.2704
Q2Q067	Q8N336	0.15109
Q2Q067	Q8TBR4	0.15526
Q2Q067	Q96G23	0.249
Q2Q067	Q99549	0.5967
Q2Q067	Q9BU89	0.16533
Q2Q067	Q9HCG1	0.17676
Q2Q067	P61923	0.33189
Q2Q067	Q12959	0.89825
Q2Q067	O43502	0.24716
Q2Q067	P63146	0.18529
Q2Q067	O43593	0.18506
Q2Q067	Q9Y297	0.26871
Q2Q067	P0C2W7	0.19215
Q2Q067	Q96G25	0.97578
Q2Q067	Q9ULW3	0.81105
Q2Q067	A6XGL0	0.18759
Q2Q067	P06746	0.44745
Q2Q067	O43745	0.70022
Q2Q067	Q9BZE7	0.29573
Q2Q067	P61927	0.67882
Q2Q067	Q6P280	0.25546
Q2Q067	Q9NYF0	0.22391
Q2Q067	Q9NRG4	0.70392
Q2Q067	Q5RKV6	0.16261
Q2Q067	O75884	0.62748
Q2Q067	O95678	0.12106
Q2Q067	Q9Y399	0.49845
Q2Q067	Q8WXC3	0.65637
Q2Q067	Q5PRF9	0.57881
Q2Q067	P60520	0.21691
Q2Q067	Q9BUI4	0.17968
Q2Q067	Q76KD6	0.22122
Q2Q067	Q9BY77	0.32468
Q2Q067	Q99966	0.25065
Q2Q067	Q9ULE6	0.66387
Q2Q067	Q9NS25	0.4381
Q2Q067	O15162	0.084687
Q2Q067	O95721	0.29435
Q2Q067	Q9UDY4	0.79848
Q2Q067	O95159	0.29654
Q2Q067	Q8TAU3	0.12519
Q2Q067	P39023	1.0298
Q2Q067	Q15173	0.32421
Q2Q067	O15061	0.6837
Q2Q067	Q9Y448	0.7815
Q2Q067	Q9UQB3	0.51091
Q2Q067	Q92994	0.30729
Q2Q067	Q9NX05	0.23892
Q2Q067	Q5QP74	0.19215
Q2Q067	Q8N5J4	0.52127
Q2Q067	Q14691	0.21101
Q2Q067	Q9UL58	0.34566
Q2Q067	P24522	0.5346
Q2Q067	P38159	0.60383
Q2Q067	Q13469	1.1637
Q2Q067	Q9H1A3	0.28783
Q2Q067	Q8N0Z2	0.26485
Q2Q067	Q9UMX2	0.19131
Q2Q067	P0CG35	0.34514
Q2Q067	Q5U623	0.75208
Q2Q067	P52747	0.7753
Q2Q067	Q15004	0.82494

Q2Q067	Q13137	1.0462
Q2Q067	O43264	0.56817
Q2Q067	P04264	0.22852
Q2Q067	Q9Y3A5	0.63233
Q2Q067	Q9Y5P8	0.63371
Q2Q067	Q3LI60	0.19215
Q2Q067	Q9UHV2	0.97663
Q2Q067	Q9H4B0	0.26213
Q2Q067	P78412	0.25814
Q2Q067	Q4KN68	0.0029586
Q2Q067	P0CW23	0.18954
Q2Q067	O60287	0.57829
Q2Q067	Q14CS0	0.3846
Q2Q067	Q96SR6	0.10324
Q2Q067	P0C860	0.32398
Q2Q067	Q1ZZU3	0.65109
Q2Q067	Q15003	0.70011
Q2Q067	P55347	0.47374
Q2Q067	Q9NZH4	0.36819
Q2Q067	O94811	0.36864
Q2Q067	P62487	0.20584
Q2Q067	Q86X60	0.11744
Q2Q067	O75396	0.18423
Q2Q067	P60896	0.19215
Q2Q067	O15127	0.61071
Q2Q067	Q9UBF1	0.77285
Q2Q067	Q8N1A0	0.13334
Q2Q067	Q9UHI6	0.36011
Q2Q067	K7EIZ4	0.19215
Q2Q067	Q9UKC9	0.48063
Q2Q067	A6NLW8	0.27517
Q2Q067	Q9UK73	0.71298
Q2Q067	A6NGH7	0.1265
Q2Q067	Q5THR3	0.31018
Q2Q067	Q8N812	0.20739
Q2Q067	P78317	0.61126
Q2Q067	P11532	0.488
Q2Q067	Q14929	0.095275
Q2Q067	Q99504	0.4532
Q2Q067	Q5T619	0.092671
Q2Q067	P62081	1.0552
Q2Q067	Q9Y2Q9	0.34109
Q2Q067	Q6ZSN1	0.15872
Q2Q067	P61247	1.1759
Q2Q067	Q9GZR5	0.11923
Q2Q067	Q9BSV6	0.14822
Q2Q067	Q96CJ1	1.2231
Q2Q067	P23769	0.39645
Q2Q067	Q8NEY8	0.43815
Q2Q067	Q8WUM4	1.0558
Q2Q067	P62304	0.76738
Q2Q067	P29372	0.42387
Q2Q067	Q8NFW9	0.2636
Q2Q067	Q29RF7	0.93936
Q2Q067	P54253	0.94427
Q2Q067	P62714	0.72383
Q2Q067	Q96L92	0.72097
Q2Q067	Q14593	0.24672
Q2Q067	Q9UBS3	0.59006
Q2Q067	Q6RVD6	0.19215
Q2Q067	Q15929	0.19354
Q2Q067	O94992	1.1074
Q2Q067	O14980	1.0988
Q2Q067	Q3LI76	0.33428
Q2Q067	Q8N655	0.19215
Q2Q067	P35548	0.66903
Q2Q067	Q86T75	0.3158
Q2Q067	Q5VWM6	0.50615
Q2Q067	P55199	1.0226
Q2Q067	O75391	0.39794
Q2Q067	Q9UHB9	0.33983
Q2Q067	Q4G112	0.32768
Q2Q067	Q9NZ38	0.0034562
Q2Q067	Q9UIV1	1.2119
Q2Q067	Q8TB69	0.22332
Q2Q067	Q8NCA5	0.30415

Q2Q067	Q86Y46	0.16378
Q2Q067	Q13428	0.74222
Q2Q067	Q8TB03	0.23419
Q2Q067	Q9BVI4	0.26767
Q2Q067	P84103	0.57338
Q2Q067	P61254	0.55702
Q2Q067	A6PW82	0.28103
Q2Q067	Q9UI66	0.19215
Q2Q067	O60645	0.36981
Q2Q067	Q8N8I6	0.19215
Q2Q067	Q99743	0.44653
Q2Q067	P86481	0.19215
Q2Q067	O75817	0.83239
Q2Q067	Q9UI10	0.35277
Q2Q067	P20719	0.34943
Q2Q067	Q5T6S3	0.26767
Q2Q067	Q15072	0.14006
Q2Q067	Q07157	0.65562
Q2Q067	Q6X4W1	0.56337
Q2Q067	Q15935	0.29124
Q2Q067	A6NEM1	0.35087
Q2Q067	Q9Y4C2	0.38834
Q2Q067	Q7Z7K6	0.25055
Q2Q067	P59047	0.26213
Q2Q067	Q9H8S9	0.45294
Q2Q067	Q9H4K1	0.13168
Q2Q067	P17987	0.1401
Q2Q067	Q8NB91	0.17168
Q2Q067	P15036	0.83597
Q2Q067	B9ZVM9	0.65755
Q2Q067	Q15388	0.088916
Q2Q067	Q8NFV5	0.23617
Q2Q067	Q8IZS5	0.16407
Q2Q067	Q9BYR6	0.46381
Q2Q067	Q5VZQ5	0.42632
Q2Q067	Q96C00	0.26461
Q2Q067	P62249	0.4969
Q2Q067	Q8N1G2	0.25458
Q2Q067	Q9BYK8	0.36866
Q2Q067	Q8IYB3	0.36892
Q2Q067	Q5H9R7	0.76926
Q2Q067	P30307	0.7347
Q2Q067	Q9UEU5	0.2716
Q2Q067	Q9GZZ1	0.35731
Q2Q067	Q2TAL8	0.55978
Q2Q067	Q9H2C0	0.53534
Q2Q067	Q8IVP9	0.12984
Q2Q067	Q6W2J9	0.39006
Q2Q067	Q05048	0.75247
Q2Q067	P20073	0.41715
Q2Q067	Q5VT03	0.4993
Q2Q067	P51826	0.35697
Q2Q067	Q14186	1.2564
Q2Q067	Q63HK3	0.32906
Q2Q067	Q9UII2	0.18937
Q2Q067	Q92674	0.3827
Q2Q067	Q8N8X9	0.094384
Q2Q067	P49901	0.19215
Q2Q067	A8MZF0	0.29573
Q2Q067	Q99608	0.15494
Q2Q067	Q5TA31	0.4504
Q2Q067	Q6ZW54	0.5886
Q2Q067	P78352	0.5949
Q2Q067	P40426	0.31986
Q2Q067	Q8TD35	0.29573
Q2Q067	O75717	0.3687
Q2Q067	Q59GR2	0.25814
Q2Q067	Q3LI62	0.19215
Q2Q067	Q6XCG6	0.19215
Q2Q067	Q15699	0.47902
Q2Q067	Q86Y13	0.24905
Q2Q067	P53677	0.16059
Q2Q067	Q9UII5	0.15472
Q2Q067	Q9C0D2	0.32508
Q2Q067	Q12800	0.33871
Q2Q067	A8MZ59	0.25814

Q2Q067	Q15532	0.9955
Q2Q067	Q96NS8	0.011195
Q2Q067	Q9UJM3	0.37745
Q2Q067	P59051	0.014148
Q2Q067	Q9GZY1	0.40863
Q2Q067	Q5HYI7	0.52812
Q2Q067	O60828	0.78818
Q2Q067	Q8NE65	0.21673
Q2Q067	P04732	0.17228
Q2Q067	O75582	0.24816
Q2Q067	O14508	0.28692
Q2Q067	A2RU30	0.2567
Q2Q067	Q05084	0.35642
Q2Q067	P26678	0.20139
Q2Q067	A8MUV8	0.23774
Q2Q067	O94913	0.31311
Q2Q067	O15131	1.0961
Q2Q067	Q96KR1	0.21109
Q2Q067	Q9P243	0.30054
Q2Q067	Q9ULM2	0.12578
Q2Q067	Q15047	0.60094
Q2Q067	Q7Z2V1	0.19215
Q2Q067	Q9HB71	0.53074
Q2Q067	Q6NT89	0.46329
Q2Q067	Q8WW18	0.29573
Q2Q067	Q14938	0.2275
Q2Q067	O75529	0.62313
Q2Q067	Q5T035	0.23031
Q2Q067	Q9UM13	0.68627
Q2Q067	Q9NP64	0.20997
Q2Q067	Q9BQ70	1.3375
Q2Q067	Q9UG56	0.082841
Q2Q067	P62280	0.58547
Q2Q067	O43310	0.46475
Q2Q067	Q96N38	0.23896
Q2Q067	Q14997	0.15484
Q2Q067	A8MWA4	0.28061
Q2Q067	P49356	0.055029
Q2Q067	P62328	0.57525
Q2Q067	P83731	0.78122
Q2Q067	Q9NX55	0.126
Q2Q067	Q569H4	0.29573
Q2Q067	O00471	0.20737
Q2Q067	Q9UBF2	0.19276
Q2Q067	Q9BYV8	0.59644
Q2Q067	A1L4L8	0.23043
Q2Q067	Q5VTQ0	0.03809
Q2Q067	Q9UGP4	0.40894
Q2Q067	Q86WX3	1.0915
Q2Q067	Q96A22	0.13147
Q2Q067	P40337	0.88045
Q2Q067	Q5SVQ8	0.13712
Q2Q067	Q99750	0.37113
Q2Q067	Q9BW27	0.79856
Q2Q067	Q6NXP2	0.58076
Q2Q067	Q9BTC8	0.65355
Q2Q067	Q9Y6P5	0.15879
Q2Q067	Q8NA03	0.17761
Q2Q067	O43924	0.049969
Q2Q067	O43768	0.0786
Q2Q067	O15235	0.69895
Q2Q067	Q5H9F3	0.5573
Q2Q067	Q9NWV4	0.32203
Q2Q067	A8MQ14	0.12001
Q2Q067	Q14498	0.71097
Q2Q067	Q96M53	0.6806
Q2Q067	P56693	0.61852
Q2Q067	Q9NYJ1	0.54683
Q2Q067	Q92748	0.80162
Q2Q067	Q9Y6H5	0.74221
Q2Q067	Q9BZW7	0.24135
Q2Q067	P05787	1.4311
Q2Q067	Q96LB3	0.57283
Q2Q067	A4GXA9	0.52312
Q2Q067	Q502W7	0.22955
Q2Q067	O14787	0.9249

52669	Q2Q067	O43236	0.26293
52670	Q2Q067	Q96BT3	0.52964
52671	Q2Q067	Q9H9C1	0.47086
52672	Q2Q067	Q8NDA8	0.28583
52673	Q2Q067	Q08J23	0.58512
52674	Q2Q067	Q8N7R0	0.32768
52675	Q2Q067	A6NHP3	0.23617
52676	Q2Q067	Q96CX3	0.1094
52677	Q2Q067	Q9BTP7	0.98908
52678	Q2Q067	Q8IWS0	0.4602
52679	Q2Q067	Q8NHQ1	1.3488
52680	Q2Q067	Q969S8	0.78192
52681	Q2Q067	A6NH11	0.23677
52682	Q2Q067	O14867	0.44301
52683	Q2Q067	P25791	0.44052
52684	Q2Q067	Q9BSU3	0.42308
52685	Q2Q067	Q96F24	0.64765
52686	Q2Q067	Q5VT33	0.1131
52687	Q2Q067	Q9Y6Y0	0.46721
52688	Q2Q067	P17032	0.098292
52689	Q2Q067	Q6ZRP0	0.29573
52690	Q2Q067	Q86V81	0.84896
52691	Q2Q067	P11171	0.46556
52692	Q2Q067	P48788	0.55237
52693	Q2Q067	Q99728	0.25007
52694	Q2Q067	Q9NXB0	0.37844
52695	Q2Q067	Q8IWZ8	0.21707
52696	Q2Q067	Q9BV19	0.15266
52697	Q2Q067	Q9H8E8	0.69857
52698	Q2Q067	Q6PGN9	0.76172
52699	Q2Q067	Q2M3C7	0.44855
52700	Q2Q067	Q86TA1	0.24537
52701	Q2Q067	P61576	0.36503
52702	Q2Q067	P19532	0.74798
52703	Q2Q067	P52758	0.43552
52704	Q2Q067	Q96BD5	0.45567
52705	Q2Q067	Q53GS7	0.44998
52706	Q2Q067	Q6P9G9	0.068077
52707	Q2Q067	Q6AI08	0.21869
52708	Q2Q067	P26358	0.36604
52709	Q2Q067	Q9ULX9	0.45688
52710	Q2Q067	Q04695	0.36996
52711	Q2Q067	P51948	0.45496
52712	Q2Q067	Q9NSG2	0.20414
52713	Q2Q067	Q13561	0.75444
52714	Q2Q067	Q92546	0.48301
52715	Q2Q067	Q9Y2T7	0.16801
52716	Q2Q067	Q52M93	0.050676
52717	Q2Q067	Q9H2W6	0.18515
52718	Q2Q067	Q96G03	0.27675
52719	Q2Q067	P31350	0.36998
52720	Q2Q067	Q66GS9	0.33645
52721	Q2Q067	Q96F07	0.42663
52722	Q2Q067	O75618	1.1491
52723	Q2Q067	Q9Y221	0.78026
52724	Q2Q067	Q9UBW8	0.35354
52725	Q2Q067	P35527	0.20138
52726	Q2Q067	P15621	0.22556
52727	Q2Q067	Q8N0T1	0.21773
52728	Q2Q067	A8MUA0	0.10188
52729	Q2Q067	Q6GPH4	0.37129
52730	Q2Q067	Q92831	0.86249
52731	Q2Q067	Q96EH3	0.071065
52732	Q2Q067	P60900	0.4445
52733	Q2Q067	Q92782	0.54353
52734	Q2Q067	Q8TEM1	0.15609
52735	Q2Q067	P46976	0.43257
52736	Q2Q067	Q14585	0.096646
52737	Q2Q067	Q8TBZ9	0.59043
52738	Q2Q067	Q14106	0.18125
52739	Q2Q067	Q6IN85	0.16498
52740	Q2Q067	O43711	0.38188
52741	Q2Q067	Q9NQX6	0.10835
52742	Q2Q067	Q9HAH7	0.25984
52743	Q2Q067	Q96MT3	0.87839
52744	Q2Q067	Q86UY5	0.0086481

Q2Q067	P17022	0.058453
Q2Q067	Q9H5J8	0.97805
Q2Q067	Q9BPZ7	0.28461
Q2Q067	Q15050	0.36233
Q2Q067	Q9H8T0	0.38239
Q2Q067	D6RB28	0.35087
Q2Q067	Q96BW9	0.18187
Q2Q067	Q9UL46	0.30616
Q2Q067	Q16143	0.30318
Q2Q067	Q07666	0.41267
Q2Q067	Q9BZD4	0.74384
Q2Q067	Q99576	0.46068
Q2Q067	Q14653	0.67565
Q2Q067	Q68DI1	0.12293
Q2Q067	P51504	0.12014
Q2Q067	Q9UBC9	0.44764
Q2Q067	Q9GZY8	0.13705
Q2Q067	P67870	0.25144
Q2Q067	Q9NQ89	0.16984
Q2Q067	Q6PK04	0.563
Q2Q067	Q9NX53	0.28783
Q2Q067	Q9Y6N9	0.29806
Q2Q067	Q8TEX9	0.38871
Q2Q067	O60934	0.18361
Q2Q067	P04733	0.17228
Q2Q067	Q9UPX8	0.27864
Q2Q067	Q9UKY1	0.35561
Q2Q067	Q9HD64	0.086969
Q2Q067	Q8WWW0	0.26515
Q2Q067	Q9H9H5	0.26302
Q2Q067	P34896	0.072845
Q2Q067	Q96T37	0.29022
Q2Q067	P61289	0.86538
Q2Q067	Q96LY2	0.32771
Q2Q067	Q9H9Y2	0.17106
Q2Q067	O75505	0.25814
Q2Q067	Q96A73	0.5634
Q2Q067	Q8N8C0	0.12775
Q2Q067	Q13495	0.64204
Q2Q067	Q5T4H9	0.19215
Q2Q067	Q9Y2D9	0.58991
Q2Q067	Q96DE5	0.53951
Q2Q067	Q96SZ4	0.13104
Q2Q067	Q15654	0.77947
Q2Q067	Q5T0L3	0.61368
Q2Q067	Q96DB2	0.21383
Q2Q067	Q96BY7	0.067745
Q2Q067	Q96KW2	0.3869
Q2Q067	Q5BN46	0.015082
Q2Q067	Q9HD34	0.276
Q2Q067	P56270	0.53182
Q2Q067	Q96DF8	0.57902
Q2Q067	Q6IQ32	0.43053
Q2Q067	Q659A1	0.11284
Q2Q067	A2RRD8	0.13766
Q2Q067	Q15306	0.84729
Q2Q067	Q14746	0.29922
Q2Q067	B4DXR9	0.24252
Q2Q067	Q9UKD1	0.75185
Q2Q067	Q8N6G1	0.19215
Q2Q067	Q6NX45	0.10964
Q2Q067	P62263	0.54942
Q2Q067	P51816	0.28754
Q2Q067	Q9BXY8	0.3233
Q2Q067	O43482	0.43553
Q2Q067	Q86US8	0.55593
Q2Q067	O96017	0.61168
Q2Q067	P51959	0.44026
Q2Q067	Q15370	1.1359
Q2Q067	P17098	0.24084
Q2Q067	Q9BU61	0.67207
Q2Q067	O60832	0.66642
Q2Q067	O75437	0.21652
Q2Q067	Q8NF99	0.24359
Q2Q067	P12755	0.16421
Q2Q067	Q9UNN5	0.66039

Q2Q067	Q9BSH5	0.34486
Q2Q067	Q9Y4X5	0.37901
Q2Q067	O14948	0.43476
Q2Q067	Q14687	0.28783
Q2Q067	Q9P1G2	0.16666
Q2Q067	Q5MJ68	0.18657
Q2Q067	P61572	0.36503
Q2Q067	Q13360	0.13061
Q2Q067	A8MUU9	0.041221
Q2Q067	O43303	0.83056
Q2Q067	Q8N1I0	0.22781
Q2Q067	Q6Q795	0.19215
Q2Q067	Q9UBD5	0.55511
Q2Q067	Q9UBF6	0.67217
Q2Q067	Q9BRT6	0.26064
Q2Q067	Q8IXQ4	0.29573
Q2Q067	O43678	0.29601
Q2Q067	Q92624	0.83087
Q2Q067	Q9UPN7	0.93763
Q2Q067	Q96C90	0.33265
Q2Q067	Q9NQ32	0.10598
Q2Q067	Q9BTE7	0.24809
Q2Q067	Q8N1E6	0.30239
Q2Q067	P60410	0.46381
Q2Q067	Q8TES7	0.55549
Q2Q067	Q86UB2	0.34167
Q2Q067	Q8N8H1	0.21059
Q2Q067	Q9HCC9	0.38464
Q2Q067	Q14978	0.44204
Q2Q067	Q00610	0.89233
Q2Q067	Q96S59	0.93087
Q2Q067	Q9H1I8	0.17504
Q2Q067	P84022	0.57434
Q2Q067	Q8WXW3	0.35292
Q2Q067	Q9HB19	0.48472
Q2Q067	Q8NDC0	0.25997
Q2Q067	P61571	0.36503
Q2Q067	P61966	0.2696
Q2Q067	Q5MJ70	0.23617
Q2Q067	Q92796	0.29068
Q2Q067	Q9NZ63	0.32989
Q2Q067	Q9UKZ1	0.34234
Q2Q067	Q9NZL4	0.2662
Q2Q067	O76095	0.056502
Q2Q067	Q96RT7	0.30342
Q2Q067	P60014	0.41862
Q2Q067	Q969G3	1.0426
Q2Q067	Q6EEV4	0.19215
Q2Q067	Q8WYR1	0.21949
Q2Q067	Q16222	0.18225
Q2Q067	Q8NBA8	0.050719
Q2Q067	P60866	0.68165
Q2Q067	Q8TAT8	0.042768
Q2Q067	O14641	0.70016
Q2Q067	P78414	0.25814
Q2Q067	P43358	0.37541
Q2Q067	P62906	0.69861
Q2Q067	A6NKN8	0.47586
Q2Q067	Q63ZY3	0.87257
Q2Q067	Q86SX3	0.77799
Q2Q067	Q6ZTR5	0.007447
Q2Q067	Q9NXV2	1.3531
Q2Q067	Q99856	0.69763
Q2Q067	A8MVJ9	0.31076
Q2Q067	Q6P9A3	0.089995
Q2Q067	I3L0S3	0.18858
Q2Q067	Q9NSP4	0.065359
Q2Q067	Q5SV97	0.36776
Q2Q067	Q8N9T2	0.19215
Q2Q067	P55212	0.45752
Q2Q067	P31949	0.32321
Q2Q067	Q69YI7	0.77707
Q2Q067	P82650	0.15784
Q2Q067	Q6P9F0	0.19934
Q2Q067	P50221	0.58373
Q2Q067	B1APH4	0.16825

Q2Q067	Q6P1X5	0.88281
Q2Q067	P46939	0.5748
Q2Q067	P68871	0.19188
Q2Q067	Q68DN1	0.20111
Q2Q067	Q9C0D0	0.19632
Q2Q067	Q5XKR9	0.41636
Q2Q067	Q9BQC6	0.42309
Q2Q067	Q9UL33	0.14849
Q2Q067	Q9BQ83	0.55537
Q2Q067	P10914	0.43506
Q2Q067	Q8N5M1	0.45243
Q2Q067	P41252	0.6402
Q2Q067	Q92539	0.5881
Q2Q067	Q8WTP9	0.56155
Q2Q067	H3BTG2	0.35676
Q2Q067	P15927	0.44711
Q2Q067	Q9ULM3	0.79657
Q2Q067	Q9NV39	0.19817
Q2Q067	A9QM74	0.55585
Q2Q067	Q86T82	0.28022
Q2Q067	P52815	0.59141
Q2Q067	A8MZG2	0.29573
Q2Q067	Q06787	0.85959
Q2Q067	Q8N448	0.29429
Q2Q067	P41227	0.32975
Q2Q067	Q8N140	0.85807
Q2Q067	Q66K41	0.50607
Q2Q067	Q9NZH5	0.2278
Q2Q067	Q96JM7	0.36067
Q2Q067	Q99622	0.056995
Q2Q067	Q15102	0.18314
Q2Q067	P56277	0.37076
Q2Q067	A6NDZ8	0.083005
Q2Q067	Q14533	0.76812
Q2Q067	P25063	0.19215
Q2Q067	Q9UI72	0.19215
Q2Q067	P0C7X5	0.1331
Q2Q067	Q8IYH5	0.42909
Q2Q067	Q13330	0.46645
Q2Q067	Q6IQ49	0.11794
Q2Q067	Q8NC74	0.098973
Q2Q067	Q8TEE9	0.59276
Q2Q067	Q5VIR6	0.45548
Q2Q067	Q15024	0.70039
Q2Q067	Q8N3C0	0.23328
Q2Q067	Q9NPD8	0.16359
Q2Q067	O14933	0.24582
Q2Q067	Q5T1N1	0.60003
Q2Q067	Q7Z3B3	0.099314
Q2Q067	Q8NEH6	0.51039
Q2Q067	P09972	0.60856
Q2Q067	O00716	1.0864
Q2Q067	Q15437	0.23106
Q2Q067	P0CJ89	0.27517
Q2Q067	Q96IZ7	0.74909
Q2Q067	Q7Z2X7	0.27875
Q2Q067	O60284	0.16141
Q2Q067	O14862	0.54443
Q2Q067	Q5T5A4	0.14848
Q2Q067	Q13310	0.37021
Q2Q067	Q86WZ0	0.36666
Q2Q067	Q12834	0.773
Q2Q067	P55060	0.84838
Q2Q067	Q8IUC0	0.17889
Q2Q067	A5D8V6	0.21598
Q2Q067	Q15643	0.89117
Q2Q067	O75177	0.46797
Q2Q067	Q8NDZ9	0.19215
Q2Q067	O75528	0.72373
Q2Q067	Q9H379	0.29403
Q2Q067	Q6ZS46	0.41154
Q2Q067	Q96HJ3	0.29573
Q2Q067	Q6ZU65	0.15082
Q2Q067	Q8WUU5	0.27248
Q2Q067	P16383	0.58843
Q2Q067	P54840	0.16949

Q2Q067	O60220	0.24913
Q2Q067	Q9NQ31	0.60393
Q2Q067	Q96BQ5	0.35134
Q2Q067	P56279	0.4108
Q2Q067	P41002	0.90366
Q2Q067	P53992	1.0081
Q2Q067	Q9Y6K9	0.97261
Q2Q067	O95628	0.62707
Q2Q067	P36873	0.71627
Q2Q067	P0CJ90	0.27517
Q2Q067	Q96GC6	1.1176
Q2Q067	Q96RY5	0.52852
Q2Q067	P51668	0.56922
Q2Q067	Q8NET4	0.36766
Q2Q067	P57081	0.88359
Q2Q067	P69208	0.19215
Q2Q067	Q92925	0.54643
Q2Q067	Q76KX8	0.24459
Q2Q067	Q96FQ6	0.24695
Q2Q067	Q9NVE4	0.15811
Q2Q067	Q8WW14	0.10032
Q2Q067	Q9H0W8	0.33044
Q2Q067	Q00056	0.31739
Q2Q067	Q5T9G4	0.53071
Q2Q067	A6NHJ4	0.25938
Q2Q067	Q2TAK8	0.61715
Q2Q067	Q9NUW8	0.63095
Q2Q067	Q8NCU7	0.51068
Q2Q067	Q14232	0.11373
Q2Q067	Q9Y5J6	0.1495
Q2Q067	Q92843	0.44311
Q2Q067	Q15021	0.79968
Q2Q067	Q9P2R6	1.0528
Q2Q067	Q01658	0.46171
Q2Q067	Q8NEF3	0.10748
Q2Q067	Q6NSW7	0.32768
Q2Q067	P28347	1.0068
Q2Q067	Q15468	0.12309
Q2Q067	Q96ES7	0.12155
Q2Q067	Q8N3F8	0.36106
Q2Q067	Q96MW1	0.29573
Q2Q067	Q8N895	0.46528
Q2Q067	Q96N22	0.24759
Q2Q067	Q92541	0.98668
Q2Q067	A8MXY4	0.16589
Q2Q067	Q8N141	0.24938
Q2Q067	P0CG34	0.34514
Q2Q067	Q9NQP4	0.93882
Q2Q067	Q96G28	0.10619
Q2Q067	P31276	0.58233
Q2Q067	P82914	0.34576
Q2Q067	Q96HQ2	0.59644
Q2Q067	Q2VPK5	0.68166
Q2Q067	P22531	0.4396
Q2Q067	Q9UKJ5	0.20853
Q2Q067	Q02878	0.86759
Q2Q067	Q8IYF3	0.41833
Q2Q067	Q7Z3Y8	0.24865
Q2Q067	P46777	1.2478
Q2Q067	O94888	0.37669
Q2Q067	P25440	0.44349
Q2Q067	Q674X7	0.33682
Q2Q067	Q05519	0.64186
Q2Q067	Q9BVC3	0.5637
Q2Q067	O15499	0.23001
Q2Q067	Q96IL0	0.14247
Q2Q067	Q03924	0.099343
Q2Q067	Q8TBZ0	0.23272
Q2Q067	O60826	0.35962
Q2Q067	Q9BXL8	0.11478
Q2Q067	Q8TAT6	0.4564
Q2Q067	Q92562	0.25618
Q2Q067	Q96LX8	0.13171
Q2Q067	Q8N6T0	0.1112
Q2Q067	Q96E11	0.36691
Q2Q067	Q9BVC4	0.38322

Q2Q067	Q8WWC4	0.3021
Q2Q067	P55854	0.48173
Q2Q067	Q9UBK9	0.9743
Q2Q067	O95486	0.37983
Q2Q067	O95424	0.098644
Q2Q067	P26368	0.50935
Q2Q067	Q9NRJ2	0.19215
Q2Q067	Q5MIZ7	0.34658
Q2Q067	Q96QU4	0.35987
Q2Q067	P50748	0.7517
Q2Q067	Q92769	0.37839
Q2Q067	P0CL82	0.019044
Q2Q067	A8MVM7	0.19215
Q2Q067	P28062	0.30294
Q2Q067	O43715	0.60731
Q2Q067	Q12904	0.16744
Q2Q067	Q8TBB1	0.70049
Q2Q067	Q9BVL2	0.66174
Q2Q067	Q5JNZ3	0.14538
Q2Q067	Q96EP1	0.50656
Q2Q067	P0CW71	0.19215
Q2Q067	Q3LI72	0.19215
Q2Q067	O14497	0.68177
Q2Q067	Q03701	0.36517
Q2Q067	Q7Z7F7	0.30983
Q2Q067	Q9NRL3	0.36948
Q2Q067	Q9Y2Q1	0.23552
Q2Q067	Q96D46	0.56745
Q2Q067	Q9H091	0.16259
Q2Q067	A6NHX0	0.42645
Q2Q067	O00255	0.18783
Q2Q067	Q5VTH2	0.3129
Q2Q067	P62899	0.65612
Q2Q067	O15105	0.30414
Q2Q067	Q6PK81	0.10998
Q2Q067	Q5JRC9	0.32957
Q2Q067	Q96NG5	0.2131
Q2Q067	Q8ND56	0.36151
Q2Q067	Q969H9	0.19215
Q2Q067	O43251	0.31738
Q2Q067	Q13114	0.39935
Q2Q067	P78344	0.39826
Q2Q067	Q06330	0.55419
Q2Q067	P15559	0.19366
Q2Q067	O14713	0.3134
Q2Q067	Q8N1L1	0.19215
Q2Q067	O75496	0.91921
Q2Q067	H3BMG3	0.10388
Q2Q067	Q6P2Q9	0.55483
Q2Q067	Q7LG56	0.23867
Q2Q067	P06396	0.4134
Q2Q067	Q96RL7	0.47185
Q2Q067	P14859	1.2183
Q2Q067	Q9BQ90	0.77585
Q2Q067	O75190	0.7221
Q2Q067	Q96A09	0.23631
Q2Q067	Q1RN00	0.19215
Q2Q067	Q9NR50	0.5329
Q2Q067	Q15543	1.032
Q2Q067	Q9UHU1	0.19215
Q2Q067	Q9UMS0	1.082
Q2Q067	O14530	0.26249
Q2Q067	Q9UHV7	0.5241
Q2Q067	Q8IZ40	0.61441
Q2Q067	Q96PM5	0.41147
Q2Q067	Q5SY13	0.19215
Q2Q067	Q86UW9	0.83797
Q2Q067	Q7Z3K6	0.56295
Q2Q067	P60368	0.13835
Q2Q067	P56282	0.12754
Q2Q067	A6NNF4	0.16589
Q2Q067	O95644	0.85682
Q2Q067	P24864	0.62119
Q2Q067	Q9GZV5	1.0546
Q2Q067	P09467	0.31993
Q2Q067	Q96EK7	0.25011

53125	Q2Q067	Q96KH6	0.20696
53126	Q2Q067	O75444	0.45861
53127	Q2Q067	Q13127	0.52563
53128	Q2Q067	Q9P2J8	0.14687
53129	Q2Q067	Q8N9E0	0.29573
53130	Q2Q067	O95780	0.26444
53131	Q2Q067	Q7RTS9	0.69343
53132	Q2Q067	Q5TBC7	0.19429
53133	Q2Q067	P54278	0.36292
53134	Q2Q067	Q9HB07	0.20036
53135	Q2Q067	O95067	0.55782
53136	Q2Q067	P49848	0.74102
53137	Q2Q067	A8MTZ7	0.013691
53138	Q2Q067	Q9UQ84	0.23423
53139	Q2Q067	Q9BQ89	0.55269
53140	Q2Q067	Q8IYW2	0.19215
53141	Q2Q067	P18669	0.59899
53142	Q2Q067	O75843	0.62243
53143	Q2Q067	P08708	0.45742
53144	Q2Q067	Q96JM3	0.30197
53145	Q2Q067	O95447	0.062404
53146	Q2Q067	Q8NHY5	0.47754
53147	Q2Q067	Q8N3F0	0.19189
53148	Q2Q067	Q9BSJ2	1.1259
53149	Q2Q067	O43541	0.63458
53150	Q2Q067	Q969Q6	0.20448
53151	Q2Q067	P41567	0.44098
53152	Q2Q067	Q5TI25	0.72826
53153	Q2Q067	Q6PF04	0.10934
53154	Q2Q067	Q9NSA3	0.78176
53155	Q2Q067	Q9HCU9	1.1108
53156	Q2Q067	Q8WTX7	0.21796
53157	Q2Q067	Q9NWA0	1.0006
53158	Q2Q067	Q6NZY4	0.57421
53159	Q2Q067	Q9H8M7	0.80582
53160	Q2Q067	Q75L30	0.19215
53161	Q2Q067	Q5JR59	0.58169
53162	Q2Q067	Q9HD42	0.96738
53163	Q2Q067	P12525	0.28643
53164	Q2Q067	Q14135	0.39593
53165	Q2Q067	Q9Y2V2	0.23707
53166	Q2Q067	P28324	1.4495
53167	Q2Q067	Q9NPI8	0.79638
53168	Q2Q067	Q2M1V0	0.32768
53169	Q2Q067	Q9BR84	0.25938
53170	Q2Q067	Q9NQG5	1.1012
53171	Q2Q067	Q9BV20	0.1163
53172	Q2Q067	Q9H116	0.3242
53173	Q2Q067	Q9BUW7	0.29573
53174	Q2Q067	Q53EL6	1.0571
53175	Q2Q067	O00625	0.5364
53176	Q2Q067	Q9H869	1.3764
53177	Q2Q067	Q16719	0.32377
53178	Q2Q067	Q6ZW13	0.19215
53179	Q2Q067	P29474	0.59305
53180	Q2Q067	Q86Y97	0.38387
53181	Q2Q067	Q9Y6X2	0.68746
53182	Q2Q067	Q96EH5	0.31757
53183	Q2Q067	P50991	0.22812
53184	Q2Q067	P78345	0.83239
53185	Q2Q067	Q8IU53	0.30456
53186	Q2Q067	P50750	0.32865
53187	Q2Q067	B2RV13	0.29537
53188	Q2Q067	Q9UNM6	0.21423
53189	Q2Q067	O43598	0.14352
53190	Q2Q067	Q9Y2N7	0.59498
53191	Q2Q067	Q99732	0.17771
53192	Q2Q067	Q96E29	0.25377
53193	Q2Q067	P08865	0.82696
53194	Q2Q067	Q9H2J4	0.6157
53195	Q2Q067	Q96JB2	0.77765
53196	Q2Q067	Q8IZM0	0.036535
53197	Q2Q067	Q9UBB9	0.3683
53198	Q2Q067	Q9NZE8	0.34099
53199	Q2Q067	Q9UBW7	0.50531
53200	Q2Q067	Q96DE0	0.52521

Q2Q067	Q9BYR4	0.34715
Q2Q067	Q9ULH7	0.86805
Q2Q067	P11161	0.43705
Q2Q067	Q9Y5B0	0.53599
Q2Q067	Q9NWT6	0.46768
Q2Q067	Q07020	0.77652
Q2Q067	Q96LC9	0.53279
Q2Q067	Q6P4R8	1.1095
Q2Q067	Q9Y6R9	0.31304
Q2Q067	Q9UDY2	0.9378
Q2Q067	Q8IXS0	0.29573
Q2Q067	Q15544	0.88756
Q2Q067	Q9NTX7	0.46641
Q2Q067	Q8IZU0	0.46789
Q2Q067	Q9H9G7	0.70956
Q2Q067	Q8IYT1	0.26415
Q2Q067	Q9Y603	1.2469
Q2Q067	Q9H4M3	0.21437
Q2Q067	Q9NWT8	0.79659
Q2Q067	Q96MC4	0.41621
Q2Q067	P62913	1.1154
Q2Q067	Q8TAE8	0.98322
Q2Q067	A2AJT9	0.28979
Q2Q067	Q12906	1.1711
Q2Q067	Q8WXE1	0.69055
Q2Q067	Q96AP0	0.058987
Q2Q067	Q9NZM5	0.2044
Q2Q067	P0C880	0.19215
Q2Q067	Q9BT25	0.45603
Q2Q067	Q9HAP2	0.65234
Q2Q067	Q8IUC2	0.41492
Q2Q067	P25788	0.71276
Q2Q067	Q9UN79	0.93491
Q2Q067	Q92529	0.23318
Q2Q067	O94855	0.37983
Q2Q067	Q9UL42	0.82285
Q2Q067	O75152	0.17304
Q2Q067	Q9BYQ9	0.34715
Q2Q067	Q9HD36	0.44153
Q2Q067	O95561	0.33241
Q2Q067	Q96Q89	0.367
Q2Q067	Q13233	0.50718
Q2Q067	Q5SQS8	0.18589
Q2Q067	O15265	1.0666
Q2Q067	Q969U7	0.58946
Q2Q067	Q9HD40	0.47685
Q2Q067	O14602	0.33755
Q2Q067	Q05BQ5	0.19432
Q2Q067	O60783	0.27648
Q2Q067	Q719H9	0.71882
Q2Q067	P82094	1.3206
Q2Q067	Q8WXD5	0.91066
Q2Q067	Q08211	0.24611
Q2Q067	P48382	0.95927
Q2Q067	Q9H095	0.12544
Q2Q067	O43679	0.20599
Q2Q067	Q99733	0.73896
Q2Q067	A6NDR6	0.25814
Q2Q067	O00268	1.1435
Q2Q067	Q00587	0.1208
Q2Q067	Q9Y5U2	0.27963
Q2Q067	P09874	0.65642
Q2Q067	P51532	0.39878
Q2Q067	Q9H6K5	0.29573
Q2Q067	Q8NI51	0.57281
Q2Q067	P62269	0.72891
Q2Q067	A7MCY6	0.29324
Q2Q067	A0JLT2	0.6262
Q2Q067	P60228	0.9759
Q2Q067	P42575	0.28737
Q2Q067	Q6P2C8	0.93857
Q2Q067	Q86V87	0.28054
Q2Q067	Q13426	0.36253
Q2Q067	P15622	0.12037
Q2Q067	Q5JSZ5	0.45822
Q2Q067	Q9BR76	0.25203

Q2Q067	Q8NA77	0.38647
Q2Q067	Q5SZD1	0.030898
Q2Q067	A4D0V7	0.46408
Q2Q067	Q6RSH7	0.38366
Q2Q067	P43357	0.28783
Q2Q067	P78386	0.17395
Q2Q067	P42025	0.29978
Q2Q067	Q9H6D7	0.45285
Q2Q067	P0DI81	1.0626
Q2Q067	Q9C0F1	0.15787
Q2Q067	O14656	0.1222
Q2Q067	Q9H4L7	0.28997
Q2Q067	P57077	0.81701
Q2Q067	Q2T9L4	0.045843
Q2Q067	Q9H6U6	0.77469
Q2Q067	Q53GL7	0.42253
Q2Q067	Q3SXR2	0.097006
Q2Q067	Q96K62	0.12473
Q2Q067	P08579	0.23294
Q2Q067	Q9BUL8	0.64903
Q2Q067	Q02410	0.4695
Q2Q067	Q9HCU8	0.45491
Q2Q067	Q7Z3K3	0.4342
Q2Q067	Q5T200	0.49872
Q2Q067	P62136	0.64024
Q2Q067	Q9NP92	0.44968
Q2Q067	Q53HC0	0.55049
Q2Q067	Q96PU5	0.24726
Q2Q067	Q3KP31	0.22136
Q2Q067	Q5JVG8	0.21596
Q2Q067	O75170	0.41832
Q2Q067	O75419	0.85684
Q2Q067	P0C7V6	0.41066
Q2Q067	Q13616	0.63441
Q2Q067	Q99618	0.50762
Q2Q067	Q99550	0.29368
Q2Q067	O43830	0.07691
Q2Q067	P0C7T7	0.19215
Q2Q067	Q2NL98	0.51994
Q2Q067	P13807	0.38518
Q2Q067	Q96MT8	0.99208
Q2Q067	Q9UIL1	0.66124
Q2Q067	Q99848	0.12439
Q2Q067	P0DI82	0.27065
Q2Q067	Q16254	1.0289
Q2Q067	Q9Y6G3	0.1688
Q2Q067	Q6ZSG2	0.14952
Q2Q067	Q14525	0.16267
Q2Q067	P62380	0.10365
Q2Q067	P52597	0.45981
Q2Q067	Q92530	0.66661
Q2Q067	Q9H8W3	0.26101
Q2Q067	Q68CJ9	0.7447
Q2Q067	P08697	0.088986
Q2Q067	Q0VDD5	0.19215
Q2Q067	Q8NI29	0.19476
Q2Q067	Q9UQN3	0.45283
Q2Q067	O43918	0.98354
Q2Q067	O15078	0.59182
Q2Q067	P41212	0.80779
Q2Q067	Q8TEB1	0.24229
Q2Q067	P61236	0.15082
Q2Q067	O75251	0.14207
Q2Q067	Q9UKX7	1.0952
Q2Q067	A8MVP8	0.0051645
Q2Q067	Q9C0I3	0.44766
Q2Q067	Q9HD47	0.12585
Q2Q067	Q8TAY7	0.8034
Q2Q067	Q6K0P9	0.19747
Q2Q067	A5PLN9	0.20866
Q2Q067	O94868	0.28783
Q2Q067	Q96Q83	0.51771
Q2Q067	P0CJ86	0.27517
Q2Q067	Q9BYP8	0.19215
Q2Q067	Q8IUI4	0.14271
Q2Q067	Q9NVD7	0.25107

Q2Q067	Q49A33	0.21234
Q2Q067	P50613	0.16905
Q2Q067	Q9UHV9	0.80141
Q2Q067	P07814	0.17255
Q2Q067	Q8IW19	0.34829
Q2Q067	P15408	0.60615
Q2Q067	O75306	0.17028
Q2Q067	Q5T953	0.39148
Q2Q067	O15198	0.4728
Q2Q067	P48634	0.81743
Q2Q067	A6NCL1	0.16771
Q2Q067	Q8TF46	0.20595
Q2Q067	Q15742	0.32419
Q2Q067	Q5VWM5	0.3955
Q2Q067	Q6P161	0.050442
Q2Q067	O14746	0.50018
Q2Q067	Q8NEP4	0.29573
Q2Q067	Q8TAD1	0.1321
Q2Q067	P23246	0.74507
Q2Q067	Q6IS14	0.22578
Q2Q067	Q12873	0.52831
Q2Q067	Q9NVC6	0.55772
Q2Q067	P86452	0.44885
Q2Q067	Q9NX46	0.28347
Q2Q067	Q9UNX4	0.25362
Q2Q067	Q8TAI1	0.17874
Q2Q067	P14678	0.84317
Q2Q067	Q96CV9	0.4484
Q2Q067	Q9UN37	0.4286
Q2Q067	Q9Y5S9	0.78715
Q2Q067	Q9UHY8	0.21701
Q2Q067	Q8IWJ2	0.65581
Q2Q067	Q6DCA0	0.048023
Q2Q067	Q9UHT4	0.19215
Q2Q067	Q86YA3	0.46888
Q2Q067	P59990	0.34715
Q2Q067	P25208	0.67783
Q2Q067	Q14677	0.45812
Q2Q067	Q969P5	0.083655
Q2Q067	P49354	0.28904
Q2Q067	O95810	0.1401
Q2Q067	Q9BW85	0.52606
Q2Q067	Q8WVJ9	0.61729
Q2Q067	Q7L8S5	0.15009
Q2Q067	Q15327	0.45975
Q2Q067	Q96H20	0.97764
Q2Q067	P62424	1.1042
Q2Q067	P48668	0.24811
Q2Q067	Q9ULU4	0.49831
Q2Q067	Q92830	0.76579
Q2Q067	Q4G0N7	0.26286
Q2Q067	Q8TC05	0.38025
Q2Q067	P04406	0.43996
Q2Q067	A6NME0	0.086493
Q2Q067	O15164	0.2597
Q2Q067	Q9GZX7	0.22546
Q2Q067	Q00005	0.39272
Q2Q067	Q9BQ95	1.0645
Q2Q067	Q3SY52	0.097807
Q2Q067	Q96EU6	0.20221
Q2Q067	Q9Y2T2	0.48849
Q2Q067	P0C7H8	0.028225
Q2Q067	P49366	0.38667
Q2Q067	Q9UGK8	0.63032
Q2Q067	P78560	0.53874
Q2Q067	Q9H467	0.99517
Q2Q067	P61088	0.72685
Q2Q067	P57055	0.35678
Q2Q067	Q13506	0.39213
Q2Q067	P35226	0.56358
Q2Q067	Q9H0R1	0.10509
Q2Q067	Q68D10	0.29573
Q2Q067	Q8N1X5	0.19215
Q2Q067	B4DX44	0.24123
Q2Q067	Q15329	1.2808
Q2Q067	Q9Y4W2	0.4697

Q2Q067	Q8N8E1	0.141
Q2Q067	P35869	0.38948
Q2Q067	Q9H160	0.41814
Q2Q067	Q5VVC0	0.14578
Q2Q067	Q8TD16	0.18846
Q2Q067	Q9H7P6	0.45961
Q2Q067	Q9HB75	0.59831
Q2Q067	Q8NI08	0.79678
Q2Q067	Q5SNX5	0.19215
Q2Q067	P25786	0.59507
Q2Q067	Q7Z4U5	0.50891
Q2Q067	Q9NTI5	0.81387
Q2Q067	Q9UFF9	0.54407
Q2Q067	Q6L8G4	0.19215
Q2Q067	Q01081	0.50209
Q2Q067	P62917	0.79151
Q2Q067	Q99615	0.76055
Q2Q067	Q4G0G2	0.062331
Q2Q067	Q96BK5	0.49728
Q2Q067	Q8N3X1	0.15082
Q2Q067	Q96M63	0.02824
Q2Q067	O75530	0.60426
Q2Q067	Q7L2K0	0.025513
Q2Q067	Q9Y2J4	0.28511
Q2Q067	O75533	0.27774
Q2Q067	Q5TCQ9	0.51783
Q2Q067	Q5VU43	0.91543
Q2Q067	Q8N988	0.24518
Q2Q067	P57772	0.29999
Q2Q067	P59923	0.16182
Q2Q067	Q9Y6B2	0.92479
Q2Q067	Q8TC84	0.32989
Q2Q067	Q9BQY4	0.91181
Q2Q067	Q8N8R7	0.47001
Q2Q067	Q16543	0.39807
Q2Q067	Q96QR8	0.25392
Q2Q067	Q13439	0.62465
Q2Q067	Q9NVR5	0.37008
Q2Q067	Q14865	0.29149
Q2Q067	Q96CT7	0.048796
Q2Q067	Q13094	0.58586
Q2Q067	O95397	0.50547
Q2Q067	Q9GZN2	0.41768
Q2Q067	P0CJ85	0.27517
Q2Q067	Q96RS0	0.59831
Q2Q067	Q8TEQ6	0.79711
Q2Q067	Q9NRG9	0.88098
Q2Q067	Q08379	0.24553
Q2Q067	Q9NRI7	0.19215
Q2Q067	Q9BV86	0.27257
Q2Q067	Q13045	1.0426
Q2Q067	Q9Y4L5	0.34282
Q2Q067	Q9UMS6	0.59922
Q2Q067	P62829	0.96199
Q2Q067	Q9NZZ3	0.97999
Q2Q067	Q5VWN6	0.029137
Q2Q067	Q9Y592	0.39615
Q2Q067	Q9Y6H1	0.46303
Q2Q067	Q92733	0.7732
Q2Q067	Q8N2C9	0.0074981
Q2Q067	Q9Y605	0.85968
Q2Q067	Q8TE02	1.1888
Q2Q067	Q9BRX2	0.29713
Q2Q067	Q969J2	0.08952
Q2Q067	Q9P2M1	0.47001
Q2Q067	Q16514	0.88551
Q2Q067	Q5VUA4	0.38433
Q2Q067	Q658Y4	0.23348
Q2Q067	Q8N616	0.19215
Q2Q067	Q49A88	0.21748
Q2Q067	Q6UB98	0.32989
Q2Q067	A2RUB6	0.062424
Q2Q067	Q9NYT6	0.087195
Q2Q067	Q9BVJ6	0.76251
Q2Q067	Q8NA31	0.32608
Q2Q067	Q8NHW3	0.30206

Q2Q067	P78396	0.88691
Q2Q067	Q96FX7	0.14337
Q2Q067	Q3SY84	0.15455
Q2Q067	Q13951	0.69524
Q2Q067	O00567	0.80842
Q2Q067	P20962	0.26036
Q2Q067	Q15185	0.49607
Q2Q067	P0DM63	0.2076
Q2Q067	Q494X3	0.2352
Q2Q067	O15116	0.85257
Q2Q067	Q96GM5	0.73649
Q2Q067	Q6NSI3	0.46166
Q2Q067	Q9Y4E1	0.23137
Q2Q067	A6NM28	0.087389
Q2Q067	Q86TC9	0.36967
Q2Q067	P82970	0.65236
Q2Q067	Q9Y2L8	0.2806
Q2Q067	Q9NR77	0.17731
Q2Q067	Q8IYB9	0.17393
Q2Q067	Q14157	0.27733
Q2Q067	P46100	0.53938
Q2Q067	Q9BSQ5	0.21053
Q2Q067	Q9NRY2	0.74516
Q2Q067	Q6ZVQ6	0.64833
Q2Q067	Q9BY27	0.1902
Q2Q067	P0DMC3	0.19215
Q2Q067	Q16621	0.5124
Q2Q067	P56211	0.1133
Q2Q067	Q9H410	0.86114
Q2Q067	P46783	1.3783
Q2Q067	Q92993	0.97777
Q2Q067	P25815	0.50011
Q2Q067	Q9UGL9	0.19215
Q2Q067	Q8IYD1	0.25773
Q2Q067	Q8IX07	0.30822
Q2Q067	Q96DG6	0.020614
Q2Q067	Q9BRS8	0.15525
Q2Q067	O76021	0.18936
Q2Q067	O60291	0.41047
Q2Q067	Q5TB80	0.77652
Q2Q067	O60907	0.78214
Q2Q067	Q53G59	0.47555
Q2Q067	Q6UWZ7	0.4372
Q2Q067	O00198	0.3284
Q2Q067	Q9P2I0	0.8431
Q2Q067	Q1W6H9	0.57734
Q2Q067	Q5RGN0	0.3158
Q2Q067	P99999	0.71729
Q2Q067	Q5SYE7	0.25742
Q2Q067	Q6ZVF9	0.29429
Q2Q067	Q6IA86	0.56015
Q2Q067	Q8WWM1	0.29682
Q2Q067	Q8TB05	0.29573
Q2Q067	P10599	0.37112
Q2Q067	Q8TCY9	0.18241
Q2Q067	Q32M78	0.20406
Q2Q067	O15527	0.47257
Q2Q067	Q9H9Z2	0.72373
Q2Q067	O60225	0.12144
Q2Q067	Q96EF9	0.13335
Q2Q067	Q8NFH3	0.96831
Q2Q067	Q9BVC6	0.25552
Q2Q067	O00505	1.0699
Q2Q067	O60299	0.22169
Q2Q067	Q9BXS5	0.78023
Q2Q067	Q15599	0.39808
Q2Q067	P14651	0.82501
Q2Q067	Q9UIA9	0.40708
Q2Q067	Q9BUH6	0.21267
Q2Q067	A6NK89	0.33428
Q2Q067	Q8TEA8	0.49464
Q2Q067	Q9P278	0.6859
Q2Q067	Q9Y3C8	0.25661
Q2Q067	Q8NAE3	0.14783
Q2Q067	Q96EN9	0.28783
Q2Q067	Q7Z465	0.70091

Q2Q067	Q9H0B6	0.59695
Q2Q067	P50749	0.68057
Q2Q067	P53805	0.62064
Q2Q067	Q701N2	0.19215
Q2Q067	P53041	0.57333
Q2Q067	Q03468	0.35123
Q2Q067	Q6UUV7	0.95107
Q2Q067	O75030	0.59186
Q2Q067	O94850	0.13591
Q2Q067	Q99996	0.47016
Q2Q067	Q9H7U1	0.5887
Q2Q067	P0CW24	0.063253
Q2Q067	P61574	0.36503
Q2Q067	O14771	0.34111
Q2Q067	Q16890	0.39259
Q2Q067	P57678	0.93385
Q2Q067	O75920	0.59206
Q2Q067	Q9UPW6	0.75656
Q2Q067	Q9NQZ8	0.097501
Q2Q067	P0CH99	0.33562
Q2Q067	P25685	0.30525
Q2Q067	Q5SWL7	0.33217
Q2Q067	Q9BYD3	0.42449
Q2Q067	Q6ZWC4	0.013407
Q2Q067	P61244	0.92522
Q2Q067	Q96JE7	0.46306
Q2Q067	Q8WUW1	0.10774
Q2Q067	Q8NHZ8	1.2126
Q2Q067	Q8NFD5	0.8695
Q2Q067	Q8WUI4	0.34989
Q2Q067	Q9BUN5	0.39517
Q2Q067	Q9P0J0	0.43792
Q2Q067	Q8N823	0.16182
Q2Q067	P17041	0.085739
Q2Q067	P13984	0.54364
Q2Q067	O60516	0.22678
Q2Q067	Q9NZJ4	0.053608
Q2Q067	Q6P9B9	0.14385
Q2Q067	Q8N9K5	0.12582
Q2Q067	P62495	0.6309
Q2Q067	Q4VC05	0.24573
Q2Q067	Q8WVP5	0.46852
Q2Q067	P55055	0.27905
Q2Q067	A8MUQ0	0.54439
Q2Q067	Q9UIH9	0.33767
Q2Q067	Q13243	0.73564
Q2Q067	Q8IY85	0.24057
Q2Q067	Q9UK41	0.70008
Q2Q067	Q9ULW0	0.75066
Q2Q067	P09661	0.6216
Q2Q067	P38936	0.24536
Q2Q067	H0Y7S4	0.0356
Q2Q067	Q9BSG1	0.1979
Q2Q067	Q13618	0.4084
Q2Q067	P40925	0.50653
Q2Q067	Q9NWQ4	0.25028
Q2Q067	P0CJ79	0.13064
Q2Q067	Q3MJ62	0.064855
Q2Q067	Q969S3	0.69024
Q2Q067	Q66K80	0.19215
Q2Q067	P12956	0.28914
Q2Q067	Q15796	0.6625
Q2Q067	Q8N7M2	0.2352
Q2Q067	Q9BT49	0.37438
Q2Q067	O60765	0.1235
Q2Q067	Q8NB50	0.17365
Q2Q067	O95232	0.47333
Q2Q067	O75475	1.437
Q2Q067	P20248	1.0139
Q2Q067	Q8N0V3	0.38294
Q2Q067	Q9P2G4	0.29573
Q2Q067	Q6ZUF6	0.039038
Q2Q067	Q6ZV65	0.1916
Q2Q067	Q9BYR8	0.46381
Q2Q067	Q8IWZ3	0.95545
Q2Q067	P21912	0.23634

Q2Q067	P36578	1.1205
Q2Q067	Q5JQS6	0.14012
Q2Q067	Q8IYB0	0.19215
Q2Q067	O43790	0.24811
Q2Q067	Q9H0W9	0.59309
Q2Q067	Q12980	0.30116
Q2Q067	Q9NVU0	0.29186
Q2Q067	Q9NZU7	0.33072
Q2Q067	Q14739	0.24525
Q2Q067	Q6PGP7	0.71583
Q2Q067	Q15369	1.4885
Q2Q067	Q3ZCT1	0.11131
Q2Q067	P50570	0.2484
Q2Q067	Q02241	0.60864
Q2Q067	P0CG43	0.19215
Q2Q067	Q02080	0.44277
Q2Q067	O60303	0.089781
Q2Q067	Q8N9N2	0.29007
Q2Q067	Q8N344	0.56295
Q2Q067	Q5SNV9	0.14361
Q2Q067	Q68E01	0.51307
Q2Q067	O60216	0.73233
Q2Q067	Q96DM1	0.40861
Q2Q067	Q9UKV8	0.82272
Q2Q067	Q9P2R7	0.12719
Q2Q067	P60412	0.34715
Q2Q067	P39748	0.25775
Q2Q067	Q8N1Q8	0.2254
Q2Q067	Q86VQ0	0.22681
Q2Q067	O75665	0.68434
Q2Q067	Q5VTE6	0.005233
Q2Q067	Q9NSI6	0.77944
Q2Q067	Q9C0C2	0.12993
Q2Q067	Q9NRL2	0.97584
Q2Q067	O00321	0.36394
Q2Q067	P62857	0.53096
Q2Q067	Q7Z7C8	0.39993
Q2Q067	Q9Y2P8	0.25341
Q2Q067	P69905	0.23079
Q2Q067	Q15366	0.97393
Q2Q067	Q96KS0	0.15817
Q2Q067	O96020	0.97371
Q2Q067	O75164	1.0338
Q2Q067	Q8WVV9	0.084811
Q2Q067	Q16401	0.44118
Q2Q067	Q2M329	0.20623
Q2Q067	Q96BY2	0.44081
Q2Q067	O00139	0.30244
Q2Q067	Q5HYK9	0.14282
Q2Q067	Q96BP2	1.1637
Q2Q067	Q6WBX8	0.40347
Q2Q067	Q96S19	0.044202
Q2Q067	O96007	0.35731
Q2Q067	A8MWP6	0.19215
Q2Q067	Q9NRA8	1.0807
Q2Q067	Q9UBU8	0.72779
Q2Q067	P12883	0.029906
Q2Q067	P62875	0.15766
Q2Q067	Q8NHU0	0.070895
Q2Q067	Q9HAV4	1.1922
Q2Q067	P16333	0.6178
Q2Q067	P78337	0.43456
Q2Q067	Q8N0W5	0.0050811
Q2Q067	Q96C34	0.34894
Q2Q067	Q02556	0.45623
Q2Q067	Q96AQ1	0.32771
Q2Q067	Q5TAB7	0.39269
Q2Q067	Q9NTX9	0.033089
Q2Q067	Q0VFX4	0.18567
Q2Q067	Q9H4H8	0.30155
Q2Q067	Q99496	0.41054
Q2Q067	Q3L8U1	0.79413
Q2Q067	P12524	0.28643
Q2Q067	A4QMS7	0.12785
Q2Q067	Q9BZS9	0.19215
Q2Q067	Q53EZ4	0.61171

Q2Q067	Q96IR3	0.36085
Q2Q067	Q96QF7	0.090284
Q2Q067	P23760	0.88981
Q2Q067	O00472	0.53722
Q2Q067	Q5PR19	0.14285
Q2Q067	P09914	0.40286
Q2Q067	P13489	0.40547
Q2Q067	Q04725	0.84299
Q2Q067	Q53G26	0.62959
Q2Q067	Q5VTR2	0.65587
Q2Q067	O95218	0.54912
Q2Q067	P15259	0.4389
Q2Q067	Q494R0	0.19215
Q2Q067	Q96NB1	0.071453
Q2Q067	Q8N9Z0	0.20884
Q2Q067	P80294	0.68253
Q2Q067	Q96S94	0.32541
Q2Q067	Q99543	0.53207
Q2Q067	Q9HBH7	0.27598
Q2Q067	O14593	0.71517
Q2Q067	A6NH13	0.077732
Q2Q067	Q96AT1	0.29573
Q2Q067	O75312	0.20523
Q2Q067	Q9NRY5	0.5901
Q2Q067	O60308	0.20737
Q2Q067	Q92785	0.54201
Q2Q067	Q7L5D6	0.54305
Q2Q067	O14770	0.50626
Q2Q067	Q96NL0	0.093297
Q2Q067	Q6ZS72	0.035036
Q2Q067	Q96JN0	0.54127
Q2Q067	P24928	0.79577
Q2Q067	Q9BUK0	0.47723
Q2Q067	Q2VY69	0.13644
Q2Q067	P50453	0.67963
Q2Q067	Q969H6	0.86242
Q2Q067	Q7Z4T9	0.6529
Q2Q067	Q8IYX3	0.49265
Q2Q067	Q9Y6J9	0.84579
Q2Q067	Q6ZN84	0.00073997
Q2Q067	Q99607	1.146
Q2Q067	Q8N8E3	0.13549
Q2Q067	A1L190	0.45857
Q2Q067	P19338	0.523
Q2Q067	Q9NX63	0.76994
Q2Q067	Q9UJ98	0.19368
Q2Q067	Q7Z591	0.6833
Q2Q067	P37198	0.94595
Q2Q067	Q96FA3	0.27313
Q2Q067	Q9H6W3	0.22721
Q2Q067	P19419	1.4007
Q2Q067	O76009	0.1595
Q2Q067	Q86VE0	0.75201
Q2Q067	A6NF01	0.53604
Q2Q067	Q96T23	0.64822
Q2Q067	Q7Z2X4	0.21413
Q2Q067	A6NCN8	0.29573
Q2Q067	Q13363	0.41999
Q2Q067	Q9H0A9	0.65658
Q2Q067	Q8WTV1	0.51793
Q2Q067	Q9H0A8	0.47001
Q2Q067	P49639	0.27943
Q2Q067	A1A5D9	0.37499
Q2Q067	P61956	0.66633
Q2Q067	O95456	0.43156
Q2Q067	Q8WTW4	0.27195
Q2Q067	O43809	0.7152
Q2Q067	Q08AG7	0.62715
Q2Q067	P62879	0.45831
Q2Q067	Q8N7U7	0.27961
Q2Q067	O94986	1.1801
Q2Q067	Q86X95	1.0119
Q2Q067	Q4KMZ1	0.19545
Q2Q067	Q9UQF2	0.49242
Q2Q067	Q8NE22	0.51037
Q2Q067	P51523	0.11099

53809	Q2Q067	H3BNL8	0.62577
53810	Q2Q067	Q5VWP3	0.45084
53811	Q2Q067	Q9UKV3	0.60252
53812	Q2Q067	Q13503	0.65819
53813	Q2Q067	O75122	0.23468
53814	Q2Q067	Q9H4A6	0.35272
53815	Q2Q067	Q9BYD2	0.40045
53816	Q2Q067	Q6P444	0.24596
53817	Q2Q067	Q96HA7	0.72884
53818	Q2Q067	Q86WG3	0.27301
53819	Q2Q067	Q86YW9	0.35753
53820	Q2Q067	Q15014	1.1838
53821	Q2Q067	Q9UQK1	0.31962
53822	Q2Q067	Q96PI1	0.35595
53823	Q2Q067	O95777	0.60859
53824	Q2Q067	C9JC47	0.19215
53825	Q2Q067	Q9H7L9	0.60114
53826	Q2Q067	Q8NFJ8	0.39592
53827	Q2Q067	Q7Z5V6	0.33218
53828	Q2Q067	O00232	0.22677
53829	Q2Q067	Q15029	0.24706
53830	Q2Q067	O94874	0.78286
53831	Q2Q067	Q9H963	0.18125
53832	Q2Q067	Q9Y3F1	0.19215
53833	Q2Q067	Q58F21	0.25544
53834	Q2Q067	A6NCI8	0.43502
53835	Q2Q067	Q92499	0.3782
53836	Q2Q067	Q8NI27	0.48653
53837	Q2Q067	P0C841	0.19215
53838	Q2Q067	P78316	0.62208
53839	Q2Q067	O15370	0.41049
53840	Q2Q067	P12036	0.38138
53841	Q2Q067	Q99687	0.51701
53842	Q2Q067	Q9UKT7	0.37815
53843	Q2Q067	Q969R5	0.12247
53844	Q2Q067	Q8TAC9	0.21229
53845	Q2Q067	Q8N4B4	0.0048627
53846	Q2Q067	Q9NYW8	0.073244
53847	Q2Q067	Q96NS1	0.15082
53848	Q2Q067	Q9Y3D5	0.317
53849	Q2Q067	O76064	0.25902
53850	Q2Q067	P41162	0.32805
53851	Q2Q067	Q9NX70	1.0414
53852	Q2Q067	Q5TZA2	0.60841
53853	Q2Q067	Q0VDD7	0.80408
53854	Q2Q067	Q8WY41	0.37563
53855	Q2Q067	Q13309	0.5599
53856	Q2Q067	Q9C073	0.39992
53857	Q2Q067	Q92621	1.1037
53858	Q2Q067	Q86TG7	0.47799
53859	Q2Q067	O95476	0.20316
53860	Q2Q067	Q15819	0.57992
53861	Q2Q067	P54920	0.41764
53862	Q2Q067	Q5BKZ1	0.44438
53863	Q2Q067	A2RTY3	0.090801
53864	Q2Q067	Q9UJC5	0.2992
53865	Q2Q067	A8MX80	0.36346
53866	Q2Q067	Q3B726	0.14307
53867	Q2Q067	Q9BYN7	0.11099
53868	Q2Q067	Q96E09	0.41087
53869	Q2Q067	Q9UN36	0.48375
53870	Q2Q067	P62072	0.21425
53871	Q2Q067	Q9NP98	0.29468
53872	Q2Q067	Q9BYZ2	0.28317
53873	Q2Q067	Q9UK99	0.33387
53874	Q2Q067	Q96GE5	0.22136
53875	Q2Q067	Q15257	0.45136
53876	Q2Q067	Q9NWF9	0.76376
53877	Q2Q067	Q9NQ33	0.49715
53878	Q2Q067	Q86UP8	0.020629
53879	Q2Q067	P62847	0.52089
53880	Q2Q067	Q13216	0.41003
53881	Q2Q067	P09016	0.20026
53882	Q2Q067	Q07866	0.57641
53883	Q2Q067	Q9Y6Z5	0.4296
53884	Q2Q067	Q96IV0	0.41955

Q2Q067	E9PQ53	0.41088
Q2Q067	Q8WWQ0	0.59548
Q2Q067	Q8N7Z2	0.23387
Q2Q067	Q2NKJ3	0.2483
Q2Q067	Q9NX04	0.18379
Q2Q067	Q5T0Z8	0.29573
Q2Q067	Q8N720	0.87239
Q2Q067	P51786	0.1134
Q2Q067	Q9BWT1	0.5376
Q2Q067	Q9BQ69	0.42245
Q2Q067	O43812	0.33577
Q2Q067	Q15466	0.57433
Q2Q067	P19838	0.80098
Q2Q067	Q8IZX4	0.32539
Q2Q067	Q7Z739	0.35458
Q2Q067	Q3LI61	0.19215
Q2Q067	Q9BX70	0.50941
Q2Q067	Q96RU7	0.40416
Q2Q067	O75143	0.57818
Q2Q067	Q9BTA0	0.29573
Q2Q067	P81274	0.503
Q2Q067	O75564	0.15072
Q2Q067	Q9Y3E7	0.74027
Q2Q067	Q9H4S2	0.23981
Q2Q067	O75436	0.65274
Q2Q067	Q02833	0.28004
Q2Q067	O94805	0.65921
Q2Q067	Q9NRI5	0.31353
Q2Q067	O75822	0.69547
Q2Q067	Q8NDM7	0.22433
Q2Q067	A8K0S8	0.25814
Q2Q067	Q96QK1	0.67258
Q2Q067	Q9Y697	0.68656
Q2Q067	O15318	0.39305
Q2Q067	Q14764	1.0591
Q2Q067	Q9NNW7	0.2469
Q2Q067	Q14145	1.2608
Q2Q067	Q8IWD4	0.29573
Q2Q067	O43299	1.1628
Q2Q067	P43351	0.6546
Q2Q067	Q8IX90	0.54913
Q2Q067	P78395	0.70004
Q2Q067	O60675	0.44562
Q2Q067	Q99962	0.23041
Q2Q067	A6NGY1	0.40607
Q2Q067	P28749	1.3398
Q2Q067	Q68DK7	0.49031
Q2Q067	A8K5M9	0.43931
Q2Q067	Q8NG31	0.61982
Q2Q067	Q9UI25	0.19215
Q2Q067	O15397	0.78973
Q2Q067	Q9HCZ1	0.1371
Q2Q067	Q8NB42	0.24653
Q2Q067	P09012	0.33137
Q2Q067	P43268	0.47798
Q2Q067	P20823	0.26562
Q2Q067	A1L3X0	0.27937
Q2Q067	Q56P03	0.3005
Q2Q067	Q14CN4	0.16378
Q2Q067	Q8IZQ1	0.57051
Q2Q067	Q02952	0.095475
Q2Q067	Q8N4S0	0.29573
Q2Q067	Q68DX3	0.37964
Q2Q067	Q15051	0.53775
Q2Q067	Q96NM4	0.30214
Q2Q067	A6NKU9	0.23617
Q2Q067	O95865	0.35963
Q2Q067	Q8TDB6	0.35545
Q2Q067	O43504	0.14358
Q2Q067	P49207	0.65612
Q2Q067	O75943	0.18524
Q2Q067	Q9P1Q0	0.3571
Q2Q067	P78549	0.24732
Q2Q067	Q15583	0.29195
Q2Q067	P23193	1.2059
Q2Q067	Q9H0C5	0.50935

Q2Q067	Q16186	0.51632
Q2Q067	Q9UFN0	0.66387
Q2Q067	Q9ULW8	0.24394
Q2Q067	Q96NL8	0.30292
Q2Q067	P54098	0.41903
Q2Q067	Q13228	1.167
Q2Q067	O75360	0.48529
Q2Q067	Q8WXC7	0.29877
Q2Q067	P07738	0.39498
Q2Q067	Q9UM11	1.1325
Q2Q067	Q70EK8	0.35448
Q2Q067	Q658L1	0.37222
Q2Q067	Q3ZLR7	0.41066
Q2Q067	Q9H9H4	0.34221
Q2Q067	Q9H7E9	0.29573
Q2Q067	A6NJG6	0.32768
Q2Q067	Q9HBT7	0.1308
Q2Q067	Q96CD0	0.28783
Q2Q067	Q7L590	1.0005
Q2Q067	Q9C035	0.5585
Q2Q067	Q53HM4	0.086378
Q2Q067	Q8NHS9	0.061239
Q2Q067	Q13144	0.51879
Q2Q067	P23258	0.25072
Q2Q067	Q13155	0.64457
Q2Q067	Q9H816	0.46804
Q2Q067	Q96GX8	0.29573
Q2Q067	O95373	0.99159
Q2Q067	Q86VP1	0.44412
Q2Q067	Q9H9L3	0.39744
Q2Q067	Q9Y383	0.38285
Q2Q067	P17861	0.71423
Q2Q067	Q96CF2	0.13146
Q2Q067	P23396	0.80307
Q2Q067	Q8IWC1	0.51943
Q2Q067	A6NMN3	0.23345
Q2Q067	O76014	0.1595
Q2Q067	Q6V702	0.047142
Q2Q067	Q9H6Z9	0.51439
Q2Q067	Q07687	0.50565
Q2Q067	Q8TBZ5	0.11543
Q2Q067	P0C852	0.19215
Q2Q067	Q9NSI2	0.29573
Q2Q067	Q8TE69	0.28783
Q2Q067	Q86UD0	0.32989
Q2Q067	Q9HAH1	0.17187
Q2Q067	Q9BVT8	0.37351
Q2Q067	P13645	0.24722
Q2Q067	P61201	0.48844
Q2Q067	Q9H1B7	0.54659
Q2Q067	Q7RTU4	0.331
Q2Q067	Q9UJX4	0.79494
Q2Q067	Q96F83	0.23538
Q2Q067	A8MUI8	0.12312
Q2Q067	Q8N371	0.25169
Q2Q067	Q86VM9	0.08833
Q2Q067	Q9Y2P7	0.35911
Q2Q067	O43913	0.32451
Q2Q067	Q8N0V1	0.20711
Q2Q067	Q09161	0.85247
Q2Q067	Q6P3V2	0.044968
Q2Q067	Q12968	0.69183
Q2Q067	Q9Y5J7	0.22811
Q2Q067	Q1W209	0.19215
Q2Q067	P28074	0.58405
Q2Q067	O75346	0.21624
Q2Q067	A6NLJ0	0.4501
Q2Q067	Q8NEG2	0.24764
Q2Q067	Q5T6C5	0.41879
Q2Q067	P53618	0.75935
Q2Q067	Q7L266	0.20407
Q2Q067	Q9H009	0.26705
Q2Q067	P54136	0.70579
Q2Q067	P25789	0.71276
Q2Q067	Q15572	0.52261
Q2Q067	A6NCJ1	0.075582

Q2Q067	Q93008	0.3667
Q2Q067	Q8N5U0	0.19215
Q2Q067	Q14588	0.16182
Q2Q067	Q8WV24	0.58814
Q2Q067	Q6ZRM9	0.29537
Q2Q067	Q6PJG2	0.73738
Q2Q067	O14529	0.38741
Q2Q067	Q92540	0.97845
Q2Q067	P52630	0.74354
Q2Q067	Q6P3X8	0.23464
Q2Q067	Q9P013	0.45511
Q2Q067	Q9UPY8	0.92011
Q2Q067	O43791	0.54707
Q2Q067	Q9H0U6	0.21747
Q2Q067	O00629	0.54258
Q2Q067	O60248	0.53834
Q2Q067	Q9HCE1	0.67098
Q2Q067	Q9UKD2	0.092757
Q2Q067	Q5T5Y3	0.10527
Q2Q067	Q9H501	0.72614
Q2Q067	Q14839	0.53856
Q2Q067	Q2M2W7	0.29573
Q2Q067	Q8N7R1	0.3413
Q2Q067	Q9UBC1	0.47546
Q2Q067	Q96LL4	0.29573
Q2Q067	Q13620	0.69543
Q2Q067	Q6ZMV8	0.25223
Q2Q067	Q9NRX3	0.34211
Q2Q067	Q6UVJ0	0.73827
Q2Q067	Q9Y543	0.33048
Q2Q067	O15504	0.55455
Q2Q067	Q9UPV9	0.73538
Q2Q067	Q9Y6D9	0.89613
Q2Q067	Q8WW01	0.16169
Q2Q067	Q96H79	0.31077
Q2Q067	P15336	0.91556
Q2Q067	O60885	0.72097
Q2Q067	Q5HYJ3	0.27696
Q2Q067	Q7L592	0.1846
Q2Q067	Q9Y2X0	0.49579
Q2Q067	Q96HT8	0.76267
Q2Q067	O95996	0.22899
Q2Q067	Q86XD5	0.087168
Q2Q067	O95073	1.0622
Q2Q067	Q8N9W8	0.63352
Q2Q067	Q9Y4K1	0.36388
Q2Q067	P59998	0.032071
Q2Q067	Q14592	0.11369
Q2Q067	Q9BY65	0.17246
Q2Q067	Q8IXH7	1.211
Q2Q067	Q9HCG8	0.13524
Q2Q067	P21399	0.34387
Q2Q067	Q9UQ35	0.27937
Q2Q067	Q13526	0.54209
Q2Q067	Q8TB33	0.0020931
Q2Q067	A6NLX3	0.086897
Q2Q067	O94864	0.52148
Q2Q067	Q8N5Q1	0.63352
Q2Q067	Q6AZW8	0.11061
Q2Q067	Q6NSZ9	0.058453
Q2Q067	Q9BVW5	0.93848
Q2Q067	P12757	0.31397
Q2Q067	A6NMK8	0.24138
Q2Q067	Q8WU39	0.19746
Q2Q067	P28838	0.32575
Q2Q067	A6NI86	0.36408
Q2Q067	O43615	0.12862
Q2Q067	Q9H2K0	0.15085
Q2Q067	P35813	0.24988
Q2Q067	Q8N1K5	0.19622
Q2Q067	Q9BUJ2	1.1326
Q2Q067	P42768	0.32835
Q2Q067	Q86V15	0.24936
Q2Q067	Q9UKT4	0.85641
Q2Q067	P28698	0.71774
Q2Q067	A2IDD5	0.24519

54113	Q2Q067	Q9H3P2	1.0781
54114	Q2Q067	Q6NUQ1	0.46762
54115	Q2Q067	Q8NFZ5	0.91099
54116	Q2Q067	Q9BYV9	0.36753
54117	Q2Q067	Q6ZNG0	0.095101
54118	Q2Q067	Q6J272	0.45578
54119	Q2Q067	P25963	0.6963
54120	Q2Q067	Q9UHT9	0.19215
54121	Q2Q067	Q8N9F8	0.12347
54122	Q2Q067	Q99619	0.50289
54123	Q2Q067	P0CG47	0.43777
54124	Q2Q067	Q9H503	0.24679
54125	Q2Q067	Q9P2K3	1.2155
54126	Q2Q067	Q5TG92	0.24542
54127	Q2Q067	P40121	0.36665
54128	Q2Q067	Q96MY1	0.41206
54129	Q2Q067	P04053	0.16257
54130	Q2Q067	P19013	0.3611
54131	Q2Q067	Q9UGK3	0.63634
54132	Q2Q067	P20042	0.45905
54133	Q2Q067	A1L170	0.19215
54134	Q2Q067	P0C7V5	0.16874
54135	Q2Q067	Q8IVD9	0.13223
54136	Q2Q067	P20290	1.1159
54137	Q2Q067	O43186	0.91667
54138	Q2Q067	Q13619	0.37811
54139	Q2Q067	Q12983	0.15809
54140	Q2Q067	P17021	0.12412
54141	Q2Q067	Q13569	0.47474
54142	Q2Q067	Q86U86	1.0201
54143	Q2Q067	Q86UW6	0.057907
54144	Q2Q067	O76042	0.26541
54145	Q2Q067	Q7RTV0	0.46192
54146	Q2Q067	Q6ZTI0	0.19215
54147	Q2Q067	Q9Y2M5	0.78992
54148	Q2Q067	Q9BPZ3	0.41707
54149	Q2Q067	O60573	0.54014
54150	Q2Q067	Q9HB03	0.27937
54151	Q2Q067	Q86XI8	0.32983
54152	Q2Q067	Q9H5Z6	0.29573
54153	Q2Q067	P08235	0.24961
54154	Q2Q067	Q14686	0.61404
54155	Q2Q067	Q6PJ61	0.47865
54156	Q2Q067	Q9UNY4	0.43574
54157	Q2Q067	Q8WV99	0.17572
54158	Q2Q067	P57740	0.79639
54159	Q2Q067	P06744	0.58779
54160	Q2Q067	O95983	1.2526
54161	Q2Q067	Q96T76	0.87561
54162	Q2Q067	Q8N998	0.1321
54163	Q2Q067	Q96BD8	0.57652
54164	Q2Q067	Q9H446	0.28783
54165	Q2Q067	Q8N865	0.067777
54166	Q2Q067	Q04917	0.24793
54167	Q2Q067	Q96QT6	0.90151
54168	Q2Q067	P04818	0.08183
54169	Q2Q067	Q9BZK7	0.93529
54170	Q2Q067	Q6QNY1	0.72768
54171	Q2Q067	Q9UI95	1.2173
54172	Q2Q067	Q9H900	0.4861
54173	Q2Q067	Q9Y3D0	0.93292
54174	Q2Q067	Q9HC62	0.44458
54175	Q2Q067	Q8N584	0.41526
54176	Q2Q067	O75934	0.65319
54177	Q2Q067	Q8N769	0.04082
54178	Q2Q067	Q9NWW0	0.1321
54179	Q2Q067	O14920	0.53843
54180	Q2Q067	Q9Y5N6	0.58096
54181	Q2Q067	Q15545	0.916
54182	Q2Q067	Q9NV56	0.51626
54183	Q2Q067	Q9UID6	0.49754
54184	Q2Q067	Q14872	0.82318
54185	Q2Q067	A6H8Z2	0.099505
54186	Q2Q067	A6NGY3	0.014439
54187	Q2Q067	Q969H0	0.71405
54188	Q2Q067	P11441	0.55479

Q2Q067	Q5T655	0.19215
Q2Q067	Q9UDV6	0.53405
Q2Q067	Q8N9Z2	0.28913
Q2Q067	P33552	0.24853
Q2Q067	Q3KNV8	0.88293
Q2Q067	P27348	0.27005
Q2Q067	O75688	0.30336
Q2Q067	P63173	0.55038
Q2Q067	Q15170	0.91417
Q2Q067	Q8N1V2	0.4017
Q2Q067	O95271	0.55337
Q2Q067	A4D1E1	0.34832
Q2Q067	Q7Z5W3	0.30807
Q2Q067	Q12789	0.83172
Q2Q067	P18621	0.66583
Q2Q067	Q9BPW8	0.2403
Q2Q067	O75638	0.66876
Q2Q067	Q9NZN8	0.76576
Q2Q067	Q5CZA5	0.14861
Q2Q067	Q03111	1.2772
Q2Q067	Q9BYQ0	0.34715
Q2Q067	P31946	0.57881
Q2Q067	Q9Y3C5	0.38958
Q2Q067	Q717R9	0.11898
Q2Q067	Q96HQ0	0.10921
Q2Q067	Q99590	0.54874
Q2Q067	Q9H6V9	0.14806
Q2Q067	Q32P41	0.33795
Q2Q067	Q3ZCQ2	0.19215
Q2Q067	Q13416	0.52064
Q2Q067	Q6NUR6	0.33189
Q2Q067	Q96JN8	0.097927
Q2Q067	Q9BRR8	0.45395
Q2Q067	Q9UKN5	0.24654
Q2Q067	P0DKX0	0.20817
Q2Q067	Q9BXN6	0.1321
Q2Q067	O60759	0.33914
Q2Q067	Q9UNY5	0.23106
Q2Q067	Q8TB72	0.48365
Q2Q067	P22415	0.52116
Q2Q067	Q9H4D5	0.27675
Q2Q067	Q8NA72	0.16939
Q2Q067	Q96N06	0.20389
Q2Q067	Q9BQE6	0.18439
Q2Q067	Q9NS73	0.71625
Q2Q067	P10244	0.5915
Q2Q067	Q8N3L3	0.44763
Q2Q067	Q9H707	0.11835
Q2Q067	Q96MG7	0.78122
Q2Q067	Q6VB84	0.45162
Q2Q067	O75150	0.45876
Q2Q067	A8MX34	0.043653
Q2Q067	P0C7P2	0.08037
Q2Q067	Q9UM07	0.32946
Q2Q067	Q96H12	0.59249
Q2Q067	Q06455	0.68894
Q2Q067	Q13761	0.7696
Q2Q067	Q9NSJ1	0.13291
Q2Q067	Q9H5V9	0.26016
Q2Q067	Q9NY12	0.17397
Q2Q067	Q6ZT83	0.029944
Q2Q067	Q9UI32	0.32457
Q2Q067	Q9BYA6	0.19215
Q2Q067	Q6ULP2	0.60143
Q2Q067	P13647	0.84255
Q2Q067	O75410	0.71458
Q2Q067	Q5VZR2	0.54396
Q2Q067	E5RJ46	0.19215
Q2Q067	Q9UKT9	0.46923
Q2Q067	Q6ZUL3	0.19215
Q2Q067	P82675	0.26409
Q2Q067	Q9C000	0.23977
Q2Q067	P41208	0.56902
Q2Q067	P52292	1.0043
Q2Q067	Q2KHT3	0.27949
Q2Q067	Q9H0A6	0.322

Q2Q067	Q8N9Z9	0.5973
Q2Q067	Q96HZ7	0.19215
Q2Q067	Q9UMQ3	0.70586
Q2Q067	Q5HYK7	1.1753
Q2Q067	Q92567	0.097372
Q2Q067	Q6ZS92	0.05235
Q2Q067	O15155	0.050295
Q2Q067	Q5JVS0	0.77575
Q2Q067	Q96IY1	0.88845
Q2Q067	Q5SVZ6	0.52342
Q2Q067	P52298	0.60569
Q2Q067	Q9NR09	0.34827
Q2Q067	Q9H9J2	0.13576
Q2Q067	Q3YBR2	0.47307
Q2Q067	P52655	1.0625
Q2Q067	B3KS81	0.075701
Q2Q067	Q6ZP98	0.033666
Q2Q067	Q8TCJ0	0.52452
Q2Q067	Q9UI43	0.36696
Q2Q067	P19484	0.56917
Q2Q067	O14646	0.56733
Q2Q067	Q13901	1.0971
Q2Q067	Q96NY9	0.59748
Q2Q067	P0CG12	0.63746
Q2Q067	P16989	0.24429
Q2Q067	O95238	0.27218
Q2Q067	B4DU55	0.14861
Q2Q067	H3BNL1	0.19958
Q2Q067	Q6PG37	0.24987
Q2Q067	P31314	0.64403
Q2Q067	Q9BPX1	0.24031
Q2Q067	O60290	0.31054
Q2Q067	Q86XK3	0.65109
Q2Q067	O75373	0.12589
Q2Q067	O75113	0.86437
Q2Q067	C9J6K1	0.20143
Q2Q067	Q9BYX4	0.22796
Q2Q067	Q6RUI8	0.16407
Q2Q067	Q9Y618	1.0608
Q2Q067	Q8IW36	0.22586
Q2Q067	Q14209	1.0888
Q2Q067	Q9BRP1	0.44985
Q2Q067	Q96IK1	0.046572
Q2Q067	Q9UFG5	0.28783
Q2Q067	O95619	0.82652
Q2Q067	P20618	0.7081
Q2Q067	Q96Q35	0.21453
Q2Q067	Q19AV6	0.6616
Q2Q067	Q05BW5	0.1197
Q2Q067	Q96CS4	0.0891
Q2Q067	A6NEF3	0.37471
Q2Q067	Q9BTL4	0.23433
Q2Q067	P45984	0.31783
Q2Q067	Q15628	0.59619
Q2Q067	Q9H0U9	0.32889
Q2Q067	Q13642	0.39105
Q2Q067	Q49AA0	0.22377
Q2Q067	P43356	0.90472
Q2Q067	A6NIH7	0.14363
Q2Q067	Q9HBM6	0.64399
Q2Q067	Q96LR7	0.4017
Q2Q067	P42771	0.57159
Q2Q067	Q8N490	0.19295
Q2Q067	Q8IZT6	0.29741
Q2Q067	P17096	0.56949
Q2Q067	Q9NP79	0.21927
Q2Q067	Q6L8G8	0.19215
Q2Q067	O95817	0.40658
Q2Q067	P14652	0.75998
Q2Q067	Q9UNE7	0.29173
Q2Q067	Q96GS4	0.55202
Q2Q067	Q04724	0.87158
Q2Q067	P48147	0.17299
Q2Q067	O95831	0.82038
Q2Q067	Q9ULG1	0.25781
Q2Q067	O75956	0.12532

Q2Q067	P48595	0.15224
Q2Q067	P35269	1.1671
Q2Q067	Q86SQ7	0.92008
Q2Q067	Q6PIF2	0.22309
Q2Q067	Q8TE85	0.26235
Q2Q067	Q15427	0.26438
Q2Q067	Q9H6Z4	1.0909
Q2Q067	Q8TC76	0.8034
Q2Q067	Q13425	0.41362
Q2Q067	P11474	0.3714
Q2Q067	Q99623	1.0884
Q2Q067	P09497	0.36942
Q2Q067	Q14139	0.52171
Q2Q067	Q8IUR6	0.96386
Q2Q067	O76015	0.1595
Q2Q067	Q8N987	0.18628
Q2Q067	O60763	0.33937
Q2Q067	Q9GZU2	0.4615
Q2Q067	Q9UKK6	0.65001
Q2Q067	Q86TJ5	0.090948
Q2Q067	O94925	0.51299
Q2Q067	Q12824	0.96773
Q2Q067	Q6NSI8	0.36551
Q2Q067	Q6DKK2	0.92814
Q2Q067	Q15714	0.77243
Q2Q067	Q9Y2X3	0.62828
Q2Q067	Q9BXV9	0.19215
Q2Q067	O00161	0.83252
Q2Q067	Q8N6Y0	0.57595
Q2Q067	P80723	0.2424
Q2Q067	A1L188	0.30457
Q2Q067	O43513	1.173
Q2Q067	O43325	0.47607
Q2Q067	Q9UBL0	0.1095
Q2Q067	Q9NS23	0.26693
Q2Q067	Q6PI48	0.51597
Q2Q067	Q0VG06	0.44196
Q2Q067	Q00653	1.2521
Q2Q067	Q8IVC4	0.10131
Q2Q067	Q9UHN1	0.068535
Q2Q067	Q9Y5J3	0.33325
Q2Q067	Q15833	0.31713
Q2Q067	Q8N488	0.48407
Q2Q067	Q96MB7	0.23984
Q2Q067	P0CG48	0.42887
Q2Q067	P49459	0.18529
Q2Q067	Q9UK10	0.16182
Q2Q067	Q9NRX2	0.22339
Q2Q067	P42166	0.10415
Q2Q067	A8MUZ8	0.25083
Q2Q067	Q9UPA5	0.11064
Q2Q067	P29083	1.1985
Q2Q067	P04731	0.18499
Q2Q067	A6NJB7	0.48545
Q2Q067	O75791	0.99055
Q2Q067	Q5SVJ3	0.13859
Q2Q067	Q8N8D1	0.34559
Q2Q067	Q5JUK9	0.071824
Q2Q067	O14925	0.11495
Q2Q067	P60953	0.52014
Q2Q067	Q96GG9	0.23957
Q2Q067	P0C5Y4	0.18143
Q2Q067	P0CZ20	0.1398
Q2Q067	Q5JUQ0	0.0064825
Q2Q067	Q9BZE2	0.6962
Q2Q067	A6NJI1	0.11178
Q2Q067	O75382	0.23749
Q2Q067	P25205	0.31163
Q2Q067	Q9NY43	0.4479
Q2Q067	O60504	0.67904
Q2Q067	Q9ULL0	0.32402
Q2Q067	Q9HAI6	0.36248
Q2Q067	O75154	0.77122
Q2Q067	Q96EK4	1.1978
Q2Q067	P07315	0.553
Q2Q067	Q86WR0	0.13106

54417	Q2Q067	Q96SE7	0.1529
54418	Q2Q067	Q9P0U1	0.11286
54419	Q2Q067	A6QL64	0.24591
54420	Q2Q067	O14556	0.45488
54421	Q2Q067	Q9NWB7	0.54182
54422	Q2Q067	P40855	0.17848
54423	Q2Q067	Q08752	0.31714
54424	Q2Q067	Q6ICG8	0.71678
54425	Q2Q067	A1L168	0.29573
54426	Q2Q067	Q13895	0.71903
54427	Q2Q067	P35251	0.26843
54428	Q2Q067	Q8N6M0	0.050661
54429	Q2Q067	Q9HCK8	0.33915
54430	Q2Q067	P46013	0.057215
54431	Q2Q067	P47974	0.25497
54432	Q2Q067	Q86YI8	0.51239
54433	Q2Q067	P40926	0.31757
54434	Q2Q067	Q9H7B2	0.46797
54435	Q2Q067	Q13409	0.42104
54436	Q2Q067	Q9BQ65	0.23303
54437	Q2Q067	Q14676	0.59527
54438	Q2Q067	P62316	0.81631
54439	Q2Q067	Q8N7Y1	0.19215
54440	Q2Q067	A6NNA2	0.41991
54441	Q2Q067	Q7RTU0	0.331
54442	Q2Q067	P61575	0.36503
54443	Q2Q067	Q96EZ4	0.19215
54444	Q2Q067	Q9P242	0.41813
54445	Q2Q067	Q8N7H5	0.90831
54446	Q2Q067	O43318	0.56984
54447	Q2Q067	Q86TS9	0.28685
54448	Q2Q067	P47928	0.42634
54449	Q2Q067	Q9UJA5	0.54666
54450	Q2Q067	Q7L945	0.13415
54451	Q2Q067	Q8NDQ6	0.14494
54452	Q2Q067	Q92600	1.1929
54453	Q2Q067	O75764	0.15182
54454	Q2Q067	A6NHR8	0.19215
54455	Q2Q067	Q9UI54	0.19215
54456	Q2Q067	Q495M9	0.2632
54457	Q2Q067	Q13106	0.088399
54458	Q2Q067	O43395	0.77663
54459	Q2Q067	P49336	0.30371
54460	Q2Q067	Q9GZT9	0.28162
54461	Q2Q067	Q9ULZ2	0.73769
54462	Q2Q067	Q86XF7	0.26079
54463	Q2Q067	Q5TEC3	0.10451
54464	Q2Q067	Q96AY2	0.70921
54465	Q2Q067	Q8IYN6	0.41087
54466	Q2Q067	O95487	0.71201
54467	Q2Q067	Q9UIL8	0.26767
54468	Q2Q067	Q9Y294	0.98793
54469	Q2Q067	Q9HA77	0.22897
54470	Q2Q067	P61758	0.58553
54471	Q2Q067	Q96PU4	0.30404
54472	Q2Q067	Q9H4B6	0.6052
54473	Q2Q067	Q969V6	1.3959
54474	Q2Q067	Q9UHD4	0.36735
54475	Q2Q067	Q6KB66	0.16378
54476	Q2Q067	Q8TED0	0.2608
54477	Q2Q067	Q658K8	0.25783
54478	Q2Q067	Q9P016	0.28009
54479	Q2Q067	Q5T230	1.238
54480	Q2Q067	Q9H2L5	0.35152
54481	Q2Q067	Q99700	0.7581
54482	Q2Q067	Q6ZNM6	0.39825
54483	Q2Q067	O15182	0.29326
54484	Q2Q067	O43633	0.0924
54485	Q2Q067	Q6NZ67	0.65427
54486	Q2Q067	O14964	0.59632
54487	Q2Q067	O95125	0.10046
54488	Q2Q067	Q9P1Z2	0.80685
54489	Q2Q067	A6NIN4	0.19215
54490	Q2Q067	Q96PK6	0.6901
54491	Q2Q067	Q9Y2S6	0.19215
54492	Q2Q067	Q9UIF9	0.66537

Q2Q067	P58304	0.2505
Q2Q067	O60583	1.0344
Q2Q067	Q8WVZ9	0.37541
Q2Q067	Q3LI59	0.19215
Q2Q067	Q8TCC3	0.050442
Q2Q067	Q8NAQ8	0.76219
Q2Q067	Q8IYE0	0.40359
Q2Q067	Q9Y4P8	0.86574
Q2Q067	P00338	0.44348
Q2Q067	O43765	0.56998
Q2Q067	Q68CP9	0.49369
Q2Q067	Q6ZVN8	0.16496
Q2Q067	O14737	0.20547
Q2Q067	Q5JSJ4	0.69602
Q2Q067	Q6ZV60	0.19215
Q2Q067	Q96D15	0.23084
Q2Q067	Q9GZN7	0.23416
Q2Q067	Q8IXT1	0.14088
Q2Q067	Q7Z6M4	0.3396
Q2Q067	Q9NUS5	0.49249
Q2Q067	A6NHY6	0.0020931
Q2Q067	Q7Z4H3	0.17558
Q2Q067	A6NCS6	0.16295
Q2Q067	Q9BVS5	0.23308
Q2Q067	P24863	1.1027
Q2Q067	Q9H808	0.54509
Q2Q067	P27986	0.56117
Q2Q067	Q14146	0.19215
Q2Q067	Q16385	0.77981
Q2Q067	Q8NCU4	0.10943
Q2Q067	O94916	0.1921
Q2Q067	Q5T2W1	0.38598
Q2Q067	P17019	0.24839
Q2Q067	Q05086	0.36811
Q2Q067	Q9BXS6	0.35465
Q2Q067	Q0VFZ6	0.29573
Q2Q067	P0CG31	0.10282
Q2Q067	Q9NP71	0.57066
Q2Q067	P16455	0.15841
Q2Q067	Q9UKF6	0.72295
Q2Q067	P28070	0.48151
Q2Q067	P43897	0.5868
Q2Q067	Q8IXM7	0.030976
Q2Q067	Q9NPJ4	1.216
Q2Q067	Q8N693	0.45786
Q2Q067	Q8NDZ2	0.093968
Q2Q067	Q9Y2G2	0.46379
Q2Q067	Q9H9L7	0.20402
Q2Q067	Q96M85	0.19215
Q2Q067	Q14244	0.36124
Q2Q067	O60682	0.33408
Q2Q067	Q9BYH8	0.29711
Q2Q067	Q6ZUC2	0.037726
Q2Q067	Q8TAQ2	1.3657
Q2Q067	Q86WJ1	0.26769
Q2Q067	Q01831	0.6541
Q2Q067	Q52MB2	0.54194
Q2Q067	Q8WU49	0.19215
Q2Q067	Q3LHN2	0.19215
Q2Q067	Q9NYF3	0.18893
Q2Q067	P39019	0.89125
Q2Q067	Q32MH5	0.19038
Q2Q067	Q92844	0.43515
Q2Q067	O75558	0.49308
Q2Q067	P0C7X2	0.29369
Q2Q067	B1ANY3	0.12253
Q2Q067	Q0VG73	0.19215
Q2Q067	Q14493	0.90013
Q2Q067	Q4LEZ3	0.53771
Q2Q067	Q96RE7	0.97958
Q2Q067	Q8WUM0	0.71047
Q2Q067	Q8NEG4	0.18146
Q2Q067	P62256	0.1399
Q2Q067	O96018	0.40012
Q2Q067	Q9BQE4	0.30448
Q2Q067	Q92793	0.69902

Q2Q067	Q86U70	0.22923
Q2Q067	Q12894	0.37525
Q2Q067	Q6ZTC4	0.19215
Q2Q067	P59052	0.21828
Q2Q067	Q8WYK2	0.34507
Q2Q067	Q6ZSA8	0.19215
Q2Q067	Q9Y2G7	0.27402
Q2Q067	Q14582	0.27371
Q2Q067	Q9BW92	0.14821
Q2Q067	Q5T8I3	0.036233
Q2Q067	Q8IYX0	0.22687
Q2Q067	Q6ZS94	0.19215
Q2Q067	Q70IA6	0.33544
Q2Q067	Q92734	0.086426
Q2Q067	Q9NY27	0.83126
Q2Q067	Q93052	0.28159
Q2Q067	Q01860	0.71188
Q2Q067	Q9H204	1.1366
Q2Q067	P18848	1.1446
Q2Q067	Q5VZT2	0.079214
Q2Q067	Q69YH5	0.70287
Q2Q067	O94817	0.47665
Q2Q067	Q5TGS1	0.49191
Q2Q067	Q6ZR54	0.19215
Q2Q067	Q9UJW7	0.087195
Q2Q067	Q9NWZ8	0.77708
Q2Q067	Q6IEG0	0.28458
Q2Q067	Q96MF7	0.50143
Q2Q067	Q9NSC2	0.30573
Q2Q067	P28066	0.64272
Q2Q067	P68036	0.70287
Q2Q067	P63000	0.53952
Q2Q067	Q9BZR8	0.40255
Q2Q067	A8MU93	0.29573
Q2Q067	Q5T749	0.36489
Q2Q067	Q03393	0.052168
Q2Q067	O00165	0.70942
Q2Q067	B3EWG5	0.29573
Q2Q067	Q6DHV5	0.040826
Q2Q067	Q5T8I9	0.060217
Q2Q067	Q92670	0.11083
Q2Q067	Q2M2I5	0.24903
Q2Q067	B1AK76	0.45637
Q2Q067	Q6ZRQ5	0.5726
Q2Q067	P41250	0.083964
Q2Q067	Q8NA57	0.38348
Q2Q067	Q9BT78	0.97885
Q2Q067	Q9UGJ1	0.23823
Q2Q067	Q5HYN5	0.068648
Q2Q067	Q8IW50	0.29573
Q2Q067	Q5C9Z4	0.68611
Q2Q067	P0CG29	0.2324
Q2Q067	P35579	0.76187
Q2Q067	Q9P287	0.63694
Q2Q067	P82664	0.51696
Q2Q067	Q8IXQ6	0.8177
Q2Q067	O95613	0.74518
Q2Q067	Q9UL18	1.0094
Q2Q067	P35606	0.3668
Q2Q067	Q14683	0.40589
Q2Q067	Q8WVM7	0.74085
Q2Q067	P52736	0.59639
Q2Q067	Q8N9G6	0.38062
Q2Q067	Q96FJ2	0.73009
Q2Q067	P61353	0.68644
Q2Q067	Q92845	0.21744
Q2Q067	Q8N3Z6	0.219
Q2Q067	Q01826	0.78354
Q2Q067	Q5VWT5	0.29297
Q2Q067	Q8WTW3	0.43424
Q2Q067	P63272	0.98552
Q2Q067	Q96NJ3	0.092025
Q2Q067	Q96DM3	0.12918
Q2Q067	Q9BTD1	0.11911
Q2Q067	O75800	0.37316
Q2Q067	Q6ZRH4	0.7143

Q2Q067	P14324	0.30544
Q2Q067	Q86XN6	0.16407
Q2Q067	P0C5J1	0.0062999
Q2Q067	O15371	0.66708
Q2Q067	Q63HN1	0.041424
Q2Q067	Q8N5L8	0.59554
Q2Q067	Q04637	0.56617
Q2Q067	Q9BZQ2	0.20753
Q2Q067	Q49MG5	0.81452
Q2Q067	Q96L21	0.22517
Q2Q067	P21675	0.40917
Q2Q067	Q8WWB5	0.37929
Q2Q067	Q9BRU9	0.27054
Q2Q067	P30305	0.34443
Q2Q067	Q9H607	0.20751
Q2Q067	Q9NXK8	0.54027
Q2Q067	Q96FN9	0.2423
Q2Q067	O14519	0.16694
Q2Q067	Q9GZX5	0.78838
Q2Q067	Q8N5J2	0.64851
Q2Q067	Q3SYG4	0.3028
Q2Q067	Q9Y375	0.55492
Q2Q067	Q86WI3	0.49128
Q2Q067	P43487	0.2174
Q2Q067	Q9NP87	0.027903
Q2Q067	Q9P1F3	0.36214
Q2Q067	P53803	0.43971
Q2Q067	Q5TBE3	0.17069
Q2Q067	Q9H8G1	0.24672
Q2Q067	Q9H9A7	0.56702
Q2Q067	P20810	0.10732
Q2Q067	Q96SW2	0.5059
Q2Q067	Q9H3F6	0.46195
Q2Q067	Q96AN2	0.13154
Q2Q067	Q8N4J0	0.41546
Q2Q067	Q7Z745	0.24419
Q2Q067	Q9H2F5	0.5648
Q2Q067	Q15007	0.38473
Q2Q067	P13994	0.37883
Q2Q067	Q49AM1	0.37468
Q2Q067	Q5T5N4	0.011803
Q2Q067	Q9GZU8	0.20538
Q2Q067	Q96MN9	0.57845
Q2Q067	Q9BRQ3	0.37843
Q2Q067	Q9BQ52	0.13114
Q2Q067	Q99436	0.7081
Q2Q067	Q15853	0.67957
Q2Q067	Q9GZV4	0.6574
Q2Q067	Q9BZF9	0.52962
Q2Q067	P24941	0.60816
Q2Q067	Q5T319	0.19215
Q2Q067	Q8IWN6	0.17426
Q2Q067	Q96MU7	0.69944
Q2Q067	Q96NA2	0.54692
Q2Q067	Q9NPA8	0.38862
Q2Q067	Q96RN5	1.2481
Q2Q067	Q8TAA3	0.33536
Q2Q067	Q9BQA5	0.82823
Q2Q067	Q8N7B7	0.047422
Q2Q067	P13010	0.3567
Q2Q067	Q8IY92	0.38211
Q2Q067	Q9H875	0.23232
Q2Q067	Q14241	0.24567
Q2Q067	Q7L1V2	0.50098
Q2Q067	Q9NUQ3	0.62219
Q2Q067	Q9BZ01	0.10585
Q2Q067	P78347	0.55159
Q2Q067	Q9UKA2	0.40312
Q2Q067	P49006	0.43756
Q2Q067	P17025	0.097501
Q2Q067	P30047	0.698
Q2Q067	O43592	0.28929
Q2Q067	Q6NSH3	0.28186
Q2Q067	Q9H489	0.16209
Q2Q067	Q9UKK3	0.6119
Q2Q067	P49321	0.39458

Q2Q067	P07320	0.43779
Q2Q067	P84101	0.54319
Q2Q067	P62266	0.98376
Q2Q067	Q9BU64	0.16343
Q2Q067	Q9BRT9	0.2581
Q2Q067	Q96M91	0.49951
Q2Q067	Q8N4C6	0.17376
Q2Q067	P42677	0.88927
Q2Q067	Q9Y3U8	0.87684
Q2Q067	O60508	0.34093
Q2Q067	Q6NUI1	0.40369
Q2Q067	P0C5K6	0.19215
Q2Q067	Q8IYJ2	0.34262
Q2Q067	A6NK75	0.15308
Q2Q067	Q9H0G5	0.59475
Q2Q067	Q9UJX6	0.55799
Q2Q067	Q8WUX9	0.47274
Q2Q067	Q494X1	0.097326
Q2Q067	P62306	0.36035
Q2Q067	Q9NRD1	0.12874
Q2Q067	Q5BJE1	0.091411
Q2Q067	A1A4G5	0.19215
Q2Q067	P35232	0.5356
Q2Q067	P63220	0.60047
Q2Q067	P43359	0.5323
Q2Q067	Q9UBD0	1.1534
Q2Q067	P49593	0.21652
Q2Q067	Q9H840	0.91039
Q2Q067	Q5T9C2	0.29573
Q2Q067	Q538Z0	0.19215
Q2Q067	P0C838	0.16605
Q2Q067	Q15652	0.42512
Q2Q067	P62841	0.76298
Q2Q067	O60869	0.64648
Q2Q067	Q3SY46	0.18753
Q2Q067	Q8WYQ3	0.30457
Q2Q067	P61962	0.64054
Q2Q067	Q8IV03	0.54663
Q2Q067	O60518	0.92214
Q2Q067	Q9HBD4	0.63303
Q2Q067	O14512	0.40695
Q2Q067	Q8NE71	0.30646
Q2Q067	Q8TDP1	0.26412
Q2Q067	O95229	0.76205
Q2Q067	Q9UBX3	0.3044
Q2Q067	Q9NWW7	0.34234
Q2Q067	Q9UNZ5	0.29573
Q2Q067	Q96CN0	0.19215
Q2Q067	Q8N587	0.19721
Q2Q067	Q8WXS3	0.54261
Q2Q067	Q9Y5B9	0.25371
Q2Q067	Q17RB0	0.32676
Q2Q067	Q8TC56	0.63352
Q2Q067	Q8N7Q3	0.16587
Q2Q067	Q9Y678	0.56898
Q2Q067	Q7Z398	0.090424
Q2Q067	P83881	0.79659
Q2Q067	Q8N6I1	0.48818
Q2Q067	P06132	0.28987
Q2Q067	P62987	0.34173
Q2Q067	Q9UHZ2	0.19215
Q2Q067	Q9BYR3	0.34715
Q2Q067	Q75QN2	0.57387
Q2Q067	Q92466	0.62869
Q2Q067	P13682	0.13461
Q2Q067	Q8IYI8	0.15228
Q2Q067	P26651	0.70354
Q2Q067	Q86V71	0.17393
Q2Q067	Q04743	0.29077
Q2Q067	Q53T94	0.39009
Q2Q067	O43242	0.24416
Q2Q067	Q09666	0.6659
Q2Q067	Q2VPJ9	0.29573
Q2Q067	Q9H4A5	0.42637
Q2Q067	P06730	0.59023
Q2Q067	P61583	0.56753

Q2Q067	P45973	0.72647
Q2Q067	P07476	0.23421
Q2Q067	Q8NCR3	0.05824
Q2Q067	Q8TEK3	0.33646
Q2Q067	P52744	0.12365
Q2Q067	Q9BX66	0.34532
Q2Q067	Q6ZRT6	0.29573
Q2Q067	Q15121	0.41476
Q2Q067	Q99689	0.084184
Q2Q067	P55327	0.30757
Q2Q067	P08670	0.15912
Q2Q067	Q14188	0.75905
Q2Q067	P60411	0.34715
Q2Q067	A6NE82	0.083005
Q2Q067	Q9UGR2	0.3848
Q2Q067	Q02577	0.60493
Q2Q067	Q9BWU0	0.50521
Q2Q067	P58505	0.15831
Q2Q067	Q9NVV2	0.19215
Q2Q067	Q86Y01	0.5697
Q2Q067	Q93075	0.36219
Q2Q067	Q96MC9	0.57527
Q2Q067	Q6PII3	0.18937
Q2Q067	A6NN14	0.16589
Q2Q067	Q8N9M1	0.020318
Q2Q067	Q96JL9	0.093779
Q2Q067	P55771	0.8544
Q2Q067	P51116	0.54346
Q2Q067	Q9UNQ2	0.23005
Q2Q067	P28370	0.40773
Q2Q067	Q96E16	0.42683
Q2Q067	O14777	0.58838
Q2Q067	Q9BYS1	0.18143
Q2Q067	Q9UKG1	0.35594
Q2Q067	Q9H8H0	0.15082
Q2Q067	P50222	0.61971
Q2Q067	Q16537	0.26328
Q2Q067	Q86YV9	0.45174
Q2Q067	Q9BYR7	0.612
Q2Q067	Q9NVM9	1.0684
Q2Q067	Q9H974	0.65431
Q2Q067	Q16576	0.40926
Q2Q067	Q676U5	0.5938
Q2Q067	Q562F6	0.75083
Q2Q067	P0CB33	0.21506
Q2Q067	Q8IXR5	0.13528
Q2Q067	Q5T4T6	0.63267
Q2Q067	Q6ZNH5	0.11824
Q2Q067	Q9H040	0.4778
Q2Q067	Q9UHA2	0.14678
Q2Q067	Q8WWR8	0.062727
Q2Q067	Q96A44	0.4314
Q2Q067	Q9BVN2	0.50579
Q2Q067	K9M1U5	0.19215
Q2Q067	Q8N7W2	0.30398
Q2Q067	Q9NWS9	0.058453
Q2Q067	Q8TDR0	0.44569
Q2Q067	P60006	0.2755
Q2Q067	Q3ZCX4	0.12173
Q2Q067	Q8IV54	0.18774
Q2Q067	Q9UJ94	0.19215
Q2Q067	Q9BX82	0.22861
Q2Q067	Q03060	0.94836
Q2Q067	O75953	0.76677
Q2Q067	Q8N7M0	0.068878
Q2Q067	P59942	0.31749
Q2Q067	Q9Y2W2	0.70292
Q2Q067	P0DJG4	0.44149
Q2Q067	Q5T870	0.33506
Q2Q067	Q9UHR6	0.53273
Q2Q067	Q7Z6J9	0.39165
Q2Q067	Q9NTM9	0.1805
Q2Q067	Q01850	0.85476
Q2Q067	O43402	0.42689
Q2Q067	Q8NC38	0.028349
Q2Q067	Q9UGY1	0.1644

Q2Q067	P49589	0.23058
Q2Q067	Q8NA47	0.29573
Q2Q067	Q9H7T9	0.66026
Q2Q067	Q9UBL3	0.2639
Q2Q067	Q6STE5	0.72873
Q2Q067	O95453	0.4386
Q2Q067	P30260	1.0912
Q2Q067	Q14154	0.46376
Q2Q067	Q6PI77	0.30886
Q2Q067	Q9BZG8	0.17656
Q2Q067	Q5T7W7	0.29573
Q2Q067	Q9Y3B9	0.3108
Q2Q067	Q8N128	0.19215
Q2Q067	Q86XK2	0.68917
Q2Q067	Q13404	1.1048
Q2Q067	P49721	0.46321
Q2Q067	O14558	0.75457
Q2Q067	Q9HCM9	0.43663
Q2Q067	P42226	0.81651
Q2Q067	P49406	0.44787
Q2Q067	P04259	0.16907
Q2Q067	Q9P0T4	0.15194
Q2Q067	Q9BSY4	0.22513
Q2Q067	P80297	0.1848
Q2Q067	B2CW77	0.19215
Q2Q067	Q8IY57	1.0804
Q2Q067	Q96PT4	0.20973
Q2Q067	Q5VWG9	0.75763
Q2Q067	A7E2U8	0.16298
Q2Q067	Q5U5X8	0.44143
Q2Q067	Q9BPU9	0.11821
Q2Q067	Q5H9L2	1.0622
Q2Q067	Q9UPT5	0.51375
Q2Q067	Q8NC51	0.68607
Q2Q067	Q5HY98	0.20168
Q2Q067	Q04837	0.50628
Q2Q067	Q86VS8	0.34707
Q2Q067	Q5W5X9	0.1308
Q2Q067	Q9Y316	0.22173
Q2Q067	Q6B0B8	0.20513
Q2Q067	Q9BRQ4	0.18858
Q2Q067	Q9BRJ6	0.28759
Q2Q067	P13051	0.6816
Q2Q067	O75792	0.62546
Q2Q067	Q9Y2W1	0.49282
Q2Q067	P11802	0.68089
Q2Q067	Q8TBN0	0.23226
Q2Q067	Q9UGB4	0.19215
Q2Q067	Q16612	0.12099
Q2Q067	Q969E8	0.61461
Q2Q067	A6NJ88	0.44763
Q2Q067	Q9UIF8	0.83615
Q2Q067	Q96LT6	0.062368
Q2Q067	Q96IZ0	0.75083
Q2Q067	Q9Y296	0.21109
Q2Q067	Q9HAT8	0.58735
Q2Q067	Q9UH90	0.26638
Q2Q067	Q92597	0.51709
Q2Q067	P0CG42	0.19215
Q2Q067	Q15637	0.32376
Q2Q067	Q9UL03	0.6471
Q2Q067	P23497	0.95366
Q2Q067	O95707	0.62866
Q2Q067	Q6ZVT6	0.29799
Q2Q067	A6NLF2	0.24331
Q2Q067	P02489	0.27733
Q2Q067	A6NJ46	0.27517
Q2Q067	P78371	0.54367
Q2Q067	Q8NA19	0.32208
Q2Q067	Q75VX8	0.48064
Q2Q067	P09496	0.25578
Q2Q067	Q8NEK5	0.10714
Q2Q067	Q9Y421	0.28802
Q2Q067	Q6TDU7	0.21694
Q2Q067	Q9BYG4	0.64686
Q2Q067	Q9HBX3	0.19215

Q2Q067	Q9Y692	0.7094
Q2Q067	P55198	0.86945
Q2Q067	Q96BN8	0.0088176
Q2Q067	O60756	0.19215
Q2Q067	Q71SY5	1.0545
Q2Q067	Q6XYB7	0.32768
Q2Q067	Q9BPX3	0.84697
Q2Q067	P14672	0.64871
Q2Q067	Q9Y535	0.24506
Q2Q067	Q66K74	0.18112
Q2Q067	Q6X4T0	0.045008
Q2Q067	Q5UE93	0.48785
Q2Q067	Q8NDZ0	0.066494
Q2Q067	O15063	0.12426
Q2Q067	Q969X6	1.2086
Q2Q067	Q9Y252	0.38865
Q2Q067	Q86Z20	0.54696
Q2Q067	P46527	0.35675
Q2Q067	Q6ICG7	0.19215
Q2Q067	Q9P015	0.29141
Q2Q067	Q8WXI9	0.47763
Q2Q067	P23284	0.3348
Q2Q067	P14653	0.34715
Q2Q067	P49720	0.4633
Q2Q067	O00463	0.28027
Q2Q067	Q13952	0.72246
Q2Q067	P15923	0.60179
Q2Q067	P10242	0.97441
Q2Q067	Q8NB12	0.26907
Q2Q067	Q6P9B6	0.28783
Q2Q067	O43309	0.1126
Q2Q067	O14978	0.37704
Q2Q067	Q9P2W9	0.12791
Q2Q067	O60422	0.32817
Q2Q067	P35637	0.36892
Q2Q067	Q9NRR5	0.69817
Q2Q067	O60810	0.34439
Q2Q067	Q6ZN79	0.20479
Q2Q067	Q99717	0.43825
Q2Q067	P06733	0.90363
Q2Q067	A7E2V4	0.7741
Q2Q067	Q15834	1.0846
Q2Q067	P51580	0.048096
Q2Q067	Q9H299	0.04381
Q2Q067	Q8TDN4	0.56222
Q2Q067	Q9BXF6	0.54172
Q2Q067	P15407	0.70602
Q2Q067	Q9ULK2	0.41879
Q2Q067	Q9Y675	0.35987
Q2Q067	Q8NCD3	0.48788
Q2Q067	Q9H7R5	0.17365
Q2Q067	Q8IUC3	0.41492
Q2Q067	Q9UBI1	0.28783
Q2Q067	Q6ZTI6	0.43678
Q2Q067	Q02040	0.23213
Q2Q067	P22626	0.50288
Q2Q067	Q12857	0.34044
Q2Q067	A6NFQ2	0.21043
Q2Q067	Q9UH77	0.56809
Q2Q067	Q96DZ1	0.23454
Q2Q067	P27469	0.68347
Q2Q067	Q9C0F3	0.29295
Q2Q067	Q9BXR0	0.66528
Q2Q067	P78318	0.45704
Q2Q067	Q69YN4	0.14045
Q2Q067	P21673	0.24866
Q2Q067	O60870	0.36278
Q2Q067	Q9BYU5	0.17348
Q2Q067	Q9BTL3	0.5188
Q2Q067	P02533	0.84232
Q2Q067	Q8N972	0.16182
Q2Q067	C9JFL3	0.19215
Q2Q067	Q01546	0.23657
Q2Q067	Q6ZYL4	0.5539
Q2Q067	Q96CS2	0.45603
Q2Q067	P11172	0.19175

Q2Q067	Q8NHZ7	0.27948
Q2Q067	Q8ND76	0.52785
Q2Q067	Q13568	0.99226
Q2Q067	Q8TAG6	0.28217
Q2Q067	A6NI87	0.24415
Q2Q067	Q0VD86	0.32989
Q2Q067	Q3B7T1	0.39593
Q2Q067	Q13490	0.4373
Q2Q067	Q12816	0.25165
Q2Q067	Q9H0M5	0.087195
Q2Q067	Q9UL40	0.58706
Q2Q067	Q9UHQ1	0.39745
Q2Q067	Q9BYQ4	0.34715
Q2Q067	O60809	0.23856
Q2Q067	Q9UMX0	0.74746
Q2Q067	Q8N3U4	0.64546
Q2Q067	Q6W349	0.27117
Q2Q067	Q96BN2	0.55997
Q2Q067	P51808	0.24236
Q2Q067	Q6PJG6	0.5463
Q2Q067	A6NFY7	0.29289
Q2Q067	Q7L190	0.75962
Q2Q067	Q5W0V3	0.39515
Q2Q067	Q13765	1.1259
Q2Q067	C9JLW8	0.39201
Q2Q067	P50914	1.0505
Q2Q067	P46776	1.0382
Q2Q067	Q9BWH6	0.35453
Q2Q067	Q6TFL3	0.06639
Q2Q067	Q8N8P6	0.28355
Q2Q067	Q9Y281	0.46094
Q2Q067	Q9BUH8	0.35983
Q2Q067	O15287	0.91508
Q2Q067	Q2NL68	0.29573
Q2Q067	Q9NVV4	0.45273
Q2Q067	Q8NDN9	0.57472
Q2Q067	Q6IPX3	0.48462
Q2Q067	Q8WVK2	0.34939
Q2Q067	Q75MW2	0.40129
Q2Q067	P50452	0.63006
Q2Q067	P27816	0.37839
Q2Q067	Q7L4I2	0.86202
Q2Q067	P18583	0.32302
Q2Q067	P0CL80	0.019044
Q2Q067	Q9ULC4	0.34109
Q2Q067	Q7Z417	0.75561
Q2Q067	Q6R327	0.10297
Q2Q067	A0JNW5	0.27888
Q2Q067	Q86WH2	0.095084
Q2Q067	Q96M29	0.27079
Q2Q067	O75376	0.41585
Q2Q067	Q6ZN08	0.20699
Q2Q067	Q13042	1.2279
Q2Q067	Q9HA58	0.19215
Q2Q067	P35900	0.80198
Q2Q067	Q96KT6	0.19215
Q2Q067	P57086	0.52078
Q2Q067	Q5JRK9	0.56155
Q2Q067	Q8NHY6	0.17262
Q2Q067	Q96EF6	0.28838
Q2Q067	Q9NNX9	0.50252
Q2Q067	P31689	0.21633
Q2Q067	Q9H7H0	0.63713
Q2Q067	Q9UK39	0.47893
Q2Q067	Q6ZTN6	0.25703
Q2Q067	Q8TBZ8	0.11921
Q2Q067	Q16384	0.26023
Q2Q067	P27815	0.27142
Q2Q067	P61586	0.086378
Q2Q067	Q86VN1	0.4456
Q2Q067	Q8ND04	0.60077
Q2Q067	Q96CM3	0.35441
Q2Q067	Q9UIE0	0.10351
Q2Q067	Q5HYC2	0.013015
Q2Q067	Q92556	0.21248
Q2Q067	Q86WV5	0.28751

Q2Q067	Q16637	0.91809
Q2Q067	Q9Y468	0.89613
Q2Q067	Q92917	0.79042
Q2Q067	Q96PG8	0.19215
Q2Q067	Q15651	0.59073
Q2Q067	Q3LHN1	0.19215
Q2Q067	Q9Y222	0.95215
Q2Q067	Q96DV4	0.18823
Q2Q067	Q8IXJ9	0.72893
Q2Q067	Q68DY9	0.11859
Q2Q067	P11940	0.41331
Q2Q067	Q5T5S1	0.26593
Q2Q067	Q5VIY5	0.22332
Q2Q067	P0CG24	0.096646
Q2Q067	Q14331	0.21951
Q2Q067	O75290	0.21203
Q2Q067	Q96AJ1	0.66981
Q2Q067	Q3T8J9	0.66073
Q2Q067	Q5VT40	0.14645
Q2Q067	H0Y354	0.2014
Q2Q067	Q9H5I1	0.52917
Q2Q067	Q15691	0.59811
Q2Q067	Q9UMX1	0.38377
Q2Q067	P22681	0.32162
Q2Q067	Q9H081	0.69508
Q2Q067	O60888	0.32529
Q2Q067	Q96N11	0.080189
Q2Q067	O95905	0.76119
Q2Q067	Q8WXF1	0.91836
Q2Q067	Q9NP77	0.17929
Q2Q067	Q99814	0.39116
Q2Q067	P82909	0.29299
Q2Q067	Q8WVK7	0.5628
Q2Q067	Q6P1K8	0.37742
Q2Q067	Q9H7E2	0.40074
Q2Q067	Q8WXB4	0.29295
Q2Q067	P60510	0.79182
Q2Q067	Q8IYL3	0.4501
Q2Q067	Q14689	0.2891
Q2Q067	Q5XG85	0.19215
Q2Q067	P49711	0.23837
Q2Q067	P40425	0.68399
Q2Q067	H3BN30	0.19215
Q2Q067	A2A288	0.27756
Q2Q067	P26371	0.19215
Q2Q067	Q9NT62	0.4061
Q2Q067	Q9GZQ3	0.58655
Q2Q067	Q8N5A5	0.82604
Q2Q067	Q9H3M7	0.793
Q2Q067	O15111	0.58744
Q2Q067	Q93062	0.75782
Q2Q067	Q969E4	0.39593
Q2Q067	P35658	1.0644
Q2Q067	P63162	0.62351
Q2Q067	Q9Y250	0.95669
Q2Q067	O75368	0.091338
Q2Q067	Q16649	0.19221
Q2Q067	Q86UD1	0.08103
Q2Q067	P83916	0.65607
Q2Q067	Q6ZQY7	0.21688
Q2Q067	P05423	0.045505
Q2Q067	Q6NXR4	0.071917
Q2Q067	Q5T7N7	0.19215
Q2Q067	Q9NVV9	1.0089
Q2Q067	Q53ET0	0.95107
Q2Q067	Q8TAP9	1.3725
Q2Q067	Q8IW35	1.1355
Q2Q067	Q9P0V3	0.5366
Q2Q067	P62854	0.89742
Q2Q067	Q16352	0.15904
Q2Q067	P17480	1.0261
Q2Q067	Q96KF2	0.19215
Q2Q067	Q9BQ66	0.34715
Q2Q067	Q92841	0.56233
Q2Q067	Q9BRJ7	0.33213
Q2Q067	Q9HAW4	0.41415

55177	Q2Q067	Q9NZM4	0.68842
55178	Q2Q067	O76011	0.20138
55179	Q2Q067	P23297	0.50745
55180	Q2Q067	Q96ED9	0.40636
55181	Q2Q067	Q5T1J5	0.46303
55182	Q2Q067	O43324	1.1875
55183	Q2Q067	Q9Y2Z0	0.40817
55184	Q2Q067	P62308	0.76738
55185	Q2Q067	Q00987	0.24833
55186	Q2Q067	P05783	1.3037
55187	Q2Q067	Q9HC16	0.082948
55188	Q2Q067	P59037	0.47948
55189	Q2Q067	P49247	0.32814
55190	Q2Q067	Q96A26	0.060523
55191	Q2Q067	Q9UMS4	0.58015
55192	Q2Q067	Q8TCZ7	0.3653
55193	Q2Q067	Q9P2J5	0.2541
55194	Q2Q067	Q0VG99	0.25704
55195	Q2Q067	Q9BWM5	0.10781
55196	Q2Q067	Q7Z4H9	0.22946
55197	Q2Q067	Q9BS18	0.36764
55198	Q2Q067	B3EWG3	0.29573
55199	Q2Q067	Q8TF32	0.2627
55200	Q2Q067	Q96GM8	0.16332
55201	Q2Q067	P09001	0.27695
55202	Q2Q067	Q13867	0.31023
55203	Q2Q067	P62753	1.0004
55204	Q2Q067	Q86VP3	0.1701
55205	Q2Q067	Q9BTE3	0.64252
55206	Q2Q067	Q16600	0.11257
55207	Q2Q067	Q2M3X9	0.10324
55208	Q2Q067	Q14602	0.25221
55209	Q2Q067	P08397	0.082511
55210	Q2Q067	Q9NTK5	0.36121
55211	Q2Q067	O14595	0.18238
55212	Q2Q067	P52737	0.58783
55213	Q2Q067	O14893	0.68045
55214	Q2Q067	O15083	0.48996
55215	Q2Q067	Q6ZRP5	0.19215
55216	Q2Q067	Q6PI98	0.56735
55217	Q2Q067	Q9NR46	0.36835
55218	Q2Q067	Q6ZV50	0.30214
55219	Q2Q067	Q96JK9	0.94709
55220	Q2Q067	Q6P1L5	0.66876
55221	Q2Q067	Q7Z794	0.25607
55222	Q2Q067	Q9HCI5	0.58736
55223	Q2Q067	Q9BX26	0.70346
55224	Q2Q067	Q14781	0.76031
55225	Q2Q067	Q9UQR1	0.81979
55226	Q2Q067	P61578	0.36503
55227	Q2Q067	Q9NY74	0.51037
55228	Q2Q067	Q9NXX6	0.36585
55229	Q2Q067	Q8N668	0.76974
55230	Q2Q067	A6NEQ2	0.19215
55231	Q2Q067	Q3LI66	0.19215
55232	Q2Q067	Q96HI0	0.45788
55233	Q2Q067	Q96QD9	0.74944
55234	Q2Q067	Q6ZVC0	0.41813
55235	Q2Q067	Q8TAF7	0.22688
55236	Q2Q067	A6NI79	0.44645
55237	Q2Q067	P05388	1.3866
55238	Q2Q067	Q5XKE5	0.16378
55239	Q2Q067	Q8N7K0	0.16182
55240	Q2Q067	Q86UT8	0.63552
55241	Q2Q067	Q13023	0.57576
55242	Q2Q067	Q9H9F9	1.1446
55243	Q2Q067	Q8IUF8	0.41474
55244	Q2Q067	Q8NEZ5	0.76375
55245	Q2Q067	Q13542	0.22574
55246	Q2Q067	P49754	0.42108
55247	Q2Q067	Q14207	0.93013
55248	Q2Q067	O14802	0.059031
55249	Q2Q067	Q9H3H3	0.17807
55250	Q2Q067	Q9H7D7	0.32989
55251	Q2Q067	Q86YD1	0.43789
55252	Q2Q067	Q8NFH5	0.58316

Q2Q067	Q15776	0.42224
Q2Q067	Q6NSW5	0.59569
Q2Q067	Q6MZT1	0.37048
Q2Q067	Q66K89	0.80655
Q2Q067	Q8IYD8	0.2662
Q2Q067	Q9P031	1.0622
Q2Q067	P0C7V0	0.19215
Q2Q067	Q15154	0.5453
Q2Q067	Q6Q759	0.3409
Q2Q067	Q08AG5	0.28629
Q2Q067	Q14129	0.21494
Q2Q067	Q53QV2	0.34416
Q2Q067	P52294	1.1063
Q2Q067	Q7LBC6	0.13449
Q2Q067	Q99459	1.1226
Q2Q067	Q6PCE3	0.21723
Q2Q067	Q96IT6	0.19215
Q2Q067	Q8N983	0.28685
Q2Q067	P35711	0.99574
Q2Q067	Q9UBY9	0.97029
Q2Q067	O14599	0.42055
Q2Q067	Q12874	0.44296
Q2Q067	Q9NQR7	0.19215
Q2Q067	Q9NTI7	0.27612
Q2Q067	P42224	0.25809
Q2Q067	O60671	0.50231
Q2Q067	P0C879	0.19215
Q2Q067	Q5VT28	0.19025
Q2Q067	Q15436	0.67823
Q2Q067	Q96LP2	0.0063133
Q2Q067	Q6ZQT7	0.19215
Q2Q067	Q9H9E3	0.40446
Q2Q067	O95190	0.3724
Q2Q067	Q5RHP9	0.15076
Q2Q067	Q96IU4	0.34381
Q2Q067	O75132	0.1794
Q2Q067	A6NKF2	0.62076
Q2Q067	Q86W11	0.080718
Q2Q067	E7EW31	0.19215
Q2Q067	P60842	0.096503
Q2Q067	Q8WYA6	0.72943
Q2Q067	O15360	0.77154
Q2Q067	P49918	0.65241
Q2Q067	P0C7I6	0.41634
Q2Q067	P51522	0.10512
Q2Q067	Q96FF9	0.7783
Q2Q067	Q6L8H2	0.19215
Q2Q067	P07197	0.72757
Q2Q067	Q9NXR7	0.35979
Q2Q067	P22736	0.28716
Q2Q067	P32929	0.28033
Q2Q067	Q9BUA3	0.22848
Q2Q067	Q9NPA3	0.679
Q2Q067	Q96EY1	0.45981
Q2Q067	Q96K31	0.22027
Q2Q067	Q16836	0.54671
Q2Q067	Q5M9Q1	0.56383
Q2Q067	Q6ZN17	1.1649
Q2Q067	A6NEJ1	0.12144
Q2Q067	Q9NRR3	0.44144
Q2Q067	Q86X55	0.75837
Q2Q067	O94763	0.76772
Q2Q067	Q7Z7L8	0.5317
Q2Q067	O14598	0.43298
Q2Q067	Q8NFG4	0.66644
Q2Q067	Q8TC71	0.34074
Q2Q067	O00479	0.40838
Q2Q067	O75674	0.31065
Q2Q067	Q71F78	0.21703
Q2Q067	Q9UN30	0.29394
Q2Q067	O15205	0.49835
Q2Q067	O15037	0.25087
Q2Q067	Q96N23	0.28161
Q2Q067	Q13227	0.66652
Q2Q067	O15392	0.25893
Q2Q067	P63208	0.61478

Q2Q067	Q96BN6	0.07306
Q2Q067	Q7RTU3	0.331
Q2Q067	P18124	0.64646
Q2Q067	P62851	0.84318
Q2Q067	Q96PM9	0.29295
Q2Q067	Q96BR6	0.19239
Q2Q067	Q6L9T8	0.2014
Q2Q067	Q9Y255	0.02603
Q2Q067	Q9BTM9	0.60343
Q2Q067	Q8IYN2	0.68354
Q2Q067	Q01534	0.17836
Q2Q067	Q00839	0.32314
Q2Q067	Q9Y2V0	0.012657
Q2Q067	Q96IR2	0.17365
Q2Q067	Q6RFH8	0.84719
Q2Q067	Q06587	0.75155
Q2Q067	Q13617	0.6907
Q2Q067	Q9UI36	0.38708
Q2Q067	Q9ULM6	1.0399
Q2Q067	P25398	0.70288
Q2Q067	Q9Y6Y1	0.4808
Q2Q067	P55209	0.71127
Q2Q067	Q32NC0	0.078975
Q2Q067	Q969W8	0.19891
Q2Q067	Q96RL1	0.72257
Q2Q067	P07864	0.23751
Q2Q067	Q14442	0.085141
Q2Q067	Q6ZNA1	0.1529
Q2Q067	A7MD48	0.25021
Q2Q067	Q15056	0.30053
Q2Q067	A6NKH3	0.22158
Q2Q067	Q9H0I3	0.40058
Q2Q067	Q15361	0.75669
Q2Q067	P43355	1.0298
Q2Q067	Q9HCM7	0.46067
Q2Q067	Q96QE5	0.85675
Q2Q067	Q8IWE2	0.36405
Q2Q067	Q9HCS7	0.42486
Q2Q067	O95201	0.58991
Q2Q067	Q96EA4	0.70461
Q2Q067	Q1RMZ1	0.26132
Q2Q067	O43345	0.15472
Q2Q067	Q15759	0.25547
Q2Q067	Q96JJ3	0.1634
Q2Q067	Q9NYV6	0.52468
Q2Q067	Q9NQ92	0.90299
Q2Q067	O95153	0.59445
Q2Q067	Q3KP22	0.035759
Q2Q067	Q8TDD2	0.26613
Q2Q067	Q701N4	0.19215
Q2Q067	Q96GV9	0.34825
Q2Q067	Q16829	0.27641
Q2Q067	Q9UKT5	0.39402
Q2Q067	P62937	0.71116
Q2Q067	Q9BTA9	0.9345
Q2Q067	Q15797	0.2318
Q2Q067	Q9H1D9	0.52885
Q2Q067	Q5FWF6	0.22668
Q2Q067	Q9Y608	0.28344
Q2Q067	A6NNV3	0.23617
Q2Q067	P78537	0.71698
Q2Q067	Q16526	0.36035
Q2Q067	Q9Y4Z0	0.74207
Q2Q067	Q68CZ6	0.45285
Q2Q067	O60936	0.78617
Q2Q067	Q8N393	0.10262
Q2Q067	Q5TG53	0.19215
Q2Q067	Q9H633	0.21528
Q2Q067	Q9BUV0	0.29573
Q2Q067	P23409	0.082963
Q2Q067	P62877	0.57674
Q2Q067	Q9H1Y0	0.3353
Q2Q067	A8MTZ0	0.1618
Q2Q067	O43660	0.26829
Q2Q067	Q8TAL5	0.021499
Q2Q067	Q9BXY0	0.15082

Q2Q067	Q9P0W2	0.68024
Q2Q067	O75969	0.19461
Q2Q067	P23508	0.30324
Q2Q067	F8WFD2	0.18974
Q2Q067	Q14194	0.37021
Q2Q067	Q8N163	0.70197
Q2Q067	P07199	0.36696
Q2Q067	O75157	0.19596
Q2Q067	Q96HY6	0.28028
Q2Q067	Q6UXR8	0.19215
Q2Q067	Q8WTZ3	0.2176
Q2Q067	Q9NQ50	0.37344
Q2Q067	Q8NAP3	0.32935
Q2Q067	Q9BZA5	0.44921
Q2Q067	Q6P1M9	0.14781
Q2Q067	P61960	0.12104
Q2Q067	Q96ND8	0.25317
Q2Q067	Q96LW9	0.078878
Q2Q067	P62910	0.68644
Q2Q067	Q86V20	0.35815
Q2Q067	Q12830	0.93115
Q2Q067	Q3MIS6	0.23615
Q2Q067	O43708	0.42655
Q2Q067	Q5T0J7	0.15651
Q2Q067	H3BQW9	0.20873
Q2Q067	Q9UL36	0.092391
Q2Q067	Q9BT81	0.37886
Q2Q067	Q9H1E3	0.5166
Q2Q067	Q53FN2	0.081486
Q2Q067	Q06547	1.0749
Q2Q067	P02795	0.56547
Q2Q067	Q8TAQ5	0.16182
Q2Q067	Q13501	0.42554
Q2Q067	Q969T9	0.56472
Q2Q067	Q8NEZ2	0.69312
Q2Q067	O95571	0.48914
Q2Q067	Q96CG3	0.29472
Q2Q067	Q5XUX1	0.28783
Q2Q067	Q92901	0.65612
Q2Q067	Q6ZU35	0.29573
Q2Q067	A4D0Y5	0.19215
Q2Q067	Q13105	0.46045
Q2Q067	Q9Y2S0	0.088781
Q2Q067	O15446	0.15775
Q2Q067	Q9NV72	0.22615
Q2Q067	Q6ZNG1	0.17365
Q2Q067	Q9Y651	0.30116
Q2Q067	Q86TU7	0.22227
Q2Q067	Q9Y3A3	0.44873
Q2Q067	Q9BT92	0.53675
Q2Q067	P68104	0.72576
Q2Q067	Q9BYR2	0.34715
Q2Q067	Q8IYN0	0.14642
Q2Q067	P46781	1.0735
Q2Q067	P54821	0.23179
Q2Q067	Q2TAZ0	0.28783
Q2Q067	Q53HV7	0.62087
Q2Q067	Q9NZ52	0.42151
Q2Q067	Q9H0N5	0.50243
Q2Q067	Q9NYP3	0.26737
Q2Q067	P48200	0.45288
Q2Q067	Q96JC4	0.13985
Q2Q067	Q49AR2	0.10332
Q2Q067	P82673	0.03469
Q2Q067	P09234	0.54036
Q2Q067	Q9H814	0.40666
Q2Q067	O15015	0.58991
Q2Q067	Q02078	0.4685
Q2Q067	Q9NZD4	0.27078
Q2Q067	Q8IYI6	0.38485
Q2Q067	Q8IUB9	0.19215
Q2Q067	Q9GZU7	0.50024
Q2Q067	Q9BYC9	0.71324
Q2Q067	Q9UPG8	0.26702
Q2Q067	Q96LM1	0.29717
Q2Q067	Q96DN6	0.36406

Q2Q067	Q9P1T7	1.3561
Q2Q067	Q8IY81	0.67503
Q2Q067	P43360	0.37541
Q2Q067	Q14695	0.33095
Q2Q067	Q9UJW9	1.006
Q2Q067	Q9BXP5	1.0962
Q2Q067	Q7Z699	0.24346
Q2Q067	Q5W0A0	0.069344
Q2Q067	Q5T0F9	0.37026
Q2Q067	A8MU76	0.36785
Q2Q067	A6NJ08	0.023567
Q2Q067	Q96LI6	0.49272
Q2Q067	Q96LS8	0.12149
Q2Q067	O14576	0.3666
Q2Q067	P14868	0.36433
Q2Q067	P62310	0.73525
Q2Q067	O00422	1.1916
Q2Q067	P27635	0.6597
Q2Q067	Q06546	1.2638
Q2Q067	Q9Y2A4	0.24211
Q2Q067	Q6TGC4	0.1952
Q2Q067	O14879	0.67845
Q2Q067	Q07352	0.94002
Q2Q067	O75821	0.53888
Q2Q067	Q6P0N0	0.49413
Q2Q067	Q92665	0.75625
Q2Q067	P21506	0.12798
Q2Q067	Q9BRK4	0.52591
Q2Q067	Q96FF7	0.19215
Q2Q067	P40424	0.66752
Q2Q067	Q14584	0.099703
Q2Q067	Q13888	0.53202
Q2Q067	Q9Y6R6	0.16182
Q2Q067	Q6ZV56	0.19215
Q2Q067	Q5MJ10	0.40408
Q2Q067	Q13367	0.43742
Q2Q067	Q96B26	0.72226
Q2Q067	Q9BUR4	0.78256
Q2Q067	Q9H0E9	0.27776
Q2Q067	Q8NG50	0.033243
Q2Q067	P31269	0.62409
Q2Q067	Q5SRE5	0.60242
Q2Q067	Q86XU0	0.11633
Q2Q067	Q9BYT5	0.026204
Q2Q067	P53567	0.71039
Q2Q067	Q16548	0.49625
Q2Q067	Q9BSH3	0.082154
Q2Q067	Q96IU2	0.6958
Q2Q067	Q8N443	0.15958
Q2Q067	Q2M238	0.026067
Q2Q067	P17014	0.088992
Q2Q067	Q6PI97	0.019436
Q2Q067	Q8N4M7	0.55862
Q2Q067	P49715	0.26309
Q2Q067	Q969L4	0.67441
Q2Q067	Q8N6U2	0.16254
Q2Q067	Q8IYK2	0.30879
Q2Q067	Q15276	0.11491
Q2Q067	P42772	0.82255
Q2Q067	Q8N365	0.29573
Q2Q067	Q8N108	1.1031
Q2Q067	Q15750	0.53869
Q2Q067	P43364	0.81743
Q2Q067	Q5QJE6	0.58597
Q2Q067	Q8WWR9	0.13202
Q2Q067	Q9Y224	1.4946
Q2Q067	Q8IUD6	0.33425
Q2Q067	Q6A163	0.1595
Q2Q067	O95900	0.37176
Q2Q067	Q6ZMS7	0.13488
Q2Q067	Q9NZY2	0.19215
Q2Q067	Q9NPC6	0.45775
Q2Q067	Q8NFH8	0.36855
Q2Q067	P17023	0.07693
Q2Q067	Q9Y242	0.24
Q2Q067	Q537H7	0.021168

Q2Q067	O15372	0.66707
Q2Q067	O15160	0.098048
Q2Q067	Q9Y3X0	0.25461
Q2Q067	A8MXK9	0.056025
Q2Q067	Q13347	0.69547
Q2Q067	Q8NDP4	0.12171
Q2Q067	Q6ZN01	0.54853
Q2Q067	Q9H2G4	0.64407
Q2Q067	Q9UPZ3	0.29328
Q2Q067	Q96EB1	1.1189
Q2Q067	Q5VT06	0.54771
Q2Q067	O60921	0.76636
Q2Q067	P18146	0.71039
Q2Q067	Q9UMY1	0.55436
Q2Q067	P48539	0.15397
Q2Q067	P61513	0.65311
Q2Q067	O75901	0.31125
Q2Q067	P28715	0.26787
Q2Q067	Q7Z6E9	0.29485
Q2Q067	Q03112	0.24904
Q2Q067	Q96N53	0.19215
Q2Q067	Q5T6M2	0.52198
Q2Q067	P0CG20	0.094965
Q2Q067	Q15906	0.90378
Q2Q067	Q96CW6	0.4403
Q2Q067	Q9ULD2	0.28553
Q2Q067	O43261	0.28783
Q2Q067	Q15650	0.741
Q2Q067	Q52LA3	0.25483
Q2Q067	P41214	0.32308
Q2Q067	P17947	0.76782
Q2Q067	Q15428	0.27906
Q2Q067	Q9BV73	0.59325
Q2Q067	Q9NW38	0.34433
Q2Q067	P17028	0.71534
Q2Q067	Q9P1J3	0.19215
Q2Q067	Q96T21	0.33324
Q2Q067	Q76FK4	0.26013
Q2Q067	Q92966	0.5105
Q2Q067	Q9BXW9	0.75446
Q2Q067	Q9NXF7	0.24229
Q2Q067	O96028	0.26507
Q2Q067	O95478	0.27472
Q2Q067	A6NGR9	0.10872
Q2Q067	Q8IYS8	0.203
Q2Q067	P31260	0.31482
Q2Q067	Q8N9B5	0.74328
Q2Q067	O76075	0.088878
Q2Q067	Q96QA6	0.15082
Q2Q067	Q5T7N2	0.051652
Q2Q067	P32519	1.351
Q2Q067	O60477	0.46956
Q2Q067	P51946	0.58237
Q2Q067	Q9UHB4	0.69243
Q2Q067	Q8WX92	1.4633
Q2Q067	P15822	0.81374
Q2Q067	A8MVA2	0.34715
Q2Q067	Q10586	0.45169
Q2Q067	Q9UKL3	0.83173
Q2Q067	Q03936	0.22595
Q2Q067	Q00403	1.0773
Q2Q067	Q96M11	0.50313
Q2Q067	Q0VF49	0.19215
Q2Q067	Q9HBQ8	0.37471
Q2Q067	Q8WTS6	0.41774
Q2Q067	Q13064	0.1746
Q2Q067	A8MSY0	0.17929
Q2Q067	P61582	0.6269
Q2Q067	Q9BXF3	0.62569
Q2Q067	Q9H1K1	0.92173
Q2Q067	Q92764	0.12452
Q2Q067	Q14693	0.36437
Q2Q067	Q96CW5	0.854
Q2Q067	Q9Y5P3	0.34734
Q2Q067	Q5BKY9	0.29573
Q2Q067	Q9H6F5	0.27346

Q2Q067	Q6NZ36	0.18889
Q2Q067	P61581	0.48164
Q2Q067	Q9BXJ9	0.63108
Q2Q067	Q969F1	0.53813
Q2Q067	Q8N2W9	1.0436
Q2Q067	Q9Y2G0	0.13247
Q2Q067	P14625	0.11874
Q2Q067	P78543	0.64694
Q2Q067	Q9NUP9	0.19112
Q2Q067	P17024	0.11858
Q2Q067	Q7Z401	0.28495
Q2Q067	Q9BSF0	0.13376
Q2Q067	A6NF83	0.38305
Q2Q067	Q9C002	0.11333
Q2Q067	Q9UK61	0.31972
Q2Q067	Q6NV74	0.29573
Q2Q067	Q7Z5L9	0.54659
Q2Q067	O95149	0.40765
Q2Q067	Q08945	1.1419
Q2Q067	Q9Y4Y9	0.8048
Q2Q067	Q8N9T8	0.73271
Q2Q067	P62945	0.78122
Q2Q067	Q71UM5	0.59435
Q2Q067	O14490	0.18368
Q2Q067	P24539	0.21749
Q2Q067	Q00534	0.57395
Q2Q067	Q71F56	0.38241
Q2Q067	Q14738	0.679
Q2Q067	Q96A56	0.94809
Q2Q067	Q9NX20	0.39144
Q2Q067	Q5TBB1	0.46264
Q2Q067	F2Z3M2	0.19215
Q2Q067	A8K8V0	0.15483
Q2Q067	P98168	0.65295
Q2Q067	Q96LI9	0.11502
Q2Q067	Q9UBS8	0.5892
Q2Q067	Q2NKX9	0.29573
Q2Q067	Q9H1J1	0.63642
Q2Q067	O60716	0.4503
Q2Q067	P0DMB1	0.28994
Q2Q067	Q15773	0.54845
Q2Q067	P33076	0.48422
Q2Q067	Q6P2E9	1.0441
Q2Q067	Q9NX95	0.16774
Q2Q067	Q7Z3I7	0.10896
Q2Q067	Q9UK58	0.32541
Q2Q067	P23025	0.14463
Q2Q067	Q14999	0.99891
Q2Q067	P51003	0.082232
Q2Q067	Q14397	0.7035
Q2Q067	Q9NUP7	0.49321
Q2Q067	Q03938	0.2426
Q2Q067	P17661	0.46281
Q2Q067	Q8TC90	0.36804
Q2Q067	Q8TF39	0.24938
Q2Q067	O75691	0.75875
Q2Q067	O60224	0.12144
Q2Q067	Q9Y314	1.0074
Q2Q067	O15297	0.5428
Q2Q067	A8MZA4	0.23387
Q2Q067	P35237	0.31689
Q2Q067	Q96JB5	0.27508
Q2Q067	Q3C1V8	0.65617
Q2Q067	P08651	0.29736
Q2Q067	Q9H606	0.18694
Q2Q067	O95059	0.19174
Q2Q067	B3EWG6	0.29573
Q2Q067	Q17RA5	0.19215
Q2Q067	Q8TAP6	0.91292
Q2Q067	O95429	0.18833
Q2Q067	Q9NRC8	1.186
Q2Q067	Q9NQR4	0.23372
Q2Q067	P62979	0.24073
Q85601	Q53EZ4	0.83584
Q85601	Q5T230	1.4611
Q85601	P43351	0.70282

Q85601	Q53EQ6	0.31563
Q85601	Q14139	0.56913
Q85601	Q9UQR1	0.869
Q85601	Q9HCZ1	0.24497
Q85601	Q8TEM1	0.23406
Q85601	Q8N8C0	0.33856
Q85601	Q9Y534	0.27039
Q85601	Q13367	0.5528
Q85601	Q9UJX2	0.62375
Q85601	P13646	0.60904
Q85601	Q5SVZ6	0.58384
Q85601	Q5TA45	0.27948
Q85601	Q3YBR2	0.50265
Q85601	Q03701	0.40583
Q85601	Q6R2W3	0.40732
Q85601	F5H284	0.41838
Q85601	Q14457	0.59947
Q85601	Q99569	0.34708
Q85601	Q96RK1	0.73522
Q85601	Q9H492	0.27226
Q85601	Q9BW61	0.29245
Q85601	Q9ULM2	0.23415
Q85601	Q13596	0.31572
Q85601	Q8NHQ1	1.4287
Q85601	Q9HCG1	0.35977
Q85601	Q9UBZ4	0.19423
Q85601	P54259	1.0389
Q85601	Q9HAY2	0.083938
Q85601	Q9NVM9	1.1708
Q85601	P37802	0.59014
Q85601	Q8N7M0	0.29611
Q85601	A6NNL0	0.052748
Q85601	Q8NBT2	0.78187
Q85601	Q9H4M3	0.22947
Q85601	O14530	0.29336
Q85601	A6NFF2	0.15883
Q85601	Q9UNN4	0.4428
Q85601	P51946	0.58511
Q85601	Q8IYI6	0.47741
Q85601	Q16890	0.39179
Q85601	P46783	1.3535
Q85601	Q96QK1	0.77042
Q85601	A6NNJ1	0.38628
Q85601	P32969	0.78249
Q85601	Q5T870	0.22853
Q85601	Q9NV31	0.77712
Q85601	O95619	0.8366
Q85601	P0CL83	0.5512
Q85601	Q8IXK0	0.33686
Q85601	P10144	0.37105
Q85601	Q6IPT2	0.61067
Q85601	Q9NQG7	0.29038
Q85601	Q9BSU1	0.49715
Q85601	P01106	0.44321
Q85601	Q9P2R6	1.1313
Q85601	Q3KNS6	0.27274
Q85601	Q16612	0.21227
Q85601	Q92618	0.15915
Q85601	Q9ULL0	0.079905
Q85601	P18146	0.72781
Q85601	A4D1E1	0.17469
Q85601	Q494X3	0.51008
Q85601	Q5VYK3	0.3294
Q85601	Q8N1N4	0.54466
Q85601	Q9H3K6	0.15967
Q85601	P0C7V6	0.60189
Q85601	P52657	1.1978
Q85601	Q9UI26	0.44324
Q85601	P10243	0.59997
Q85601	P28066	0.50362
Q85601	Q96IZ0	0.78792
Q85601	P17480	1.0184
Q85601	Q5SZL2	0.29784
Q85601	Q96FQ6	0.3678
Q85601	Q9UBW5	0.53217
Q85601	Q9HCE1	0.68731

Q85601	O75373	0.3518
Q85601	Q8N3X6	0.38445
Q85601	P61163	0.080961
Q85601	Q8N1G0	0.23504
Q85601	O14925	0.1344
Q85601	Q969S8	0.8072
Q85601	O00401	0.65167
Q85601	Q9H4A5	0.76777
Q85601	Q9BZI7	0.5723
Q85601	Q9ULW3	0.72865
Q85601	Q68CJ9	0.77593
Q85601	Q8N196	0.72062
Q85601	P08048	0.063932
Q85601	Q969E4	0.45278
Q85601	Q8N8X9	0.15738
Q85601	Q9H5I1	0.61828
Q85601	O95625	0.23736
Q85601	Q99816	0.48045
Q85601	O60664	0.64168
Q85601	P30281	0.79451
Q85601	Q9UNM6	0.043919
Q85601	Q86TC9	0.41884
Q85601	Q7Z6E9	0.51348
Q85601	Q5PR19	0.24756
Q85601	Q8IY42	0.58572
Q85601	Q8IYN2	1.1171
Q85601	Q8NE22	0.36467
Q85601	Q96CN9	0.56478
Q85601	Q6ZQN3	0.28407
Q85601	Q6NX45	0.30406
Q85601	Q6PD62	0.63548
Q85601	O14639	0.67109
Q85601	O15198	0.4669
Q85601	Q9UGY1	0.18048
Q85601	O43765	0.61084
Q85601	Q9UPN9	0.52676
Q85601	Q9BRT2	0.41231
Q85601	Q5MJ07	0.17798
Q85601	P61513	0.641
Q85601	P22674	0.41032
Q85601	P30405	0.41838
Q85601	O75182	1.1498
Q85601	Q96J02	0.29623
Q85601	P78317	0.72144
Q85601	P60900	0.29628
Q85601	Q8NI51	0.68416
Q85601	Q99543	0.60297
Q85601	Q63HQ0	0.29117
Q85601	Q96MH2	1.2631
Q85601	Q9NZC7	0.387
Q85601	Q6N069	0.23097
Q85601	Q9UPZ3	0.3451
Q85601	Q8IXT2	0.13144
Q85601	O14490	0.21086
Q85601	Q9Y250	1.0078
Q85601	Q03989	0.29804
Q85601	Q6P435	0.24823
Q85601	Q99497	0.10065
Q85601	P61981	0.52884
Q85601	P49454	0.60589
Q85601	O60225	0.1323
Q85601	Q9HBH1	0.4727
Q85601	Q9Y2X0	0.49674
Q85601	P20700	0.34811
Q85601	Q05DH4	0.51349
Q85601	O00186	0.76455
Q85601	Q6UX04	0.37848
Q85601	Q9NRL3	0.4461
Q85601	Q14978	0.54092
Q85601	Q9BUL9	0.92982
Q85601	O00213	0.43411
Q85601	P08729	1.4372
Q85601	Q15637	0.35183
Q85601	Q8NHW5	0.23796
Q85601	Q92585	0.97005
Q85601	Q8IWJ2	0.70332

Q85601	Q5SRE5	0.63596
Q85601	P17097	0.22177
Q85601	P54920	0.42692
Q85601	Q9NRC8	1.3084
Q85601	P51786	0.28387
Q85601	Q96CJ1	1.2412
Q85601	Q15014	1.2761
Q85601	Q8TE85	0.28792
Q85601	Q96M02	0.089366
Q85601	P69905	0.26103
Q85601	Q6QNY0	0.453
Q85601	Q9NRN7	0.50494
Q85601	Q32P41	0.42706
Q85601	Q15532	0.99318
Q85601	Q9P2D3	0.78495
Q85601	Q969J2	0.28525
Q85601	O75161	0.73523
Q85601	Q8N9K5	0.33324
Q85601	P78424	0.25161
Q85601	Q9NZU5	0.3841
Q85601	O15511	0.17646
Q85601	Q15542	1.0029
Q85601	Q9UHL9	0.33524
Q85601	Q32M78	0.41841
Q85601	P28698	0.73618
Q85601	P02538	0.64802
Q85601	Q8IZC7	0.20522
Q85601	Q9BRR8	0.51058
Q85601	Q92664	0.13833
Q85601	O00458	0.55047
Q85601	Q5RKV6	0.16112
Q85601	Q92734	0.15525
Q85601	O75496	0.93006
Q85601	P49639	0.24257
Q85601	P15621	0.4573
Q85601	Q9NWS1	1.0405
Q85601	Q8WXC7	0.41805
Q85601	Q9Y608	0.3283
Q85601	Q96BW1	0.23411
Q85601	O75971	0.63409
Q85601	P09629	0.94441
Q85601	F8WFD2	0.0035824
Q85601	Q63HK3	0.42016
Q85601	P26373	0.85396
Q85601	Q96JM3	0.43543
Q85601	Q4VC44	0.34052
Q85601	O15234	0.69419
Q85601	Q6PGN9	0.87183
Q85601	Q9BQ52	0.20652
Q85601	Q52LA3	0.15697
Q85601	P49756	0.35585
Q85601	Q96MU7	0.65242
Q85601	Q7Z739	0.30691
Q85601	P61978	0.89653
Q85601	P09234	0.53619
Q85601	O43913	0.34368
Q85601	Q6PJG6	0.62145
Q85601	P0CG00	0.24501
Q85601	Q96CB8	0.60464
Q85601	P51815	0.24802
Q85601	Q9H7T9	0.77512
Q85601	Q969W3	0.66653
Q85601	Q9Y2M0	0.42227
Q85601	Q9UHF7	0.5276
Q85601	P02489	0.33296
Q85601	Q9BRU9	0.22662
Q85601	Q9Y5T5	0.3555
Q85601	P62857	0.48227
Q85601	Q96S65	0.98924
Q85601	Q13487	1.1552
Q85601	P36954	0.20346
Q85601	Q9ULR0	0.58204
Q85601	Q86WV6	0.066901
Q85601	A8K5M9	0.47412
Q85601	P52815	0.49984
Q85601	Q96G28	0.22222

Q85601	Q9Y3Y2	0.60593
Q85601	P54198	1.0554
Q85601	Q6ZR52	0.35363
Q85601	Q7Z4Q2	0.50242
Q85601	Q15643	1.0185
Q85601	O15527	0.49379
Q85601	Q99440	0.34284
Q85601	Q8N5Y2	0.093673
Q85601	Q9NQX6	0.31946
Q85601	Q96LC9	0.56756
Q85601	Q15370	1.0998
Q85601	Q6P2E9	1.0826
Q85601	O15126	0.29796
Q85601	Q13064	0.30039
Q85601	Q9Y3I1	0.89867
Q85601	O43598	0.20944
Q85601	O43715	0.62853
Q85601	Q7Z5D8	0.2625
Q85601	Q64ET8	0.33413
Q85601	Q92837	0.54459
Q85601	Q9HCS7	0.41569
Q85601	Q9UKV0	0.47138
Q85601	Q16543	0.40373
Q85601	Q96QR8	0.36511
Q85601	P08151	0.37549
Q85601	P60174	0.22831
Q85601	Q9H2F5	0.60595
Q85601	Q9Y4R8	0.1799
Q85601	O43493	0.32124
Q85601	P62244	0.65109
Q85601	Q13409	0.56296
Q85601	Q96GA3	0.76879
Q85601	Q6PEW1	0.11394
Q85601	Q6ZTI6	0.49989
Q85601	P46087	0.63965
Q85601	Q86W11	0.26361
Q85601	Q9ULX3	0.38276
Q85601	P09001	0.23391
Q85601	Q14746	0.38227
Q85601	O60346	0.23449
Q85601	Q8NA42	0.32641
Q85601	P11831	0.58038
Q85601	Q8WX93	0.27531
Q85601	Q8N137	0.91377
Q85601	Q9Y2T7	0.16139
Q85601	Q5T7V8	1.2798
Q85601	Q96BY7	0.21592
Q85601	Q14929	0.29322
Q85601	P07315	0.60917
Q85601	Q9H7E2	0.48909
Q85601	Q96T76	0.98752
Q85601	Q4LE39	0.93481
Q85601	Q14525	0.57121
Q85601	Q00536	0.21058
Q85601	Q9BQA1	1.0289
Q85601	Q15853	0.62632
Q85601	Q9BWC9	0.33334
Q85601	P63104	0.99323
Q85601	Q9H4X1	0.66666
Q85601	Q8NFW9	0.36471
Q85601	Q96CV9	0.54213
Q85601	Q9BZL1	0.075178
Q85601	Q9UMY1	0.49334
Q85601	Q6TDU7	0.050024
Q85601	Q8TF39	0.47651
Q85601	A6NHX0	0.37151
Q85601	Q9HBT8	0.25932
Q85601	Q9UPG8	0.32454
Q85601	Q8ND90	0.72645
Q85601	O14896	0.56525
Q85601	Q02078	0.42287
Q85601	Q9P1U0	0.24173
Q85601	Q96JK9	0.95809
Q85601	O00629	0.63007
Q85601	Q9BQC6	0.4222
Q85601	P53675	0.31245

56013	Q85601	O60239	0.34525
56014	Q85601	Q96EL2	0.33745
56015	Q85601	P17676	0.82024
56016	Q85601	P15036	0.80146
56017	Q85601	Q5SZD4	0.26929
56018	Q85601	Q9NTK5	0.38722
56019	Q85601	P35321	0.42949
56020	Q85601	Q92575	0.98214
56021	Q85601	Q9BXR0	1.0205
56022	Q85601	Q16666	0.62688
56023	Q85601	Q9BYD1	0.82143
56024	Q85601	P78386	0.59315
56025	Q85601	Q96C34	0.56737
56026	Q85601	Q5HYR2	0.42695
56027	Q85601	P80294	0.82454
56028	Q85601	Q17RC7	0.17046
56029	Q85601	Q96A73	0.59692
56030	Q85601	P09874	0.75725
56031	Q85601	O75688	0.36875
56032	Q85601	Q8NC51	0.66443
56033	Q85601	P54136	0.74221
56034	Q85601	O43324	1.1838
56035	Q85601	O60481	0.48963
56036	Q85601	Q13309	0.50889
56037	Q85601	Q9NSD9	0.47301
56038	Q85601	Q14593	0.52008
56039	Q85601	P62913	0.96947
56040	Q85601	Q96DB2	0.29035
56041	Q85601	P62633	0.75856
56042	Q85601	Q9BUE0	0.99267
56043	Q85601	Q9P2G1	0.32982
56044	Q85601	Q9ULZ2	0.8334
56045	Q85601	P09913	0.57298
56046	Q85601	O75360	0.47218
56047	Q85601	P17040	0.7007
56048	Q85601	Q9ULW8	0.35517
56049	Q85601	Q6ZN11	0.29342
56050	Q85601	Q9UHB4	0.64392
56051	Q85601	P11473	0.3983
56052	Q85601	O95602	0.69449
56053	Q85601	Q9UPX8	0.32367
56054	Q85601	Q2VY69	0.34062
56055	Q85601	Q53FT3	0.28883
56056	Q85601	Q3MJ62	0.27663
56057	Q85601	O75843	0.67375
56058	Q85601	Q8NCR3	0.20341
56059	Q85601	Q9NRP2	0.37355
56060	Q85601	O94864	0.53771
56061	Q85601	P08107	0.5604
56062	Q85601	Q8N720	0.97565
56063	Q85601	Q86XU0	0.31419
56064	Q85601	P62253	0.66549
56065	Q85601	P55036	0.40012
56066	Q85601	P34949	0.15804
56067	Q85601	Q9BXT5	0.45162
56068	Q85601	Q9UJK0	0.40165
56069	Q85601	O00534	0.22094
56070	Q85601	Q92546	0.44379
56071	Q85601	Q92614	0.14547
56072	Q85601	Q06455	0.76621
56073	Q85601	A6NGY1	0.46463
56074	Q85601	Q96SZ4	0.23061
56075	Q85601	Q56P03	0.38682
56076	Q85601	P83916	0.64402
56077	Q85601	Q9UHY8	0.23573
56078	Q85601	P27816	0.52058
56079	Q85601	Q9NZU7	0.39583
56080	Q85601	Q9UNY5	0.41246
56081	Q85601	Q75VX8	0.36411
56082	Q85601	P25208	0.62407
56083	Q85601	Q9H1I8	0.28491
56084	Q85601	Q9P0J0	0.47949
56085	Q85601	Q14586	0.25802
56086	Q85601	Q96MA1	0.25289
56087	Q85601	P22681	0.4208
56088	Q85601	Q9UJW0	0.61354

Q85601	Q9H6Z9	0.48193
Q85601	O75145	0.44987
Q85601	P13807	0.41596
Q85601	O14529	0.37591
Q85601	O15535	0.24212
Q85601	O43502	0.33083
Q85601	O15499	0.17895
Q85601	P02545	0.70597
Q85601	Q6EEV6	0.15956
Q85601	Q5T9G4	0.72122
Q85601	Q96S99	0.18945
Q85601	Q9NPA5	0.03995
Q85601	P62879	0.48684
Q85601	O15315	0.17029
Q85601	P11168	0.11351
Q85601	Q9UJW8	0.26111
Q85601	P33076	0.51825
Q85601	Q9HAU4	0.3815
Q85601	Q9BT49	0.60554
Q85601	Q13495	0.96538
Q85601	Q8NBB4	0.33082
Q85601	P31946	0.55624
Q85601	Q9UPT5	0.59042
Q85601	Q9NQV7	0.32042
Q85601	P68133	0.1273
Q85601	O95229	0.84892
Q85601	Q9H8M2	0.12117
Q85601	Q6VUC0	0.46815
Q85601	A2BFH1	0.41838
Q85601	Q9Y2J2	0.40749
Q85601	Q96JB3	0.14292
Q85601	Q6X4W1	0.81023
Q85601	Q9UKL3	0.99189
Q85601	Q96DE5	0.77206
Q85601	Q53ET0	1.5062
Q85601	A6NHJ4	0.51874
Q85601	Q9NPD3	0.29968
Q85601	Q9HAV4	1.0917
Q85601	Q96PK6	0.67816
Q85601	O94925	0.52928
Q85601	Q9BSH4	0.57595
Q85601	Q9UIA9	0.52513
Q85601	Q9ULJ3	0.64573
Q85601	Q13127	0.55803
Q85601	Q9ULN7	0.58341
Q85601	Q9NS56	0.3887
Q85601	P16157	0.25001
Q85601	Q9NP79	0.34292
Q85601	Q99733	0.77641
Q85601	Q9NVD7	0.28387
Q85601	P14373	0.33721
Q85601	O43316	0.23878
Q85601	Q9BXH1	0.33715
Q85601	Q9NY87	0.27561
Q85601	Q96LB3	0.69517
Q85601	Q9H707	0.32213
Q85601	O43148	0.8035
Q85601	Q96HA7	0.82587
Q85601	P62854	0.82503
Q85601	O15027	0.56456
Q85601	P26447	0.24274
Q85601	Q2M3X9	0.31208
Q85601	Q8N1G2	0.3469
Q85601	P48444	0.49215
Q85601	Q7L590	1.0642
Q85601	Q9UJX6	0.57029
Q85601	Q9NP97	0.64482
Q85601	Q00610	0.89452
Q85601	Q16763	0.76908
Q85601	P78346	0.57931
Q85601	Q8TDR0	0.53674
Q85601	Q9GZW5	0.5118
Q85601	Q8IUE1	0.32996
Q85601	Q96M95	0.08493
Q85601	Q08AM6	0.22856
Q85601	O75528	0.67935

56165	Q85601	P23025	0.21206
56166	Q85601	Q9H2H8	0.41446
56167	Q85601	Q9NZH4	0.82051
56168	Q85601	O75143	0.66359
56169	Q85601	P82930	0.23982
56170	Q85601	P55273	0.23207
56171	Q85601	Q9Y2T2	0.55686
56172	Q85601	Q96MX3	0.2804
56173	Q85601	P31749	0.51456
56174	Q85601	Q16236	0.70242
56175	Q85601	Q6V9R5	0.46434
56176	Q85601	Q7Z4H3	0.24491
56177	Q85601	Q5EBL2	0.23545
56178	Q85601	C9JN71	0.36472
56179	Q85601	P99999	0.71828
56180	Q85601	P62993	0.24712
56181	Q85601	O76013	0.57121
56182	Q85601	Q86XA9	0.16218
56183	Q85601	O75928	0.90382
56184	Q85601	O00541	0.41038
56185	Q85601	Q9BTE3	0.79474
56186	Q85601	Q9NYK6	0.12492
56187	Q85601	Q86VP1	0.42515
56188	Q85601	P24539	0.21524
56189	Q85601	P02533	0.80646
56190	Q85601	P24385	0.57805
56191	Q85601	Q53TQ3	0.65253
56192	Q85601	Q7Z2Z1	0.72232
56193	Q85601	Q13490	0.46505
56194	Q85601	Q7Z7K6	0.36399
56195	Q85601	Q8TDB6	0.54871
56196	Q85601	Q8N3Z6	0.63294
56197	Q85601	Q7Z340	0.28926
56198	Q85601	P31689	0.62541
56199	Q85601	Q9BRU2	0.50791
56200	Q85601	O43924	0.10185
56201	Q85601	Q9NUP9	0.25634
56202	Q85601	Q9HCI6	0.44445
56203	Q85601	P14316	0.95521
56204	Q85601	Q9BTP7	1.0759
56205	Q85601	P09496	0.29788
56206	Q85601	O14829	0.29398
56207	Q85601	Q5RGN0	0.46437
56208	Q85601	Q6ZN17	1.1721
56209	Q85601	P17096	0.56154
56210	Q85601	Q3LI76	0.3247
56211	Q85601	Q9Y383	0.6951
56212	Q85601	Q9H0E7	0.28572
56213	Q85601	Q9BYR3	0.17342
56214	Q85601	P06396	0.39315
56215	Q85601	Q8WV37	0.55776
56216	Q85601	Q9NUP1	0.67833
56217	Q85601	Q9Y2W2	0.68707
56218	Q85601	P82912	0.13386
56219	Q85601	Q9UQN3	0.51425
56220	Q85601	Q96SR6	0.31208
56221	Q85601	Q5JNZ3	0.35232
56222	Q85601	A6NK39	0.26877
56223	Q85601	A6NIE6	0.38764
56224	Q85601	P62081	0.95151
56225	Q85601	Q96ND8	0.48756
56226	Q85601	Q5VWI1	0.92413
56227	Q85601	Q8NEF3	0.067446
56228	Q85601	O75154	0.88221
56229	Q85601	O95831	0.75889
56230	Q85601	Q9H5P4	0.39218
56231	Q85601	P26639	0.3236
56232	Q85601	P50452	0.65696
56233	Q85601	Q9H5F2	0.056236
56234	Q85601	O75112	0.36365
56235	Q85601	P41134	0.34456
56236	Q85601	Q9Y324	0.51234
56237	Q85601	Q86TA1	0.32369
56238	Q85601	Q6UVJ0	0.86345
56239	Q85601	Q6P2D8	0.26522
56240	Q85601	P63146	0.75667

Q85601	Q6PG37	0.4826
Q85601	Q9H0S4	0.062964
Q85601	O43390	0.4471
Q85601	P51116	0.53086
Q85601	Q96K62	0.25678
Q85601	Q15834	1.1825
Q85601	O14933	0.67993
Q85601	Q8N8G2	0.93149
Q85601	Q99575	0.7077
Q85601	Q02241	0.74051
Q85601	Q6P5R6	0.16468
Q85601	Q8IYB3	0.26886
Q85601	P10768	0.3075
Q85601	Q9NPI6	0.98163
Q85601	P61582	0.50359
Q85601	Q5T2W1	0.45137
Q85601	O14813	0.32759
Q85601	Q15038	1.3908
Q85601	Q8WVC0	1.0172
Q85601	Q8WZ19	0.085341
Q85601	Q9Y4Z2	0.26076
Q85601	Q8NFH4	0.94794
Q85601	Q96EK4	1.3031
Q85601	P14868	0.53295
Q85601	Q96RL1	0.78178
Q85601	Q92917	0.81269
Q85601	Q9NQW6	0.36215
Q85601	Q9UD71	0.67861
Q85601	O43708	0.4296
Q85601	Q5JVS0	0.80389
Q85601	P61353	0.63754
Q85601	Q9BZE2	0.99823
Q85601	Q14765	0.40309
Q85601	Q15750	0.49663
Q85601	Q96A35	0.22165
Q85601	Q01831	0.6933
Q85601	Q5TYW1	0.23373
Q85601	O94986	1.2566
Q85601	O95149	0.42501
Q85601	Q9UG56	0.069329
Q85601	A6NFI3	0.25155
Q85601	P0DMB2	0.40554
Q85601	Q8TAA5	0.15218
Q85601	Q6J272	0.43043
Q85601	Q15388	0.17257
Q85601	P60369	0.17342
Q85601	Q9Y2S7	0.37972
Q85601	Q7Z591	0.7194
Q85601	Q9NQP4	0.92786
Q85601	Q9BSF8	0.44273
Q85601	Q8N228	0.35025
Q85601	Q9NR22	0.34147
Q85601	Q14258	1.1077
Q85601	P53672	0.45348
Q85601	Q8N7B1	0.48996
Q85601	P62891	0.53692
Q85601	O96017	0.68679
Q85601	Q07666	0.38949
Q85601	O14910	0.61887
Q85601	Q9BQC3	0.73233
Q85601	Q9NSB4	0.43164
Q85601	Q8NFH3	0.94794
Q85601	Q9UC07	0.33398
Q85601	Q9BXF3	0.64247
Q85601	Q15154	0.58697
Q85601	Q92636	0.31212
Q85601	Q03188	0.35005
Q85601	P46776	0.87396
Q85601	Q96CS4	0.29188
Q85601	Q9H1D9	0.53094
Q85601	Q14689	0.34277
Q85601	Q92754	0.50278
Q85601	Q9NX08	0.30691
Q85601	Q02556	0.45491
Q85601	P17028	0.73424
Q85601	P51522	0.27754

Q85601	O60563	1.5422
Q85601	P42568	0.88121
Q85601	Q6NUQ1	0.55827
Q85601	P51808	0.45905
Q85601	P61968	0.30088
Q85601	Q9C002	0.16534
Q85601	Q9BZQ2	0.28402
Q85601	Q8N7M2	0.51008
Q85601	Q9P0W2	0.69802
Q85601	Q969W8	0.4005
Q85601	Q9NUA8	0.25219
Q85601	Q8TDD2	0.37485
Q85601	Q9UHD1	0.23859
Q85601	Q9BWX5	0.44233
Q85601	Q8IUI4	0.024346
Q85601	A6NK75	0.38145
Q85601	P36405	0.28994
Q85601	Q96NT1	0.97031
Q85601	Q9H900	0.55274
Q85601	P31150	0.14549
Q85601	O00471	0.29941
Q85601	Q5VWT5	0.15215
Q85601	Q8NHY5	1.0082
Q85601	Q01538	0.25156
Q85601	Q8N0V3	0.20454
Q85601	P60368	0.043883
Q85601	Q6ZYL4	0.71016
Q85601	P0CB33	0.47854
Q85601	Q5XKE5	0.47806
Q85601	Q8N8E3	0.17433
Q85601	Q8WV24	0.68711
Q85601	Q9BR77	0.2015
Q85601	Q99687	0.48385
Q85601	Q6ZRH4	1.169
Q85601	Q9P2K5	0.12152
Q85601	Q93009	0.41255
Q85601	Q9ULD5	0.21694
Q85601	P47813	0.95003
Q85601	P09086	0.36102
Q85601	P08779	0.67587
Q85601	Q96A65	0.22651
Q85601	P21506	0.33241
Q85601	Q9BUX1	0.79509
Q85601	Q15398	1.0112
Q85601	Q9BSB4	0.31361
Q85601	Q6XYB7	0.32996
Q85601	Q15466	0.59583
Q85601	Q13884	0.51394
Q85601	Q01892	0.39719
Q85601	Q8N2H4	1.188
Q85601	Q96BP3	0.39478
Q85601	Q8N371	0.32176
Q85601	Q9UPW6	0.7876
Q85601	A6NJ46	0.27808
Q85601	Q7Z6M4	0.32095
Q85601	Q86X55	0.74104
Q85601	Q9H7P6	0.58802
Q85601	Q9BVS5	0.27037
Q85601	Q8WV44	0.43447
Q85601	Q15004	0.856
Q85601	Q86VS8	0.43012
Q85601	P82933	0.56602
Q85601	Q86Z20	0.96654
Q85601	Q6ZMR3	0.49983
Q85601	O15534	0.2614
Q85601	Q96AH0	0.30118
Q85601	Q8IUR6	0.94308
Q85601	Q9UQ80	0.99514
Q85601	Q3SY84	0.49278
Q85601	Q96JP2	0.21058
Q85601	P18077	0.43553
Q85601	Q7L804	0.61046
Q85601	Q6AI08	0.2144
Q85601	P50539	0.23243
Q85601	Q12901	0.38699
Q85601	O95684	0.86376

Q85601	Q9H706	0.30691
Q85601	Q08999	1.3764
Q85601	P56270	0.53744
Q85601	Q9NYJ8	0.5534
Q85601	Q9H0N5	0.84344
Q85601	O95347	0.48583
Q85601	Q9H8G1	0.52008
Q85601	Q8IYL3	0.20715
Q85601	P15976	0.30803
Q85601	Q14008	0.93152
Q85601	O75909	1.0706
Q85601	Q969H0	0.73138
Q85601	A1L3X0	0.54781
Q85601	Q9BQG0	0.73881
Q85601	Q3SXZ3	0.35256
Q85601	Q8IYD8	0.37551
Q85601	Q6IN85	0.30982
Q85601	Q5T0L3	1.2136
Q85601	Q96SC8	0.15598
Q85601	Q96BP2	1.2261
Q85601	Q52M58	0.016113
Q85601	Q6UUV9	1.5436
Q85601	P25787	0.4982
Q85601	Q8WWK9	0.36901
Q85601	Q96MT4	0.10176
Q85601	Q9GZZ1	0.42632
Q85601	P46060	0.2863
Q85601	Q9UJY4	0.49979
Q85601	Q9BY41	0.49946
Q85601	Q9BWH6	0.30032
Q85601	Q96EE3	0.60109
Q85601	Q14194	0.42574
Q85601	P61313	0.87396
Q85601	P41091	0.075188
Q85601	Q9UL59	0.31481
Q85601	Q9Y2R5	0.16086
Q85601	O94776	1.0617
Q85601	Q5C9Z4	0.6325
Q85601	O60888	0.37537
Q85601	Q2KHR2	0.23821
Q85601	Q9BQQ3	0.19147
Q85601	Q9UQ84	0.31837
Q85601	Q6Q759	0.41576
Q85601	O00165	0.77123
Q85601	Q7RTV0	0.41513
Q85601	Q9Y3D5	0.15711
Q85601	Q7L945	0.31315
Q85601	Q8WXE1	0.74037
Q85601	Q92994	0.43515
Q85601	Q86SG2	0.27273
Q85601	P61962	0.72744
Q85601	O15265	1.1631
Q85601	Q96H20	1.0443
Q85601	O75396	0.25941
Q85601	Q96RT1	0.29127
Q85601	Q9UQC2	0.26123
Q85601	Q9C0G0	0.034064
Q85601	Q99613	0.68618
Q85601	Q16384	0.35321
Q85601	Q8TBK2	0.52369
Q85601	P49406	0.44234
Q85601	P05388	1.2109
Q85601	P50479	0.32982
Q85601	P19484	0.61389
Q85601	Q13243	0.63967
Q85601	P50750	0.41449
Q85601	Q9UBQ5	1.0283
Q85601	P17019	0.47479
Q85601	O75691	0.80216
Q85601	Q52LR7	0.51352
Q85601	Q96NG8	0.31529
Q85601	Q5VV17	0.020218
Q85601	Q9Y2Z0	0.49016
Q85601	Q14202	0.31871
Q85601	Q9HD47	0.22925
Q85601	Q7L4I2	1.2837

Q85601	P15172	0.35304
Q85601	P49789	0.63948
Q85601	Q04724	0.89452
Q85601	Q17RY6	0.30939
Q85601	O75190	0.72468
Q85601	Q9UN36	0.58221
Q85601	Q9HCK5	0.62448
Q85601	B2RV13	0.11433
Q85601	O95059	0.04101
Q85601	Q92547	0.68618
Q85601	Q9H040	0.58752
Q85601	Q5HYK7	1.2402
Q85601	P05413	0.25825
Q85601	P62714	0.80429
Q85601	Q9ULK2	0.79691
Q85601	Q5HY98	0.40802
Q85601	Q96MC9	0.55426
Q85601	Q9GZV4	0.58359
Q85601	P0CG29	0.35473
Q85601	A6NNA5	0.16654
Q85601	Q8N2N9	0.1977
Q85601	Q96BD5	0.59171
Q85601	Q9NVD3	0.066076
Q85601	P14921	0.8125
Q85601	Q8IYF3	0.4174
Q85601	Q6B0B8	0.2124
Q85601	P18887	0.67327
Q85601	B9ZVM9	1.0414
Q85601	Q969S2	0.14825
Q85601	Q9ULV5	0.42204
Q85601	Q9UBS3	0.60494
Q85601	O60437	0.78594
Q85601	Q9UL33	0.33243
Q85601	Q9BZE4	0.34768
Q85601	Q8IUC6	0.22221
Q85601	Q92558	0.37137
Q85601	Q2M2Z5	0.71476
Q85601	Q6P1L6	0.24948
Q85601	Q9H320	0.27102
Q85601	P63151	0.38747
Q85601	Q03052	0.36887
Q85601	Q8N8L2	0.26852
Q85601	Q9HB09	0.58717
Q85601	Q6ZN57	0.27324
Q85601	Q96BR6	0.38547
Q85601	Q9ULK4	1.1733
Q85601	Q6NZ36	0.31289
Q85601	Q5VYV7	0.30691
Q85601	Q6ZMS7	0.34677
Q85601	Q96HQ0	0.30526
Q85601	Q5JSH3	0.24526
Q85601	Q9HD36	0.44977
Q85601	Q96JM2	0.24984
Q85601	P62495	0.54199
Q85601	Q8N9R8	0.66054
Q85601	Q9NP92	0.5641
Q85601	Q9Y5K6	0.27184
Q85601	Q5VTE0	0.65989
Q85601	P17661	0.44395
Q85601	Q9Y2N7	0.61566
Q85601	Q9BQD3	0.36464
Q85601	A6NKH3	0.17681
Q85601	Q86WV5	0.36843
Q85601	Q9BRA2	0.52661
Q85601	Q9NPG3	0.81431
Q85601	Q659C4	0.44911
Q85601	Q96CW1	0.53952
Q85601	Q6NVV0	0.0062555
Q85601	P86452	0.52553
Q85601	Q9UQL6	0.5214
Q85601	Q8NB12	0.43737
Q85601	Q9Y3B7	0.87707
Q85601	Q15629	0.21048
Q85601	P13682	0.33475
Q85601	P09917	0.24236
Q85601	P13051	0.95553

Q85601	P08651	0.27919
Q85601	O14879	0.69926
Q85601	Q9H489	0.63412
Q85601	Q9UGR2	0.45
Q85601	Q15833	0.34385
Q85601	O14579	0.43636
Q85601	Q15293	0.24763
Q85601	Q86YA3	0.33347
Q85601	Q3B7T1	0.45278
Q85601	Q9UIF9	0.68509
Q85601	Q3L8U1	0.93471
Q85601	Q7Z713	0.23411
Q85601	P13533	0.42967
Q85601	Q15486	0.36486
Q85601	Q86VM9	0.21504
Q85601	Q86V15	0.43653
Q85601	O95487	0.75672
Q85601	P57772	0.1819
Q85601	Q9BXW9	0.8225
Q85601	Q6STE5	0.74559
Q85601	Q9H6D7	0.65651
Q85601	O75151	0.63486
Q85601	Q96JF6	0.24796
Q85601	Q9BT25	0.6577
Q85601	Q8TBE0	0.79568
Q85601	O95671	0.21076
Q85601	Q2V2M9	0.33461
Q85601	Q99547	0.65741
Q85601	Q7Z4T9	0.71018
Q85601	Q9UBB4	0.69763
Q85601	P21399	0.35882
Q85601	O15182	0.46019
Q85601	Q5PSV4	0.41331
Q85601	O75398	0.26549
Q85601	Q9H321	0.29282
Q85601	Q8WUN7	0.44273
Q85601	P17032	0.29823
Q85601	Q9Y3M2	0.30815
Q85601	A8MTZ0	0.22515
Q85601	P49589	0.28679
Q85601	Q9P253	0.1619
Q85601	Q9NZF1	0.033739
Q85601	Q16656	0.42101
Q85601	Q99871	0.66313
Q85601	Q9C0F0	0.43526
Q85601	Q9NQS7	0.97485
Q85601	A8CG34	0.35769
Q85601	Q8IY18	0.45417
Q85601	Q6PF04	0.28195
Q85601	Q5T6M2	0.4715
Q85601	P48634	0.87447
Q85601	Q9UL58	0.55761
Q85601	Q9Y3D0	1.0174
Q85601	P60002	0.64354
Q85601	Q96F07	0.45729
Q85601	O95153	0.65356
Q85601	Q96DC7	1.1041
Q85601	P17036	0.37229
Q85601	Q6UXP7	0.08194
Q85601	Q4U2R6	0.82143
Q85601	Q6DD87	0.24776
Q85601	Q96RT8	0.44028
Q85601	Q9HCL3	0.46743
Q85601	O15151	0.23627
Q85601	Q13439	0.73916
Q85601	Q14CW9	0.92932
Q85601	Q9NRR5	0.7184
Q85601	Q9BYG5	0.69973
Q85601	P04732	0.26807
Q85601	Q9BW71	0.61353
Q85601	O95447	0.039897
Q85601	Q86U70	0.23897
Q85601	Q9Y613	0.6135
Q85601	Q06546	1.2105
Q85601	O14599	0.44263
Q85601	Q9HCK1	0.59445

Q85601	Q05048	0.61245
Q85601	P23508	0.41383
Q85601	P61289	0.70459
Q85601	Q9Y2L8	0.50068
Q85601	P60331	0.17342
Q85601	Q6W349	0.0019713
Q85601	P06730	0.5129
Q85601	Q8IY63	0.45941
Q85601	Q9P2D1	0.20471
Q85601	Q93015	0.094911
Q85601	P50548	0.35477
Q85601	P62304	0.61651
Q85601	Q9GZU7	0.56237
Q85601	O95251	0.95255
Q85601	P07196	0.50402
Q85601	Q9NY61	0.96593
Q85601	Q9Y603	1.2207
Q85601	Q96P70	1.0115
Q85601	Q8N5T8	0.014388
Q85601	Q13769	0.53982
Q85601	Q15329	1.2562
Q85601	Q8WW38	0.60882
Q85601	Q16531	0.68698
Q85601	O95295	0.47071
Q85601	Q3LHN0	0.36976
Q85601	Q12769	0.99377
Q85601	Q8TAC9	0.4198
Q85601	Q9UK58	0.36692
Q85601	P57060	0.51969
Q85601	P56645	0.25571
Q85601	Q01130	0.60688
Q85601	Q8IWV7	0.16498
Q85601	Q58FF8	0.58819
Q85601	P00480	0.096384
Q85601	Q9P2D0	0.24728
Q85601	Q8TC05	0.19623
Q85601	Q8TAQ5	0.35685
Q85601	A6NIX2	1.1079
Q85601	O14713	0.33086
Q85601	Q96MC4	0.49605
Q85601	Q8TDY3	0.11637
Q85601	O95049	0.46996
Q85601	Q02386	0.25356
Q85601	Q0VG99	0.25935
Q85601	Q86YE8	0.35685
Q85601	Q9H0W8	0.25594
Q85601	Q9H307	0.62961
Q85601	P43364	0.87447
Q85601	Q15554	0.51667
Q85601	O00463	0.40793
Q85601	O95166	0.25156
Q85601	Q9UJW3	0.68369
Q85601	Q9BYN7	0.23504
Q85601	Q8NAP3	0.42211
Q85601	O60506	0.27208
Q85601	Q68CZ2	0.33837
Q85601	Q14240	0.12356
Q85601	O15194	0.07935
Q85601	Q6P1J9	0.65102
Q85601	Q9H379	0.053121
Q85601	Q9P206	0.10358
Q85601	Q6P1R3	0.61143
Q85601	Q14549	0.3482
Q85601	Q9HBT7	0.33141
Q85601	P41220	0.44772
Q85601	Q9UI95	1.3525
Q85601	Q92540	0.99281
Q85601	Q8NFV5	0.15217
Q85601	Q9UBU9	0.41756
Q85601	A6NJR5	0.15217
Q85601	Q5FWF6	0.50115
Q85601	Q96FH0	0.23255
Q85601	O60763	0.46912
Q85601	Q5MIZ7	0.50035
Q85601	Q6GPH4	0.54266
Q85601	P56282	0.29436

Q85601	Q96A44	0.49751
Q85601	Q96HR8	0.94664
Q85601	Q14244	0.57757
Q85601	Q96CS3	0.55184
Q85601	P51161	0.22891
Q85601	Q96GT9	0.033402
Q85601	Q8N814	0.46598
Q85601	A6NDR6	0.22476
Q85601	Q32MQ0	0.3878
Q85601	Q9H0C8	0.48256
Q85601	Q96JE7	0.80621
Q85601	Q9Y606	0.95198
Q85601	Q9P2Y5	0.3312
Q85601	Q86WX3	1.1459
Q85601	Q9UBP0	0.2492
Q85601	Q12857	0.33573
Q85601	Q5VTA0	0.21119
Q85601	Q8WXF5	0.30028
Q85601	Q16621	0.51617
Q85601	Q13158	0.43747
Q85601	Q9NQR1	0.70355
Q85601	Q9UBX3	0.3138
Q85601	Q8IYN0	0.3511
Q85601	Q93083	0.28384
Q85601	Q99471	0.85973
Q85601	Q19AV6	1.0556
Q85601	Q75MW2	0.63202
Q85601	Q9BYQ6	0.17342
Q85601	Q14331	0.26834
Q85601	O95714	0.24897
Q85601	Q7Z398	0.27319
Q85601	Q9H081	0.76113
Q85601	P49427	0.71895
Q85601	P0DKL9	0.22322
Q85601	P17020	0.23662
Q85601	P06400	0.68784
Q85601	P0CJ87	0.27808
Q85601	Q06330	0.55059
Q85601	P48378	0.35025
Q85601	Q6ZP82	0.30691
Q85601	Q9Y3C5	0.54471
Q85601	Q969V6	1.4633
Q85601	Q96BV0	0.14522
Q85601	Q92733	0.8435
Q85601	P08670	0.1621
Q85601	O75306	0.088033
Q85601	Q9UKZ1	0.07327
Q85601	Q7Z465	0.67559
Q85601	O75969	0.26233
Q85601	Q13360	0.21568
Q85601	A8MXT2	0.074529
Q85601	P05787	1.3992
Q85601	P35579	0.72294
Q85601	Q92979	0.36034
Q85601	Q9UQK1	0.32904
Q85601	Q96PY6	0.34212
Q85601	Q9HD40	0.5402
Q85601	Q9Y4W2	0.52047
Q85601	Q8N7R0	0.32996
Q85601	Q5T5J6	0.62257
Q85601	Q68E01	0.58042
Q85601	Q96QT6	1.0006
Q85601	Q96BF6	0.39891
Q85601	P12036	0.37769
Q85601	P63173	0.44684
Q85601	P25788	0.56503
Q85601	Q9H7S9	0.77083
Q85601	Q8WTT2	0.32895
Q85601	B4DXR9	0.48246
Q85601	Q16548	0.53158
Q85601	O75312	0.29157
Q85601	P52758	0.54086
Q85601	P56279	0.4939
Q85601	Q9UIL8	0.44456
Q85601	O43541	0.61274
Q85601	Q9H5L6	0.44059

Q85601	P38935	0.52983
Q85601	O95677	0.18933
Q85601	P53567	0.65379
Q85601	Q8N5Q1	0.61067
Q85601	O95336	0.30631
Q85601	Q96LX8	0.25378
Q85601	Q96MP8	0.28648
Q85601	Q12798	0.75373
Q85601	Q8WWW0	0.44409
Q85601	Q9P258	0.75673
Q85601	Q9H7U1	0.53923
Q85601	P36952	0.44379
Q85601	Q96DE9	0.0095678
Q85601	Q96IK1	0.25452
Q85601	B1APH4	0.41767
Q85601	O60290	0.462
Q85601	Q8TD23	0.59385
Q85601	Q8N8J6	0.14203
Q85601	O60907	0.81225
Q85601	Q9NQ89	0.21086
Q85601	Q8N4B4	0.096051
Q85601	Q9H0U6	0.16934
Q85601	Q9BYQ8	0.17342
Q85601	P49593	0.23655
Q85601	P13349	0.3025
Q85601	Q9P2J5	0.28687
Q85601	Q13310	0.33513
Q85601	P23588	0.2373
Q85601	Q9NX20	0.41909
Q85601	P39687	1.3964
Q85601	Q3LI81	0.36976
Q85601	Q53FD0	0.46154
Q85601	Q9H7D7	0.44273
Q85601	A8MWA4	0.47865
Q85601	Q96T23	0.74481
Q85601	P50995	0.84142
Q85601	Q9BR84	0.51874
Q85601	Q6ZNG0	0.29924
Q85601	Q8TCC3	0.096586
Q85601	Q9Y2Y9	0.79713
Q85601	O15111	0.56444
Q85601	P12980	0.88215
Q85601	Q9P2N5	0.092264
Q85601	Q12796	1.4021
Q85601	Q96E11	0.29765
Q85601	Q14103	0.74831
Q85601	Q8WV92	0.33148
Q85601	Q9BX66	0.37986
Q85601	P54845	0.37984
Q85601	P81274	0.55664
Q85601	Q9BYR8	0.36976
Q85601	Q14966	0.093242
Q85601	Q9Y535	0.31717
Q85601	Q13148	0.41095
Q85601	Q15797	0.20726
Q85601	P15622	0.33129
Q85601	Q15773	0.73083
Q85601	Q86YS3	0.1723
Q85601	Q9Y294	1.045
Q85601	Q9BUL8	0.70256
Q85601	P50549	0.92949
Q85601	P14649	0.23442
Q85601	Q96BQ5	0.28632
Q85601	Q96H40	0.45522
Q85601	Q969X6	1.2392
Q85601	Q9H8M7	0.81549
Q85601	Q2M3W8	0.47651
Q85601	Q6NZ67	0.76106
Q85601	Q96EU6	0.21827
Q85601	P82921	0.19944
Q85601	O75800	0.55351
Q85601	Q96Q06	0.45148
Q85601	Q9Y2X8	0.84493
Q85601	O95391	0.20973
Q85601	Q13888	0.60111
Q85601	O15116	0.7323

Q85601	A6NHQ2	0.17525
Q85601	Q6PF05	0.41532
Q85601	P14653	0.36728
Q85601	Q96SN8	0.54678
Q85601	Q8N7U7	0.33004
Q85601	Q8N5J2	0.80151
Q85601	Q00005	0.48982
Q85601	Q8N1B3	0.55825
Q85601	Q9Y283	0.3204
Q85601	Q7Z6R9	0.37155
Q85601	Q9BZE3	0.45111
Q85601	Q9C086	1.5253
Q85601	O43248	0.3357
Q85601	O60315	0.3178
Q85601	Q15036	0.20782
Q85601	Q96BT3	0.58839
Q85601	Q53G26	0.6202
Q85601	P19013	0.53559
Q85601	Q9UP83	1.3101
Q85601	Q8N5F7	1.3374
Q85601	Q8N8R7	0.53217
Q85601	P16455	0.12464
Q85601	Q9BS18	0.47296
Q85601	Q7Z7C8	0.515
Q85601	P62072	0.2988
Q85601	Q14587	0.23373
Q85601	Q03933	0.72386
Q85601	Q9HAF1	1.0938
Q85601	Q13155	0.66899
Q85601	Q5H9M0	0.77709
Q85601	A2RRD8	0.35853
Q85601	Q16719	0.35607
Q85601	Q8NDY6	0.50494
Q85601	Q9H0E3	0.59933
Q85601	Q8NHY6	0.36725
Q85601	Q8TEA8	0.56335
Q85601	O75935	0.98736
Q85601	Q96GX2	0.57132
Q85601	P17027	0.24382
Q85601	Q9Y3Q8	1.2713
Q85601	Q9BXS5	0.78977
Q85601	Q5TD94	0.1602
Q85601	Q01105	0.80248
Q85601	Q9BSF0	0.31662
Q85601	P10746	0.18815
Q85601	Q9BUI4	0.21882
Q85601	Q9UM07	0.48976
Q85601	Q9BU02	0.44767
Q85601	O60813	0.35214
Q85601	Q92665	0.76108
Q85601	Q7Z4H7	1.0083
Q85601	Q9Y6Z5	0.32555
Q85601	C9JE40	0.79215
Q85601	Q71F78	0.066686
Q85601	Q9Y6J0	0.19372
Q85601	Q13112	1.4944
Q85601	Q9H467	1.0637
Q85601	Q9H2W6	0.24725
Q85601	Q9H9T3	0.85073
Q85601	Q5VVM6	0.11502
Q85601	Q9UDY8	0.2681
Q85601	Q9UKT4	0.91064
Q85601	Q9BQ95	1.1106
Q85601	A8MUV8	0.45887
Q85601	Q9HC16	0.15547
Q85601	Q96IM9	0.051044
Q85601	P16333	0.63078
Q85601	Q15006	0.36308
Q85601	Q8NEU8	0.46037
Q85601	Q9NR11	0.26517
Q85601	P07814	0.095726
Q85601	Q13216	0.45555
Q85601	P02042	0.21071
Q85601	Q8N684	0.30538
Q85601	O75400	0.86897
Q85601	P78406	0.54407

Q85601	Q9Y6X2	0.80526
Q85601	Q03936	0.49862
Q85601	Q96P15	0.44379
Q85601	Q08AG5	0.47311
Q85601	Q9Y4C2	0.087078
Q85601	Q9H9F9	1.2785
Q85601	Q8N7R1	0.4324
Q85601	Q13573	0.56464
Q85601	O60308	0.448
Q85601	P59022	0.21923
Q85601	Q8IXQ3	0.40137
Q85601	Q3LI73	0.12421
Q85601	Q56P42	0.23411
Q85601	Q9Y2Y1	0.079874
Q85601	Q9BPX1	0.21026
Q85601	P57740	0.78245
Q85601	Q9H116	0.4026
Q85601	Q13951	0.65543
Q85601	Q2M1K9	0.32175
Q85601	A1L4L8	0.2683
Q85601	Q9BPZ7	0.37079
Q85601	Q86SE8	0.10355
Q85601	Q8TF01	0.22014
Q85601	A8MQ11	0.26267
Q85601	Q5M9Q1	0.91137
Q85601	Q8IYW4	0.097374
Q85601	O75330	0.032524
Q85601	Q14790	0.26841
Q85601	Q92560	0.19596
Q85601	P62877	0.65851
Q85601	Q99417	1.5168
Q85601	Q9UHV7	0.53444
Q85601	P0CG30	0.35473
Q85601	Q9H8S9	0.50726
Q85601	Q5EE01	0.66487
Q85601	P43487	0.3375
Q85601	Q92925	0.56238
Q85601	Q53GS7	0.58454
Q85601	Q6ZN79	0.44594
Q85601	Q9Y4P1	0.15616
Q85601	Q15459	0.49001
Q85601	Q9Y651	0.29874
Q85601	Q6ZU67	0.20174
Q85601	Q15424	0.6905
Q85601	Q8N815	0.029075
Q85601	P51826	0.41232
Q85601	Q9NUS5	0.59556
Q85601	Q96SW2	0.58941
Q85601	Q17R31	0.1199
Q85601	Q99966	0.23916
Q85601	Q9NWS9	0.14382
Q85601	P0CW01	0.47415
Q85601	Q9H3C7	0.51707
Q85601	Q8N680	0.030777
Q85601	P40337	0.92223
Q85601	Q8WTP9	0.48065
Q85601	O75030	0.59539
Q85601	Q14684	0.29799
Q85601	P23396	0.70236
Q85601	O76009	0.56535
Q85601	B1AL46	0.052748
Q85601	Q7RTT3	0.1323
Q85601	Q6P1W5	0.30691
Q85601	Q9NR48	0.45985
Q85601	Q9BRG1	1.1953
Q85601	Q9NRG9	0.95732
Q85601	P35244	0.58868
Q85601	Q86UA6	0.86102
Q85601	A6NHP3	0.15217
Q85601	P56377	0.40398
Q85601	Q99962	0.26823
Q85601	P15822	0.9109
Q85601	Q9BZF9	0.56708
Q85601	P62273	0.6715
Q85601	Q9NVX0	1.1722
Q85601	Q07352	0.96008

57001	Q85601	Q8NHZ8	1.1937
57002	Q85601	Q9Y5J9	0.36408
57003	Q85601	Q9UL18	0.91108
57004	Q85601	Q9BUR4	0.76452
57005	Q85601	Q330K2	0.25558
57006	Q85601	A8MVS1	0.51279
57007	Q85601	Q33E94	0.90177
57008	Q85601	Q9BR46	0.39384
57009	Q85601	Q14695	0.045486
57010	Q85601	Q9Y5J6	0.23724
57011	Q85601	P15407	0.60512
57012	Q85601	Q8N7G0	0.32996
57013	Q85601	O95433	0.55163
57014	Q85601	P49336	0.348
57015	Q85601	Q6P4D5	0.13627
57016	Q85601	Q15007	0.41196
57017	Q85601	Q96T21	0.23184
57018	Q85601	Q8WYP5	0.69735
57019	Q85601	P46778	0.53692
57020	Q85601	Q8N6Q8	0.40516
57021	Q85601	Q8NET4	0.47206
57022	Q85601	A8MVW5	0.84854
57023	Q85601	Q96EK7	0.32352
57024	Q85601	Q14872	0.86407
57025	Q85601	Q86UL8	0.30124
57026	Q85601	Q8NDW4	0.28521
57027	Q85601	Q9UKN5	0.32886
57028	Q85601	Q99708	0.71058
57029	Q85601	Q8TC84	0.44273
57030	Q85601	P18848	1.1636
57031	Q85601	Q9BTX9	0.60224
57032	Q85601	P10155	0.44362
57033	Q85601	Q96LT7	0.34081
57034	Q85601	Q6NSZ9	0.16158
57035	Q85601	A4GXA9	0.50747
57036	Q85601	Q14533	0.73612
57037	Q85601	Q7Z3Y8	0.56535
57038	Q85601	Q9HC62	0.53611
57039	Q85601	Q15654	0.91786
57040	Q85601	P62829	1.0901
57041	Q85601	Q9HBF4	0.27561
57042	Q85601	Q9H9Y2	0.17841
57043	Q85601	Q5H9L4	0.13504
57044	Q85601	Q8WVY7	0.29101
57045	Q85601	P61604	0.7587
57046	Q85601	Q99457	0.72919
57047	Q85601	O75832	0.34497
57048	Q85601	Q8N5X7	0.057444
57049	Q85601	Q96D03	0.23369
57050	Q85601	Q9P0W5	0.42435
57051	Q85601	Q4G0N7	0.13085
57052	Q85601	Q8NHV4	0.83842
57053	Q85601	Q14814	0.60477
57054	Q85601	Q86TX2	0.20044
57055	Q85601	Q13506	0.44716
57056	Q85601	Q9P299	0.3048
57057	Q85601	Q9UDV6	0.59532
57058	Q85601	Q0VDD7	1.0786
57059	Q85601	Q13129	0.80582
57060	Q85601	Q8WV60	0.22702
57061	Q85601	O15304	1.0978
57062	Q85601	P19419	1.3196
57063	Q85601	P40763	0.34573
57064	Q85601	O15155	0.1451
57065	Q85601	P18669	0.68342
57066	Q85601	P30050	0.67535
57067	Q85601	Q05195	0.57599
57068	Q85601	O95989	0.20108
57069	Q85601	Q7L266	0.31754
57070	Q85601	Q8N3S3	0.34535
57071	Q85601	Q9NWB7	0.62582
57072	Q85601	Q3BBV2	0.46437
57073	Q85601	Q8IV36	0.55151
57074	Q85601	Q9NY27	0.90459
57075	Q85601	O14978	0.48564
57076	Q85601	Q9H0A6	0.50637

57077	Q85601	Q13426	0.37448
57078	Q85601	P28702	0.42036
57079	Q85601	Q86X83	0.30691
57080	Q85601	Q14241	0.38545
57081	Q85601	Q8TBZ9	1.0465
57082	Q85601	Q03112	0.26561
57083	Q85601	Q96A04	0.55173
57084	Q85601	Q9UMS4	0.5981
57085	Q85601	Q8ND76	0.60582
57086	Q85601	Q6P1M3	0.96691
57087	Q85601	P60006	0.098824
57088	Q85601	Q9UHJ9	0.23343
57089	Q85601	P35080	0.39563
57090	Q85601	Q06787	0.76464
57091	Q85601	Q49AN0	0.19762
57092	Q85601	Q9Y697	0.68453
57093	Q85601	Q5TI25	1.1879
57094	Q85601	Q8NFG4	0.73997
57095	Q85601	P28069	0.75337
57096	Q85601	A0PJX2	0.11453
57097	Q85601	O60583	1.0619
57098	Q85601	P17030	0.31301
57099	Q85601	P62753	0.9568
57100	Q85601	P21912	0.22872
57101	Q85601	Q75QN2	0.58748
57102	Q85601	Q9UKA2	0.50627
57103	Q85601	P32519	1.3867
57104	Q85601	Q6PI26	1.1608
57105	Q85601	P14859	1.1649
57106	Q85601	O95793	0.19089
57107	Q85601	Q93050	0.19447
57108	Q85601	Q6P047	0.23425
57109	Q85601	P79522	0.51489
57110	Q85601	Q9UDY2	0.95632
57111	Q85601	Q9Y468	0.94204
57112	Q85601	Q9BVJ6	0.78003
57113	Q85601	Q9NQ92	0.92865
57114	Q85601	Q9H0R8	0.23785
57115	Q85601	Q9Y4A5	0.81288
57116	Q85601	P61201	0.53411
57117	Q85601	O60663	0.62482
57118	Q85601	Q8TF74	0.4558
57119	Q85601	P27815	0.38367
57120	Q85601	Q01534	0.48372
57121	Q85601	P52740	0.23373
57122	Q85601	A6NCC3	0.19777
57123	Q85601	P11172	0.24693
57124	Q85601	Q96KM6	0.24704
57125	Q85601	E9PAV3	0.43898
57126	Q85601	P61956	0.67961
57127	Q85601	Q8TDB4	0.19268
57128	Q85601	Q12982	0.26253
57129	Q85601	Q8NFT6	0.080342
57130	Q85601	Q9UBP6	0.78746
57131	Q85601	Q1ED39	0.40862
57132	Q85601	Q6ZWJ1	0.26891
57133	Q85601	Q13868	0.53805
57134	Q85601	Q5XKP0	0.17482
57135	Q85601	Q6ZMY9	0.46919
57136	Q85601	Q8TF20	0.34917
57137	Q85601	P23769	0.41287
57138	Q85601	Q6NVH7	0.61236
57139	Q85601	Q86TU7	0.29926
57140	Q85601	Q8NEK8	0.056455
57141	Q85601	P41970	0.91163
57142	Q85601	P30622	0.31687
57143	Q85601	Q9BUG6	0.1704
57144	Q85601	P42772	0.83753
57145	Q85601	P27635	0.61112
57146	Q85601	Q6PCD5	0.25357
57147	Q85601	Q9HC44	1.1837
57148	Q85601	Q9H825	0.22935
57149	Q85601	Q49MG5	1.0561
57150	Q85601	Q13415	0.27467
57151	Q85601	Q9Y6P5	0.12766
57152	Q85601	Q9NPI8	0.8558

Q85601	Q9H1A4	0.80113
Q85601	O60825	0.098147
Q85601	Q9UHV9	0.81008
Q85601	Q96MG7	0.8768
Q85601	Q15573	0.45563
Q85601	Q9BYD9	0.23763
Q85601	Q460N3	0.32411
Q85601	Q9Y2Y4	0.33186
Q85601	Q5TG08	0.27158
Q85601	P61326	0.832
Q85601	Q9NVM4	0.28518
Q85601	Q9H0E9	0.31576
Q85601	Q9Y2Y0	1.0662
Q85601	Q9P1Y5	0.47913
Q85601	P56555	0.45994
Q85601	A6NLJ0	0.20715
Q85601	Q9NYF0	0.25933
Q85601	Q9UBW8	0.45647
Q85601	Q2M2I5	0.65053
Q85601	Q9HCM1	0.078626
Q85601	Q969Z0	0.30153
Q85601	Q9Y5N6	0.54307
Q85601	Q9NWT6	0.46684
Q85601	O95789	0.28688
Q85601	P22626	0.40714
Q85601	Q5W1A9	0.52851
Q85601	Q12894	0.38673
Q85601	P62826	0.71799
Q85601	Q9P1A6	0.15593
Q85601	O60759	0.40385
Q85601	E9PKD4	0.018234
Q85601	Q9NR83	0.23802
Q85601	Q9NY30	0.39819
Q85601	Q86WB0	1.1662
Q85601	O15015	0.66443
Q85601	Q9H609	0.030777
Q85601	O15516	0.25817
Q85601	K7EIQ3	0.55422
Q85601	P78545	0.86389
Q85601	Q9UMS6	0.6286
Q85601	Q86WP2	0.51936
Q85601	O60220	0.35269
Q85601	O75437	0.47119
Q85601	Q1ZZU3	0.72103
Q85601	Q8IWE2	0.12713
Q85601	P61573	0.42275
Q85601	Q5SV97	0.15398
Q85601	P62324	0.68795
Q85601	Q13106	0.27099
Q85601	Q9UL41	0.38942
Q85601	P07438	0.28384
Q85601	O60810	0.7262
Q85601	Q7Z5W3	0.48406
Q85601	Q9BXD5	0.27456
Q85601	Q9Y2X9	0.66648
Q85601	O60613	0.37128
Q85601	Q9UHX1	0.50971
Q85601	Q9UKK3	0.68803
Q85601	Q9HAT8	0.55461
Q85601	Q86WZ6	0.25228
Q85601	Q6NUN9	0.7856
Q85601	Q9UJW7	0.25802
Q85601	Q96BY2	0.45037
Q85601	Q92844	0.52095
Q85601	Q9Y242	0.34999
Q85601	Q969T9	0.63313
Q85601	Q96PM5	0.52296
Q85601	Q8N888	0.70485
Q85601	Q7L5Y9	0.065327
Q85601	Q5XUX1	0.30691
Q85601	Q15287	0.86643
Q85601	O75420	0.46266
Q85601	Q12800	0.34317
Q85601	Q96PU4	0.42938
Q85601	Q8WUM0	0.6988
Q85601	Q9BS31	0.20185

57229	Q85601	Q9Y3A5	0.67777
57230	Q85601	Q16829	0.29411
57231	Q85601	Q5T7N2	0.10593
57232	Q85601	Q9H3P2	1.0803
57233	Q85601	B4DU55	0.3562
57234	Q85601	Q8N1F7	0.64329
57235	Q85601	Q93052	0.47173
57236	Q85601	Q658L1	0.35687
57237	Q85601	Q8WYA1	0.37626
57238	Q85601	Q9UER7	0.92678
57239	Q85601	Q8IVP9	0.19068
57240	Q85601	P12757	0.35694
57241	Q85601	Q8IYH5	0.59189
57242	Q85601	Q9BYC8	0.80739
57243	Q85601	O00148	0.31771
57244	Q85601	Q8IYJ2	0.030099
57245	Q85601	Q9Y3A2	0.8919
57246	Q85601	O14709	0.35685
57247	Q85601	Q96T51	0.21499
57248	Q85601	Q71F23	0.68614
57249	Q85601	O43865	0.18326
57250	Q85601	Q9HBL8	0.011802
57251	Q85601	Q14596	0.2581
57252	Q85601	O95678	0.48476
57253	Q85601	P63000	0.49747
57254	Q85601	Q5T681	0.30691
57255	Q85601	Q5TID7	0.22094
57256	Q85601	A6NE82	0.14127
57257	Q85601	Q8NAQ8	1.0544
57258	Q85601	Q96SQ5	0.30086
57259	Q85601	Q99728	0.32588
57260	Q85601	Q6ZNG1	0.3621
57261	Q85601	Q3KP31	0.44626
57262	Q85601	Q969H6	0.88988
57263	Q85601	P48200	0.44186
57264	Q85601	O75534	0.033036
57265	Q85601	Q9NVR2	0.98546
57266	Q85601	Q96PT3	0.17792
57267	Q85601	Q09FC8	0.36697
57268	Q85601	O75794	0.83607
57269	Q85601	Q9BYC9	0.67372
57270	Q85601	Q9H3M9	0.24397
57271	Q85601	Q9Y4L5	0.49757
57272	Q85601	Q96NL0	0.039649
57273	Q85601	Q9UBS4	0.49536
57274	Q85601	Q96IS3	0.23786
57275	Q85601	Q5U5X8	0.20879
57276	Q85601	Q9HC77	0.86436
57277	Q85601	Q5VWX1	0.63445
57278	Q85601	Q04637	0.47994
57279	Q85601	Q86V71	0.37553
57280	Q85601	P01011	0.46483
57281	Q85601	Q16589	0.40753
57282	Q85601	Q9Y5J7	0.31533
57283	Q85601	P82932	0.48697
57284	Q85601	P23381	0.23118
57285	Q85601	Q9BVN2	0.63997
57286	Q85601	Q52M93	0.22722
57287	Q85601	Q15545	0.95348
57288	Q85601	O60318	0.63842
57289	Q85601	Q9NZJ0	0.82491
57290	Q85601	Q6TGC4	0.18125
57291	Q85601	Q4KN68	0.14073
57292	Q85601	Q9BXM0	0.69575
57293	Q85601	Q96H86	0.30217
57294	Q85601	Q9BU76	0.10565
57295	Q85601	Q13616	0.56057
57296	Q85601	P15880	0.85397
57297	Q85601	Q8IXW0	0.43036
57298	Q85601	P17026	0.25896
57299	Q85601	Q8IZL8	0.87012
57300	Q85601	F5GYI3	0.44476
57301	Q85601	O43815	0.24741
57302	Q85601	Q9UMS0	1.1206
57303	Q85601	P53803	0.69977
57304	Q85601	P17022	0.15323

Q85601	Q66K74	0.20382
Q85601	Q9H4D5	0.34438
Q85601	Q8TDN6	0.33075
Q85601	P80303	0.21328
Q85601	Q9BQ48	0.2264
Q85601	Q6P050	0.35579
Q85601	Q8N5A5	0.88447
Q85601	O60284	0.29935
Q85601	P46379	0.25226
Q85601	Q8WU03	0.60461
Q85601	Q92610	0.13618
Q85601	Q9BTA9	0.98373
Q85601	Q6ZP68	0.3752
Q85601	Q9BZZ5	0.44011
Q85601	Q9ULH7	0.89664
Q85601	Q86VX9	1.0727
Q85601	Q7Z2W4	0.47974
Q85601	Q9BV86	0.37897
Q85601	O14686	0.4087
Q85601	Q9P0M9	0.20232
Q85601	Q8TAW3	0.29665
Q85601	Q8N8U2	0.43066
Q85601	P56537	1.1846
Q85601	Q86UU0	1.0294
Q85601	Q96KR1	0.27194
Q85601	Q16520	0.93729
Q85601	O95931	0.812
Q85601	Q9UBU8	0.80726
Q85601	Q2NKJ3	0.33247
Q85601	Q12981	0.11152
Q85601	Q9UMR2	0.18918
Q85601	O43264	0.62727
Q85601	Q9BTW9	0.19749
Q85601	Q14204	0.43908
Q85601	P42695	0.37827
Q85601	Q99504	0.63531
Q85601	P37837	0.21314
Q85601	Q9H0M5	0.25802
Q85601	P52744	0.28555
Q85601	Q14188	0.76874
Q85601	Q3KP66	0.52811
Q85601	A6NEF3	0.36009
Q85601	Q2TAK8	0.68972
Q85601	P36639	0.46161
Q85601	Q2M218	0.31208
Q85601	Q14738	0.73465
Q85601	Q7L2E3	0.26894
Q85601	Q8N4M7	0.52218
Q85601	Q8N9W8	0.61067
Q85601	Q6Y7W6	0.45625
Q85601	Q15723	1.2665
Q85601	Q12772	0.24967
Q85601	Q9H7Z6	0.23381
Q85601	O60936	0.64646
Q85601	Q9GZX7	0.3587
Q85601	P83881	0.77044
Q85601	Q15121	0.46765
Q85601	Q2TAZ0	0.30691
Q85601	Q49B96	0.21739
Q85601	Q96LX7	0.040277
Q85601	Q8IVE0	0.10052
Q85601	Q9BRQ6	0.9351
Q85601	Q16613	0.099648
Q85601	A7E2U8	0.29566
Q85601	Q9H9H4	0.55347
Q85601	P25490	0.26892
Q85601	Q8IZ26	0.31448
Q85601	Q9Y3C0	0.27462
Q85601	Q53HL2	1.003
Q85601	Q96G25	0.95395
Q85601	Q07020	0.67122
Q85601	P14136	0.4788
Q85601	P98170	0.2987
Q85601	Q6ZN55	0.2978
Q85601	Q86VN1	0.55443
Q85601	Q9UNL4	0.47794

Q85601	Q9Y5N5	0.27167
Q85601	Q6IPX3	0.69016
Q85601	Q8N895	0.53895
Q85601	Q8IXJ9	0.78478
Q85601	Q9P209	0.8423
Q85601	Q96ES7	0.1849
Q85601	Q9HCH5	0.62811
Q85601	O43364	0.21789
Q85601	Q9NRR4	0.40467
Q85601	Q9BY44	0.38911
Q85601	Q9BX82	0.46104
Q85601	Q5MJ10	0.17798
Q85601	Q71F56	0.41257
Q85601	Q9Y2S0	0.12685
Q85601	Q96NG5	0.43917
Q85601	O00327	0.24098
Q85601	Q6ICG8	0.98185
Q85601	Q8WXE9	0.55826
Q85601	Q96JB2	0.85702
Q85601	Q9BXS6	0.60089
Q85601	O60232	0.84357
Q85601	Q5VWQ0	0.73867
Q85601	Q5T8I9	0.18886
Q85601	P0C7X2	0.49647
Q85601	O43747	0.70114
Q85601	Q96IU4	0.47431
Q85601	Q96MY1	0.13475
Q85601	P30153	0.53282
Q85601	Q9Y2G2	0.43487
Q85601	Q9NPJ8	0.19087
Q85601	Q8WYA6	0.69048
Q85601	P61218	0.27725
Q85601	P61923	0.44012
Q85601	Q14157	0.20159
Q85601	Q5VTL8	0.13542
Q85601	Q8NF99	0.36056
Q85601	Q13045	1.1607
Q85601	P24522	0.58994
Q85601	Q9Y618	1.0493
Q85601	O00268	1.1466
Q85601	O94913	0.27748
Q85601	Q96RL7	0.79605
Q85601	Q9NWK9	0.29901
Q85601	P31948	0.98121
Q85601	P83436	0.45159
Q85601	P39019	0.75947
Q85601	Q96AY2	0.8436
Q85601	Q9H1H9	0.62043
Q85601	Q2QGD7	0.71271
Q85601	Q9Y6X9	0.40866
Q85601	Q9UPM8	0.2822
Q85601	O75792	0.70545
Q85601	O14503	0.90936
Q85601	Q7RTU4	0.35333
Q85601	Q8TDY2	0.90525
Q85601	O15083	0.55526
Q85601	Q6PJ61	0.61091
Q85601	Q15172	0.2938
Q85601	Q8IX12	0.93602
Q85601	Q8NDP4	0.33691
Q85601	Q96IR3	0.22107
Q85601	Q03252	0.32784
Q85601	Q9BSH3	0.18011
Q85601	Q13642	0.52476
Q85601	Q86XN6	0.34745
Q85601	Q9Y3E1	0.42546
Q85601	Q96CG3	0.31205
Q85601	Q8WW22	0.61984
Q85601	P35712	0.53342
Q85601	Q658K8	0.17164
Q85601	Q9Y376	0.20504
Q85601	Q9C099	1.0968
Q85601	Q96KD3	0.61067
Q85601	Q8WTW4	0.34112
Q85601	Q9H079	0.11712
Q85601	Q10587	0.31301

Q85601	Q9Y2Z2	0.4166
Q85601	P26641	0.3773
Q85601	Q9UIV1	1.2122
Q85601	P57075	0.36962
Q85601	Q14588	0.35685
Q85601	P06733	0.94057
Q85601	O43299	1.2455
Q85601	P08621	0.21791
Q85601	Q6ISB3	0.75682
Q85601	Q9UII5	0.34917
Q85601	P33316	0.62298
Q85601	Q8NG31	0.68072
Q85601	Q7Z2F6	0.36366
Q85601	Q5TB30	0.22332
Q85601	Q6IQ23	0.34738
Q85601	Q08378	0.42404
Q85601	Q5T653	0.12175
Q85601	P55145	0.52859
Q85601	Q5U5X0	0.28489
Q85601	Q9BRP4	0.39421
Q85601	Q96G21	0.87497
Q85601	Q15583	0.28862
Q85601	Q86X02	0.23754
Q85601	Q96QE5	0.84806
Q85601	Q86UD0	0.44273
Q85601	O43159	0.86375
Q85601	Q9H4T2	0.21005
Q85601	Q969L4	0.54521
Q85601	Q96JC4	0.34256
Q85601	Q9Y3U8	0.85824
Q85601	Q9GZQ3	0.74748
Q85601	Q96PU5	0.26407
Q85601	Q9UNX4	0.32032
Q85601	P11532	0.55083
Q85601	O95243	0.78481
Q85601	Q71UM5	0.64925
Q85601	P10242	0.96031
Q85601	Q5SRH9	0.2768
Q85601	Q9BYR5	0.17342
Q85601	Q00059	0.88259
Q85601	Q8IVV7	0.1691
Q85601	Q15173	0.358
Q85601	Q96S95	0.34448
Q85601	Q9P013	0.34749
Q85601	Q9BQ15	0.69149
Q85601	Q9NR50	0.52538
Q85601	Q969T4	0.66163
Q85601	Q8TF40	0.67907
Q85601	Q9NR28	0.83878
Q85601	Q8TBZ8	0.33231
Q85601	Q8IZS6	0.10543
Q85601	Q6NXT1	0.44793
Q85601	Q15306	0.88646
Q85601	Q8WXF1	0.87826
Q85601	Q9Y296	0.24464
Q85601	P46108	0.47433
Q85601	Q14592	0.31944
Q85601	Q9GZV5	1.0516
Q85601	Q9NP61	0.15994
Q85601	P61758	0.62383
Q85601	A6NFQ7	0.32996
Q85601	Q9UBU7	0.18355
Q85601	Q5JWF8	0.059966
Q85601	Q6ZQR2	0.95851
Q85601	Q86VP6	0.73502
Q85601	A6NK53	0.32578
Q85601	P61970	0.43591
Q85601	O15078	0.64765
Q85601	Q9NQZ8	0.29598
Q85601	Q9Y248	0.68519
Q85601	Q9BZR9	0.36094
Q85601	Q9NVF7	0.15623
Q85601	O14497	0.65923
Q85601	Q99767	0.26494
Q85601	Q7Z6G3	0.42435
Q85601	Q14591	0.22599

Q85601	Q14764	1.0478
Q85601	P0CV98	0.47415
Q85601	Q9Y5B9	0.33569
Q85601	Q9H974	1.1119
Q85601	O96019	0.95333
Q85601	Q9NUQ6	0.44273
Q85601	O00151	0.32086
Q85601	P18846	0.89421
Q85601	Q5SWA1	0.39244
Q85601	Q49AM1	0.38793
Q85601	Q14135	0.45278
Q85601	A1L020	0.24871
Q85601	Q9UBD5	0.51828
Q85601	Q8N1L9	0.82627
Q85601	Q8N8H1	0.43069
Q85601	O00757	0.052019
Q85601	Q14669	0.37872
Q85601	Q9NWF9	0.90252
Q85601	Q9BYN8	0.45531
Q85601	O43633	0.35918
Q85601	Q14106	0.39383
Q85601	Q96B36	0.82412
Q85601	P58317	0.32159
Q85601	Q5TAQ9	0.26501
Q85601	Q9BWD3	0.17478
Q85601	Q9BYP9	0.17342
Q85601	Q9NQ33	0.54057
Q85601	Q9UGP5	0.15546
Q85601	O60884	0.61098
Q85601	P62701	0.42389
Q85601	Q9C0F3	0.4507
Q85601	P06744	0.5954
Q85601	Q6ZRM9	0.07997
Q85601	Q9NRX3	0.28656
Q85601	Q99814	0.39512
Q85601	O15392	0.31084
Q85601	P62241	0.63007
Q85601	P47974	0.31317
Q85601	P82675	0.18591
Q85601	Q86WI3	0.52892
Q85601	P62424	0.96913
Q85601	Q8TF32	0.47998
Q85601	P27797	0.49982
Q85601	P16383	0.56205
Q85601	Q9ULR5	0.48638
Q85601	Q15046	0.4088
Q85601	P49959	0.21375
Q85601	P27540	0.45254
Q85601	Q15776	0.54641
Q85601	A8MQ14	0.33896
Q85601	Q86XK2	0.84447
Q85601	Q02833	0.27688
Q85601	O15226	0.68555
Q85601	Q3MIS6	0.4637
Q85601	Q3KQV3	0.35068
Q85601	O75290	0.4755
Q85601	Q9NR56	0.40669
Q85601	Q8N8D1	0.21225
Q85601	P53999	1.2563
Q85601	Q96SE7	0.34313
Q85601	Q13363	0.4173
Q85601	P0CG48	0.38014
Q85601	Q8IUW3	0.51618
Q85601	B4DX44	0.52111
Q85601	Q9BRS8	0.28869
Q85601	P49005	0.57237
Q85601	Q7Z614	0.46832
Q85601	Q8IUD6	0.45959
Q85601	P21673	0.24146
Q85601	Q92552	0.68709
Q85601	D6RGH6	1.2222
Q85601	Q9P242	0.49979
Q85601	O43678	0.46284
Q85601	P30304	0.61658
Q85601	O75448	0.49069
Q85601	P11171	0.54943

57609	Q85601	P26358	0.41192
57610	Q85601	Q9HAW0	0.5505
57611	Q85601	Q15386	0.27509
57612	Q85601	Q8IUE0	0.32996
57613	Q85601	Q86V81	0.66105
57614	Q85601	Q9UJJ7	0.29565
57615	Q85601	Q8NA19	0.41299
57616	Q85601	O94888	0.41463
57617	Q85601	P19338	0.47871
57618	Q85601	Q9P2F9	0.23029
57619	Q85601	O43776	0.44735
57620	Q85601	Q6NSI3	0.27847
57621	Q85601	Q92834	0.34976
57622	Q85601	Q7L5D6	0.69734
57623	Q85601	Q9Y3D9	0.40077
57624	Q85601	O14646	0.68604
57625	Q85601	Q5BJF6	0.54742
57626	Q85601	Q8N402	0.013005
57627	Q85601	P04259	0.29484
57628	Q85601	Q9Y3M9	0.21995
57629	Q85601	P17038	0.35363
57630	Q85601	Q13469	1.1836
57631	Q85601	O95155	0.2281
57632	Q85601	Q9P255	0.3582
57633	Q85601	Q92831	0.83185
57634	Q85601	O14519	0.21194
57635	Q85601	Q5SWL7	0.40413
57636	Q85601	Q7Z2X4	0.2584
57637	Q85601	O00303	0.28168
57638	Q85601	Q9UBI1	0.30691
57639	Q85601	Q9Y2I6	0.2286
57640	Q85601	Q5VIR6	0.83133
57641	Q85601	Q86Y37	0.096562
57642	Q85601	P49006	0.5503
57643	Q85601	Q9H3R5	0.56057
57644	Q85601	Q9BUJ2	0.98171
57645	Q85601	A2AJT9	0.40717
57646	Q85601	P83731	0.67535
57647	Q85601	Q9NS23	0.46717
57648	Q85601	Q96FX7	0.26475
57649	Q85601	Q9ULE4	0.38313
57650	Q85601	Q9UMX3	0.5441
57651	Q85601	Q14203	1.0161
57652	Q85601	P49754	0.55242
57653	Q85601	Q14674	0.34323
57654	Q85601	Q96DX5	1.0999
57655	Q85601	Q6ZP65	0.12603
57656	Q85601	Q14690	0.50624
57657	Q85601	Q01082	0.4779
57658	Q85601	A6NM28	0.24786
57659	Q85601	O60516	0.2404
57660	Q85601	P55795	0.044924
57661	Q85601	Q6FI81	0.46181
57662	Q85601	Q7LBR1	0.93272
57663	Q85601	O75494	0.48444
57664	Q85601	Q6PK81	0.30557
57665	Q85601	Q9H0W9	0.68688
57666	Q85601	O60682	0.32532
57667	Q85601	Q9BTT0	0.24373
57668	Q85601	Q96B02	0.34744
57669	Q85601	Q99549	0.70898
57670	Q85601	P59037	0.40089
57671	Q85601	Q7RTU5	0.41
57672	Q85601	Q9BW66	0.95888
57673	Q85601	Q00978	0.99137
57674	Q85601	P17987	0.28891
57675	Q85601	P07199	0.6359
57676	Q85601	Q96CW5	0.922
57677	Q85601	Q66LE6	0.45703
57678	Q85601	Q6ZN06	0.47911
57679	Q85601	Q9Y3C7	0.90947
57680	Q85601	Q2VPK5	0.72283
57681	Q85601	O76075	0.11089
57682	Q85601	P17031	0.33241
57683	Q85601	Q92562	0.31444
57684	Q85601	Q96AQ7	0.56781

Q85601	Q8IWY8	0.30203
Q85601	Q9UPV9	0.78103
Q85601	Q7Z745	0.068541
Q85601	Q15653	0.99871
Q85601	P17014	0.26841
Q85601	P51948	0.49986
Q85601	P56181	0.31992
Q85601	Q9NZN8	0.78474
Q85601	O75940	0.54579
Q85601	P63241	0.42065
Q85601	P61024	0.64091
Q85601	Q96MU6	0.25692
Q85601	P59990	0.17342
Q85601	Q8N2W9	1.179
Q85601	Q96EA4	0.81946
Q85601	Q5T1R4	0.26895
Q85601	P0DMB1	0.12166
Q85601	P23297	0.54937
Q85601	Q8TDN4	0.65176
Q85601	Q9BYD2	0.18773
Q85601	P19532	0.80405
Q85601	Q9NZQ3	0.68539
Q85601	Q53HC0	0.6391
Q85601	O15164	0.30384
Q85601	P10074	0.43473
Q85601	P27469	0.65292
Q85601	Q9ULW0	0.90726
Q85601	P78337	0.47953
Q85601	P18615	0.85075
Q85601	Q9UHD4	0.60783
Q85601	P62899	0.53692
Q85601	Q9H0F6	0.16715
Q85601	Q8NEP9	0.4755
Q85601	Q5VVH5	0.67368
Q85601	O60551	0.012632
Q85601	Q9NRY5	0.7954
Q85601	Q9NPD8	0.70402
Q85601	P52594	0.71285
Q85601	Q86SQ9	0.037422
Q85601	Q12815	0.32326
Q85601	P51959	0.46954
Q85601	Q9NV72	0.49058
Q85601	Q49A33	0.42548
Q85601	P41002	1.0265
Q85601	P50749	0.77142
Q85601	O60216	0.81022
Q85601	Q9UJN7	0.29276
Q85601	P25685	0.35605
Q85601	Q92499	0.39211
Q85601	Q9BW92	0.18872
Q85601	Q8WUH2	0.68878
Q85601	Q86SE9	1.15
Q85601	Q15560	1.1072
Q85601	O43439	0.99403
Q85601	Q92698	0.325
Q85601	P20042	0.40394
Q85601	P13196	0.31075
Q85601	Q15742	0.36508
Q85601	P21953	0.044157
Q85601	P52565	0.076593
Q85601	O60732	0.30691
Q85601	Q8WVM7	0.79622
Q85601	Q9UJ41	0.33721
Q85601	Q9UFF9	0.52259
Q85601	Q9Y244	0.43351
Q85601	Q6P1K8	0.60791
Q85601	Q13144	0.51759
Q85601	Q5TA31	0.6271
Q85601	A6NEJ1	0.1323
Q85601	Q6PIJ6	0.34036
Q85601	P0CG34	0.34646
Q85601	Q16594	0.59884
Q85601	P02795	0.67625
Q85601	P12270	0.66547
Q85601	Q86VD1	0.47469
Q85601	Q9H6Y2	0.45754

Q85601	Q9BX26	1.2608
Q85601	Q7KZF4	0.9223
Q85601	P42166	0.1051
Q85601	Q9BT92	0.6393
Q85601	Q96D15	0.2366
Q85601	Q9NYJ1	0.60066
Q85601	Q6NT76	0.53051
Q85601	Q9HAH1	0.41282
Q85601	Q16385	0.75053
Q85601	Q9ULE0	0.025837
Q85601	Q8WZ33	0.9452
Q85601	P62945	0.67535
Q85601	O95801	0.59067
Q85601	Q08AN1	0.35685
Q85601	P05062	0.25209
Q85601	Q9NZV7	0.5777
Q85601	Q9NSK7	0.69694
Q85601	Q6PF18	0.10745
Q85601	Q9HD34	0.34692
Q85601	Q96JJ3	0.22638
Q85601	P05423	0.011362
Q85601	O43639	0.40857
Q85601	Q16629	0.5623
Q85601	Q13114	0.4463
Q85601	O75934	0.63461
Q85601	Q99743	0.46562
Q85601	Q9UHD9	0.84611
Q85601	P06454	0.55036
Q85601	Q8IZT6	0.44049
Q85601	Q7Z3Y9	0.65053
Q85601	Q9BYQ3	0.17342
Q85601	Q6P5X5	0.46258
Q85601	Q15596	0.66539
Q85601	Q10567	0.66655
Q85601	Q9Y4B6	0.91845
Q85601	O00472	0.60012
Q85601	Q7Z6I5	0.70261
Q85601	P32189	0.12021
Q85601	Q5VSL9	0.81542
Q85601	O14972	0.33489
Q85601	Q03923	0.5423
Q85601	Q8N3X1	0.23708
Q85601	O43791	0.54465
Q85601	Q9H6Z4	1.126
Q85601	P52736	0.64331
Q85601	O95983	1.2724
Q85601	Q9NRY2	0.82948
Q85601	P63172	0.88034
Q85601	P0CI01	0.15217
Q85601	Q99608	0.19284
Q85601	O43303	0.88925
Q85601	O95503	0.9346
Q85601	P56211	0.18549
Q85601	O95644	0.867
Q85601	Q9BRP8	0.84437
Q85601	Q8N823	0.35685
Q85601	Q9Y285	0.32126
Q85601	Q6H8Q1	0.4492
Q85601	Q9BTE1	0.2393
Q85601	O14786	0.26941
Q85601	Q9BZV1	1.1103
Q85601	Q92782	0.72731
Q85601	C9J3I9	0.24699
Q85601	Q9P2W9	0.23007
Q85601	Q4G0F5	0.22527
Q85601	Q6P2H3	0.66448
Q85601	Q9UK45	0.64797
Q85601	P17024	0.34274
Q85601	Q14149	0.2444
Q85601	O96004	0.49151
Q85601	Q8WUW1	0.14428
Q85601	P17017	0.25157
Q85601	Q15714	0.80572
Q85601	Q5JY77	0.53217
Q85601	Q9H714	0.32406
Q85601	P49321	0.54318

Q85601	Q5VUG0	0.33843
Q85601	Q8IYT1	0.16823
Q85601	Q9Y692	0.81539
Q85601	Q96PV6	0.74358
Q85601	O75886	1.1736
Q85601	O14744	0.95954
Q85601	Q2VWA4	0.14677
Q85601	Q9BQ89	0.69883
Q85601	Q9NTI5	0.88649
Q85601	F5H4B4	0.27158
Q85601	Q08211	0.2283
Q85601	P62316	0.73261
Q85601	Q9BZL4	0.53217
Q85601	Q15054	0.62168
Q85601	Q9GZS1	0.46613
Q85601	Q9BR76	0.30241
Q85601	O94906	0.18673
Q85601	A6NKF1	0.98946
Q85601	P41162	0.34071
Q85601	P51668	0.5612
Q85601	P48147	0.099878
Q85601	O60304	0.26172
Q85601	Q29RF7	1.0294
Q85601	P60372	0.17342
Q85601	P35237	0.4028
Q85601	Q7Z401	0.33155
Q85601	Q9UJY5	0.51602
Q85601	Q9Y2D9	0.66443
Q85601	Q15436	0.70253
Q85601	Q8WXU2	0.76138
Q85601	P59923	0.35685
Q85601	Q8N2I2	0.32741
Q85601	Q96FX2	0.96174
Q85601	Q8NDM7	0.47911
Q85601	P31350	0.34814
Q85601	P49247	0.40225
Q85601	Q00613	0.88771
Q85601	A6NKU9	0.15217
Q85601	Q86V48	0.41133
Q85601	Q13398	0.45392
Q85601	Q9HAN9	0.26242
Q85601	Q9BYR2	0.17342
Q85601	Q53GI3	0.33218
Q85601	Q9UK11	0.38013
Q85601	Q96LR5	0.68983
Q85601	Q2KJ03	0.22952
Q85601	O15525	0.3792
Q85601	Q13867	0.34721
Q85601	P68036	0.74875
Q85601	O15405	1.0093
Q85601	Q9Y3E5	0.54486
Q85601	Q07955	0.4732
Q85601	Q8IYE0	0.45506
Q85601	Q9BYR0	0.17342
Q85601	Q86UD4	0.23314
Q85601	P49770	0.24497
Q85601	O43463	0.83832
Q85601	P80723	0.26754
Q85601	Q15651	0.75901
Q85601	Q9NP71	0.54876
Q85601	P55055	0.34325
Q85601	Q04864	1.2378
Q85601	P20290	1.1199
Q85601	P17010	0.35675
Q85601	P49711	0.28095
Q85601	Q93008	0.47433
Q85601	Q9UID6	0.52887
Q85601	P55769	0.72149
Q85601	O43345	0.34917
Q85601	Q9UIH9	0.44148
Q85601	Q9BZE9	0.53308
Q85601	Q8TAF7	0.44666
Q85601	Q96R06	1.025
Q85601	Q15059	1.1508
Q85601	Q13952	0.63635
Q85601	Q96BW9	0.046395

Q85601	Q8WWB5	0.39346
Q85601	Q8N612	0.33981
Q85601	Q9H008	0.50987
Q85601	Q658Y4	0.11028
Q85601	Q9H1B4	0.34963
Q85601	Q8N184	0.22783
Q85601	Q9BSV6	0.12236
Q85601	Q92600	1.202
Q85601	P0CW00	0.47415
Q85601	Q9NZP6	0.73813
Q85601	O75694	0.65755
Q85601	Q9H089	0.065354
Q85601	Q9BUY5	0.38602
Q85601	Q8TF47	0.44666
Q85601	Q9NVU0	0.34399
Q85601	O95278	0.24408
Q85601	Q04917	0.2928
Q85601	Q9C0E2	0.9044
Q85601	A6NNV3	0.15217
Q85601	Q9NWT8	0.8447
Q85601	Q9H7H0	0.65762
Q85601	Q96GX9	0.39127
Q85601	O43617	0.3729
Q85601	Q9BV57	0.67387
Q85601	Q8WYR4	0.40293
Q85601	O43745	0.75611
Q85601	Q6A162	0.49362
Q85601	P24534	0.56684
Q85601	Q9NSQ0	0.28946
Q85601	P61077	0.84493
Q85601	O75474	0.685
Q85601	P59998	0.10187
Q85601	Q13427	0.42908
Q85601	Q9P2R7	0.18355
Q85601	Q9UJP4	0.34819
Q85601	Q9UJL9	0.25255
Q85601	P63279	0.95217
Q85601	Q504Q3	0.20317
Q85601	Q9H777	0.17836
Q85601	Q9H095	0.13285
Q85601	P16118	0.048629
Q85601	P02100	0.21071
Q85601	P27694	0.59008
Q85601	O14776	1.1056
Q85601	Q6ZRQ5	0.63646
Q85601	O94817	0.51065
Q85601	Q96AQ9	0.35099
Q85601	Q8IYI8	0.37155
Q85601	O43542	0.62826
Q85601	Q9NYW8	0.23651
Q85601	Q8WUB8	0.49575
Q85601	P51610	0.52523
Q85601	A1L3Z2	0.1242
Q85601	O75953	0.72315
Q85601	Q14145	1.3273
Q85601	Q00597	0.87279
Q85601	Q96FI4	0.046803
Q85601	A6NI86	0.53871
Q85601	A8MXY4	0.35363
Q85601	Q9NWB6	0.45278
Q85601	Q9UBB9	0.32033
Q85601	Q7Z3Y7	0.43373
Q85601	Q02080	0.43699
Q85601	P62263	0.45907
Q85601	Q9P015	0.3236
Q85601	P15259	0.68269
Q85601	Q96EN9	0.30691
Q85601	Q9BQI9	0.60265
Q85601	Q6ZSR6	0.092509
Q85601	O14508	0.30918
Q85601	Q5VT03	0.052748
Q85601	Q9NQ55	0.39508
Q85601	O00198	0.11728
Q85601	Q12834	0.80713
Q85601	P12755	0.18972
Q85601	Q9UHA3	0.29583

Q85601	P08697	0.48679
Q85601	Q13541	0.82449
Q85601	Q9NZ81	0.7685
Q85601	Q9GZS3	1.1737
Q85601	Q96FA3	0.50393
Q85601	Q9Y2L1	0.47911
Q85601	P62861	0.78596
Q85601	O95125	0.29137
Q85601	Q8IVD9	0.27808
Q85601	Q8N3L3	0.64397
Q85601	Q96FZ7	0.0591
Q85601	Q8N0T1	0.060104
Q85601	Q9ULU4	0.62624
Q85601	Q8IYB7	0.1794
Q85601	Q9H9C1	0.58292
Q85601	Q8TC90	0.053868
Q85601	Q12830	1.0786
Q85601	Q3MHD2	0.3103
Q85601	Q8N6M0	0.11358
Q85601	Q8WTW3	0.49486
Q85601	Q5VWP3	0.34989
Q85601	Q8NCD3	0.5611
Q85601	Q02878	0.92243
Q85601	Q70YC4	0.10442
Q85601	Q9Y5Q3	0.84634
Q85601	Q8TC94	0.09652
Q85601	Q9NS25	0.22764
Q85601	Q9BYD6	0.13092
Q85601	O75607	0.68022
Q85601	Q09161	0.66542
Q85601	Q5JTW2	0.41939
Q85601	O96015	0.095656
Q85601	Q9Y6E7	0.45303
Q85601	P30793	0.21691
Q85601	P0DM35	0.61713
Q85601	H3BNL1	0.06625
Q85601	Q66K41	0.80902
Q85601	P0C1Z6	1.2944
Q85601	Q8IZX4	0.36401
Q85601	Q6TFL3	0.24285
Q85601	Q499Z4	0.36859
Q85601	Q9HA77	0.15236
Q85601	O15504	0.64926
Q85601	O95273	0.95972
Q85601	Q13404	1.075
Q85601	Q14974	1.1196
Q85601	O15040	0.30691
Q85601	P30047	0.68998
Q85601	Q86YV9	0.52102
Q85601	Q9H7R0	0.45863
Q85601	Q9UQF2	0.4945
Q85601	Q9BV47	0.43786
Q85601	Q9UBG7	0.20316
Q85601	Q9Y6J9	0.80676
Q85601	Q8WX92	1.475
Q85601	P10914	0.42804
Q85601	Q9Y6H1	0.45749
Q85601	Q9NQG5	1.1118
Q85601	Q6VN20	0.41722
Q85601	P08047	0.81026
Q85601	Q5VWG9	0.8798
Q85601	Q96DI7	0.56944
Q85601	Q12888	0.83329
Q85601	Q9NRF9	0.049858
Q85601	Q8IWE5	0.40289
Q85601	Q8TEP8	0.84777
Q85601	Q96E09	0.15623
Q85601	Q9NNW5	0.52681
Q85601	Q969R5	0.2939
Q85601	Q8N6M9	0.25399
Q85601	Q99623	1.1373
Q85601	Q86Y26	0.23411
Q85601	Q9H6W3	0.38183
Q85601	P11802	0.71107
Q85601	Q9BZQ8	0.27536
Q85601	Q8WUX9	0.54483

58065	Q85601	Q13257	0.7841
58066	Q85601	Q8NDW8	0.07941
58067	Q85601	Q6PK04	0.7463
58068	Q85601	P30154	0.29047
58069	Q85601	O60806	0.46238
58070	Q85601	P27986	0.51594
58071	Q85601	Q9H267	0.27986
58072	Q85601	Q9HC78	0.25392
58073	Q85601	Q8IZH2	0.17653
58074	Q85601	Q86TB9	0.81298
58075	Q85601	Q9UM82	0.70127
58076	Q85601	O94829	1.0478
58077	Q85601	Q12840	0.32423
58078	Q85601	P53370	0.52773
58079	Q85601	O43156	0.30691
58080	Q85601	Q8N9F8	0.3341
58081	Q85601	Q6IQ21	0.38106
58082	Q85601	P54252	0.4911
58083	Q85601	Q9UH92	0.9312
58084	Q85601	O43663	0.93053
58085	Q85601	Q7Z3C6	0.18695
58086	Q85601	Q9H501	0.83262
58087	Q85601	O43752	0.10883
58088	Q85601	P07737	0.86799
58089	Q85601	Q8N5C8	0.51487
58090	Q85601	O75152	0.30436
58091	Q85601	Q9UKN8	0.54113
58092	Q85601	Q9H0I3	0.50191
58093	Q85601	Q96MT3	1.023
58094	Q85601	Q8IWP9	0.56423
58095	Q85601	P60412	0.17342
58096	Q85601	Q08209	0.42864
58097	Q85601	P07954	0.13144
58098	Q85601	Q9H9Y6	0.73346
58099	Q85601	Q01954	0.23811
58100	Q85601	O14598	0.29282
58101	Q85601	O95201	0.66443
58102	Q85601	P52630	0.75059
58103	Q85601	P82979	0.37644
58104	Q85601	Q96D70	0.18591
58105	Q85601	Q9HAU0	0.22989
58106	Q85601	P80217	0.68462
58107	Q85601	P55771	0.86377
58108	Q85601	O95707	0.66791
58109	Q85601	P39023	0.91979
58110	Q85601	Q5VIY5	0.48095
58111	Q85601	Q96NY9	0.73144
58112	Q85601	Q92889	0.28062
58113	Q85601	Q9UKC9	0.53813
58114	Q85601	Q5VXH4	0.39986
58115	Q85601	Q9H5Z6	0.30691
58116	Q85601	Q9UBW7	0.74644
58117	Q85601	Q8NE18	0.98223
58118	Q85601	P38919	0.1849
58119	Q85601	P60709	0.1273
58120	Q85601	Q9UFW8	0.42685
58121	Q85601	P23258	0.33875
58122	Q85601	Q9NPC6	0.44329
58123	Q85601	Q5VTH2	0.50188
58124	Q85601	P61254	0.92236
58125	Q85601	Q6XQN6	0.28493
58126	Q85601	Q96C86	0.91904
58127	Q85601	Q9BWU0	0.51865
58128	Q85601	Q8TAP9	1.55
58129	Q85601	Q5H9L2	1.0775
58130	Q85601	Q5VSY0	0.42807
58131	Q85601	P51531	0.70188
58132	Q85601	Q9H944	1.0147
58133	Q85601	O14757	0.58553
58134	Q85601	P26651	0.72892
58135	Q85601	Q13562	0.24578
58136	Q85601	Q96CF2	0.25144
58137	Q85601	O43399	0.5636
58138	Q85601	P14678	0.71073
58139	Q85601	O15371	0.65807
58140	Q85601	Q9Y592	0.34831

Q85601	Q96AJ1	0.71342
Q85601	A8MQ03	0.51676
Q85601	Q96QA6	0.22094
Q85601	Q9P219	0.44686
Q85601	P01100	0.52602
Q85601	Q9NXW9	0.050174
Q85601	Q6UYE1	0.44165
Q85601	Q16587	0.27674
Q85601	Q9NP77	0.26877
Q85601	Q96LY2	0.26137
Q85601	Q9UKA1	0.2751
Q85601	Q8NG57	0.28453
Q85601	O75155	0.76094
Q85601	Q96L92	0.75331
Q85601	Q5TC12	0.43305
Q85601	Q9Y2R9	0.52881
Q85601	Q9P2K3	1.2253
Q85601	Q7Z5K2	0.80067
Q85601	Q96ST8	0.95843
Q85601	Q9HBM6	0.66365
Q85601	Q15572	0.83482
Q85601	Q96EY4	0.025737
Q85601	O14734	0.19758
Q85601	Q15022	0.66823
Q85601	O75167	0.16374
Q85601	Q7Z589	0.72806
Q85601	O75943	0.27475
Q85601	Q05481	0.35363
Q85601	Q92674	0.39777
Q85601	Q08752	0.34797
Q85601	Q9H869	1.4396
Q85601	Q05BW5	0.16675
Q85601	Q14934	0.52688
Q85601	Q86W54	0.27647
Q85601	Q8N6I1	0.7162
Q85601	O15370	0.43108
Q85601	P25789	0.56503
Q85601	O00170	0.71442
Q85601	Q8N1E6	0.37168
Q85601	Q13263	0.24788
Q85601	P15924	0.28681
Q85601	P28715	0.33211
Q85601	Q9H6Q4	0.36939
Q85601	O60291	0.57057
Q85601	Q8TE69	0.30691
Q85601	Q76KD6	0.1715
Q85601	Q8NG50	0.08841
Q85601	Q9BSJ6	0.50114
Q85601	P11387	0.66195
Q85601	Q68DI1	0.20591
Q85601	Q53H80	0.40153
Q85601	Q8IVN3	0.65462
Q85601	Q8NHW3	0.30267
Q85601	Q63HM1	0.31838
Q85601	Q8TCI5	0.64143
Q85601	P52746	0.15724
Q85601	P41212	0.79031
Q85601	Q9UQB3	0.56667
Q85601	Q9GZY1	0.57995
Q85601	O15060	0.10687
Q85601	O95397	0.78981
Q85601	Q86VY4	0.49249
Q85601	Q15397	0.10584
Q85601	Q9BXF9	0.17482
Q85601	P41250	0.26219
Q85601	Q13405	0.60679
Q85601	Q8N7W2	0.30709
Q85601	O43516	0.26053
Q85601	Q14526	0.61919
Q85601	Q5HYW3	0.47423
Q85601	P0C7T5	0.31707
Q85601	Q7Z794	0.60912
Q85601	Q9NS73	0.7671
Q85601	Q5U623	0.86074
Q85601	Q13137	1.153
Q85601	Q8IYS8	0.16318

Q85601	Q8N831	0.72964
Q85601	Q6IQ32	0.46356
Q85601	Q8N0Z6	0.40068
Q85601	Q96MR7	1.398
Q85601	O60716	0.50682
Q85601	Q96FV9	0.7404
Q85601	Q96GM5	0.70305
Q85601	O75820	0.25157
Q85601	P22415	0.48296
Q85601	O00233	0.49743
Q85601	Q9P267	0.30676
Q85601	Q9NRR3	0.57279
Q85601	O96000	0.38937
Q85601	P56277	0.20355
Q85601	P62487	0.34189
Q85601	O75122	0.34746
Q85601	Q16222	0.26636
Q85601	Q9UNH5	0.72671
Q85601	Q8N6H7	0.095847
Q85601	P51398	1.174
Q85601	Q9H0B3	0.35296
Q85601	O60294	0.31922
Q85601	Q8NEA6	0.27834
Q85601	Q86WQ0	0.49427
Q85601	Q13515	0.52501
Q85601	Q3ZCX4	0.31466
Q85601	A8MSI8	1.0161
Q85601	O60518	0.97198
Q85601	Q9Y675	0.11206
Q85601	Q9NVK5	0.17272
Q85601	Q9Y6R6	0.35685
Q85601	Q5VZQ5	0.16431
Q85601	Q9H0A9	0.87042
Q85601	Q8TF50	0.31798
Q85601	Q14566	0.19588
Q85601	P0CJ79	0.34347
Q85601	O96020	0.96446
Q85601	Q9BVC4	0.41749
Q85601	A6NFY7	0.12949
Q85601	Q9H9A7	0.58122
Q85601	Q9GZX5	0.85087
Q85601	Q14CN4	0.38819
Q85601	P30305	0.44078
Q85601	Q16633	0.6041
Q85601	Q14686	0.83382
Q85601	Q8NEY3	0.24738
Q85601	Q9BQY4	0.83988
Q85601	P01009	0.58305
Q85601	Q9UBL3	0.37294
Q85601	Q14677	0.52978
Q85601	O43361	0.23119
Q85601	O14965	0.32114
Q85601	Q96B67	0.32317
Q85601	Q9BRT9	0.32153
Q85601	Q9NW07	0.29941
Q85601	Q8WYQ5	0.61385
Q85601	Q92688	0.23411
Q85601	Q9BY77	0.34382
Q85601	Q9H4I2	0.80688
Q85601	Q9Y266	0.81943
Q85601	B5ME19	0.10003
Q85601	Q9H0G5	0.47357
Q85601	Q5SXM2	0.63642
Q85601	Q08AD1	0.55282
Q85601	O60870	0.76742
Q85601	Q13503	0.67458
Q85601	P36508	0.085001
Q85601	Q9Y3D3	0.61351
Q85601	P19388	0.64744
Q85601	Q9NP66	1.0098
Q85601	Q5T160	0.58369
Q85601	Q8TC21	0.3052
Q85601	Q9BVC3	0.57287
Q85601	Q9UPV0	1.674
Q85601	P29084	1.2529
Q85601	A6ZKI3	0.014132

Q85601	P43360	0.39393
Q85601	Q6ZW54	0.57078
Q85601	Q9BWT1	0.54914
Q85601	Q8NBZ0	1.3122
Q85601	Q86WV1	0.63596
Q85601	P49207	0.56747
Q85601	P60228	0.96807
Q85601	O75817	0.86917
Q85601	Q9UMX2	0.23662
Q85601	O75818	0.31289
Q85601	Q8NI35	0.30687
Q85601	Q9BXW4	0.28257
Q85601	Q6PJT7	0.96236
Q85601	P31260	0.3006
Q85601	Q16851	0.42688
Q85601	Q7Z3K3	0.57886
Q85601	P23409	0.061646
Q85601	Q9BW85	0.57104
Q85601	Q9H2J4	0.64168
Q85601	Q8TCE6	0.63405
Q85601	P35637	0.36552
Q85601	Q8IWL8	0.33985
Q85601	O75570	0.15733
Q85601	Q96KQ4	0.93945
Q85601	Q86WR7	0.002423
Q85601	Q9UK12	0.29749
Q85601	O43681	0.076137
Q85601	Q96F83	0.38237
Q85601	Q8NHZ7	0.35546
Q85601	Q6MZT1	0.16673
Q85601	Q8TEB1	0.26501
Q85601	P10599	0.36373
Q85601	Q96HQ2	0.51797
Q85601	P11940	0.30426
Q85601	Q9XRX5	0.30691
Q85601	Q9NX65	0.25071
Q85601	O60296	0.55208
Q85601	Q5VY09	0.10715
Q85601	Q14186	1.2123
Q85601	P50222	0.60616
Q85601	Q9UII2	0.29944
Q85601	Q5T215	0.25485
Q85601	Q53T94	0.43283
Q85601	Q7Z6J0	0.37823
Q85601	Q8NDQ6	0.36823
Q85601	P51508	0.31208
Q85601	Q9Y5Q8	0.68797
Q85601	Q86XR8	1.0712
Q85601	Q96GC5	0.60679
Q85601	Q9Y6B2	1.0017
Q85601	Q8WYN3	0.54068
Q85601	Q92830	0.7091
Q85601	Q9H2C0	0.62105
Q85601	Q9BYQ0	0.17342
Q85601	Q5HYW2	0.10358
Q85601	Q9UKX2	0.51716
Q85601	O76003	0.24797
Q85601	Q9UH99	0.17456
Q85601	Q9BPX3	0.95902
Q85601	Q86XL3	0.34348
Q85601	Q9UK13	0.4637
Q85601	O75444	0.46027
Q85601	Q04725	0.86798
Q85601	Q9Y291	0.32562
Q85601	Q9Y2K3	0.51716
Q85601	Q9Y316	0.18002
Q85601	Q8NFJ8	0.41298
Q85601	Q9Y512	0.070431
Q85601	P60411	0.17342
Q85601	P40424	0.68028
Q85601	Q96TA1	0.37785
Q85601	Q96Q89	0.5345
Q85601	Q96LK0	0.082628
Q85601	Q14653	0.64119
Q85601	Q14164	0.256
Q85601	Q9C0C6	0.42921

Q85601	Q9Y6M1	0.31661
Q85601	Q8N3Y1	0.23501
Q85601	Q13123	0.48226
Q85601	Q9H2K2	0.4227
Q85601	Q6P9F0	0.3298
Q85601	Q00577	0.52822
Q85601	P38432	0.70754
Q85601	Q9BQ70	1.3752
Q85601	P20618	0.55261
Q85601	Q8NCE8	0.22279
Q85601	A2RTY3	0.29059
Q85601	Q92764	0.49362
Q85601	Q6ZN08	0.47241
Q85601	P62910	0.63754
Q85601	Q09666	0.67291
Q85601	A8MUA0	0.074848
Q85601	Q9BSJ2	1.2259
Q85601	Q15170	1.2578
Q85601	Q14151	0.63079
Q85601	Q17RB0	0.18373
Q85601	Q5T6F2	0.37239
Q85601	Q9GZU2	0.55913
Q85601	Q9NQR4	0.342
Q85601	O15131	1.1156
Q85601	Q96LI5	0.58716
Q85601	Q9UKT7	0.44103
Q85601	Q9Y6G3	0.21762
Q85601	Q9BW27	0.79769
Q85601	Q8TEA1	0.3843
Q85601	Q9Y6F1	0.31952
Q85601	P49716	0.40244
Q85601	Q92624	0.95818
Q85601	Q92541	1.0075
Q85601	Q9Y305	0.31106
Q85601	P12524	0.24393
Q85601	Q8NBF1	0.30995
Q85601	Q03468	0.43375
Q85601	Q86T75	0.46437
Q85601	O60711	0.52082
Q85601	Q13352	0.56493
Q85601	Q9Y6K9	0.93812
Q85601	Q8WW24	0.47167
Q85601	Q969S3	0.81425
Q85601	Q04726	1.0193
Q85601	P0CJ88	0.27808
Q85601	Q9H693	0.49978
Q85601	A6NNH0	0.37151
Q85601	P07864	0.51979
Q85601	Q9P0U4	0.38382
Q85601	Q8IWS0	0.64406
Q85601	Q96QU4	0.11206
Q85601	Q8N1W2	0.20923
Q85601	P25791	0.52433
Q85601	O15117	0.46369
Q85601	B1AK76	0.72454
Q85601	P69892	0.21071
Q85601	Q9Y6Y0	0.51682
Q85601	Q6P1M9	0.067225
Q85601	P46976	0.47383
Q85601	P61576	0.42275
Q85601	Q12968	0.74986
Q85601	Q9NRX2	0.080202
Q85601	Q8N339	0.26192
Q85601	Q86UW9	1.0046
Q85601	Q9HB96	0.85803
Q85601	Q9NXB0	0.71708
Q85601	Q15650	0.97814
Q85601	P17535	0.93121
Q85601	Q14154	0.46951
Q85601	Q9NQX1	0.55479
Q85601	Q2M3C7	0.5088
Q85601	O15355	0.97576
Q85601	P51532	0.46107
Q85601	Q9H814	0.32123
Q85601	Q7Z3Z0	0.65053
Q85601	Q8WWY6	0.45278

Q85601	Q9H607	0.52454
Q85601	Q8N983	0.38107
Q85601	Q9NVV9	1.1119
Q85601	A1L443	0.41643
Q85601	Q96JC9	0.98642
Q85601	Q9Y295	0.16619
Q85601	P82909	0.3334
Q85601	P62310	0.57719
Q85601	P49354	0.32494
Q85601	P48788	0.55831
Q85601	Q8NA31	0.69948
Q85601	O75461	0.31906
Q85601	Q9H808	0.59026
Q85601	Q04695	0.61937
Q85601	Q9H3J6	0.31705
Q85601	O15235	0.65885
Q85601	Q9BRJ7	0.23559
Q85601	Q5D0E6	0.71202
Q85601	Q6PI98	0.69936
Q85601	A6NGD5	0.24501
Q85601	Q15555	0.19135
Q85601	Q9H7B2	0.59847
Q85601	Q9H981	1.1956
Q85601	Q9H9G7	0.62448
Q85601	Q86TP1	0.84729
Q85601	Q66GS9	0.43133
Q85601	P41227	0.35218
Q85601	Q9Y221	0.71902
Q85601	Q96RN5	1.2874
Q85601	Q8WTZ3	0.54425
Q85601	Q6NUR6	0.33369
Q85601	Q5T280	0.75349
Q85601	Q6ZNM6	0.27807
Q85601	Q6P597	0.15687
Q85601	Q9NXK8	0.59558
Q85601	P30307	0.74217
Q85601	P62249	0.62339
Q85601	Q9BTM9	0.63526
Q85601	A6NJL1	0.14537
Q85601	O00159	0.10814
Q85601	P60371	0.17342
Q85601	O75351	0.35344
Q85601	Q8N6F7	0.38721
Q85601	Q9BUT9	0.65162
Q85601	P62280	0.46957
Q85601	Q13435	0.33181
Q85601	Q9BYV9	0.3452
Q85601	Q9NP64	0.32117
Q85601	Q86SQ7	0.96487
Q85601	Q8TED0	0.37018
Q85601	Q9Y2B5	0.015978
Q85601	O75665	0.74902
Q85601	Q8NEK5	0.31166
Q85601	Q8N4W9	0.35685
Q85601	Q9Y6K5	0.171
Q85601	Q9H8T0	0.48015
Q85601	Q5XG87	0.19723
Q85601	Q92785	0.71958
Q85601	Q96N38	0.44393
Q85601	P62314	0.75147
Q85601	Q9NWW7	0.056741
Q85601	Q6PJG2	0.79574
Q85601	Q9UK41	0.75787
Q85601	Q6ICB0	0.22109
Q85601	Q8N587	0.41898
Q85601	Q9UIS9	0.5967
Q85601	Q16537	0.35772
Q85601	Q8N344	0.61167
Q85601	Q9H9E3	0.47267
Q85601	Q3ZLR7	0.60189
Q85601	Q8IXF0	0.37679
Q85601	P0DKX0	0.41402
Q85601	Q9H171	0.1766
Q85601	P50991	0.3524
Q85601	Q9BXY8	0.43646
Q85601	P13984	0.58119

Q85601	P52742	0.31741
Q85601	Q02930	0.82676
Q85601	Q9H175	1.0813
Q85601	O43768	0.20509
Q85601	Q9BUK0	0.46889
Q85601	Q9H9H5	0.23747
Q85601	Q9UK73	0.75079
Q85601	Q6P3W6	0.5858
Q85601	Q7LG56	0.30283
Q85601	P63208	0.56811
Q85601	Q16600	0.30669
Q85601	Q9UMQ3	0.72237
Q85601	Q8NCQ2	0.31985
Q85601	Q9UBX0	0.18918
Q85601	Q92681	0.50404
Q85601	Q8N140	0.97576
Q85601	Q9Y5A6	0.76515
Q85601	Q9BW60	0.54781
Q85601	A6NJG6	0.32996
Q85601	P78364	0.11142
Q85601	Q8NDT2	0.70395
Q85601	Q96F24	0.7162
Q85601	Q15013	0.87467
Q85601	O00423	0.50943
Q85601	P56945	0.55736
Q85601	Q9P0T4	0.51717
Q85601	Q9BTC0	0.35868
Q85601	Q8N1Q8	0.31225
Q85601	Q9H7R5	0.3621
Q85601	P15170	0.43964
Q85601	Q8TAY7	1.2384
Q85601	Q9UKK6	0.69123
Q85601	Q99551	0.67614
Q85601	Q969G3	0.99321
Q85601	O76064	0.35812
Q85601	Q13901	1.0326
Q85601	Q96D46	0.67304
Q85601	Q9Y448	0.95148
Q85601	Q96M61	0.30691
Q85601	P11166	0.51847
Q85601	Q13098	0.25218
Q85601	Q96JB5	0.30051
Q85601	Q76N32	0.16485
Q85601	Q9Y375	0.58968
Q85601	P62306	0.25364
Q85601	O15049	1.115
Q85601	Q9HAU5	0.83184
Q85601	Q8N726	0.61718
Q85601	Q9UNN5	0.6553
Q85601	Q9UBY9	1.0044
Q85601	Q5JUW0	0.42505
Q85601	O14948	0.41785
Q85601	Q96JN0	0.43765
Q85601	O94811	0.46837
Q85601	Q9BPZ2	0.11417
Q85601	P35573	0.53811
Q85601	Q8N1V2	0.49205
Q85601	Q9C0I3	0.61281
Q85601	P17098	0.46069
Q85601	Q6PII5	0.27846
Q85601	Q9BSM1	1.1396
Q85601	Q96MN9	0.63622
Q85601	P05783	1.3089
Q85601	O95232	0.4564
Q85601	P47914	0.75257
Q85601	A6NNA2	0.22379
Q85601	P80297	0.28331
Q85601	O14556	0.40264
Q85601	Q70IA6	0.49011
Q85601	Q9P0L1	0.31855
Q85601	O60508	0.31055
Q85601	Q9GZT9	0.29579
Q85601	Q9HD15	0.61064
Q85601	Q14582	0.29416
Q85601	Q96N23	0.061982
Q85601	Q8NB42	0.46793

Q85601	P54098	0.43286
Q85601	Q8TEQ6	0.67546
Q85601	P84098	0.53692
Q85601	Q96L21	0.44662
Q85601	Q9UL36	0.26904
Q85601	Q96S59	0.96682
Q85601	Q8WXW3	0.67691
Q85601	Q8NHQ8	0.69012
Q85601	Q9H1A7	0.078638
Q85601	Q7Z7F7	0.53795
Q85601	Q9BY43	0.23409
Q85601	P25205	0.39368
Q85601	Q12988	0.44697
Q85601	P54727	0.7372
Q85601	Q8WVZ9	0.39393
Q85601	P12882	0.51716
Q85601	O95045	0.13239
Q85601	Q15928	0.48462
Q85601	Q5TAX3	0.7593
Q85601	Q9H6Q3	0.23771
Q85601	O95810	0.25915
Q85601	Q96D98	0.56772
Q85601	Q9ULJ1	0.20123
Q85601	Q8IY92	0.43168
Q85601	Q9UGK3	0.75929
Q85601	Q8N1K5	0.037193
Q85601	Q9H788	0.99905
Q85601	Q9H322	0.22152
Q85601	O15266	0.51854
Q85601	Q92621	1.0545
Q85601	O14641	0.91156
Q85601	Q13428	0.80299
Q85601	Q04323	0.29914
Q85601	P23511	1.1869
Q85601	Q9H2L5	0.56881
Q85601	Q53FN2	0.43378
Q85601	Q9ULX6	0.4606
Q85601	P40222	0.43034
Q85601	Q9NVI1	0.72835
Q85601	Q96AA8	0.16774
Q85601	Q96MR9	0.41154
Q85601	Q6P9A3	0.28926
Q85601	G3V211	0.26611
Q85601	P40306	0.2902
Q85601	Q562F6	0.80833
Q85601	Q9UGJ1	0.35201
Q85601	O14929	0.80404
Q85601	Q5XKR9	0.18341
Q85601	P0CJ90	0.27808
Q85601	Q96CM4	0.060673
Q85601	O95260	0.13334
Q85601	Q9Y4I5	0.25865
Q85601	Q8WW12	0.63253
Q85601	Q9Y6Q2	0.25242
Q85601	Q969U7	0.58031
Q85601	Q3B726	0.2395
Q85601	P78537	0.71602
Q85601	Q5MCW4	0.23549
Q85601	Q9BXF6	0.64939
Q85601	Q8NI29	0.20965
Q85601	P34896	0.1538
Q85601	Q9UI43	0.43925
Q85601	P0CJ89	0.27808
Q85601	P14618	0.21841
Q85601	P14635	0.29826
Q85601	Q9UKV3	0.59809
Q85601	Q9HAI6	0.39507
Q85601	Q8TES7	0.62243
Q85601	P15927	0.47514
Q85601	Q96N20	0.29141
Q85601	Q13084	1.0547
Q85601	Q8IXJ6	0.44872
Q85601	Q9NZH5	0.2953
Q85601	Q96JM7	0.42113
Q85601	Q15010	0.66046
Q85601	P08708	0.41302

Q85601	Q8IU81	0.32443
Q85601	O43920	0.52048
Q85601	Q68DK7	0.59147
Q85601	P04264	0.24418
Q85601	Q9UJ04	0.43856
Q85601	Q15124	0.0581
Q85601	Q92759	0.68569
Q85601	Q9UM00	1.2572
Q85601	Q96EV8	0.26869
Q85601	Q9HAW4	0.45285
Q85601	O95402	1.0285
Q85601	O95373	1.0699
Q85601	Q9H2U2	0.61239
Q85601	Q4L180	0.29744
Q85601	A6NJ88	0.50922
Q85601	Q8NFZ5	0.91545
Q85601	Q9UKJ3	0.57521
Q85601	P0CH99	0.60067
Q85601	Q7Z3K6	0.61167
Q85601	Q9UJ98	0.43151
Q85601	Q15437	0.3832
Q85601	Q9BRR0	0.39725
Q85601	Q9HCC9	0.50361
Q85601	Q13042	1.2683
Q85601	Q9BXP5	1.1175
Q85601	Q9UJX5	0.81417
Q85601	Q16649	0.1743
Q85601	Q6ZV65	0.29968
Q85601	P32780	0.75513
Q85601	A2A288	0.37394
Q85601	Q99933	0.81753
Q85601	Q01546	0.53559
Q85601	Q5JSZ5	0.34802
Q85601	P55060	0.88193
Q85601	Q15528	1.2058
Q85601	Q12933	0.56751
Q85601	Q969E8	0.76821
Q85601	Q8WTS6	0.47897
Q85601	Q9Y4P8	0.90804
Q85601	Q92973	1.1218
Q85601	Q9BV73	0.66203
Q85601	P51965	0.68983
Q85601	Q8TC56	0.61067
Q85601	P63162	0.53183
Q85601	Q9Y4Z0	0.59942
Q85601	P58340	0.48604
Q85601	Q9UM63	0.26495
Q85601	Q96M29	0.2844
Q85601	O75170	0.65636
Q85601	O75529	0.60544
Q85601	B3KS81	0.14306
Q85601	Q9UBL0	0.28883
Q85601	Q99873	0.62354
Q85601	Q14657	1.1485
Q85601	Q9Y3A3	0.59577
Q85601	P16415	0.34784
Q85601	P18847	1.1558
Q85601	Q9Y2D8	0.62427
Q85601	Q8IZW8	0.352
Q85601	O60543	0.32301
Q85601	Q4W5G0	0.3562
Q85601	Q5VZE5	0.31265
Q85601	P60370	0.17342
Q85601	Q8WV07	0.36585
Q85601	Q9H7X3	0.27623
Q85601	Q96N22	0.46418
Q85601	Q8IXQ5	0.34482
Q85601	P62136	0.68883
Q85601	Q9UBF2	0.32595
Q85601	O95948	0.38258
Q85601	Q9NYP9	0.44466
Q85601	Q9NXZ1	0.22094
Q85601	P22531	0.48792
Q85601	Q9HD26	0.93765
Q85601	Q12906	1.0894
Q85601	Q9BU64	0.2216

58749	Q85601	Q9H5J4	0.54781
58750	Q85601	Q96BK5	0.56752
58751	Q85601	Q9Y2G7	0.51519
58752	Q85601	Q9UL40	0.62881
58753	Q85601	Q6VY07	0.58636
58754	Q85601	P36956	0.25724
58755	Q85601	Q5TGS1	0.49887
58756	Q85601	Q9NQT4	0.44095
58757	Q85601	Q9UDV7	0.22809
58758	Q85601	Q5VZK9	0.24265
58759	Q85601	O43790	0.59315
58760	Q85601	Q13838	0.20603
58761	Q85601	Q5TBC7	0.22389
58762	Q85601	Q03924	0.26034
58763	Q85601	A6NDZ8	0.14127
58764	Q85601	P78543	0.66016
58765	Q85601	Q5VVS0	0.68876
58766	Q85601	Q9BQ90	0.80204
58767	Q85601	Q86YP4	0.87517
58768	Q85601	P49257	0.15735
58769	Q85601	Q96QU8	1.0199
58770	Q85601	Q13526	0.56993
58771	Q85601	Q9Y2J8	0.27437
58772	Q85601	O95416	0.41877
58773	Q85601	I3L1I5	0.2125
58774	Q85601	Q96MB7	0.38963
58775	Q85601	Q9UKW6	0.48607
58776	Q85601	Q9Y2A4	0.46674
58777	Q85601	Q7Z406	0.38986
58778	Q85601	P13929	0.19822
58779	Q85601	P35711	0.9437
58780	Q85601	P62258	0.85659
58781	Q85601	Q9H8E8	0.71981
58782	Q85601	Q9Y5B0	0.5743
58783	Q85601	Q92769	0.38354
58784	Q85601	Q9H444	0.75643
58785	Q85601	A6NFD8	0.2553
58786	Q85601	Q8N398	0.50435
58787	Q85601	Q9UBF6	0.82327
58788	Q85601	P59991	0.28316
58789	Q85601	Q96CX3	0.31716
58790	Q85601	P49720	0.32736
58791	Q85601	Q9UBF1	0.82202
58792	Q85601	Q9NVP1	0.073846
58793	Q85601	Q69383	0.42275
58794	Q85601	Q9NQC7	0.23176
58795	Q85601	Q8N859	0.481
58796	Q85601	P42575	0.28376
58797	Q85601	Q15973	0.25925
58798	Q85601	Q8N6T3	0.085439
58799	Q85601	Q14683	0.47454
58800	Q85601	Q2TAM9	0.41643
58801	Q85601	Q86Y79	0.2038
58802	Q85601	Q96KS0	0.12673
58803	Q85601	Q9C005	0.67065
58804	Q85601	Q0PNE2	0.41581
58805	Q85601	P63261	0.1273
58806	Q85601	Q96DE0	0.49983
58807	Q85601	P40121	0.43477
58808	Q85601	Q9Y678	0.64808
58809	Q85601	O60765	0.31611
58810	Q85601	P16220	0.89316
58811	Q85601	Q9UHV2	1.3689
58812	Q85601	Q9UPQ4	0.26238
58813	Q85601	Q99717	0.45393
58814	Q85601	Q96Q05	0.2753
58815	Q85601	Q6PF06	0.60031
58816	Q85601	Q9BQ87	0.45278
58817	Q85601	Q9H469	0.26661
58818	Q85601	Q9H0A8	0.53217
58819	Q85601	Q15699	0.47481
58820	Q85601	P52435	0.59245
58821	Q85601	Q9UKD2	0.11049
58822	Q85601	Q6PGQ7	1.1081
58823	Q85601	Q9H1Y0	0.34271
58824	Q85601	Q9Y2L5	0.018984

Q85601	Q9Y3B2	0.74131
Q85601	Q96LM1	0.053839
Q85601	O14920	0.51586
Q85601	Q08J23	0.68001
Q85601	Q96GS4	0.45413
Q85601	Q92804	0.1918
Q85601	Q04206	0.65196
Q85601	Q8TB03	0.13329
Q85601	O15069	0.39619
Q85601	Q8WXI9	0.61135
Q85601	A5YKK6	1.0584
Q85601	Q9BZD4	0.80465
Q85601	P45973	0.76939
Q85601	Q8TF63	0.019456
Q85601	P42768	0.32931
Q85601	Q99618	0.63956
Q85601	P42226	0.81952
Q85601	Q9P2J8	0.33301
Q85601	O95780	0.50474
Q85601	Q7RTS9	0.79491
Q85601	P49848	0.74107
Q85601	P52738	0.26944
Q85601	Q5HYC2	0.050346
Q85601	Q8N4J0	0.13604
Q85601	Q15035	0.12009
Q85601	O60535	0.55767
Q85601	Q96SD1	0.10075
Q85601	A8MV24	0.30462
Q85601	Q16576	0.43235
Q85601	Q9ULM3	0.77908
Q85601	P04733	0.26807
Q85601	Q147U1	0.32338
Q85601	Q9BRD0	0.3958
Q85601	Q8WW35	0.30691
Q85601	O95628	0.6637
Q85601	Q96LR7	0.55927
Q85601	Q8IXM2	0.17756
Q85601	O75394	0.087903
Q85601	Q9HCU9	1.1402
Q85601	Q9NZL3	0.69462
Q85601	Q8IYL2	0.035405
Q85601	Q96K17	0.38713
Q85601	Q9H875	0.1221
Q85601	Q96FA7	0.67443
Q85601	O95453	0.42994
Q85601	P62318	0.71454
Q85601	P36873	0.77927
Q85601	Q8WW14	0.10567
Q85601	P36871	0.23274
Q85601	Q8TEX9	0.52907
Q85601	Q9UM13	0.69316
Q85601	P53990	0.56687
Q85601	P02511	0.41614
Q85601	Q8WXC3	0.68383
Q85601	Q12972	0.72428
Q85601	P42766	0.8239
Q85601	Q8TCS8	0.063162
Q85601	Q9Y5V3	0.80082
Q85601	Q04727	0.66551
Q85601	P27348	0.31999
Q85601	Q9Y6Y1	0.56813
Q85601	Q6ICB4	0.20544
Q85601	Q5VZR2	0.41643
Q85601	Q15050	0.60958
Q85601	Q96G01	0.20772
Q85601	Q9H668	0.26893
Q85601	Q15029	0.25171
Q85601	Q6NUP7	0.45743
Q85601	Q8NFH8	0.41733
Q85601	P54725	0.64358
Q85601	Q12873	0.74097
Q85601	O43679	0.23803
Q85601	Q8IXQ6	0.86431
Q85601	Q99502	0.19626
Q85601	P19012	0.65618
Q85601	Q14678	0.53304

Q85601	Q6SJ96	0.30319
Q85601	P48668	0.29484
Q85601	Q9NRX1	0.11529
Q85601	Q99576	0.49374
Q85601	Q96E29	0.2333
Q85601	Q96NM4	0.35025
Q85601	Q9NZE8	0.24719
Q85601	P98169	0.16053
Q85601	Q96G23	0.21925
Q85601	A6NDY2	0.41169
Q85601	A2RTX5	0.31009
Q85601	P20585	0.069378
Q85601	Q8TAB7	0.55584
Q85601	Q8N9A8	0.327
Q85601	Q9Y6N9	0.34388
Q85601	A6NGH7	0.03624
Q85601	Q5VT06	0.70295
Q85601	Q9H8N7	0.25422
Q85601	Q9NWZ8	0.79341
Q85601	P40425	0.71734
Q85601	Q8NAV1	0.27791
Q85601	P52597	0.35289
Q85601	Q9C0F1	0.3295
Q85601	P28062	0.19715
Q85601	Q96T92	0.2566
Q85601	Q9BRK4	0.80461
Q85601	Q9NWW0	0.23411
Q85601	Q8TB72	0.4606
Q85601	O60573	0.49787
Q85601	Q8WY36	0.35025
Q85601	Q8WWQ0	0.62682
Q85601	P57081	0.92091
Q85601	Q08ER8	0.3562
Q85601	Q9UBZ9	0.54528
Q85601	Q76FK4	0.26936
Q85601	Q70EK8	0.48644
Q85601	P09972	0.61758
Q85601	P21980	0.30238
Q85601	O15014	0.99461
Q85601	Q5SVQ8	0.34583
Q85601	H3BTG2	0.059472
Q85601	Q6DKK2	1.0861
Q85601	Q9NS26	0.10376
Q85601	Q68DX3	0.46837
Q85601	Q9UN79	0.90111
Q85601	A6NKN8	0.32037
Q85601	Q13765	1.1467
Q85601	Q9C075	0.49847
Q85601	Q14192	0.74043
Q85601	Q9BYZ2	0.51979
Q85601	Q8NA57	0.058715
Q85601	Q495Y8	0.15217
Q85601	P11142	0.39211
Q85601	Q9UHI6	0.38026
Q85601	Q9H6B1	0.42155
Q85601	Q6NZY4	0.62781
Q85601	P23284	0.3785
Q85601	P59282	0.1732
Q85601	Q9UI36	0.42238
Q85601	Q05BQ5	0.37736
Q85601	Q15691	0.7221
Q85601	Q96M11	0.49129
Q85601	Q96N58	0.33023
Q85601	Q9NVX2	0.25829
Q85601	Q3C1V8	0.69877
Q85601	E7ETH6	0.29082
Q85601	O75347	0.86916
Q85601	P41218	0.69977
Q85601	Q9NVS2	0.20731
Q85601	P49721	0.33721
Q85601	Q8NFD5	0.86361
Q85601	A6NLC8	1.1466
Q85601	O60447	0.27633
Q85601	P62269	0.71568
Q85601	Q96AB3	0.75676
Q85601	O95190	0.44854

Q85601	Q71SY5	1.0633
Q85601	Q9NXT0	0.28473
Q85601	Q9UPA5	0.23693
Q85601	Q15058	0.39558
Q85601	Q8N3V7	0.30177
Q85601	A6NJB7	0.8046
Q85601	P46939	0.70764
Q85601	Q15051	0.63616
Q85601	P08238	0.59133
Q85601	Q8TC76	1.2384
Q85601	Q8TBR4	0.36001
Q85601	Q92905	0.73096
Q85601	P0DI81	1.0828
Q85601	P11055	0.49425
Q85601	Q17R26	0.33678
Q85601	Q9UKJ5	0.31486
Q85601	Q8IYM9	0.23121
Q85601	Q9BPZ3	0.47384
Q85601	Q53HV7	0.68591
Q85601	Q92843	0.43463
Q85601	P42704	0.9074
Q85601	P68104	0.74396
Q85601	O60248	0.56083
Q85601	Q7KZ85	0.36784
Q85601	Q7Z7A3	0.59048
Q85601	Q13425	0.51399
Q85601	A6NLF2	0.28453
Q85601	Q96MT8	1.1268
Q85601	Q02577	0.63064
Q85601	Q15025	0.50214
Q85601	Q96BD8	0.71027
Q85601	Q9HB21	0.22805
Q85601	H3BNL8	0.8759
Q85601	Q9Y3C6	0.41446
Q85601	O75427	0.46754
Q85601	P13645	0.62632
Q85601	Q6P4R8	1.1785
Q85601	Q10570	0.67495
Q85601	Q2TAL8	0.73779
Q85601	Q9Y2V2	0.3826
Q85601	Q9H4L7	0.37974
Q85601	Q63ZY3	0.917
Q85601	Q9Y6D9	0.99597
Q85601	P0DJG4	0.37717
Q85601	Q99848	0.20362
Q85601	Q9UJT9	0.47915
Q85601	A6NFL8	0.36009
Q85601	Q8N1B4	0.57724
Q85601	P62312	0.72853
Q85601	Q7Z3V5	0.34313
Q85601	P50570	0.33077
Q85601	O15297	0.62316
Q85601	A8MT69	0.5523
Q85601	Q5JVG2	0.22174
Q85601	Q9NUU7	0.12738
Q85601	Q5T5D7	0.23741
Q85601	Q6P3V2	0.21678
Q85601	P33552	0.48731
Q85601	Q9NYG5	0.89025
Q85601	O94868	0.30691
Q85601	Q9UHQ7	0.34176
Q85601	Q9P2I0	0.72998
Q85601	Q96B01	0.5406
Q85601	Q8NHM5	0.24145
Q85601	Q99750	0.44206
Q85601	Q8N7Q3	0.40278
Q85601	Q9HB58	0.42194
Q85601	Q5T5Y3	0.19313
Q85601	Q96NJ6	0.2572
Q85601	Q13330	0.62062
Q85601	Q8IYX0	0.52859
Q85601	A8MTY7	0.17342
Q85601	Q15562	0.8268
Q85601	P35269	1.1459
Q85601	Q9UI30	0.355
Q85601	P55735	0.58386

Q85601	Q15942	0.36531
Q85601	Q1MSJ5	0.90957
Q85601	O75618	1.1705
Q85601	P30279	0.75314
Q85601	O60921	0.87251
Q85601	Q5UIP0	0.7346
Q85601	Q7RTS7	0.30573
Q85601	Q9BW30	0.17594
Q85601	Q13228	1.2014
Q85601	Q8TA86	0.78197
Q85601	Q6P1X5	0.94103
Q85601	A9QM74	0.64351
Q85601	Q676U5	0.66804
Q85601	Q96RK0	0.98599
Q85601	Q9P031	1.0775
Q85601	Q99594	1.1076
Q85601	P22736	0.33614
Q85601	Q01543	0.41988
Q85601	Q14585	0.26341
Q85601	Q9NSI6	0.85296
Q85601	Q9HA38	0.12328
Q85601	Q9Y262	0.78361
Q85601	P52948	0.98646
Q85601	Q9NX18	0.82462
Q85601	Q9Y6X8	0.7345
Q85601	Q6NSI8	0.53008
Q85601	Q0VGE8	0.27192
Q85601	P41236	0.29012
Q85601	P61086	0.66863
Q85601	P54105	0.89605
Q85601	Q9Y222	1.0356
Q85601	Q9Y5K5	0.39149
Q85601	P51825	0.95867
Q85601	P14324	0.38597
Q85601	Q3SY00	0.46698
Q85601	Q15744	0.31821
Q85601	P20248	1.0879
Q85601	Q9Y6G5	0.30691
Q85601	Q9H8X3	0.046811
Q85601	Q6ZVX7	0.33546
Q85601	Q96A09	0.32844
Q85601	Q9BVL4	0.83521
Q85601	O14576	0.48682
Q85601	Q5VX52	0.045849
Q85601	A6NF01	0.73813
Q85601	Q6ZVC0	0.49979
Q85601	Q5JSJ4	0.79698
Q85601	Q13772	0.40531
Q85601	Q92529	0.22811
Q85601	Q96LW1	0.44666
Q85601	P55957	0.45454
Q85601	Q96N21	0.087345
Q85601	O14907	0.50144
Q85601	Q6UWZ7	0.5094
Q85601	Q9H446	0.30691
Q85601	Q9Y2P8	0.36246
Q85601	O14771	0.51744
Q85601	Q9NQ31	0.6177
Q85601	Q96GC6	1.1959
Q85601	Q86XF7	0.57451
Q85601	Q9BWW4	0.59856
Q85601	Q8IVI9	0.26628
Q85601	Q9Y2Q9	0.51005
Q85601	Q9NRH1	0.046709
Q85601	Q9BXJ9	0.73231
Q85601	Q6ZT77	0.32091
Q85601	A7MCY6	0.30033
Q85601	Q96N95	0.36056
Q85601	Q86UY8	0.082163
Q85601	Q9BZG8	0.24982
Q85601	Q8TAX9	0.11589
Q85601	P35548	0.62341
Q85601	Q9UET6	0.63322
Q85601	P25815	0.53671
Q85601	Q9NWV8	0.68771
Q85601	O75346	0.42716

Q85601	Q9BYX4	0.27942
Q85601	O60281	0.23085
Q85601	Q86VQ0	0.36803
Q85601	O75530	0.62919
Q85601	Q15024	0.63781
Q85601	Q96QD9	0.66063
Q85601	Q8NA77	0.66564
Q85601	Q96GM8	0.26102
Q85601	Q8WXF8	1.1205
Q85601	Q8TD16	0.34126
Q85601	O00273	0.27785
Q85601	Q3KQU3	0.67812
Q85601	P35251	0.54569
Q85601	Q9Y6K1	0.41313
Q85601	Q5VZB9	0.13144
Q85601	Q96B97	0.38402
Q85601	Q99459	1.0318
Q85601	P25786	0.42578
Q85601	O15205	0.53206
Q85601	Q8IVM0	0.53217
Q85601	Q6ZV50	0.35025
Q85601	Q9ULC6	0.67611
Q85601	Q9UKX7	1.0399
Q85601	Q01081	0.46858
Q85601	P35680	0.27516
Q85601	Q9H3H9	0.81942
Q85601	Q8IZD4	0.99091
Q85601	O14862	0.53011
Q85601	P05412	0.82835
Q85601	Q8IZL2	0.90799
Q85601	Q9Y543	0.35965
Q85601	P17029	0.40292
Q85601	Q9BYV8	0.65594
Q85601	Q9BYD3	0.37248
Q85601	Q9NPJ4	1.4797
Q85601	Q6ZTZ1	0.15138
Q85601	O95159	0.25798
Q85601	Q6ZRS2	0.43405
Q85601	Q9NQ87	0.48689
Q85601	Q86V97	0.39393
Q85601	Q9NWN3	0.11079
Q85601	Q9H2P0	0.23466
Q85601	Q9Y4A8	0.51624
Q85601	Q99909	0.1323
Q85601	Q8ND83	0.64429
Q85601	P11388	0.23538
Q85601	Q96RS0	0.63241
Q85601	Q9UQ35	0.098785
Q85601	Q9NWU5	0.16289
Q85601	Q96T88	0.35764
Q85601	P29083	1.2587
Q85601	Q9ULM6	1.006
Q85601	Q76L83	0.579
Q85601	P35749	0.51716
Q85601	Q7Z7A1	1.2059
Q85601	Q9Y281	0.49304
Q85601	P31040	0.61317
Q85601	P49450	0.26478
Q85601	Q92985	0.61376
Q85601	A6NP11	0.47998
Q85601	Q15327	0.44772
Q85601	Q3ZCQ8	0.074459
Q85601	Q9Y6X4	0.63409
Q85601	Q9BXY0	0.22094
Q85601	Q9HAV7	0.15218
Q85601	Q8IZU1	0.52287
Q85601	Q9UGC7	0.16954
Q85601	Q8TE02	1.2405
Q85601	Q9NZM4	0.83263
Q85601	Q9P2M1	0.53217
Q85601	Q7Z4U5	0.47271
Q85601	Q16514	0.85038
Q85601	Q15929	0.51514
Q85601	P61571	0.42275
Q85601	Q9BYE7	0.85006
Q85601	Q05519	0.52715

Q85601	Q8NEM1	0.47479
Q85601	P28347	0.97128
Q85601	Q9Y3F4	0.83317
Q85601	Q9UBT6	0.14377
Q85601	O95257	0.30906
Q85601	Q99615	0.81088
Q85601	O14656	0.21293
Q85601	P22033	0.16304
Q85601	P55822	0.33116
Q85601	O14613	0.090252
Q85601	Q5JR98	0.57776
Q85601	P82970	0.72651
Q85601	Q9NR77	0.11041
Q85601	Q14160	0.55452
Q85601	Q8N5N7	0.096586
Q85601	P78352	0.57108
Q85601	Q70Z53	0.64005
Q85601	P61579	0.42275
Q85601	Q14691	0.27431
Q85601	Q9Y4K1	0.429
Q85601	Q9HBH7	0.31713
Q85601	O00255	0.20587
Q85601	P35789	0.46557
Q85601	Q9UMS5	0.39428
Q85601	A6NK89	0.39529
Q85601	Q5TB80	0.99182
Q85601	Q6ZNA1	0.34313
Q85601	Q96IL0	0.11737
Q85601	P67809	0.5219
Q85601	P78396	0.90272
Q85601	P18621	0.61843
Q85601	Q8IYD1	0.38439
Q85601	Q8IX07	0.35191
Q85601	O75663	0.45644
Q85601	P18850	0.61224
Q85601	P12035	0.49278
Q85601	A6H8Y1	0.72281
Q85601	A6NL46	0.53989
Q85601	A8MSY0	0.26877
Q85601	Q8ND04	0.57242
Q85601	O96018	0.45308
Q85601	Q569K6	0.16068
Q85601	Q9Y676	0.23408
Q85601	Q01826	0.7568
Q85601	Q9NUW8	0.73305
Q85601	Q3KNT7	0.1041
Q85601	A6NDD8	0.46437
Q85601	Q6TAR4	0.31043
Q85601	Q14590	0.25802
Q85601	Q9NYZ3	0.13637
Q85601	Q9H902	0.12846
Q85601	P05387	0.53692
Q85601	Q9UKI9	1.2717
Q85601	Q8NI27	0.41556
Q85601	Q6U7Q0	0.31481
Q85601	Q8IXW5	0.67475
Q85601	Q9HAP2	0.714
Q85601	Q9NQ29	0.76753
Q85601	O94921	0.20163
Q85601	Q99550	0.48083
Q85601	A8MVA2	0.17342
Q85601	Q8N668	0.8235
Q85601	O00399	0.21107
Q85601	Q9NYK5	0.41111
Q85601	Q13625	0.42937
Q85601	Q9BSH5	0.37166
Q85601	P41219	0.42248
Q85601	P10244	0.60258
Q85601	Q8IZU0	0.32773
Q85601	P13994	0.39471
Q85601	P42771	0.54658
Q85601	Q9BYK8	0.42373
Q85601	P53041	0.71071
Q85601	A6NKF2	0.6668
Q85601	P53680	0.17962
Q85601	Q86UC2	0.090855

Q85601	Q9P2B4	0.2596
Q85601	Q15323	0.57121
Q85601	Q9UC06	0.33046
Q85601	P57086	0.48953
Q85601	O75376	0.39862
Q85601	Q9HB03	0.54781
Q85601	P60673	0.22639
Q85601	Q5JTD0	1.014
Q85601	Q99612	0.33002
Q85601	Q9NTK1	0.36172
Q85601	O00470	0.61384
Q85601	P42224	0.25227
Q85601	Q9NXG0	0.075608
Q85601	Q6NUI1	0.32095
Q85601	Q9H5H4	0.2988
Q85601	Q8N883	0.26649
Q85601	Q8N9N5	0.17951
Q85601	Q9NX05	0.29038
Q85601	O00425	0.38222
Q85601	Q96HT8	1.0195
Q85601	O00762	0.53262
Q85601	Q9BVA0	0.24767
Q85601	O94818	0.40862
Q85601	Q9NTX7	0.61804
Q85601	Q9NPA3	1.128
Q85601	Q96EB1	1.1249
Q85601	Q6ZRF7	0.22879
Q85601	A8MU76	0.59101
Q85601	Q9Y462	0.69057
Q85601	Q86YI8	0.68137
Q85601	P60410	0.36976
Q85601	Q01201	1.0942
Q85601	Q8IW35	1.2526
Q85601	Q15369	1.4177
Q85601	Q9BYQ9	0.17342
Q85601	Q6RW13	0.61172
Q85601	Q13185	0.77515
Q85601	O75582	0.25797
Q85601	P83369	0.57475
Q85601	P78344	0.35663
Q85601	Q8WV99	0.31044
Q85601	Q9BRJ2	0.35401
Q85601	Q9UPP1	0.45928
Q85601	Q8NCA9	0.17891
Q85601	Q6ZTU2	0.3554
Q85601	Q14061	0.21339
Q85601	P06748	0.42839
Q85601	Q5TCQ9	0.53803
Q85601	P28072	0.36955
Q85601	Q8N108	1.1716
Q85601	Q16254	1.0233
Q85601	O00566	0.52956
Q85601	P06132	0.40087
Q85601	Q96IP4	0.22677
Q85601	P08235	0.38006
Q85601	A5PLL1	0.23411
Q85601	Q7Z7K2	0.19496
Q85601	Q53TS8	0.045146
Q85601	Q96I59	0.40226
Q85601	Q14865	0.27274
Q85601	O43711	0.33472
Q85601	Q92783	0.62742
Q85601	Q6ZN32	0.32996
Q85601	Q9NZZ3	1.0146
Q85601	Q12816	0.3353
Q85601	Q14CX7	0.11557
Q85601	Q9H7N4	0.55181
Q85601	Q9NRL2	1.0693
Q85601	O75717	0.45182
Q85601	Q8NFH5	0.65007
Q85601	Q9UNX3	0.95826
Q85601	P49356	0.14845
Q85601	Q53QV2	0.51773
Q85601	Q9UN30	0.31192
Q85601	Q8N0X7	0.12616
Q85601	A9YTQ3	0.31551

Q85601	Q01658	0.4524
Q85601	Q9NW38	0.57219
Q85601	P22392	0.27933
Q85601	Q53EL6	0.9849
Q85601	P17023	0.25965
Q85601	Q96NI8	0.40366
Q85601	Q86YC2	0.70674
Q85601	P15121	0.2827
Q85601	Q9NV88	0.58748
Q85601	Q96F45	0.13825
Q85601	P60842	0.081216
Q85601	Q9BX40	0.6395
Q85601	Q9BT78	1.0402
Q85601	Q3KNV8	1.0124
Q85601	Q9NQ48	0.22344
Q85601	Q8N6T7	0.7136
Q85601	O43829	0.66734
Q85601	P82650	0.2061
Q85601	Q9H063	0.31186
Q85601	Q00403	1.1565
Q85601	Q92796	0.31669
Q85601	Q99689	0.18901
Q85601	Q9UDY4	0.83815
Q85601	P0CI00	0.59643
Q85601	Q9NPB6	0.73072
Q85601	Q8TD17	0.21952
Q85601	Q9UKV8	0.777
Q85601	Q8N2Z9	0.617
Q85601	Q8TBN0	0.25155
Q85601	Q8WWM7	0.45629
Q85601	Q9UJX3	0.75873
Q85601	Q13200	0.34911
Q85601	Q2M3D2	0.19689
Q85601	Q9GZR2	0.14046
Q85601	Q9UDW3	0.39181
Q85601	Q9Y2J4	0.31586
Q85601	Q96SK3	0.33301
Q85601	Q9ULG1	0.3907
Q85601	Q86UE4	0.42116
Q85601	P61572	0.42275
Q85601	O14787	0.97419
Q85601	Q13033	0.69293
Q85601	P62277	1.0607
Q85601	Q6ZS17	0.40578
Q85601	P05121	0.40287
Q85601	P61586	0.41676
Q85601	Q96C90	0.72564
Q85601	Q9P0R6	0.12102
Q85601	Q9NZD4	0.22245
Q85601	P57077	0.81038
Q85601	Q14209	1.0839
Q85601	P0CW23	0.0033438
Q85601	Q99590	0.64132
Q85601	Q3SY52	0.25357
Q85601	Q4V348	0.23373
Q85601	Q9HB75	0.66976
Q85601	Q5SNV9	0.15838
Q85601	Q13569	0.53046
Q85601	O95456	0.4877
Q85601	O00512	0.84935
Q85601	Q6PB30	0.3347
Q85601	A8MV65	0.6425
Q85601	O94983	1.3507
Q85601	O96007	0.25437
Q85601	Q96NJ3	0.27477
Q85601	C9JBD0	0.51292
Q85601	Q9H074	0.39976
Q85601	P12883	0.42967
Q85601	P11474	0.45661
Q85601	Q13015	0.36536
Q85601	O43830	0.21822
Q85601	Q9BV68	0.32982
Q85601	P22061	0.22004
Q85601	Q8TA94	0.50616
Q85601	Q14315	0.32294
Q85601	Q9C0D0	0.27901

Q85601	P50613	0.23004
Q85601	O00479	0.22868
Q85601	Q6P9B6	0.30691
Q85601	Q6P1L5	0.70874
Q85601	O60293	0.023553
Q85601	Q9BQN1	0.61318
Q85601	Q6PIF2	0.30814
Q85601	P15923	0.57651
Q85601	Q86T29	0.24382
Q85601	Q9HBZ2	0.36971
Q85601	O95905	0.82902
Q85601	Q9H4H8	0.48115
Q85601	O14964	0.68622
Q85601	Q8IUC3	0.19997
Q85601	O43395	0.77919
Q85601	Q99496	0.52824
Q85601	Q13356	0.37952
Q85601	P36402	0.50187
Q85601	Q8N5J4	0.52414
Q85601	Q5H9J7	0.75921
Q85601	Q13118	0.35551
Q85601	Q96JI7	0.66917
Q85601	O94927	0.65651
Q85601	P49459	0.75667
Q85601	Q8N9Z0	0.413
Q85601	Q32MZ4	1.1437
Q85601	O14593	0.74057
Q85601	P10827	0.30334
Q85601	Q8WUF5	0.90221
Q85601	O75920	0.53899
Q85601	Q9C0C2	0.20143
Q85601	O75884	0.78365
Q85601	O43290	0.92898
Q85601	Q5MJ68	0.30022
Q85601	O43615	0.18182
Q85601	P58304	0.29838
Q85601	Q3ZCT1	0.2542
Q85601	Q96N06	0.0049316
Q85601	O43812	0.33281
Q85601	P50453	0.6813
Q85601	Q3B820	0.22617
Q85601	Q9UHJ3	0.33843
Q85601	Q5HYK9	0.32689
Q85601	P28065	0.52148
Q85601	O00267	1.0411
Q85601	P07900	0.59162
Q85601	Q96A56	1.0032
Q85601	Q69YQ0	0.32946
Q85601	Q8TAF3	0.7018
Q85601	P22670	0.81966
Q85601	Q8N5L8	0.75315
Q85601	O60384	0.4606
Q85601	Q9H6U6	0.83192
Q85601	P30414	0.37076
Q85601	Q9H410	0.95919
Q85601	Q96PV7	0.002423
Q85601	Q14181	0.48339
Q85601	Q96ST2	0.048128
Q85601	Q8IYW5	0.32282
Q85601	P32243	0.82415
Q85601	O95478	0.30989
Q85601	Q8TBB1	0.89474
Q85601	P42773	0.36929
Q85601	P35268	0.57838
Q85601	Q8IYA6	0.48306
Q85601	Q6ZVF9	0.17604
Q85601	O14867	0.41765
Q85601	Q9H201	0.15033
Q85601	Q96EG3	0.24722
Q85601	Q6VVB1	0.45052
Q85601	Q5T1B0	0.017737
Q85601	Q15633	0.73409
Q85601	P48382	0.94565
Q85601	Q6ZW49	0.45397
Q85601	Q2M1V0	0.32996
Q85601	Q8IVC4	0.33107

Q85601	Q5JTC6	0.51778
Q85601	Q9UHK0	0.31655
Q85601	Q9BQ66	0.17342
Q85601	O75340	0.42354
Q85601	Q96T58	0.61375
Q85601	Q6IE81	1.1652
Q85601	Q8N693	0.46012
Q85601	Q02447	0.53876
Q85601	O75558	0.57427
Q85601	O95630	0.26274
Q85601	O75132	0.3014
Q85601	Q09028	0.61593
Q85601	Q9H8Q6	0.42897
Q85601	P56693	0.62105
Q85601	O94993	0.22899
Q85601	O75446	1.1128
Q85601	Q96EK6	0.24602
Q85601	O94992	1.2993
Q85601	P55854	0.55188
Q85601	Q96T17	0.5147
Q85601	P23193	1.3006
Q85601	Q6IB77	0.21134
Q85601	Q6NSH3	0.26275
Q85601	P07741	0.29291
Q85601	P29966	0.6503
Q85601	Q9BTC8	0.80236
Q85601	Q8IY81	0.72213
Q85601	Q8NE65	0.41818
Q85601	Q5T8A7	0.7082
Q85601	Q96N77	0.4507
Q85601	Q9H4A6	0.45283
Q85601	Q68EA5	0.51008
Q85601	P43897	0.83286
Q85601	Q8WWY3	0.2811
Q85601	Q8N5U6	1.2392
Q85601	Q8WUU5	0.36139
Q85601	Q13601	0.40628
Q85601	Q68DK2	0.97998
Q85601	O14980	0.97828
Q85601	O95486	0.46446
Q85601	Q5H9I0	0.51907
Q85601	P67775	0.74297
Q85601	Q0D2I5	0.032492
Q85601	Q99684	0.39106
Q85601	Q06265	0.043245
Q85601	P53805	0.61145
Q85601	P0C7M4	0.22476
Q85601	P62979	0.42618
Q85601	Q8WY41	0.45449
Q85601	O75334	0.53887
Q85601	Q9H0C5	0.56117
Q85601	O43402	0.44852
Q85601	O95476	0.29522
Q85601	Q9BYQ7	0.24199
Q85601	O95751	0.5794
Q85601	Q7L2H7	0.56583
Q85601	P50402	0.14245
Q85601	A8MX80	0.48715
Q85601	Q8IW19	0.47439
Q85601	Q96GN5	0.90605
Q85601	P55265	0.35162
Q85601	Q13347	0.68618
Q85601	Q14207	0.94752
Q85601	Q15185	0.51947
Q85601	A6NMK8	0.069652
Q85601	Q96RY5	0.92224
Q85601	Q8WU90	1.0051
Q85601	P61575	0.42275
Q85601	O43309	0.29938
Q85601	O94973	0.414
Q85601	Q6ZS46	0.14684
Q85601	A8K554	0.0096984
Q85601	Q99436	0.55261
Q85601	Q9BSU3	0.57753
Q85601	E9PQ53	0.45141
Q85601	Q8TEK3	0.38665

Q85601	P62847	0.50417
Q85601	P48059	0.27985
Q85601	Q8N7Z2	0.43599
Q85601	Q8N319	0.88732
Q85601	Q12872	0.48278
Q85601	P50616	0.34763
Q85601	P29474	0.55459
Q85601	Q96PC3	0.37967
Q85601	A1A5D9	0.4266
Q85601	Q9P1F3	0.42724
Q85601	Q5SXJ2	1.1879
Q85601	Q9C035	0.67191
Q85601	Q9NUP7	0.68595
Q85601	O15360	0.85288
Q85601	Q9BQ69	0.45446
Q85601	Q8TF68	0.17544
Q85601	O00204	0.48026
Q85601	Q7Z6J9	0.43992
Q85601	Q7Z6B0	0.56037
Q85601	Q99583	0.40766
Q85601	Q8ND07	0.91064
Q85601	Q3SYG4	0.45195
Q85601	O60927	0.47351
Q85601	Q9BX70	0.56934
Q85601	Q96RU7	0.41875
Q85601	O75382	0.38199
Q85601	Q8NDF8	0.4516
Q85601	Q15628	0.63136
Q85601	Q14146	0.22094
Q85601	Q9NQ35	0.56585
Q85601	O75436	0.71451
Q85601	Q07157	0.672
Q85601	Q52MB2	1.0838
Q85601	Q8N5P1	0.27685
Q85601	P17544	1.0969
Q85601	O75822	0.68618
Q85601	Q0D2K3	0.61619
Q85601	A8K0S8	0.22476
Q85601	Q6ZVK8	0.44238
Q85601	H0Y7S4	0.17874
Q85601	Q96S94	0.36692
Q85601	Q6GMV2	0.2559
Q85601	Q8TDG2	0.11637
Q85601	Q9NZI6	0.54905
Q85601	P28070	0.35054
Q85601	Q8IX90	0.71489
Q85601	O75323	0.25139
Q85601	D6RB28	0.36182
Q85601	P28749	1.3532
Q85601	Q9NXF7	0.26501
Q85601	Q9HBD4	0.63336
Q85601	P20073	0.4097
Q85601	P84103	0.44933
Q85601	P09467	0.36812
Q85601	Q9NW08	0.075132
Q85601	P55199	1.002
Q85601	Q8NE71	0.34177
Q85601	P35658	0.98869
Q85601	O15446	0.26237
Q85601	Q6PGQ1	0.090372
Q85601	Q6NS38	0.1836
Q85601	P68871	0.21136
Q85601	Q9NUL3	0.59396
Q85601	P41567	0.57053
Q85601	Q05516	0.74507
Q85601	P31249	0.55631
Q85601	O75901	0.56929
Q85601	Q9HCU8	0.48603
Q85601	P13489	0.45993
Q85601	Q14397	0.67815
Q85601	Q6ZS27	0.30272
Q85601	Q9P1Q0	0.38973
Q85601	Q9NTW7	0.44079
Q85601	Q9BQ67	0.44273
Q85601	Q8N3U4	0.70179
Q85601	Q9HD33	0.53386

Q85601	O95996	0.37452
Q85601	Q9H9L3	0.41513
Q85601	Q92797	0.76638
Q85601	Q7L2R6	0.48095
Q85601	Q86T82	0.37009
Q85601	Q14980	1.1479
Q85601	P26196	0.51162
Q85601	Q5H9F3	0.6922
Q85601	O75150	0.58822
Q85601	O14602	0.30529
Q85601	O95363	0.26326
Q85601	O00482	0.28056
Q85601	Q9UK39	0.45512
Q85601	Q14152	1.1177
Q85601	Q96IF1	0.43578
Q85601	Q04446	0.083373
Q85601	P0DI82	0.37836
Q85601	P40925	0.52398
Q85601	Q86US8	0.63152
Q85601	Q96DM1	0.5015
Q85601	P61244	0.88429
Q85601	P51504	0.28078
Q85601	Q9ULE6	0.70162
Q85601	Q86Y97	0.44708
Q85601	P09914	0.35572
Q85601	Q2VPB7	0.52847
Q85601	Q9BQA5	0.86907
Q85601	P05198	0.62266
Q85601	P60608	0.27602
Q85601	Q9NX70	1.0544
Q85601	H0YKK7	0.36182
Q85601	P84022	0.52723
Q85601	Q9Y3T9	1.0543
Q85601	O95376	0.45704
Q85601	O60260	0.51261
Q85601	P61580	0.33886
Q85601	Q06830	0.25096
Q85601	Q8IWC1	0.66237
Q85601	Q6ZN19	0.24382
Q85601	Q9P0M2	0.11226
Q85601	P78345	0.86917
Q85601	Q6ZN01	0.57302
Q85601	Q5TG92	0.021192
Q85601	Q9NPG2	0.04707
Q85601	Q0D2J5	0.25527
Q85601	Q9BWF2	0.27439
Q85601	Q99607	1.1477
Q85601	Q9NPI1	1.0584
Q85601	Q6ZSS3	0.19331
Q85601	Q15233	0.54537
Q85601	Q9BY27	0.40938
Q85601	P53618	0.79161
Q85601	Q6NSW5	0.71297
Q85601	Q9NT62	0.45066
Q85601	Q3BBV1	1.1879
Q85601	O95777	0.4945
Q85601	Q96JZ2	0.47122
Q85601	Q9UJX4	0.8039
Q85601	P60520	0.24293
Q85601	P42677	0.87727
Q85601	Q7RTT5	0.1323
Q85601	A6NKD9	1.0127
Q85601	O95400	0.73661
Q85601	Q9P2J3	0.26296
Q85601	Q5VTR2	0.74573
Q85601	Q9HCK8	0.44042
Q85601	Q9H2G9	0.46468
Q85601	P31271	0.15545
Q85601	Q9BXG8	0.56813
Q85601	Q9UM11	1.1792
Q85601	Q5THR3	0.36002
Q85601	Q9H9Q2	0.26218
Q85601	Q86VE0	0.91598
Q85601	P63220	0.5756
Q85601	Q96CD0	0.30691
Q85601	Q9Y252	0.53214

Q85601	Q14584	0.31607
Q85601	P49715	0.24028
Q85601	Q9Y6B7	0.26685
Q85601	Q9H009	0.39619
Q85601	Q6NT89	0.31638
Q85601	P05120	0.5571
Q85601	Q5JR59	1.0463
Q85601	Q99766	0.32668
Q85601	Q9UIF8	0.96049
Q85601	P50748	0.85215
Q85601	Q9BTL3	0.45592
Q85601	Q8TAP6	0.98771
Q85601	Q8WY54	0.89818
Q85601	Q96EH5	0.29456
Q85601	Q8TF45	0.24382
Q85601	A1A4V9	0.16683
Q85601	Q16082	0.96818
Q85601	P10072	0.30741
Q85601	Q9Y615	0.41156
Q85601	Q06547	1.0447
Q85601	Q8TAA3	0.23663
Q85601	Q9C0D7	0.5008
Q85601	Q14159	0.84504
Q85601	P09497	0.40767
Q85601	P14866	0.36386
Q85601	A1A580	0.12421
Q85601	Q8NEF9	0.70872
Q85601	O15055	0.24427
Q85601	P62837	0.84493
Q85601	Q6ZQV5	0.35685
Q85601	Q9Y2D1	1.1737
Q85601	Q12959	0.81923
Q85601	Q9UL46	0.23001
Q85601	Q8TB69	0.48095
Q85601	Q6IA86	0.62651
Q85601	Q96H79	0.43005
Q85601	Q96RE9	0.27609
Q85601	O60287	0.54149
Q85601	Q9UGP4	0.50377
Q85601	Q9NPC3	0.18232
Q85601	O60885	0.74725
Q85601	Q15326	1.3408
Q85601	Q8WVK7	0.69369
Q85601	P01008	0.50961
Q85601	Q8IV54	0.39447
Q85601	O76095	0.24388
Q85601	Q6P2Q9	0.42471
Q85601	O15037	0.15015
Q85601	O14746	0.50341
Q85601	Q9P2T0	0.17003
Q85601	Q9Y5Z7	0.44239
Q85601	Q99460	0.073424
Q85601	O43296	0.33357
Q85601	Q96IY1	1.0036
Q85601	Q6RSH7	0.38278
Q85601	Q5T6S3	0.44456
Q85601	Q96EF6	0.56397
Q85601	O00231	0.31995
Q85601	Q96DN6	0.085767
Q85601	A6XGL0	0.07969
Q85601	Q5T1J5	0.45749
Q85601	Q15937	0.31898
Q85601	P78385	0.57095
Q85601	P14317	0.24754
Q85601	O95639	0.37546
Q85601	Q9UKY7	0.27624
Q85601	O60671	0.57712
Q85601	Q49AA0	0.42883
Q85601	Q15759	0.26024
Q85601	Q86WG3	0.29197
Q85601	Q96IZ7	0.81436
Q85601	A2IDD5	0.56034
Q85601	Q9UNS2	0.68562
Q85601	Q15906	0.92172
Q85601	Q9NYV6	0.55014
Q85601	P41161	0.1922

59813	Q85601	Q16204	0.27235
59814	Q85601	P55347	0.46007
59815	Q85601	P08727	0.65053
59816	Q85601	P0CG47	0.38823
59817	Q85601	Q8WYH8	0.6445
59818	Q85601	Q99996	0.58352
59819	Q85601	O95394	0.77304
59820	Q85601	P04053	0.30571
59821	Q85601	Q9BPU9	0.22596
59822	Q85601	P83876	0.90466
59823	Q85601	P31949	0.33539
59824	Q85601	Q96QC0	0.8009
59825	Q85601	P63165	0.99903
59826	Q85601	P45984	0.33373
59827	Q85601	Q8N782	0.39148
59828	Q85601	Q9BSY4	0.14226
59829	Q85601	P49918	0.71902
59830	Q85601	P0C7V5	0.39609
59831	Q85601	P53677	0.27389
59832	Q85601	P07197	0.73455
59833	Q85601	P07195	0.51979
59834	Q85601	O76011	0.65053
59835	Q85601	Q9UGU0	0.4567
59836	Q85601	Q8WYJ6	0.22655
59837	Q85601	I6L9F6	0.1936
59838	Q85601	Q8IZQ1	0.66974
59839	Q85601	Q8IYX3	0.51024
59840	Q85601	Q6ZSB9	0.26338
59841	Q85601	Q8IXM3	0.41683
59842	Q85601	Q5VU43	1.0486
59843	Q85601	P37275	0.80059
59844	Q85601	Q96Q83	0.55486
59845	Q85601	Q9UJM3	0.43184
59846	Q85601	Q9BYH8	0.35203
59847	Q85601	O43933	0.28557
59848	Q85601	Q8WUY3	0.33707
59849	Q85601	Q96RJ6	0.38487
59850	Q85601	Q12983	0.15033
59851	Q85601	Q9BRP1	0.81769
59852	Q85601	Q99567	0.79786
59853	Q85601	Q4G163	0.15891
59854	Q85601	Q96C74	0.3025
59855	Q85601	O00193	0.40862
59856	Q85601	O95218	0.47274
59857	Q85601	Q96S55	0.30528
59858	Q85601	Q5W0V3	0.37171
59859	Q85601	Q7Z7L8	0.5865
59860	Q85601	Q8IXH7	1.1826
59861	Q85601	Q9BZK7	0.9479
59862	Q85601	Q9UPR3	1.0283
59863	Q85601	P35754	0.4084
59864	Q85601	P56524	0.3562
59865	Q85601	P61581	0.33886
59866	Q85601	O75638	0.70874
59867	Q85601	Q9NZM5	0.1358
59868	Q85601	Q9UK10	0.35685
59869	Q85601	Q8IYB9	0.37553
59870	Q85601	Q96CS2	0.6577
59871	Q85601	Q86TJ5	0.27901
59872	Q85601	Q8IY85	0.3446
59873	Q85601	Q5EBM4	0.13188
59874	Q85601	P60953	0.48547
59875	Q85601	Q6ZNA4	0.68882
59876	Q85601	O43447	0.56819
59877	Q85601	Q9NSC5	0.45206
59878	Q85601	P60510	0.94439
59879	Q85601	Q8IXH6	1.2338
59880	Q85601	Q5TKA1	0.4182
59881	Q85601	Q9BUK6	0.088619
59882	Q85601	Q9UPN7	1.0006
59883	Q85601	Q12789	0.84031
59884	Q85601	Q9UIF7	0.45846
59885	Q85601	Q9C073	0.28218
59886	Q85601	P84101	0.444
59887	Q85601	Q9BYR7	0.95215
59888	Q85601	Q8N584	0.28954

Q85601	Q99627	0.45647
Q85601	P62328	0.74911
Q85601	Q9UKG1	0.42863
Q85601	Q8N163	0.68828
Q85601	Q92870	0.70969
Q85601	P54278	0.40936
Q85601	Q9BZA5	0.57772
Q85601	P31274	0.11617
Q85601	Q13416	0.49719
Q85601	P14625	0.47529
Q85601	P62508	0.55105
Q85601	Q86TS9	0.31905
Q85601	Q9NPC7	0.20159
Q85601	Q92845	0.25621
Q85601	P02792	0.091884
Q85601	Q9BTV5	0.5661
Q85601	Q9Y2H8	0.31465
Q85601	Q9NRI5	0.3971
Q85601	Q5T6C5	0.79691
Q85601	O15379	0.56347
Q85601	A6NKD2	0.47415
Q85601	O60422	0.31892
Q85601	Q5VTD9	0.29486
Q85601	Q8N187	0.65088
Q85601	A8MT65	0.43523
Q85601	Q8IUC1	0.17154
Q85601	Q6NZI2	1.0757
Q85601	Q6ICC9	0.14915
Q85601	Q8NEG4	0.22895
Q85601	O15287	0.97951
Q85601	A4D1E9	0.50026
Q85601	Q00839	0.25022
Q85601	Q8N9N2	0.33188
Q85601	Q8N8A2	0.30691
Q85601	Q8NBR6	1.043
Q85601	P52292	0.99784
Q85601	O15453	0.43364
Q85601	Q08945	1.2245
Q85601	P18583	0.21812
Q85601	P52737	0.66077
Q85601	Q6ULP2	0.74669
Q85601	P13647	0.83409
Q85601	Q9UGN5	0.23351
Q85601	Q14999	1.0211
Q85601	Q9H832	0.71893
Q85601	Q9HBQ8	0.36009
Q85601	Q96AE4	0.50785
Q85601	Q9BZE1	0.31905
Q85601	A6NCN2	0.56056
Q85601	Q8N8Z8	0.26087
Q85601	Q92750	0.66232
Q85601	P0C838	0.039046
Q85601	Q7Z6K1	0.61884
Q85601	Q9Y2P0	0.3051
Q85601	Q12905	1.0661
Q85601	Q96RE7	1.0907
Q85601	P18754	1.1489
Q85601	Q7L190	0.79565
Q85601	Q5TZA2	0.6997
Q85601	P17947	0.68691
Q85601	Q9UBQ0	0.413
Q85601	Q9UK61	0.16983
Q85601	O43189	0.6581
Q85601	P38646	0.56814
Q85601	P21675	0.43567
Q85601	O15127	0.73273
Q85601	Q8IYE1	0.069201
Q85601	Q4G0A6	1.0129
Q85601	Q14532	0.43235
Q85601	Q15910	0.451
Q85601	Q9HB19	0.58404
Q85601	Q9NR09	0.46276
Q85601	Q9NX01	0.18972
Q85601	Q8TEV9	0.29797
Q85601	Q6NXS1	0.37994
Q85601	Q9GZL7	0.69538

Q85601	Q9UGK8	0.70708
Q85601	Q12765	0.19835
Q85601	Q2TAY7	0.23411
Q85601	P08133	0.65183
Q85601	Q8WWC4	0.25358
Q85601	Q96MW7	0.26569
Q85601	O15084	0.74616
Q85601	Q8IVF1	0.68211
Q85601	P46782	0.48716
Q85601	P04156	0.45508
Q85601	Q96BN2	0.57389
Q85601	P46937	0.80712
Q85601	Q08050	0.49872
Q85601	Q6P2C8	0.984
Q85601	Q6P9G9	0.23611
Q85601	Q9NPA8	0.452
Q85601	Q8TAD8	1.3007
Q85601	P34932	0.25755
Q85601	P11844	0.45348
Q85601	P07902	0.44927
Q85601	Q9UHC1	0.46569
Q85601	Q9UBS8	0.75535
Q85601	Q7Z6Z7	0.34731
Q85601	O75113	0.90095
Q85601	Q8IW36	0.41128
Q85601	Q9NX53	0.30691
Q85601	Q9UHY7	0.25572
Q85601	Q12962	1.0919
Q85601	Q13156	0.31224
Q85601	Q5T5S1	0.29947
Q85601	Q6WBX8	0.42899
Q85601	Q6UN15	0.53151
Q85601	A3KN83	0.65721
Q85601	Q9BRH9	0.30507
Q85601	Q06732	0.1945
Q85601	Q9H6J7	0.22094
Q85601	A6NIH7	0.21378
Q85601	Q4LE28	0.86988
Q85601	Q9NQ50	0.39826
Q85601	P25440	0.53346
Q85601	P61574	0.42275
Q85601	Q8WUM4	1.0897
Q85601	Q16836	0.58616
Q85601	P19387	0.51723
Q85601	P47897	0.15004
Q85601	Q92966	0.52171
Q85601	P04792	0.39423
Q85601	O14974	0.3667
Q85601	O75164	1.0999
Q85601	Q86TG7	0.63057
Q85601	Q6RFH5	0.28079
Q85601	Q6ZN28	0.66442
Q85601	O75486	0.76646
Q85601	P28370	0.53132
Q85601	Q96LL4	0.30691
Q85601	Q7Z2E3	0.55772
Q85601	Q13077	0.30817
Q85601	P08397	0.12718
Q85601	Q9UHB9	0.23248
Q85601	Q9NX63	0.82378
Q85601	P82094	1.392
Q85601	P35232	0.56245
Q85601	Q969M7	0.50956
Q85601	Q9Y6D6	0.6295
Q85601	Q9H098	0.16626
Q85601	C9JLW8	0.48854
Q85601	Q9Y5Y2	0.31185
Q85601	Q16533	0.49002
Q85601	Q8WYR1	0.26653
Q85601	P35869	0.41025
Q85601	P63272	1.0154
Q85601	Q13432	0.49494
Q85601	P23526	0.156
Q85601	P48556	0.28592
Q85601	O60356	0.59589
Q85601	O00743	0.8226

Q85601	Q9GZN2	0.40758
Q85601	Q6ZU65	0.22094
Q85601	Q9H1Z4	0.44273
Q85601	Q8N6Y0	0.61302
Q85601	P35226	0.63936
Q85601	O60783	0.22814
Q85601	A6PW82	0.25205
Q85601	O15105	0.29133
Q85601	Q9H8Y8	0.3516
Q85601	O60826	0.30177
Q85601	Q6PJP8	0.76941
Q85601	O43325	0.65006
Q85601	A7E2V4	1.106
Q85601	Q6ZMW2	0.27186
Q85601	Q8NCT3	0.096861
Q85601	Q6PI48	0.53389
Q85601	Q16534	0.24205
Q85601	Q5T1N1	0.52145
Q85601	P35527	0.65053
Q85601	A6NDX5	0.34569
Q85601	Q8N9Z9	0.63675
Q85601	Q96T37	0.2362
Q85601	P06746	0.58587
Q85601	Q92890	0.84087
Q85601	P48595	0.53196
Q85601	P46779	1.0109
Q85601	O15397	0.85646
Q85601	Q9H1A3	0.30691
Q85601	P04731	0.28384
Q85601	P69891	0.21071
Q85601	Q969F9	0.64742
Q85601	P21127	0.19986
Q85601	Q9NV06	0.26385
Q85601	Q7Z460	0.45881
Q85601	O76031	0.21672
Q85601	Q01850	0.90193
Q85601	A0JLT2	0.81271
Q85601	Q8IWZ3	0.92963
Q85601	Q02410	0.51848
Q85601	Q8NHR7	0.1968
Q85601	O94763	0.76278
Q85601	P12004	0.22697
Q85601	Q9BSK1	0.23095
Q85601	Q86WJ1	0.43773
Q85601	Q9BYR4	0.17342
Q85601	Q9UK76	0.50873
Q85601	O60504	0.72529
Q85601	O14645	0.063327
Q85601	Q9UHB7	1.3188
Q85601	Q7L9L4	0.83348
Q85601	O00625	0.53963
Q85601	P0CG31	0.33659
Q85601	Q96HI0	0.56814
Q85601	P52655	1.1688
Q85601	Q8IVT2	0.32966
Q85601	Q8NA92	0.48281
Q85601	Q8N972	0.35685
Q85601	Q59F38	0.12414
Q85601	Q5SW79	0.88838
Q85601	Q6A163	0.56535
Q85601	Q6UUV7	1.5062
Q85601	Q9NRA8	1.1263
Q85601	Q7RTT6	0.21427
Q85601	Q9Y2M5	0.85527
Q85601	Q504T8	0.22094
Q85601	Q9HCD5	1.0775
Q85601	Q13342	0.76749
Q85601	Q96AQ1	0.26137
Q85601	O75157	0.26223
Q85601	Q96Q35	0.20289
Q85601	Q7RTU0	0.35333
Q85601	O94892	0.25103
Q85601	Q9UK99	0.37327
Q85601	A9UHW6	0.68598
Q85601	Q8NDN9	0.70013
Q85601	Q5HYI7	0.6849

60117	Q85601	Q9UJA5	0.50971
60118	Q85601	Q7L3S4	0.3434
60119	Q85601	P62750	0.71935
60120	Q85601	Q9NY74	0.44273
60121	Q85601	Q9Y5R5	0.22314
60122	Q85601	Q9BW11	0.38487
60123	Q85601	P30740	0.5571
60124	Q85601	Q9NPE3	0.70791
60125	Q85601	Q107X0	0.2935
60126	Q85601	Q9NNX9	0.41572
60127	Q85601	O00567	0.78841
60128	Q85601	O43823	0.14095
60129	Q85601	P54840	0.27263
60130	Q85601	Q96EI5	0.64454
60131	Q85601	P35580	0.45053
60132	Q85601	Q96M63	0.10546
60133	Q85601	Q8TD86	0.31552
60134	Q85601	Q92670	0.25371
60135	Q85601	P0CW19	0.089127
60136	Q85601	P0CG35	0.34646
60137	Q85601	O96006	0.52318
60138	Q85601	Q5JXB2	0.57619
60139	Q85601	O15350	0.3653
60140	Q85601	P22087	0.55179
60141	Q85601	Q9Y6I3	0.063115
60142	Q85601	P62851	0.76152
60143	Q85601	Q96KG9	0.35576
60144	Q85601	Q99990	0.58101
60145	Q85601	Q5SQN1	0.34826
60146	Q85601	O00291	0.52911
60147	Q85601	P46013	0.11435
60148	Q85601	P63010	0.44784
60149	Q85601	Q9BU61	0.69211
60150	Q85601	Q8NEY8	0.43176
60151	Q85601	Q96TC7	0.5413
60152	Q85601	A8MUQ0	0.86025
60153	Q85601	Q9UBX2	0.27808
60154	Q85601	Q08AG7	0.73064
60155	Q85601	Q92945	0.66688
60156	Q85601	O14893	0.60858
60157	Q85601	Q9NZJ4	0.11739
60158	Q85601	Q9NWS8	0.43365
60159	Q85601	Q9NRG4	0.82855
60160	Q85601	Q13547	0.80362
60161	Q85601	P43356	0.91823
60162	Q85601	Q00994	0.80404
60163	Q85601	Q9H5Q4	0.42946
60164	Q85601	P50221	0.59282
60165	Q85601	P35908	0.43137
60166	Q85601	Q99081	0.45844
60167	Q85601	Q7L0Y3	0.92633
60168	Q85601	Q96C00	0.38071
60169	Q85601	Q9H9E1	0.28584
60170	Q85601	Q96DG6	0.12173
60171	Q85601	P31314	0.58769
60172	Q85601	Q9BTT4	0.64203
60173	Q85601	A8MTY0	0.49058
60174	Q85601	Q58FF3	0.58819
60175	Q85601	P49116	0.60785
60176	Q85601	Q86WZ0	0.055025
60177	Q85601	Q9NWA0	1.2185
60178	Q85601	Q05086	0.37964
60179	Q85601	O75764	0.28588
60180	Q85601	Q9UFB7	0.27524
60181	Q85601	Q6PEX3	0.12132
60182	Q85601	O75635	0.53196
60183	Q85601	Q92597	0.61836
60184	Q85601	Q9BQ83	0.67767
60185	Q85601	Q9H2T7	0.47128
60186	Q85601	Q66K89	0.87707
60187	Q85601	Q6ECI4	0.33301
60188	Q85601	Q9HCH0	0.84314
60189	Q85601	Q4LEZ3	0.82218
60190	Q85601	Q5T619	0.30138
60191	Q85601	O94916	0.21845
60192	Q85601	Q9GZR5	0.25401

60193	Q85601	P14651	0.21781
60194	Q85601	Q9BWD1	0.25837
60195	Q85601	Q9UKY1	0.40299
60196	Q85601	O76015	0.56535
60197	Q85601	Q9NZ63	0.44273
60198	Q85601	O43660	0.24685
60199	Q85601	Q5MNV8	0.015793
60200	Q85601	A8MUU9	0.20599
60201	Q85601	Q8TD26	0.25567
60202	Q85601	P62841	0.65289
60203	Q85601	Q14687	0.30691
60204	Q85601	Q9H2G4	0.74715
60205	Q85601	Q96CX2	0.12893
60206	Q85601	Q8N3F8	0.56503
60207	Q85601	Q6PCE3	0.27478
60208	Q85601	Q86YW9	0.38726
60209	Q85601	P78559	0.76712
60210	Q85601	P24928	0.84064
60211	Q85601	Q96M53	0.88952
60212	Q85601	O75362	0.76406
60213	Q85601	Q5TYX0	0.39986
60214	Q85601	P62140	0.79298
60215	Q85601	P28074	0.43342
60216	Q85601	O75626	0.21959
60217	Q85601	Q96F86	0.58011
60218	Q85601	Q96DV4	0.25881
60219	Q85601	O15372	0.65806
60220	Q85601	P49792	0.84652
60221	Q85601	Q9Y6H5	0.79138
60222	Q85601	Q9H0P0	0.23155
60223	Q85601	Q96NL3	0.33129
60224	Q85601	A6NKC0	0.38628
60225	Q85601	Q8NCX0	0.4165
60226	Q85601	Q7Z699	0.32678
60227	Q85601	P62308	0.61651
60228	Q85601	Q86U86	1.0582
60229	Q85601	Q13505	0.57244
60230	Q85601	P24863	1.0796
60231	Q85601	Q96P71	0.54597
60232	Q85601	P62256	0.64759
60233	Q85601	Q8WW33	0.30188
60234	Q85601	Q6IPR1	0.24479
60235	Q85601	Q674X7	0.47037
60236	Q85601	P61236	0.22094
60237	Q85601	P52298	0.46662
60238	Q85601	Q96JL9	0.31555
60239	Q85601	Q9P0V3	0.57627
60240	Q85601	P67870	0.32339
60241	Q85601	Q92530	0.49661
60242	Q85601	P57055	0.53242
60243	Q85601	Q9Y3R5	0.57461
60244	Q85601	P54253	0.90493
60245	Q85601	P0DI83	0.31331
60246	Q85601	Q13233	0.50148
60247	Q85601	Q9HB65	0.577
60248	Q85601	P38936	0.24747
60249	Q85601	Q96IT1	0.72157
60250	Q85601	P30876	0.63992
60251	Q85601	Q86XI2	0.87879
60252	Q85601	Q96SL8	0.22259
60253	Q85601	Q9Y473	0.36604
60254	Q85601	O00570	0.86232
60255	Q85601	Q9P243	0.40909
60256	Q85601	O94903	0.28497
60257	Q85601	Q92572	0.44823
60258	Q85601	O60930	0.27532
60259	Q85601	Q8NB50	0.3621
60260	Q85601	Q86VX2	0.39824
60261	Q85601	Q5TAB7	0.67278
60262	Q85601	P59797	0.35084
60263	Q85601	Q9Y5A7	0.67799
60264	Q85601	P04406	0.43756
60265	Q85601	O75865	0.48612
60266	Q85601	O15162	0.14547
60267	Q85601	P50502	0.61194
60268	Q85601	P62987	0.69107

Q85601	O95848	0.52955
Q85601	P38159	0.50539
Q85601	Q0VD86	0.44273
Q85601	Q14140	1.3369
Q85601	A6NNF4	0.35363
Q85601	P35900	0.78804
Q85601	P13056	0.67556
Q85601	Q6ZRV2	0.4694
Q85601	P33992	0.22948
Q85601	Q8WVM0	0.5613
Q85601	Q8IWE4	0.22361
Q85601	P48552	0.65928
Q85601	Q5QJE6	0.67276
Q85601	Q9UM54	0.13547
Q85601	Q96JN8	0.20637
Q85601	O95886	0.47854
Q85601	Q9P287	0.71896
Q85601	Q13620	0.79062
Q85601	O95613	0.81301
Q85601	P82663	0.52149
Q85601	P17025	0.29598
Q85601	P23528	0.75141
Q85601	Q00534	0.55879
Q85601	P52434	0.38455
Q85601	P40426	0.32503
Q85601	P61966	0.33417
Q85601	Q6P280	0.53556
Q85601	Q8NCN2	0.23504
Q85601	Q9BQY9	0.48095
Q85601	P24278	0.87609
Q85601	Q7Z4G1	0.98109
Q85601	Q7Z3I7	0.2712
Q85601	P55198	0.96966
Q85601	Q13618	0.40585
Q85601	P78371	0.60016
Q85601	P20962	0.13853
Q85601	P22090	0.3174
Q85601	Q8N0Z3	0.63491
Q85601	P59780	0.11413
Q85601	P55212	0.47336
Q85601	Q7Z7J5	0.56409
Q85601	Q8N7B6	0.50855
Q85601	Q13111	1.1695
Q85601	A0JNW5	0.24031
Q85601	O00287	0.044135
Q85601	O43365	0.44188
Q85601	Q9BWX1	0.46583
Q85601	Q00653	1.2012
Q85601	Q15543	1.002
Q85601	Q0VG06	0.57771
Q85601	P0C5J1	0.077411
Q85601	Q86UW6	0.1057
Q85601	O60224	0.1323
Q85601	Q9NSC2	0.33591
Q85601	O95067	0.61356
Q85601	Q9NYB0	0.70042
Q85601	Q15696	0.19106
Q85601	Q9HCE5	0.040716
Q85601	Q96EP1	0.6574
Q85601	Q9BVL2	0.6879
Q85601	Q92993	1.0514
Q85601	Q86UQ0	0.2655
Q85601	Q8WWX9	0.56952
Q85601	O95999	0.23621
Q85601	Q96C28	0.42238
Q85601	Q9NVR5	0.18366
Q85601	O00712	0.21983
Q85601	O43379	0.23994
Q85601	Q9NP87	0.18464
Q85601	Q5T1B1	0.66469
Q85601	P43268	0.45463
Q85601	P23246	0.7257
Q85601	O94952	0.43005
Q85601	P26368	0.39579
Q85601	Q9BYQ5	0.17342
Q85601	A2A368	0.0050687

Q85601	Q9H3F6	0.57939
Q85601	P60014	0.24199
Q85601	Q15652	0.3922
Q85601	P56182	0.58089
Q85601	Q96HA1	0.27234
Q85601	Q3LIE5	0.28273
Q85601	Q96LI6	0.53277
Q85601	Q53G59	0.50177
Q85601	P15336	0.85752
Q85601	P51814	0.20304
Q85601	P10828	0.56356
Q85601	Q8IYX7	0.41357
Q85601	Q9NVV4	0.53159
Q85601	Q8N3J9	0.29108
Q85601	P41182	0.2049
Q85601	Q8NEH6	0.7115
Q85601	P04198	0.80092
Q85601	Q6NSW7	0.32996
Q85601	Q8N0Y7	0.68269
Q85601	Q8WW01	0.19808
Q85601	Q9Y333	0.57419
Q85601	P41252	0.6845
Q85601	O60524	0.7181
Q85601	Q9P1T7	1.4438
Q85601	Q6RFH8	0.80608
Q85601	Q8IWL3	0.55255
Q85601	Q8IZ40	0.62744
Q85601	Q15047	0.78175
Q85601	Q16352	0.56137
Q85601	Q8TAK5	0.51059
Q85601	O43318	0.56455
Q85601	Q8IUF8	0.51676
Q85601	Q8IXY8	0.37076
Q85601	Q9NS37	0.8506
Q85601	O00422	1.2551
Q85601	Q9H299	0.12519
Q85601	Q92793	0.49738
Q85601	Q9HCG8	0.10681
Q85601	Q9H4R4	0.071132
Q85601	O75525	0.69164
Q85601	A8MUZ8	0.5338
Q85601	P39880	0.64255
Q85601	P05114	0.39536
Q85601	A6NEY3	0.36009
Q85601	Q9H4P4	0.28984
Q85601	P57071	0.15266
Q85601	Q15365	0.86619
Q85601	Q3T8J9	0.68539
Q85601	Q8N4C6	0.20678
Q85601	Q8IV03	0.59031
Q85601	Q9HAJ7	1.1032
Q85601	P11441	0.58661
Q85601	Q99456	0.65053
Q85601	A5LHX3	0.20043
Q85601	P13535	0.42967
Q85601	A6NE21	0.38628
Q85601	P39748	0.311
Q85601	Q59GR2	0.22476
Q85601	Q15697	0.53796
Q85601	P31276	0.54231
Q85601	Q9NX40	0.45409
Q85601	A6NI87	0.29386
Q85601	P19474	0.42127
Q85601	P61578	0.42275
Q85601	Q6PGP7	0.76943
Q85601	Q9H1K1	0.991
Q85601	Q96PQ5	0.25389
Q85601	Q5CZA5	0.3562
Q85601	Q14119	0.26775
Q85601	Q8IZP0	0.25964
Q85601	Q9UJU3	0.23015
Q85601	Q96NB3	0.60232
Q85601	Q8WUI4	0.41655
Q85601	Q8TDC0	0.48602
Q85601	Q8N2G8	0.21239
Q85601	Q9Y2P7	0.41378

Q85601	P40692	0.020736
Q85601	Q8N488	0.59094
Q85601	Q9BQS6	0.85017
Q85601	P82664	0.5511
Q85601	P48643	0.093816
Q85601	Q9BQW3	0.052078
Q85601	Q9BV20	0.087351
Q85601	P61583	0.46208
Q85601	Q15181	0.35841
Q85601	O60869	0.66067
Q85601	P48380	0.36319
Q85601	P53804	0.775
Q85601	P51003	0.13828
Q85601	Q9P1Z2	0.89867
Q85601	Q9BYJ9	0.43793
Q85601	O14512	0.50209
Q85601	Q8TDP1	0.054443
Q85601	Q9UPQ9	0.31398
Q85601	Q9UI32	0.32153
Q85601	Q9H1E3	0.35225
Q85601	Q96NA2	0.81162
Q85601	Q16775	0.2909
Q85601	Q15361	0.77684
Q85601	O75467	0.25481
Q85601	Q66PJ3	0.60135
Q85601	Q9HCS4	0.19771
Q85601	O60264	0.24551
Q85601	Q13023	0.6037
Q85601	Q9UIC8	0.28095
Q85601	Q6P387	0.44273
Q85601	Q92800	0.53755
Q85601	Q496M5	0.29702
Q85601	Q9HBL0	0.38212
Q85601	Q14CZ0	0.94458
Q85601	O43593	0.24489
Q85601	Q7RTU3	0.35333
Q85601	Q9NZL4	0.26708
Q85601	Q9Y6Q3	0.23957
Q85601	P78549	0.33886
Q85601	Q5BKZ1	0.49567
Q85601	Q92466	0.66746
Q85601	P51523	0.23504
Q85601	P61927	0.59299
Q85601	P57721	0.60293
Q85601	Q9Y6Q5	0.41971
Q85601	Q9NV56	0.61832
Q85601	Q96LW7	0.22546
Q85601	O75475	1.4175
Q85601	O75586	0.72122
Q85601	Q8TE76	0.31901
Q85601	P30419	0.26527
Q85601	P29692	0.29126
Q85601	O14558	0.76901
Q85601	Q5SXM1	0.36542
Q85601	P0CG12	0.94895
Q85601	Q6VMQ6	1.0068
Q85601	Q13875	0.60004
Q85601	A6NEM1	0.36182
Q85601	Q9BYG7	0.22094
Q85601	Q9NXR1	0.40331
Q85601	Q8N0Z2	0.38202
Q85601	Q9UQR0	0.27872
Q85601	Q5VWM5	0.83567
Q85601	P36578	1.027
Q85601	Q8TAV0	0.22014
Q85601	O60828	0.86811
Q85601	Q6PDB4	0.53147
Q85601	O95271	0.64192
Q85601	Q8N7J2	0.62717
Q85601	Q9BY42	0.28551
Q85601	Q9BZR8	0.43422
Q85601	Q86Y01	0.68911
Q85601	Q9UPY8	1.0962
Q85601	Q05877	0.35703
Q85601	Q9BT04	0.12374
Q85601	P61088	0.73482

60497	Q85601	Q5T3J3	1.014
60498	Q85601	Q16526	0.43508
60499	Q85601	Q8N8E2	0.030979
60500	Q85601	Q6AZW8	0.27624
60501	Q85601	Q9NX95	0.33039
60502	Q85601	O00505	1.078
60503	Q85601	Q96K30	0.86615
60504	Q85601	Q06416	0.32996
60505	Q85601	P07738	0.71167
60506	Q85601	P41214	0.56892
60507	Q85601	O95071	0.25694
60508	Q85601	Q9NXV2	1.3776
60509	Q85601	O00257	1.0268
60510	Q85601	Q8IY57	1.2114
60511	Q85601	Q9UHQ1	0.48754
60512	Q85601	Q8IZT9	0.32773
60513	Q85601	Q96K80	0.30436
60514	Q85601	P19447	0.17925
60515	Q85601	P35813	0.32149
60516	Q85601	Q7LBC6	0.19072
60517	Q85601	Q5SWL8	0.22854
60518	Q85601	O94855	0.46446
60519	Q85601	O75381	0.093015
60520	Q85601	Q4G112	0.32996
60521	Q85601	Q8TCZ7	0.17067
60522	Q85601	Q3LI83	0.30526
60523	Q85601	Q14527	0.38727
60524	Q85601	Q99732	0.25289
60525	Q85601	Q6P589	0.54448
60526	Q85601	Q460N5	0.43876
60527	Q85601	P07093	0.48695
60528	Q85601	Q06323	0.66336
60529	Q85601	Q13895	0.81358
60530	Q85601	Q14129	0.44911
60531	Q85601	O60675	0.4338
60532	Q85601	Q8NC74	0.32582
60533	Q85601	P17677	0.12482
60534	Q85601	Q9NP98	0.29985
60535	Q85601	Q6ZVQ6	0.80526
60536	Q85601	O43255	0.46825
60537	Q85601	P35606	0.45069
60538	Q85601	Q8WXS3	0.52129
60539	Q85601	Q6IPW1	0.44312
60540	Q85601	P43358	0.39393
60541	Q85601	Q9UJC5	0.31265
60542	Q85601	Q96NS1	0.22094
60543	Q85601	P59942	0.15779
60544	Q85601	Q76KX8	0.47842
60545	Q85601	Q13829	0.772
60546	Q85601	Q96JS3	0.3411
60547	Q85601	Q9H9J2	0.3715
60548	Q85601	Q8NEZ2	0.82693
60549	Q85601	Q9H9L7	0.29751
60550	Q85601	Q53HM4	0.41676
60551	Q85601	O94805	0.68682
60552	Q85601	Q9UKT8	0.30575
60553	Q85601	A6NN14	0.35363
60554	Q85601	Q6IV72	0.28727
60555	Q85601	P10515	0.36422
60556	Q85601	P30260	1.0724
60557	Q85601	O15160	0.11656
60558	Q85601	Q5VWM1	0.21119
60559	Q85601	Q6PI77	0.23411
60560	Q85601	O00167	0.76642
60561	Q85601	P05386	1.0109
60562	Q85601	Q9NZ52	0.4798
60563	Q85601	A6NIY4	0.15217
60564	Q85601	Q9BSQ5	0.27614
60565	Q85601	Q6Q0C0	0.29694
60566	Q85601	Q7Z5L9	1.0383
60567	Q85601	Q9NXR8	0.36762
60568	Q85601	O14618	0.52629
60569	Q85601	Q96MY7	0.69781
60570	Q85601	Q63HK5	0.34582
60571	Q85601	O75419	0.88082
60572	Q85601	Q68DY9	0.32778

60573	Q85601	Q9Y3C4	0.33622
60574	Q85601	O75807	0.45325
60575	Q85601	Q9H963	0.38556
60576	Q85601	Q6P582	0.38744
60577	Q85601	Q8IX03	1.1331
60578	Q85601	Q5T200	0.72102
60579	Q85601	Q9H633	0.27714
60580	Q85601	Q92900	0.62568
60581	Q85601	Q7Z7L9	0.35574
60582	Q85601	P17275	0.8753
60583	Q85601	Q04837	0.50733
60584	Q85601	A8K8V0	0.29979
60585	Q85601	O76042	0.34315
60586	Q85601	Q86WH2	0.23992
60587	Q85601	Q9Y3B9	0.37346
60588	Q85601	Q9NY12	0.11152
60589	Q85601	Q8N141	0.47651
60590	Q85601	Q15257	0.44473
60591	Q85601	Q9Y2W1	0.74335
60592	Q85601	Q9H816	0.60266
60593	Q85601	Q9UIE0	0.29689
60594	Q85601	O00411	0.5138
60595	Q85601	Q9ULD0	0.25856
60596	Q85601	Q9UH77	0.61228
60597	Q85601	P43355	1.105
60598	Q85601	O95163	0.69488
60599	Q85601	Q9H160	0.52674
60600	Q85601	Q9UFN0	0.70162
60601	Q85601	Q5VUA4	0.5491
60602	Q85601	Q86U42	0.59136
60603	Q85601	Q9UBK9	1.0315
60604	Q85601	P78358	0.53217
60605	Q85601	Q16637	0.80459
60606	Q85601	Q9BWK5	0.10715
60607	Q85601	Q9P270	0.50492
60608	Q85601	P0CV99	0.47415
60609	Q85601	Q9BVT8	0.096518
60610	Q85601	Q9UL03	0.6603
60611	Q85601	O43310	0.4272
60612	Q85601	P43359	0.89264
60613	Q85601	Q96EZ8	0.87961
60614	Q85601	P58557	0.54045
60615	Q85601	Q9BTE7	0.35501
60616	Q85601	Q6ZMV8	0.51618
60617	Q85601	Q96I27	0.22137
60618	Q85601	Q9UIL1	0.75722
60619	Q85601	Q00987	0.27074
60620	Q85601	Q92922	1.0359
60621	Q85601	Q8N2M8	0.44232
60622	Q85601	Q8WXQ3	0.75655
60623	Q85601	Q8NF91	0.55864
60624	Q85601	O75531	0.96342
60625	Q85601	O95571	0.60703
60626	Q85601	P60660	0.29254
60627	Q85601	P14672	0.658
60628	Q85601	Q8N660	1.1879
60629	Q85601	O94901	0.1732
60630	Q85601	Q8NCK3	0.38119
60631	Q85601	Q9UPU9	0.29653
60632	Q85601	Q9H7L9	0.58157
60633	Q85601	Q9BS34	0.23195
60634	Q85601	O94842	0.65684
60635	Q85601	P31943	0.29634
60636	Q85601	P60604	0.84493
60637	Q85601	Q8TCJ0	0.70616
60638	Q85601	P82914	0.36105
60639	Q85601	Q14671	0.15118
60640	Q85601	Q14602	0.3273
60641	Q85601	Q96CA5	0.28697
60642	Q85601	Q7L1Q6	0.39547
60643	Q85601	Q9BU89	0.14569
60644	Q85601	Q8IYF1	0.26678
60645	Q85601	Q8TBZ5	0.32035
60646	Q85601	O60238	0.24065
60647	Q85601	Q9BSG1	0.3414
60648	Q85601	P55209	0.72964

Q85601	A0AVK6	0.38629
Q85601	Q96HA8	0.99895
Q85601	O43583	0.42393
Q85601	Q9BXX3	0.23715
Q85601	Q9NXX6	0.45765
Q85601	Q9P032	0.47401
Q85601	O95073	1.0775
Q85601	O60684	0.45049
Q85601	Q5VWM6	0.55878
Q85601	P02794	0.83798
Q85601	Q86UB2	0.23911
Q85601	Q9GZY8	0.0909
Q85601	Q96KP1	0.29032
Q85601	Q8N1I0	0.29301
Q85601	Q68CP9	0.57799
Q85601	P19838	0.74405
Q85601	Q9H910	0.50873
Q85601	Q9BYQ2	0.17342
Q85601	Q8IUD2	0.8735
Q85601	Q9Y536	0.34528
Q85601	Q6PD74	0.07822
Q85601	Q96EY5	0.42771
Q85601	Q8NG27	0.31785
Q85601	O95429	0.2471
Q85601	P20823	0.24546
Q85601	Q14568	0.58819
Q85601	Q9NSA3	0.79026
Q85601	Q9Y5L0	1.0116
Q85601	Q9H0U9	0.62675
Q85601	O43592	0.35643
Q85601	Q9BRX2	0.46286
Q85601	A8K8P3	0.80069
Q85601	Q96B23	0.70273
Q85601	Q12824	0.95921
Q85601	Q8TDR4	0.43441
Q85601	Q99741	0.54331
Q85601	Q96ED9	0.50596
Q85601	A6NCW3	0.38171
Q85601	Q9Y3B6	0.42169
Q85601	P29508	0.5571
Q85601	P62166	0.26764
Q85601	Q15935	0.44819
Q85601	Q0VDF9	0.32329
Q85601	O75123	0.27785
Q85601	Q13568	0.99579
Q85601	O76021	0.3407
Q85601	Q9UKT5	0.42017
Q85601	Q9UNE7	0.31603
Q85601	Q9C0H6	0.29592
Q85601	P0C7X5	0.34201
Q85601	P12525	0.24393
Q85601	Q8WV22	0.30525
Q85601	Q07021	0.84521
Q85601	P23490	0.76464
Q85601	B2RXF5	0.39835
Q85601	Q9BV97	0.31257
Q85601	O95171	0.22473
Q85601	O43900	0.34039
Q85601	Q86TU6	0.40682
Q85601	O75175	1.3902
Q85601	Q9H6R4	0.25211
Q85601	Q6ZNK6	0.47043
Q85601	Q8NFC6	0.44287
Q85601	Q9H9Z2	0.72802
Q85601	Q9UND3	0.35037
Q85601	Q12837	0.55614
Q85601	Q13619	0.40128
Q85601	O43172	0.62322
Q85601	Q7Z417	0.7281
Q85601	Q9NVN8	0.31781
Q85601	Q14511	0.74532
Q85601	Q8TAQ2	1.3452
Q85601	Q9UBC9	0.49294
Q85601	P08865	0.81009
Q85601	Q6P0N0	0.92676
Q85601	Q06609	0.47698

Q85601	Q14137	0.80661
Q85601	Q6P1N0	0.36804
Q85601	Q6AW86	0.2426
Q85601	Q9UKL0	1.2317
Q85601	O60244	0.53444
Q85601	A8MWS5	0.031834
Q85601	Q9ULC4	0.4109
Q85601	Q9H211	0.71344
Q85601	Q96E16	0.56465
Q85601	O14628	0.47825
Q85601	Q9Y399	0.69618
Q85601	P0CG24	0.25538
Q85601	O94874	0.84465
Q85601	Q9NR46	0.44185
Q85601	Q13485	0.61727
Q85601	Q8N0Y2	0.19248
Q85601	Q5JRK9	0.48065
Q85601	Q9NTG7	0.58959
Q85601	P62380	0.15879
Q85601	Q5CZC0	0.57575
Q85601	Q9GZM3	0.078638
Q85601	P61247	1.0467
Q85601	Q9C0D2	0.32222
Q85601	Q9BVI4	0.33254
Q85601	Q86XK3	0.72103
Q85601	Q9Y5B6	0.5411
Q85601	Q9H204	1.2311
Q85601	Q96HR3	0.78003
Q85601	Q15003	0.81542
Q85601	Q96CM3	0.33888
Q85601	Q5UE93	0.50691
Q85601	Q96PQ6	0.45965
Q85601	Q9UHN1	0.15329
Q85601	O14818	0.52419
Q85601	Q9NSJ1	0.32039
Q85601	Q7Z3B4	0.86448
Q85601	P48454	0.15271
Q85601	Q03111	1.3273
Q85601	Q8IYN6	0.15623
Q85601	Q9P278	0.79241
Q85601	Q93075	0.36782
Q85601	Q9UNH7	0.21881
Q85601	Q8WTR7	0.61682
Q85601	Q86UE3	0.34313
Q85601	Q96A49	1.1033
Q85601	P62937	0.68795
Q85601	O75925	0.64922
Q85601	O14737	0.18434
Q85601	Q8N9B5	0.80931
Q85601	Q96E14	0.28852
Q85601	Q96FF9	0.88564
Q85601	P16989	0.27533
Q85601	O95817	0.44201
Q85601	Q9BSN8	0.46923
Q85601	Q9UMX1	0.66109
Q85601	P69208	0.21862
Q85601	Q5T1C6	0.38073
Q85601	Q9H583	1.0138
Q85601	Q96LR2	0.579
Q85601	P78395	0.73702
Q85601	Q9Y2X3	0.68675
Q85601	Q9UN37	0.51619
Q85601	O43482	0.49992
Q85601	Q9BYR6	0.36976
Q85601	Q6P1K2	1.2022
Q85601	P00338	0.51979
Q85601	Q8WXB4	0.4507
Q85601	Q14257	0.59027
Q85601	O75564	0.32629
Q85601	Q9GZM8	0.11916
Q85601	Q9BUP3	0.15572
Q85601	A6NEV1	0.016027
Q85601	O14595	0.27475
Q85601	Q06730	0.1945
Q85601	O14662	0.50559
Q85601	Q6NXP2	0.56949

Q85601	E9PI22	0.12166
Q85601	Q6QNY1	0.79556
Q85601	P78347	0.59367
Q85601	Q8NHY2	1.1508
Q85601	Q6K0P9	0.25406
Q85601	Q93074	0.48189
Q85601	O75683	0.18732
Q85601	Q9UHR5	1.233
Q85601	Q7RTV3	0.42458
Q85601	Q9P000	0.15603
Q85601	Q14232	0.13937
Q85601	Q5H9R7	0.89784
Q85601	Q06587	0.8641
Q85601	Q3LI68	0.56205
Q85601	Q9Y237	0.38149
Q85601	Q9GZR1	0.44343
Q85601	Q9Y253	0.38148
Q85601	P24941	0.64414
Q85601	Q9H091	0.3431
Q85601	Q99700	0.7383
Q85601	O75391	0.40327
Q85601	Q8TBC5	0.14382
Q85601	Q9BY31	0.20447
Q85601	Q9UNA4	0.38806
Q85601	Q14498	0.6239
Q85601	Q9UBD0	1.1552
Q85601	Q9HB71	0.55656
Q85601	Q9Y3E7	0.80521
Q85601	Q9Y623	0.51716
Q85601	Q8TF09	0.37628
Q85601	Q9UBB5	1.1835
Q85601	Q8NEG2	0.14697
Q85601	Q969Q6	0.30938
Q85601	P62736	0.1273
Q85601	P42025	0.42566
Q85601	Q9HD42	1.0544
Q85601	Q7L8S5	0.1957
Q85601	Q14264	0.13723
Q85601	P27695	0.47938
Q85601	Q05639	0.48937
Q85601	A6QL64	0.1977
Q85601	O75478	0.47717
Q85601	Q8NC26	0.43748
Q85601	Q9H3M7	0.80144
Q85601	P0C7X3	0.21452
Q85601	Q9NZI5	0.55032
Q85601	Q13907	0.36557
Q85601	Q8TAT6	0.54294
Q85601	Q96KN1	0.41804
Q85601	Q8IXQ9	0.40774
Q85601	Q6NX49	0.23154
Q85601	P11161	0.48778
Q85601	Q9Y6X3	1.0014
Q85601	Q9UKF6	0.65374
Q85601	Q9P0J6	0.28738
Q85601	Q5TEC3	0.30549
Q85601	P52294	1.099
Q85601	Q9H4S2	0.25468
Q85601	Q8N5R6	0.46276
Q85601	Q01664	0.54678
Q85601	Q86Y25	0.32151
Q85601	Q5PRF9	0.67729
Q85601	Q9BVP2	0.22073
Q85601	Q53GL7	0.52503
Q85601	Q9HB07	0.2432
Q85601	Q92539	0.64055
Q85601	O43167	0.66443
Q85601	Q9UL42	0.84009
Q85601	O00161	0.86656
Q85601	Q9NY43	0.42222
Q85601	Q99676	0.33301
Q85601	Q9NU63	0.26501
Q85601	Q58F21	0.28359
Q85601	Q9UEG4	0.32458
Q85601	Q86VP3	0.24427
Q85601	P53539	0.05508

Q85601	P17861	0.64185
Q85601	Q9Y3Y4	0.72184
Q85601	P30084	0.14954
Q85601	Q6I9Y2	0.70776
Q85601	O15519	0.24415
Q85601	A6NI79	0.79453
Q85601	Q96HW7	0.58748
Q85601	O75821	0.45305
Q85601	Q1W6H9	1.0334
Q85601	O14777	0.64654
Q85601	Q8TAE8	0.98919
Q85601	P31269	0.61174
Q85601	Q96QG7	0.27773
Q85601	Q96N03	0.30691
Q85601	Q96PE6	0.25817
Q85601	P49790	1.1051
Q85601	Q01860	0.71185
Q85601	Q15544	1.0988
Q85601	O14802	0.23159
Q85601	Q14676	0.67893
Q85601	Q8N1A0	0.51757
Q85601	Q9UNY4	0.54198
Q85601	Q03060	0.52421
Q85601	Q96AQ6	0.6418
Q85601	P09132	0.19159
Q85601	O00716	1.0909
Q85601	Q9UID3	0.058561
Q85601	Q9BR11	0.030716
Q85601	O43684	0.73129
Q85601	Q9H246	0.062588
Q85601	A6NF83	0.54771
Q85601	P63167	1.4751
Q85601	Q07866	0.69561
Q85601	Q6ZN30	0.27806
Q85601	O15382	0.083993
Q85601	Q9BWM5	0.31039
Q85601	O14604	0.3618
Q85601	Q8IZD2	0.38167
Q85601	Q14493	0.76474
Q85601	Q8WTV1	0.9441
Q85601	O00139	0.47037
Q85601	Q5JNZ5	0.58749
Q85601	P46100	0.70071
Q85601	P52743	0.35842
Q85601	Q9NXR7	0.45196
Q85601	Q9H5J8	0.98599
Q85601	Q96K75	0.28997
Q85601	Q13227	0.89008
Q85601	Q96B26	0.66329
Q85601	Q7L5N1	1.1436
Q85601	O60832	0.62067
Q85601	Q00444	0.26861
Q85601	O75410	0.81047
Q85601	Q8TF46	0.24037
Q85601	Q15072	0.26529
Q85601	P55895	0.72084
Q85601	Q8N5D0	0.55289
Q85601	Q08379	0.31744
Q85601	O60645	0.40819
Q85601	P0C7I6	0.56164
Q85601	P46777	1.108
Q85601	Q14749	0.6289
Q85601	P07320	0.50881
Q85601	Q13105	0.51774
Q85601	Q9HBM1	0.76436
Q85601	P52747	0.79534
Q85601	P28324	1.4499
Q85601	Q9Y3V2	0.87447
Q85601	Q8TEE9	0.96706
Q85601	Q9Y224	1.5525
Q85601	Q96EY1	0.43061
Q85601	Q86UT8	0.63409
Q85601	Q9UJY1	0.44665
Q85601	Q8N5M1	0.46241
Q85601	Q96G03	0.34124
Q85601	Q9H0B6	0.69114

Q85601	O15318	0.32016
Q85601	Q969I3	0.28917
Q85601	Q6UB98	0.44273
Q85601	Q9NPJ6	0.88491
Q85601	Q13887	0.40176
Q85601	P20749	0.41947
Q85601	P78318	0.70034
Q85601	Q9UKT9	0.49262
Q85601	Q9H4B6	0.6298
Q85601	Q9UJT2	0.51896
Q85601	Q96PM9	0.4507
Q85601	Q99638	0.60089
Q85601	P53992	1.05
Q85601	Q9BYQ4	0.17342
Q85601	P17035	0.25157
Q85601	Q05084	0.42095
Q85601	P09661	0.48367
Q85601	Q8NCF5	0.5366
Q85601	Q9NSB2	0.52968
Q85601	P51587	0.20156
Q85601	Q6IQ49	0.22664
Q85601	Q96I24	0.46909
Q85601	P61457	1.0544
Q85601	Q86W56	0.78896
Q85601	Q86V20	0.53968
Q85601	Q9NXV6	0.6297
Q85601	O95551	0.85587
Q85601	Q9BSW2	0.81632
Q85601	Q495M9	0.30418
Q85601	Q8NF64	1.3998
Q85601	Q9UJG1	0.52318
Q85601	O43918	1.1122
Q85601	Q15631	0.67878
Q85601	P40926	0.2868
Q85601	P62917	0.8426
Q85601	Q7Z353	0.22476
Q85601	Q96AV8	0.36573
Q85601	Q9ULD2	0.50254
Q85601	A6NCI8	0.74095
Q85601	Q9ULX9	0.43921
Q85601	Q8NEQ6	0.13649
Q85601	Q16401	0.69289
Q85601	Q96N16	0.82953
Q85601	Q07687	0.45813
Q85601	Q86Y46	0.38819
Q85601	Q9H0R1	0.22943
Q85601	Q8TAU3	0.30086
Q85601	Q9BT81	0.41682
Q85601	Q86X51	0.021782
Q85601	Q9NSD4	0.3085
Q85601	Q8HWS3	0.32893
Q85601	Q9BSC4	0.22094
Q85601	Q9NVC6	0.57019
Q85601	Q8WYK2	0.33701
Q85601	Q92901	0.53692
Q85601	O60784	0.37325
Q85601	Q14938	0.21226
Q85601	O43513	1.1362
Q85601	A6NGN4	0.31479
Q85601	P33240	0.37136
Q85601	Q9BRV8	0.57655
Q85601	Q86XI8	0.48333
Q85601	Q15561	0.86197
Q85601	Q15021	0.90272
Q85601	A6NCL1	0.23306
Q85601	Q86V87	0.29416
Q85601	Q5JVG8	0.43626
Q85601	Q9ULJ6	0.67462
Q85601	Q9Y2G9	0.50646
Q85601	P81877	0.47332
Q85601	Q8TDH9	0.16483
Q85601	Q68CZ6	0.65651
Q85601	O95721	0.41187
Q85601	Q8IUC2	0.19997
Q85601	Q01844	0.60555
Q85601	Q15366	0.84759

Q85601	Q86VW0	0.21411
Q85601	Q69YH5	0.86736
Q85601	P98182	0.24385
Q85601	Q9ULD9	1.0374
Q85601	Q86SX3	1.3695
Q85601	Q99856	0.69112
Q85601	Q9H1B7	1.0383
Q85601	Q01094	0.79845
Q85601	Q9HC52	0.82264
Q85601	Q6P161	0.096586
Q85601	Q9UMX0	0.75358
Q85601	O14770	0.47655
Q85601	P23760	0.89634
Q85601	Q96DB5	0.40071
Q85601	Q9Y605	1.0114
Q85601	Q8N7H5	0.93884
Q85601	Q9UI10	0.34591
Q85601	Q9NZI7	0.2809
Q85601	O00189	0.39573
Q85601	Q13136	0.27847
Q85601	P78560	0.52916
Q85601	P18124	0.53419
Q85601	P46781	0.93932
Q85601	O15144	0.22199
Q85601	Q9Y4X5	0.51813
Q85601	P49419	0.60607
Q85601	Q16186	0.59685
Q85601	Q5FBB7	0.84022
Q85601	A6NG73	0.26877
Q85601	Q9P2M7	0.44775
Q85601	Q86X95	1.1472
Q85601	P0CJ86	0.27808
Q85601	P40855	0.18588
Q85601	Q7L1V2	0.64982
Q85601	Q96GV9	0.42859
Q85601	P62875	0.16371
Q85601	P11308	0.5673
Q85601	P51955	0.28986
Q85601	P15408	0.58289
Q85601	O43805	0.62133
Q85601	O95997	0.95013
Q85601	O95198	0.43876
Q85601	Q6NVV1	0.30606
Q85601	Q93034	0.7442
Q85601	Q9NYF8	1.2225
Q85601	Q86Y13	0.35301
Q85601	A6NLW8	0.27808
Q85601	Q86SZ2	0.54013
Q85601	Q9NVW2	0.45447
Q85601	P54274	0.46876
Q85601	Q96GG9	0.26518
Q85601	Q9NR55	0.3675
Q85601	P55197	1.5024
Q85601	Q9UHR6	0.88837
Q85601	Q99698	0.23958
Q85601	Q8TD31	0.18278
Q85601	P25398	0.67193
Q85601	Q8NCY6	0.066453
Q85601	P60413	0.17342
Q85601	Q9NUQ3	0.71742
Q85601	Q8N9G6	0.58181
Q85601	Q6MZP7	0.61771
Q85601	Q9BUH8	0.43846
Q85601	Q9UHA2	0.13257
Q85601	Q8IY31	0.55516
Q85601	Q70EL1	0.37528
Q85601	Q9Y5J3	0.33498
Q85601	Q8N7K0	0.35685
Q85601	Q2NL98	0.42183
Q85601	Q5VSR9	0.17798
Q85601	Q86YF9	0.15496
Q85601	Q9ULQ0	0.72106
Q85601	O00410	1.2006
Q85601	Q96LA8	0.73526
Q85601	Q5JVX7	0.25806
Q85601	Q8IX15	0.52968

Q85601	P60866	0.58002
Q85601	Q9H7C4	0.39325
Q85601	P13640	0.70858
Q85601	P17039	0.24382
Q85601	Q96DF8	0.87719
Q85601	Q8NA72	0.33054
Q85601	Q12874	0.4347
Q85601	Q14739	0.26015
Q85601	Q9UJW9	1.3317
Q85601	Q6L8Q7	0.21804
Q85601	Q5T0F9	0.4287
Q85601	P15884	0.63294
Q85601	Q5D1E8	0.55548
Q85601	O76014	0.56535
Q85601	Q6P2D0	0.24529
Q85601	Q15599	0.46943
Q85601	A6NP61	0.38149
Q85601	Q8WVJ9	0.61808
Q85601	Q13794	0.42491
Q85601	Q99747	0.52433
Q85601	Q86YD1	0.65604
Q85601	Q9H9Q4	0.49527
Q85601	O43809	0.59647
Q85601	Q96ME1	0.018414
Q85601	Q9NQE9	0.43583
Q85601	O43236	0.35007
Q85601	Q96M91	0.3251
Q85601	Q8N393	0.2815
Q85601	Q9HCX3	0.2381
Q85601	Q9NX46	0.46527
Q85601	Q6ZNH5	0.29511
Q85601	Q96IU2	0.84291
Q85601	Q96NB1	0.18363
Q85601	P55210	0.2495
Q85601	Q9NWD9	0.33708
Q85601	P60409	0.17342
Q85601	Q9Y5S9	0.60352
Q85601	Q6P9A1	0.4386
Q85601	P51114	0.046507
Q85601	Q15796	0.62535
Q85601	Q99619	0.56371
Q85601	Q14839	0.76144
Q85601	Q02543	0.67535
Q85601	Q9BVW5	1.0943
Q85601	Q8NCE0	0.44197
Q85601	Q8NI08	0.78563
Q85601	P13010	0.46288
Q85601	P17482	1.0884
Q85601	Q9H503	0.34451
Q85601	Q96FZ2	0.81589
Q85601	Q5TBE3	0.27805
Q85601	Q96CB9	0.62356
Q85601	Q5VZF2	0.098392
Q85601	Q9Y5P8	0.61544
Q85601	Q2NL82	0.27965
Q85601	Q13287	0.53128
Q85601	Q96FN9	0.19276
Q85601	Q86UP8	0.069336
Q85601	Q5T953	0.10715
Q85601	P12956	0.41369
Q85601	Q96LW9	0.29347
Q85601	P20226	0.99054
Q85601	Q6ZMV5	0.24311
Q85601	Q96IV0	0.54749
Q85601	P20265	0.63686
Q85601	Q9HCM9	0.51307
Q85601	Q5EBL4	0.48196
Q85601	P45877	0.41838
Q85601	Q7Z7F1	0.2923
Q85601	Q9BWJ5	0.39471
Q85601	Q8N6U2	0.1156
Q85601	P0C7M6	0.015638
Q85601	O95359	0.38902
Q85601	Q8IYX8	0.14399
Q85601	Q10586	0.41727
Q85601	Q9HCM7	0.68293

Q85601	Q9ULV3	0.30359
Q85601	P62906	0.5806
Q85601	Q16670	0.27829
Q85601	P10073	0.37085
Q85601	Q8TEL6	0.4024
Q85601	Q5VZL5	0.66684
Q85601	Q8N490	0.36256
Q85601	O00321	0.33555
Q85601	Q9NX55	0.28432
Q85601	Q96P63	0.53297
Q85601	P50914	0.91075
Q85601	P78316	0.81914
Q85601	Q9ULW6	0.63254
Q85601	Q719H9	0.79244
Q85601	O95238	0.28217
Q85601	Q6KB66	0.27556
Q85601	O95865	0.41795
Q85601	O43257	0.32369
Q85601	Q8N3J5	0.14718
Q85601	Q9Y2Q1	0.5019
Q85601	P0CJ85	0.27808
Q85601	P47928	0.43184
Q85601	Q9UBC3	0.29334
Q85601	Q13501	0.49482
Q85601	Q6IS14	0.27703
Q85601	Q8TBY8	0.67192
Q85601	Q16143	0.40639
Q85601	Q9BQE4	0.34004
Q85601	Q9P086	0.99267
Q85601	Q5ZEY9	0.65143
Q85601	P05204	0.3992
Q85601	Q3BBV0	0.39434
Q85601	Q96RT7	0.44028
Q85601	Q8WXD5	0.86569
Q85601	P46527	0.35121
Q85601	Q68DY1	0.47479
Q85601	Q9NVU7	0.45699
Q85601	Q5TBB1	0.56804
Q85601	P0C860	0.39201
Q85601	Q8IWV8	0.312
Q85601	Q96CW6	0.39619
Q85601	Q13278	0.1053
Q85601	Q13094	0.59128
Q85601	Q9UN81	0.080004
Q85601	P49910	0.75423
Q85601	Q86TJ2	0.54197
Q85601	Q9H840	0.86538
Q85601	Q9HCI5	0.63819
Q85601	O75177	0.65721
Q85601	Q96MF7	0.68427
Q85601	Q5VT98	0.19615
Q85601	Q9BZK3	0.44131
Q85601	Q6W2J9	0.42563
Q85601	P62266	0.90541
Q85601	Q8N988	0.45816
Q85601	Q92748	1.0327
Q85601	Q9NYT6	0.25802
Q85601	P55345	0.38065
Q85601	Q9H1L0	0.011592
Q85601	Q9BRA0	0.23411
Q85601	O96028	0.44617
Q85601	Q96H12	0.59705
Q85601	A1L190	0.59889
Q85601	Q15102	0.23493
Q85601	Q6IEG0	0.38447
Q85601	Q9NS91	0.29817
Q85601	Q5T4T6	1.1728
Q85601	O75081	1.2518
Q85601	P49750	1.0592
Q85601	Q8IU60	0.87575
Q85601	A8MZA4	0.43599
Q85601	P40429	0.76028
Q85601	Q9HBE1	0.18618
Q85601	P52741	0.26593
Q85601	Q8N9Y4	1.0652
Q85601	Q96KW2	0.51785

Q85601	O75674	0.40441
Q85601	O15481	0.0040392
Q85601	O75791	1.0297
Q85601	Q96IR2	0.3621
Q85601	Q8N3C0	0.37436
Q85601	O60299	0.4493
Q85601	Q13542	0.30615
Q85601	Q96QF0	0.61439
Q85601	Q8NEZ5	0.80077
Q85601	Q14693	0.40171
Q85601	Q8WVP5	0.51467
Q85601	P17021	0.21987
Q85601	Q13561	0.9038
Q85601	O15061	0.69172
Q85601	Q969P5	0.18526
Q85601	P35227	0.76277
Q85601	A8MYZ0	0.14353
Q85601	O95561	0.43524
Q85601	Q9UBC1	0.48716
Q85601	Q8ND56	0.40244
Q85601	P35638	0.7996
Q85601	P32929	0.2836
Q85601	O95782	0.44251
Q85601	P29372	0.48233
Q85601	Q9UNK9	0.1279
Q85601	Q12986	0.30709
Q85601	Q8WWL7	0.93873
Q85601	P98168	0.73128
Q85601	Q13761	0.78343
Q85601	P24864	0.64356
Q85601	P19367	0.3186
Q85601	Q9NPF5	0.95515
Q85601	Q96RS6	0.23411
Q85601	Q9UJ78	0.1443
Q85601	Q5MJ70	0.15217
Q85601	Q5SW24	0.17152
Q85601	Q9BYG4	0.73056
Q85601	Q92988	0.51984
Q85601	P0CJ78	0.22175
Q85601	Q8N9T8	0.89002
Q85601	Q6XD76	0.3033
Q85601	O60934	0.21425
Q85601	P62888	0.67122
Q85601	P41208	0.67183
Q85601	Q969F1	0.57203
Q85601	Q9UNQ2	0.22422
Q85601	Q9NRD1	0.22461
Q85601	P36543	0.25967
Q85601	Q96GE5	0.44626
Q85601	Q5F1R6	0.13209
Q85601	P59047	0.32635
Q85601	Q9H1J1	0.7755
Q85601	Q9HAZ2	0.41573
Q85601	Q14781	0.78798
Q85601	Q9BZW7	0.32881
Q85601	Q14CS0	0.49805
Q85601	Q8NCU7	0.35559
Q85601	Q96ST3	0.75696
Q85601	Q9H9D4	0.59532
Q85601	Q96K58	0.24355
Q85601	Q03938	0.49372
Q85601	Q93062	0.71565
Q85601	O75505	0.22476
Q85601	Q9UNS1	0.58477
Q85601	Q6P1L8	0.48917
Q85601	Q8TBZ0	0.42109
Q85601	Q9UH90	0.47944
Q85601	P25963	0.65776
Q85601	P63313	0.34646
Q85601	Q9Y314	1.0165
Q85601	Q13617	0.63773
Q85601	P43357	0.30691
Q85601	Q15819	0.60636
Q85601	Q93073	0.50624
Q85601	P17041	0.20836
Q85601	Q9Y4Y9	0.65294

Q85601	O60381	0.20822
Q85601	Q9H2U1	0.30851
Q85601	A5D8V6	0.33266
Q85601	Q69YI7	0.92402
Q85601	P05813	0.11642
Q85601	P10589	0.337
Q85601	Q3KRA6	0.53899
Q85601	Q8IUR0	0.78953
P03346	O95166	0.35361
P03346	Q96HJ3	0.0077221
P03346	O43402	0.58768
P03346	Q3LI63	0.36736
P03346	O75390	0.2886
P03346	Q5T751	0.02884
P03346	Q13439	0.91311
P03346	Q86VQ1	0.20041
P03346	Q15172	0.49945
P03346	Q8TEM1	0.3066
P03346	Q13425	0.67643
P03346	Q08J23	0.77685
P03346	Q6PCB5	0.32217
P03346	O60921	0.92444
P03346	P08107	0.62758
P03346	Q86UQ0	0.27382
P03346	Q9HCE5	0.2425
P03346	Q9BVJ6	1.0223
P03346	P13646	0.40838
P03346	E7ETH6	0.29208
P03346	Q9UPR3	1.1579
P03346	Q6PRD7	0.20041
P03346	Q96T92	0.4754
P03346	Q9Y6N9	0.45149
P03346	Q9NVU0	0.47837
P03346	Q15723	1.5382
P03346	Q5F1R6	0.29473
P03346	A6NK53	0.4412
P03346	O60437	0.95254
P03346	Q6R2W3	0.66866
P03346	A6NKD9	0.21102
P03346	P02792	0.20776
P03346	Q92681	0.65264
P03346	Q12888	0.93194
P03346	Q13596	0.47468
P03346	O75179	0.54311
P03346	Q6P3S1	0.27403
P03346	Q5JU85	0.25055
P03346	P31271	0.35862
P03346	Q06330	0.63292
P03346	B2CW77	0.37007
P03346	O15014	0.16261
P03346	Q9NVM9	1.2737
P03346	P37802	0.61307
P03346	Q56VL3	0.10111
P03346	Q8TEX9	0.72929
P03346	Q99081	0.71921
P03346	Q9BSI4	0.40892
P03346	A8MU46	0.35118
P03346	Q6KB66	0.51027
P03346	Q9NSC5	0.65663
P03346	Q7Z3K3	0.63786
P03346	Q15773	0.4712
P03346	Q9NZJ0	0.87744
P03346	P85037	0.30304
P03346	Q9NPG3	1.0533
P03346	Q5VZR2	0.13835
P03346	Q3C1V8	0.95636
P03346	Q8IYI6	0.72129
P03346	Q06945	0.26791
P03346	P43357	0.66787
P03346	Q9BW60	0.17942
P03346	A8MU93	0.0077221
P03346	Q66LE6	0.54012
P03346	Q96G01	0.30728
P03346	Q5T870	0.016444
P03346	P59282	0.20041
P03346	P0CL83	0.32217

P03346	Q9NQG7	0.47425
P03346	Q9Y606	0.54269
P03346	Q9BSU1	0.48715
P03346	P15924	0.39773
P03346	P10244	0.90277
P03346	Q6SJ96	0.62171
P03346	Q9NSI2	0.0077221
P03346	Q3KNS6	0.31436
P03346	P53805	0.89744
P03346	Q5T5Y3	0.24315
P03346	Q9NQ39	0.26641
P03346	O75382	0.48695
P03346	Q0VFZ6	0.0077221
P03346	Q15833	0.50672
P03346	Q14781	1.0654
P03346	Q13588	0.28579
P03346	Q9BRD0	0.13004
P03346	Q9Y5S9	0.96812
P03346	Q8NEA9	0.49726
P03346	Q8N1N4	0.51027
P03346	P62136	0.62219
P03346	Q9UI26	0.66815
P03346	Q96NG5	0.31256
P03346	O00204	0.59274
P03346	Q8IYK2	0.089753
P03346	P54105	1.0322
P03346	Q5JVG2	0.31436
P03346	Q9NPC8	0.37474
P03346	Q5SZL2	0.35785
P03346	Q5BKX5	0.0077221
P03346	Q9UBW5	0.8379
P03346	Q96FX7	0.26326
P03346	Q96FV9	1.1932
P03346	Q6P582	0.38127
P03346	Q96A35	0.36869
P03346	Q9Y6D9	0.93575
P03346	Q9Y3Z3	0.24674
P03346	Q9NRI5	0.41938
P03346	O95400	0.99518
P03346	Q9H0A8	0.8379
P03346	P21283	0.2957
P03346	Q969S3	0.77714
P03346	Q13886	0.26545
P03346	O14925	0.3672
P03346	Q969S8	0.90291
P03346	Q9H4A5	0.015986
P03346	Q9BZI7	0.87372
P03346	O15265	1.1472
P03346	P41567	0.24325
P03346	Q9Y6Y1	0.8829
P03346	P08048	0.31436
P03346	Q86UT5	0.35488
P03346	P25787	0.58476
P03346	Q9BZE0	0.39211
P03346	Q9H5V9	0.01482
P03346	Q9NS91	0.40413
P03346	Q9BYR9	0.40421
P03346	Q5JUW0	0.18405
P03346	O60664	0.83759
P03346	Q12901	0.30845
P03346	P30281	0.92277
P03346	Q86TC9	0.65924
P03346	O60739	0.15258
P03346	Q05481	0.30845
P03346	Q6ZU52	0.66787
P03346	Q14695	0.018128
P03346	Q9Y6J9	1.0763
P03346	Q96CN9	0.7532
P03346	Q96AP0	0.3118
P03346	Q9BTM9	0.8612
P03346	Q9Y6X9	0.37702
P03346	Q8N7U7	0.66978
P03346	P31249	0.82379
P03346	Q8TC90	0.047438
P03346	Q8N2Z9	0.68496
P03346	P21980	0.36432

61485	P03346	Q6PD62	0.82089
61486	P03346	Q7KZ85	0.67774
61487	P03346	P0C860	0.66665
61488	P03346	O95201	0.85529
61489	P03346	Q9NWS9	0.34285
61490	P03346	O75691	0.96902
61491	P03346	Q96ST3	0.85354
61492	P03346	Q9UPN9	0.61911
61493	P03346	Q9Y6K9	0.85059
61494	P03346	Q9Y2A0	0.32273
61495	P03346	P12004	0.30954
61496	P03346	Q75MW2	0.013232
61497	P03346	Q86YV9	0.035399
61498	P03346	Q6P9B9	0.41065
61499	P03346	P49716	0.7783
61500	P03346	P54821	0.50689
61501	P03346	Q96J02	0.32915
61502	P03346	P19012	1.0238
61503	P03346	Q7Z398	0.31436
61504	P03346	Q8NI51	0.74193
61505	P03346	Q8N3V7	0.32588
61506	P03346	O76021	0.35979
61507	P03346	Q9UKA2	0.68012
61508	P03346	O75995	0.29221
61509	P03346	Q96AB3	0.94726
61510	P03346	Q9Y253	0.451
61511	P03346	O14490	0.4623
61512	P03346	Q96D70	0.32442
61513	P03346	Q9BXU7	0.37305
61514	P03346	P59047	0.40262
61515	P03346	Q03989	0.64621
61516	P03346	Q8NB91	0.47294
61517	P03346	P23381	0.39962
61518	P03346	Q9NVS2	0.35603
61519	P03346	Q9NS26	0.37392
61520	P03346	B1AL46	0.083147
61521	P03346	Q6PB30	0.12075
61522	P03346	Q96AC1	0.27921
61523	P03346	Q15427	0.63876
61524	P03346	P47902	0.52805
61525	P03346	Q15058	0.4559
61526	P03346	Q9HBH1	0.1523
61527	P03346	P05387	0.66528
61528	P03346	Q9Y399	0.42537
61529	P03346	Q05DH4	0.11947
61530	P03346	Q5T6C5	0.021938
61531	P03346	P05412	0.74008
61532	P03346	Q9UKS7	0.3141
61533	P03346	Q4G0F5	0.37782
61534	P03346	Q96A04	0.19265
61535	P03346	Q96N20	0.28776
61536	P03346	P51991	0.29183
61537	P03346	O00213	0.48095
61538	P03346	Q6P444	0.12222
61539	P03346	O95639	0.44661
61540	P03346	P08729	1.4081
61541	P03346	Q8NHW5	0.40389
61542	P03346	Q9Y467	0.30822
61543	P03346	Q92585	1.1044
61544	P03346	Q0P6D6	0.17073
61545	P03346	P17097	0.12564
61546	P03346	Q9H0H0	0.28295
61547	P03346	Q9H5Y0	0.11048
61548	P03346	P46779	1.1135
61549	P03346	Q96CJ1	1.4506
61550	P03346	Q3YBM2	0.10902
61551	P03346	Q9ULC6	0.096187
61552	P03346	Q3SY00	0.074014
61553	P03346	Q96GY3	0.79591
61554	P03346	Q9ULR5	0.77353
61555	P03346	Q969H4	0.37958
61556	P03346	Q8IUC2	0.36736
61557	P03346	P56279	0.71939
61558	P03346	Q9BVL4	0.042492
61559	P03346	Q3KNV8	1.0307
61560	P03346	Q9UQK1	0.56439

P03346	Q13077	0.51585
P03346	Q12874	0.68494
P03346	Q7Z713	0.49807
P03346	Q12830	0.99924
P03346	P78424	0.58965
P03346	Q9NZU5	0.40809
P03346	P61966	0.38948
P03346	Q15542	1.0313
P03346	P63119	0.25941
P03346	Q9UHL9	0.62534
P03346	Q96LW1	0.31436
P03346	O75603	0.2075
P03346	Q9GZT8	1.0537
P03346	P02538	0.96257
P03346	Q99615	1.026
P03346	Q9BRR8	0.091185
P03346	Q9Y3A2	1.0224
P03346	Q9UFG5	0.66787
P03346	P82930	0.37322
P03346	O00463	0.50566
P03346	Q5T6F0	0.33351
P03346	Q5RKV6	0.37388
P03346	Q9H2U2	0.49126
P03346	P09972	0.61814
P03346	Q8IY22	0.3715
P03346	P55735	0.79328
P03346	Q99828	0.34258
P03346	P49639	0.43096
P03346	Q7Z7A1	1.252
P03346	P43378	0.26119
P03346	Q16621	0.70942
P03346	P46063	0.26106
P03346	Q8ND76	0.74759
P03346	P57071	0.31436
P03346	Q9BT25	0.61124
P03346	Q5T5D7	0.31436
P03346	Q86U42	0.87824
P03346	Q5FBB7	0.79884
P03346	O75592	0.22812
P03346	Q4VC44	0.53049
P03346	Q9H7Z3	0.12821
P03346	O15234	1.0632
P03346	Q9NRX4	0.17976
P03346	Q7Z7K2	0.31436
P03346	Q9NWT1	0.31438
P03346	Q96PE7	0.23435
P03346	Q96MU7	1.0368
P03346	Q9NWQ4	0.025377
P03346	Q9NQ92	1.1894
P03346	P61978	1.2176
P03346	Q99742	0.37607
P03346	Q86V48	0.272
P03346	Q5VIR6	0.78353
P03346	Q6L8H1	0.40421
P03346	Q8WYQ3	0.15058
P03346	P28070	0.41915
P03346	Q9HBT8	0.31436
P03346	P15863	0.29181
P03346	Q96FJ2	0.78047
P03346	Q8TA86	0.70811
P03346	Q15562	1.0268
P03346	Q9UGC7	0.37518
P03346	Q9UKT7	0.67239
P03346	Q969W3	0.079386
P03346	Q6T4R5	0.22607
P03346	Q9UHF7	0.72708
P03346	O95456	0.74679
P03346	Q9Y238	0.18007
P03346	Q86XT2	0.23325
P03346	P46937	0.85015
P03346	Q96S65	0.80066
P03346	Q13487	0.84503
P03346	Q9H0C8	0.55545
P03346	Q9ULR0	0.49972
P03346	Q13263	0.33358
P03346	Q9NZR4	0.31775

P03346	Q96RK0	1.3467
P03346	Q86Y46	0.51027
P03346	Q494X3	0.31436
P03346	Q9Y3Y2	0.80207
P03346	Q01534	0.50617
P03346	Q8TDG2	0.18249
P03346	A8MXY4	0.31436
P03346	O15042	0.61867
P03346	Q1ED39	0.48532
P03346	O60443	0.13037
P03346	Q9H1M0	0.25042
P03346	Q6L8G8	0.40421
P03346	Q9BXS6	0.53688
P03346	P35453	0.37872
P03346	A5PLN7	0.0077221
P03346	Q9NQX6	0.31436
P03346	Q9BU61	0.93433
P03346	Q15370	1.1586
P03346	A6NCI8	0.088756
P03346	Q504Q3	0.35865
P03346	Q9C0I3	0.074845
P03346	Q9ULM6	1.1227
P03346	Q9P267	0.24689
P03346	Q9Y2B0	0.35908
P03346	Q9H6D7	0.6137
P03346	O00743	0.86072
P03346	Q96P70	1.2452
P03346	Q16589	0.54657
P03346	Q9Y4Z2	0.4895
P03346	O75364	0.37074
P03346	O43752	0.29222
P03346	P50135	0.29983
P03346	Q8N0Z2	0.52744
P03346	P31260	0.68712
P03346	Q9NR83	0.46284
P03346	Q96QR8	0.49779
P03346	Q5TD97	0.25563
P03346	Q96DF8	0.43067
P03346	P12524	0.66312
P03346	Q96QK1	0.95549
P03346	Q96GA3	0.65405
P03346	Q9C075	0.47854
P03346	O43172	1.0046
P03346	Q7Z4G1	1.1129
P03346	Q06520	0.35198
P03346	Q9BXT5	0.054477
P03346	Q9H1A3	0.66787
P03346	O75564	0.36833
P03346	Q9ULX3	0.23621
P03346	P78396	1.0035
P03346	Q14746	0.61116
P03346	O60346	0.25925
P03346	Q08AI8	0.0077221
P03346	Q6RSH7	0.60896
P03346	Q96HJ5	0.2721
P03346	Q9UBS3	0.80265
P03346	P98182	0.32702
P03346	O95405	0.29681
P03346	Q15438	0.28448
P03346	Q86Z20	0.19101
P03346	Q8NFH4	0.9376
P03346	Q7Z5V6	0.69317
P03346	Q3SY84	0.48986
P03346	Q9UBB4	0.72181
P03346	Q99698	0.3692
P03346	O75608	0.31836
P03346	Q9NRZ9	0.26085
P03346	Q16352	0.48875
P03346	Q14320	0.23226
P03346	Q14525	0.47854
P03346	Q15050	0.51875
P03346	B5ME19	0.26396
P03346	P45984	0.3645
P03346	Q00536	0.12605
P03346	Q8IW50	0.0077221
P03346	Q93009	0.43093

P03346	Q8WUM0	0.72663
P03346	Q9H4X1	0.38122
P03346	Q3LI62	0.36736
P03346	Q9BZL1	0.22802
P03346	P50219	0.35523
P03346	Q5TID7	0.48532
P03346	Q8TF39	0.34285
P03346	Q8ND56	0.15872
P03346	Q9Y6K1	0.48829
P03346	Q9P0M2	0.28742
P03346	Q86VQ0	0.62992
P03346	Q96NL6	0.1536
P03346	P09086	0.64479
P03346	Q02078	0.51952
P03346	Q6L8G5	0.40421
P03346	O00629	0.79747
P03346	Q13542	0.52615
P03346	O60239	0.57378
P03346	Q96EL2	0.53303
P03346	O95159	0.22769
P03346	Q9GZR1	0.61944
P03346	Q96KR7	0.19728
P03346	Q9BRX5	0.27113
P03346	Q02386	0.18062
P03346	P38935	0.52354
P03346	P35321	0.6687
P03346	Q8IVM0	0.8379
P03346	Q7L9B9	0.12849
P03346	Q96B97	0.54128
P03346	Q16666	0.71647
P03346	Q9BYD1	1.0411
P03346	Q9NQX1	0.70756
P03346	Q8N573	0.27564
P03346	Q96A73	0.071131
P03346	P16083	0.19498
P03346	P52954	0.29339
P03346	Q86UW9	0.96795
P03346	Q8NC51	0.96509
P03346	Q96ES7	0.43902
P03346	Q2NL98	0.20041
P03346	Q14739	0.46788
P03346	Q09161	0.89647
P03346	P31040	0.6884
P03346	Q8WXA3	0.39353
P03346	P10827	0.29735
P03346	Q9P2G1	0.4708
P03346	Q9UMS5	0.63182
P03346	Q8N4W9	0.31436
P03346	P22061	0.08464
P03346	Q9H9P5	0.2879
P03346	Q9HB65	0.77121
P03346	E9PGG2	0.66978
P03346	Q969U7	0.87323
P03346	P35813	0.34007
P03346	P49910	0.83464
P03346	P0CJ88	0.6797
P03346	P11473	0.47939
P03346	Q86U70	0.34185
P03346	Q1A5X6	0.0077221
P03346	P63173	0.56339
P03346	O15524	0.31897
P03346	Q3MJ62	0.34285
P03346	Q86UU5	0.090554
P03346	P31268	0.58644
P03346	Q5JNZ5	0.26013
P03346	Q9Y281	0.77626
P03346	P56181	0.44494
P03346	P25789	0.60446
P03346	Q9NQC8	0.27766
P03346	P28715	0.37917
P03346	P55036	0.56941
P03346	Q96N21	0.32091
P03346	Q86UR1	0.35153
P03346	Q7Z5Q5	0.28643
P03346	Q8WXF1	1.1933
P03346	P50570	0.37221

P03346	Q94892	0.31436
P03346	P02549	0.25731
P03346	Q15788	0.38446
P03346	P28324	1.7076
P03346	P06730	0.66164
P03346	Q08AF8	0.11048
P03346	P62917	0.77029
P03346	Q13895	1.0021
P03346	Q9NSK0	0.20059
P03346	Q16082	1.1541
P03346	P23511	1.476
P03346	Q9NY74	0.65405
P03346	O15397	1.105
P03346	Q9HBD4	0.65913
P03346	Q8N653	0.40553
P03346	Q75VX8	0.12284
P03346	Q6NT89	0.39616
P03346	Q12988	0.63076
P03346	A8MS94	0.11048
P03346	Q9H115	0.20354
P03346	P45973	0.80014
P03346	A6NGD5	0.35994
P03346	Q8N9K3	0.0077221
P03346	Q9UJW0	0.70358
P03346	O14598	0.072694
P03346	O75394	0.36869
P03346	P13807	0.52153
P03346	Q00005	0.65814
P03346	O15535	0.34285
P03346	Q8IX12	1.1735
P03346	O14908	0.35749
P03346	Q8IZM8	0.31436
P03346	P07196	0.75215
P03346	Q9NP64	0.46537
P03346	Q92560	0.33481
P03346	Q6PEX3	0.36736
P03346	O76064	0.28794
P03346	P62249	0.52229
P03346	Q9NPA5	0.31436
P03346	P55198	1.0375
P03346	Q7Z3C6	0.40129
P03346	Q5JWR5	0.20151
P03346	P62913	1.036
P03346	Q9BTC0	0.38439
P03346	Q9HAU4	0.47666
P03346	Q9Y657	0.17655
P03346	B3EWG3	0.0077221
P03346	Q3SY89	0.30149
P03346	Q9UPT5	0.78834
P03346	O15111	0.5684
P03346	P86452	0.73972
P03346	Q15366	1.1483
P03346	Q9NQV7	0.09464
P03346	O95229	0.88832
P03346	Q6ZVN8	0.38048
P03346	Q99811	0.36987
P03346	A2RRD8	0.31436
P03346	Q6X4W1	0.085953
P03346	Q6NZ67	0.93117
P03346	Q6P1L6	0.31436
P03346	Q7L5A3	0.32217
P03346	Q53ET0	1.0631
P03346	Q96LR2	0.17204
P03346	P52434	0.35827
P03346	Q6QHK4	0.40854
P03346	P0CG31	0.31436
P03346	Q9HAH1	0.31436
P03346	Q15061	0.48532
P03346	P60660	0.48407
P03346	Q53GT1	0.24691
P03346	Q9HAV4	1.374
P03346	Q9BV68	0.4708
P03346	Q06787	1.1282
P03346	Q9Y283	0.48185
P03346	Q8TAY7	0.2489
P03346	Q99687	0.86901

P03346	Q8IZD9	0.47044
P03346	Q4G0A6	0.10934
P03346	P49406	0.66389
P03346	O95402	1.3271
P03346	Q08AN1	0.31436
P03346	Q9Y2Z0	0.62591
P03346	Q9P2W9	0.49831
P03346	Q9NY87	0.51434
P03346	Q7Z5Y7	0.3559
P03346	O95677	0.21762
P03346	Q96HA7	0.92502
P03346	Q9H8N7	0.44027
P03346	Q96MP8	0.4624
P03346	A6NJ78	0.052605
P03346	Q96NJ6	0.31644
P03346	P35325	0.1267
P03346	Q5XKR9	0.1098
P03346	O15027	0.35285
P03346	A6NJ88	0.037931
P03346	Q96HW7	0.88882
P03346	A2A288	0.36027
P03346	P12270	0.67176
P03346	O95625	0.4117
P03346	P12956	0.37332
P03346	Q49A33	0.31436
P03346	P78346	0.38004
P03346	Q14207	1.1312
P03346	Q9H2G9	0.68677
P03346	Q9BRR0	0.50363
P03346	Q8IUE1	0.71675
P03346	O43290	1.1428
P03346	Q9H2H8	0.61887
P03346	Q9NZH4	0.37793
P03346	Q9UHD9	1.0361
P03346	P55273	0.34327
P03346	O14576	0.5285
P03346	Q08379	0.60748
P03346	C9JN71	0.33838
P03346	Q9NVI1	0.93052
P03346	Q8WX93	0.5483
P03346	Q6PJG6	0.87916
P03346	Q99966	0.28724
P03346	Q92979	0.58902
P03346	Q6ZRF7	0.31436
P03346	Q96C55	0.31436
P03346	P24385	0.57196
P03346	Q7Z2Z1	0.87087
P03346	Q9NPJ6	1.0502
P03346	Q9UGP4	0.50346
P03346	Q7L2Z9	0.1652
P03346	P61579	0.74357
P03346	Q7Z340	0.31436
P03346	A2RU30	0.17173
P03346	Q9BPZ3	0.75006
P03346	Q01201	1.2761
P03346	Q9BRU2	0.77395
P03346	Q9P2N7	0.33151
P03346	Q5T1R4	0.46405
P03346	O14829	0.44379
P03346	Q9BU68	0.015848
P03346	Q8TBN0	0.58254
P03346	Q9H765	0.47271
P03346	Q8N7R0	0.71675
P03346	Q9P031	1.3712
P03346	Q96MR9	0.31436
P03346	Q96FQ6	0.40893
P03346	Q9P0J6	0.35035
P03346	O60907	0.9283
P03346	Q96CM4	0.3216
P03346	Q8N888	0.06799
P03346	Q8N983	0.54515
P03346	Q9HCU8	0.63576
P03346	Q9Y4P1	0.41895
P03346	Q8WV37	0.61543
P03346	Q9NZV7	0.32639
P03346	Q8WTZ3	0.49903

P03346	P62861	0.59083
P03346	Q9UD57	0.44258
P03346	Q9NSV4	0.25725
P03346	P82912	0.34123
P03346	A3KN83	0.13259
P03346	Q14315	0.47632
P03346	Q9H469	0.52327
P03346	Q6ICB0	0.15286
P03346	P50221	0.88868
P03346	P80303	0.3114
P03346	O96006	0.23908
P03346	Q06609	0.56365
P03346	P04406	0.4566
P03346	P18847	1.4119
P03346	Q08170	0.351
P03346	Q8IV13	0.32217
P03346	P50452	0.83471
P03346	O94915	0.46683
P03346	Q9NQG5	1.3274
P03346	Q96C00	0.51556
P03346	Q8N1L9	1.2652
P03346	Q13362	0.36461
P03346	O75112	0.42222
P03346	Q9NQ55	0.65145
P03346	Q9Y324	0.47318
P03346	Q96S95	0.54986
P03346	P07900	0.54452
P03346	P13489	0.72993
P03346	Q6P2D8	0.50299
P03346	Q9BV90	0.42391
P03346	Q15928	0.34297
P03346	Q8NBJ4	0.04142
P03346	Q86YD1	0.76096
P03346	Q8N163	0.95118
P03346	Q96MF7	0.56794
P03346	P30279	0.74976
P03346	Q9Y5R8	0.26633
P03346	Q15834	1.3132
P03346	P29373	0.37665
P03346	Q5XG87	0.32566
P03346	Q8N8G2	1.1121
P03346	Q13228	1.4162
P03346	P83111	0.30744
P03346	Q9NRN7	0.59542
P03346	Q8IYB3	0.69873
P03346	P05783	1.3154
P03346	P57055	0.30852
P03346	Q96HY6	0.6139
P03346	Q9NPI6	1.1149
P03346	P61582	0.32217
P03346	Q14135	0.74266
P03346	Q5T2W1	0.73663
P03346	O14813	0.59994
P03346	Q8WZ19	0.36195
P03346	Q14678	0.68265
P03346	Q9H091	0.40481
P03346	Q96EK4	1.3957
P03346	Q5HYJ3	0.3708
P03346	Q9NQW6	0.45355
P03346	Q96PU8	0.558
P03346	Q9BQE9	0.018583
P03346	Q7RTS1	0.42611
P03346	P62277	1.0516
P03346	Q02878	0.89181
P03346	Q9Y6B7	0.47607
P03346	Q9UDY2	1.0997
P03346	Q9UKY7	0.37538
P03346	P41091	0.21796
P03346	Q8IYA6	0.17073
P03346	O75427	0.77888
P03346	Q5CZC0	0.14441
P03346	Q9NWU2	0.32217
P03346	Q9BWM5	0.31436
P03346	O95149	0.63942
P03346	Q96PU5	0.35656
P03346	Q6TGC4	0.35113

62017	P03346	Q14591	0.31436
62018	P03346	Q99612	0.47502
62019	P03346	P0CG33	0.11048
62020	P03346	P29374	0.57232
62021	P03346	P68036	0.80924
62022	P03346	Q9BUY5	0.31436
62023	P03346	Q7Z3Y8	0.58681
62024	P03346	P60369	0.40421
62025	P03346	Q99873	0.7549
62026	P03346	Q9UEG4	0.31436
62027	P03346	Q7Z591	0.70946
62028	P03346	P58012	0.44874
62029	P03346	Q9BSF8	0.65405
62030	P03346	Q14258	0.96218
62031	P03346	Q9Y5Q8	0.96338
62032	P03346	Q8N0U4	0.20041
62033	P03346	Q9HB19	0.80031
62034	P03346	O95551	0.81525
62035	P03346	Q9BYU5	0.40421
62036	P03346	P0CAP2	0.19564
62037	P03346	Q14814	0.79961
62038	P03346	P78337	0.69282
62039	P03346	O96017	0.64279
62040	P03346	Q8IXH7	1.2815
62041	P03346	Q99598	0.22257
62042	P03346	P60006	0.16466
62043	P03346	P36508	0.30991
62044	P03346	Q9BXF3	0.89565
62045	P03346	Q9NQZ5	0.14441
62046	P03346	Q93062	1.0871
62047	P03346	Q96S94	0.70286
62048	P03346	Q9Y2P0	0.31436
62049	P03346	A6NN14	0.31436
62050	P03346	O95397	0.17705
62051	P03346	Q6ZRQ5	0.8272
62052	P03346	Q9NX70	1.3404
62053	P03346	Q9H9R9	0.20041
62054	P03346	Q8NCA9	0.31436
62055	P03346	Q8WY54	0.87888
62056	P03346	O75446	1.1355
62057	P03346	Q86XR8	1.0762
62058	P03346	Q8IXM2	0.35777
62059	P03346	Q9P032	0.74971
62060	P03346	P14373	0.43268
62061	P03346	Q14511	0.80823
62062	P03346	O15522	0.32126
62063	P03346	Q8WYB5	0.31511
62064	P03346	Q96SD1	0.23342
62065	P03346	Q9NS69	0.33771
62066	P03346	Q52M93	0.33838
62067	P03346	Q9H116	0.5775
62068	P03346	Q86UT8	0.19349
62069	P03346	Q9UP83	1.4519
62070	P03346	Q7RTS3	0.28657
62071	P03346	Q5HYW3	0.06162
62072	P03346	Q8TF40	0.81274
62073	P03346	Q12905	1.3597
62074	P03346	O00471	0.51716
62075	P03346	Q96LY2	0.035326
62076	P03346	P49247	0.10717
62077	P03346	P25685	0.55324
62078	P03346	Q9Y3C0	0.6156
62079	P03346	Q32MQ0	0.33966
62080	P03346	Q9UBK8	0.3176
62081	P03346	Q8NHY5	0.043795
62082	P03346	Q01538	0.20768
62083	P03346	Q9HCS5	0.30298
62084	P03346	Q12769	1.035
62085	P03346	Q14671	0.43352
62086	P03346	Q8N8E3	0.35785
62087	P03346	Q86WZ0	0.024832
62088	P03346	Q9BWT6	0.34355
62089	P03346	Q8NAF0	0.31436
62090	P03346	Q8WV24	0.8543
62091	P03346	Q16626	0.3776
62092	P03346	Q9BR77	0.17073

P03346	Q3KNW1	0.29876
P03346	A6NFD8	0.52759
P03346	Q8IVW6	0.45236
P03346	Q15916	0.31436
P03346	Q9P2K5	0.21518
P03346	Q9ULD5	0.31644
P03346	P47813	0.22577
P03346	Q96KR1	0.50963
P03346	Q8N2Y8	0.33623
P03346	P08779	0.99925
P03346	Q96A65	0.34809
P03346	Q9BUX1	0.90682
P03346	P46100	0.63978
P03346	Q8N9B5	0.91437
P03346	Q6PCD5	0.24588
P03346	Q9GZR2	0.4593
P03346	Q5JTW2	0.54312
P03346	Q96GM5	0.91711
P03346	Q9UPW6	0.95034
P03346	A6NJ46	0.6797
P03346	Q9Y4C2	0.04631
P03346	Q8N4Q1	0.28165
P03346	Q9H2T7	0.65396
P03346	Q9H0A9	0.18187
P03346	Q9C005	0.85182
P03346	Q96RT1	0.43573
P03346	Q659A1	0.32217
P03346	O00567	1.0379
P03346	Q8TAB7	0.10854
P03346	P16220	0.83731
P03346	Q3LI64	0.49573
P03346	O15392	0.22634
P03346	O60765	0.34402
P03346	Q86VS8	0.51929
P03346	Q9BV97	0.18405
P03346	P82933	0.82079
P03346	O43684	0.76034
P03346	P98171	0.4059
P03346	Q96NS5	0.4168
P03346	Q68DX3	0.66523
P03346	Q9BYR2	0.40421
P03346	Q8IZC7	0.31436
P03346	Q5TAP6	0.072987
P03346	Q96JP2	0.31558
P03346	Q9UKD2	0.38434
P03346	P09651	0.41588
P03346	Q6P2S7	0.20041
P03346	Q9UQR1	0.90998
P03346	O75150	0.54455
P03346	O43447	0.90052
P03346	O95684	0.93666
P03346	Q9H706	0.66787
P03346	Q08999	1.5259
P03346	P56270	0.79233
P03346	Q96EL3	0.37322
P03346	H0YKK7	0.015632
P03346	P17039	0.31436
P03346	Q8TEA8	0.29687
P03346	P08727	0.70581
P03346	Q2QGD7	0.78596
P03346	Q99633	0.42359
P03346	O60888	0.71012
P03346	Q12840	0.34571
P03346	P61581	0.32217
P03346	O75362	0.89268
P03346	Q9UKL0	1.3912
P03346	O00479	0.28899
P03346	Q5T8I9	0.34901
P03346	Q99460	0.24711
P03346	Q8IX06	0.19743
P03346	B2RD01	0.29934
P03346	Q9BQG0	0.85473
P03346	Q6P161	0.37322
P03346	Q92734	0.47639
P03346	Q9NX02	0.27397
P03346	Q9Y3C6	0.1282

P03346	Q96BP2	1.3827
P03346	Q9Y2P7	0.65117
P03346	Q9UM11	1.1515
P03346	Q9H3M9	0.099429
P03346	P59103	0.174
P03346	Q96BU1	0.21289
P03346	Q8N6M9	0.32295
P03346	P23610	0.34434
P03346	Q8N8B7	0.34797
P03346	Q9UH77	0.82842
P03346	Q8NAP8	0.27653
P03346	A7MCY6	0.64944
P03346	Q86UE4	0.5164
P03346	Q96MT4	0.028327
P03346	Q9H2W2	0.31302
P03346	O14639	0.64739
P03346	Q86US8	0.66704
P03346	Q96PV6	0.66787
P03346	O60248	0.87002
P03346	Q96EE3	0.5754
P03346	Q5TA76	0.02884
P03346	Q5HYK9	0.31436
P03346	Q8N3U4	0.80821
P03346	P06401	0.30356
P03346	Q5T0L3	0.063008
P03346	Q9BYG8	0.34138
P03346	Q9UL59	0.31436
P03346	Q8IW19	0.53882
P03346	O43918	1.0302
P03346	Q3SYF9	0.36736
P03346	Q96HQ2	0.074789
P03346	O60287	0.50071
P03346	Q9H0M5	0.31436
P03346	P43355	1.3154
P03346	Q96D03	0.18705
P03346	Q96FF9	0.92293
P03346	Q96BA8	0.38626
P03346	P17676	0.90299
P03346	Q7RTV0	0.82488
P03346	P55265	0.52873
P03346	Q96P71	0.7022
P03346	O60732	0.66787
P03346	Q9H2Z4	0.44791
P03346	Q03052	0.62115
P03346	Q9UIV1	1.2819
P03346	O43167	0.85529
P03346	P78414	0.66978
P03346	Q16384	0.4707
P03346	Q9BSU3	0.67596
P03346	P06748	0.46184
P03346	Q99567	0.84082
P03346	Q96N16	0.38993
P03346	Q99543	0.76865
P03346	Q9Y4X4	0.44999
P03346	P19484	0.83584
P03346	Q15323	0.4917
P03346	P17098	0.34464
P03346	Q9UBQ5	1.1695
P03346	Q13616	0.68917
P03346	P29474	0.49774
P03346	Q86V71	0.31436
P03346	O15370	0.70372
P03346	Q52LR7	0.67697
P03346	O14901	0.37359
P03346	Q9UNL4	0.49684
P03346	Q9H2H0	0.31327
P03346	Q8IZT9	0.32217
P03346	P0C7T5	0.43488
P03346	Q8N1N5	0.70085
P03346	Q8NC26	0.31436
P03346	P12757	0.51782
P03346	Q96C86	0.93398
P03346	Q8NFV4	0.23421
P03346	Q96NJ3	0.31436
P03346	Q96JB2	0.97756
P03346	O75190	0.9094

P03346	P36402	0.711
P03346	Q59GN2	0.26013
P03346	Q9ULW3	1.1111
P03346	O75175	1.5435
P03346	Q9NWF9	0.83187
P03346	O75586	0.94731
P03346	P62633	0.73529
P03346	Q96M11	0.62166
P03346	Q8IZW8	0.62882
P03346	Q13489	0.26936
P03346	Q15036	0.35362
P03346	P14866	0.82995
P03346	P05413	0.48904
P03346	Q9H5P4	0.73341
P03346	Q9ULK2	0.021938
P03346	P15336	0.76253
P03346	O14633	0.090938
P03346	Q9UIF8	1.0526
P03346	Q9GZV4	0.81623
P03346	Q14764	1.3615
P03346	O15511	0.46897
P03346	Q70EK8	0.11093
P03346	Q9BTK6	0.78688
P03346	Q5VWP3	0.46875
P03346	Q8ND07	0.02348
P03346	Q693B1	0.25645
P03346	A2RUB1	0.0077221
P03346	Q9BS16	0.30284
P03346	Q92800	0.71272
P03346	Q5MJ70	0.24687
P03346	Q99607	1.4279
P03346	P55327	0.54942
P03346	Q9BTL3	0.86184
P03346	Q5JR59	0.83673
P03346	Q86Y97	0.66005
P03346	Q9BZE4	0.47105
P03346	P63010	0.49483
P03346	Q2M2Z5	0.89893
P03346	Q4VC05	0.1373
P03346	P23526	0.27712
P03346	Q99569	0.48418
P03346	O60716	0.61418
P03346	Q9BSD3	0.49501
P03346	Q9NRX1	0.4493
P03346	Q9UL46	0.31172
P03346	Q9BQY4	1.2815
P03346	Q9P0W2	0.98902
P03346	P54098	0.62685
P03346	Q96HQ0	0.31436
P03346	O14787	1.2325
P03346	Q96G27	0.13083
P03346	Q05925	0.28165
P03346	O43639	0.52501
P03346	Q9NU63	0.11664
P03346	Q8IXQ9	0.19045
P03346	O75531	1.055
P03346	Q86WV5	0.55886
P03346	Q9BRA2	0.76653
P03346	O14737	0.51019
P03346	A5D8V6	0.44579
P03346	Q9H9L3	0.66406
P03346	Q5VWM5	0.13291
P03346	Q13129	0.90427
P03346	Q9Y2V2	0.56604
P03346	Q1KMD3	0.31389
P03346	Q8TCY9	0.38134
P03346	Q9Y6R7	0.25935
P03346	P78415	0.43683
P03346	Q13515	0.28247
P03346	Q9BSC4	0.48532
P03346	Q9BYG7	0.48532
P03346	Q9UFM2	0.32217
P03346	Q9H8W5	0.26638
P03346	Q2VPB7	0.82273
P03346	Q2M218	0.31436
P03346	Q8NFH8	0.70824

P03346	P62280	0.60402
P03346	P39023	0.97199
P03346	P36956	0.34264
P03346	A8MX34	0.36736
P03346	Q8NHU6	0.39484
P03346	P52739	0.30845
P03346	Q7Z5K2	0.79964
P03346	P23258	0.42942
P03346	O15209	0.31436
P03346	Q05066	0.84269
P03346	Q9UGR2	0.58215
P03346	P0CJ78	0.31436
P03346	Q5T9G4	0.11273
P03346	Q16836	0.47466
P03346	Q8N187	0.93502
P03346	Q3B8N5	0.45838
P03346	Q13449	0.23273
P03346	Q6ZP68	0.10317
P03346	Q8N141	0.31436
P03346	Q7Z4H9	0.32217
P03346	P35527	0.4258
P03346	Q86SG2	0.48449
P03346	Q96DN6	0.40606
P03346	Q9H9A5	0.27623
P03346	Q8WYH8	0.66329
P03346	Q8TAE8	1.1514
P03346	P57772	0.02108
P03346	Q5VTR2	0.70801
P03346	Q8WYR1	0.37743
P03346	O60688	0.28191
P03346	Q7Z3V5	0.31436
P03346	Q9H2P0	0.30078
P03346	A6NNW6	0.13007
P03346	Q8IU81	0.52293
P03346	P14625	0.20272
P03346	Q03828	0.53574
P03346	Q6P1Q0	0.19489
P03346	Q9Y3M2	0.53651
P03346	P50502	0.8208
P03346	Q29RF7	1.1167
P03346	Q9P253	0.34863
P03346	Q107X0	0.49807
P03346	Q16656	0.67954
P03346	Q99871	0.58118
P03346	Q9C0F0	0.6213
P03346	Q8IZQ1	0.80442
P03346	Q00613	1.0835
P03346	Q9P016	0.32217
P03346	O15144	0.4638
P03346	Q6ZT77	0.31436
P03346	Q9UL58	0.34285
P03346	H3BTG2	0.053998
P03346	Q9BQ61	0.0077221
P03346	Q8NCN2	0.4412
P03346	Q96F07	0.73014
P03346	Q96DC7	0.11078
P03346	P35638	0.90684
P03346	Q9BQA1	1.1125
P03346	Q92622	0.2745
P03346	Q9UKS6	0.45921
P03346	P35548	0.67359
P03346	O75489	0.27897
P03346	Q02535	0.25594
P03346	Q96RT8	0.49672
P03346	O43516	0.47715
P03346	Q9NZL4	0.58319
P03346	Q96LC9	0.71437
P03346	O75901	0.53338
P03346	Q9BV20	0.25587
P03346	Q8N823	0.31436
P03346	Q5TA79	0.02884
P03346	Q8WW38	0.58882
P03346	Q6ZQV5	0.31436
P03346	Q9H6Q3	0.39484
P03346	Q9H4E7	0.30962
P03346	Q9Y613	0.81756

P03346	P07148	0.28329
P03346	P52747	0.91894
P03346	Q7Z6J0	0.45115
P03346	O94888	0.68635
P03346	Q9H633	0.47406
P03346	O43598	0.36276
P03346	Q9BYU1	0.62328
P03346	P23508	0.61141
P03346	Q6AI39	0.014834
P03346	O00167	0.74077
P03346	Q9Y2L8	0.34285
P03346	Q96DB2	0.37504
P03346	P07320	0.74096
P03346	P08235	0.5016
P03346	Q8TAK5	0.75531
P03346	Q8IVI9	0.43719
P03346	Q06547	1.3202
P03346	Q93015	0.24025
P03346	P62304	0.96834
P03346	O95155	0.37573
P03346	Q9HD90	0.34553
P03346	Q9BYR0	0.40421
P03346	Q7Z4H7	0.91493
P03346	Q14159	0.097226
P03346	Q9H305	0.33838
P03346	O75530	0.84893
P03346	P11831	0.54448
P03346	Q6ZW61	0.36636
P03346	Q8N5T8	0.0096925
P03346	Q8TAV0	0.10094
P03346	P11388	0.27006
P03346	P31270	0.29828
P03346	A2AJT9	0.14441
P03346	O95171	0.31428
P03346	P83876	1.161
P03346	Q15329	1.4551
P03346	Q9UHR5	1.4582
P03346	Q3KQV3	0.31436
P03346	Q9NQE9	0.53016
P03346	O95295	0.51879
P03346	O43296	0.31436
P03346	O43502	0.46204
P03346	Q9UKX7	1.1367
P03346	Q9Y5Z7	0.61242
P03346	P57060	0.12821
P03346	P52758	0.65761
P03346	O95685	0.13234
P03346	Q96A44	0.80377
P03346	Q9BPU9	0.35918
P03346	Q9H3J6	0.23222
P03346	Q9NV56	0.59651
P03346	Q0VD86	0.65405
P03346	Q8TAQ5	0.31436
P03346	P11277	0.36475
P03346	Q9BS18	0.64128
P03346	O95376	0.51259
P03346	P20073	0.54575
P03346	O43379	0.4243
P03346	Q9UKF6	0.85617
P03346	Q93008	0.52875
P03346	Q6PEW1	0.2862
P03346	Q9BXA9	0.2629
P03346	Q86TJ5	0.31436
P03346	Q0VG99	0.56934
P03346	Q99627	0.64501
P03346	O95751	0.89531
P03346	Q32P41	0.5669
P03346	P28347	1.2907
P03346	Q9Y5W3	0.27234
P03346	O95777	0.99127
P03346	Q15554	0.60191
P03346	Q8IY31	0.69715
P03346	P13929	0.23686
P03346	P84101	0.47084
P03346	Q9H7E2	0.69328
P03346	Q8N612	0.65183

62473	P03346	O00541	0.54749
62474	P03346	Q86VX2	0.67432
62475	P03346	Q9UJT9	0.49978
62476	P03346	O00458	0.0048076
62477	P03346	O43747	0.73375
62478	P03346	O60930	0.42005
62479	P03346	Q3ZLR7	0.061177
62480	P03346	P42768	0.50419
62481	P03346	Q96QC0	0.12543
62482	P03346	Q9UQ80	1.3364
62483	P03346	Q9ULJ1	0.17073
62484	P03346	Q8IUE0	0.71675
62485	P03346	Q9Y3D7	0.18731
62486	P03346	P62987	0.26228
62487	P03346	Q96K75	0.31436
62488	P03346	O43309	0.34285
62489	P03346	O15379	0.61709
62490	P03346	Q6ZUV0	0.16914
62491	P03346	P61927	0.62242
62492	P03346	Q8NFV5	0.040024
62493	P03346	Q9P2H0	0.66787
62494	P03346	A6NJR5	0.040024
62495	P03346	Q5FWF6	0.31436
62496	P03346	Q9ULM2	0.31436
62497	P03346	Q460N3	0.58912
62498	P03346	P27694	0.5525
62499	P03346	Q5MIZ7	0.65084
62500	P03346	Q8TC71	0.60623
62501	P03346	Q9NP62	0.24492
62502	P03346	Q6ZSB9	0.31436
62503	P03346	Q96DZ1	0.52394
62504	P03346	Q9UNX3	0.59833
62505	P03346	Q96CS3	0.76262
62506	P03346	P20700	0.59061
62507	P03346	Q7Z3I7	0.31436
62508	P03346	P02489	0.41799
62509	P03346	Q9UKT9	0.61775
62510	P03346	Q14011	0.46555
62511	P03346	Q96KQ4	1.0991
62512	P03346	P28072	0.42723
62513	P03346	Q9H503	0.63188
62514	P03346	Q8IY42	0.18359
62515	P03346	P25815	0.78586
62516	P03346	Q6ZUT4	0.036288
62517	P03346	O95900	0.058374
62518	P03346	Q9P0T4	0.31436
62519	P03346	Q12857	0.62522
62520	P03346	Q99594	1.3764
62521	P03346	Q06732	0.30845
62522	P03346	A6NCW3	0.051589
62523	P03346	Q9BUP3	0.3855
62524	P03346	Q13158	0.51695
62525	P03346	Q92674	0.58969
62526	P03346	Q96H40	0.31436
62527	P03346	Q9NQR1	0.82009
62528	P03346	Q8NFD5	1.1291
62529	P03346	Q9UBX3	0.47383
62530	P03346	Q9BRP1	0.28097
62531	P03346	Q9NTK1	0.14441
62532	P03346	Q9BX26	0.36633
62533	P03346	Q99471	1.1105
62534	P03346	Q7Z5D8	0.66978
62535	P03346	Q9Y5K8	0.29845
62536	P03346	Q71SY5	1.2331
62537	P03346	Q8N3X6	0.74923
62538	P03346	O75694	0.6949
62539	P03346	Q92990	0.27399
62540	P03346	O95714	0.25585
62541	P03346	P21127	0.38747
62542	P03346	Q8N668	0.9041
62543	P03346	Q8NFW5	0.28171
62544	P03346	Q9UJ04	0.22545
62545	P03346	Q9UIL1	0.89622
62546	P03346	P49427	0.55213
62547	P03346	P23297	0.69686
62548	P03346	P48443	0.29353

P03346	Q8IXW5	0.72837
P03346	P06400	0.6333
P03346	P0CJ87	0.6797
P03346	Q8N7M2	0.31436
P03346	Q9H3K2	0.31831
P03346	O75123	0.31436
P03346	P48378	0.70946
P03346	Q9Y3C5	0.60132
P03346	Q4G112	0.71675
P03346	P25963	0.63309
P03346	Q96IQ9	0.27653
P03346	Q8N365	0.0077221
P03346	O75717	0.72588
P03346	Q14590	0.31436
P03346	Q9UKZ1	0.29996
P03346	Q7Z465	0.94068
P03346	O75969	0.38966
P03346	Q9NR28	0.97883
P03346	Q9HD42	1.0545
P03346	P0CH99	0.31436
P03346	A6NLC8	0.14736
P03346	P35579	0.59517
P03346	P61956	0.96086
P03346	P40426	0.60625
P03346	Q9ULP0	0.18381
P03346	Q14774	0.24495
P03346	Q9UHP9	0.35139
P03346	P0CB47	0.24944
P03346	Q96I25	0.62422
P03346	Q9Y6M1	0.5981
P03346	Q9UHV9	1.0346
P03346	P78352	0.59603
P03346	Q96K62	0.31436
P03346	Q9H788	1.1922
P03346	P62857	0.54082
P03346	Q92572	0.66302
P03346	Q9NW07	0.27529
P03346	O75143	0.7576
P03346	Q96MW5	0.24239
P03346	O00425	0.66259
P03346	P25788	0.60446
P03346	Q8WTT2	0.48728
P03346	Q6V9R5	0.31436
P03346	P35789	0.30845
P03346	Q96Q35	0.25662
P03346	Q9GZW5	0.71564
P03346	Q8N6F7	0.59604
P03346	O75312	0.28033
P03346	Q9Y5N6	0.8517
P03346	Q8WU90	0.953
P03346	Q9UM82	0.095474
P03346	Q12815	0.64315
P03346	Q8WYJ6	0.40868
P03346	O94875	0.25457
P03346	P62736	0.10464
P03346	O95336	0.40684
P03346	Q8NEF9	0.94205
P03346	Q8NEZ5	0.83047
P03346	Q9BVC4	0.61635
P03346	P52740	0.30845
P03346	O14656	0.34574
P03346	Q14691	0.49738
P03346	Q9HBH7	0.40297
P03346	P35326	0.051514
P03346	Q99784	0.27912
P03346	Q9NR11	0.31436
P03346	Q9BSH3	0.31628
P03346	Q9NRR3	0.67693
P03346	Q9Y5U2	0.0004879
P03346	P60520	0.47776
P03346	Q9BYM8	0.26426
P03346	A8MT69	0.67014
P03346	Q8TD23	0.68219
P03346	Q9H171	0.48683
P03346	Q2TAL8	0.12821
P03346	Q15291	0.35197

62625	P03346	Q9BYT5	0.40421
62626	P03346	Q9H0U6	0.28895
62627	P03346	O95260	0.36922
62628	P03346	Q08AG5	0.31436
62629	P03346	Q9GZP8	0.23885
62630	P03346	P51532	0.49528
62631	P03346	Q9Y2D8	0.90008
62632	P03346	Q9BYQ8	0.40421
62633	P03346	Q14149	0.22619
62634	P03346	P13349	0.54017
62635	P03346	Q99728	0.38016
62636	P03346	Q9UJ72	0.11346
62637	P03346	Q96IL0	0.16708
62638	P03346	P53680	0.24199
62639	P03346	Q53FD0	0.072131
62640	P03346	Q9C0E2	1.1931
62641	P03346	Q8TCC3	0.37322
62642	P03346	Q5SQN1	0.63881
62643	P03346	Q9P2N5	0.17397
62644	P03346	O43711	0.70304
62645	P03346	Q9H147	0.32217
62646	P03346	Q6TFL4	0.16082
62647	P03346	Q8N7J2	0.16832
62648	P03346	Q00577	0.72177
62649	P03346	Q8WWC4	0.14441
62650	P03346	Q96M53	0.20758
62651	P03346	P49754	0.57176
62652	P03346	Q8IZP0	0.40502
62653	P03346	P35226	0.59869
62654	P03346	P54845	0.63031
62655	P03346	Q14CX7	0.20041
62656	P03346	Q969W8	0.31436
62657	P03346	Q9NZM5	0.25053
62658	P03346	P28074	0.50625
62659	P03346	Q9UMR2	0.30472
62660	P03346	Q56P03	0.55781
62661	P03346	Q13148	0.83133
62662	P03346	Q92614	0.33814
62663	P03346	Q15797	0.31096
62664	P03346	Q70SY1	0.22489
62665	P03346	Q86XK3	0.93048
62666	P03346	Q9Y3C1	0.44008
62667	P03346	Q14188	0.9282
62668	P03346	Q9GZV5	1.1069
62669	P03346	O60393	0.53731
62670	P03346	P27469	0.85174
62671	P03346	P50549	1.2238
62672	P03346	Q00059	1.0768
62673	P03346	Q969X6	1.4533
62674	P03346	Q7LBC6	0.35116
62675	P03346	P49721	0.39017
62676	P03346	Q6ZTU2	0.14315
62677	P03346	Q9BX40	0.07292
62678	P03346	Q7Z3Z0	0.56472
62679	P03346	Q12772	0.38786
62680	P03346	Q96SZ4	0.3828
62681	P03346	O95391	0.38013
62682	P03346	A6NHQ2	0.47653
62683	P03346	Q2NKX9	0.0077221
62684	P03346	Q96EZ8	0.97144
62685	P03346	Q8N1B3	0.92701
62686	P03346	Q96MU6	0.31436
62687	P03346	Q9BY42	0.12821
62688	P03346	Q8NFZ5	1.03
62689	P03346	Q9BZE3	0.75924
62690	P03346	Q92841	0.76902
62691	P03346	Q9UBI9	0.084219
62692	P03346	Q8TB05	0.0077221
62693	P03346	Q9C086	1.3348
62694	P03346	O60315	0.42363
62695	P03346	Q9Y2J8	0.089801
62696	P03346	Q13541	0.94031
62697	P03346	Q9UMS6	0.8913
62698	P03346	O75410	0.90173
62699	P03346	Q15154	0.67625
62700	P03346	O60563	1.4423

P03346	Q9NRG9	0.84905
P03346	Q4L180	0.50297
P03346	Q15653	1.0848
P03346	O43734	0.36847
P03346	P0C7Q3	0.3447
P03346	Q8WXF8	1.0874
P03346	Q3LI66	0.36736
P03346	Q9BWX5	0.52607
P03346	P48382	1.3272
P03346	Q03933	0.96667
P03346	O60290	0.44737
P03346	Q9Y4Y9	1.0498
P03346	O60238	0.34015
P03346	Q9H7B2	0.48532
P03346	Q6P1K8	0.58027
P03346	O75935	0.83661
P03346	Q96R06	0.94529
P03346	P17027	0.31436
P03346	Q9Y3Q8	1.4983
P03346	Q9UHB4	0.81821
P03346	Q5MCW4	0.31436
P03346	Q96AQ6	0.76887
P03346	Q01105	0.9195
P03346	Q5TCQ9	0.67473
P03346	Q8IXM3	0.6134
P03346	Q13347	0.91246
P03346	Q9H1A7	0.34613
P03346	Q5T280	0.12821
P03346	Q9UNA4	0.58081
P03346	Q9NZL9	0.3273
P03346	O15225	0.12821
P03346	Q96GV9	0.72835
P03346	Q9BV57	0.63506
P03346	Q92543	0.36184
P03346	Q96HZ4	0.52404
P03346	O00139	0.38589
P03346	P51786	0.33736
P03346	Q13884	0.70507
P03346	Q15038	1.174
P03346	Q15013	1.072
P03346	Q9UKI9	1.506
P03346	Q9H467	1.2782
P03346	O60268	0.20041
P03346	Q9UDY8	0.35986
P03346	Q4G0X4	0.3559
P03346	P51808	0.4321
P03346	Q99626	0.2616
P03346	P08579	0.62522
P03346	O94763	0.9523
P03346	Q8N137	0.99816
P03346	P07992	0.22388
P03346	Q9BRV8	0.81344
P03346	P07814	0.25862
P03346	Q6ZU65	0.48532
P03346	Q14247	0.38215
P03346	O60841	0.33694
P03346	O75400	1.0982
P03346	P78406	0.59153
P03346	Q12837	0.76453
P03346	Q8NHY2	1.0862
P03346	Q03936	0.43781
P03346	Q9UFN0	0.96272
P03346	Q16559	0.33959
P03346	Q9H9G7	0.9068
P03346	Q9H9F9	1.3617
P03346	Q96JG9	0.31436
P03346	Q5T7W7	0.12821
P03346	O60308	0.27763
P03346	Q00056	0.46888
P03346	Q56P42	0.49807
P03346	Q69YH5	0.93524
P03346	P26678	0.12727
P03346	Q15911	0.38173
P03346	O75159	0.25263
P03346	Q9BPX1	0.40948
P03346	P57740	0.81471

P03346	Q15691	0.69333
P03346	P55795	0.41851
P03346	P56182	0.39855
P03346	Q2M1K9	0.51803
P03346	P17275	0.9366
P03346	Q5XKE5	0.51027
P03346	P78347	0.8781
P03346	Q9BPZ7	0.22315
P03346	Q9BUK6	0.22289
P03346	Q32MZ4	1.4131
P03346	Q96JI7	0.82463
P03346	O96020	1.1053
P03346	Q86T29	0.31436
P03346	A8MUV8	0.31436
P03346	P82094	1.6111
P03346	Q8NEP4	0.0077221
P03346	Q8IUD6	0.51477
P03346	O95628	0.7576
P03346	Q99733	1.019
P03346	Q147U1	0.31436
P03346	Q5EE01	0.75503
P03346	Q6ZWB6	0.18903
P03346	P0CG34	0.18695
P03346	Q13322	0.24418
P03346	Q6UX04	0.31049
P03346	Q9UBD5	0.8176
P03346	P02100	0.079165
P03346	Q8TD17	0.30647
P03346	P54274	0.53047
P03346	P61578	0.74357
P03346	Q9UBV8	0.44472
P03346	Q6ZNK6	0.11316
P03346	Q14587	0.30845
P03346	Q9Y5N5	0.57383
P03346	Q9P2D0	0.44053
P03346	P52738	0.30845
P03346	Q70IA6	0.59206
P03346	Q9NQV6	0.31436
P03346	Q8TF45	0.31436
P03346	P49116	0.65381
P03346	Q9ULZ2	0.94204
P03346	P0CB33	0.31436
P03346	Q8IWV1	0.094509
P03346	Q15466	0.73748
P03346	Q9UJQ4	0.2964
P03346	P42575	0.42735
P03346	Q96LM6	0.32217
P03346	Q96M61	0.66787
P03346	Q8N680	0.31436
P03346	Q9H4A6	0.539
P03346	Q99689	0.29649
P03346	Q6XYB7	0.71675
P03346	P24863	1.2736
P03346	Q8TF42	0.25957
P03346	O43678	0.042351
P03346	Q9NZ09	0.20041
P03346	P35244	0.6789
P03346	Q9NZD4	0.13291
P03346	Q7Z7C8	0.72567
P03346	Q9NZI6	0.74458
P03346	P49257	0.1441
P03346	Q9UIU6	0.32493
P03346	Q8WXH0	0.2744
P03346	O95178	0.076823
P03346	P56377	0.46913
P03346	Q99962	0.41567
P03346	P14316	1.1903
P03346	Q9Y5M1	0.14265
P03346	Q4G0J3	0.27663
P03346	Q15004	1.0139
P03346	Q9UNW9	0.29353
P03346	Q9NS39	0.35297
P03346	O14818	0.57414
P03346	Q8N699	0.32217
P03346	Q9UL18	1.1618
P03346	P43487	0.41721

P03346	P05423	0.23946
P03346	Q9UK59	0.19758
P03346	P51580	0.22531
P03346	Q9Y3Y4	0.75202
P03346	A8MVS1	0.31436
P03346	P24278	0.99545
P03346	Q96BZ8	0.12821
P03346	Q8TBK2	0.77445
P03346	Q9P2W1	0.30977
P03346	Q6WBX8	0.69682
P03346	P43243	0.36115
P03346	P49336	0.51587
P03346	Q6P4D5	0.030447
P03346	Q15007	0.68744
P03346	Q8WYN3	0.84255
P03346	Q7Z6J9	0.47419
P03346	P17861	0.88041
P03346	Q8WV99	0.4159
P03346	Q8WYP5	0.84454
P03346	P46778	0.66528
P03346	Q8WVV9	0.50036
P03346	Q96KM6	0.42081
P03346	Q9BRT9	0.54885
P03346	Q8NEP9	0.31436
P03346	A8MVW5	0.84596
P03346	O15360	1.0037
P03346	P23769	0.40313
P03346	Q08AD1	0.7583
P03346	Q96QU8	1.2014
P03346	Q14872	0.88058
P03346	Q5TBB1	0.44207
P03346	Q9UBZ9	0.67138
P03346	Q99708	0.81332
P03346	Q8TC84	0.65405
P03346	Q3LI67	0.36736
P03346	O43159	0.96934
P03346	Q8NE65	0.45059
P03346	Q9NSI6	1.0217
P03346	Q96LT7	0.56579
P03346	Q9BYQ2	0.40421
P03346	P49207	0.66528
P03346	Q96SI1	0.3559
P03346	Q5VIY5	0.31436
P03346	Q52LG2	0.36736
P03346	Q5BKY1	0.35029
P03346	Q9HD15	0.84135
P03346	Q9BSJ6	0.81053
P03346	Q14527	0.41511
P03346	Q53GL7	0.68363
P03346	Q8WV07	0.12821
P03346	P35250	0.24636
P03346	P61604	0.89852
P03346	Q99457	0.26935
P03346	Q9ULG1	0.3561
P03346	P62258	0.86843
P03346	Q8IYL2	0.11829
P03346	P43360	0.7115
P03346	Q9BW85	0.15973
P03346	Q96HR3	0.9704
P03346	Q8N5X7	0.32031
P03346	Q8N693	0.75837
P03346	Q8WW12	0.93479
P03346	P25490	0.38428
P03346	P0CG12	0.32217
P03346	P0C7M4	0.66978
P03346	Q8NHV4	0.827
P03346	Q9NRR5	0.87944
P03346	O43615	0.38522
P03346	Q8N336	0.22247
P03346	Q15583	0.5099
P03346	Q6P1X6	0.14441
P03346	Q9NQP4	1.1073
P03346	O95848	0.65552
P03346	Q99436	0.61155
P03346	Q8N5U6	1.2104
P03346	Q6ZS17	0.20041

62929	P03346	O15304	0.93239
62930	P03346	P19419	1.4977
62931	P03346	O95983	1.5008
62932	P03346	Q5VYV7	0.66787
62933	P03346	P17152	0.11747
62934	P03346	Q9H4S2	0.58682
62935	P03346	Q9NZU7	0.48041
62936	P03346	Q5VZE5	0.48194
62937	P03346	Q7L266	0.46489
62938	P03346	Q9UBF1	1.0697
62939	P03346	Q96A56	1.1279
62940	P03346	Q5H9K5	0.12127
62941	P03346	Q9UMX2	0.40841
62942	P03346	P80723	0.43277
62943	P03346	P24941	0.542
62944	P03346	Q15637	0.52708
62945	P03346	Q96FS4	0.26014
62946	P03346	Q9NS18	0.29487
62947	P03346	Q9NSB4	0.4983
62948	P03346	Q8IV36	0.70802
62949	P03346	Q9NY27	1.0556
62950	P03346	O14978	0.53898
62951	P03346	Q68CZ1	0.23235
62952	P03346	Q8NA19	0.57808
62953	P03346	Q13426	0.43065
62954	P03346	P55957	0.55552
62955	P03346	Q96IU4	0.63477
62956	P03346	Q15633	0.98749
62957	P03346	Q9Y483	0.29285
62958	P03346	Q9GZL7	0.88792
62959	P03346	Q5T749	0.20041
62960	P03346	O75971	0.84595
62961	P03346	Q9UIF3	0.27341
62962	P03346	Q8TBZ9	0.11896
62963	P03346	P51610	0.62549
62964	P03346	Q0PNE2	0.48755
62965	P03346	Q9UMS4	0.89313
62966	P03346	Q9NVR5	0.13727
62967	P03346	Q6P1M3	1.1397
62968	P03346	Q15629	0.19702
62969	P03346	Q49AN0	0.33723
62970	P03346	Q9BQ66	0.40421
62971	P03346	Q8IXQ5	0.54624
62972	P03346	O75505	0.66978
62973	P03346	Q15573	0.73389
62974	P03346	Q8TE76	0.41006
62975	P03346	P0DJD3	0.39713
62976	P03346	Q8NHX4	0.030538
62977	P03346	Q14789	0.16731
62978	P03346	Q9NXG0	0.2135
62979	P03346	Q9UH99	0.31937
62980	P03346	Q6P5R6	0.26013
62981	P03346	Q6PI26	1.2996
62982	P03346	P55895	0.5582
62983	P03346	P14859	1.4216
62984	P03346	Q6P280	0.31436
62985	P03346	Q969U6	0.43555
62986	P03346	Q9NSC2	0.3293
62987	P03346	Q5T0B9	0.31436
62988	P03346	O95177	0.12821
62989	P03346	O75486	0.72982
62990	P03346	P30304	0.68407
62991	P03346	Q8IZU0	0.48532
62992	P03346	Q9P0U1	0.34671
62993	P03346	O43313	0.28901
62994	P03346	P48380	0.58858
62995	P03346	Q9BZE2	0.27126
62996	P03346	Q8IYA7	0.40452
62997	P03346	Q93073	0.09655
62998	P03346	Q8N393	0.31436
62999	P03346	Q7Z4T9	0.9529
63000	P03346	Q14498	1.059
63001	P03346	Q16531	0.81024
63002	P03346	P25208	0.99829
63003	P03346	Q13156	0.35755
63004	P03346	P50548	0.7271

P03346	Q9H814	0.57057
P03346	Q9BVL2	0.72769
P03346	P62937	0.68285
P03346	Q9P2D3	0.037241
P03346	Q8IWI9	0.61719
P03346	Q96AH0	0.29287
P03346	O60506	0.67399
P03346	P23246	1.0834
P03346	Q96I27	0.31436
P03346	Q8WVR3	0.0077221
P03346	Q8TF20	0.31436
P03346	A8MXZ3	0.40421
P03346	Q13888	0.47329
P03346	Q49MI3	0.49573
P03346	Q9Y6D5	0.2691
P03346	Q8NF99	0.48931
P03346	Q8NE22	0.14894
P03346	P42772	0.89187
P03346	Q92925	0.79576
P03346	Q5T7P2	0.02884
P03346	Q5XKL5	0.48532
P03346	O95359	0.46427
P03346	Q8IX15	0.86572
P03346	P35237	0.4892
P03346	Q27J81	0.40488
P03346	Q6MZP7	0.91345
P03346	P52741	0.43781
P03346	O75865	0.19974
P03346	Q9NR55	0.74127
P03346	Q8TD26	0.39509
P03346	P56282	0.284
P03346	P55197	1.4434
P03346	Q96MG7	1.0555
P03346	Q9C0A1	0.33644
P03346	O75474	0.19883
P03346	P20264	0.38212
P03346	Q15149	0.29123
P03346	Q8WZ33	1.0595
P03346	Q9Y6Z2	0.12821
P03346	Q86YE8	0.31436
P03346	Q8NA72	0.48271
P03346	Q969X5	0.33219
P03346	P15923	0.6404
P03346	P0CG48	0.3483
P03346	Q8TDY3	0.18249
P03346	P78371	0.70595
P03346	Q5QJE6	0.88516
P03346	P59780	0.31594
P03346	Q8TEK3	0.62033
P03346	Q13618	0.56752
P03346	O60759	0.66918
P03346	Q9H307	0.9812
P03346	Q8TAD7	0.0077221
P03346	Q8NCY6	0.32217
P03346	Q6ZMY9	0.31436
P03346	Q8WTW3	0.76711
P03346	P62266	0.93627
P03346	Q86WB0	0.93708
P03346	Q6VB84	0.82071
P03346	Q9H1K1	1.0109
P03346	P78413	0.56441
P03346	Q9NZI8	0.32823
P03346	Q9BZV1	1.2424
P03346	Q58WW2	0.40762
P03346	O75886	1.2996
P03346	O75820	0.33736
P03346	Q86WP2	0.82039
P03346	Q8WXK3	0.4168
P03346	Q86X83	0.66787
P03346	P61573	0.74357
P03346	P05787	1.4645
P03346	Q9BQY9	0.77922
P03346	P25800	0.33777
P03346	A8MTZ0	0.53853
P03346	Q8IUD2	1.1112
P03346	Q9HC52	1.0098

P03346	Q8IV53	0.27403
P03346	P15172	0.44942
P03346	Q8NEZ2	0.80799
P03346	Q9H2G4	0.79429
P03346	Q8NCN5	0.19849
P03346	Q8N5M1	0.77251
P03346	Q70Z53	0.96082
P03346	Q8WXD5	1.1182
P03346	Q15796	0.62201
P03346	Q9BXS5	0.80569
P03346	Q9UKK3	0.86273
P03346	Q9H3S7	0.28533
P03346	Q86WZ6	0.31436
P03346	Q15257	0.51431
P03346	P41225	0.30193
P03346	P56693	0.77478
P03346	Q9UII4	0.4434
P03346	K7EIQ3	0.10013
P03346	Q969T9	0.67303
P03346	Q96PM5	0.57174
P03346	O75475	1.3577
P03346	P49750	1.3411
P03346	P56555	0.19669
P03346	Q49A88	0.17073
P03346	Q15185	0.65103
P03346	O14744	1.0467
P03346	Q9BW11	0.72512
P03346	Q96A26	0.34785
P03346	P23760	1.1996
P03346	Q8IZ20	0.31436
P03346	Q9BWT7	0.4803
P03346	P56524	0.30994
P03346	Q6NSW7	0.71675
P03346	P15408	0.88458
P03346	Q5JT18	0.11221
P03346	O75970	0.34021
P03346	Q9UL68	0.20768
P03346	Q9H3P2	1.1924
P03346	Q9UK11	0.29135
P03346	O76009	0.47854
P03346	Q6UWF3	0.058829
P03346	Q8N1F7	0.7092
P03346	Q9NTM9	0.42423
P03346	Q9H329	0.23737
P03346	Q9P2F5	0.091138
P03346	P17535	1.2441
P03346	Q9NUW8	0.82428
P03346	P60608	0.10147
P03346	P13010	0.43325
P03346	P04732	0.26681
P03346	Q86YW9	0.66979
P03346	Q8IYH5	0.69313
P03346	Q9BYC8	0.52493
P03346	O43298	0.31436
P03346	Q16401	0.42833
P03346	Q15327	0.54356
P03346	O14709	0.34285
P03346	Q96T51	0.3324
P03346	Q8ND83	0.12237
P03346	P19622	0.56791
P03346	Q6NX45	0.31436
P03346	Q156A1	0.39361
P03346	Q9UNS1	0.69573
P03346	Q7Z3G6	0.27687
P03346	Q9H0R1	0.39797
P03346	Q96KH6	0.032912
P03346	O95678	0.29497
P03346	O00410	1.2954
P03346	Q8NCR6	0.2975
P03346	Q5T681	0.66787
P03346	Q8N9W5	0.2256
P03346	O00712	0.43428
P03346	Q86SZ2	0.19974
P03346	A6NE82	0.46683
P03346	P05114	0.43324
P03346	Q9H4P4	0.35636

P03346	P07738	0.13177
P03346	O75335	0.47367
P03346	Q5PSV4	0.69594
P03346	Q3SY52	0.31436
P03346	A5LHX3	0.31946
P03346	Q04743	0.60536
P03346	Q969H6	1.0569
P03346	O75534	0.51545
P03346	Q96NA2	0.29144
P03346	Q71F56	0.70213
P03346	P19367	0.35383
P03346	Q15906	1.2523
P03346	O00287	0.38899
P03346	Q9ULE6	0.96272
P03346	Q8IV03	0.1189
P03346	P15121	0.31372
P03346	Q9HC77	0.87076
P03346	Q9NQY0	0.55748
P03346	O75170	0.7731
P03346	P60709	0.17041
P03346	P26641	0.66337
P03346	Q92858	0.27936
P03346	Q9UJY1	0.73028
P03346	Q9NX40	0.71965
P03346	Q9Y5J7	0.38256
P03346	Q93074	0.62696
P03346	Q53GS7	0.7748
P03346	P78358	0.8379
P03346	Q63HM1	0.45465
P03346	P78344	0.64438
P03346	P58557	0.32514
P03346	Q5TD94	0.32867
P03346	Q96QT6	0.99279
P03346	Q8IVF2	0.32217
P03346	Q99426	0.40646
P03346	O60318	0.12686
P03346	C9JLW8	0.035984
P03346	Q9NPI1	1.1453
P03346	P31276	0.93576
P03346	Q5H9F3	0.83748
P03346	O95707	0.84413
P03346	P36543	0.41387
P03346	Q9NVK5	0.37004
P03346	P17026	0.27098
P03346	P19013	0.63855
P03346	O43815	0.44982
P03346	Q99856	0.99026
P03346	Q9UMS0	1.2158
P03346	P49789	0.71335
P03346	O43255	0.49178
P03346	P53803	0.25184
P03346	P17022	0.34285
P03346	P61254	0.56901
P03346	P23409	0.3371
P03346	Q96B42	0.15106
P03346	O14757	0.57005
P03346	Q66K74	0.39023
P03346	A6NFQ7	0.71675
P03346	Q96E16	0.18669
P03346	Q9BQ48	0.35035
P03346	Q6P050	0.044653
P03346	Q8N5A5	0.98821
P03346	P10746	0.070149
P03346	Q8WU03	0.017346
P03346	Q7Z7J5	0.79885
P03346	Q9ULH7	1.1244
P03346	Q8TBC5	0.34285
P03346	Q86VX9	0.18908
P03346	O95347	0.54682
P03346	Q9HBG6	0.27148
P03346	Q9Y678	0.73223
P03346	Q9H9Y6	0.79813
P03346	P38919	0.43752
P03346	P0C7H9	0.34408
P03346	P27348	0.60811
P03346	Q5BKY9	0.0077221

P03346	P56537	1.3571
P03346	Q14141	0.24173
P03346	Q9NX95	0.40627
P03346	Q16520	1.3202
P03346	O95931	1.072
P03346	Q12986	0.46222
P03346	Q5HY98	0.31436
P03346	Q12981	0.32001
P03346	Q86WG5	0.29048
P03346	O60573	0.77468
P03346	O43264	0.67768
P03346	P35227	0.83862
P03346	Q86TG7	0.76047
P03346	P62495	0.78457
P03346	Q7Z589	0.74315
P03346	Q04206	0.59224
P03346	Q06250	0.12821
P03346	P18054	0.26966
P03346	Q2TAK8	0.90302
P03346	Q8N660	0.2685
P03346	P36639	0.44095
P03346	Q8IZ40	0.90354
P03346	Q5T3J3	1.286
P03346	Q9UIC8	0.55281
P03346	Q68DI1	0.31436
P03346	Q7L2E3	0.47278
P03346	P33076	0.67986
P03346	Q8N9W8	0.65405
P03346	Q8IVG9	0.34294
P03346	Q96GX8	0.0077221
P03346	Q9UBU7	0.28145
P03346	P07910	0.65875
P03346	B9ZVM9	0.13682
P03346	O14682	0.24083
P03346	Q9HAJ7	0.72377
P03346	Q12802	0.29716
P03346	Q9BSB4	0.59468
P03346	Q86UE3	0.31436
P03346	Q53H80	0.61993
P03346	O95782	0.50253
P03346	Q71UM5	0.74156
P03346	Q86WU2	0.27165
P03346	P51826	0.70369
P03346	Q2TAZ0	0.66787
P03346	Q49B96	0.33623
P03346	P07476	0.1775
P03346	Q9NTX7	0.62836
P03346	Q8TAK6	0.45852
P03346	Q8N9Y4	0.078026
P03346	Q9H9D4	0.81093
P03346	O00170	0.78527
P03346	Q6W0C5	0.21901
P03346	Q9BRQ6	0.60438
P03346	Q8IV54	0.41915
P03346	Q8NA47	0.0077221
P03346	Q9NXR1	0.40251
P03346	P23786	0.22883
P03346	P0CJ85	0.6797
P03346	Q9P2S5	0.33677
P03346	Q99816	0.47178
P03346	P24864	0.62892
P03346	O15061	0.90394
P03346	Q9NX18	1.0071
P03346	Q96RS6	0.49807
P03346	Q53HL2	0.99405
P03346	P53618	0.82388
P03346	P14136	0.27393
P03346	Q9UQC2	0.42636
P03346	Q9UKT8	0.59623
P03346	Q9UMY4	0.3759
P03346	Q8IYN6	0.033045
P03346	P98170	0.32274
P03346	Q0VG06	0.7527
P03346	Q5T200	0.479
P03346	Q9NZD8	0.92951
P03346	Q3MIS6	0.31436

P03346	Q8N9K5	0.31436
P03346	Q8N895	0.72538
P03346	H3BNL1	0.0057171
P03346	Q9P209	0.98246
P03346	Q9ULD0	0.47651
P03346	P52737	0.85532
P03346	Q96BP3	0.35639
P03346	Q9HCH5	0.72116
P03346	Q9Y383	0.37335
P03346	O95238	0.62145
P03346	Q8N684	0.72199
P03346	Q12906	1.4304
P03346	Q9NXV2	1.5071
P03346	Q7L3S4	0.31436
P03346	O95163	0.86669
P03346	O94776	1.0655
P03346	P28698	0.80408
P03346	Q6ZMS7	0.49207
P03346	Q07021	0.85059
P03346	Q9ULJ3	0.81535
P03346	P60329	0.40421
P03346	P13056	0.76403
P03346	Q7Z739	0.66787
P03346	Q9NQ69	0.32758
P03346	Q6ICG8	0.15615
P03346	Q9BVT8	0.18669
P03346	H3BNL8	0.093534
P03346	O00321	0.59456
P03346	Q16222	0.39917
P03346	Q5T4T6	0.48532
P03346	O60232	0.88126
P03346	Q9H1L0	0.037061
P03346	Q96FH0	0.57969
P03346	Q9UN30	0.67357
P03346	Q13835	0.45247
P03346	Q96MY1	0.0030483
P03346	Q6ZRV2	0.05054
P03346	Q6PJG2	1.0775
P03346	P61218	0.39077
P03346	Q5T753	0.02884
P03346	P11142	0.46893
P03346	Q14157	0.54888
P03346	Q9UM13	0.75019
P03346	P25686	0.33635
P03346	A8MSY0	0.51797
P03346	Q01850	1.1499
P03346	O00268	1.1351
P03346	P52951	0.41368
P03346	Q9NWK9	0.51534
P03346	P31948	1.1289
P03346	O95671	0.30272
P03346	Q9H1H9	0.53583
P03346	Q8WW59	0.44546
P03346	P82979	0.73986
P03346	Q8IXJ6	0.30827
P03346	Q13023	0.76475
P03346	O60806	0.7711
P03346	Q9P2T0	0.50795
P03346	Q92886	0.40266
P03346	Q6ZS46	0.097853
P03346	Q96J94	0.24392
P03346	P02794	0.83316
P03346	P41161	0.58257
P03346	O75940	0.99671
P03346	Q8TDY2	1.0268
P03346	P18583	0.67782
P03346	C9J3V5	0.16168
P03346	Q9UHN1	0.33966
P03346	P56177	0.68689
P03346	Q96CS4	0.31436
P03346	Q8NFG4	0.95898
P03346	P48668	0.56559
P03346	Q9NPB6	0.71273
P03346	Q9P0L1	0.34285
P03346	Q9BQ13	0.3559
P03346	Q9NUL3	0.90311

P03346	Q15056	0.50024
P03346	Q96M29	0.098591
P03346	P60174	0.31474
P03346	O60244	0.69619
P03346	Q9C035	0.62746
P03346	Q9BYW2	0.27122
P03346	Q9H7J1	0.11316
P03346	P28062	0.27531
P03346	Q9H583	1.2195
P03346	Q96IF1	0.39035
P03346	Q96FA7	0.21218
P03346	Q9H4W6	0.3142
P03346	Q8WTW4	0.63302
P03346	A8MWD9	0.43888
P03346	Q9ULM3	1.1015
P03346	Q13190	0.26687
P03346	Q8WWB5	0.024172
P03346	Q9Y2Z2	0.093068
P03346	Q92826	0.38236
P03346	Q86XG9	0.20041
P03346	Q04741	0.25665
P03346	Q9BYE4	0.1267
P03346	P57075	0.57332
P03346	Q6ZQN3	0.14944
P03346	P08621	0.75238
P03346	Q6ISB3	1.0208
P03346	Q86VN1	0.73862
P03346	Q63HK5	0.44917
P03346	Q00587	0.41991
P03346	Q5TB30	0.52474
P03346	P15173	0.27649
P03346	Q08378	0.55199
P03346	Q14532	0.45506
P03346	P55854	0.81086
P03346	Q5T653	0.36869
P03346	Q8IXT5	0.20671
P03346	Q9NX01	0.52949
P03346	Q08050	0.55808
P03346	Q96G21	1.1327
P03346	Q8IUC1	0.51027
P03346	Q9GZL8	0.12821
P03346	Q9NVA1	0.14661
P03346	Q9BPY8	0.45864
P03346	Q17R26	0.18405
P03346	Q5JSS6	0.11646
P03346	Q9H8E8	0.93209
P03346	P11940	0.59808
P03346	Q9UMX0	0.95095
P03346	Q8IX03	1.2138
P03346	Q9UNX4	0.55433
P03346	P60953	0.45024
P03346	Q9Y2U9	0.32217
P03346	Q14209	1.2864
P03346	O00193	0.12821
P03346	P12525	0.66312
P03346	P78386	0.48588
P03346	P42226	1.0337
P03346	Q13242	0.38429
P03346	Q8N5I9	0.0077221
P03346	P0DI83	0.48532
P03346	Q9BQP7	0.14441
P03346	Q53QW1	0.0077221
P03346	P52742	0.35506
P03346	Q6UN15	0.41273
P03346	Q8WW35	0.66787
P03346	Q8WV22	0.35126
P03346	P69208	0.39865
P03346	Q9UDV7	0.32869
P03346	Q9H825	0.46931
P03346	Q6MZT1	0.20055
P03346	Q8IUR6	1.211
P03346	P18754	1.1134
P03346	Q9C0D4	0.31436
P03346	Q9BYE7	0.93606
P03346	Q15306	0.96747
P03346	Q9H2C0	0.84908

P03346	P29692	0.53711
P03346	Q9NPG2	0.18476
P03346	Q8WTR7	0.80821
P03346	P60409	0.40421
P03346	O60296	0.75029
P03346	Q9BWD3	0.042514
P03346	Q96RL1	0.91191
P03346	P31943	0.77544
P03346	Q9Y5Q3	1.0804
P03346	Q14129	0.18789
P03346	Q96C03	0.19801
P03346	Q9NVP1	0.32037
P03346	Q9NT62	0.66634
P03346	Q9BR76	0.58858
P03346	Q9P206	0.079707
P03346	P15622	0.31436
P03346	Q9BXM0	0.8808
P03346	Q9NVF7	0.033045
P03346	Q99767	0.52381
P03346	Q96T21	0.46003
P03346	E9PI22	0.03215
P03346	Q8NDQ6	0.4754
P03346	Q9UBC3	0.35859
P03346	Q7Z6K1	0.30661
P03346	Q08495	0.37177
P03346	Q75QN2	0.88882
P03346	Q93050	0.31235
P03346	Q9P2Y4	0.46703
P03346	Q96NU7	0.16571
P03346	P23759	0.44525
P03346	Q9NUQ6	0.65405
P03346	Q96HM7	0.021317
P03346	O95630	0.30098
P03346	P62993	0.25485
P03346	Q5SWA1	0.0070612
P03346	P10588	0.3835
P03346	Q3KRA6	0.11292
P03346	Q14457	0.61186
P03346	Q96QS3	0.31976
P03346	Q13522	0.43501
P03346	O76014	0.47854
P03346	Q9HCK0	0.31644
P03346	Q8IVC4	0.31436
P03346	A6NCN2	0.51027
P03346	Q00839	0.7396
P03346	O60832	0.86912
P03346	Q9H7D7	0.65405
P03346	Q96AE4	0.87019
P03346	O43396	0.29833
P03346	Q12824	0.9908
P03346	Q9NQ33	0.81208
P03346	P14652	0.26926
P03346	P63279	0.75888
P03346	Q96AV8	0.6776
P03346	O43482	0.65128
P03346	Q8TA94	0.31436
P03346	Q9C0F3	0.59404
P03346	Q6PG37	0.31436
P03346	P06703	0.24825
P03346	Q9NRX3	0.0042209
P03346	O75396	0.5114
P03346	P42574	0.24622
P03346	Q96IY1	0.98886
P03346	Q96TC7	0.51061
P03346	Q7L9L4	0.9099
P03346	Q9NYG5	0.73939
P03346	Q9H2W6	0.4377
P03346	Q9NZW5	0.38073
P03346	O43186	1.125
P03346	Q9NX65	0.31436
P03346	Q16526	0.60722
P03346	O00505	1.173
P03346	Q9H8G2	0.055233
P03346	P0C7I0	0.34408
P03346	Q13247	0.38429
P03346	Q8IYF3	0.79132

P03346	Q8IZY5	0.34086
P03346	Q9NYH9	0.20732
P03346	Q96GG9	0.60572
P03346	Q8TF32	0.31436
P03346	Q9NRJ4	0.49512
P03346	O00257	1.1586
P03346	Q9Y2D1	1.4334
P03346	P27540	0.55369
P03346	Q9NWV4	0.12821
P03346	Q8N5J4	0.86768
P03346	A8MQ14	0.31436
P03346	Q86XK2	0.77334
P03346	Q9P1Y5	0.12027
P03346	Q86TX2	0.31629
P03346	Q5JSZ5	0.66787
P03346	Q9NP08	0.50207
P03346	Q86UD4	0.31436
P03346	P58340	0.7624
P03346	Q96SE7	0.31436
P03346	Q86U86	1.2708
P03346	Q9UIF7	0.44976
P03346	Q6ZRP0	0.0077221
P03346	Q96AY2	0.91889
P03346	Q99666	0.29545
P03346	Q96DM1	0.02649
P03346	Q8N319	0.23171
P03346	Q969V6	1.5183
P03346	Q9BUR4	1.0566
P03346	Q9HCG1	0.32241
P03346	Q86WQ0	0.80058
P03346	Q9BTT4	0.88548
P03346	P49005	0.73761
P03346	Q7Z614	0.70874
P03346	Q9NZI5	0.77514
P03346	Q15942	0.3631
P03346	P58304	0.53667
P03346	P21673	0.44554
P03346	Q6P0N0	0.46306
P03346	Q92552	0.96448
P03346	Q8NFH5	0.65126
P03346	D6RGH6	1.408
P03346	P62424	1.0566
P03346	P16989	0.51604
P03346	Q13438	0.3358
P03346	P26358	0.48717
P03346	P62273	0.5294
P03346	Q96NL8	0.20041
P03346	P28289	0.30407
P03346	Q15386	0.51528
P03346	A6NJT0	0.32488
P03346	Q8WWZ8	0.30386
P03346	Q6IPT2	0.15847
P03346	Q9UK41	0.81621
P03346	P62714	0.79091
P03346	Q15560	1.0868
P03346	P67775	0.69165
P03346	Q9P258	0.6922
P03346	P19338	0.85432
P03346	Q9P2F9	0.31436
P03346	Q8TAF7	0.31436
P03346	O43776	0.077096
P03346	Q9UBL3	0.54182
P03346	P55884	0.47197
P03346	Q14204	0.39303
P03346	Q6P3W6	0.20041
P03346	Q5VVS0	0.099891
P03346	Q7L5D6	0.32035
P03346	Q99717	0.59502
P03346	P0C7X2	0.31644
P03346	P0C7W0	0.0077221
P03346	Q8IZE3	0.31629
P03346	O14646	0.84901
P03346	Q92748	0.9834
P03346	P20823	0.33472
P03346	P49459	0.26571
P03346	Q6XZF7	0.41963

P03346	Q9H0E3	0.83226
P03346	Q9Y3M9	0.31436
P03346	Q96BR9	0.31644
P03346	Q9P299	0.50306
P03346	P17038	0.31436
P03346	Q3SY46	0.36736
P03346	P15559	0.33425
P03346	Q9BYD2	0.35035
P03346	Q92688	0.49807
P03346	Q92831	0.80513
P03346	Q9UBQ0	0.23882
P03346	Q9H4Q3	0.24147
P03346	P25440	0.74785
P03346	Q99638	0.69529
P03346	Q96IK1	0.31764
P03346	P0CJ89	0.6797
P03346	Q02575	0.30595
P03346	Q96RE7	1.2139
P03346	O14613	0.25795
P03346	A6NK75	0.31436
P03346	Q9H609	0.31436
P03346	O15350	0.39063
P03346	P49918	0.8359
P03346	Q9H4B0	0.17422
P03346	Q7Z2X4	0.32308
P03346	Q96N38	0.31436
P03346	O95153	0.88554
P03346	Q9NXK8	0.85722
P03346	O15105	0.32916
P03346	P26599	0.51652
P03346	Q9NZM4	0.66787
P03346	O75626	0.39311
P03346	Q9NTW7	0.31436
P03346	Q6NT04	0.31623
P03346	Q9Y697	0.74987
P03346	Q9BUJ2	1.3931
P03346	Q9H173	0.22607
P03346	P83731	0.777
P03346	Q8NDY6	0.81266
P03346	Q9UI08	0.38271
P03346	Q8ND04	0.80865
P03346	A6NFY7	0.19558
P03346	Q14203	1.0185
P03346	P60866	0.68912
P03346	Q99502	0.19123
P03346	Q9Y3S2	0.12714
P03346	Q9BSM1	1.0963
P03346	Q96EK7	0.62751
P03346	Q9UBX0	0.51937
P03346	Q8TAF3	0.90189
P03346	P09467	0.34447
P03346	Q15847	0.12821
P03346	Q9Y5K6	0.43381
P03346	Q9Y534	0.77514
P03346	O14579	0.55508
P03346	Q9NWW7	0.0055509
P03346	P69905	0.38076
P03346	Q92945	1.0841
P03346	Q9BX66	0.45129
P03346	Q9BYE0	0.44815
P03346	O00189	0.54953
P03346	Q9H0W9	0.83215
P03346	O60682	0.64543
P03346	Q13526	0.66868
P03346	Q13287	0.75968
P03346	Q9NRL2	1.0377
P03346	Q14586	0.12564
P03346	Q16254	1.1321
P03346	Q7RTU5	0.71524
P03346	Q00978	1.1771
P03346	Q00994	0.85383
P03346	Q68CZ2	0.64872
P03346	Q9H379	0.052842
P03346	Q6PI98	0.80838
P03346	P49458	0.12229
P03346	Q96CW5	0.92204

P03346	Q05084	0.54689
P03346	P13667	0.44143
P03346	Q9P1F3	0.031128
P03346	Q9NZJ4	0.2776
P03346	P29084	1.3881
P03346	Q86XI2	0.67152
P03346	Q04864	1.4649
P03346	Q92562	0.36869
P03346	Q7RTT6	0.50821
P03346	Q96AQ7	0.75005
P03346	Q08211	0.46177
P03346	A8K010	0.10398
P03346	P0CW22	0.26013
P03346	Q13769	0.85549
P03346	Q8NFW8	0.25265
P03346	Q96B26	0.90032
P03346	P17014	0.32869
P03346	P22557	0.26423
P03346	P35573	0.54666
P03346	P63241	0.60747
P03346	P61024	0.84472
P03346	O60902	0.27238
P03346	O75030	0.79704
P03346	Q3I5F7	0.31089
P03346	Q13610	0.38523
P03346	P59990	0.40421
P03346	Q92670	0.33573
P03346	A8MVA2	0.40421
P03346	Q9Y2X8	0.097659
P03346	Q9NXW9	0.14527
P03346	P37198	0.84762
P03346	Q9ULL0	0.042085
P03346	P0DMB1	0.03215
P03346	P20618	0.61155
P03346	Q9BYQ6	0.40421
P03346	Q8TDN4	0.74915
P03346	Q6ZNB5	0.3001
P03346	Q9H213	0.21317
P03346	Q53HC0	0.88011
P03346	Q9Y5R5	0.4512
P03346	Q8N587	0.31436
P03346	Q9BQQ7	0.3017
P03346	P10074	0.58416
P03346	O43316	0.48603
P03346	Q9UM63	0.45516
P03346	Q9BXY8	0.55091
P03346	Q14749	0.75573
P03346	P62899	0.66528
P03346	Q9H0F6	0.30748
P03346	P55060	1.142
P03346	Q5VVH5	0.90654
P03346	P0CI00	0.052348
P03346	Q8NB42	0.31436
P03346	Q86SQ9	0.2697
P03346	Q8NFH3	0.9376
P03346	P80294	0.82607
P03346	Q8N1G4	0.61963
P03346	Q96N03	0.66787
P03346	O60224	0.50821
P03346	Q9NV72	0.31436
P03346	P14653	0.60023
P03346	Q6NXP2	0.13639
P03346	P41002	1.0998
P03346	Q969G9	0.32226
P03346	P42771	0.61052
P03346	P63272	0.91126
P03346	Q5T1N1	0.20041
P03346	O95067	0.67346
P03346	Q969Q0	0.40345
P03346	Q96KN7	0.26083
P03346	Q86VK4	0.31436
P03346	Q3SXZ3	0.31436
P03346	Q8WUH2	0.95094
P03346	O00192	0.39219
P03346	Q9NR33	0.45672
P03346	Q8TE85	0.51353

P03346	Q9UI95	1.3116
P03346	Q8N5F7	1.4735
P03346	Q92698	0.43068
P03346	P35658	1.0245
P03346	P13196	0.3707
P03346	O00570	1.2085
P03346	Q5T5J6	0.0041285
P03346	P52298	0.61199
P03346	P52565	0.24952
P03346	P49593	0.27913
P03346	O00409	0.53018
P03346	P31269	1.0022
P03346	Q9HAN9	0.40198
P03346	O00232	0.24174
P03346	Q9Y376	0.44312
P03346	Q14152	1.1998
P03346	O94842	0.97768
P03346	Q5TA31	0.6447
P03346	A6NEJ1	0.50821
P03346	Q6PIJ6	0.49807
P03346	Q15269	0.39995
P03346	O75330	0.10891
P03346	Q96FC7	0.32015
P03346	Q96RJ6	0.72512
P03346	Q9UBC9	0.73821
P03346	Q6ISU1	0.12063
P03346	Q9H0B3	0.14441
P03346	Q9NXH3	0.13021
P03346	Q15652	0.60922
P03346	A8MUQ0	0.10245
P03346	O43390	0.9144
P03346	Q96BV0	0.31436
P03346	A6NK39	0.51797
P03346	Q9H165	0.27659
P03346	P05937	0.3744
P03346	Q09FC8	0.52569
P03346	P55212	0.5604
P03346	A2RU54	0.27791
P03346	Q8N859	0.31436
P03346	O60662	0.25349
P03346	Q8WTP9	0.11615
P03346	Q68E01	0.77609
P03346	Q70YC5	0.50381
P03346	P52743	0.30845
P03346	Q9NRF2	0.28246
P03346	Q9HD34	0.42864
P03346	P13378	0.35755
P03346	O15090	0.27009
P03346	Q9HCS7	0.77413
P03346	O60810	0.13291
P03346	P82909	0.55092
P03346	P46976	0.55502
P03346	Q96JJ3	0.45997
P03346	Q86Z02	0.26531
P03346	Q96EH3	0.35757
P03346	Q16629	0.71368
P03346	Q13114	0.45103
P03346	P17483	0.40257
P03346	Q9BYQ3	0.40421
P03346	Q96DG6	0.32467
P03346	Q8NBB4	0.34285
P03346	Q9NV92	0.20982
P03346	P42166	0.41424
P03346	Q5TYM5	0.32015
P03346	Q9HCI6	0.051831
P03346	Q9Y4B6	1.1309
P03346	O00472	0.82513
P03346	Q7Z6I5	0.078049
P03346	O75151	0.62579
P03346	O14972	0.30942
P03346	P55196	0.29903
P03346	Q96C90	0.13021
P03346	O75821	0.75623
P03346	Q03923	0.2741
P03346	Q9NRF9	0.41315
P03346	Q8TEE9	0.91815

P03346	Q8N3X1	0.48532
P03346	Q96BY2	0.74329
P03346	O75764	0.47702
P03346	Q58A44	0.20041
P03346	O43818	0.40136
P03346	O00291	0.66993
P03346	Q96EI5	0.74266
P03346	P0CI01	0.040024
P03346	P60842	0.2192
P03346	Q9UKJ5	0.51667
P03346	Q12983	0.21047
P03346	Q9P278	0.92959
P03346	O75208	0.13301
P03346	A0JNW5	0.0046665
P03346	P56211	0.31284
P03346	O95644	1.0229
P03346	Q8NCP5	0.31436
P03346	Q8TAP4	0.26636
P03346	Q9BYC9	0.92832
P03346	Q6H8Q1	0.44572
P03346	P55072	0.31183
P03346	Q9BTE1	0.38066
P03346	Q6XD76	0.65096
P03346	P22626	0.84628
P03346	Q6IBW4	0.059658
P03346	Q9UJW8	0.31436
P03346	Q9BZE7	0.12821
P03346	Q8TDC0	0.78707
P03346	Q9H0E2	0.41487
P03346	Q9BWK5	0.20041
P03346	Q86V15	0.5379
P03346	Q9BYH8	0.63558
P03346	Q9UI30	0.67304
P03346	Q6P3W7	0.2786
P03346	Q8IYX8	0.19876
P03346	Q5JY77	0.8379
P03346	Q8IWE4	0.16249
P03346	Q969P5	0.46321
P03346	P49321	0.50122
P03346	Q9Y3B2	0.99024
P03346	O15194	0.17092
P03346	Q01094	0.90913
P03346	P09496	0.33995
P03346	Q9BY31	0.31436
P03346	P36871	0.27618
P03346	Q9UID3	0.33905
P03346	Q569H4	0.0077221
P03346	P46939	0.62909
P03346	Q13829	0.91986
P03346	Q2VWA4	0.41921
P03346	Q7L273	0.3559
P03346	Q9BQ89	0.82175
P03346	Q6NZI2	1.2277
P03346	Q9NUA8	0.24742
P03346	P17032	0.30845
P03346	Q9BZL4	0.8379
P03346	Q9H1Q7	0.017694
P03346	O94906	0.59299
P03346	Q86T82	0.50066
P03346	F5GYI3	0.10275
P03346	Q9BT92	0.76351
P03346	Q03393	0.18587
P03346	P51668	0.60607
P03346	P49715	0.30636
P03346	A5YKK6	1.2404
P03346	Q9BUQ8	0.30911
P03346	Q9NPJ4	1.4753
P03346	Q8NAV2	0.32217
P03346	Q8N2I2	0.31436
P03346	Q6NVV1	0.44865
P03346	Q9Y333	0.9835
P03346	Q9UEU5	0.6395
P03346	Q4V328	0.037097
P03346	Q9ULX9	0.74398
P03346	P16157	0.35835
P03346	P17096	0.57141

P03346	Q13398	0.31436
P03346	Q66K89	0.78892
P03346	Q53GI3	0.34285
P03346	Q9C0F1	0.4555
P03346	Q92754	0.6818
P03346	Q8NF64	1.2739
P03346	P26038	0.26393
P03346	P35268	0.67797
P03346	Q9HD47	0.44146
P03346	Q9Y3E5	0.72765
P03346	Q07955	0.83833
P03346	Q8N554	0.31436
P03346	P41252	0.78233
P03346	Q9BWG4	0.31505
P03346	Q5T447	0.44665
P03346	A6NJL1	0.34285
P03346	P17025	0.31436
P03346	O43463	0.83694
P03346	O60784	0.66751
P03346	Q9BR61	0.42689
P03346	Q9Y5J3	0.42775
P03346	P0C7H8	0.40421
P03346	O15055	0.48958
P03346	Q96AX9	0.32276
P03346	P15621	0.52569
P03346	Q01469	0.49166
P03346	P31274	0.52225
P03346	Q9UBC0	0.43979
P03346	P17010	0.51548
P03346	Q16342	0.29065
P03346	A6NEY3	0.028905
P03346	O43345	0.31436
P03346	A6NDN3	0.11048
P03346	Q6P9A1	0.31436
P03346	Q15059	1.3786
P03346	P59817	0.27653
P03346	A8MZA4	0.0055218
P03346	Q9BUL9	1.1361
P03346	P02545	0.80404
P03346	Q9H1B4	0.67477
P03346	Q53HI1	0.085
P03346	A8MT65	0.31436
P03346	Q96LK0	0.28986
P03346	Q01860	0.82991
P03346	A8K0Z3	0.24163
P03346	P61571	0.74357
P03346	Q9NZP6	0.046204
P03346	Q92750	0.79022
P03346	Q9HCX3	0.31436
P03346	Q02410	0.7377
P03346	Q92843	0.68455
P03346	Q9ULJ6	0.71354
P03346	O75182	1.291
P03346	O75896	0.11635
P03346	Q04917	0.4713
P03346	Q6EKJ0	0.41934
P03346	Q9NWT8	1.0257
P03346	Q5EBL8	0.27837
P03346	Q0D2I5	0.36736
P03346	Q5T440	0.20736
P03346	P09565	0.066272
P03346	Q64ET8	0.32217
P03346	P61077	0.50466
P03346	Q13427	0.64676
P03346	Q8N8U2	0.73269
P03346	Q96SQ7	0.39968
P03346	O95997	1.0108
P03346	Q2V2M9	0.48977
P03346	Q8TDB4	0.15367
P03346	Q96H20	1.1484
P03346	Q9BTP7	1.2551
P03346	O14867	0.71603
P03346	Q9BZE9	0.67074
P03346	Q9BVI4	0.61859
P03346	P09132	0.52288
P03346	Q08945	1.2584

P03346	Q9H3H9	0.74266
P03346	Q9UIA9	0.71827
P03346	Q9NYW8	0.3493
P03346	P20719	0.34215
P03346	Q5T6F2	0.059122
P03346	O14602	0.687
P03346	Q9NXV6	0.7221
P03346	O60809	0.13291
P03346	Q8N0Z3	0.66142
P03346	Q658L1	0.092368
P03346	Q8TD31	0.11766
P03346	Q93034	0.76791
P03346	O00151	0.36662
P03346	P60014	0.40421
P03346	Q13351	0.24179
P03346	O43805	0.73629
P03346	Q5BJF2	0.32996
P03346	P22103	0.10501
P03346	Q9BQI9	0.75728
P03346	Q8WWX9	0.68371
P03346	O00186	0.88556
P03346	O14508	0.46528
P03346	P62826	0.6236
P03346	Q8TB72	0.81129
P03346	Q00987	0.24456
P03346	Q8TEW0	0.27001
P03346	Q96FZ2	0.096385
P03346	P61962	0.98152
P03346	Q9BWG6	0.31086
P03346	Q12834	0.81339
P03346	O00566	0.65759
P03346	P0CG40	0.26056
P03346	A5A3E0	0.12449
P03346	O15519	0.48875
P03346	O15198	0.63064
P03346	Q0VGE8	0.31436
P03346	Q9NZ81	0.88516
P03346	Q9Y664	0.18574
P03346	O75444	0.69469
P03346	P30305	0.42144
P03346	Q96FA3	0.36925
P03346	P62879	0.61016
P03346	Q8N7K0	0.31436
P03346	Q9BT78	1.2317
P03346	Q6PGP7	1.0249
P03346	Q6QNY0	0.58253
P03346	Q96FZ7	0.25747
P03346	Q9UKJ3	0.31701
P03346	Q96A49	0.32217
P03346	Q9H4T2	0.34285
P03346	Q8IYB7	0.34269
P03346	Q9BQ90	1.0087
P03346	O95793	0.52023
P03346	Q53T94	0.57036
P03346	Q8TF09	0.20578
P03346	Q5U5X8	0.089479
P03346	Q8WUW1	0.254
P03346	Q8NCD3	0.73851
P03346	Q8NCQ2	0.055129
P03346	P78426	0.28291
P03346	Q8TC94	0.18249
P03346	Q8NDX6	0.33838
P03346	Q9NS25	0.47043
P03346	P09001	0.31971
P03346	Q9NSD4	0.31436
P03346	Q86SE8	0.2959
P03346	Q9Y6E7	0.52517
P03346	Q9P086	1.2846
P03346	Q9UKV3	0.78243
P03346	Q9Y255	0.30778
P03346	Q66K41	0.086253
P03346	P0C1Z6	1.4882
P03346	Q9ULX6	0.59749
P03346	Q15051	0.79777
P03346	Q499Z4	0.31436
P03346	Q8N3C0	0.47934

P03346	Q13404	1.1721
P03346	O15083	0.78869
P03346	Q13620	0.89117
P03346	P0CJ90	0.6797
P03346	Q9BT04	0.14422
P03346	P30047	0.79526
P03346	Q93045	0.35564
P03346	Q6P9G9	0.34285
P03346	O15534	0.45236
P03346	A7MD48	0.18768
P03346	Q9BV47	0.5336
P03346	Q69YN4	0.33682
P03346	B3EWF7	0.32217
P03346	O94855	0.52905
P03346	A6NJV1	0.0037973
P03346	P62891	0.66528
P03346	Q14980	1.0226
P03346	P21912	0.27179
P03346	Q9P013	0.90122
P03346	P08047	0.70163
P03346	Q9BQW3	0.35613
P03346	Q9BXP5	1.3763
P03346	Q9H098	0.072414
P03346	Q92870	0.92967
P03346	Q9H582	0.31436
P03346	Q8TBY8	0.20041
P03346	Q5TAG4	0.20041
P03346	P08708	0.47675
P03346	Q8TEP8	0.96084
P03346	P12980	1.192
P03346	P16452	0.29561
P03346	Q13889	0.30541
P03346	Q9H0E9	0.48631
P03346	Q9BYD5	0.17719
P03346	Q12816	0.5544
P03346	O95433	0.75642
P03346	Q8IYJ3	0.40761
P03346	Q86Y26	0.49807
P03346	O14503	1.0133
P03346	P11802	0.72154
P03346	O95999	0.27343
P03346	Q15853	0.88596
P03346	Q96LW9	0.34285
P03346	Q6P2H3	0.76205
P03346	Q8TF68	0.3829
P03346	Q9Y215	0.30704
P03346	P06733	0.93815
P03346	P20226	0.96545
P03346	Q6PCE3	0.22356
P03346	Q9H5F2	0.34434
P03346	O76031	0.30017
P03346	Q9HC78	0.46161
P03346	Q8IZH2	0.39212
P03346	Q9H8Q6	0.07938
P03346	P41218	0.87411
P03346	O00422	1.4224
P03346	Q05048	1.044
P03346	P20265	0.85574
P03346	Q9H5H4	0.33052
P03346	Q9NRG4	0.77723
P03346	Q8N9F8	0.31436
P03346	Q7L4I2	0.23762
P03346	Q92766	0.29568
P03346	Q86YF9	0.33308
P03346	Q9H501	0.98376
P03346	O95561	0.053798
P03346	Q7Z6B0	0.60475
P03346	Q96JM2	0.4606
P03346	Q6IS14	0.48137
P03346	Q92796	0.50101
P03346	Q8N5C8	0.50165
P03346	Q8TDD2	0.52439
P03346	P98168	0.81958
P03346	Q96MT3	0.87479
P03346	Q9Y316	0.4389
P03346	P18850	0.84134

P03346	A6NDB9	0.15886
P03346	Q9H963	0.31436
P03346	Q15024	0.87553
P03346	P17480	1.2563
P03346	Q6ZNE5	0.61745
P03346	O75347	0.16678
P03346	Q9H0R8	0.37923
P03346	Q9UK10	0.31436
P03346	Q13813	0.3054
P03346	Q3B7T1	0.74266
P03346	O00303	0.50424
P03346	Q9H869	1.628
P03346	Q9P2W6	0.20041
P03346	P22670	1.1506
P03346	Q7Z5L9	0.79377
P03346	Q8WY36	0.70946
P03346	Q69YQ0	0.47141
P03346	Q9NZN8	0.9554
P03346	Q14119	0.46294
P03346	O95600	0.31436
P03346	Q8IX01	0.33697
P03346	Q9H5Z6	0.66787
P03346	O94868	0.66787
P03346	Q9Y371	0.2666
P03346	O15056	0.28243
P03346	Q96NY9	0.78341
P03346	P61088	0.72665
P03346	Q8N8E2	0.24287
P03346	Q96CX3	0.31436
P03346	Q16637	1.1259
P03346	Q9UFW8	0.73648
P03346	Q9UNQ2	0.26563
P03346	Q14CZ0	0.22272
P03346	A8MUZ8	0.31436
P03346	O95453	0.53243
P03346	P52736	0.83914
P03346	P35908	0.52749
P03346	Q6XQN6	0.3658
P03346	O75167	0.52182
P03346	P37837	0.20987
P03346	Q9H5J8	1.3467
P03346	Q8TDW5	0.23488
P03346	Q9NX58	0.39353
P03346	P52756	0.4042
P03346	Q14919	0.41971
P03346	O43768	0.3578
P03346	P30153	0.60863
P03346	Q8NFI3	0.23423
P03346	Q03111	1.5813
P03346	Q9H2U1	0.45689
P03346	Q9BY27	0.32217
P03346	Q5VUG0	0.68149
P03346	Q9Y592	0.12821
P03346	P98169	0.32702
P03346	Q9H0A6	0.55337
P03346	O75381	0.24195
P03346	Q6P6B1	0.0077221
P03346	Q8N1F1	0.14441
P03346	Q9P219	0.68674
P03346	A6NEF3	0.028905
P03346	Q8TF05	0.41243
P03346	Q13424	0.46403
P03346	Q6UYE1	0.051053
P03346	Q8N490	0.31689
P03346	A6NNA2	0.12119
P03346	Q5I0X4	0.0077221
P03346	P57076	0.45623
P03346	P61572	0.74357
P03346	Q9UKA1	0.46008
P03346	Q9Y5P8	0.8507
P03346	O75155	1.0805
P03346	Q5TC12	0.73559
P03346	P48553	0.33524
P03346	Q13098	0.43746
P03346	P53370	0.65166
P03346	Q5SRE5	0.71791

P03346	P20132	0.22179
P03346	O43593	0.4401
P03346	Q96ST8	0.22843
P03346	Q3LI58	0.36736
P03346	Q5TA82	0.02884
P03346	Q96G25	1.2401
P03346	Q13432	0.66539
P03346	Q14592	0.31436
P03346	Q16236	0.84607
P03346	O75943	0.40255
P03346	Q15561	1.0846
P03346	Q6NZ36	0.42259
P03346	Q14934	0.67256
P03346	Q86W54	0.57809
P03346	Q9BRK4	0.71933
P03346	Q9H9A7	0.89689
P03346	Q8N1E6	0.6483
P03346	Q8WXC3	0.88862
P03346	Q9Y3P8	0.020273
P03346	Q96MG8	0.19165
P03346	Q8TD47	0.30258
P03346	Q6NYC8	0.17453
P03346	Q14103	1.0714
P03346	Q76KD6	0.32864
P03346	O75832	0.42388
P03346	Q9UHQ7	0.094527
P03346	Q9BTX1	0.20452
P03346	Q8IVN3	0.41686
P03346	O14530	0.61951
P03346	O14618	0.52128
P03346	Q9BWF3	0.28623
P03346	Q9Y5B0	0.57394
P03346	Q9H902	0.34547
P03346	Q5JTD0	1.2039
P03346	Q9C029	0.23964
P03346	P46782	0.54595
P03346	Q9UQB3	0.79197
P03346	Q9HB31	0.39029
P03346	O60225	0.50821
P03346	Q9HAU0	0.55673
P03346	O15060	0.31436
P03346	P41250	0.1624
P03346	Q8TAM2	0.30919
P03346	O15318	0.23596
P03346	Q8N7W2	0.66787
P03346	Q86Y79	0.11387
P03346	O60663	0.78011
P03346	Q14526	0.74671
P03346	Q96SW2	0.8129
P03346	Q9UKN8	0.77023
P03346	Q7Z794	0.55691
P03346	Q5U623	1.0523
P03346	Q9BQ65	0.35698
P03346	Q16650	0.54723
P03346	Q13137	1.1247
P03346	Q14CS0	0.62071
P03346	Q9Y222	1.2519
P03346	Q7Z333	0.28045
P03346	O43680	0.24082
P03346	Q15532	1.2082
P03346	Q9HAV7	0.17358
P03346	Q9C0H6	0.44985
P03346	Q92575	1.121
P03346	Q99417	1.645
P03346	Q9UHC1	0.56148
P03346	Q8N0Z6	0.56885
P03346	Q96MR7	0.27129
P03346	P07438	0.27009
P03346	P61353	0.6799
P03346	Q8IY63	0.68302
P03346	O00233	0.58983
P03346	O96004	0.58969
P03346	P62487	0.37796
P03346	P17036	0.60049
P03346	O15069	0.6555
P03346	P61960	0.47575

P03346	Q8WVL7	0.30755
P03346	Q6ZNH5	0.31436
P03346	P51398	1.3214
P03346	P01106	0.48852
P03346	O60294	0.63318
P03346	O43364	0.43764
P03346	Q6ZMW2	0.31436
P03346	Q8IU60	0.96431
P03346	Q3ZCX4	0.31436
P03346	Q8TBZ5	0.31436
P03346	Q4LE28	0.98457
P03346	O60518	1.2688
P03346	Q5M9Q1	0.15996
P03346	Q9Y675	0.34434
P03346	Q5TA50	0.17705
P03346	Q5T230	1.4509
P03346	Q9UJX6	0.61969
P03346	Q9BS34	0.31436
P03346	Q9UL40	0.77516
P03346	Q9UI32	0.47494
P03346	P41162	0.70008
P03346	Q13155	0.87419
P03346	Q9BRT3	0.15767
P03346	Q14566	0.34049
P03346	Q15555	0.25776
P03346	P0CJ79	0.31436
P03346	Q8WVK2	0.38097
P03346	P78560	0.76726
P03346	P45381	0.22643
P03346	Q6P1N0	0.61151
P03346	Q6ZPB5	0.14682
P03346	B4DX44	0.31436
P03346	O43583	0.68756
P03346	O75448	0.63786
P03346	P14649	0.44517
P03346	P62877	0.50146
P03346	Q96DX5	1.2584
P03346	Q03701	0.75777
P03346	P51843	0.24336
P03346	Q92527	0.29284
P03346	Q6UW56	0.40614
P03346	Q5TB80	0.92611
P03346	Q92879	0.39352
P03346	Q96CF2	0.44645
P03346	Q9Y421	0.57053
P03346	Q68DU8	0.18903
P03346	Q96MN9	0.79679
P03346	Q96B67	0.61654
P03346	Q9NZZ3	1.1792
P03346	Q9BWW4	0.73202
P03346	O00299	0.22344
P03346	Q9UHB7	1.5103
P03346	P25205	0.49675
P03346	Q9Y266	0.62158
P03346	P16333	0.71742
P03346	Q969S2	0.14583
P03346	Q9H0G5	0.93336
P03346	P41208	0.62414
P03346	O60870	0.18796
P03346	Q9NRS6	0.29372
P03346	O43592	0.62456
P03346	P62854	0.86594
P03346	Q8NFT6	0.20022
P03346	P16278	0.2394
P03346	Q9UHP3	0.48479
P03346	Q9NV06	0.53085
P03346	Q5H9R7	1.0703
P03346	Q12926	0.53704
P03346	P07197	0.92971
P03346	P19388	0.71915
P03346	Q9NP66	1.3021
P03346	Q9Y608	0.64342
P03346	Q14493	1.0553
P03346	P38646	0.71143
P03346	Q9HC44	0.70946
P03346	P0C7V9	0.052605

P03346	O75925	0.66727
P03346	Q9Y3D9	0.60118
P03346	Q9H910	0.35026
P03346	Q8NBF1	0.52062
P03346	O43365	0.65189
P03346	Q9Y3U8	0.83414
P03346	O75817	1.0575
P03346	Q9BXX3	0.65882
P03346	Q15528	1.4515
P03346	Q6ZNA1	0.31436
P03346	Q8NI35	0.50147
P03346	Q9Y2X3	0.85545
P03346	O60303	0.3318
P03346	Q01082	0.56272
P03346	Q92621	1.1216
P03346	P82650	0.43065
P03346	Q15699	0.76147
P03346	P28702	0.49108
P03346	P35637	0.63167
P03346	O75635	0.17334
P03346	Q96A72	0.37216
P03346	Q8IWL8	0.49807
P03346	Q9NSA3	0.92242
P03346	Q5MJ10	0.10148
P03346	O75570	0.35664
P03346	Q9HCK5	0.9068
P03346	Q9UL49	0.24934
P03346	Q9NP71	0.60917
P03346	Q9Y2V7	0.21668
P03346	Q68D10	0.12821
P03346	Q3T8J9	1.0209
P03346	Q6ZTQ3	0.47956
P03346	O60583	1.0197
P03346	Q9H8W4	0.42264
P03346	Q8TCB0	0.25642
P03346	Q8TCJ0	0.67774
P03346	P21506	0.31436
P03346	P28358	0.49
P03346	Q14186	1.3715
P03346	P50222	0.96383
P03346	Q9UII2	0.2692
P03346	O14777	0.69975
P03346	A6NF36	0.10483
P03346	Q96M43	0.20041
P03346	P51508	0.30845
P03346	Q14139	0.79417
P03346	Q9Y3R5	0.08417
P03346	Q6ICC9	0.007188
P03346	Q5VWI1	0.13503
P03346	Q07666	0.76836
P03346	P62318	1.0401
P03346	Q14687	0.66787
P03346	Q9NVH2	0.52498
P03346	Q92830	0.77131
P03346	Q9BYQ0	0.40421
P03346	Q9BYE3	0.02884
P03346	Q5HYW2	0.079707
P03346	Q9Y330	0.31436
P03346	Q9UK13	0.31436
P03346	Q2TAY7	0.49807
P03346	Q9BXW6	0.46742
P03346	Q8N0Y2	0.34285
P03346	Q5T953	0.054055
P03346	A6NK89	0.19866
P03346	Q92499	0.57503
P03346	Q9Y5B6	0.81133
P03346	Q5T686	0.066022
P03346	P04733	0.26681
P03346	Q7L4P6	0.11048
P03346	Q9C0D2	0.35785
P03346	Q8N8Z8	0.31436
P03346	Q5MJ68	0.53166
P03346	O75161	0.59038
P03346	Q86VM9	0.32105
P03346	Q96BN2	0.79965
P03346	P0CG30	0.55716

P03346	Q9BT49	0.36317
P03346	P18848	1.2955
P03346	P60411	0.40421
P03346	P04080	0.23731
P03346	Q9Y6D6	0.73621
P03346	P59998	0.3837
P03346	Q3LI77	0.36736
P03346	Q96B36	0.97009
P03346	Q14582	0.64204
P03346	Q9Y6F6	0.20843
P03346	Q9GZM3	0.34613
P03346	Q2M1V0	0.71675
P03346	Q8N954	0.16667
P03346	Q96JL9	0.31436
P03346	Q53HV7	0.84229
P03346	Q9P243	0.53478
P03346	Q8IYI8	0.31436
P03346	P99999	0.71944
P03346	Q3LHN0	0.36736
P03346	O00327	0.42918
P03346	Q8ND82	0.27653
P03346	Q8WW18	0.0077221
P03346	Q6L8G9	0.40421
P03346	Q9H2K2	0.45644
P03346	Q58F21	0.49877
P03346	Q99700	0.88628
P03346	Q9BQ70	1.5977
P03346	Q10567	0.74337
P03346	Q16890	0.63019
P03346	Q7Z4V5	0.35728
P03346	Q09666	0.98011
P03346	Q6NUQ1	0.80994
P03346	Q9BZ29	0.22818
P03346	Q9UKL3	1.1191
P03346	Q15170	0.7505
P03346	Q86TB9	1.0281
P03346	Q14151	1.0819
P03346	P12036	0.59177
P03346	Q2M2I5	0.58798
P03346	Q14153	0.12821
P03346	Q9NQR4	0.45141
P03346	Q9H4I8	0.35247
P03346	Q96EP0	0.29887
P03346	P14921	0.90073
P03346	P47914	0.5786
P03346	Q9HAW4	0.67337
P03346	Q9NWW0	0.49807
P03346	Q9BW27	0.78422
P03346	Q9H1I8	0.46683
P03346	P10155	0.733
P03346	O00161	1.0128
P03346	Q06710	0.2727
P03346	Q92794	0.3239
P03346	P13640	0.7077
P03346	Q9NS56	0.35429
P03346	Q8NAA4	0.3142
P03346	P07316	0.27132
P03346	Q9P215	0.22407
P03346	Q96PI1	0.16848
P03346	P18669	0.53998
P03346	Q9BUN8	0.13626
P03346	Q9Y5J6	0.13307
P03346	Q93083	0.27009
P03346	P84022	0.46882
P03346	O43148	1.0228
P03346	Q15643	1.1347
P03346	Q15742	0.54606
P03346	O14628	0.31436
P03346	Q86Y25	0.31436
P03346	Q8HWS3	0.6285
P03346	Q14676	0.81288
P03346	Q12904	0.20406
P03346	Q8TAG6	0.015025
P03346	P11308	0.91968
P03346	Q04726	1.2415
P03346	Q9H063	0.5776

P03346	Q8IWS0	0.65171
P03346	Q86SE9	1.1176
P03346	Q9HB71	0.74225
P03346	Q96QU4	0.32217
P03346	Q8N1W2	0.31436
P03346	Q9H9E1	0.46253
P03346	P25791	0.56684
P03346	Q14694	0.2755
P03346	Q96DT7	0.34395
P03346	Q14847	0.24749
P03346	Q8IYX0	0.31436
P03346	Q9Y6Y0	0.71184
P03346	Q8TBC3	0.3559
P03346	Q6PI47	0.3559
P03346	P08133	0.86043
P03346	Q9BW71	0.92446
P03346	Q12968	0.86955
P03346	Q86V81	1.0104
P03346	O43513	1.3586
P03346	O75843	0.79291
P03346	P11171	0.60687
P03346	P31275	0.47959
P03346	Q9NXB0	0.55633
P03346	O14980	0.99933
P03346	Q8N5Q1	0.15847
P03346	Q2M3C7	0.80513
P03346	Q6IPR1	0.14441
P03346	Q8IWE2	0.20041
P03346	Q9NVX0	1.0192
P03346	Q14264	0.20187
P03346	O76041	0.39195
P03346	Q8NHY6	0.31436
P03346	Q8WWY6	0.74266
P03346	P30154	0.64032
P03346	A1L443	0.13835
P03346	Q9Y295	0.38451
P03346	P09917	0.31318
P03346	P49354	0.48802
P03346	P17542	0.2696
P03346	P48444	0.64186
P03346	Q14232	0.26348
P03346	Q04695	0.6272
P03346	Q96IZ0	0.93292
P03346	Q96DE5	0.78189
P03346	Q9BRJ7	0.19151
P03346	Q14978	0.55489
P03346	O95232	0.82701
P03346	Q96CV9	0.69102
P03346	Q9NSB2	0.45278
P03346	Q9BRH9	0.31436
P03346	Q7Z3T8	0.22867
P03346	O96019	0.99164
P03346	P31350	0.41787
P03346	Q66GS9	0.54506
P03346	P51116	0.90296
P03346	Q13166	0.12821
P03346	Q9Y6Q2	0.17721
P03346	Q9Y221	1.0659
P03346	Q9BPZ2	0.38912
P03346	Q9Y285	0.42426
P03346	O60508	0.66701
P03346	Q9Y224	1.5799
P03346	Q9HCS4	0.40583
P03346	P49419	0.69827
P03346	Q8TCE9	0.29604
P03346	Q9UHY8	0.55168
P03346	Q9Y692	0.94282
P03346	Q9BUT9	0.14751
P03346	Q3LI81	0.36736
P03346	P20340	0.25176
P03346	Q96AA8	0.1222
P03346	P43897	0.29557
P03346	Q9BYV9	0.72849
P03346	Q8WUU5	0.50432
P03346	P50479	0.4708
P03346	A7E2V4	0.18508

P03346	Q14863	0.53117
P03346	O75665	0.8202
P03346	Q92973	1.2454
P03346	Q9ULW0	0.88417
P03346	Q8NEJ9	0.14793
P03346	Q9H8T0	0.60863
P03346	Q9Y2R5	0.39239
P03346	P10144	0.5244
P03346	P10242	1.1363
P03346	Q9NTK5	0.54635
P03346	Q96D15	0.52803
P03346	P08865	0.77937
P03346	O60481	0.64743
P03346	Q9UI15	0.31193
P03346	Q9UIS9	0.73489
P03346	Q6ZN11	0.31436
P03346	Q9Y237	0.52498
P03346	Q5VTA0	0.13291
P03346	P17661	0.64018
P03346	O95231	0.44258
P03346	Q8IVT2	0.060929
P03346	A6NDE4	0.35549
P03346	Q8IXF0	0.60186
P03346	Q9C0J9	0.36662
P03346	Q8N3R9	0.35052
P03346	Q8NB15	0.31436
P03346	P02511	0.4092
P03346	P51784	0.44991
P03346	Q6ECI4	0.4412
P03346	Q15436	0.79968
P03346	P61236	0.48532
P03346	Q9UNY5	0.34285
P03346	Q9H175	1.3844
P03346	Q4LEZ3	0.11085
P03346	Q8IYM9	0.25665
P03346	Q8WXE0	0.22802
P03346	Q9H9H5	0.21597
P03346	Q9Y3E7	0.90636
P03346	Q9UK76	0.32217
P03346	Q7LG56	0.28167
P03346	Q6ZW54	0.079753
P03346	Q8WXE9	0.77418
P03346	O43236	0.43364
P03346	Q496M5	0.34337
P03346	A9UHW6	1.0857
P03346	Q13227	0.78822
P03346	Q8N140	1.1421
P03346	Q96DP5	0.051194
P03346	Q6L8G4	0.40421
P03346	Q7L804	0.76845
P03346	Q8N960	0.2409
P03346	O14556	0.4887
P03346	Q96EA4	0.81999
P03346	Q96L21	0.46101
P03346	Q9Y2P8	0.38528
P03346	Q8NDT2	1.0366
P03346	Q96F24	0.94081
P03346	P13984	0.68238
P03346	P56945	0.62757
P03346	Q8N1G0	0.4412
P03346	O43829	0.8711
P03346	Q13609	0.17275
P03346	Q9H7R5	0.31436
P03346	Q9H9E3	0.73683
P03346	Q9NPP4	0.31859
P03346	Q8TF47	0.30647
P03346	O43541	0.72044
P03346	O75391	0.32442
P03346	Q8N5D0	0.72307
P03346	O75157	0.42708
P03346	Q8WYA6	1.0212
P03346	Q9P021	0.11392
P03346	Q15599	0.66945
P03346	Q96TA1	0.58613
P03346	Q9NQS1	0.29429
P03346	Q96S55	0.35281

P03346	O60493	0.39777
P03346	Q9GZU7	0.68923
P03346	Q9NXR7	0.58204
P03346	Q15398	1.1724
P03346	O75340	0.59523
P03346	Q9P0K7	0.28316
P03346	Q99990	0.83994
P03346	Q16667	0.25693
P03346	Q9UNN5	0.78133
P03346	P22736	0.37751
P03346	Q9UBY9	1.1468
P03346	Q5EBM4	0.31436
P03346	Q86UT6	0.24323
P03346	O14948	0.73517
P03346	Q7Z3Y9	0.47854
P03346	Q15020	0.49054
P03346	A6NKF1	1.0318
P03346	O00423	0.66229
P03346	Q08752	0.6146
P03346	P17706	0.33617
P03346	Q9Y2S7	0.41262
P03346	Q9Y2H0	0.22214
P03346	Q86XN7	0.0077221
P03346	Q92625	0.394
[truncated: 509,173 more chars]
